# HIF-1α Stabilization Hampers the Chondrogenic Differentiation of Aged Bone Marrow Mesenchymal Stem Cells by Adenosine A_2A_/A_2B_ Receptors Imbalance

**DOI:** 10.1021/acsptsci.5c00190

**Published:** 2025-06-24

**Authors:** Rui Pinto-Cardoso, Catarina Bessa-Andrês, Flávio Pereira-Costa, Rafael Neiva, Ricardo Martins-Ferreira, Bárbara Guerra-Leal, Diogo Nóbrega Catelas, Adélio Vilaça, António Oliveira, Julie Pelletier, Jean Sévigny, José Bernardo Noronha-Matos, Paulo Correia-de-Sá

**Affiliations:** † Laboratório de Farmacologia e Neurobiologia, 26706 Universidade do Porto (ICBAS-UP), 4050-313 Porto, Portugal; ‡ Center for Drug Discovery and Innovative Medicines (MedInUP)/RISE-Health: Health Research Network, 26706 Universidade do Porto (ICBAS-UP), 4050-313 Porto, Portugal; § Laboratório de Imunogenética, Departamento de Patologia e Imunologia Molecular, 26706 Universidade do Porto (ICBAS-UP), 4050-313 Porto, Portugal; ∥ Unit for Multidisciplinary Research in Biomedicine (UMIB)/ITR−Laboratory for Integrative and Translational Research in Population Health, Instituto de Ciências Biomédicas Abel Salazar − Universidade do Porto (ICBAS-UP), 4050-313 Porto, Portugal; ⊥ Serviço de Ortopedia, 674892 Centro Hospitalar Universitário de Santo António, 4099-001 Porto, Portugal; # Centre de Recherche en Rhumatologie et Immunologie, 12369 University Laval, 2325, rue de l’Université Québec, Québec G1 V 0A6, Canada

**Keywords:** mesenchymal stem cells, HIF-1α, chondrogenesis, adenosine-sensitive receptors, ecto-nucleotidases, ENT-1 transporter

## Abstract

Trauma and excessive motion are deleterious to cartilage by triggering HIF-1α stabilization, leading to changes in extracellular adenosine formation via CD73. How these changes affect adenosine A_2A_/A_2B_ receptors activation balance in chondrogenesis is unclear. We used bone marrow mesenchymal stem cells (BM-MSCs) from aged women to investigate the impact of HIF-1α overactivation on adenosine formation from ATP breakdown and subsequent A_2A_/A_2B_ receptor tone-regulating chondrogenesis. The chondrogenic differentiation of BM-MSCs from 27 postmenopausal (*Pm*) women was induced for 14 days with or without DMOG, a prolyl-4-hydroxylase (PHD) inhibitor that increases HIF-1α intracellular accumulation. Chondrogenesis was ascertained by the nuclear translocation of SOX9, type II/X collagen, and MMP13 production and by transcriptomic RNAseq analysis. Changes in the density of adenosine receptors, ecto-NTPDases, and CD73 were assessed by immunofluorescence confocal microscopy. The kinetics of ATP hydrolysis and adenosine formation was performed by HPLC. DMOG-induced HIF-1α transcriptional activity increased cartilage damage biomarkers (MMP13 and type X collagen), while decreasing cell viability, SOX9 nuclear translocation, and type II collagen production. DMOG upregulated pro-inflammatory genes and down-regulated chondrogenic gene transcripts determined by RNAseq. HIF-1α stabilization decreased CD39/CD73 amounts and activity, thus reducing adenosine formation. Hence, changes in the A_2A_/A_2B_ receptor tone resulted in preferential activation of the SCH 442416-sensitive A_2A_ receptor, which is deleterious to the cartilage. In conclusion, HIF-1α overactivation hampers the chondrogenic differentiation of aged BM-MSCs by decreasing adenosine formation from ATP hydrolysis via CD39/CD73, leading to preferential activation of the high-affinity A_2A_ versus the chondroprotective A_2B_ receptor.

## Graphical abstract

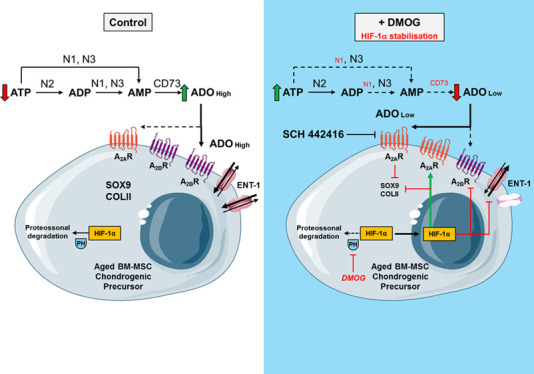

Cartilage, as an avascular tissue, has a limited oxygen supply. It is now widely accepted that hypoxia plays a critical (yet unresolved) role in cartilage homeostasis.^1–3^ However, oxygen-tension-associated mechanisms and their impact on chondrogenesis are still a matter of debate, mainly due to erratic experimental conditions. Confounding factors include cell origin, passage numbers, donors’ age, cartilage lesion scores, oxygen tension, culture media, and cell differentiation status on isolation.^4,5^ For instance, aged chondrocytes react differently to extrinsic stressors (e.g., hypoxia), thus resulting in distinct proliferation rates; the energy metabolism, response to growth factors, and autophagy protective mechanisms of aged chondrocytes are also affected,^6^ ultimately resulting in degenerative osteoarthritic diseases.^7^

The autologous transplantation of chondrocytes is used worldwide to tackle cartilage defects, yet unsolved questions remain regarding the ability of these cells to grow and differentiate in transplanted foci. Moreover, limitations to cartilage autotransplantation also involve donor sites’ accessibility, surgical risk, and tissue/cell yield, in addition to age- and pathology-associated impairments of cell differentiation.^8–10^ New approaches have emerged to overcome part of these constraints considering the use of multipotent mesenchymal stromal cells (MSCs) from different sources,^11^ although consensus in the literature regarding the ideal conditions to promote chondrogenic vs osteogenic differentiation is still missing.^12,13^ Difficulties in translating preclinical studies into clinical practice concerning osteochondral repair are related, in part, to the lack of standardized experimental procedures as previously mentioned.

During joint motion, thus favoring hypoxia and mechanical stress, both cartilage resident cells and transplanted MSCs release huge amounts of adenine nucleotides, including ATP, to the microenvironment.^14^ The peak and permanence of adenine nucleotides and adenosine formation in the extracellular milieu is balanced by the ability of the cells to release and inactivate purines by high-affinity NTPDases, ecto-5′-nucleotidase/CD73, and adenosine deaminase. These paths also determine activation of nucleoside- vs nucleotide-specific P1 and P2 purinoceptors in the vicinity, respectively.^15–17^ Our group recently reviewed data from the literature, gathering information about the putative interplay between hypoxia and the purinergic signaling cascade to osteochondral homeostasis.^4^

Increasing evidence shows that endogenously generated adenosine is paramount to control both the chondrogenic^18^ and/or the osteogenic^19^ fate of human bone marrow-derived MSCs (BM-MSCs). The low-affinity A_2B_ receptor is involved in the osteogenic differentiation of BM-MSCs, since its activation promotes cell growth and differentiation, translated into increases in alkaline phosphatase (ALP) activity and bone nodule formation. In osteogenic differentiating cells, the A_2B_ receptor signaling tone is balanced by the activation of colocalized A_1_ and A_2A_ receptors, depending on the cells' differentiation stage.^20^ Conversely, the high-affinity A_2A_ receptor subtype seems to promote cartilage homeostasis, while reducing chondrocyte cell senescence^21^ via its anti-inflammatory and antiapoptotic properties.^22,23^ The A_2B_ receptor activation normally suppresses the chondrogenic commitment and is deleterious to cartilage cells.^24^ Regrettably, most of this information derives from animal research or the use of immortalized human cell lines, while pending information is still necessary in nonmodified human BM-MSCs undergoing chondrogenic differentiation.

The hypoxia-inducible factor (HIF)-1α is a key transcription factor that responds to low oxygen tension to regulate the growth and differentiation of multiple cell types, including chondrocytes.^25–27^ HIF-1α modulates important chondrogenic transcription factors, as for instance the sex-determining region Y (SRY)-box transcription factor 9 (SOX9).^28^ Hypoxia, putatively via HIF-1α, may also regulate the transcription of several players of the purinergic signaling cascade, such as ecto-nucleotidases and P1 purinoceptors, in noncartilaginous and bone tissues–for a review, see e.g.^4^ Yet, the link between HIF-1α transcriptional activation by hypoxia and the expression and function of purinergic signaling players in the osteochondral unit is still missing in both health and disease conditions. This prompted us to explore the impact of HIF-1α overactivation under normoxic conditions using a cell-permeable prolyl-4-hydroxylase inhibitor, dimethyloxallyl glycine (DMOG),^29,30^ on the expression and function of high-affinity A_2A_ and low-affinity A_2B_ receptors in chondrogenic-differentiating aged BM-MSCs from postmenopausal (*Pm*) women. Given its relevance in this endeavor, we also investigated the putative changes operated by DMOG in the extracellular ATP breakdown and adenosine formation by NTPDases.

## Results

### Upregulation of HIF-1α in Aged BM-MSCs Favors the Transcription of Genes Contributing to Deficits in Chondrogenesis and Cartilage Damage

Mounting evidence in the literature shows that hypoxia influences chondrogenesis and that HIF-1α transcriptional activation by hypoxia may alter the expression and function of several players of the purinergic signaling cascade in the osteochondral unit.^4^ Here, we used a cell-permeable prolyl-4-hydroxylase competitive inhibitor, DMOG (30 μM), to explore the impact of HIF-1α stabilization and activation on chondrogenesis under normoxic conditions, focusing on the expression and function of high-affinity A_2A_ and low-affinity A_2B_ receptors, as well as on the extracellular ATP breakdown and adenosine formation by NTPDases in aged BM-MSCs from *Pm* women undergoing chondrogenic differentiation.

Figure 1A shows that following DMOG (30 μM) application, the HIF-1α immunoreactivity increases in the cytoplasm, which subsequently translocates to the nucleus of BM-MSCs undergoing chondrogenic differentiation, without affecting cells’ morphology. DMOG (30 μM)-induced stabilization of HIF-1α at culture days 7 and 14 favors gene transcription of *COL10A1* (Figure 1B), with a similar (yet not significant) tendency regarding the MMP13 gene transcription in some experiments; curiously, COL10A1 and MMP13 are both associated with deleterious cartilage hypertrophy.^31,32^

**Figure 1:**
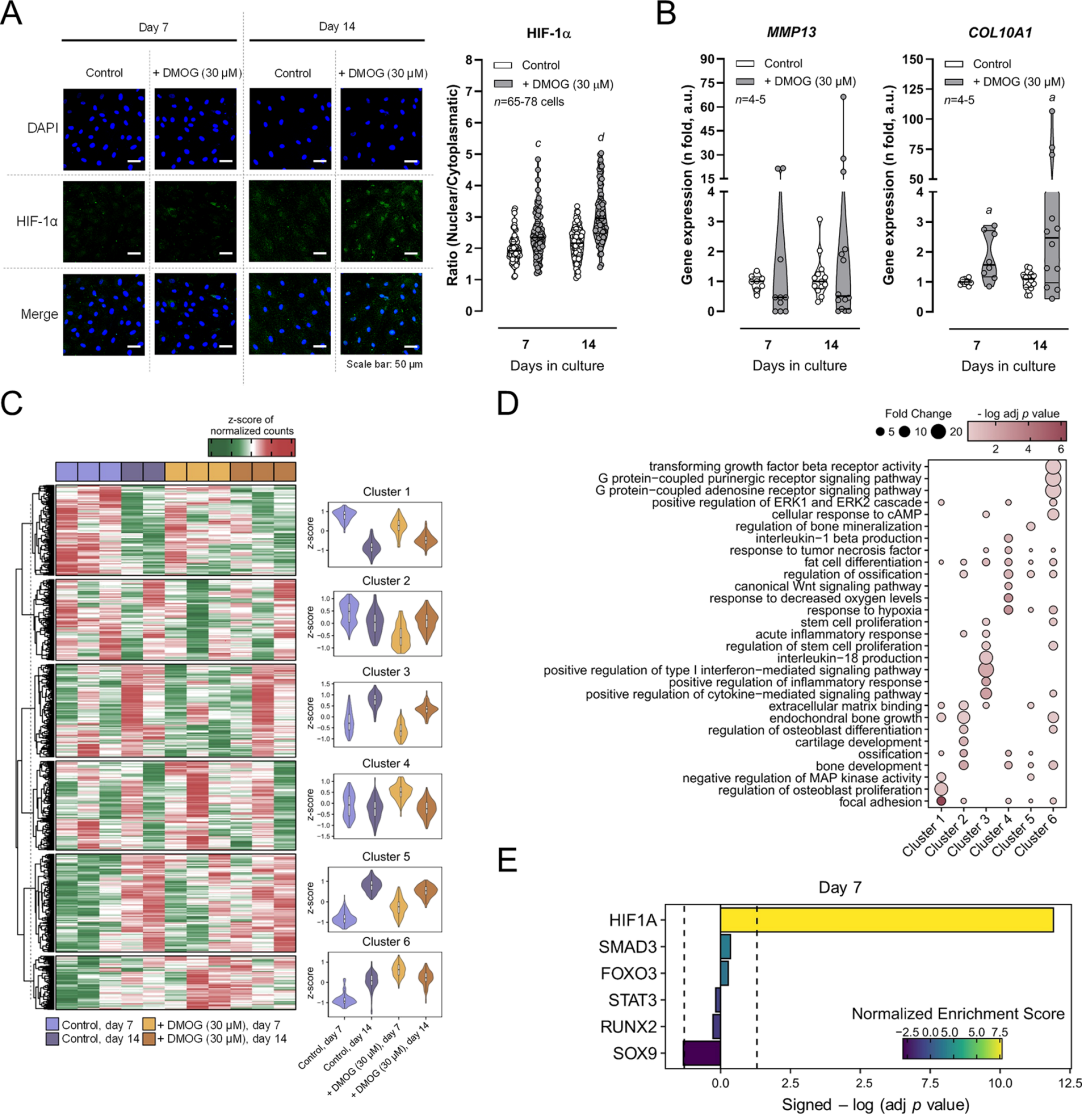
Upregulation of HIF-1α transcriptional activity impacts the chondrogenic differentiation of human bone marrow mesenchymal stem cells (BM-MSCs). The cells were allowed to grow for 7 and 14 days in a chondrogenic-inducing medium in either the absence or presence of DMOG (30 μM). In Panel A are representative images of the immunocytochemical detection of HIF-1α protein levels (green) by confocal microscopy. Blue dots represent nuclei staining with DAPI. The scale bar is 50 μm; The right-hand side panel shows immunofluorescence intensity graphs computed from acquired confocal microscopy images. Ordinates represent fluorescence intensity per cell (arbitrary units, a.u.) of the HIF-1α immunoreactivity as a function of the number of days in culture (7 and 14 days). Plotted data correspond to 65–78 cells from a 79-year-old woman. ^*c*^ *P <*0.001 and ^*d*^ *P <*0.0001 (Kruskal–Wallis test with uncorrected Dunn’s test) represent significant differences. Panel B shows the relative expression of matrix metalloproteinase-13 (*MMP13*) and type X collagen (*COL10A1*) mRNA levels detected by RT-PCR. Ordinates represent the fold-change (arbitrary units, a.u.) of the indicated mRNA levels normalized to ubiquitin C (*UBC*) determined in the same samples. Represented are triplicates from four to five *Pm* women (68 ± 6 years old). ^*a*^ *P <*0.05 (Kruskal–Wallis test with uncorrected Dunn’s test) represent significant differences. Panel C presents a heat map of the differentially expressed genes (DEGs) obtained from the pairwise comparison between all studied conditions. Gene expression is represented as the z-score of the normalized counts. The significance cutoff was raw *P*-value < 0.01 and absolute log2FoldChange >0.5. Unsupervised clustering divided the DEGs in six clusters (1–6). Represented are violin plots of the mean z-score of each DEG cluster for the indicated experimental conditions. Panel D represents a selected list of enriched gene ontology (GO) terms from indicated clusters (raw *P*-value < 0.05). The significance of enrichment is represented by the negative of the log of the adjusted *P*-value and the enrichment fold change. Panel E presents a bar plot showing the translational activity of relevant transcription factors (TF) involved in BM-MSC differentiation; the enrichment is represented by the positive or negative Normalized Enrichment Score (NES) and the statistical significance by the adjusted *P*-value (FDR). The ladder is presented as the signed −log (adjusted *P*-value), which is calculated as the −log10­(adjusted *P*-value/(NES/absolute­(NES)). Zero represents the identity between the TF activities obtained in the absence or presence of DMOG (30 μM) at culture day 7. Differential expression analysis was performed by using the DESeq2 R package (see the Materials and Methods section for further details).

The RNA sequencing analysis of both control and DMOG (30 μM)-treated chondrogenic-differentiating BM-MSCs revealed significant changes in gene expression patterns between the two studied groups (Figure 1C). The unsupervised clustering of DEGs (raw *P* < 0.05, total of 799 genes; see Supplementary Table 1) from all pairwise comparisons provided six different clusters (1–6) (see Figure 1C,D, and Supplementary Table 2). Data show that stem cell differentiation-related genes (Cluster 1) decrease in chondrogenic differentiating BM-MSCs from culture day 7 to 14, while the opposite is observed regarding gene transcripts related to inflammatory responses (Cluster 3), bone development (Cluster 5), and purinergic signaling (Cluster 6). The upregulation of HIF-1α transcriptional activity by DMOG (30 μM) increased DEGs concerning Clusters 4, 5, and 6 at culture day 7, while decreasing Cluster 2 genes at the same time point (Figure 1C); genes from Clusters 1 and 3 were unaffected by DMOG (30 μM) throughout the culture period.

From GO analysis (Figure 1D; see Supplementary Table 3), the most prominent results include the enrichment of hypoxia-related terms in Clusters 4 and 6, purinergic signaling-related terms in Cluster 6, and stem cell differentiation-related terms in Cluster 2. It is noteworthy that, besides the expression of genes related to hypoxia, the upregulation of HIF-1α favored the transcription of genes encoding for pro-inflammatory cytokines (Cluster 4) and G protein-coupled purinoceptors (Cluster 6) in aged BM-MSCs (Figure 1D), while stem cell differentiation-related and cartilage development transcripts were significantly decreased (Cluster 2). The analysis of the regulon enrichment based on gene expression of transcription factor targets confirmed the increased activity of HIF-1α and the decreased activity of SOX9 in the presence of DMOG (30 μM; Figure 1E).

Overall, these results suggest that DMOG-induced stabilization of HIF-1α may contribute to hamper the chondrogenic differentiation of aged BM-MSCs.

### DMOG-Induced HIF-1α Stabilization Downregulates NTPDase1 and Ecto-5′-nucleotidase/CD73 Immunoreactivity in Chondrogenic-Differentiating BM-MSCs Isolated from *Pm* Women

Previous studies from our group showed that the extracellular breakdown of ATP and adenosine formation is impaired in aged *Pm* BM-MSCs, thus limiting the osteogenic differentiation capability of these cells.^33^ This prompted us to explore whether the same occurred when these cells were challenged by chondrogenic differentiating conditions, given the putative role of these purines in the osteochondral unit homeostasis.^4^

Human BM-MSCs undergoing osteogenic differentiation exhibit immunoreactivity against ecto-NTPDase1, −2 and −3, and ecto-5′-nucleotidase/CD73 on the plasma membrane; the density of these nucleotide-metabolizing enzymes typically increases upon differentiation of the cells into mature osteoblasts.^16,33^ Figure 2 shows that chondrogenic-differentiating BM-MSCs also express all of these enzymes, yet their relative abundance slightly differs from that of the osteogenic differentiating lineage. While osteogenic-differentiating BM-MSCs exhibit high levels of NTPDase1 and −3 at all differentiation stages, the NTPDase2 immunoreactivity only becomes evident in mature osteoblasts.^16^ A distinct pattern was evident in chondrogenic-differentiating BM-MSCs, given that at early culture stages (day 7) the cells predominantly express NTPDase2 (ATPase or CD39L1; EC 3.6.1.3), followed by NTPDase3 and NTPDase1, with very small amounts of NTPDase8 (Figure 2A). In contrast to that observed in osteogenic differentiating cells, NTPDase2 (but also NTPDase3) was downregulated (*P* < 0.05) during the chondrogenic differentiation of the cells from culture day 7 to 14 (Figure 2B). Typically, pluripotent BM-MSCs exhibit immunoreactivity against ecto-5′-nucleotidase/CD73, yet while the density of this adenosine-forming enzyme progressively increases to become a hallmark of osteoprogenitor cells–see e.g.,^34^ its level remained roughly constant in cells cultured with the chondrogenic differentiation cocktail (Figure 2A,B).

**Figure 2:**
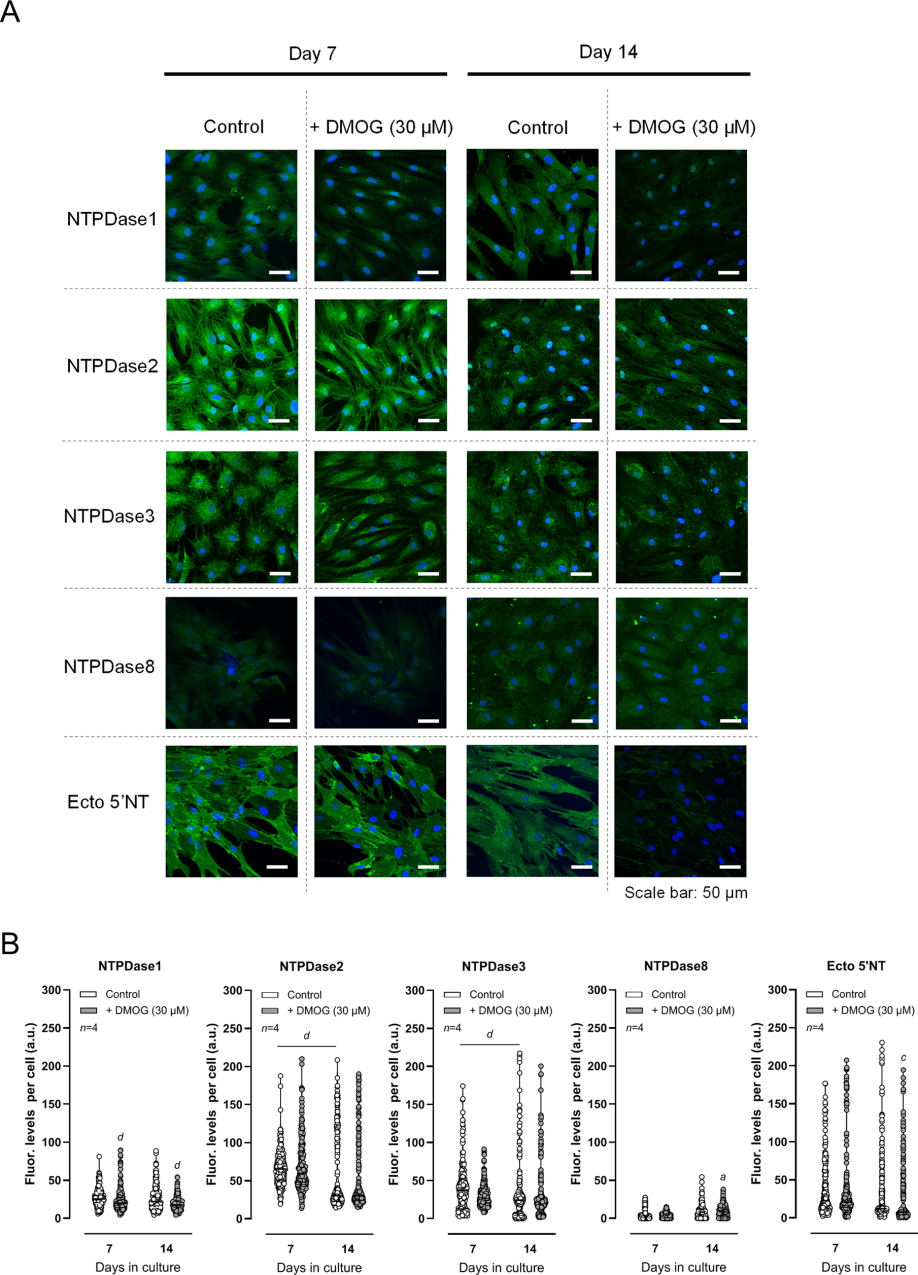
Changes in the pattern of ecto-nucleotidase protein amounts in chondrogenic-differentiating BM-MSCs from *Pm* women upon exposure to the prolyl hydroxylase inhibitor, DMOG. Panel A shows representative images of the immunocytochemical detection of NTPDase1, −2, −3, −8 and ecto-5′-nucleotidase/CD73 (ecto-5′NT). Shown is the time-related immunoreactivity (green) detected by confocal microscopy. Blue dots represent nuclei staining with DAPI. The scale bar is 50 μm. Panel B shows immunofluorescence intensity graphs computed from confocal microscopy images acquired as in Panel A. Ordinates represent fluorescence intensity per cell (arbitrary units, a.u.) of the enzymes’ immunoreactivity as a function of the number of days in culture (day 7 and 14). A total of 134–240 cells were analyzed from four *Pm* women (84 ± 3 years old). ^*a*^ *P <*0.05, ^*c*^ *P <*0.001 and ^*d*^ *P <*0.0001 (Kruskal–Wallis test with uncorrected Dunn’s test) represent significant differences.

In the presence of DMOG (30 μM), the immunoreactivity against NTPDase1 (the enzyme that dephosphorylates ATP directly to AMP, also called CD39 or apyrase, EC 3.6.1.5), significantly (*P* < 0.05) decreased when the chondrogenic-differentiating cells were exposed to this drug for 7 and 14 days compared to nonexposed cells (Figure 2B). The upregulation of HIF-1α transcriptional activity by DMOG (30 μM) for 14 days also slightly decreased the density of the ecto-5′-nucleotidase/CD73 immunoreactivity in these cells (Figure 2B).

### DMOG-Induced HIF-1α Stabilization Decreases Both ATP-Derived Extracellular Adenosine Formation and the Amount of ENT-1 Transporters in Chondrogenic-Differentiating BM-MSCs from *Pm* Women

Figure 3 shows the kinetics of the extracellular catabolism of ATP (10 μM) and AMP (10 μM) and metabolite formation in chondrogenic-differentiating BM-MSCs from *Pm* women cultured for 7 and 14 days in the absence or presence of DMOG (30 μM). ATP (10 μM) was sequentially dephosphorylated into ADP, AMP, adenosine (ADO), inosine (INO), and hypoxanthine (HX) in chondrogenic-differentiating BM-MSCs; at the end of the 30 min incubation period, ADO was the most represented ATP metabolite. Co-localization of NTPDase1, 2, and 3 ensures the sequential dephosphorylation of ATP and ADP directly to AMP, and justifies the appearance of small amounts of ADP in culture media. Despite the downregulation of NTPDase2 and 3 in chondrogenic differentiating cells (culture day 7 vs 14; see Figure 2B), such modifications are probably not enough to influence the kinetics of the extracellular ATP breakdown and metabolites formation (Figure 3A–D). The negligible accumulation of AMP, INO, and HX in the cultures also sustains the idea that pluripotent human BM-MSCs possess high adenosine-forming capability from AMP dephosphorylation, via ecto-5′-nucleotidase/CD73, but low adenosine deaminase (ADA) activity. This feature ensures fast AMP dephosphorylation and ADO accumulation, as the main extracellular ATP metabolite.^20,33^

**Figure 3:**
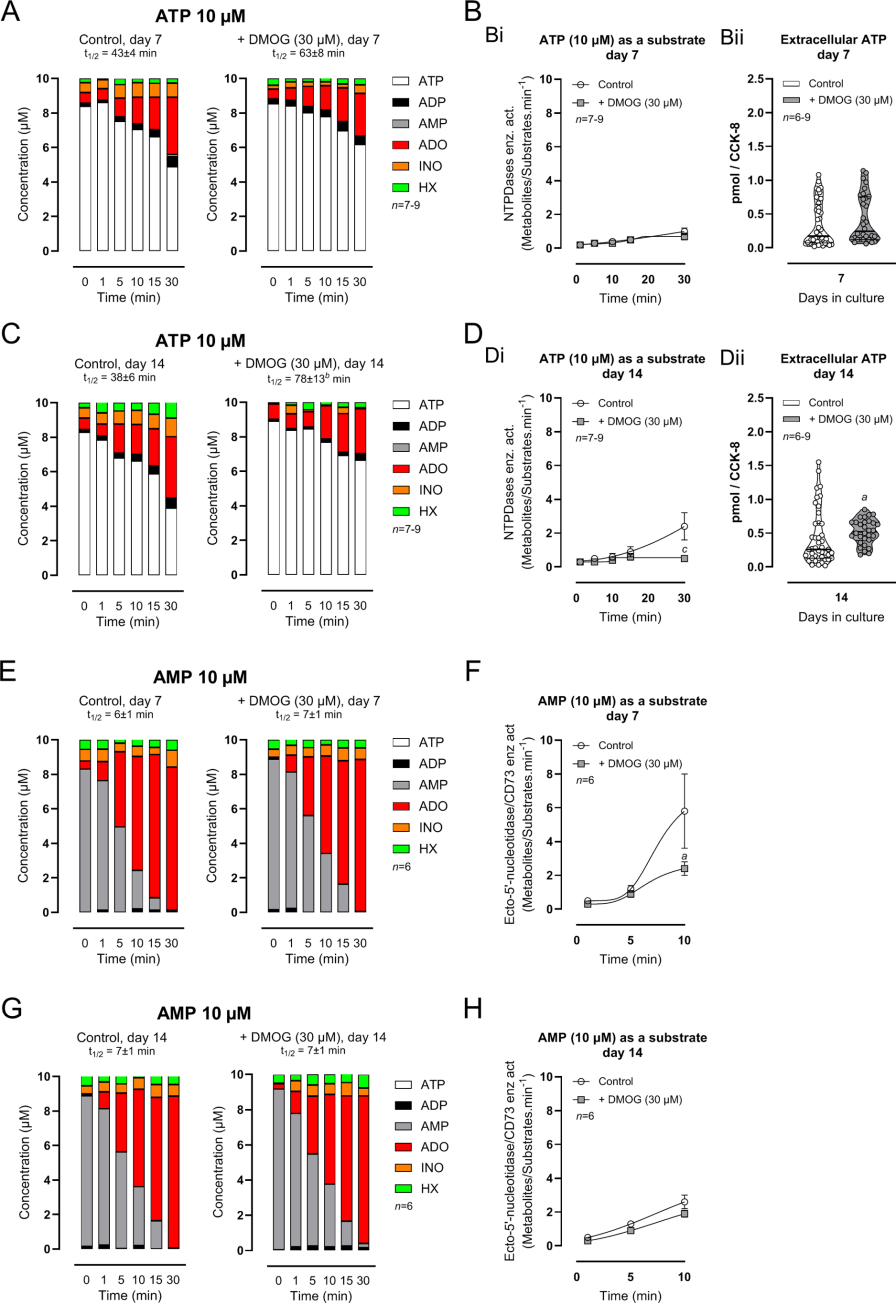
Upregulation of HIF-1α transcriptional activity attenuates adenosine formation from the extracellular catabolism of ATP (Panels A and C) and AMP (Panels E and G) in chondrogenic-differentiating BM-MSCs from *Pm* women grown in culture for 7 and 14 days. ATP (10 μM) and AMP (10 μM) were added to the incubation medium (Tyrode’s solution) at time zero to BM-MSCs cultures exposed or not to DMOG (30 μM), for 7 and 14 days. 75-μL samples were collected at the indicated times for HPLC analysis to quantify the kinetics of substrates disappearance and formation of metabolites. ATP (white bar), ADP (black), AMP (gray bar), adenosine (ADO, red), inosine (INO, orange), hypoxanthine (HX, green). The half-life time (*t*_1/2_) for each initial substrate is shown for comparison. Panels Bi and Di represent the enzymatic activity of NTPDases; panels F and H represent the enzymatic activity of ecto-5′-nucleotidase/CD73. Values were obtained by quantifying the ratio [Metabolites]:[Substrates] per min from seven to nine *Pm* women (74 ± 4 years old); cells from each individual were tested in duplicate. In panels Bii and Dii, ordinates represent the extracellular ATP accumulation (pmol/CCK-8) during 30 min in Tyrode’s solution (no drugs); values were corrected for cell viability/proliferation (CCK-8 assay). The ATP content of the samples was measured using the luciferin-luciferase bioluminescence assay (75 μL/well; see Materials and Methods for details). Dots in the violin plots are triplicates from six to nine *Pm* women (72 ± 4 years old). ^*a*^ *P <*0.05, ^*b*^ *P <*0.01 and ^*c*^ *P <*0.001 (Kruskal–Wallis test with uncorrected Dunn’s test) represent significant differences.

Exposure of chondrogenic-differentiating BM-MSCs to DMOG (30 μM) increased the half-life of extracellular ATP from 43 ± 4 to 63 ± 8 min and from 38 ± 6 to 78 ± 13 min at culture days 7 and 14, respectively (Figure 3A,C). Downregulation of NTPDase1 in chondrogenic-differentiating BM-MSCs treated with DMOG (30 μM) functionally translates into a reduction in the ATP metabolizing capacity and AMP formation by these cells, which was more evident at 14 days of cell culture (Figure 3Di). At this time point, we found that the incubation medium collected from DMOG (30 μM)-treated chondrogenic-differentiating BM-MSCs had higher (*P* < 0.05) levels of ATP compared to nontreated cells, providing that proliferation/viability of the cells were normalized by CCK-8 values and the incubation time was kept at 30 min (Figure 3Dii).

Using AMP (10 μM) as the substrate of ecto-5′-nucleotidase/CD73, we found that treatment of chondrogenic-differentiating BM-MSCs with DMOG (30 μM) resulted in the reduction of ADO formation at culture day 7 compared with nontreated cells; ADO amounts remained constant thereafter (Figure 3E–H). This finding may be explained since DMOG (30 μM)-induced a partial loss of ecto-5′-nucleotidase/CD73 in the plasma membrane of chondrogenic-differentiating BM-MSCs (see Figure 2A,B). Moreover, high extracellular ATP accumulation can feed-forwardly inhibit ecto-5′-nucleotidase/CD73 by binding to its catalytic site and, thus, further reduce ADO formation from AMP in the cultures^35,36^ (see Figure 3B,D).

Besides ADO originating from the extracellular catabolism of adenine nucleotides, the nucleoside is released as such from human BM-MSCs, via a dipyridamole-sensitive type 1 equilibrative nucleoside transporter (ENT-1).^20^ This ADO release path may be susceptible to modulation by hypoxia,^37^ yet no data is available regarding the effect of HIF-1α stabilization caused by DMOG on ENT-1 amounts in chondrogenic-differentiating BM-MSCs. Here, we used the intrinsic fluorescent photophysical properties of dipyridamole (an ENT-1 inhibitor) to tag the ENT-1 transporter on the plasma membrane of these cells.^38^ Supplementary Figure 3A shows that ENT-1 transporters fluorescently labeled with dipyridamole (0.5 μM; excitation overlap with DAPI at 405 nm) were significantly (*P* < 0.05) downregulated in chondrogenic-differentiating BM-MSCs exposed to DMOG (30 μM) for 7 and 14 days compared to nonexposed cells. Using a nonfluorescent ENT-1 inhibitor, S-(4-nitrobenzyl)-6-thioinosine (NBTI, 50 μM), we discarded any effect of ENT-1 inhibition on cell proliferation/viability (Supplementary Figure 3B). To further validate this finding, ENT-1 was labeled using a specific antibody, and immunoreactivity analysis revealed a significant decline in ENT-1 expression in DMOG-treated cells when compared to controls on both culture days 7 and 14 (Supplementary Figure 3C).

### Density of Adenosine A_2A_ Receptors Overcomes That of the Chondroprotective A_2B_ Subtype in DMOG-Treated Chondrogenic-differentiating BM-MSCs from *Pm* Women

ADO-sensitive receptors are present in chondrocytes and chondrocyte-like cells isolated from rodents and bovine^39–41^ ; however, much less information is available concerning the expression and function of ADO receptor subtypes in human cells undergoing chondrogenic differentiation.^18^ Nonetheless, increasing evidence points toward endogenous ADO accumulation resulting from the extracellular catabolism of adenine nucleotides, via ecto-5′-nucleotidase/CD73, as a major determinant of osteogenic differentiation of human BM-MSCs through activation of the most abundant low-affinity A_2B_ receptor subtype compared to the colocalized high-affinity A_2A_ receptor.^16,20,33^

Using immunofluorescence confocal microscopy, we show in Figure 4 (panels A and B) that both A_2A_ and A_2B_ receptor subtypes are expressed evenly in chondrogenic-differentiating human BM-MSCs at all culture stages (7 and 14 days; Figure 4B). Treatment with DMOG (30 μM) transiently downregulated (*P* < 0.05) the A_2B_ receptor density at culture day 7, while disproportionally increasing the A_2A_ receptor amount when this drug treatment was prolonged to 14 days (Figure 4A,B). Overall, DMOG (30 μM) exposure favors A_2A_ receptors’ overexpression in human BM-MSCs undergoing chondrogenic differentiation, leading to an increase in the A_2A_ versus A_2B_ receptor tone, whose functional impact may be strengthened given the higher affinity of the nucleoside for the A_2A_ receptor (Figure 4B).

**Figure 4:**
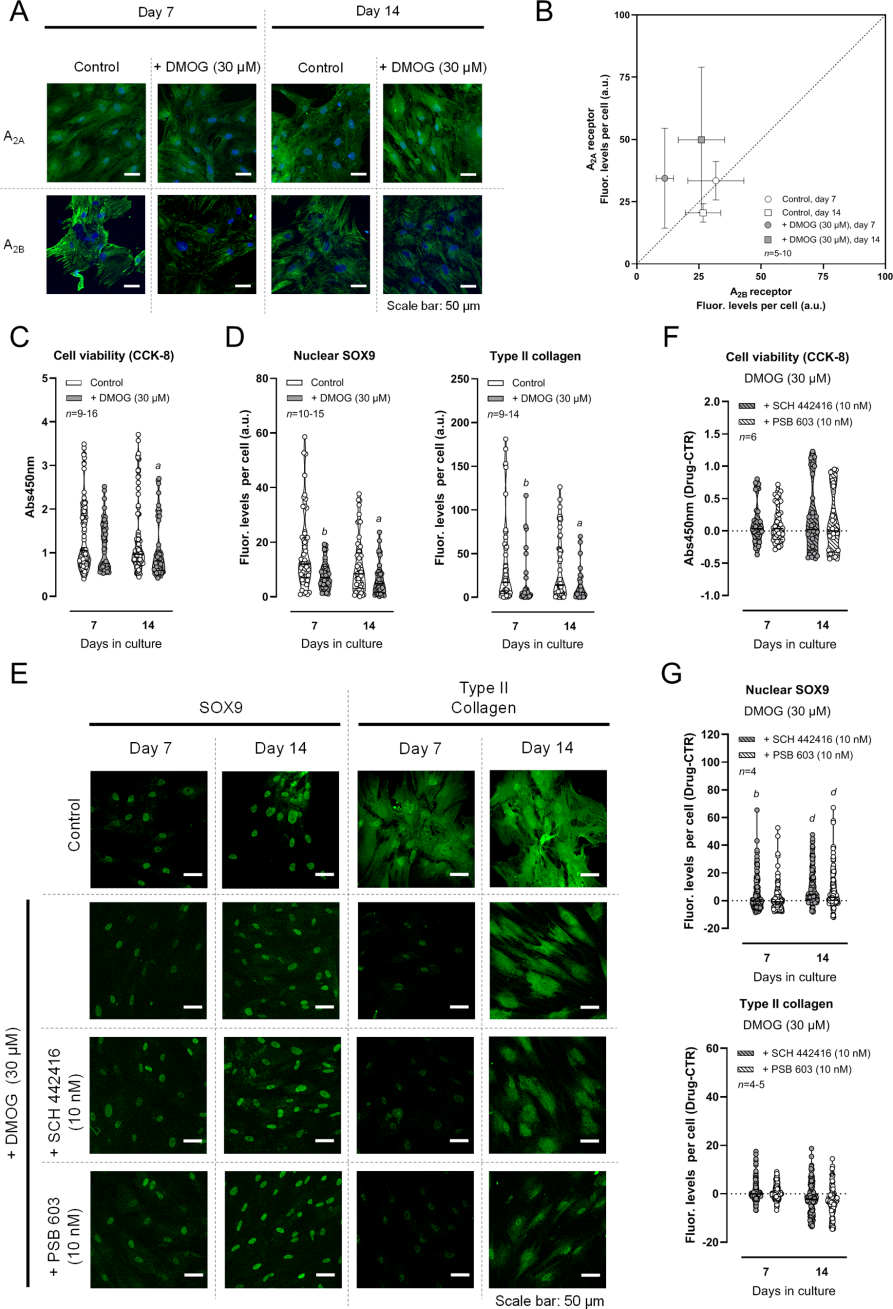
Upregulation of HIF-1α transcriptional activity promotes the tonic activation of high-affinity A_2A_ adenosine receptors in human BM-MSCs undergoing chondrogenic differentiation. Chondrogenic-differentiated BM-MSCs were allowed to grow for 7 and 14 days in the absence or presence of DMOG (30 μM) and/or of selective adenosine A_2A_ (SCH 442416, 10 nM) and A_2B_ (PSB 603, 10 nM) receptor antagonists. Panel A shows representative images of the immunocytochemical detection (green) of the indicated adenosine-sensitive receptors by confocal microscopy. Blue dots represent nuclei staining with DAPI. The scale bar is 50 μm. Panel B shows the relative immunoreactivity of adenosine A_2A_ (ordinates) and A_2B_ (abscissas) receptors in chondrogenic-differentiating BM-MSCs in control conditions and after exposure to DMOG (30 μM) for 7 and 14 days. The dashed line represents equal immunoreactivity for both receptors. Dots represent mean ± SEM. A total of 166–424 cells were analyzed from five to ten *Pm* women (76 ± 3 years old). Panel C represents cell viability/proliferation data using the CCK-8 assay; ordinates represent absorbance values measured at 450 nm. Truncated violin plots represent pooled data from nine to 16 *Pm* women (75 ± 2 years old); four to eight replicates were made per individual. Panel D shows the immunofluorescence intensity graphs computed from acquired confocal microscopy images stained against nuclear SOX9 and type II collagen chondrogenic markers. Ordinates represent fluorescence intensity per cell (arbitrary units, a.u.). A total of 340–729 cells were analyzed from nine to 15 *Pm* women (75 ± 3 years old). Panel E shows representative micrographs of the immunocytochemical detection (green) of nuclear SOX9 and type II collagen by confocal microscopy at all aforementioned experimental conditions. The scale bar is 50 μm. Panels F and G show data from the cell viability/proliferation CCK-8 assay (F) and immunofluorescence confocal microscopy to detect nuclear SOX9 and type II collagen (G). Zero represents the identity between CCK-8 values, nuclear SOX9 and type II collagen levels in the absence and presence of the DMOG (30 μM) (horizontal dashed line). A total of 160–458 cells were analyzed from four to six *Pm* women (80 ± 5 years old). ^*a*^ *P <*0.05, ^*b*^ *P <*0.01, ^*c*^ *P <*0.001, and ^*d*^ *P <*0.0001 (Kruskal–Wallis test with uncorrected Dunn’s test) represent significant differences.

Besides DMOG (30 μM)-induced upregulation of genes, like *MMP13* and *COL10A1,* that are detrimental to cartilage^31,32^ (Figure 1B), we show here that it also significantly (*P* < 0.05) attenuated the nuclear translocation of SOX9, a transcription factor that is essential for early chondrogenic differentiation of BM-MSC. This finding occurred along with decreases in cell proliferation/viability and type II collagen production (a late chondrogenic marker comprising 90% of the collagen in hyaline articular cartilage^42^) at all culture stages in the presence of DMOG (30 μM) (Figure 4C–E).

DMOG (30 μM)-induced inhibition of SOX9 nuclear translocation was mimicked by the selective A_2A_ receptor agonist, CGS21680 (10 nM, *n* = 5), and by inhibition of ATP breakdown into adenosine by NTPDases with ARL67156 (50 μM, *n* = 5) in chondrogenic-differentiating BM-MSCs allowed to grow in culture for 7 days (data not shown). These preliminary findings strengthen the hypothesis that HIF-1α stabilization favors the tonic activation of high-affinity A_2A_ receptors by low adenosine amounts. Moreover, selective blockage of the A_2A_ receptor with SCH 442416 (10 nM) partially rehabilitated the nuclear translocation of SOX9 in chondrogenically differentiating BM-MSCs exposed to DMOG (30 μM) at all evaluated time points (culture days 7 and 14; Figure 4E, G). SCH 442416 (10 nM) did not change proliferation/viability (Figure 4F) and the production of the late chondrogenic marker, type II collagen, by these cells (Figure 4E,G; see also Supplementary Figure 4). The selective A_2B_ receptor antagonist, PSB 603 (10 nM), had only a minor blocking effect on the DMOG (30 μM)-induced decrease of the nuclear translocation of SOX9 when BM-MSCs were cultured for 14 days in the chondrogenic-enrichment medium, without repercussions on the other evaluated parameters (Figure 4F,G). Blockage of the low-affinity A_2B_ receptor with PSB 603 (10 nM) had only a minor decreasing effect on type II collagen production when the cells were exposed for 14 days to the chondrogenic-inducing medium without DMOG (30 μM; Supplementary Figure 4).

## Discussion

The articular cartilage is an avascular tissue and, thus, evolves under low oxygen tension conditions; this microenvironment may become harsher after trauma and during chronic inflammatory conditions (e.g., osteoarthritis).^4,43^ The HIF transcription factor is the master regulator of cellular responses to hypoxia.^44,45^ HIF is a heterodimer formed by tunable HIF-α subunits (1α, 2α, and 3α) and constitutively expressed HIF-1β (also known as aryl hydrocarbon receptor nuclear translocator or ARNT).^46,47^ Upon activation by hypoxia (low O_2_), HIF-1α resists to degradation and translocates to the nucleus, where it dimerizes with HIF-1β,^48^ and this complex induces the expression of several transcriptional targets by binding to hypoxia-responsive elements (HRE).^44,45^ Stabilization of HIF-1α may also be achieved in normoxia by exposure of the cells to the α-ketoglutarate analogue, DMOG.^29,30^ This compound competitively inhibits HIF-1α hydroxylation by prolyl hydroxylase (PHD), among other minor effects on NF-kβ, TNF-α, AMPK, or histone demethylase systems.^49–54^

Besides the characteristic hypoxic microenvironment of articular cartilage, further increases in the endogenous levels of HIF-1α are frequent in osteoarthritic joints.^55^ Interestingly, the RNAseq analysis performed in DMOG-treated chondrogenic-differentiating BM-MSCs in this study showed significant increases in the expression of pro-inflammatory cytokines (*e.g., IL-18, TNF*-α, *IFN, IL-1*β; see Supplementary Tables 1 and 3). Overexpression of chondrocyte hypertrophy maturation markers, such as *VEGFA*, *MMP13*, and *COL10A1*,^31,32^ was also observed in DMOG-treated cells (Supplementary Table 1). As expected, exposure of BM-MSCs to DMOG dramatically increased HIF-1α transcriptional activity, which negatively correlated with the transcription of early chondrogenic gene markers, such as SOX9 (Figure 1E). The translocation of the SOX9 transcription factor to the nucleus was also diminished in DMOG-treated chondrogenic-differentiating BM-MSCs, and these cells produced less type II collagen. Overall, these findings suggest that DMOG-induced persistent upregulation of HIF-1α transcriptional activity might be detrimental to chondrogenesis and may eventually result in progressive cartilage deterioration in osteoarthritic joints.^43,56^ This is, however, paradoxical considering that the putative promotion of autophagy by DMOG should be chondroprotective,^57–59^ thus prompting the investigation of alternative hypotheses.

The influence of hypoxia on the purinergic signaling cascade in cartilage and bone is scarce, and the underlying mechanisms are controversial. Inspired on available data from other cell systems, we hypothesized that stabilization of HIF-1α by persistent hypoxia (or DMOG under normoxic conditions) could differentially affect the expression and activity of ATP metabolizing and ADO forming enzymes in osteogenic- and chondrogenic-differentiating BM-MSCs.^4^ In fact, the intracellular stabilization of HIF-1α transcriptional activity is associated with NTPDase1/CD39 and/or ecto-5′-nucleotidase/CD73 overexpression in human endothelial cells,^60^ gastric carcinoma cells,^60,61^ human metastatic colon carcinoma T84 cells, and HepaRG and Chang liver cell lines.^62^ NTPDase1/CD39 gene transcription and protein synthesis were also upregulated ex vivo by exposure of human microvascular epithelial cells (HMEC-1) to hypoxia for 8 and 24 h; a similar outcome was obtained in the ischemic myocardium of C57BL/6 mice upon treatment with DMOG.^61^ However, these findings are not consensual, since chronic exposure to hypoxia delayed the extracellular breakdown of ATP by fibroblasts and endothelial cells, thus increasing the tonic activation of ATP-sensitive receptors.^63^ Moreover, hypoxia reduced the activity of NTPDases and ecto-5′-nucleotidase/CD73 without affecting their density in endothelial cells of vasa vasorum^64^ by a mechanism associated with the production of pro-inflammatory cytokines, such as IL-1, IL-6, and TNF-α.^63,64^

Data obtained in the present study show that chondrogenic-differentiating BM-MSCs from *Pm* women are able to metabolize adenine nucleotides (e.g., ATP and AMP) and generate ADO in the extracellular milieu. This competence is also shared by BM-MSCs undergoing osteogenic differentiation, as previously demonstrated,^16,20,33,65^ but the plasma membrane density of nucleotide-metabolizing enzyme subtypes differs when the cells are exposed to chondrogenic vs osteogenic-differentiating conditions. While osteogenic-differentiating BM-MSCs show high NTPDase1 and NTPDase3 protein amounts at all differentiation stages, the NTPDase2 (ATPase or CD39L1; EC 3.6.1.3) only became evident in mature osteoblasts.^16^ This pattern is distinct from that observed in BM-MSCs undergoing chondrogenic differentiation (this study). During chondrogenic differentiation of BM-MSCs, the NTPDase2 enzyme predominates at early culture stages, followed by NTPDase3 and NTPDase1, with very small amounts of NTPDase8 being observed at culture day 7 (Figure 2A). The relative abundance of NTPDase2, an ATPase that converts ATP into ADP in chondrogenic-differentiating BM-MSCs, could anticipate a putative role of ADP-sensitive receptors in chondrogenesis.^4^ Notwithstanding this, our enzymatic kinetic experiments did not support this hypothesis, since we detected only small amounts of ADP in culture media of chondrogenic-differentiating BM-MSCs when ATP was the substrate (Figure 3). These results may be biased by the simultaneous presence of NTPDase1 and NTPDase3 isoforms in the plasma membrane of these cells (Figure 2), which may contribute to bypass ADP formation/accumulation by ATP (and ADP) dephosphorylation directly to AMP. These results agree with previous findings showing that NTPDase1 and NTPDase3 gene transcripts are upregulated in mature chondrocytes compared to undifferentiated cells.^66^ DMOG-induced extracellular ATP accumulation emphasizes a role mediated by P2 purinoceptors upon hypoxia in human chondrogenesis.^15–17^ Although the levels of P2X4 and P2Y_11_ receptor gene transcripts increase under hypoxia conditions, the influence of these receptors in the chondrogenic differentiation of aged BM-MSCs has never been explored and thus warrants further investigation.

Another important difference among osteogenic- vs chondrogenic-differentiating BM-MSCs is that the density of nucleotide-metabolizing enzymes tends to increase upon osteoblast maturation (7 < 14 day), while both NTPDase2 and NTPDase3 were downregulated during chondrogenic differentiation of the same cells (7 > 14 day). A similar feature occurred concerning the density of the ADO-forming enzyme, ecto-5′-nucleotidase/CD73, that is responsible for the extracellular catabolism of AMP. The upregulation of this enzyme is a hallmark of osteoprogenitor cells, see, e.g.^20,34^ Yet, the immunoreactivity of ecto-5′-nucleotidase/CD73 in chondrogenic-differentiating BM-MSCs remained roughly constant throughout the entire culture period (Figure 2), and the same happened considering the extracellular formation of ADO when AMP was used as a substrate (Figure 3). In fact, ADO was the most represented extracellular ATP metabolite in BM-MSCs undergoing chondrogenic differentiation. This finding is explained given that these cells exhibit high ecto-5′-nucleotidase/CD73 protein amounts and lack ADA activity, yielding very small amounts of ADO metabolites, like INO and HX.^20^

Microenvironment changes, such as variations in oxygen tension, mechanical stress, and inflammation, have a tremendous impact on the release, extracellular metabolism, and activity of purinergic signaling players involved in osteogenic and chondrogenic differentiation of BM-MSCs, making them putative candidates for pharmacological manipulation.^4,18^ In this study, we investigated whether impairment of the chondrogenic differentiation capability of aged BM-MSCs exposed to DMOG could be associated with changes in the extracellular ADO accumulation and activity, as previously predicted in other cell types (e.g., fibroblasts, endothelial cells) submitted to hypoxia.^63,64^ Data show that chondrogenic-differentiating BM-MSC cultures accumulate higher extracellular ATP amounts when exposed to DMOG and that this drug dramatically increases the HIF-1α transcriptional activity under normoxic conditions. This feature may result specifically from the downregulation of NTPDase1/CD39 in the plasma membrane of aged BM-MSCs, while the subsequent reduction of extracellular ADO accumulation may be a consequence of multiple concurring mechanisms. First, DMOG also reduced the density of the ADO-forming enzyme, ecto-5′-nucleotidase/CD73, in chondrogenic-differentiating BM-MSCs. Second, the DMOG-induced extracellular ATP accumulation may contribute to feed-forwardly inhibiting the catalytic activity of ecto-5′-nucleotidase/CD73, further reducing ADO formation from AMP dephosphorylation.^35,36^ Finally, the density of the ENT-1 nucleoside transporter, which was fluorescently tagged with dipyridamole and by a specific antibody, significantly decreased after exposure of BM-MSCs undergoing chondrogenic differentiation to DMOG, which eventually may limit the outflow of ADO from affected cells. The latter effect does not depend on changes in BM-MSCs’ viability/proliferation, because the nonfluorescent ENT-1 inhibitor, NBTI, had no measurable effect. Unfortunately, due to methodological constraints (lack of chromatographic detection limit for such small ADO amounts), we were unable to quantify the endogenous accumulation of ADO in the samples used to measure ATP through the highly sensitive luciferin-luciferase bioluminescence kit.

Modifications in ADO extracellular amounts resulting from deficits in the catabolism of released ATP, as well as in the nucleoside release via ENT-1 transporters, may be critical to determine whether ADO activates preferentially high-affinity (A_2A_), low-affinity (A_2B_), or both, receptor subtypes driving pluripotent BM-MSCs to proliferation and/or differentiation.^18,20^ In aged BM-MSCs, ADO accumulation in the extracellular fluid facilitates activation of the most abundant low-affinity A_2B_ receptor subtype to promote the osteogenic differentiation of the cells.^16,20,33^ Although ADO-sensitive receptors are present in chondrocytes and chondrocyte-like cells from rodents and bovines,^39–41^ their expression and function in human chondrocyte progenitors have been insufficiently explored.^18^ We show here that A_2A_ and A_2B_ receptor immunoreactivity is evenly distributed in human BM-MSCs undergoing chondrogenic differentiation, and it remains roughly constant at all culture stages (7 and 14 days; Figure 4B). Interestingly, the persistent transcriptional activity of HIF-1α induced by DMOG in normoxic conditions transiently downregulated the A_2B_ receptor subtype at culture day 7 and disproportionally upregulated the A_2A_ receptor in more differentiated cells, resulting from the prolongation of DMOG treatment until day 14 (Figure 4A,B). DMOG-induced overexpression of the A_2A_ receptor in chondrogenic-differentiating BM-MSCs may affect the A_2A_/A_2B_ receptors balance toward the preferential activation of high-affinity A_2A_ receptors by low ADO amounts (see above). We confirmed this trend given that one could partially rehabilitate the chondrogenesis of DMOG-exposed BM-MSCs, which was indicated by restoration of SOX9 translocation to the nucleus (an early chondrogenic marker) upon selectively blocking the A_2A_ receptor with SCH 442416 at all culture time points. Although the rehabilitation effect of SCH 442416 was only partial, since it failed to affect BM-MSCs proliferation/viability and type II collagen production (a late chondrogenic marker), selective blockage of the A_2B_ receptor with PSB 603 had even less of an effect. Indeed, SOX9 plays a pivotal role in the regulation of type II collagen gene expression by binding to its promoter during chondrogenic differentiation.^67^ Yet, no positive correlation between SOX9 and type II collagen expression has been observed in adult articular chondrocytes.^68^ This feature has been attributed to the time dynamics of chondrogenesis, where early increases in SOX9 transcriptional levels are followed (yet noncoincidently) by type II collagen production.^69–71^

Upregulation of the A_2A_ receptor transcription and activity has also been observed in endothelial cells of the human lung microvasculature (HLMVECs) and pulmonary artery (HPAECs) submitted to hypoxia, involving a mechanism related to HIF-2α transcriptional activity.^72^ However, the opposite was also verified in human umbilical vein endothelial cells (HUVECs), where hypoxia switched the A_2A_/A_2B_ receptor transcriptional ratio from a predominant A_2A_ receptor subtype under normoxia conditions to a dominant A_2B_ “angiogenic” receptor phenotype upon 3 h of hypoxia (4.6% O_2_), via a mechanism involving the expression of VEGF independently of HIF-1α.^73^ Likewise, hypoxia-induced A_2B_ receptor overexpression was also observed in human lung fibroblasts,^74^ as well as in HMEC-1 and in the colonic mucosa of mice with TNBS-induced colitis.^75^ Thus, it seems that controversial results concerning the impact of hypoxia on A_2A_/A_2B_ receptor balance may emerge depending on the experimental conditions, namely, the origin of the cells/tissue, the duration of the hypoxic stimulus, and whether the receptors are activated by the natural ADO ligand or enzymatically stable agonists. This study was designed to explore the impact of HIF-1α transcriptional activation by DMOG under normoxic conditions on the expression and function of ATP metabolizing enzymes and ADO formation in chondrogenic-differentiating BM-MSCs obtained from aged (*Pm*) women. The influence of DMOG-induced transcriptional HIF-1α activation was also assessed concerning the density and function of high-affinity A_2A_ and low-affinity A_2B_ receptors in these cells. Overall, the data suggest that DMOG-induced HIF-1α transcriptional activity hampers the chondrogenic differentiation of *Pm* BM-MSCs. The mechanism(s) underlying the impairment of the chondrogenic fate may be due to deficient extracellular ADO formation and/or release from aged BM-MSCs via the CD39/CD73 cascade and ENT-1 transporters, respectively. The functional repercussion of ADO deficiency is highlighted as a consequence of DMOG-induced disruption of the A_2A_/A_2B_ receptors equilibrium, leading to a preferential activation of the high-affinity A_2A_ receptor subtype in aged BM-MSCs from early chondrogenic commitment stages. Notwithstanding the fact that HIF-1α stabilization influences A_2A_/A_2B_ receptor expression and/or activation balance, we did not explore in this study the downstream molecular pathways responsible for their differential effects. This limitation deserves investigation in a dedicated study, also considering that both A_2A_ and A_2B_ receptors share overlapping intracellular signaling pathways and/or act as “biased” receptors (see, e.g.^76^).

Age-related changes in BM-MSCs compromise their clinical potential for both bone and cartilage regeneration. In aged women, BM-MSCs’ ability to differentiate into osteoblasts and chondrocytes may be impaired due to hormonal decline, cell senescence, and alterations in the local microenvironment, such as prolonged/exacerbated hypoxia. Aging cells acquire a senescence-associated secretory phenotype (SASP). This phenotype is characterized by the secretion of proteins, such as cytokines, chemokines, and growth factors, as well as anti-inflammatory factors and/or adenine-based purines (i.e., ATP and adenosine), which may affect the communication with neighboring cells via autocrine/paracrine mechanisms.^77^ Unfortunately, due to clinical limitations of bone marrow sampling, we were unable to evaluate the impact of HIF-1α stabilization in BM-MSCs from younger females undergoing chondrogenic differentiation. Yet, preliminary data show that the chondrogenic differentiation of younger BM-MSCs (47 ± 1 years old, n = 3) expresses higher levels of chondrogenic biomarkers, namely, SOX9 and type II collagen, compared to cells from aged women (unpublished results). Notwithstanding this limitation may deserve further research efforts, evaluation of the chondrogenic differentiation of putatively affected BM-MSCs from PM women may open new avenues to restore chondrogenesis in the population most affected by degenerative osteochondral lesions.

In summary, this study gathers compelling evidence on the interplay between HIF-1α and the purinergic signaling cascade in chondrogenic differentiation of aged human BM-MSCs while paving the way for novel therapeutic strategies to ameliorate and/or prevent osteochondral damage in osteoarthritic joints in aged *Pm* women.

## Materials and Methods

### Human Bone Marrow-Derived Mesenchymal Stem Cell (BM-MSC) Isolation and Culture

Bone marrow samples were isolated from the neck of the femur of twenty-seven female patients (75 ± 2 years old) undergoing total hip arthroplasty due to noninflammatory degenerative osteoarthrosis. Handling of bone marrow samples and cell culturing were performed as previously described.^14,33^ To avoid in vitro phenotypic changes and senescence of the cells, we limited their usage to the first subcultures. No pooled samples from different individuals were carried out under any circumstances.

Before differentiation of the cells into the desired lineages, they were plated onto plastic bottom wells at an initial density of 2.5 × 10^4^ cells/mL and allowed to expand until 80% confluence in a humidified atmosphere of 95% air plus 5% CO_2_, at 37 °C, for 15 days. The expansion medium consisted of α-minimal essential medium (α-MEM) supplemented with 10% fetal bovine serum (FBS), 100 U/mL penicillin, 100 μg/mL streptomycin, and 2.5 μg/mL amphotericin B. Plastic nonadherent cells were discarded 5 days after plating; from this time point onward, we started culture medium changes twice a week.

### Multipotent Capability of BM-MSCs and Viability/Proliferation Assays

Under the aforementioned experimental conditions, cultured cell yields were highly enriched in multipotent BM-MSCs; see, e.g.,^16^ Supplementary Figure 1 shows that plastic-adherent BM-MSCs isolated under appropriate condition media can differentiate into chondrocyte, adipocyte, and osteocyte cell lineages; see below, cf.^33,78,79^

The osteogenic differentiation of BM-MSCs was performed by incubating the cells with α-MEM supplemented with ascorbic acid (50 μg/mL), β-glycerophosphate (10 mM), and dexamethasone (10 nM) for 35 days. BM-MSCs’ osteogenic differentiation was confirmed by increases in (1) the activity of alkaline phosphatase (ALP), (2) the amount of the osterix transcription factor (Western blot analysis at 21-day cultures), and (3) the formation of mineralized bone nodules (Alizarin Red staining at 35-day cultures),^14,33^ as shown in Supplementary Figure 1A.

For adipogenic differentiation and assessment, expanded cells were allowed to grow for 21 days in Dulbecco’s modified Eagle’s medium (DMEM) – high glucose supplemented with FBS (10%), insulin (10 μL/mL), dexamethasone (100 nM), indomethacin (0.5 mM), 3-isobutyl-1-methylxantine (IBMX, 60 μM), penicillin (100 U/mL), streptomycin (100 μg/mL), and amphotericin B (2.5 μg/mL). We used Western blot analysis to assess increases in the density of the adipogenic marker, peroxisome proliferator-activated receptor γ (PPAR-γ).^80^ Identification of intracellular lipid droplets was performed at culture day 21. This was done after washing the cells three times with phosphate-buffered saline (PBS) 1×, fixation with 4% paraformaldehyde (PFA),^81^ and incubation with the Oil Red dye for 2 h. Cell documentation was made in an Olympus CKX41 microscope (Olympus, Tokyo, Japan) equipped with a digital camera (Olympus SC3, Tokyo, Japan) running the Olympus Analysis GetIT 5.1 imaging software (Olympus, Tokyo, Japan). Incubation of BM-MSCs with the adipogenic cocktail decreased their fibroblast-like morphology and favored progressive accumulation of round lipid droplets in the cytoplasm of the cells (see Supplementary Figure 1A). Increases in PPAR-γ amounts in cell homogenates also confirmed the adipogenic differentiation of cultured BM-MSCs (Supplementary Figure 1A).

For the chondrogenic differentiation, once BM-MSCs reached 80% confluence, the expansion medium was changed to a chondrogenic differentiation cocktail. The latter consisted of a DMEM - high glucose medium supplemented with FBS (2.5%), transforming growth factor-β3 (TGF-β3, 10 ng/mL), insulin-transferrin-selenium (ITS) supplement (100×, 1%), ascorbic acid (50 μg/mL), l-proline (40 μg/mL), dexamethasone (100 nM), penicillin (100 U/mL), streptomycin (100 μg/mL), and amphotericin B (2.5 μg/mL).^82,83^ To ascertain the chondrogenic differentiation of BM-MSCs, we evaluated the amount of the SOX9 transcription factor, a master regulator of chondrogenesis, in total cell homogenates by Western blot analysis at culture days 7 and 21 (Supplementary Figure 1B). We used the Toluidine Blue O assay to evaluate the cell matrix deposition of polysaccharides.^84^ To this end, the cells were fixed with 4% PFA at room temperature for 10 min. Then, the cells were washed out with sterile PBS 1× and, subsequently, exposed to 1% acetic acid for 10–15 s. Finally, the cells were stained with 1% Toluidine Blue O at room temperature for 5 min and then washed out with PBS 1× three times. Cell documentation was made in an Olympus CKX41 microscope (Olympus, Tokyo, Japan) equipped with a digital camera (Olympus SC3, Tokyo, Japan) running the Olympus Analysis GetIT 5.1 imaging software (Olympus, Tokyo, Japan). Incubation of BM-MSCs with the chondrogenic cocktail increased cell matrix deposition of polysaccharides (see Supplementary Figure 1A). Using immunofluorescence confocal microscopy at culture days 7 and 14, Supplementary Figure 1C shows parallel increases in the nuclear translocation (DNA binding) of SOX9 and type II collagen production by BM-MSCs undergoing chondrogenic differentiation compared to cells cultured in the standard expansion medium.

To monitor cell viability/proliferation, we used in this study the premixed, ready-to-use Cell Counting Kit-8 (CCK-8) high-sensitivity assay, according to the manufacturer’s instructions.^85^ Absorbance was measured at 450 nm using a Synergy HT microplate reader (RRID: SCR_020536; BioTek Instruments, Vermont, USA).

### Induction of HIF-1α Transcriptional Activity in Chondrogenic-Differentiating BM-MSCs under Normoxic Conditions

Chondrogenic-differentiating BM-MSCs were allowed to grow, for 14 days, in 95% air plus 5% CO_2_ humidified atmosphere (normoxic conditions) in the absence (control) or presence of the cell permeable competitive inhibitor of prolyl hydroxylase, dimethyloxallyl glycine (DMOG, 30 μM), which promotes stabilization and intracellular accumulation of HIF-1α protein.^29,30^ The concentration of DMOG (30 μM) was chosen as it gave the best results compared to other concentrations ranging from 10 to 300 μM appraised during the optimization protocols using chondrogenic-differentiating BM-MSCs isolated from adult female Wistar rats (5.5 ± 2.0 months old, n = 4; *Rattus norvegicus*; Charles River, RGD Cat. No. 13508588, RRID:RGD_13508588). These animals, which were euthanized for other research purposes, were used in order to reduce the number of experimental animals, as determined by the ARRIVE 2.0 guidelines. BM-MSCs were harvested by flushing rat femoral diaphysis with the expansion α-MEM medium (see above) onto culture plates, via an 18-G needle coupled to a 5 mL syringe.^86^ The procedures to induce chondrogenesis and the phenotypic characterization of rat BM-MSCs were similar to those mentioned above for the Human cells, unless otherwise stated (see Supplementary Figure 2).

In some experiments, the human BM-MSCs undergoing chondrogenic differentiation were also incubated with adenosine-modifying drugs, like selective A_2A_ and A_2B_ receptor antagonists, SCH 442416 (10 nM)^87^ and PSB 603 (10 nM),^88^ respectively, and ENT-1 transport inhibitors, namely dipyridamole (0.5 μM) or NBTI (50 μM). When used, these drugs were included in culture media from day 0 onward, and thus, they were renewed whenever changing culture media, i.e., twice a week.

### Immunofluorescence Staining and Confocal Microscopy Imaging

For imaging purposes, chondrogenic-differentiating BM-MSCs were plated and allowed to grow in glass-bottom chamber slides for 7 or 14 days under the conditions described above for induction of the HIF-1α transcriptional activity under normoxic conditions. For cell fixation, we used 4% PFA in PBS, with subsequent 1.5 h incubation with a blocking buffer I containing FBS (10%), bovine serum albumin (BSA, 1%), Triton X (0.1%), and NaN_3_ (0.05%). Fixed cells were incubated for 2 h, in the dark, with epitope-specific primary antibodies diluted in blocking buffer II with an identical composition to blocking buffer I, except concerning FBS (5%). In this study, we used HIF-1α 1:200 (ab1 [H1alpha67], mouse), SOX9 5 μg/mL (ab185966, rabbit), type II collagen 1:100 (ab34712, rabbit), ecto-5′-nucleotidase 1:1000 (h5′NT-2_L_I_5_, rabbit), NTPDase1 1:1000 (hN1–1_C_I_5_, guinea pig), NTPDase2 1:200 (hN2-Kw3I4, rabbit), NTPDase3 1:200 (hN3–1_C_I_4_, guinea pig), NTPDase8 1:1000 (hN8-C5_S_, mouse), A_2A_ receptor 1:100 (A2aR21-A, rabbit), A_2B_ receptor 1:50 (AAR-003, rabbit) and ENT-1 1:50 (sc-377283, mouse). After washout of the primary antibodies, the cells were incubated with secondary antibodies diluted in the blocking buffer II, namely Alexa Fluor 488 1:1500 (antirabbit), Alexa Fluor 568 1:1500 (antimouse), Alexa-Fluor 647 1:150 (antiguinea pig), and/or TRITC 1:150 (antiguinea pig), for 1 h in the dark. Negative controls were performed in the absence of the primary antibodies as previously described (Supplementary Figure 5).^33^ Glass slides mounted with Vectashield antifade mounting medium with DAPI (H-1200–10; Vector Laboratories, Inc.; Newark, CA 94560, USA) were stored at 4 °C until observation using an Olympus FV1000 laser-scanning confocal microscope running the FluoView Advanced Software for FV1000 (v. 4.0.3.4; Olympus, Tokyo, Japan). The microscope settings and acquisition parameters were kept unaltered during documentation to allow comparisons between the acquired micrographs. The technical details about immunofluorescence staining, confocal microscopy observation, and standardization procedures for image comparisons were extensively described in previously published papers.^14,33^ Image analyses to assess the average fluorescence intensity per cell (outlined as a region of interest, ROI) and background subtraction were made with ImageJ 1.37c software (NIH, Bethesda, MD, USA) after confocal micrograph files were imported in the Olympus Multi TIFF format (Tokyo, Japan). Manuscript figures depict typical immunofluorescence micrographs for each experimental condition.

### Quantification of Extracellular ATP and Kinetics of Adenosine Formation from Adenine Nucleotides

Measurements of extracellular ATP in culture media were performed by bioluminescence using the luciferin-luciferase ATP bioluminescence assay kit HS II (Roche Applied Science, Indianapolis, Indiana, USA) in a multidetection microplate reader (Synergy HT, BioTek Instruments, Vermont, USA), according to the manufacturer’s instructions.^33,89^ Briefly, chondrogenic-differentiating BM-MSCs cultured for 7 and 14 days in the absence or presence of DMOG (30 μM) were washed twice with gassed (95% O_2_ plus 5% CO_2_) Tyrode’s solution (137 mM NaCl, 2.7 mM KCl, 1.8 mM CaCl_2_, 1 mM MgCl_2_, 0.4 mM NaH_2_PO_4_, 11.9 mM NaHCO_3_, and 11.2 mM glucose, pH 7.4). After maintaining the cells in the gassed Tyrode’s solution for 30 min, at 37 °C, the incubation fluid was removed, snap-frozen in liquid nitrogen, and kept at −80 °C until ATP quantifications. The ATP measurements were made in duplicate against freshly prepared high-purity ATP external standards; we made two to four replicas per individual experiment. The amount of ATP per culture well (in pmol) was normalized for cell viability/proliferation using the CCK-8 result obtained in the same cell population. We found a negligible (<0.1 mU/mL) lactate dehydrogenase (LDH; EC 1.1.1.27) activity in the samples used for extracellular ATP quantification.^90^ This ensures the integrity of cells throughout the experimental period, which otherwise would complicate data interpretation due to intracellular ATP contamination of the analyzed fluid.

The time course of the extracellular catabolism of ATP and AMP and metabolites formation was evaluated by High-Performance Liquid Chromatography (HPLC) in chondrogenic-differentiating BM-MSCs cultured for 7 and 14 days in the absence or presence of DMOG (30 μM), following a methodology previously described by our group.^20,33^ In brief, after washing out the culture medium, we incubated the cells for 30 min at 37 °C with ATP or AMP (100 μM) made in gassed Tyrode’s solution (zero time). At predefined time points, we collected 75 μL samples of the incubation medium for HPLC analysis (LaChrom Elite, Merck, Frankfurt, Germany) of substrate disappearance and formation of metabolites. We plotted the variations in the concentration of substrates and the formation of metabolites as a function of time (progress curves). The parameters analyzed were the half-life time (*t*_1/2_) of the initial substrate, the kinetics of metabolite appearance, and the concentration of the substrate or any product remaining at the end of the experiment. The activity of NTPDases and ecto-5′-nucleotidase/CD73 was calculated by the [Metabolites]:[Substrate] ratio divided by the time point in minutes. Adenine nucleotides, namely ATP or AMP (100 μM), did not suffer from spontaneous degradation over time during 30 min in the absence of the cells. The incubation fluid remaining at the end of the protocol had a negligible (<0.1 mU/mL) lactate dehydrogenase (LDH; EC 1.1.1.27) activity, demonstrating that cell integrity was maintained during the whole procedure.^90^

### RNA Extraction, Reverse Transcription, and Real-Time PCR

Chondrogenic-differentiating BM-MSCs were detached from culture plates at days 7 and 14 by incubating the cells with trypsin-EDTA (0.04%) and type I collagenase (0.025%) in PBS (pH 7.4) for 10 min. The resulting cell suspensions were centrifuged (5000 rpm) for 10 min, at 4 °C; after removing the supernatants, the pellets were snap-frozen at −80 °C. The total RNA was extracted using the RNAeasy Mini kit (Qiagen, Germany) and, subsequently, quantified using a Nanodrop 1000 Spectrophotometer (RRID:SCR_016517, Thermo Fisher Scientific, USA); the RNA samples with 260/280 nm absorbance ratios of 1.5–2.0 were considered eligible for processing. The cDNA synthesis of eligible samples was performed using the Nzy First-Strand cDNA Synthesis Kit (Nzy First-Strand cDNA Synthesis Kit). Matrix metalloproteinase 13 (*MMP13*; Hs00942584_m1), type X collagen (*COL10A1*; Hs00166657_m1), and Ubiquitin C (*UBC*, reference gene; Hs00824723_m1) gene transcripts were quantified by Real-Time PCR using commercially available Taqman probes (Taqman Kits, Applied Biosystems, USA) in a Corbett Rotor Gene 600 Thermocycler (Corbett Research, UK). Each reaction was performed in triplicate per individual, and the average Ct value was used in the analysis. The relative expression was calculated using the 2^–ΔΔCT^ method.^91^

### Library Preparation for Transcriptome Sequencing (RNAseq) and Analysis

RNA sequencing libraries of human BM-MSC samples (RIN greater than or equal to 7.7) undergoing chondrogenic differentiation were prepared and sequenced by an outsourcing company, Novogene (Cambridge), using 150-bp paired-end reads on the Illumina NovaSeq 6000 platform (two to three biological replicates per group). Each sample yielded over 40 million reads. Fastq files were aligned to the GRCh38/hg38 transcriptome using HISAT2 (2.0.5)^92^ with default settings, *nd* gene-specific read counts were assigned using featureCounts (v1.5.0-p3).^93^ Differentially expressed genes (DEGs) were calculated using DESeq2.^94,95^ Differential expression was analyzed using DESeq2 (v1.20.0) for four groups, considering genes with at least ten raw counts in a minimum of three samples.^94,95^ Donor variation was controlled as a covariate. Genes with a raw *P* < 0.01 and absolute log2FoldChange >0.5 were deemed significant. Heatmaps and individual gene expression levels were represented as normalized count values and FPKM, respectively. Heatmaps were created with z-scores using heatmap2 (gplots package) and refined in the Heatmap function of ComplexHeatmap.^96,97^ Functional enrichment was analyzed for transcription factor (TF) activity with DoRothEA^98^ and for Gene Ontology (GO) using the enrichGO function in clusterProfiler.^99^

### Ethics Approval, Humans Consent To Participate and Use of Experimental Animals

Informed consent to use the biological material that would otherwise be discarded was obtained. All procedures were approved by the Ethics Committees of Centro Hospitalar de Vila Nova de Gaia–Espinho (registration n° 137/2018–2, endorsed on January 10, 2019) via the Gabinete Coordenador de Investigação/DEFI–Centro Hospitalar Universitário de Santo António (CHUdSA, registration n° 2021–002­(001-DEFI-001-CE, endorsed on September 01, 2021), and of Instituto de Ciências Biomédicas Abel Salazar (Medical School) of the University of Porto. The investigation conforms to the principles outlined in the Declaration of Helsinki. The use of animals in the DMOG optimization protocol was done for obvious ethical and practical reasons *vis-à-vis* the Human alternative. Experimental procedures and care of the animals followed the recommendations of the European Convention for the Protection of Vertebrate Animals for Experimental and Other Scientific Purposes (ETS 123), Directive 2010/63/EU, and Portuguese rules (DL 113/2013). We obtained approval for all experiments involving animals from the national authority, Direção Geral de Alimentação e Veterinária, and the ICBAS Animal Ethical Committee (No. 224/2017).

### Reagents and Antibodies

α-Minimum essential media (MEM, cat. no. 0894), adenosine (ADO; Cat. No. 9251), adenosine 5′-monophosphate (AMP) sodium salt (Cat. No. 1752), 4-[2-[[6-amino-9-(nethyl-β-D-ribofuranuronamidosyl)-9H-purin-2-yl]­amino]­ethyl] benzene propanoic acid hydrochloride (CGS21680, Cat. No. C141), amphotericin B (Cat. No. A2942), ascorbic acid (Cat. No. A4034), adenosine-5′-triphosphate (ATP, Cat. No. 7699), β-glycerophosphate (Cat. No. G9891), dipyridamole (Cat. No. 9766), and dexamethasone (Cat. No. D4902), Dulbecco’s Modified Eagle Medium (DMEM)–high glucose (Cat. No. D7777), dimethyloxallyl glycine (DMOG, Cat. No. D3695), fetal bovine serum (FBS; Cat. No. F9665), 3-isobutyl-1-methylxantine (IBMX, Cat. No. I5879), indomethacin (Cat. No. I7378), insulin (Cat. No. I9278), Insulin-transferrin-selenium x100 (Cat. No. I3146), l-Proline (A4034), phosphate buffered saline (PBS; Cat. No. D1408), Penicillin-Streptomycin (Cat. No. P0781), Transforming Growth Factor (TGF)-β3 (Cat. No. SRP3171) were supplied by Sigma-Aldrich (St. Louis, MO, USA; RRID: SCR_008988). 6-N,N-Diethyl-D-β,γ-dibromomethyleneATP trisodium salt (ARL 67156, Cat. No. 1283), 2-(2-Furanyl)-7-[3-(4-methoxyphenyl)­propyl]-7H-pyrazolo­[4,3-*e*]­[1,2,4]­triazolo­[1,5-*c*]­pyrimidin-5-amine (SCH 442416, Cat. No. 2463), 8-[4-[4-(4-chlorophenzyl)­piperazide-1-sulfonyl)­phenyl]]-1-propylxanthine (PSB 603, Cat. No. 3198) and 6-S-[(4-nitrophenyl)­methyl]-6-thioinosine (NBTI, Cat. No. 2924) were purchased from Tocris Bioscience (Bristol, United Kingdom). The stock solutions of SCH 442416, PSB 603, dipyridamole, and NBTI were prepared in dimethyl sulfoxide (DMSO; Merck, Germany); the maximal percentage of DMSO used in the cultures was 0.05% v/v, as it proved to be harmless to cells. We used distilled water or a culture medium to dissolve all other drugs. Stock solutions were stored as frozen aliquots at −20 °C.

Cell Counting Kit-8 (CCK-8; Cat. No. CK04) was purchased from Dojindo Laboratories (Tokyo, Japan). ATP bioluminescence assay kit HS II was purchased from Roche Applied Science (Indianapolis, Indiana, USA). RNAeasy Mini kit was purchased from Qiagen Pty Ltd. (Hilden, Germany; RRID:SCR_008539). NZY First Strand cDNA Synthesis kit (Cat. No. MB12501) and NZYSpeedy One-step RT-qPCR Probe Master Mix (MB40501) were purchased from NZYTech, Lda–Genes & Enzymes (Lisbon, Portugal). TaqMan probes for matrix metalloproteinase 13 (MMP13, Hs00942584_m1), type X collagen (COL10A1, Hs00166657_m1), and Ubiquitin C (UBC, Hs00824723_m1) were purchased from Thermo Fisher Scientific (MA, USA; RRID:SCR_008452).

Primary antihuman and secondary conjugated antibodies used in this work were previously validated.^16,89,100,101^ Primary antibodies included: anti-A_2A_ receptor (rabbit, Alpha Diagnostic International Cat# A2AR21-A, RRID:AB_1609239) purchased from Alpha Diagnostics International (Texas, USA), anti-A_2B_ receptor (rabbit, Alomone Laboratories Cat# AAR-003, RRID:AB_2039709) purchased from Alomone Laboratories (Jerusalem, Israel; RRID:SCR_013570). The anti-HIF-1α (mouse, Abcam catalog no. ab1, RRID:AB_296474), anti-SOX9 (rabbit, Abcam catalog no. ab185966, RRID:AB_2728660), antitype II collagen (rabbit, Abcam catalog no. ab34712, RRID:AB_731688), and anti-OSX (rabbit, Abcam catalog no. ab22552, RRID: AB_2194492) were purchased from Abcam (Cambridge, UK; RRID:SCR_012931). The anti-ENT-1 (mouse, Santa Cruz Biotechnology cat. no. sc-377283) and anti-PPAR-γ (mouse, Santa Cruz Biotechnology cat. no. sc-7273, RRID:AB_628115) were purchased from Santa Cruz Biotechnology (Texas, USA). The antihuman ecto-NTPDases and CD73 antibodies were developed and kindly supplied by JP and JS at the Centre de Recherche en Rhumatologie et Immunologie, University of Laval, Québec, QC, Canada (for additional information, see http://ectonucleotidases-ab.com); these included h5′NT-2_L_I_5_ (rabbit), hN1–1_C_I_5_ (guinea pig), hN2-kw3I4 (rabbit), hN3–1_C_I_4_ (guinea pig), and hN8-C5_S_ (mouse). The secondary antibodies Alexa Fluor 488 (antirabbit, Molecular Probes cat. no. A-21206 (also A21206), RRID:AB_2535792) and Alexa Fluor 568 (antimouse, Thermo Fisher Scientific cat. no. A10037, RRID:AB_2534013) were purchased from Molecular Probes (Invitrogen, Carlsbad, CA, USA). The Alexa Fluor 647 (antiguinea pig, Jackson ImmunoResearch Laboratories Cat# 706-605-148, RRID:AB_2340476) and Rhodamine (TRITC) (antiguinea pig, Jackson ImmunoResearch Laboratories Cat# 706-025-148, RRID:AB_2340445) were purchased from Jackson ImmunoResearch (Newmarket, Suffolk, UK).

### Presentation of Data and Statistical Analysis

Data obtained in this study was expressed as dot plots from an *n* number of individuals. Shapiro-Wilk normality test was performed, and subsequent statistical data analyses included parametric (one-way ANOVA with Fisher’s LSD test) or nonparametric (Kruskal–Wallis test with uncorrected Dunn’s test) tests, with a confidence level of 0.05, i.e., a 95% confidence interval. Significant differences were considered for *P* < 0.05. Prism 10.0.2 software (RRID: SCR_002798; GraphPad Software, CA, United States) was used for statistical data analysis.

## Supplementary Material



## Data Availability

Any additional information required that supports the findings of this study is available from the corresponding author upon reasonable request.

## Notes

Evidence of the multipotent differentiation capability of plastic-adherent MSCs obtained from the bone marrow (BM) of a postmenopausal (*Pm*) woman; experiments designed to optimize DMOG-induced upregulation of the HIF-1α transcriptional activity in BM-MSCs from adult female rats; upregulation of HIF-1α transcriptional activity reduces the amount of the equilibrative nucleoside transporter1 (ENT-1) in chondrogenic-differentiating BM-MSCs from *Pm* women; effect of the selective A_2A_ and A_2B_ receptor antagonists on human chondrogenesis under control conditions; negative controls of immunofluorescence staining using human BM-MSC cultures grown for 7 days in chondrogenic-inducing medium; RNA sequencing data – Differential expression results for all pairwise comparison; RNA sequencing data, DEGs grouped by cluster; and RNA sequencing data, gene ontology (GO) enrichment for DEGs by the cluster (PDF)

## Abbreviations

ADA: adenosine deaminase
ADO: adenosine
ADP: adenosine 5′-diphosphate
AMP: adenosine 5′-monophosphate
ATP: adenosine 5′-triphosphate
ARL 67156: 6-N,N-Diethyl-d-β,γ-dibromomethyleneATP trisodium salt
a.u.: arbitrary units
BM-MSCs: bone-marrow mesenchymal stem cells
CCK-8: Cell Counting Kit-8
CGS21680: 4-[2-[[6-amino-9-(nethyl-β-d-ribofuranuronamidosyl)-9H-purin-2-yl]­amino]­ethyl] benzene propanoic acid hydrochloride
COL10A1: type X collagen
DEGs: differentially expressed genes
DMEM: dulbecco’s modified eagle’s medium–high glucose
DMOG: dimethyloxallyl glycine
ENT-1: equilibrative nucleoside transporter 1
FBS: fetal bovine serum
GO: gene ontology
HIF: hypoxia-inducible factor
HMEC-1: human microvascular endothelial cells-1
HPLC: high-performance liquid chromatography
HRE: hypoxia responsive element
HX: hypoxanthine
INO: inosine
MMP-13: matrix metalloproteinase 13
NBTI: 4-nitrobenzylthioinosine
PBS: dulbecco’s phosphate buffered Saline
PFA: paraformaldehyde
PHD: prolyl-4-hydroxylase
*Pm*: postmenopausal
PSB 603: 8-[4-[4-(4-Chlorophenzyl)­piperazide-1-sulfonyl)­phenyl]]-1-propylxanthine
ROI: region of interest
SCH 442416: 2-(2-Furanyl)-7-[3-(4-methoxyphenyl)­propyl]-7H-pyrazolo­[4,3-*e*]­[1,2,4]­triazolo­[1,5-*c*]­pyrimidin-5-amine
SOX9: sex-determining region Y (SYR) box transcription factor 9
α-MEM: α-minimal essential medium
