# Supplementary material for: HIF-1α Stabilization Hampers the Chondrogenic Differentiation of Aged Bone Marrow Mesenchymal Stem Cells by Adenosine A2A/A2B Receptors Imbalance
Source: ACS Pharmacol Transl Sci. 2025 Jun 24;8(7):2075–92. doi: 10.1021/acsptsci.5c00190 (PMC12261222; doi:10.1021/acsptsci.5c00190)
Supplement: Supplementary file 1 [file pt5c00190_si_001.pdf]

Supporting Information

HIF-1α stabilisation hampers the chondrogenic differentiation of aged bone marrow mesenchymal stem cells by adenosine A_{2A}/A_{2B} receptors imbalance

Rui Pinto-Cardoso[1,2], Catarina Bessa-Andrês[1,2], Flávio Pereira-Costa[1,2], Rafael Neiva[1,2], Ricardo Martins-Ferreira[3,4], Bárbara Guerra-Leal[3,4], Diogo Nóbrega Catelas[5], Adélio Vilaça[5], António Oliveira[5], Julie Pelletier[6], Jean Sévigny[6] José Bernardo Noronha-Matos[1,2], & Paulo Correia-de-Sá[1,2*]

[1]Laboratório de Farmacologia e Neurobiologia, [2]Center for Drug Discovery and Innovative Medicines (MedInUP)/RISE-Health: Health Research Network, [3]Laboratório de Imunogenética, Departamento de Patologia e Imunologia Molecular, and [4]Unit for Multidisciplinary Research in Biomedicine (UMIB)/ITR – Laboratory for Integrative and Translational Research in Population Health, Instituto de Ciências Biomédicas Abel Salazar - Universidade do Porto (ICBAS-UP), 4050-313, Porto, Portugal; [5]Serviço de Ortopedia, Centro Hospitalar Universitário de Santo António, 4099-001, Porto, Portugal; and [6]Centre de Recherche en Rhumatologie et Immunologie, University Laval, 2325, rue de l'Université Québec (Québec) G1V 0A6, Canada.

Correspondence to (*): Paulo Correia-de-Sá, MD, PhD. E-mail address: farmacol@icbas.up.pt; ORCID: 0000-0002-6114-9189.

Laboratório de Farmacologia e Neurobiologia / MedInUP / RISE-Health, Instituto de Ciências Biomédicas de Abel Salazar (ICBAS) – Universidade do Porto (UP), Jorge Viterbo Ferreira Street, 228, 4050-313 Porto, Portugal. Tel.: 351-220428212; Fax: 351-220428090.

Supplementary Figure 1 – Pinto-Cardoso et al., 2025

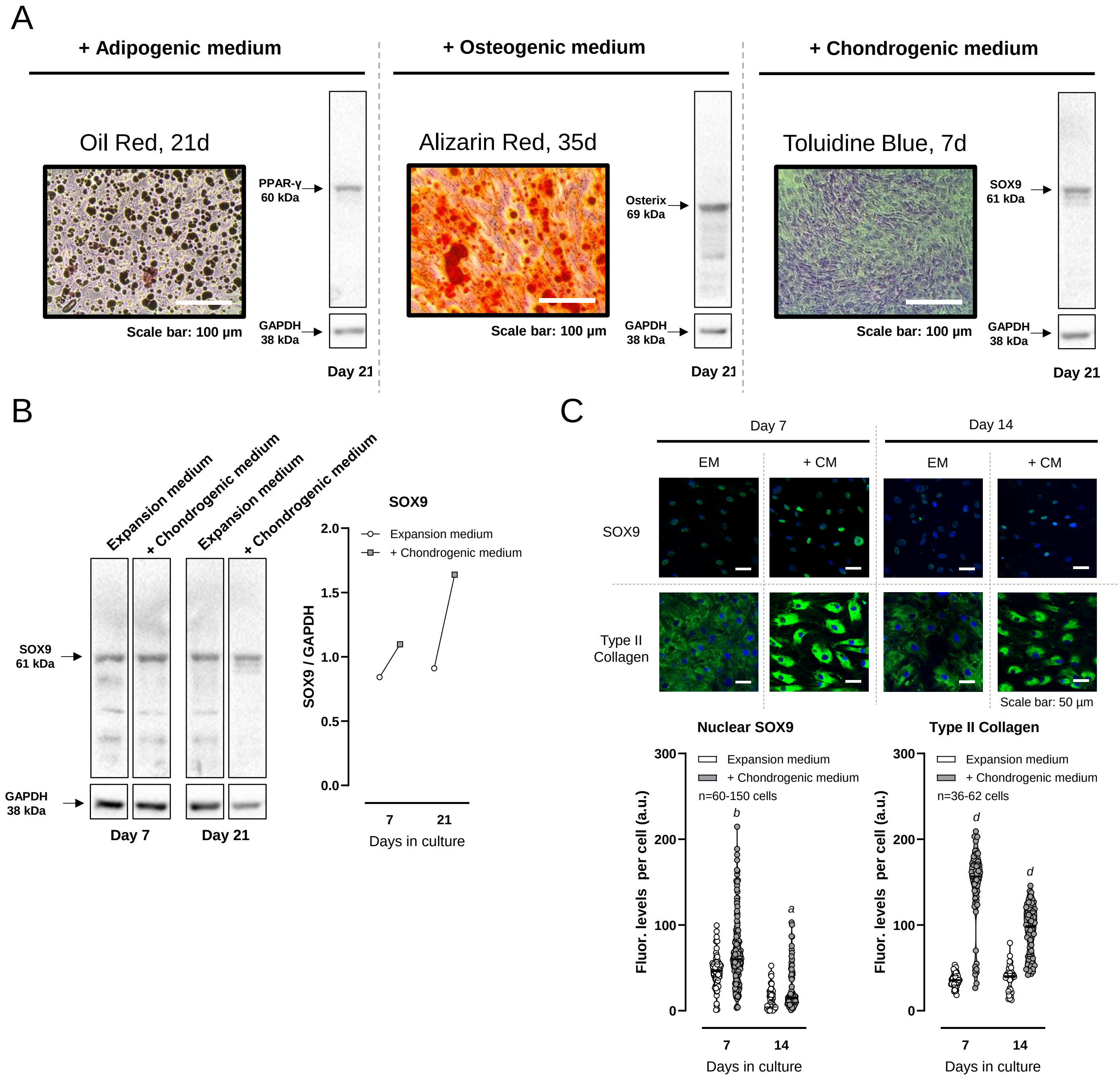

Supplementary Figure 1 – Evidence of the multipotent differentiation capability of plastic-adherent MSCs obtained from the bone marrow (BM) of a post-menopausal (Pm) woman (75-year-old). The cells were allowed to grow and differentiate using the adipogenic, osteogenic and chondrogenic cocktails for 7, 14, and/or 21 days. **Panel A** shows microscopic pictures of lipid droplets (oil red assay, day 21), matrix mineralization (alizarin red assay, day 35) and polysaccharide deposition (toluidine blue assay, day 7) pending the use of adipogenic, osteogenic and chondrogenic cocktails, respectively. The scale bar is 100 µm. Moreover, increases in the relative protein density of PPAR-γ (60 kDa), Osterix, (69 kDa) and SOX9 (61 kDa) transcription factor biomarkers of adipogenesis, osteogenesis and chondrogenesis were also obtained using lineage-specific differentiation cocktails; data was normalized by GAPDH (39 kDa) protein amounts. **Panel B** depicts immunoblots showing increases in the amounts of the SOX9 transcription factor (61 kDa) in BM-MSCs from a Pm 75-year-old woman treated with the chondrogenic-inducing medium compared to the incubation with expansion cocktail, both at culture day 7 and 21; GAPDH (39 kDa) immunoreactivity was used as normalizer. **Panel C** shows representative confocal micrographs of SOX9 and type II collagen in BM-MSCs from a Pm women grown in culture for 7 and 14 days in the presence of the expansion medium (EM) or the chondrogenic-differentiation (CM) cocktail. Blue dots represent nuclei staining with DAPI. Scale bar is 50 µm. Box and whiskers plots show the immunofluorescence intensity computed from acquired confocal microscopy images. Ordinates represent fluorescence intensity per cell (arbitrary units, a.u.) of the chondrogenic markers, SOX9 and type II collagen, obtained in 7- and 14-day cultures. A total of 36-150 cells were analysed from two to three Pm women (71±2 years old). ^{a}P<0.05, ^{b}P<0.01 and ^{d}P<0.0001 (Kruskal-Wallis test with uncorrected Dunn's test) represent significant differences.

Supplementary Figure 2 – Pinto-Cardoso et al., 2025

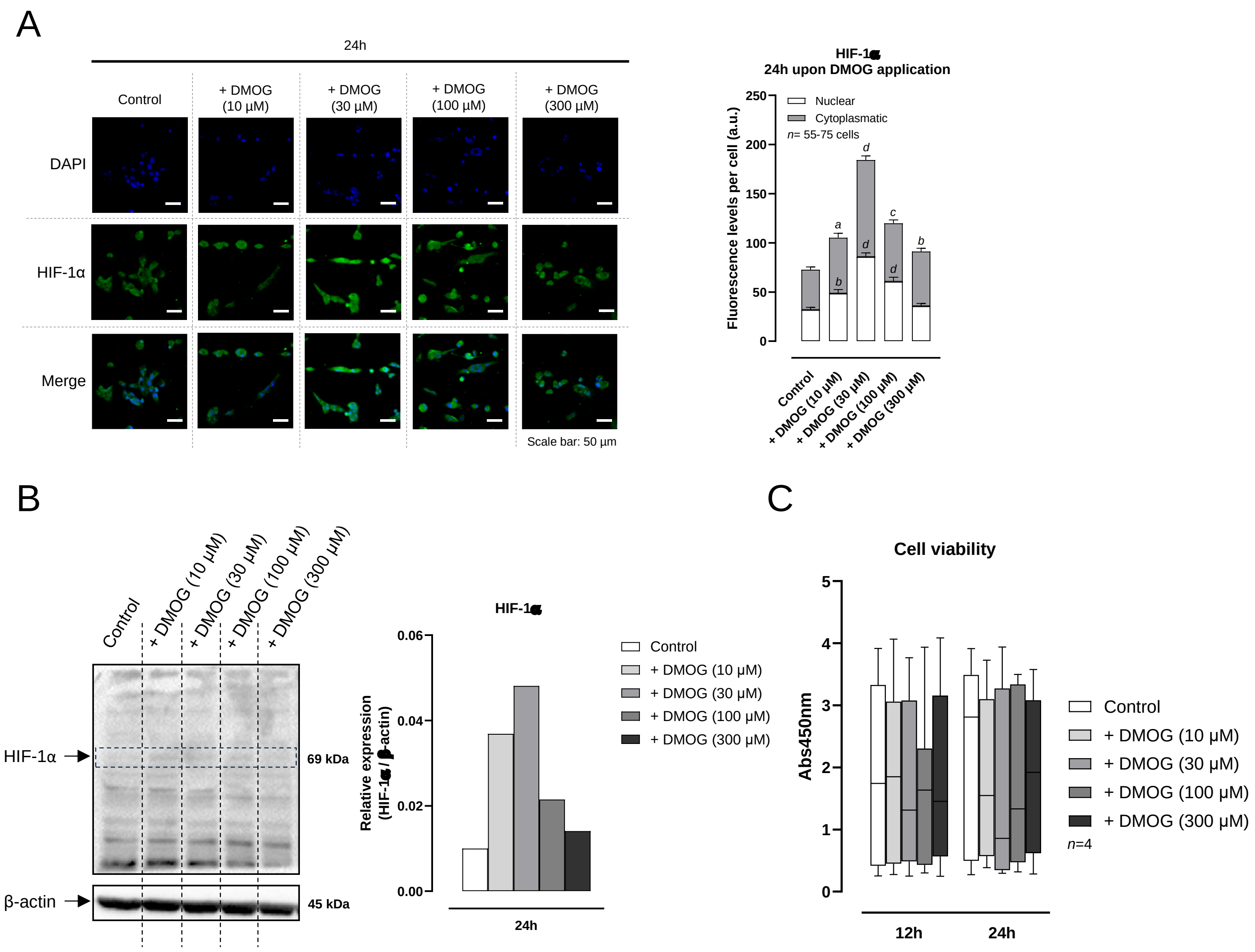

Supplementary Figure 2 – Experiments designed to optimize DMOG-induced upregulation of the HIF-1α transcriptional activity in BM-MSCs from adult female rats. Chondrogenic-differentiating rat BM-MSCs grew for 24 h in culture in either absence (control) or presence of DMOG used in concentrations ranging from 10 to 300 µM. **Panel A** shows confocal micrographs illustrating the immunocytochemical detection (green) of HIF-1α protein density in cultured BM-MSCs from a 3-mounth old female Wistar rat. Blue dots represent nuclei stained with DAPI. The scale bar is 50 µm. The right-hand side graph shows the computed average HIF-1α immunofluorescence intensity (ordinates, arbitrary units, a.u.) in the nucleus (white bars) and cytoplasm (grey bars) of cultured rat BM-MSCs. We analysed 55-75 cells from four animals. The vertical bars represent mean±S.E.M. $^{a}P<0.05$, $^{b}P<0.01$, $^{c}P<0.001$ and $^{d}P<0.0001$ (Kruskal-Wallis test with uncorrected Dunn's test) represent significant differences as compared to control values. **Panel B** shows a typical uncropped immunoblot stained against the HIF-1α protein (69 kDa) from BM-MSC homogenates of a 3-month-old female rat; β-actin (45 kDa) protein amounts were used as housekeeping protein for normalization purposes. The relative amount of HIF-1α corrected by the β-actin content of cell homogenates treated with increasing concentrations of DMOG is shown the right hand-side graph (grey bars). **Panel C** shows that increasing concentrations of DMOG (10-300 µM) did not affect the viability and/or proliferation of BM-MSCs when applied for 12 to 24 h, as determined by the CCK-8 assay; ordinates represent absorbance values measured at 450 nm. Boxes and whiskers represent pooled data from four female Wistar rat (5.5±2 months old); four replicates were made per individual experiment.

Supplementary Figure 3 – Pinto-Cardoso et al., 2025

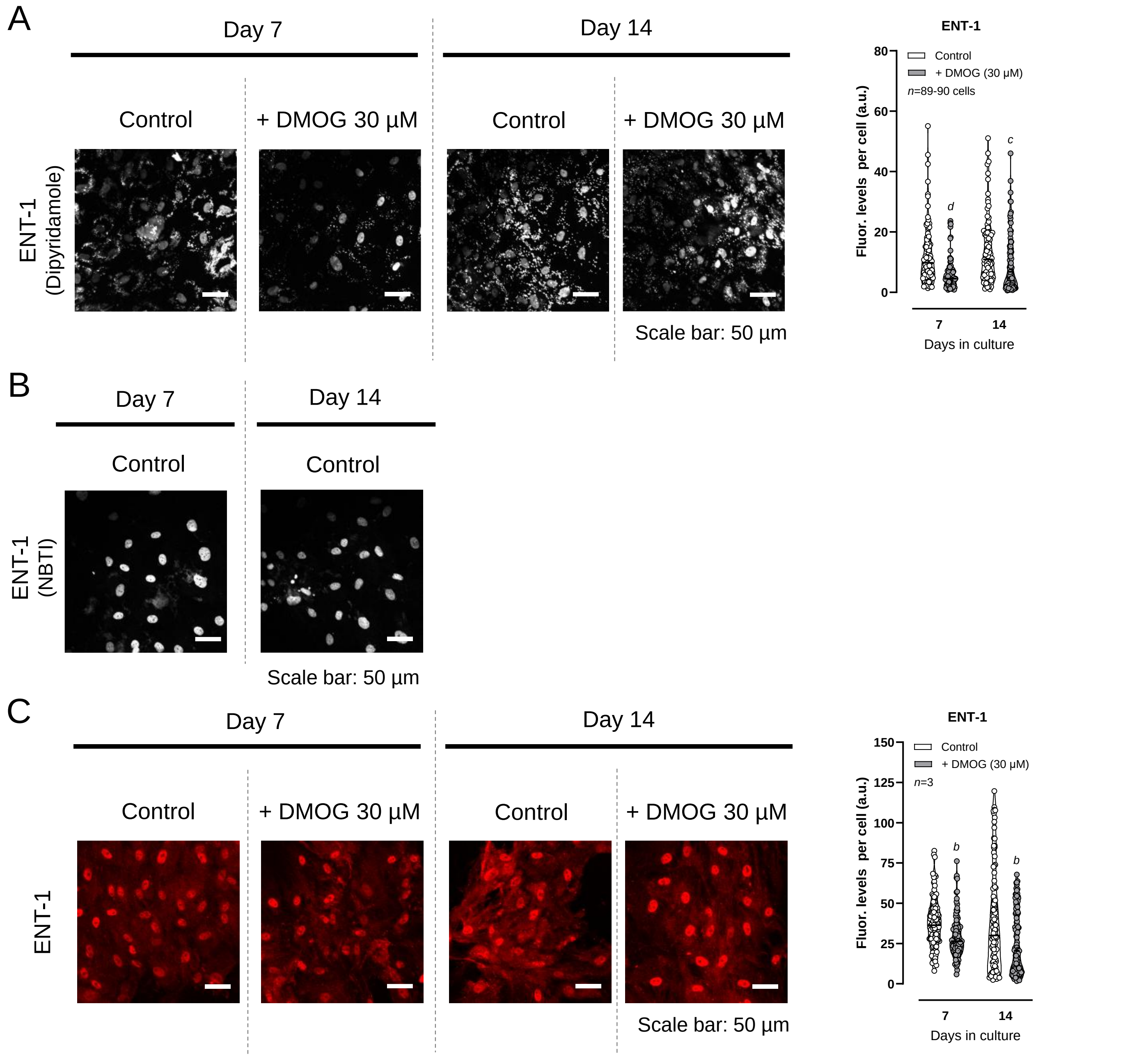

Supplementary Figure 3 – Upregulation of HIF-1α transcriptional activity reduces the amount of the equilibrative nucleoside transporter 1 (ENT-1) in chondrogenic-differentiating BM-MSCs from Pm women. **Panel A** shows representative confocal images of BM-MSCs fluorescently tagged with dipyridamole (0.5 µM). Exposure to DMOG (30 µM) significantly decreased the density of ENT1 transporters in the plasma membrane. Please note that the absorption spectra of dipyridamole at physiological pH (7.4) overlaps with that of DAPI at 405 nm and, therefore, the white dots in cells centre denotes nuclei stained with DAPI contained in the Vectashield® antifade mounting medium (H-1200-10; Vector Laboratories, Inc.). Scale bar is 50 µm. The right-hand side graphs show the obtained fluorescence intensity computed from acquired confocal microscopy images. Ordinates represent fluorescence intensity per cell (arbitrary units, a.u.) of ENT-1 upon background (no cells) and nuclear DAPI fluorescence being subtracted from all ROIs. A total of 89-90 cells were analysed. $^{c}P<0.001$ and $^{d}P<0.0001$ (Kruskal-Wallis test with uncorrected Dunn's test) represent significant differences. **Panel B** shows representative confocal images of BM-MSCs incubated with NBTI (50 µM), instead of dipyridamole (0.5 µM); all other experimental conditions were alike. White dots in the cells centre represent nuclei staining with DAPI. Scale bar is 50 µm. **Panel C** shows representative images of the immunocytochemical detection of the adenosine transporter ENT-1 (red staining) in cultured BM-MSCs undergoing chondrogenic differentiation in the absence and in the presence of DMOG (30 µM). The scale bar is 50 µm. The right-hand side graphs show the ENT-1 immunofluorescence intensity computed from confocal microscopy images. Ordinates represent fluorescence intensity per cell (arbitrary units, a.u.) of ENT-1 immunoreactivity as a function of the number of days in culture (day 7 and 14). A total of 91-109 cells were analysed from three Pm women (86±5 years old). $^{a}P<0.05$ and $^{b}P<0.01$ (Kruskal-Wallis test with uncorrected Dunn's test) represent significant differences.

Supplementary Figure 4 – Pinto-Cardoso et al., 2025

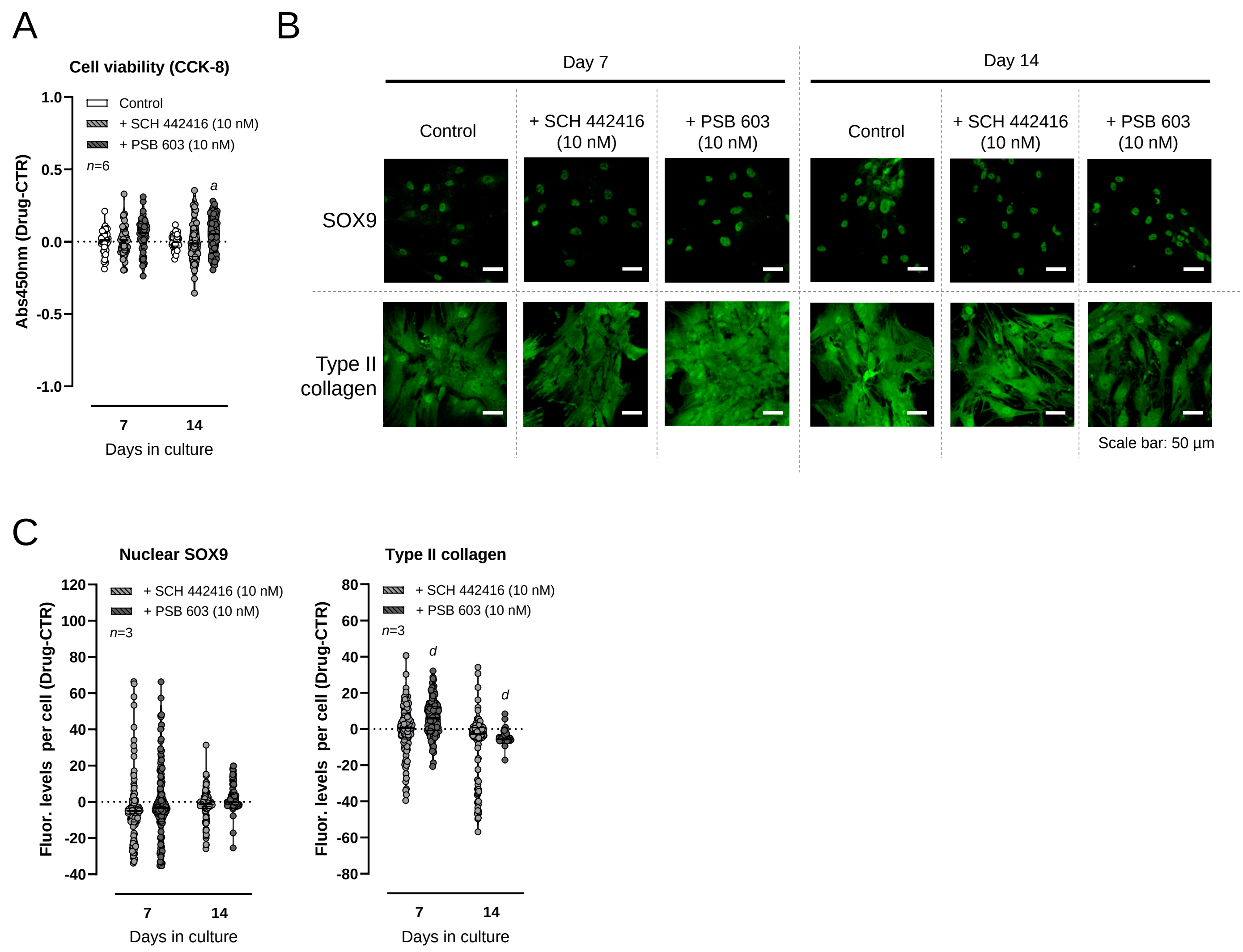

Supplementary Figure 4 – The effect of the selective A_{2A} and A_{2B} receptor antagonists on human chondrogenesis under control conditions. Human BM-MSCs were allowed to grow for 7 and 14 days of culture in a chondrogenic medium in the absence or presence of selective A_{2A} (SCH 442416, 10 nM) and A_{2B} (PSB 603, 10 nM) adenosine receptor antagonists. **Panel A** represents cell viability/proliferation data using the CCK-8 assay; ordinates represent absorbance values measured at 450 nm. Truncated violin plots represent pooled data from six Pm women (70±4 years old); four to eight replicates were made per individual. **Panel B** shows representative images of the immunocytochemical detection (green) of nuclear SOX9 and type II collagen under control conditions for 7 and 14 days in the absence or presence of selective adenosine receptor antagonists. The scale bar is 50 µm. **Panel C** shows the immunofluorescence intensity graphs computed from acquired confocal microscopy images stained against chondrogenic markers, nuclear SOX9 and type II collagen. Ordinates represent fluorescence intensity per cell (arbitrary units, a.u.). Zero represents the identity between CCK-8 values, nuclear SOX9 and type II collagen levels in the absence and presence of the selective adenosine receptor antagonists (horizontal dashed line). A total of 99-130 cells were analysed from three Pm women (72±5 years old). $^{a}P<0.05$ (Kruskal-Wallis test with uncorrected Dunn's test) represent significant differences.

Supplementary Figure 5 – Pinto-Cardoso et al., 2025

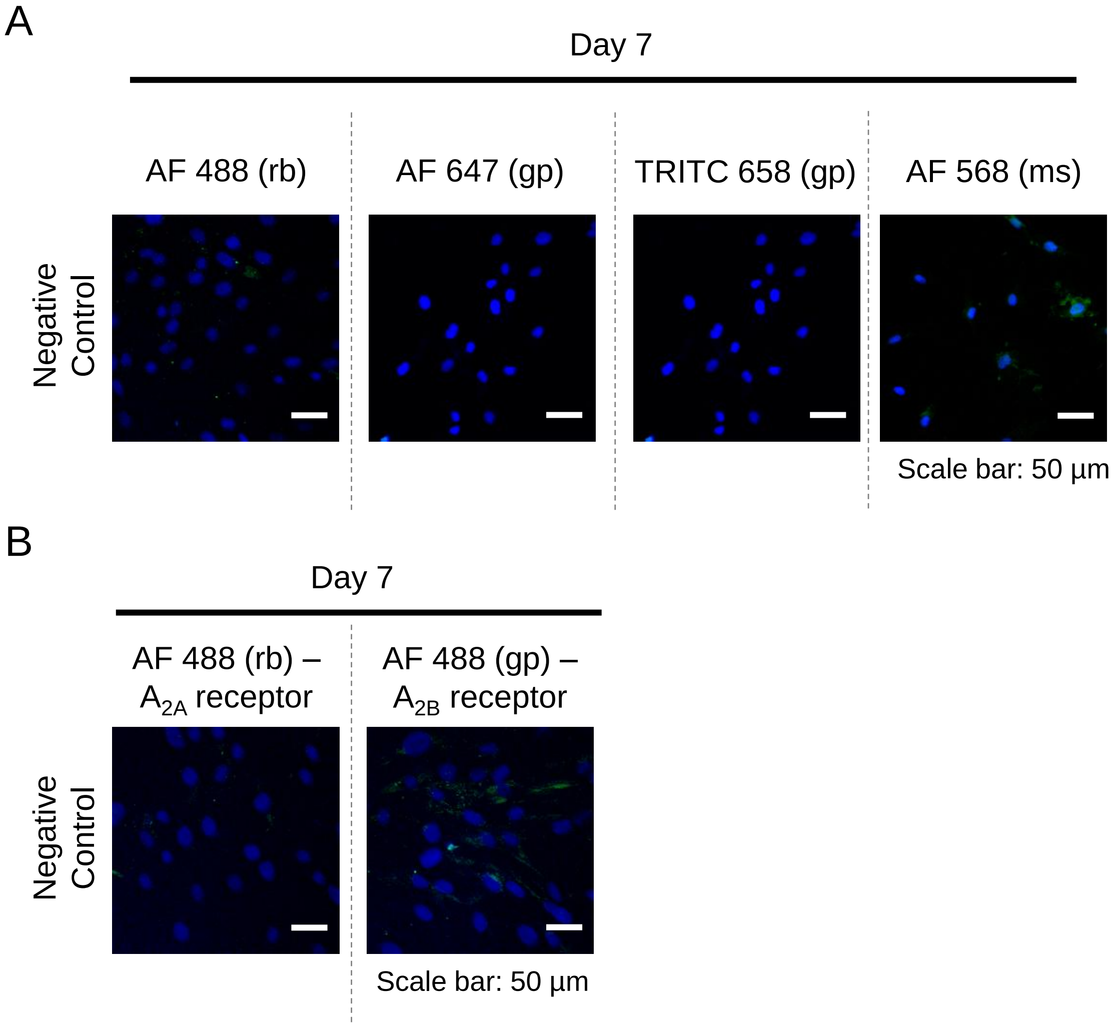

Supplementary Figure 5 – Negative controls of immunofluorescence staining using human BM-MSC cultures allowed growing for 7 days in a chondrogenic-inducing medium (first subculture). **Panel A and B** shows the immunofluorescence staining detected in cells incubated with the secondary antibodies, but where primary antibodies were omitted, for ectonucleotidases (**Panel A**) and A_{2A} and A_{2B} receptors (**Panel B**). Blue dots represent nuclei stained with DAPI contained in the VectaShield mounting medium. The scale bar is 50 µm. gp, guinea pig; ms, mouse; rb, rabbit.

Supplemental table 1: Differential expression results for all pairwise comparison

CTR_d14 vs CTR_d7

baseMean	log2FoldChange	lfcSE	stat	pvalue	padj	gene_id	gene_name
836617.341	0.080946176	0.475128431	0.170366937	0.864721572	0.999467288	ENSG00000115414	FN1
607209.4415	-0.197632717	0.977843936	-0.202110695	0.839830187	0.999467288	ENSG00000108821	COL1A1
377493.6585	-0.056257973	0.210152462	-0.267700755	0.788929669	0.999467288	ENSG00000164692	COL1A2
276619.4416	-0.432291254	0.520111928	-0.831150434	0.405888652	0.990045328	ENSG00000120708	TGFBI
221928.4399	-0.291021927	0.202531082	-1.436924761	0.150739373	0.936396974	ENSG00000113140	SPARC
211230.9357	0.724853604	1.207588381	0.600248905	0.548340365	0.99483168	ENSG00000198804	MT-CO1
148280.5096	-0.095975444	0.272607189	-0.35206498	0.724789531	0.999456245	ENSG00000141753	IGFBP4
138468.9625	0.484756679	0.335346675	1.445538947	0.148306561	0.936396974	ENSG00000137801	THBS1
117801.0114	-0.413992512	0.247611222	-1.671945676	0.094535035	0.921147998	ENSG00000156508	EEF1A1
118430.1866	-0.32902484	0.309260008	-1.063910081	0.287369436	0.965284229	ENSG00000168542	COL3A1
115574.4082	-0.028850239	0.971091837	-0.029709074	0.976299075	0.999527751	ENSG00000146674	IGFBP3
112693.5513	1.024011929	0.447605844	2.287753708	0.022151868	0.744905651	ENSG00000198886	MT-ND4
103486.6261	-0.472482281	0.403738311	-1.170268632	0.241892881	0.962465655	ENSG00000133110	POSTN
106699.4452	-0.144332329	0.463007532	-0.311727821	0.755247382	0.999467288	ENSG00000105664	COMP
97763.92112	0.305853015	0.350818659	0.871826535	0.383303016	0.985655313	ENSG00000210082	MT-RNR2
91610.65186	-0.155808086	0.233230247	-0.66804408	0.504105455	0.993513219	ENSG00000075624	ACTB
83506.4108	0.856832755	0.406231618	2.109222222	0.034925405	0.784512596	ENSG00000198712	MT-CO2
74009.85526	0.319508995	0.748686004	0.426759673	0.669554382	0.997568624	ENSG00000106366	SERPINE1
71158.15676	0.713351435	0.432765123	1.648357035	0.099279427	0.921147998	ENSG00000198938	MT-CO3
68770.8487	-0.048531189	0.22291587	-0.217710785	0.82765446	0.999467288	ENSG00000184009	ACTG1
65040.01421	0.985573824	0.40845799	2.412913562	0.015825572	0.696728876	ENSG00000198727	MT-CYB
63307.6516	0.060968797	0.358015409	0.170296572	0.864776907	0.999467288	ENSG00000144810	COL8A1
61510.12721	0.006510091	0.196152225	0.033188971	0.973523893	0.999527751	ENSG00000163430	FSTL1
58724.66708	-0.689604453	0.315903436	-2.182959648	0.029038782	0.773201806	ENSG00000087086	FTL
57129.01918	-0.233222267	0.217851565	-1.070555852	0.284369183	0.963310634	ENSG00000196924	FLNA
56096.11109	0.060401306	0.246840438	0.244697776	0.80669045	0.999467288	ENSG00000060718	COL11A1
56743.08966	-0.274690869	0.809657893	-0.339267822	0.734407981	0.999467288	ENSG00000130635	COL5A1
54810.87725	0.175912901	0.751970225	0.233935992	0.815034671	0.999467288	ENSG00000134333	LDHA
53292.75387	0.090767393	0.33487642	0.27104743	0.786354553	0.999467288	ENSG00000111799	COL12A1
51918.76701	-0.388460706	0.264492583	-1.468701699	0.141913716	0.935644597	ENSG00000182492	BGN
50980.594	-0.177740802	0.723866907	-0.245543484	0.806035643	0.999467288	ENSG00000111640	GAPDH
52425.69871	0.804874833	0.42165119	1.908864135	0.056279621	0.847598083	ENSG00000198786	MT-ND5
50476.64981	0.968370054	0.461417922	2.09868323	0.035844834	0.785058983	ENSG00000198899	MT-ATP6
48792.28758	-0.064392113	0.197057279	-0.326768507	0.743842981	0.999467288	ENSG00000087245	MMP2
50560.21165	0.325856036	0.434988402	0.74911431	0.453788311	0.99135833	ENSG00000163359	COL6A3
48464.61863	0.384838615	0.248242983	1.550249723	0.12108159	0.923922314	ENSG00000118523	CTGF
46133.68263	-0.20669687	0.258672376	-0.799068202	0.424250867	0.991301167	ENSG00000122786	CALD1
46304.07993	-0.103206219	0.278740525	-0.37025911	0.711189438	0.99900742	ENSG00000166033	HTRA1
45389.13007	-0.351117806	0.281094853	-1.249107914	0.211625606	0.954310328	ENSG00000187498	COL4A1
41790.81693	0.080160432	0.227666197	0.352096326	0.724766024	0.999456245	ENSG00000164733	CTSB
41726.45627	0.122013074	0.315285434	0.386992424	0.698761817	0.998869317	ENSG00000183087	GAS6
41783.49206	-0.207653099	0.937699711	-0.221449464	0.824742481	0.999467288	ENSG00000100234	TIMP3
41820.9389	-0.252204916	0.348319889	-0.724061196	0.469028166	0.99135833	ENSG00000150687	PRSS23
41894.64371	0.738450837	0.253171618	2.916799458	0.003536431	0.425638902	ENSG00000011465	DCN
39170.92811	-0.213561105	0.291620982	-0.73232421	0.463970706	0.99135833	ENSG00000186340	THBS2
38111.68277	-0.306179298	0.233955616	-1.308706764	0.1906337	0.949094286	ENSG00000167460	TPM4
36184.82522	0.033540894	0.278292666	0.120523816	0.904068215	0.999527751	ENSG00000182718	ANXA2
36316.05462	-0.315513817	0.227378821	-1.387613042	0.165254908	0.943223078	ENSG00000142173	COL6A2
35856.89208	-0.347610952	0.220411314	-1.577101217	0.114772237	0.923922314	ENSG00000137809	ITGA11
35024.25571	-0.214512535	0.379935647	-0.564602285	0.572344311	0.99483168	ENSG00000166147	FBN1
34194.66747	0.079274949	0.239101856	0.331553047	0.740226779	0.999467288	ENSG00000128595	CALU
33296.79179	0.310854848	0.521377007	0.596218943	0.551028973	0.99483168	ENSG00000139329	LUM
33004.13867	-0.366100775	0.320802555	-1.141202802	0.253785539	0.962465655	ENSG00000134871	COL4A2
32829.04842	-0.036278281	0.206796702	-0.175429689	0.860742014	0.999467288	ENSG00000150093	ITGB1
32782.32368	0.152887257	0.478300877	0.319646617	0.749236231	0.999467288	ENSG00000074800	ENO1
32069.82685	0.271484934	0.455264379	0.596323688	0.550959009	0.99483168	ENSG00000135919	SERPINE2
31986.37454	-0.540115937	0.367756862	-1.468676708	0.141920498	0.935644597	ENSG00000167996	FTH1

32366.78973	1.013823898	0.372129273	2.724386318	0.00644211	0.516907781	ENSG00000198763	MT-ND2
31841.52048	-0.141651972	0.209988796	-0.674569191	0.499949513	0.993513219	ENSG00000100345	MYH9
31372.31377	-0.03552743	0.275792057	-0.128819627	0.897500375	0.999467288	ENSG00000067225	PKM
30670.96394	0.481640871	0.304816559	1.580100742	0.114083797	0.923922314	ENSG00000026025	VIM
29548.26541	-0.31743991	0.356716019	-0.889895303	0.373522106	0.982521685	ENSG00000161638	ITGA5
30259.14507	-0.460444178	0.478502092	-0.962261578	0.335918224	0.977691382	ENSG00000049540	ELN
28336.76926	-0.72516923	0.429885446	-1.686889466	0.091624608	0.916034901	ENSG00000107796	ACTA2
28409.42277	0.529915024	0.504265544	1.050865026	0.293320585	0.970706863	ENSG00000152952	PLOD2
27652.73637	-0.837231974	0.336518037	-2.487926	0.012849047	0.667111125	ENSG00000149591	TAGLN
27389.22967	-0.290554917	0.265845888	-1.092944937	0.274417977	0.962465655	ENSG00000197746	PSAP
27392.96074	-0.36019757	0.383991047	-0.938036376	0.348225717	0.977691382	ENSG00000117984	CTSD
26584.13069	0.180241375	0.276544495	0.651762659	0.514554294	0.993513219	ENSG00000166923	GREM1
26355.51285	-0.778706566	0.318605897	-2.444105942	0.014521162	0.686410066	ENSG00000101335	MYL9
26472.09916	-0.622290221	0.290375751	-2.143051614	0.032108954	0.775963115	ENSG00000125868	DSTN
26260.76334	-0.027559957	0.195403546	-0.14104123	0.887837371	0.999467288	ENSG00000204262	COL5A2
25783.96577	-0.481921143	0.310010582	-1.554531269	0.120057744	0.923922314	ENSG00000140545	MFGE8
26116.77625	0.164679511	0.358634559	0.459184724	0.646101519	0.997368416	ENSG00000123384	LRP1
25571.05347	0.027336152	0.240837205	0.113504687	0.909630447	0.999527751	ENSG00000164176	EDIL3
25777.4986	-0.281758194	0.30120596	-0.935433659	0.349564857	0.977691382	ENSG00000168036	CTNNB1
25525.12132	-0.556104845	0.234572249	-2.370718823	0.017753531	0.726053908	ENSG00000167658	EEF2
24587.69524	-0.146862024	0.291928504	-0.503075313	0.614911322	0.99483168	ENSG00000166710	B2M
24710.73282	0.485452081	0.50114284	0.968690045	0.332699861	0.976026345	ENSG00000113739	STC2
24360.54709	0.396945659	0.477049314	0.83208517	0.405360872	0.990045328	ENSG00000113083	LOX
23070.33227	-0.242526628	0.171588457	-1.41342041	0.157532148	0.939464191	ENSG00000142156	COL6A1
23191.05697	0.872979441	0.393977128	2.21581249	0.026704347	0.765090753	ENSG00000198888	MT-ND1
22248.48224	0.16308273	0.265358127	0.614575976	0.538834784	0.99483168	ENSG00000125148	MT2A
22717.93142	0.660322922	0.404831564	1.631105329	0.102868096	0.92169898	ENSG00000198695	MT-ND6
21979.71542	0.131538018	0.224040208	0.587117906	0.557124523	0.99483168	ENSG00000082781	ITGB5
22264.58879	-0.126923088	0.312149971	-0.40660932	0.684294951	0.998869317	ENSG00000119681	LTBP2
21719.95951	-0.055354507	0.210161514	-0.263390315	0.792249755	0.999467288	ENSG00000087460	GNAS
21577.88254	-0.227067804	0.289626309	-0.784002683	0.433038536	0.991301167	ENSG00000163453	IGFBP7
21634.67525	0.09386416	0.307289383	0.305458519	0.760016958	0.999467288	ENSG00000152661	GJA1
21632.12089	-0.2054809	0.23585577	-0.87121422	0.383637197	0.985655313	ENSG00000087116	ADAMTS2
21394.2198	-0.401216248	0.209311685	-1.916836353	0.055258725	0.847598083	ENSG00000072110	ACTN1
21198.02628	-0.544713637	0.21064405	-2.585943615	0.009711282	0.626160891	ENSG00000140416	TPM1
21142.30039	-0.450511109	0.239849175	-1.878310017	0.060338767	0.858996918	ENSG00000089157	RPLP0
20308.85716	0.178293139	0.285479501	0.6245392	0.532273535	0.99483168	ENSG00000038427	VCAN
19460.72559	-0.155374827	0.231089156	-0.672358797	0.501355307	0.993513219	ENSG00000149257	SERPINH1
19139.8078	0.008693904	0.289927977	0.029986426	0.976077878	0.999527751	ENSG00000185624	P4HB
18390.19484	-0.76502598	0.224987184	-3.400309144	0.000673097	0.195790358	ENSG00000106211	HSPB1
18766.80717	0.021378622	0.237736556	0.089925682	0.928346273	0.999527751	ENSG00000169504	CLIC4
18318.67559	-0.204309558	0.783448782	-0.260782278	0.794260412	0.999467288	ENSG00000100644	HIF1A
18407.38737	0.340093804	0.210204322	1.617920132	0.105679811	0.92169898	ENSG00000138061	CYP1B1
18405.39355	0.358163159	0.223492263	1.602575205	0.109028472	0.923922314	ENSG00000115310	RTN4
18211.84073	-0.343667499	0.227366679	-1.511512155	0.130658014	0.929373153	ENSG00000133112	TPT1
17259.75467	-0.029134702	0.204108796	-0.142741041	0.886494702	0.999467288	ENSG00000035862	TIMP2
17825.14095	-0.281156545	0.787513896	-0.357017885	0.721078417	0.99900742	ENSG00000134013	LOXL2
16817.20538	-0.158115426	0.868541593	-0.18204704	0.85554581	0.999467288	ENSG00000102144	PGK1
16192.88485	-0.701118344	0.36720548	-1.909335188	0.056218866	0.847598083	ENSG00000198467	TPM2
16497.9693	0.327913624	0.286136995	1.14600219	0.251794246	0.962465655	ENSG00000134294	SLC38A2
16197.7282	-0.295275191	0.41164694	-0.717302045	0.473187746	0.99135833	ENSG00000044574	HSPA5
15907.28784	-0.247134118	0.282508044	-0.874786128	0.381690286	0.985369679	ENSG00000157227	MMP14
15855.78397	-0.131456721	0.167305214	-0.785729969	0.432025702	0.991301167	ENSG00000110321	EIF4G2
15534.25906	0.065068761	0.299870027	0.216989879	0.828216232	0.999467288	ENSG00000135404	CD63
15628.16899	-0.306775614	0.161293858	-1.901967113	0.057175454	0.852306368	ENSG00000115677	HDLBP
15388.11596	-0.041113726	0.279353931	-0.147174326	0.882994425	0.999467288	ENSG00000150991	UBC
15460.93301	-0.466631754	0.327680828	-1.424043501	0.15443388	0.939432741	ENSG00000011028	MRC2
15311.76414	0.093672023	0.28976914	0.323264317	0.746495075	0.999467288	ENSG00000166598	HSP90B1
15425.18546	0.176214299	0.328798354	0.535934248	0.592003994	0.99483168	ENSG00000091986	CCDC80
15107.7331	0.595154308	0.230701673	2.57975723	0.009886979	0.630529247	ENSG00000102265	TIMP1
14847.59317	0.41768019	0.369032689	1.13182437	0.25770828	0.962465655	ENSG00000141429	GALNT1
14282.47785	-0.002126576	0.338552065	-0.006281385	0.994988213	0.999527751	ENSG00000205542	TMSB4X
14286.09099	-0.027258386	0.257723203	-0.10576613	0.91576791	0.999527751	ENSG00000034510	TMSB10
14409.595	-0.032179883	0.194165715	-0.165734116	0.868366196	0.999467288	ENSG00000164111	ANXA5

14553.55817	-0.181525394	0.205824956	-0.881940645	0.377808906	0.982609538	ENSG00000138758		11-Sep
14083.66717	0.461597514	0.310830586	1.485045343	0.137531813	0.934144427	ENSG00000142871	CYR61	
14011.77016	-0.068886837	0.296296634	-0.232492809	0.816155272	0.999467288	ENSG00000100097	LGALS1	
14085.80676	0.779918656	0.23352359	3.339785316	0.000838432	0.21163807	ENSG00000124942	AHNAK	
14446.24864	-0.260111055	0.381846627	-0.681192492	0.495749698	0.993513219	ENSG00000130508	PXDN	
14031.77336	-0.096046831	0.257160591	-0.373489698	0.708784011	0.99900742	ENSG00000109971	HSPA8	
13559.98103	-0.19850312	0.2437877	-0.814245836	0.415504132	0.990226751	ENSG00000142192	APP	
13564.91962	-0.311337209	0.209810436	-1.483897632	0.13783608	0.934144427	ENSG00000174444	RPL4	
13361.59477	-0.370315049	0.260812708	-1.419850481	0.155651215	0.939464191	ENSG00000179218	CALR	
13134.83224	-0.607887139	0.260264124	-2.335654754	0.019509242	0.737623006	ENSG00000142541	RPL13A	
13035.17263	0.023492523	0.185765029	0.126463647	0.899364923	0.999527751	ENSG00000183255	PTTG1IP	
13158.61836	0.235719147	0.829256564	0.28425358	0.776216057	0.999467288	ENSG00000137573	SULF1	
12995.31561	-0.379359354	0.240248944	-1.579026102	0.11433007	0.923922314	ENSG00000100316	RPL3	
13107.04106	0.028819595	0.297769521	0.096784906	0.922897211	0.999527751	ENSG00000127022	CANX	
12353.97944	-0.077204046	0.239823133	-0.321920763	0.747512723	0.999467288	ENSG00000067560	RHOA	
12864.0602	0.420722161	0.737439395	0.57051761	0.568326682	0.99483168	ENSG00000041982	TNC	
12124.71943	-0.231183056	0.284619174	-0.812253978	0.416645912	0.990226751	ENSG00000177469	CAVIN1	
12076.05961	-0.068709751	0.310834551	-0.221049272	0.825054068	0.999467288	ENSG00000129116	PALLD	
12235.90148	-0.126522093	0.284859968	-0.444155398	0.656930236	0.997568624	ENSG00000140937	CDH11	
12140.65935	-0.639264302	0.238921451	-2.675625401	0.007458996	0.563130099	ENSG00000137818	RPLP1	
12099.55332	0.09572872	0.205371634	0.466124355	0.641126491	0.996553586	ENSG00000136156	ITM2B	
12112.67539	-0.391298355	0.277997263	-1.4075619	0.159260836	0.939625921	ENSG00000138448	ITGAV	
12057.48812	0.68246251	0.381195535	1.790321363	0.073402264	0.888663898	ENSG00000211459	MT-RNR1	
11927.04718	-0.199305508	0.257992504	-0.772524414	0.439803893	0.991301167	ENSG00000086062	B4GALT1	
11817.50089	0.239284214	0.19853706	1.205237019	0.228111815	0.958865115	ENSG00000139644	TMBIM6	
11792.3872	-0.190114164	0.489199412	-0.388623042	0.697555019	0.998869317	ENSG00000111669	TPI1	
11577.05612	0.006838443	0.236091682	0.0289652	0.976892345	0.999527751	ENSG00000169604	ANTXR1	
11636.90902	-0.054529283	0.21763529	-0.250553498	0.802159338	0.999467288	ENSG00000185896	LAMP1	
11426.0024	-0.107683947	0.236685432	-0.454966519	0.649133326	0.997381078	ENSG00000135862	LAMC1	
10935.42532	-0.46883918	0.313548555	-1.495268189	0.134844464	0.933741583	ENSG00000092841	MYL6	
11082.33266	0.288582287	0.385193303	0.749188225	0.453743765	0.99135833	ENSG00000196230	TUBB	
11239.87856	-0.411617874	0.285075148	-1.44389252	0.148769211	0.936396974	ENSG00000006016	CRLF1	
11129.38546	0.01159456	0.353222426	0.032825096	0.973814066	0.999527751	ENSG00000083444	PLOD1	
10946.91787	-0.177207881	0.33836236	-0.523722204	0.60047177	0.99483168	ENSG00000136235	GPNMB	
10926.22891	-0.011540022	0.196174144	-0.058825397	0.95309118	0.999527751	ENSG00000164924	YWHAZ	
10891.5412	-0.422925328	0.232084503	-1.82229025	0.068410957	0.87420149	ENSG00000140988	RPS2	
10903.30452	-0.117166902	0.186714582	-0.627518751	0.530319249	0.99483168	ENSG00000070756	PABPC1	
10835.6103	-0.583054461	0.28825291	-2.022718386	0.04310219	0.806698895	ENSG00000166741	NNMT	
10806.17779	-0.234776402	0.356757067	-0.658084797	0.510483644	0.993513219	ENSG00000164694	FNDC1	
10503.18336	0.005359053	0.293680724	0.018247889	0.985441099	0.999527751	ENSG00000196262	PPIA	
10727.70693	0.224798348	0.270082786	0.832331269	0.405221985	0.990045328	ENSG00000188783	PRELP	
10510.72107	-0.324365055	0.208135042	-1.558435581	0.119130028	0.923922314	ENSG00000147403	RPL10	
10325.75907	0.331735165	0.246028142	1.348362677	0.177541762	0.946844293	ENSG00000134243	SORT1	
10337.17725	-0.329567281	0.186266913	-1.769328094	0.076839138	0.897052166	ENSG00000168028	RPSA	
10363.26904	-0.046405404	0.26464152	-0.17535194	0.860803102	0.999467288	ENSG00000182534	MXRA7	
10161.04013	-0.562820585	0.386134835	-1.457575266	0.144957651	0.935644597	ENSG00000124762	CDKN1A	
10256.04896	0.598853388	0.305802688	1.958299947	0.050194827	0.839078573	ENSG00000083857	FAT1	
10117.63405	0.030714253	0.162276284	0.189271358	0.849880142	0.999467288	ENSG00000009307	CSDE1	
10187.193	0.023366984	0.244310212	0.095644731	0.923802738	0.999527751	ENSG00000058262	SEC61A1	
10118.67358	-0.447653703	0.21859805	-2.047839413	0.040575737	0.796070921	ENSG00000198034	RPS4X	
9987.239551	0.051835447	0.266617198	0.194418993	0.84584782	0.999467288	ENSG00000096384	HSP90AB1	
9836.460983	-0.334925171	0.290698731	-1.1521384	0.249264206	0.962465655	ENSG00000100243	CYB5R3	
9940.85848	-0.091855675	0.249050787	-0.368823066	0.712259611	0.99900742	ENSG00000084234	APLP2	
9970.63155	0.207116698	0.19311575	1.072500288	0.283495385	0.963267117	ENSG00000134250	NOTCH2	
9867.783581	0.297592991	0.282559261	1.053205581	0.292246781	0.970706863	ENSG00000150938	CRIM1	
9891.133849	-0.400066922	0.200854225	-1.991827268	0.046390013	0.818574412	ENSG00000174748	RPL15	
9811.705189	0.229994127	0.276046698	0.833171084	0.404748248	0.989803411	ENSG00000113389	NPR3	
9718.950593	0.170505461	0.399685584	0.426598976	0.669671444	0.997568624	ENSG00000154096	THY1	
9757.806183	0.324753099	0.22283357	1.4573796	0.145011625	0.935644597	ENSG00000067167	TRAM1	
9800.732982	-0.596704895	0.264431193	-2.256560155	0.024035573	0.744948012	ENSG00000167526	RPL13	
9837.888166	0.401942398	0.388234181	1.035309144	0.300524561	0.972186492	ENSG00000198840	MT-ND3	
9705.941146	0.031585257	0.191267612	0.165136464	0.868836573	0.999467288	ENSG00000137076	TLN1	
9601.042423	-0.085823985	0.198812823	-0.43168234	0.665972305	0.997568624	ENSG00000065978	YBX1	
9458.826656	0.692813738	0.315780961	2.193969317	0.028237615	0.769339074	ENSG00000110092	CCND1	

9472.45754	-0.290471451	0.209631831	-1.38562665	0.165860936	0.943223078	ENSG00000130402	ACTN4
9367.558377	-0.295301821	0.267855246	-1.102467938	0.27025829	0.962465655	ENSG00000172757	CFL1
9501.508071	-0.147761615	0.259877799	-0.568581138	0.569640428	0.99483168	ENSG00000173812	EIF1
9494.115672	-0.699665938	0.289573746	-2.416192585	0.015683764	0.696728876	ENSG00000158747	NBL1
9455.816265	-0.061950335	0.198077686	-0.31275777	0.754464701	0.999467288	ENSG00000141756	FKBP10
9248.490102	-0.215416679	0.174303641	-1.235870214	0.21650681	0.954900762	ENSG00000204628	RACK1
9281.806554	0.052558528	0.263265117	0.199641066	0.841761309	0.999467288	ENSG00000167004	PDIA3
9109.810063	0.547035214	0.375981181	1.454953711	0.145682077	0.935644597	ENSG00000085063	CD59
9149.155993	-0.283070631	0.239539695	-1.181727441	0.237313866	0.962465655	ENSG00000148303	RPL7A
9364.517135	-0.407984684	0.287681252	-1.418183079	0.156137321	0.939464191	ENSG00000113721	PDGFRB
9197.091567	-0.470614996	0.348217737	-1.351496335	0.176536493	0.946278819	ENSG00000004399	PLXND1
9127.104641	-0.080463972	0.180049807	-0.446898409	0.654948412	0.997568624	ENSG00000197324	LRP10
8892.265936	-0.150751219	0.252670214	-0.596632334	0.550752878	0.99483168	ENSG00000108518	PFN1
9008.065783	-0.208460108	0.450917565	-0.462302034	0.643864741	0.997332006	ENSG00000116774	OLFML3
8986.337143	-0.387222604	0.388501022	-0.996709359	0.318905606	0.974768742	ENSG00000122641	INHBA
9062.659944	-0.109607664	0.456843707	-0.239923769	0.810389354	0.999467288	ENSG00000142798	HSPG2
8840.570075	-0.617727837	0.254500048	-2.4272209	0.015214989	0.694606015	ENSG00000105372	RPS19
8686.903539	-0.073668519	0.195816723	-0.376211582	0.706759606	0.99900742	ENSG00000078369	GNB1
8551.422826	-0.066732147	0.265069237	-0.251753647	0.801231488	0.999467288	ENSG00000071127	WDR1
8652.023975	0.197515968	0.227185391	0.869404355	0.384626002	0.985655313	ENSG00000080824	HSP90AA1
8558.223275	-0.135620898	0.203149205	-0.667592563	0.504393706	0.993513219	ENSG00000177697	CD151
8744.625655	-0.795147954	0.332911982	-2.38846301	0.016919011	0.714671438	ENSG00000106624	AEBP1
8531.916456	-0.309370338	0.242747173	-1.274454956	0.202502227	0.954310328	ENSG00000162734	PEA15
8563.292137	0.142860254	0.28975242	0.493042488	0.621982567	0.99483168	ENSG00000136240	KDELR2
8564.469905	-0.471232966	0.257302758	-1.831433794	0.067035821	0.87420149	ENSG00000154380	ENAH
8633.068742	-0.005295054	0.256874917	-0.020613355	0.983554087	0.999527751	ENSG00000196576	PLXNB2
8496.150642	-0.180679144	0.45921224	-0.393454547	0.693983788	0.998869317	ENSG00000141526	SLC16A3
8400.994147	-0.079513974	0.239533471	-0.331953499	0.739924372	0.999467288	ENSG00000168374	ARF4
8483.756881	-0.146682694	0.311749347	-0.470514838	0.637987236	0.996553586	ENSG00000049323	LTBP1
8272.71137	0.199553405	0.296217064	0.673672887	0.500519303	0.993513219	ENSG00000143933	CALM2
8264.910805	-0.038586892	0.248827553	-0.155074837	0.876762319	0.999467288	ENSG00000172270	BSG
8285.810046	-0.285373912	0.201887372	-1.413530271	0.157499867	0.939464191	ENSG00000089009	RPL6
8196.324938	-0.285260637	0.227508983	-1.2538434	0.209898931	0.954310328	ENSG00000137154	RPS6
8130.147698	-0.632876909	0.221226839	-2.860760073	0.004226268	0.462224997	ENSG00000161016	RPL8
8079.665786	-0.187869132	0.366358786	-0.512800945	0.608090568	0.99483168	ENSG00000116260	QSOX1
8108.085754	-0.667143544	0.256972834	-2.5961637	0.009427114	0.621716014	ENSG00000142534	RPS11
8106.018108	0.021374864	0.213079462	0.10031405	0.920095004	0.999527751	ENSG00000138760	SCARB2
8182.062988	-0.324928851	0.342761387	-0.947973907	0.343142738	0.977691382	ENSG00000171603	CLSTN1
7998.452907	0.013488663	0.212766109	0.063396672	0.949450637	0.999527751	ENSG00000138071	ACTR2
8170.714858	-0.751982328	0.383576307	-1.960450411	0.049943169	0.837780702	ENSG00000112655	PTK7
7938.041303	0.673629549	0.229369131	2.936879718	0.003315327	0.409258657	ENSG00000178209	PLEC
7961.685641	0.302132803	0.206799443	1.460994277	0.144017012	0.935644597	ENSG00000141367	CLTC
7875.593601	-0.132246467	0.216304805	-0.611389406	0.540941814	0.99483168	ENSG00000277443	MARCKS
7928.560506	-0.331974637	0.42726401	-0.776977768	0.437171887	0.991301167	ENSG00000148848	ADAM12
7958.360478	-0.465046788	0.385054907	-1.207741493	0.227146712	0.958865115	ENSG00000124225	PMEPA1
7714.121049	0.073741796	0.198943152	0.370667675	0.710885069	0.99900742	ENSG00000101160	CTSZ
7697.248902	0.132228977	0.292689199	0.451772658	0.651432769	0.997449432	ENSG00000035403	VCL
7716.509289	0.196969554	0.182884677	1.077015076	0.28147353	0.963267117	ENSG00000111897	SERINC1
7750.597594	-0.25025693	0.230108154	-1.087562198	0.276788427	0.962465655	ENSG00000082641	NFE2L1
7745.195872	-0.170006626	0.247420593	-0.687115913	0.492009687	0.993513219	ENSG00000073712	FERMT2
7674.425758	-0.149267079	0.19760504	-0.75538093	0.450020452	0.99135833	ENSG00000168385	2-Sep
7682.781916	0.001716855	0.227709616	0.00753967	0.993984271	0.999527751	ENSG00000110651	CD81
7726.379816	-0.067760137	0.378873842	-0.178846175	0.858058488	0.999467288	ENSG00000171314	PGAM1
7674.367612	-0.052073567	0.903248908	-0.057651404	0.954026304	0.999527751	ENSG00000114480	GBE1
7471.812146	-0.784649671	0.300854244	-2.608072465	0.009105368	0.611285922	ENSG00000198959	TGM2
7634.765152	-0.431883089	0.211626703	-2.040777849	0.041272913	0.800562177	ENSG00000108298	RPL19
7628.208565	-0.216208904	0.180361232	-1.198754862	0.230623279	0.959575179	ENSG00000229117	RPL41
7642.772993	-0.619207095	0.220309577	-2.810622686	0.004944573	0.472943531	ENSG00000231500	RPS18
7866.095953	0.825237134	0.418180849	1.973397723	0.048450267	0.833264304	ENSG00000212907	MT-ND4L
7556.75922	-0.215153292	0.207387861	-1.037444001	0.299528981	0.972186492	ENSG00000104904	OAZ1
7748.619181	0.102956713	0.836614591	0.123063492	0.902056821	0.999527751	ENSG00000122884	P4HA1
7554.714508	-0.563857275	0.185450698	-3.040469946	0.002362093	0.355371917	ENSG00000161203	AP2M1
7554.228213	0.212735192	0.25334338	0.839710878	0.401070513	0.988492517	ENSG00000145730	PAM
7547.219193	-0.493158521	0.232152009	-2.12429142	0.033645783	0.775963115	ENSG00000149273	RPS3

7530.481226	0.186172081	0.242256404	0.768491887	0.442195009	0.99135833	ENSG00000148248	SURF4
7507.418517	0.062893658	0.209590477	0.300078796	0.764117053	0.999467288	ENSG00000112697	TMEM30A
7503.486447	-0.009651463	0.227888843	-0.042351626	0.966218391	0.999527751	ENSG00000147065	MSN
7505.517368	0.06993991	0.211788969	0.330233962	0.741223187	0.999467288	ENSG00000170348	TMED10
7383.466402	-0.230511156	0.201952735	-1.141411413	0.253698757	0.962465655	ENSG00000123562	MORF4L2
7450.589071	-0.627405538	0.316117124	-1.984724935	0.047175083	0.827156057	ENSG00000206503	HLA-A
7386.909347	0.193547023	0.236694364	0.817708628	0.413523583	0.990226751	ENSG00000068697	LAPTM4A
7388.404333	-0.131979948	0.267483561	-0.493413307	0.621720582	0.99483168	ENSG00000030582	GRN
7436.195623	-0.47487079	0.231560836	-2.050738792	0.040292391	0.795004104	ENSG00000196205	EEF1A1P5
7253.674441	0.289794726	0.250754948	1.155688962	0.247808405	0.962465655	ENSG00000135046	ANXA1
7329.219106	0.225150086	0.296489114	0.759387364	0.44762087	0.99135833	ENSG00000157514	TSC22D3
7286.928251	-0.245543568	0.209561845	-1.17169978	0.241317611	0.962465655	ENSG00000167123	CERCAM
7323.118765	0.126742199	0.220960898	0.573595598	0.566241491	0.99483168	ENSG00000198542	ITGBL1
7210.11223	0.17481466	0.310957146	0.562182481	0.573991696	0.99483168	ENSG00000115419	GLS
7320.922971	-0.135558133	0.29859916	-0.453980289	0.649843014	0.997381078	ENSG00000172037	LAMB2
7207.233254	0.003765311	0.206492912	0.018234576	0.98545172	0.999527751	ENSG00000158710	TAGLN2
7257.41198	-0.099029984	0.204201767	-0.484961446	0.627703694	0.99483168	ENSG00000047849	MAP4
7151.35254	-0.800348064	0.250998223	-3.188660274	0.001429337	0.264665659	ENSG00000159176	CSRP1
7222.994626	-0.198022277	0.304201169	-0.650958303	0.515073404	0.993513219	ENSG00000070404	FSTL3
7113.30262	-0.166781515	0.272919723	-0.611100997	0.54113272	0.99483168	ENSG00000197043	ANXA6
7144.681774	-0.09165701	0.309306573	-0.296330626	0.7669776	0.999467288	ENSG00000136830	FAM129B
7075.496	0.916520437	0.379141463	2.417357442	0.015633657	0.696728876	ENSG00000105974	CAV1
7127.977221	0.050805434	0.251225345	0.202230528	0.839736509	0.999467288	ENSG00000101439	CST3
7058.316714	0.083013267	0.242826297	0.341862754	0.732454181	0.999467288	ENSG00000173706	HEG1
7069.557106	-0.076555184	0.178813668	-0.428128257	0.668557745	0.997568624	ENSG00000122642	FKBP9
6990.108805	1.826519039	1.228254772	1.48708483	0.136992408	0.934144427	ENSG00000167772	ANGPTL4
7040.959298	-0.194702534	0.234266511	-0.831115524	0.405908371	0.990045328	ENSG00000123989	CHPF
7078.773206	-0.497381787	0.23126325	-2.150716925	0.03149855	0.775963115	ENSG00000142937	RPS8
7032.417119	0.738574074	1.076486446	0.686096956	0.49265197	0.993513219	ENSG00000117394	SLC2A1
6857.44383	-0.132627387	0.266067629	-0.498472464	0.618151073	0.99483168	ENSG00000101608	MYL12A
6913.804711	-0.207579437	0.183803697	-1.129353984	0.258748536	0.962465655	ENSG00000143761	ARF1
6956.149669	-0.502573315	0.227208398	-2.211948673	0.026970212	0.765286378	ENSG00000105640	RPL18A
6931.662727	0.089427531	0.276786486	0.323092115	0.746625481	0.999467288	ENSG00000136026	CKAP4
6960.372999	-1.199572367	0.303630996	-3.950757272	7.79E-05	0.06618658	ENSG00000168461	RAB31
6856.921216	-0.545855648	0.317171254	-1.721012359	0.085248581	0.901307093	ENSG00000204525	HLA-C
6845.103998	-0.140211715	0.314125815	-0.446355275	0.655340634	0.997568624	ENSG00000110955	ATP5F1B
6876.171639	0.049840896	0.187909264	0.265239164	0.790825227	0.999467288	ENSG00000174437	ATP2A2
6754.098803	0.066715768	0.287089173	0.232386917	0.81623751	0.999467288	ENSG00000136153	LMO7
6847.537884	0.091731438	0.237643161	0.386004958	0.699492997	0.998869317	ENSG00000044115	CTNNA1
6714.130847	-0.041624118	0.23223081	-0.179235986	0.857752408	0.999467288	ENSG00000114353	GNAI2
6777.626156	0.142655262	0.204539689	0.697445387	0.485524106	0.992558339	ENSG00000135535	CD164
6753.339607	-0.227642589	0.314997799	-0.722679935	0.469876546	0.99135833	ENSG00000024422	EHD2
6742.45737	-0.093394716	0.218501575	-0.427432691	0.669064201	0.997568624	ENSG00000135677	GNS
6628.029001	-0.231921206	0.24548929	-0.944730442	0.344796511	0.977691382	ENSG00000002586	CD99
6654.457235	-0.042496282	0.252925966	-0.16801866	0.866568599	0.999467288	ENSG00000179222	MAGED1
6590.018707	-0.218337082	0.287233813	-0.760137116	0.447172629	0.99135833	ENSG00000050165	DKK3
6546.80093	0.50776534	0.908480361	0.558917244	0.5762182	0.99483168	ENSG00000104419	NDRG1
6697.881527	-0.153855059	0.295083525	-0.521394945	0.602091672	0.99483168	ENSG00000089597	GANAB
6706.233399	-0.149928668	0.457379405	-0.327799343	0.743063384	0.999467288	ENSG00000072682	P4HA2
6460.160291	-0.468823278	0.257639589	-1.819686487	0.068806762	0.87420149	ENSG00000109846	CRYAB
6538.236402	-0.519101075	0.208283117	-2.492285899	0.012692383	0.661790909	ENSG00000065534	MYLK
6510.510622	0.248353088	0.246977667	1.00556901	0.314622937	0.974768742	ENSG00000114850	SSR3
6466.578176	-0.181351861	0.262958334	-0.68966006	0.490407988	0.993513219	ENSG00000197879	MYO1C
6543.634461	0.155384983	0.223664151	0.694724579	0.487227922	0.993513219	ENSG00000134352	IL6ST
6400.943676	-0.481324108	0.191825578	-2.50917585	0.012101322	0.648356243	ENSG00000077549	CAPZB
6432.863435	-0.417914821	0.212870508	-1.963234948	0.04961888	0.836346249	ENSG00000008988	RPS20
6379.117151	0.089890702	0.252819798	0.355552463	0.722175749	0.99906455	ENSG00000115091	ACTR3
6422.743444	-0.037922427	0.192081055	-0.197429295	0.843491606	0.999467288	ENSG00000162576	MXRA8
6366.40529	0.075883978	0.203835021	0.372281356	0.709683378	0.99900742	ENSG00000104763	ASAH1
6418.91495	-0.021674674	0.27943857	-0.077565078	0.938174023	0.999527751	ENSG00000118257	NRP2
6289.557795	0.119904345	0.336405499	0.356428015	0.721520052	0.99900742	ENSG00000115380	EFEMP1
6391.527892	0.472837282	0.295815852	1.59841766	0.109950057	0.923922314	ENSG00000170017	ALCAM
6206.369668	0.429504427	0.3855549	1.113990322	0.265283338	0.962465655	ENSG00000077942	FBLN1
6174.622557	-0.214621995	0.218898594	-0.980463107	0.326857572	0.976026345	ENSG00000108828	VAT1

6268.761676	-0.00994521	0.23479833	-0.042356393	0.96621459	0.999527751	ENSG00000116209	TMEM59
6234.389927	-0.568727857	0.22512694	-2.526254102	0.011528606	0.647889729	ENSG00000166441	RPL27A
6348.749697	-0.381398257	0.343097111	-1.111633542	0.266295745	0.962465655	ENSG00000133816	MICAL2
6261.06314	-0.352976057	0.20444321	-1.726523749	0.084253231	0.901307093	ENSG00000145425	RPS3A
6279.969621	0.24489301	0.29230446	0.83780114	0.402142399	0.988592117	ENSG00000106105	GARS
6237.352426	-0.466987958	0.22508251	-2.074741206	0.038010526	0.790190505	ENSG00000197756	RPL37A
6097.979112	0.595078454	0.251300556	2.367994978	0.017884779	0.726053908	ENSG00000134531	EMP1
5983.880118	0.10906368	0.383081539	0.284700954	0.77587326	0.999467288	ENSG00000197956	S100A6
6139.083284	0.146777878	0.319287436	0.459704522	0.645728323	0.997368416	ENSG00000196549	MME
6144.039977	0.044088063	0.212775832	0.207204277	0.835850327	0.999467288	ENSG00000197965	MPZL1
5970.031256	-0.119506064	0.342783961	-0.348633769	0.727364273	0.999467288	ENSG00000142089	IFITM3
6076.610362	-0.043636077	0.182876603	-0.238609405	0.811408472	0.999467288	ENSG00000114867	EIF4G1
6052.055158	-0.036185221	0.230089307	-0.157265981	0.87503523	0.999467288	ENSG00000170275	CRTAP
5944.675189	-0.110059549	0.395554602	-0.278241103	0.780827285	0.999467288	ENSG00000115457	IGFBP2
6196.501933	-0.124881889	0.391336668	-0.319116249	0.749638363	0.999467288	ENSG00000099194	SCD
6045.679014	0.960647046	1.150952922	0.83465364	0.403912751	0.988927059	ENSG00000112715	VEGFA
6171.613985	0.232071661	0.845247903	0.274560469	0.783653941	0.999467288	ENSG00000103257	SLC7A5
6172.8038	-0.160351929	0.325798075	-0.492181941	0.62259073	0.99483168	ENSG00000265972	TXNIP
6061.017366	-0.278459177	0.211632294	-1.315768835	0.188251642	0.949094286	ENSG00000122406	RPL5
6018.888896	0.085025179	0.217325154	0.391234873	0.695623633	0.998869317	ENSG00000005893	LAMP2
5988.08992	-0.480916569	0.351587847	-1.367841847	0.171361586	0.944422693	ENSG00000103196	CRISPLD2
5947.064053	-0.64872445	0.347837958	-1.86501914	0.062178721	0.866005086	ENSG00000116016	EPAS1
5961.724936	-0.334485408	0.216971346	-1.541610971	0.123168158	0.927012392	ENSG00000108107	RPL28
5897.588008	-0.801983859	0.2925497	-2.741359364	0.006118555	0.499617804	ENSG00000169100	SLC25A6
5881.713286	0.153498436	0.210791718	0.728199559	0.466491436	0.99135833	ENSG00000113719	ERGIC1
5985.60767	0.028576697	0.364503755	0.078398909	0.937510742	0.999527751	ENSG00000197081	IGF2R
5873.109276	-2.144415238	0.726963297	-2.949826003	0.003179529	0.408098513	ENSG00000117318	ID3
5843.713221	-0.067602276	0.229942411	-0.293996551	0.768760539	0.999467288	ENSG00000163466	ARPC2
6063.700677	-0.177487167	0.359886963	-0.49317476	0.62188911	0.99483168	ENSG00000107731	UNC5B
5990.731765	-0.816575833	0.427726115	-1.909109135	0.056248015	0.847598083	ENSG00000143153	ATP1B1
5839.033633	0.019522017	0.179943171	0.108489901	0.913607091	0.999527751	ENSG00000134308	YWHAQ
5780.979832	-0.530742207	0.281847605	-1.883082198	0.059689229	0.858656911	ENSG00000127314	RAP1B
5840.490999	-0.15861717	0.205700801	-0.771106236	0.440643965	0.99135833	ENSG00000172638	EFEMP2
5568.916562	0.794732193	0.508578315	1.562654501	0.118133882	0.923922314	ENSG00000147872	PLIN2
5807.320877	0.233954697	0.256388767	0.912499794	0.361505678	0.979465649	ENSG00000118705	RPN2
5729.364963	0.046696268	0.260622753	0.179171876	0.857802746	0.999467288	ENSG00000162909	CAPN2
5668.865636	0.36768413	0.319626249	1.150356493	0.249997072	0.962465655	ENSG00000160789	LMNA
5733.717283	-0.061798775	0.18268723	-0.338276383	0.735154919	0.999467288	ENSG00000165119	HNRNPK
5697.338901	-0.086063838	0.249798796	-0.344532638	0.730445755	0.999467288	ENSG00000075415	SLC25A3
5744.429309	0.261835883	0.210452451	1.24415697	0.213441797	0.954310328	ENSG00000163399	ATP1A1
5693.55996	-0.058154471	0.205458375	-0.283047459	0.777140457	0.999467288	ENSG00000138674	SEC31A
5743.416853	0.219219321	0.255282401	0.858732604	0.390488058	0.98765184	ENSG00000131711	MAP1B
5692.3653	-0.297160414	0.199545662	-1.489185035	0.136438651	0.934144427	ENSG00000112306	RPS12
5656.489432	-0.319492353	0.95697476	-0.333856614	0.73848776	0.999467288	ENSG00000148344	PTGES
5587.308027	-0.221125742	0.240808556	-0.918263641	0.358480857	0.979427314	ENSG00000139926	FRMD6
5560.796527	-0.260749744	0.205965745	-1.265985975	0.205518104	0.954310328	ENSG00000163110	PDLIM5
5620.7279	-0.263859424	0.281074227	-0.938753534	0.347857301	0.977691382	ENSG00000164574	GALNT10
5604.091642	0.04651643	0.200586806	0.231901745	0.816614329	0.999467288	ENSG00000061676	NCKAP1
5579.429646	-0.499272985	0.207856881	-2.402003639	0.016305546	0.698816035	ENSG00000117519	CNN3
5600.557898	0.216652486	0.374217534	0.57894798	0.562624275	0.99483168	ENSG00000128272	ATF4
5518.661231	-0.038635994	0.247689022	-0.155985897	0.876044138	0.999467288	ENSG00000096060	FKBP5
5589.127031	0.181258726	0.484684806	0.373972371	0.708424872	0.99900742	ENSG00000104765	BNIP3L
5519.672071	0.24338492	0.190889766	1.275002453	0.202308374	0.954310328	ENSG00000077147	TM9SF3
5473.460701	-0.018199511	0.476413926	-0.038201048	0.969527385	0.999527751	ENSG00000067057	PFKP
5448.691627	-0.208770776	0.285420792	-0.731449082	0.464504895	0.99135833	ENSG00000102024	PLS3
5477.01369	-0.550095061	0.224514196	-2.450157144	0.014279388	0.684434275	ENSG00000164587	RPS14
5392.489413	-0.055930891	0.243695542	-0.229511342	0.818471506	0.999467288	ENSG00000106991	ENG
5339.398261	-0.045675334	0.2620329	-0.174311448	0.861620703	0.999467288	ENSG00000115641	FHL2
5376.124816	0.004580466	0.190278142	0.024072476	0.980794798	0.999527751	ENSG00000185787	MORF4L1
5354.653406	0.068630296	0.196008823	0.350138811	0.726234526	0.999467288	ENSG00000108953	YWHAE
5398.194047	-0.561464699	0.223900721	-2.507650251	0.01215369	0.648356243	ENSG00000234745	HLA-B
5316.665464	0.222624605	0.243335532	0.914887371	0.36025076	0.979465649	ENSG00000063660	GPC1
5317.037527	0.192866454	0.203427644	0.948083799	0.343086795	0.977691382	ENSG00000152558	TMEM123
5335.427028	0.088268069	0.244133253	0.361556927	0.717683157	0.99900742	ENSG00000163902	RPN1

5290.077117	0.016139389	0.238938934	0.067546084	0.946146976	0.999527751	ENSG00000163191	S100A11
5261.519914	0.477592282	0.286365471	1.667771891	0.095361018	0.921147998	ENSG00000168615	ADAM9
5326.023612	0.024650517	0.40179242	0.061351374	0.951079378	0.999527751	ENSG00000105220	GPI
5343.607083	-0.252568702	0.263630858	-0.958039221	0.338042982	0.977691382	ENSG00000187109	NAP1L1
5308.89743	0.087003403	0.242883303	0.358210721	0.720185625	0.99900742	ENSG00000163527	STT3B
5246.816429	0.447040455	0.279818104	1.597610908	0.110129597	0.923922314	ENSG00000122862	SRGN
5220.962319	0.005866318	0.288236251	0.020352466	0.983762203	0.999527751	ENSG00000088832	FKBP1A
5276.692993	0.090974581	0.230266976	0.395083056	0.692781593	0.998869317	ENSG00000071967	CYBRD1
5245.052505	-0.103589758	0.219134655	-0.472721937	0.636411574	0.99653524	ENSG00000162704	ARPC5
5249.766963	-0.382498925	0.234581539	-1.630558509	0.10298351	0.92169898	ENSG00000105193	RPS16
5233.070695	-0.394337892	0.336035494	-1.173500714	0.240595071	0.962465655	ENSG00000076706	MCAM
5214.193099	-0.321861464	0.235816457	-1.364881263	0.172290372	0.944422693	ENSG00000112977	DAP
5320.668592	0.676835612	0.456144593	1.483818118	0.137857179	0.934144427	ENSG00000135069	PSAT1
5172.740614	-0.241976786	0.202661588	-1.193994328	0.232480187	0.959575179	ENSG00000144713	RPL32
5177.108672	-0.119501198	0.282068849	-0.423659679	0.671814025	0.997568624	ENSG00000140092	FBLN5
5165.077155	-0.296053665	0.173947122	-1.701975066	0.088760043	0.910413547	ENSG00000221983	UBA52
5091.208432	0.479387827	0.270077412	1.775001561	0.075897629	0.897052166	ENSG00000138119	MYOF
5105.507453	-0.115781118	0.209097102	-0.553719382	0.579770908	0.99483168	ENSG00000197702	PARVA
5105.066524	-0.071689161	0.218819708	-0.327617476	0.743200906	0.999467288	ENSG00000129353	SLC44A2
5111.521471	0.044504817	0.19890168	0.223752848	0.822949622	0.999467288	ENSG00000086598	TMED2
5122.840184	-0.353648613	0.226068291	-1.564344169	0.117736764	0.923922314	ENSG00000125691	RPL23
5118.101557	0.272133336	0.233464413	1.16563091	0.243763705	0.962465655	ENSG00000198363	ASPH
5095.909994	0.048771535	0.18377652	0.265385018	0.790712876	0.999467288	ENSG00000166913	YWHAB
5065.490464	0.137181394	0.397245133	0.345331843	0.72984491	0.999467288	ENSG00000167552	TUBA1A
4838.616538	0.360952823	0.954866482	0.378013921	0.705420252	0.99900742	ENSG00000115461	IGFBP5
5084.568994	-0.211690276	0.203517882	-1.040155655	0.29826759	0.972186492	ENSG00000138326	RPS24
5077.625064	0.313347985	0.281894725	1.111578039	0.266319619	0.962465655	ENSG00000077782	FGFR1
4957.563868	-0.176490988	0.420684229	-0.419533169	0.674826519	0.998869317	ENSG00000120129	DUSP1
5073.73191	0.306697084	0.237047238	1.293822642	0.19572669	0.954310328	ENSG00000140575	IQGAP1
5008.436446	0.070307495	0.274053398	0.256546701	0.797528724	0.999467288	ENSG00000176014	TUBB6
5000.494327	0.005476826	0.197851189	0.027681544	0.977916144	0.999527751	ENSG00000148180	GSN
5132.456318	-0.170909401	0.930584789	-0.183658065	0.854281709	0.999467288	ENSG00000123500	COL10A1
5050.789643	0.070973857	0.219214502	0.323764422	0.746116394	0.999467288	ENSG00000075420	FNDC3B
5020.260226	-0.566805912	0.225555203	-2.51293654	0.011973087	0.648356243	ENSG00000083845	RPS5
4995.076006	-0.078980216	0.180724018	-0.43702114	0.662096017	0.997568624	ENSG00000260032	NORAD
4906.203025	-0.161796204	0.306408306	-0.528041181	0.597470754	0.99483168	ENSG00000002834	LASP1
4941.439659	-0.175621838	0.339433343	-0.517397131	0.604878962	0.99483168	ENSG00000138080	EMILIN1
4931.96433	0.504636917	0.275102098	1.834362298	0.066600232	0.87420149	ENSG00000065308	TRAM2
4970.165011	-0.258557576	0.229798085	-1.125151138	0.260524995	0.962465655	ENSG00000147604	RPL7
4970.33691	0.364751684	0.232695083	1.567509201	0.116995717	0.923922314	ENSG00000162733	DDR2
4921.856166	-0.03167998	0.17559191	-0.180418222	0.85682425	0.999467288	ENSG00000165280	VCP
4967.306043	-0.508262559	0.212638229	-2.390268962	0.016836038	0.714671438	ENSG00000162244	RPL29
4903.036541	-0.182902996	0.193342041	-0.946007368	0.344144828	0.977691382	ENSG00000143947	RPS27A
5001.961284	0.291783019	1.010175208	0.288843972	0.772700781	0.999467288	ENSG00000176171	BNIP3
4876.29646	-0.234157542	0.251101708	-0.932520706	0.351067491	0.977691382	ENSG00000197958	RPL12
4905.121143	0.11350101	0.178226123	0.636837116	0.524230947	0.99483168	ENSG00000132824	SERINC3
4825.067037	-0.270437533	0.373109001	-0.724821789	0.468561367	0.99135833	ENSG00000119280	C1orf198
4857.582724	0.11662318	0.337725873	0.345319058	0.729854521	0.999467288	ENSG00000206538	VGLL3
4873.987854	-0.208430817	0.358242047	-0.581815614	0.560690885	0.99483168	ENSG00000079308	TNS1
4872.769315	-0.091873131	0.269214337	-0.341263887	0.732904932	0.999467288	ENSG00000118816	CCNI
4794.784138	-0.401159806	0.281410113	-1.425534432	0.154002774	0.939432741	ENSG00000064666	CNN2
4855.362872	-0.168338235	0.260674762	-0.645778798	0.51842262	0.993513219	ENSG00000070614	NDST1
4804.168556	-0.475934319	0.202550358	-2.349708608	0.018788113	0.726053908	ENSG00000131469	RPL27
4765.481916	0.265785824	0.253436862	1.048725993	0.294304248	0.970706863	ENSG00000213625	LEPROT
4768.587521	-0.035372486	0.218299793	-0.16203628	0.871277284	0.999467288	ENSG00000126247	CAPNS1
4829.079369	0.146999552	0.206788124	0.710870377	0.477164565	0.99135833	ENSG00000152291	TGOLN2
4783.4558	0.065218824	0.292405242	0.223042594	0.823502357	0.999467288	ENSG00000181163	NPM1
4845.082838	-0.281125584	0.207907954	-1.352163675	0.176322961	0.946278819	ENSG00000213639	PPP1CB
4858.417221	0.351605053	0.348305659	1.009472697	0.312747989	0.973427483	ENSG00000096696	DSP
4807.005191	0.08375681	0.216714973	0.386483726	0.699138453	0.998869317	ENSG00000169439	SDC2
4784.173485	-0.30366296	0.220446627	-1.377489709	0.168360894	0.943223078	ENSG00000133030	MPRIP
4802.01719	-0.02323136	0.231947773	-0.100157719	0.920219112	0.999527751	ENSG00000075223	SEMA3C
4783.270769	0.16548151	0.261552404	0.632689693	0.526936302	0.99483168	ENSG00000074695	LMAN1
4787.311159	0.472855657	0.224580016	2.105510831	0.035246866	0.784512596	ENSG00000197102	DYNC1H1

4782.949715	0.017388649	0.263608671	0.065963872	0.947406589	0.999527751	ENSG00000182752	PAPPA
4739.834462	-0.161733245	0.184488226	-0.8766589	0.380671937	0.983746147	ENSG00000108946	PRKAR1A
4734.122038	0.054214972	0.279729672	0.193812017	0.846323078	0.999467288	ENSG00000109099	PMP22
4796.700292	0.032456703	0.288525566	0.1124916	0.91043363	0.999527751	ENSG00000196305	IARS
4683.562643	0.058561943	0.198108544	0.295605335	0.767531499	0.999467288	ENSG00000136238	RAC1
4662.872722	-0.350511333	0.467138961	-0.750336329	0.453052168	0.99135833	ENSG00000156804	FBXO32
4682.18321	0.0834329	0.196041284	0.425588421	0.670407779	0.997568624	ENSG00000215301	DDX3X
4618.89525	-0.000906166	0.211143973	-0.004291697	0.996575732	0.999527751	ENSG00000204592	HLA-E
4701.589748	0.11611698	0.22665166	0.512314714	0.608430769	0.99483168	ENSG00000108654	DDX5
4640.044279	-0.596827952	0.282955593	-2.109263671	0.034921829	0.784512596	ENSG00000170889	RPS9
4653.109074	-0.198845944	0.201554827	-0.986560069	0.323858354	0.975412329	ENSG00000170027	YWHAG
4625.493089	-0.408450136	0.309698851	-1.31886229	0.187215155	0.949094286	ENSG00000198431	TXNRD1
4625.852447	-0.173361533	0.239215754	-0.724707842	0.468631283	0.99135833	ENSG00000064601	CTSA
4651.7364	0.579613529	0.263431072	2.200247388	0.027789348	0.769167109	ENSG00000166825	ANPEP
4598.421448	0.068701209	0.205254923	0.334711624	0.737842632	0.999467288	ENSG00000130985	UBA1
4656.976384	-0.790154405	0.494089487	-1.599213149	0.10977325	0.923922314	ENSG00000107562	CXCL12
4570.909811	0.122356935	0.245524779	0.49834862	0.618238344	0.99483168	ENSG00000198668	CALM1
4555.549302	0.093799521	0.241837349	0.387862011	0.698118153	0.998869317	ENSG00000113732	ATP6V0E1
4580.265928	-0.188338449	0.241245202	-0.780693037	0.434983057	0.991301167	ENSG00000198755	RPL10A
4586.815155	0.148790845	0.225455392	0.659956915	0.509281478	0.993513219	ENSG00000182220	ATP6AP2
4500.987982	0.124804315	0.497984421	0.250618915	0.802108757	0.999467288	ENSG00000183160	TMEM119
4554.204618	-0.341984377	0.201648342	-1.695944399	0.08989641	0.910413547	ENSG00000142676	RPL11
4537.750363	0.080070165	0.181234581	0.441804009	0.658631037	0.997568624	ENSG00000078808	SDF4
4529.500154	-0.152153855	0.228702721	-0.665290968	0.505864406	0.993513219	ENSG00000182670	TTC3
4537.108977	0.193580721	0.203830529	0.949714064	0.342257561	0.977691382	ENSG00000143384	MCL1
4672.425231	-0.066459465	0.348734027	-0.190573501	0.848859752	0.999467288	ENSG00000104415	WISP1
4493.210374	0.106159248	0.251623897	0.421896526	0.673100546	0.998473603	ENSG00000110880	CORO1C
4495.554344	-0.691862423	0.318732323	-2.170669158	0.02995619	0.773201806	ENSG00000213626	LBH
4500.477197	-0.097608001	0.177598118	-0.549600425	0.582593467	0.99483168	ENSG00000075785	RAB7A
4509.328042	-0.035265619	0.21121231	-0.16696763	0.867395519	0.999467288	ENSG00000104805	NUCB1
4517.424225	0.521627224	0.299179759	1.743524448	0.081242048	0.897807954	ENSG00000145623	OSMR
4523.556257	0.286549625	0.226707509	1.263961774	0.206243747	0.954310328	ENSG00000103335	PIEZO1
4469.357512	0.197713249	0.209282505	0.944719429	0.344802135	0.977691382	ENSG00000135926	TMBIM1
4502.710254	-0.239528355	0.185297724	-1.292667554	0.196126064	0.954310328	ENSG00000105887	MTPN
4473.685922	-0.518078335	0.296544163	-1.747052881	0.080628166	0.897807954	ENSG00000161011	SQSTM1
4424.127197	0.758184907	0.519670917	1.45897121	0.144573032	0.935644597	ENSG00000148677	ANKRD1
4596.601229	-0.725908492	0.334312689	-2.171345915	0.029905034	0.773201806	ENSG00000107957	SH3PXD2A
4501.20087	-0.432724514	0.218125543	-1.983832376	0.04727453	0.827156057	ENSG00000071082	RPL31
4567.886494	0.354331407	0.442787892	0.800228309	0.423578531	0.991301167	ENSG00000168003	SLC3A2
4477.245016	-0.402334452	0.197773216	-2.034322238	0.041919116	0.804159726	ENSG00000163682	RPL9
4496.045824	0.298496289	0.269119842	1.109157491	0.267362247	0.962465655	ENSG00000125844	RRBP1
4457.677523	0.569925006	0.38858911	1.466652026	0.14247074	0.935644597	ENSG00000069849	ATP1B3
4409.407716	0.396399144	0.4667243	0.849321846	0.395702228	0.988378925	ENSG00000074410	CA12
4418.044621	-0.050832064	0.246410078	-0.206290524	0.83656398	0.999467288	ENSG00000073921	PICALM
4459.675905	0.15346556	0.338984596	0.452721339	0.650749412	0.997449432	ENSG00000184575	XPOT
4362.995517	0.555925162	0.263976159	2.105967314	0.035207192	0.784512596	ENSG00000111186	WNT5B
4408.01383	0.294590015	0.209249629	1.40784008	0.159178429	0.939523519	ENSG00000137845	ADAM10
4381.037828	0.113829421	0.218156558	0.521778588	0.6018245	0.99483168	ENSG00000166311	SMPD1
4371.806965	-0.124267284	0.302373946	-0.410972195	0.681092923	0.998869317	ENSG00000152234	ATP5F1A
4385.725145	-0.14434907	0.206359899	-0.699501554	0.484238642	0.991892545	ENSG00000095139	ARCN1
4362.215055	-0.218356531	0.181287325	-1.204477649	0.228405016	0.95933456	ENSG00000100934	SEC23A
4426.369917	-0.135474029	0.293412865	-0.461718094	0.644283494	0.997332006	ENSG00000090863	GLG1
4327.025146	-0.000447596	0.23555243	-0.001900196	0.998483864	0.999527751	ENSG00000118680	MYL12B
4388.308043	0.128031882	0.243556142	0.525677081	0.59911259	0.99483168	ENSG00000125304	TM9SF2
4317.440006	0.137730385	0.30568048	0.450569776	0.652299654	0.997449432	ENSG00000130309	COLGALT1
4327.803219	-0.748667543	0.394170736	-1.899348364	0.057518688	0.852306368	ENSG00000143878	RHOB
4324.200537	-0.345477684	0.194982201	-1.771842157	0.076420762	0.897052166	ENSG00000063177	RPL18
4312.49961	0.180285638	0.269830576	0.668143841	0.504041779	0.993513219	ENSG00000117385	P3H1
4328.490705	-0.002103607	0.315006322	-0.006677982	0.994671781	0.999527751	ENSG00000169764	UGP2
4284.564931	-0.013328047	0.2181545	-0.061094531	0.951283925	0.999527751	ENSG00000170759	KIF5B
4262.134718	-0.062548078	0.327344378	-0.191077295	0.848465036	0.999467288	ENSG00000135486	HNRNPA1
4262.823123	-0.001775169	0.281380952	-0.006308774	0.99496636	0.999527751	ENSG00000142552	RCN3
4295.917492	-0.350718012	0.236111834	-1.485389388	0.137440705	0.934144427	ENSG00000105223	PLD3
4239.947058	-0.110675073	0.316968811	-0.349167076	0.726963882	0.999467288	ENSG00000114019	AMOTL2

4225.908163	0.280746369	0.452439312	0.620517186	0.534917343	0.99483168	ENSG00000140285	FGF7
4308.725925	0.101462207	0.399493309	0.253977236	0.799513144	0.999467288	ENSG00000072274	TFRC
4287.581192	0.286235508	0.34174205	0.837577665	0.402267941	0.988592117	ENSG00000051108	HERPUD1
4297.878373	-0.230584974	0.215040466	-1.07228643	0.2835914	0.963290356	ENSG00000174807	CD248
4214.322159	-0.495151146	0.184752418	-2.680079373	0.00736047	0.563130099	ENSG00000196923	PDLIM7
4254.658368	-0.055522861	0.278852355	-0.19911204	0.842175103	0.999467288	ENSG00000168487	BMP1
4264.277179	-0.031647525	0.354548904	-0.089261381	0.928874185	0.999527751	ENSG00000071537	SEL1L
4295.731346	0.089747223	0.312049581	0.287605652	0.773648618	0.999467288	ENSG00000105281	SLC1A5
4220.172658	-0.258599133	0.267732871	-0.965884884	0.334101787	0.976026345	ENSG00000140941	MAP1LC3B
4221.2582	-0.070319685	0.296249016	-0.237366814	0.812372233	0.999467288	ENSG00000182326	C1S
4170.194397	-0.032683189	0.239074103	-0.136707358	0.891262114	0.999467288	ENSG00000023191	RNH1
4155.633756	-0.272204721	0.171156155	-1.590388152	0.111747339	0.923922314	ENSG00000138069	RAB1A
4166.058097	-0.005748222	0.262370542	-0.021908792	0.982520712	0.999527751	ENSG00000151348	EXT2
4152.250553	-0.306855581	0.190971728	-1.606811562	0.108095711	0.923922314	ENSG00000104388	RAB2A
4133.154832	-0.025064213	0.209415231	-0.119686678	0.904731354	0.999527751	ENSG00000122218	COPA
4109.968065	-0.235708086	0.167154427	-1.410121711	0.158503748	0.939464191	ENSG00000162368	CMPK1
4103.760198	0.104513093	0.222941621	0.468791302	0.639218818	0.996553586	ENSG00000140391	TSPAN3
4116.287242	0.034748107	0.201117846	0.172774856	0.862828402	0.999467288	ENSG00000180398	MCFD2
4111.273145	0.087784155	0.206602179	0.424894624	0.670913494	0.997568624	ENSG00000182934	SRPRA
4074.055061	0.094498115	0.205177666	0.460567261	0.645109105	0.997368416	ENSG00000071054	MAP4K4
4058.259072	-0.12421476	0.223189355	-0.556544285	0.57783883	0.99483168	ENSG00000172380	GNG12
4060.135434	-0.316734405	0.224460401	-1.411092573	0.15821732	0.939464191	ENSG00000159840	ZYX
4093.069713	-0.116710903	0.340981843	-0.342278939	0.732140984	0.999467288	ENSG00000115415	STAT1
4104.202998	0.329032012	0.273864744	1.20143983	0.229580634	0.959575179	ENSG00000106799	TGFBR1
4032.655201	-0.202474276	0.181237177	-1.117178496	0.263918013	0.962465655	ENSG00000179010	MRFAP1
4137.524209	0.029872428	0.452256154	0.066052011	0.947336417	0.999527751	ENSG00000170558	CDH2
4043.182878	0.635721773	0.254453953	2.498376484	0.012476361	0.657649947	ENSG00000115306	SPTBN1
4054.094617	-0.428327169	0.281395593	-1.522153083	0.127970728	0.928627271	ENSG00000060237	WNK1
4025.72953	0.380500208	0.309013407	1.231338832	0.218196164	0.956884001	ENSG00000091136	LAMB1
4080.339024	0.276081755	0.365440674	0.755476264	0.449963269	0.99135833	ENSG00000163171	CDC42EP3
4020.825305	-0.45849481	0.421901872	-1.086733291	0.277154697	0.962465655	ENSG00000125249	RAP2A
4092.513875	-1.109035951	0.415262764	-2.670684801	0.007569669	0.563130099	ENSG00000116679	IVNS1ABP
3957.172724	-0.425644908	0.299120336	-1.422988866	0.154739383	0.939432741	ENSG00000140264	SERF2
4037.388989	-0.157974549	0.193745544	-0.815371264	0.414859828	0.990226751	ENSG00000132475	H3F3B
3954.831162	0.365656405	0.276485798	1.322514241	0.18599697	0.949094286	ENSG00000163513	TGFBR2
4000.688147	0.239173324	0.208094996	1.149346831	0.250412995	0.962465655	ENSG00000082153	BZW1
3966.839359	0.213005441	0.213960091	0.995538184	0.319474583	0.974964731	ENSG00000143753	DEGS1
3995.558412	-0.353620711	0.296691909	-1.191878513	0.233308884	0.959684953	ENSG00000152049	KCNE4
4035.279894	-0.08789782	0.336975285	-0.260843522	0.79421318	0.999467288	ENSG00000113013	HSPA9
3916.0381	-0.334500398	0.291623181	-1.14702952	0.251369421	0.962465655	ENSG00000175274	TP53I11
3967.469159	0.197586841	0.229953324	0.859247596	0.390203929	0.987407688	ENSG00000179820	MYADM
3962.917156	0.040390174	0.210460463	0.191913358	0.847810074	0.999467288	ENSG00000135387	CAPRIN1
3935.432651	-0.069718142	0.224811164	-0.310118683	0.756470705	0.999467288	ENSG00000187514	PTMA
3995.443968	-0.078415271	0.407759754	-0.192307528	0.847501322	0.999467288	ENSG00000162407	PLPP3
3960.204675	0.486276836	0.337508235	1.440785097	0.149645405	0.936396974	ENSG00000167601	AXL
3947.953849	0.263816505	0.278218241	0.948235832	0.34300941	0.977691382	ENSG00000124783	SSR1
3932.114233	0.17363203	0.204507873	0.849023698	0.395868106	0.988378925	ENSG00000213585	VDAC1
3946.660035	-0.218865682	0.228541944	-0.957660892	0.338233783	0.977691382	ENSG00000188846	RPL14
3912.341825	-0.380792532	0.256126246	-1.486737646	0.137084116	0.934144427	ENSG00000168268	NT5DC2
3920.212264	0.03415927	0.211718625	0.161342772	0.871823437	0.999467288	ENSG00000071553	ATP6AP1
3940.230513	-0.088557133	0.33404083	-0.265108708	0.790925719	0.999467288	ENSG00000136205	TNS3
3889.705292	0.283946163	0.228247215	1.244029034	0.213488877	0.954310328	ENSG00000164294	GPX8
3818.009704	-0.313782378	0.224103254	-1.40016877	0.161462785	0.942964826	ENSG00000129250	KIF1C
3909.139128	-0.899771787	0.263385551	-3.416177475	0.000635068	0.195790358	ENSG00000021762	OSBPL5
3886.397614	-0.324860966	0.254253162	-1.277706692	0.201352865	0.954310328	ENSG00000131473	ACLY
3864.918563	-0.319425561	0.388105795	-0.82303734	0.410486759	0.990226751	ENSG00000100600	LGMN
3822.955752	0.252698624	0.312975255	0.807407678	0.419431645	0.990226751	ENSG00000119655	NPC2
3845.965732	-0.407618092	0.216632885	-1.881607644	0.059889308	0.858996918	ENSG00000131236	CAP1
3843.420524	0.120939428	0.231189791	0.523117513	0.600892479	0.99483168	ENSG00000115053	NCL
3859.390898	0.229188382	0.182608719	1.255079076	0.209450055	0.954310328	ENSG00000184743	ATL3
3783.560002	0.14602485	0.357792584	0.408127099	0.683180366	0.998869317	ENSG00000156976	EIF4A2
3827.414743	0.257594392	0.20287111	1.269744088	0.204175803	0.954310328	ENSG00000197694	SPTAN1
3785.141497	-0.186677177	0.195526946	-0.954738878	0.339709764	0.977691382	ENSG00000085733	CTTN
3805.616258	-0.430366092	0.220396748	-1.95268803	0.050856573	0.842188482	ENSG00000136942	RPL35

3853.068972	0.247891516	0.348701926	0.710898041	0.47714742	0.99135833	ENSG00000104635	SLC39A14
3789.893805	0.269417668	0.29633249	0.90917357	0.363258513	0.98048242	ENSG00000049860	HEXB
3813.996673	0.234066331	0.206997315	1.130769889	0.258151957	0.962465655	ENSG00000153187	HNRNPU
3833.153214	0.120304614	0.243359126	0.494350124	0.621058932	0.99483168	ENSG00000134910	STT3A
3788.562664	0.027748839	0.251641273	0.110271413	0.912194128	0.999527751	ENSG00000104331	IMPAD1
3776.241836	0.098575374	0.19522297	0.504937377	0.613602821	0.99483168	ENSG00000133872	SARAF
3759.505338	-0.054384025	0.213540034	-0.254678356	0.798971535	0.999467288	ENSG00000092199	HNRNPC
3803.655567	0.009104028	0.291288069	0.031254381	0.975066671	0.999527751	ENSG00000145431	PDGFC
3733.604579	-0.404009742	0.217730076	-1.855553211	0.063517268	0.868202292	ENSG00000137409	MTCH1
3740.653994	0.05745613	0.294322019	0.195215194	0.845224485	0.999467288	ENSG00000050405	LIMA1
3723.39952	0.590888921	0.27977939	2.11198159	0.034688028	0.784512596	ENSG00000144746	ARL6IP5
3655.733041	0.661256959	0.42252096	1.565027589	0.11757644	0.923922314	ENSG00000135821	GLUL
3726.08981	0.446881897	0.303960715	1.470196231	0.141508615	0.935644597	ENSG00000005884	ITGA3
3761.378574	-0.227623985	0.269121817	-0.845802793	0.397662767	0.988378925	ENSG00000135506	OS9
3763.312215	0.31239514	0.260535951	1.199048112	0.230509238	0.959575179	ENSG00000166340	TPP1
3746.747745	0.025437454	0.231236861	0.110006053	0.912404575	0.999527751	ENSG00000244038	DDOST
3709.239416	0.080167534	0.287342936	0.278996015	0.780247883	0.999467288	ENSG00000206527	HACD2
3710.200473	0.128176702	0.283955644	0.451396915	0.651703506	0.997449432	ENSG00000105438	KDELR1
3713.508811	0.171825844	0.215233578	0.798322669	0.424683266	0.991301167	ENSG00000132341	RAN
3707.839722	-0.253575123	0.207764944	-1.220490416	0.22227902	0.956884001	ENSG00000106682	EIF4H
3678.034966	-0.138095587	0.18398691	-0.750572894	0.452909739	0.99135833	ENSG00000184432	COPB2
3680.509837	0.345165823	0.193156052	1.786979072	0.073940871	0.889199003	ENSG00000083312	TNPO1
3721.167799	0.060555164	0.318365984	0.190206138	0.849147601	0.999467288	ENSG00000120594	PLXDC2
3736.150797	0.006747632	0.298635006	0.022594913	0.981973401	0.999527751	ENSG00000157613	CREB3L1
3669.725566	-0.124678221	0.23579206	-0.528763442	0.596969561	0.99483168	ENSG00000132507	EIF5A
3653.001539	0.28725818	0.275759375	1.041698692	0.297551395	0.972186492	ENSG00000213719	CLIC1
3658.872386	0.018697534	0.222311847	0.084104983	0.932972963	0.999527751	ENSG00000184840	TMED9
3671.267156	0.027632198	0.190018371	0.145418562	0.88438041	0.999467288	ENSG00000067182	TNFRSF1A
3691.954537	0.870268862	0.363685117	2.392918548	0.016714951	0.712135817	ENSG00000026508	CD44
3659.797504	0.104404832	0.212388977	0.491573684	0.62302075	0.99483168	ENSG00000175166	PSMD2
3655.887668	0.129498161	0.927249916	0.139658315	0.888929963	0.999467288	ENSG00000143387	CTSK
3630.312389	0.25503775	0.333977532	0.763637448	0.445083326	0.99135833	ENSG00000122566	HNRNPA2B1
3700.492256	-0.017045644	0.32121394	-0.053066326	0.957679061	0.999527751	ENSG00000152377	SPOCK1
3656.666835	-0.00208681	0.242799886	-0.008594775	0.993142446	0.999527751	ENSG00000069275	NUCKS1
3622.781106	0.38314669	0.241856448	1.584190514	0.113150371	0.923922314	ENSG00000143549	TPM3
3669.611344	-0.040838978	0.201821443	-0.202352027	0.839641529	0.999467288	ENSG00000183853	KIRREL1
3616.512434	-0.043887845	0.320457434	-0.136953741	0.89106736	0.999467288	ENSG00000143819	EPHX1
3623.066325	0.103096417	0.411148286	0.250752394	0.80200555	0.999467288	ENSG00000103888	CEMIP
3601.038357	0.046266031	0.291729484	0.158592236	0.873990146	0.999467288	ENSG00000103855	CD276
3612.818229	-0.118762527	0.235085478	-0.505188701	0.613426306	0.99483168	ENSG00000108679	LGALS3BP
3606.878787	0.74616569	0.482470386	1.546552311	0.121971237	0.923922314	ENSG00000197930	ERO1A
3510.652718	-0.718487356	0.45370389	-1.583604133	0.113283833	0.923922314	ENSG00000137124	ALDH1B1
3611.155661	-0.05030917	0.186775157	-0.26935686	0.787655081	0.999467288	ENSG00000125991	ERGIC3
3613.567889	0.02361558	0.216680398	0.108988076	0.913211948	0.999527751	ENSG00000143870	PDIA6
3583.920799	0.034664455	0.202278428	0.171370004	0.863932839	0.999467288	ENSG00000147140	NONO
3600.627392	0.458485189	0.239639824	1.913226201	0.055719101	0.847598083	ENSG00000127603	MACF1
3571.190603	-0.249084089	0.278150669	-0.89550059	0.37051955	0.981455968	ENSG00000129038	LOXL1
3558.380039	0.055029503	0.234819746	0.234347851	0.81471494	0.999467288	ENSG00000131446	MGAT1
3566.185953	0.072059192	0.255792174	0.281709916	0.77816595	0.999467288	ENSG00000156642	NPTN
3545.292653	-0.128066076	0.246701056	-0.51911442	0.603680953	0.99483168	ENSG00000197226	TBC1D9B
3524.265414	-0.523843841	0.257642168	-2.033222456	0.042030053	0.804797577	ENSG00000071282	LMCD1
3543.023022	-0.315970446	0.201450491	-1.568476921	0.116769872	0.923922314	ENSG00000145741	BTF3
3555.933645	0.301073597	0.248839645	1.209910091	0.226313395	0.958865115	ENSG00000156515	HK1
3616.029193	-0.014503777	0.456076318	-0.031801207	0.974630584	0.999527751	ENSG00000128591	FLNC
3582.054951	0.062588221	0.313953128	0.199355302	0.841984823	0.999467288	ENSG00000122863	CHST3
3553.310991	-0.568049289	0.257315936	-2.207594672	0.027272541	0.768732301	ENSG00000145592	RPL37
3548.131178	-0.012239703	0.2602857	-0.047024109	0.962494012	0.999527751	ENSG00000187079	TEAD1
3509.206355	-0.156844576	0.202467602	-0.774665053	0.438537606	0.991301167	ENSG00000197712	FAM114A1
3497.224749	0.799798269	0.280186276	2.854523355	0.004310146	0.462224997	ENSG00000136068	FLNB
3540.319609	0.076617179	0.203388644	0.37670333	0.706394088	0.99900742	ENSG00000130175	PRKCSH
3530.919174	-0.163185934	0.240210957	-0.679344258	0.49691976	0.993513219	ENSG00000130147	SH3BP4
3597.856007	-0.128156361	0.85899064	-0.14919413	0.881400452	0.999467288	ENSG00000168209	DDIT4
3532.704784	0.02408886	0.265538031	0.090717176	0.927717322	0.999527751	ENSG00000031698	SARS
3492.988014	-0.367655558	0.226807577	-1.621002101	0.105017188	0.92169898	ENSG00000177156	TALDO1

3476.644159	0.155434523	0.279533616	0.556049485	0.578177026	0.99483168	ENSG00000198856	OSTC
3601.726078	-0.416650962	0.994457657	-0.418973054	0.675235825	0.998869317	ENSG00000139211	AMIGO2
3515.241882	0.16222587	0.222897239	0.727805651	0.466732565	0.99135833	ENSG00000171867	PRNP
3596.118811	-0.602147267	0.510660856	-1.179152975	0.238337267	0.962465655	ENSG00000074181	NOTCH3
3413.675751	0.679494489	1.017647436	0.667711101	0.504318023	0.993513219	ENSG00000111674	ENO2
3463.419198	-0.543105109	0.235721226	-2.304014441	0.021221833	0.73997555	ENSG00000182899	RPL35A
3446.891673	-0.320177218	0.203078866	-1.576615155	0.114884103	0.923922314	ENSG00000131143	COX4I1
3436.781657	-0.048243478	0.881287704	-0.054742031	0.956343984	0.999527751	ENSG00000122861	PLAU
3475.763679	0.000595195	0.326607144	0.001822358	0.998545969	0.999527751	ENSG00000075618	FSCN1
3439.731065	0.292472208	0.198114804	1.47627639	0.13986971	0.935644597	ENSG00000117868	ESYT2
3444.74345	-0.514376317	0.219117834	-2.347487231	0.018900521	0.726053908	ENSG00000168610	STAT3
3430.899239	0.490759649	0.40814182	1.202424317	0.229199173	0.959575179	ENSG00000127083	OMD
3391.682595	0.185081723	0.391996869	0.472151023	0.636818995	0.99653524	ENSG00000164932	CTHRC1
3378.007278	0.52462219	0.327112072	1.603799537	0.108758247	0.923922314	ENSG00000197614	MFAP5
3394.591747	-0.091050236	0.365680311	-0.248988619	0.803369585	0.999467288	ENSG00000164096	C4orf3
3416.977402	-0.245320556	0.363410317	-0.67505116	0.499643261	0.993513219	ENSG00000112769	LAMA4
3400.422802	-0.192980736	0.206563592	-0.934243705	0.350178196	0.977691382	ENSG00000114391	RPL24
3387.245401	0.119284426	0.222168849	0.536908869	0.591330564	0.99483168	ENSG00000093010	COMT
3353.539376	0.176980612	0.307908686	0.574782785	0.565438211	0.99483168	ENSG00000153904	DDAH1
3382.095401	0.335047159	0.300535271	1.114834735	0.264921248	0.962465655	ENSG00000147027	TMEM47
3375.09043	0.216315872	0.225486695	0.959328761	0.337393151	0.977691382	ENSG00000163069	SGCB
3343.585766	-0.165673789	0.255588268	-0.648205766	0.516851873	0.993513219	ENSG00000176658	MYO1D
3395.055269	0.210606572	0.21348829	0.986501753	0.323886956	0.975412329	ENSG00000147649	MTDH
3403.655821	-0.125810646	0.230497082	-0.545823163	0.5851875	0.99483168	ENSG00000136010	ALDH1L2
3320.000297	-0.459122395	0.492476486	-0.932272724	0.3511956	0.977691382	ENSG00000130821	SLC6A8
3339.275726	0.072377396	0.387237809	0.186906843	0.851733674	0.999467288	ENSG00000154188	ANGPT1
3354.372466	0.116466194	0.229472189	0.507539472	0.611776348	0.99483168	ENSG00000079246	XRCC5
3379.23328	-0.115749596	0.272988851	-0.424008511	0.671559606	0.997568624	ENSG00000151176	PLBD2
3322.735028	-0.324286447	0.204620958	-1.58481541	0.11300828	0.923922314	ENSG00000112335	SNX3
3336.405728	0.016025228	0.236681053	0.067708114	0.94601799	0.999527751	ENSG00000057608	GDI2
3345.780644	0.138408581	0.232884611	0.594322574	0.552296383	0.99483168	ENSG00000129083	COPB1
3257.783722	-0.40636715	0.420950999	-0.965354997	0.334367034	0.976026345	ENSG00000172403	SYNPO2
3319.120958	-0.432689357	0.26211079	-1.650788038	0.098781863	0.921147998	ENSG00000105701	FKBP8
3315.726669	-0.247510375	0.223927982	-1.105312395	0.269024249	0.962465655	ENSG00000174099	MSRB3
3318.658161	0.135131954	0.232814047	0.580428695	0.561625559	0.99483168	ENSG00000081087	OSTM1
3334.310054	0.169250449	0.272239826	0.621696142	0.534141689	0.99483168	ENSG00000078098	FAP
3331.957778	0.030967937	0.265474882	0.1166511	0.907136542	0.999527751	ENSG00000106397	PLOD3
3305.325237	-0.028971088	0.186592724	-0.155263761	0.876613384	0.999467288	ENSG00000039560	RAI14
3310.287084	0.223142366	0.263853192	0.845706524	0.397716483	0.988378925	ENSG00000133318	RTN3
3300.898089	-0.582615216	0.24812534	-2.348068177	0.018871067	0.726053908	ENSG00000177600	RPLP2
3290.627024	-0.180076524	0.20783264	-0.866449679	0.38624361	0.98647506	ENSG00000181789	COPG1
3254.568255	0.081781258	0.231971804	0.35254827	0.724427125	0.999456245	ENSG00000137575	SDCBP
3262.092848	-0.233534279	0.217441127	-1.074011537	0.282817511	0.963267117	ENSG00000265808	SEC22B
3247.853511	0.588638971	0.345967901	1.701426543	0.088862921	0.910413547	ENSG00000116678	LEPR
3266.467624	-0.162914054	0.224724991	-0.724948541	0.4684836	0.99135833	ENSG00000196531	NACA
3296.538755	-0.176721233	0.272334565	-0.648912241	0.516395102	0.993513219	ENSG00000099860	GADD45B
3307.204895	-0.246266616	0.337220529	-0.730283583	0.465216861	0.99135833	ENSG00000168140	VASN
3250.296066	-0.274158987	0.244118994	-1.123054713	0.261414258	0.962465655	ENSG00000123091	RNF11
3285.500283	0.148627226	0.206255605	0.720597272	0.471157332	0.99135833	ENSG00000134440	NARS
3257.992284	-0.274705541	0.220945595	-1.24331757	0.213750832	0.954310328	ENSG00000063046	EIF4B
3253.94005	-0.408786702	0.200374418	-2.040114227	0.04133895	0.800562177	ENSG00000114942	EEF1B2
3238.652912	-0.084564535	0.215784417	-0.391893616	0.69513682	0.998869317	ENSG00000110917	MLEC
3214.205907	0.032351377	0.283143091	0.114258049	0.909033237	0.999527751	ENSG00000071242	RPS6KA2
3240.129786	-0.001121787	0.183353332	-0.00611817	0.995118437	0.999527751	ENSG00000109332	UBE2D3
3232.316066	0.007227495	0.341178188	0.02118393	0.983098933	0.999527751	ENSG00000134363	FST
3224.691007	-0.583943878	0.177394558	-3.29178012	0.000995554	0.228234741	ENSG00000116871	MAP7D1
3223.613028	-0.112845857	0.177161073	-0.636967562	0.524145973	0.99483168	ENSG00000087274	ADD1
3188.219775	0.047162824	0.204574	0.230541631	0.817670918	0.999467288	ENSG00000118985	ELL2
3185.52411	0.214144795	0.275960871	0.775996954	0.437750784	0.991301167	ENSG00000128791	TWSG1
3198.646097	0.134931738	0.193636419	0.696830373	0.485908956	0.992923464	ENSG00000065150	IPO5
3213.520695	0.124016715	0.210513147	0.589116246	0.555783296	0.99483168	ENSG00000107581	EIF3A
3316.534347	0.774666993	0.752729111	1.029144458	0.303411798	0.972793733	ENSG00000154175	ABI3BP
3220.82689	0.19370775	0.220963277	0.876651327	0.380676051	0.983746147	ENSG00000136628	EPRS
3188.717301	-0.438407418	0.223372382	-1.962675122	0.049683936	0.836346249	ENSG00000118181	RPS25

3182.876534	-0.340141277	0.206383962	-1.648099367	0.099332282	0.921147998	ENSG00000115268	RPS15	
3195.914729	0.017394225	0.187906073	0.092568721	0.926246194	0.999527751	ENSG00000122545		7-Sep
3216.830749	-0.252186934	0.254467049	-0.991039645	0.321666222	0.975337095	ENSG00000196498	NCOR2	
3303.094317	-0.802978277	0.446398906	-1.798790872	0.072051768	0.885654197	ENSG00000235750	KIAA0040	
3158.87045	-0.009997708	0.269398814	-0.03711118	0.970396357	0.999527751	ENSG00000142864	SERBP1	
3150.074974	-0.215638257	0.200494719	-1.075530857	0.282137125	0.963267117	ENSG00000172354	GNB2	
3244.611526	1.007738211	0.984215405	1.023900059	0.305882482	0.973427483	ENSG00000163661	PTX3	
3186.457927	-0.220392647	0.261749086	-0.84199968	0.399788132	0.988384114	ENSG00000105329	TGFB1	
3191.442178	0.250323327	0.269358587	0.929331155	0.352717494	0.977691382	ENSG00000100664	EIF5	
3142.022097	-0.246373927	0.209987571	-1.173278614	0.240684096	0.962465655	ENSG00000141522	ARHGDIA	
3139.004676	0.616267507	0.273587346	2.252543898	0.024287922	0.747954063	ENSG00000049449	RCN1	
3149.781879	0.006848449	0.203110313	0.03371788	0.973102121	0.999527751	ENSG00000163697	APBB2	
3153.997829	0.070316345	0.233148126	0.301595153	0.762960704	0.999467288	ENSG00000150403	TMCO3	
3165.329619	-0.133747191	0.230044752	-0.581396401	0.560973322	0.99483168	ENSG00000165678	GHITM	
3114.702744	-0.127055371	0.213223536	-0.595878733	0.551256242	0.99483168	ENSG00000105568	PPP2R1A	
3152.637019	-0.048922068	0.274915447	-0.177953144	0.858759774	0.999467288	ENSG00000151414	NEK7	
3149.242942	0.712487472	0.219894709	3.240130132	0.001194752	0.244243321	ENSG00000114251	WNT5A	
3119.666732	0.155322854	0.188615519	0.823489261	0.410229821	0.990226751	ENSG00000116005	PCYOX1	
3132.520642	-0.172839968	0.231211251	-0.747541339	0.454736859	0.99135833	ENSG00000128245	YWHAH	
3095.93273	0.046085695	0.277528117	0.166057751	0.868111502	0.999467288	ENSG00000142669	SH3BGRL3	
3127.28103	-0.281409093	0.224472477	-1.253646312	0.20997059	0.954310328	ENSG00000102181	CD99L2	
3107.105261	-0.423748568	0.261434431	-1.620859832	0.105047703	0.92169898	ENSG00000182197	EXT1	
3080.470368	-0.239768593	0.263984141	-0.90826893	0.363736153	0.98048242	ENSG00000160691	SHC1	
3116.021407	-0.39094999	0.222949066	-1.753539484	0.079509448	0.897052166	ENSG00000198561	CTNND1	
3083.417036	-0.015375031	0.23212039	-0.066237315	0.947188889	0.999527751	ENSG00000119318	RAD23B	
3100.763049	-0.141474146	0.200951839	-0.704020162	0.481420218	0.991525194	ENSG00000150961	SEC24D	
3087.718966	-0.177981745	0.329901388	-0.539499837	0.58954201	0.99483168	ENSG00000178695	KCTD12	
3041.427714	-0.345135834	0.276133911	-1.249885725	0.211341295	0.954310328	ENSG00000143774	GUK1	
3084.978646	-0.145495524	0.219038489	-0.664246383	0.506532631	0.993513219	ENSG00000137693	YAP1	
3045.349048	0.277411595	0.357118388	0.776805689	0.43727342	0.991301167	ENSG00000106333	PCOLCE	
3076.670325	0.06544237	0.192102985	0.340662952	0.733357332	0.999467288	ENSG00000153113	CAST	
3068.573172	-0.223276843	0.214779693	-1.039562166	0.298543362	0.972186492	ENSG00000100380	ST13	
3077.511585	0.234751283	0.249618935	0.940438605	0.346992628	0.977691382	ENSG00000183291	SELENOF	
3054.723661	-0.200235997	0.210125768	-0.952934042	0.340623491	0.977691382	ENSG00000088986	DYNLL1	
3051.791066	-0.05786329	0.249351349	-0.232055252	0.8164951	0.999467288	ENSG00000060138	YBX3	
3070.646426	0.431613386	0.253606973	1.701898731	0.088774354	0.910413547	ENSG00000133657	ATP13A3	
3110.888628	0.201130137	0.375323582	0.535884626	0.59203829	0.99483168	ENSG00000134369	NAV1	
3076.34853	0.130603911	0.22664479	0.57624934	0.56444666	0.99483168	ENSG00000134970	TMED7	
3105.796824	0.070368943	0.285536587	0.246444574	0.805338105	0.999467288	ENSG00000100219	XBP1	
3044.706171	-0.059466684	0.19674231	-0.302256715	0.762456373	0.999467288	ENSG00000196419	XRCC6	
3034.781902	-0.494318075	0.255947018	-1.931329692	0.053442294	0.847598083	ENSG00000184216	IRAK1	
3015.395859	-0.172441776	0.261803638	-0.658668374	0.510108746	0.993513219	ENSG00000140319	SRP14	
3041.46269	0.213156254	0.241130979	0.883985355	0.376704119	0.982609538	ENSG00000164040	PGRMC2	
3057.784749	-0.072037753	0.20495749	-0.351476555	0.725230858	0.999467288	ENSG00000175582	RAB6A	
3080.038905	-0.488606403	0.28145752	-1.735986316	0.082566254	0.899922174	ENSG00000157933	SKI	
3052.437855	0.028611422	0.266711136	0.107274944	0.914570862	0.999527751	ENSG00000171812	COL8A2	
3037.396643	-0.288738615	0.211965564	-1.362195868	0.173136079	0.944422693	ENSG00000138757	G3BP2	
3038.364278	0.158471837	0.209805677	0.755326739	0.450052959	0.99135833	ENSG00000006125	AP2B1	
3054.128969	0.245652774	0.260242755	0.943937034	0.345201826	0.977691382	ENSG00000126777	KTN1	
3008.714688	0.028285871	0.193390292	0.146263137	0.883713666	0.999467288	ENSG00000079332	SAR1A	
3055.181098	0.257576457	0.299537377	0.859914244	0.389836316	0.987216662	ENSG00000008710	PKD1	
3009.398215	0.07249143	0.197499439	0.367046258	0.713584514	0.99900742	ENSG00000049656	CLPTM1L	
3027.365734	0.123538142	0.185935543	0.664413806	0.506425499	0.993513219	ENSG00000110958	PTGES3	
3046.907453	0.23991244	0.308426851	0.777858475	0.436652451	0.991301167	ENSG00000166250	CLMP	
2995.895558	-0.019552029	0.165210721	-0.118346004	0.905793507	0.999527751	ENSG00000167986	DDB1	
2945.681325	-0.734504186	0.777202046	-0.945062086	0.34462718	0.977691382	ENSG00000119630	PGF	
2984.73384	-0.092870314	0.248909059	-0.373109416	0.709067011	0.99900742	ENSG00000102316	MAGED2	
2975.149771	-0.126643006	0.250914625	-0.504725487	0.613751657	0.99483168	ENSG00000155254	MARVELD1	
2952.736476	-0.664674028	0.254577351	-2.610892234	0.009030635	0.611285922	ENSG00000162458	FBLIM1	
2986.858614	0.048925911	0.207554656	0.235725433	0.813645733	0.999467288	ENSG00000196141	SPATS2L	
2968.145582	-0.328143552	0.209960648	-1.562881209	0.118080538	0.923922314	ENSG00000197894	ADH5	
2957.180642	-0.027308516	0.200441235	-0.136242008	0.891629967	0.999467288	ENSG00000089063	TMEM230	
2998.455901	0.253541262	0.224439731	1.129663011	0.258618249	0.962465655	ENSG00000112081	SRSF3	
2951.582664	0.376596933	0.336988423	1.117536708	0.263764913	0.962465655	ENSG00000152518	ZFP36L2	

2962.588321	-0.168491512	0.168237101	-1.001512217	0.316579237	0.974768742	ENSG00000084733	RAB10
2946.209403	0.272504216	0.251900762	1.081791949	0.279344985	0.963267117	ENSG00000152601	MBNL1
2989.218664	0.406895624	0.804202094	0.505961905	0.612883393	0.99483168	ENSG00000119938	PPP1R3C
2948.682563	-0.185918545	0.191844633	-0.969109959	0.332490329	0.976026345	ENSG00000197157	SND1
2953.162616	0.187051618	0.236853837	0.789734377	0.42968291	0.991301167	ENSG00000123131	PRDX4
3027.243309	0.092514382	0.514354418	0.179865049	0.857258515	0.999467288	ENSG00000134107	BHLHE40
2829.375113	-0.230930741	1.009743245	-0.228702437	0.819100201	0.999467288	ENSG00000165507	DEPP1
2960.035628	0.121226389	0.211867238	0.57218091	0.567199419	0.99483168	ENSG00000125977	EIF2S2
2942.518277	-0.099622424	0.21813543	-0.456699877	0.647886779	0.997368416	ENSG00000178104	PDE4DIP
2937.023049	-0.172848269	0.196744724	-0.878540806	0.379650303	0.983373687	ENSG00000122026	RPL21
2940.400413	-0.087113515	0.223306693	-0.390107049	0.69645739	0.998869317	ENSG00000155660	PDIA4
2909.007893	-0.183578115	0.238081469	-0.771072673	0.440663857	0.99135833	ENSG00000255302	EID1
2935.70711	-0.255950553	0.226950245	-1.127782666	0.259411716	0.962465655	ENSG00000266412	NCOA4
2939.277239	-0.140187284	0.172829781	-0.811129212	0.417291472	0.990226751	ENSG00000140943	MBTPS1
2916.47012	-0.126743427	0.203879714	-0.621657863	0.534166865	0.99483168	ENSG00000074842	MYDGF
2918.940169	1.233595992	0.411299796	2.999262352	0.002706342	0.380278034	ENSG00000135318	NT5E
2933.405504	0.260294473	0.215608635	1.207254402	0.227334185	0.958865115	ENSG00000129625	REEP5
2978.688395	0.633682952	0.393598274	1.609973909	0.107403552	0.923922314	ENSG00000248527	MTATP6P1
2932.758728	-0.339564098	0.217479772	-1.561359456	0.118438962	0.923922314	ENSG00000110700	RPS13
2907.968576	-0.340131422	0.289146353	-1.176329629	0.23946318	0.962465655	ENSG00000164713	BRI3
2925.429257	0.077410533	0.202259358	0.382729054	0.70192067	0.998869317	ENSG00000178719	GRINA
2906.33006	0.153234785	0.23031062	0.665339638	0.505833283	0.993513219	ENSG00000183726	TMEM50A
2908.459955	0.260921105	0.19727486	1.322627248	0.185959368	0.949094286	ENSG00000185825	BCAP31
2952.859694	-0.055172785	0.452012716	-0.12206025	0.902851302	0.999527751	ENSG00000198743	SLC5A3
2917.065031	-0.19285505	0.210602113	-0.915731789	0.359807588	0.979465649	ENSG00000171863	RPS7
2897.745587	-0.020665536	0.249655387	-0.082776248	0.934029456	0.999527751	ENSG00000136810	TXN
2942.709928	-0.434719353	0.304744391	-1.426504855	0.153722667	0.939432741	ENSG00000168264	IRF2BP2
2894.484086	0.162957012	0.214014419	0.761430062	0.446400237	0.99135833	ENSG00000173402	DAG1
2910.963162	0.306808398	0.222239049	1.380533256	0.167422517	0.943223078	ENSG00000141424	SLC39A6
2893.92135	-0.132083575	0.393523803	-0.335643167	0.737139968	0.999467288	ENSG00000159403	C1R
2959.828846	0.239255971	0.381001692	0.627965639	0.53002645	0.99483168	ENSG00000065911	MTHFD2
2879.539694	-0.011899088	0.209264679	-0.056861428	0.954655581	0.999527751	ENSG00000124155	PIGT
2865.430641	-0.269894742	0.412628573	-0.654086411	0.513056136	0.993513219	ENSG00000115594	IL1R1
2849.460371	-0.013660907	0.226403172	-0.06033885	0.951885761	0.999527751	ENSG00000112473	SLC39A7
2815.749693	-0.266074675	0.253970759	-1.0476587	0.294795882	0.970706863	ENSG00000084207	GSTP1
2838.981808	-0.005182232	0.242984918	-0.02132738	0.982984503	0.999527751	ENSG00000129562	DAD1
2861.083706	-0.050805441	0.175042705	-0.290245975	0.771628066	0.999467288	ENSG00000119335	SET
2970.520816	-0.870866683	1.031340025	-0.844403069	0.398444208	0.988378925	ENSG00000119927	GPAM
2857.350827	0.037843826	0.203028134	0.186396957	0.852133479	0.999467288	ENSG00000205339	IPO7
2834.940011	-0.169690984	0.238987277	-0.710041917	0.477678143	0.99135833	ENSG00000143641	GALNT2
2842.681773	0.190761494	0.250589246	0.761251716	0.446506734	0.99135833	ENSG00000105971	CAV2
2841.027993	-0.097491412	0.203347013	-0.479433704	0.631630121	0.99483168	ENSG00000177239	MAN1B1
2832.235303	0.121310374	0.264295594	0.458995066	0.646237709	0.997368416	ENSG00000101294	HM13
2837.628457	0.184309722	0.216740063	0.850372189	0.395118193	0.988378925	ENSG00000104853	CLPTM1
2785.621033	-0.414253899	0.375880059	-1.102090651	0.270422264	0.962465655	ENSG00000233276	GPX1
2823.948615	0.004001876	0.21263539	0.018820366	0.984984407	0.999527751	ENSG00000008441	NFIX
2844.684616	-0.327567157	0.202291972	-1.619279076	0.105387231	0.92169898	ENSG00000160710	ADAR
2831.643907	0.113856543	0.214220468	0.531492366	0.59507763	0.99483168	ENSG00000124486	USP9X
2811.117814	-0.027888688	0.236294864	-0.118024945	0.906047893	0.999527751	ENSG00000111321	LTBR
2840.40059	-0.147916326	0.221016824	-0.669253691	0.503333662	0.993513219	ENSG00000157637	SLC38A10
2791.794025	-0.423225472	0.258587249	-1.636683455	0.101696628	0.92169898	ENSG00000104964	AES
2795.242944	0.450445881	0.468045977	0.962396651	0.335850395	0.977691382	ENSG00000123416	TUBA1B
2807.816899	0.064664451	0.215290988	0.300358374	0.763903811	0.999467288	ENSG00000166734	CASC4
2816.721907	-0.01393995	0.178653807	-0.078027724	0.937806	0.999527751	ENSG00000155506	LARP1
2827.370401	-0.35826713	0.32439064	-1.104431159	0.269406151	0.962465655	ENSG00000006327	TNFRSF12A
2824.807541	-0.052154799	0.208987008	-0.249560007	0.80292763	0.999467288	ENSG00000197858	GPAA1
2791.674373	-0.175182682	0.211885672	-0.826779276	0.408362178	0.990226751	ENSG00000143612	C1orf43
2793.101377	0.29184061	0.20367562	1.43286963	0.151895098	0.938052926	ENSG00000058668	ATP2B4
2817.547187	-0.770188588	0.48337446	-1.593358052	0.11107989	0.923922314	ENSG00000171617	ENC1
2792.37222	-0.184981756	0.248718648	-0.743738989	0.457034397	0.99135833	ENSG00000120438	TCP1
2785.694058	-0.330434323	0.208415778	-1.585457329	0.112862465	0.923922314	ENSG00000113758	DBN1
2759.170481	-0.264918008	0.25961123	-1.020441249	0.307519239	0.973427483	ENSG00000155366	RHOC
2780.222832	-0.120568342	0.190203884	-0.633890007	0.526152607	0.99483168	ENSG00000163956	LRPAP1
2743.284608	-0.249967217	0.300660742	-0.831392937	0.405751688	0.990045328	ENSG00000100300	TSPO

2801.038823	-0.318471222	0.221549093	-1.437474726	0.150583149	0.936396974	ENSG00000124422	USP22	
2786.095201	-0.277302534	0.240378609	-1.153607369	0.248661176	0.962465655	ENSG00000075426	FOSL2	
2784.992916	-0.171532251	0.178553934	-0.960674721	0.336715745	0.977691382	ENSG00000023734	STRAP	
2779.193504	0.143203916	0.235098958	0.609121867	0.542443664	0.99483168	ENSG00000111817	DSE	
2808.354141	0.529065417	0.492881788	1.073412388	0.283086129	0.963267117	ENSG00000162433	AK4	
2766.403006	-0.008113514	0.282136643	-0.028757391	0.977058084	0.999527751	ENSG00000079739	PGM1	
2756.367904	0.078549513	0.206552883	0.380287662	0.703731893	0.99900742	ENSG00000116237	ICMT	
2754.492319	-0.420095623	0.219241425	-1.916132517	0.05534823	0.847598083	ENSG00000198918	RPL39	
2735.548578	-0.304463687	0.266330822	-1.143178566	0.25296446	0.962465655	ENSG00000176871	WSB2	
2758.79996	0.039087836	0.236064406	0.165581234	0.868486517	0.999467288	ENSG00000048828	FAM120A	
2777.093315	0.638459512	0.283823121	2.249497893	0.024480837	0.749756093	ENSG00000167978	SRRM2	
2753.983103	-0.121756743	0.188718598	-0.645176171	0.518813026	0.993513219	ENSG00000184007	PTP4A2	
2737.645305	-0.087560033	0.282278033	-0.310190743	0.756415909	0.999467288	ENSG00000033100	CHPF2	
2749.074369	-0.012763203	0.234674984	-0.05438672	0.956627059	0.999527751	ENSG00000124067	SLC12A4	
2726.026167	0.611269217	0.329357155	1.855946375	0.063461202	0.868202292	ENSG00000102007	PLP2	
2752.275889	0.093718976	0.235063492	0.398696434	0.690116896	0.998869317	ENSG00000077232	DNAJC10	
2729.030758	-0.03212726	0.195821869	-0.164063698	0.869680999	0.999467288	ENSG00000113558	SKP1	
2749.371049	-0.086470572	0.221378418	-0.39060073	0.696092386	0.998869317	ENSG00000173517	PEAK1	
2747.567208	0.101522918	0.223955337	0.453317698	0.65031999	0.997381078	ENSG00000177311	ZBTB38	
2727.021333	-0.218859657	0.219638094	-0.99645582	0.319028723	0.974768742	ENSG00000159335	PTMS	
2724.847388	-0.126759907	0.207992822	-0.609443661	0.542230405	0.99483168	ENSG00000169714	CNBP	
2743.607155	0.170457097	0.227811065	0.748238883	0.454316081	0.99135833	ENSG00000170248	PDCD6IP	
2713.233989	-0.052525219	0.247655454	-0.212089894	0.832036904	0.999467288	ENSG00000184640		9-Sep
2709.191424	0.320745653	0.254329389	1.261142706	0.207257437	0.954310328	ENSG00000152894	PTPRK	
2739.686151	-0.236012984	0.252138505	-0.936044987	0.349250026	0.977691382	ENSG00000198612	COPS8	
2783.461229	-0.453946692	0.506476888	-0.896283133	0.370101565	0.981455968	ENSG00000168159	RNF187	
2796.29796	-0.150328417	0.41055418	-0.366159753	0.714245872	0.99900742	ENSG00000187735	TCEA1	
2699.192231	-0.103851166	0.310635139	-0.334318797	0.738139008	0.999467288	ENSG00000244486	SCARF2	
2719.982	-0.232785582	0.424198352	-0.54876588	0.583166129	0.99483168	ENSG00000141959	PFKL	
2752.313979	0.205996607	0.283105438	0.727632109	0.46683882	0.99135833	ENSG00000204217	BMPR2	
2711.732638	0.039509765	0.179046145	0.220668054	0.825350907	0.999467288	ENSG00000173726	TOMM20	
2695.895855	-0.271839415	0.222966171	-1.219195783	0.222769892	0.956884001	ENSG00000197728	RPS26	
2699.471612	-0.049113707	0.228794909	-0.214662588	0.830030393	0.999467288	ENSG00000166272	WBP1L	
2672.429651	0.093734762	0.251903445	0.372105915	0.709813993	0.99900742	ENSG00000136436	CALCOCO2	
2696.67617	0.158453742	0.207346847	0.764196536	0.44475013	0.99135833	ENSG00000106609	TMEM248	
2631.506563	0.574918048	0.333361771	1.724607013	0.084598319	0.901307093	ENSG00000067082	KLF6	
2690.852748	0.248172786	0.219790644	1.129132625	0.25884189	0.962465655	ENSG00000153827	TRIP12	
2701.946797	-0.422606999	0.253937729	-1.664215084	0.096069455	0.921147998	ENSG00000168710	AHCYL1	
2695.45262	-0.122161429	0.20108777	-0.607503028	0.543517134	0.99483168	ENSG00000107372	ZFAND5	
2664.905614	-0.261397265	0.166812674	-1.567010816	0.117112163	0.923922314	ENSG00000197256	KANK2	
2669.353023	-0.205450679	0.235987592	-0.8705995	0.38397287	0.985655313	ENSG00000157240	FZD1	
2684.209217	0.127455052	0.242721349	0.525108533	0.599507744	0.99483168	ENSG00000113407	TARS	
2627.37903	-0.117816323	0.293276179	-0.40172483	0.687886549	0.998869317	ENSG00000013364	MVP	
2668.566786	-0.303955838	0.240975173	-1.261357483	0.207180079	0.954310328	ENSG00000175390	EIF3F	
2648.206353	-0.152609148	0.20375655	-0.748977879	0.453870539	0.99135833	ENSG00000022840	RNF10	
2687.266483	-0.35220807	0.311461383	-1.1308242	0.258129092	0.962465655	ENSG00000175215	CTDSP2	
2617.078823	-0.368160864	0.341769345	-1.077220266	0.281381874	0.963267117	ENSG00000189043	NDUFA4	
2696.599276	-0.174580839	0.40202067	-0.434258365	0.664100834	0.997568624	ENSG00000097007	ABL1	
2651.33429	0.043693559	0.192237482	0.227289487	0.820198648	0.999467288	ENSG00000169223	LMAN2	
2662.368404	-0.376087863	0.202965771	-1.852962011	0.063887804	0.868202292	ENSG00000166963	MAP1A	
2648.601113	0.172646751	0.20004143	0.863054971	0.388107244	0.986799595	ENSG00000011304	PTBP1	
2573.830983	0.366280747	0.426294076	0.859220823	0.390218697	0.987407688	ENSG00000185201	IFITM2	
2657.509798	-0.048027997	0.186791793	-0.257120486	0.797085764	0.999467288	ENSG00000007168	PAFAH1B1	
2643.311965	0.177386097	0.185592632	0.955781996	0.339182386	0.977691382	ENSG00000067141	NEO1	
2645.759867	0.311359129	0.200659218	1.551681163	0.120738532	0.923922314	ENSG00000008952	SEC62	
2628.947628	-0.271342451	0.337910894	-0.80300001	0.421974729	0.991301167	ENSG00000181019	NQO1	
2655.41475	0.144096759	0.184910984	0.779276362	0.435816936	0.991301167	ENSG00000100528	CNIH1	
2608.56583	-0.203288966	0.356313091	-0.570534654	0.568315125	0.99483168	ENSG00000176046	NUPR1	
2627.908842	-0.173168517	0.192455265	-0.89978581	0.368234247	0.981269214	ENSG00000128989	ARPP19	
2596.158642	0.434450918	0.385806948	1.126083707	0.26013009	0.962465655	ENSG00000107738	VSIR	
2629.570454	0.291306438	0.238966844	1.219024501	0.222834893	0.956884001	ENSG00000129128	SPCS3	
2707.516256	0.217800896	0.3848382	0.565954462	0.571424737	0.99483168	ENSG00000144476	ACKR3	
2625.513603	0.047102862	0.205402247	0.229320091	0.818620139	0.999467288	ENSG00000100030	MAPK1	
2630.593344	-0.434934162	0.310048644	-1.402793306	0.160678484	0.941856092	ENSG00000140564	FURIN	

2611.810499	0.097447387	0.184479206	0.528229654	0.59733995	0.99483168	ENSG00000136758	YME1L1
2617.301749	-0.177012543	0.209842433	-0.843549801	0.398921026	0.988378925	ENSG00000179295	PTPN11
2558.768328	0.636190608	0.348240362	1.826872121	0.067718999	0.87420149	ENSG00000000971	CFH
2602.689509	-0.398566044	0.377275768	-1.056431602	0.290771074	0.968990916	ENSG00000122203	KIAA1191
2570.558504	-0.171502957	0.276960486	-0.619232583	0.53576315	0.99483168	ENSG00000122359	ANXA11
2603.268154	-0.067800175	0.195360555	-0.347051504	0.728552626	0.999467288	ENSG00000106263	EIF3B
2560.108325	-0.227163157	0.315240814	-0.720601987	0.471154431	0.99135833	ENSG00000131378	RFTN1
2643.117554	0.131354468	0.364589001	0.360280939	0.718637052	0.99900742	ENSG00000140105	WARS
2609.430518	0.075230583	0.264066898	0.284892138	0.775726779	0.999467288	ENSG00000085662	AKR1B1
2557.424911	-0.250417391	0.240149422	-1.042756586	0.297061043	0.971789954	ENSG00000134590	RTL8C
2604.092882	-0.052182515	0.213802856	-0.244068371	0.807177868	0.999467288	ENSG00000198663	C6orf89
2572.959491	-0.150930923	0.239725186	-0.629599775	0.528956471	0.99483168	ENSG00000135624	CCT7
2600.091138	0.275489917	0.279109711	0.987030928	0.323627477	0.975412329	ENSG00000168214	RBPJ
2605.274573	-0.775798795	0.32849284	-2.36169164	0.018191767	0.726053908	ENSG00000177954	RPS27
2576.475743	-0.303689678	0.270055948	-1.124543562	0.260782501	0.962465655	ENSG00000172667	ZMAT3
2589.83554	-0.122289206	0.236879721	-0.516250212	0.605679665	0.99483168	ENSG00000138801	PAPSS1
2558.264511	-0.087900914	0.183586657	-0.478797945	0.632082378	0.994860874	ENSG00000049245	VAMP3
2597.046272	-0.915643644	0.771825969	-1.186334331	0.235490294	0.962465655	ENSG00000146197	SCUBE3
2595.143949	-0.006610195	0.257340358	-0.025686586	0.979507323	0.999527751	ENSG00000101955	SRPX
2566.939351	-0.063321536	0.179132442	-0.353490054	0.723721084	0.999456245	ENSG00000097033	SH3GLB1
2596.638959	-0.072521301	0.269345599	-0.269249995	0.787737309	0.999467288	ENSG00000103512	NOMO1
2557.571225	0.925786494	0.442322842	2.093010817	0.036348189	0.789688071	ENSG00000116285	ERRFI1
2577.635163	0.166379776	0.210719186	0.789580575	0.429772756	0.991301167	ENSG00000174231	PRPF8
2528.152958	0.422185463	0.287047537	1.470785874	0.141349034	0.935644597	ENSG00000166073	GPR176
2535.554558	-0.101939441	0.263498874	-0.386868602	0.698853487	0.998869317	ENSG00000117592	PRDX6
2545.845164	-0.003019976	0.192124659	-0.015718835	0.987458701	0.999527751	ENSG00000116489	CAPZA1
2546.17356	-0.107941467	0.208972896	-0.516533335	0.605481963	0.99483168	ENSG00000198961	PJA2
2545.966198	0.493663129	0.315069319	1.566839734	0.117152157	0.923922314	ENSG00000188229	TUBB4B
2545.259062	-0.359169189	0.280154341	-1.282040417	0.199828468	0.954310328	ENSG00000134686	PHC2
2535.445508	-0.308268912	0.220116398	-1.400481359	0.161369222	0.942964826	ENSG00000055208	TAB2
2543.55423	0.350225484	0.222395078	1.574789724	0.115304989	0.923922314	ENSG00000086758	HUWE1
2526.945129	0.067821992	0.208903826	0.324656533	0.745441036	0.999467288	ENSG00000143742	SRP9
2495.078012	-0.058033785	0.344845994	-0.168288993	0.866355933	0.999467288	ENSG00000134986	NREP
2526.699973	0.036013529	0.207167821	0.173837464	0.8619932	0.999467288	ENSG00000155096	AZIN1
2484.082377	0.374697481	0.373924576	1.002067007	0.316311231	0.974768742	ENSG00000134824	FADS2
2530.16784	0.200556572	0.19530293	1.026899962	0.304467572	0.973427483	ENSG00000131507	NDFIP1
2527.929445	-0.386035812	0.231605304	-1.66678312	0.095557538	0.921147998	ENSG00000157483	MYO1E
2532.843949	0.330930843	0.31831091	1.039646561	0.298504137	0.972186492	ENSG00000008394	MGST1
2540.47935	0.123345468	0.213486386	0.57776737	0.563421188	0.99483168	ENSG00000108424	KPNB1
2485.541544	-0.343115896	0.310361548	-1.105536103	0.268927359	0.962465655	ENSG00000131171	SH3BGRL
2562.188762	-0.070025826	0.264938788	-0.264309452	0.791541478	0.999467288	ENSG00000151491	EPS8
2543.088562	0.390719343	0.358241526	1.090658996	0.27542296	0.962465655	ENSG00000120889	TNFRSF10B
2550.705651	-0.137332199	0.356278826	-0.385462701	0.699894636	0.998869317	ENSG00000206190	ATP10A
2499.500338	-0.199616467	0.250283997	-0.79755985	0.425125958	0.991301167	ENSG00000166794	PPIB
2483.668052	0.036781044	0.298081116	0.123392736	0.901796109	0.999527751	ENSG00000166557	TMED3
2505.544141	0.26708141	0.202557022	1.318549255	0.187319848	0.949094286	ENSG00000017260	ATP2C1
2497.821383	0.227916848	0.201911933	1.12879335	0.258985019	0.962465655	ENSG00000114554	PLXNA1
2491.492197	0.542162242	0.273587901	1.981674778	0.047515652	0.827997046	ENSG00000185650	ZFP36L1
2474.29206	0.234194411	0.452282023	0.517806145	0.60459353	0.99483168	ENSG00000142949	PTPRF
2417.948921	0.201894821	0.442806865	0.455943295	0.648430755	0.997368416	ENSG00000180447	GAS1
2516.537339	0.23237575	0.23893526	0.972546917	0.330778536	0.976026345	ENSG00000025796	SEC63
2473.433322	0.243835001	0.184931287	1.318516764	0.187330717	0.949094286	ENSG00000165476	REEP3
2454.398524	-0.153318819	0.196228628	-0.781327478	0.434609913	0.991301167	ENSG00000161013	MGAT4B
2450.328128	-0.115077525	0.221418798	-0.519727892	0.603253244	0.99483168	ENSG00000108829	LRRC59
2450.030948	-0.024001008	0.186770951	-0.128505036	0.897749313	0.999467288	ENSG00000120705	ETF1
2447.276156	-0.107159854	0.202808134	-0.528380453	0.597235302	0.99483168	ENSG00000180879	SSR4
2479.221453	0.219886224	0.290751601	0.756268317	0.44948834	0.99135833	ENSG00000038382	TRIO
2477.068159	-0.162892761	0.221394845	-0.735756789	0.461878722	0.99135833	ENSG00000184014	DENND5A
2486.486246	-0.417519079	0.494043805	-0.845105382	0.398052004	0.988378925	ENSG00000105509	HAS1
2469.23638	0.249310835	0.282011585	0.884044656	0.376672107	0.982609538	ENSG00000166986	MARS
2430.56223	0.447780131	0.28796116	1.555001831	0.119945633	0.923922314	ENSG00000128641	MYO1B
2445.568647	-0.212517423	0.194921536	-1.090271639	0.275593504	0.962465655	ENSG00000101367	MAPRE1
2445.333835	0.162222726	0.306409061	0.529431882	0.596505886	0.99483168	ENSG00000189403	HMGB1
2474.228781	-0.194295656	0.342309153	-0.567602867	0.57030466	0.99483168	ENSG00000198380	GFPT1

2420.331006	-0.19444098	0.205096897	-0.948044471	0.343106815	0.977691382	ENSG00000198898	CAPZA2
2427.41412	-0.168671444	0.206779567	-0.815706534	0.414668001	0.990226751	ENSG00000145391	SETD7
2417.732665	-0.606685835	0.206110499	-2.943497967	0.00324526	0.408098513	ENSG00000176788	BASP1
2359.333235	0.719432773	0.427374873	1.683376393	0.09230225	0.921147998	ENSG00000162616	DNAJB4
2426.625515	-0.012774874	0.1958138	-0.065239907	0.947982987	0.999527751	ENSG00000070214	SLC44A1
2392.95357	-0.390109245	0.298469897	-1.307030456	0.191202368	0.949234949	ENSG00000168994	PXDC1
2417.148231	-0.313793998	0.198739993	-1.578917226	0.114355044	0.923922314	ENSG00000105323	HNRNPUL1
2449.87308	0.10317614	0.251433523	0.410351567	0.681548069	0.998869317	ENSG00000176915	ANKLE2
2433.225517	0.236333323	0.273049416	0.865533157	0.386746225	0.986711841	ENSG00000187134	AKR1C1
2433.827256	-0.037506344	0.304930654	-0.122999587	0.902107425	0.999527751	ENSG00000173482	PTPRM
2421.032626	-0.163320057	0.242256008	-0.674163083	0.500207637	0.993513219	ENSG00000115806	GORASP2
2413.102016	0.115461667	0.222239225	0.519537749	0.603385796	0.99483168	ENSG00000148358	GPR107
2401.639488	0.065087536	0.202941554	0.320720594	0.748422139	0.999467288	ENSG00000104695	PPP2CB
2429.044819	-0.442123214	0.317646143	-1.391873391	0.163960741	0.943223078	ENSG00000150907	FOXO1
2399.833189	0.105879034	0.180867607	0.585395225	0.558281999	0.99483168	ENSG00000091527	CDV3
2379.304334	0.050763828	0.248324746	0.20442517	0.838021262	0.999467288	ENSG00000117450	PRDX1
2406.93134	0.182753118	0.271420773	0.673320306	0.500743537	0.993513219	ENSG00000144381	HSPD1
2383.387962	-0.035718077	0.225831243	-0.15816269	0.874328603	0.999467288	ENSG00000070087	PFN2
2388.548027	-0.214498886	0.225945666	-0.94933835	0.342448555	0.977691382	ENSG00000101150	TPD52L2
2395.556187	-0.075959741	0.243247416	-0.31227358	0.754832617	0.999467288	ENSG00000104408	EIF3E
2412.010038	0.038196365	0.26879567	0.14210186	0.886999549	0.999467288	ENSG00000072364	AFF4
2395.449728	-0.095573446	0.218526193	-0.43735465	0.661854168	0.997568624	ENSG00000131724	IL13RA1
2404.028323	0.043038262	0.373514907	0.115225017	0.90826677	0.999527751	ENSG00000182199	SHMT2
2380.133407	-0.251548161	0.228743914	-1.09969335	0.271465753	0.962465655	ENSG00000186591	UBE2H
2417.362137	-0.462878162	0.315727908	-1.466066667	0.142630126	0.935644597	ENSG00000149115	TNKS1BP1
2370.621503	-0.166449123	0.179475281	-0.927420878	0.353708055	0.977691382	ENSG00000196961	AP2A1
2363.795898	-0.153860037	0.206020784	-0.746818036	0.455173407	0.99135833	ENSG00000123240	OPTN
2344.936722	0.139363246	0.275191142	0.506423443	0.612559421	0.99483168	ENSG00000235162	C12orf75
2355.158915	0.008496711	0.440879509	0.019272184	0.984623974	0.999527751	ENSG00000176845	METRNL
2377.602325	-0.078457477	0.24413122	-0.321374206	0.747926828	0.999467288	ENSG00000112531	QKI
2371.492393	1.298245249	0.470500035	2.759288315	0.00579274	0.499617804	ENSG00000197747	S100A10
2356.160887	-0.249034041	0.25094234	-0.99239547	0.321004648	0.975337095	ENSG00000116288	PARK7
2388.165746	0.3291554	0.255320944	1.289182918	0.197334502	0.954310328	ENSG00000139641	ESYT1
2357.766134	-0.296240157	0.206251214	-1.436307457	0.150914873	0.936396974	ENSG00000106153	CHCHD2
2346.297687	0.208572	0.241618555	0.863228404	0.3880119	0.986799595	ENSG00000154978	VOPP1
2362.318302	-0.616615358	0.284078983	-2.17057718	0.029963149	0.773201806	ENSG00000171604	CXXC5
2329.892084	0.183800566	0.421715407	0.435840291	0.662952609	0.997568624	ENSG00000100196	KDELR3
2383.634256	-0.070114961	0.235024084	-0.298330963	0.765450575	0.999467288	ENSG00000112308	C6orf62
2357.672675	-0.249246738	0.295653555	-0.843036499	0.399208031	0.988378925	ENSG00000103502	CDIPT
2349.429672	-0.067729514	0.241495224	-0.280459022	0.77912536	0.999467288	ENSG00000163468	CCT3
2333.159376	-0.226026738	0.202202696	-1.11782257	0.26364278	0.962465655	ENSG00000131100	ATP6V1E1
2348.320887	-0.107545285	0.248332204	-0.43307023	0.66496375	0.997568624	ENSG00000163931	TKT
2397.174112	-0.340755553	0.375420317	-0.907664125	0.364055703	0.98048242	ENSG00000149428	HYOU1
2358.870637	0.090324066	0.210654734	0.428777764	0.668084962	0.997568624	ENSG00000130726	TRIM28
2354.560739	-0.070331615	0.294244769	-0.239024181	0.811086833	0.999467288	ENSG00000008294	SPAG9
2342.987551	-0.002646767	0.226928722	-0.01166343	0.99069414	0.999527751	ENSG00000084754	HADHA
2343.191137	0.058097582	0.225096435	0.258100854	0.796329073	0.999467288	ENSG00000170266	GLB1
2346.756681	0.049651178	0.193323523	0.25682947	0.797310419	0.999467288	ENSG00000134108	ARL8B
2346.732015	0.073550409	0.346253387	0.212417876	0.831781041	0.999467288	ENSG00000140945	CDH13
2318.34653	-0.077485113	0.24658479	-0.31423314	0.753343975	0.999467288	ENSG00000122705	CLTA
2328.193736	-0.330204332	0.195077184	-1.692685555	0.090515336	0.912150398	ENSG00000151135	TMEM263
2328.101973	-0.383719562	0.230715208	-1.663174118	0.096277587	0.921147998	ENSG00000109475	RPL34
2293.025763	-0.405041802	0.773630332	-0.523559877	0.600584694	0.99483168	ENSG00000164125	FAM198B
2309.280244	-0.348153333	0.23346358	-1.491253297	0.135895005	0.934144427	ENSG00000111328	CDK2AP1
2304.458953	-0.197752641	0.204371789	-0.967612223	0.333238071	0.976026345	ENSG00000203879	GDI1
2302.350448	-0.114583695	0.213202673	-0.537440238	0.590963555	0.99483168	ENSG00000126524	SBDS
2317.578916	0.192545057	0.198954322	0.967785244	0.333151635	0.976026345	ENSG00000162736	NCSTN
2311.25951	-0.141845314	0.246449171	-0.575556061	0.564915287	0.99483168	ENSG00000177628	GBA
2287.662813	-0.760744389	0.384650202	-1.977756372	0.047956197	0.830493236	ENSG00000174307	PHLDA3
2314.134005	0.060663251	0.225845007	0.268605679	0.788233142	0.999467288	ENSG00000145817	YIPF5
2293.770801	-0.345777478	0.22715246	-1.522226427	0.127952356	0.928627271	ENSG00000233927	RPS28
2305.532916	-0.092187441	0.191414567	-0.481611418	0.630082014	0.99483168	ENSG00000065000	AP3D1
2308.895196	0.002385614	0.210072672	0.011356135	0.99093931	0.999527751	ENSG00000162434	JAK1
2316.006707	-0.200795782	0.28356909	-0.708101796	0.478882044	0.99135833	ENSG00000113657	DPYSL3

2276.280455	0.323175186	0.241728559	1.336934237	0.181244104	0.949094286	ENSG00000165915	SLC39A13
2277.835265	-0.256990441	0.229906939	-1.117802017	0.26365156	0.962465655	ENSG00000065154	OAT
2278.429945	0.482216612	0.223688743	2.155748226	0.03110333	0.775963115	ENSG00000101856	PGRMC1
2282.641016	0.033526525	0.273643014	0.122519207	0.902487835	0.999527751	ENSG00000163479	SSR2
2266.827105	-0.128686378	0.2027841	-0.634597968	0.525690654	0.99483168	ENSG00000159346	ADIPOR1
2263.108546	-0.098549102	0.204177675	-0.482663457	0.629334714	0.99483168	ENSG00000010256	UQCRC1
2263.196103	-0.193833638	0.209571735	-0.924903534	0.355016088	0.977691382	ENSG00000138107	ACTR1A
2279.6046	0.249127653	0.278923021	0.893177093	0.371762343	0.981955904	ENSG00000134851	TMEM165
2222.112462	-0.605152973	0.244358263	-2.476498918	0.013267803	0.670420654	ENSG00000173641	HSPB7
2294.274202	0.242653573	0.22367173	1.084864735	0.277981569	0.962465655	ENSG00000100201	DDX17
2281.590803	0.277877878	0.244865813	1.134816965	0.256452017	0.962465655	ENSG00000159140	SON
2246.071199	-0.121823996	0.245087354	-0.497063573	0.619144221	0.99483168	ENSG00000160014	CALM3
2255.394318	-0.221012041	0.208452009	-1.06025383	0.289029138	0.966307369	ENSG00000135916	ITM2C
2253.790383	-0.020704241	0.240236428	-0.08618277	0.931321127	0.999527751	ENSG00000136295	TTYH3
2245.13357	-0.042077886	0.228851281	-0.183865632	0.854118866	0.999467288	ENSG00000074416	MGLL
2246.777713	-0.400371132	0.24674797	-1.62259139	0.104676782	0.92169898	ENSG00000126934	MAP2K2
2268.788291	0.162413683	0.219247535	0.740777694	0.458828242	0.99135833	ENSG00000118762	PKD2
2235.614473	-0.195353731	0.240487388	-0.812324224	0.416605614	0.990226751	ENSG00000159377	PSMB4
2260.668719	0.116127439	0.232715783	0.499009725	0.617772533	0.99483168	ENSG00000159461	AMFR
2266.617688	-0.147816366	0.271550892	-0.544341302	0.58620663	0.99483168	ENSG00000171862	PTEN
2252.313155	-0.043898964	0.180246521	-0.243549579	0.807579682	0.999467288	ENSG00000135018	UBQLN1
2261.301488	-0.088199382	0.234813406	-0.375614765	0.707203313	0.99900742	ENSG00000108582	CPD
2241.159349	-0.663117215	0.308914498	-2.146604382	0.031824791	0.775963115	ENSG00000167468	GPX4
2231.402799	-0.111623905	0.19652506	-0.567988147	0.570043016	0.99483168	ENSG00000129925	TMEM8A
2254.74266	0.35550798	0.205145967	1.73295135	0.083104316	0.901307093	ENSG00000170876	TMEM43
2262.35336	-0.300500663	0.226731398	-1.325359728	0.185051867	0.949094286	ENSG00000106723	SPIN1
2243.257314	-0.282166135	0.241236303	-1.169666967	0.242135017	0.962465655	ENSG00000142208	AKT1
2205.671857	-0.480889325	0.408019884	-1.178592867	0.238560333	0.962465655	ENSG00000133142	TCEAL4
2216.883857	0.096119076	1.051693879	0.091394538	0.927179102	0.999527751	ENSG00000172061	LRRC15
2250.48214	-0.182550099	0.295356281	-0.618067435	0.536530887	0.99483168	ENSG00000174705	SH3PXD2B
2226.643193	-0.029925601	0.256881369	-0.116495803	0.907259613	0.999527751	ENSG00000180628	PCGF5
2240.748393	-0.074511071	0.199769126	-0.372985918	0.709158925	0.99900742	ENSG00000138768	USO1
2214.646718	-0.064694771	0.265860488	-0.243341052	0.807741205	0.999467288	ENSG00000102804	TSC22D1
2239.430757	-0.456869471	0.218384444	-2.092042193	0.036434742	0.789688071	ENSG00000117122	MFAP2
2291.273428	-0.365135364	0.397664491	-0.91819957	0.358514394	0.979427314	ENSG00000139508	SLC46A3
2269.263098	0.090490812	0.334422168	0.270588556	0.786707498	0.999467288	ENSG00000155304	HSPA13
2269.269502	-0.208415482	0.334029239	-0.623943828	0.532664475	0.99483168	ENSG00000135269	TES
2241.264505	0.059695874	0.213354574	0.279796553	0.779633596	0.999467288	ENSG00000120451	SNX19
2228.337476	-0.422686963	0.191528474	-2.20691448	0.027320035	0.768732301	ENSG00000146535	GNA12
2299.278732	-1.06900706	0.462647912	-2.310627653	0.02085343	0.73997555	ENSG00000162591	MEGF6
2220.305475	0.120224538	0.235082088	0.511415137	0.609060398	0.99483168	ENSG00000148700	ADD3
2208.939668	0.142031908	0.242006554	0.586892815	0.557275696	0.99483168	ENSG00000182240	BACE2
2243.009135	0.293131574	0.235060713	1.247046226	0.212380549	0.954310328	ENSG00000166479	TMX3
2213.524019	0.315294426	0.219597033	1.435786364	0.151063141	0.936396974	ENSG00000113583	C5orf15
2199.436643	-0.552672759	0.260430507	-2.122150607	0.033825093	0.777923272	ENSG00000103187	COTL1
2209.869746	0.088090749	0.276315441	0.318805016	0.749874374	0.999467288	ENSG00000132155	RAF1
2199.15317	-0.03859467	0.280562931	-0.137561545	0.890586949	0.999467288	ENSG00000112078	KCTD20
2233.603425	0.083395021	0.249072312	0.334822526	0.737758967	0.999467288	ENSG00000237550	RPL9P9
2214.657754	0.720040114	0.365874206	1.967999118	0.049068139	0.835720678	ENSG00000151914	DST
2153.045244	-0.483897546	0.374317741	-1.292745424	0.196099122	0.954310328	ENSG00000120738	EGR1
2210.433519	-0.062242988	0.183169671	-0.339810557	0.733999198	0.999467288	ENSG00000114354	TFG
2201.07307	0.160180726	0.212237839	0.75472275	0.450415353	0.99135833	ENSG00000086289	EPDR1
2188.319981	0.082700052	0.244660976	0.338018972	0.735348891	0.999467288	ENSG00000160209	PDXK
2219.645926	-0.108737401	0.218416961	-0.497843209	0.618594558	0.99483168	ENSG00000158195	WASF2
2196.067055	-0.037773525	0.18897585	-0.19988546	0.841570162	0.999467288	ENSG00000089737	DDX24
2216.23808	-0.090987416	0.28941333	-0.314385712	0.753228106	0.999467288	ENSG00000164741	DLC1
2176.645088	0.105036651	0.174756267	0.601046549	0.547808983	0.99483168	ENSG00000072778	ACADVL
2185.329335	-0.317486277	0.199395932	-1.592240491	0.111330678	0.923922314	ENSG00000169567	HINT1
2199.927189	0.043147377	0.191960454	0.224772216	0.822156481	0.999467288	ENSG00000151239	TWF1
2208.681576	-0.423048356	0.226696281	-1.866145992	0.062020943	0.865910462	ENSG00000177425	PAWR
2169.010616	-0.017770918	0.269854442	-0.065853717	0.947494289	0.999527751	ENSG00000111229	ARPC3
2195.695066	0.088047084	0.249783031	0.352494257	0.724467624	0.999456245	ENSG00000116044	NFE2L2
2182.021844	-0.040259964	0.185917461	-0.216547516	0.828560991	0.999467288	ENSG00000007202	KIAA0100
2195.49111	0.274181407	0.238279689	1.150670489	0.249867822	0.962465655	ENSG00000115524	SF3B1

2179.199031	-0.256146626	0.41906114	-0.611239271	0.541041188	0.99483168	ENSG00000198732	SMOC1
2164.915534	-0.128569246	0.320193627	-0.401535932	0.688025589	0.998869317	ENSG00000123143	PKN1
2179.287476	-0.049138734	0.17350701	-0.283208924	0.777016688	0.999467288	ENSG00000204469	PRRC2A
2195.492829	-0.395704749	0.263694989	-1.500615355	0.133455078	0.933741583	ENSG00000177733	HNRNPA0
2180.423152	-0.27378528	0.197925554	-1.383274037	0.166580856	0.943223078	ENSG00000174695	TMEM167A
2172.370674	-0.391622721	0.359009588	-1.090841956	0.275342431	0.962465655	ENSG00000022267	FHL1
2159.727018	-0.271610348	0.206084902	-1.317953648	0.187519165	0.949094286	ENSG00000115234	SNX17
2163.517698	0.200467196	0.242396409	0.827022136	0.408224515	0.990226751	ENSG00000137207	YIPF3
2172.494129	0.128923421	0.168867711	0.763458095	0.445190245	0.99135833	ENSG00000101558	VAPA
2135.418822	0.500556476	0.476805778	1.049812101	0.29380451	0.970706863	ENSG00000164442	CITED2
2159.428479	0.074002909	0.224486714	0.329653845	0.741661533	0.999467288	ENSG00000119777	TMEM214
2151.363709	-0.159239983	0.215615774	-0.738535869	0.460188879	0.99135833	ENSG00000183283	DAZAP2
2176.590455	-0.067362516	0.227895534	-0.295585064	0.767546981	0.999467288	ENSG00000168906	MAT2A
2148.702864	0.034737413	0.242474899	0.143261892	0.886083351	0.999467288	ENSG00000129103	SUMF2
2190.765649	0.398966339	0.377058499	1.058101965	0.290008959	0.967933358	ENSG00000139514	SLC7A1
2147.47906	0.024424175	0.22315595	0.109448908	0.912846444	0.999527751	ENSG00000164292	RHOBTB3
2146.106015	0.013576664	0.173975696	0.078037705	0.93779806	0.999527751	ENSG00000078618	NRDC
2154.742936	0.247041757	0.239970672	1.029466455	0.303260535	0.972793733	ENSG00000137509	PRCP
2156.991959	0.026189575	0.262544813	0.09975278	0.920540597	0.999527751	ENSG00000155876	RRAGA
2180.007655	-0.579497741	0.418747508	-1.383883437	0.166394149	0.943223078	ENSG00000134030	CTIF
2140.271546	0.403596425	0.29628377	1.362195521	0.173136189	0.944422693	ENSG00000092964	DPYSL2
2154.69776	-0.039598476	0.300619158	-0.131723061	0.895203345	0.999467288	ENSG00000109929	SC5D
2154.448848	-0.098461376	0.200739836	-0.490492462	0.623785462	0.99483168	ENSG00000135956	TMEM127
2165.578122	-0.224646467	0.209242259	-1.07361901	0.282993474	0.963267117	ENSG00000137710	RDX
2140.813105	0.06425186	0.261088729	0.246092047	0.805610979	0.999467288	ENSG00000138944	SHISAL1
2119.725482	0.210126914	0.422347878	0.497520943	0.618821738	0.99483168	ENSG00000197381	ADARB1
2151.981572	0.362787649	0.262510879	1.381990912	0.166974491	0.943223078	ENSG00000074696	HACD3
2140.93556	-0.127332281	0.337700502	-0.377056832	0.706131371	0.99900742	ENSG00000048392	RRM2B
2118.513741	0.310941083	0.30287086	1.026645757	0.3045873	0.973427483	ENSG00000122870	BICC1
2116.61354	-0.265419064	0.456866253	-0.580955722	0.561270296	0.99483168	ENSG00000106538	RARRES2
2159.626885	-0.075887156	0.328161436	-0.231249464	0.817121004	0.999467288	ENSG00000168056	LTBP3
2160.934264	0.237990255	0.472054882	0.50415802	0.614150338	0.99483168	ENSG00000133639	BTG1
2134.046605	0.562281749	0.298954258	1.880828701	0.059995225	0.858996918	ENSG00000062716	VMP1
2119.920807	-0.413757408	0.311037743	-1.330248234	0.183436497	0.949094286	ENSG00000072163	LIMS2
2124.675528	0.071893215	0.254340355	0.282665385	0.777433353	0.999467288	ENSG00000134684	YARS
2156.16694	0.301169534	0.305259101	0.986602965	0.323837316	0.975412329	ENSG00000182158	CREB3L2
2096.698801	-0.033721518	0.293987833	-0.114703788	0.908679911	0.999527751	ENSG00000110811	P3H3
2107.687939	0.112468126	0.265034738	0.424352396	0.671308832	0.997568624	ENSG00000156261	CCT8
2146.945315	-0.294147742	0.266835365	-1.102356662	0.270306645	0.962465655	ENSG00000132294	EFR3A
2123.851273	-0.300486717	0.279904664	-1.073532367	0.283032325	0.963267117	ENSG00000100241	SBF1
2104.012925	-0.186939129	0.210637407	-0.887492548	0.374813776	0.982609538	ENSG00000146701	MDH2
2124.154616	-0.358481829	0.23686093	-1.513469651	0.130160404	0.929373153	ENSG00000120742	SERP1
2143.115909	0.203571693	0.393135419	0.517815702	0.604586861	0.99483168	ENSG00000150630	VEGFC
2104.761441	0.139886255	0.205533702	0.680600083	0.496124574	0.993513219	ENSG00000164754	RAD21
2097.296388	0.088734485	0.223775255	0.396533949	0.691711169	0.998869317	ENSG00000177706	FAM20C
2112.392011	0.178983791	0.217494434	0.82293504	0.410544935	0.990226751	ENSG00000103342	GSPT1
2133.578445	-0.120970342	0.371041813	-0.326028866	0.744402515	0.999467288	ENSG00000198053	SIRPA
2072.076965	0.085702807	0.33525411	0.255635366	0.798232407	0.999467288	ENSG00000188042	ARL4C
2097.777057	-0.140703501	0.182129168	-0.772547873	0.439790004	0.991301167	ENSG00000084623	EIF3I
2109.853973	0.088614019	0.1742638	0.50850503	0.611099213	0.99483168	ENSG00000196914	ARHGEF12
2104.914074	-0.281730676	0.216521453	-1.301167494	0.193201129	0.950382812	ENSG00000198242	RPL23A
2091.819396	0.608308831	0.481922974	1.262253231	0.20685768	0.954310328	ENSG00000167553	TUBA1C
2096.879464	0.071545799	0.317204854	0.225550769	0.821550835	0.999467288	ENSG00000090861	AARS
2094.70653	0.108959739	0.188523026	0.57796515	0.563287648	0.99483168	ENSG00000129636	ITFG1
2089.786749	-0.039682477	0.227917899	-0.174108649	0.861780076	0.999467288	ENSG00000143162	CREG1
2111.617502	-0.420402121	0.317640757	-1.323514415	0.185664366	0.949094286	ENSG00000171298	GAA
2084.111465	-0.122014027	0.188256439	-0.648126711	0.516902999	0.993513219	ENSG00000147677	EIF3H
2107.463871	-0.233396606	0.20528014	-1.136966324	0.255552364	0.962465655	ENSG00000162512	SDC3
2073.116797	-0.047905781	0.228638673	-0.209526151	0.834037523	0.999467288	ENSG00000110906	KCTD10
2076.933359	-0.046584893	0.222078882	-0.209767326	0.833849275	0.999467288	ENSG00000143321	HDGF
2089.542608	0.524432828	0.274068526	1.913509865	0.055682812	0.847598083	ENSG00000169715	MT1E
2079.890555	0.162786299	0.270842479	0.601036808	0.547815471	0.99483168	ENSG00000145012	LPP
2072.187037	0.161511019	0.248704162	0.649410196	0.516073275	0.993513219	ENSG00000117298	ECE1
2093.82409	0.337651044	0.333536578	1.012335877	0.311377486	0.973427483	ENSG00000144320	LNPK

2084.010365	0.067654563	0.236963888	0.285505795	0.775256665	0.999467288	ENSG00000167323	STIM1
2081.79776	0.108842829	0.242352764	0.449109088	0.653352965	0.997527562	ENSG00000169180	XPO6
2081.275395	-0.056906814	0.265498893	-0.214339177	0.830282569	0.999467288	ENSG00000145860	RNF145
2080.029435	-0.227875419	0.186926411	-1.219064856	0.222819577	0.956884001	ENSG00000115216	NRBP1
2062.441185	-0.123126601	0.195024127	-0.631340353	0.527818007	0.99483168	ENSG00000204463	BAG6
2047.226863	-0.081519441	0.311351872	-0.261824155	0.793457016	0.999467288	ENSG00000162614	NEXN
2085.261383	0.194683856	0.300456861	0.64795943	0.517011191	0.993513219	ENSG00000124831	LRRFIP1
2081.397635	-0.132056533	0.232818867	-0.567207181	0.57057343	0.99483168	ENSG00000152102	FAM168B
2072.888717	0.000798832	0.182458421	0.004378159	0.996506745	0.999527751	ENSG00000090060	PAPOLA
2095.141727	-0.348156544	0.308487876	-1.128590688	0.259070541	0.962465655	ENSG00000197555	SIPA1L1
2059.062717	-0.261153911	0.2614907	-0.998712042	0.317934206	0.974768742	ENSG00000165757	JCAD
2059.727352	0.020653821	0.188452947	0.109596701	0.912729227	0.999527751	ENSG00000119396	RAB14
2067.743472	0.193278906	0.187604134	1.03024865	0.302893297	0.972793733	ENSG00000107863	ARHGAP21
2075.567227	-0.119136012	0.244237745	-0.487787061	0.625700677	0.99483168	ENSG00000013288	MAN2B2
2056.425374	-0.041793027	0.177664747	-0.235235337	0.814026081	0.999467288	ENSG00000165637	VDAC2
2156.1189	0.156315004	1.146411553	0.136351561	0.891543365	0.999467288	ENSG00000101255	TRIB3
2023.68644	-0.430034465	0.263366776	-1.632834906	0.102503724	0.92169898	ENSG00000198832	SELENOM
2068.570388	0.278854492	0.225741473	1.235282505	0.216725382	0.954900762	ENSG00000144724	PTPRG
2050.039106	0.028441465	0.275931251	0.103074459	0.917903876	0.999527751	ENSG00000068912	ERLEC1
2049.551389	0.032456332	0.257272328	0.126155548	0.899608798	0.999527751	ENSG00000146731	CCT6A
2034.771783	-0.187378747	0.24982138	-0.750050882	0.45322406	0.99135833	ENSG00000148484	RSU1
2044.214745	-0.243982579	0.199814131	-1.221047669	0.222067972	0.956884001	ENSG00000113384	GOLPH3
2039.068539	0.195660817	0.211908413	0.923327274	0.355836679	0.978364583	ENSG00000147526	TACC1
2044.998649	-0.056567578	0.219684019	-0.25749519	0.796796528	0.999467288	ENSG00000012660	ELOVL5
2044.851825	-0.073794022	0.199271432	-0.370319122	0.711144728	0.99900742	ENSG00000090621	PABPC4
2080.383793	-1.234954259	0.491630964	-2.511953779	0.012006481	0.648356243	ENSG00000101665	SMAD7
2019.933811	-0.130900184	0.230419038	-0.568096218	0.569969636	0.99483168	ENSG00000185088	RPS27L
2025.915208	-0.041177622	0.195018176	-0.211147609	0.832772091	0.999467288	ENSG00000014216	CAPN1
1989.752465	0.494052529	0.431021238	1.146237091	0.251697065	0.962465655	ENSG00000204381	LAYN
2024.111995	-0.340123158	0.255330113	-1.332091832	0.182830014	0.949094286	ENSG00000117411	B4GALT2
2049.722391	-0.079427471	0.219026379	-0.362638834	0.716874697	0.99900742	ENSG00000225733	FGD5-AS1
2039.597237	-0.364384223	0.24194525	-1.506060662	0.132051602	0.932626545	ENSG00000154122	ANKH
2021.46844	0.166271079	0.26187449	0.634926598	0.525476289	0.99483168	ENSG00000153071	DAB2
2037.65801	-0.006399438	0.184827707	-0.034623802	0.972379722	0.999527751	ENSG00000168066	SF1
2026.913171	-0.10627088	0.203882155	-0.521236789	0.602201829	0.99483168	ENSG00000143418	CERS2
2038.758964	-0.39746142	0.235527528	-1.687537011	0.09150014	0.915903452	ENSG00000173457	PPP1R14B
2009.723178	-0.041920986	0.200681888	-0.208892723	0.834531985	0.999467288	ENSG00000204843	DCTN1
2019.683925	-0.098370577	0.186292357	-0.528044084	0.597468739	0.99483168	ENSG00000188554	NBR1
2024.727033	0.034902235	0.229252743	0.152243476	0.878994905	0.999467288	ENSG00000095787	WAC
2029.516388	-0.121639836	0.192048338	-0.633381352	0.52648464	0.99483168	ENSG00000087365	SF3B2
2013.85209	-0.070885895	0.191911704	-0.369367232	0.711854019	0.99900742	ENSG00000135720	DYNC1LI2
1993.919106	-0.004122145	0.286055336	-0.014410305	0.988502638	0.999527751	ENSG00000231991	ANXA2P2
1998.539242	-0.141241728	0.192568563	-0.733462024	0.463276682	0.99135833	ENSG00000157593	SLC35B2
2001.067561	-0.430241753	0.308815977	-1.393197843	0.163559972	0.943223078	ENSG00000167291	TBC1D16
2020.861623	0.201029701	0.18995419	1.058306221	0.289915858	0.967933358	ENSG00000144224	UBXN4
1999.674654	-0.273907799	0.222192054	-1.23275245	0.217668137	0.956884001	ENSG00000184900	SUMO3
2009.876889	0.172833157	0.334464307	0.516746194	0.605333345	0.99483168	ENSG00000135047	CTSL
1998.774167	-0.337434148	0.201927943	-1.671062179	0.094709397	0.921147998	ENSG00000106636	YKT6
2038.891653	-0.114640028	0.391891463	-0.292530047	0.76988139	0.999467288	ENSG00000196365	LONP1
1999.591052	-0.131426786	0.202641828	-0.648566917	0.516618345	0.993513219	ENSG00000105993	DNAJB6
1983.330916	-0.019836541	0.222289308	-0.089237494	0.928893169	0.999527751	ENSG00000165410	CFL2
2006.66662	-0.037157909	0.235988667	-0.157456327	0.874885224	0.999467288	ENSG00000059573	ALDH18A1
2005.973198	0.21213511	0.23412263	0.906085459	0.364890621	0.981216764	ENSG00000135316	SYNCRIP
2011.398163	0.027590161	0.216362542	0.127518195	0.898530273	0.999490224	ENSG00000111530	CAND1
1997.24528	0.251155392	0.243682716	1.030665596	0.302697663	0.972793733	ENSG00000134153	EMC7
1977.628508	-0.185278432	0.232461224	-0.79702941	0.425433951	0.991301167	ENSG00000170004	CHD3
2021.257479	-0.070529444	0.353223453	-0.199673729	0.841735761	0.999467288	ENSG00000136960	ENPP2
2052.644929	-0.608462246	0.500539034	-1.215613978	0.224132007	0.956884001	ENSG00000171793	CTPS1
1958.421292	0.064959026	0.354393682	0.183296232	0.854565591	0.999467288	ENSG00000132386	SERPINF1
1973.906602	-0.235008217	0.219226765	-1.071986888	0.283725921	0.963310634	ENSG00000157020	SEC13
2007.479292	-0.020107675	0.258281845	-0.077851678	0.937946038	0.999527751	ENSG00000165527	ARF6
1979.674349	-0.262203155	0.215510894	-1.216658474	0.223734185	0.956884001	ENSG00000119729	RHOQ
1995.474863	-0.104858946	0.186688588	-0.561678391	0.57433516	0.99483168	ENSG00000099246	RAB18
1998.946847	-0.098150598	0.242614737	-0.404553324	0.685805874	0.998869317	ENSG00000081154	PCNP

1994.136874	0.395505413	0.311541043	1.269513028	0.204258147	0.954310328	ENSG00000057019	DCBLD2
1993.184996	-0.325362578	0.179460309	-1.813005787	0.069830929	0.878035218	ENSG00000188643	S100A16
1980.938952	0.111370607	0.222980568	0.499463285	0.617453046	0.99483168	ENSG00000170606	HSPA4
1988.466251	-0.131500775	0.199397551	-0.65949042	0.509580896	0.993513219	ENSG00000091039	OSBPL8
1975.229992	-0.163420935	0.195593085	-0.835514887	0.403427869	0.988927059	ENSG00000018408	WWTR1
2004.119746	-0.323811626	0.327760183	-0.98795291	0.323175711	0.975337095	ENSG00000183688	RFLNB
1970.179343	0.276476774	0.26693254	1.035755229	0.300316349	0.972186492	ENSG00000118855	MFSD1
2017.620251	-0.101407455	0.369014112	-0.274806443	0.783464948	0.999467288	ENSG00000136603	SKIL
1974.31308	0.312751283	0.251743757	1.24233978	0.214111224	0.954310328	ENSG00000123983	ACSL3
1965.398537	-0.265563131	0.177668173	-1.494714145	0.134989061	0.933741583	ENSG00000009335	UBE3C
1942.097757	-0.633686744	0.382521546	-1.656604054	0.097599552	0.921147998	ENSG00000173511	VEGFB
1964.04355	-0.01833486	0.180156424	-0.101771892	0.918937737	0.999527751	ENSG00000153250	RBMS1
1958.614841	0.304596016	0.271873931	1.120357564	0.262561421	0.962465655	ENSG00000149131	SERPING1
1958.312811	-0.145376841	0.257146117	-0.565347213	0.571837621	0.99483168	ENSG00000159202	UBE2Z
1963.013736	-0.308651781	0.204439278	-1.509747947	0.131107752	0.930053663	ENSG00000148730	EIF4EBP2
1945.348535	0.058406209	0.319297564	0.182920936	0.854860055	0.999467288	ENSG00000119138	KLF9
1946.579786	0.096141976	0.219876236	0.437254967	0.661926451	0.997568624	ENSG00000164054	SHISA5
1995.271577	-0.693823231	0.249185171	-2.784368061	0.005363214	0.487175435	ENSG00000084636	COL16A1
1951.951051	0.02871556	0.202887554	0.141534359	0.887447818	0.999467288	ENSG00000113575	PPP2CA
1939.397424	0.543175621	0.463127371	1.172842842	0.240858834	0.962465655	ENSG00000159399	HK2
1938.721016	-0.04748123	0.281501677	-0.168671214	0.866055262	0.999467288	ENSG00000014641	MDH1
1923.255109	-0.229724284	0.257210174	-0.893138404	0.371783059	0.981955904	ENSG00000254999	BRK1
1969.671887	0.137253112	0.270435747	0.507525775	0.611785956	0.99483168	ENSG00000127463	EMC1
1933.270137	-0.026361391	0.249591831	-0.105618002	0.915885441	0.999527751	ENSG00000141696	P3H4
1933.758343	-0.173461825	0.213219695	-0.813535662	0.415911009	0.990226751	ENSG00000147416	ATP6V1B2
1936.414057	-0.106215852	0.181025012	-0.58674683	0.557373751	0.99483168	ENSG00000110696	C11orf58
1939.315531	-0.10081456	0.194996206	-0.5170078	0.605150714	0.99483168	ENSG00000100320	RBFOX2
1931.760577	-0.294610189	0.346030598	-0.851399241	0.394547613	0.988378925	ENSG00000005022	SLC25A5
1950.638578	0.0262668	0.246401863	0.106601468	0.915105154	0.999527751	ENSG00000102580	DNAJC3
1944.418239	0.38886256	0.299087196	1.300164518	0.193544589	0.950382812	ENSG00000105976	MET
1955.300477	0.231626125	0.306867959	0.754807135	0.450364712	0.99135833	ENSG00000140526	ABHD2
1925.633308	0.331344293	0.250211915	1.324254656	0.185418484	0.949094286	ENSG00000117335	CD46
1926.966884	-0.910398101	0.213630457	-4.26155574	2.03E-05	0.028520548	ENSG00000158186	MRAS
1937.955517	0.044699592	0.207656294	0.215257584	0.829566498	0.999467288	ENSG00000027697	IFNGR1
1926.403014	0.083244241	0.207051755	0.402045572	0.687650488	0.998869317	ENSG00000165102	HGSNAT
1918.523556	0.009638078	0.282712941	0.034091395	0.97280427	0.999527751	ENSG00000184371	CSF1
1925.814107	-0.106397962	0.375830744	-0.283100741	0.777099614	0.999467288	ENSG00000074590	NUAK1
1934.482894	0.135615431	0.296176493	0.457887218	0.64703347	0.997368416	ENSG00000141458	NPC1
1921.311804	-0.112290761	0.203783808	-0.551028869	0.581613885	0.99483168	ENSG00000136802	LRRC8A
1909.168067	0.152079856	0.248329479	0.61241161	0.540265462	0.99483168	ENSG00000198911	SREBF2
1923.610049	0.063058096	0.218650524	0.288396731	0.77304307	0.999467288	ENSG00000056097	ZFR
1895.832526	-0.285970499	0.386564067	-0.739775172	0.459436427	0.99135833	ENSG00000138623	SEMA7A
1909.630121	-0.422729104	0.218660477	-1.933267093	0.053203303	0.847598083	ENSG00000283041	AC008038.1
1896.022021	-0.057505474	0.219819161	-0.261603557	0.793627102	0.999467288	ENSG00000198792	TMEM184B
1916.735399	-0.136837059	0.281857288	-0.485483486	0.627333424	0.99483168	ENSG00000107331	ABCA2
1882.953994	-0.466028486	0.202935652	-2.296434767	0.021651035	0.73997555	ENSG00000107130	NCS1
1902.936014	0.249129684	0.186583647	1.335217143	0.181805288	0.949094286	ENSG00000145907	G3BP1
1891.249849	-0.182452656	0.236235003	-0.772335401	0.439915803	0.99135833	ENSG00000084090	STARD7
1899.594442	-0.000553522	0.179800423	-0.003078536	0.997543687	0.999527751	ENSG00000129515	SNX6
1891.045133	-0.098065681	0.250142031	-0.392039999	0.69502866	0.998869317	ENSG00000116459	ATP5PB
1900.797195	0.011800054	0.193897113	0.060857297	0.951472859	0.999527751	ENSG00000069329	VPS35
1885.726093	-0.029454543	0.273964413	-0.107512297	0.91438257	0.999527751	ENSG00000104332	SFRP1
1904.455485	-0.026022566	0.197809629	-0.131553584	0.895337401	0.999467288	ENSG00000169710	FASN
1893.849499	0.035154202	0.283813002	0.12386396	0.901422989	0.999527751	ENSG00000101474	APMAP
1927.924984	0.235428993	0.314069498	0.749607952	0.45349086	0.99135833	ENSG00000144136	SLC20A1
1892.708738	0.396317575	0.233159467	1.699770459	0.08917411	0.910413547	ENSG00000148175	STOM
1897.966461	-0.03303582	0.19382476	-0.170441689	0.864662788	0.999467288	ENSG00000162923	WDR26
1887.504798	-0.333105868	0.247659084	-1.345017769	0.178619496	0.9469453	ENSG00000161202	DVL3
1885.044493	-0.256003836	0.210423942	-1.216609829	0.223752701	0.956884001	ENSG00000119801	YPEL5
1916.221315	0.595588536	0.372722467	1.597941065	0.110056094	0.923922314	ENSG00000092621	PHGDH
1900.084578	-0.201121067	0.316106106	-0.636245437	0.524616462	0.99483168	ENSG00000064042	LIMCH1
1896.288028	0.662000894	0.330448855	2.003338439	0.045140974	0.813244956	ENSG00000170540	ARL6IP1
1887.366499	0.161676705	0.213108883	0.758657745	0.44805732	0.99135833	ENSG00000140612	SEC11A
1888.284888	0.049369116	0.318447396	0.155030678	0.876797132	0.999467288	ENSG00000151632	AKR1C2

1894.829517	0.12336754	0.325498474	0.379011115	0.70467961	0.99900742	ENSG00000130779	CLIP1		
1842.289674	-0.222499064	0.379033004	-0.58701765	0.557191853	0.99483168	ENSG00000131389	SLC6A6		
1850.089705	-0.282049655	0.275893489	-1.022313561	0.306632517	0.973427483	ENSG00000089220	PEBP1		
1869.589204	-0.130512333	0.227463629	-0.573772315	0.566121886	0.99483168	ENSG00000178952	TUFM		
1869.386944	0.241628734	0.236139193	1.023247055	0.30619105	0.973427483	ENSG00000076716	GPC4		
1885.354524	0.206592152	0.204876796	1.008372622	0.313275611	0.973427483	ENSG00000127947	PTPN12		
1873.235988	-0.090426401	0.183806534	-0.491965107	0.622744011	0.99483168	ENSG00000070367	EXOC5		
1865.786869	-0.116902964	0.200124739	-0.584150486	0.559119071	0.99483168	ENSG00000124214	STAU1		
1868.816211	-0.350990627	0.24365887	-1.4405001	0.149725962	0.936396974	ENSG00000169564	PCBP1		
1873.583336	0.118616525	0.246647147	0.48091586	0.6305763	0.99483168	ENSG00000151726	ACSL1		
1834.295575	1.248889182	0.295355619	4.228425339	2.35E-05	0.028520548	ENSG00000170961	HAS2		
1924.626088	-1.480963073	0.796894038	-1.858419066	0.063109527	0.868202292	ENSG00000171223	JUNB		
1869.670288	0.094799233	0.213048845	0.444964783	0.656345205	0.997568624	ENSG00000065135	GNAI3		
1872.745909	0.018172203	0.187666572	0.09683239	0.922859501	0.999527751	ENSG00000132463	GRSF1		
1888.241306	-0.114646998	0.259623485	-0.441589472	0.658786304	0.997568624	ENSG00000163297	ANTXR2		
1866.86617	-0.05809471	0.229162632	-0.253508651	0.799875177	0.999467288	ENSG00000166226	CCT2		
1863.554651	0.09484026	0.18975238	0.499810646	0.617208414	0.99483168	ENSG00000129351	ILF3		
1871.279056	-0.561662673	0.35733235	-1.571821509	0.115991952	0.923922314	ENSG00000210140	MT-TC		
1849.624674	-0.212129896	0.21602389	-0.981974245	0.326112537	0.976026345	ENSG00000167193	CRK		
1859.15273	0.297240787	0.224550149	1.32371672	0.185597143	0.949094286	ENSG00000143183	TMCO1		
1876.045255	-0.769413696	0.223311793	-3.445468259	0.000570071	0.195790358	ENSG00000128283	CDC42EP1		
1833.676127	-0.282324586	0.469361876	-0.601507281	0.547502163	0.99483168	ENSG00000162551	ALPL		
1836.43055	-0.155442577	0.505294066	-0.307627949	0.75836545	0.999467288	ENSG00000120549	KIAA1217		
1856.932114	-0.333038114	0.196707977	-1.693058507	0.090444331	0.912150398	ENSG00000182446	NPLOC4		
1849.867475	-0.031235325	0.250483454	-0.124700151	0.900760938	0.999527751	ENSG00000182087	TMEM259		
1852.673572	-0.350290352	0.31522796	-1.111228688	0.266469926	0.962465655	ENSG00000172216	CEBPB		
1865.016053	0.159553399	0.217621661	0.733168743	0.463455517	0.99135833	ENSG00000090054	SPTLC1		
1858.923927	-0.003637472	0.189152527	-0.019230363	0.984657336	0.999527751	ENSG00000102753	KPNA3		
1872.738609	-0.192103754	0.396045797	-0.485054393	0.627637762	0.99483168	ENSG00000091542	ALKBH5		
1837.367922	-0.189059908	0.267730604	-0.706157256	0.480090349	0.99135833	ENSG00000155324	GRAMD2B		
1875.034365	0.2463217	0.522098533	0.471791597	0.637075549	0.99653524	ENSG00000135766	EGLN1		
1859.299555	0.062079984	0.208668975	0.297504622	0.76608128	0.999467288	ENSG00000163531	NFASC		
1869.575565	-0.76787372	0.379706831	-2.022280498	0.043147382	0.806698895	ENSG00000124766	SOX4		
1845.684297	-0.019964532	0.250943862	-0.07955776	0.936588991	0.999527751	ENSG00000115484	CCT4		
1838.743727	-0.265110772	0.192837897	-1.374785641	0.169197911	0.944252485	ENSG00000111540	RAB5B		
1856.928408	1.000892865	0.424619076	2.357154733	0.018415572	0.726053908	ENSG00000105825	TFPI2		
1847.916768	-0.023159662	0.249004076	-0.093009167	0.925896278	0.999527751	ENSG00000100242	SUN2		
1808.039739	0.286366268	0.243977604	1.173739982	0.24049919	0.962465655	ENSG00000162337	LRP5		
1849.417865	-0.017484872	0.194851416	-0.089734384	0.928498291	0.999527751	ENSG00000106052	TAX1BP1		
1875.757787	-0.75280074	0.346863678	-2.170307207	0.029983581	0.773201806	ENSG00000151468	CCDC3		
1840.587387	-0.28961046	0.202365848	-1.431123196	0.152394911	0.938209588	ENSG00000162889	MAPKAPK2		
1849.419379	0.429411805	0.267169411	1.607264108	0.107996444	0.923922314	ENSG00000145495			6-Mar
1818.808357	-0.13250078	0.262400815	-0.504955673	0.61358997	0.99483168	ENSG00000142168	SOD1		
1834.302482	0.056394078	0.220407722	0.255862532	0.798056987	0.999467288	ENSG00000168092	PAFAH1B2		
1815.748453	0.505493542	0.498172471	1.014695856	0.31025083	0.973427483	ENSG00000156466	GDF6		
1823.667894	0.136684957	0.220885525	0.618804501	0.536045157	0.99483168	ENSG00000157916	RER1		
1822.694146	-0.161226288	0.216622357	-0.744273536	0.456711008	0.99135833	ENSG00000115762	PLEKHB2		
1840.429976	0.103657424	0.248104505	0.417797429	0.676095231	0.998869317	ENSG00000146433	TMEM181		
1822.11389	0.077951532	0.18655653	0.417844029	0.676061157	0.998869317	ENSG00000273749	CYFIP1		
1837.092433	-0.407156783	0.30548507	-1.332820564	0.182590696	0.949094286	ENSG00000175061	LRRC75A-AS1		
1822.817669	0.004694114	0.183171285	0.02562691	0.979554922	0.999527751	ENSG00000141905	NFIC		
1820.322029	-0.508578807	0.230038895	-2.210838338	0.027047034	0.765324405	ENSG00000042753	AP2S1		
1819.411028	-0.596239038	0.263551645	-2.262323339	0.023677433	0.744948012	ENSG00000149806	FAU		
1827.431256	0.059632965	0.227491734	0.262132448	0.793219332	0.999467288	ENSG00000162430	SELENON		
1822.019405	0.030523051	0.207089256	0.1473908	0.882823567	0.999467288	ENSG00000075151	EIF4G3		
1797.782119	0.276950589	0.28344769	0.977078308	0.328530386	0.976026345	ENSG00000149485	FADS1		
1819.500066	0.250577527	0.293693836	0.853193007	0.393552281	0.988378925	ENSG00000125912	NCLN		
1830.505585	0.033849371	0.20271983	0.166976123	0.867388836	0.999467288	ENSG00000156599	ZDHHC5		
1845.096565	-0.511852895	0.422751044	-1.210766721	0.225984823	0.958865115	ENSG00000198853	RUSC2		
1816.098305	-0.037052206	0.229891178	-0.161172805	0.8719573	0.999467288	ENSG00000077380	DYNC1I2		
1811.764947	-0.19006229	0.200588704	-0.947522398	0.343372649	0.977691382	ENSG00000165672	PRDX3		
1817.641432	0.119227454	0.230495673	0.517265478	0.604970849	0.99483168	ENSG00000172260	NEGR1		
1812.438037	-0.316329805	0.261438914	-1.209956851	0.226295451	0.958865115	ENSG00000176978	DPP7		
1861.259284	-0.058315381	0.358345124	-0.162735243	0.870726897	0.999467288	ENSG00000166750	SLFN5		

1823.022278	-0.415711113	0.249408522	-1.666787924	0.095556582	0.921147998	ENSG00000169926	KLF13
1816.757813	-0.179153508	0.201024274	-0.891203355	0.372820085	0.982329093	ENSG00000163320	CGGBP1
1806.318679	-0.278420964	0.200106655	-1.391362841	0.164115427	0.943223078	ENSG00000171858	RPS21
1803.797599	0.02519662	0.240063859	0.104957988	0.916409145	0.999527751	ENSG00000173692	PSMD1
1813.581094	-0.431925391	0.224704016	-1.922197028	0.054580971	0.847598083	ENSG00000122042	UBL3
1778.906505	0.472377728	0.361282767	1.307501412	0.191042476	0.949234949	ENSG00000116133	DHCR24
1800.649647	-0.169318656	0.192048775	-0.881644031	0.377969337	0.982609538	ENSG00000132963	POMP
1803.200027	-0.315277746	0.859698629	-0.366730545	0.71382002	0.99900742	ENSG00000101825	MXRA5
1801.294218	-0.195761893	0.201435203	-0.971835556	0.331132364	0.976026345	ENSG00000086061	DNAJA1
1807.915895	-0.176464618	0.189077995	-0.933290086	0.350670212	0.977691382	ENSG00000127946	HIP1
1790.562282	-0.320518082	0.221815348	-1.444977025	0.148464339	0.936396974	ENSG00000110237	ARHGEF17
1793.975529	-0.289239676	0.186659021	-1.54956173	0.121246744	0.923922314	ENSG00000099341	PSMD8
1776.891319	0.149079254	0.298658245	0.499163363	0.617664303	0.99483168	ENSG00000168528	SERINC2
1810.203081	-0.254959231	0.259918599	-0.980919536	0.326632423	0.976026345	ENSG00000198752	CDC42BPB
1774.753832	-0.158303697	0.283711726	-0.557973755	0.576862306	0.99483168	ENSG00000198682	PAPSS2
1777.660929	0.671954272	0.281728413	2.385113611	0.017073848	0.715620174	ENSG00000142227	EMP3
1801.704921	0.208707286	0.228926256	0.911679115	0.361937661	0.979465649	ENSG00000138600	SPPL2A
1787.504171	-0.081384014	0.189972766	-0.428398319	0.668361148	0.997568624	ENSG00000177731	FLII
1756.658243	-0.875709196	0.412119556	-2.124891149	0.033595697	0.775963115	ENSG00000167641	PPP1R14A
1794.053323	0.238940204	0.192460146	1.241504846	0.214419309	0.954310328	ENSG00000084112	SSH1
1797.816846	0.136670861	0.218227433	0.62627718	0.531133151	0.99483168	ENSG00000144028	SNRNP200
1766.60465	-0.695570729	0.319685712	-2.175795487	0.029570556	0.773201806	ENSG00000105698	USF2
1787.047104	-0.015106355	0.202076571	-0.0747556	0.940409169	0.999527751	ENSG00000100353	EIF3D
1788.380946	-0.048368405	0.243004179	-0.199043512	0.842228708	0.999467288	ENSG00000161671	EMC10
1762.102007	-0.131624677	0.310479722	-0.42393969	0.671609797	0.997568624	ENSG00000116171	SCP2
1799.570146	-0.037194975	0.278465505	-0.133571212	0.89374165	0.999467288	ENSG00000116685	KIAA2013
1787.527874	-0.057211249	0.172762448	-0.331155585	0.740526967	0.999467288	ENSG00000149547	EI24
1799.979755	-0.148020271	0.264358459	-0.559922582	0.575532245	0.99483168	ENSG00000100994	PYGB
1803.859214	0.63013771	0.335670282	1.877252004	0.060483563	0.858996918	ENSG00000127481	UBR4
1791.950914	0.18075311	0.293378436	0.616109049	0.53782255	0.99483168	ENSG00000177119	ANO6
1797.02699	0.450584482	0.488962243	0.921511811	0.356783278	0.979427314	ENSG00000173530	TNFRSF10D
1812.550176	0.256158499	0.409665303	0.62528727	0.531782534	0.99483168	ENSG00000155380	SLC16A1
1775.076108	-0.217675151	0.290190318	-0.750111695	0.453187437	0.99135833	ENSG00000130382	MLLT1
1819.399419	-0.58310231	0.321333394	-1.814633401	0.069580266	0.877912272	ENSG00000020181	ADGRA2
1788.002674	0.063124859	0.202684178	0.311444431	0.755462781	0.999467288	ENSG00000121892	PDS5A
1766.303192	-0.108977034	0.237781732	-0.458307008	0.64673189	0.997368416	ENSG00000156052	GNAQ
1773.305084	0.18606779	0.240162661	0.774757365	0.438483047	0.991301167	ENSG00000066322	ELOVL1
1807.242846	-0.178858025	0.269843197	-0.662822066	0.50744452	0.993513219	ENSG00000047932	GOPC
1794.169415	0.24051282	0.292554103	0.822113986	0.411012026	0.990226751	ENSG00000117139	KDM5B
1770.242619	-0.382321046	0.228772242	-1.671186341	0.094684877	0.921147998	ENSG00000154556	SORBS2
1765.672883	0.228186194	0.211290184	1.079965902	0.280157364	0.963267117	ENSG00000010404	IDS
1752.884306	0.184823269	0.251518156	0.734830728	0.462442591	0.99135833	ENSG00000203485	INF2
1799.182614	-0.9103209	0.402149028	-2.263640681	0.023596224	0.744948012	ENSG00000165617	DACT1
1750.895109	0.402382138	0.248516626	1.619135686	0.105418072	0.92169898	ENSG00000124795	DEK
1768.168088	-0.087752584	0.205406625	-0.427213992	0.669223471	0.997568624	ENSG00000114978	MOB1A
1760.362812	-0.096480923	0.236231332	-0.408417134	0.682967457	0.998869317	ENSG00000130741	EIF2S3
1779.368841	0.473488425	0.25428378	1.862047301	0.062596423	0.868202292	ENSG00000198408	OGA
1766.198085	-0.120942689	0.185552826	-0.651796533	0.514532438	0.993513219	ENSG00000177830	CHID1
1751.578863	-0.468342763	0.243341532	-1.92463144	0.054275483	0.847598083	ENSG00000159069	FBXW5
1759.462456	-0.441418491	0.212773373	-2.074594606	0.038024122	0.790190505	ENSG00000107223	EDF1
1762.299623	-0.139239556	0.199018903	-0.699629807	0.484158523	0.991892545	ENSG00000106615	RHEB
1761.718784	0.234123726	0.210284441	1.113366851	0.265550906	0.962465655	ENSG00000177565	TBL1XR1
1771.290107	0.184543354	0.215422194	0.856658968	0.391633386	0.988355932	ENSG00000067704	IARS2
1770.877568	-0.457145261	0.22425425	-2.038513252	0.041498631	0.800562177	ENSG00000186111	PIP5K1C
1759.984973	0.132133341	0.225943909	0.584805945	0.558678207	0.99483168	ENSG00000084073	ZMPSTE24
1738.713227	-0.19579982	0.257645479	-0.759958299	0.447279512	0.99135833	ENSG00000140682	TGFB1I1
1753.469731	0.210857859	0.346569794	0.608413837	0.542913036	0.99483168	ENSG00000196937	FAM3C
1762.160765	0.113212844	0.202974859	0.557767816	0.577002942	0.99483168	ENSG00000119537	KDSR
1783.714053	-0.46619739	0.338935964	-1.375473363	0.168984738	0.943792179	ENSG00000159216	RUNX1
1742.977379	-0.376887603	0.274390952	-1.373542385	0.169583794	0.944422693	ENSG00000167671	UBXN6
1737.797252	0.017839644	0.221094357	0.08068792	0.935690144	0.999527751	ENSG00000103769	RAB11A
1752.287406	-0.473343433	0.200437258	-2.361554121	0.018198515	0.726053908	ENSG00000257923	CUX1
1790.72427	-0.201971429	0.400986265	-0.503686652	0.61448159	0.99483168	ENSG00000103489	XYLT1
1761.09534	0.20224741	0.260654141	0.775922488	0.437794754	0.991301167	ENSG00000104723	TUSC3

1755.395265	-0.316151151	0.214374476	-1.474761157	0.140276767	0.935644597	ENSG00000125534	PPDPF
1758.831172	0.134355909	0.253633568	0.529724478	0.596302974	0.99483168	ENSG00000131873	CHSY1
1733.476247	-0.201828198	0.300738624	-0.671108338	0.502151515	0.993513219	ENSG00000158470	B4GALT5
1729.045279	-0.14332916	0.219400158	-0.653277377	0.513577474	0.993513219	ENSG00000101182	PSMA7
1731.920215	0.208155352	0.274302193	0.758854128	0.447939822	0.99135833	ENSG00000231925	TAPBP
1736.789684	-0.033097743	0.239516762	-0.138185497	0.890093817	0.999467288	ENSG00000171067	C11orf24
1765.307191	-0.601704569	0.287836436	-2.090439201	0.036578365	0.789688071	ENSG00000031081	ARHGAP31
1749.281346	0.311564666	0.238334815	1.307256205	0.191125713	0.949234949	ENSG00000133706	LARS
1738.953246	0.511473913	0.260258267	1.965255202	0.049384708	0.836346249	ENSG00000082898	XPO1
1746.662327	-0.049708266	0.220771734	-0.225156842	0.821857263	0.999467288	ENSG00000169756	LIMS1
1733.065746	0.156784796	0.210080908	0.746306732	0.455482146	0.99135833	ENSG00000180370	PAK2
1742.881311	0.048987327	0.212748894	0.230258903	0.817890593	0.999467288	ENSG00000136813	ECPAS
1732.543464	-0.060812579	0.253477866	-0.23991278	0.810397873	0.999467288	ENSG00000133104	SPART
1738.606374	-0.362118834	0.321853749	-1.125103671	0.260545106	0.962465655	ENSG00000156639	ZFAND3
1738.597806	-0.331337357	0.295863638	-1.119898883	0.262756854	0.962465655	ENSG00000171206	TRIM8
1724.49459	-0.391570949	0.270863824	-1.445637678	0.148278853	0.936396974	ENSG00000175220	ARHGAP1
1721.013061	-0.019192223	0.19442745	-0.098711489	0.921367346	0.999527751	ENSG00000153786	ZDHHC7
1715.27071	-0.315885611	0.208592661	-1.514365888	0.129933066	0.929373153	ENSG00000089693	MLF2
1716.198288	-0.262280773	0.219937751	-1.192522755	0.233056334	0.959575179	ENSG00000165458	INPPL1
1736.307892	-0.013966656	0.324470723	-0.043044426	0.96566612	0.999527751	ENSG00000160007	ARHGAP35
1728.508867	-0.50136215	0.340475872	-1.472533568	0.140876849	0.935644597	ENSG00000157570	TSPAN18
1711.966214	-0.178500566	0.21726816	-0.821567992	0.411322813	0.990226751	ENSG00000112079	STK38
1728.050282	0.360709642	0.333068513	1.082989318	0.278813164	0.963231086	ENSG00000115902	SLC1A4
1747.010586	0.036807974	0.3045739	0.120850718	0.903809278	0.999527751	ENSG00000260916	CCPG1
1707.835251	-0.203741742	0.194622278	-1.046857245	0.295165424	0.970706863	ENSG00000169813	HNRNPF
1685.465559	0.317475509	0.755783181	0.420061622	0.674440438	0.998869317	ENSG00000136542	GALNT5
1715.584386	-0.01960571	0.212211781	-0.09238747	0.926390195	0.999527751	ENSG00000198677	TTC37
1702.879964	-0.14190083	0.250085746	-0.567408705	0.570436537	0.99483168	ENSG00000136193	SCRN1
1677.115457	-0.407726493	0.311373254	-1.309446097	0.190383285	0.949094286	ENSG00000178980	SELENOW
1702.931669	0.663175989	0.295388206	2.245099756	0.024761729	0.749756093	ENSG00000164032	H2AFZ
1697.223322	-0.099836941	0.246064245	-0.405735264	0.684937129	0.998869317	ENSG00000115504	EHBP1
1718.717713	0.143154071	0.253043235	0.565729691	0.571577548	0.99483168	ENSG00000115963	RND3
1734.396026	-0.20165307	0.311679766	-0.646988005	0.517639706	0.993513219	ENSG00000091490	SEL1L3
1700.035273	-0.224214198	0.21660799	-1.035115086	0.300615168	0.972186492	ENSG00000115694	STK25
1688.012391	-0.130063577	0.226864285	-0.57331006	0.566434775	0.99483168	ENSG00000113328	CCNG1
1712.067444	0.162050419	0.210805495	0.768720089	0.442059496	0.99135833	ENSG00000112851	ERBIN
1695.960949	0.042696007	0.303467674	0.140693756	0.888111878	0.999467288	ENSG00000170144	HNRNPA3
1695.616439	-0.035821479	0.194647983	-0.184032111	0.853988265	0.999467288	ENSG00000143569	UBAP2L
1705.54271	0.482184877	0.224477063	2.148036285	0.031710873	0.775963115	ENSG00000008282	SYPL1
1684.943179	-0.149542699	0.296872482	-0.503727046	0.6144532	0.99483168	ENSG00000102317	RBM3
1696.148774	0.370119269	0.222890166	1.660545534	0.096804756	0.921147998	ENSG00000117906	RCN2
1701.891224	0.083314165	0.256860321	0.324355915	0.745668592	0.999467288	ENSG00000088812	ATRN
1690.440906	-0.060468825	0.197431352	-0.306277725	0.759393198	0.999467288	ENSG00000129187	DCTD
1681.368627	-0.397997139	0.245431655	-1.621621052	0.104884512	0.92169898	ENSG00000163041	H3F3A
1704.685105	0.402070304	0.259774761	1.54776508	0.121678867	0.923922314	ENSG00000109180	OCIAD1
1696.715731	0.582115175	0.374483957	1.554446228	0.120078014	0.923922314	ENSG00000185950	IRS2
1684.776339	0.086605879	0.218294212	0.396739234	0.691559765	0.998869317	ENSG00000111843	TMEM14C
1681.254083	-0.307304448	0.210520145	-1.459738913	0.144361843	0.935644597	ENSG00000125817	CENPB
1679.088118	0.277630966	0.208842428	1.329380089	0.183722603	0.949094286	ENSG00000113648	H2AFY
1682.349794	-0.183542487	0.234909393	-0.781333115	0.434606598	0.991301167	ENSG00000140740	UQCRC2
1692.876014	0.102370473	0.234309353	0.43690306	0.662181652	0.997568624	ENSG00000100258	LMF2
1672.930936	-0.057261953	0.256145131	-0.223552767	0.82310532	0.999467288	ENSG00000138029	HADHB
1681.838493	-0.629304855	0.275347003	-2.285497382	0.02228368	0.744905651	ENSG00000186468	RPS23
1697.382553	0.148375736	0.277882091	0.533952134	0.593374651	0.99483168	ENSG00000173905	GOLIM4
1690.335696	0.275446988	0.28809845	0.956086325	0.339028623	0.977691382	ENSG00000116560	SFPQ
1684.585813	-0.595600397	0.222353392	-2.678620688	0.007392609	0.563130099	ENSG00000130255	RPL36
1703.583267	-0.317123988	0.331479431	-0.956692809	0.338722329	0.977691382	ENSG00000054598	FOXC1
1688.798928	-0.324041833	0.239977622	-1.350300208	0.176919706	0.946540546	ENSG00000108100	CCNY
1685.333873	0.21027975	0.214590598	0.97991129	0.327129909	0.976026345	ENSG00000135829	DHX9
1674.229499	0.436655621	0.785993083	0.555546392	0.578520987	0.99483168	ENSG00000017483	SLC38A5
1665.70193	-0.365804396	0.256856832	-1.424156773	0.154401095	0.939432741	ENSG00000137312	FLOT1
1665.877908	-0.232639925	0.221052271	-1.052420423	0.292606702	0.970706863	ENSG00000160075	SSU72
1682.564394	-0.35834989	0.202626566	-1.768523728	0.076973389	0.897052166	ENSG00000164091	WDR82
1724.936311	-0.88278088	1.01962231	-0.86579204	0.386604215	0.986711841	ENSG00000100292	HMOX1

1675.513292	-0.348078102	0.259953701	-1.339000369	0.180570553	0.949094286	ENSG00000213949	ITGA1
1684.668123	0.175935591	0.25985587	0.677050672	0.498373805	0.993513219	ENSG00000101337	TM9SF4
1700.196842	-0.582662889	0.403700282	-1.443305627	0.148934395	0.936396974	ENSG00000151718	WWC2
1660.57172	-0.097297836	0.314596886	-0.309277811	0.757110209	0.999467288	ENSG00000145901	TNIP1
1667.49179	0.12764463	0.206246137	0.618894647	0.535985765	0.99483168	ENSG00000124767	GLO1
1662.501243	-0.049466204	0.326255372	-0.151618053	0.879488194	0.999467288	ENSG00000105953	OGDH
1654.216477	-0.282220348	0.20716265	-1.362312885	0.173099163	0.944422693	ENSG00000136279	DBNL
1627.818063	0.626629801	0.480445423	1.304268435	0.192142074	0.949261383	ENSG00000186480	INSIG1
1664.732164	0.227461463	0.256997934	0.885071171	0.376118247	0.982609538	ENSG00000091317	CMTM6
1660.963665	0.138615353	0.221371805	0.626165348	0.531206493	0.99483168	ENSG00000158290	CUL4B
1652.863333	-0.139014047	0.213146353	-0.652199981	0.514272172	0.993513219	ENSG00000188612	SUMO2
1668.311082	0.319573899	0.204053096	1.566131098	0.117317929	0.923922314	ENSG00000152104	PTPN14
1657.172104	-0.230306368	0.199596401	-1.153860324	0.248557438	0.962465655	ENSG00000175203	DCTN2
1675.589163	-0.268463877	0.234775615	-1.143491317	0.252834658	0.962465655	ENSG00000113712	CSNK1A1
1655.267429	0.852839839	0.487943705	1.747824246	0.080494466	0.897807954	ENSG00000059804	SLC2A3
1682.988852	-0.448727109	0.35889137	-1.250314571	0.211184658	0.954310328	ENSG00000169902	TPST1
1649.838766	0.087386189	0.205132688	0.425998363	0.670109039	0.997568624	ENSG00000166888	STAT6
1672.005396	0.015036341	0.228608855	0.065773222	0.947558376	0.999527751	ENSG00000092148	HECTD1
1653.919502	-0.094955301	0.187075069	-0.507578595	0.611748905	0.99483168	ENSG00000078304	PPP2R5C
1652.286921	-0.493526525	0.228528662	-2.15958261	0.030804996	0.775963115	ENSG00000079950	STX7
1667.908297	-0.01451473	0.280382572	-0.051767588	0.958713882	0.999527751	ENSG00000204256	BRD2
1654.006855	0.157971129	0.245162988	0.64435146	0.519347553	0.993513219	ENSG00000052802	MSMO1
1665.861786	-0.019853581	0.352872447	-0.056262768	0.955132479	0.999527751	ENSG00000120254	MTHFD1L
1647.126993	-0.054479573	0.196537428	-0.277196936	0.781628892	0.999467288	ENSG00000168439	STIP1
1660.019928	0.085654363	0.283587504	0.302038566	0.762622664	0.999467288	ENSG00000111711	GOLT1B
1686.244638	1.027328668	0.357104047	2.876832893	0.004016883	0.45638972	ENSG00000225630	MTND2P28
1658.415809	0.231785172	0.237720982	0.975030352	0.329545204	0.976026345	ENSG00000127483	HP1BP3
1652.937216	0.087510649	0.218165559	0.401120366	0.688331507	0.998869317	ENSG00000186432	KPNA4
1636.163245	-0.114440377	0.191540041	-0.597474952	0.550190326	0.99483168	ENSG00000177885	GRB2
1640.995544	0.064911905	0.242267093	0.267935297	0.788749124	0.999467288	ENSG00000198843	SELENOT
1652.236882	-0.218760573	0.201555622	-1.085360809	0.277761883	0.962465655	ENSG00000110367	DDX6
1661.904091	0.219240058	0.283485599	0.773372821	0.43930177	0.991301167	ENSG00000131016	AKAP12
1653.552363	-0.068519225	0.327140365	-0.209449009	0.834097737	0.999467288	ENSG00000127526	SLC35E1
1630.234109	-0.355182958	0.284151151	-1.249978953	0.211307236	0.954310328	ENSG00000154518	ATP5MC3
1643.724043	0.159416314	0.21047752	0.757403041	0.448808435	0.99135833	ENSG00000150753	CCT5
1633.69824	-0.187742341	0.284762135	-0.659295314	0.509706151	0.993513219	ENSG00000102401	ARMCX3
1638.582733	0.005304354	0.186989064	0.028367188	0.977369294	0.999527751	ENSG00000124570	SERPINB6
1643.593757	-0.142934136	0.185661374	-0.769864691	0.441380161	0.99135833	ENSG00000132128	LRRC41
1666.994556	-0.56942845	0.307931256	-1.8492064	0.064428015	0.868202292	ENSG00000162849	KIF26B
1638.784348	0.476014521	0.209130276	2.27616264	0.022836277	0.744905651	ENSG00000133687	TMTC1
1659.936377	1.780351879	0.329712718	5.39970641	6.68E-08	0.000482035	ENSG00000245532	NEAT1
1620.446397	-0.348343332	0.245046417	-1.421540196	0.155159777	0.939432741	ENSG00000123349	PFDN5
1636.412554	-0.16238498	0.248328895	-0.653910937	0.513169187	0.993513219	ENSG00000101152	DNAJC5
1660.255198	0.392208356	0.322445977	1.216353698	0.223850214	0.956884001	ENSG00000117143	UAP1
1638.903302	-0.120317967	0.21722978	-0.553874182	0.579664955	0.99483168	ENSG00000175115	PACS1
1646.035552	0.11846019	0.309756469	0.382430078	0.702142384	0.998869317	ENSG00000183723	CMTM4
1619.933318	0.719844583	0.284317406	2.531834374	0.011346757	0.645201642	ENSG00000124145	SDC4
1635.673527	-0.306415306	0.195395132	-1.568182908	0.116838452	0.923922314	ENSG00000119487	MAPKAP1
1642.37278	0.324215907	0.205794589	1.575434553	0.115156174	0.923922314	ENSG00000108588	CCDC47
1620.044048	-0.124017876	0.223816903	-0.554104158	0.579507565	0.99483168	ENSG00000008018	PSMB1
1674.547499	-0.043523508	0.450197522	-0.096676471	0.922983325	0.999527751	ENSG00000090020	SLC9A1
1659.409653	-0.069039931	0.358322934	-0.192675166	0.847213374	0.999467288	ENSG00000104368	PLAT
1626.281374	-0.125326089	0.199295997	-0.628843983	0.529451202	0.99483168	ENSG00000116688	MFN2
1649.804445	-1.067780362	0.241808364	-4.415812367	1.01E-05	0.020763122	ENSG00000166482	MFAP4
1629.008225	0.430022688	0.33609391	1.279471823	0.200730956	0.954310328	ENSG00000221968	FADS3
1647.398555	0.285734442	0.279858927	1.020994561	0.307257016	0.973427483	ENSG00000136868	SLC31A1
1617.044128	-0.281259804	0.181516326	-1.549501413	0.121261232	0.923922314	ENSG00000159720	ATP6V0D1
1632.781033	0.046463285	0.215510965	0.21559592	0.829302737	0.999467288	ENSG00000109111	SUPT6H
1616.621314	-0.062098571	0.217761714	-0.285167534	0.775515792	0.999467288	ENSG00000128829	EIF2AK4
1628.70032	-0.183940379	0.248555081	-0.7400387	0.459276512	0.99135833	ENSG00000182095	TNRC18
1581.34021	1.363946008	0.483174482	2.82288502	0.004759364	0.472943531	ENSG00000148926	ADM
1642.707502	-0.393990686	0.245687199	-1.603627242	0.108796242	0.923922314	ENSG00000123066	MED13L
1603.48335	-0.546324147	0.489733207	-1.115554632	0.264612821	0.962465655	ENSG00000161544	CYGB
1636.978735	-0.605780345	0.2702833	-2.241279228	0.025007994	0.754051048	ENSG00000019144	PHLDB1

1618.941441	0.039147278	0.184381876	0.212316299	0.83186028	0.999467288	ENSG00000086232	EIF2AK1
1616.482135	-0.149784345	0.193655414	-0.773458081	0.439251328	0.991301167	ENSG00000111676	ATN1
1607.347591	0.099258213	0.342734018	0.289607124	0.772116816	0.999467288	ENSG00000168938	PPIC
1631.521549	-0.537682081	0.331785886	-1.62056948	0.105110002	0.92169898	ENSG00000132613	MTSS1L
1621.606005	-0.163613119	0.458934458	-0.356506503	0.721461283	0.99900742	ENSG00000116132	PRRX1
1593.020189	0.076295805	0.35990411	0.211989256	0.832115416	0.999467288	ENSG00000116717	GADD45A
1629.4265	-0.156158204	0.515501868	-0.302924614	0.761947313	0.999467288	ENSG00000102359	SRPX2
1603.859023	0.082696131	0.224627184	0.368148368	0.712762607	0.99900742	ENSG00000114416	FXR1
1612.754017	-0.022631894	0.234801886	-0.09638719	0.923213066	0.999527751	ENSG00000144567	RETREG2
1602.420572	0.024487099	0.244583354	0.100117601	0.920250962	0.999527751	ENSG00000196743	GM2A
1606.305595	-0.180783996	0.186436861	-0.96967947	0.332206287	0.976026345	ENSG00000087053	MTMR2
1620.291028	-0.151595277	0.238938433	-0.634453298	0.525785036	0.99483168	ENSG00000015171	ZMYND11
1624.199154	0.407442307	0.913295238	0.446123324	0.655508166	0.997568624	ENSG00000170525	PFKFB3
1611.60527	-0.362422249	0.25240408	-1.435881106	0.151036176	0.936396974	ENSG00000162413	KLHL21
1604.648344	-0.087146811	0.195706603	-0.445293155	0.656107914	0.997568624	ENSG00000121579	NAA50
1599.411622	-0.213448562	0.231160092	-0.923379814	0.355809307	0.978364583	ENSG00000177963	RIC8A
1590.692964	-0.385896639	0.218405282	-1.76688327	0.077247779	0.897052166	ENSG00000174282	ZBTB4
1597.266898	-0.200509879	0.217062125	-0.923744199	0.355619514	0.978364583	ENSG00000130340	SNX9
1592.552718	0.261074382	0.211741149	1.232988403	0.217580091	0.956884001	ENSG00000110108	TMEM109
1588.113076	-0.014703736	0.204248529	-0.071989434	0.942610317	0.999527751	ENSG00000185651	UBE2L3
1614.33199	-0.263913924	0.207221774	-1.273582016	0.202811589	0.954310328	ENSG00000140931	CMTM3
1599.925932	0.409751386	0.22055351	1.857832081	0.063192864	0.868202292	ENSG00000173821	RNF213
1607.183077	0.271461028	0.249448346	1.088245453	0.276486764	0.962465655	ENSG00000115170	ACVR1
1575.072201	0.222592964	0.303791197	0.732716967	0.463731073	0.99135833	ENSG00000198121	LPAR1
1602.651766	-1.253222561	0.486228561	-2.577435104	0.009953657	0.630529247	ENSG00000179403	VWA1
1590.586024	0.382046927	0.20675343	1.847838402	0.064625724	0.868202292	ENSG00000196776	CD47
1599.977767	0.012960528	0.207809214	0.062367435	0.950270228	0.999527751	ENSG00000164880	INTS1
1612.155505	-3.34E-05	0.333526873	-0.000100132	0.999920106	0.999920106	ENSG00000103222	ABCC1
1574.693684	-0.14641412	0.254216444	-0.575942761	0.564653872	0.99483168	ENSG00000105401	CDC37
1592.256626	-0.028962205	0.231567203	-0.125070411	0.900467808	0.999527751	ENSG00000151929	BAG3
1592.118363	0.029438561	0.221803393	0.132723675	0.894411919	0.999467288	ENSG00000100991	TRPC4AP
1578.474847	-0.543054838	0.269385316	-2.015903632	0.043810054	0.807325344	ENSG00000172809	RPL38
1561.515341	-0.650320674	0.3365533	-1.932296237	0.053322953	0.847598083	ENSG00000240342	RPS2P5
1563.972699	0.031588053	0.321833025	0.09815044	0.921812835	0.999527751	ENSG00000042445	RETSAT
1614.073975	-0.285733067	0.354368228	-0.806316832	0.420060188	0.990680811	ENSG00000150764	DIXDC1
1597.193912	-0.481436553	0.233204616	-2.064438351	0.038976173	0.793985738	ENSG00000100129	EIF3L
1585.995292	-0.221747326	0.189224304	-1.171875505	0.241247043	0.962465655	ENSG00000001629	ANKIB1
1578.995932	-0.241257043	0.203137977	-1.187651109	0.234970893	0.961317886	ENSG00000179889	PDXDC1
1591.024395	0.113942143	0.353379611	0.322435532	0.74712277	0.999467288	ENSG00000060982	BCAT1
1572.934683	-0.076412665	0.204746919	-0.373205442	0.708995546	0.99900742	ENSG00000115935	WIPF1
1576.13657	0.470905415	0.282472171	1.667086047	0.095497296	0.921147998	ENSG00000156535	CD109
1568.053803	-0.326602177	0.196627187	-1.661022478	0.096708932	0.921147998	ENSG00000055070	SZRD1
1597.445367	-1.02534042	0.278370012	-3.683372407	0.000230169	0.118725853	ENSG00000130592	LSP1
1579.645976	0.162922759	0.27062026	0.602034596	0.547151109	0.99483168	ENSG00000110619	CARS
1574.156024	0.437030253	0.213975323	2.042432958	0.041108603	0.800562177	ENSG00000131051	RBM39
1566.21275	0.01855289	0.200082642	0.092726135	0.926121134	0.999527751	ENSG00000065882	TBC1D1
1594.307178	0.203679067	0.489678312	0.415944635	0.677450517	0.998869317	ENSG00000181458	TMEM45A
1575.494414	-0.084360926	0.18337269	-0.460051748	0.645479077	0.997368416	ENSG00000174780	SRP72
1550.013767	-0.017385448	1.013955542	-0.017146164	0.986320011	0.999527751	ENSG00000162745	OLFML2B
1526.440207	-0.130079281	0.477842069	-0.27222233	0.785451073	0.999467288	ENSG00000007933	FMO3
1604.193398	-0.850267845	0.514394762	-1.6529481	0.098341427	0.921147998	ENSG00000115468	EFHD1
1586.687166	-0.157247556	0.248824766	-0.631961035	0.527412339	0.99483168	ENSG00000140262	TCF12
1572.920681	0.19745082	0.229449362	0.860542033	0.389490324	0.987216662	ENSG00000032444	PNPLA6
1570.759644	0.160584461	0.221500761	0.724983792	0.468461974	0.99135833	ENSG00000067900	ROCK1
1592.082631	-0.146453055	0.336464197	-0.435270843	0.663365846	0.997568624	ENSG00000108175	ZMIZ1
1568.38043	-0.005075061	0.373291105	-0.01359545	0.989152734	0.999527751	ENSG00000196526	AFAP1
1543.21255	-0.24119855	0.301296817	-0.800534679	0.42340108	0.991301167	ENSG00000123933	MXD4
1566.261636	0.088651719	0.322382894	0.274988907	0.783324761	0.999467288	ENSG00000062485	CS
1556.129362	-0.069169684	0.262938626	-0.26306399	0.792501259	0.999467288	ENSG00000050820	BCAR1
1569.370693	0.038431668	0.221640847	0.173396141	0.862340058	0.999467288	ENSG00000101346	POFUT1
1561.375645	0.267286319	0.221009793	1.209386765	0.226514291	0.958865115	ENSG00000148110	MFSD14B
1566.060551	1.008051805	0.518992792	1.942323324	0.052097971	0.846840376	ENSG00000107984	DKK1
1557.596621	-0.140169484	0.196214728	-0.7143678	0.474999777	0.99135833	ENSG00000108344	PSMD3
1559.826051	0.385731641	0.236554769	1.630622979	0.102969897	0.92169898	ENSG00000118689	FOXO3

1561.741975	0.22414131	0.341665178	0.656026203	0.511807265	0.993513219	ENSG00000198830	HMGN2
1598.129825	0.316700774	0.510701408	0.62012904	0.535172834	0.99483168	ENSG00000104812	GYS1
1600.594387	-0.281239739	0.384293398	-0.73183599	0.464268679	0.99135833	ENSG00000155090	KLF10
1548.52008	-0.137901775	0.219710896	-0.627651051	0.530232558	0.99483168	ENSG00000134779	TPGS2
1565.889064	0.134460978	0.220605587	0.609508489	0.542187447	0.99483168	ENSG00000049239	H6PD
1551.051661	-0.227983086	0.305054316	-0.747352436	0.454850848	0.99135833	ENSG00000172115	CYCS
1552.592411	0.037306158	0.199564998	0.186937379	0.851709732	0.999467288	ENSG00000198176	TFDP1
1553.971264	-0.063303987	0.231046445	-0.273988145	0.784093732	0.999467288	ENSG00000065427	KARS
1549.060527	-0.343849664	0.19476728	-1.765438546	0.07749009	0.897052166	ENSG00000151327	FAM177A1
1559.039011	0.000367701	0.244645302	0.001502997	0.998800782	0.999527751	ENSG00000100811	YY1
1571.435117	0.240114502	0.278781482	0.861300042	0.389072812	0.986937571	ENSG00000115866	DARS
1555.569132	-0.037021169	0.242301063	-0.152789958	0.878563917	0.999467288	ENSG00000079785	DDX1
1551.128828	-0.210455954	0.239748153	-0.877820959	0.380040889	0.983585589	ENSG00000116251	RPL22
1549.018919	-0.237449674	0.2364163	-1.004370995	0.315199825	0.974768742	ENSG00000186318	BACE1
1536.104858	-0.101816916	0.198555567	-0.512788017	0.608099613	0.99483168	ENSG00000119408	NEK6
1555.358839	-0.110293179	0.223066015	-0.494441877	0.620994145	0.99483168	ENSG00000134247	PTGFRN
1534.115185	-0.073926686	0.2929465	-0.252355586	0.800766226	0.999467288	ENSG00000008256	CYTH3
1541.59113	-0.023676874	0.267668396	-0.088455993	0.92951426	0.999527751	ENSG00000169554	ZEB2
1565.028128	-0.677070766	0.274282085	-2.468519828	0.013567314	0.672931175	ENSG00000104687	GSR
1569.308007	0.294911576	0.412010106	0.715787239	0.474122733	0.99135833	ENSG00000138685	FGF2
1540.000642	-0.154509351	0.21335815	-0.724178342	0.468956253	0.99135833	ENSG00000153130	SCOC
1551.155586	-0.124279614	0.224794556	-0.552858647	0.580360206	0.99483168	ENSG00000082701	GSK3B
1544.55591	0.286629138	0.187594182	1.527921254	0.12653209	0.928627271	ENSG00000055211	GINM1
1538.724687	-0.188495903	0.18560314	-1.015585744	0.309826697	0.973427483	ENSG00000115993	TRAK2
1547.043662	-0.163155986	0.206885668	-0.788628752	0.430329021	0.991301167	ENSG00000099331	MYO9B
1526.760836	-0.287758796	0.283537057	-1.014889547	0.310158482	0.973427483	ENSG00000170315	UBB
1546.079397	-0.288605792	0.277223126	-1.041059584	0.297847895	0.972186492	ENSG00000128335	APOL2
1517.217545	-0.041028231	0.360896094	-0.11368433	0.909488035	0.999527751	ENSG00000112096	SOD2
1522.349583	-0.337513656	0.275893187	-1.223349007	0.221197905	0.956884001	ENSG00000127184	COX7C
1543.468819	-0.20607493	0.224234442	-0.919015509	0.35808746	0.979427314	ENSG00000215021	PHB2
1545.467499	-0.208839236	0.208263093	-1.00276642	0.315973575	0.974768742	ENSG00000100504	PYGL
1538.852988	0.09006559	0.331389432	0.27178172	0.785789861	0.999467288	ENSG00000256235	SMIM3
1543.259266	0.234112416	0.258883226	0.904316668	0.365827511	0.981269214	ENSG00000172939	OXSR1
1549.077266	0.534602938	0.268015615	1.99467086	0.04607879	0.815583282	ENSG00000253729	PRKDC
1546.910004	0.100051996	0.219568817	0.455674885	0.648623784	0.997368416	ENSG00000160216	AGPAT3
1562.389436	-0.487681828	0.365147838	-1.335573641	0.181688671	0.949094286	ENSG00000105429	MEGF8
1536.531318	-0.448879166	0.335619297	-1.337465307	0.1810708	0.949094286	ENSG00000019549	SNAI2
1532.931038	-0.017847131	0.233923913	-0.0762946	0.939184722	0.999527751	ENSG00000149577	SIDT2
1515.005522	-0.053353794	0.23809764	-0.224083675	0.822692195	0.999467288	ENSG00000125971	DYNLRB1
1522.109531	-0.260167544	0.285343175	-0.911770693	0.361889441	0.979465649	ENSG00000275832	ARHGAP23
1516.53297	-0.063151158	0.3649873	-0.173022892	0.862633434	0.999467288	ENSG00000118564	FBXL5
1539.100055	-0.577278222	0.251963696	-2.29111666	0.021956667	0.743057397	ENSG00000167693	NXN
1532.370748	-0.097959461	0.183157636	-0.534836895	0.592762648	0.99483168	ENSG00000131467	PSME3
1553.038864	0.155741255	0.256361205	0.607507111	0.543514425	0.99483168	ENSG00000124193	SRSF6
1530.799755	-0.052574167	0.199866609	-0.263046275	0.792514913	0.999467288	ENSG00000197111	PCBP2
1524.910692	0.196212247	0.231246325	0.848498877	0.396160197	0.988378925	ENSG00000129657	SEC14L1
1521.349972	-0.146651835	0.225727534	-0.649685189	0.515895593	0.993513219	ENSG00000197006	METTL9
1513.186215	-0.417280052	0.259300616	-1.609252064	0.107561236	0.923922314	ENSG00000100804	PSMB5
1527.139365	0.475605667	0.309047751	1.538939099	0.123819165	0.927794614	ENSG00000070081	NUCB2
1528.552361	-0.542900379	0.483846584	-1.12205066	0.261840901	0.962465655	ENSG00000136859	ANGPTL2
1522.182079	-0.290949582	0.304069205	-0.956853169	0.338641371	0.977691382	ENSG00000182809	CRIP2
1535.130199	-0.158900913	0.261207492	-0.608332141	0.542967208	0.99483168	ENSG00000164889	SLC4A2
1534.651606	0.289206449	0.248227806	1.165084821	0.24398466	0.962465655	ENSG00000175216	CKAP5
1531.718845	-0.084197687	0.285475751	-0.294938142	0.768041134	0.999467288	ENSG00000126012	KDM5C
1528.227114	0.340226988	0.301616214	1.128012926	0.25931446	0.962465655	ENSG00000099783	HNRNPM
1517.707588	0.16303192	0.261170853	0.62423474	0.532473435	0.99483168	ENSG00000189060	H1F0
1530.111788	-0.128661557	0.287366316	-0.447726648	0.654350488	0.997568624	ENSG00000138413	IDH1
1534.696293	-0.013709338	0.249236345	-0.055005372	0.956134184	0.999527751	ENSG00000080845	DLGAP4
1546.382769	0.316886405	0.315897346	1.003130949	0.315797684	0.974768742	ENSG00000075568	TMEM131
1511.25879	-0.418442752	0.24177008	-1.73074663	0.083496965	0.901307093	ENSG00000173598	NUDT4
1530.575336	0.132292829	0.360176463	0.36730004	0.713395224	0.99900742	ENSG00000173546	CSPG4
1513.362659	0.122218047	0.198832911	0.614677147	0.538767955	0.99483168	ENSG00000148672	GLUD1
1496.653523	0.699199832	0.950727923	0.735436307	0.462073817	0.99135833	ENSG00000173432	SAA1
1528.464205	-0.23077806	0.277507576	-0.831609945	0.405629147	0.990045328	ENSG00000211455	STK38L

1520.034443	0.170464379	0.223101041	0.764068056	0.444826687	0.99135833	ENSG00000101557	USP14
1518.108807	-0.068746161	0.188591981	-0.364523245	0.715467319	0.99900742	ENSG00000143621	ILF2
1511.861342	-0.54267493	0.283405492	-1.914835613	0.055513471	0.847598083	ENSG00000178996	SNX18
1515.388183	-0.242960605	0.326921628	-0.743176907	0.457374583	0.99135833	ENSG00000135631	RAB11FIP5
1510.095008	0.283402768	0.216519038	1.308904615	0.190566663	0.949094286	ENSG00000116962	NID1
1524.472362	-0.324127494	0.221633471	-1.462448307	0.1436184	0.935644597	ENSG00000100266	PACSIN2
1508.199814	-0.109515521	0.20183517	-0.5425988	0.587406065	0.99483168	ENSG00000114573	ATP6V1A
1514.711652	-0.646621406	0.255197597	-2.5338068	0.011283092	0.644117389	ENSG00000128487	SPECC1
1507.689309	-0.043666586	0.225667634	-0.193499549	0.846567759	0.999467288	ENSG00000138279	ANXA7
1518.570667	0.357169998	0.232577681	1.535701944	0.124611498	0.927794614	ENSG00000134954	ETS1
1512.285304	0.295177429	0.197188791	1.496928031	0.134411985	0.933741583	ENSG00000089154	GCN1
1506.344439	-0.383941551	0.231493541	-1.658541095	0.0972083	0.921147998	ENSG00000197451	HNRNPAB
1498.532636	-0.06740454	0.279298591	-0.24133505	0.809295447	0.999467288	ENSG00000141736	ERBB2
1504.956711	-0.599848729	0.261140276	-2.297036439	0.021616691	0.73997555	ENSG00000156482	RPL30
1524.158639	0.031262726	0.2969697	0.105272443	0.916159628	0.999527751	ENSG00000147010	SH3KBP1
1507.30951	0.413277028	0.261268402	1.581810216	0.113692902	0.923922314	ENSG00000115307	AUP1
1499.934953	0.06550701	0.190691761	0.343523023	0.731205024	0.999467288	ENSG00000178913	TAF7
1509.281868	0.351866276	0.23552229	1.493982913	0.135180085	0.933741583	ENSG00000152795	HNRNPDL
1496.379314	0.005415872	0.19446825	0.027849646	0.97778207	0.999527751	ENSG00000126067	PSMB2
1503.652277	-0.003933075	0.176652215	-0.022264509	0.982236959	0.999527751	ENSG00000146223	RPL7L1
1514.964336	0.016842501	0.275254404	0.061188853	0.951208807	0.999527751	ENSG00000136731	UGGT1
1485.889589	0.206020741	0.293492753	0.701961937	0.482702901	0.991759385	ENSG00000183722	LHFPL6
1501.934554	-0.006694065	0.18652588	-0.035888128	0.971371563	0.999527751	ENSG00000134882	UBAC2
1489.78142	-0.050018812	0.240671237	-0.207830453	0.835361354	0.999467288	ENSG00000165629	ATP5F1C
1489.455751	0.291893234	0.247511172	1.179313372	0.238273416	0.962465655	ENSG00000142166	IFNAR1
1464.714119	-0.106698768	0.236073422	-0.451972809	0.651288571	0.997449432	ENSG00000167815	PRDX2
1500.076909	-0.0321965	0.235674192	-0.136614449	0.891335555	0.999467288	ENSG00000143776	CDC42BPA
1471.607393	-0.048285411	0.278744744	-0.173224474	0.862474987	0.999467288	ENSG00000112514	CUTA
1486.741595	0.492419274	0.31413384	1.567546095	0.116987101	0.923922314	ENSG00000166130	IKBIP
1481.562671	-0.262302426	0.19366904	-1.354384912	0.175613609	0.946278819	ENSG00000160310	PRMT2
1516.644477	-0.340071115	0.376490437	-0.903266276	0.366384591	0.981269214	ENSG00000182704	TSKU
1461.089718	-0.116032498	0.331517309	-0.350004344	0.726335438	0.999467288	ENSG00000160213	CSTB
1490.680999	0.155785873	0.20381955	0.76433234	0.444669218	0.99135833	ENSG00000106771	TMEM245
1481.657303	0.11361582	0.199844022	0.568522483	0.569680244	0.99483168	ENSG00000122565	CBX3
1480.890174	-0.121649328	0.180905189	-0.672447975	0.50129855	0.993513219	ENSG00000171055	FEZ2
1479.443755	-0.069134039	0.215402834	-0.320952317	0.748246526	0.999467288	ENSG00000139684	ESD
1487.515949	0.089845592	0.202363187	0.443981896	0.657055673	0.997568624	ENSG00000085719	CPNE3
1494.128803	0.716789233	0.379641831	1.888067053	0.059016949	0.853473128	ENSG00000154734	ADAMTS1
1464.101084	0.020615082	0.26774432	0.076995403	0.938627202	0.999527751	ENSG00000160932	LY6E
1483.535421	0.032011721	0.203520298	0.157290067	0.875016248	0.999467288	ENSG00000121774	KHDRBS1
1456.661679	-0.527296101	0.303716736	-1.736144367	0.082538311	0.899922174	ENSG00000126458	RRAS
1461.891753	-0.239998597	0.279506988	-0.858649719	0.390533799	0.98765184	ENSG00000105355	PLIN3
1474.712365	0.480547038	0.239190195	2.009058266	0.044530955	0.811047391	ENSG00000115084	SLC35F5
1504.551246	0.041676816	0.353978412	0.117738298	0.906275021	0.999527751	ENSG00000134109	EDEM1
1459.505769	-0.532583885	0.278969595	-1.909110865	0.056247792	0.847598083	ENSG00000126432	PRDX5
1487.743418	-0.723881101	0.398144376	-1.818137197	0.069043166	0.87549644	ENSG00000198848	CES1
1496.714266	0.450681558	0.255193261	1.766040199	0.077389105	0.897052166	ENSG00000181222	POLR2A
1477.007375	-0.285008552	0.237573024	-1.199667149	0.230268636	0.959575179	ENSG00000213741	RPS29
1468.520366	-0.2507355	0.253775962	-0.988019111	0.323143289	0.975337095	ENSG00000124172	ATP5F1E
1468.850869	-0.614177175	0.502726601	-1.221692215	0.221824041	0.956884001	ENSG00000221869	CEBPD
1473.350137	-0.050776125	0.204794881	-0.247936496	0.804183542	0.999467288	ENSG00000089248	ERP29
1473.571782	0.028492132	0.190130399	0.149855744	0.880878429	0.999467288	ENSG00000182149	IST1
1469.863319	-0.038102003	0.192931528	-0.197489772	0.843444285	0.999467288	ENSG00000100554	ATP6V1D
1466.665681	-0.102929474	0.235618082	-0.436848791	0.662221012	0.997568624	ENSG00000110756	HPS5
1337.049567	-0.41298539	1.475588435	-0.279878441	0.779570768	0.999467288	ENSG00000174697	LEP
1448.768445	0.003694166	0.293105819	0.012603523	0.98994411	0.999527751	ENSG00000143198	MGST3
1467.24706	-0.132860356	0.25055708	-0.530259837	0.595931789	0.99483168	ENSG00000089053	ANAPC5
1471.60093	0.232639581	0.263581416	0.882609953	0.377447049	0.982609538	ENSG00000160746	ANO10
1470.613224	-0.048957272	0.29500726	-0.165952772	0.868194117	0.999467288	ENSG00000107438	PDLIM1
1474.995408	0.113455157	0.174643056	0.649640237	0.515924636	0.993513219	ENSG00000108256	NUFIP2
1474.911222	-0.3276903	0.225044326	-1.456114469	0.145360978	0.935644597	ENSG00000143515	ATP8B2
1483.378152	0.183834555	0.23255681	0.790493104	0.429239848	0.991301167	ENSG00000148396	SEC16A
1464.326607	-0.262428453	0.217523283	-1.206438452	0.227648477	0.958865115	ENSG00000101421	CHMP4B
1469.735509	-0.172596823	0.188204195	-0.917072135	0.359104842	0.979465649	ENSG00000269378	AC022149.1

1483.266369	0.149776302	0.370281096	0.404493514	0.685849846	0.998869317	ENSG00000125629	INSIG2
1451.042299	-0.324811454	0.245740275	-1.321767275	0.186245659	0.949094286	ENSG00000125970	RALY
1464.285511	-0.412099729	0.189778132	-2.171481635	0.029894784	0.773201806	ENSG00000119242	CCDC92
1477.339263	-0.237823552	0.209286397	-1.136354562	0.255808204	0.962465655	ENSG00000012983	MAP4K5
1445.309232	-0.262389259	0.296290546	-0.885580936	0.375843388	0.982609538	ENSG00000168653	NDUFS5
1487.601821	0.94254977	0.315764659	2.984975495	0.00283601	0.384679448	ENSG00000169045	HNRNPH1
1462.082749	0.042521608	0.184189989	0.230857326	0.817425645	0.999467288	ENSG00000143337	TOR1AIP1
1459.880538	-0.093772542	0.194355779	-0.482478795	0.629465858	0.99483168	ENSG00000196715	VKORC1L1
1454.632193	-0.100384924	0.271680834	-0.36949579	0.71175821	0.99900742	ENSG00000117410	ATP6V0B
1456.31053	0.247476707	0.204482861	1.210256478	0.226180492	0.958865115	ENSG00000102158	MAGT1
1478.920931	-0.047210397	0.328985855	-0.143502816	0.885893088	0.999467288	ENSG00000106070	GRB10
1479.696768	0.151968005	0.288426977	0.526885545	0.598273073	0.99483168	ENSG00000101782	RIOK3
1453.4488	-0.163150061	0.312607472	-0.521900707	0.601739466	0.99483168	ENSG00000105137	SYDE1
1440.386388	0.082096191	0.265006297	0.309789586	0.756720971	0.999467288	ENSG00000186298	PPP1CC
1465.487324	0.424442026	0.247715471	1.713425586	0.086634273	0.901483284	ENSG00000064393	HIPK2
1460.181329	-0.108531926	0.318502673	-0.34075672	0.733286735	0.999467288	ENSG00000141580	WDR45B
1471.40046	0.02065996	0.308632242	0.066940382	0.946629165	0.999527751	ENSG00000136986	DERL1
1447.942325	-0.256773973	0.209837815	-1.223678264	0.221073622	0.956884001	ENSG00000116786	PLEKHM2
1444.410564	-0.129115542	0.211163135	-0.611449258	0.540902201	0.99483168	ENSG00000159842	ABR
1455.117872	0.122727436	0.200471432	0.612194137	0.54040932	0.99483168	ENSG00000029364	SLC39A9
1455.600797	-0.124920366	0.204973953	-0.609445071	0.542229471	0.99483168	ENSG00000257103	LSM14A
1449.273786	0.156903932	0.245522232	0.639062013	0.52278259	0.994616524	ENSG00000068366	ACSL4
1447.292878	-0.157306163	0.190725323	-0.824778591	0.409497304	0.990226751	ENSG00000087302	RTRAF
1466.913201	0.144096629	0.317193257	0.454286547	0.649622598	0.997381078	ENSG00000069869	NEDD4
1439.726433	-0.272314967	0.22382737	-1.216629435	0.223745238	0.956884001	ENSG00000109113	RAB34
1441.156823	-0.001751828	0.205087819	-0.008541844	0.993184678	0.999527751	ENSG00000177879	AP3S1
1444.491993	-0.452762712	0.266952123	-1.696044621	0.08987743	0.910413547	ENSG00000005243	COPZ2
1431.87471	-0.240485301	0.264437245	-0.909422959	0.363126907	0.98048242	ENSG00000185222	TCEAL9
1438.986833	-0.501225573	0.277962743	-1.803211348	0.071355029	0.883866242	ENSG00000100439	ABHD4
1432.685878	-0.415398369	0.255155886	-1.628017977	0.103521074	0.92169898	ENSG00000141985	SH3GL1
1446.858739	-0.177938866	0.197344345	-0.901666907	0.367233837	0.981269214	ENSG00000198833	UBE2J1
1442.487363	0.121360862	0.192634807	0.630004847	0.528691413	0.99483168	ENSG00000144043	TEX261
1438.613388	0.423649019	0.306199574	1.383571551	0.166489685	0.943223078	ENSG00000099139	PCSK5
1446.662719	0.152636569	0.21677436	0.704126487	0.481354006	0.991525194	ENSG00000187555	USP7
1454.508089	-0.026652311	0.318186046	-0.083763292	0.933244634	0.999527751	ENSG00000167522	ANKRD11
1447.608223	-1.310209125	0.460830293	-2.843148864	0.00446702	0.469826318	ENSG00000170345	FOS
1430.628372	-0.080187561	0.205833741	-0.389574425	0.696851267	0.998869317	ENSG00000120805	ARL1
1413.383347	-1.253841972	0.399195843	-3.140919411	0.001684184	0.296642256	ENSG00000130176	CNN1
1428.644113	0.223128871	0.205611558	1.085196153	0.277834788	0.962465655	ENSG00000135679	MDM2
1427.696746	-0.376463184	0.299851213	-1.255499953	0.209297325	0.954310328	ENSG00000164543	STK17A
1434.844329	-0.401677382	0.289656641	-1.386736312	0.165522184	0.943223078	ENSG00000063245	EPN1
1430.372465	-0.159288537	0.224766582	-0.708684252	0.478520439	0.99135833	ENSG00000111142	METAP2
1536.035815	-0.213789607	1.350853262	-0.158262642	0.874249844	0.999467288	ENSG00000185633	NDUFA4L2
1422.439756	0.056089812	0.240214137	0.233499213	0.81537378	0.999467288	ENSG00000092820	EZR
1433.041145	-0.22557749	0.208661721	-1.081067906	0.279666909	0.963267117	ENSG00000122068	FYTTD1
1407.065909	-0.529973323	0.278828745	-1.900712656	0.05733966	0.852306368	ENSG00000228474	OST4
1449.152424	-0.032222437	0.417550301	-0.077170191	0.938488155	0.999527751	ENSG00000159200	RCAN1
1422.971329	-0.042724003	0.180379523	-0.236856172	0.812768372	0.999467288	ENSG00000078140	UBE2K
1426.423209	0.331187631	0.227996923	1.452596935	0.146335697	0.936017041	ENSG00000139921	TMX1
1439.960624	0.18251617	0.241127894	0.756926821	0.449093706	0.99135833	ENSG00000134313	KIDINS220
1417.268038	-0.23641091	0.241047549	-0.980764627	0.326708826	0.976026345	ENSG00000174851	YIF1A
1438.578506	-0.169074959	0.272535813	-0.620377033	0.53500959	0.99483168	ENSG00000179051	RCC2
1414.76151	-1.196923236	0.864237186	-1.384947623	0.166068484	0.943223078	ENSG00000157168	NRG1
1416.942797	0.034723123	0.241344036	0.143873963	0.885599997	0.999467288	ENSG00000161057	PSMC2
1415.991595	-0.035822114	0.19584194	-0.182913395	0.854865972	0.999467288	ENSG00000136448	NMT1
1414.564566	-1.916290261	0.887847618	-2.158354905	0.030900249	0.775963115	ENSG00000115738	ID2
1400.692493	0.042861903	0.402983898	0.106361328	0.915295675	0.999527751	ENSG00000177606	JUN
1423.58197	0.101298173	0.199488049	0.507790685	0.611600143	0.99483168	ENSG00000101266	CSNK2A1
1422.738889	-0.20517637	0.339966885	-0.603518694	0.546163684	0.99483168	ENSG00000174080	CTSF
1423.196989	0.082795688	0.193364155	0.428185296	0.668516221	0.997568624	ENSG00000138085	ATRAID
1412.28973	-0.357182503	0.206048855	-1.733484532	0.083009584	0.901307093	ENSG00000107745	MICU1
1429.109167	0.10300639	0.233217617	0.441674995	0.658724407	0.997568624	ENSG00000160551	TAOK1
1413.105628	-0.046679256	0.302528658	-0.154296971	0.877375586	0.999467288	ENSG00000184867	ARMCX2
1410.103512	0.149169983	0.208574949	0.715186477	0.474493824	0.99135833	ENSG00000138434	ITPRID2

1418.985527	0.091209695	0.258944084	0.352237031	0.724660507	0.999456245	ENSG00000029363	BCLAF1
1402.145959	-0.470830251	0.296322654	-1.588910752	0.112080543	0.923922314	ENSG00000139112	GABARAPL1
1410.581609	0.074347752	0.186406709	0.398846974	0.690005963	0.998869317	ENSG00000064726	BTBD1
1410.848722	0.043028848	0.21514324	0.200000928	0.841479856	0.999467288	ENSG00000167930	FAM234A
1386.049823	-0.163183982	0.305355462	-0.534406625	0.593060236	0.99483168	ENSG00000131981	LGALS3
1413.055799	-0.337649793	0.190918598	-1.768553702	0.076968383	0.897052166	ENSG00000072062	PRKACA
1402.869588	-0.069243142	0.232574872	-0.297724088	0.765913757	0.999467288	ENSG00000135272	MDFIC
1401.633034	0.422793516	0.275584604	1.534169579	0.12498794	0.927794614	ENSG00000113621	TXNDC15
1412.805643	0.184559561	0.180680218	1.021470765	0.307031453	0.973427483	ENSG00000189091	SF3B3
1447.146091	-0.612914699	0.448973969	-1.365145289	0.17220739	0.944422693	ENSG00000169231	THBS3
1411.417779	-0.24260777	0.224807421	-1.079180433	0.2805073	0.963267117	ENSG00000102225	CDK16
1396.018897	-0.095933105	0.309614441	-0.309846997	0.756677311	0.999467288	ENSG00000204386	NEU1
1410.412219	-0.234106925	0.232651043	-1.006257791	0.314291579	0.974768742	ENSG00000167110	GOLGA2
1407.301353	-0.032755316	0.203768506	-0.160747685	0.872292131	0.999467288	ENSG00000084652	TXLNA
1408.548079	-0.738669357	0.2161714	-3.417054047	0.000633027	0.195790358	ENSG00000214717	ZBED1
1392.483345	-0.070200415	0.220368915	-0.318558608	0.750061245	0.999467288	ENSG00000085998	POMGNT1
1398.075546	-0.960559974	0.375227001	-2.559943641	0.010468914	0.635400047	ENSG00000105373	NOP53
1399.30148	0.367160118	0.299684305	1.225156311	0.220516332	0.956884001	ENSG00000105810	CDK6
1405.647762	0.650819053	0.852247597	0.763650207	0.445075721	0.99135833	ENSG00000175040	CHST2
1421.556008	0.243392734	0.335264481	0.725972322	0.467855738	0.99135833	ENSG00000131018	SYNE1
1383.093708	0.194659194	0.301619625	0.645379735	0.518681132	0.993513219	ENSG00000093167	LRRFIP2
1412.789031	-0.174504816	0.211059938	-0.826802175	0.408349197	0.990226751	ENSG00000182831	C16orf72
1394.930168	-0.17397627	0.240806235	-0.722474106	0.47000304	0.99135833	ENSG00000132002	DNAJB1
1393.022414	-0.04868113	0.222115872	-0.219169976	0.826517643	0.999467288	ENSG00000146112	PPP1R18
1394.843094	-0.052256635	0.18118783	-0.288411397	0.773031844	0.999467288	ENSG00000077721	UBE2A
1398.293242	0.314222527	0.237250656	1.324432702	0.18535938	0.949094286	ENSG00000016864	GLT8D1
1394.17727	0.317823435	0.224009445	1.418794794	0.155958851	0.939464191	ENSG00000089280	FUS
1403.989918	0.139125747	0.233331807	0.596257103	0.551003483	0.99483168	ENSG00000125505	MBOAT7
1385.267195	-0.172431784	0.392302237	-0.439538111	0.660271673	0.997568624	ENSG00000068878	PSME4
1400.437127	-0.220535107	0.214155418	-1.029789997	0.303108597	0.972793733	ENSG00000153989	NUS1
1402.468432	0.04100356	0.212621218	0.192847921	0.847078073	0.999467288	ENSG00000196591	HDAC2
1392.616245	0.390137949	0.438065985	0.890591742	0.373148231	0.982329093	ENSG00000099250	NRP1
1392.972991	0.023946634	0.275643645	0.086875334	0.930770606	0.999527751	ENSG00000150457	LATS2
1383.80291	-0.373132188	0.326433629	-1.143056824	0.253014999	0.962465655	ENSG00000163346	PBXIP1
1401.501629	-0.428822984	0.29304359	-1.463341971	0.143373829	0.935644597	ENSG00000179195	ZNF664
1386.899887	0.011351776	0.185997917	0.061031737	0.951333934	0.999527751	ENSG00000168288	MMADHC
1420.375993	-0.13917825	0.33632016	-0.413826665	0.679001054	0.998869317	ENSG00000101384	JAG1
1383.987258	0.020022125	0.229326585	0.087308345	0.930426421	0.999527751	ENSG00000073578	SDHA
1383.592433	0.182814368	0.260854876	0.700827875	0.48341044	0.991892545	ENSG00000064115	TM7SF3
1383.196218	0.014483871	0.218511017	0.066284399	0.947151404	0.999527751	ENSG00000164506	STXBP5
1376.174992	-0.134328309	0.212023753	-0.633553112	0.526372509	0.99483168	ENSG00000079805	DNM2
1386.657242	-0.267022476	0.259130951	-1.030453812	0.302797023	0.972793733	ENSG00000104164	BLOC1S6
1385.66347	-0.130941306	0.197248487	-0.663839345	0.506793141	0.993513219	ENSG00000119689	DLST
1388.077	-0.277795927	0.215351922	-1.28996261	0.19706364	0.954310328	ENSG00000117859	OSBPL9
1385.291769	0.17148608	0.211776578	0.809749981	0.418083886	0.990226751	ENSG00000147475	ERLIN2
1377.042254	-0.263435708	0.24101539	-1.093024427	0.274383075	0.962465655	ENSG00000136888	ATP6V1G1
1360.331497	-0.295915587	0.284503802	-1.040111186	0.298288247	0.972186492	ENSG00000103202	NME4
1371.046046	-0.47890333	0.266352025	-1.798008968	0.072175587	0.885710197	ENSG00000173786	CNP
1364.777941	-0.439177595	0.28216284	-1.556468582	0.119596708	0.923922314	ENSG00000182054	IDH2
1385.839845	0.097403779	0.185512175	0.525053297	0.599546141	0.99483168	ENSG00000109814	UGDH
1378.512412	-0.243424876	0.224754331	-1.083070906	0.278776952	0.963231086	ENSG00000107341	UBE2R2
1385.433302	0.193774321	0.202280806	0.957947147	0.338089411	0.977691382	ENSG00000136827	TOR1A
1387.06589	-0.301100615	0.282068423	-1.06747367	0.285757997	0.964826902	ENSG00000142627	EPHA2
1356.901523	-0.441953683	0.320419563	-1.379296816	0.167803258	0.943223078	ENSG00000166681	BEX3
1387.988804	0.213872548	0.315727165	0.677396727	0.498154275	0.993513219	ENSG00000130827	PLXNA3
1372.241096	0.000679897	0.201651752	0.003371638	0.997309827	0.999527751	ENSG00000150459	SAP18
1365.46368	0.039157376	0.257222725	0.152231402	0.879004427	0.999467288	ENSG00000168575	SLC20A2
1361.924737	-0.127900899	0.24572075	-0.520513219	0.602705917	0.99483168	ENSG00000118515	SGK1
1390.986299	0.183485619	0.276985602	0.662437389	0.507690948	0.993513219	ENSG00000116641	DOCK7
1383.905211	0.193462495	0.283011982	0.683584116	0.494237818	0.993513219	ENSG00000144674	GOLGA4
1385.387238	0.181417649	0.201553138	0.900098361	0.368067908	0.981269214	ENSG00000138138	ATAD1
1382.11258	-0.010945866	0.207945224	-0.05263822	0.958020164	0.999527751	ENSG00000074657	ZNF532
1388.415656	-0.088592938	0.287682929	-0.307953405	0.758117788	0.999467288	ENSG00000185164	NOMO2
1384.171818	0.038810727	0.210380233	0.184478964	0.853637728	0.999467288	ENSG00000180776	ZDHHC20

1354.970683	0.038993933	0.370052107	0.105374168	0.916078913	0.999527751	ENSG00000068305	MEF2A
1352.003237	0.069357283	0.481197625	0.144134716	0.885394091	0.999467288	ENSG00000130164	LDLR
1364.669141	-0.307850017	0.258148596	-1.19253028	0.233053385	0.959575179	ENSG00000123144	TRIR
1387.732986	-0.086991735	0.332971647	-0.261258686	0.793893024	0.999467288	ENSG00000164465	DCBLD1
1369.908914	0.026337705	0.212451638	0.123970353	0.901338748	0.999527751	ENSG00000090615	GOLGA3
1366.45813	-0.030345363	0.213526932	-0.142114921	0.886989233	0.999467288	ENSG00000006007	GDE1
1374.026439	-0.018167396	0.193967488	-0.09366207	0.925377602	0.999527751	ENSG00000185728	YTHDF3
1354.96389	-0.012145153	0.225912258	-0.053760487	0.957125991	0.999527751	ENSG00000127884	ECHS1
1368.808316	-0.02115563	0.210185748	-0.100652067	0.919826663	0.999527751	ENSG00000164244	PRRC1
1369.667549	0.020523822	0.202045852	0.10158002	0.919090039	0.999527751	ENSG00000168175	MAPK1IP1L
1372.048843	0.336125822	0.18242543	1.842538194	0.065396465	0.868202292	ENSG00000080815	PSEN1
1366.922447	0.091991353	0.232203747	0.396166531	0.69198218	0.998869317	ENSG00000112249	ASCC3
1364.213182	-0.29876272	0.257421681	-1.160596572	0.245806	0.962465655	ENSG00000100284	TOM1
1358.256658	-0.042252886	0.227820896	-0.185465367	0.852864042	0.999467288	ENSG00000111481	COPZ1
1365.092099	-0.172273701	0.255510549	-0.674233224	0.50016305	0.993513219	ENSG00000139793	MBNL2
1374.092814	-0.08407268	0.278883357	-0.301461805	0.763062372	0.999467288	ENSG00000214517	PPME1
1361.006921	-0.072920016	0.186454324	-0.391087825	0.69573232	0.998869317	ENSG00000196235	SUPT5H
1368.888345	-0.138755855	0.230907884	-0.600914325	0.547897052	0.99483168	ENSG00000108061	SHOC2
1385.552703	-0.141856541	0.364757221	-0.388906738	0.697345138	0.998869317	ENSG00000155850	SLC26A2
1359.876165	-0.275006398	0.229756556	-1.196946905	0.23132725	0.959575179	ENSG00000100403	ZC3H7B
1356.939455	0.007839647	0.211154761	0.037127491	0.970383352	0.999527751	ENSG00000152492	CCDC50
1362.735421	0.108702084	0.238965554	0.454885993	0.64919126	0.997381078	ENSG00000122591	FAM126A
1360.590498	0.616672114	0.261197215	2.360944443	0.018228461	0.726053908	ENSG00000106392	C1GALT1
1359.048649	-0.364140549	0.231830774	-1.570717047	0.116248388	0.923922314	ENSG00000108861	DUSP3
1369.500234	0.149454187	0.277160692	0.539232982	0.589726105	0.99483168	ENSG00000173120	KDM2A
1347.045282	-0.202108671	0.232869117	-0.867906718	0.385445401	0.986047049	ENSG00000158109	TPRG1L
1370.462153	-0.123959031	0.26818906	-0.462207633	0.64393243	0.997332006	ENSG00000010017	RANBP9
1355.541107	-0.138017823	0.219381517	-0.629122384	0.529268937	0.99483168	ENSG00000174306	ZHX3
1351.435876	-0.114124173	0.205140892	-0.556320937	0.577991477	0.99483168	ENSG00000122034	GTF3A
1346.62666	-0.004563486	0.277984198	-0.016416352	0.986902235	0.999527751	ENSG00000106348	IMPDH1
1364.474227	-0.287307722	0.237467116	-1.209884242	0.226323315	0.958865115	ENSG00000130723	PRRC2B
1346.046503	-0.035015495	0.210441674	-0.1663905	0.86784965	0.999467288	ENSG00000175387	SMAD2
1350.320405	0.165137014	0.25743132	0.641479888	0.521210944	0.993513219	ENSG00000152127	MGAT5
1357.584011	0.179005285	0.210454439	0.850565499	0.395010762	0.988378925	ENSG00000106460	TMEM106B
1352.416078	-0.306166267	0.217628254	-1.40683143	0.15947738	0.939986013	ENSG00000166887	VPS39
1345.746452	-0.235801807	0.26472749	-0.890734119	0.373071826	0.982329093	ENSG00000100823	APEX1
1356.223493	0.06126151	0.23610889	0.259462955	0.795278061	0.999467288	ENSG00000165732	DDX21
1349.811068	-0.160082375	0.199690407	-0.801652804	0.422753824	0.991301167	ENSG00000117505	DR1
1346.051507	-0.355576346	0.250867297	-1.417388199	0.156369461	0.939464191	ENSG00000147853	AK3
1363.940211	0.102098418	0.244937437	0.41683468	0.676799334	0.998869317	ENSG00000074054	CLASP1
1349.897952	0.172745866	0.204178651	0.846052539	0.397523436	0.988378925	ENSG00000099917	MED15
1342.060182	0.200892084	0.211950556	0.947825229	0.343218435	0.977691382	ENSG00000010244	ZNF207
1362.307327	-0.236331345	0.264448302	-0.893676925	0.371494775	0.981955904	ENSG00000117020	AKT3
1341.868304	-0.084327044	0.224682219	-0.375316944	0.707424766	0.99900742	ENSG00000090487	SPG21
1358.537815	0.306827934	0.229691661	1.335825309	0.181606379	0.949094286	ENSG00000117523	PRRC2C
1351.212512	0.005328912	0.190002072	0.028046602	0.977624983	0.999527751	ENSG00000153147	SMARCA5
1340.859652	-0.155497106	0.189481237	-0.820646459	0.411847676	0.990226751	ENSG00000136930	PSMB7
1332.777819	-0.408528183	0.234143657	-1.744775787	0.081023905	0.897807954	ENSG00000099817	POLR2E
1343.923566	-0.234048837	0.213252701	-1.097518749	0.272414689	0.962465655	ENSG00000088247	KHSRP
1368.220077	-0.19931315	0.414977593	-0.480298584	0.631015096	0.99483168	ENSG00000198768	APCDD1L
1350.80099	0.690179316	0.456581366	1.511623926	0.130629561	0.929373153	ENSG00000182481	KPNA2
1345.002634	-0.179658808	0.192579805	-0.932905752	0.350868632	0.977691382	ENSG00000141551	CSNK1D
1336.18742	-0.279982068	0.208433565	-1.343267662	0.179185319	0.9469453	ENSG00000088256	GNA11
1345.093857	0.296067774	0.236221799	1.253346536	0.210079619	0.954310328	ENSG00000173230	GOLGB1
1331.840986	0.179730122	0.237432592	0.756973255	0.449065886	0.99135833	ENSG00000006042	TMEM98
1331.456401	-0.313079197	0.230683471	-1.357180885	0.174723743	0.946176003	ENSG00000106244	PDAP1
1329.959752	-0.113598326	0.215132957	-0.528037767	0.597473123	0.99483168	ENSG00000125743	SNRPD2
1350.737558	0.0175092	0.220074427	0.079560359	0.936586924	0.999527751	ENSG00000005007	UPF1
1327.905656	-0.163306773	0.267913111	-0.609551255	0.54215911	0.99483168	ENSG00000124098	FAM210B
1337.036352	-0.010639144	0.276121338	-0.038530683	0.969264568	0.999527751	ENSG00000125944	HNRNPR
1344.02651	-0.346878752	0.271331245	-1.278432756	0.201096881	0.954310328	ENSG00000054965	FAM168A
1332.19159	-0.302950157	0.192088169	-1.577141155	0.114763049	0.923922314	ENSG00000017797	RALBP1
1338.217757	0.096426073	0.217464128	0.443411395	0.657468195	0.997568624	ENSG00000139218	SCAF11
1342.115248	0.005773353	0.208075795	0.027746395	0.97786442	0.999527751	ENSG00000136051	WASHC4

1331.692572	0.088867109	0.186474516	0.476564364	0.633672364	0.995376704	ENSG00000105612	DNASE2
1348.792858	0.917065795	0.515018653	1.780645787	0.074970335	0.893390506	ENSG00000147852	VLDLR
1318.100562	-0.447935459	0.234391904	-1.911053462	0.055997711	0.847598083	ENSG00000167657	DAPK3
1337.490684	-0.693882005	0.31699537	-2.188934195	0.028601623	0.770696352	ENSG00000127418	FGFRL1
1336.560714	0.007890775	0.221422988	0.035636658	0.971572078	0.999527751	ENSG00000114439	BBX
1336.396375	-0.122910753	0.206521555	-0.595147338	0.551744988	0.99483168	ENSG00000198952	SMG5
1333.893607	-0.081897644	0.254211212	-0.322163778	0.747328624	0.999467288	ENSG00000176407	KCMF1
1315.945838	0.115788403	0.223611571	0.517810426	0.604590543	0.99483168	ENSG00000159348	CYB5R1
1321.036025	-0.100474793	0.224342476	-0.447863441	0.654251755	0.997568624	ENSG00000196975	ANXA4
1317.801721	-0.555040719	0.250568476	-2.215125889	0.026751425	0.765090753	ENSG00000125753	VASP
1306.460268	-0.381980169	0.222431994	-1.717289689	0.085926255	0.901307093	ENSG00000175550	DRAP1
1331.0846	-0.226086104	0.293743656	-0.769671442	0.441494815	0.99135833	ENSG00000004455	AK2
1314.989307	-0.223475806	0.222292654	-1.005322498	0.314741585	0.974768742	ENSG00000230989	HSBP1
1318.976644	-0.449300365	0.295478304	-1.520586651	0.128363599	0.928627271	ENSG00000255112	CHMP1B
1333.963384	-0.086257162	0.3107079	-0.277614963	0.781307944	0.999467288	ENSG00000150347	ARID5B
1317.848902	-0.394147273	0.188497628	-2.090993285	0.036528666	0.789688071	ENSG00000142186	SCYL1
1334.231984	0.130736201	0.269924322	0.484343909	0.628141819	0.99483168	ENSG00000110422	HIPK3
1319.449021	0.393579488	0.263125211	1.495787831	0.134708953	0.933741583	ENSG00000149084	HSD17B12
1327.428068	-0.086136093	0.190767115	-0.451524848	0.65161132	0.997449432	ENSG00000205581	HMGN1
1336.815316	-0.015354914	0.301623507	-0.050907552	0.959399188	0.999527751	ENSG00000169967	MAP3K2
1327.79818	-0.249363965	0.242146761	-1.029805082	0.303101515	0.972793733	ENSG00000152767	FARP1
1311.279996	-0.214682918	0.237519692	-0.903853134	0.366073283	0.981269214	ENSG00000117758	STX12
1326.133763	0.339084469	0.243552089	1.392246194	0.163847859	0.943223078	ENSG00000152332	UHMK1
1327.53615	0.050326309	0.195572268	0.257328451	0.796925231	0.999467288	ENSG00000154845	PPP4R1
1325.170393	-0.01921706	0.226557666	-0.084821938	0.932402952	0.999527751	ENSG00000120686	UFM1
1333.089289	-0.292329365	0.260487549	-1.1222393	0.261760707	0.962465655	ENSG00000069956	MAPK6
1328.926611	0.029152679	0.292950541	0.099513996	0.920730176	0.999527751	ENSG00000010810	FYN
1322.0016	0.265758765	0.234255595	1.134482039	0.256592404	0.962465655	ENSG00000072042	RDH11
1318.500343	-0.01289314	0.205730775	-0.062669962	0.950029317	0.999527751	ENSG00000107862	GBF1
1307.836298	0.233574285	0.269381605	0.867075854	0.38590045	0.986238739	ENSG00000214063	TSPAN4
1311.537455	-0.146428054	0.277569113	-0.527537276	0.597820538	0.99483168	ENSG00000135048	CEMIP2
1322.99702	-0.12813402	0.20484033	-0.625531213	0.53162247	0.99483168	ENSG00000134001	EIF2S1
1321.868493	0.012623054	0.253093332	0.049875095	0.960221924	0.999527751	ENSG00000058272	PPP1R12A
1318.315125	0.048674237	0.234118943	0.207903884	0.835304017	0.999467288	ENSG00000117114	ADGRL2
1309.822052	0.024683276	0.205004021	0.120403864	0.904163231	0.999527751	ENSG00000100225	FBXO7
1321.118032	0.056284336	0.204572013	0.275132143	0.783214718	0.999467288	ENSG00000054118	THRAP3
1310.730913	-0.217240245	0.220394435	-0.98568843	0.324286029	0.975494399	ENSG00000243317	STMP1
1300.031171	-0.217767003	0.250036575	-0.870940594	0.38378659	0.985655313	ENSG00000132471	WBP2
1303.945183	-0.423001969	0.256461884	-1.649375584	0.099070713	0.921147998	ENSG00000078902	TOLLIP
1316.531564	0.125797558	0.179569733	0.700549895	0.483583957	0.991892545	ENSG00000055917	PUM2
1308.810305	0.148896598	0.360274763	0.41328623	0.679396917	0.998869317	ENSG00000149218	ENDOD1
1313.824988	-0.034162476	0.20231501	-0.168857844	0.865908458	0.999467288	ENSG00000087111	PIGS
1293.469879	-0.027852784	0.2840267	-0.098063964	0.921881502	0.999527751	ENSG00000132274	TRIM22
1309.615578	-0.041299514	0.205156434	-0.201307426	0.840458196	0.999467288	ENSG00000166200	COPS2
1317.050535	0.357279143	0.249568022	1.431590234	0.152261126	0.938052926	ENSG00000213593	TMX2
1311.852209	0.061317845	0.509524929	0.12034317	0.904211308	0.999527751	ENSG00000119950	MXI1
1296.469502	-0.174958168	0.213688221	-0.818754387	0.41292656	0.990226751	ENSG00000071859	FAM50A
1310.527155	-0.120631024	0.183853605	-0.656125421	0.511743429	0.993513219	ENSG00000167881	SRP68
1294.467919	0.444190092	0.266149291	1.668950874	0.095127116	0.921147998	ENSG00000159363	ATP13A2
1294.122274	-0.130665278	0.245992067	-0.531176797	0.595296271	0.99483168	ENSG00000143252	SDHC
1305.684393	0.141736175	0.338314412	0.418948087	0.675254073	0.998869317	ENSG00000144867	SRPRB
1292.709624	0.106194938	0.218584217	0.48583077	0.627087156	0.99483168	ENSG00000130202	NECTIN2
1304.183164	0.189339331	0.292908006	0.646412278	0.518012391	0.993513219	ENSG00000113273	ARSB
1312.539204	-0.312025936	0.251227821	-1.242003908	0.21423512	0.954310328	ENSG00000125875	TBC1D20
1304.443205	-0.289195674	0.191408641	-1.510880976	0.130818779	0.929373153	ENSG00000119383	PTPA
1310.505794	-0.51788604	0.317347056	-1.631923253	0.102695655	0.92169898	ENSG00000132000	PODNL1
1308.383427	0.079402909	0.193153253	0.411087606	0.681008298	0.998869317	ENSG00000092201	SUPT16H
1300.692269	-0.00635655	0.25589324	-0.024840632	0.980182081	0.999527751	ENSG00000112992	NNT
1292.070543	-0.125638306	0.264845457	-0.474383467	0.635226481	0.995506752	ENSG00000133243	BTBD2
1301.651369	0.340745386	0.207823216	1.639592496	0.101089924	0.92169898	ENSG00000182944	EWSR1
1307.29673	0.056472095	0.19284305	0.292839671	0.769644704	0.999467288	ENSG00000141380	SS18
1299.842653	-0.08798647	0.218450445	-0.402775422	0.687113444	0.998869317	ENSG00000070413	DGCR2
1295.973184	-0.150282909	0.205725394	-0.730502469	0.465083104	0.99135833	ENSG00000109919	MTCH2
1309.033116	-0.063533722	0.216827642	-0.293014864	0.76951079	0.999467288	ENSG00000126261	UBA2

1297.732022	0.236616537	0.190723713	1.240624636	0.214744447	0.954310328	ENSG00000115839	RAB3GAP1
1300.910246	-0.048506739	0.200635246	-0.241765791	0.808961648	0.999467288	ENSG00000131966	ACTR10
1302.670674	0.130645696	0.254569442	0.513202587	0.607809616	0.99483168	ENSG00000163602	RYBP
1292.413225	0.369813426	0.215274747	1.717867198	0.085820841	0.901307093	ENSG00000164828	SUN1
1299.480927	-0.070081199	0.212864217	-0.329229591	0.741982158	0.999467288	ENSG00000114650	SCAP
1306.969949	-0.768011025	0.314739832	-2.440145623	0.014681342	0.686410066	ENSG00000131435	PDLIM4
1300.530231	0.325001195	0.237406024	1.368967769	0.17100935	0.944422693	ENSG00000153201	RANBP2
1285.261475	-0.001676708	0.232076828	-0.007224795	0.994235498	0.999527751	ENSG00000186866	POFUT2
1298.555234	0.251292165	0.207178976	1.212923098	0.225159224	0.958865115	ENSG00000138246	DNAJC13
1314.031882	0.087938736	0.291557621	0.301617003	0.762944045	0.999467288	ENSG00000100596	SPTLC2
1300.99264	0.022889543	0.220660138	0.103732116	0.917381939	0.999527751	ENSG00000009954	BAZ1B
1291.499063	0.190952142	0.330698905	0.57741994	0.563655807	0.99483168	ENSG00000170581	STAT2
1286.952157	0.217044086	0.214170111	1.013419122	0.31086001	0.973427483	ENSG00000130177	CDC16
1290.630018	-0.057281216	0.18618071	-0.307664615	0.758337547	0.999467288	ENSG00000134287	ARF3
1275.615016	-0.389364583	0.289031452	-1.347135684	0.177936537	0.9469453	ENSG00000123159	GIPC1
1290.06829	0.368167275	0.212021413	1.736462695	0.082482055	0.899922174	ENSG00000109606	DHX15
1274.379635	-0.31720839	0.27183842	-1.166900506	0.243250552	0.962465655	ENSG00000051523	CYBA
1293.038438	0.178333988	0.295568007	0.603360255	0.546269056	0.99483168	ENSG00000120694	HSPH1
1286.400957	-0.1762297	0.242211012	-0.727587481	0.466866146	0.99135833	ENSG00000116649	SRM
1281.017572	0.20629625	0.28486282	0.724195069	0.468945985	0.99135833	ENSG00000105854	PON2
1268.303727	-0.020123804	0.31051469	-0.064807897	0.948326954	0.999527751	ENSG00000079459	FDFT1
1285.996537	0.177739637	0.314579155	0.565007675	0.572068544	0.99483168	ENSG00000131238	PPT1
1290.906942	-0.018651092	0.18273027	-0.102068977	0.918701925	0.999527751	ENSG00000173674	EIF1AX
1274.739627	0.301208363	0.232951106	1.293011086	0.196007225	0.954310328	ENSG00000143537	ADAM15
1296.754026	0.158137749	0.268367533	0.589258125	0.555688131	0.99483168	ENSG00000169826	CSGALNACT2
1285.104242	-0.016335037	0.202745426	-0.080569204	0.935784559	0.999527751	ENSG00000140367	UBE2Q2
1287.488673	-0.065390696	0.251333587	-0.260174922	0.794728848	0.999467288	ENSG00000125107	CNOT1
1271.438026	-0.380200004	0.254098371	-1.496270921	0.13458307	0.933741583	ENSG00000004142	POLDIP2
1285.49022	0.175935265	0.261201598	0.673561213	0.50059032	0.993513219	ENSG00000077097	TOP2B
1281.749064	0.317005245	0.322659356	0.982476531	0.32586514	0.976026345	ENSG00000169032	MAP2K1
1289.772076	-0.39293299	0.519148581	-0.756879638	0.449121975	0.99135833	ENSG00000143341	HMCN1
1272.289744	0.043431213	0.237916986	0.18254776	0.855152877	0.999467288	ENSG00000148834	GSTO1
1279.153917	-0.191091497	0.194074156	-0.984631347	0.324805186	0.976026345	ENSG00000134419	RPS15A
1278.925888	0.102412217	0.181671534	0.56372187	0.572943431	0.99483168	ENSG00000204574	ABCF1
1282.366593	-0.210742679	0.296089898	-0.711752343	0.476618149	0.99135833	ENSG00000142657	PGD
1279.021442	0.079714757	0.183936309	0.433382389	0.664736992	0.997568624	ENSG00000132670	PTPRA
1279.733293	-0.113150205	0.191025435	-0.592330571	0.553629248	0.99483168	ENSG00000080371	RAB21
1273.727891	0.032904012	0.218291804	0.150734068	0.880185498	0.999467288	ENSG00000117362	APH1A
1274.608054	-0.028580349	0.200433297	-0.142592822	0.886611767	0.999467288	ENSG00000185627	PSMD13
1264.581505	0.583923612	0.305380081	1.912120824	0.055860699	0.847598083	ENSG00000163659	TIPARP
1292.027666	-0.258167799	0.378038143	-0.682914684	0.494660755	0.993513219	ENSG00000099998	GGT5
1273.86141	-0.109451547	0.223793171	-0.489074562	0.62478891	0.99483168	ENSG00000151240	DIP2C
1280.057784	-0.21696279	0.199564081	-1.087183571	0.27695569	0.962465655	ENSG00000105270	CLIP3
1279.632001	0.061911741	0.292176053	0.211898751	0.832186025	0.999467288	ENSG00000134853	PDGFRA
1244.057888	0.505055996	0.397660469	1.270068401	0.204060266	0.954310328	ENSG00000154856	APCDD1
1268.672883	-0.422953499	0.252783818	-1.673182649	0.094291345	0.921147998	ENSG00000105221	AKT2
1242.592566	0.070797724	1.565521322	0.045223098	0.963929484	0.999527751	ENSG00000159167	STC1
1252.071077	0.287326454	0.393322954	0.730510264	0.465078342	0.99135833	ENSG00000170624	SGCD
1263.715002	-0.13639729	0.234098983	-0.582647938	0.560130327	0.99483168	ENSG00000179364	PACS2
1277.847269	0.036532698	0.1973343	0.185131009	0.853126281	0.999467288	ENSG00000103429	BFAR
1263.622666	-0.007171907	0.242316186	-0.029597309	0.976388211	0.999527751	ENSG00000041357	PSMA4
1283.507897	-0.247611842	0.409103955	-0.60525409	0.545010181	0.99483168	ENSG00000149380	P4HA3
1272.832061	-0.287705358	0.212081275	-1.356580672	0.174914487	0.946176003	ENSG00000179134	SAMD4B
1260.489886	0.194305438	0.270109333	0.719358473	0.471920076	0.99135833	ENSG00000138698	RAP1GDS1
1280.289789	-0.238435499	0.268261277	-0.888818176	0.374100806	0.982609538	ENSG00000176903	PNMA1
1270.611877	-0.054812718	0.186257976	-0.294283869	0.768540998	0.999467288	ENSG00000076043	REXO2
1257.964972	-0.21578316	0.234691739	-0.919432279	0.357869512	0.979427314	ENSG00000111652	COPS7A
1272.493076	-0.253801156	0.343798481	-0.738226518	0.460376811	0.99135833	ENSG00000144579	CTDSP1
1238.654669	-0.346620384	0.397950619	-0.871013557	0.383746751	0.985655313	ENSG00000177700	POLR2L
1268.041141	-0.340768547	0.307902515	-1.106741682	0.268405628	0.962465655	ENSG00000183010	PYCR1
1267.679168	-0.106006712	0.269954955	-0.392682966	0.694553654	0.998869317	ENSG00000140450	ARRDC4
1271.165216	0.143136059	0.222444216	0.643469457	0.519919527	0.993513219	ENSG00000118217	ATF6
1288.270302	0.171371913	0.252826286	0.677824745	0.49788282	0.993513219	ENSG00000080503	SMARCA2
1250.570564	-0.308249624	0.367309646	-0.839209172	0.401351941	0.988527641	ENSG00000128656	CHN1

1247.364144	0.619851495	0.326724106	1.897170991	0.057805374	0.852306368	ENSG00000148516	ZEB1	
1259.824452	0.214027222	0.21822517	0.980763227	0.326709516	0.976026345	ENSG00000120265	PCMT1	
1268.138871	0.110324513	0.235249876	0.468967356	0.639092969	0.996553586	ENSG00000103249	CLCN7	
1270.984648	-0.420409695	0.221270326	-1.89998226	0.057435448	0.852306368	ENSG00000101350	KIF3B	
1285.732919	-0.351951421	0.263221775	-1.337090828	0.181192991	0.949094286	ENSG00000131791	PRKAB2	
1254.315023	0.143729395	0.25742494	0.558335159	0.57661554	0.99483168	ENSG00000102038	SMARCA1	
1272.833903	0.276420872	0.251264523	1.100118987	0.271280282	0.962465655	ENSG00000113580	NR3C1	
1287.147974	0.28505153	0.339486475	0.839655041	0.401101829	0.988492517	ENSG00000128050	PAICS	
1250.509645	-0.071920814	0.231779618	-0.310298269	0.756334147	0.999467288	ENSG00000126698	DNAJC8	
1236.936741	-0.327724895	0.428511373	-0.764798592	0.444391486	0.99135833	ENSG00000099953	MMP11	
1261.599485	-0.028369764	0.195080797	-0.145425714	0.884374763	0.999467288	ENSG00000113282	CLINT1	
1275.282196	0.233688508	0.248130659	0.941796187	0.346296998	0.977691382	ENSG00000113194	FAF2	
1251.247208	-0.202278722	0.203252196	-0.995210512	0.31963389	0.974964731	ENSG00000185236	RAB11B	
1261.659867	-0.164325138	0.219571199	-0.748391128	0.454224272	0.99135833	ENSG00000159658	EFCAB14	
1243.510951	0.103783816	0.229099821	0.453006971	0.650543722	0.997381078	ENSG00000104824	HNRNPL	
1248.175159	-0.495240818	0.273714156	-1.809335786	0.070398853	0.87954613	ENSG00000054148	PHPT1	
1258.13302	-0.370992407	0.233311724	-1.590114717	0.111808949	0.923922314	ENSG00000175224	ATG13	
1258.730927	-0.257089909	0.236986503	-1.084829328	0.277997253	0.962465655	ENSG00000139645	ANKRD52	
1241.519951	-0.409112964	0.330501172	-1.237856319	0.215769341	0.954310328	ENSG00000131408	NR1H2	
1259.469864	0.235912357	0.190173819	1.240509123	0.214787142	0.954310328	ENSG00000140299	BNIP2	
1256.789896	-0.535336947	0.289745906	-1.847608323	0.064659025	0.868202292	ENSG00000171148	TADA3	
1256.775288	-0.309679453	0.352906513	-0.877511301	0.380208983	0.983585589	ENSG00000102996	MMP15	
1258.810245	-0.311965941	0.185556277	-1.681247035	0.092714941	0.921147998	ENSG00000108312	UBTF	
1279.634328	0.122478631	0.454467091	0.269499449	0.787545366	0.999467288	ENSG00000116584	ARHGEF2	
1253.734845	0.023815275	0.200244141	0.118931194	0.905329868	0.999527751	ENSG00000119414	PPP6C	
1255.023362	-0.225421792	0.210193246	-1.072450214	0.283517864	0.963267117	ENSG00000101444	AHCY	
1228.917883	0.619806735	0.457893945	1.353603255	0.175862988	0.946278819	ENSG00000127863	TNFRSF19	
1250.879052	-0.159946393	0.200262645	-0.798683116	0.42447418	0.991301167	ENSG00000182827	ACBD3	
1247.377763	-0.532302448	0.302905974	-1.757319084	0.078863437	0.897052166	ENSG00000140511	HAPLN3	
1256.397732	-0.096313753	0.18288907	-0.52662389	0.598454799	0.99483168	ENSG00000166181	API5	
1253.519296	0.333027855	0.296411132	1.123533561	0.261210955	0.962465655	ENSG00000110841	PPFIBP1	
1253.184481	0.246821906	0.237965423	1.03721752	0.299634495	0.972186492	ENSG00000188706	ZDHHC9	
1245.951306	-0.124871915	0.242961654	-0.513957297	0.607281847	0.99483168	ENSG00000134375	TIMM17A	
1255.441732	-0.28264152	0.197216727	-1.433151863	0.151814443	0.938052926	ENSG00000116221	MRPL37	
1258.301912	0.106055854	0.203586556	0.520937414	0.602410371	0.99483168	ENSG00000123684	LPGAT1	
1241.026102	-0.064177427	0.313537484	-0.204688211	0.837815732	0.999467288	ENSG00000119812	FAM98A	
1247.086496	-0.230831374	0.190477062	-1.21185917	0.225566294	0.958865115	ENSG00000063244	U2AF2	
1253.223756	0.069390736	0.190739464	0.363798525	0.716008464	0.99900742	ENSG00000122958	VPS26A	
1259.255956	0.163430848	0.240345865	0.679981939	0.496515897	0.993513219	ENSG00000065613	SLK	
1256.170298	-0.303341521	0.220228022	-1.377397471	0.168389394	0.943223078	ENSG00000006451	RALA	
1247.873403	-0.014644317	0.233732311	-0.062654228	0.950041847	0.999527751	ENSG00000087470	DNM1L	
1245.035435	-0.41644616	0.257378156	-1.61803226	0.105655646	0.92169898	ENSG00000156467	UQCRB	
1257.224686	0.18221298	0.266977916	0.682502071	0.494921534	0.993513219	ENSG00000152700	SAR1B	
1249.446303	0.148276856	0.216960377	0.683428271	0.494336261	0.993513219	ENSG00000127616	SMARCA4	
1248.520404	0.273082798	0.228777533	1.193660908	0.232610638	0.959575179	ENSG00000132305	IMMT	
1249.373409	-0.105404461	0.193197639	-0.545578412	0.585355767	0.99483168	ENSG00000131508	UBE2D2	
1252.658948	0.189481468	0.183478755	1.03271612	0.301736768	0.972793733	ENSG00000186522		10-Sep
1246.414837	0.145059049	0.251130769	0.57762356	0.563518297	0.99483168	ENSG00000131871	SELENOS	
1238.21515	0.123889742	0.225793466	0.548686124	0.58322087	0.99483168	ENSG00000122729	ACO1	
1250.914873	-0.266108359	0.209538264	-1.26997501	0.204093532	0.954310328	ENSG00000188895	MSL1	
1235.386671	-0.174280114	0.21921838	-0.795006853	0.426609516	0.991301167	ENSG00000105520	PLPPR2	
1230.535095	-0.474188162	0.259997542	-1.823817862	0.068179613	0.87420149	ENSG00000205302	SNX2	
1239.933378	0.147794551	0.252300681	0.585787364	0.558018416	0.99483168	ENSG00000105968	H2AFV	
1251.199054	-0.161483732	0.209297955	-0.771549499	0.440381294	0.99135833	ENSG00000104324	CPQ	
1242.569294	-0.004341206	0.179933053	-0.024126787	0.980751476	0.999527751	ENSG00000014824	SLC30A9	
1245.576425	-0.62890732	0.222359635	-2.82833402	0.004679096	0.47075409	ENSG00000113716	HMGXB3	
1240.808089	-0.450971054	0.225526544	-1.999636256	0.045539556	0.813244956	ENSG00000115944	COX7A2L	
1242.842166	-0.078739196	0.191135623	-0.411954585	0.680372711	0.998869317	ENSG00000183020	AP2A2	
1241.36473	-0.052072642	0.26171182	-0.198969394	0.842286686	0.999467288	ENSG00000132432	SEC61G	
1246.911736	0.057573641	0.236394891	0.243548585	0.807580453	0.999467288	ENSG00000118200	CAMSAP2	
1238.62898	-0.108049364	0.196460048	-0.549981359	0.582332159	0.99483168	ENSG00000181904	C5orf24	
1241.568337	-0.129201693	0.177523547	-0.72780031	0.466735835	0.99135833	ENSG00000107758	PPP3CB	
1241.876599	-0.072753259	0.261291126	-0.278437544	0.780676503	0.999467288	ENSG00000099622	CIRBP	
1239.094853	-0.184321799	0.188184075	-0.979476076	0.327344804	0.976026345	ENSG00000157954	WIPI2	

1234.554901	-0.413669298	0.185306033	-2.23235742	0.02559135	0.760526466	ENSG00000058091	CDK14
1246.662292	0.093150037	0.30812105	0.302316368	0.762410902	0.999467288	ENSG00000077235	GTF3C1
1230.199247	-0.145082566	0.194376976	-0.746397893	0.455427092	0.99135833	ENSG00000276293	PIP4K2B
1240.10512	-0.056443107	0.203519817	-0.277334697	0.78152312	0.999467288	ENSG00000171490	RSL1D1
1232.536906	-0.274013041	0.192746197	-1.421626186	0.155134799	0.939432741	ENSG00000116473	RAP1A
1231.433785	0.073143343	0.241585456	0.302763853	0.762069832	0.999467288	ENSG00000130638	ATXN10
1198.568532	-0.522368298	0.297407681	-1.756404866	0.079019303	0.897052166	ENSG00000151729	SLC25A4
1224.094673	-0.343887685	0.219727057	-1.565067544	0.117567073	0.923922314	ENSG00000179950	PUF60
1233.822589	0.01327192	0.31957202	0.041530293	0.966873143	0.999527751	ENSG00000154511	FAM69A
1227.206966	-0.222840018	0.213006292	-1.046166366	0.295484229	0.970706863	ENSG00000160714	UBE2Q1
1240.158754	0.063625237	0.223017539	0.285292528	0.775420037	0.999467288	ENSG00000004897	CDC27
1226.243989	-0.035911619	0.185325373	-0.193776049	0.846351241	0.999467288	ENSG00000116199	FAM20B
1241.285639	0.285383519	0.235486302	1.211890103	0.225554451	0.958865115	ENSG00000169018	FEM1B
1226.688834	0.227097201	0.241675744	0.939677261	0.347383132	0.977691382	ENSG00000158417	EIF5B
1217.590394	-0.084147404	0.220650612	-0.381360394	0.702935846	0.99900742	ENSG00000205531	NAP1L4
1235.048565	-0.227066847	0.231020346	-0.982886795	0.325663158	0.976026345	ENSG00000107854	TNKS2
1227.326001	0.35302382	0.26194969	1.347677948	0.177761988	0.9469453	ENSG00000140443	IGF1R
1225.755631	-0.104575449	0.18771333	-0.557101879	0.577457824	0.99483168	ENSG00000025800	KPNA6
1232.05716	0.348260323	0.236686189	1.471401116	0.141182672	0.935644597	ENSG00000085365	SCAMP1
1251.189207	0.399988154	0.772863156	0.51754072	0.604778751	0.99483168	ENSG00000204291	COL15A1
1224.851133	0.383860831	0.244135822	1.572324895	0.115875222	0.923922314	ENSG00000107566	ERLIN1
1231.782971	-0.983143301	1.169825919	-0.840418463	0.400673801	0.988492517	ENSG00000115884	SDC1
1216.966863	-0.1071597	0.282228941	-0.379690685	0.704175035	0.99900742	ENSG00000214022	REPIN1
1219.708774	-0.27708371	0.19493475	-1.421417728	0.155195356	0.939432741	ENSG00000103353	UBFD1
1224.84062	0.065699549	0.20672123	0.317817133	0.750623654	0.999467288	ENSG00000170242	USP47
1200.056314	0.299250982	0.342129572	0.874671488	0.381752678	0.985369679	ENSG00000145349	CAMK2D
1226.248938	0.453958297	0.242769703	1.869913299	0.061495859	0.864834166	ENSG00000146648	EGFR
1226.873982	-0.053839196	0.19944389	-0.269946579	0.787201352	0.999467288	ENSG00000198231	DDX42
1226.739968	-0.208764656	0.215981307	-0.966586687	0.333750693	0.976026345	ENSG00000172239	PAIP1
1214.010687	-0.300977006	0.210969802	-1.426635481	0.153684992	0.939432741	ENSG00000115561	CHMP3
1209.275279	0.226223527	0.265140138	0.853222482	0.393535938	0.988378925	ENSG00000159128	IFNGR2
1230.897623	-0.338458656	0.218076332	-1.552019206	0.120657628	0.923922314	ENSG00000119638	NEK9
1224.349901	0.101420079	0.226084767	0.448593155	0.653725172	0.997527562	ENSG00000138095	LRPPRC
1222.37961	0.136109724	0.236892365	0.574563574	0.565586493	0.99483168	ENSG00000118363	SPCS2
1208.104174	-0.089483195	0.300934981	-0.297350594	0.766198859	0.999467288	ENSG00000178035	IMPDH2
1212.886937	0.304057754	0.34959714	0.869737533	0.384443856	0.985655313	ENSG00000104341	LAPTM4B
1200.003676	-0.311545939	0.239454847	-1.301063404	0.193236753	0.950382812	ENSG00000196821	C6orf106
1183.995632	0.13753858	0.34599187	0.397519687	0.690984272	0.998869317	ENSG00000136861	CDK5RAP2
1220.208774	0.308116399	0.217710927	1.415254638	0.15699385	0.939464191	ENSG00000058063	ATP11B
1212.528561	-0.129013523	0.282802096	-0.456197196	0.648248181	0.997368416	ENSG00000164253	WDR41
1221.398482	0.302694051	0.216975738	1.395059442	0.162997915	0.943223078	ENSG00000156113	KCNMA1
1220.716606	-0.106093503	0.275387472	-0.38525174	0.700050913	0.998869317	ENSG00000159592	GPBP1L1
1208.386728	0.086163392	0.202929127	0.424598444	0.671129428	0.997568624	ENSG00000186063	AIDA
1190.18926	-0.536679292	0.503946921	-1.064952021	0.286897642	0.965284229	ENSG00000183691	NOG
1207.963095	-0.165666605	0.325056795	-0.509654336	0.61029365	0.99483168	ENSG00000121691	CAT
1214.3991	-0.082433725	0.244011503	-0.337827208	0.735493405	0.999467288	ENSG00000061273	HDAC7
1195.482498	-0.38912982	0.293359578	-1.326460255	0.184687292	0.949094286	ENSG00000087088	BAX
1216.032627	-0.010592747	0.207244244	-0.051112381	0.95923597	0.999527751	ENSG00000196504	PRPF40A
1202.006724	-0.129606815	0.242627588	-0.53418004	0.593216977	0.99483168	ENSG00000112062	MAPK14
1219.120474	0.014862531	0.224802914	0.066113606	0.947287379	0.999527751	ENSG00000104067	TJP1
1222.540804	0.205400396	0.255804314	0.80295908	0.421998386	0.991301167	ENSG00000253352	TUG1
1246.729014	0.166618352	0.480794226	0.346548155	0.728930801	0.999467288	ENSG00000100889	PCK2
1198.581046	-0.120053096	0.240956943	-0.498234642	0.618318668	0.99483168	ENSG00000028528	SNX1
1212.249321	0.099544742	0.236798844	0.420376809	0.674210207	0.998869317	ENSG00000054793	ATP9A
1203.621965	-0.153391001	0.192815405	-0.795532915	0.426303572	0.991301167	ENSG00000141002	TCF25
1201.776205	0.191401782	0.240254232	0.796663518	0.425646477	0.991301167	ENSG00000136816	TOR1B
1206.674731	-0.21834515	0.184400588	-1.184080553	0.236381177	0.962465655	ENSG00000189241	TSPYL1
1208.072694	0.279288988	0.312823816	0.892799632	0.371964483	0.981955904	ENSG00000184500	PROS1
1207.774787	0.01341195	0.235200521	0.057023469	0.9545265	0.999527751	ENSG00000166747	AP1G1
1200.281531	-0.181579388	0.202971505	-0.89460532	0.370998107	0.981955904	ENSG00000186660	ZFP91
1195.672394	-0.306921652	0.200287425	-1.532406003	0.125422277	0.928208753	ENSG00000124357	NAGK
1191.888911	0.086088237	0.310139604	0.277578987	0.781335564	0.999467288	ENSG00000114450	GNB4
1200.905314	0.078478463	0.192572191	0.407527497	0.683620603	0.998869317	ENSG00000144455	SUMF1
1199.900239	0.408627078	0.390231934	1.047139001	0.295035474	0.970706863	ENSG00000135052	GOLM1

1187.701358	-0.023100158	0.235613405	-0.098042633	0.921898441	0.999527751	ENSG00000177889	UBE2N
1204.199161	0.094440475	0.193999124	0.486808771	0.626393854	0.99483168	ENSG00000115486	GGCX
1152.680399	-0.617715751	0.424920909	-1.453719358	0.146024129	0.936017041	ENSG00000064205	WISP2
1213.991632	-0.565815713	0.350012616	-1.616558052	0.105973713	0.922095158	ENSG00000149212	SESN3
1198.082773	-0.044209071	0.198398951	-0.22282916	0.823668472	0.999467288	ENSG00000241973	PI4KA
1200.522408	-0.001668234	0.207796764	-0.008028202	0.99359449	0.999527751	ENSG00000055130	CUL1
1195.08667	0.267314639	0.239568306	1.115818047	0.264500027	0.962465655	ENSG00000136527	TRA2B
1201.3966	-0.112950761	0.219163572	-0.515371965	0.606293119	0.99483168	ENSG00000143164	DCAF6
1144.360623	0.744202144	0.51898373	1.433960452	0.151583549	0.938052926	ENSG00000184292	TACSTD2
1189.180241	0.172549948	0.318149996	0.542354079	0.587574608	0.99483168	ENSG00000119977	TCTN3
1195.339205	0.032176585	0.24119817	0.133403107	0.893874588	0.999467288	ENSG00000153317	ASAP1
1195.228576	0.254551712	0.309488465	0.822491758	0.410797075	0.990226751	ENSG00000073008	PVR
1196.289344	-0.178398018	0.337494721	-0.528594988	0.597086438	0.99483168	ENSG00000067113	PLPP1
1201.132667	-0.327114918	0.282312748	-1.158696942	0.246579738	0.962465655	ENSG00000100221	JOSD1
1184.695595	-0.237690595	0.267696198	-0.887911732	0.374588233	0.982609538	ENSG00000068323	TFE3
1185.678647	0.030139817	0.212151802	0.142067219	0.887026911	0.999467288	ENSG00000111666	CHPT1
1187.124082	-0.014441984	0.20385105	-0.070845766	0.943520507	0.999527751	ENSG00000165169	DYNLT3
1197.323573	-0.035499747	0.282612693	-0.125612712	0.9000385	0.999527751	ENSG00000099942	CRKL
1182.061976	-0.20740803	0.203556457	-1.0189214	0.308240283	0.973427483	ENSG00000159352	PSMD4
1191.802226	-0.219638071	0.184375302	-1.191255379	0.233553345	0.959684953	ENSG00000163930	BAP1
1200.55	-0.904145225	0.349022246	-2.59050887	0.009583415	0.622034307	ENSG00000137507	LRRC32
1179.137906	-0.20626477	0.325895596	-0.632916714	0.526788032	0.99483168	ENSG00000107816	LZTS2
1190.932763	0.085278273	0.222136428	0.38390044	0.70105224	0.998869317	ENSG00000137876	RSL24D1
1187.516723	-0.500192812	0.317014254	-1.577824358	0.114605966	0.923922314	ENSG00000179632	MAF1
1218.29703	-0.501385469	0.400729299	-1.251182457	0.210867917	0.954310328	ENSG00000160161	CILP2
1177.994284	-0.131167154	0.259157793	-0.506128537	0.612766418	0.99483168	ENSG00000113387	SUB1
1163.253476	-0.222776222	0.367641698	-0.60596016	0.544541209	0.99483168	ENSG00000112576	CCND3
1193.470148	-0.251000555	0.283171669	-0.88639007	0.375407368	0.982609538	ENSG00000100299	ARSA
1170.033865	-0.458171671	0.257197049	-1.781403299	0.07484659	0.893390506	ENSG00000124299	PEPD
1193.817844	-0.133597505	0.228757687	-0.584013185	0.559211441	0.99483168	ENSG00000109572	CLCN3
1193.456837	0.536639875	0.258505603	2.07593131	0.037900304	0.790190505	ENSG00000112378	PERP
1193.426983	0.10099067	0.332028836	0.304162347	0.761004209	0.999467288	ENSG00000147650	LRP12
1185.689283	-0.069749492	0.184615761	-0.377808976	0.705572505	0.99900742	ENSG00000066044	ELAVL1
1177.313445	-0.005674425	0.242979876	-0.023353477	0.981368315	0.999527751	ENSG00000163683	SMIM14
1210.734908	0.209208056	0.432565574	0.483644721	0.628638031	0.99483168	ENSG00000237973	MTCO1P12
1188.678476	-0.088215764	0.2978938	-0.296131589	0.76712959	0.999467288	ENSG00000121064	SCPEP1
1185.886787	-0.153761553	0.283854431	-0.541691574	0.588030995	0.99483168	ENSG00000173039	RELA
1162.713703	0.031785457	0.195758487	0.162370775	0.871013884	0.999467288	ENSG00000255248	MIR100HG
1170.626582	-0.314799061	0.22239262	-1.415510378	0.156918908	0.939464191	ENSG00000172531	PPP1CA
1177.868638	0.405106277	0.21710574	1.865939966	0.062049765	0.865910462	ENSG00000138594	TMOD3
1180.304126	0.182960738	0.264835882	0.690845728	0.489662495	0.993513219	ENSG00000168488	ATXN2L
1180.970451	0.203258929	0.250185405	0.812433201	0.416543102	0.990226751	ENSG00000104517	UBR5
1183.347914	0.265650626	0.314095883	0.845762841	0.397685059	0.988378925	ENSG00000133065	SLC41A1
1185.370314	0.098918378	0.235814516	0.419475356	0.674868761	0.998869317	ENSG00000101126	ADNP
1171.622992	0.140743089	0.202687203	0.694385668	0.487440379	0.993513219	ENSG00000119820	YIPF4
1161.550005	0.203091847	0.314939014	0.644860871	0.519017351	0.993513219	ENSG00000103994	ZNF106
1176.819792	-0.405384811	0.331265943	-1.223744304	0.221048701	0.956884001	ENSG00000186350	RXRA
1179.127202	0.264626155	0.235106598	1.125558179	0.260352578	0.962465655	ENSG00000154473	BUB3
1176.917947	0.125518912	0.21140526	0.593735993	0.552688705	0.99483168	ENSG00000121741	ZMYM2
1174.050435	0.046105645	0.183703588	0.250978469	0.801830755	0.999467288	ENSG00000198700	IPO9
1176.296729	-0.048614131	0.282373901	-0.172162266	0.863309962	0.999467288	ENSG00000137831	UACA
1177.053309	0.334289385	0.273142636	1.223863803	0.221003611	0.956884001	ENSG00000103978	TMEM87A
1176.976442	-0.13941772	0.2358125	-0.591222773	0.554371166	0.99483168	ENSG00000166326	TRIM44
1177.761969	-0.290668057	0.336445996	-0.86393674	0.387622642	0.986799595	ENSG00000135480	KRT7
1168.654877	-0.267836303	0.236011416	-1.134844692	0.256440397	0.962465655	ENSG00000147533	GOLGA7
1217.088857	-0.84032348	0.89931327	-0.934405739	0.350094639	0.977691382	ENSG00000177283	FZD8
1167.905398	0.018586461	0.199128565	0.093338998	0.925634251	0.999527751	ENSG00000165934	CPSF2
1160.198879	0.289089784	0.243399028	1.187719551	0.234943919	0.961317886	ENSG00000100997	ABHD12
1176.644732	0.199237908	0.232495988	0.856952029	0.391471397	0.988355932	ENSG00000197217	ENTPD4
1163.02008	0.352065736	0.223215511	1.577245838	0.114738969	0.923922314	ENSG00000117500	TMED5
1156.71365	-0.355559797	0.234289263	-1.517610294	0.12911267	0.928627271	ENSG00000013306	SLC25A39
1163.04777	-0.386216847	0.215720378	-1.79035866	0.073396272	0.888663898	ENSG00000025293	PHF20
1158.710104	-0.146278284	0.23494043	-0.622618612	0.533535178	0.99483168	ENSG00000131165	CHMP1A
1172.175732	-0.029145847	0.214333835	-0.135983417	0.89183439	0.999467288	ENSG00000197771	MCMBP

1169.10816	-0.070970671	0.300440654	-0.236221928	0.813260465	0.999467288	ENSG00000213614	HEXA
1155.852466	-0.462480507	0.268841416	-1.720272549	0.085382911	0.901307093	ENSG00000136003	ISCU
1192.662547	-0.307299943	0.452169211	-0.67961271	0.49674972	0.993513219	ENSG00000166173	LARP6
1164.663307	0.064956896	0.209520294	0.310026752	0.756540613	0.999467288	ENSG00000125166	GOT2
1159.27654	0.209516453	0.229522393	0.912836653	0.361328458	0.979465649	ENSG00000138385	SSB
1168.562994	-0.325030997	0.22250088	-1.460807693	0.144068224	0.935644597	ENSG00000100422	CERK
1164.501194	-0.021435102	0.204311214	-0.104913975	0.916444069	0.999527751	ENSG00000139687	RB1
1165.402438	-0.019047915	0.308239271	-0.061795875	0.950725389	0.999527751	ENSG00000106823	ECM2
1174.669829	-0.05675778	0.29005828	-0.195677159	0.844862863	0.999467288	ENSG00000107771	CCSER2
1181.499471	-0.183571593	0.309384654	-0.59334421	0.552950817	0.99483168	ENSG00000120063	GNA13
1159.593185	0.221866319	0.211132617	1.05083867	0.293332692	0.970706863	ENSG00000134318	ROCK2
1165.423741	0.404825901	0.382736485	1.057714425	0.290185657	0.967933358	ENSG00000178202	KDELC2
1161.28565	0.045575044	0.244173389	0.18665033	0.851934802	0.999467288	ENSG00000116521	SCAMP3
1168.417186	0.002594704	0.197310361	0.01315037	0.989507825	0.999527751	ENSG00000149136	SSRP1
1157.394255	-0.069102186	0.233832357	-0.295520205	0.76759652	0.999467288	ENSG00000064655	EYA2
1158.799931	0.236535291	0.222821978	1.061543807	0.288442835	0.966288987	ENSG00000125037	EMC3
1166.02656	-0.523565784	0.274361024	-1.908309629	0.056351209	0.847792202	ENSG00000259956	RBM15B
1153.875435	0.109848842	0.252177567	0.435601165	0.663126126	0.997568624	ENSG00000114902	SPCS1
1162.048569	-0.024367089	0.215635723	-0.113001172	0.910029626	0.999527751	ENSG00000023228	NDUFS1
1143.122022	-0.385462747	0.263571421	-1.462460329	0.143615108	0.935644597	ENSG00000112695	COX7A2
1150.41564	-0.262640785	0.315909499	-0.831379828	0.405759091	0.990045328	ENSG00000104870	FCGRT
1139.47126	2.457193892	0.412306676	5.959626742	2.53E-09	3.65E-05	ENSG00000251562	MALAT1
1150.987916	-0.239782687	0.195837102	-1.22439867	0.220801871	0.956884001	ENSG00000125818	PSMF1
1144.907857	0.163508985	0.259944884	0.629014053	0.529339856	0.99483168	ENSG00000105677	TMEM147
1153.832395	0.147247346	0.194358271	0.757607823	0.448685796	0.99135833	ENSG00000102531	FNDC3A
1150.320558	-0.24346191	0.218937072	-1.112017749	0.266130519	0.962465655	ENSG00000100568	VTI1B
1163.640908	-0.076426091	0.28820154	-0.265182798	0.790868646	0.999467288	ENSG00000013375	PGM3
1159.635004	-0.375966645	0.370906219	-1.013643411	0.310752935	0.973427483	ENSG00000181852	RNF41
1148.000543	-0.133428738	0.185037624	-0.721089774	0.470854283	0.99135833	ENSG00000148337	CIZ1
1146.916333	-0.022993287	0.192163827	-0.119654609	0.904756759	0.999527751	ENSG00000006715	VPS41
1144.152214	0.136474887	0.195341252	0.698648571	0.484771681	0.991892545	ENSG00000132383	RPA1
1152.756119	0.387819286	0.324354271	1.195665728	0.231827031	0.959575179	ENSG00000124207	CSE1L
1149.617988	-0.092674573	0.188205586	-0.492411382	0.622428554	0.99483168	ENSG00000211460	TSN
1129.574096	-0.307467656	0.294472233	-1.044131232	0.296424678	0.970706863	ENSG00000204941	PSG5
1116.654268	-0.669919866	0.791878503	-0.845988195	0.39755933	0.988378925	ENSG00000159251	ACTC1
1150.151471	-0.602416765	0.330686089	-1.821717891	0.068497802	0.87420149	ENSG00000143013	LMO4
1137.451591	0.105988766	0.232123106	0.45660584	0.647954381	0.997368416	ENSG00000142230	SAE1
1152.706558	-0.071203437	0.198723634	-0.358303818	0.720115961	0.99900742	ENSG00000109171	SLAIN2
1161.119589	0.29272856	0.339312624	0.86271049	0.388296664	0.986799595	ENSG00000148841	ITPRIP
1142.597423	-0.332365305	0.263260405	-1.262496369	0.206770232	0.954310328	ENSG00000178982	EIF3K
1151.786101	0.08571769	0.221691649	0.386652768	0.699013287	0.998869317	ENSG00000120137	PANK3
1134.168848	0.504862259	0.246382827	2.049096782	0.040452652	0.795991355	ENSG00000152818	UTRN
1142.51758	-0.217791287	0.308132391	-0.706810752	0.479684092	0.99135833	ENSG00000142327	RNPEPL1
1138.55234	-0.212929809	0.264943785	-0.803679199	0.42158227	0.991301167	ENSG00000185122	HSF1
1142.228141	0.073596971	0.213850798	0.344151025	0.730732711	0.999467288	ENSG00000158669	GPAT4
1150.609955	-0.351111672	0.364210554	-0.964034863	0.335028447	0.976928484	ENSG00000188157	AGRN
1142.400993	-0.068584624	0.228259716	-0.300467491	0.76382059	0.999467288	ENSG00000149100	EIF3M
1143.532819	-0.119803634	0.206597403	-0.57988935	0.561989238	0.99483168	ENSG00000164961	WASHC5
1140.023523	0.239642859	0.222281718	1.078104223	0.280987247	0.963267117	ENSG00000156471	PTDSS1
1140.807423	-0.228162043	0.230852214	-0.988346781	0.322982842	0.975337095	ENSG00000131626	PPFIA1
1142.301227	0.435375762	0.234509666	1.856536528	0.063377121	0.868202292	ENSG00000141279	NPEPPS
1135.61091	0.108655576	0.244461666	0.444468771	0.656703703	0.997568624	ENSG00000107959	PITRM1
1132.35325	-0.026259162	0.195450806	-0.134351771	0.893124417	0.999467288	ENSG00000134046	MBD2
1137.792935	0.312279477	0.292474264	1.067716088	0.285648599	0.964826902	ENSG00000155760	FZD7
1130.962379	-0.255156117	0.227324624	-1.122430614	0.261679394	0.962465655	ENSG00000105058	FAM32A
1114.002506	-0.48527382	0.33517708	-1.44781326	0.147669283	0.936396974	ENSG00000163431	LMOD1
1131.952436	0.056360968	0.222881139	0.252874553	0.800365155	0.999467288	ENSG00000140497	SCAMP2
1146.02418	0.114150666	0.229740621	0.496867578	0.619282436	0.99483168	ENSG00000047315	POLR2B
1129.006572	0.048492563	0.271424205	0.178659684	0.858204927	0.999467288	ENSG00000132842	AP3B1
1124.738972	-0.055376989	0.241144771	-0.22964209	0.818369898	0.999467288	ENSG00000165916	PSMC3
1122.770272	0.100968058	0.267904993	0.37688009	0.706262718	0.99900742	ENSG00000140464	PML
1127.489302	-0.48298386	0.20503205	-2.355650543	0.018490303	0.726053908	ENSG00000112658	SRF
1149.934775	-0.163948426	0.396499604	-0.413489507	0.679248009	0.998869317	ENSG00000187840	EIF4EBP1
1129.625297	0.147625291	0.256626059	0.575254486	0.565119198	0.99483168	ENSG00000113643	RARS

1129.954723	-0.081477687	0.303039954	-0.268867806	0.788031412	0.999467288	ENSG00000091140	DLD
1123.296596	-0.17463885	0.243609647	-0.716879864	0.473448227	0.99135833	ENSG00000137106	GRHPR
1139.584608	-0.742076341	0.307068432	-2.416648099	0.015664153	0.696728876	ENSG00000165655	ZNF503
1143.34492	0.053215307	0.301396436	0.176562495	0.859852055	0.999467288	ENSG00000133026	MYH10
1124.860753	-0.084558328	0.265829833	-0.318091944	0.750415195	0.999467288	ENSG00000165802	NSMF
1110.478081	0.089763038	0.305990719	0.29335216	0.769252988	0.999467288	ENSG00000182551	ADI1
1122.175323	-0.23950464	0.266996691	-0.897032238	0.369701715	0.981455968	ENSG00000109079	TNFAIP1
1114.857405	-0.376418624	0.258103019	-1.458404579	0.144729059	0.935644597	ENSG00000198258	UBL5
1138.262025	0.197974811	0.287869277	0.687724694	0.491626167	0.993513219	ENSG00000142892	PIGK
1149.804259	-0.293620172	0.333596003	-0.880166938	0.378768881	0.982609538	ENSG00000112773	TENT5A
1131.681021	0.097248256	0.203296406	0.478356985	0.632396144	0.994860874	ENSG00000010322	NISCH
1125.118895	-0.206827397	0.189892459	-1.08918173	0.276073754	0.962465655	ENSG00000185963	BICD2
1111.144314	-0.324881697	0.264880214	-1.226523083	0.220001894	0.956884001	ENSG00000174903	RAB1B
1123.800085	0.007289404	0.189437872	0.038479126	0.969305674	0.999527751	ENSG00000116030	SUMO1
1134.192761	0.080754416	0.233321263	0.346108261	0.729261355	0.999467288	ENSG00000165671	NSD1
1115.040918	-0.024309201	0.274078684	-0.088694241	0.929324909	0.999527751	ENSG00000167645	YIF1B
1116.023666	-0.245408079	0.245145945	-1.001069299	0.316793306	0.974768742	ENSG00000124333	VAMP7
1122.977578	-0.163828225	0.183803206	-0.891324087	0.37275533	0.982329093	ENSG00000015676	NUDCD3
1122.294025	0.0542963	0.193211876	0.281019475	0.778695463	0.999467288	ENSG00000158604	TMED4
1129.779038	-0.073960195	0.217650606	-0.339811572	0.733998433	0.999467288	ENSG00000013561	RNF14
1130.147727	0.136082141	0.888295875	0.153194611	0.878244807	0.999467288	ENSG00000114023	FAM162A
1132.422403	-0.013194147	0.247431283	-0.05332449	0.957473368	0.999527751	ENSG00000115464	USP34
1114.856975	-0.222009772	0.242463447	-0.915642232	0.359854573	0.979465649	ENSG00000168090	COPS6
1132.801501	-0.126288058	0.198669709	-0.635668408	0.524992572	0.99483168	ENSG00000136021	SCYL2
1136.740818	0.773623394	0.369885845	2.091519329	0.036481536	0.789688071	ENSG00000006652	IFRD1
1113.071699	0.095197373	0.290981475	0.327159564	0.743547203	0.999467288	ENSG00000128294	TPST2
1075.555091	0.601144105	0.79614147	0.755071967	0.450205802	0.99135833	ENSG00000145681	HAPLN1
1129.011872	-1.087124608	0.306552097	-3.546296433	0.000390686	0.173156217	ENSG00000182541	LIMK2
1121.337189	0.005637155	0.19477677	0.028941618	0.976911153	0.999527751	ENSG00000088930	XRN2
1112.508889	-0.011665575	0.26036445	-0.044804791	0.964262906	0.999527751	ENSG00000197496	SLC2A10
1112.393102	-0.114965295	0.231751643	-0.496071112	0.61984424	0.99483168	ENSG00000184304	PRKD1
1106.51444	-0.388235226	0.222708675	-1.743242489	0.081291267	0.897807954	ENSG00000149357	LAMTOR1
1155.627361	-0.368813036	1.114834328	-0.330823179	0.740778052	0.999467288	ENSG00000213859	KCTD11
1119.353	0.292012388	0.202191149	1.44423922	0.148671696	0.936396974	ENSG00000227500	SCAMP4
1123.404847	0.074624538	0.223570214	0.333785688	0.738541284	0.999467288	ENSG00000114062	UBE3A
1118.571853	0.042694755	0.214345409	0.199186701	0.842116702	0.999467288	ENSG00000242247	ARFGAP3
1110.342373	0.100105735	0.215656098	0.464191532	0.642510528	0.996798617	ENSG00000078668	VDAC3
1114.307578	-0.216569235	0.311637135	-0.694940398	0.487092654	0.993513219	ENSG00000107404	DVL1
1111.940811	-0.0612828	0.243568348	-0.251604121	0.801347073	0.999467288	ENSG00000102893	PHKB
1112.308051	-0.074656727	0.208609629	-0.357877666	0.720434865	0.99900742	ENSG00000161217	PCYT1A
1106.905764	-0.013487383	0.303621905	-0.04442164	0.964568312	0.999527751	ENSG00000130522	JUND
1110.586197	-0.0720705	0.203455758	-0.354231801	0.723165173	0.999456245	ENSG00000056586	RC3H2
1118.009484	0.456886231	0.217270951	2.102840852	0.035479684	0.785058983	ENSG00000023318	ERP44
1114.337462	-0.191702133	0.227067081	-0.844253301	0.398527875	0.988378925	ENSG00000185989	RASA3
1113.714736	-0.170307861	0.353215019	-0.482164835	0.629688855	0.99483168	ENSG00000103507	BCKDK
1129.752568	-0.078909581	0.297452104	-0.265284997	0.790789921	0.999467288	ENSG00000115758	ODC1
1127.166974	0.096541264	0.362339754	0.26643851	0.78990151	0.999467288	ENSG00000122176	FMOD
1112.709737	-0.1903718	0.242326803	-0.785599435	0.432102195	0.991301167	ENSG00000076067	RBMS2
1109.073769	-0.008966589	0.211640914	-0.042366991	0.966206142	0.999527751	ENSG00000047249	ATP6V1H
1097.570714	-0.248005261	0.266648641	-0.930082602	0.352328318	0.977691382	ENSG00000126267	COX6B1
1098.657278	-0.20526732	0.219711502	-0.93425842	0.350170607	0.977691382	ENSG00000163812	ZDHHC3
1106.900312	0.144226644	0.312810558	0.461067059	0.644750494	0.997368416	ENSG00000155508	CNOT8
1113.627643	0.292688977	0.222601812	1.314854425	0.188558832	0.949094286	ENSG00000078674	PCM1
1106.557609	-0.138741758	0.206837653	-0.670776118	0.502363164	0.993513219	ENSG00000108349	CASC3
1103.915168	0.167295109	0.202813151	0.824873085	0.409443648	0.990226751	ENSG00000134644	PUM1
1105.050508	0.156641094	0.188566321	0.830694967	0.40614597	0.990226751	ENSG00000136938	ANP32B
1105.92332	0.330948762	0.223285407	1.482178195	0.138292887	0.934144427	ENSG00000169905	TOR1AIP2
1123.106501	-0.156915904	0.310769405	-0.504927131	0.613610018	0.99483168	ENSG00000175727	MLXIP
1096.90559	0.041730515	0.266266096	0.156724854	0.875461699	0.999467288	ENSG00000180817	PPA1
1102.138644	0.033759689	0.184303242	0.183174691	0.854660952	0.999467288	ENSG00000205937	RNPS1
1113.612237	-0.923922888	0.205287769	-4.50062316	6.78E-06	0.019571574	ENSG00000157216	SSBP3
1124.51339	1.068000324	1.369743419	0.779708308	0.435562588	0.991301167	ENSG00000163083	INHBB
1100.96976	-0.147494296	0.220683576	-0.66835194	0.503908966	0.993513219	ENSG00000101752	MIB1
1088.971519	-0.297911058	0.242575184	-1.228118449	0.219402506	0.956884001	ENSG00000180304	OAZ2

1093.180788	-0.231063807	0.219111728	-1.054547874	0.291632154	0.97025964	ENSG00000102898	NUTF2
1101.625182	0.165620675	0.226062239	0.732633081	0.463782249	0.99135833	ENSG00000117899	MESD
1103.628374	-0.60917087	0.442167678	-1.377691994	0.168298404	0.943223078	ENSG00000130066	SAT1
1094.242927	0.045331223	0.199037699	0.227751944	0.819839088	0.999467288	ENSG00000128463	EMC4
1100.202255	0.110052093	0.252143328	0.436466408	0.662498366	0.997568624	ENSG00000132466	ANKRD17
1093.588328	0.120653839	0.280015696	0.430882416	0.66655387	0.997568624	ENSG00000147955	SIGMAR1
1095.504652	-0.295302816	0.269691468	-1.094965361	0.273531812	0.962465655	ENSG00000165424	ZCCHC24
1101.793175	0.234606186	0.200873022	1.167932774	0.242833883	0.962465655	ENSG00000197045	GMFB
1097.845281	-0.126405188	0.197009544	-0.641619615	0.521120194	0.993513219	ENSG00000174238	PITPNA
1100.64804	-0.154581586	0.246393967	-0.627375693	0.530412997	0.99483168	ENSG00000179833	SERTAD2
1103.192738	-0.299319622	0.215837513	-1.386782202	0.165508186	0.943223078	ENSG00000153560	UBP1
1096.633397	-0.11273646	0.190303939	-0.592402135	0.553581337	0.99483168	ENSG00000126457	PRMT1
1100.176366	-0.088679108	0.178144526	-0.497793054	0.618629912	0.99483168	ENSG00000105176	URI1
1091.484492	0.141980489	0.216355897	0.656235819	0.511672405	0.993513219	ENSG00000001461	NIPAL3
1105.399914	-0.074923712	0.213315322	-0.351234555	0.725412388	0.999467288	ENSG00000275052	PPP4R3B
1085.210906	0.013615339	0.350162713	0.038882891	0.968983757	0.999527751	ENSG00000197448	GSTK1
1095.905813	0.023089862	0.235541687	0.098028769	0.921909449	0.999527751	ENSG00000168216	LMBRD1
1100.641115	0.190983325	0.221444924	0.86244165	0.388444529	0.986799595	ENSG00000110713	NUP98
1096.95001	0.234174764	0.182056224	1.286277163	0.19834635	0.954310328	ENSG00000139842	CUL4A
1091.075608	-0.13874257	0.22207988	-0.624741739	0.532140574	0.99483168	ENSG00000083799	CYLD
1109.824068	-0.643552032	0.472919849	-1.360805712	0.173575096	0.944422693	ENSG00000172164	SNTB1
1098.05769	-0.01719553	0.324898243	-0.052925894	0.957790953	0.999527751	ENSG00000068438	FTSJ1
1098.856732	0.100670697	0.233614547	0.430926492	0.66652182	0.997568624	ENSG00000145740	SLC30A5
1087.959927	-0.374724733	0.241388204	-1.55237384	0.120572799	0.923922314	ENSG00000131653	TRAF7
1100.54902	-0.034958513	0.212967671	-0.164149391	0.869613541	0.999467288	ENSG00000139505	MTMR6
1102.052822	0.185864938	0.226192681	0.821710662	0.41124159	0.990226751	ENSG00000126773	PCNX4
1085.10469	-0.229375942	0.204263162	-1.122943263	0.261461592	0.962465655	ENSG00000034713	GABARAPL2
1095.538656	0.135129138	0.237251767	0.56956009	0.568976104	0.99483168	ENSG00000173744	AGFG1
1092.949579	-0.141329838	0.189488086	-0.745850788	0.455757556	0.99135833	ENSG00000130227	XPO7
1097.065534	-0.016743688	0.211631514	-0.07911718	0.936939419	0.999527751	ENSG00000144566	RAB5A
1090.15603	-0.162880255	0.241266354	-0.675105552	0.499608707	0.993513219	ENSG00000101199	ARFGAP1
1086.97666	0.347893693	0.197853383	1.758340886	0.078689525	0.897052166	ENSG00000122707	RECK
1093.131329	-0.005696687	0.207726544	-0.027423974	0.978121577	0.999527751	ENSG00000124181	PLCG1
1090.193818	0.249779288	0.231615217	1.078423479	0.280844814	0.963267117	ENSG00000139324	TMTC3
1100.486549	-0.102201104	0.224945102	-0.454337986	0.64958558	0.997381078	ENSG00000132912	DCTN4
1090.351719	-0.00405275	0.234510542	-0.017281739	0.986211854	0.999527751	ENSG00000116857	TMEM9
1098.529848	0.433789507	0.290931289	1.491037655	0.135951609	0.934144427	ENSG00000070961	ATP2B1
1118.929876	-0.518955669	0.394893162	-1.314167272	0.188789919	0.949094286	ENSG00000105643	ARRDC2
1094.131736	-0.024322208	0.221735834	-0.109690021	0.912655215	0.999527751	ENSG00000141027	NCOR1
1096.290872	0.095208908	0.226475686	0.420393506	0.674198012	0.998869317	ENSG00000023516	AKAP11
1089.846448	-0.30449001	0.245957138	-1.237979966	0.21572349	0.954310328	ENSG00000143079	CTTNBP2NL
1084.851934	-0.226358456	0.301844515	-0.749917406	0.453304451	0.99135833	ENSG00000124813	RUNX2
1091.68067	-0.230202292	0.222162931	-1.036186777	0.300115015	0.972186492	ENSG00000072803	FBXW11
1078.022272	-0.248887747	0.208585171	-1.193218798	0.232783694	0.959575179	ENSG00000102882	MAPK3
1081.321641	0.041369852	0.316294532	0.130795342	0.895937208	0.999467288	ENSG00000106772	PRUNE2
1082.198552	-0.189354303	0.209139305	-0.905397973	0.365254589	0.981269214	ENSG00000187446	CHP1
1098.400886	-0.300815221	0.353418412	-0.851158884	0.394681099	0.988378925	ENSG00000177169	ULK1
1082.907172	-0.022282226	0.238850563	-0.093289399	0.925673654	0.999527751	ENSG00000108784	NAGLU
1078.167865	-0.159744238	0.241441078	-0.661628249	0.508209499	0.993513219	ENSG00000146425	DYNLT1
1082.170447	0.217721732	0.270554402	0.804724413	0.420978728	0.991301167	ENSG00000102054	RBBP7
1088.996452	-0.005752554	0.270141243	-0.021294616	0.983010638	0.999527751	ENSG00000108510	MED13
1090.418566	0.011956111	0.24954315	0.047911997	0.961786378	0.999527751	ENSG00000170471	RALGAPB
1087.762435	-0.190626052	0.224895389	-0.847620985	0.396649083	0.988378925	ENSG00000029725	RABEP1
1076.917845	-0.478095133	0.2662532	-1.79564089	0.072551648	0.885710197	ENSG00000146122	DAAM2
1074.619027	-0.086144352	0.21772325	-0.395659869	0.692355965	0.998869317	ENSG00000132356	PRKAA1
1083.47982	0.154363313	0.247421753	0.623887396	0.532701538	0.99483168	ENSG00000170921	TANC2
1087.163526	0.108887239	0.23694359	0.459549208	0.645839824	0.997368416	ENSG00000198492	YTHDF2
1092.430824	-0.927907081	0.29013686	-3.198170273	0.001383026	0.264250513	ENSG00000136848	DAB2IP
1073.259067	0.402638203	0.234800423	1.71481038	0.086379998	0.901307093	ENSG00000206560	ANKRD28
1067.905018	-0.306475127	0.234045662	-1.30946724	0.190376128	0.949094286	ENSG00000053254	FOXN3
1088.523462	-0.166128746	0.259909803	-0.639178454	0.522706847	0.994616524	ENSG00000169641	LUZP1
1075.086605	0.126122046	0.25436004	0.495840644	0.620006846	0.99483168	ENSG00000120725	SIL1
1075.413219	0.221753871	0.190837276	1.162005008	0.245233431	0.962465655	ENSG00000111077	TNS2
1066.122297	-0.394132047	0.223574384	-1.762867641	0.077922816	0.897052166	ENSG00000165502	RPL36AL

1094.340678	0.011888029	0.299628889	0.039675843	0.968351561	0.999527751	ENSG00000166224	SGPL1
1079.330164	0.031132131	0.235672811	0.132098949	0.894906027	0.999467288	ENSG00000088888	MAVS
1073.366484	-0.315264885	0.228911266	-1.377236215	0.168439229	0.943223078	ENSG00000204673	AKT1S1
1067.878163	-0.072227374	0.202571111	-0.35655318	0.721426333	0.99900742	ENSG00000169895	SYAP1
1069.74181	0.144414164	0.278758704	0.518061541	0.604415332	0.99483168	ENSG00000089159	PXN
1055.278497	-0.000680563	0.295371599	-0.002304092	0.998161602	0.999527751	ENSG00000204580	DDR1
1069.110807	0.037342628	0.198385394	0.188232746	0.850694203	0.999467288	ENSG00000171302	CANT1
1075.779341	-0.054158488	0.217356223	-0.249169255	0.80322986	0.999467288	ENSG00000005700	IBTK
1079.974933	0.463987281	0.335877692	1.381417381	0.167150665	0.943223078	ENSG00000114120	SLC25A36
1060.034883	0.022530648	0.24654968	0.091383804	0.927187631	0.999527751	ENSG00000181061	HIGD1A
1066.758005	0.18426995	0.244360021	0.754092052	0.450793949	0.99135833	ENSG00000119314	PTBP3
1061.415225	-0.087597517	0.212997349	-0.411261069	0.680881113	0.998869317	ENSG00000176087	SLC35A4
1062.146418	-0.249703564	0.206700501	-1.208045277	0.227029847	0.958865115	ENSG00000136854	STXBP1
1068.717929	-0.272595271	0.198134652	-1.375808162	0.168881034	0.943792179	ENSG00000162521	RBBP4
1075.399195	-0.001367717	0.337649649	-0.0040507	0.996768018	0.999527751	ENSG00000107679	PLEKHA1
1076.045278	0.227254512	0.239297474	0.949673676	0.342278089	0.977691382	ENSG00000154305	MIA3
1061.844298	-0.039159658	0.229832365	-0.170383564	0.864708497	0.999467288	ENSG00000157538	VPS26C
1061.549555	0.099793815	0.227883752	0.437915446	0.661447579	0.997568624	ENSG00000166086	JAM3
1059.816831	-0.17288478	0.192960176	-0.895960937	0.370273626	0.981455968	ENSG00000100280	AP1B1
1057.773391	0.241458673	0.237330173	1.017395597	0.308965276	0.973427483	ENSG00000164023	SGMS2
1059.7536	-0.09680515	0.195339024	-0.495575065	0.620194249	0.99483168	ENSG00000013275	PSMC4
1061.857994	-0.421291501	0.214983521	-1.959645556	0.050037233	0.838384865	ENSG00000100412	ACO2
1059.349289	0.174572372	0.21381098	0.816479921	0.414225703	0.990226751	ENSG00000133961	NUMB
1050.413134	0.431084276	0.366336459	1.176744124	0.239297651	0.962465655	ENSG00000116729	WLS
1052.585471	-0.318496958	0.266200254	-1.196456249	0.231518562	0.959575179	ENSG00000173660	UQCRH
1054.359553	0.283379739	0.325857274	0.869643742	0.384495125	0.985655313	ENSG00000106080	FKBP14
1060.288114	0.167414111	0.189261971	0.884562861	0.376392445	0.982609538	ENSG00000198162	MAN1A2
1070.605444	-0.636102462	0.263842709	-2.410915441	0.015912537	0.696728876	ENSG00000153823	PID1
1066.694337	-0.012445804	0.257704143	-0.048294931	0.961481195	0.999527751	ENSG00000023287	RB1CC1
1060.559657	-0.030762888	0.189409093	-0.162415054	0.870979017	0.999467288	ENSG00000169139	UBE2V2
1054.215669	-0.208709486	0.216263044	-0.965072358	0.334508571	0.976026345	ENSG00000166847	DCTN5
1057.90002	0.117371027	0.374395246	0.313494972	0.75390464	0.999467288	ENSG00000165030	NFIL3
1057.43643	-0.138769944	0.184796278	-0.750934731	0.452691937	0.99135833	ENSG00000100220	RTCB
1060.174015	-0.855245092	0.301886456	-2.83300252	0.004611302	0.47075409	ENSG00000176170	SPHK1
1054.829734	0.384275483	0.262427355	1.464311839	0.143108764	0.935644597	ENSG00000109736	MFSD10
1057.032651	-0.109807165	0.256431829	-0.428211916	0.668496841	0.997568624	ENSG00000012232	EXTL3
1063.362311	-0.170724186	0.221469588	-0.770869659	0.440784193	0.99135833	ENSG00000066455	GOLGA5
1050.295188	0.597563064	0.327638541	1.823848508	0.068174978	0.87420149	ENSG00000123146	ADGRE5
1054.286686	0.164178603	0.287296491	0.571460525	0.567687512	0.99483168	ENSG00000198925	ATG9A
1054.671653	-0.210699664	0.205290007	-1.026351292	0.304726028	0.973427483	ENSG00000183576	SETD3
1054.667119	-0.150808788	0.259567751	-0.580999708	0.561240651	0.99483168	ENSG00000071564	TCF3
1061.651782	-0.102003164	0.240031319	-0.424957729	0.67086749	0.997568624	ENSG00000078747	ITCH
1047.684129	-0.124137946	0.252262037	-0.492099196	0.622649221	0.99483168	ENSG00000172053	QARS
1060.463473	0.029197678	0.292817499	0.099712888	0.920572268	0.999527751	ENSG00000130024	PHF10
1064.557994	-0.045900203	0.260875055	-0.175947074	0.860335523	0.999467288	ENSG00000092607	TBX15
1056.155828	-0.045751321	0.216583853	-0.211240684	0.832699466	0.999467288	ENSG00000166579	NDEL1
1034.570767	-0.300479547	0.27281777	-1.101392871	0.270725707	0.962465655	ENSG00000172725	CORO1B
1048.314278	-0.169772441	0.296611544	-0.572373006	0.5670693	0.99483168	ENSG00000118508	RAB32
1045.148466	0.066173508	0.28828443	0.229542427	0.818447348	0.999467288	ENSG00000164163	ABCE1
1044.929684	-0.50424109	0.257390186	-1.959053282	0.050106547	0.838573419	ENSG00000127920	GNG11
1068.481689	-0.776215127	1.080886487	-0.718128255	0.47267821	0.99135833	ENSG00000146477	SLC22A3
1044.854362	0.000558126	0.215348024	0.002591739	0.997932093	0.999527751	ENSG00000155463	OXA1L
1037.089346	0.220310915	0.251302008	0.876677893	0.380661618	0.983746147	ENSG00000107819	SFXN3
1045.383182	0.572225116	0.406126324	1.408983074	0.158840173	0.939464191	ENSG00000143127	ITGA10
1041.694807	0.300344789	0.307494657	0.976747994	0.328693928	0.976026345	ENSG00000026103	FAS
1052.402415	0.188575467	0.243727554	0.77371419	0.439099826	0.991301167	ENSG00000169446	MMGT1
1037.486846	-0.1509616	0.228272079	-0.661323104	0.508405128	0.993513219	ENSG00000115241	PPM1G
1053.753904	-0.269304151	0.226013926	-1.191537865	0.233442501	0.959684953	ENSG00000067955	CBFB
1040.347874	-0.341624458	0.238614756	-1.431698788	0.152230043	0.938052926	ENSG00000135390	ATP5MC2
1037.007258	-0.323734674	0.247328632	-1.308925177	0.190559697	0.949094286	ENSG00000163681	SLMAP
1044.986713	0.176490962	0.253539791	0.696107546	0.486361481	0.993286039	ENSG00000197386	HTT
1040.077897	0.11665741	0.178815488	0.652389854	0.514149708	0.993513219	ENSG00000093183	SEC22C
1049.622722	-0.058220937	0.241975306	-0.240606936	0.809859774	0.999467288	ENSG00000166503	HDGFL3
1051.63684	-0.012766816	0.377845186	-0.033788485	0.973045819	0.999527751	ENSG00000183258	DDX41

1024.997056	-0.436401891	0.303558277	-1.437621454	0.15054149	0.936396974	ENSG00000103363	ELOB
1045.548609	-0.064744719	0.222916243	-0.290444154	0.771476469	0.999467288	ENSG00000108039	XPNPEP1
1036.931255	-0.193886492	0.23566464	-0.822722031	0.410666084	0.990226751	ENSG00000004059	ARF5
1034.194944	0.527739476	0.246180689	2.14370785	0.032056303	0.775963115	ENSG00000101333	PLCB4
1039.827672	0.291412119	0.3048094	0.956047023	0.339048478	0.977691382	ENSG00000116406	EDEM3
1041.807803	0.096888359	0.24526065	0.395042414	0.692811586	0.998869317	ENSG00000138668	HNRNPD
1039.828116	0.017920447	0.236882978	0.075651054	0.939696718	0.999527751	ENSG00000111885	MAN1A1
1038.243961	0.121476276	0.238300701	0.509760466	0.610219285	0.99483168	ENSG00000171928	TVP23B
1038.228049	-0.080040036	0.294819992	-0.271487818	0.786015868	0.999467288	ENSG00000075391	RASAL2
1033.093478	0.255663131	0.312890099	0.817102017	0.41387013	0.990226751	ENSG00000172893	DHCR7
1035.550966	0.046172674	0.280410572	0.164660961	0.869210846	0.999467288	ENSG00000143924	EML4
1037.613594	-0.149556329	0.2104744	-0.710567787	0.477352111	0.99135833	ENSG00000119402	FBXW2
1028.962866	0.014798138	0.289304545	0.051150727	0.959205414	0.999527751	ENSG00000204520	MICA
1040.650429	-0.184599776	0.226331437	-0.815617035	0.414719203	0.990226751	ENSG00000205730	ITPRIPL2
1039.1913	0.554101501	0.276938313	2.000812004	0.045412653	0.813244956	ENSG00000102172	SMS
1034.64306	-0.124381553	0.232128638	-0.535830278	0.592075854	0.99483168	ENSG00000159692	CTBP1
1049.10163	0.568674486	0.361009676	1.575233362	0.115202589	0.923922314	ENSG00000020577	SAMD4A
1032.79522	-0.062641905	0.202064224	-0.310009876	0.756553446	0.999467288	ENSG00000108671	PSMD11
1031.050568	0.176659286	0.307025558	0.575389511	0.565027896	0.99483168	ENSG00000136378	ADAMTS7
1034.725456	-0.155533156	0.284897922	-0.545925907	0.585116869	0.99483168	ENSG00000158864	NDUFS2
1033.61461	-0.151604845	0.26802724	-0.56563223	0.571643814	0.99483168	ENSG00000114686	MRPL3
1035.121859	-0.079779502	0.201193823	-0.396530574	0.691713658	0.998869317	ENSG00000110768	GTF2H1
1036.833171	-0.035826702	0.278509428	-0.128637304	0.897644647	0.999467288	ENSG00000116157	GPX7
1034.11759	-0.093245605	0.246995594	-0.377519305	0.705787721	0.99900742	ENSG00000214078	CPNE1
1040.405533	-0.358221376	0.243819256	-1.469208715	0.141776187	0.935644597	ENSG00000196704	AMZ2
1044.95455	0.138466311	0.302025034	0.458459713	0.6466222	0.997368416	ENSG00000185070	FLRT2
1030.16292	-0.11403347	0.201884971	-0.564843778	0.572180027	0.99483168	ENSG00000090581	GNPTG
1029.452563	0.081564111	0.207700177	0.392701209	0.694540178	0.998869317	ENSG00000090316	MAEA
1027.224254	0.106582571	0.222279427	0.479498139	0.631584292	0.99483168	ENSG00000085832	EPS15
1031.365833	-0.033908737	0.208553661	-0.162589987	0.870841271	0.999467288	ENSG00000115233	PSMD14
1033.882774	-0.084247176	0.215146768	-0.391580021	0.695368552	0.998869317	ENSG00000115365	LANCL1
1029.627541	-0.195001345	0.202180349	-0.964492079	0.334799278	0.976475357	ENSG00000086065	CHMP5
1037.205672	-0.271717207	0.231318457	-1.174645595	0.240136534	0.962465655	ENSG00000065833	ME1
1031.265845	-0.124993886	0.196695454	-0.635469118	0.525122502	0.99483168	ENSG00000110048	OSBP
1033.665519	-0.188504326	0.232406792	-0.811096459	0.417310279	0.990226751	ENSG00000003056	M6PR
1024.161876	-0.254025168	0.286401598	-0.886954435	0.375103432	0.982609538	ENSG00000111670	GNPTAB
1029.474298	-0.111182239	0.200921787	-0.553360789	0.580016383	0.99483168	ENSG00000113845	TIMMDC1
1014.616762	0.485095343	0.276992704	1.751292854	0.079895478	0.897302013	ENSG00000184347	SLIT3
1028.47904	0.836237205	0.324711485	2.575323767	0.010014629	0.630908184	ENSG00000185567	AHNAK2
1027.724459	-0.079182558	0.222606885	-0.355705789	0.722060909	0.99906455	ENSG00000119048	UBE2B
1028.703791	0.131780086	0.190505018	0.691740758	0.489100146	0.993513219	ENSG00000198791	CNOT7
1021.17724	-0.103041637	0.284340469	-0.362388222	0.717061939	0.99900742	ENSG00000189067	LITAF
1038.034457	0.233085685	0.238048704	0.979151245	0.327505254	0.976026345	ENSG00000197601	FAR1
991.7872429	0.361412699	0.461185902	0.783659468	0.433239951	0.991301167	ENSG00000154721	JAM2
1031.977936	-0.255568732	0.219486066	-1.164396158	0.244263504	0.962465655	ENSG00000167491	GATAD2A
1036.13102	-0.388814887	0.338094815	-1.150017303	0.250136745	0.962465655	ENSG00000186174	BCL9L
1027.762697	-0.17209477	0.211003829	-0.815600224	0.414728821	0.990226751	ENSG00000131374	TBC1D5
1025.913307	-0.118140225	0.226379605	-0.521867795	0.601762383	0.99483168	ENSG00000039523	RIPOR1
1026.942589	-0.02692827	0.286983623	-0.093832078	0.92524255	0.999527751	ENSG00000100106	TRIOBP
1053.079477	-0.059088833	0.386871315	-0.152735111	0.878607171	0.999467288	ENSG00000070371	CLTCL1
1023.508633	-0.134934613	0.222258217	-0.60710742	0.543779626	0.99483168	ENSG00000126461	SCAF1
1020.460864	0.071685595	0.326711593	0.219415524	0.826326379	0.999467288	ENSG00000123505	AMD1
1013.114066	0.804902183	0.284684486	2.827348248	0.004693526	0.47075409	ENSG00000003436	TFPI
1027.543323	0.10368449	0.289130013	0.358608532	0.719887963	0.99900742	ENSG00000143702	CEP170
1033.867753	0.081748417	0.299785072	0.272690085	0.785091457	0.999467288	ENSG00000100647	SUSD6
1020.110803	-0.17605916	0.206895673	-0.85095622	0.394793673	0.988378925	ENSG00000188986	NELFB
1020.415986	-0.060809402	0.215315334	-0.282420211	0.77762132	0.999467288	ENSG00000140350	ANP32A
1026.008561	-0.090659988	0.222792805	-0.406925114	0.68406299	0.998869317	ENSG00000102910	LONP2
1027.135625	0.147989506	0.239415227	0.618129047	0.536490277	0.99483168	ENSG00000213281	NRAS
1021.877823	0.265314158	0.213045183	1.245342208	0.213005987	0.954310328	ENSG00000136451	VEZF1
1014.872118	-0.006833889	0.321948262	-0.021226667	0.983064842	0.999527751	ENSG00000162144	CYB561A3
1009.823711	-0.407824516	0.255324907	-1.597276668	0.110204049	0.923922314	ENSG00000130811	EIF3G
1017.241385	-0.076184336	0.262874099	-0.289813019	0.771959287	0.999467288	ENSG00000124006	OBSL1
1010.007203	-0.273831929	0.254412319	-1.076331249	0.281779138	0.963267117	ENSG00000143420	ENSA

1020.386081	-0.520640043	0.226326617	-2.300392461	0.021425996	0.73997555	ENSG00000160877	NACC1
1017.558537	-0.329722244	0.287733409	-1.145929649	0.251824262	0.962465655	ENSG00000154277	UCHL1
1019.526032	0.234113248	0.214220795	1.092859576	0.274455459	0.962465655	ENSG00000137936	BCAR3
1014.335201	-0.296044015	0.19703093	-1.502525588	0.132961425	0.933741583	ENSG00000089351	GRAMD1A
1016.688317	0.016289635	0.239414457	0.06803948	0.945754206	0.999527751	ENSG00000137497	NUMA1
1013.739555	-0.307254578	0.276654195	-1.11060878	0.266736782	0.962465655	ENSG00000133275	CSNK1G2
1016.176145	-0.116166622	0.192061242	-0.604841564	0.545284274	0.99483168	ENSG00000099995	SF3A1
1008.197429	-0.277559818	0.249967571	-1.110383304	0.26683389	0.962465655	ENSG00000108819	PPP1R9B
1018.716352	0.162082527	0.815354842	0.198787716	0.842428806	0.999467288	ENSG00000275993	SIK1B
1011.372897	0.224881735	0.203923739	1.10277369	0.270125456	0.962465655	ENSG00000188529	SRSF10
1004.23802	-0.129590281	0.287596611	-0.450597385	0.652279752	0.997449432	ENSG00000140374	ETFA
1011.515447	0.199125611	0.250273153	0.795633124	0.426245308	0.991301167	ENSG00000178033	CALHM5
1014.363993	-0.021454286	0.191202923	-0.112206893	0.910659364	0.999527751	ENSG00000116906	GNPAT
1012.306483	-0.120013023	0.186323193	-0.644112098	0.519502746	0.993513219	ENSG00000111667	USP5
1014.233523	0.158935298	0.196792797	0.80762762	0.419304982	0.990226751	ENSG00000108883	EFTUD2
1016.110496	0.086145546	0.23435202	0.367590371	0.713178696	0.99900742	ENSG00000198836	OPA1
1014.6085	-0.093225941	0.212854016	-0.437980655	0.661400307	0.997568624	ENSG00000174574	AKIRIN1
1008.282554	-0.629513905	0.289308354	-2.175927159	0.029560708	0.773201806	ENSG00000175183	CSRP2
1016.642143	-0.23401054	0.203459802	-1.150156139	0.250079568	0.962465655	ENSG00000136485	DCAF7
1003.828631	-0.149873267	0.225374551	-0.664996411	0.506052788	0.993513219	ENSG00000240849	TMEM189
1019.846137	-0.028178626	0.298167897	-0.094505903	0.924707293	0.999527751	ENSG00000163629	PTPN13
1011.098418	0.478798756	0.222638398	2.150566838	0.031510405	0.775963115	ENSG00000092969	TGFB2
1013.833041	-0.054027487	0.196433655	-0.275041907	0.783284042	0.999467288	ENSG00000215717	TMEM167B
998.2068776	-0.345180071	0.274694835	-1.256594689	0.208900439	0.954310328	ENSG00000164405	UQCRQ
1017.80121	-0.091958704	0.206901656	-0.444456103	0.656712859	0.997568624	ENSG00000163785	RYK
1010.630597	-0.443594006	0.227160958	-1.952773966	0.050846385	0.842188482	ENSG00000130881	LRP3
1015.260354	-0.917774706	0.343815479	-2.669381578	0.007599106	0.563130099	ENSG00000122644	ARL4A
1018.374448	0.166954766	0.250391684	0.666774405	0.504916244	0.993513219	ENSG00000168137	SETD5
1006.307368	0.314589299	0.27882726	1.128258761	0.259210654	0.962465655	ENSG00000163378	EOGT
1000.55442	-0.340606287	0.237184611	-1.436038728	0.150991321	0.936396974	ENSG00000163444	TMEM183A
1005.542297	-0.366832557	0.290370378	-1.263326377	0.206471911	0.954310328	ENSG00000142784	WDTC1
1005.65493	0.409063924	0.29604613	1.381757376	0.167046211	0.943223078	ENSG00000139370	SLC15A4
1011.72553	-0.140802033	0.236138411	-0.596269081	0.550995483	0.99483168	ENSG00000138078	PREPL
1008.821965	0.006103859	0.217573996	0.028054173	0.977618944	0.999527751	ENSG00000204394	VARS
1005.840461	-0.274088656	0.266336593	-1.029106265	0.303429742	0.972793733	ENSG00000160445	ZER1
1001.56154	0.034808796	0.297447739	0.117024913	0.906840311	0.999527751	ENSG00000177666	PNPLA2
1003.575874	-0.185437555	0.272920138	-0.679457209	0.496848212	0.993513219	ENSG00000071655	MBD3
1003.522858	-0.027516098	0.187524945	-0.146733003	0.883342768	0.999467288	ENSG00000166233	ARIH1
1004.281427	-0.519098654	0.239996369	-2.162943781	0.030545504	0.775963115	ENSG00000104979	C19orf53
995.6268223	-0.36414144	0.22412647	-1.624714116	0.104223488	0.92169898	ENSG00000141552	ANAPC11
1011.577946	0.144883881	0.244016372	0.593746558	0.552681638	0.99483168	ENSG00000124201	ZNFX1
991.5687446	-0.191806309	0.251555046	-0.762482455	0.44577211	0.99135833	ENSG00000170043	TRAPPC1
1006.573635	-0.152605881	0.213822471	-0.713703664	0.47541044	0.99135833	ENSG00000169251	NMD3
1001.803139	-0.256631511	0.220158492	-1.165667099	0.243749067	0.962465655	ENSG00000196396	PTPN1
1014.407049	0.228722596	0.292042761	0.783181873	0.433520318	0.991301167	ENSG00000134575	ACP2
1011.686528	0.244704803	0.209705018	1.166900082	0.243250723	0.962465655	ENSG00000110330	BIRC2
1002.262424	-0.233195537	0.217017987	-1.07454474	0.282578604	0.963267117	ENSG00000160058	BSDC1
997.9614311	-0.372178702	0.269790532	-1.379509869	0.167737606	0.943223078	ENSG00000183741	CBX6
992.5874435	-0.259530998	0.223522347	-1.161096426	0.245602688	0.962465655	ENSG00000160211	G6PD
1003.197794	-0.334231748	0.234840944	-1.42322604	0.154670639	0.939432741	ENSG00000110047	EHD1
1009.632765	0.407500372	0.322813603	1.262339531	0.206826638	0.954310328	ENSG00000072310	SREBF1
1006.236103	0.193262485	0.237184826	0.814818082	0.415176449	0.990226751	ENSG00000109323	MANBA
984.5737037	-0.30111315	0.271170554	-1.110419792	0.266818173	0.962465655	ENSG00000128524	ATP6V1F
1004.443536	0.229462192	0.22931447	1.00064419	0.316998858	0.974768742	ENSG00000096746	HNRNPH3
990.954141	-0.192993211	0.250099805	-0.77166478	0.440312996	0.99135833	ENSG00000175334	BANF1
999.9989623	0.223477652	0.238598499	0.936626396	0.348950769	0.977691382	ENSG00000136478	TEX2
998.0693217	-0.070859875	0.196405358	-0.360783817	0.718261061	0.99900742	ENSG00000164209	SLC25A46
1004.368706	-0.294860992	0.272323016	-1.082761921	0.278914112	0.963231086	ENSG00000107263	RAPGEF1
1009.869409	0.111006627	0.254721993	0.435795218	0.662985314	0.997568624	ENSG00000100731	PCNX1
1006.756331	0.016728433	0.234470644	0.071345532	0.943122758	0.999527751	ENSG00000176853	FAM91A1
1006.031145	0.171720748	0.229230333	0.749118781	0.453785616	0.99135833	ENSG00000151553	FAM160B1
1001.962104	0.310210649	0.258978136	1.197825632	0.230984906	0.959575179	ENSG00000080822	CLDND1
997.327336	-0.422341914	0.286399145	-1.474661923	0.140303458	0.935644597	ENSG00000140743	CDR2
1010.143362	-0.459607477	0.250242261	-1.836650112	0.066261564	0.873987009	ENSG00000165006	UBAP1

994.4669348	-0.139365164	0.208868558	-0.667238598	0.50461974	0.993513219	ENSG00000170142	UBE2E1
999.145732	-0.100520115	0.241069463	-0.41697573	0.67669616	0.998869317	ENSG00000133606	MKRN1
999.9458652	0.100790511	0.374167471	0.269372725	0.787642873	0.999467288	ENSG00000167695	FAM57A
1000.967881	0.137353139	0.402023362	0.34165462	0.732610827	0.999467288	ENSG00000087074	PPP1R15A
988.6614039	-0.156855769	0.199954331	-0.784457972	0.432771434	0.991301167	ENSG00000140553	UNC45A
1000.272801	-0.119716088	0.24178747	-0.495129411	0.620508774	0.99483168	ENSG00000163349	HIPK1
1005.236423	-0.436676896	0.210459748	-2.074871326	0.037998462	0.790190505	ENSG00000180228	PRKRA
999.9413242	-0.629109363	0.282770318	-2.224806928	0.026094202	0.762934669	ENSG00000259330	INAFM2
1002.337946	0.085505148	0.253308567	0.337553323	0.735699822	0.999467288	ENSG00000113615	SEC24A
998.0735841	0.015978489	0.206850685	0.077246488	0.93842746	0.999527751	ENSG00000006831	ADIPOR2
1007.660538	-0.35169684	0.271513024	-1.295322169	0.195209115	0.953843167	ENSG00000166454	ATMIN
997.8101648	-0.179295205	0.298698182	-0.600255427	0.548336019	0.99483168	ENSG00000079432	CIC
1013.784605	-0.542398067	0.455605869	-1.19049842	0.23385055	0.959884875	ENSG00000112759	SLC29A1
994.7686425	0.424834783	0.275389005	1.542671552	0.122910488	0.926620728	ENSG00000001036	FUCA2
997.1753534	-0.197317253	0.202745973	-0.973224031	0.33044197	0.976026345	ENSG00000131558	EXOC4
991.7138651	-0.114755724	0.201243877	-0.570232128	0.568520268	0.99483168	ENSG00000107175	CREB3
984.6280779	0.414380376	0.270128243	1.534013516	0.125026328	0.927794614	ENSG00000119899	SLC17A5
990.1471543	-0.327289319	0.204911022	-1.597226528	0.110215222	0.923922314	ENSG00000167778	SPRYD3
991.4219962	-0.097036438	0.255737935	-0.379437012	0.70436337	0.99900742	ENSG00000134905	CARS2
983.4958011	0.187343988	0.345270214	0.542601073	0.5874045	0.99483168	ENSG00000107798	LIPA
990.5800473	0.145932582	0.267845804	0.544838036	0.585864916	0.99483168	ENSG00000142002	DPP9
993.0497323	0.495574461	0.266603252	1.858846275	0.063048931	0.868202292	ENSG00000033627	ATP6V0A1
993.816073	-0.20649478	0.298333708	-0.692160405	0.488836601	0.993513219	ENSG00000149256	TENM4
995.2795543	0.78699062	0.366251186	2.14877289	0.031652407	0.775963115	ENSG00000139278	GLIPR1
987.4620987	-0.1571736	0.218770918	-0.718439187	0.472486532	0.99135833	ENSG00000166848	TERF2IP
987.1165819	-0.293120518	0.215259002	-1.36171085	0.173289156	0.944422693	ENSG00000149923	PPP4C
989.4750809	-0.201543491	0.21749831	-0.926643941	0.354111433	0.977691382	ENSG00000099956	SMARCB1
997.6749779	-0.044266653	0.278380348	-0.159015008	0.87365705	0.999467288	ENSG00000115295	CLIP4
983.353237	-0.258112977	0.292165324	-0.883448364	0.37699407	0.982609538	ENSG00000115107	STEAP3
981.35707	-0.048246665	0.242816451	-0.198696032	0.842500528	0.999467288	ENSG00000022277	RTF2
992.5369948	-0.22203754	0.284506457	-0.780430583	0.435137472	0.991301167	ENSG00000103043	VAC14
992.911251	-0.299729276	0.240699035	-1.245245025	0.213041697	0.954310328	ENSG00000169047	IRS1
1001.396753	-0.086752057	0.24636888	-0.352122629	0.724746299	0.999456245	ENSG00000141012	GALNS
991.2916191	0.019117392	0.207458575	0.092150406	0.926578541	0.999527751	ENSG00000018510	AGPS
983.0939588	0.28314887	0.259689794	1.090334993	0.275565606	0.962465655	ENSG00000099797	TECR
990.0071573	-0.226515974	0.237486539	-0.953805528	0.34018209	0.977691382	ENSG00000102858	MGRN1
981.9183144	-0.095992539	0.240516007	-0.399110813	0.689811555	0.998869317	ENSG00000179262	RAD23A
998.6849451	-0.016341471	0.263221027	-0.062082697	0.950496976	0.999527751	ENSG00000063978	RNF4
987.6948917	-0.123565764	0.242371396	-0.509819914	0.610177633	0.99483168	ENSG00000187231	SESTD1
985.7782945	0.151640947	0.193077339	0.785389666	0.432225138	0.991301167	ENSG00000155097	ATP6V1C1
1006.946702	0.319940877	0.306005314	1.045540265	0.295773341	0.970706863	ENSG00000170385	SLC30A1
990.1927365	0.415513844	0.257041219	1.61652612	0.105980611	0.922095158	ENSG00000057252	SOAT1
984.724575	-0.218275083	0.205519502	-1.062065064	0.288206149	0.966288987	ENSG00000143393	PI4KB
982.4389584	0.523388147	0.251829836	2.078340498	0.037678009	0.790190505	ENSG00000187210	GCNT1
991.3015126	-0.146285713	0.212389842	-0.688760402	0.490974058	0.993513219	ENSG00000142207	URB1
984.7435049	0.110289951	0.22999828	0.479525109	0.63156511	0.99483168	ENSG00000178252	WDR6
984.2291365	0.175345093	0.210287639	0.833834521	0.404374239	0.98939135	ENSG00000148634	HERC4
989.7333295	-0.104842106	0.313746896	-0.334161414	0.738257759	0.999467288	ENSG00000076513	ANKRD13A
986.7418962	-0.30556214	0.215875402	-1.415456033	0.156934831	0.939464191	ENSG00000140718	FTO
988.6323896	-0.087179581	0.188327396	-0.462915023	0.643425279	0.997097166	ENSG00000166068	SPRED1
989.9044556	-2.311483454	1.329736907	-1.738301346	0.082157729	0.899345331	ENSG00000132854	KANK4
984.6996709	-0.102490526	0.191268939	-0.535845114	0.5920656	0.99483168	ENSG00000104969	SGTA
977.0335378	-0.011877207	0.351525829	-0.033787579	0.973046541	0.999527751	ENSG00000173548	SNX33
985.9450056	0.126045173	0.211851306	0.594970008	0.551863519	0.99483168	ENSG00000090520	DNAJB11
986.5192092	0.469086852	0.211572442	2.217145335	0.026613162	0.764376062	ENSG00000116754	SRSF11
982.702312	0.176585166	0.181927915	0.970632602	0.331731265	0.976026345	ENSG00000149187	CELF1
977.7402789	0.321170203	0.405233156	0.792556577	0.428036216	0.991301167	ENSG00000189184	PCDH18
981.632006	0.144848293	0.181528918	0.79793509	0.424908159	0.991301167	ENSG00000103197	TSC2
980.6371878	-0.025266916	0.1993532	-0.126744472	0.899142646	0.999527751	ENSG00000137177	KIF13A
986.2973341	0.399842134	0.224683787	1.779577155	0.075145188	0.893390506	ENSG00000109133	TMEM33
1006.859011	-0.076606393	0.392968045	-0.19494306	0.845437524	0.999467288	ENSG00000234741	GAS5
983.738912	-0.626598881	0.296424765	-2.113854697	0.03452768	0.783572981	ENSG00000112210	RAB23
977.0729423	-0.242347591	0.219595148	-1.103610864	0.269761976	0.962465655	ENSG00000111832	RWDD1
1013.435829	-0.54433633	0.449189393	-1.211819199	0.225581597	0.958865115	ENSG00000099864	PALM

985.3879442	-0.186525473	0.238100918	-0.783388301	0.433399124	0.991301167	ENSG00000011451	WIZ	
978.1598717	0.396168634	0.216910639	1.826414033	0.06778792	0.87420149	ENSG00000075790	BCAP29	
963.1231154	-0.174332029	0.32109415	-0.542931191	0.58717718	0.99483168	ENSG00000087077	TRIP6	
974.5509645	-0.124507886	0.214532404	-0.580368669	0.561666029	0.99483168	ENSG00000103018	CYB5B	
992.9291437	0.018412772	0.352868325	0.052180293	0.958385036	0.999527751	ENSG00000127663	KDM4B	
976.2192607	-0.600766247	0.185262356	-3.242786391	0.001183669	0.244243321	ENSG00000148120	C9orf3	
974.3874454	0.197797505	0.211827387	0.933767383	0.350423898	0.977691382	ENSG00000196712	NF1	
973.0403016	-0.412337412	0.239372059	-1.722579543	0.084964587	0.901307093	ENSG00000079999	KEAP1	
979.8066752	-0.323179308	0.268915966	-1.201785497	0.229446646	0.959575179	ENSG00000165175	MID1IP1	
976.5158142	-0.027735045	0.209629069	-0.132305339	0.894742784	0.999467288	ENSG00000124164	VAPB	
975.0347619	0.264791869	0.246039563	1.076216628	0.281830385	0.963267117	ENSG00000139624	CERS5	
976.1905676	0.227521422	0.19657735	1.157414228	0.247103164	0.962465655	ENSG00000100401	RANGAP1	
970.0472234	0.162894682	0.255454104	0.637667117	0.523690395	0.99483168	ENSG00000106299	WASL	
965.7786974	0.495431362	0.262162545	1.889786971	0.058786457	0.853319392	ENSG00000213853	EMP2	
979.1387247	0.400544141	0.216226	1.852432832	0.063963694	0.868202292	ENSG00000122299	ZC3H7A	
967.5758145	-0.141932207	0.232288531	-0.611016852	0.541188424	0.99483168	ENSG00000099968	BCL2L13	
970.4819524	-0.039674576	0.353857242	-0.112120289	0.910728031	0.999527751	ENSG00000006576	PHTF2	
975.9040399	-0.126593865	0.320012046	-0.395590936	0.692406825	0.998869317	ENSG00000138764	CCNG2	
965.8232719	0.059631329	0.260269741	0.229113568	0.818780648	0.999467288	ENSG00000133835	HSD17B4	
971.0807848	-0.342206475	0.22091758	-1.549023285	0.121376123	0.923922314	ENSG00000151502	VPS26B	
973.6261782	0.009209865	0.214665393	0.042903354	0.965778575	0.999527751	ENSG00000104131	EIF3J	
969.0672681	0.11844608	0.180151209	0.657481459	0.510871389	0.993513219	ENSG00000107779	BMPR1A	
960.7483768	-0.05761655	0.237589389	-0.242504726	0.808389095	0.999467288	ENSG00000167315	ACAA2	
979.6723443	-0.256900168	0.378465824	-0.678793571	0.497268668	0.993513219	ENSG00000128590	DNAJB9	
971.6328558	0.206435604	0.25403013	0.812642203	0.416423228	0.990226751	ENSG00000050130	JKAMP	
972.5353775	0.023532074	0.192815083	0.122044777	0.902863556	0.999527751	ENSG00000163605	PPP4R2	
967.2726162	-0.231141366	0.193738376	-1.193059271	0.232846161	0.959575179	ENSG00000114503	NCBP2	
964.2194335	-0.338202903	0.210329517	-1.607966911	0.107842426	0.923922314	ENSG00000121067	SPOP	
958.890856	-0.236817937	0.22364038	-1.058922976	0.28963486	0.967638364	ENSG00000169976	SF3B5	
968.4621345	0.268844092	0.264849087	1.015084083	0.310065749	0.973427483	ENSG00000111145	ELK3	
976.9486489	-0.265380478	0.28944328	-0.916865224	0.359213269	0.979465649	ENSG00000136048	DRAM1	
970.2007088	0.039739376	0.203884671	0.194911052	0.845462582	0.999467288	ENSG00000164323	CFAP97	
970.20378	0.123771043	0.23906595	0.517727609	0.604648333	0.99483168	ENSG00000181704	YIPF6	
965.5166174	0.012349428	0.203668794	0.060634857	0.951650013	0.999527751	ENSG00000132153	DHX30	
969.0790038	0.172186933	0.323646704	0.532021276	0.59471126	0.99483168	ENSG00000170153	RNF150	
960.1090571	0.099102535	0.193222471	0.512893426	0.608025872	0.99483168	ENSG00000072958	AP1M1	
960.1115575	-0.005714503	0.246289562	-0.023202374	0.981488845	0.999527751	ENSG00000130414	NDUFA10	
954.5032554	-0.070870324	0.224344192	-0.315899972	0.752078433	0.999467288	ENSG00000186815	TPCN1	
969.3202265	-0.402356747	0.220671525	-1.823328796	0.068253607	0.87420149	ENSG00000182606	TRAK1	
971.0564695	-0.297541274	0.303165325	-0.981448896	0.326371424	0.976026345	ENSG00000102572	STK24	
994.6988101	-1.38940319	0.871721403	-1.593861507	0.110967058	0.923922314	ENSG00000171246	NPTX1	
951.9423251	-0.327380769	0.235958534	-1.38745043	0.165304457	0.943223078	ENSG00000105254	TBCB	
967.4866247	0.062610871	0.255816284	0.244749357	0.806650509	0.999467288	ENSG00000151779	NBAS	
963.968343	0.562479028	0.220630748	2.549413596	0.010790425	0.638240244	ENSG00000147162	OGT	
964.6718065	0.365320828	0.269851923	1.353782562	0.175805759	0.946278819	ENSG00000154229	PRKCA	
963.5665549	0.148560197	0.241608011	0.61488109	0.538633252	0.99483168	ENSG00000142599	RERE	
961.6642604	0.146928934	0.274839367	0.534599304	0.592926965	0.99483168	ENSG00000054523	KIF1B	
955.2571866	-0.095143498	0.202301545	-0.470305344	0.63813688	0.996553586	ENSG00000169727	GPS1	
962.5341052	0.08963299	0.221448362	0.404757972	0.685655425	0.998869317	ENSG00000136536		7-Mar
960.0160455	0.220248421	0.213558423	1.031326314	0.302387822	0.972793733	ENSG00000197170	PSMD12	
949.8061033	0.020630883	0.239147311	0.086268513	0.931252968	0.999527751	ENSG00000106628	POLD2	
947.2001822	-0.125071759	0.258740748	-0.483386403	0.6288214	0.99483168	ENSG00000162627	SNX7	
949.4487925	0.809473294	0.414073036	1.954904625	0.050594329	0.842188482	ENSG00000162496	DHRS3	
951.5249223	0.024050436	0.202901145	0.118532775	0.905645528	0.999527751	ENSG00000174243	DDX23	
959.4980162	-0.436044336	0.248750398	-1.752939247	0.079612436	0.897052166	ENSG00000188636	RTL6	
953.358437	-0.113258862	0.257483061	-0.439869176	0.660031862	0.997568624	ENSG00000006282	SPATA20	
966.9937937	0.281885153	0.340330891	0.828267902	0.407518797	0.990226751	ENSG00000108854	SMURF2	
954.4290478	0.324111585	0.302194731	1.072525601	0.283484021	0.963267117	ENSG00000147274	RBMX	
959.4168045	0.132214927	0.229548554	0.575978042	0.564630023	0.99483168	ENSG00000099904	ZDHHC8	
956.9555019	0.208504709	0.252853293	0.824607448	0.409594492	0.990226751	ENSG00000085224	ATRX	
955.2185804	-0.049901423	0.242799272	-0.205525425	0.837161636	0.999467288	ENSG00000154174	TOMM70	
940.8049001	-0.596398071	0.347985838	-1.713857306	0.086554936	0.901307093	ENSG00000147224	PRPS1	
952.6202532	-0.018018876	0.226505685	-0.07955154	0.936593939	0.999527751	ENSG00000100883	SRP54	
941.6850416	-0.174989047	0.211199118	-0.828550082	0.407359044	0.990226751	ENSG00000134248	LAMTOR5	

954.3208896	0.529946212	0.22340083	2.372176563	0.017683638	0.726053908	ENSG00000110911	SLC11A2
952.7451036	-0.08767026	0.254940518	-0.343885156	0.730932655	0.999467288	ENSG00000091436	MAP3K20
952.6368581	0.276177641	0.207386682	1.331703839	0.182957527	0.949094286	ENSG00000109501	WFS1
959.4197888	-0.157201024	0.25067615	-0.627108019	0.530588431	0.99483168	ENSG00000139726	DENR
951.4591656	-0.189321151	0.214941508	-0.880803123	0.378424389	0.982609538	ENSG00000068308	OTUD5
935.5109634	-0.183857515	0.32031843	-0.573983566	0.565978922	0.99483168	ENSG00000013583	HEBP1
954.7600701	-0.337457727	0.330471362	-1.021140608	0.307187826	0.973427483	ENSG00000012822	CALCOCO1
954.0074697	0.203984641	0.202087942	1.009385513	0.312789783	0.973427483	ENSG00000161904	LEMD2
946.0683425	-0.268835054	0.21701159	-1.238805055	0.215417701	0.954310328	ENSG00000169021	UQCRFS1
952.4491392	-0.024908289	0.209876277	-0.118680821	0.905528232	0.999527751	ENSG00000108468	CBX1
953.4880856	-0.152262261	0.238677618	-0.637941095	0.523512025	0.99483168	ENSG00000168246	UBTD2
952.0751125	0.037046332	0.205811408	0.180001352	0.857151506	0.999467288	ENSG00000180957	PITPNB
948.9980295	-0.160073295	0.189641932	-0.844081759	0.39862372	0.988378925	ENSG00000144895	EIF2A
951.7568628	-0.46831215	0.258084023	-1.814572418	0.069589644	0.877912272	ENSG00000213442	RPL18AP3
936.7751612	0.026194746	0.368483286	0.071088016	0.943327706	0.999527751	ENSG00000137331	IER3
948.0686008	-0.185513368	0.213755328	-0.867877162	0.385461583	0.986047049	ENSG00000139990	DCAF5
945.5431332	0.01460593	0.213694433	0.068349606	0.945507336	0.999527751	ENSG00000168958	MFF
944.7927369	-0.021098773	0.209132738	-0.100886994	0.919640167	0.999527751	ENSG00000113141	IK
954.9536063	-0.112677498	0.36128746	-0.31187769	0.755133479	0.999467288	ENSG00000134717	BTF3L4
949.2967548	0.243305249	0.201775857	1.205819428	0.227887123	0.958865115	ENSG00000112701	SENP6
946.5572565	0.020748249	0.197898332	0.104842969	0.916500414	0.999527751	ENSG00000134371	CDC73
904.4880864	0.393228853	0.496442358	0.792093678	0.428306056	0.991301167	ENSG00000073756	PTGS2
949.0487801	0.631790121	0.215070644	2.93759348	0.003307704	0.409258657	ENSG00000152223	EPG5
948.6558785	0.307350642	0.216504112	1.41960649	0.155722275	0.939464191	ENSG00000151923	TIAL1
956.3689241	-0.403689849	0.456424101	-0.884462167	0.376446777	0.982609538	ENSG00000140044	JDP2
943.4074423	0.048942712	0.208504306	0.234732378	0.814416456	0.999467288	ENSG00000170035	UBE2E3
941.7537397	-0.379953072	0.221452037	-1.715735274	0.086210506	0.901307093	ENSG00000103275	UBE2I
963.3831343	-0.498691115	0.468967788	-1.063380317	0.287609515	0.965808935	ENSG00000123095	BHLHE41
946.2793747	0.045140945	0.38363727	0.117665691	0.906332552	0.999527751	ENSG00000129474	AJUBA
941.3995623	-0.205634618	0.274364881	-0.74949322	0.453559984	0.99135833	ENSG00000120896	SORBS3
940.9182623	-0.053423714	0.242668055	-0.22015141	0.825753237	0.999467288	ENSG00000175826	CTDNEP1
937.7825743	0.374437364	0.296963621	1.260886308	0.207349812	0.954310328	ENSG00000131504	DIAPH1
944.3256984	-0.099788197	0.21385942	-0.466606508	0.64078143	0.996553586	ENSG00000132388	UBE2G1
938.8733751	-0.22891258	0.285658091	-0.801351639	0.422928104	0.991301167	ENSG00000087191	PSMC5
936.5897257	-0.381929197	0.230991296	-1.653435449	0.098242273	0.921147998	ENSG00000111412	C12orf49
935.6254614	-0.328831424	0.24786317	-1.326665128	0.184619481	0.949094286	ENSG00000196642	RABL6
940.9075109	0.082030525	0.193548468	0.423824204	0.671694025	0.997568624	ENSG00000144118	RALB
941.0803143	-0.131081884	0.21116806	-0.620746736	0.534766274	0.99483168	ENSG00000105576	TNPO2
948.1101298	-0.181017986	0.238127727	-0.760171813	0.447151891	0.99135833	ENSG00000141867	BRD4
941.8827231	-0.296523539	0.298395925	-0.993725162	0.320356689	0.975316319	ENSG00000109762	SNX25
937.1574888	-0.197087931	0.230576478	-0.854761652	0.392683112	0.988378925	ENSG00000264364	DYNLL2
935.4494196	-0.190884472	0.249997052	-0.763546894	0.445137307	0.99135833	ENSG00000115073	ACTR1B
941.2342047	0.057870245	0.219376889	0.263793719	0.791938875	0.999467288	ENSG00000107651	SEC23IP
950.9118174	-0.333043459	0.256868721	-1.296551242	0.194785638	0.952688442	ENSG00000141627	DYM
939.8007182	-0.257079135	0.268014304	-0.959199308	0.337458349	0.977691382	ENSG00000051620	HEBP2
933.0778303	-0.298764039	0.216452125	-1.38027769	0.167501161	0.943223078	ENSG00000023902	PLEKHO1
934.9869165	0.461251876	0.290525963	1.587644253	0.112366805	0.923922314	ENSG00000182667	NTM
941.6476413	0.003173363	0.252026025	0.012591408	0.989953775	0.999527751	ENSG00000133818	RRAS2
934.4129064	-0.138985084	0.196699604	-0.706585483	0.479824113	0.99135833	ENSG00000141543	EIF4A3
929.8200383	-0.059794083	0.25498673	-0.234498805	0.814597761	0.999467288	ENSG00000011454	RABGAP1
942.7273962	0.129490794	0.208346201	0.621517423	0.534259235	0.99483168	ENSG00000107290	SETX
952.8742288	0.428377434	0.453318518	0.944981104	0.344668523	0.977691382	ENSG00000166123	GPT2
934.2738467	-0.102370596	0.187386845	-0.546306202	0.584855474	0.99483168	ENSG00000125841	NRSN2
943.8932732	-0.048670896	0.347583438	-0.140026512	0.888639043	0.999467288	ENSG00000146374	RSPO3
933.6135318	0.031403675	0.225547101	0.139233337	0.889265766	0.999467288	ENSG00000093144	ECHDC1
940.834599	-0.224726914	0.204965242	-1.096414744	0.272897314	0.962465655	ENSG00000121644	DESI2
938.6004485	-0.114864939	0.195481852	-0.587598989	0.55680149	0.99483168	ENSG00000120008	WDR11
936.8487997	0.131487524	0.197441846	0.665955706	0.505439413	0.993513219	ENSG00000075711	DLG1
935.5247082	0.299007582	0.221124453	1.352214007	0.176306864	0.946278819	ENSG00000047410	TPR
929.3328349	-0.622723468	0.202395913	-3.076759097	0.002092643	0.328522281	ENSG00000148411	NACC2
922.9805654	0.42288151	0.298525737	1.416566335	0.156609757	0.939464191	ENSG00000135678	CPM
920.3552937	-0.10901376	0.261774925	-0.416440803	0.677087477	0.998869317	ENSG00000149932	TMEM219
934.7096747	0.038989147	0.185528553	0.210151733	0.83354925	0.999467288	ENSG00000085491	SLC25A24
940.027407	0.083282925	0.251349633	0.331342936	0.740385463	0.999467288	ENSG00000174227	PIGG

921.081396	-0.145632324	0.267869454	-0.543669022	0.586669253	0.99483168	ENSG00000168291	PDHB
927.2630593	-0.466235038	0.301095716	-1.548461216	0.121511292	0.923922314	ENSG00000101940	WDR13
932.3800362	-0.114868396	0.18295329	-0.627856409	0.530098009	0.99483168	ENSG00000130640	TUBGCP2
925.6141412	-0.237091866	0.198983032	-1.191518005	0.233450293	0.959684953	ENSG00000164062	APEH
939.8471796	0.214186481	0.216938045	0.987316359	0.323487573	0.975412329	ENSG00000136044	APPL2
932.0662054	-0.345259154	0.193729014	-1.782175768	0.074720573	0.893390506	ENSG00000134452	FBH1
922.49946	-0.030770297	0.277050135	-0.111064003	0.911565594	0.999527751	ENSG00000128923	MINDY2
922.4114769	-0.53136866	0.26541437	-2.002034249	0.045281048	0.813244956	ENSG00000175416	CLTB
922.3522566	-0.241222391	0.221414488	-1.089460737	0.27595076	0.962465655	ENSG00000135930	EIF4E2
934.3918534	-0.053557443	0.218200564	-0.245450526	0.806107611	0.999467288	ENSG00000172845	SP3
928.0963069	0.132124143	0.303763252	0.434957627	0.663593185	0.997568624	ENSG00000137216	TMEM63B
921.9193902	0.017759595	0.238600603	0.074432314	0.940666397	0.999527751	ENSG00000085449	WDFY1
928.8481277	-0.336965805	0.231305282	-1.456801165	0.145171274	0.935644597	ENSG00000105063	PPP6R1
925.2427111	-0.343592938	0.268918855	-1.277682586	0.201361368	0.954310328	ENSG00000167792	NDUFV1
931.7297586	-0.028608255	0.223868483	-0.127790454	0.898314805	0.999490224	ENSG00000163428	LRRC58
934.0999772	0.08841522	0.304821253	0.290055958	0.771773429	0.999467288	ENSG00000106638	TBL2
929.9095112	0.01027208	0.198930957	0.05163641	0.958818407	0.999527751	ENSG00000069345	DNAJA2
923.7011091	0.298139428	0.232525751	1.282178109	0.199780174	0.954310328	ENSG00000169213	RAB3B
927.1415912	0.327373547	0.257987385	1.268951762	0.204458269	0.954310328	ENSG00000087502	ERGIC2
930.3527389	0.220208315	0.233500158	0.943075659	0.345642205	0.977691382	ENSG00000164930	FZD6
917.8852092	0.231257195	0.349069256	0.662496599	0.507653014	0.993513219	ENSG00000132646	PCNA
926.4875419	-0.236464517	0.1981533	-1.193341301	0.232735734	0.959575179	ENSG00000135966	TGFBRAP1
918.7826206	-0.173380015	0.222408172	-0.779557751	0.435651232	0.991301167	ENSG00000100138	SNU13
920.1838457	0.200897423	0.232514678	0.864020391	0.387576689	0.986799595	ENSG00000166619	BLCAP
924.0450117	-0.108845225	0.193086889	-0.563711112	0.572950754	0.99483168	ENSG00000101577	LPIN2
924.4910113	-0.590032997	0.268487667	-2.197616763	0.027976428	0.769167109	ENSG00000106683	LIMK1
935.3841129	-0.546889781	0.345115295	-1.584658199	0.113044014	0.923922314	ENSG00000179918	SEPHS2
923.0479118	0.078550685	0.197447344	0.397831054	0.690754725	0.998869317	ENSG00000135932	CAB39
921.638553	-0.347849559	0.263201763	-1.321608013	0.186298714	0.949094286	ENSG00000089472	HEPH
928.2473133	-0.034114428	0.212604978	-0.160459215	0.872519347	0.999467288	ENSG00000120690	ELF1
923.1714418	0.133246781	0.201993945	0.659657303	0.509473773	0.993513219	ENSG00000134759	ELP2
920.1362172	0.477870948	0.257911609	1.852847762	0.063904182	0.868202292	ENSG00000104756	KCTD9
916.8624488	-0.175222681	0.245362751	-0.714137252	0.475142313	0.99135833	ENSG00000152484	USP12
922.1490112	-0.285387415	0.2443765	-1.16781857	0.242879956	0.962465655	ENSG00000186635	ARAP1
922.7343555	0.354444262	0.203926801	1.738095535	0.082193981	0.899345331	ENSG00000128731	HERC2
934.299968	-0.221841724	0.322598131	-0.687672068	0.491659314	0.993513219	ENSG00000131149	GSE1
922.8822676	-0.086226449	0.210208684	-0.410194513	0.681663266	0.998869317	ENSG00000198900	TOP1
926.2536564	0.408775989	0.797976074	0.512265971	0.608464877	0.99483168	ENSG00000187957	DNER
943.0346881	0.007742383	0.452781208	0.017099612	0.986357149	0.999527751	ENSG00000172432	GTPBP2
914.9443614	-0.168625242	0.231745444	-0.727631317	0.466839304	0.99135833	ENSG00000141503	MINK1
925.060823	0.167358743	0.215243652	0.77753161	0.436845193	0.991301167	ENSG00000125827	TMX4
923.7779377	-0.281607032	0.301615398	-0.933662651	0.350477937	0.977691382	ENSG00000153814	JAZF1
936.1748135	-0.028584561	0.460440291	-0.062080929	0.950498384	0.999527751	ENSG00000081052	COL4A4
919.9249107	-0.112715912	0.199725158	-0.5643551	0.572512489	0.99483168	ENSG00000163636	PSMD6
921.8208718	0.145784988	0.251571341	0.579497599	0.562253466	0.99483168	ENSG00000131263	RLIM
917.5332517	-0.112979132	0.188800673	-0.598404288	0.549570205	0.99483168	ENSG00000074319	TSG101
914.9763328	-0.015862981	0.232121568	-0.06833911	0.945515692	0.999527751	ENSG00000141349	G6PC3
911.3723211	-0.152985574	0.245726292	-0.622585288	0.533557082	0.99483168	ENSG00000172500	FIBP
918.8465924	-0.063985018	0.248924745	-0.257045632	0.797143547	0.999467288	ENSG00000038274	MAT2B
917.3754289	-0.091306644	0.210289115	-0.434195767	0.664146286	0.997568624	ENSG00000100614	PPM1A
912.2662416	0.38193816	0.241026713	1.584629996	0.113050426	0.923922314	ENSG00000120948	TARDBP
921.7783587	0.260872106	0.31102893	0.838739039	0.401615766	0.988592117	ENSG00000163872	YEATS2
939.6825745	0.345593137	0.46666655	0.740556907	0.458962145	0.99135833	ENSG00000130766	SESN2
908.4219429	-0.317632661	0.221051678	-1.436915854	0.150741904	0.936396974	ENSG00000141759	TXNL4A
905.3699165	-0.370366068	0.245855538	-1.506437766	0.131954832	0.932626545	ENSG00000130724	CHMP2A
901.4846344	-0.100946618	0.30285682	-0.33331466	0.738896774	0.999467288	ENSG00000132589	FLOT2
919.5378062	0.455202645	0.491684783	0.925801776	0.354549004	0.977691382	ENSG00000183671	GPR1
902.0270191	-0.167467973	0.263151095	-0.63639474	0.524519168	0.99483168	ENSG00000142507	PSMB6
913.1808129	-0.155020823	0.208059396	-0.745079652	0.456223569	0.99135833	ENSG00000135341	MAP3K7
904.2277542	-0.30152225	0.247703505	-1.217270822	0.223501192	0.956884001	ENSG00000138867	GUCD1
900.5202294	0.241075878	0.28140617	0.856682984	0.39162011	0.988355932	ENSG00000103381	CPPED1
914.3373549	-2.423122543	1.103883004	-2.195090001	0.028157142	0.769167109	ENSG00000125968	ID1
909.9984977	0.062898352	0.194072292	0.324097537	0.745864191	0.999467288	ENSG00000143727	ACP1
903.1660761	-0.279143711	0.235063819	-1.187523084	0.235021357	0.961317886	ENSG00000100364	KIAA0930

915.0823915	0.047938765	0.19960278	0.240170831	0.810197825	0.999467288	ENSG00000270647	TAF15
909.2376625	0.116523492	0.24025137	0.485006567	0.627671687	0.99483168	ENSG00000108010	GLRX3
905.5391983	0.316879613	0.203012721	1.560885501	0.118550769	0.923922314	ENSG00000005486	RHBDD2
905.3968676	-0.152279568	0.233221109	-0.65294076	0.513794471	0.993513219	ENSG00000179091	CYC1
898.2448828	-0.22250876	0.268876316	-0.827550613	0.407925047	0.990226751	ENSG00000117448	AKR1A1
907.4546038	-0.11721537	0.243035051	-0.482298212	0.629594117	0.99483168	ENSG00000108091	CCDC6
910.61171	-0.136971591	0.212196423	-0.645494346	0.518606881	0.993513219	ENSG00000112339	HBS1L
909.8211844	-0.078987326	0.202873799	-0.389342175	0.697023042	0.998869317	ENSG00000103544	VPS35L
916.2187381	0.477175031	0.259635562	1.837864686	0.066082347	0.872419869	ENSG00000213064	SFT2D2
908.2565393	-0.326404953	0.215173358	-1.516939435	0.129281976	0.928627271	ENSG00000274523	RCC1L
918.8990248	0.391685844	0.283104899	1.383536088	0.16650055	0.943223078	ENSG00000100813	ACIN1
914.9992424	0.218390422	0.272507187	0.801411604	0.422893399	0.991301167	ENSG00000100393	EP300
903.6924055	0.080640905	0.234039518	0.344561064	0.730424382	0.999467288	ENSG00000078269	SYNJ2
952.784989	-0.425605091	0.369504694	-1.15182594	0.249392606	0.962465655	ENSG00000179954	SSC5D
900.3276127	-0.115247757	0.221470262	-0.520375765	0.602801698	0.99483168	ENSG00000160948	VPS28
905.9326971	0.17090006	0.289686469	0.589948369	0.555225265	0.99483168	ENSG00000111859	NEDD9
915.2710372	-0.222356507	0.21105092	-1.053568053	0.292080722	0.97066771	ENSG00000167635	ZNF146
898.7036486	0.209149779	0.296787726	0.704711685	0.480989678	0.991427704	ENSG00000198715	GLMP
900.673683	0.460239373	0.235758446	1.952164938	0.050918625	0.842188482	ENSG00000071626	DAZAP1
902.6389456	-0.286397109	0.244778376	-1.170026183	0.241990432	0.962465655	ENSG00000184887	BTBD6
901.8465923	0.146241086	0.23745948	0.615857012	0.537988895	0.99483168	ENSG00000142687	KIAA0319L
901.3184192	-0.084610028	0.212899764	-0.397417199	0.691059835	0.998869317	ENSG00000271601	LIX1L
900.95557	-0.3321083	0.261579485	-1.269626707	0.204217631	0.954310328	ENSG00000107521	HPS1
908.1069932	-0.0615483	0.337852923	-0.182174832	0.855445523	0.999467288	ENSG00000157985	AGAP1
909.9356841	0.353923426	0.225843944	1.56711497	0.117087821	0.923922314	ENSG00000052841	TTC17
915.8307597	-0.109466379	0.455929126	-0.240095166	0.810256481	0.999467288	ENSG00000130513	GDF15
901.7587271	-0.008254873	0.209997368	-0.039309411	0.968643704	0.999527751	ENSG00000092108	SCFD1
912.6600032	-0.331331052	0.391633733	-0.84602276	0.397540048	0.988378925	ENSG00000138942	RNF185
892.8563895	-0.191811235	0.262164742	-0.7316439	0.464385946	0.99135833	ENSG00000167863	ATP5PD
899.0595307	-0.118365609	0.246263405	-0.480646358	0.630767862	0.99483168	ENSG00000100632	ERH
904.7701256	-0.259789235	0.235653165	-1.102422005	0.270278249	0.962465655	ENSG00000124209	RAB22A
897.524407	-0.120685323	0.212485221	-0.567970431	0.570055046	0.99483168	ENSG00000162517	PEF1
899.7450105	-0.389202981	0.248791866	-1.564371806	0.117730278	0.923922314	ENSG00000168936	TMEM129
897.9656342	0.075621116	0.270071556	0.280004001	0.779474435	0.999467288	ENSG00000153395	LPCAT1
900.9354777	-0.137197044	0.193620898	-0.708585926	0.478581473	0.99135833	ENSG00000009844	VTA1
902.7313515	-0.372925958	0.257575963	-1.447829037	0.14766487	0.936396974	ENSG00000236552	RPL13AP5
891.1776932	0.00402426	0.302850226	0.013287953	0.98939806	0.999527751	ENSG00000173915	ATP5MD
901.0786394	-0.254480261	0.304953875	-0.834487713	0.404006208	0.988927059	ENSG00000175137	SH3BP5L
911.8248276	-0.703751264	0.334326901	-2.104979475	0.035293095	0.784512596	ENSG00000238227	TMEM250
899.8447744	-0.056149875	0.185795783	-0.30221286	0.762489801	0.999467288	ENSG00000150760	DOCK1
957.2090541	-0.252116952	0.511792885	-0.492615196	0.622284508	0.99483168	ENSG00000157766	ACAN
897.5642122	0.074351753	0.204731489	0.363167158	0.716480019	0.99900742	ENSG00000145782	ATG12
896.2427939	-0.346632656	0.252600364	-1.372257151	0.1699834	0.944422693	ENSG00000130725	UBE2M
895.6346671	0.519587421	0.340284458	1.526920812	0.126780704	0.928627271	ENSG00000161547	SRSF2
900.3827478	0.136931171	0.291143057	0.47032264	0.638124525	0.996553586	ENSG00000100968	NFATC4
885.2617144	-0.441263491	0.256201281	-1.722331321	0.085009517	0.901307093	ENSG00000243927	MRPS6
885.0561229	-0.322562929	0.285767363	-1.128760563	0.258998853	0.962465655	ENSG00000106400	ZNHIT1
892.5062487	-0.203164738	0.30087721	-0.675241367	0.499522429	0.993513219	ENSG00000136521	NDUFB5
874.9202778	0.203587203	0.451487893	0.450925054	0.652043566	0.997449432	ENSG00000109686	SH3D19
913.1646023	0.295510629	0.33974815	0.869793194	0.384413431	0.985655313	ENSG00000096433	ITPR3
884.2311031	1.040548509	0.981080086	1.060615258	0.288864786	0.966288987	ENSG00000117632	STMN1
900.4002988	0.296504707	0.235373695	1.259718963	0.207770762	0.954310328	ENSG00000144357	UBR3
897.0109609	-0.207855832	0.200598723	-1.036177245	0.300119461	0.972186492	ENSG00000142453	CARM1
896.5940809	0.058262277	0.217977658	0.267285545	0.789249315	0.999467288	ENSG00000106261	ZKSCAN1
910.9074391	-0.433233961	0.317759306	-1.363402906	0.172755566	0.944422693	ENSG00000153885	KCTD15
934.5432377	-1.246643826	0.553055278	-2.254103477	0.024189659	0.747235409	ENSG00000134198	TSPAN2
896.8937775	0.172684291	0.183591036	0.940592172	0.346913895	0.977691382	ENSG00000102699	PARP4
900.1852593	0.427566915	0.242675366	1.761888412	0.078088153	0.897052166	ENSG00000135317	SNX14
894.8259914	-0.085650796	0.247729729	-0.345742903	0.729535938	0.999467288	ENSG00000114999	TTL
921.4679505	-0.612903806	0.399990306	-1.532296649	0.125449248	0.928208753	ENSG00000130707	ASS1
896.6991646	0.061888482	0.21040446	0.294140545	0.76865051	0.999467288	ENSG00000070770	CSNK2A2
885.4980536	-0.491589987	0.236949563	-2.074660867	0.038017977	0.790190505	ENSG00000108528	SLC25A11
895.4306047	-0.161174268	0.246693307	-0.653338636	0.51353799	0.993513219	ENSG00000185359	HGS
894.2234362	-0.183793178	0.20481661	-0.897354849	0.369529597	0.981455968	ENSG00000090372	STRN4

894.3479343	-0.070662257	0.221495647	-0.31902323	0.749708898	0.999467288	ENSG00000084463	WBP11
890.8142922	-0.154965878	0.191643199	-0.808616632	0.418735695	0.990226751	ENSG00000184787	UBE2G2
891.4688421	0.043439686	0.240040434	0.180968203	0.856392534	0.999467288	ENSG00000064419	TNPO3
887.6513532	-0.161150693	0.229889899	-0.700990753	0.483308786	0.991892545	ENSG00000090273	NUDC
895.3896939	-0.084453673	0.219044696	-0.385554521	0.699826621	0.998869317	ENSG00000119541	VPS4B
885.7074832	-0.187715357	0.221078596	-0.849088788	0.395831888	0.988378925	ENSG00000083937	CHMP2B
895.4460985	0.135942347	0.259661621	0.523536544	0.600600927	0.99483168	ENSG00000100815	TRIP11
890.5140078	-0.103787172	0.28656646	-0.362174875	0.717221354	0.99900742	ENSG00000232573	RPL3P4
876.383097	-0.110826068	0.333482664	-0.332329322	0.739640601	0.999467288	ENSG00000180891	CUEDC1
901.846138	-0.212092455	0.254936099	-0.831943598	0.405440781	0.990045328	ENSG00000023909	GCLM
898.1808439	-0.056882492	0.318278914	-0.178719009	0.858158342	0.999467288	ENSG00000123094	RASSF8
879.1571222	-0.296690635	0.239386491	-1.239379189	0.215205105	0.954310328	ENSG00000198356	ASNA1
885.4493802	-0.222865956	0.220383496	-1.011264273	0.311889961	0.973427483	ENSG00000204619	PPP1R11
886.816385	0.147841297	0.223905482	0.660284401	0.509071338	0.993513219	ENSG00000164466	SFXN1
889.2261143	-0.083880961	0.277644598	-0.302116308	0.762563401	0.999467288	ENSG00000118263	KLF7
886.2209729	-0.28940113	0.299911005	-0.964956686	0.334566507	0.976026345	ENSG00000173281	PPP1R3B
899.5466696	0.007069997	0.292962275	0.02413279	0.980746688	0.999527751	ENSG00000127870	RNF6
863.0825982	0.324369133	0.327686959	0.989875014	0.322235214	0.975337095	ENSG00000108389	MTMR4
875.7590532	-0.348112158	0.208258709	-1.671537095	0.094615638	0.921147998	ENSG00000145050	MANF
886.059106	-0.357224338	0.261598903	-1.365542184	0.172082704	0.944422693	ENSG00000173020	GRK2
885.2734892	-0.459432137	0.398508656	-1.152878688	0.248960181	0.962465655	ENSG00000176720	BOK
878.7153773	-0.13826271	0.25886284	-0.534115711	0.593261481	0.99483168	ENSG00000139433	GLTP
889.6682891	0.090865828	0.218469421	0.41592012	0.677468456	0.998869317	ENSG00000090432	MUL1
879.733701	-0.180396828	0.215298597	-0.837891333	0.402091737	0.988592117	ENSG00000078804	TP53INP2
873.8526663	-0.084787809	0.252941428	-0.335207284	0.737468727	0.999467288	ENSG00000277791	PSMB3
886.1467099	-0.175575832	0.224046917	-0.783656543	0.433241668	0.991301167	ENSG00000107164	FUBP3
888.2932249	0.017785778	0.387204708	0.045933786	0.963363025	0.999527751	ENSG00000037637	FBXO42
877.5359215	-0.026876514	0.36514222	-0.073605604	0.941324212	0.999527751	ENSG00000146278	PNRC1
885.3298449	-0.061641849	0.208946046	-0.295013235	0.76798377	0.999467288	ENSG00000138814	PPP3CA
884.0551296	0.216809589	0.214340486	1.011519536	0.311767836	0.973427483	ENSG00000124198	ARFGEF2
887.3718515	0.003823386	0.259693265	0.014722699	0.98825341	0.999527751	ENSG00000161091	MFSD12
882.9521063	0.275405156	0.230790332	1.193313225	0.232746725	0.959575179	ENSG00000164944	VIRMA
875.5535794	0.047912006	0.270906729	0.17685794	0.859619977	0.999467288	ENSG00000265354	TIMM23
880.4182387	-0.234330159	0.290908268	-0.805512199	0.420524169	0.991289469	ENSG00000071051	NCK2
878.2414299	0.12314807	0.219574207	0.560849433	0.574900185	0.99483168	ENSG00000105939	ZC3HAV1
875.6634594	0.110326423	0.220771465	0.499731353	0.617264253	0.99483168	ENSG00000247556	OIP5-AS1
873.7114611	-0.314630655	0.349203383	-0.900995438	0.367590745	0.981269214	ENSG00000137267	TUBB2A
876.8185429	-0.240766225	0.196160742	-1.227392509	0.2196751	0.956884001	ENSG00000101161	PRPF6
881.1474067	0.212669226	0.246178003	0.863883955	0.387651642	0.986799595	ENSG00000185591	SP1
882.6931019	-0.456953489	0.36614021	-1.248028698	0.212020548	0.954310328	ENSG00000197063	MAFG
870.7193567	-0.189657326	0.207342162	-0.914706994	0.360345471	0.979465649	ENSG00000054116	TRAPPC3
877.9618801	0.002773102	0.220599557	0.012570751	0.989970256	0.999527751	ENSG00000011405	PIK3C2A
871.4750326	0.163619786	0.230486257	0.709889555	0.477772628	0.99135833	ENSG00000116350	SRSF4
892.9385872	0.041079053	0.466053165	0.088142418	0.929763482	0.999527751	ENSG00000099875	MKNK2
879.1801323	-0.015360829	0.226116925	-0.067933123	0.945838871	0.999527751	ENSG00000154001	PPP2R5E
878.8388424	0.113556463	0.232836668	0.487708676	0.625756205	0.99483168	ENSG00000143324	XPR1
864.5949154	-0.282146627	0.251249746	-1.122972787	0.261449052	0.962465655	ENSG00000106367	AP1S1
872.237172	-0.017185377	0.230038445	-0.074706542	0.940448202	0.999527751	ENSG00000011007	ELOA
876.1225022	0.019640009	0.225134965	0.0872366	0.930483448	0.999527751	ENSG00000198793	MTOR
869.5640405	-0.382039795	0.38802337	-0.984579343	0.324830741	0.976026345	ENSG00000196139	AKR1C3
890.4800941	0.098846715	0.377773479	0.261656046	0.79358663	0.999467288	ENSG00000075275	CELSR1
866.0476456	-0.038488457	0.245979829	-0.15646997	0.875662588	0.999467288	ENSG00000126088	UROD
864.1008265	-0.74363918	0.290982644	-2.555613525	0.010600078	0.638240244	ENSG00000225663	MCRIP1
863.810762	-0.15443548	0.51469914	-0.300050005	0.764139013	0.999467288	ENSG00000196562	SULF2
874.9264873	0.193097174	0.309737082	0.623422849	0.533006687	0.99483168	ENSG00000073792	IGF2BP2
867.6940382	-0.381269375	0.230629208	-1.65317038	0.098296193	0.921147998	ENSG00000147164	SNX12
885.0329831	0.071548954	0.290091057	0.246643088	0.805184455	0.999467288	ENSG00000176624	MEX3C
866.7038861	-0.119733968	0.229093306	-0.522642806	0.601222846	0.99483168	ENSG00000242372	EIF6
863.6114484	-0.374822813	0.297899285	-1.258219916	0.208312234	0.954310328	ENSG00000129968	ABHD17A
871.8815417	0.035672016	0.191002061	0.186762468	0.851846875	0.999467288	ENSG00000125755	SYMPK
874.0141211	-0.107287496	0.198935608	-0.539307654	0.589674589	0.99483168	ENSG00000173950	XXYLT1
882.8703266	0.17683204	0.319367201	0.553695056	0.579787559	0.99483168	ENSG00000100342	APOL1
870.1127408	-0.029356442	0.229011654	-0.128187544	0.898000557	0.999467288	ENSG00000108523	RNF167
869.8614419	-0.095763911	0.192317167	-0.4979478	0.618520835	0.99483168	ENSG00000138593	SECISBP2L

880.6441785	-0.657348072	0.295854255	-2.221864515	0.026292463	0.763890866	ENSG00000125826	RBCK1
872.2245366	0.103369273	0.263629432	0.392100654	0.694983844	0.998869317	ENSG00000153066	TXNDC11
874.9618089	0.340915408	0.445080425	0.765963607	0.443697957	0.99135833	ENSG00000145555	MYO10
866.067484	-0.290759985	0.223006356	-1.303819276	0.192295209	0.949261383	ENSG00000110931	CAMKK2
874.3032759	-0.002209428	0.23887925	-0.00924914	0.99262036	0.999527751	ENSG00000137414	FAM8A1
872.1353567	0.155799499	0.281694483	0.553079696	0.580208839	0.99483168	ENSG00000173852	DPY19L1
870.0724729	0.424949851	0.269351725	1.577676369	0.114639978	0.923922314	ENSG00000094916	CBX5
870.5037579	0.29803406	0.264872117	1.125199829	0.260504366	0.962465655	ENSG00000113916	BCL6
867.1945305	0.054406929	0.200875869	0.270848508	0.78650755	0.999467288	ENSG00000082805	ERC1
861.9235387	-0.276634102	0.238718178	-1.158831323	0.246524947	0.962465655	ENSG00000180964	TCEAL8
864.0903981	-0.012158199	0.337185986	-0.036057841	0.971236238	0.999527751	ENSG00000180694	TMEM64
872.444882	-0.913833067	0.283997102	-3.217754901	0.001291982	0.252163401	ENSG00000172986	GXYLT2
865.7825843	-0.409009105	0.296307003	-1.380355851	0.167477106	0.943223078	ENSG00000104774	MAN2B1
862.372526	0.908116525	0.290925902	3.12147017	0.001799505	0.302525475	ENSG00000139289	PHLDA1
863.1904498	-0.35491482	0.231718634	-1.531662833	0.125605659	0.928627271	ENSG00000108561	C1QBP
867.609561	-0.103504141	0.195048477	-0.530658544	0.595655419	0.99483168	ENSG00000091164	TXNL1
860.1222983	-0.422943825	0.197959355	-2.1365185	0.032637175	0.775963115	ENSG00000160991	ORAI2
848.9057087	0.182397442	0.355971901	0.512392807	0.608376124	0.99483168	ENSG00000081320	STK17B
851.8057997	0.111922179	0.329573664	0.339596855	0.734160147	0.999467288	ENSG00000129055	ANAPC13
859.5960398	-0.186035977	0.208205933	-0.893519096	0.37157925	0.981955904	ENSG00000140859	KIFC3
859.692055	0.113298096	0.23715828	0.477731985	0.632840976	0.994909189	ENSG00000138363	ATIC
846.8260945	-0.206121029	0.378941913	-0.543938324	0.586483916	0.99483168	ENSG00000138650	PCDH10
864.7809791	0.363323755	0.203848019	1.782326642	0.07469598	0.893390506	ENSG00000143771	CNIH4
862.3554131	-0.019583584	0.200525667	-0.097661232	0.922201301	0.999527751	ENSG00000104660	LEPROTL1
864.3156764	-0.172091325	0.225187988	-0.764211832	0.444741017	0.99135833	ENSG00000078061	ARAF
866.7242657	0.136082897	0.244413429	0.556773404	0.577682258	0.99483168	ENSG00000168172	HOOK3
853.3297543	-0.354513108	0.25923024	-1.367560775	0.171449602	0.944422693	ENSG00000130311	DDA1
856.1241267	-0.255549795	0.237209789	-1.077315553	0.281339316	0.963267117	ENSG00000143222	UFC1
861.2307877	-0.237953681	0.210754536	-1.129056035	0.258874196	0.962465655	ENSG00000122126	OCRL
861.5068512	-0.091040203	0.20904435	-0.435506641	0.66319472	0.997568624	ENSG00000198218	QRICH1
860.6729152	0.132477718	0.220168906	0.60170948	0.547367539	0.99483168	ENSG00000169992	NLGN2
862.9272669	-0.659959598	0.273741021	-2.410890391	0.01591363	0.696728876	ENSG00000084731	KIF3C
857.8555286	-0.110112475	0.205801569	-0.535041959	0.592620844	0.99483168	ENSG00000117408	IPO13
861.6596884	-0.020838386	0.391208704	-0.053266673	0.957519433	0.999527751	ENSG00000079150	FKBP7
862.7452289	0.019823449	0.231355068	0.085684091	0.931717549	0.999527751	ENSG00000114331	ACAP2
856.0094811	-0.123918335	0.238822988	-0.518871054	0.603850664	0.99483168	ENSG00000135250	SRPK2
858.7181033	-0.001021606	0.214528117	-0.004762108	0.996200402	0.999527751	ENSG00000133704	IPO8
861.0158862	0.10571505	0.206800502	0.511193394	0.609215644	0.99483168	ENSG00000278311	GGNBP2
858.7624826	-0.106320477	0.21341433	-0.498188086	0.618351479	0.99483168	ENSG00000004487	KDM1A
852.7571675	0.022665128	0.21153408	0.10714646	0.914672789	0.999527751	ENSG00000067066	SP100
855.1805531	-0.607737173	0.287015709	-2.117435227	0.034222927	0.782919999	ENSG00000168256	NKIRAS2
843.4398626	-0.145285999	0.280037454	-0.518809168	0.603893824	0.99483168	ENSG00000090266	NDUFB2
848.8864399	0.678991134	0.461022144	1.472795054	0.140806306	0.935644597	ENSG00000138356	AOX1
847.5738986	-0.441879815	0.279283715	-1.582189694	0.113606272	0.923922314	ENSG00000135940	COX5B
851.6878945	-0.216604083	0.34286653	-0.631744612	0.527553772	0.99483168	ENSG00000075142	SRI
850.2196871	-0.080974165	0.220565412	-0.367120866	0.713528864	0.99900742	ENSG00000154582	ELOC
857.0572517	-0.011905547	0.222109998	-0.053602029	0.95725224	0.999527751	ENSG00000141646	SMAD4
859.3334525	-0.076738484	0.22756591	-0.337214323	0.735955341	0.999467288	ENSG00000143575	HAX1
855.2802432	-0.098248463	0.195483965	-0.502590903	0.615251926	0.99483168	ENSG00000169105	CHST14
857.9366524	0.060211353	0.215517139	0.279380808	0.779952598	0.999467288	ENSG00000095261	PSMD5
858.7134805	-0.509591296	0.381515329	-1.335703331	0.18164626	0.949094286	ENSG00000210135	MT-TN
844.5989114	0.02572025	0.339600858	0.075736704	0.939628574	0.999527751	ENSG00000114698	PLSCR4
857.709339	0.100219	0.211201898	0.474517514	0.635130911	0.995506752	ENSG00000163946	FAM208A
848.6932445	-0.040192452	0.204743943	-0.19630594	0.844370712	0.999467288	ENSG00000101846	STS
853.3554236	0.166657403	0.377061906	0.441989498	0.658496805	0.997568624	ENSG00000196968	FUT11
853.5531968	-0.242656506	0.215724106	-1.124846504	0.260654084	0.962465655	ENSG00000166548	TK2
867.958273	-0.421985796	0.525371623	-0.803213911	0.421851106	0.991301167	ENSG00000148053	NTRK2
852.7182684	0.278383774	0.22022882	1.26406605	0.206206321	0.954310328	ENSG00000157837	SPPL3
857.9618084	0.176879078	0.242539836	0.72927846	0.465831345	0.99135833	ENSG00000005339	CREBBP
850.4436705	-0.022695439	0.189413791	-0.119819357	0.904626248	0.999527751	ENSG00000143437	ARNT
848.917498	0.12288528	0.287157144	0.427937394	0.668696701	0.997568624	ENSG00000276023	DUSP14
855.5302695	0.363373133	0.205414944	1.76897126	0.076898671	0.897052166	ENSG00000100650	SRSF5
851.387149	-0.357666978	0.219747258	-1.627628856	0.103603607	0.92169898	ENSG00000164896	FASTK
846.5777348	-0.043613642	0.213019896	-0.204739759	0.837775455	0.999467288	ENSG00000176393	RNPEP

845.1830168	-0.224438595	0.25286236	-0.88759195	0.374760285	0.982609538	ENSG00000114125	RNF7
857.5800551	0.191163148	0.29705762	0.643522116	0.519885369	0.993513219	ENSG00000166025	AMOTL1
854.2492352	-0.297271144	0.258633971	-1.149389396	0.25039545	0.962465655	ENSG00000197122	SRC
869.7103246	-0.43438329	0.443500059	-0.97944359	0.327360848	0.976026345	ENSG00000171992	SYNPO
856.8744763	-0.073208036	0.281062543	-0.260468845	0.794502145	0.999467288	ENSG00000011275	RNF216
844.7868618	-0.366821566	0.234092919	-1.566991291	0.117116727	0.923922314	ENSG00000188690	UROS
851.3344379	0.179956081	0.197372131	0.911760341	0.361894892	0.979465649	ENSG00000185722	ANKFY1
854.3088091	0.344029636	0.252851355	1.360600327	0.173640028	0.944422693	ENSG00000115760	BIRC6
849.9277817	-0.295968647	0.28211595	-1.049102848	0.294130785	0.970706863	ENSG00000178951	ZBTB7A
857.5269049	-0.164179612	0.310967868	-0.527963267	0.597524832	0.99483168	ENSG00000047644	WWC3
856.0719356	0.809053503	0.864506393	0.935856009	0.349347329	0.977691382	ENSG00000186918	ZNF395
851.9125576	0.131116172	0.205060636	0.639401958	0.522561475	0.994616524	ENSG00000146463	ZMYM4
852.9961864	0.032509401	0.196441862	0.16549121	0.868557368	0.999467288	ENSG00000140157	NIPA2
847.6718645	-0.141013894	0.212698847	-0.662974418	0.507346938	0.993513219	ENSG00000120992	LYPLA1
827.5904364	-0.599099197	0.379056009	-1.580503098	0.113991698	0.923922314	ENSG00000196507	TCEAL3
846.6055738	-0.149857103	0.191537443	-0.782390644	0.433985031	0.991301167	ENSG00000125651	GTF2F1
841.9153969	-0.078690898	0.277214268	-0.283863089	0.776515304	0.999467288	ENSG00000126945	HNRNPH2
842.8039754	-0.344155157	0.205200679	-1.677163829	0.09351045	0.921147998	ENSG00000172057	ORMDL3
848.6556504	0.329968934	0.259482567	1.271642014	0.203500342	0.954310328	ENSG00000153214	TMEM87B
849.6100509	-0.285198528	0.215173362	-1.325436032	0.185026573	0.949094286	ENSG00000278540	ACACA
838.6507014	-0.51663237	0.242711307	-2.128587976	0.033288364	0.775963115	ENSG00000114853	ZBTB47
847.6179371	0.33298892	0.297853555	1.117961878	0.263583275	0.962465655	ENSG00000113441	LNPEP
851.3692903	0.141104865	0.24834925	0.568171092	0.569918799	0.99483168	ENSG00000119682	AREL1
835.2956403	-0.047848885	0.30954166	-0.15457979	0.877152604	0.999467288	ENSG00000115685	PPP1R7
862.4534384	0.214019769	0.442018623	0.484187221	0.628253005	0.99483168	ENSG00000153879	CEBPG
835.8448247	0.767069641	0.374273812	2.049487879	0.040414432	0.795991355	ENSG00000171451	DSEL
842.7512275	-0.134950268	0.21232609	-0.635580241	0.525050051	0.99483168	ENSG00000218283	MORF4L1P1
841.5676928	-0.07669579	0.205376462	-0.373440019	0.708820979	0.99900742	ENSG00000164975	SNAPC3
844.9780229	0.39491147	0.411213961	0.960355211	0.336876472	0.977691382	ENSG00000189320	FAM180A
842.786948	0.193062312	0.206442839	0.935185321	0.349692802	0.977691382	ENSG00000100612	DHRS7
836.76004	-0.393480587	0.25583369	-1.538032725	0.124040614	0.927794614	ENSG00000088367	EPB41L1
845.4830312	-0.224518054	0.257295187	-0.872608837	0.382876322	0.985655313	ENSG00000181915	ADO
844.7225368	-0.302603657	0.232241422	-1.302970217	0.19258493	0.949261383	ENSG00000124788	ATXN1
840.1268691	-0.734491889	0.402493183	-1.824855476	0.068022842	0.87420149	ENSG00000227051	C14orf132
850.2681095	-0.122229261	0.256884224	-0.475814587	0.634206477	0.99544042	ENSG00000036257	CUL3
846.004994	0.119810124	0.242966487	0.493113784	0.621932192	0.99483168	ENSG00000113658	SMAD5
843.5357012	0.231173935	0.268148081	0.862112957	0.388625362	0.986799595	ENSG00000169490	TM2D2
834.1071923	-0.059636596	0.221558689	-0.269168394	0.787800101	0.999467288	ENSG00000113163	COL4A3BP
841.414908	0.187298894	0.203228752	0.92161612	0.356728847	0.979427314	ENSG00000110075	PPP6R3
836.3377227	-0.127568935	0.204061014	-0.625150944	0.531871995	0.99483168	ENSG00000143106	PSMA5
840.4962973	-0.115864971	0.197330192	-0.587162917	0.557094295	0.99483168	ENSG00000119042	SATB2
838.3048863	0.132112833	0.21620263	0.61106025	0.541159694	0.99483168	ENSG00000168283	BMI1
832.4662739	-0.156570883	0.438743102	-0.356862325	0.721194876	0.99900742	ENSG00000198108	CHSY3
844.5816833	-0.165608941	0.224010324	-0.739291554	0.459729977	0.99135833	ENSG00000166783	MARF1
830.3434119	0.267978388	0.383550398	0.698678425	0.48475302	0.991892545	ENSG00000104549	SQLE
839.6067437	-0.069442998	0.235374514	-0.295031936	0.767969484	0.999467288	ENSG00000141644	MBD1
825.0929458	-0.242385557	0.264028221	-0.918028976	0.358603696	0.979427314	ENSG00000105669	COPE
838.5142801	-0.356622193	0.278885861	-1.27873888	0.200989023	0.954310328	ENSG00000087152	ATXN7L3
832.9140186	-0.188681769	0.212817668	-0.886588836	0.375300307	0.982609538	ENSG00000065518	NDUFB4
866.8877245	1.071227003	0.47789562	2.241550158	0.02499046	0.754051048	ENSG00000151012	SLC7A11
835.9679003	-0.049708142	0.194329681	-0.255792845	0.798110799	0.999467288	ENSG00000126804	ZBTB1
839.5913233	-0.291607244	0.268810525	-1.084805901	0.278007631	0.962465655	ENSG00000196628	TCF4
835.9731195	0.123375143	0.217156342	0.568139719	0.5699401	0.99483168	ENSG00000125734	GPR108
835.2830193	0.261959223	0.190010754	1.378654723	0.168001236	0.943223078	ENSG00000159899	NPR2
847.0965537	-0.157778277	0.263057687	-0.599785845	0.548648968	0.99483168	ENSG00000165417	GTF2A1
838.4440407	-0.134796953	0.225058806	-0.598941033	0.549212207	0.99483168	ENSG00000143398	PIP5K1A
834.3506562	-0.23774277	0.235544474	-1.009332826	0.312815042	0.973427483	ENSG00000092203	TOX4
827.4190061	-0.094878088	0.255403054	-0.371483766	0.710277245	0.99900742	ENSG00000265491	RNF115
837.0179157	-0.115103408	0.292531753	-0.39347321	0.693970007	0.998869317	ENSG00000155313	USP25
832.1814795	0.372824865	0.304017435	1.226327249	0.220075551	0.956884001	ENSG00000177508	IRX3
838.3518728	-0.041973782	0.253841072	-0.165354572	0.868664907	0.999467288	ENSG00000197622	CDC42SE1
848.1631639	-0.133636226	0.28593235	-0.467370081	0.640235124	0.996553586	ENSG00000165650	PDZD8
828.4266744	-0.130559203	0.329845786	-0.39581892	0.692238618	0.998869317	ENSG00000145675	PIK3R1
828.7518895	-0.157061469	0.200856267	-0.781959518	0.434238365	0.991301167	ENSG00000167770	OTUB1

832.8392577	-0.200394535	0.229998451	-0.871286454	0.383597765	0.985655313	ENSG00000185619	PCGF3
825.3999573	0.261177044	0.282331604	0.925071938	0.354928489	0.977691382	ENSG00000163565	IFI16
818.6333094	-0.2525923	0.795453859	-0.31754488	0.750830191	0.999467288	ENSG00000121005	CRISPLD1
841.7922552	0.011472843	0.303657051	0.037782237	0.969861307	0.999527751	ENSG00000012174	MBTPS2
832.2612585	0.164356008	0.199971272	0.821898098	0.411134896	0.990226751	ENSG00000221914	PPP2R2A
837.4705306	-0.033442015	0.211607749	-0.15803776	0.874427044	0.999467288	ENSG00000088205	DDX18
829.9637746	0.062276598	0.237049451	0.262715637	0.792769764	0.999467288	ENSG00000015532	XYLT2
820.4558149	0.245661973	0.343677852	0.71480304	0.474730757	0.99135833	ENSG00000171552	BCL2L1
833.315849	-0.348542406	0.256431704	-1.359201693	0.174082685	0.945216623	ENSG00000112033	PPARD
836.0946303	-0.002996283	0.23806891	-0.012585779	0.989958266	0.999527751	ENSG00000108021	FAM208B
840.7829201	0.068761259	0.295328379	0.232829839	0.815893542	0.999467288	ENSG00000163848	ZNF148
833.9225122	-0.208023444	0.433518065	-0.47984954	0.631334384	0.99483168	ENSG00000221866	PLXNA4
829.9979483	-0.490467564	0.504961442	-0.971297061	0.331400372	0.976026345	ENSG00000135074	ADAM19
809.767226	-0.356767367	0.322496441	-1.10626761	0.268610707	0.962465655	ENSG00000246705	H2AFJ
817.6099104	0.322112699	0.347133719	0.927921089	0.353448504	0.977691382	ENSG00000134291	TMEM106C
817.0659801	-0.28663889	0.275891099	-1.038956646	0.2988249	0.972186492	ENSG00000095383	TBC1D2
826.9915652	0.068414672	0.220373786	0.310448321	0.756220053	0.999467288	ENSG00000100519	PSMC6
823.6285149	-0.395246213	0.20902297	-1.890922385	0.058634706	0.852830872	ENSG00000100591	AHSA1
826.4275625	-0.34119585	0.278613818	-1.224619268	0.220718705	0.956884001	ENSG00000100605	ITPK1
830.7069119	-0.492497887	0.249553704	-1.97351464	0.048436958	0.833264304	ENSG00000109089	CDR2L
832.3570846	0.154855544	0.24842919	0.623338765	0.533061929	0.99483168	ENSG00000082996	RNF13
829.0276212	0.192084809	0.208684201	0.920456881	0.357334056	0.979427314	ENSG00000101972	STAG2
823.7728974	0.110322275	0.249061635	0.442951699	0.657800672	0.997568624	ENSG00000170515	PA2G4
819.8645352	-0.258753294	0.318113853	-0.813398382	0.415989687	0.990226751	ENSG00000090565	RAB11FIP3
824.4752756	0.265486876	0.208582961	1.272811909	0.203084794	0.954310328	ENSG00000171456	ASXL1
827.3695348	-0.177085143	0.196838075	-0.899648822	0.368307167	0.981269214	ENSG00000138381	ASNSD1
825.1631432	0.735785313	0.28081649	2.620164195	0.008788744	0.604581883	ENSG00000146242	TPBG
825.3212455	-0.1630044	0.266924389	-0.610676307	0.541413894	0.99483168	ENSG00000071575	TRIB2
828.5668489	0.205815335	0.235365423	0.874450175	0.381873143	0.985369679	ENSG00000118873	RAB3GAP2
825.6098894	-0.048503171	0.272689382	-0.177869671	0.85882533	0.999467288	ENSG00000185803	SLC52A2
816.5100606	0.337498779	0.304937425	1.106780443	0.268388866	0.962465655	ENSG00000124151	NCOA3
833.7752821	0.064830848	0.240390292	0.269689961	0.787398785	0.999467288	ENSG00000136770	DNAJC1
830.2368049	0.169624608	0.240142684	0.706349264	0.479970964	0.99135833	ENSG00000164168	TMEM184C
823.8282119	0.078928109	0.23557998	0.335037424	0.737596856	0.999467288	ENSG00000146676	PURB
823.4616383	-0.599485939	0.285975628	-2.096283319	0.036057066	0.785478429	ENSG00000126351	THRA
821.2642833	-0.619729374	0.278559637	-2.224763719	0.026097104	0.762934669	ENSG00000159792	PSKH1
828.9636463	-0.039735766	0.270821832	-0.146722904	0.883350739	0.999467288	ENSG00000099814	CEP170B
818.7545935	-0.068406393	0.26008906	-0.263011419	0.792541778	0.999467288	ENSG00000178741	COX5A
825.2300477	0.112472611	0.241302635	0.466106023	0.641139612	0.996553586	ENSG00000158615	PPP1R15B
824.224355	-0.142563036	0.185981013	-0.766546188	0.44335138	0.99135833	ENSG00000159579	RSPRY1
819.2996672	0.298789527	0.211480158	1.412848985	0.157700132	0.939464191	ENSG00000196586	MYO6
832.5794157	-0.440875111	0.369194062	-1.194155476	0.232417156	0.959575179	ENSG00000100852	ARHGAP5
815.4074335	0.18079595	0.258036218	0.700661136	0.483514515	0.991892545	ENSG00000173221	GLRX
823.6891575	-0.197821018	0.223269898	-0.886017418	0.375608141	0.982609538	ENSG00000175029	CTBP2
824.2088462	0.280986331	0.209975032	1.338189249	0.180834753	0.949094286	ENSG00000164031	DNAJB14
827.8314274	0.101707265	0.24650301	0.412600498	0.679899336	0.998869317	ENSG00000179387	ELMOD2
820.5176087	-0.101829679	0.187138356	-0.544141143	0.58634435	0.99483168	ENSG00000146457	WTAP
812.4842215	-0.079208317	0.298241629	-0.265584377	0.790559319	0.999467288	ENSG00000167085	PHB
817.3281568	-0.045932606	0.209820078	-0.218914256	0.826716841	0.999467288	ENSG00000116954	RRAGC
824.0366836	-0.063756784	0.219103513	-0.290989327	0.771059482	0.999467288	ENSG00000173473	SMARCC1
825.8463265	0.590467311	0.319529959	1.847924725	0.064613233	0.868202292	ENSG00000101236	RNF24
820.0969103	0.020624133	0.21005128	0.098186181	0.921784455	0.999527751	ENSG00000117528	ABCD3
820.8580055	-0.183351001	0.197007653	-0.930679586	0.352019333	0.977691382	ENSG00000100227	POLDIP3
820.3326437	0.036152018	0.185049108	0.195364454	0.845107642	0.999467288	ENSG00000183207	RUVBL2
819.8186002	-0.520576608	0.339660367	-1.532638655	0.125364912	0.928208753	ENSG00000203875	SNHG5
821.7764967	-0.544228527	0.28749243	-1.893018632	0.058355392	0.852621724	ENSG00000213553	RPLP0P6
807.4981581	-0.17999971	0.28503611	-0.631497917	0.527715011	0.99483168	ENSG00000164919	COX6C
808.670672	0.384656636	0.31349909	1.226978476	0.219830681	0.956884001	ENSG00000103066	PLA2G15
805.4935067	-0.41475477	0.285855923	-1.45092243	0.146801461	0.936396974	ENSG00000006118	TMEM132A
815.6421034	0.278921341	0.193368037	1.442437668	0.149178944	0.936396974	ENSG00000175348	TMEM9B
812.6757586	-0.374684621	0.233732519	-1.603048746	0.108923892	0.923922314	ENSG00000125733	TRIP10
811.0910428	0.445608437	0.227995012	1.954465732	0.050646164	0.842188482	ENSG00000112893	MAN2A1
819.7798718	-0.132608292	0.252074976	-0.526066861	0.598841753	0.99483168	ENSG00000134852	CLOCK
805.297653	-0.346897721	0.311115945	-1.115011062	0.264845681	0.962465655	ENSG00000241553	ARPC4

812.5877083	-0.197507323	0.24467432	-0.807225387	0.419536642	0.990226751	ENSG00000108219	TSPAN14
814.1485594	0.028838631	0.21483148	0.134238387	0.893214072	0.999467288	ENSG00000013374	NUB1
814.0759197	-0.291833082	0.21699715	-1.344870571	0.178667035	0.9469453	ENSG00000142875	PRKACB
817.7535864	-0.049932909	0.22164883	-0.225279371	0.821761947	0.999467288	ENSG00000130939	UBE4B
822.4646918	-0.050951941	0.351449745	-0.144976462	0.884729456	0.999467288	ENSG00000131759	RARA
818.3386519	-0.165338296	0.224362596	-0.736924511	0.461168257	0.99135833	ENSG00000101166	PRELID3B
801.0776989	-0.172964045	0.415414643	-0.416364825	0.677143063	0.998869317	ENSG00000042493	CAPG
814.3529751	-0.330277884	0.216450827	-1.525879518	0.127039873	0.928627271	ENSG00000184203	PPP1R2
812.1133054	-0.043771092	0.196212999	-0.223079468	0.823473658	0.999467288	ENSG00000167005	NUDT21
811.5225819	-0.187136874	0.25428809	-0.73592465	0.461776555	0.99135833	ENSG00000143499	SMYD2
812.5952003	-0.041226928	0.229682313	-0.17949544	0.857548698	0.999467288	ENSG00000065548	ZC3H15
802.9681226	-0.660126445	0.312175751	-2.114598726	0.034464162	0.783572981	ENSG00000138835	RGS3
816.7643326	0.185293832	0.379251744	0.488577404	0.625140914	0.99483168	ENSG00000183963	SMTN
813.8545149	-0.082830544	0.223360628	-0.37083771	0.710758412	0.99900742	ENSG00000108406	DHX40
811.2151084	-0.035752486	0.200289876	-0.178503709	0.858327408	0.999467288	ENSG00000115966	ATF2
810.5836263	-0.162428358	0.22798445	-0.712453673	0.476183889	0.99135833	ENSG00000035687	ADSS
810.8714229	0.031025744	0.212659958	0.14589368	0.884005321	0.999467288	ENSG00000138430	OLA1
815.228489	0.067342342	0.273404773	0.246310045	0.805442235	0.999467288	ENSG00000155975	VPS37A
799.8792925	-0.20344608	0.368701559	-0.551790668	0.581091782	0.99483168	ENSG00000120913	PDLIM2
802.3663108	0.156509831	0.248430099	0.629995448	0.528697562	0.99483168	ENSG00000143149	ALDH9A1
802.3493379	-0.166594691	0.270204597	-0.616550173	0.537531469	0.99483168	ENSG00000111269	CREBL2
809.6159226	0.239663183	0.214870826	1.115382611	0.264686498	0.962465655	ENSG00000072501	SMC1A
807.8961335	0.126038455	0.219259004	0.574838218	0.565400717	0.99483168	ENSG00000107937	GTPBP4
810.7989227	0.040050714	0.227040866	0.176403107	0.859977264	0.999467288	ENSG00000049618	ARID1B
802.1226104	-0.529099549	0.218060531	-2.426388426	0.015249939	0.694606015	ENSG00000106803	SEC61B
806.8229224	0.070837469	0.230737755	0.307004241	0.758840144	0.999467288	ENSG00000145715	RASA1
811.4912188	0.057465846	0.214478584	0.267932792	0.788751052	0.999467288	ENSG00000171566	PLRG1
811.7149889	-0.113611397	0.265871533	-0.427316889	0.669148533	0.997568624	ENSG00000086015	MAST2
793.2239714	-0.667050137	0.292757723	-2.278505692	0.022696466	0.744905651	ENSG00000173113	TRMT112
801.2068957	-0.132449279	0.260213957	-0.509001442	0.610751213	0.99483168	ENSG00000137970	RPL7P9
805.3101291	-0.044506925	0.211777666	-0.21015873	0.833543789	0.999467288	ENSG00000213676	ATF6B
799.7499378	-0.329112053	0.216754828	-1.518360886	0.128923446	0.928627271	ENSG00000110717	NDUFS8
807.6200254	0.05310146	0.224188771	0.236860481	0.812765029	0.999467288	ENSG00000129292	PHF20L1
802.3234705	-0.413832845	0.25340344	-1.633098768	0.102448226	0.92169898	ENSG00000169057	MECP2
808.309981	0.077848458	0.255525325	0.30466044	0.760624782	0.999467288	ENSG00000182022	CHST15
791.586126	0.024713291	0.313219529	0.078900862	0.937111478	0.999527751	ENSG00000173442	EHBP1L1
809.173926	-0.18463846	0.301628763	-0.612138108	0.540446387	0.99483168	ENSG00000123096	SSPN
795.7626021	0.165444595	0.265872177	0.622271184	0.533763565	0.99483168	ENSG00000141232	TOB1
798.3196581	-0.331427597	0.210110812	-1.577394303	0.114704826	0.923922314	ENSG00000157191	NECAP2
801.2479111	0.150746619	0.222895514	0.67631069	0.498843407	0.993513219	ENSG00000162378	ZYG11B
804.9178523	-0.079754301	0.30036106	-0.265528098	0.790602667	0.999467288	ENSG00000126062	TMEM115
813.7045093	0.319936163	0.312838211	1.02268889	0.306454966	0.973427483	ENSG00000011422	PLAUR
796.780942	0.005508608	0.259642409	0.021216134	0.983073244	0.999527751	ENSG00000204370	SDHD
799.7560626	-0.036173456	0.191651518	-0.188745995	0.8502919	0.999467288	ENSG00000122692	SMU1
800.1950967	-0.214240732	0.222481973	-0.962957715	0.335568744	0.977411637	ENSG00000086666	ZFAND6
787.845971	0.044517307	0.293607482	0.151621842	0.879485205	0.999467288	ENSG00000160285	LSS
795.9789629	-0.143238174	0.21785102	-0.657505177	0.510856143	0.993513219	ENSG00000070540	WIPI1
804.2143585	0.017938215	0.21392022	0.083854697	0.933171959	0.999527751	ENSG00000075413	MARK3
795.6390165	-0.031314102	0.211522334	-0.148041585	0.88230995	0.999467288	ENSG00000136636	KCTD3
790.2894789	-0.027244701	0.254935754	-0.106868891	0.914892993	0.999527751	ENSG00000144645	OSBPL10
797.541045	0.506939769	0.3180426	1.59393669	0.110950216	0.923922314	ENSG00000167325	RRM1
796.4793264	-0.12857113	0.241651476	-0.532051913	0.594690041	0.99483168	ENSG00000204310	AGPAT1
794.516913	-0.313012486	0.211376934	-1.480826127	0.138652912	0.934605957	ENSG00000182473	EXOC7
791.7062765	-0.309278079	0.258449683	-1.196666506	0.231436567	0.959575179	ENSG00000126254	RBM42
789.7489335	-0.096692768	0.234450818	-0.412422394	0.680029852	0.998869317	ENSG00000182117	NOP10
807.4708451	-0.204911268	0.310719993	-0.659472426	0.509592447	0.993513219	ENSG00000162702	ZNF281
787.9926117	-0.109402768	0.331730015	-0.329794602	0.741555166	0.999467288	ENSG00000110315	RNF141
791.3490019	-0.122167877	0.245528203	-0.497571665	0.61878598	0.99483168	ENSG00000130733	YIPF2
792.8258369	-0.481104191	0.278389217	-1.728171067	0.083957559	0.901307093	ENSG00000124702	KLHDC3
794.4991744	-0.250929987	0.223971713	-1.120364639	0.262558407	0.962465655	ENSG00000127511	SIN3B
804.0610633	-0.164809248	0.321719288	-0.512276554	0.608457472	0.99483168	ENSG00000144711	IQSEC1
797.3785995	0.049301592	0.209276735	0.235580856	0.81375793	0.999467288	ENSG00000162819	BROX
795.8422498	0.319672668	0.203101258	1.573957103	0.115497367	0.923922314	ENSG00000163625	WDFY3
799.4485523	-0.021997852	0.274002691	-0.080283341	0.936011907	0.999527751	ENSG00000064932	SBNO2

794.0261305	-0.300661627	0.229678871	-1.309052179	0.190516676	0.949094286	ENSG00000253719	ATXN7L3B
791.059248	-0.218165707	0.230121154	-0.948047164	0.343105444	0.977691382	ENSG00000095321	CRAT
792.2364947	0.164284975	0.19850591	0.827607474	0.407892834	0.990226751	ENSG00000155016	CYP2U1
804.798037	0.366625626	0.496147028	0.738945525	0.459940077	0.99135833	ENSG00000069667	RORA
790.675635	-0.325211139	0.228948616	-1.420454704	0.155475347	0.939464191	ENSG00000102178	UBL4A
799.9096565	0.148094717	0.251129762	0.589713925	0.555382458	0.99483168	ENSG00000117713	ARID1A
789.1692992	-0.176167218	0.237068202	-0.743107747	0.45741645	0.99135833	ENSG00000182636	NDN
787.9145218	0.035055628	0.20521084	0.170827367	0.86435951	0.999467288	ENSG00000131788	PIAS3
797.7945042	0.346885613	0.23558664	1.472433294	0.140903908	0.935644597	ENSG00000163714	U2SURP
788.5307278	-0.581998968	0.369669282	-1.574377414	0.115400222	0.923922314	ENSG00000131669	NINJ1
790.2000014	0.198875135	0.203790085	0.975882287	0.329122801	0.976026345	ENSG00000176105	YES1
798.7436114	0.406631462	0.264188698	1.539170543	0.123762667	0.927794614	ENSG00000132718	SYT11
788.3511247	0.120692185	0.21251115	0.567933425	0.570080175	0.99483168	ENSG00000113269	RNF130
795.8244841	0.221963755	0.26013858	0.853251965	0.393519592	0.988378925	ENSG00000105726	ATP13A1
792.1695359	-0.247994301	0.242525284	-1.022550298	0.30652052	0.973427483	ENSG00000115825	PRKD3
789.8034698	0.244574738	0.237362616	1.030384404	0.302829592	0.972793733	ENSG00000123200	ZC3H13
788.9553102	-0.100822351	0.276372828	-0.364805585	0.715256536	0.99900742	ENSG00000116604	MEF2D
794.2557934	0.23567085	0.240514456	0.979861476	0.327154501	0.976026345	ENSG00000089057	SLC23A2
800.2518307	-0.351458374	0.313119414	-1.122441975	0.261674566	0.962465655	ENSG00000177674	AGTRAP
780.4481977	-0.36679467	0.212961338	-1.722353329	0.085005533	0.901307093	ENSG00000138073	PREB
778.5873463	0.196037661	0.242148415	0.809576478	0.418183631	0.990226751	ENSG00000065054	SLC9A3R2
790.2865591	-0.059763557	0.222104576	-0.269078461	0.787869305	0.999467288	ENSG00000144283	PKP4
782.6664378	0.007179831	0.237755722	0.03019835	0.975908864	0.999527751	ENSG00000170445	HARS
791.0680581	0.234729656	0.203175089	1.155307261	0.247964623	0.962465655	ENSG00000160131	VMA21
779.2712942	-0.281825336	0.27771027	-1.014817838	0.310192669	0.973427483	ENSG00000154723	ATP5PF
786.8664351	-0.079222637	0.199552265	-0.397001944	0.691366027	0.998869317	ENSG00000151148	UBE3B
787.9293762	0.059378739	0.257682298	0.230433907	0.817754616	0.999467288	ENSG00000153029	MR1
780.3002834	0.034419355	0.222339129	0.154805658	0.87697453	0.999467288	ENSG00000113312	TTC1
794.4824349	-0.120773955	0.236474883	-0.510726355	0.609542684	0.99483168	ENSG00000102921	N4BP1
782.6050128	-1.065483044	0.318347365	-3.346919627	0.000817149	0.210751479	ENSG00000102802	MEDAG
783.5422657	0.089527049	0.307944057	0.290725043	0.771261617	0.999467288	ENSG00000100567	PSMA3
791.2785033	-0.142407987	0.234508757	-0.607260848	0.543677817	0.99483168	ENSG00000169398	PTK2
778.3533447	-0.769120925	0.381683205	-2.015076678	0.043896615	0.807325344	ENSG00000196683	TOMM7
787.9952649	0.207108355	0.205393279	1.008350203	0.31328637	0.973427483	ENSG00000148719	DNAJB12
779.735916	-0.060371868	0.222837143	-0.27092372	0.786449701	0.999467288	ENSG00000178234	GALNT11
793.7682858	0.90143361	0.382573256	2.356237912	0.01846109	0.726053908	ENSG00000137309	HMGA1
790.171127	0.462079823	0.217470397	2.124794135	0.033603794	0.775963115	ENSG00000162695	SLC30A7
783.149307	-0.059581963	0.228443702	-0.260816835	0.794233761	0.999467288	ENSG00000170832	USP32
798.2199979	0.673961965	0.320198668	2.104824388	0.035306597	0.784512596	ENSG00000166833	NAV2
779.5349972	-0.024252223	0.251983009	-0.096245471	0.923325618	0.999527751	ENSG00000132676	DAP3
778.4170354	0.324280574	0.262453799	1.235572033	0.216617686	0.954900762	ENSG00000145390	USP53
779.4036103	-0.133958773	0.202482895	-0.661580688	0.508239988	0.993513219	ENSG00000048140	TSPAN17
780.9609012	-0.293243366	0.208718164	-1.40497291	0.160029334	0.941239523	ENSG00000169221	TBC1D10B
773.9126121	-0.262100839	0.275380602	-0.951776697	0.341210242	0.977691382	ENSG00000168394	TAP1
787.2169511	-0.563473554	0.227516724	-2.476624763	0.013263126	0.670420654	ENSG00000122694	GLIPR2
776.8402502	0.04706866	0.302054883	0.155828171	0.876168465	0.999467288	ENSG00000104852	SNRNP70
778.3126891	-0.407078594	0.224291652	-1.814952052	0.069531278	0.877912272	ENSG00000100239	PPP6R2
778.6396203	-0.4422275	0.272892661	-1.620518111	0.105121027	0.92169898	ENSG00000143158	MPC2
775.4923622	-0.619890733	0.34617113	-1.790706036	0.073340481	0.888663898	ENSG00000013016	EHD3
785.6919996	0.263137028	0.232896334	1.129846159	0.258541054	0.962465655	ENSG00000075292	ZNF638
778.8312703	0.230305881	0.221490413	1.039800673	0.298432516	0.972186492	ENSG00000172081	MOB3A
772.9663857	0.323939281	0.289708613	1.118155505	0.263500584	0.962465655	ENSG00000003402	CFLAR
792.5507568	0.152786019	0.401452045	0.380583486	0.703512337	0.99900742	ENSG00000143815	LBR
783.5196616	-0.110201221	0.194844861	-0.565584436	0.57167631	0.99483168	ENSG00000149658	YTHDF1
782.7300077	0.134921375	0.212204342	0.635808738	0.524901092	0.99483168	ENSG00000115904	SOS1
772.7516048	-0.240334423	0.405226187	-0.593087097	0.553122865	0.99483168	ENSG00000127528	KLF2
776.033243	-0.041916451	0.280477321	-0.149446847	0.881201048	0.999467288	ENSG00000135924	DNAJB2
772.8580189	-0.126175297	0.307214083	-0.410708049	0.681286624	0.998869317	ENSG00000112208	BAG2
780.3155599	0.077084098	0.239176053	0.322290202	0.747232855	0.999467288	ENSG00000143797	MBOAT2
776.6486449	-0.096288405	0.235468632	-0.40892243	0.682596588	0.998869317	ENSG00000166946	CCNDBP1
773.6165567	0.148025191	0.208426859	0.710202091	0.477578824	0.99135833	ENSG00000172493	AFF1
787.7734242	0.207613447	0.269547161	0.770230511	0.441163168	0.99135833	ENSG00000092439	TRPM7
774.5451256	0.452255664	0.277377882	1.630467649	0.103002697	0.92169898	ENSG00000133789	SWAP70
783.0459736	0.058980161	0.224750693	0.262424826	0.792993935	0.999467288	ENSG00000005812	FBXL3

799.4543048	-0.725600234	0.449612598	-1.613834302	0.106563372	0.922165836	ENSG00000142632	ARHGEF19
773.0561342	-0.123941719	0.204831135	-0.605092185	0.545117746	0.99483168	ENSG00000146007	ZMAT2
785.8467162	-0.397878358	0.283001582	-1.405922733	0.15974707	0.940573556	ENSG00000164603	BMT2
779.2558544	-0.620626108	0.244906847	-2.534131303	0.011272648	0.644117389	ENSG00000149823	VPS51
767.8924237	-0.205700934	0.246243064	-0.835357269	0.403516582	0.988927059	ENSG00000115204	MPV17
779.2975639	-0.247863646	0.202792947	-1.222249839	0.221613162	0.956884001	ENSG00000164830	OXR1
774.4791298	-0.324710411	0.211271431	-1.536934786	0.12430928	0.927794614	ENSG00000141425	RPRD1A
753.6339185	0.158364153	0.39881491	0.397086841	0.691303423	0.998869317	ENSG00000173918	C1QTNF1
785.207941	0.037490092	0.25157339	0.149022484	0.881535891	0.999467288	ENSG00000065883	CDK13
774.7601064	0.39067037	0.267116584	1.462546294	0.143591568	0.935644597	ENSG00000005810	MYCBP2
779.1594788	-0.197463009	0.241429836	-0.817889835	0.413420095	0.990226751	ENSG00000100029	PES1
781.9848377	0.047928591	0.259681772	0.184566636	0.853568957	0.999467288	ENSG00000198265	HELZ
776.0576621	-0.010854968	0.315676386	-0.034386379	0.972569045	0.999527751	ENSG00000163870	TPRA1
769.7742646	-0.229970704	0.227791966	-1.009564599	0.312703937	0.973427483	ENSG00000182180	MRPS16
768.8025723	-0.549261877	0.291351236	-1.885222403	0.059399817	0.857911562	ENSG00000214253	FIS1
780.4275678	0.12351477	0.297998263	0.41448151	0.678521506	0.998869317	ENSG00000179094	PER1
779.6598172	0.17820877	0.290891311	0.612630088	0.540120959	0.99483168	ENSG00000107036	RIC1
766.9040243	-0.336116958	0.219136081	-1.533827546	0.125072084	0.927794614	ENSG00000068903	SIRT2
766.9263539	-0.281982431	0.228209897	-1.235627527	0.216597048	0.954900762	ENSG00000101363	MANBAL
780.2884812	-0.316168802	0.289964533	-1.0903706	0.275549927	0.962465655	ENSG00000140948	ZCCHC14
772.7502928	0.162231957	0.240948118	0.673306596	0.500752257	0.993513219	ENSG00000105135	ILVBL
775.0727722	0.180896212	0.265722419	0.68077136	0.496016175	0.993513219	ENSG00000133226	SRRM1
775.2447697	0.01276238	0.206400539	0.061833075	0.950695764	0.999527751	ENSG00000111450	STX2
767.4787279	-0.016359186	0.262252362	-0.062379558	0.950260574	0.999527751	ENSG00000141556	TBCD
780.5707011	-9.16E-05	0.273133845	-0.000335377	0.999732408	0.9998586	ENSG00000035681	NSMAF
770.7183147	0.264559137	0.210607459	1.256171735	0.209053712	0.954310328	ENSG00000101290	CDS2
766.9284509	-0.016933837	0.226484523	-0.074768184	0.940399156	0.999527751	ENSG00000198160	MIER1
771.2662925	0.0987447	0.207536103	0.475795288	0.634220227	0.99544042	ENSG00000121060	TRIM25
764.5880198	0.014423858	0.326219686	0.044215168	0.964732892	0.999527751	ENSG00000152556	PFKM
773.7994532	0.204044081	0.264434839	0.771623293	0.440337574	0.99135833	ENSG00000052795	FNIP2
767.2519016	0.262204762	0.266036971	0.9855952	0.324331794	0.975494399	ENSG00000164050	PLXNB1
756.4173467	0.560447349	0.370079378	1.514397675	0.129925009	0.929373153	ENSG00000105472	CLEC11A
777.3255171	-0.240524277	0.310126055	-0.775569395	0.438003277	0.991301167	ENSG00000183098	GPC6
775.7772082	0.009944921	0.305732995	0.032528124	0.974050888	0.999527751	ENSG00000168297	PXK
749.7420779	0.508860226	0.41054609	1.239471617	0.215170893	0.954310328	ENSG00000253368	TRNP1
772.9422272	-0.073598029	0.267967333	-0.274652988	0.783582853	0.999467288	ENSG00000174233	ADCY6
764.1935155	0.083603777	0.306189581	0.273045794	0.784818014	0.999467288	ENSG00000091483	FH
766.0087223	0.018783147	0.265762089	0.070676547	0.943655187	0.999527751	ENSG00000152684	PELO
773.0147716	-0.14398789	0.259072145	-0.555782983	0.57835922	0.99483168	ENSG00000169410	PTPN9
771.2832539	0.082514088	0.214957388	0.383862536	0.701080335	0.998869317	ENSG00000164164	OTUD4
768.0036878	-0.199934977	0.249617798	-0.800964428	0.423152239	0.991301167	ENSG00000164402	8-Sep
763.5054806	-0.173449223	0.22927693	-0.756505345	0.449346269	0.99135833	ENSG00000136877	FPGS
767.6599481	-0.031489405	0.191051034	-0.164821956	0.869084122	0.999467288	ENSG00000164902	PHAX
769.7275119	-0.31403645	0.276037256	-1.137659657	0.255262626	0.962465655	ENSG00000111424	VDR
759.6760914	-0.394590466	0.23513532	-1.678142042	0.093319373	0.921147998	ENSG00000089486	CDIP1
757.414326	-0.021257098	0.242736561	-0.08757271	0.930216293	0.999527751	ENSG00000185043	CIB1
762.2350955	-0.224427378	0.249185735	-0.900642962	0.367778184	0.981269214	ENSG00000168894	RNF181
758.822326	-0.032504143	0.272417298	-0.119317471	0.905023843	0.999527751	ENSG00000113456	RAD1
760.0557587	-0.066732413	0.246748963	-0.270446579	0.786816709	0.999467288	ENSG00000115128	SF3B6
766.234143	-0.009434546	0.217614311	-0.043354438	0.965418997	0.999527751	ENSG00000155827	RNF20
764.1212795	0.554032059	0.323072212	1.714886142	0.086366104	0.901307093	ENSG00000089327	FXYD5
762.5212453	0.027687284	0.226497404	0.122241065	0.902708105	0.999527751	ENSG00000134283	PPHLN1
758.2780126	0.134114943	0.284543079	0.471334406	0.637401949	0.99653524	ENSG00000196547	MAN2A2
774.1405504	0.506786791	0.319036095	1.588493586	0.11217477	0.923922314	ENSG00000157106	SMG1
757.3188578	-0.530186582	0.228550975	-2.31977388	0.020353113	0.73997555	ENSG00000108774	RAB5C
753.4805059	-1.014522982	0.352687704	-2.876547634	0.004020515	0.45638972	ENSG00000101400	SNTA1
762.7124143	0.692185112	0.243789447	2.8392743	0.004521627	0.469826318	ENSG00000109685	NSD2
753.115196	0.498456115	0.324588024	1.53565775	0.124622342	0.927794614	ENSG00000169499	PLEKHA2
758.544395	-0.276446386	0.233872213	-1.182040326	0.2371897	0.962465655	ENSG00000197355	UAP1L1
758.2861453	-0.056853973	0.280669731	-0.20256539	0.839474744	0.999467288	ENSG00000213923	CSNK1E
757.7043484	0.447359724	0.254354228	1.758805928	0.078610478	0.897052166	ENSG00000197912	SPG7
766.287001	0.061327961	0.244121522	0.251218986	0.801644806	0.999467288	ENSG00000108094	CUL2
754.8433685	0.266165198	0.266134427	1.000115622	0.317254557	0.974768742	ENSG00000176619	LMNB2
763.575047	0.149844905	0.239651689	0.625261208	0.531799635	0.99483168	ENSG00000162104	ADCY9

760.1246271	0.001911085	0.213492994	0.008951514	0.992857821	0.999527751	ENSG00000172534	HCFC1	
760.3611132	0.284832384	0.229365801	1.241825865	0.214300817	0.954310328	ENSG00000107929	LARP4B	
762.066479	0.186517006	0.246365317	0.757074936	0.449004969	0.99135833	ENSG00000100888	CHD8	
755.88231	-0.287501957	0.200828968	-1.431576134	0.152265164	0.938052926	ENSG00000105402	NAPA	
765.330769	-0.013355292	0.236160909	-0.05655166	0.954902342	0.999527751	ENSG00000077254	USP33	
755.7573082	-0.076877555	0.265321055	-0.289752939	0.772005253	0.999467288	ENSG00000189266	PNRC2	
762.930741	0.251534888	0.285651795	0.880564702	0.378553471	0.982609538	ENSG00000116977	LGALS8	
755.6956922	-0.087840308	0.39074906	-0.224799793	0.822135027	0.999467288	ENSG00000126603	GLIS2	
760.7218151	0.186000428	0.292065286	0.636845378	0.524225565	0.99483168	ENSG00000183578	TNFAIP8L3	
755.313085	0.082130077	0.281114155	0.292159165	0.770164932	0.999467288	ENSG00000145246	ATP10D	
755.7962089	-0.226199601	0.197046266	-1.147951726	0.250988493	0.962465655	ENSG00000117419	ERI3	
763.8849851	-0.087198882	0.274776254	-0.317345043	0.750981803	0.999467288	ENSG00000116698	SMG7	
756.6468865	-0.074010018	0.228590202	-0.323767237	0.746114262	0.999467288	ENSG00000106829	TLE4	
753.0303434	0.193929816	0.187035102	1.036863213	0.299799611	0.972186492	ENSG00000204120	GIGYF2	
756.3188273	0.231857321	0.217895315	1.064076668	0.287293969	0.965284229	ENSG00000103042	SLC38A7	
752.8017841	-0.107816389	0.206042373	-0.523272897	0.600784359	0.99483168	ENSG00000124226	RNF114	
756.8385668	-0.407897688	0.200175182	-2.037703599	0.041579584	0.80071191	ENSG00000122490	PQLC1	
756.9961762	0.198940234	0.216656092	0.918230512	0.358498198	0.979427314	ENSG00000103194	USP10	
753.2852948	-0.27052924	0.21447114	-1.261378292	0.207172586	0.954310328	ENSG00000130313	PGLS	
757.3230662	-0.342557188	0.225221137	-1.520981522	0.128264475	0.928627271	ENSG00000176095	IP6K1	
755.573921	-0.008922913	0.347568047	-0.02567242	0.979518622	0.999527751	ENSG00000113504	SLC12A7	
746.5806088	-0.312002675	0.233494724	-1.336230086	0.18147408	0.949094286	ENSG00000137100	DCTN3	
750.7146776	-0.137227285	0.19522388	-0.702922639	0.482103962	0.991525194	ENSG00000100387	RBX1	
761.4713506	-0.057734545	0.257781693	-0.223966817	0.822783123	0.999467288	ENSG00000244462	RBM12	
744.7803277	-0.34597296	0.277358769	-1.24738425	0.212256639	0.954310328	ENSG00000111639	MRPL51	
744.0782989	0.193902909	0.296775062	0.653366587	0.513519974	0.993513219	ENSG00000205726	ITSN1	
753.5637801	-0.463701457	0.248280784	-1.867649398	0.061810956	0.865910462	ENSG00000109436	TBC1D9	
756.2985897	0.197718154	0.237912941	0.831052543	0.405943948	0.990045328	ENSG00000126821	SGPP1	
750.5486192	-0.742500101	0.270873856	-2.741128697	0.006122852	0.499617804	ENSG00000115325	DOK1	
764.7254227	2.205333199	1.299045076	1.697657179	0.089572484	0.910413547	ENSG00000131747	TOP2A	
755.3121262	0.129513629	0.230902508	0.560901786	0.574864493	0.99483168	ENSG00000102189	EEA1	
750.3676693	0.030192617	0.221679439	0.136199449	0.891663611	0.999467288	ENSG00000198954	KIF1BP	
749.587856	-0.3319084	0.214800918	-1.545190789	0.122300122	0.924322688	ENSG00000149930	TAOK2	
744.6047757	0.150934491	0.284083845	0.531302621	0.595209089	0.99483168	ENSG00000006744	ELAC2	
747.618671	-0.100059275	0.248658917	-0.402395682	0.687392847	0.998869317	ENSG00000111144	LTA4H	
756.077706	0.010805904	0.239442398	0.045129451	0.964004127	0.999527751	ENSG00000136381	IREB2	
747.4470368	-0.275336849	0.193573893	-1.422386279	0.154914144	0.939432741	ENSG00000169230	PRELID1	
749.8222034	-0.333080131	0.199624831	-1.668530562	0.09521045	0.921147998	ENSG00000165283	STOML2	
748.5255021	-0.19397753	0.264954659	-0.732115945	0.464097803	0.99135833	ENSG00000170145	SIK2	
754.6790753	-0.180502016	0.221515653	-0.814849937	0.415158213	0.990226751	ENSG00000198060		5-Mar
742.6151293	-0.475868572	0.262708375	-1.81139475	0.070079768	0.878416974	ENSG00000198931	APRT	
759.6633576	0.302772078	0.352489682	0.858953024	0.390366434	0.987574427	ENSG00000145685	LHFPL2	
753.8852531	-0.154613619	0.241719745	-0.639640006	0.522406667	0.994611059	ENSG00000185009	AP3M1	
762.9083488	0.440197614	0.318184026	1.383468617	0.166521224	0.943223078	ENSG00000114423	CBLB	
750.8760455	-0.079279972	0.221590866	-0.357776354	0.720510688	0.99900742	ENSG00000172466	ZNF24	
749.9530213	0.123605724	0.221884482	0.557072414	0.577477954	0.99483168	ENSG00000115145	STAM2	
750.7801913	0.145233162	0.215880144	0.672749054	0.501106954	0.993513219	ENSG00000084093	REST	
751.6041206	-0.032361798	0.242915229	-0.133222597	0.89401734	0.999467288	ENSG00000198642	KLHL9	
744.9121691	0.006768301	0.193718816	0.034938792	0.972128548	0.999527751	ENSG00000085377	PREP	
741.3615628	-0.150280928	0.276299625	-0.543905652	0.586506399	0.99483168	ENSG00000171530	TBCA	
749.164804	-0.278351068	0.217954411	-1.277106837	0.201564531	0.954310328	ENSG00000165156	ZHX1	
746.7545297	0.121640077	0.228144519	0.533171157	0.593915106	0.99483168	ENSG00000163938	GNL3	
749.971915	0.067864553	0.245518503	0.27641319	0.782230727	0.999467288	ENSG00000136699	SMPD4	
740.210157	0.490914415	0.419580506	1.170012448	0.24199596	0.962465655	ENSG00000110090	CPT1A	
747.1641199	0.074157981	0.211874449	0.350009082	0.726331882	0.999467288	ENSG00000150593	PDCD4	
743.4339228	-0.222974116	0.198689922	-1.122221567	0.261768245	0.962465655	ENSG00000144021	CIAO1	
743.0056908	0.233099513	0.248065572	0.939668939	0.347387402	0.977691382	ENSG00000122515	ZMIZ2	
754.1338226	0.071085227	0.248428668	0.286139388	0.774771365	0.999467288	ENSG00000062650	WAPL	
738.0322164	-0.716819143	0.272323111	-2.632237636	0.008482451	0.604581883	ENSG00000103260	METRN	
741.6748821	0.081011772	0.263976783	0.306889763	0.758927281	0.999467288	ENSG00000090674	MCOLN1	
749.708636	0.246702685	0.259264401	0.951548627	0.341325946	0.977691382	ENSG00000005889	ZFX	
744.9905229	0.252390498	0.213867305	1.180126612	0.237949862	0.962465655	ENSG00000109184	DCUN1D4	
767.3852905	-0.488502539	0.40963073	-1.192543683	0.233048133	0.959575179	ENSG00000148572	NRBF2	
752.9586821	0.342513491	0.307260676	1.114732597	0.264965028	0.962465655	ENSG00000139496	NUP58	

761.0149786	-0.48096894	0.361224488	-1.33149594	0.18302588	0.949094286	ENSG00000100139	MICALL1
737.6786587	-0.202581939	0.249509722	-0.811920024	0.416837524	0.990226751	ENSG00000119185	ITGB1BP1
744.3515073	-0.213112512	0.219783554	-0.969647222	0.332222366	0.976026345	ENSG00000114302	PRKAR2A
735.8852899	-0.264819695	0.267289857	-0.990758489	0.321803524	0.975337095	ENSG00000002549	LAP3
761.8906305	-1.133813982	0.321135065	-3.530645221	0.000414547	0.176097288	ENSG00000147883	CDKN2B
744.105285	-0.131189161	0.239887162	-0.546878624	0.584462122	0.99483168	ENSG00000140577	CRTC3
743.2795586	-0.076551837	0.263668897	-0.29033321	0.771561335	0.999467288	ENSG00000126858	RHOT1
733.7023212	-0.235695644	0.248311005	-0.949195317	0.342521283	0.977691382	ENSG00000117118	SDHB
739.1334595	0.511193193	0.295865967	1.727786399	0.084026527	0.901307093	ENSG00000112902	SEMA5A
734.3133345	-0.014220272	0.262463554	-0.054179986	0.956791766	0.999527751	ENSG00000128609	NDUFA5
744.8612696	0.226126049	0.240697204	0.939462717	0.347493226	0.977691382	ENSG00000066777	ARFGEF1
740.202644	-0.023563327	0.192108494	-0.122656354	0.902379228	0.999527751	ENSG00000149480	MTA2
754.0802503	-0.437387801	0.424033767	-1.031492855	0.302309757	0.972793733	ENSG00000251493	FOXD1
756.1120566	-1.051298644	0.515763269	-2.038335623	0.04151638	0.800562177	ENSG00000187955	COL14A1
740.7201532	-0.237455125	0.245444385	-0.967449816	0.333319217	0.976026345	ENSG00000166295	ANAPC16
751.3261924	-0.092463795	0.296373652	-0.311983857	0.755052792	0.999467288	ENSG00000167470	MIDN
735.0522438	0.006430376	0.2466062	0.026075482	0.979197133	0.999527751	ENSG00000106028	SSBP1
738.08678	-0.136551674	0.195458959	-0.698620698	0.484789105	0.991892545	ENSG00000136280	CCM2
738.8304863	0.156094885	0.204551459	0.763108147	0.445398902	0.99135833	ENSG00000062725	APPBP2
739.0426642	0.111305358	0.22524081	0.494161596	0.62119206	0.99483168	ENSG00000036054	TBC1D23
753.9248459	-0.317444013	0.410928366	-0.772504503	0.439815681	0.991301167	ENSG00000115318	LOXL3
742.5048423	-0.198005047	0.248542284	-0.796665436	0.425645363	0.991301167	ENSG00000255529	POLR2M
739.4977097	-0.337603427	0.213854741	-1.57865767	0.114414599	0.923922314	ENSG00000178464	RPL10P16
745.392124	0.190655075	0.269115009	0.708452032	0.47866459	0.99135833	ENSG00000152683	SLC30A6
743.8628786	0.489043488	0.277712523	1.760970237	0.078243441	0.897052166	ENSG00000138688	KIAA1109
736.0138722	-0.248440538	0.229478485	-1.082631071	0.27897221	0.963231086	ENSG00000186184	POLR1D
740.774384	0.142356818	0.214490208	0.663698445	0.506883335	0.993513219	ENSG00000147548	NSD3
733.0018112	-0.095993063	0.224451564	-0.427678298	0.668885352	0.997568624	ENSG00000168724	DNAJC21
738.9430216	0.142262534	0.213162897	0.667388817	0.504523807	0.993513219	ENSG00000156162	DPY19L4
745.0066168	0.115221679	0.278025547	0.414428388	0.678560402	0.998869317	ENSG00000104361	NIPAL2
739.6691229	-0.046940907	0.195443831	-0.240175947	0.81019386	0.999467288	ENSG00000188725	SMIM15
734.1675787	0.030407549	0.21697218	0.140144922	0.888545488	0.999467288	ENSG00000111596	CNOT2
730.5283659	-0.268205677	0.274440841	-0.977280479	0.328430315	0.976026345	ENSG00000064961	HMG20B
739.3285486	-0.106077171	0.232209999	-0.45681569	0.647803527	0.997368416	ENSG00000101310	SEC23B
734.741931	-0.2658039	0.330253139	-0.804848975	0.420906836	0.991301167	ENSG00000135931	ARMC9
738.3756037	0.569971731	0.219870637	2.59230491	0.009533524	0.622034307	ENSG00000129003	VPS13C
735.3631502	0.164518062	0.193354592	0.850861932	0.394846054	0.988378925	ENSG00000174953	DHX36
734.7618058	0.274650637	0.213182593	1.288335196	0.197629307	0.954310328	ENSG00000147854	UHRF2
724.3064861	-0.110414845	0.332577147	-0.331997692	0.739891001	0.999467288	ENSG00000211445	GPX3
734.5476273	-0.173373963	0.224430313	-0.772506892	0.439814267	0.991301167	ENSG00000048544	MRPS10
733.0928735	-0.165334429	0.22289572	-0.741756859	0.458234663	0.99135833	ENSG00000112146	FBXO9
737.722857	0.099223602	0.261262212	0.379785507	0.704104642	0.99900742	ENSG00000005483	KMT2E
733.1832092	-0.460667101	0.264658176	-1.740611636	0.081751677	0.897807954	ENSG00000177951	BET1L
736.750354	-0.203608777	0.214570937	-0.948911253	0.342665751	0.977691382	ENSG00000050748	MAPK9
729.8941902	-0.054897872	0.21258309	-0.258241952	0.796220183	0.999467288	ENSG00000188021	UBQLN2
731.9289563	0.195670209	0.217352269	0.900244614	0.367990089	0.981269214	ENSG00000157800	SLC37A3
724.1337975	0.525887903	0.286958677	1.832625894	0.066858225	0.87420149	ENSG00000181523	SGSH
733.7601217	-0.00590031	0.194429613	-0.030346764	0.975790501	0.999527751	ENSG00000028203	VEZT
731.3759918	0.196197213	0.205385334	0.95526399	0.339444213	0.977691382	ENSG00000151893	CACUL1
738.6635533	-0.43724841	0.267391248	-1.635238302	0.101999102	0.92169898	ENSG00000233476	EEF1A1P6
729.9162364	-0.245223656	0.224627337	-1.091691061	0.274968916	0.962465655	ENSG00000135446	CDK4
724.1261891	-0.121063697	0.285354559	-0.424257098	0.671378324	0.997568624	ENSG00000172340	SUCLG2
725.4949544	-0.431479295	0.293708163	-1.469074914	0.141812471	0.935644597	ENSG00000125648	SLC25A23
728.2623762	-0.17870428	0.214444118	-0.833337289	0.404654531	0.989741812	ENSG00000180104	EXOC3
734.3010304	-0.254579384	0.233347948	-1.090986172	0.275278967	0.962465655	ENSG00000235552	RPL6P27
738.0486305	0.156198718	0.332008438	0.47046611	0.638022041	0.996553586	ENSG00000162298	SYVN1
734.1458615	-0.011567292	0.249620654	-0.046339483	0.96303967	0.999527751	ENSG00000034677	RNF19A
721.9254331	0.216691921	0.247183326	0.876644573	0.380679721	0.983746147	ENSG00000206053	JPT2
733.2965426	-0.096709731	0.221461547	-0.436688593	0.662337203	0.997568624	ENSG00000074603	DPP8
732.1026978	0.120542735	0.202402937	0.595558234	0.551470385	0.99483168	ENSG00000138459	SLC35A5
755.026973	0.017571801	0.817627768	0.021491199	0.982853824	0.999527751	ENSG00000128965	CHAC1
738.3617187	0.188029524	0.258261976	0.728057324	0.466578496	0.99135833	ENSG00000119523	ALG2
723.8648712	-0.393754858	0.285966144	-1.376928238	0.168534436	0.943223078	ENSG00000133612	AGAP3
723.3639506	-0.178572598	0.234725834	-0.760770958	0.446793884	0.99135833	ENSG00000167118	URM1

728.3640201	0.025818653	0.209009862	0.123528398	0.901688688	0.999527751	ENSG00000070778	PTPN21
726.7504678	-0.479047986	0.253406918	-1.890429787	0.058700503	0.852928935	ENSG00000237172	B3GNT9
724.1171933	-0.105985486	0.250126363	-0.423727771	0.67176436	0.997568624	ENSG00000140632	GLYR1
729.0512593	0.02148572	0.308747239	0.06959	0.944519997	0.999527751	ENSG00000135686	KLHL36
729.8052817	-0.100805544	0.20110445	-0.501259638	0.616188407	0.99483168	ENSG00000173545	ZNF622
720.1200678	-0.4385823	0.240675159	-1.822299827	0.068409504	0.87420149	ENSG00000140990	NDUFB10
759.1533978	0.259250907	0.475727573	0.544956656	0.585783329	0.99483168	ENSG00000100003	SEC14L2
728.8128653	0.106211233	0.215016629	0.493967528	0.621329113	0.99483168	ENSG00000035928	RFC1
728.0632767	-0.14865597	0.274462854	-0.541625096	0.5880768	0.99483168	ENSG00000175970	UNC119B
723.2137086	0.07617616	0.224456304	0.3393808	0.73432288	0.999467288	ENSG00000008838	MED24
720.1492081	0.334408259	0.332425369	1.005964918	0.314432446	0.974768742	ENSG00000162694	EXTL2
725.615698	-0.428835541	0.209514536	-2.046805676	0.040677168	0.796070921	ENSG00000143294	PRCC
729.4679082	0.272821757	0.248203378	1.099186319	0.271686805	0.962465655	ENSG00000197136	PCNX3
749.3152961	0.492480673	0.456889673	1.077898454	0.281079075	0.963267117	ENSG00000114270	COL7A1
726.9621127	0.375549754	0.294273171	1.276194335	0.201886831	0.954310328	ENSG00000110719	TCIRG1
724.6093357	-0.034441805	0.211006168	-0.16322653	0.870340079	0.999467288	ENSG00000062598	ELMO2
725.3567402	-0.351979764	0.297513783	-1.183070448	0.236781229	0.962465655	ENSG00000122390	NAA60
729.2009765	0.196341685	0.29010415	0.67679723	0.498534616	0.993513219	ENSG00000102606	ARHGEF7
721.0409966	-0.038191479	0.249885258	-0.152836064	0.878527556	0.999467288	ENSG00000105856	HBP1
723.5106526	-0.214348329	0.25182829	-0.851168584	0.394675712	0.988378925	ENSG00000214485	RPL7P1
727.1712114	-0.372645784	0.23068518	-1.615386755	0.106226967	0.922165836	ENSG00000172985	SH3RF3
722.8460674	0.303258689	0.27158314	1.116632974	0.264151287	0.962465655	ENSG00000101361	NOP56
742.2445598	0.175981922	0.499425886	0.352368443	0.724561965	0.999456245	ENSG00000117479	SLC19A2
723.4341116	-0.473008541	0.267191585	-1.770297297	0.076677628	0.897052166	ENSG00000116337	AMPD2
724.4929734	0.153416418	0.221988365	0.691101165	0.489501968	0.993513219	ENSG00000037474	NSUN2
708.6911194	-0.09581732	1.103738814	-0.086811589	0.930821276	0.999527751	ENSG00000162692	VCAM1
711.9422961	0.245868841	0.28329652	0.867885142	0.385457214	0.986047049	ENSG00000146416	AIG1
713.5763264	-0.258579065	0.247229218	-1.045908195	0.29560342	0.970706863	ENSG00000140995	DEF8
728.7253328	0.148344409	0.282194559	0.525681324	0.599109642	0.99483168	ENSG00000157500	APPL1
715.8789121	-0.016217319	0.279996061	-0.057919811	0.953812502	0.999527751	ENSG00000111237	VPS29
724.1758399	0.188518495	0.275924486	0.683224956	0.494464705	0.993513219	ENSG00000177200	CHD9
724.272026	-0.156263767	0.205526731	-0.760308727	0.447070067	0.99135833	ENSG00000141030	COPS3
717.179497	0.263567411	0.346786665	0.760027525	0.447238132	0.99135833	ENSG00000115875	SRSF7
718.772266	0.065986202	0.227908789	0.289528994	0.772176595	0.999467288	ENSG00000136754	ABI1
722.4371458	0.124774985	0.205860508	0.606114237	0.544438898	0.99483168	ENSG00000211456	SACM1L
714.9927507	0.063760096	0.26001199	0.245219831	0.806286222	0.999467288	ENSG00000115649	CNPPD1
720.3873243	-0.122025969	0.279506194	-0.436576976	0.662418163	0.997568624	ENSG00000183580	FBXL7
713.8696563	-0.34063296	0.201334286	-1.691877556	0.090669322	0.912569352	ENSG00000204356	NELFE
720.5226151	0.031355991	0.218679514	0.143387876	0.885983857	0.999467288	ENSG00000114030	KPNA1
713.961136	0.276343252	0.326153029	0.847280961	0.396838536	0.988378925	ENSG00000198951	NAGA
719.5503739	0.266147348	0.260514937	1.021620299	0.306960646	0.973427483	ENSG00000162493	PDPN
715.3744779	-0.20533208	0.287721266	-0.7136493	0.475444065	0.99135833	ENSG00000059122	FLYWCH1
721.8797045	0.143982999	0.227026879	0.634211244	0.525942971	0.99483168	ENSG00000213699	SLC35F6
718.4025476	-0.025713961	0.304087329	-0.084561105	0.932610322	0.999527751	ENSG00000115275	MOGS
721.4801049	0.139545305	0.222446459	0.627320862	0.530448931	0.99483168	ENSG00000116984	MTR
718.1549714	-0.403163618	0.291896759	-1.381185658	0.167221883	0.943223078	ENSG00000163071	SPATA18
708.7661769	-0.272953221	0.308682549	-0.884252192	0.37656009	0.982609538	ENSG00000108840	HDAC5
711.9838693	0.525960001	0.292023415	1.801088453	0.071688939	0.884210289	ENSG00000196159	FAT4
711.2388753	0.442003343	0.353746734	1.249490952	0.211485561	0.954310328	ENSG00000147459	DOCK5
710.0708298	-0.031335396	0.230046597	-0.136213256	0.891652696	0.999467288	ENSG00000181929	PRKAG1
722.9276007	0.040988177	0.238305761	0.171998263	0.863438894	0.999467288	ENSG00000181555	SETD2
715.4840938	-0.051265492	0.192640671	-0.266119773	0.790146967	0.999467288	ENSG00000198420	TCAF1
718.3632933	0.009740726	0.297083909	0.032787792	0.973843813	0.999527751	ENSG00000000003	TSPAN6
717.0888164	0.046093682	0.228771036	0.201483908	0.840320211	0.999467288	ENSG00000136045	PWP1
715.2279834	-0.374817117	0.18992983	-1.973450493	0.04844426	0.833264304	ENSG00000100325	ASCC2
721.8234957	-1.050123664	0.520625251	-2.017043277	0.043690997	0.807325344	ENSG00000105880	DLX5
718.681095	-0.108450131	0.211259713	-0.513349798	0.607706655	0.99483168	ENSG00000101193	GID8
714.7357947	0.138775083	0.268142721	0.517541862	0.604777954	0.99483168	ENSG00000170776	AKAP13
722.4095888	-0.016898036	0.286081135	-0.059067287	0.952898515	0.999527751	ENSG00000114982	KANSL3
714.3321762	-0.12058725	0.204227833	-0.590454533	0.55488596	0.99483168	ENSG00000110107	PRPF19
716.3330385	-0.219807618	0.234593159	-0.936973689	0.348772092	0.977691382	ENSG00000011566	MAP4K3
718.1150072	0.292084995	0.272208444	1.073019595	0.283262325	0.963267117	ENSG00000138802	SEC24B
716.1904536	-0.193122684	0.199679469	-0.967163449	0.333462331	0.976026345	ENSG00000107186	MPDZ
716.1072573	0.0588701	0.264754909	0.222356974	0.824036003	0.999467288	ENSG00000167543	TP53I13

716.8673058	0.285272427	0.28714691	0.993472042	0.320479969	0.975316319	ENSG00000115970	THADA
711.4628495	0.083475713	0.28431172	0.293606302	0.76905876	0.999467288	ENSG00000171150	SOCS5
708.9015845	-0.103568636	0.236522524	-0.437880646	0.661472806	0.997568624	ENSG00000179933	C14orf119
718.2200918	-0.16519716	0.22983661	-0.718759121	0.472289349	0.99135833	ENSG00000244398	AC116533.1
712.4670715	-0.189909921	0.264580771	-0.717776731	0.472894963	0.99135833	ENSG00000132254	ARFIP2
716.196325	0.06180158	0.21082766	0.293137913	0.769416739	0.999467288	ENSG00000086475	SEPHS1
708.9267815	-0.119723432	0.259524309	-0.461318758	0.644569929	0.997368416	ENSG00000163754	GYG1
706.0160283	0.337410843	0.275282078	1.225691282	0.220314872	0.956884001	ENSG00000168807	SNTB2
707.7336674	-0.19402454	0.24191657	-0.802030799	0.422535143	0.991301167	ENSG00000197780	TAF13
694.5815589	-0.343118136	0.451020678	-0.760759213	0.4468009	0.99135833	ENSG00000124785	NRN1
707.452364	-0.123246101	0.252726478	-0.48766596	0.625786467	0.99483168	ENSG00000128016	ZFP36
710.2487694	-0.213334462	0.273414825	-0.780259307	0.43523826	0.991301167	ENSG00000115183	TANC1
719.2569707	0.201475691	0.302042557	0.667044051	0.504743996	0.993513219	ENSG00000223496	EXOSC6
711.913755	-0.10252394	0.195567954	-0.524236912	0.60011377	0.99483168	ENSG00000186575	NF2
708.8196799	-0.42700918	0.236614463	-1.804662212	0.071127557	0.88331496	ENSG00000176340	COX8A
703.9525009	-0.530966945	0.255110293	-2.081323096	0.037404343	0.790190505	ENSG00000168802	CHTF8
713.173833	-0.726196054	0.336806542	-2.156122174	0.031074126	0.775963115	ENSG00000115129	TP53I3
719.9105176	0.209002211	0.264099429	0.791376989	0.428724034	0.991301167	ENSG00000119596	YLPM1
713.2994124	-0.415604099	0.271554667	-1.530462003	0.125902412	0.928627271	ENSG00000160113	NR2F6
716.9291	0.113367855	0.270983404	0.418357189	0.675685982	0.998869317	ENSG00000157827	FMNL2
715.590153	0.052141809	0.248355106	0.209948609	0.833707783	0.999467288	ENSG00000085788	DDHD2
717.9553837	0.033067346	0.272168806	0.121495724	0.903298401	0.999527751	ENSG00000146373	RNF217
715.4897814	0.046848445	0.231152331	0.20267347	0.839390261	0.999467288	ENSG00000136937	NCBP1
710.7895771	-0.203106808	0.283004042	-0.717681651	0.4729536	0.99135833	ENSG00000156860	FBRS
706.201319	-0.421598079	0.241210312	-1.747844344	0.080490984	0.897807954	ENSG00000007080	CCDC124
717.3661146	-0.024708315	0.262996046	-0.093949379	0.925149369	0.999527751	ENSG00000161533	ACOX1
718.5337126	-0.023022438	0.235380139	-0.097809603	0.922083482	0.999527751	ENSG00000101191	DIDO1
714.4756134	0.086155807	0.274851977	0.31346257	0.753929254	0.999467288	ENSG00000169919	GUSB
708.6083945	0.088633432	0.216199054	0.409962166	0.681833701	0.998869317	ENSG00000087263	OGFOD1
712.7674717	0.086660089	0.222332469	0.389777029	0.696701431	0.998869317	ENSG00000157540	DYRK1A
708.0031474	-0.187685272	0.208212599	-0.901411696	0.367369465	0.981269214	ENSG00000166135	HIF1AN
708.1062618	0.026468379	0.200661515	0.131905605	0.895058955	0.999467288	ENSG00000221823	PPP3R1
711.4143121	-0.173470865	0.228731588	-0.758403623	0.44820939	0.99135833	ENSG00000198742	SMURF1
714.8268919	0.036577978	0.223417823	0.163720053	0.869951531	0.999467288	ENSG00000131023	LATS1
711.1830501	0.309246659	0.254339792	1.215879973	0.224030648	0.956884001	ENSG00000072849	DERL2
706.9289973	-0.061273634	0.220656895	-0.277687374	0.781252354	0.999467288	ENSG00000114544	SLC41A3
704.7534379	0.025507035	0.23020647	0.110800686	0.911774401	0.999527751	ENSG00000173418	NAA20
713.7549798	-0.319459293	0.252329632	-1.266039548	0.205498924	0.954310328	ENSG00000182307	C8orf33
695.9918053	2.021225196	0.951197826	2.124926216	0.03359277	0.775963115	ENSG00000164761	TNFRSF11B
704.3270795	0.161336032	0.306768139	0.525921737	0.598942585	0.99483168	ENSG00000128708	HAT1
705.2123652	-0.384119575	0.245039424	-1.567582753	0.11697854	0.923922314	ENSG00000111786	SRSF9
709.784946	-0.152984762	0.204547696	-0.747917307	0.454510038	0.99135833	ENSG00000158863	FAM160B2
700.5737182	-0.134047011	0.283337772	-0.473099685	0.636142062	0.996399501	ENSG00000163541	SUCLG1
715.5596238	0.336964759	0.306498672	1.099400386	0.271593463	0.962465655	ENSG00000111252	SH2B3
703.1445536	0.203201933	0.21647504	0.938685277	0.347892355	0.977691382	ENSG00000123130	ACOT9
702.0666793	0.323053217	0.2349711	1.374863623	0.169173729	0.944252485	ENSG00000155229	MMS19
704.3305216	-0.28596106	0.251870499	-1.13534956	0.256228885	0.962465655	ENSG00000137942	FNBP1L
699.1460225	0.176374834	0.259969004	0.678445627	0.497489188	0.993513219	ENSG00000132581	SDF2
696.7165014	-0.077325233	0.215820446	-0.358285023	0.720130025	0.99900742	ENSG00000165775	FUNDC2
711.7051521	-0.192179709	0.226760693	-0.847500096	0.396716433	0.988378925	ENSG00000138592	USP8
706.9336432	-0.2948843	0.241074389	-1.223208743	0.221250864	0.956884001	ENSG00000141568	FOXK2
705.384378	-0.151544077	0.275630223	-0.549809364	0.582450135	0.99483168	ENSG00000170088	TMEM192
695.2507837	-0.386604834	0.316195765	-1.222675561	0.221452261	0.956884001	ENSG00000165886	UBTD1
695.6255054	-0.334156959	0.253962842	-1.315771065	0.188250894	0.949094286	ENSG00000163382	NAXE
697.4780668	0.465531257	0.318400814	1.462091918	0.143716023	0.935644597	ENSG00000168785	TSPAN5
696.7768851	0.389249349	0.366395213	1.062375642	0.288065187	0.966288987	ENSG00000187688	TRPV2
704.5674662	0.04256416	0.220144688	0.193346295	0.846687772	0.999467288	ENSG00000119912	IDE
703.6479218	-0.656664027	0.26409932	-2.486428313	0.012903256	0.667111125	ENSG00000151208	DLG5
704.4910423	0.018935537	0.258033787	0.073383943	0.941500595	0.999527751	ENSG00000234456	MAGI2-AS3
707.2903608	-0.621902795	0.394525243	-1.576332075	0.114949293	0.923922314	ENSG00000142279	WTIP
699.1860055	-0.041960344	0.202780095	-0.206925361	0.83606815	0.999467288	ENSG00000104142	VPS18
699.490806	-0.351040817	0.21548406	-1.629080205	0.103296041	0.92169898	ENSG00000204590	GNL1
698.1165308	-0.170778681	0.272215268	-0.627366283	0.530419164	0.99483168	ENSG00000130520	LSM4
698.2178828	-0.170203076	0.244442652	-0.696290415	0.486246975	0.993197447	ENSG00000156026	MCU

698.0032973	-0.424735048	0.272455846	-1.558913323	0.119016897	0.923922314	ENSG00000149557	FEZ1
698.5494102	-0.022542365	0.328204179	-0.068683967	0.94524118	0.999527751	ENSG00000073969	NSF
705.0732865	0.372015445	0.226826052	1.640091347	0.100986175	0.92169898	ENSG00000104133	SPG11
698.1567985	0.206007002	0.221612345	0.9295827	0.352587188	0.977691382	ENSG00000055332	EIF2AK2
692.8275721	0.164097564	0.357342033	0.459217076	0.646078289	0.997368416	ENSG00000189159	JPT1
700.2693948	-0.030521371	0.210702639	-0.14485519	0.884825206	0.999467288	ENSG00000073614	KDM5A
700.4600051	-0.015325814	0.231153163	-0.066301556	0.947137745	0.999527751	ENSG00000135241	PNPLA8
699.5111434	-0.068694057	0.197739777	-0.34739625	0.728293651	0.999467288	ENSG00000130764	LRRC47
696.8176556	0.09209118	0.209931849	0.438671791	0.66089937	0.997568624	ENSG00000138443	ABI2
701.8801378	-0.451982456	0.326476307	-1.384426515	0.166227895	0.943223078	ENSG00000165801	ARHGEF40
700.0843226	-0.208971034	0.23812407	-0.877572071	0.380175992	0.983585589	ENSG00000133103	COG6
700.1405007	-0.274847793	0.236869359	-1.160334938	0.245912465	0.962465655	ENSG00000145337	PYURF
705.7826044	0.306954463	0.296932832	1.033750497	0.30125282	0.972793733	ENSG00000033178	UBA6
692.0909552	-0.147296244	0.256194835	-0.574938381	0.565332971	0.99483168	ENSG00000147123	NDUFB11
694.8472949	-0.172084856	0.205777134	-0.836268113	0.403004088	0.988927059	ENSG00000188677	PARVB
693.5564484	0.333845586	0.352485272	0.947119249	0.343578017	0.977691382	ENSG00000115355	CCDC88A
698.4897197	-0.207795372	0.210151698	-0.988787504	0.322767119	0.975337095	ENSG00000175662	TOM1L2
692.7325822	-0.130922172	0.335336787	-0.39041995	0.696226038	0.998869317	ENSG00000104320	NBN
695.1093329	-0.170003014	0.200104626	-0.849570635	0.395563844	0.988378925	ENSG00000136143	SUCLA2
691.5077788	-0.144884513	0.311455011	-0.465186007	0.641798259	0.996696873	ENSG00000095739	BAMBI
705.2759927	0.037231479	0.28351136	0.131322705	0.895520032	0.999467288	ENSG00000120053	GOT1
688.5763201	-0.309597795	0.244894278	-1.264209997	0.206154664	0.954310328	ENSG00000129245	FXR2
695.2832985	0.08495124	0.227086641	0.374091756	0.708336052	0.99900742	ENSG00000114098	ARMC8
698.7502716	0.239386392	0.274168047	0.873137457	0.382588159	0.98555814	ENSG00000205060	SLC35B4
696.4524075	0.286612021	0.20512246	1.397272734	0.162331572	0.943223078	ENSG00000123908	AGO2
695.736766	0.200244062	0.201998483	0.991314678	0.321531948	0.975337095	ENSG00000062194	GPBP1
693.5260402	-0.010220183	0.206912789	-0.049393677	0.960605567	0.999527751	ENSG00000112237	CCNC
695.0036996	0.190319713	0.258867243	0.735201993	0.462216485	0.99135833	ENSG00000197535	MYO5A
694.1163831	0.368496889	0.239752509	1.536988662	0.124296086	0.927794614	ENSG00000109738	GLRB
690.0455687	-0.404844061	0.220123868	-1.839164765	0.065890955	0.872086825	ENSG00000103005	USB1
690.8799054	-0.091485702	0.204254521	-0.4479005	0.654225008	0.997568624	ENSG00000113068	PFDN1
694.3683896	0.090404799	0.208044221	0.434546072	0.663891946	0.997568624	ENSG00000089006	SNX5
690.1380576	-0.215657128	0.211829909	-1.018067416	0.308645919	0.973427483	ENSG00000114316	USP4
691.6370289	-0.060207336	0.245065928	-0.245678119	0.805931412	0.999467288	ENSG00000105197	TIMM50
686.5183619	-0.071692543	0.243103828	-0.294905037	0.768066424	0.999467288	ENSG00000171307	ZDHHC16
665.2800573	0.106068856	0.45301267	0.234141036	0.814875489	0.999467288	ENSG00000104936	DMPK
698.4312709	-0.069194565	0.232865286	-0.297144184	0.766356433	0.999467288	ENSG00000147044	CASK
695.4388206	-0.101409949	0.277574665	-0.365342959	0.714855415	0.99900742	ENSG00000092096	SLC22A17
691.3847122	-0.190232439	0.199781138	-0.952204205	0.340993429	0.977691382	ENSG00000122550	KLHL7
696.0306309	-0.388754311	0.293010604	-1.326758507	0.18458858	0.949094286	ENSG00000105245	NUMBL
695.8785146	0.30248469	0.292064059	1.035679265	0.300351799	0.972186492	ENSG00000153956	CACNA2D1
689.0771475	0.026661344	0.210686819	0.126544906	0.899300605	0.999527751	ENSG00000164024	METAP1
687.5852185	0.018475367	0.240416863	0.076847217	0.938745088	0.999527751	ENSG00000198171	DDRGK1
696.9762025	0.258252075	0.302230402	0.854487415	0.392834979	0.988378925	ENSG00000121578	B4GALT4
694.9216725	0.037966237	0.212246672	0.178877891	0.858033583	0.999467288	ENSG00000165219	GAPVD1
689.5154446	-0.051823862	0.21791474	-0.237817148	0.812022918	0.999467288	ENSG00000185504	FAAP100
689.7457936	-0.170851361	0.239609085	-0.713042084	0.475819716	0.99135833	ENSG00000102119	EMD
687.7496235	-0.892741328	0.256137635	-3.485396932	0.000491407	0.1830637	ENSG00000204103	MAFB
690.2380336	-0.23179188	0.251917604	-0.920109894	0.357515335	0.979427314	ENSG00000141510	TP53
693.5212705	0.091026717	0.222820825	0.408519794	0.682892101	0.998869317	ENSG00000144747	TMF1
688.5130453	0.031570516	0.234557553	0.134596032	0.892931279	0.999467288	ENSG00000144744	UBA3
692.3824073	-0.101277844	0.210734446	-0.480594633	0.630804631	0.99483168	ENSG00000163939	PBRM1
688.8906419	0.111720284	0.28726273	0.388913257	0.697340315	0.998869317	ENSG00000102241	HTATSF1
682.9004006	-0.363996801	0.270933241	-1.343492588	0.179112525	0.9469453	ENSG00000148218	ALAD
679.5562809	0.324252294	0.493951349	0.656445812	0.511537322	0.993513219	ENSG00000137563	GGH
679.3003698	-0.206511266	0.327284875	-0.630983227	0.528051492	0.99483168	ENSG00000115756	HPCAL1
685.2569126	-0.303600446	0.19904135	-1.525313441	0.127180937	0.928627271	ENSG00000023330	ALAS1
686.385513	-0.436514032	0.196496436	-2.221485752	0.026318079	0.763890866	ENSG00000102978	POLR2C
689.6571854	0.020876801	0.223526095	0.0933976	0.925587697	0.999527751	ENSG00000076201	PTPN23
699.9779966	0.250173423	0.34299279	0.729383912	0.465766856	0.99135833	ENSG00000272391	POM121C
684.3845934	0.138501066	0.26572777	0.521214122	0.602217618	0.99483168	ENSG00000134697	GNL2
686.1194432	-0.113628946	0.278873083	-0.407457559	0.683671959	0.998869317	ENSG00000108604	SMARCD2
691.2128995	0.112596114	0.237090054	0.474908634	0.634852097	0.995506752	ENSG00000198586	TLK1
685.9073491	-0.187277665	0.26773376	-0.699492155	0.484244514	0.991892545	ENSG00000114796	KLHL24

687.5358327	-0.102776156	0.209765289	-0.489957882	0.624163703	0.99483168	ENSG00000140750	ARHGAP17
679.5885309	-0.089551626	0.334341431	-0.267844836	0.788818757	0.999467288	ENSG00000175573	C11orf68
693.6022212	-0.417110471	0.253775722	-1.643618499	0.10025503	0.92169898	ENSG00000183386	FHL3
688.1117809	0.198504268	0.205339673	0.96671172	0.333688166	0.976026345	ENSG00000120733	KDM3B
693.5124432	-0.020184468	0.221518497	-0.091118657	0.927398308	0.999527751	ENSG00000100796	PPP4R3A
680.8087122	-0.136025513	0.217893394	-0.624275527	0.532446653	0.99483168	ENSG00000004478	FKBP4
685.2718807	0.1176153	0.215944237	0.544655887	0.58599021	0.99483168	ENSG00000144848	ATG3
677.9256852	-0.159714737	0.252474519	-0.632597451	0.526996552	0.99483168	ENSG00000166595	CIAO2B
690.6485705	0.116493506	0.209394241	0.556335769	0.577981339	0.99483168	ENSG00000036549	AC118549.1
692.6114069	-0.239296089	0.420597692	-0.568942942	0.569394862	0.99483168	ENSG00000168077	SCARA3
693.2365903	0.259208675	0.272112073	0.952580577	0.340802622	0.977691382	ENSG00000167202	TBC1D2B
685.5226394	0.018365503	0.224427771	0.081832579	0.934779849	0.999527751	ENSG00000112276	BVES
687.3866636	0.195967133	0.365340081	0.53639648	0.591684564	0.99483168	ENSG00000147100	SLC16A2
677.5652426	-0.262803201	0.255968442	-1.026701571	0.304561009	0.973427483	ENSG00000012061	ERCC1
679.7430177	0.814976318	0.258166998	3.156779618	0.001595219	0.287996778	ENSG00000106546	AHR
679.8475258	0.320309141	0.319301629	1.003155362	0.315785907	0.974768742	ENSG00000143401	ANP32E
689.4978014	0.299817321	0.254232419	1.17930405	0.238277126	0.962465655	ENSG00000180488	MIGA1
668.8547483	-0.04443958	0.536612375	-0.082815049	0.933998603	0.999527751	ENSG00000109107	ALDOC
682.677507	0.029241361	0.204297387	0.143131351	0.886186446	0.999467288	ENSG00000198015	MRPL42
685.570483	0.023355982	0.223902064	0.104313382	0.916920658	0.999527751	ENSG00000166197	NOLC1
681.8010349	0.097389451	0.187624039	0.519067018	0.603714007	0.99483168	ENSG00000176783	RUFY1
688.748451	-0.122231181	0.248601163	-0.49167582	0.622948534	0.99483168	ENSG00000188647	PTAR1
677.3999488	0.430628034	0.977082926	0.440728235	0.659409753	0.997568624	ENSG00000136999	NOV
684.8500278	-0.377614781	0.256850268	-1.470174761	0.141514428	0.935644597	ENSG00000126903	SLC10A3
683.3160479	0.181400246	0.214335836	0.846336525	0.397365038	0.988378925	ENSG00000163655	GMPS
687.8367139	-0.331462217	0.240784071	-1.376595285	0.16863741	0.943311428	ENSG00000198876	DCAF12
689.2996201	-0.082617798	0.226236418	-0.365183459	0.714974465	0.99900742	ENSG00000100697	DICER1
691.9327882	-0.291217877	0.231962428	-1.255452789	0.209314436	0.954310328	ENSG00000137492	THAP12
685.6751267	0.012375137	0.234978743	0.052664922	0.957998888	0.999527751	ENSG00000116539	ASH1L
688.6247837	0.349797446	0.247199846	1.415039098	0.157057033	0.939464191	ENSG00000196367	TRRAP
684.1657576	0.331182492	0.264408765	1.252539765	0.210373248	0.954310328	ENSG00000149639	SOGA1
682.1820709	0.031265098	0.201526483	0.155141388	0.876709855	0.999467288	ENSG00000188976	NOC2L
683.3781599	-0.077613386	0.217545409	-0.356768669	0.721264994	0.99900742	ENSG00000149532	CPSF7
683.2250192	-0.170195913	0.206682565	-0.823465263	0.410243463	0.990226751	ENSG00000129691	ASH2L
682.8205657	0.304320223	0.208169339	1.461887832	0.14377195	0.935644597	ENSG00000164190	NIPBL
669.0688261	0.041195847	0.398159387	0.103465718	0.917593356	0.999527751	ENSG00000272398	CD24
675.4807811	0.156281633	0.361964604	0.431759435	0.665916265	0.997568624	ENSG00000079819	EPB41L2
677.1333528	-0.163513801	0.231602818	-0.706009549	0.480182199	0.99135833	ENSG00000123472	ATPAF1
682.5370985	-0.03727668	0.270534612	-0.137788948	0.890407219	0.999467288	ENSG00000151458	ANKRD50
683.5461681	-0.076111703	0.211605841	-0.35968621	0.719081806	0.99900742	ENSG00000143368	SF3B4
676.5248072	-0.875279592	0.34510201	-2.53629236	0.011203316	0.644117389	ENSG00000203805	PLPP4
674.5046704	-0.096394461	0.215674659	-0.446943844	0.654915606	0.997568624	ENSG00000154813	DPH3
675.8360089	0.035175114	0.306702115	0.114688201	0.908692266	0.999527751	ENSG00000143799	PARP1
718.4834019	-1.077477957	0.356016383	-3.026484195	0.002474158	0.360084787	ENSG00000163520	FBLN2
690.4133204	-0.218152123	0.359625322	-0.606609462	0.544110119	0.99483168	ENSG00000076356	PLXNA2
678.3523297	0.028994893	0.217745677	0.133159441	0.894067287	0.999467288	ENSG00000043093	DCUN1D1
678.746256	-0.277528913	0.218686936	-1.269069464	0.20441629	0.954310328	ENSG00000132612	VPS4A
673.376907	-0.089938811	0.278489217	-0.322952581	0.746731154	0.999467288	ENSG00000164904	ALDH7A1
678.6177184	-0.047582399	0.228184051	-0.208526402	0.83481797	0.999467288	ENSG00000225178	RPSAP58
681.5083641	-0.041566752	0.238695251	-0.174141511	0.861754251	0.999467288	ENSG00000122741	DCAF10
676.0680774	0.239081868	0.294576957	0.811610897	0.417014937	0.990226751	ENSG00000113161	HMGCR
668.8679813	0.086583284	0.370435685	0.233733648	0.815191764	0.999467288	ENSG00000067064	IDI1
680.5070899	-0.405189597	0.263143215	-1.539806365	0.12360756	0.927794614	ENSG00000198909	MAP3K3
678.4989161	-0.074221389	0.228075247	-0.325425008	0.744859431	0.999467288	ENSG00000044090	CUL7
682.6703144	0.128467104	0.240165243	0.534911308	0.592711189	0.99483168	ENSG00000148468	FAM171A1
671.5377564	0.697194611	0.440841878	1.581507216	0.11376211	0.923922314	ENSG00000112562	SMOC2
678.927028	0.164234033	0.217556347	0.75490343	0.450306927	0.99135833	ENSG00000159131	GART
680.736037	-0.178652052	0.214694407	-0.832122527	0.405339788	0.990045328	ENSG00000103479	RBL2
683.4923477	-0.41624127	0.305654751	-1.361802063	0.17326036	0.944422693	ENSG00000164916	FOXK1
674.9229453	0.097280448	0.249576511	0.389782067	0.696697706	0.998869317	ENSG00000161813	LARP4
683.5690979	0.111844421	0.260153742	0.429916633	0.667256286	0.997568624	ENSG00000124222	STX16
674.9594706	-0.219626799	0.210941175	-1.041175571	0.297794071	0.972186492	ENSG00000118900	UBN1
682.1245141	-0.178702502	0.338100329	-0.528548737	0.59711853	0.99483168	ENSG00000181588	MEX3D
675.6169946	-0.185353722	0.193919148	-0.955829909	0.339158175	0.977691382	ENSG00000104442	ARMC1

678.3956224	0.348287649	0.23366231	1.490559814	0.136077102	0.934144427	ENSG00000171634	BPTF
675.0860165	0.327293833	0.201942454	1.620728217	0.105075939	0.92169898	ENSG00000143653	SCCPDH
676.0270949	-0.090250743	0.224316066	-0.402337401	0.687435733	0.998869317	ENSG00000198648	STK39
674.2864936	-0.149929787	0.362054382	-0.414108473	0.678794668	0.998869317	ENSG00000225614	ZNF469
674.1483434	-0.185877746	0.254774226	-0.729578299	0.465647991	0.99135833	ENSG00000154945	ANKRD40
676.6747554	0.274225074	0.194344845	1.411023143	0.15823779	0.939464191	ENSG00000065243	PKN2
677.1938061	1.00618302	0.298192157	3.374277275	0.000740098	0.201683631	ENSG00000143322	ABL2
675.8221007	-0.14675785	0.249726629	-0.587674013	0.556751122	0.99483168	ENSG00000141447	OSBPL1A
672.6604061	-0.504462582	0.395263797	-1.276268117	0.201860757	0.954310328	ENSG00000171051	FPR1
677.7447275	0.044084272	0.256697959	0.171735967	0.863645108	0.999467288	ENSG00000067334	DNTTIP2
667.4434559	-0.190286655	0.246656142	-0.771465301	0.440431182	0.99135833	ENSG00000164258	NDUFS4
679.5528553	-0.348553587	0.560526591	-0.621832385	0.534052089	0.99483168	ENSG00000104435	STMN2
675.413948	0.134798103	0.3867775	0.348515886	0.727452785	0.999467288	ENSG00000173210	ABLIM3
677.8101253	-0.280341732	0.215252905	-1.302383035	0.192785479	0.949321643	ENSG00000137815	RTF1
674.2824006	-0.055568949	0.186571335	-0.29784291	0.765823063	0.999467288	ENSG00000066654	THUMPD1
676.12508	-0.283895835	0.228717535	-1.241250849	0.214513096	0.954310328	ENSG00000100461	RBM23
677.9873924	0.146065348	0.284295971	0.513779169	0.607406394	0.99483168	ENSG00000089876	DHX32
680.0979547	-0.026230044	0.257744402	-0.101767657	0.918941098	0.999527751	ENSG00000085982	USP40
671.960344	-0.020260663	0.253642412	-0.079878847	0.936333614	0.999527751	ENSG00000133703	KRAS
673.6165411	-0.111809855	0.217782111	-0.513402384	0.607669878	0.99483168	ENSG00000100485	SOS2
670.5169375	0.246165015	0.215161248	1.144095496	0.252584035	0.962465655	ENSG00000162613	FUBP1
670.7355215	-0.013997497	0.288863825	-0.048457079	0.96135197	0.999527751	ENSG00000135823	STX6
672.6389198	0.091248677	0.245997546	0.37093328	0.710687227	0.99900742	ENSG00000004700	RECQL
674.7507923	-0.201994186	0.301860251	-0.669164574	0.503390502	0.993513219	ENSG00000171475	WIPF2
664.8175758	0.163479335	0.283888368	0.575857813	0.564711292	0.99483168	ENSG00000106780	MEGF9
664.6512749	-1.15077163	0.338180035	-3.402837275	0.0006669	0.195790358	ENSG00000149596	JPH2
686.7425389	-0.267477758	0.340522692	-0.78549173	0.432165317	0.991301167	ENSG00000102595	UGGT2
675.2308497	0.166646577	0.244905636	0.68045219	0.496218184	0.993513219	ENSG00000060339	CCAR1
666.1153179	-0.099110711	0.220845421	-0.448778657	0.653591337	0.997527562	ENSG00000160410	SHKBP1
667.2573372	0.023160871	0.20988844	0.110348483	0.912133008	0.999527751	ENSG00000168883	USP39
677.540321	-0.095677406	0.272719479	-0.350827181	0.725718005	0.999467288	ENSG00000105696	TMEM59L
668.0341811	-0.287890772	0.247207659	-1.1645706	0.24419285	0.962465655	ENSG00000131943	C19orf12
666.6512337	0.192579266	0.248393653	0.775298661	0.438163201	0.991301167	ENSG00000090686	USP48
669.7045163	2.203397312	1.373876055	1.603781727	0.108762174	0.923922314	ENSG00000011426	ANLN
666.8028223	0.397208421	0.219627054	1.808558706	0.070519589	0.87954613	ENSG00000007384	RHBDF1
666.0632067	0.535904449	0.281675776	1.902557813	0.057098268	0.852306368	ENSG00000115652	UXS1
671.9130531	0.2720438	0.290074263	0.937841908	0.348325661	0.977691382	ENSG00000143514	TP53BP2
676.0936045	-0.185866147	0.246611818	-0.753678994	0.451041998	0.99135833	ENSG00000102226	USP11
669.466147	0.111519953	0.249158587	0.447586232	0.654451841	0.997568624	ENSG00000183955	KMT5A
668.9416159	-0.017113604	0.234165861	-0.073083259	0.941739864	0.999527751	ENSG00000151465	CDC123
658.4029375	-0.486751182	0.313292583	-1.55366328	0.120264756	0.923922314	ENSG00000126005	MMP24OS
685.3243034	-0.695609739	0.496749694	-1.400322432	0.161416786	0.942964826	ENSG00000060656	PTPRU
669.5713909	-0.234113075	0.252844979	-0.925915463	0.354489915	0.977691382	ENSG00000136720	HS6ST1
664.2115531	-0.528725332	0.33923034	-1.558602724	0.119090438	0.923922314	ENSG00000160685	ZBTB7B
669.0277891	-0.361794697	0.218282481	-1.657460994	0.097426309	0.921147998	ENSG00000113391	FAM172A
694.8944853	0.653134658	1.017872647	0.641666381	0.521089822	0.993513219	ENSG00000135842	FAM129A
664.5043282	0.132423253	0.200252898	0.661280085	0.50843271	0.993513219	ENSG00000101158	NELFCD
664.0312048	-0.030458226	0.211833417	-0.143783857	0.88567115	0.999467288	ENSG00000249915	PDCD6
662.9995267	-0.755567343	0.293556377	-2.57384067	0.010057658	0.630908184	ENSG00000146540	C7orf50
654.1562568	0.154768764	0.331009004	0.467566627	0.640094535	0.996553586	ENSG00000170955	CAVIN3
674.0806822	-0.176763851	0.398698355	-0.443352347	0.657510898	0.997568624	ENSG00000181788	SIAH2
662.0696757	-0.112613364	0.259686398	-0.433651377	0.66454162	0.997568624	ENSG00000136819	C9orf78
667.1880437	-0.296238402	0.212250793	-1.39569986	0.162804896	0.943223078	ENSG00000176022	B3GALT6
662.9190767	0.052851235	0.23971441	0.220475835	0.82550059	0.999467288	ENSG00000164081	TEX264
662.4197455	0.759724249	0.290791565	2.612607582	0.008985441	0.611285922	ENSG00000165124	SVEP1
667.1204255	-0.098983326	0.19498603	-0.507643168	0.611703611	0.99483168	ENSG00000111605	CPSF6
662.4197275	0.193566575	0.219877046	0.880340076	0.378675109	0.982609538	ENSG00000132485	ZRANB2
662.6735268	0.03408484	0.234848784	0.145135263	0.884604077	0.999467288	ENSG00000108509	CAMTA2
661.4990089	0.049503264	0.213670525	0.231680358	0.816786288	0.999467288	ENSG00000163950	SLBP
667.945307	0.009732252	0.282609524	0.034437099	0.9725286	0.999527751	ENSG00000152620	NADK2
661.1715693	-0.046907199	0.219911255	-0.213300584	0.831092518	0.999467288	ENSG00000057663	ATG5
657.6879211	-0.228368929	0.21202645	-1.077077551	0.281445621	0.963267117	ENSG00000162735	PEX19
665.5521659	-0.348690669	0.208223515	-1.674597943	0.094013143	0.921147998	ENSG00000263001	GTF2I
666.5058342	-0.130875166	0.203329153	-0.643661589	0.519794903	0.993513219	ENSG00000071189	SNX13

662.0020704	0.166377242	0.238832236	0.696628078	0.486035579	0.993041714	ENSG00000182979	MTA1
663.0000945	-0.167334638	0.206943589	-0.80860025	0.418745121	0.990226751	ENSG00000149182	ARFGAP2
678.1326318	0.577330821	0.499091279	1.156763994	0.247368796	0.962465655	ENSG00000117525	F3
650.5256893	0.003331352	0.361158972	0.00922406	0.992640369	0.999527751	ENSG00000155368	DBI
661.4158727	0.016837645	0.220588301	0.076330635	0.939156054	0.999527751	ENSG00000183864	TOB2
662.6774575	-0.209157956	0.237196278	-0.881792741	0.377888898	0.982609538	ENSG00000163558	PRKCI
662.7758169	-0.341032953	0.240533448	-1.417819249	0.156243543	0.939464191	ENSG00000125952	MAX
666.7769928	0.030347222	0.229428018	0.13227339	0.894768054	0.999467288	ENSG00000107625	DDX50
658.2219794	0.048888809	0.229097009	0.213397848	0.831016659	0.999467288	ENSG00000273841	TAF9
660.0286408	0.191566419	0.316605737	0.605063005	0.545137134	0.99483168	ENSG00000139263	LRIG3
654.064444	-0.431794037	0.245480536	-1.75897464	0.078581816	0.897052166	ENSG00000119421	NDUFA8
660.2884544	0.045082015	0.199971111	0.22544264	0.821634943	0.999467288	ENSG00000168259	DNAJC7
659.5894312	-0.114277117	0.243931782	-0.468479819	0.6394415	0.996553586	ENSG00000183431	SF3A3
663.2550241	0.214391747	0.235817106	0.90914417	0.36327403	0.98048242	ENSG00000108055	SMC3
658.1536231	-0.035380622	0.222833986	-0.158775698	0.873845596	0.999467288	ENSG00000198589	LRBA
658.2156678	-0.055051378	0.215118575	-0.255911782	0.798018957	0.999467288	ENSG00000114744	COMMD2
654.9825813	-0.126310658	0.27265807	-0.463256629	0.64318043	0.997097166	ENSG00000164346	NSA2
668.8369471	0.436233382	0.445791192	0.978559894	0.327797484	0.976026345	ENSG00000074527	NTN4
667.3515841	-0.085843564	0.275445495	-0.311653541	0.755303839	0.999467288	ENSG00000144233	AMMECR1L
661.6816186	0.062826965	0.277596614	0.22632468	0.820948905	0.999467288	ENSG00000133812	SBF2
652.3718075	0.042496114	0.23226534	0.182963649	0.854826541	0.999467288	ENSG00000165389	SPTSSA
659.2972787	0.046431862	0.237485085	0.195514856	0.844989908	0.999467288	ENSG00000119321	FKBP15
656.0379325	0.096383669	0.268185001	0.359392466	0.71930151	0.99900742	ENSG00000168591	TMUB2
657.4589685	0.144221077	0.207872531	0.693795743	0.487810312	0.993513219	ENSG00000160633	SAFB
657.4232883	-0.108637852	0.231250576	-0.469784135	0.638509251	0.996553586	ENSG00000198087	CD2AP
656.0853411	-0.291785929	0.216992249	-1.344683646	0.178727418	0.9469453	ENSG00000115520	COQ10B
649.087805	0.072798049	0.279369202	0.26058008	0.794416353	0.999467288	ENSG00000107104	KANK1
658.0428305	-0.25830485	0.21632317	-1.194069275	0.232450871	0.959575179	ENSG00000131899	LLGL1
661.5732051	0.26332179	0.245304734	1.073447653	0.283070314	0.963267117	ENSG00000173273	TNKS
661.2188661	0.129940397	0.308412373	0.421320311	0.673521201	0.99853019	ENSG00000166889	PATL1
650.2646347	-0.042395251	0.308034993	-0.137631284	0.89053183	0.999467288	ENSG00000144824	PHLDB2
658.6006094	-0.476030723	0.305891147	-1.556209549	0.119658272	0.923922314	ENSG00000118046	STK11
654.9145893	-0.055325777	0.271652624	-0.203663696	0.838616314	0.999467288	ENSG00000068120	COASY
654.8342256	0.09857343	0.266433001	0.369974551	0.711401453	0.99900742	ENSG00000137996	RTCA
658.082796	-0.282838387	0.254732382	-1.110335426	0.266854513	0.962465655	ENSG00000164823	OSGIN2
637.9370628	-0.293518101	0.416949886	-0.703964938	0.481454609	0.991525194	ENSG00000176485	PLA2G16
657.081067	0.075368007	0.202536291	0.372120998	0.709802763	0.99900742	ENSG00000083520	DIS3
654.3497792	-0.159181363	0.196803843	-0.808832595	0.418611445	0.990226751	ENSG00000101843	PSMD10
649.4250491	0.034777999	0.322915217	0.107700092	0.914233596	0.999527751	ENSG00000099337	KCNK6
654.5487768	-0.134106017	0.261131509	-0.513557392	0.607561476	0.99483168	ENSG00000215193	PEX26
655.4719668	-0.118034236	0.26606276	-0.443633057	0.657307902	0.997568624	ENSG00000169733	RFNG
648.7054849	-0.537451331	0.267738648	-2.007372992	0.044709962	0.812259102	ENSG00000099795	NDUFB7
654.4139727	0.668744549	0.303096553	2.206374642	0.027357779	0.768732301	ENSG00000121989	ACVR2A
654.9211204	0.13097329	0.214619925	0.610256903	0.541691641	0.99483168	ENSG00000163634	THOC7
655.2132901	0.316153679	0.21721328	1.455498849	0.145531209	0.935644597	ENSG00000121879	PIK3CA
650.7605069	0.346379543	0.490473477	0.706214626	0.480054676	0.99135833	ENSG00000164308	ERAP2
654.7175871	0.159884365	0.262023728	0.610190408	0.541735683	0.99483168	ENSG00000180182	MED14
661.0090144	-0.103412702	0.287461651	-0.359744339	0.719038331	0.99900742	ENSG00000099991	CABIN1
657.1767137	0.157125193	0.21867426	0.718535383	0.472427239	0.99135833	ENSG00000163162	RNF149
653.4209415	0.025431837	0.193506425	0.13142632	0.895438069	0.999467288	ENSG00000103051	COG4
652.0693197	-0.006710978	0.238729222	-0.028111253	0.977573419	0.999527751	ENSG00000177189	RPS6KA3
658.0208223	0.108175715	0.252974259	0.427615503	0.668931077	0.997568624	ENSG00000124786	SLC35B3
652.6867668	-0.12733392	0.193281922	-0.658798909	0.510024908	0.993513219	ENSG00000197265	GTF2E2
650.4018564	0.049493263	0.239159394	0.206946767	0.836051432	0.999467288	ENSG00000103966	EHD4
650.6677389	0.087699471	0.224039102	0.391447163	0.695466737	0.998869317	ENSG00000131748	STARD3
652.9739174	-0.340711293	0.22042234	-1.545720332	0.122172125	0.923922314	ENSG00000100348	TXN2
653.8607946	-0.280769793	0.217600614	-1.290298716	0.196946963	0.954310328	ENSG00000111737	RAB35
648.2795632	-0.09042966	0.26555973	-0.340524749	0.733461388	0.999467288	ENSG00000008283	CYB561
649.6226038	0.326610495	0.382683058	0.853475189	0.393395841	0.988378925	ENSG00000102312	PORCN
656.2330566	-0.115785492	0.282269426	-0.410194947	0.681662947	0.998869317	ENSG00000196588	MRTFA
647.0521208	-0.312436758	0.248277978	-1.258415106	0.208241672	0.954310328	ENSG00000100938	GMPR2
652.3371718	0.27783226	0.198601996	1.39893992	0.161831	0.943223078	ENSG00000205765	C5orf51
647.5778243	0.014969539	0.252597277	0.059262472	0.952743051	0.999527751	ENSG00000103653	CSK
658.3436147	-0.138865539	0.311114061	-0.446349287	0.655344959	0.997568624	ENSG00000281649	EBLN3P

651.7304721	-0.269794347	0.214147816	-1.25985103	0.207723107	0.954310328	ENSG00000164305	CASP3
656.699046	-0.335805357	0.238230103	-1.409584064	0.158662535	0.939464191	ENSG00000052723	SIKE1
645.7090699	-0.010147717	0.245797433	-0.041284878	0.967068788	0.999527751	ENSG00000131475	VPS25
646.1598832	-0.158037365	0.258895983	-0.610428031	0.541578304	0.99483168	ENSG00000147400	CETN2
652.0342444	-0.0617651	0.204756113	-0.301652042	0.762917331	0.999467288	ENSG00000175221	MED16
652.9046768	-0.159882306	0.271633498	-0.588595688	0.556132527	0.99483168	ENSG00000072840	EVC
653.7253864	0.20912459	0.252356678	0.82868657	0.407281787	0.990226751	ENSG00000146802	TMEM168
637.5288555	-0.231898455	0.298475786	-0.77694227	0.437192831	0.991301167	ENSG00000110711	AIP
650.5824348	0.163283114	0.200463693	0.814527119	0.415343043	0.990226751	ENSG00000156171	DRAM2
634.892596	0.81109427	0.437746609	1.852885328	0.063898797	0.868202292	ENSG00000138131	LOXL4
642.5548797	-0.369859277	0.255786919	-1.445966351	0.14818664	0.936396974	ENSG00000175756	AURKAIP1
641.255529	-0.24651999	0.274361245	-0.898523368	0.368906593	0.981455968	ENSG00000158828	PINK1
648.7045446	0.190726217	0.248882164	0.766331398	0.44347914	0.99135833	ENSG00000131778	CHD1L
654.3903062	-0.330342634	0.262370587	-1.259068853	0.208005463	0.954310328	ENSG00000100335	MIEF1
650.0498496	0.003813642	0.200831413	0.018989269	0.984849666	0.999527751	ENSG00000178950	GAK
646.7376541	0.017636214	0.248893707	0.070858418	0.943510438	0.999527751	ENSG00000124562	SNRPC
653.442232	0.186893529	0.26708381	0.699756114	0.484079626	0.991892545	ENSG00000121073	SLC35B1
646.544011	-0.274117538	0.252154899	-1.087099791	0.27699271	0.962465655	ENSG00000180818	HOXC10
650.0411734	0.097170248	0.221116208	0.439453302	0.660333112	0.997568624	ENSG00000119760	SUPT7L
647.8836494	0.000751405	0.217519011	0.003454433	0.997243767	0.999527751	ENSG00000135655	USP15
644.9051656	-0.313453518	0.220806233	-1.419586367	0.155728137	0.939464191	ENSG00000127922	SEM1
648.9214253	0.213607963	0.25324185	0.843493931	0.398952258	0.988378925	ENSG00000067248	DHX29
646.4001088	-0.332778704	0.223116565	-1.491501555	0.135829863	0.934144427	ENSG00000162191	UBXN1
655.6436052	-0.533931304	0.26323112	-2.028374546	0.042522036	0.806698895	ENSG00000156011	PSD3
645.8092052	-0.308795792	0.219927924	-1.404077241	0.16029585	0.941607907	ENSG00000070010	UFD1
652.6580425	0.168397392	0.247859665	0.679406198	0.496880524	0.993513219	ENSG00000048707	VPS13D
649.0022709	0.108456632	0.246799823	0.439451823	0.660334183	0.997568624	ENSG00000168827	GFM1
643.9012315	-0.065105938	0.220952989	-0.294659685	0.768253863	0.999467288	ENSG00000088833	NSFL1C
628.8012891	-0.118155851	0.516409298	-0.228802718	0.819022254	0.999467288	ENSG00000143196	DPT
645.0845069	0.244244705	0.212725792	1.148166866	0.250899685	0.962465655	ENSG00000099290	WASHC2A
644.794514	-0.164891848	0.206900073	-0.796963703	0.425472112	0.991301167	ENSG00000143889	HNRNPLL
640.4908657	-0.484294027	0.293365056	-1.650823832	0.098774552	0.921147998	ENSG00000005238	FAM214B
626.4513336	0.555852171	0.385670827	1.44126061	0.149511071	0.936396974	ENSG00000100075	SLC25A1
648.5113265	-0.083116861	0.214054386	-0.388297863	0.697795619	0.998869317	ENSG00000133313	CNDP2
645.5971666	0.097983326	0.2548627	0.384455341	0.70064099	0.998869317	ENSG00000178927	CYBC1
642.4365253	-0.09369444	0.23986397	-0.390614896	0.696081913	0.998869317	ENSG00000155959	VBP1
648.8479399	0.175500182	0.237323463	0.739497815	0.459604766	0.99135833	ENSG00000118482	PHF3
639.8140805	-0.48901694	0.280827493	-1.741342829	0.081623503	0.897807954	ENSG00000143862	ARL8A
638.3151035	-0.152828794	0.265822511	-0.574927958	0.56534002	0.99483168	ENSG00000120509	PDZD11
643.8959095	0.058183622	0.26251979	0.221635185	0.82459789	0.999467288	ENSG00000116161	CACYBP
641.0796582	-0.005831491	0.232668669	-0.0250635	0.980004314	0.999527751	ENSG00000185721	DRG1
642.7526905	-0.037899105	0.206097684	-0.183889038	0.854100504	0.999467288	ENSG00000137055	PLAA
643.1008549	0.04370696	0.245289304	0.178185348	0.858577416	0.999467288	ENSG00000136560	TANK
650.8797515	-0.374888846	0.425967047	-0.880088843	0.378811183	0.982609538	ENSG00000166886	NAB2
649.4343128	-0.361536796	0.267445701	-1.351813825	0.17643488	0.946278819	ENSG00000167977	KCTD5
651.069539	0.154609295	0.267792694	0.577346951	0.563705103	0.99483168	ENSG00000170881	RNF139
638.5955217	0.167658531	0.327802194	0.511462504	0.609027237	0.99483168	ENSG00000149541	B3GAT3
646.7231449	-0.044296391	0.279767267	-0.158333	0.874194406	0.999467288	ENSG00000168300	PCMTD1
644.4114151	0.082725359	0.213025384	0.388335685	0.697767633	0.998869317	ENSG00000164144	ARFIP1
644.224868	-0.006286983	0.223125329	-0.028176914	0.97752105	0.999527751	ENSG00000164597	COG5
640.4343299	-0.147211977	0.21193722	-0.694601811	0.487304877	0.993513219	ENSG00000097021	ACOT7
642.3983668	0.346537248	0.237903999	1.456626413	0.145219532	0.935644597	ENSG00000076108	BAZ2A
641.5512119	0.025405187	0.369180938	0.068815001	0.945136877	0.999527751	ENSG00000042286	AIFM2
635.2984062	-0.246758108	0.23122566	-1.067174414	0.285893084	0.964826902	ENSG00000106665	CLIP2
660.5743645	0.676496441	0.917816263	0.737071752	0.461078716	0.99135833	ENSG00000128165	ADM2
640.088642	-0.276754962	0.248249137	-1.114827488	0.264924355	0.962465655	ENSG00000204227	RING1
633.1292493	-0.00183159	0.305849542	-0.005988533	0.99522187	0.999527751	ENSG00000175602	CCDC85B
642.0509694	-0.04705961	0.227990451	-0.206410441	0.836470316	0.999467288	ENSG00000181830	SLC35C1
635.0862685	0.033946643	0.25321182	0.134064213	0.893351798	0.999467288	ENSG00000125835	SNRPB
638.3438097	0.058060521	0.226345733	0.256512551	0.79755509	0.999467288	ENSG00000113048	MRPS27
637.7348137	-0.128343022	0.291374079	-0.440475084	0.659593054	0.997568624	ENSG00000104325	DECR1
637.1889496	-0.059665215	0.20670781	-0.28864519	0.77285291	0.999467288	ENSG00000111361	EIF2B1
631.8597215	0.13090486	0.32001958	0.40905266	0.682501016	0.998869317	ENSG00000144677	CTDSPL
642.8763578	0.085809267	0.217329927	0.394834105	0.692965324	0.998869317	ENSG00000139697	SBNO1

635.6028624	-0.046106482	0.207903501	-0.221768663	0.824493975	0.999467288	ENSG00000137161	CNPY3
642.9375619	-0.286403976	0.330829452	-0.865714869	0.386646544	0.986711841	ENSG00000119669	IRF2BPL
634.2977422	-0.091047702	0.220821333	-0.412313887	0.680109371	0.998869317	ENSG00000134825	TMEM258
641.5707116	-0.160100214	0.202177191	-0.791880691	0.428430247	0.991301167	ENSG00000119616	FCF1
647.3155298	-0.028706412	0.38648124	-0.074276339	0.940790503	0.999527751	ENSG00000136869	TLR4
640.2884759	-0.113734366	0.233598404	-0.486879892	0.626343449	0.99483168	ENSG00000143155	TIPRL
635.7923756	-0.152838402	0.225434511	-0.677972515	0.49778912	0.993513219	ENSG00000100304	TTLL12
638.2876293	0.116698107	0.247692261	0.471141514	0.637539681	0.99653524	ENSG00000087087	SRRT
631.2233119	0.042960826	0.282535057	0.152054852	0.879143673	0.999467288	ENSG00000116791	CRYZ
645.2352822	-0.035206255	0.271417637	-0.12971248	0.896793909	0.999467288	ENSG00000159267	HLCS
642.7601767	0.029247461	0.31801101	0.091969964	0.926721904	0.999527751	ENSG00000116704	SLC35D1
637.948253	0.206433729	0.213791113	0.965586108	0.334251329	0.976026345	ENSG00000083896	YTHDC1
636.0141852	0.42632637	0.309637249	1.376857502	0.168556309	0.943223078	ENSG00000244405	ETV5
646.7562164	0.374481875	0.296743164	1.261973047	0.206958485	0.954310328	ENSG00000080345	RIF1
630.3751331	-0.095969758	0.259406838	-0.369958476	0.71141343	0.99900742	ENSG00000198722	UNC13B
635.6969034	-0.064035517	0.270098205	-0.237082349	0.812592905	0.999467288	ENSG00000171720	HDAC3
631.3102632	-0.603660819	0.27825427	-2.169457523	0.030047966	0.773201806	ENSG00000099624	ATP5F1D
634.710227	0.202508739	0.394071431	0.513888405	0.607330014	0.99483168	ENSG00000001084	GCLC
636.2933027	0.059399377	0.205422249	0.289157465	0.77246088	0.999467288	ENSG00000185127	C6orf120
637.0096753	-0.497696955	0.282372255	-1.762556153	0.077975378	0.897052166	ENSG00000162236	STX5
637.0443257	-0.163056919	0.220984191	-0.737866895	0.460595338	0.99135833	ENSG00000100014	SPECC1L
642.8086544	0.303014271	0.313076705	0.967859525	0.333114531	0.976026345	ENSG00000166471	TMEM41B
639.1504539	0.28936891	0.234964823	1.231541413	0.218120438	0.956884001	ENSG00000144468	RHBDD1
641.3947249	0.153569358	0.255206336	0.601745867	0.547343314	0.99483168	ENSG00000164187	LMBRD2
646.861916	0.399896661	0.88074741	0.454042392	0.649798315	0.997381078	ENSG00000136826	KLF4
623.463079	0.116999692	0.293879791	0.398120918	0.690541056	0.998869317	ENSG00000104884	ERCC2
629.9296866	0.022068315	0.311182273	0.070917649	0.943463297	0.999527751	ENSG00000156110	ADK
629.6080471	0.077096906	0.225996507	0.341142025	0.732996665	0.999467288	ENSG00000137824	RMDN3
631.5811523	-0.260696009	0.228532056	-1.140741538	0.253977495	0.962465655	ENSG00000146282	RARS2
639.2067795	0.029757636	0.267171013	0.111380479	0.911314639	0.999527751	ENSG00000166170	BAG5
637.3172999	0.092184281	0.214485454	0.429792693	0.667346449	0.997568624	ENSG00000170852	KBTBD2
639.0419363	0.182820369	0.283423292	0.645043559	0.518898958	0.993513219	ENSG00000151694	ADAM17
630.5322931	-0.011747277	0.217895053	-0.053912547	0.95700484	0.999527751	ENSG00000074201	CLNS1A
634.0549665	-0.06752909	0.264514434	-0.255294536	0.79849562	0.999467288	ENSG00000111961	SASH1
635.3363492	-0.147264661	0.19178047	-0.767881427	0.442557633	0.99135833	ENSG00000160767	FAM189B
634.904393	-0.266605893	0.201459869	-1.323369736	0.185712451	0.949094286	ENSG00000011114	BTBD7
634.7016511	0.042680506	0.221103571	0.193033996	0.846932344	0.999467288	ENSG00000166266	CUL5
627.2940188	0.769400822	0.28172027	2.731080802	0.006312699	0.512215252	ENSG00000104219	ZDHHC2
628.6034708	-0.186857004	0.234455381	-0.796983219	0.425460778	0.991301167	ENSG00000111775	COX6A1
633.3619004	-0.021363386	0.235933565	-0.090548312	0.927851504	0.999527751	ENSG00000164332	UBLCP1
631.3482554	0.086913	0.241289379	0.360202346	0.71869582	0.99900742	ENSG00000166128	RAB8B
634.9221047	-0.134329275	0.207357817	-0.647813895	0.517105327	0.993513219	ENSG00000160294	MCM3AP
633.8094889	-0.112515466	0.245778515	-0.457792113	0.647101803	0.997368416	ENSG00000172292	CERS6
624.5498235	-1.060841442	0.371450732	-2.855941182	0.004290946	0.462224997	ENSG00000137834	SMAD6
642.1518587	-0.518387274	0.424281687	-1.221799783	0.221783351	0.956884001	ENSG00000133216	EPHB2
633.7064111	-0.019956778	0.20398701	-0.097833572	0.922064449	0.999527751	ENSG00000213995	NAXD
626.5435615	-0.126900701	0.272967408	-0.464893232	0.642007918	0.996723676	ENSG00000169692	AGPAT2
628.3513674	-0.21937716	0.242550404	-0.90446009	0.365751488	0.981269214	ENSG00000008130	NADK
626.502223	0.385055352	0.214425328	1.795754986	0.072533493	0.885710197	ENSG00000071462	BUD23
634.4485699	0.066236055	0.237809809	0.278525329	0.780609124	0.999467288	ENSG00000039319	ZFYVE16
628.3307543	0.028348468	0.276352917	0.102580674	0.918295782	0.999527751	ENSG00000184164	CRELD2
634.3682898	0.070270386	0.326029086	0.215534101	0.829350928	0.999467288	ENSG00000010818	HIVEP2
627.8594901	-0.137553492	0.199033848	-0.691106029	0.489498912	0.993513219	ENSG00000101365	IDH3B
635.6591051	-0.063503932	0.270586494	-0.234689954	0.814449386	0.999467288	ENSG00000151292	CSNK1G3
632.3631281	-0.101458037	0.223716563	-0.453511514	0.650180454	0.997381078	ENSG00000204569	PPP1R10
633.6396127	-0.214323597	0.271706972	-0.788804186	0.430226463	0.991301167	ENSG00000075975	MKRN2
629.2441106	0.19552001	0.251813242	0.776448486	0.437484226	0.991301167	ENSG00000163820	FYCO1
628.0082655	-0.180500707	0.242033369	-0.745767855	0.455807661	0.99135833	ENSG00000130706	ADRM1
628.3239195	0.051426693	0.216637346	0.237386093	0.812357277	0.999467288	ENSG00000173542	MOB1B
628.5320304	-0.046385871	0.214567845	-0.216182772	0.828845282	0.999467288	ENSG00000148229	POLE3
622.5545649	-0.10732766	0.381018923	-0.281685906	0.778184363	0.999467288	ENSG00000184897	H1FX
630.9799533	0.278719114	0.228836342	1.217984486	0.223229868	0.956884001	ENSG00000160584	SIK3
640.5237741	0.683059186	0.42297888	1.614877761	0.106337169	0.922165836	ENSG00000115548	KDM3A
628.6553488	0.438145984	0.23760636	1.843999398	0.065183228	0.868202292	ENSG00000127990	SGCE

631.9351772	0.187408486	0.236616356	0.792035216	0.428340142	0.991301167	ENSG00000061987	MON2
628.5076902	0.003898326	0.254688528	0.015306249	0.987787857	0.999527751	ENSG00000171681	ATF7IP
616.6952943	-0.075842031	0.408807797	-0.18552002	0.852821179	0.999467288	ENSG00000108639	SYNGR2
621.0052117	-0.224453758	0.279485915	-0.803095063	0.421919791	0.991301167	ENSG00000145022	TCTA
626.9979372	0.234282294	0.317625504	0.737605423	0.460754259	0.99135833	ENSG00000109066	TMEM104
617.5289082	-0.145727885	0.385280625	-0.378238291	0.705253582	0.99900742	ENSG00000088280	ASAP3
631.7118318	-0.012681553	0.234655605	-0.054043255	0.956900702	0.999527751	ENSG00000143207	COP1
625.6777598	-0.244955855	0.259547044	-0.943782103	0.345281008	0.977691382	ENSG00000160087	UBE2J2
623.0256686	-0.45104601	0.255178116	-1.767573244	0.077132274	0.897052166	ENSG00000007520	TSR3
630.4489003	0.357858611	0.241456887	1.482080779	0.138318802	0.934144427	ENSG00000115977	AAK1
627.9770577	-0.33043135	0.229471196	-1.439968747	0.149876241	0.936396974	ENSG00000130703	OSBPL2
628.101568	0.065202054	0.275291181	0.236847596	0.812775026	0.999467288	ENSG00000105426	PTPRS
631.1348804	-0.400279699	0.36821626	-1.087077736	0.277002456	0.962465655	ENSG00000188938	FAM120AOS
624.538754	0.259044391	0.239742359	1.080511561	0.279914442	0.963267117	ENSG00000145725	PPIP5K2
632.9445866	0.376364621	0.280155864	1.343411543	0.179138751	0.9469453	ENSG00000156671	SAMD8
640.2583015	-0.291795483	0.446193093	-0.65396683	0.513133176	0.993513219	ENSG00000008513	ST3GAL1
628.9736015	-0.029563948	0.221735178	-0.133329986	0.893932414	0.999467288	ENSG00000143756	FBXO28
620.9683317	0.224053974	0.257428582	0.870353912	0.384107025	0.985655313	ENSG00000128951	DUT
621.8929474	-0.028573707	0.211469125	-0.135119995	0.892517001	0.999467288	ENSG00000105397	TYK2
626.6166779	-1.031751475	0.24409345	-4.226870798	2.37E-05	0.028520548	ENSG00000196405	EVL
625.8632632	0.103356046	0.250624415	0.412394165	0.68005054	0.998869317	ENSG00000182544	MFSD5
623.5827777	0.06957926	0.249705299	0.278645507	0.780516886	0.999467288	ENSG00000100347	SAMM50
624.2779024	-0.260803845	0.25998232	-1.003159927	0.315783704	0.974768742	ENSG00000145919	BOD1
621.5932552	-0.122664503	0.256341947	-0.478519043	0.632280824	0.994860874	ENSG00000071889	FAM3A
615.8950193	0.197366708	0.292858324	0.673932384	0.500354303	0.993513219	ENSG00000244509	APOBEC3C
618.4261983	-0.550366563	0.242083761	-2.273455106	0.022998769	0.744948012	ENSG00000053371	AKR7A2
624.9698851	0.087371142	0.21652517	0.403514943	0.686569444	0.998869317	ENSG00000169084	DHRSX
608.1132592	0.162811289	0.408967405	0.398103337	0.690554014	0.998869317	ENSG00000168899	VAMP5
617.7919476	0.240181518	0.271836532	0.88355129	0.376938484	0.982609538	ENSG00000167775	CD320
622.7310674	0.168267891	0.239363044	0.702981915	0.48206702	0.991525194	ENSG00000074755	ZZEF1
616.6712656	-0.259565712	0.262234442	-0.989823114	0.322260585	0.975337095	ENSG00000127837	AAMP
604.0051604	-0.040845728	0.289401102	-0.141138814	0.887760282	0.999467288	ENSG00000134202	GSTM3
617.1219894	-0.080537122	0.361140113	-0.223007967	0.823529306	0.999467288	ENSG00000131174	COX7B
615.9903952	0.215488467	0.301114805	0.715635576	0.474216401	0.99135833	ENSG00000106853	PTGR1
610.0879913	0.227036175	0.41994006	0.540639478	0.588756102	0.99483168	ENSG00000189221	MAOA
615.4515523	0.249449112	0.271527204	0.91868921	0.358258155	0.979427314	ENSG00000165996	HACD1
619.6769381	-0.159966678	0.209066708	-0.765146585	0.444184262	0.99135833	ENSG00000155252	PI4K2A
620.9131055	0.130747955	0.263193083	0.496775804	0.61934716	0.99483168	ENSG00000214655	ZSWIM8
633.9205921	2.382686175	0.554825142	4.294481262	1.75E-05	0.028100023	ENSG00000111371	SLC38A1
618.5841027	-0.093576025	0.200885732	-0.465817179	0.641346367	0.996553586	ENSG00000103035	PSMD7
619.2879748	0.045081028	0.207955076	0.216782531	0.828377827	0.999467288	ENSG00000005194	CIAPIN1
624.1895007	0.276318739	0.270675332	1.02084936	0.307325815	0.973427483	ENSG00000010292	NCAPD2
627.5713236	0.019106571	0.284856953	0.067074267	0.94652258	0.999527751	ENSG00000101966	XIAP
617.8584347	-0.264696727	0.21222228	-1.247261721	0.212301548	0.954310328	ENSG00000116478	HDAC1
614.6180796	-0.074873554	0.250131488	-0.299336779	0.764683095	0.999467288	ENSG00000168002	POLR2G
614.2785975	-0.122844184	0.234077388	-0.524801582	0.599721132	0.99483168	ENSG00000112110	MRPL18
626.1001233	0.307009261	0.280493961	1.094530733	0.273722276	0.962465655	ENSG00000204178	MACO1
620.9002828	-0.488742624	0.324721803	-1.505111819	0.132295331	0.933741583	ENSG00000125520	SLC2A4RG
615.8551549	-0.026985234	0.202624929	-0.133178253	0.894052409	0.999467288	ENSG00000114745	GORASP1
619.1958187	-0.171643068	0.260295059	-0.659417312	0.509627828	0.993513219	ENSG00000154059	IMPACT
617.2468519	-0.163879027	0.257487641	-0.636453952	0.524480586	0.99483168	ENSG00000170871	KIAA0232
619.8124255	0.120548261	0.219498335	0.549198974	0.582868909	0.99483168	ENSG00000265241	RBM8A
611.9574335	0.118691532	0.394183772	0.301107099	0.763332828	0.999467288	ENSG00000100714	MTHFD1
616.9886697	0.188488931	0.204674513	0.920920382	0.357091996	0.979427314	ENSG00000093000	NUP50
616.6685705	0.143463183	0.300398738	0.477575851	0.632952122	0.994963811	ENSG00000077420	APBB1IP
615.7651457	0.206450466	0.274689839	0.751576639	0.452305697	0.99135833	ENSG00000214160	ALG3
632.8060265	0.112785037	0.484394059	0.232837367	0.815887696	0.999467288	ENSG00000147454	SLC25A37
626.8238959	-0.150296903	0.414674077	-0.362445861	0.717018873	0.99900742	ENSG00000102452	NALCN
625.0900218	-0.150225792	0.338010946	-0.444440611	0.656724057	0.997568624	ENSG00000166716	ZNF592
619.9241179	-0.028184515	0.308572429	-0.091338409	0.9272237	0.999527751	ENSG00000101654	RNMT
615.5166533	0.075435136	0.234332543	0.321914897	0.747517167	0.999467288	ENSG00000164134	NAA15
618.3652846	-0.796256499	0.308789531	-2.578638263	0.00991906	0.630529247	ENSG00000178882	RFLNA
620.8396591	-0.218608264	0.238620067	-0.916135289	0.359595941	0.979465649	ENSG00000108587	GOSR1
618.012401	0.133268947	0.257504098	0.517541073	0.604778504	0.99483168	ENSG00000107789	MINPP1

612.3031503	-0.254161287	0.199835764	-1.271850855	0.203426116	0.954310328	ENSG00000126581	BECN1
619.2691221	0.284267981	0.261559362	1.086820135	0.277116307	0.962465655	ENSG00000014123	UFL1
616.1373672	0.103316029	0.226220251	0.456705484	0.647882749	0.997368416	ENSG00000096401	CDC5L
614.8332217	-0.399342697	0.282150882	-1.415351581	0.156965438	0.939464191	ENSG00000007047	MARK4
612.6921858	-0.251914947	0.222506516	-1.132168852	0.257563453	0.962465655	ENSG00000163703	CRELD1
616.196218	0.062330675	0.253272659	0.246101079	0.805603988	0.999467288	ENSG00000105819	PMPCB
610.3737663	-0.361920198	0.238066838	-1.520246172	0.128449117	0.928627271	ENSG00000169217	CD2BP2
613.9527267	0.348579068	0.543026651	0.641918897	0.520925844	0.993513219	ENSG00000143369	ECM1
606.8100207	-0.257644339	0.256495704	-1.004478182	0.315148183	0.974768742	ENSG00000242485	MRPL20
608.0596526	-0.198740713	0.289945375	-0.685441915	0.493065102	0.993513219	ENSG00000106049	HIBADH
610.4115157	-0.390385678	0.23079923	-1.69145139	0.090750624	0.912649921	ENSG00000105723	GSK3A
616.0850784	-0.084792851	0.217219596	-0.390355442	0.696273732	0.998869317	ENSG00000162928	PEX13
614.6903021	0.085205818	0.238028622	0.357964591	0.720369812	0.99900742	ENSG00000071894	CPSF1
614.8059455	0.106307969	0.261048824	0.407234046	0.683836098	0.998869317	ENSG00000102781	KATNAL1
609.8814284	-0.277515276	0.213012551	-1.302811855	0.192639003	0.949261383	ENSG00000125656	CLPP
609.4213971	-0.015962464	0.397849522	-0.040121864	0.967995971	0.999527751	ENSG00000068001	HYAL2
608.7790854	0.173258087	0.25075266	0.69095214	0.489595618	0.993513219	ENSG00000116266	STXBP3
610.8959252	0.110914838	0.220599304	0.502788702	0.615112838	0.99483168	ENSG00000153339	TRAPPC8
617.716122	-0.505100678	0.250715676	-2.014635408	0.043942865	0.807325344	ENSG00000144560	VGLL4
612.3368343	-0.019389592	0.234252528	-0.082772177	0.934032693	0.999527751	ENSG00000107897	ACBD5
610.3646421	0.147115437	0.227853055	0.645659268	0.518500045	0.993513219	ENSG00000186501	TMEM222
609.2404553	-0.061168287	0.342145586	-0.178778535	0.8581116	0.999467288	ENSG00000068971	PPP2R5B
603.3128253	-0.154469238	0.260153902	-0.593760989	0.552671984	0.99483168	ENSG00000107537	PHYH
607.8196789	-0.165333642	0.296364998	-0.557871688	0.576932006	0.99483168	ENSG00000099901	RANBP1
611.3734119	-0.270100197	0.275101845	-0.981818925	0.326189063	0.976026345	ENSG00000126767	ELK1
605.6291884	-0.112056893	0.272944942	-0.410547607	0.681404288	0.998869317	ENSG00000158042	MRPL17
613.2582488	-0.405202985	0.219813976	-1.843390451	0.065272023	0.868202292	ENSG00000031823	RANBP3
613.6475856	0.057696807	0.234439836	0.246104962	0.805600982	0.999467288	ENSG00000070061	ELP1
604.414253	0.059280948	0.265785634	0.223040452	0.823504023	0.999467288	ENSG00000161526	SAP30BP
619.817938	-0.084202647	0.380490355	-0.221300344	0.824858582	0.999467288	ENSG00000109756	RAPGEF2
599.7833061	-0.194696406	0.326834946	-0.595702535	0.551373965	0.99483168	ENSG00000117280	RAB29
609.7257498	-0.012128316	0.207110806	-0.05855955	0.95330293	0.999527751	ENSG00000177105	RHOG
609.1574855	0.216023024	0.223720109	0.965595023	0.334246867	0.976026345	ENSG00000171109	MFN1
610.8963351	-0.227948749	0.439049877	-0.519186455	0.603630723	0.99483168	ENSG00000173599	PC
610.5621725	-0.250574764	0.238375361	-1.051177282	0.293177175	0.970706863	ENSG00000172086	KRCC1
608.2888016	0.155630392	0.303259846	0.513191557	0.607817332	0.99483168	ENSG00000179163	FUCA1
610.9414831	0.224798133	0.218757124	1.027615143	0.304130898	0.973427483	ENSG00000153936	HS2ST1
606.8401589	-0.03869483	0.294980823	-0.131177444	0.895634939	0.999467288	ENSG00000126778	SIX1
605.7866665	-0.213634847	0.209363625	-1.020400977	0.307538331	0.973427483	ENSG00000185340	GAS2L1
608.8042929	-0.262916414	0.21344199	-1.231793303	0.218026306	0.956884001	ENSG00000204713	TRIM27
607.935868	-0.207659688	0.240751936	-0.862546286	0.388386974	0.986799595	ENSG00000174775	HRAS
606.5839066	-0.274012504	0.221779073	-1.235520108	0.216636998	0.954900762	ENSG00000137500	CCDC90B
602.6649974	-0.321263563	0.273350593	-1.175280287	0.239882599	0.962465655	ENSG00000107874	CUEDC2
611.7506689	0.180568537	0.329337341	0.548278361	0.583500783	0.99483168	ENSG00000089902	RCOR1
613.0711953	0.11509521	0.237536473	0.484536999	0.628004813	0.99483168	ENSG00000101596	SMCHD1
606.2368126	0.081481907	0.25239221	0.322838439	0.7468176	0.999467288	ENSG00000163166	IWS1
608.3591199	-0.085565501	0.221936879	-0.385539806	0.699837521	0.998869317	ENSG00000173456	RNF26
615.7146902	-0.302987458	0.314522399	-0.963325533	0.335384184	0.977237217	ENSG00000141564	RPTOR
607.9130249	-0.037575636	0.259206328	-0.144964192	0.884739144	0.999467288	ENSG00000167114	SLC27A4
603.1647205	-0.219558754	0.235375339	-0.932802709	0.350921842	0.977691382	ENSG00000100028	SNRPD3
606.0915623	0.123597994	0.297798654	0.415038793	0.678113505	0.998869317	ENSG00000134982	APC
617.6687253	1.42757616	0.38395955	3.7180379	0.000200776	0.111531153	ENSG00000109046	WSB1
613.2225282	-0.256481593	0.271518975	-0.944617565	0.344854156	0.977691382	ENSG00000110925	CSRNP2
596.1593542	-0.375453532	0.312464354	-1.201588362	0.229523053	0.959575179	ENSG00000184076	UQCR10
605.4186703	-0.397076751	0.209322548	-1.896961193	0.05783306	0.852306368	ENSG00000117640	MTFR1L
606.69588	-0.360293798	0.240148104	-1.500298325	0.133537143	0.933741583	ENSG00000108433	GOSR2
606.7360699	0.370475241	0.256516776	1.444253458	0.148667692	0.936396974	ENSG00000140836	ZFHX3
606.2909253	0.486928429	0.340105401	1.431698607	0.152230095	0.938052926	ENSG00000151835	SACS
601.2574493	0.125941253	0.235501376	0.534779265	0.592802503	0.99483168	ENSG00000127054	INTS11
608.9288263	-0.466484882	0.274852901	-1.697216512	0.089655734	0.910413547	ENSG00000116095	PLEKHA3
604.8088552	-0.261537706	0.314991551	-0.830300701	0.406368792	0.990226751	ENSG00000131828	PDHA1
606.2767196	0.351535195	0.216592609	1.623024893	0.104584084	0.92169898	ENSG00000164985	PSIP1
609.9625935	0.101412744	0.263132883	0.385405056	0.699937337	0.998869317	ENSG00000090989	EXOC1
608.8094911	-0.364394349	0.269949922	-1.349859063	0.177061195	0.946540546	ENSG00000118496	FBXO30

605.0556337	-0.145960941	0.217749574	-0.670315623	0.50265661	0.993513219	ENSG00000170632	ARMC10
604.7842577	0.077675254	0.238814229	0.325253876	0.744988936	0.999467288	ENSG00000127948	POR
602.6058985	-0.256537271	0.197003146	-1.302198851	0.192848418	0.949321643	ENSG00000136247	ZDHHC4
600.4271658	-0.164329914	0.234681804	-0.700224348	0.483787208	0.991892545	ENSG00000117691	NENF
599.3958332	-0.308807122	0.264758558	-1.166372578	0.243463841	0.962465655	ENSG00000135362	PRR5L
597.9337195	0.062959739	0.266870657	0.235918551	0.813495873	0.999467288	ENSG00000090924	PLEKHG2
594.9338238	-0.233298322	0.282903168	-0.824657864	0.40956586	0.990226751	ENSG00000241468	ATP5MF
605.0473079	-0.136233589	0.27871117	-0.488798454	0.624984393	0.99483168	ENSG00000077585	GPR137B
602.0033088	-0.319454478	0.237568702	-1.344682508	0.178727786	0.9469453	ENSG00000105700	KXD1
603.437796	0.258198102	0.198773834	1.298954178	0.193959655	0.950624691	ENSG00000100603	SNW1
595.9429466	-0.276291491	0.293359076	-0.941820156	0.346284724	0.977691382	ENSG00000184983	NDUFA6
599.7742876	-0.193655177	0.261958072	-0.739260203	0.45974901	0.99135833	ENSG00000082014	SMARCD3
599.4870598	-0.113250053	0.223275271	-0.507221658	0.6119993	0.99483168	ENSG00000138663	COPS4
601.5939877	0.008002873	0.240998301	0.033207175	0.973509376	0.999527751	ENSG00000156256	USP16
604.962163	0.035271927	0.257536777	0.136958798	0.891063362	0.999467288	ENSG00000177479	ARIH2
606.6747715	0.34863218	0.264077121	1.32019078	0.186771329	0.949094286	ENSG00000137776	SLTM
601.8215299	-0.104062229	0.219947111	-0.473123874	0.636124805	0.996399501	ENSG00000077238	IL4R
602.1491768	-0.118864473	0.291205884	-0.408180189	0.683141392	0.998869317	ENSG00000166398	KIAA0355
604.888096	-0.084160575	0.25967248	-0.324102788	0.745860216	0.999467288	ENSG00000118922	KLF12
604.098351	0.162790167	0.207672363	0.783879784	0.433110653	0.991301167	ENSG00000067369	TP53BP1
602.6495118	-0.089300913	0.193641487	-0.461166221	0.644679354	0.997368416	ENSG00000131375	CAPN7
606.7828433	0.324771012	0.325429973	0.997975106	0.31829143	0.974768742	ENSG00000164300	SERINC5
602.5275857	-0.302386556	0.279435721	-1.082132786	0.279193529	0.963267117	ENSG00000177732	SOX12
598.7430029	-0.242654924	0.257658173	-0.941770725	0.346310036	0.977691382	ENSG00000106635	BCL7B
600.2974619	-0.01672712	0.240948645	-0.06942193	0.944653773	0.999527751	ENSG00000072210	ALDH3A2
601.2632166	0.245928193	0.229844551	1.069976171	0.284630035	0.963419638	ENSG00000105983	LMBR1
599.2809333	0.265394398	0.247160798	1.073772219	0.282924784	0.963267117	ENSG00000048649	RSF1
598.4006462	-0.459749257	0.352474117	-1.304348984	0.192114621	0.949261383	ENSG00000120837	NFYB
609.6153439	0.413707912	0.498733243	0.829517417	0.406811685	0.990226751	ENSG00000153531	ADPRHL1
601.9638041	-1.048814698	0.477538068	-2.196295475	0.028070801	0.769167109	ENSG00000230202	AL450405.1
603.0951549	0.212870356	0.286020599	0.74424834	0.456726248	0.99135833	ENSG00000164938	TP53INP1
599.7381484	-0.033368606	0.245091689	-0.136147441	0.891704724	0.999467288	ENSG00000125870	SNRPB2
602.5721703	-1.504988315	0.428774775	-3.509974005	0.000448151	0.181542056	ENSG00000130203	APOE
602.9028541	0.31867526	0.260658387	1.222578193	0.221489054	0.956884001	ENSG00000136997	MYC
596.7059151	-0.133016002	0.226130994	-0.588225434	0.556380988	0.99483168	ENSG00000197713	RPE
607.9199726	-0.113348507	0.286994137	-0.394950602	0.692879344	0.998869317	ENSG00000129071	MBD4
597.8054471	-0.110459715	0.223763669	-0.493644548	0.621557233	0.99483168	ENSG00000204231	RXRB
598.5033784	0.13962367	0.225009618	0.62052312	0.534913437	0.99483168	ENSG00000165416	SUGT1
594.3516099	0.177021367	0.241735407	0.732293912	0.463989195	0.99135833	ENSG00000104960	PTOV1
601.4996697	-0.322108407	0.312644287	-1.030271209	0.30288271	0.972793733	ENSG00000111911	HINT3
596.1795075	0.169863098	0.306017215	0.555076936	0.578842038	0.99483168	ENSG00000135929	CYP27A1
596.9313998	0.023170411	0.237499012	0.09756003	0.922281665	0.999527751	ENSG00000168397	ATG4B
602.2756175	0.160140545	0.231234696	0.692545486	0.488594831	0.993513219	ENSG00000217128	FNIP1
598.8677894	0.348760035	0.288576894	1.208551489	0.226835203	0.958865115	ENSG00000095319	NUP188
601.2449273	-0.049837638	0.246984091	-0.201784812	0.840084956	0.999467288	ENSG00000146872	TLK2
604.5408481	-0.389041029	0.257566123	-1.510451082	0.130928363	0.929694367	ENSG00000125686	MED1
600.3718392	0.221697169	0.218671933	1.013834586	0.310661689	0.973427483	ENSG00000076928	ARHGEF1
600.7512895	0.011308288	0.273653868	0.041323327	0.967038136	0.999527751	ENSG00000169299	PGM2
593.7555402	-0.071212998	0.235243188	-0.302720765	0.762102672	0.999467288	ENSG00000164172	MOCS2
604.7299404	-0.175323249	0.229351669	-0.764429793	0.44461116	0.99135833	ENSG00000087448	KLHL42
599.5722702	-0.493088156	0.250365491	-1.969473323	0.048898763	0.83480477	ENSG00000134874	DZIP1
591.6724361	0.297308653	0.309045333	0.96202279	0.336038156	0.977691382	ENSG00000102471	NDFIP2
596.5557122	0.264249178	0.252922697	1.04478238	0.29612356	0.970706863	ENSG00000204842	ATXN2
597.8030741	-0.096935523	0.23598438	-0.410770931	0.68124051	0.998869317	ENSG00000166908	PIP4K2C
600.4247482	0.1689805	0.231516864	0.729884195	0.465460974	0.99135833	ENSG00000023041	ZDHHC6
598.4100508	-0.137555338	0.222366668	-0.618596928	0.536181926	0.99483168	ENSG00000139168	ZCRB1
592.7185511	-0.311403167	0.23258772	-1.338863321	0.180615172	0.949094286	ENSG00000089289	IGBP1
591.8843805	-0.100328231	0.241300973	-0.415780467	0.677570654	0.998869317	ENSG00000277258	PCGF2
596.3529532	-0.12710719	0.217344986	-0.584817676	0.558670318	0.99483168	ENSG00000163161	ERCC3
596.5494965	0.210074614	0.232128675	0.904992088	0.365469579	0.981269214	ENSG00000039123	MTREX
611.1290845	0.025731088	0.467425917	0.055048485	0.956099837	0.999527751	ENSG00000108846	ABCC3
591.6915603	-0.18039133	0.237964426	-0.758060074	0.44841502	0.99135833	ENSG00000140307	GTF2A2
593.2911292	-0.386319691	0.212648842	-1.816702534	0.069262674	0.877509468	ENSG00000157350	ST3GAL2
591.5004465	0.21559125	0.395997052	0.544426402	0.586148082	0.99483168	ENSG00000154678	PDE1C

597.3374337	0.271846557	0.246480942	1.102911061	0.27006579	0.962465655	ENSG00000090905	TNRC6A
594.9125212	0.308164108	0.280821666	1.097365855	0.272481493	0.962465655	ENSG00000129255	MPDU1
596.9583861	0.426794968	0.275575264	1.548741938	0.121443768	0.923922314	ENSG00000157224	CLDN12
597.4010867	0.223719614	0.220089605	1.016493324	0.309394525	0.973427483	ENSG00000116062	MSH6
578.3523621	0.392802375	0.424329308	0.925701732	0.354601007	0.977691382	ENSG00000085117	CD82
597.0714781	-0.222178231	0.253852211	-0.875226692	0.381450573	0.985209339	ENSG00000066629	EML1
603.3413455	-0.450933619	0.291456845	-1.547171139	0.121821983	0.923922314	ENSG00000029993	HMGB3
592.0540205	0.071408209	0.259878623	0.274775233	0.783488927	0.999467288	ENSG00000127955	GNAI1
602.0461604	-0.157719819	0.312412007	-0.504845572	0.613667305	0.99483168	ENSG00000088387	DOCK9
593.9238124	-0.324314832	0.293195163	-1.106139777	0.268666024	0.962465655	ENSG00000206418	RAB12
589.9441255	-0.278118331	0.197421631	-1.408753085	0.158908192	0.939464191	ENSG00000123124	WWP1
583.7686092	0.08093693	0.254337302	0.318226737	0.750312953	0.999467288	ENSG00000157110	RBPMS
591.3993207	-0.034324313	0.253082543	-0.135624973	0.892117762	0.999467288	ENSG00000136738	STAM
588.0341518	0.550369658	0.451555417	1.218830818	0.222908412	0.956884001	ENSG00000076003	MCM6
593.0174483	0.09587504	0.295160807	0.324823074	0.74531498	0.999467288	ENSG00000119979	FAM45A
589.4714242	-0.202135474	0.242161913	-0.834712081	0.403879838	0.988927059	ENSG00000136783	NIPSNAP3A
594.1381927	-0.061373412	0.201236021	-0.304982236	0.760379682	0.999467288	ENSG00000171853	TRAPPC12
596.0418597	0.042539143	0.246988608	0.172231193	0.863255776	0.999467288	ENSG00000088766	CRLS1
589.1397966	-0.349037021	0.245354974	-1.422579763	0.154858014	0.939432741	ENSG00000198858	R3HDM4
605.4543587	-0.371268909	0.398931249	-0.93065888	0.352030047	0.977691382	ENSG00000163637	PRICKLE2
590.8653333	-0.012682507	0.196897579	-0.064411696	0.948642418	0.999527751	ENSG00000109270	LAMTOR3
592.7652427	0.150550287	0.225015817	0.669065355	0.503453789	0.993513219	ENSG00000100722	ZC3H14
584.0739232	0.029806725	0.277831909	0.107283302	0.914564231	0.999527751	ENSG00000104823	ECH1
587.0309975	-0.100608499	0.211408353	-0.475896518	0.634148103	0.99544042	ENSG00000011485	PPP5C
590.0328454	0.42027499	0.234919882	1.789014141	0.073612538	0.888663898	ENSG00000120697	ALG5
539.0358711	-1.955052078	1.946669399	-1.004306165	0.315231063	0.974768742	ENSG00000106819	ASPN
588.4754521	-0.359834476	0.2021317	-1.78019814	0.07504354	0.893390506	ENSG00000178057	NDUFAF3
588.6298756	-0.087990386	0.210065938	-0.418870316	0.675310912	0.998869317	ENSG00000119446	RBM18
596.8336238	0.037395194	0.258380381	0.144729233	0.884924658	0.999467288	ENSG00000102974	CTCF
589.7721536	0.236378344	0.308607114	0.765952349	0.443704656	0.99135833	ENSG00000108106	UBE2S
588.3547862	-0.034382457	0.209930144	-0.163780468	0.869903968	0.999467288	ENSG00000164609	SLU7
587.7472902	0.572715831	0.264048641	2.16897852	0.030084315	0.773201806	ENSG00000162402	USP24
584.0410833	0.011865497	0.25489467	0.046550589	0.962871414	0.999527751	ENSG00000171204	TMEM126B
590.1389094	-0.083658095	0.28773556	-0.29074646	0.771245236	0.999467288	ENSG00000075945	KIFAP3
580.8638717	0.769205633	0.346952153	2.217036631	0.026620588	0.764376062	ENSG00000131386	GALNT15
584.2449177	0.328053899	0.229425346	1.429893883	0.152747478	0.939432741	ENSG00000141699	RETREG3
594.4133821	-0.436137026	0.473768252	-0.920570392	0.357274766	0.979427314	ENSG00000198948	MFAP3L
582.6321825	-0.150853779	0.230608204	-0.654156169	0.513011197	0.993513219	ENSG00000196411	EPHB4
579.9996196	-0.307887921	0.287883517	-1.069487842	0.284849905	0.96393795	ENSG00000106605	BLVRA
589.5351447	0.16224829	0.21168439	0.766463176	0.443400755	0.99135833	ENSG00000198815	FOXJ3
587.2330869	-0.038127089	0.244606661	-0.155871016	0.876134692	0.999467288	ENSG00000117682	DHDDS
584.2154866	0.472361031	0.391799979	1.205617806	0.22796489	0.958865115	ENSG00000125257	ABCC4
588.0040789	-0.071321197	0.218525249	-0.326375085	0.744140586	0.999467288	ENSG00000124356	STAMBP
574.1461613	-0.084817612	0.375838714	-0.225675559	0.821453769	0.999467288	ENSG00000034152	MAP2K3
592.6808947	-0.027304166	0.250732927	-0.108897409	0.913283862	0.999527751	ENSG00000198301	SDAD1
582.7621638	0.489265181	0.419271491	1.16694121	0.243234112	0.962465655	ENSG00000123342	MMP19
587.9502025	-0.421166157	0.261033724	-1.613454965	0.1066457	0.922325655	ENSG00000167566	NCKAP5L
587.0581404	0.103691482	0.196256541	0.528346628	0.597258774	0.99483168	ENSG00000164022	AIMP1
579.3204286	-0.271117106	0.260858541	-1.039326159	0.298653073	0.972186492	ENSG00000001617	SEMA3F
579.5233336	-0.278943715	0.261402245	-1.067105278	0.285924299	0.964826902	ENSG00000134996	OSTF1
586.0897794	-0.169681929	0.210768012	-0.805064902	0.420782228	0.991301167	ENSG00000167716	WDR81
584.4544089	-0.066141792	0.228894386	-0.288962056	0.772610414	0.999467288	ENSG00000198373	WWP2
580.0970428	-0.421452401	0.220136675	-1.914503344	0.055555872	0.847598083	ENSG00000212747	RTL8B
589.7915579	0.121156032	0.263795873	0.459279483	0.64603348	0.997368416	ENSG00000186566	GPATCH8
582.1868433	-0.628005412	0.228388776	-2.749720996	0.005964603	0.499617804	ENSG00000142459	EVI5L
583.0464472	-0.132678414	0.261800568	-0.506791923	0.612300824	0.99483168	ENSG00000198369	SPRED2
587.2676194	-0.027530766	0.328328935	-0.083851172	0.933174762	0.999527751	ENSG00000165434	PGM2L1
588.1130183	0.292049531	0.315467473	0.925767491	0.354566825	0.977691382	ENSG00000094975	SUCO
584.1682052	0.375949685	0.232063723	1.620027812	0.105226303	0.92169898	ENSG00000130714	POMT1
585.3639269	0.585052831	0.211690249	2.763721207	0.005714634	0.499617804	ENSG00000173575	CHD2
586.9157924	-0.67741116	0.245018514	-2.764734591	0.005696913	0.499617804	ENSG00000167157	PRRX2
583.059599	-0.288096253	0.232541001	-1.238905189	0.215380612	0.954310328	ENSG00000182218	HHIPL1
581.8996223	0.142240678	0.221526083	0.642094494	0.520811831	0.993513219	ENSG00000010270	STARD3NL
577.9850892	-0.54787655	0.266451963	-2.056192581	0.039763957	0.793985738	ENSG00000074071	MRPS34

580.1915376	0.144168185	0.209997055	0.686524796	0.492382234	0.993513219	ENSG00000077713	SLC25A43
578.9722516	0.054551229	0.202271025	0.269693741	0.787395877	0.999467288	ENSG00000161204	ABCF3
577.1546382	0.057733859	0.280516747	0.205812521	0.83693736	0.999467288	ENSG00000165688	PMPCA
578.5809537	-0.332917718	0.238966317	-1.393157506	0.163572167	0.943223078	ENSG00000167965	MLST8
582.8379616	-0.045033639	0.214444374	-0.210001496	0.833666506	0.999467288	ENSG00000067596	DHX8
581.2527878	0.081041215	0.250904344	0.322996459	0.746697924	0.999467288	ENSG00000135334	AKIRIN2
580.2974939	0.230837315	0.246686423	0.935752006	0.349400887	0.977691382	ENSG00000081177	EXD2
584.3755993	-0.165142552	0.241380203	-0.684159469	0.493874473	0.993513219	ENSG00000065559	MAP2K4
578.1158097	-0.086725436	0.23504414	-0.368975105	0.712146281	0.99900742	ENSG00000160695	VPS11
589.3824387	0.043497438	0.292579114	0.148668978	0.881814841	0.999467288	ENSG00000065526	SPEN
584.0030658	-0.408917616	0.226877481	-1.80237199	0.071486899	0.883977125	ENSG00000103495	MAZ
579.9474693	-0.033135798	0.22721783	-0.145832739	0.88405343	0.999467288	ENSG00000120675	DNAJC15
578.7445432	0.532964929	0.224905482	2.369728495	0.017801152	0.726053908	ENSG00000137449	CPEB2
580.710441	-0.231261279	0.221216544	-1.045406795	0.295834998	0.970706863	ENSG00000179115	FARSA
580.5408056	-0.398022935	0.204466625	-1.946640117	0.051577895	0.843646143	ENSG00000040633	PHF23
580.8286905	-0.384448773	0.23265547	-1.652438143	0.098445265	0.921147998	ENSG00000182952	HMGN4
585.8321366	-0.305350664	0.28517703	-1.070740739	0.28428602	0.963310634	ENSG00000161542	PRPSAP1
576.7007698	-0.505275798	0.249194768	-2.027634054	0.042597611	0.806698895	ENSG00000092330	TINF2
579.1858506	-0.155890209	0.260786728	-0.597768954	0.549994109	0.99483168	ENSG00000066697	MSANTD3
580.4779007	-0.321017916	0.301650332	-1.064205409	0.287235657	0.965284229	ENSG00000107140	TESK1
579.6154381	-0.480203169	0.22720885	-2.113487961	0.034559024	0.783572981	ENSG00000103549	RNF40
555.2673407	0.397940307	0.845928131	0.470418577	0.638055995	0.996553586	ENSG00000145244	CORIN
579.145486	0.084325685	0.253253851	0.332969014	0.739157673	0.999467288	ENSG00000171135	JAGN1
574.717553	0.012135872	0.266474895	0.045542271	0.963675083	0.999527751	ENSG00000137478	FCHSD2
580.6897776	-0.102668752	0.236370573	-0.434355052	0.664030632	0.997568624	ENSG00000143157	POGK
575.9712659	0.019508059	0.217158718	0.089833184	0.928419778	0.999527751	ENSG00000225921	NOL7
578.5693573	-0.134810747	0.224839236	-0.599587282	0.548781325	0.99483168	ENSG00000061794	MRPS35
569.1723586	-0.105106695	0.384848099	-0.273112158	0.784767001	0.999467288	ENSG00000124615	MOCS1
575.9724201	-0.381437202	0.210214347	-1.814515554	0.06959839	0.877912272	ENSG00000128928	IVD
581.1031303	-0.184586553	0.279451852	-0.660530789	0.508913267	0.993513219	ENSG00000114784	EIF1B
595.5553884	-0.706829732	0.943101363	-0.749473768	0.453571703	0.99135833	ENSG00000129009	ISLR
579.7142382	0.134912456	0.228894263	0.589409511	0.555586598	0.99483168	ENSG00000064313	TAF2
579.5490775	0.022143006	0.288395901	0.076779891	0.938798648	0.999527751	ENSG00000082212	ME2
575.5132214	-0.055749441	0.224092075	-0.248779175	0.8035316	0.999467288	ENSG00000144635	DYNC1LI1
574.6540868	-0.317286473	0.35004862	-0.906406866	0.36472054	0.981076669	ENSG00000069535	MAOB
574.3204192	0.211199175	0.334660407	0.631085037	0.527984924	0.99483168	ENSG00000136450	SRSF1
576.4475462	-0.238637536	0.242645042	-0.983484079	0.325369248	0.976026345	ENSG00000187713	TMEM203
573.709069	-0.042284298	0.21502225	-0.196650801	0.844100813	0.999467288	ENSG00000185129	PURA
570.1279818	-0.238581921	0.364903185	-0.653822523	0.513226154	0.993513219	ENSG00000006534	ALDH3B1
567.2523437	-0.354919795	0.250793744	-1.415185997	0.157013969	0.939464191	ENSG00000127445	PIN1
572.0025286	-0.290051684	0.282530024	-1.026622516	0.304598248	0.973427483	ENSG00000072506	HSD17B10
574.3555663	-0.01713118	0.199508027	-0.085867123	0.931572047	0.999527751	ENSG00000196792	STRN3
574.6080213	0.03014974	0.215739561	0.139750632	0.888857021	0.999467288	ENSG00000140829	DHX38
568.8362554	-0.267452956	0.221547946	-1.207201246	0.227354651	0.958865115	ENSG00000159228	CBR1
576.9425902	-0.224296126	0.239414952	-0.936850954	0.348835231	0.977691382	ENSG00000132694	ARHGEF11
570.6551501	-0.460455791	0.241510322	-1.906567745	0.056576585	0.84929896	ENSG00000103415	HMOX2
575.0044524	0.225484387	0.209742728	1.075052225	0.282351347	0.963267117	ENSG00000112144	ICK
574.854749	0.020423641	0.202531306	0.100841897	0.919675967	0.999527751	ENSG00000134453	RBM17
569.5385324	-0.41521144	0.266379431	-1.558721854	0.119062227	0.923922314	ENSG00000187778	MCRS1
583.7502775	0.048449156	0.329213647	0.147166304	0.883000757	0.999467288	ENSG00000150995	ITPR1
579.1725804	-0.090091245	0.247954627	-0.363337626	0.71635269	0.99900742	ENSG00000074356	NCBP3
570.3506362	0.021838047	0.29646626	0.073661155	0.941280008	0.999527751	ENSG00000138777	PPA2
583.2949532	-0.515860829	0.343380662	-1.502300177	0.133019603	0.933741583	ENSG00000145147	SLIT2
573.3141812	-0.410178561	0.259579968	-1.580162616	0.114069631	0.923922314	ENSG00000136807	CDK9
577.7356068	-0.2565706	0.214842325	-1.194227438	0.232389013	0.959575179	ENSG00000117751	PPP1R8
579.868131	-0.839419416	0.391942229	-2.141691695	0.032218299	0.775963115	ENSG00000168453	HR
573.2624489	-0.010509386	0.323163862	-0.032520301	0.974057126	0.999527751	ENSG00000116120	FARSB
571.3607703	0.179873802	0.260057767	0.69166864	0.489145445	0.993513219	ENSG00000136271	DDX56
575.2330063	0.186376647	0.23306735	0.799668624	0.423902816	0.991301167	ENSG00000112739	PRPF4B
566.3827263	-0.109597986	0.254816153	-0.430106116	0.667118451	0.997568624	ENSG00000143507	DUSP10
589.6291003	-0.53132763	0.446776793	-1.18924626	0.234342776	0.960525552	ENSG00000015413	DPEP1
570.1102687	-0.22897399	0.235250686	-0.97331912	0.330394722	0.976026345	ENSG00000147457	CHMP7
568.1071441	-0.270693102	0.223304626	-1.21221448	0.22543029	0.958865115	ENSG00000104529	EEF1D
575.3006539	0.036581173	0.291358406	0.125553861	0.900085087	0.999527751	ENSG00000125503	PPP1R12C

567.781097	-0.84258835	0.275373146	-3.059805805	0.002214805	0.343961675	ENSG00000173156	RHOD
568.1779286	-0.130636122	0.287877674	-0.453790392	0.6499797	0.997381078	ENSG00000178802	MPI
563.7025272	-0.040669346	0.288769137	-0.140836886	0.887998803	0.999467288	ENSG00000164751	PEX2
571.9493057	-0.073566816	0.197448074	-0.372588165	0.709454983	0.99900742	ENSG00000134265	NAPG
570.8821625	0.2625162	0.286555231	0.916110303	0.359609045	0.979465649	ENSG00000064607	SUGP2
572.4062207	-0.099051448	0.226785748	-0.436762225	0.662283797	0.997568624	ENSG00000220842	RPL21P16
568.0940057	-0.211577371	0.250366057	-0.845072108	0.39807058	0.988378925	ENSG00000180900	SCRIB
570.5578121	0.128231524	0.261446664	0.490469154	0.623801952	0.99483168	ENSG00000141076	UTP4
566.6718364	-0.35025967	0.216430019	-1.618350684	0.105587044	0.92169898	ENSG00000131043	AAR2
570.0290595	0.080817875	0.261496425	0.309059197	0.757276496	0.999467288	ENSG00000100503	NIN
563.1608404	-0.175838284	0.24053748	-0.731022392	0.464765476	0.99135833	ENSG00000055950	MRPL43
574.3316895	0.019798752	0.280411038	0.07060618	0.943711192	0.999527751	ENSG00000100522	GNPNAT1
569.789026	-0.078982438	0.21985696	-0.359244656	0.719412073	0.99900742	ENSG00000116747	TROVE2
570.1609999	-0.046595576	0.19696007	-0.236573715	0.812987514	0.999467288	ENSG00000118518	RNF146
563.5071302	-0.166131941	0.231557236	-0.71745519	0.473093276	0.99135833	ENSG00000004779	NDUFAB1
569.5101794	-0.27978686	0.281070735	-0.995432199	0.319526105	0.974964731	ENSG00000160703	NLRX1
569.977419	0.134551649	0.339082518	0.396810928	0.691506892	0.998869317	ENSG00000218336	TENM3
574.4864797	-0.209043777	0.324139807	-0.644918559	0.518979964	0.993513219	ENSG00000152990	ADGRA3
565.4577328	-0.482295368	0.268805429	-1.794217362	0.072778482	0.886289726	ENSG00000171469	ZNF561
570.1893303	-0.39176656	0.209718243	-1.86806142	0.06175351	0.865910462	ENSG00000168118	RAB4A
567.6187027	-0.253738379	0.193501429	-1.311299768	0.189756507	0.949094286	ENSG00000116198	CEP104
567.2556161	-0.01158276	0.20661593	-0.056059374	0.955294508	0.999527751	ENSG00000123575	FAM199X
568.4894311	-0.199259669	0.228332087	-0.872674845	0.382840333	0.985655313	ENSG00000111647	UHRF1BP1L
562.0691566	-0.036110906	0.292933136	-0.123273544	0.90189049	0.999527751	ENSG00000100983	GSS
570.574852	0.411278009	0.248562891	1.654623536	0.098000886	0.921147998	ENSG00000244754	N4BP2L2
565.0926423	-0.199144075	0.24031295	-0.828686409	0.407281878	0.990226751	ENSG00000067829	IDH3G
560.1015407	0.188122386	0.344234282	0.54649521	0.584725579	0.99483168	ENSG00000133935	ERG28
572.3683918	0.058669838	0.229302128	0.255862598	0.798056936	0.999467288	ENSG00000066117	SMARCD1
569.9014899	0.324968162	0.245491079	1.323747341	0.18558697	0.949094286	ENSG00000152749	GPR180
553.3072074	-0.51511461	0.419028265	-1.229307552	0.218956517	0.956884001	ENSG00000161281	COX7A1
564.3801166	-0.329551337	0.215601365	-1.528521569	0.126383092	0.928627271	ENSG00000134330	IAH1
568.3431854	0.013907846	0.243058735	0.057220102	0.954369865	0.999527751	ENSG00000011198	ABHD5
545.6910436	0.905788381	1.349842816	0.671032486	0.502199835	0.993513219	ENSG00000171388	APLN
568.6896621	0.261869709	0.296599078	0.882908034	0.377285963	0.982609538	ENSG00000110218	PANX1
564.0016026	-0.158415432	0.310879945	-0.509571087	0.610351984	0.99483168	ENSG00000188215	DCUN1D3
569.3923557	0.091605172	0.294602551	0.310944937	0.755842482	0.999467288	ENSG00000070882	OSBPL3
567.5224538	0.150849322	0.271380411	0.555859288	0.578307051	0.99483168	ENSG00000007541	PIGQ
567.3805564	0.451487941	0.238118728	1.89606229	0.057951808	0.852306368	ENSG00000172269	DPAGT1
567.6286451	-0.054171271	0.221412635	-0.244662061	0.806718107	0.999467288	ENSG00000181090	EHMT1
567.3175181	-0.045513727	0.21808999	-0.208692417	0.83468836	0.999467288	ENSG00000146085	MUT
563.1205122	0.478840513	0.208462642	2.297008747	0.021618271	0.73997555	ENSG00000023608	SNAPC1
564.4214182	0.136618804	0.233160137	0.585944087	0.557913089	0.99483168	ENSG00000165733	BMS1
561.6519478	-0.100207017	0.232904118	-0.430250089	0.667013729	0.997568624	ENSG00000196998	WDR45
564.1970366	-0.073530082	0.256191757	-0.287011897	0.774103211	0.999467288	ENSG00000108557	RAI1
559.7710415	0.094143949	0.237901278	0.395726959	0.692306465	0.998869317	ENSG00000168924	LETM1
561.6944214	0.021650696	0.230876589	0.093776057	0.925287052	0.999527751	ENSG00000198730	CTR9
561.8236436	-0.430069766	0.217321247	-1.978958675	0.047820659	0.829140192	ENSG00000149792	MRPL49
558.0616785	-0.033225427	0.277834191	-0.119587252	0.904810119	0.999527751	ENSG00000027847	B4GALT7
564.7742514	0.451348306	0.259350045	1.740305485	0.081805391	0.897807954	ENSG00000171988	JMJD1C
563.2691622	-0.173343639	0.231789686	-0.747848802	0.454551362	0.99135833	ENSG00000105325	FZR1
564.2914369	0.159050619	0.24789495	0.641604916	0.521129741	0.993513219	ENSG00000156273	BACH1
562.9921344	-0.115039128	0.207032062	-0.555658517	0.57844432	0.99483168	ENSG00000105671	DDX49
565.31966	-0.103056545	0.255706449	-0.403026773	0.686928529	0.998869317	ENSG00000124535	WRNIP1
562.5067045	0.283815658	0.198838283	1.427369289	0.153473478	0.939432741	ENSG00000160679	CHTOP
557.3115362	-0.155323483	0.251076371	-0.61863043	0.53615985	0.99483168	ENSG00000275023	MLLT6
553.2949036	0.073717013	0.338671963	0.217664943	0.827690181	0.999467288	ENSG00000115828	QPCT
562.8325539	0.262235348	0.211856903	1.237794683	0.2157922	0.954310328	ENSG00000168538	TRAPPC11
553.8830382	-0.73394493	0.293612964	-2.49970206	0.012429779	0.657594515	ENSG00000182154	MRPL41
555.242687	-0.608655598	0.292390253	-2.081654883	0.037374004	0.790190505	ENSG00000103266	STUB1
563.0373808	0.22853186	0.273110213	0.836775223	0.402718927	0.98885914	ENSG00000197563	PIGN
562.8546613	-0.326371124	0.201030329	-1.623491963	0.10448428	0.92169898	ENSG00000020129	NCDN
562.507273	0.471640002	0.216944812	2.174009134	0.029704449	0.773201806	ENSG00000024048	UBR2
556.4162785	-0.122603754	0.231151073	-0.530405297	0.595830954	0.99483168	ENSG00000084070	SMAP2
563.1645978	0.107317538	0.238270258	0.450402575	0.652420188	0.997449432	ENSG00000068654	POLR1A

561.5585312	-0.354133384	0.229717249	-1.541605539	0.123169479	0.927012392	ENSG00000100897	DCAF11
563.4374939	0.099477223	0.21844664	0.455384542	0.648832614	0.997368416	ENSG00000121749	TBC1D15
563.5558479	0.186029674	0.274323772	0.678139092	0.497683506	0.993513219	ENSG00000189339	SLC35E2B
565.5564258	0.100895135	0.255057907	0.395577366	0.692416837	0.998869317	ENSG00000038219	BOD1L1
558.993722	-0.227845616	0.240063436	-0.949105868	0.34256677	0.977691382	ENSG00000112186	CAP2
560.8723488	-0.246403934	0.255031213	-0.966171671	0.333958286	0.976026345	ENSG00000107021	TBC1D13
566.9005979	0.164524308	0.281154453	0.585174114	0.558430648	0.99483168	ENSG00000204852	TCTN1
559.7495949	0.111906445	0.38373203	0.291626543	0.770572179	0.999467288	ENSG00000188352	FOCAD
558.081206	0.104319969	0.248840914	0.419223539	0.675052771	0.998869317	ENSG00000111011	RSRC2
557.3662969	0.179390967	0.285890661	0.627481032	0.530343966	0.99483168	ENSG00000164548	TRA2A
553.7859464	-0.441985287	0.236013494	-1.872711936	0.061108174	0.863586458	ENSG00000169857	AVEN
556.5431762	0.16396845	0.242135565	0.677176233	0.498294145	0.993513219	ENSG00000151247	EIF4E
556.8556023	-0.044526101	0.239465846	-0.185939257	0.852492397	0.999467288	ENSG00000113360	DROSHA
560.7739365	-0.015447659	0.266572788	-0.057949121	0.953789156	0.999527751	ENSG00000050393	MCUR1
558.6490435	-0.052891983	0.261585652	-0.202197568	0.839762274	0.999467288	ENSG00000172046	USP19
558.8237916	-0.090698287	0.244520032	-0.37092375	0.710694325	0.99900742	ENSG00000108599	AKAP10
561.6843562	0.236652486	0.285169817	0.829865125	0.406615045	0.990226751	ENSG00000112763	BTN2A1
556.8107226	-0.345648001	0.240180366	-1.439118473	0.150116959	0.936396974	ENSG00000135070	ISCA1
547.0898835	-0.711490807	0.313297971	-2.270971641	0.023148695	0.744948012	ENSG00000125995	ROMO1
559.732113	0.125269881	0.31585904	0.396600589	0.691662018	0.998869317	ENSG00000144642	RBMS3
554.9655737	0.203844533	0.227757458	0.895007063	0.370783312	0.981849219	ENSG00000172175	MALT1
556.2685945	1.448695765	1.104203812	1.311982217	0.189526135	0.949094286	ENSG00000088325	TPX2
553.4510627	-0.181151885	0.289191509	-0.626408036	0.53104734	0.99483168	ENSG00000186834	HEXIM1
554.6400321	0.199673599	0.2918733	0.684110535	0.49390537	0.993513219	ENSG00000164934	DCAF13
548.4715324	-0.489352793	0.279700551	-1.749559633	0.08019433	0.897807954	ENSG00000182575	NXPH3
556.8051458	0.257810531	0.276340544	0.932945008	0.350848362	0.977691382	ENSG00000103657	HERC1
553.4284922	0.213393065	0.203818615	1.046975348	0.295110948	0.970706863	ENSG00000137955	RABGGTB
554.1621816	0.03199701	0.237430204	0.134763858	0.892798582	0.999467288	ENSG00000121022	COPS5
564.2461676	0.339349342	0.303570375	1.117860538	0.263626561	0.962465655	ENSG00000060749	QSER1
548.3003753	-0.030818349	0.264301801	-0.116602871	0.907174763	0.999527751	ENSG00000204351	SKIV2L
555.0218009	0.122009555	0.216250522	0.564204673	0.572614848	0.99483168	ENSG00000137275	RIPK1
549.2480937	-0.086097841	0.264007257	-0.326119222	0.744334155	0.999467288	ENSG00000116898	MRPS15
554.5717809	-0.361751518	0.348018095	-1.039461808	0.298590011	0.972186492	ENSG00000163512	AZI2
558.0937825	0.14378024	0.204624649	0.702653573	0.482271667	0.991525194	ENSG00000075856	SART3
562.41366	-0.285917518	0.384707758	-0.743207051	0.457356335	0.99135833	ENSG00000134278	SPIRE1
542.512798	-0.390584075	0.362554228	-1.077312149	0.281340837	0.963267117	ENSG00000166347	CYB5A
553.207797	0.006169158	0.251260674	0.02455282	0.980411652	0.999527751	ENSG00000059758	CDK17
571.0594918	-0.451647733	0.4325102	-1.044247588	0.296370855	0.970706863	ENSG00000230838	LINC01614
554.6432734	0.461813758	0.266228689	1.734650611	0.082802709	0.899952548	ENSG00000105835	NAMPT
551.9007887	0.092707361	0.231458887	0.400534893	0.688762588	0.998869317	ENSG00000141252	VPS53
552.955289	0.272968421	0.230271009	1.185422437	0.235850465	0.962465655	ENSG00000071794	HLTF
550.9216466	0.174180791	0.256165826	0.679953268	0.496534051	0.993513219	ENSG00000171155	C1GALT1C1
552.9575499	-0.034141505	0.231931522	-0.14720511	0.882970127	0.999467288	ENSG00000110514	MADD
549.9942864	-0.46061023	0.290616414	-1.584942242	0.112979458	0.923922314	ENSG00000146574	CCZ1B
551.2263044	-0.100176923	0.229012314	-0.437430289	0.661799322	0.997568624	ENSG00000149091	DGKZ
552.922118	-0.390932785	0.24003366	-1.628658183	0.1033854	0.92169898	ENSG00000172331	BPGM
554.173693	-0.026166604	0.368467314	-0.071014724	0.943386037	0.999527751	ENSG00000141562	NARF
521.8002212	0.684508403	1.080085364	0.633753985	0.526241387	0.99483168	ENSG00000029559	IBSP
559.6504138	0.002296884	0.34068823	0.006741893	0.994620788	0.999527751	ENSG00000198146	ZNF770
553.014919	0.471372501	0.323758988	1.455936416	0.145410198	0.935644597	ENSG00000135905	DOCK10
548.0107164	-0.032180167	0.210804408	-0.152654144	0.878671024	0.999467288	ENSG00000145293	ENOPH1
547.5628669	-0.25962005	0.229840487	-1.129566218	0.258659052	0.962465655	ENSG00000106554	CHCHD3
544.7082864	-0.378657749	0.262811962	-1.440793432	0.14964305	0.936396974	ENSG00000179526	SHARPIN
548.0111941	-0.125089305	0.238466685	-0.524556735	0.59989137	0.99483168	ENSG00000148925	BTBD10
548.914453	-0.062084399	0.215313663	-0.288343981	0.773083444	0.999467288	ENSG00000123213	NLN
553.9684528	0.350733398	0.259469854	1.351730819	0.176461442	0.946278819	ENSG00000153207	AHCTF1
546.0911353	-0.183019471	0.206535411	-0.886140881	0.375541616	0.982609538	ENSG00000175467	SART1
548.3836938	0.469118498	0.264439827	1.774008489	0.076061746	0.897052166	ENSG00000119707	RBM25
549.0783168	-0.043450981	0.228189097	-0.190416551	0.848982729	0.999467288	ENSG00000171262	FAM98B
543.68952	-0.195882932	0.267781808	-0.731502013	0.464472576	0.99135833	ENSG00000076604	TRAF4
543.5077928	-0.571892591	0.330830515	-1.728657317	0.083870445	0.901307093	ENSG00000175130	MARCKSL1
560.5660922	0.2509901	0.380290774	0.659995237	0.509256886	0.993513219	ENSG00000183337	BCOR
546.7365454	-0.228523102	0.232626709	-0.982359693	0.325922676	0.976026345	ENSG00000125445	MRPS7
543.9523293	-0.130792212	0.246268532	-0.53109592	0.595352312	0.99483168	ENSG00000254986	DPP3

548.0013277	-0.242479497	0.200955489	-1.20663286	0.227573566	0.958865115	ENSG00000178988	MRFAP1L1
546.321403	0.147187962	0.243713719	0.603937941	0.545884903	0.99483168	ENSG00000169062	UPF3A
548.3571697	-0.286870419	0.225452933	-1.272418215	0.203224566	0.954310328	ENSG00000119401	TRIM32
544.9812494	0.680301796	0.546057803	1.245842093	0.212822373	0.954310328	ENSG00000136108	CKAP2
544.8388039	-0.091087641	0.23860071	-0.381757627	0.702641152	0.998869317	ENSG00000185104	FAF1
542.5803659	-0.201954657	0.2407788	-0.838755975	0.401606261	0.988592117	ENSG00000101079	NDRG3
550.8834278	-0.04148197	0.222582233	-0.18636694	0.852157016	0.999467288	ENSG00000115808	STRN
552.3461489	-0.157709027	0.22259969	-0.708487182	0.478642769	0.99135833	ENSG00000172775	FAM192A
548.2549419	0.130951062	0.202892417	0.645421171	0.518654287	0.993513219	ENSG00000158435	CNOT11
558.7546938	-0.560254041	0.316361139	-1.770931922	0.076572023	0.897052166	ENSG00000137193	PIM1
543.9245452	-0.074768475	0.274956122	-0.271928753	0.785676802	0.999467288	ENSG00000150768	DLAT
544.0655569	0.111023021	0.247377819	0.448799418	0.653576359	0.997527562	ENSG00000135452	TSPAN31
540.8898902	0.795592883	0.270026838	2.946347441	0.00321551	0.408098513	ENSG00000184584	TMEM173
544.2823023	-0.286018207	0.225425459	-1.268792834	0.204514962	0.954310328	ENSG00000185800	DMWD
544.4566511	-0.086030855	0.247505224	-0.347592078	0.728146556	0.999467288	ENSG00000054611	TBC1D22A
544.7136078	0.027030324	0.20663665	0.13081089	0.895924908	0.999467288	ENSG00000116752	BCAS2
546.0967092	0.152877721	0.200243596	0.763458726	0.445189868	0.99135833	ENSG00000178691	SUZ12
543.7862254	0.228217885	0.240926201	0.947252248	0.343510257	0.977691382	ENSG00000174915	PTDSS2
544.7996752	0.053018196	0.227487782	0.233059532	0.815715178	0.999467288	ENSG00000111802	TDP2
544.9057019	-0.209663015	0.237872492	-0.88140925	0.378096353	0.982609538	ENSG00000144591	GMPPA
543.9021866	-0.103255078	0.230774651	-0.447428162	0.654565946	0.997568624	ENSG00000166164	BRD7
534.2474359	-0.270647244	0.279893468	-0.966965203	0.333561429	0.976026345	ENSG00000166925	TSC22D4
548.9962777	0.467319371	0.264025176	1.769980338	0.076730416	0.897052166	ENSG00000054983	GALC
539.0563286	-0.136697583	0.284254358	-0.480898811	0.630588418	0.99483168	ENSG00000214194	SMIM30
547.2770607	-0.038460759	0.246339054	-0.156129359	0.875931057	0.999467288	ENSG00000159459	UBR1
545.4511719	-0.252990245	0.355329212	-0.711988309	0.476472016	0.99135833	ENSG00000197530	MIB2
543.5880488	0.016499682	0.210394939	0.078422427	0.937492034	0.999527751	ENSG00000136950	ARPC5L
539.5092525	0.057021726	0.25364688	0.224807519	0.822129016	0.999467288	ENSG00000173757	STAT5B
560.3926408	-0.100840166	0.386709086	-0.260764925	0.794273794	0.999467288	ENSG00000166401	SERPINB8
540.6522222	-0.136616266	0.240281416	-0.568567758	0.569649511	0.99483168	ENSG00000006712	PAF1
543.1635652	0.179866018	0.214820269	0.837286064	0.402431791	0.988658335	ENSG00000166855	CLPX
545.0529255	-0.012166434	0.228768595	-0.053182274	0.957586679	0.999527751	ENSG00000163960	UBXN7
532.284581	-0.382278245	0.351743498	-1.086809699	0.27712092	0.962465655	ENSG00000175600	SUGCT
546.1458693	0.119991484	0.225759015	0.531502514	0.5950706	0.99483168	ENSG00000174840	PDE12
541.8627462	-0.296920434	0.245800505	-1.207973248	0.227057553	0.958865115	ENSG00000127125	PPCS
539.2271398	-0.388874077	0.308476324	-1.260628601	0.207442689	0.954310328	ENSG00000150867	PIP4K2A
546.8601308	-0.435017714	0.255996175	-1.699313335	0.089260161	0.910413547	ENSG00000227081	AC005912.1
545.5375552	-0.409083987	0.241166011	-1.696275461	0.089833725	0.910413547	ENSG00000172765	TMCC1
538.9913047	-0.109617511	0.22918139	-0.478300229	0.632436533	0.994860874	ENSG00000167674	HDGFL2
535.3877982	0.14713155	0.39473559	0.372734442	0.709346099	0.99900742	ENSG00000178573	MAF
546.7224647	0.159928726	0.243077313	0.657933578	0.510580813	0.993513219	ENSG00000134744	TUT4
536.0197674	-0.536668805	0.330753024	-1.622566587	0.104682088	0.92169898	ENSG00000145494	NDUFS6
542.8054164	0.035643227	0.201603209	0.176798907	0.859666348	0.999467288	ENSG00000136504	KAT7
540.1634252	0.451038043	0.244718764	1.84308729	0.065316267	0.868202292	ENSG00000162604	TM2D1
543.8998817	-0.182623465	0.247049277	-0.739218779	0.45977416	0.99135833	ENSG00000170185	USP38
537.9299659	-0.178966133	0.259770034	-0.688940639	0.490860624	0.993513219	ENSG00000213523	SRA1
546.3627238	-0.309334645	0.241126119	-1.282874896	0.199535909	0.954310328	ENSG00000166225	FRS2
539.5570626	-0.348000988	0.375293713	-0.927276358	0.353783066	0.977691382	ENSG00000198816	ZNF358
543.7898217	-0.062255828	0.297867857	-0.209004856	0.834444448	0.999467288	ENSG00000225648	SBDSP1
545.4120684	0.323500492	0.231144347	1.399560476	0.161644977	0.943223078	ENSG00000159593	NAE1
535.8653505	-0.02160019	0.262043846	-0.082429677	0.934305038	0.999527751	ENSG00000205352	PRR13
548.9453704	0.375601656	0.28573232	1.314522825	0.188670322	0.949094286	ENSG00000183495	EP400
539.9973415	-0.299094185	0.273508134	-1.093547678	0.274153408	0.962465655	ENSG00000198728	LDB1
536.6487616	-0.252349455	0.260666786	-0.968092095	0.332998377	0.976026345	ENSG00000266472	MRPS21
534.3658285	-0.076315504	0.324965237	-0.234842054	0.814331326	0.999467288	ENSG00000011638	TMEM159
543.4743942	0.285128098	0.27206205	1.048025987	0.294626634	0.970706863	ENSG00000177728	TMEM94
541.5582632	-0.256271249	0.229999231	-1.114226546	0.26518201	0.962465655	ENSG00000101052	IFT52
538.4134884	0.055281058	0.253043238	0.218464869	0.827066927	0.999467288	ENSG00000172890	NADSYN1
547.9395808	-0.178663553	0.376271481	-0.474826188	0.634910865	0.995506752	ENSG00000171310	CHST11
534.7321509	-0.184665237	0.256345709	-0.720375768	0.471293666	0.99135833	ENSG00000077684	JADE1
537.6942877	-0.165289851	0.253092302	-0.653081306	0.513703863	0.993513219	ENSG00000187954	CYHR1
538.771297	0.18830382	0.201061649	0.936547673	0.348991279	0.977691382	ENSG00000128607	KLHDC10
535.8186741	0.692107771	0.447757084	1.545721544	0.122171832	0.923922314	ENSG00000079215	SLC1A3
541.7830179	0.270418533	0.260301526	1.038866494	0.298866831	0.972186492	ENSG00000105778	AVL9

538.2709589	-0.184462708	0.227643375	-0.810314413	0.417759494	0.990226751	ENSG00000076321	KLHL20
540.0112204	-0.468410827	0.267388291	-1.751800069	0.079808192	0.897052166	ENSG00000100842	EFS
542.5619907	0.273393985	0.242875805	1.125653439	0.260312239	0.962465655	ENSG00000198646	NCOA6
533.1226194	0.397515145	0.297827712	1.334715102	0.181969609	0.949094286	ENSG00000164619	BMPER
537.3416259	0.287886603	0.2599936	1.107283424	0.268171409	0.962465655	ENSG00000065923	SLC9A7
541.4801266	0.027313748	0.244507064	0.111709443	0.911053792	0.999527751	ENSG00000139372	TDG
539.0631692	-0.375209065	0.215292984	-1.742783523	0.081371436	0.897807954	ENSG00000168495	POLR3D
542.973885	0.321552379	0.287622505	1.117966686	0.263581222	0.962465655	ENSG00000167779	IGFBP6
536.6728883	-0.114893279	0.225158831	-0.510276584	0.609857705	0.99483168	ENSG00000176623	RMDN1
533.9395646	-0.014413599	0.365267025	-0.039460445	0.968523289	0.999527751	ENSG00000075239	ACAT1
547.7219906	0.840922491	0.557244288	1.509073327	0.131280045	0.930361967	ENSG00000152256	PDK1
555.6898518	-2.291105239	0.834056662	-2.746941956	0.006015378	0.499617804	ENSG00000112559	MDFI
530.3627968	1.033360244	0.348713138	2.963353346	0.00304307	0.403220772	ENSG00000151892	GFRA1
536.6116535	0.250171699	0.2087898	1.198198855	0.230839611	0.959575179	ENSG00000198818	SFT2D1
537.6729925	0.841062129	0.456314507	1.843163249	0.065305179	0.868202292	ENSG00000091129	NRCAM
537.0249237	-0.069048066	0.206906105	-0.33371691	0.738593188	0.999467288	ENSG00000105202	FBL
538.694475	0.09964534	0.273741636	0.364012363	0.715848778	0.99900742	ENSG00000157450	RNF111
534.9202088	-0.117334096	0.206044575	-0.569459769	0.569044166	0.99483168	ENSG00000165861	ZFYVE1
537.211463	0.810847418	0.937966447	0.8644738	0.387327667	0.986799595	ENSG00000114268	PFKFB4
534.2645703	-0.161675413	0.265219082	-0.609591931	0.542132158	0.99483168	ENSG00000164615	CAMLG
539.2929174	0.086122306	0.224790921	0.383121815	0.701629445	0.998869317	ENSG00000086589	RBM22
536.8713545	0.698297538	0.273961934	2.548885268	0.010806785	0.638240244	ENSG00000155561	NUP205
536.3111229	-0.023944451	0.228280019	-0.104890702	0.916462537	0.999527751	ENSG00000114520	SNX4
531.5752758	0.126286277	0.255907116	0.49348482	0.621670063	0.99483168	ENSG00000122335	SERAC1
528.8537325	-0.23402453	0.269811551	-0.867362904	0.385743201	0.986238739	ENSG00000138175	ARL3
533.0374013	0.331327937	0.2380002	1.392133019	0.163882121	0.943223078	ENSG00000196187	TMEM63A
537.2866864	0.282955758	0.257834764	1.097430593	0.272453206	0.962465655	ENSG00000176142	TMEM39A
535.9716051	-0.228133486	0.296510088	-0.769395361	0.441658642	0.99135833	ENSG00000141858	SAMD1
536.4888749	0.186300938	0.212711167	0.875839951	0.381117048	0.984700093	ENSG00000137200	CMTR1
533.4816492	-0.149036678	0.210195312	-0.70903902	0.478300263	0.99135833	ENSG00000176986	SEC24C
539.1705867	-0.050164758	0.283204389	-0.177132699	0.859404158	0.999467288	ENSG00000107560	RAB11FIP2
527.9629682	-0.414746306	0.235197539	-1.763395607	0.07783379	0.897052166	ENSG00000051128	HOMER3
539.5246353	0.045917089	0.276630588	0.165987026	0.86816716	0.999467288	ENSG00000164151	ICE1
530.7332114	-0.406512733	0.29600551	-1.373328264	0.16965032	0.944422693	ENSG00000128309	MPST
535.2977499	0.291776122	0.37372095	0.780732583	0.434959793	0.991301167	ENSG00000149483	TMEM138
534.1361121	0.537272699	0.231205902	2.323784536	0.020137045	0.73997555	ENSG00000125676	THOC2
544.8606749	0.124871672	0.415025412	0.300877172	0.763508158	0.999467288	ENSG00000155189	AGPAT5
531.8039872	-0.299296664	0.207124133	-1.445011068	0.148454777	0.936396974	ENSG00000180901	KCTD2
535.4704237	-0.200644083	0.311978677	-0.643133963	0.520137179	0.993513219	ENSG00000177030	DEAF1
540.0206965	-0.347654236	0.264734041	-1.313220748	0.189108574	0.949094286	ENSG00000224470	ATXN1L
526.0784855	-0.619776045	0.472890167	-1.310613096	0.189988512	0.949094286	ENSG00000159423	ALDH4A1
532.5381655	0.130004885	0.205288413	0.633279214	0.526551324	0.99483168	ENSG00000275700	AATF
533.695865	-0.249522491	0.256712529	-0.971991866	0.331054595	0.976026345	ENSG00000163597	SNHG16
533.3380686	-0.213240631	0.207933163	-1.025524871	0.305115596	0.973427483	ENSG00000130338	TULP4
536.7250933	-0.263378592	0.254251332	-1.035898572	0.300249464	0.972186492	ENSG00000109787	KLF3
544.2229315	0.482722427	0.428304901	1.127053242	0.259719971	0.962465655	ENSG00000175414	ARL10
529.5287626	0.204068401	0.308991795	0.660433074	0.508975953	0.993513219	ENSG00000256043	CTSO
533.7386898	1.351247623	0.959387627	1.40844804	0.158998442	0.939523519	ENSG00000249992	TMEM158
530.544167	-0.001940912	0.223958933	-0.008666374	0.993085321	0.999527751	ENSG00000069248	NUP133
536.4960914	-0.035128131	0.249215389	-0.140954902	0.887905569	0.999467288	ENSG00000167721	TSR1
533.9566474	0.114613855	0.229740399	0.498884199	0.617860966	0.99483168	ENSG00000135968	GCC2
527.2597751	-0.362888005	0.240739164	-1.507390815	0.131710512	0.931891739	ENSG00000100764	PSMC1
530.3395948	-0.107252295	0.223278554	-0.480351979	0.630977134	0.99483168	ENSG00000103111	MON1B
531.7027049	0.303330182	0.259611291	1.168401347	0.242644912	0.962465655	ENSG00000172469	MANEA
531.7532084	0.230148085	0.271142156	0.848809672	0.395987207	0.988378925	ENSG00000134049	IER3IP1
530.7329419	-0.405157649	0.330707609	-1.225123454	0.220528709	0.956884001	ENSG00000125170	DOK4
531.1231963	-0.170628023	0.216742599	-0.787238061	0.43114252	0.991301167	ENSG00000053770	AP5M1
524.9424472	-0.028901548	0.28096276	-0.102866117	0.91806923	0.999527751	ENSG00000136717	BIN1
524.8248695	-0.615492723	0.359020133	-1.71436827	0.086461112	0.901307093	ENSG00000213722	DDAH2
525.167676	-0.116527166	0.267550171	-0.435533888	0.663174947	0.997568624	ENSG00000147669	POLR2K
528.3547664	-0.367894352	0.213852981	-1.720314348	0.085375317	0.901307093	ENSG00000063322	MED29
530.3088215	-0.465907033	0.247922525	-1.879244469	0.06021112	0.858996918	ENSG00000143486	EIF2D
532.354798	-0.343903671	0.258405209	-1.330869728	0.18323188	0.949094286	ENSG00000066084	DIP2B
519.4806724	0.935251725	0.360062936	2.59746736	0.009391405	0.621716014	ENSG00000081923	ATP8B1

532.0954629	-0.081385605	0.21143671	-0.384917101	0.700298835	0.998869317	ENSG00000131013	PPIL4
530.4060753	-0.599615355	0.363944915	-1.647544257	0.099446227	0.921147998	ENSG00000120885	CLU
533.3904904	-0.679050273	0.330942638	-2.051866984	0.040182591	0.795004104	ENSG00000164188	RANBP3L
530.8662702	0.241059429	0.239222605	1.007678304	0.313608924	0.973633446	ENSG00000198689	SLC9A6
531.6349059	-0.176667936	0.256028314	-0.690032805	0.490173558	0.993513219	ENSG00000135108	FBXO21
521.9881813	0.132228347	0.264388496	0.500128976	0.616984265	0.99483168	ENSG00000021355	SERPINB1
527.0083866	-0.055618324	0.253352975	-0.219528996	0.826237995	0.999467288	ENSG00000115514	TXNDC9
527.9648757	-0.022236322	0.229610823	-0.096843528	0.922850656	0.999527751	ENSG00000114742	WDR48
525.0512804	0.024102222	0.236816943	0.10177575	0.918934674	0.999527751	ENSG00000163702	IL17RC
524.8544094	-0.046935718	0.266467044	-0.176140798	0.86018333	0.999467288	ENSG00000136213	CHST12
519.5010436	-0.787732952	0.345490163	-2.280044517	0.022605048	0.744905651	ENSG00000063854	HAGH
526.0545413	0.067388867	0.202301886	0.333110423	0.739050932	0.999467288	ENSG00000154222	CC2D1B
524.0794353	-0.269692781	0.250064468	-1.07849301	0.2808138	0.963267117	ENSG00000204272	NBDY
522.9309938	-0.049915445	0.27833593	-0.179335254	0.857674467	0.999467288	ENSG00000152219	ARL14EP
528.6283124	0.136501595	0.2215919	0.616004445	0.537891586	0.99483168	ENSG00000085721	RRN3
522.7926627	-0.263793182	0.281384477	-0.937483065	0.348510131	0.977691382	ENSG00000170892	TSEN34
531.9243969	0.059845307	0.265472211	0.225429648	0.821645049	0.999467288	ENSG00000117625	RCOR3
526.5230997	-0.219894431	0.247641477	-0.887954771	0.374565081	0.982609538	ENSG00000264522	OTUD7B
523.4194044	0.217744116	0.233885845	0.930984587	0.351861537	0.977691382	ENSG00000169871	TRIM56
525.3589033	-0.106710515	0.206926221	-0.51569354	0.606068467	0.99483168	ENSG00000167699	GLOD4
522.88202	0.121818568	0.240316422	0.506909043	0.61221864	0.99483168	ENSG00000214046	SMIM7
531.426412	0.324912267	0.262302883	1.238691178	0.215459887	0.954310328	ENSG00000143442	POGZ
523.3670298	-0.115105295	0.20958364	-0.549209354	0.582861786	0.99483168	ENSG00000117395	EBNA1BP2
524.9099932	-0.302683385	0.298620622	-1.013605097	0.310771225	0.973427483	ENSG00000114779	ABHD14B
527.1336995	-0.107259462	0.353159933	-0.303713565	0.761346123	0.999467288	ENSG00000033170	FUT8
530.1478163	-0.130495449	0.244931575	-0.532783285	0.594183606	0.99483168	ENSG00000154114	TBCEL
520.6074639	0.552876447	0.394721834	1.400673587	0.161311705	0.942964826	ENSG00000113594	LIFR
530.2429759	-0.097291462	0.245576471	-0.396175828	0.691975322	0.998869317	ENSG00000188419	CHM
525.7961209	0.297501503	0.220305414	1.350404866	0.176886151	0.946540546	ENSG00000239305	RNF103
524.5201276	0.170530026	0.240911552	0.707853254	0.479036392	0.99135833	ENSG00000128585	MKLN1
522.9229589	-0.193005056	0.214044742	-0.901704263	0.367213988	0.981269214	ENSG00000139613	SMARCC2
527.7300472	0.26534365	0.22768084	1.165419321	0.2438493	0.962465655	ENSG00000124789	NUP153
504.2182869	0.620857403	1.257755077	0.493623452	0.621572135	0.99483168	ENSG00000163235	TGFA
526.3468344	-0.119859352	0.222672498	-0.538276408	0.590386237	0.99483168	ENSG00000104343	UBE2W
520.9378986	0.238130443	0.229394115	1.038084361	0.299230782	0.972186492	ENSG00000175745	NR2F1
529.8241665	-0.199094199	0.249002475	-0.799567152	0.423961626	0.991301167	ENSG00000161021	MAML1
525.5953969	0.203037784	0.262126382	0.774579736	0.438588035	0.991301167	ENSG00000124571	XPO5
523.6230121	-0.171353779	0.230454166	-0.743548197	0.457149853	0.99135833	ENSG00000140521	POLG
523.5119714	-0.197807693	0.206977301	-0.95569752	0.339225076	0.977691382	ENSG00000146729	NIPSNAP2
527.4862785	0.470676234	0.261315304	1.801181276	0.071674313	0.884210289	ENSG00000143624	INTS3
525.2901966	0.101554466	0.24472661	0.414971082	0.678163072	0.998869317	ENSG00000167258	CDK12
520.3365438	-0.197393646	0.260697913	-0.757173864	0.448945706	0.99135833	ENSG00000258289	CHURC1
525.1499638	-0.863761557	0.386526938	-2.234673633	0.025438783	0.759116406	ENSG00000135424	ITGA7
522.4778869	-0.130275417	0.202752735	-0.642533464	0.520526869	0.993513219	ENSG00000137210	TMEM14B
519.7295796	-0.265804995	0.259779193	-1.02319586	0.30621525	0.973427483	ENSG00000074964	ARHGEF10L
522.6452698	0.110490545	0.245525047	0.450017407	0.652697889	0.997471445	ENSG00000103160	HSDL1
529.3496929	0.223846652	0.275263165	0.813209612	0.416097889	0.990226751	ENSG00000186594	MIR22HG
515.0579624	-0.468745718	0.270530525	-1.732690678	0.083150662	0.901307093	ENSG00000185052	SLC24A3
522.218532	-0.018627221	0.237818394	-0.0783254	0.937569213	0.999527751	ENSG00000116212	LRRC42
518.576936	-0.047110964	0.241512499	-0.195066361	0.845340996	0.999467288	ENSG00000104946	TBC1D17
519.4036858	-0.230576443	0.2463253	-0.936064803	0.349239824	0.977691382	ENSG00000130150	MOSPD2
527.0702032	0.375922537	0.278101411	1.351746239	0.176456508	0.946278819	ENSG00000122257	RBBP6
526.8649886	0.015709346	0.252544771	0.062204204	0.950400215	0.999527751	ENSG00000125484	GTF3C4
521.4264408	-0.093871216	0.274717513	-0.341700879	0.73257601	0.999467288	ENSG00000164330	EBF1
525.7839253	-0.1222947	0.250520004	-0.488163414	0.625434097	0.99483168	ENSG00000113742	CPEB4
522.7206455	-0.123324944	0.257298556	-0.479306787	0.631720395	0.99483168	ENSG00000198825	INPP5F
512.8328549	-0.078168239	0.36135123	-0.21632205	0.828736722	0.999467288	ENSG00000198947	DMD
526.023679	-0.221933812	0.361451387	-0.614007361	0.539210463	0.99483168	ENSG00000182168	UNC5C
520.7689626	-0.074021252	0.357394616	-0.207113505	0.835921215	0.999467288	ENSG00000065485	PDIA5
520.9414982	-0.547717075	0.284375679	-1.926033468	0.054100195	0.847598083	ENSG00000124733	MEA1
521.106018	0.493702067	0.24927334	1.980565056	0.047640071	0.827997046	ENSG00000177707	NECTIN3
519.4745298	0.092015022	0.231922456	0.396749085	0.6915525	0.998869317	ENSG00000168522	FNTA
521.4650932	0.14181632	0.230835687	0.614360467	0.538977153	0.99483168	ENSG00000140259	MFAP1
521.4589471	0.447026737	0.214831614	2.080823805	0.037450036	0.790190505	ENSG00000102908	NFAT5

519.9249717	0.006855572	0.243912692	0.028106664	0.977577079	0.999527751	ENSG00000112584	FAM120B	
515.4489294	0.000630471	0.231189287	0.002727077	0.99782411	0.999527751	ENSG00000080189	SLC35C2	
519.9758061	-0.102048929	0.205843819	-0.495759014	0.620064445	0.99483168	ENSG00000148481	MINDY3	
521.7038057	0.429013379	0.362228041	1.184373737	0.236265152	0.962465655	ENSG00000183696	UPP1	
524.1941192	0.245101694	0.264546569	0.926497344	0.354187577	0.977691382	ENSG00000151067	CACNA1C	
521.4826436	-0.014707762	0.217950964	-0.067481979	0.946198008	0.999527751	ENSG00000140992	PDPK1	
523.0604503	0.013418392	0.327263102	0.041001848	0.967294422	0.999527751	ENSG00000025772	TOMM34	
517.7766903	0.572522042	0.318731058	1.796254327	0.072454078	0.885710197	ENSG00000159063	ALG8	
516.5651856	-0.137986796	0.250139397	-0.551639596	0.581195302	0.99483168	ENSG00000245910	SNHG6	
516.3797116	-0.032413819	0.240213509	-0.134937537	0.892661261	0.999467288	ENSG00000111726	CMAS	
514.3991535	0.195554996	0.247692459	0.789507265	0.429815585	0.991301167	ENSG00000108592	FTSJ3	
517.3556385	-0.220902214	0.221615409	-0.996781833	0.318870418	0.974768742	ENSG00000158941	CCAR2	
516.9705502	-1.167125738	0.343433535	-3.398403527	0.000677804	0.195790358	ENSG00000172201	ID4	
518.561821	0.055393476	0.203838165	0.271752228	0.785812539	0.999467288	ENSG00000218739	CEBPZOS	
518.2616251	-0.554554278	0.257851056	-2.150676775	0.031501721	0.775963115	ENSG00000171680	PLEKHG5	
519.1124861	0.045564332	0.211397488	0.215538665	0.82934737	0.999467288	ENSG00000136518	ACTL6A	
518.3377932	0.137790045	0.207079786	0.665395923	0.505797292	0.993513219	ENSG00000242802	AP5Z1	
515.095496	-0.128083866	0.248461425	-0.515508056	0.606198042	0.99483168	ENSG00000136270	TBRG4	
519.2821636	-0.004317389	0.204274048	-0.021135279	0.983137743	0.999527751	ENSG00000198964	SGMS1	
519.1885574	-0.19276988	0.250587908	-0.769270481	0.441732757	0.99135833	ENSG00000115363	EVA1A	
519.5977239	0.317657227	0.244549129	1.298950553	0.193960899	0.950624691	ENSG00000078142	PIK3C3	
519.1377154	-0.088189885	0.261379064	-0.337402253	0.735813687	0.999467288	ENSG00000129933	MAU2	
514.336556	-0.360728399	0.221394228	-1.629348706	0.103239221	0.92169898	ENSG00000152082	MZT2B	
520.091256	0.368023159	0.265211391	1.387659698	0.165240694	0.943223078	ENSG00000153914	SREK1	
510.9290193	-0.179407326	0.234148067	-0.766213143	0.44354949	0.99135833	ENSG00000120306	CYSTM1	
516.9999568	0.124604977	0.204630999	0.608925226	0.542574003	0.99483168	ENSG00000138398	PPIG	
520.2416699	0.090064427	0.262292195	0.343374408	0.73131681	0.999467288	ENSG00000172869	DMXL1	
518.0694993	-0.104199871	0.197001398	-0.528929604	0.596854285	0.99483168	ENSG00000172661	WASHC2C	
516.767122	-0.1399528	0.206838654	-0.676627881	0.498642085	0.993513219	ENSG00000143622	RIT1	
515.3667799	0.023234618	0.248866559	0.093361754	0.925616174	0.999527751	ENSG00000068745	IP6K2	
519.2649249	-0.276535135	0.33569694	-0.823764242	0.410073529	0.990226751	ENSG00000197724	PHF2	
516.7859363	0.130183271	0.223185191	0.583297085	0.559693325	0.99483168	ENSG00000178904	DPY19L3	
514.2753669	0.062460693	0.248675302	0.251173688	0.801679827	0.999467288	ENSG00000213024	NUP62	
514.2109658	0.292822391	0.216290366	1.353839266	0.175787663	0.946278819	ENSG00000130529	TRPM4	
517.6546158	-0.030942026	0.237165851	-0.130465773	0.896197932	0.999467288	ENSG00000140694	PARN	
518.2902139	-0.463054119	0.349911046	-1.323348101	0.185719642	0.949094286	ENSG00000169991	IFFO2	
519.4616306	-0.015431864	0.268386402	-0.05749868	0.954147958	0.999527751	ENSG00000130559	CAMSAP1	
514.1551627	-0.222009022	0.236132478	-0.940188419	0.347120921	0.977691382	ENSG00000119865	CNRIP1	
514.1387891	-0.258672953	0.47424705	-0.545439246	0.585451455	0.99483168	ENSG00000150540	HNMT	
504.626047	0.014682221	0.350515659	0.04188749	0.96658839	0.999527751	ENSG00000092010	PSME1	
512.8582099	-0.014910162	0.281208477	-0.053021738	0.957714588	0.999527751	ENSG00000174684	B4GAT1	
514.8163543	-0.046750982	0.236162009	-0.197961486	0.843075197	0.999467288	ENSG00000133131	MORC4	
517.2009207	0.374172573	0.267793725	1.397241751	0.162340886	0.943223078	ENSG00000198862	LTN1	
519.5449178	0.469816453	0.256721676	1.830061487	0.067240745	0.87420149	ENSG00000033867	SLC4A7	
520.3923767	0.13710216	0.247334312	0.554319208	0.579360407	0.99483168	ENSG00000163608	NEPRO	
515.2131163	0.123019148	0.207562771	0.59268407	0.553392605	0.99483168	ENSG00000102081	FMR1	
514.5175269	-0.261229662	0.297525185	-0.878008568	0.379939069	0.983585589	ENSG00000165406		8-Mar
512.8577825	-0.031984448	0.258448212	-0.123755735	0.901508681	0.999527751	ENSG00000164808	SPIDR	
515.6810571	-0.008791631	0.253225368	-0.034718605	0.972304125	0.999527751	ENSG00000137522	RNF121	
514.5179215	0.074276088	0.254816325	0.291488736	0.770677557	0.999467288	ENSG00000149313	AASDHPPT	
515.7585708	-0.05751869	0.270820178	-0.212387017	0.831805114	0.999467288	ENSG00000174738	NR1D2	
518.7879326	0.574244404	0.403024939	1.424835905	0.154204641	0.939432741	ENSG00000113369	ARRDC3	
508.934577	0.338880936	0.213762978	1.585311638	0.112895546	0.923922314	ENSG00000180773	SLC36A4	
513.333216	-0.183643625	0.220062141	-0.834508037	0.40399476	0.988927059	ENSG00000117475	BLZF1	
513.9869395	-0.006354618	0.219576801	-0.028940296	0.976912207	0.999527751	ENSG00000114209	PDCD10	
510.168874	-0.471722916	0.384199184	-1.22780822	0.219518969	0.956884001	ENSG00000167797	CDK2AP2	
514.3786212	-0.038881995	0.247283589	-0.157236457	0.875058497	0.999467288	ENSG00000141385	AFG3L2	
510.0242209	0.213603999	0.282724797	0.755519154	0.449937544	0.99135833	ENSG00000072786	STK10	
516.1021524	0.105958503	0.218008018	0.486030305	0.62694568	0.99483168	ENSG00000146476	ARMT1	
512.701424	0.328476167	0.267698424	1.227038106	0.219808269	0.956884001	ENSG00000096063	SRPK1	
514.5518221	0.021838547	0.27140043	0.080466148	0.935866519	0.999527751	ENSG00000103148	NPRL3	
508.6185115	0.042405686	0.229890362	0.18446048	0.853652228	0.999467288	ENSG00000160753	RUSC1	
521.0742197	-0.597905686	0.39936046	-1.497157945	0.134352165	0.933741583	ENSG00000183496	MEX3B	
508.2078886	-0.336919138	0.257000793	-1.310965364	0.189869466	0.949094286	ENSG00000100418	DESI1	

506.8003826	-0.039530491	0.321636173	-0.12290437	0.902182825	0.999527751	ENSG00000086189	DIMT1
511.8559599	-0.584115979	0.253850465	-2.30102387	0.021390282	0.73997555	ENSG00000007866	TEAD3
511.4786646	-0.120003824	0.307444827	-0.390326372	0.696295225	0.998869317	ENSG00000176490	DIRAS1
500.4471294	-0.525845069	0.380004567	-1.383786182	0.166423935	0.943223078	ENSG00000280143	AP000892.3
506.4541853	-0.320647182	0.275811485	-1.162559211	0.245008388	0.962465655	ENSG00000147684	NDUFB9
516.1527046	-0.494920879	0.213990651	-2.31281543	0.020732789	0.73997555	ENSG00000184110	EIF3C
502.8083055	-0.058551661	0.344118454	-0.170149726	0.864892388	0.999467288	ENSG00000072422	RHOBTB1
513.8882984	0.183846171	0.304950799	0.602871584	0.546594121	0.99483168	ENSG00000171824	EXOSC10
508.4050705	0.096992545	0.25721515	0.377087216	0.706108791	0.99900742	ENSG00000178467	P4HTM
512.9403326	0.225068175	0.291138939	0.773061053	0.439486249	0.991301167	ENSG00000084774	CAD
512.0599661	0.128346662	0.429395213	0.298901008	0.765015578	0.999467288	ENSG00000177426	TGIF1
509.4574518	0.088113118	0.211512348	0.416586166	0.67698113	0.998869317	ENSG00000196363	WDR5
509.2530184	-0.488089678	0.229745173	-2.124482841	0.033629789	0.775963115	ENSG00000169189	NSMCE1
511.072003	-0.00056371	0.215433004	-0.002616636	0.997912229	0.999527751	ENSG00000196428	TSC22D2
509.3261439	-0.071071665	0.265223936	-0.267968518	0.788723552	0.999467288	ENSG00000163904	SENP2
510.7862523	-0.44839004	0.247562588	-1.811218901	0.070106973	0.878416974	ENSG00000109790	KLHL5
516.4178785	-0.153554542	0.290331113	-0.528894543	0.596878607	0.99483168	ENSG00000196072	BLOC1S2
493.0388942	-0.528156557	0.890215323	-0.593290796	0.552986558	0.99483168	ENSG00000103044	HAS3
507.0277725	0.122534669	0.238438568	0.513904566	0.607318715	0.99483168	ENSG00000179832	MROH1
503.8508532	-0.241447156	0.227273261	-1.062364991	0.288070021	0.966288987	ENSG00000179271	GADD45GIP1
509.4512767	0.377572905	0.218464258	1.728305159	0.083933529	0.901307093	ENSG00000114127	XRN1
511.9715334	0.168851453	0.224596799	0.751798125	0.45217247	0.99135833	ENSG00000110066	KMT5B
503.7286347	-0.155697445	0.251352684	-0.619438165	0.535627746	0.99483168	ENSG00000241839	PLEKHO2
508.0070299	-0.314614339	0.283723088	-1.10887817	0.267482742	0.962465655	ENSG00000187147	RNF220
500.8398731	-0.413286129	0.32915921	-1.255581238	0.209267837	0.954310328	ENSG00000125356	NDUFA1
508.8192469	0.198842118	0.28071747	0.708335388	0.478737006	0.99135833	ENSG00000139163	ETNK1
502.9569205	-0.327356458	0.463534299	-0.706218415	0.48005232	0.99135833	ENSG00000197635	DPP4
501.7343009	-0.250591213	0.248740484	-1.007440401	0.313723185	0.973633446	ENSG00000110442	COMMD9
507.8579907	-0.76279316	0.233669077	-3.264416373	0.001096898	0.243730821	ENSG00000178188	SH2B1
506.3021962	-0.405733719	0.269762049	-1.504042989	0.132570298	0.933741583	ENSG00000004975	DVL2
503.2047468	-0.153130612	0.229842273	-0.66624216	0.505256329	0.993513219	ENSG00000117305	HMGCL
504.9364566	-0.260997153	0.237958379	-1.096818502	0.27272074	0.962465655	ENSG00000096070	BRPF3
513.0554813	0.497104967	0.43144932	1.152174643	0.249249315	0.962465655	ENSG00000170485	NPAS2
507.589317	0.044947738	0.216463359	0.207645943	0.835505429	0.999467288	ENSG00000174165	ZDHHC24
501.0736606	-0.132254263	0.231111452	-0.57225318	0.567150464	0.99483168	ENSG00000105341	DMAC2
509.7672495	0.480190609	0.37447889	1.282290196	0.199740866	0.954310328	ENSG00000181744	C3orf58
497.6290759	-0.146628481	0.529350202	-0.276997119	0.781782318	0.999467288	ENSG00000083720	OXCT1
511.009548	-0.353129428	0.280934801	-1.256980004	0.208760876	0.954310328	ENSG00000176946	THAP4
505.1997662	0.001572264	0.208001028	0.007558924	0.993968909	0.999527751	ENSG00000138614	INTS14
507.826788	0.087071877	0.241717546	0.360221584	0.718681435	0.99900742	ENSG00000070018	LRP6
502.6869344	0.185825981	0.22676328	0.819471219	0.412517616	0.990226751	ENSG00000135090	TAOK3
503.5626475	-0.182379192	0.272979502	-0.668105813	0.504066052	0.993513219	ENSG00000124120	TTPAL
504.7055685	0.302497645	0.258433634	1.170504168	0.241798138	0.962465655	ENSG00000130816	DNMT1
517.8669913	-0.527369206	0.37386285	-1.410595374	0.158363957	0.939464191	ENSG00000183023	SLC8A1
507.6905229	0.320396739	0.215778883	1.48483825	0.137586677	0.934144427	ENSG00000163104	SMARCAD1
516.6604393	0.606878974	0.54543493	1.112651465	0.265858148	0.962465655	ENSG00000196517	SLC6A9
507.5420978	0.130858912	0.213567165	0.612729546	0.540055183	0.99483168	ENSG00000164329	TENT2
504.7993191	-0.680624636	0.209862814	-3.243188355	0.001182	0.244243321	ENSG00000183779	ZNF703
505.7460607	-0.051137585	0.216393593	-0.236317464	0.813186336	0.999467288	ENSG00000118260	CREB1
506.2962578	0.211687932	0.230865405	0.916932235	0.359178151	0.979465649	ENSG00000169375	SIN3A
503.0131578	0.118664597	0.228867519	0.518485968	0.604119248	0.99483168	ENSG00000134077	THUMPD3
499.7326916	-0.54391254	0.438935782	-1.239161999	0.21528551	0.954310328	ENSG00000274180	NATD1
502.1335772	-0.379376515	0.265951468	-1.426487765	0.153727596	0.939432741	ENSG00000203950	RTL8A
507.7907288	-0.259985455	0.322206629	-0.80689046	0.419729596	0.990386301	ENSG00000174996	KLC2
503.7579612	0.012061411	0.214531535	0.056222089	0.955164884	0.999527751	ENSG00000120533	ENY2
500.4898889	1.399438153	0.46715103	2.995686754	0.002738276	0.380278034	ENSG00000262655	SPON1
499.504209	-0.414733592	0.260780574	-1.590354626	0.111754892	0.923922314	ENSG00000182512	GLRX5
497.2334951	-0.266109162	0.310990555	-0.855682457	0.392173447	0.988378925	ENSG00000178605	GTPBP6
501.4234206	-0.183609609	0.218003755	-0.842231406	0.399658438	0.988384114	ENSG00000182872	RBM10
504.2474203	-0.09390601	0.260768244	-0.360112907	0.718762701	0.99900742	ENSG00000156931	VPS8
501.1976947	-0.209422889	0.233163153	-0.898181754	0.369088659	0.981455968	ENSG00000167196	FBXO22
505.9415521	-0.070175441	0.221670423	-0.316575573	0.751565673	0.999467288	ENSG00000086102	NFX1
500.1254121	0.083126686	0.241342772	0.344434121	0.730519832	0.999467288	ENSG00000126214	KLC1
502.1753451	-0.310675993	0.222438695	-1.396681425	0.162509392	0.943223078	ENSG00000109917	ZPR1

501.4289573	0.0004933	0.260928917	0.001890555	0.998491557	0.999527751	ENSG00000143179	UCK2
509.402272	0.175355189	0.322348116	0.543993219	0.586446139	0.99483168	ENSG00000174106	LEMD3
501.7646697	-0.037707267	0.218530971	-0.172548846	0.863006064	0.999467288	ENSG00000132591	ERAL1
502.9406266	0.237285219	0.232038364	1.022612016	0.306491326	0.973427483	ENSG00000128699	ORMDL1
501.5057207	0.008396217	0.2178649	0.038538641	0.969258223	0.999527751	ENSG00000139977	NAA30
495.2953099	-0.37353984	0.306469092	-1.218849957	0.222901146	0.956884001	ENSG00000099804	CDC34
499.3317792	-0.105965977	0.274381984	-0.386198743	0.699349485	0.998869317	ENSG00000109854	HTATIP2
496.2841309	-0.385403769	0.251180726	-1.534368401	0.124939047	0.927794614	ENSG00000142686	C1orf216
502.8388886	0.200295772	0.218456575	0.916867678	0.359211983	0.979465649	ENSG00000081307	UBA5
497.4982593	0.182683676	0.28171649	0.648466392	0.516683341	0.993513219	ENSG00000170653	ATF7
496.5665986	-0.098195572	0.273515729	-0.359012521	0.719585722	0.99900742	ENSG00000103591	AAGAB
500.7516229	-0.138245609	0.209097944	-0.661152406	0.50851458	0.993513219	ENSG00000122376	SHLD2
503.8999398	0.363640936	0.358233947	1.015093459	0.31006128	0.973427483	ENSG00000164654	MIOS
497.764289	-0.193406246	0.252298716	-0.766576416	0.443333402	0.99135833	ENSG00000156411	ATP5MPL
495.9618473	-0.485036882	0.244797902	-1.981376789	0.047549035	0.827997046	ENSG00000086504	MRPL28
502.7793417	-0.453672779	0.273854732	-1.656618368	0.097596656	0.921147998	ENSG00000160360	GPSM1
501.0689276	-0.20404226	0.281548363	-0.724714779	0.468627026	0.99135833	ENSG00000123992	DNPEP
497.3033935	-0.360613936	0.273687749	-1.317610807	0.187633966	0.949094286	ENSG00000108578	BLMH
499.1207756	0.101637784	0.2336572	0.434986742	0.663572051	0.997568624	ENSG00000163875	MEAF6
501.7765184	-0.309012473	0.235238819	-1.313611735	0.188976898	0.949094286	ENSG00000120709	FAM53C
498.0021564	-0.727142656	0.377112872	-1.928183072	0.053832359	0.847598083	ENSG00000153048	CARHSP1
502.744498	1.786771729	1.274277398	1.402184275	0.160860226	0.942134731	ENSG00000134057	CCNB1
501.2090407	-0.216630306	0.275908891	-0.785151594	0.432364692	0.991301167	ENSG00000107185	RGP1
500.9231546	0.213912181	0.223734522	0.956098229	0.339022609	0.977691382	ENSG00000083642	PDS5B
505.2777646	0.087849995	0.250895597	0.350145622	0.726229414	0.999467288	ENSG00000137770	CTDSPL2
508.2627054	0.082744967	0.364031988	0.227301363	0.820189413	0.999467288	ENSG00000177410	ZFAS1
495.0827275	-0.362464017	0.270808173	-1.338453021	0.180748805	0.949094286	ENSG00000099800	TIMM13
495.6569036	-0.310239032	0.275870898	-1.124580499	0.260766841	0.962465655	ENSG00000100413	POLR3H
493.220603	-0.13998311	0.293874743	-0.476335968	0.633835045	0.995376704	ENSG00000253276	CCDC71L
501.4144192	0.358440048	0.24592728	1.45750422	0.144977247	0.935644597	ENSG00000158711	ELK4
496.9293957	0.307637361	0.2127909	1.445726115	0.148254037	0.936396974	ENSG00000105771	SMG9
498.2604017	-0.08842329	0.20243671	-0.436794741	0.662260214	0.997568624	ENSG00000196850	PPTC7
502.6259286	0.260207187	0.288753917	0.901138208	0.36751484	0.981269214	ENSG00000145780	FEM1C
498.0484383	-0.33309743	0.324705463	-1.02584486	0.304964717	0.973427483	ENSG00000162729	IGSF8
500.4032535	-0.08314156	0.271868652	-0.305815177	0.759745372	0.999467288	ENSG00000116273	PHF13
503.9453599	-0.186146461	0.288517731	-0.645182051	0.518809216	0.993513219	ENSG00000065802	ASB1
497.9154801	-0.31866844	0.253996502	-1.254617436	0.209617671	0.954310328	ENSG00000087008	ACOX3
498.8058735	-0.05574233	0.213849902	-0.260661005	0.79435394	0.999467288	ENSG00000154727	GABPA
492.3491625	0.10884968	0.272663162	0.399209336	0.689738965	0.998869317	ENSG00000082146	STRADB
499.7187293	0.249693861	0.289314745	0.863052663	0.388108513	0.986799595	ENSG00000110395	CBL
490.3819736	-0.325979753	0.302750342	-1.076727944	0.281601824	0.963267117	ENSG00000160613	PCSK7
494.9203497	-0.19621622	0.220748905	-0.88886611	0.374075041	0.982609538	ENSG00000230124	ACBD6
497.2295522	-0.130315009	0.203030562	-0.641849225	0.520971084	0.993513219	ENSG00000111962	UST
502.3751025	-0.033486885	0.351733788	-0.095205197	0.924151842	0.999527751	ENSG00000166484	MAPK7
494.2935767	0.117917137	0.239057335	0.493258812	0.621829727	0.99483168	ENSG00000165487	MICU2
495.0890538	-0.075180375	0.200829494	-0.374349272	0.708144479	0.99900742	ENSG00000124532	MRS2
497.6262143	0.104651322	0.237575542	0.440497034	0.65957716	0.997568624	ENSG00000138303	ASCC1
498.4358834	-0.33419558	0.274655239	-1.216782106	0.22368713	0.956884001	ENSG00000103423	DNAJA3
491.6056163	0.005364078	0.239689731	0.022379259	0.982145425	0.999527751	ENSG00000135002	RFK
499.7133737	0.139183197	0.259338345	0.536685764	0.591484691	0.99483168	ENSG00000114861	FOXP1
498.0624376	-0.189027984	0.246797739	-0.765922674	0.443722313	0.99135833	ENSG00000197860	SGTB
494.280773	0.16791787	0.229948195	0.730242175	0.465242167	0.99135833	ENSG00000040531	CTNS
485.262568	-0.57668616	0.286484302	-2.012976471	0.044117105	0.807325344	ENSG00000184254	ALDH1A3
489.1707994	-0.69021749	0.289438303	-2.384679165	0.017094022	0.715620174	ENSG00000197982	C1orf122
492.1145124	-0.158475498	0.249640684	-0.634814388	0.525549479	0.99483168	ENSG00000107829	FBXW4
493.9400622	0.030640017	0.46062251	0.066518713	0.946964859	0.999527751	ENSG00000164970	FAM219A
498.2082831	-0.129746557	0.37649659	-0.344615489	0.730383459	0.999467288	ENSG00000157077	ZFYVE9
488.3395132	0.082411127	0.276943711	0.297573562	0.766028655	0.999467288	ENSG00000124541	RRP36
494.0570123	0.194504098	0.26688259	0.72880025	0.466123858	0.99135833	ENSG00000114988	LMAN2L
490.6518876	0.119298843	0.5533001	0.215613269	0.829289212	0.999467288	ENSG00000127824	TUBA4A
489.4712937	-0.228703393	0.322764331	-0.708577036	0.478586991	0.99135833	ENSG00000159674	SPON2
491.2460994	0.471495109	0.269029445	1.752578081	0.079674456	0.897052166	ENSG00000197586	ENTPD6
494.8331976	-0.089530425	0.240036436	-0.37298681	0.709158261	0.99900742	ENSG00000137692	DCUN1D5
485.6717093	-0.208795765	0.296051738	-0.705267822	0.480643581	0.991417002	ENSG00000138796	HADH

493.4705017	0.372122278	0.248837496	1.495442949	0.134798879	0.933741583	ENSG00000055609	KMT2C
486.8245529	-0.099713917	0.213426759	-0.46720438	0.64035366	0.996553586	ENSG00000123739	PLA2G12A
487.0578542	-0.304984319	0.246426059	-1.237630145	0.215853232	0.954310328	ENSG00000171222	SCAND1
493.8055378	0.238349076	0.22240008	1.071713088	0.283848921	0.963310634	ENSG00000135837	CEP350
487.4458548	-0.045518734	0.230692223	-0.197313692	0.843582066	0.999467288	ENSG00000051382	PIK3CB
486.2092164	-0.376817219	0.243465965	-1.547720311	0.12168965	0.923922314	ENSG00000110697	PITPNM1
492.0838181	-0.340666693	0.288786159	-1.179650349	0.238139309	0.962465655	ENSG00000247596	TWF2
487.8635777	0.667833849	0.371658452	1.79690209	0.072351165	0.885710197	ENSG00000120802	TMPO
491.7197339	0.365841897	0.221676693	1.650339923	0.098873431	0.921147998	ENSG00000171940	ZNF217
488.2754997	-0.405754574	0.332783711	-1.219274143	0.222740159	0.956884001	ENSG00000188313	PLSCR1
489.4090865	0.161257822	0.255154153	0.632001556	0.527385861	0.99483168	ENSG00000145354	CISD2
491.7273691	0.163949721	0.236828973	0.692270541	0.488767446	0.993513219	ENSG00000038002	AGA
491.7601689	-0.080646617	0.218860513	-0.36848409	0.712512306	0.99900742	ENSG00000102786	INTS6
490.1330713	-0.465014089	0.30533887	-1.522944292	0.127772643	0.928627271	ENSG00000112679	DUSP22
486.6843467	-0.085330612	0.271885543	-0.31384755	0.753636828	0.999467288	ENSG00000090097	PCBP4
490.1675666	-0.448576804	0.282034083	-1.590505655	0.111720872	0.923922314	ENSG00000161791	FMNL3
502.2364352	0.853863634	0.507589166	1.682194362	0.092531157	0.921147998	ENSG00000162998	FRZB
486.3658397	-0.047350855	0.205021733	-0.230955296	0.817349532	0.999467288	ENSG00000133884	DPF2
476.9104554	-0.976463379	0.420275372	-2.323389484	0.020158239	0.73997555	ENSG00000214530	STARD10
486.7520706	0.064161973	0.209178568	0.306733015	0.759046598	0.999467288	ENSG00000144659	SLC25A38
487.2887991	0.178589262	0.276933829	0.64488063	0.519004545	0.993513219	ENSG00000135365	PHF21A
481.7821175	0.743063988	0.264239402	2.812086244	0.00492213	0.472943531	ENSG00000198824	CHAMP1
487.4836848	0.120618323	0.213405589	0.565206954	0.571933007	0.99483168	ENSG00000144580	CNOT9
490.2164716	-0.119686506	0.263456849	-0.454292635	0.649618216	0.997381078	ENSG00000128602	SMO
465.4129386	0.468071826	1.019504623	0.459116923	0.646150204	0.997368416	ENSG00000135245	HILPDA
485.3445388	-0.141814538	0.328499777	-0.431703604	0.665956847	0.997568624	ENSG00000167397	VKORC1
481.0137045	-0.117342307	0.322105735	-0.36429748	0.715635881	0.99900742	ENSG00000185551	NR2F2
484.1601726	-0.186380353	0.236039014	-0.789616725	0.429751638	0.991301167	ENSG00000169718	DUS1L
479.7245379	-1.881577614	0.496250955	-3.791584875	0.000149689	0.093998167	ENSG00000168874	ATOH8
486.4599119	-0.239460179	0.218083972	-1.09801824	0.272196524	0.962465655	ENSG00000010803	SCMH1
497.2237504	-0.099144193	0.394797084	-0.251126963	0.80171595	0.999467288	ENSG00000104881	PPP1R13L
484.7253894	-0.274632666	0.227584239	-1.206729723	0.227536248	0.958865115	ENSG00000148334	PTGES2
490.3522523	0.24731477	0.293234527	0.843402625	0.399003304	0.988378925	ENSG00000162607	USP1
484.6163173	0.140808253	0.221751375	0.634982546	0.525439798	0.99483168	ENSG00000166260	COX11
487.5195438	-0.185128815	0.252438149	-0.733363065	0.46333702	0.99135833	ENSG00000160218	TRAPPC10
484.7977513	0.020649612	0.240076209	0.086012739	0.931456291	0.999527751	ENSG00000163539	CLASP2
485.0177942	0.687854059	0.298514476	2.304256963	0.021208223	0.73997555	ENSG00000108848	LUC7L3
478.7088026	-0.27346852	0.301385299	-0.907371797	0.364210218	0.98048242	ENSG00000157978	LDLRAP1
484.1023876	-0.254642101	0.261207406	-0.974865549	0.329626957	0.976026345	ENSG00000213619	NDUFS3
478.2198071	0.004552069	0.384682419	0.011833317	0.9905586	0.999527751	ENSG00000135596	MICAL1
482.6493515	-0.062931302	0.332358625	-0.189347582	0.849820404	0.999467288	ENSG00000137547	MRPL15
486.6079748	0.377875877	0.213491502	1.769980879	0.076730326	0.897052166	ENSG00000132549	VPS13B
477.9569233	0.262672336	0.356968951	0.735840849	0.461827559	0.99135833	ENSG00000119326	CTNNAL1
489.5928393	0.110568351	0.298781279	0.370064523	0.711334415	0.99900742	ENSG00000090857	PDPR
477.9304243	0.089942651	0.32651415	0.275463256	0.782960352	0.999467288	ENSG00000168701	TMEM208
483.2004909	-0.227349164	0.278134771	-0.817406479	0.413696174	0.990226751	ENSG00000111906	HDDC2
485.6185614	-0.212600659	0.270343587	-0.786409106	0.43162785	0.991301167	ENSG00000109519	GRPEL1
483.9950024	0.017603707	0.21469405	0.081994389	0.934651175	0.999527751	ENSG00000158545	ZC3H18
491.7565254	0.052414082	0.3636382	0.144137997	0.885391501	0.999467288	ENSG00000169855	ROBO1
484.1295706	0.150294446	0.240389473	0.625212262	0.531831755	0.99483168	ENSG00000115446	UNC50
479.0194823	-0.129917801	0.252028302	-0.515488934	0.6062114	0.99483168	ENSG00000163866	SMIM12
484.9286012	-0.121509859	0.232907118	-0.521709514	0.601872599	0.99483168	ENSG00000163001	CFAP36
480.9050337	-0.04294311	0.218496045	-0.196539531	0.844187895	0.999467288	ENSG00000196655	TRAPPC4
480.2312073	0.176965716	0.341804765	0.51773917	0.604640265	0.99483168	ENSG00000134901	KDELC1
480.8066727	-0.19238941	0.262474458	-0.732983359	0.463568579	0.99135833	ENSG00000182247	UBE2E2
481.1553674	-0.515762622	0.391262509	-1.318200977	0.187436378	0.949094286	ENSG00000143344	RGL1
485.6913069	0.586752528	0.258424714	2.270496964	0.023177447	0.744948012	ENSG00000003756	RBM5
483.2441716	0.002458178	0.252675775	0.009728584	0.992237835	0.999527751	ENSG00000124177	CHD6
481.258287	-0.566610589	0.243171638	-2.330085012	0.019801658	0.73975641	ENSG00000134775	FHOD3
484.0606336	0.18695064	0.276749324	0.675523384	0.4993433	0.993513219	ENSG00000241878	PISD
480.9682964	-0.095965536	0.20583852	-0.466217578	0.641059769	0.996553586	ENSG00000084072	PPIE
480.5919482	0.055351489	0.230373475	0.240268501	0.810122113	0.999467288	ENSG00000128789	PSMG2
483.2573706	-0.044590822	0.22041968	-0.202299641	0.839682481	0.999467288	ENSG00000198860	TSEN15
479.3875968	0.209283504	0.258919998	0.808294089	0.418921305	0.990226751	ENSG00000185475	TMEM179B

473.5503599	-0.12982204	0.282644066	-0.45931281	0.64600955	0.997368416	ENSG00000121440	PDZRN3
485.4767732	0.277496414	0.289780535	0.957608879	0.33826002	0.977691382	ENSG00000154447	SH3RF1
481.354566	-0.176659812	0.218326632	-0.809153748	0.418426716	0.990226751	ENSG00000179119	SPTY2D1
479.2921092	-0.270284691	0.34119794	-0.792163902	0.428265114	0.991301167	ENSG00000235106	BRD3OS
471.9970101	0.493530739	0.406621886	1.213733829	0.224849382	0.958865115	ENSG00000166165	CKB
480.3084502	0.334689483	0.265333195	1.261393178	0.207167225	0.954310328	ENSG00000125384	PTGER2
481.762207	-0.01075591	0.228617267	-0.047047672	0.962475233	0.999527751	ENSG00000116731	PRDM2
480.4167408	-0.228182259	0.247716583	-0.921142446	0.356976062	0.979427314	ENSG00000160953	MUM1
474.2644397	-0.122869508	0.318818846	-0.385389728	0.699948692	0.998869317	ENSG00000184752	NDUFA12
474.8003719	-0.405122418	0.265236121	-1.527402889	0.126660859	0.928627271	ENSG00000112511	PHF1
472.0736558	-0.740377666	0.333724092	-2.218532267	0.026518562	0.764376062	ENSG00000128185	DGCR6L
480.6840896	-0.289971683	0.298525634	-0.971346006	0.331376006	0.976026345	ENSG00000100906	NFKBIA
473.8767593	-0.131862431	0.274560621	-0.480267093	0.631037485	0.99483168	ENSG00000010165	METTL13
474.6993236	0.525025056	0.341096141	1.539228952	0.123748412	0.927794614	ENSG00000154736	ADAMTS5
478.5828832	-0.016229042	0.223231484	-0.072700505	0.942044446	0.999527751	ENSG00000076242	MLH1
479.9589976	0.244649742	0.263771245	0.92750725	0.353663229	0.977691382	ENSG00000165699	TSC1
480.5262289	0.531521919	0.309831008	1.715522025	0.086249561	0.901307093	ENSG00000159322	ADPGK
482.4680533	-0.159020036	0.249875679	-0.636396611	0.524517949	0.99483168	ENSG00000073417	PDE8A
480.1895769	0.265014356	0.287364875	0.922222508	0.356412525	0.979427314	ENSG00000106692	FKTN
474.1234082	-0.317573011	0.310526461	-1.022692269	0.306453368	0.973427483	ENSG00000130731	METTL26
482.603369	-0.322847841	0.362058836	-0.891699937	0.372553787	0.982259938	ENSG00000153815	CMIP
479.2465842	0.105939317	0.24843884	0.42642011	0.66980175	0.997568624	ENSG00000184220	CMSS1
481.0896323	-0.113243942	0.25747669	-0.439822113	0.66006595	0.997568624	ENSG00000125633	CCDC93
477.9767088	-0.143656688	0.241893549	-0.593883915	0.552589758	0.99483168	ENSG00000106245	BUD31
477.6501608	-0.313841211	0.223727914	-1.402780755	0.160682227	0.941856092	ENSG00000102218	RP2
471.8380921	-0.616648307	0.345942235	-1.782518134	0.074664777	0.893390506	ENSG00000250479	CHCHD10
472.5112015	-0.344594392	0.307768738	-1.119653654	0.262861382	0.962465655	ENSG00000151552	QDPR
478.4587483	-0.158187158	0.23547671	-0.671774112	0.501727512	0.993513219	ENSG00000066926	FECH
476.170184	-0.124869765	0.237145169	-0.526554119	0.598503261	0.99483168	ENSG00000132286	TIMM10B
474.0474287	0.316244701	0.318915833	0.991624333	0.321380815	0.975337095	ENSG00000101974	ATP11C
475.3272989	0.158352201	0.294556061	0.537596139	0.590855896	0.99483168	ENSG00000064102	INTS13
477.0391514	-0.250660982	0.223398115	-1.122037139	0.261846649	0.962465655	ENSG00000148331	ASB6
475.1881109	-0.221636179	0.227002355	-0.976360705	0.328885747	0.976026345	ENSG00000186716	BCR
481.2842032	0.432320738	0.293746949	1.471745457	0.141089627	0.935644597	ENSG00000188994	ZNF292
481.0253271	0.188205656	0.298497433	0.630510131	0.528360877	0.99483168	ENSG00000101109	STK4
472.559708	-0.258451543	0.237830305	-1.08670568	0.277166903	0.962465655	ENSG00000160049	DFFA
479.3558352	0.009223255	0.230712919	0.039977196	0.968111307	0.999527751	ENSG00000138081	FBXO11
469.9997048	-0.210759824	0.263287139	-0.800494185	0.423424531	0.991301167	ENSG00000198892	SHISA4
475.4721369	-0.04094406	0.216406964	-0.189199364	0.849936566	0.999467288	ENSG00000118454	ANKRD13C
473.9674205	0.724288466	0.32009129	2.262755934	0.023650739	0.744948012	ENSG00000125430	HS3ST3B1
475.3263762	-0.127670771	0.285955377	-0.446470958	0.655257087	0.997568624	ENSG00000151748	SAV1
476.7094982	0.134121341	0.21183041	0.633154327	0.526632868	0.99483168	ENSG00000145868	FBXO38
476.808884	0.105047447	0.294148817	0.357123472	0.720999374	0.99900742	ENSG00000011295	TTC19
473.9130122	0.264423484	0.249341065	1.060489111	0.288922141	0.966288987	ENSG00000119900	OGFRL1
474.0868516	-0.004737105	0.243885258	-0.019423498	0.984503265	0.999527751	ENSG00000133028	SCO1
477.5896714	-0.069198274	0.235851638	-0.29339747	0.769218359	0.999467288	ENSG00000122482	ZNF644
466.9244993	0.17332065	0.340684542	0.508742337	0.610932842	0.99483168	ENSG00000122691	TWIST1
475.7378231	0.067496692	0.217280946	0.310642481	0.756072429	0.999467288	ENSG00000133422	MORC2
473.5924151	0.200161822	0.218714644	0.915173389	0.360100613	0.979465649	ENSG00000176018	LYSMD3
474.7965818	0.114422087	0.224697156	0.509228017	0.610592407	0.99483168	ENSG00000132382	MYBBP1A
473.8116069	-0.018182374	0.296528943	-0.061317368	0.951106459	0.999527751	ENSG00000144815	NXPE3
473.0632871	-0.327485273	0.223864682	-1.462871542	0.143502532	0.935644597	ENSG00000189343	RPS2P46
473.4695514	1.845063232	0.538089087	3.428917768	0.000605993	0.195790358	ENSG00000163814	CDCP1
480.0249652	-0.882723854	0.478771203	-1.843727962	0.065222796	0.868202292	ENSG00000188483	IER5L
471.353582	-0.187059867	0.2401773	-0.778840743	0.436073534	0.991301167	ENSG00000173960	UBXN2A
478.4451427	-0.05389735	0.298871393	-0.180336262	0.85688859	0.999467288	ENSG00000037749	MFAP3
468.0664661	-0.005803819	0.261733495	-0.022174535	0.982308731	0.999527751	ENSG00000101246	ARFRP1
462.8011113	1.063629869	0.391569254	2.716326315	0.006601082	0.526737172	ENSG00000148154	UGCG
464.7284466	-0.111763498	0.315996327	-0.353686067	0.723574165	0.999456245	ENSG00000166974	MAPRE2
473.3479525	-0.117653461	0.2240228	-0.525185208	0.599454446	0.99483168	ENSG00000067208	EVI5
470.8789294	0.069130948	0.235792511	0.293185512	0.769380358	0.999467288	ENSG00000167081	PBX3
467.7035183	0.042888175	0.26451284	0.16214024	0.871195419	0.999467288	ENSG00000140365	COMMD4
475.7032767	-0.255342416	0.286972046	-0.889781493	0.373583226	0.982521685	ENSG00000143751	SDE2
476.5128245	-0.032193971	0.278378768	-0.115648083	0.907931453	0.999527751	ENSG00000151806	GUF1

469.1477032	-0.030472415	0.28128133	-0.108334296	0.913730518	0.999527751	ENSG00000165264	NDUFB6	
475.6630069	0.105474027	0.244330052	0.431686674	0.665969154	0.997568624	ENSG00000226479	TMEM185B	
475.4064643	0.00848252	0.245261988	0.034585547	0.972410227	0.999527751	ENSG00000180008	SOCS4	
472.5875431	0.140937965	0.245464897	0.574167497	0.565854462	0.99483168	ENSG00000134014	ELP3	
470.548181	-0.008118746	0.231577902	-0.035058378	0.97203319	0.999527751	ENSG00000105443	CYTH2	
469.7473459	0.186448003	0.283083195	0.658633242	0.510131311	0.993513219	ENSG00000197461	PDGFA	
471.6922603	-0.313298134	0.213118916	-1.470062535	0.141544818	0.935644597	ENSG00000136643	RPS6KC1	
465.5859032	0.129736841	0.240614549	0.53918951	0.589756098	0.99483168	ENSG00000142188	TMEM50B	
472.2327489	-0.334691148	0.314522006	-1.064126331	0.287271474	0.965284229	ENSG00000237441	RGL2	
476.8322821	-0.182102528	0.337701404	-0.539241253	0.589720399	0.99483168	ENSG00000111912	NCOA7	
466.3791043	-0.172852052	0.259199574	-0.66686858	0.504856082	0.993513219	ENSG00000141698	NT5C3B	
466.0678404	-0.055042285	0.292228455	-0.188353613	0.85059946	0.999467288	ENSG00000162384	C1orf123	
468.7544277	-0.000797025	0.242921635	-0.003280995	0.997382149	0.999527751	ENSG00000117614	SYF2	
471.8521738	0.238689806	0.218279209	1.093506833	0.274171331	0.962465655	ENSG00000121940	CLCC1	
461.1998304	-0.310099094	0.319766448	-0.969767455	0.332162419	0.976026345	ENSG00000141504	SAT2	
468.1800712	-0.266575731	0.251368699	-1.060496918	0.288918591	0.966288987	ENSG00000177096	PHETA2	
470.8641039	0.215244019	0.220589998	0.975765088	0.329180889	0.976026345	ENSG00000163811	WDR43	
467.7040789	0.304291666	0.229696715	1.324754105	0.185252721	0.949094286	ENSG00000163755	HPS3	
473.340832	-0.00214927	0.26086361	-0.008239058	0.993426257	0.999527751	ENSG00000110344	UBE4A	
468.9815542	-0.211715386	0.310669782	-0.681480461	0.495567526	0.993513219	ENSG00000070423	RNF126	
467.592128	0.06480555	0.250010447	0.259211368	0.795472161	0.999467288	ENSG00000130479	MAP1S	
464.5397903	-0.04405922	0.34821171	-0.126529977	0.899312421	0.999527751	ENSG00000155115	GTF3C6	
465.2445493	0.104466333	0.408147942	0.255952124	0.797987805	0.999467288	ENSG00000197321	SVIL	
465.8447124	-0.047871306	0.214914584	-0.222745729	0.823733409	0.999467288	ENSG00000103642	LACTB	
472.0318797	0.142636363	0.253289542	0.563135617	0.57334254	0.99483168	ENSG00000163125	RPRD2	
469.2114112	-0.138671876	0.246027376	-0.563644088	0.572996376	0.99483168	ENSG00000162980	ARL5A	
472.4005293	-0.121879995	0.264884552	-0.460124966	0.645426525	0.997368416	ENSG00000146833	TRIM4	
466.1958524	-0.079463395	0.206279949	-0.385221129	0.70007359	0.998869317	ENSG00000225190	PLEKHM1	
463.9812687	0.143945943	0.240174959	0.599337845	0.548947616	0.99483168	ENSG00000119471	HSDL2	
468.7459756	-0.402772393	0.320551561	-1.256497993	0.208935473	0.954310328	ENSG00000101849	TBL1X	
469.4510644	0.15077143	0.269483391	0.559483198	0.575831995	0.99483168	ENSG00000162688	AGL	
467.5393463	0.187767727	0.259702113	0.723011934	0.469672553	0.99135833	ENSG00000119203	CPSF3	
463.4893153	0.014592313	0.276379035	0.052798191	0.957892703	0.999527751	ENSG00000150787	PTS	
463.1672746	-0.233795962	0.297493227	-0.785886672	0.431933883	0.991301167	ENSG00000106617	PRKAG2	
464.3481704	-0.203832202	0.240780789	-0.846546782	0.397247788	0.988378925	ENSG00000126107	HECTD3	
457.9674248	0.68930685	0.376307483	1.83176493	0.06698645	0.87420149	ENSG00000166949	SMAD3	
469.6081084	0.334935071	0.215409586	1.554875423	0.119975742	0.923922314	ENSG00000185658	BRWD1	
466.5318734	-0.224121416	0.264524274	-0.847262193	0.396848995	0.988378925	ENSG00000180357	ZNF609	
463.7446539	-0.102775781	0.249367011	-0.412146659	0.680231931	0.998869317	ENSG00000124588	NQO2	
461.8975536	-0.414815749	0.319859384	-1.296869093	0.194676232	0.952475889	ENSG00000161714	PLCD3	
463.5131482	-0.180538831	0.235448813	-0.766785902	0.44320882	0.99135833	ENSG00000172936	MYD88	
464.7551816	0.037135459	0.275261741	0.134909627	0.892683328	0.999467288	ENSG00000174132	FAM174A	
451.1735044	0.785418305	0.542101226	1.448840673	0.147382082	0.936396974	ENSG00000125266	EFNB2	
461.7805714	0.159029133	0.243721848	0.652502574	0.514077013	0.993513219	ENSG00000040341	STAU2	
462.6090644	0.396444043	0.243520712	1.627968479	0.10353157	0.92169898	ENSG00000112343	TRIM38	
462.6661509	-0.301808633	0.24257506	-1.244186574	0.213430904	0.954310328	ENSG00000187051	RPS19BP1	
464.7682666	0.231951899	0.256880369	0.902956891	0.366548775	0.981269214	ENSG00000003393	ALS2	
464.0293748	-0.265345306	0.211095175	-1.256993704	0.208755915	0.954310328	ENSG00000163807	KIAA1143	
462.9510816	0.086378674	0.312045908	0.276813994	0.781922936	0.999467288	ENSG00000123643	SLC36A1	
462.0622091	0.052263578	0.228287279	0.228937757	0.818917294	0.999467288	ENSG00000140395	WDR61	
466.3812409	0.308884106	0.2399084	1.28750851	0.197917105	0.954310328	ENSG00000075539	FRYL	
459.2402591	-0.375513155	0.317068816	-1.184326986	0.23628365	0.962465655	ENSG00000156587	UBE2L6	
458.1029225	-0.563424342	0.338700386	-1.66348893	0.096214605	0.921147998	ENSG00000099785		2-Mar
461.6809945	0.080504593	0.246676583	0.326356851	0.74415438	0.999467288	ENSG00000115364	MRPL19	
460.9215883	0.0084361	0.269502855	0.031302453	0.975028334	0.999527751	ENSG00000196656	AC004057.1	
471.9310968	-0.027296479	0.308156845	-0.088579824	0.929415843	0.999527751	ENSG00000180543	TSPYL5	
464.2501311	-0.202856451	0.234299247	-0.865800694	0.386599469	0.986711841	ENSG00000181027	FKRP	
463.8287738	-0.287989557	0.271409943	-1.061086982	0.288650374	0.966288987	ENSG00000130717	UCK1	
461.5208426	-0.45232058	0.284563559	-1.589523907	0.111942161	0.923922314	ENSG00000182768	NGRN	
462.9836904	-0.178406256	0.234736412	-0.760028045	0.447237821	0.99135833	ENSG00000112640	PPP2R5D	
458.9185677	0.013486872	0.27954769	0.048245337	0.961520719	0.999527751	ENSG00000149782	PLCB3	
462.6032192	0.161472545	0.227059726	0.711145688	0.47699396	0.99135833	ENSG00000197150	ABCB8	
459.7303962	-0.042717245	0.249411792	-0.171271953	0.864009933	0.999467288	ENSG00000117153	KLHL12	
462.7986687	-0.199119811	0.537324909	-0.370576178	0.710953228	0.99900742	ENSG00000141401	IMPA2	

463.7678835	-0.300577602	0.251255961	-1.196300383	0.231579359	0.959575179	ENSG00000100744	GSKIP
468.860063	0.292094419	0.268479424	1.087958306	0.276613514	0.962465655	ENSG00000186908	ZDHHC17
462.3907392	-0.144460675	0.221883776	-0.651064613	0.515004779	0.993513219	ENSG00000139197	PEX5
473.2792936	-0.010831939	0.357899282	-0.030265328	0.975855448	0.999527751	ENSG00000081014	AP4E1
456.1925131	-0.711838922	0.325605346	-2.186201581	0.028800861	0.773201806	ENSG00000142910	TINAGL1
464.782292	-0.342986423	0.230515075	-1.487913195	0.136773787	0.934144427	ENSG00000144647	POMGNT2
461.7474894	-0.200587546	0.259235005	-0.773767208	0.439068468	0.991301167	ENSG00000071994	PDCD2
463.7480464	0.245171529	0.286556409	0.855578592	0.392230915	0.988378925	ENSG00000137075	RNF38
464.260692	0.043176214	0.240251421	0.179712626	0.857378181	0.999467288	ENSG00000136709	WDR33
462.3196934	0.392834764	0.271159017	1.448724694	0.147414481	0.936396974	ENSG00000134900	TPP2
460.4986521	-0.273502517	0.219492759	-1.246066241	0.212740077	0.954310328	ENSG00000100216	TOMM22
458.8008116	0.047706061	0.348506681	0.136887077	0.891120054	0.999467288	ENSG00000131462	TUBG1
464.6696487	0.531911818	0.245200775	2.16929093	0.030060603	0.773201806	ENSG00000173218	VANGL1
461.1612806	-0.061462199	0.261221104	-0.235288029	0.813985186	0.999467288	ENSG00000149499	EML3
464.1556647	-0.154403745	0.214464997	-0.719948464	0.471556727	0.99135833	ENSG00000132823	OSER1
457.2114633	-0.326617027	0.24607812	-1.32729	0.184412771	0.949094286	ENSG00000173486	FKBP2
461.8361619	-0.244327948	0.222560391	-1.097805171	0.272289573	0.962465655	ENSG00000111707	SUDS3
464.304513	-0.008919002	0.254927186	-0.03498647	0.972090529	0.999527751	ENSG00000136935	GOLGA1
464.9678482	-0.130394857	0.232783983	-0.560153903	0.575374466	0.99483168	ENSG00000123728	RAP2C
457.6068037	-0.022126879	0.237319754	-0.093236568	0.925715624	0.999527751	ENSG00000105928	GSDME
464.3783023	0.224762666	0.272314562	0.825378799	0.409156567	0.990226751	ENSG00000146733	PSPH
463.0031719	-0.222314807	0.275007329	-0.808395935	0.418862691	0.990226751	ENSG00000173264	GPR137
460.4842811	-0.085124056	0.235564947	-0.361361304	0.717829371	0.99900742	ENSG00000106608	URGCP
460.0397672	-0.151439214	0.227090746	-0.666866514	0.504857402	0.993513219	ENSG00000156381	ANKRD9
462.7674844	-0.010986472	0.231123882	-0.047534991	0.962086843	0.999527751	ENSG00000132361	CLUH
462.4523178	0.238417541	0.216645978	1.100493731	0.27111706	0.962465655	ENSG00000000419	DPM1
465.3316728	-0.130990569	0.231245935	-0.566455662	0.571084065	0.99483168	ENSG00000148737	TCF7L2
466.0719637	-1.070954964	0.475191182	-2.25373493	0.024212849	0.747235409	ENSG00000204387	C6orf48
461.0864255	0.19987751	0.242658884	0.823697475	0.410111474	0.990226751	ENSG00000021776	AQR
456.061145	-0.104812241	0.317221078	-0.330407557	0.741092032	0.999467288	ENSG00000182628	SKA2
463.3880718	-0.138972611	0.238749021	-0.582086624	0.560508335	0.99483168	ENSG00000048991	R3HDM1
461.8007475	0.53650024	0.232021784	2.312283921	0.020762042	0.73997555	ENSG00000197343	ZNF655
450.3140648	0.514303621	0.447728047	1.148696457	0.250681167	0.962465655	ENSG00000067798	NAV3
462.4773206	-0.484276255	0.255218741	-1.897494883	0.057762653	0.852306368	ENSG00000212802	RPL15P3
455.9185364	0.587794203	0.296580374	1.981905259	0.047489845	0.827997046	ENSG00000068028	RASSF1
463.3045015	0.027116087	0.283574148	0.095622564	0.923820344	0.999527751	ENSG00000213079	SCAF8
461.0595846	-0.162486093	0.226677546	-0.716816004	0.473487635	0.99135833	ENSG00000204560	DHX16
463.4325035	-0.115742258	0.260461904	-0.444373079	0.656772874	0.997568624	ENSG00000078246	TULP3
459.3076627	0.110297044	0.221877634	0.497107538	0.619113219	0.99483168	ENSG00000141456	PELP1
458.1026898	0.26174228	0.210847353	1.241382813	0.214464365	0.954310328	ENSG00000129493	HEATR5A
461.9381259	-0.280846197	0.28034392	-1.001791646	0.316444233	0.974768742	ENSG00000169925	BRD3
459.2046833	0.242706466	0.260283074	0.932471183	0.351093072	0.977691382	ENSG00000170502	NUDT9
459.1668466	-0.187668448	0.207575619	-0.90409678	0.365944086	0.981269214	ENSG00000055147	FAM114A2
464.2762236	0.069220861	0.278698743	0.248371629	0.803846883	0.999467288	ENSG00000164307	ERAP1
459.6404734	-0.159366729	0.305779176	-0.521182415	0.602239703	0.99483168	ENSG00000102100	SLC35A2
455.4717197	-0.144611204	0.231280505	-0.625263268	0.531798283	0.99483168	ENSG00000120727	PAIP2
455.1806325	-0.100133222	0.239536508	-0.418029062	0.675925869	0.998869317	ENSG00000166313	APBB1
458.4110986	0.051584575	0.420481438	0.122679792	0.902360666	0.999527751	ENSG00000151882	CCL28
464.0601084	-0.436604592	0.320252935	-1.363311759	0.172784278	0.944422693	ENSG00000063587	ZNF275
457.2344242	-0.082410991	0.26980832	-0.305442733	0.760028979	0.999467288	ENSG00000102900	NUP93
455.2710283	-0.160944887	0.214547929	-0.750158194	0.453159434	0.99135833	ENSG00000213341	CHUK
454.3356498	0.097523816	0.261383734	0.373105911	0.70906962	0.99900742	ENSG00000112419	PHACTR2
460.0559887	0.576243248	0.259758254	2.218382817	0.026528742	0.764376062	ENSG00000166012	TAF1D
462.3446578	-0.325040236	0.320868983	-1.012999865	0.311060225	0.973427483	ENSG00000109189	USP46
456.6238467	-0.293661596	0.210674963	-1.393908382	0.163345273	0.943223078	ENSG00000083099	LYRM2
456.7668969	0.160383271	0.221883143	0.722827652	0.469785776	0.99135833	ENSG00000005020	SKAP2
454.7842274	-0.215023893	0.238100225	-0.903081436	0.366482676	0.981269214	ENSG00000130560	UBAC1
456.7937244	-0.200842671	0.239758967	-0.837685753	0.402207217	0.988592117	ENSG00000092847	AGO1
457.0967433	0.108229814	0.269742075	0.401234455	0.688247516	0.998869317	ENSG00000112685	EXOC2
457.326231	0.198514734	0.251675116	0.788773785	0.430244234	0.991301167	ENSG00000157259	GATAD1
462.3844966	0.017774415	0.298572199	0.059531379	0.952528873	0.999527751	ENSG00000107643	MAPK8
458.1048616	-0.061233371	0.212688264	-0.287901975	0.773421775	0.999467288	ENSG00000165832	TRUB1
455.9827299	-0.182773717	0.216890856	-0.842699043	0.399396783	0.988378925	ENSG00000143319	ISG20L2
450.0758697	-0.593389326	0.332743372	-1.783324256	0.074533535	0.893390506	ENSG00000198736	MSRB1

456.2252829	-0.269085344	0.241171662	-1.115741962	0.264532603	0.962465655	ENSG00000149657	LSM14B
455.6792882	0.446509504	0.25900206	1.723961205	0.084714847	0.901307093	ENSG00000188486	H2AFX
456.6702984	0.009187577	0.241065444	0.038112378	0.969598082	0.999527751	ENSG00000156531	PHF6
456.607137	0.080388579	0.317579439	0.253129042	0.800168497	0.999467288	ENSG00000184277	TM2D3
459.680157	0.132135168	0.250506382	0.527472262	0.597865674	0.99483168	ENSG00000196865	NHLRC2
458.3776366	0.04376659	0.240073661	0.182304839	0.855343501	0.999467288	ENSG00000104643	MTMR9
451.5674263	0.049923169	0.277286934	0.180041547	0.857119952	0.999467288	ENSG00000127527	EPS15L1
459.3724494	-0.084982051	0.295594249	-0.287495616	0.773732859	0.999467288	ENSG00000083168	KAT6A
456.7163814	-0.386915983	0.230590323	-1.677936776	0.093359442	0.921147998	ENSG00000130204	TOMM40
453.0102809	-0.151469504	0.235901231	-0.642088656	0.520815621	0.993513219	ENSG00000146066	HIGD2A
452.9365219	-0.401070982	0.233323303	-1.718949527	0.085623563	0.901307093	ENSG00000108788	MLX
448.5197876	-0.108671872	0.361608054	-0.300523925	0.76377755	0.999467288	ENSG00000172819	RARG
451.5623239	-0.501888309	0.221184798	-2.269090439	0.023262827	0.744948012	ENSG00000204308	RNF5
457.9771598	0.378584155	0.254518703	1.487451217	0.136895678	0.934144427	ENSG00000129315	CCNT1
462.4272252	-0.070882041	0.28670047	-0.24723378	0.804727304	0.999467288	ENSG00000065970	FOXJ2
454.588138	-0.171701757	0.217428671	-0.789692346	0.429707463	0.991301167	ENSG00000164068	RNF123
454.976165	0.735561458	0.272934078	2.695015089	0.007038544	0.549501061	ENSG00000166508	MCM7
457.7376361	0.369970451	0.290831696	1.272111864	0.203333376	0.954310328	ENSG00000149311	ATM
451.44699	-0.248294184	0.391198618	-0.634701076	0.525623392	0.99483168	ENSG00000153714	LURAP1L
452.8163465	-0.01604659	0.238928683	-0.067160584	0.946453864	0.999527751	ENSG00000123064	DDX54
454.9031334	0.115632809	0.207782254	0.556509552	0.577862566	0.99483168	ENSG00000143093	STRIP1
454.9047059	0.258444983	0.223042548	1.158725032	0.246568284	0.962465655	ENSG00000110429	FBXO3
451.3071292	-0.054300559	0.23581411	-0.23026849	0.817883144	0.999467288	ENSG00000100417	PMM1
453.4593214	-0.186364252	0.24612912	-0.757180833	0.448941532	0.99135833	ENSG00000196663	TECPR2
460.6327576	-0.081853374	0.336706266	-0.24310024	0.807927746	0.999467288	ENSG00000155111	CDK19
452.6571065	-0.095314069	0.20160175	-0.472783935	0.636367336	0.99653524	ENSG00000129473	BCL2L2
453.1116361	-0.050411745	0.221544369	-0.227546949	0.819998466	0.999467288	ENSG00000213463	SYNJ2BP
452.7249737	-0.015661975	0.220602309	-0.070996424	0.943400602	0.999527751	ENSG00000165689	ENTR1
460.0957834	-0.357201397	0.396577575	-0.900710022	0.367742518	0.981269214	ENSG00000154144	TBRG1
454.560757	-0.167507816	0.231044039	-0.72500384	0.468449674	0.99135833	ENSG00000151116	UEVLD
454.8607247	0.011201952	0.280843466	0.039886818	0.968183361	0.999527751	ENSG00000108651	UTP6
451.5354586	-0.161874261	0.314331817	-0.514978923	0.606567748	0.99483168	ENSG00000172831	CES2
455.2205704	0.026764311	0.23489984	0.113939246	0.909285955	0.999527751	ENSG00000133393	FOPNL
448.2375426	-0.261043033	0.271356795	-0.961991877	0.336053684	0.977691382	ENSG00000104613	INTS10
451.2004491	0.005857472	0.262421357	0.022320864	0.982192006	0.999527751	ENSG00000184384	MAML2
455.4653156	0.058197221	0.243270004	0.239228921	0.810928078	0.999467288	ENSG00000115020	PIKFYVE
445.3886876	0.43515794	0.364145997	1.195009538	0.232083304	0.959575179	ENSG00000160752	FDPS
449.9084297	-0.039642522	0.242808235	-0.163266794	0.870308378	0.999467288	ENSG00000181026	AEN
448.5268547	-0.134757576	0.272982236	-0.493649618	0.621553652	0.99483168	ENSG00000147471	PLPBP
448.1959453	-0.35870281	0.28811851	-1.244983567	0.213137791	0.954310328	ENSG00000213465	ARL2
450.5506878	0.24711877	0.240655881	1.026855314	0.304488598	0.973427483	ENSG00000105186	ANKRD27
450.8623863	-0.205726641	0.217642638	-0.945249714	0.344531404	0.977691382	ENSG00000057757	PITHD1
444.4101973	-0.230881217	0.323229394	-0.714295237	0.475044637	0.99135833	ENSG00000172428	COPS9
453.4600482	-0.900047633	0.438342776	-2.053296372	0.040043843	0.794441233	ENSG00000155792	DEPTOR
450.7541596	-0.191203751	0.245923	-0.777494379	0.436867149	0.991301167	ENSG00000094880	CDC23
454.2019469	0.069901789	0.242569386	0.288172345	0.773214816	0.999467288	ENSG00000066739	ATG2B
452.9273801	-0.028952198	0.272214574	-0.106358003	0.915298313	0.999527751	ENSG00000275066	SYNRG
447.9759433	0.29996437	0.293909159	1.020602322	0.307442889	0.973427483	ENSG00000130304	SLC27A1
449.1313336	-0.160428734	0.23458252	-0.683890402	0.494044375	0.993513219	ENSG00000170860	LSM3
452.1195813	-0.082921635	0.28802824	-0.287894114	0.773427793	0.999467288	ENSG00000180530	NRIP1
448.2066739	0.23279658	0.217751525	1.069092762	0.285027873	0.964314258	ENSG00000156136	DCK
446.9289448	-0.139646146	0.237193187	-0.588744337	0.556032791	0.99483168	ENSG00000170855	TRIAP1
445.9081777	-0.059091392	0.307514219	-0.192158243	0.847618254	0.999467288	ENSG00000132669	RIN2
443.2696648	-0.08548614	0.27293387	-0.313211916	0.754119666	0.999467288	ENSG00000171735	CAMTA1
452.7004782	-0.01320116	0.286157538	-0.046132493	0.963204648	0.999527751	ENSG00000070047	PHRF1
445.7600158	-0.607198847	0.365027929	-1.663431204	0.096226151	0.921147998	ENSG00000168303	MPLKIP
451.5574955	0.03710796	0.245726284	0.151013394	0.87996515	0.999467288	ENSG00000196199	MPHOSPH8
448.9424023	-0.142737999	0.265578763	-0.537460138	0.590949813	0.99483168	ENSG00000108262	GIT1
454.6839907	-0.229162462	0.287621477	-0.796750175	0.425596137	0.991301167	ENSG00000151612	ZNF827
450.5258507	-0.030174655	0.281665502	-0.107129396	0.914686327	0.999527751	ENSG00000137145	DENND4C
448.8371402	0.11995936	0.291729364	0.411200843	0.68092527	0.998869317	ENSG00000145833	DDX46
450.6722105	0.137926248	0.253437094	0.54422281	0.586288157	0.99483168	ENSG00000122557	HERPUD2
450.7319932	0.206017406	0.272500394	0.756026085	0.449633556	0.99135833	ENSG00000118246	FASTKD2
452.3011058	0.054255449	0.26494939	0.204776653	0.837746629	0.999467288	ENSG00000131381	RBSN

448.4083725	-0.046597995	0.258598785	-0.180194176	0.857000131	0.999467288	ENSG00000088298	EDEM2
442.6883547	-0.329716316	0.28316247	-1.164406834	0.244259179	0.962465655	ENSG00000130770	ATP5IF1
451.634878	-0.044671719	0.248934018	-0.179452047	0.857582767	0.999467288	ENSG00000108443	RPS6KB1
455.5079321	-0.08481002	0.344348942	-0.246290927	0.805457033	0.999467288	ENSG00000152409	JMY
447.1017105	0.495311683	0.23048084	2.149036266	0.031631525	0.775963115	ENSG00000167548	KMT2D
448.6452093	0.003808724	0.220565546	0.017267991	0.986222821	0.999527751	ENSG00000278845	MRPL45
443.0532299	-0.788721535	0.287392951	-2.744401115	0.006062142	0.499617804	ENSG00000162520	SYNC
442.4350519	-0.293017542	0.364609408	-0.803647781	0.42160042	0.991301167	ENSG00000117228	GBP1
442.0167039	-0.06549497	0.286686018	-0.228455403	0.819292222	0.999467288	ENSG00000151276	MAGI1
439.8319329	-0.02959929	0.395386043	-0.074861748	0.940324712	0.999527751	ENSG00000082438	COBLL1
441.9121214	2.329482937	1.165023275	1.999516221	0.045552529	0.813244956	ENSG00000243137	PSG4
444.9773349	0.105102795	0.228574184	0.459819187	0.64564601	0.997368416	ENSG00000172009	THOP1
448.310127	0.320126631	0.245656317	1.303148379	0.19252411	0.949261383	ENSG00000102710	SUPT20H
445.2083872	0.072880671	0.231121454	0.315334945	0.752507355	0.999467288	ENSG00000103365	GGA2
447.4447491	0.042524206	0.244397212	0.173996281	0.861868385	0.999467288	ENSG00000119231	SENP5
450.3695834	0.30701244	0.243492157	1.26087199	0.207354971	0.954310328	ENSG00000126883	NUP214
449.4086165	0.235258718	0.285016986	0.825419992	0.409133189	0.990226751	ENSG00000173064	HECTD4
438.0614912	0.168789437	0.355448953	0.474862664	0.634884865	0.995506752	ENSG00000106571	GLI3
446.8978822	-0.191178036	0.228511466	-0.836623388	0.402804295	0.988900634	ENSG00000126756	UXT
446.0143295	-0.594777198	0.256642647	-2.317530637	0.020474844	0.73997555	ENSG00000101986	ABCD1
445.1453233	0.393684391	0.263030353	1.49672609	0.134464545	0.933741583	ENSG00000030066	NUP160
442.1604727	-0.483944526	0.350418318	-1.381048023	0.167264196	0.943223078	ENSG00000182253	SYNM
445.9660995	-0.25712241	0.227995756	-1.12775086	0.259425152	0.962465655	ENSG00000088682	COQ9
445.5737474	-0.104489625	0.368093399	-0.283867153	0.776512189	0.999467288	ENSG00000187193	MT1X
446.2301482	0.350606808	0.215597288	1.62621159	0.103904651	0.92169898	ENSG00000133398	MED10
441.203442	-0.163219293	0.253035868	-0.645044096	0.51889861	0.993513219	ENSG00000148290	SURF1
465.2967608	1.650270708	0.892706782	1.848614507	0.064513496	0.868202292	ENSG00000145423	SFRP2
444.4727443	0.249906479	0.241372993	1.035353941	0.300503647	0.972186492	ENSG00000204899	MZT1
443.8316736	-0.465547138	0.294439139	-1.581131977	0.113847865	0.923922314	ENSG00000162585	FAAP20
439.9780419	-0.233295186	0.340979912	-0.684190411	0.493854936	0.993513219	ENSG00000113552	GNPDA1
450.2583262	-0.520221377	0.296880875	-1.752289962	0.079723961	0.897052166	ENSG00000187189	TSPYL4
447.6138983	0.202156381	0.227063589	0.890307345	0.373300878	0.982329093	ENSG00000278053	DDX52
446.1750911	-0.156557475	0.223901929	-0.699223433	0.484412409	0.991892545	ENSG00000139083	ETV6
449.3139661	-0.066859727	0.254684514	-0.262519795	0.792920726	0.999467288	ENSG00000081760	AACS
443.4949186	0.18183412	0.235723532	0.771387222	0.440477447	0.99135833	ENSG00000125124	BBS2
448.2596652	0.095832944	0.36464913	0.262808645	0.792698072	0.999467288	ENSG00000095209	TMEM38B
454.3216073	0.056019985	0.417236741	0.134264267	0.893193608	0.999467288	ENSG00000131459	GFPT2
456.9740367	-0.738515675	0.407771289	-1.811102684	0.070124958	0.878416974	ENSG00000142303	ADAMTS10
447.3799373	-0.1920205	0.253353263	-0.757916031	0.448501252	0.99135833	ENSG00000109118	PHF12
441.71191	-0.319917086	0.244762759	-1.307049679	0.19119584	0.949234949	ENSG00000142546	NOSIP
438.0785466	-0.354225941	0.459274992	-0.771271999	0.440545726	0.99135833	ENSG00000145936	KCNMB1
445.6434127	0.100835089	0.220650417	0.45699025	0.647678054	0.997368416	ENSG00000136100	VPS36
445.8512192	0.104092398	0.241976296	0.430176011	0.667067611	0.997568624	ENSG00000159256	MORC3
446.4488237	-0.188961584	0.264407893	-0.714659391	0.474819536	0.99135833	ENSG00000111276	CDKN1B
444.5419814	0.005545748	0.249862559	0.022195195	0.98229225	0.999527751	ENSG00000087338	GMCL1
442.1600087	0.144095977	0.254097134	0.567090131	0.570652948	0.99483168	ENSG00000072121	ZFYVE26
443.5823268	-0.177504239	0.21767158	-0.815468141	0.414804394	0.990226751	ENSG00000234127	TRIM26
442.8952224	0.665612407	0.325761766	2.04324901	0.041027794	0.800562177	ENSG00000173376	NDNF
445.3585737	-0.10470703	0.237385074	-0.441085144	0.659151358	0.997568624	ENSG00000263465	SRSF8
446.109341	0.087875661	0.309618131	0.283819494	0.776548715	0.999467288	ENSG00000070495	JMJD6
437.9856969	-0.347171982	0.378218881	-0.917912879	0.358664479	0.979427314	ENSG00000159199	ATP5MC1
446.6589751	0.093336775	0.233446317	0.399821151	0.689288251	0.998869317	ENSG00000076053	RBM7
442.0943945	0.555667352	0.276706068	2.008150226	0.04462733	0.811779008	ENSG00000132300	PTCD3
442.237701	-0.18124295	0.258909978	-0.700023038	0.483912917	0.991892545	ENSG00000158985	CDC42SE2
439.3196224	-0.486733288	0.247745783	-1.964648125	0.049454978	0.836346249	ENSG00000188243	COMMD6
443.9566637	0.140748155	0.271537957	0.518336946	0.604223199	0.99483168	ENSG00000009830	POMT2
445.0767402	0.294046296	0.286530013	1.026232094	0.304782197	0.973427483	ENSG00000165282	PIGO
440.0141093	-0.109168003	0.204142744	-0.53476308	0.592813696	0.99483168	ENSG00000108395	TRIM37
446.2535197	0.178105446	0.275135187	0.647337941	0.517413251	0.993513219	ENSG00000005206	SPPL2B
439.4954047	0.139977806	0.297997253	0.469728512	0.638548995	0.996553586	ENSG00000179335	CLK3
438.8063377	-0.156005295	0.254636263	-0.612659381	0.540101586	0.99483168	ENSG00000257267	ZNF271P
440.4990242	0.147846794	0.24990812	0.591604601	0.554115392	0.99483168	ENSG00000266028	SRGAP2
435.4184564	-0.096913938	0.294901648	-0.328631389	0.742434319	0.999467288	ENSG00000129559	NEDD8
441.5305865	-0.318229728	0.227093397	-1.401316516	0.161119445	0.942888225	ENSG00000004961	HCCS

436.7495258	-0.412987881	0.275992301	-1.496374645	0.134556053	0.933741583	ENSG00000176101	SSNA1
439.6631707	0.049463806	0.237754629	0.208045605	0.835193358	0.999467288	ENSG00000112406	HECA
440.8708119	-0.503329632	0.230333478	-2.185221349	0.028872621	0.773201806	ENSG00000138629	UBL7
443.4606495	-0.125163389	0.249111107	-0.502440017	0.615358036	0.99483168	ENSG00000110013	SIAE
440.9158627	-0.012767087	0.244459893	-0.052225692	0.958348862	0.999527751	ENSG00000147130	ZMYM3
440.0336736	-0.417549066	0.271682772	-1.536899312	0.124317968	0.927794614	ENSG00000244313	AC024293.1
440.655413	0.004097257	0.221843432	0.018469138	0.985264598	0.999527751	ENSG00000076924	XAB2
441.480956	0.491445881	0.295363047	1.663870566	0.096138298	0.921147998	ENSG00000118058	KMT2A
441.1483485	0.284244177	0.286297295	0.992828718	0.320793432	0.975337095	ENSG00000155755	TMEM237
438.4531621	0.104024333	0.233687511	0.445142884	0.6562165	0.997568624	ENSG00000028310	BRD9
440.9341921	0.122879661	0.219601189	0.559558268	0.575780777	0.99483168	ENSG00000160208	RRP1B
437.8582556	-0.034766222	0.22838551	-0.15222604	0.879008657	0.999467288	ENSG00000136875	PRPF4
440.6572139	-0.137541432	0.377724024	-0.364132074	0.715759387	0.99900742	ENSG00000109220	CHIC2
452.6208371	-1.296641555	0.442249699	-2.931921852	0.003368715	0.412325011	ENSG00000168675	LDLRAD4
441.5411482	-0.067544756	0.348495632	-0.193818083	0.846318327	0.999467288	ENSG00000116863	ADPRHL2
432.3033961	0.535521806	0.306431408	1.747607433	0.080532027	0.897807954	ENSG00000128805	ARHGAP22
442.2230083	-0.028904532	0.278142583	-0.103919839	0.917232963	0.999527751	ENSG00000121297	TSHZ3
432.5951736	-0.450040165	0.242606617	-1.85502016	0.063593348	0.868202292	ENSG00000172301	COPRS
433.8192941	0.015790002	0.246417063	0.064078364	0.948907829	0.999527751	ENSG00000105171	POP4
438.0234658	-0.329164974	0.277028578	-1.188198618	0.234755168	0.961085539	ENSG00000182552	RWDD4
439.9580735	-0.384865419	0.300940268	-1.278876441	0.20094057	0.954310328	ENSG00000176148	TCP11L1
437.8031272	-0.011984719	0.369181967	-0.032462905	0.974102898	0.999527751	ENSG00000169255	B3GALNT1
434.307644	-0.146907601	0.257537263	-0.57043241	0.568384453	0.99483168	ENSG00000163638	ADAMTS9
434.1161584	0.086649167	0.236240424	0.366783828	0.713780272	0.99900742	ENSG00000099821	POLRMT
435.961918	0.118060075	0.260643255	0.452956572	0.650580013	0.997381078	ENSG00000058799	YIPF1
439.8217185	0.193442999	0.22812954	0.847952433	0.396464462	0.988378925	ENSG00000151881	TMEM267
435.067808	0.492310998	0.232564025	2.116883715	0.034269718	0.782919999	ENSG00000085415	SEH1L
430.7351689	-0.141278552	0.337634598	-0.418436242	0.675628193	0.998869317	ENSG00000261236	BOP1
440.0171031	-0.073070536	0.269242911	-0.271392606	0.786089088	0.999467288	ENSG00000145743	FBXL17
436.7209691	0.034363162	0.298688374	0.115046869	0.908407973	0.999527751	ENSG00000196323	ZBTB44
435.0743606	0.059468106	0.249228981	0.238608309	0.811409321	0.999467288	ENSG00000175283	DOLK
432.7618224	0.354084123	0.23066458	1.53506066	0.124768928	0.927794614	ENSG00000165322	ARHGAP12
431.7956048	-0.173943422	0.32073255	-0.54233168	0.587590035	0.99483168	ENSG00000180340	FZD2
435.6446125	-0.53632619	0.260707419	-2.057195736	0.039667402	0.793985738	ENSG00000104866	PPP1R37
432.0164923	-0.120641257	0.253210887	-0.476445774	0.633756831	0.995376704	ENSG00000139579	NABP2
431.7898234	0.003958421	0.255765189	0.015476778	0.987651811	0.999527751	ENSG00000162852	CNST
430.3083981	-0.097142474	0.278398975	-0.348932587	0.72713992	0.999467288	ENSG00000165609	NUDT5
440.8423735	-0.353328682	0.297115325	-1.189197098	0.234362116	0.960525552	ENSG00000104447	TRPS1
437.3017481	0.168136926	0.238152727	0.706004622	0.480185263	0.99135833	ENSG00000112200	ZNF451
434.7008595	0.052567127	0.210129288	0.250165636	0.802459259	0.999467288	ENSG00000111790	FGFR1OP2
436.4767753	0.038200591	0.222170711	0.171942516	0.863482721	0.999467288	ENSG00000166971	AKTIP
427.3850887	0.672371255	0.337011425	1.995099288	0.046032052	0.815583282	ENSG00000180801	ARSJ
433.679916	0.168595242	0.296071008	0.569441914	0.569056279	0.99483168	ENSG00000130826	DKC1
427.8753978	-0.450531047	0.30898881	-1.458082079	0.14481792	0.935644597	ENSG00000119673	ACOT2
436.01834	0.313742532	0.261167458	1.201307908	0.229631784	0.959575179	ENSG00000139746	RBM26
434.469436	0.19131892	0.290474551	0.658642623	0.510125285	0.993513219	ENSG00000166685	COG1
431.8153376	0.153590017	0.24630713	0.623571137	0.532909271	0.99483168	ENSG00000159921	GNE
433.2179217	-0.276305286	0.216918953	-1.273771987	0.202744236	0.954310328	ENSG00000159479	MED8
439.2539587	0.02580679	0.322568247	0.080004123	0.936233977	0.999527751	ENSG00000185344	ATP6V0A2
432.0528504	0.112197383	0.203916182	0.550213237	0.582173125	0.99483168	ENSG00000205423	CNEP1R1
430.8723906	-0.707875907	0.33313413	-2.124897583	0.03359516	0.775963115	ENSG00000134668	SPOCD1
429.0157906	0.066399117	0.217108458	0.305833857	0.759731149	0.999467288	ENSG00000149600	COMMD7
426.4645371	-0.468599354	0.280353387	-1.671459576	0.094630937	0.921147998	ENSG00000188186	LAMTOR4
427.6234428	0.351527041	0.257342773	1.365987616	0.17194285	0.944422693	ENSG00000072415	MPP5
427.2952197	-0.266456656	0.288965048	-0.922106869	0.356472834	0.979427314	ENSG00000178307	TMEM11
424.8173168	0.092910127	0.404065886	0.229938063	0.818139899	0.999467288	ENSG00000112972	HMGCS1
418.4993418	-0.702836285	0.414104076	-1.697245514	0.089650253	0.910413547	ENSG00000103145	HCFC1R1
437.79752	-0.325356804	0.412638568	-0.788478898	0.430416637	0.991301167	ENSG00000197019	SERTAD1
431.0741137	-0.484591341	0.268166645	-1.807053001	0.070754016	0.88094849	ENSG00000160094	ZNF362
433.5085474	-0.037035936	0.227352155	-0.162901186	0.870596237	0.999467288	ENSG00000224531	SMIM13
427.477187	-0.051377812	0.250876797	-0.204792999	0.837733857	0.999467288	ENSG00000272047	GTF2H5
426.9649709	0.695561307	0.47735553	1.457113751	0.145084983	0.935644597	ENSG00000114346	ECT2
432.9504658	0.657229796	0.300689536	2.185742162	0.028834474	0.773201806	ENSG00000113649	TCERG1
428.4917435	0.310306218	0.29610761	1.047950839	0.294661258	0.970706863	ENSG00000196510	ANAPC7

432.8692491	-0.186642107	0.34218889	-0.545435904	0.585453752	0.99483168	ENSG00000092445	TYRO3
425.2907288	-0.330452164	0.28297716	-1.167769735	0.242899659	0.962465655	ENSG00000181924	COA4
431.6037213	0.452751175	0.22825225	1.983556241	0.047305331	0.827156057	ENSG00000113569	NUP155
431.7007551	-0.274585658	0.23034263	-1.192074857	0.233231895	0.959684953	ENSG00000129675	ARHGEF6
432.1060086	-0.29578317	0.376837049	-0.784910005	0.432506335	0.991301167	ENSG00000205213	LGR4
428.2202273	-0.152851357	0.234810346	-0.650956655	0.515074467	0.993513219	ENSG00000181610	MRPS23
428.0557567	-0.360753621	0.219445	-1.643936392	0.100189342	0.92169898	ENSG00000146576	C7orf26
432.8112129	0.411965186	0.262743455	1.567937006	0.116895834	0.923922314	ENSG00000119285	HEATR1
436.1551412	-0.377258015	0.316165936	-1.193227899	0.232780131	0.959575179	ENSG00000161970	RPL26
427.4817629	0.137925858	0.211370825	0.652530253	0.514059163	0.993513219	ENSG00000175470	PPP2R2D
427.4725422	-0.211219303	0.26085393	-0.809722528	0.418099668	0.990226751	ENSG00000072135	PTPN18
429.917427	0.046466008	0.224095748	0.207348905	0.835737383	0.999467288	ENSG00000148943	LIN7C
421.7217894	-0.039879959	0.330307523	-0.120735848	0.903900264	0.999527751	ENSG00000025039	RRAGD
428.5667782	-0.194284635	0.259582768	-0.748449662	0.454188977	0.99135833	ENSG00000131323	TRAF3
430.3810529	0.091296131	0.246512514	0.370350897	0.711121055	0.99900742	ENSG00000185115	NSMCE3
429.9181025	0.023022967	0.225374707	0.102154173	0.918634302	0.999527751	ENSG00000101343	CRNKL1
435.7252295	-0.202061378	0.499720314	-0.404348938	0.685956144	0.998869317	ENSG00000197766	CFD
425.5769858	0.005804871	0.314997521	0.018428307	0.98529717	0.999527751	ENSG00000232119	MCTS1
427.3757003	0.09043708	0.259431579	0.348597037	0.727391853	0.999467288	ENSG00000047621	C12orf4
427.6636741	-0.011124537	0.231549312	-0.048043923	0.961681238	0.999527751	ENSG00000119953	SMNDC1
427.5648915	-0.293923239	0.23973036	-1.226057635	0.220176987	0.956884001	ENSG00000130669	PAK4
425.4258127	-0.192089737	0.254926093	-0.75351148	0.451142616	0.99135833	ENSG00000104872	PIH1D1
425.8551949	0.02522841	0.279104737	0.090390477	0.927976924	0.999527751	ENSG00000006607	FARP2
431.4724222	-0.083567631	0.260034929	-0.321370793	0.747929414	0.999467288	ENSG00000165669	FAM204A
428.128102	0.263462554	0.209678565	1.256506853	0.208932263	0.954310328	ENSG00000111358	GTF2H3
426.4380181	0.338195615	0.46580544	0.726044796	0.467811309	0.99135833	ENSG00000115525	ST3GAL5
427.0206854	-0.164489248	0.209176677	-0.786365148	0.431653595	0.991301167	ENSG00000204209	DAXX
431.3337501	-0.468639157	0.256294819	-1.82851592	0.067472159	0.87420149	ENSG00000138172	CALHM2
426.8522523	-0.072211569	0.272111782	-0.26537465	0.790720863	0.999467288	ENSG00000187239	FNBP1
430.9919894	-0.494395065	0.488402866	-1.012268969	0.311409468	0.973427483	ENSG00000145934	TENM2
423.8075108	-0.025833476	0.284315153	-0.090862115	0.927602153	0.999527751	ENSG00000172965	MIR4435-2HG
426.0661319	0.077714802	0.244588638	0.317736762	0.750684623	0.999467288	ENSG00000204070	SYS1
422.1960432	-0.119101391	0.216604176	-0.549857317	0.582417242	0.99483168	ENSG00000140688	C16orf58
423.6156343	-0.130100783	0.232505164	-0.559560833	0.575779027	0.99483168	ENSG00000183735	TBK1
420.8640833	-0.417939969	0.212016528	-1.971261262	0.048694	0.83480477	ENSG00000181104	F2R
418.4455403	0.531745303	0.336489405	1.580273539	0.114044237	0.923922314	ENSG00000162804	SNED1
429.3268214	0.04258331	0.305696554	0.139299281	0.889213658	0.999467288	ENSG00000100441	KHNYN
422.2082507	0.00185273	0.26214348	0.007067617	0.994360904	0.999527751	ENSG00000144029	MRPS5
424.4063304	0.349244039	0.265803381	1.313918719	0.188873559	0.949094286	ENSG00000147202	DIAPH2
418.8735254	-0.570791663	0.325834678	-1.751783038	0.079811122	0.897052166	ENSG00000174917	C19orf70
424.2949579	-0.025656106	0.318023805	-0.08067354	0.93570158	0.999527751	ENSG00000213672	NCKIPSD
419.6130592	0.113084451	0.234560845	0.482111373	0.629726831	0.99483168	ENSG00000081791	DELE1
424.4689832	-0.299560617	0.349078227	-0.858147525	0.390811007	0.987833983	ENSG00000137166	FOXP4
416.6471434	-0.121728472	0.371570492	-0.327605325	0.743210095	0.999467288	ENSG00000135709	KIAA0513
426.7741997	0.36922068	0.301619856	1.224125908	0.220904734	0.956884001	ENSG00000101745	ANKRD12
422.1736327	-0.280799086	0.279824221	-1.003483846	0.315627467	0.974768742	ENSG00000167969	ECI1
421.6027244	-0.202887542	0.253535815	-0.800232272	0.423576235	0.991301167	ENSG00000149196	HIKESHI
424.978007	-0.071111639	0.313059299	-0.227150701	0.82030656	0.999467288	ENSG00000083290	ULK2
419.8762084	-0.110251114	0.23053102	-0.4782485	0.632473346	0.994860874	ENSG00000116918	TSNAX
422.7229633	-0.3307648	0.25175065	-1.31385877	0.188893736	0.949094286	ENSG00000164086	DUSP7
421.0326958	-0.17459894	0.237870986	-0.73400688	0.462944545	0.99135833	ENSG00000118579	MED28
421.3114637	-0.314109843	0.265446044	-1.183328401	0.236679021	0.962465655	ENSG00000104412	EMC2
422.1799648	-0.177225205	0.286317511	-0.618981368	0.535928633	0.99483168	ENSG00000178425	NT5DC1
420.4716872	0.186531161	0.265556067	0.702417244	0.482418994	0.991543694	ENSG00000143970	ASXL2
423.8410619	0.090748306	0.252194433	0.359834691	0.718970759	0.99900742	ENSG00000089916	GPATCH2L
421.5740501	-0.50388079	0.283010724	-1.780430028	0.075005611	0.893390506	ENSG00000178397	FAM220A
420.2991558	-1.096588098	0.387432747	-2.830396004	0.004649042	0.47075409	ENSG00000103647	CORO2B
421.9620367	-0.121485085	0.253417407	-0.479387295	0.63166313	0.99483168	ENSG00000105829	BET1
420.0058122	-0.259415272	0.261041575	-0.993769944	0.320334882	0.975316319	ENSG00000163348	PYGO2
416.8760393	0.000606641	0.260348119	0.002330114	0.99814084	0.999527751	ENSG00000138386	NAB1
421.0663584	-0.097609109	0.244692033	-0.398905956	0.6899625	0.998869317	ENSG00000147364	FBXO25
418.6577093	-0.25525943	0.231950307	-1.100491879	0.271117866	0.962465655	ENSG00000103496	STX4
420.5872309	-0.082834189	0.299732115	-0.276360739	0.782271008	0.999467288	ENSG00000184939	ZFP90
418.5918447	-0.338875738	0.359205725	-0.943402944	0.345474838	0.977691382	ENSG00000175155	YPEL2

420.6542194	-0.124278067	0.277243528	-0.448263187	0.653963266	0.997527562	ENSG00000158850	B4GALT3
423.3308669	0.126612106	0.260739047	0.485589355	0.627258346	0.99483168	ENSG00000117597	UTP25
416.5046464	-0.466280672	0.284708709	-1.637746434	0.1014746	0.92169898	ENSG00000165782	PIP4P1
421.8980031	-0.318079147	0.246736186	-1.289146732	0.19734708	0.954310328	ENSG00000120963	ZNF706
414.5545456	0.140520876	0.278982938	0.503689855	0.614479339	0.99483168	ENSG00000157045	NTAN1
424.8152093	0.079390017	0.304789203	0.260475161	0.794497273	0.999467288	ENSG00000153046	CDYL
420.5423576	-0.392928645	0.328128538	-1.197483911	0.231117995	0.959575179	ENSG00000052126	PLEKHA5
424.7451793	0.097668948	0.266799781	0.366075816	0.714308503	0.99900742	ENSG00000166938	DIS3L
418.5998595	-0.148777682	0.268566378	-0.553969871	0.579599465	0.99483168	ENSG00000137992	DBT
421.2572928	0.37550042	0.405823656	0.925279773	0.354820398	0.977691382	ENSG00000169359	SLC33A1
422.5245186	0.272882249	0.259185713	1.052844489	0.292412271	0.970706863	ENSG00000120071	KANSL1
416.8925499	-0.672499168	0.346519819	-1.940723537	0.05229182	0.846840376	ENSG00000171159	C9orf16
424.5858105	-0.373168042	0.456001105	-0.818348985	0.413157943	0.990226751	ENSG00000141582	CBX4
421.6915974	-0.324355621	0.243991455	-1.32937287	0.183724984	0.949094286	ENSG00000040933	INPP4A
421.4810425	-0.126220363	0.272329874	-0.46348335	0.643017947	0.997097166	ENSG00000167106	FAM102A
417.9715711	-0.122680478	0.242021983	-0.506898077	0.612226335	0.99483168	ENSG00000150316	CWC15
419.5249884	-0.042013276	0.246799975	-0.170232092	0.864827614	0.999467288	ENSG00000165997	ARL5B
416.0583408	-0.174640045	0.26245141	-0.66541858	0.505782804	0.993513219	ENSG00000214706	IFRD2
417.0399182	0.022950915	0.259548087	0.088426446	0.929537742	0.999527751	ENSG00000092020	PPP2R3C
417.0022681	0.440070623	0.220966235	1.991574069	0.046417811	0.818574412	ENSG00000142867	BCL10
413.6000388	-0.343245829	0.226618826	-1.514639521	0.129863718	0.929373153	ENSG00000143740	SNAP47
420.7536841	-0.056679693	0.266189736	-0.212929669	0.83138182	0.999467288	ENSG00000170265	ZNF282
417.0322222	0.276926519	0.358413941	0.772644385	0.439732869	0.991301167	ENSG00000139178	C1RL
418.7750408	0.049084144	0.214934914	0.228367476	0.819360572	0.999467288	ENSG00000126226	PCID2
416.2668837	-0.004077644	0.263188592	-0.01549324	0.987638678	0.999527751	ENSG00000154803	FLCN
418.3095765	-0.217903281	0.280252136	-0.777525855	0.436848586	0.991301167	ENSG00000065989	PDE4A
421.0272097	0.196481182	0.258989777	0.758644544	0.448065219	0.99135833	ENSG00000004534	RBM6
417.4753732	0.217215258	0.292139631	0.743532321	0.457159461	0.99135833	ENSG00000133138	TBC1D8B
412.3131691	-0.671358033	0.363744497	-1.845685744	0.064937848	0.868202292	ENSG00000119632	IFI27L2
418.7890325	0.021029249	0.321326651	0.065445081	0.947819632	0.999527751	ENSG00000124383	MPHOSPH10
408.9236714	0.411897012	0.445368933	0.92484451	0.355046794	0.977691382	ENSG00000124212	PTGIS
416.3351952	-0.103092332	0.225581196	-0.457007647	0.647665549	0.997368416	ENSG00000136718	IMP4
423.7801598	0.85827565	0.348237625	2.464626419	0.013715617	0.672931175	ENSG00000175455	CCDC14
418.5960501	-0.023166429	0.238116056	-0.097290494	0.922495705	0.999527751	ENSG00000163374	YY1AP1
416.0401769	0.174875224	0.291825668	0.59924552	0.549009172	0.99483168	ENSG00000147535	PLPP5
412.5879868	-0.052041787	0.294721436	-0.17657958	0.859838635	0.999467288	ENSG00000130517	PGPEP1
412.1942389	0.164628739	0.271989359	0.605276398	0.544995361	0.99483168	ENSG00000163932	PRKCD
418.3516725	0.285052209	0.481161521	0.592425197	0.553565898	0.99483168	ENSG00000150051	MKX
416.989099	-0.162361143	0.220206174	-0.737314216	0.460931289	0.99135833	ENSG00000126391	FRMD8
415.186907	0.221805351	0.221818585	0.999940339	0.317339381	0.974768742	ENSG00000148688	RPP30
415.9242645	-0.335528737	0.247045302	-1.358166841	0.17441075	0.945931076	ENSG00000143256	PFDN2
416.4837641	0.346635026	0.236818142	1.463718204	0.143270959	0.935644597	ENSG00000104154	SLC30A4
414.8938752	0.5582317	0.423279874	1.318824101	0.187227925	0.949094286	ENSG00000188549	CCDC9B
416.8216281	0.150084631	0.244784803	0.613128873	0.53979113	0.99483168	ENSG00000197323	TRIM33
413.1106326	0.07414272	0.231941347	0.319661505	0.749224944	0.999467288	ENSG00000119718	EIF2B2
413.777841	-0.081276808	0.29787598	-0.272854521	0.784965048	0.999467288	ENSG00000181396	OGFOD3
408.9794322	-0.29105813	0.375776794	-0.77455057	0.438605275	0.991301167	ENSG00000118960	HS1BP3
412.2575303	-0.085844654	0.251084863	-0.341894979	0.732429928	0.999467288	ENSG00000091640	SPAG7
418.2981445	-0.18074357	0.233363546	-0.774515012	0.438626294	0.991301167	ENSG00000138375	SMARCAL1
419.076302	-0.116620163	0.273664432	-0.426142928	0.670003701	0.997568624	ENSG00000177463	NR2C2
416.7024371	0.125321126	0.253912716	0.493559865	0.621617051	0.99483168	ENSG00000175073	VCPIP1
413.0137797	-0.279863518	0.24477223	-1.143363028	0.252887896	0.962465655	ENSG00000159210	SNF8
414.9043747	0.350647397	0.242821159	1.444056189	0.14872317	0.936396974	ENSG00000112282	MED23
414.7656381	-0.144250525	0.233689589	-0.617274075	0.537053964	0.99483168	ENSG00000170340	B3GNT2
415.7024166	0.049456138	0.245122953	0.201760535	0.840103935	0.999467288	ENSG00000119844	AFTPH
412.8463757	-0.130117988	0.226767156	-0.573795564	0.566106151	0.99483168	ENSG00000141873	SLC39A3
427.9092613	0.494796042	0.85048717	0.581779549	0.560715181	0.99483168	ENSG00000197594	ENPP1
412.0048971	0.136744484	0.260488715	0.524953581	0.59961546	0.99483168	ENSG00000155008	APOOL
413.5049235	-0.204593436	0.268813671	-0.761097584	0.446598783	0.99135833	ENSG00000165633	VSTM4
414.1944106	0.100756671	0.235348431	0.428117031	0.668565918	0.997568624	ENSG00000115540	MOB4
415.2440351	0.198972128	0.219464235	0.906626668	0.364604254	0.980994643	ENSG00000164366	CCDC127
412.3041605	-0.478702801	0.269007486	-1.779514795	0.075155402	0.893390506	ENSG00000173272	MZT2A
420.2119622	0.101493394	0.29402064	0.345191391	0.72995049	0.999467288	ENSG00000031003	FAM13B
414.6699985	0.007314786	0.261394313	0.027983723	0.977675133	0.999527751	ENSG00000131725	WDR44

415.5432948	-0.245494542	0.229834356	-1.068136836	0.285458792	0.96478822	ENSG00000112242	E2F3
413.3236093	0.294873769	0.276516371	1.066388106	0.286248236	0.964826902	ENSG00000092931	MFSD11
411.387031	-0.328904319	0.276814978	-1.188173852	0.234764923	0.961085539	ENSG00000228300	C19orf24
413.2095534	-0.150288396	0.249276218	-0.602899052	0.546575847	0.99483168	ENSG00000100941	PNN
405.6043702	0.175735431	0.3540378	0.496374768	0.619630024	0.99483168	ENSG00000162783	IER5
411.2733774	0.245577817	0.298948673	0.821471508	0.411377747	0.990226751	ENSG00000106591	MRPL32
408.2768373	-0.383404802	0.284354396	-1.348334359	0.177550866	0.946844293	ENSG00000116586	LAMTOR2
410.4564509	-0.003908156	0.265563602	-0.01471646	0.988258387	0.999527751	ENSG00000205250	E2F4
412.836362	-0.009951628	0.250027352	-0.039802159	0.968250855	0.999527751	ENSG00000167962	ZNF598
412.0229294	-0.301502146	0.2205738	-1.366899179	0.17165691	0.944422693	ENSG00000135148	TRAFD1
411.2146883	-0.014827974	0.220203447	-0.067337612	0.946312935	0.999527751	ENSG00000100442	FKBP3
413.7923455	0.30317637	0.27494426	1.102683032	0.270164838	0.962465655	ENSG00000054267	ARID4B
413.3235667	0.04270761	0.257567044	0.165811623	0.868305199	0.999467288	ENSG00000012963	UBR7
411.417753	0.16501724	0.244236361	0.67564567	0.499265638	0.993513219	ENSG00000198522	GPN1
412.6707817	-0.220613548	0.216154489	-1.020629039	0.307430226	0.973427483	ENSG00000179021	C3orf38
414.1719757	-0.365237006	0.291949468	-1.251028159	0.210924204	0.954310328	ENSG00000134086	VHL
408.8977952	0.228843404	0.260725228	0.877718685	0.380096402	0.983585589	ENSG00000169814	BTD
416.1426832	0.162859328	0.292869669	0.556081237	0.578155321	0.99483168	ENSG00000149177	PTPRJ
410.4455803	0.866488354	0.248332972	3.48921992	0.000484432	0.1830637	ENSG00000107281	NPDC1
406.2596043	0.250138085	0.465524321	0.537325491	0.5910428	0.99483168	ENSG00000240184	PCDHGC3
409.7680259	0.282492287	0.293453155	0.962648663	0.335723868	0.977592706	ENSG00000110057	UNC93B1
411.3147992	0.425762151	0.230907498	1.843864557	0.065202882	0.868202292	ENSG00000134884	ARGLU1
436.3634061	-0.495260772	1.248559951	-0.396665592	0.691614077	0.998869317	ENSG00000150594	ADRA2A
415.1147979	0.012689307	0.282887083	0.044856439	0.964221738	0.999527751	ENSG00000110851	PRDM4
414.5473804	0.486006414	0.333088613	1.459090449	0.144540215	0.935644597	ENSG00000112941	TENT4A
411.4811933	0.05419787	0.27675668	0.195832203	0.844741503	0.999467288	ENSG00000172172	MRPL13
411.9318	0.204413693	0.246775035	0.828340246	0.407477836	0.990226751	ENSG00000166037	CEP57
408.0742099	-0.049657617	0.227596139	-0.218183039	0.8272865	0.999467288	ENSG00000148308	GTF3C5
408.5033642	-0.017436627	0.298543733	-0.058405604	0.953425551	0.999527751	ENSG00000175792	RUVBL1
412.6256635	0.845746266	0.879649805	0.961457914	0.336321977	0.977691382	ENSG00000161800	RACGAP1
409.969993	0.230907476	0.269527612	0.856711764	0.3916042	0.988355932	ENSG00000182093	WRB
411.3468495	-0.213429048	0.248056569	-0.860404739	0.389565974	0.987216662	ENSG00000128581	IFT22
410.5041159	0.028090052	0.219172094	0.128164363	0.898018902	0.999467288	ENSG00000110435	PDHX
408.8573647	0.442570723	0.277366725	1.595615781	0.110574602	0.923922314	ENSG00000157617	C2CD2
408.1413491	-0.33404476	0.267114773	-1.250566399	0.211092716	0.954310328	ENSG00000132313	MRPL35
414.7869553	0.372903088	0.389371807	0.95770439	0.338211843	0.977691382	ENSG00000127914	AKAP9
419.5320308	-0.129932539	0.507264586	-0.256143524	0.797840015	0.999467288	ENSG00000125675	GRIA3
408.2857574	-0.055147704	0.223091133	-0.247198098	0.804754917	0.999467288	ENSG00000160194	NDUFV3
409.6262834	0.297437043	0.271848214	1.094129108	0.273898358	0.962465655	ENSG00000198837	DENND4B
412.243648	-0.318457472	0.243541481	-1.307610807	0.19100535	0.949234949	ENSG00000198252	STYX
413.7340322	-0.173529442	0.270397717	-0.641756312	0.52103142	0.993513219	ENSG00000100099	HPS4
409.8356079	0.213733535	0.248409861	0.860406805	0.389564836	0.987216662	ENSG00000154640	BTG3
415.5327447	-0.23780984	0.235088151	-1.011577313	0.311740198	0.973427483	ENSG00000233913	RPL10P9
431.5577869	-0.765236626	0.919112764	-0.832581872	0.405080586	0.989962822	ENSG00000086991	NOX4
411.0905514	0.099057584	0.227231958	0.435931567	0.662886381	0.997568624	ENSG00000151422	FER
406.5773963	-0.091754686	0.245672639	-0.373483536	0.708788597	0.99900742	ENSG00000113811	SELENOK
403.8141785	0.132518747	0.268841394	0.492925383	0.622065311	0.99483168	ENSG00000148660	CAMK2G
405.4817957	-0.095409246	0.258217686	-0.369491521	0.711761392	0.99900742	ENSG00000137502	RAB30
407.5736489	0.05433718	0.218022535	0.249227356	0.80318492	0.999467288	ENSG00000163798	SLC4A1AP
404.8704669	-0.348559969	0.306697433	-1.136494577	0.255749634	0.962465655	ENSG00000100711	ZFYVE21
407.0360695	-0.061345333	0.239580413	-0.256053205	0.797909755	0.999467288	ENSG00000089050	RBBP9
407.7555598	-0.268644721	0.234181891	-1.147162661	0.2513144	0.962465655	ENSG00000143553	SNAPIN
408.7674166	0.085173157	0.225063023	0.378441362	0.705102746	0.99900742	ENSG00000133731	IMPA1
409.2719223	-0.022331076	0.212629065	-0.105023631	0.916357058	0.999527751	ENSG00000112234	FBXL4
407.9951147	-0.484493162	0.339312769	-1.427865989	0.153330435	0.939432741	ENSG00000105722	ERF
407.6057364	0.390760095	0.235446785	1.659653562	0.096984166	0.921147998	ENSG00000116138	DNAJC16
407.3720615	0.023698971	0.239388072	0.098998128	0.921139756	0.999527751	ENSG00000165271	NOL6
406.7216171	1.272163593	0.368596604	3.451370896	0.000557747	0.195790358	ENSG00000175592	FOSL1
408.0265276	0.028880098	0.243921516	0.11839914	0.905751407	0.999527751	ENSG00000126243	LRFN3
408.7061451	0.254065401	0.288317126	0.881201212	0.378208923	0.982609538	ENSG00000151461	UPF2
408.7664722	0.110180026	0.277048385	0.39769236	0.69085697	0.998869317	ENSG00000167447	SMG8
406.9268946	0.013005711	0.208575622	0.062354893	0.950280215	0.999527751	ENSG00000066855	MTFR1
406.9837277	-0.282309757	0.272700106	-1.035238898	0.300557357	0.972186492	ENSG00000235174	RPL39P3
405.4659766	-0.027905592	0.224582382	-0.124255483	0.901112993	0.999527751	ENSG00000082512	TRAF5

406.497739	-0.117308104	0.220579503	-0.531817791	0.5948522	0.99483168	ENSG00000137103	TMEM8B
408.6476558	-0.057968728	0.364001578	-0.159254056	0.873468717	0.999467288	ENSG00000151693	ASAP2
405.5200514	0.011196977	0.256518567	0.04364977	0.965183578	0.999527751	ENSG00000121898	CPXM2
412.2716455	-0.171467291	0.430031565	-0.398731872	0.690090781	0.998869317	ENSG00000140332	TLE3
403.50711	-0.024651128	0.243825029	-0.101101712	0.919469719	0.999527751	ENSG00000168818	STX18
403.7406825	0.193942993	0.274189141	0.707332872	0.479359642	0.99135833	ENSG00000065357	DGKA
406.4690929	-0.072283277	0.262969427	-0.27487331	0.783413573	0.999467288	ENSG00000163577	EIF5A2
405.8209603	-0.096419831	0.22877601	-0.421459536	0.673419553	0.99853019	ENSG00000176248	ANAPC2
405.7433249	-0.088418189	0.33564397	-0.263428505	0.792220323	0.999467288	ENSG00000107249	GLIS3
409.8753569	-0.143872087	0.267528386	-0.537782512	0.590727207	0.99483168	ENSG00000099810	MTAP
402.6345067	0.015694545	0.258046549	0.060820595	0.951502088	0.999527751	ENSG00000180198	RCC1
411.1164011	0.037649956	0.37947052	0.099217078	0.920965915	0.999527751	ENSG00000196313	POM121
405.4520834	-0.502356473	0.221942312	-2.263455171	0.023607645	0.744948012	ENSG00000099940	SNAP29
412.0027533	2.127787074	1.374584039	1.547949789	0.121634386	0.923922314	ENSG00000117724	CENPF
405.5802967	0.316539331	0.272616072	1.161117641	0.245594062	0.962465655	ENSG00000143643	TTC13
405.3205498	-0.153977831	0.221343955	-0.695649588	0.486648303	0.99350043	ENSG00000180185	FAHD1
406.6562579	0.137513567	0.235358175	0.584273596	0.559036254	0.99483168	ENSG00000116793	PHTF1
401.6842705	0.179569735	0.343501435	0.522762693	0.601139404	0.99483168	ENSG00000113460	BRIX1
374.0681804	1.38432806	1.221739057	1.133079974	0.25718067	0.962465655	ENSG00000118898	PPL
411.4286657	0.170125612	0.315095308	0.539917948	0.58925362	0.99483168	ENSG00000125772	GPCPD1
408.4664105	0.090888585	0.27333632	0.332515582	0.739499976	0.999467288	ENSG00000119041	GTF3C3
407.560973	0.152504244	0.276712827	0.551128205	0.581545791	0.99483168	ENSG00000149231	CCDC82
407.8066082	0.295455694	0.321602876	0.918697301	0.358253922	0.979427314	ENSG00000095564	BTAF1
401.5087938	-0.133733749	0.246595344	-0.542320658	0.587597627	0.99483168	ENSG00000163159	VPS72
404.1922953	-0.608568547	0.243432996	-2.499942723	0.012421339	0.657594515	ENSG00000170619	COMMD5
403.9195723	0.3147201	0.305335886	1.030734069	0.302665544	0.972793733	ENSG00000115368	WDR75
401.828765	0.307892683	0.295625382	1.0414961	0.297645362	0.972186492	ENSG00000131584	ACAP3
403.0632676	0.04291678	0.29386362	0.146043187	0.883887296	0.999467288	ENSG00000122882	ECD
405.7970753	-0.262660081	0.232794907	-1.128289634	0.25919762	0.962465655	ENSG00000120860	WASHC3
403.3912571	0.000316579	0.207664234	0.001524474	0.998783646	0.999527751	ENSG00000269556	TMEM185A
404.3633961	-0.201167339	0.257269695	-0.781931734	0.434254694	0.991301167	ENSG00000173011	TADA2B
400.9689456	-0.225046166	0.238196917	-0.944790426	0.344765881	0.977691382	ENSG00000184319	RPL23AP82
403.5241622	-0.071570536	0.218151889	-0.328076628	0.742853725	0.999467288	ENSG00000130803	ZNF317
401.2937077	-0.308408361	0.310066506	-0.994652294	0.319905404	0.974964731	ENSG00000107262	BAG1
401.7161529	0.088074377	0.243475222	0.361738563	0.717547406	0.99900742	ENSG00000135457	TFCP2
401.7196564	-0.07654313	0.24301244	-0.31497618	0.75277974	0.999467288	ENSG00000160785	SLC25A44
402.7670452	0.12889569	0.243708574	0.528892719	0.596879873	0.99483168	ENSG00000111725	PRKAB1
403.6729821	-0.581656525	0.340163127	-1.709934086	0.087278062	0.90492251	ENSG00000170604	IRF2BP1
406.6693297	-0.041743006	0.295215269	-0.141398534	0.887555112	0.999467288	ENSG00000157693	TMEM268
398.8528512	-0.097996636	0.330477258	-0.296530649	0.766824865	0.999467288	ENSG00000239672	NME1
402.3833398	-0.236230713	0.264251572	-0.89396143	0.371342528	0.981955904	ENSG00000132376	INPP5K
400.3557879	-0.120480461	0.269824757	-0.446513738	0.655226191	0.997568624	ENSG00000100982	PCIF1
396.4949585	0.151162495	0.380873057	0.39688419	0.691452864	0.998869317	ENSG00000126947	ARMCX1
397.3647039	-0.430563634	0.280639643	-1.534222426	0.124974942	0.927794614	ENSG00000227057	WDR46
397.4080676	-0.172701765	0.249594945	-0.691928134	0.488982462	0.993513219	ENSG00000184117	NIPSNAP1
388.1600144	0.193551889	0.531687861	0.364032927	0.715833421	0.99900742	ENSG00000101347	SAMHD1
400.0805244	0.470941574	0.240099652	1.961442135	0.049827469	0.836811782	ENSG00000115159	GPD2
400.040781	0.162525464	0.217350761	0.747756592	0.454606989	0.99135833	ENSG00000087269	NOP14
402.4252275	0.112009183	0.211376574	0.529903482	0.596178852	0.99483168	ENSG00000214753	HNRNPUL2
397.8227865	0.190706687	0.265465549	0.718385823	0.472519426	0.99135833	ENSG00000127419	TMEM175
401.0156097	0.07939309	0.252103909	0.314922091	0.752820809	0.999467288	ENSG00000065183	WDR3
401.4634999	0.272143319	0.31564716	0.862175725	0.388590826	0.986799595	ENSG00000198000	NOL8
399.3553166	-0.099084216	0.240450562	-0.412077291	0.680282773	0.998869317	ENSG00000111880	RNGTT
401.6453089	-0.34232844	0.263522593	-1.299047781	0.193927532	0.950624691	ENSG00000169957	ZNF768
403.8866648	-0.301489975	0.300624259	-1.002879728	0.315918895	0.974768742	ENSG00000164114	MAP9
393.6780058	-0.064474494	0.316411659	-0.203767755	0.838534992	0.999467288	ENSG00000177556	ATOX1
392.6554544	-0.186074106	0.35215481	-0.528387233	0.597230597	0.99483168	ENSG00000153179	RASSF3
400.7960452	-0.743924018	0.329117157	-2.260362313	0.023798774	0.744948012	ENSG00000137094	DNAJB5
398.1874343	-0.349389525	0.223017385	-1.566647035	0.117197217	0.923922314	ENSG00000129518	EAPP
400.8332755	0.085422586	0.235347816	0.362963155	0.716632408	0.99900742	ENSG00000121864	ZNF639
398.2518733	-0.566771397	0.233021992	-2.43226569	0.015004696	0.691682485	ENSG00000185420	SMYD3
398.483276	-0.146560548	0.305915364	-0.47908855	0.631875635	0.99483168	ENSG00000173621	LRFN4
400.5134903	0.245728595	0.238159898	1.031779897	0.302175239	0.972793733	ENSG00000178074	C2orf69
404.2054793	0.150706742	0.323289299	0.466166811	0.641096103	0.996553586	ENSG00000179562	GCC1

398.3087284	-0.559756706	0.26188727	-2.137395624	0.032565827	0.775963115	ENSG00000111678	C12orf57
398.7081338	-0.210399958	0.231507597	-0.908825288	0.363442356	0.98048242	ENSG00000162302	RPS6KA4
401.7440274	-0.065850188	0.229363365	-0.287099851	0.774035866	0.999467288	ENSG00000125779	PANK2
399.0158458	-0.169013909	0.224750286	-0.752007536	0.452046527	0.99135833	ENSG00000100281	HMGXB4
398.6732137	0.091492932	0.213846145	0.427844663	0.668764218	0.997568624	ENSG00000183520	UTP11
396.8229996	0.437135885	0.323312525	1.352053669	0.176358147	0.946278819	ENSG00000109586	GALNT7
399.468628	0.16312566	0.225788889	0.72246983	0.470005668	0.99135833	ENSG00000147601	TERF1
398.0542126	-0.058562119	0.346704542	-0.168910734	0.865866856	0.999467288	ENSG00000185112	FAM43A
398.1967399	-0.22183659	0.264187475	-0.839693817	0.401080081	0.988492517	ENSG00000205133	TRIQK
395.8683278	-0.011591321	0.255013728	-0.045453715	0.963745666	0.999527751	ENSG00000104980	TIMM44
394.4775739	-0.006338026	0.30320585	-0.020903377	0.983322733	0.999527751	ENSG00000105849	TWISTNB
401.2190309	0.044793939	0.242489572	0.184725216	0.853444567	0.999467288	ENSG00000104365	IKBKB
398.1139915	-0.001313612	0.252741207	-0.00519746	0.995853045	0.999527751	ENSG00000136631	VPS45
395.8073262	0.340599367	0.344659185	0.988220775	0.323044536	0.975337095	ENSG00000162511	LAPTM5
390.9824194	0.282722946	0.309535268	0.913378783	0.361043361	0.979465649	ENSG00000168890	TMEM150A
399.4398822	0.015968988	0.23556153	0.067791153	0.945951886	0.999527751	ENSG00000145545	SRD5A1
402.2117776	0.043422245	0.366942501	0.118335285	0.905802001	0.999527751	ENSG00000132510	KDM6B
397.0658242	-0.204412425	0.257458863	-0.793961498	0.427217846	0.991301167	ENSG00000198382	UVRAG
398.066167	0.112427656	0.241511529	0.465516724	0.641561463	0.996566167	ENSG00000151445	VIPAS39
397.3000027	-0.305844784	0.276205667	-1.107308142	0.268160725	0.962465655	ENSG00000006704	GTF2IRD1
399.0696587	0.088580643	0.25422376	0.348435738	0.727512967	0.999467288	ENSG00000165525	NEMF
399.8966362	0.000521814	0.302097267	0.001727306	0.99862181	0.999527751	ENSG00000153561	RMND5A
395.5203585	0.208719427	0.284992297	0.732368659	0.463943583	0.99135833	ENSG00000131844	MCCC2
396.4023654	-0.258020474	0.331496547	-0.77835041	0.436362465	0.991301167	ENSG00000213753	CENPBD1P1
396.3312733	0.160526383	0.240574135	0.66726368	0.504603721	0.993513219	ENSG00000165097	KDM1B
394.3775756	-0.251745471	0.242620621	-1.037609541	0.299451874	0.972186492	ENSG00000256537	SMIM10L1
395.8311904	0.271363315	0.213641945	1.27017808	0.204021204	0.954310328	ENSG00000100523	DDHD1
394.811395	0.010273267	0.23588392	0.043552215	0.965261343	0.999527751	ENSG00000129472	RAB2B
398.6201351	-0.619061679	0.256457902	-2.413892006	0.01578314	0.696728876	ENSG00000148158	SNX30
394.8790054	0.148581106	0.30117756	0.493333919	0.621776665	0.99483168	ENSG00000163029	SMC6
400.1423767	-0.1415264	0.329296968	-0.429783491	0.667353143	0.997568624	ENSG00000132031	MATN3
396.2005912	0.482371612	0.288612255	1.671348333	0.094652895	0.921147998	ENSG00000116001	TIA1
391.0376534	0.030595172	0.398454699	0.076784569	0.938794927	0.999527751	ENSG00000132329	RAMP1
398.2882452	-0.217233764	0.224181397	-0.96900888	0.332540759	0.976026345	ENSG00000227345	PARG
394.5659164	0.623047383	0.32205109	1.934622806	0.053036599	0.847598083	ENSG00000080819	CPOX
395.7303701	-0.130405454	0.255124965	-0.511143447	0.609250615	0.99483168	ENSG00000138660	AP1AR
393.4432669	-0.101616082	0.209850639	-0.484230509	0.628222287	0.99483168	ENSG00000204371	EHMT2
390.8530931	0.00941408	0.236545351	0.039798204	0.968254008	0.999527751	ENSG00000177225	GATD1
394.6979877	0.031699631	0.216692764	0.146288368	0.883693748	0.999467288	ENSG00000183751	TBL3
394.8478559	-0.165420942	0.246058002	-0.672284343	0.501402695	0.993513219	ENSG00000150776	NKAPD1
398.6233723	0.256950172	0.290192837	0.885446293	0.375915974	0.982609538	ENSG00000140598	EFL1
394.6122275	0.105033595	0.263518831	0.398580985	0.690201974	0.998869317	ENSG00000197296	FITM2
412.9210447	-0.460217886	1.010039272	-0.455643557	0.648646316	0.997368416	ENSG00000122877	EGR2
389.5247518	-0.446133797	0.300750385	-1.483402247	0.137967571	0.934144427	ENSG00000130312	MRPL34
392.5219448	0.020040906	0.242615168	0.082603682	0.934166673	0.999527751	ENSG00000105185	PDCD5
394.0063747	0.244926932	0.288930592	0.847701626	0.396604161	0.988378925	ENSG00000145687	SSBP2
397.6467892	0.26378844	0.284144382	0.928360568	0.353220566	0.977691382	ENSG00000184634	MED12
365.8274594	1.490741165	1.721744269	0.865831931	0.386582336	0.986711841	ENSG00000129521	EGLN3
392.9751282	-0.093453778	0.251013613	-0.372305615	0.709665318	0.99900742	ENSG00000132467	UTP3
390.2248725	0.084711086	0.266549497	0.317806214	0.750631937	0.999467288	ENSG00000106009	BRAT1
389.5501355	0.073593803	0.324546467	0.22675891	0.820611217	0.999467288	ENSG00000166997	CNPY4
390.8444207	-0.332355246	0.298399686	-1.113792209	0.26536834	0.962465655	ENSG00000095906	NUBP2
393.4442646	0.094052262	0.225748654	0.416623802	0.676953597	0.998869317	ENSG00000104671	DCTN6
391.3024376	0.091535723	0.286794444	0.319168396	0.749598821	0.999467288	ENSG00000107949	BCCIP
389.5584538	0.172369808	0.365123727	0.472086021	0.63686539	0.99653524	ENSG00000136732	GYPC
389.4061926	0.146617901	0.273231186	0.53660749	0.591538769	0.99483168	ENSG00000174032	SLC25A30
390.3683784	0.189254861	0.305687088	0.619113036	0.535841896	0.99483168	ENSG00000185262	UBALD2
390.7977654	-0.253350053	0.217934862	-1.162503561	0.245030979	0.962465655	ENSG00000177370	TIMM22
392.9307037	0.144110403	0.247998748	0.581093269	0.561177595	0.99483168	ENSG00000204977	TRIM13
393.4492498	0.088393875	0.218755342	0.404076418	0.686156526	0.998869317	ENSG00000144840	RABL3
400.039314	-0.132640037	0.463274534	-0.286309795	0.774640857	0.999467288	ENSG00000133794	ARNTL
389.4438665	-0.469198965	0.273595483	-1.714936815	0.086356812	0.901307093	ENSG00000161677	JOSD2
394.0795185	-0.254648199	0.26914738	-0.946129216	0.344082684	0.977691382	ENSG00000159873	CCDC117
390.3010643	-0.159940086	0.294001613	-0.544010915	0.586433962	0.99483168	ENSG00000111727	HCFC2

389.0741081	-0.402618392	0.291133068	-1.382935971	0.166684499	0.943223078	ENSG00000224877	NDUFAF8
391.861928	0.242155224	0.254862834	0.950139413	0.342041419	0.977691382	ENSG00000100425	BRD1
392.265579	0.242760313	0.227281288	1.068105145	0.285473085	0.96478822	ENSG00000198399	ITSN2
388.8627241	0.347199751	0.250140666	1.388018016	0.165131559	0.943223078	ENSG00000009413	REV3L
379.3915948	-0.490740226	0.523905636	-0.936695833	0.34891504	0.977691382	ENSG00000072952	MRVI1
392.5910272	-0.225834545	0.221289559	-1.020538634	0.307473076	0.973427483	ENSG00000160803	UBQLN4
392.4727641	-0.017898965	0.317860824	-0.056310698	0.955094297	0.999527751	ENSG00000159164	SV2A
392.6635997	0.03370105	0.250845087	0.134350049	0.893125779	0.999467288	ENSG00000183684	ALYREF
390.061563	0.216632297	0.221827362	0.976580595	0.32877683	0.976026345	ENSG00000114021	NIT2
393.0367179	-0.261589752	0.327273293	-0.799300639	0.424116109	0.991301167	ENSG00000060069	CTDP1
391.2010356	-0.042888687	0.278535738	-0.153979117	0.877626202	0.999467288	ENSG00000084676	NCOA1
390.9921441	0.064733769	0.226356081	0.285982019	0.774891893	0.999467288	ENSG00000197498	RPF2
389.1297727	0.169690781	0.262827429	0.645635739	0.518515286	0.993513219	ENSG00000100154	TTC28
391.5312352	0.209788908	0.299331142	0.700858943	0.483391049	0.991892545	ENSG00000066933	MYO9A
391.3322444	0.004886202	0.228111683	0.021420219	0.982910445	0.999527751	ENSG00000163964	PIGX
395.3759063	0.015076602	0.343741943	0.043860233	0.965015815	0.999527751	ENSG00000177150	FAM210A
393.4171709	-0.038268231	0.252405924	-0.151613837	0.879491519	0.999467288	ENSG00000113300	CNOT6
388.2578798	0.672887331	0.407582584	1.650922677	0.098754365	0.921147998	ENSG00000112118	MCM3
391.8945519	0.065595468	0.310415958	0.211314742	0.83264168	0.999467288	ENSG00000182372	CLN8
389.9133582	0.079960527	0.233337814	0.342681392	0.731838163	0.999467288	ENSG00000108179	PPIF
389.6212244	-0.10596353	0.248962283	-0.425620815	0.670384171	0.997568624	ENSG00000160563	MED27
390.4935174	-0.08625034	0.251168359	-0.343396519	0.731300178	0.999467288	ENSG00000186283	TOR3A
391.8628541	-0.414418781	0.285518248	-1.451461628	0.14665136	0.936396974	ENSG00000110492	MDK
390.3854487	-0.189913238	0.217507841	-0.873132836	0.382590678	0.98555814	ENSG00000066136	NFYC
397.3811737	-0.48831681	0.521204789	-0.936900083	0.348809956	0.977691382	ENSG00000144655	CSRNP1
385.273117	-0.382464916	0.250315788	-1.52792966	0.126530003	0.928627271	ENSG00000180992	MRPL14
395.34288	-0.187853537	0.326710324	-0.574985003	0.56530144	0.99483168	ENSG00000170677	SOCS6
386.5793463	-0.014252831	0.315239533	-0.045212702	0.96393777	0.999527751	ENSG00000197785	ATAD3A
391.5321485	0.269721087	0.231220487	1.166510333	0.243408173	0.962465655	ENSG00000165660	ABRAXAS2
395.9764728	0.549278139	0.366418425	1.499046174	0.133861654	0.933741583	ENSG00000117000	RLF
388.0298284	0.063886751	0.223206249	0.28622295	0.774707367	0.999467288	ENSG00000147419	CCDC25
383.7427685	-0.647306179	0.368778092	-1.75527287	0.079212645	0.897052166	ENSG00000166444	ST5
390.2535149	-0.209036701	0.218636534	-0.956092274	0.339025618	0.977691382	ENSG00000102870	ZNF629
388.7626414	-0.098560574	0.235940836	-0.417734275	0.676141409	0.998869317	ENSG00000152778	IFIT5
387.3916316	-0.143438229	0.222982241	-0.643271988	0.520047629	0.993513219	ENSG00000057935	MTA3
388.7459714	-0.225868839	0.300844465	-0.750782765	0.452783404	0.99135833	ENSG00000167107	ACSF2
383.1998459	0.14221221	0.299408381	0.474977386	0.634803092	0.995506752	ENSG00000106266	SNX8
387.2970027	0.570622726	0.250449362	2.278395603	0.022703018	0.744905651	ENSG00000133121	STARD13
391.0372158	-0.208687531	0.269603916	-0.7740523	0.438899864	0.991301167	ENSG00000125812	GZF1
388.0316042	0.08620808	0.305781741	0.281926841	0.777999608	0.999467288	ENSG00000068724	TTC7A
388.9763223	0.263453954	0.231306236	1.13898336	0.254710094	0.962465655	ENSG00000124275	MTRR
387.7398119	0.037241914	0.266677905	0.139651291	0.888935513	0.999467288	ENSG00000068796	KIF2A
389.4363015	-0.011391971	0.221852225	-0.051349366	0.959047132	0.999527751	ENSG00000152942	RAD17
385.8604084	0.20222402	0.341379993	0.592372206	0.553601374	0.99483168	ENSG00000277053	GTF2IP1
385.5617525	-0.029327241	0.295593016	-0.099214933	0.920967619	0.999527751	ENSG00000119333	WDR34
383.9920669	0.013902327	0.465515479	0.02986437	0.976175222	0.999527751	ENSG00000139971	ARMH4
388.8474733	0.205358134	0.229077819	0.896455778	0.370009389	0.981455968	ENSG00000116750	UCHL5
381.7198885	-0.256698956	0.342292605	-0.749940115	0.453290773	0.99135833	ENSG00000103024	NME3
389.3974057	0.019349931	0.264637174	0.073118717	0.941711647	0.999527751	ENSG00000180867	PDIA3P1
385.3906597	0.143262971	0.378502282	0.378499624	0.705059473	0.99900742	ENSG00000239306	RBM14
386.3411278	0.021481913	0.235147349	0.091355114	0.927210427	0.999527751	ENSG00000112367	FIG4
374.8134115	-1.093800123	0.876063521	-1.248539743	0.211833464	0.954310328	ENSG00000126709	IFI6
387.1463082	-0.009592903	0.246961447	-0.038843728	0.969014981	0.999527751	ENSG00000150712	MTMR12
386.8918744	0.191453154	0.272248945	0.703228268	0.481913506	0.991525194	ENSG00000139291	TMEM19
384.4017263	-0.218632402	0.236400807	-0.924837796	0.355050287	0.977691382	ENSG00000133265	HSPBP1
387.0374316	0.398002958	0.215131721	1.850043112	0.064307336	0.868202292	ENSG00000135372	NAT10
384.0435219	0.098208389	0.309999809	0.31680145	0.751394264	0.999467288	ENSG00000067221	STOML1
385.2837293	0.043991603	0.25310521	0.173807577	0.862016689	0.999467288	ENSG00000248333	CDK11B
382.8709934	-0.378507611	0.326204253	-1.160339289	0.245910694	0.962465655	ENSG00000168734	PKIG
385.3776824	0.119280396	0.243173493	0.490515616	0.623769082	0.99483168	ENSG00000117748	RPA2
387.706295	0.403091301	0.235810172	1.709388943	0.087378928	0.905318403	ENSG00000120925	RNF170
385.7730601	-0.256573536	0.280105084	-0.915990285	0.359671991	0.979465649	ENSG00000164897	TMUB1
389.6095418	-0.066794994	0.338269771	-0.197460723	0.843467015	0.999467288	ENSG00000176994	SMCR8
392.5251246	0.207526985	0.311455139	0.666314212	0.505210283	0.993513219	ENSG00000104611	SH2D4A

390.2076258	-0.226170446	0.27383661	-0.825932097	0.408842612	0.990226751	ENSG00000187866	FAM122A
384.7605892	0.349862385	0.296742499	1.179010039	0.238394179	0.962465655	ENSG00000123179	EBPL
388.2812453	-0.298069615	0.254666253	-1.170432327	0.241827033	0.962465655	ENSG00000198055	GRK6
382.5370343	0.460593092	0.338159758	1.362057669	0.173179686	0.944422693	ENSG00000185483	ROR1
389.8491993	0.00453827	0.279546169	0.016234419	0.987047377	0.999527751	ENSG00000118007	STAG1
386.5495646	-0.143468012	0.251051601	-0.571468222	0.567682295	0.99483168	ENSG00000179151	EDC3
388.2968285	0.718393445	0.543876631	1.320875736	0.186542801	0.949094286	ENSG00000113810	SMC4
390.0744905	0.086086013	0.307836388	0.279648593	0.779747122	0.999467288	ENSG00000172888	ZNF621
387.947439	-0.801896639	0.376273349	-2.131154492	0.033076417	0.775963115	ENSG00000120833	SOCS2
383.2235588	0.444527798	0.302441449	1.46979787	0.141616506	0.935644597	ENSG00000106868	SUSD1
384.619943	0.059834822	0.278721696	0.214675867	0.830020039	0.999467288	ENSG00000171467	ZNF318
383.4872915	-0.33236276	0.245897028	-1.351633908	0.176492457	0.946278819	ENSG00000168884	TNIP2
386.421419	0.016647852	0.242559488	0.068634099	0.945280876	0.999527751	ENSG00000138750	NUP54
396.5849004	-0.72880574	0.502740131	-1.449666924	0.147151424	0.936396974	ENSG00000170873	MTSS1
383.2118697	-0.06565625	0.23577946	-0.278464671	0.780655681	0.999467288	ENSG00000115946	PNO1
386.3021393	-0.041442114	0.266851499	-0.155300285	0.876584591	0.999467288	ENSG00000153006	SREK1IP1
389.2490952	-0.327677712	0.353244027	-0.927624212	0.353602534	0.977691382	ENSG00000171914	TLN2
383.981571	-0.237348176	0.216938659	-1.094079667	0.273920039	0.962465655	ENSG00000149179	C11orf49
389.2932411	-0.09473657	0.349388747	-0.271149458	0.786276084	0.999467288	ENSG00000061938	TNK2
384.4125663	-0.33912595	0.249915024	-1.356965037	0.17479232	0.946176003	ENSG00000263266	RPS7P1
385.925528	-0.014620109	0.253938415	-0.057573443	0.954088404	0.999527751	ENSG00000152242	C18orf25
383.5242319	-0.052125315	0.214854813	-0.242607154	0.808309738	0.999467288	ENSG00000100034	PPM1F
383.6644397	0.071495386	0.274003431	0.260928799	0.794147415	0.999467288	ENSG00000128908	INO80
382.2242325	0.179108829	0.228281013	0.784598012	0.432689296	0.991301167	ENSG00000109320	NFKB1
382.856719	0.304381531	0.259619435	1.172414273	0.241030772	0.962465655	ENSG00000196455	PIK3R4
380.1367289	0.08481287	0.272732339	0.310974748	0.755819819	0.999467288	ENSG00000114738	MAPKAPK3
380.5405213	-0.654401789	0.25650719	-2.551202516	0.010735193	0.638240244	ENSG00000173153	ESRRA
381.5450525	-0.275719565	0.236860266	-1.164060012	0.244399692	0.962465655	ENSG00000124380	SNRNP27
382.5517001	0.233187393	0.243285775	0.95849169	0.33781488	0.977691382	ENSG00000130962	PRRG1
378.9874053	-0.237607796	0.28881189	-0.822707805	0.410674176	0.990226751	ENSG00000220205	VAMP2
381.2858662	0.065149641	0.232147804	0.280638628	0.778987585	0.999467288	ENSG00000135622	SEMA4F
382.0751997	-0.160300289	0.300994227	-0.532569315	0.594331748	0.99483168	ENSG00000138442	WDR12
382.4108338	0.28312007	0.235891344	1.200213904	0.230056277	0.959575179	ENSG00000108559	NUP88
379.6935205	-0.522366026	0.285078682	-1.83235738	0.066898193	0.87420149	ENSG00000104915	STX10
382.9417624	-0.041288006	0.230234675	-0.179330096	0.857678517	0.999467288	ENSG00000112290	WASF1
383.6899471	-0.114332358	0.311929804	-0.366532332	0.713967891	0.99900742	ENSG00000137040	RANBP6
382.0598573	-0.052631461	0.296602065	-0.177448061	0.859156459	0.999467288	ENSG00000186951	PPARA
379.6576859	0.014731564	0.254649482	0.057850359	0.953867824	0.999527751	ENSG00000177646	ACAD9
386.2603552	-0.008914015	0.34395356	-0.025916332	0.979324073	0.999527751	ENSG00000085511	MAP3K4
381.7272884	-0.314637104	0.265720029	-1.184092538	0.236376434	0.962465655	ENSG00000114446	IFT57
377.2330114	-0.055039945	0.30602918	-0.179851952	0.857268797	0.999467288	ENSG00000095380	NANS
382.522803	0.241540857	0.213164478	1.133119641	0.257164014	0.962465655	ENSG00000117620	SLC35A3
385.9162461	-0.204787059	0.337298904	-0.607138229	0.543759182	0.99483168	ENSG00000136052	SLC41A2
384.5410705	0.681827838	0.995501141	0.684909148	0.493401253	0.993513219	ENSG00000185432	METTL7A
385.0614147	0.121904384	0.286363408	0.425698187	0.670327783	0.997568624	ENSG00000107077	KDM4C
381.6343185	-0.379031966	0.246566795	-1.537238485	0.124234919	0.927794614	ENSG00000136940	PDCL
380.4044001	0.040480622	0.246029228	0.164535826	0.869309346	0.999467288	ENSG00000145348	TBCK
380.2009701	0.006855412	0.266150091	0.025757693	0.979450606	0.999527751	ENSG00000112787	FBRSL1
381.3301	-0.146856072	0.254884937	-0.576166147	0.564502885	0.99483168	ENSG00000048471	SNX29
379.0789571	0.158688824	0.235962547	0.672516997	0.501254623	0.993513219	ENSG00000072609	CHFR
381.4201454	-0.06035309	0.285326465	-0.211522929	0.832479242	0.999467288	ENSG00000114126	TFDP2
382.0077913	0.09298675	0.242227832	0.383881361	0.701066382	0.998869317	ENSG00000177888	ZBTB41
379.0541787	-0.018704663	0.255914996	-0.073089358	0.94173501	0.999527751	ENSG00000143147	GPR161
375.1878065	-0.175921778	0.28988063	-0.606876623	0.543932793	0.99483168	ENSG00000133083	DCLK1
375.5134558	0.907325608	0.31251536	2.903299242	0.003692536	0.440754558	ENSG00000176692	FOXC2
378.4554928	-0.009504928	0.248561941	-0.038239674	0.969496589	0.999527751	ENSG00000090006	LTBP4
377.8814496	0.010477833	0.276075912	0.037952726	0.969725374	0.999527751	ENSG00000071205	ARHGAP10
384.5243407	-0.275093418	0.31004229	-0.887277081	0.374929742	0.982609538	ENSG00000198315	ZKSCAN8
382.8592669	0.273931988	0.26578236	1.030662787	0.302698982	0.972793733	ENSG00000198887	SMC5
379.4648166	0.018664112	0.224426997	0.0831634	0.933721615	0.999527751	ENSG00000167378	IRGQ
381.0470221	-0.071720078	0.255927431	-0.280235992	0.779296455	0.999467288	ENSG00000085978	ATG16L1
376.9080087	-0.085847533	0.290949403	-0.295060009	0.767948039	0.999467288	ENSG00000109445	ZNF330
377.3740275	0.395478114	0.299308757	1.321304855	0.186399736	0.949094286	ENSG00000055044	NOP58
379.4646455	0.441717701	0.277871191	1.589649146	0.111913913	0.923922314	ENSG00000164535	DAGLB

371.5945622	-0.236952863	0.379761255	-0.62395218	0.532658991	0.99483168	ENSG00000166900	STX3
376.5110625	-0.045076863	0.265304836	-0.169905921	0.865084125	0.999467288	ENSG00000169019	COMMD8
384.1125878	0.33486897	0.276041732	1.213109944	0.225087788	0.958865115	ENSG00000124782	RREB1
377.7161789	-0.10501413	0.304487001	-0.344888714	0.730178036	0.999467288	ENSG00000134574	DDB2
378.5625409	0.102747687	0.250024654	0.410950222	0.681109035	0.998869317	ENSG00000151092	NGLY1
381.3370665	0.215740092	0.244762338	0.88142683	0.378086841	0.982609538	ENSG00000175105	ZNF654
378.2524307	0.240059219	0.218779597	1.097265115	0.272525516	0.962465655	ENSG00000147874	HAUS6
380.9295648	0.185126047	0.241533617	0.76646079	0.443402174	0.99135833	ENSG00000083223	TUT7
377.9359404	-0.537446179	0.323126807	-1.663267076	0.096258986	0.921147998	ENSG00000224051	CPTP
372.0897142	-0.383595384	0.351447869	-1.091471643	0.275065403	0.962465655	ENSG00000204264	PSMB8
373.4881057	-0.100880583	0.250697084	-0.402400305	0.687389445	0.998869317	ENSG00000175634	RPS6KB2
380.615602	-0.165435362	0.319443762	-0.517885717	0.604538008	0.99483168	ENSG00000165661	QSOX2
379.8566181	0.32617469	0.280708352	1.161970023	0.245247642	0.962465655	ENSG00000210196	MT-TP
380.350943	-0.031422164	0.30919962	-0.1016242	0.91905497	0.999527751	ENSG00000168758	SEMA4C
378.9548952	-0.043291833	0.277734587	-0.155874837	0.87613168	0.999467288	ENSG00000112996	MRPS30
383.8039646	-0.452645975	0.439698899	-1.029445324	0.30327046	0.972793733	ENSG00000178860	MSC
380.9420278	0.179230206	0.262253308	0.683424005	0.494338956	0.993513219	ENSG00000096717	SIRT1
368.7748724	0.003697778	0.385529048	0.009591439	0.992347256	0.999527751	ENSG00000128274	A4GALT
378.86503	-0.203466667	0.239518947	-0.849480469	0.395613994	0.988378925	ENSG00000116497	S100PBP
378.6135029	-0.155418015	0.2402817	-0.646815861	0.517751126	0.993513219	ENSG00000180354	MTURN
379.082019	0.05524716	0.223381781	0.24732169	0.804659274	0.999467288	ENSG00000101773	RBBP8
378.908029	0.346182844	0.264407794	1.309276247	0.190440793	0.949094286	ENSG00000134987	WDR36
383.3971742	-0.452427115	0.35922885	-1.25943981	0.207871518	0.954310328	ENSG00000235531	MSC-AS1
372.4585334	0.036232542	0.287931647	0.125837303	0.899860713	0.999527751	ENSG00000182195	LDOC1
372.2042167	-0.235466641	0.302476444	-0.778462738	0.436296265	0.991301167	ENSG00000122033	MTIF3
376.7258681	0.185880799	0.222702182	0.834660879	0.403908674	0.988927059	ENSG00000021574	SPAST
376.1703716	-0.086950746	0.236788671	-0.36720822	0.713463708	0.99900742	ENSG00000100226	GTPBP1
379.5234195	0.039698138	0.255254535	0.155523732	0.876408446	0.999467288	ENSG00000180917	CMTR2
373.8460574	-0.235306544	0.25003692	-0.941087196	0.346660177	0.977691382	ENSG00000060971	ACAA1
374.0402643	0.30647435	0.285170537	1.074705519	0.282506592	0.963267117	ENSG00000145191	EIF2B5
376.6753774	0.417752138	0.288244901	1.449295851	0.147254979	0.936396974	ENSG00000186001	LRCH3
375.2742439	-0.107274603	0.239311641	-0.448263204	0.653963253	0.997527562	ENSG00000078043	PIAS2
373.782621	0.059633128	0.232564805	0.256415099	0.79763033	0.999467288	ENSG00000066027	PPP2R5A
369.1428995	0.142498142	0.367408074	0.387847063	0.698129215	0.998869317	ENSG00000043143	JADE2
371.6728501	-0.301672079	0.251241767	-1.200724236	0.229858189	0.959575179	ENSG00000126705	AHDC1
373.3063115	0.190283456	0.303546909	0.626866723	0.530746602	0.99483168	ENSG00000156709	AIFM1
375.4955807	-0.021103157	0.226390027	-0.093215928	0.925732021	0.999527751	ENSG00000101413	RPRD1B
374.1687454	0.119064608	0.291440147	0.408538799	0.682878152	0.998869317	ENSG00000087253	LPCAT2
371.533199	-1.199912284	0.299044031	-4.012493678	6.01E-05	0.054234087	ENSG00000171621	SPSB1
368.3585908	-0.109905844	0.368344521	-0.298377845	0.765414797	0.999467288	ENSG00000143314	MRPL24
376.8392391	0.3794581	0.254207022	1.492712898	0.135512356	0.934144427	ENSG00000047188	YTHDC2
373.7384235	0.464634031	0.226519325	2.051189365	0.040248509	0.795004104	ENSG00000122417	ODF2L
374.9982855	0.181496408	0.290729274	0.624279781	0.53244386	0.99483168	ENSG00000137073	UBAP2
373.4045222	0.145467378	0.254793198	0.570923317	0.568051625	0.99483168	ENSG00000157107	FCHO2
371.0872214	-0.081661353	0.255203349	-0.319985427	0.748979378	0.999467288	ENSG00000114054	PCCB
373.474479	0.207708194	0.266159493	0.780389954	0.435161379	0.991301167	ENSG00000134324	LPIN1
377.3159564	-0.057032158	0.252901295	-0.225511532	0.821581355	0.999467288	ENSG00000140950	TLDC1
369.2759203	0.042882569	0.332905972	0.128812857	0.897505732	0.999467288	ENSG00000137266	SLC22A23
366.6862147	0.301616744	0.318551323	0.946838774	0.343720941	0.977691382	ENSG00000065809	FAM107B
372.7833407	0.178655374	0.242248877	0.737486901	0.460826306	0.99135833	ENSG00000072401	UBE2D1
371.2898871	0.217305697	0.222492602	0.97668729	0.328723989	0.976026345	ENSG00000050426	LETMD1
378.4590158	0.472319888	0.355018403	1.330409591	0.183383356	0.949094286	ENSG00000136824	SMC2
381.4563491	-0.207597804	0.393320893	-0.527807719	0.597632799	0.99483168	ENSG00000198018	ENTPD7
371.0081742	0.332152064	0.246339091	1.348353048	0.177544858	0.946844293	ENSG00000109680	TBC1D19
371.2148077	-0.111722508	0.225786644	-0.49481451	0.620731061	0.99483168	ENSG00000204568	MRPS18B
359.6096149	0.422960297	0.591692506	0.714831256	0.474713319	0.99135833	ENSG00000180914	OXTR
368.3621105	-0.653684101	0.291089121	-2.245649371	0.024726476	0.749756093	ENSG00000063241	ISOC2
373.8896415	0.376090085	0.272288605	1.381218596	0.167211759	0.943223078	ENSG00000163900	TMEM41A
373.5943405	0.068724271	0.251121349	0.273669568	0.784338567	0.999467288	ENSG00000123178	SPRYD7
373.9016017	-0.042192186	0.23552097	-0.179144075	0.857824575	0.999467288	ENSG00000053372	MRTO4
372.3747128	-0.121202944	0.263856547	-0.459351665	0.645981652	0.997368416	ENSG00000070761	CFAP20
369.1923832	-0.255504814	0.254955873	-1.002153084	0.316269663	0.974768742	ENSG00000186395	KRT10
377.7504839	0.235275959	0.309855324	0.759309074	0.447667691	0.99135833	ENSG00000150527	MIA2
371.4395622	-0.001715421	0.241996196	-0.007088629	0.99434414	0.999527751	ENSG00000103319	EEF2K

366.8580396	-0.041102341	1.122328945	-0.036622365	0.970786111	0.999527751	ENSG00000152463	OLAH
368.9303759	0.155819919	0.491010322	0.317345505	0.750981452	0.999467288	ENSG00000120820	GLT8D2
372.61567	-0.223460014	0.269840515	-0.828118839	0.407603202	0.990226751	ENSG00000234797	RPS3AP6
370.2559621	-0.151850353	0.262863224	-0.577678195	0.563481404	0.99483168	ENSG00000140983	RHOT2
368.8235378	-0.209984384	0.33012179	-0.636081563	0.524723262	0.99483168	ENSG00000108960	MMD
366.735725	-0.385399755	0.319485126	-1.206315174	0.227695989	0.958865115	ENSG00000104522	TSTA3
372.7712939	-0.285285012	0.245968228	-1.159844967	0.246111932	0.962465655	ENSG00000112319	EYA4
370.6539801	0.386628857	0.409594418	0.943930971	0.345204924	0.977691382	ENSG00000110328	GALNT18
367.1631924	0.21503889	0.28868337	0.744895315	0.456335007	0.99135833	ENSG00000151366	NDUFC2
369.7350555	0.164902452	0.255777311	0.644711022	0.519114472	0.993513219	ENSG00000134444	RELCH
369.6732669	0.260154947	0.230353705	1.12937166	0.258741083	0.962465655	ENSG00000181751	C5orf30
373.7504346	-0.049456678	0.404875797	-0.122152715	0.902778074	0.999527751	ENSG00000205609	EIF3CL
371.0780111	0.298308583	0.283563975	1.051997464	0.292800713	0.970706863	ENSG00000064651	SLC12A2
370.7604468	0.478345477	0.42023766	1.138273703	0.255006211	0.962465655	ENSG00000123136	DDX39A
370.9716377	-0.170826034	0.235447158	-0.725538736	0.46812159	0.99135833	ENSG00000120029	ARMH3
367.5167306	0.072259658	0.249629425	0.28946771	0.772223486	0.999467288	ENSG00000135124	P2RX4
371.5834079	0.080560156	0.278922169	0.288826653	0.772714035	0.999467288	ENSG00000091157	WDR7
373.5065148	-0.081604561	0.357732849	-0.228115927	0.819556118	0.999467288	ENSG00000148840	PPRC1
368.4539607	-0.032031919	0.250635462	-0.127802821	0.898305017	0.999490224	ENSG00000113812	ACTR8
368.9742743	0.736196159	0.233636034	3.151038586	0.00162691	0.290092105	ENSG00000171492	LRRC8D
373.0009492	0.040601252	0.297342712	0.136546989	0.89138888	0.999467288	ENSG00000168944	CEP120
368.6703168	0.304826592	0.247676626	1.230744288	0.218418518	0.956884001	ENSG00000181827	RFX7
366.7190244	0.088811807	0.292701446	0.303421142	0.761568936	0.999467288	ENSG00000167088	SNRPD1
373.2557944	0.402260119	0.295664418	1.360529353	0.173662471	0.944422693	ENSG00000058804	NDC1
366.9153721	-0.402376796	0.293657308	-1.370225719	0.170616452	0.944422693	ENSG00000154237	LRRK1
364.1894523	-0.279811926	0.251044628	-1.114590376	0.265025996	0.962465655	ENSG00000183978	COA3
368.4985087	-0.185213704	0.232683908	-0.795988452	0.426038748	0.991301167	ENSG00000100083	GGA1
371.4447308	-0.414177817	0.355900311	-1.163746711	0.244526673	0.962465655	ENSG00000170390	DCLK2
366.2131667	0.362055659	0.269229723	1.344783386	0.178695197	0.9469453	ENSG00000204138	PHACTR4
367.2825878	-0.256320829	0.227593183	-1.126223668	0.260070858	0.962465655	ENSG00000131148	EMC8
370.2969966	0.482194635	0.359134061	1.342659156	0.179382366	0.9469453	ENSG00000126368	NR1D1
369.2835205	-0.155520793	0.269166875	-0.577785782	0.563408756	0.99483168	ENSG00000144036	EXOC6B
366.2037382	-0.070833247	0.290164568	-0.244114049	0.807142492	0.999467288	ENSG00000015153	YAF2
368.2806478	0.676492619	0.255729018	2.645349453	0.008160659	0.592283416	ENSG00000221978	CCNL2
372.2913002	0.417198608	0.34304559	1.216160826	0.223923663	0.956884001	ENSG00000088448	ANKRD10
365.0920253	-0.087218628	0.247057992	-0.353028967	0.724066724	0.999456245	ENSG00000158092	NCK1
366.0225444	-0.156386861	0.278763441	-0.561002047	0.574796141	0.99483168	ENSG00000168286	THAP11
368.5213226	0.144320473	0.295925751	0.487691499	0.625768374	0.99483168	ENSG00000119787	ATL2
367.2697958	0.289173841	0.302071094	0.957303916	0.33841388	0.977691382	ENSG00000126653	NSRP1
367.4388545	0.199406595	0.246181973	0.809996741	0.417942049	0.990226751	ENSG00000064545	TMEM161A
364.8263655	-0.261441889	0.258357821	-1.011937199	0.311568082	0.973427483	ENSG00000156253	RWDD2B
368.1085695	0.658047643	0.31091092	2.116515059	0.034301026	0.782919999	ENSG00000157214	STEAP2
364.5331955	-0.032764588	0.230594259	-0.142087612	0.887010803	0.999467288	ENSG00000132792	CTNNBL1
366.529425	-0.080311527	0.284055789	-0.282731526	0.777382647	0.999467288	ENSG00000114648	KLHL18
365.6193354	0.303843595	0.245945496	1.235410284	0.216677847	0.954900762	ENSG00000163389	POGLUT1
364.9615819	-0.128706707	0.221383628	-0.581374096	0.560988352	0.99483168	ENSG00000154124	OTULIN
365.4149117	0.112375144	0.241696777	0.464942665	0.641972516	0.996723676	ENSG00000066557	LRRC40
360.8857616	-0.432274106	0.337803116	-1.279662872	0.200663728	0.954310328	ENSG00000166780	C16orf45
361.3090711	-0.034672736	0.256194609	-0.135337492	0.892345044	0.999467288	ENSG00000170310	STX8
366.9128778	-0.087924865	0.247345406	-0.355474018	0.722234506	0.99906455	ENSG00000186141	POLR3C
364.5789173	-0.326476373	0.298933753	-1.092136201	0.27477324	0.962465655	ENSG00000164088	PPM1M
359.59689	0.140699694	0.294522281	0.477721731	0.632848275	0.994909189	ENSG00000105552	BCAT2
362.6337163	-0.040583329	0.297738119	-0.136305452	0.891579814	0.999467288	ENSG00000241837	ATP5PO
366.479694	0.160419448	0.241029061	0.665560609	0.505691992	0.993513219	ENSG00000153107	ANAPC1
361.0682397	0.117167044	0.266331902	0.439928688	0.659988757	0.997568624	ENSG00000081377	CDC14B
378.1122843	-0.411295285	0.436598415	-0.942044842	0.346169682	0.977691382	ENSG00000106003	LFNG
363.9871851	0.126927095	0.237746598	0.533875547	0.593427641	0.99483168	ENSG00000163882	POLR2H
366.0935354	0.102790903	0.370439705	0.277483492	0.78140888	0.999467288	ENSG00000198805	PNP
364.6649037	-0.256479574	0.23312164	-1.100196336	0.271246587	0.962465655	ENSG00000115207	GTF3C2
363.9313953	0.449582188	0.253480504	1.77363616	0.076123352	0.897052166	ENSG00000112983	BRD8
364.0101896	-0.408236107	0.24663934	-1.655194611	0.097885028	0.921147998	ENSG00000224597	SVIL-AS1
364.7756105	0.117811486	0.280817023	0.419531141	0.674828001	0.998869317	ENSG00000137817	PARP6
368.4894263	-0.271260996	0.277288796	-0.978261653	0.327944931	0.976026345	ENSG00000175395	ZNF25
365.002065	-0.025791434	0.245182546	-0.105192781	0.916222839	0.999527751	ENSG00000130935	NOL11

360.558573	-0.490358549	0.2605842	-1.881766234	0.059867762	0.858996918	ENSG00000127838	PNKD
361.5597746	-0.001648819	0.226953732	-0.007265002	0.994203418	0.999527751	ENSG00000172932	ANKRD13D
364.9910329	-0.261982768	0.239379412	-1.09442481	0.273768708	0.962465655	ENSG00000161999	JMJD8
361.4228039	0.305013313	0.304858729	1.000507067	0.317065179	0.974768742	ENSG00000091656	ZFHX4
364.2045724	0.350290205	0.254142552	1.378321744	0.168103973	0.943223078	ENSG00000165240	ATP7A
361.6416538	-0.120085966	0.326869606	-0.367381868	0.713334195	0.99900742	ENSG00000132970	WASF3
364.3714247	3.189943223	1.604611635	1.987984602	0.046813393	0.823539385	ENSG00000148773	MKI67
363.8036637	-0.071446217	0.256501601	-0.278541018	0.780597082	0.999467288	ENSG00000106305	AIMP2
362.1222091	-0.118520956	0.248517016	-0.476912842	0.633424187	0.995376704	ENSG00000072518	MARK2
364.0356132	-0.153301201	0.294348855	-0.520814668	0.602495884	0.99483168	ENSG00000155729	KCTD18
367.5554312	-0.534027085	0.299181058	-1.784962885	0.074267337	0.89238199	ENSG00000127334	DYRK2
361.9033393	-0.509551625	0.25498352	-1.998370818	0.045676473	0.814450986	ENSG00000215030	RPL13P12
359.9313754	-0.036532807	0.290266048	-0.125859731	0.899842959	0.999527751	ENSG00000185189	NRBP2
360.4299865	0.20986853	0.244813323	0.857259432	0.391301523	0.988355932	ENSG00000121289	CEP89
360.1216987	0.470987004	0.307732397	1.53050835	0.125890948	0.928627271	ENSG00000080493	SLC4A4
363.8478574	0.21355366	0.288928374	0.739123186	0.459832199	0.99135833	ENSG00000085433	WDR47
364.8861018	0.052922395	0.258499835	0.204728932	0.837783915	0.999467288	ENSG00000136152	COG3
359.3073685	0.2299007	0.320876419	0.716477391	0.473696623	0.99135833	ENSG00000116675	DNAJC6
360.375199	0.100525766	0.321260503	0.312910443	0.754348703	0.999467288	ENSG00000148341	SH3GLB2
359.649114	-0.488228824	0.301719512	-1.618154626	0.105629279	0.92169898	ENSG00000133424	LARGE1
361.9050942	0.078859853	0.253063014	0.31162141	0.755328261	0.999467288	ENSG00000122008	POLK
361.2637679	-0.213596667	0.235322811	-0.90767515	0.364049877	0.98048242	ENSG00000115761	NOL10
360.4583438	-0.472372842	0.362506963	-1.303072465	0.192550023	0.949261383	ENSG00000172663	TMEM134
362.6454105	-0.011915707	0.270698989	-0.044018293	0.964889823	0.999527751	ENSG00000105879	CBLL1
360.1259036	-0.31315246	0.21867405	-1.432051312	0.152129137	0.938052926	ENSG00000133059	DSTYK
355.6469908	-0.73873794	0.299976338	-2.462654037	0.013791292	0.672931175	ENSG00000141741	MIEN1
360.1902356	0.047740895	0.215951451	0.221072352	0.825036098	0.999467288	ENSG00000033800	PIAS1
361.5330346	-0.09102713	0.23879752	-0.381189595	0.703062569	0.99900742	ENSG00000132604	TERF2
358.7319089	-0.204082587	0.266885399	-0.764682475	0.444460644	0.99135833	ENSG00000106290	TAF6
362.1273707	0.266422299	0.245569866	1.084914463	0.277959542	0.962465655	ENSG00000004766	VPS50
359.0940234	-0.011939215	0.213980419	-0.05579583	0.955504457	0.999527751	ENSG00000141560	FN3KRP
354.9596949	-0.41915633	0.30146118	-1.390415608	0.16440271	0.943223078	ENSG00000137038	DMAC1
358.949272	0.296346553	0.317175261	0.934330603	0.350133383	0.977691382	ENSG00000156030	ELMSAN1
354.6839457	-0.467881678	0.284317185	-1.645632774	0.099839387	0.92169898	ENSG00000005075	POLR2J
359.88557	0.019277215	0.227746859	0.084643165	0.932545082	0.999527751	ENSG00000023697	DERA
363.3399124	0.070028492	0.311010385	0.22516448	0.821851321	0.999467288	ENSG00000073711	PPP2R3A
356.0724993	0.048933695	0.28285403	0.172999817	0.862651571	0.999467288	ENSG00000120910	PPP3CC
357.1254224	0.0250443	0.254310815	0.098479101	0.921551866	0.999527751	ENSG00000143811	PYCR2
360.7033582	-0.001052307	0.24833904	-0.004237379	0.996619071	0.999527751	ENSG00000163281	GNPDA2
359.5352272	-0.170054538	0.255977528	-0.664333854	0.506476658	0.993513219	ENSG00000100580	TMED8
360.4259164	-0.286267629	0.246146078	-1.162998947	0.24482993	0.962465655	ENSG00000166167	BTRC
355.0253542	-0.348275207	0.307194819	-1.133727478	0.25690888	0.962465655	ENSG00000101460	MAP1LC3A
353.8764202	0.11963327	0.31241461	0.382931098	0.701770853	0.998869317	ENSG00000189077	TMEM120A
353.2335735	0.279189241	0.399890649	0.698163965	0.485074659	0.992117871	ENSG00000122420	PTGFR
358.6055327	-0.006595555	0.236689094	-0.027865903	0.977769104	0.999527751	ENSG00000103264	FBXO31
358.1639698	-0.112771861	0.227215748	-0.496320621	0.61966822	0.99483168	ENSG00000138286	FAM149B1
360.2120567	0.244406161	0.265770208	0.91961459	0.357774199	0.979427314	ENSG00000006756	ARSD
365.2028403	-1.497359525	0.422627322	-3.542978524	0.000395635	0.173156217	ENSG00000233901	LINC01503
355.147186	-0.155380757	0.25323219	-0.613590068	0.539486248	0.99483168	ENSG00000100865	CINP
360.5139764	0.245304807	0.311751265	0.786860664	0.431363437	0.991301167	ENSG00000103226	NOMO3
355.776727	0.175622138	0.270959452	0.648149148	0.516888488	0.993513219	ENSG00000162976	PQLC3
356.1009162	-0.003516473	0.248035156	-0.014177317	0.988688516	0.999527751	ENSG00000132603	NIP7
357.2369173	-0.296765644	0.223531591	-1.327622836	0.184302737	0.949094286	ENSG00000144827	ABHD10
359.407718	0.235147956	0.248759164	0.945283592	0.344514113	0.977691382	ENSG00000134480	CCNH
360.269779	0.012220539	0.326301806	0.037451644	0.970124896	0.999527751	ENSG00000089094	KDM2B
357.4621902	-0.356563895	0.250145423	-1.425426423	0.154033974	0.939432741	ENSG00000100207	TCF20
357.7555478	0.208165853	0.24077322	0.864572285	0.38727359	0.986799595	ENSG00000086200	IPO11
353.4082164	-0.125807431	0.309937796	-0.405911872	0.684807354	0.998869317	ENSG00000181817	LSM10
355.8623248	-0.772626678	0.332191332	-2.325848399	0.02002664	0.73975641	ENSG00000189171	S100A13
358.8751464	-0.253144557	0.269304022	-0.939995456	0.347219892	0.977691382	ENSG00000146834	MEPCE
362.1278904	-0.050060932	0.443665912	-0.11283475	0.910161568	0.999527751	ENSG00000069998	HDHD5
351.1679921	-0.316059534	0.422497654	-0.748074058	0.454415489	0.99135833	ENSG00000243244	STON1
361.2609419	0.210278829	0.313415562	0.670926574	0.502267307	0.993513219	ENSG00000128191	DGCR8
357.8535025	-0.136920515	0.304332958	-0.449903672	0.6527799	0.997471445	ENSG00000117054	ACADM

357.647944	0.1109789	0.227133927	0.488605561	0.625120975	0.99483168	ENSG00000183530	PRR14L
354.8324763	-0.300200863	0.459461296	-0.653375738	0.513514076	0.993513219	ENSG00000101871	MID1
357.2352227	0.01179819	0.244977358	0.04816033	0.961588466	0.999527751	ENSG00000058729	RIOK2
357.1811341	0.212029557	0.227850216	0.930565527	0.352078354	0.977691382	ENSG00000143614	GATAD2B
357.1814632	0.038726092	0.270338456	0.143250401	0.886092426	0.999467288	ENSG00000164347	GFM2
356.7009313	0.589433014	0.285175714	2.066911678	0.038742477	0.793985738	ENSG00000128039	SRD5A3
357.0085884	0.191017728	0.236089419	0.809090595	0.418463038	0.990226751	ENSG00000106443	PHF14
356.5318444	0.04097085	0.223515581	0.183301985	0.854561077	0.999467288	ENSG00000100462	PRMT5
357.0765126	0.466846483	0.246274656	1.895633478	0.058008527	0.852306368	ENSG00000162231	NXF1
357.5848469	-0.117304838	0.222250119	-0.527805513	0.59763433	0.99483168	ENSG00000139350	NEDD1
355.6305033	-0.28671379	0.271719479	-1.055183058	0.291341611	0.969851196	ENSG00000135974	C2orf49
353.4034743	-0.472207864	0.294050605	-1.605872784	0.108301864	0.923922314	ENSG00000173540	GMPPB
351.8296485	0.078128931	0.281562207	0.277483727	0.781408699	0.999467288	ENSG00000142733	MAP3K6
359.3439017	0.491375386	0.38520842	1.275609152	0.202093717	0.954310328	ENSG00000189223	PAX8-AS1
349.0647538	-0.352125221	0.289563365	-1.216055841	0.223963651	0.956884001	ENSG00000184602	SNN
352.6966738	-0.036857909	0.273186435	-0.134918518	0.892676298	0.999467288	ENSG00000143457	GOLPH3L
359.2388745	-0.205843231	0.296560776	-0.694101336	0.487618661	0.993513219	ENSG00000100814	CCNB1IP1
365.2664314	-0.27655859	0.423010622	-0.653786398	0.513249431	0.993513219	ENSG00000198142	SOWAHC
355.751999	-0.009901738	0.253976981	-0.038986752	0.968900951	0.999527751	ENSG00000155438	NIFK
363.5442136	-0.05498274	0.870463198	-0.063164922	0.949635177	0.999527751	ENSG00000246430	LINC00968
351.1404996	-0.318807387	0.292764446	-1.088955272	0.27617361	0.962465655	ENSG00000167112	TRUB2
357.5426532	-0.172740113	0.276376971	-0.625016304	0.531960357	0.99483168	ENSG00000182923	CEP63
358.2204923	0.411937155	0.332458326	1.239064035	0.215321785	0.954310328	ENSG00000224078	SNHG14
353.1342773	0.221870753	0.27147571	0.817276631	0.413770358	0.990226751	ENSG00000158623	COPG2
350.741783	-0.068394607	0.292499694	-0.23382796	0.815118543	0.999467288	ENSG00000196954	CASP4
355.7417616	0.082742752	0.244628254	0.338238739	0.735183285	0.999467288	ENSG00000100109	TFIP11
353.8451752	-0.039077469	0.232973064	-0.167733852	0.866792664	0.999467288	ENSG00000105447	GRWD1
352.5725105	-0.397659612	0.311199853	-1.277827121	0.20131039	0.954310328	ENSG00000158234	FAIM
351.2434161	-0.171114417	0.543133402	-0.31505044	0.752723357	0.999467288	ENSG00000169071	ROR2
353.0910023	0.041955593	0.230337189	0.182148583	0.855466123	0.999467288	ENSG00000100395	L3MBTL2
356.851804	0.107018567	0.306368422	0.349313307	0.726854109	0.999467288	ENSG00000137221	TJAP1
347.5544294	-0.436231071	0.367991451	-1.185438057	0.235844292	0.962465655	ENSG00000183011	NAA38
353.6972564	0.36959729	0.303833188	1.216448056	0.223814287	0.956884001	ENSG00000106688	SLC1A1
353.7367588	-0.048331991	0.224092614	-0.215678644	0.829238249	0.999467288	ENSG00000197579	TOPORS
351.430061	-0.254275654	0.31682985	-0.802562174	0.422227839	0.991301167	ENSG00000138735	PDE5A
347.0889563	-0.039957356	0.427501084	-0.093467262	0.925532357	0.999527751	ENSG00000136261	BZW2
356.3361438	-0.243918364	0.258664853	-0.942989976	0.34568603	0.977691382	ENSG00000157625	TAB3
349.2919399	0.269324155	0.269390712	0.999752935	0.317430088	0.974768742	ENSG00000091947	TMEM101
353.0812536	-0.240701119	0.224766572	-1.070893757	0.284217203	0.963310634	ENSG00000159111	MRPL10
347.0469272	-0.540331852	0.524602527	-1.029983318	0.303017836	0.972793733	ENSG00000198517	MAFK
350.4097802	-0.633122717	0.254975265	-2.483075046	0.013025364	0.667111125	ENSG00000172922	RNASEH2C
354.1322276	0.16951278	0.224759916	0.75419489	0.450732206	0.99135833	ENSG00000101138	CSTF1
355.253622	-0.438409316	0.333683698	-1.313846969	0.188897708	0.949094286	ENSG00000171703	TCEA2
351.4492784	0.018275904	0.229037735	0.079794292	0.936400864	0.999527751	ENSG00000152944	MED21
354.5994469	-0.167490704	0.240656287	-0.695974767	0.486444632	0.9933154	ENSG00000164818	DNAAF5
353.891216	0.083685017	0.239538537	0.349359308	0.726819578	0.999467288	ENSG00000128915	ICE2
350.9562692	0.156545221	0.260976021	0.599845229	0.548609387	0.99483168	ENSG00000038358	EDC4
351.4575206	-0.202611733	0.239164955	-0.847163136	0.396904198	0.988378925	ENSG00000104897	SF3A2
351.8750339	-0.222994033	0.283113899	-0.787647776	0.430902761	0.991301167	ENSG00000253293	HOXA10
351.5094083	-0.556932968	0.249318021	-2.233825564	0.025494553	0.759212013	ENSG00000150779	TIMM8B
347.314023	0.529094233	0.367775914	1.438632091	0.150254789	0.936396974	ENSG00000115339	GALNT3
351.1807609	0.549371311	0.270734014	2.029192057	0.042438732	0.806698895	ENSG00000164951	PDP1
353.6290974	0.092189624	0.271503889	0.339551763	0.734194109	0.999467288	ENSG00000061936	SFSWAP
349.8503567	-0.25138399	0.246158904	-1.021226476	0.307147151	0.973427483	ENSG00000167740	CYB5D2
351.6386033	-0.140288931	0.266060781	-0.52728151	0.597998112	0.99483168	ENSG00000170584	NUDCD2
350.8181887	-0.24752563	0.23976802	-1.032354646	0.30190601	0.972793733	ENSG00000002919	SNX11
353.723748	0.257324501	0.344751702	0.746405312	0.455422611	0.99135833	ENSG00000065060	UHRF1BP1
351.6806978	0.143711236	0.256706594	0.559826818	0.575597569	0.99483168	ENSG00000136715	SAP130
352.3443645	0.23951346	0.237275369	1.009432459	0.312767278	0.973427483	ENSG00000134152	KATNBL1
348.6892451	-0.933443231	0.279808069	-3.33601256	0.000849893	0.21163807	ENSG00000125652	ALKBH7
354.8104469	-0.411079358	0.416570024	-0.986819344	0.32373121	0.975412329	ENSG00000073464	CLCN4
351.2100299	-0.03229708	0.221011833	-0.14613281	0.883816545	0.999467288	ENSG00000175606	TMEM70
349.2293739	-0.412504489	0.283937463	-1.452800504	0.146279152	0.936017041	ENSG00000153406	NMRAL1
353.6206442	0.901273848	0.349081588	2.581842981	0.009827429	0.630529247	ENSG00000167615	LENG8

351.6912462	-0.353702096	0.238662074	-1.482020537	0.138334831	0.934144427	ENSG00000129235	TXNDC17
352.7128022	0.885974557	0.537053634	1.649694743	0.099005386	0.921147998	ENSG00000164104	HMGB2
355.3002688	-0.041632681	0.261333712	-0.159308497	0.873425827	0.999467288	ENSG00000278730	AC005332.6
349.318954	-0.286074973	0.278798937	-1.026097788	0.304845493	0.973427483	ENSG00000137168	PPIL1
348.26209	-0.169752153	0.27681611	-0.613230759	0.539723769	0.99483168	ENSG00000179912	R3HDM2
349.5246301	0.258801428	0.293836614	0.880766441	0.378444247	0.982609538	ENSG00000198796	ALPK2
347.517702	-0.472363219	0.274586357	-1.720271991	0.085383012	0.901307093	ENSG00000141971	MVB12A
349.4505709	-0.122349571	0.262106385	-0.466793553	0.640647589	0.996553586	ENSG00000135900	MRPL44
346.8185391	-0.304241878	0.409780481	-0.742450877	0.457814204	0.99135833	ENSG00000168386	FILIP1L
349.2457119	0.034865307	0.235820518	0.147846791	0.882463681	0.999467288	ENSG00000164941	INTS8
345.0990601	-0.390029193	0.567604374	-0.687149731	0.491988378	0.993513219	ENSG00000119986	AVPI1
349.1619848	0.067309848	0.291542313	0.230875056	0.81741187	0.999467288	ENSG00000167925	GHDC
352.2766929	-0.494044819	0.255589226	-1.93296418	0.053240611	0.847598083	ENSG00000060491	OGFR
348.92303	-0.013448585	0.29719512	-0.045251702	0.963906684	0.999527751	ENSG00000103174	NAGPA
351.6463322	0.131356671	0.313879341	0.41849416	0.675585856	0.998869317	ENSG00000156650	KAT6B
353.3385407	0.116392079	0.271288869	0.429033744	0.667898668	0.997568624	ENSG00000139826	ABHD13
351.3779893	0.070483948	0.249087214	0.282968951	0.777200638	0.999467288	ENSG00000113595	TRIM23
344.8230285	-0.035020594	0.571473045	-0.06128127	0.951135207	0.999527751	ENSG00000106537	TSPAN13
350.0706205	0.158921754	0.287516005	0.552740548	0.580441083	0.99483168	ENSG00000163291	PAQR3
350.181984	8.90E-05	0.244621259	0.000363713	0.999709799	0.9998586	ENSG00000148498	PARD3
346.8948286	0.043913865	0.250668455	0.175187042	0.860932666	0.999467288	ENSG00000136146	MED4
352.7573293	0.030454814	0.31277948	0.097368324	0.922433899	0.999527751	ENSG00000183309	ZNF623
352.4306046	0.35764181	0.320819503	1.114775777	0.264946519	0.962465655	ENSG00000102763	VWA8
344.9220274	-0.70593795	0.284115999	-2.484682146	0.012966715	0.667111125	ENSG00000074219	TEAD2
350.1702171	0.386330235	0.237195455	1.628742149	0.103367616	0.92169898	ENSG00000135870	RC3H1
347.9726368	-0.397598455	0.263022732	-1.511650542	0.130622787	0.929373153	ENSG00000088038	CNOT3
348.4994707	-0.22948095	0.261691299	-0.876914712	0.380532964	0.983746147	ENSG00000197892	KIF13B
340.1335357	0.090590645	0.436531117	0.207523913	0.83560072	0.999467288	ENSG00000135414	GDF11
345.7799647	0.33850183	0.370368594	0.913959323	0.36073822	0.979465649	ENSG00000082497	SERTAD4
344.74382	0.154945388	0.364648374	0.424917259	0.670896992	0.997568624	ENSG00000138744	NAAA
350.9565411	0.214755036	0.261622918	0.820857124	0.411727656	0.990226751	ENSG00000124574	ABCC10
348.129837	-0.012480254	0.230020218	-0.054257202	0.956730247	0.999527751	ENSG00000007923	DNAJC11
353.3553717	0.360941956	0.316206115	1.141476839	0.253671545	0.962465655	ENSG00000146842	TMEM209
355.9559367	-0.287487339	0.447531653	-0.64238437	0.520623645	0.993513219	ENSG00000177106	EPS8L2
351.5829743	-0.481366426	0.421051722	-1.143247733	0.252935749	0.962465655	ENSG00000219438	FAM19A5
346.0363964	-0.012487241	0.258249201	-0.048353454	0.961434554	0.999527751	ENSG00000131779	PEX11B
350.0801731	0.236875873	0.272120303	0.870482174	0.384036957	0.985655313	ENSG00000138463	DIRC2
348.4686969	-0.019311248	0.262555783	-0.073551027	0.94136764	0.999527751	ENSG00000136840	ST6GALNAC4
346.7731435	-0.125450144	0.255899254	-0.490232553	0.623969348	0.99483168	ENSG00000158019	BABAM2
342.1215366	0.526839145	0.37083394	1.420687505	0.155407627	0.939464191	ENSG00000116117	PARD3B
348.4162521	-0.240024738	0.218086416	-1.100594627	0.271073125	0.962465655	ENSG00000130749	ZC3H4
340.938809	-0.409968661	0.341461303	-1.200629934	0.229894784	0.959575179	ENSG00000158716	DUSP23
347.2320095	0.094829941	0.22785143	0.416191995	0.677269517	0.998869317	ENSG00000100023	PPIL2
335.5402497	0.139688525	0.417061603	0.334934994	0.737674124	0.999467288	ENSG00000140939	NOL3
348.558526	0.073134294	0.28265007	0.258745007	0.795831992	0.999467288	ENSG00000005175	RPAP3
347.320771	-0.520987655	0.242058333	-2.152322742	0.031371944	0.775963115	ENSG00000106012	IQCE
341.3984535	-0.192015067	0.35062411	-0.547637946	0.583940529	0.99483168	ENSG00000172465	TCEAL1
348.4140429	-0.127226733	0.228471996	-0.556859202	0.577623631	0.99483168	ENSG00000148153	INIP
345.5765963	0.261790352	0.287851557	0.90946304	0.363105759	0.98048242	ENSG00000092140	G2E3
345.9222444	-0.110754221	0.228654568	-0.484373536	0.628120796	0.99483168	ENSG00000170734	POLH
346.9217279	-0.035434084	0.270334608	-0.13107491	0.895716049	0.999467288	ENSG00000085760	MTIF2
343.2752406	0.178537358	0.247847175	0.720352602	0.471307925	0.99135833	ENSG00000171503	ETFDH
342.5789249	0.153098127	0.370891962	0.412783621	0.679765153	0.998869317	ENSG00000111716	LDHB
348.6373165	0.162638501	0.280397055	0.580029279	0.561894874	0.99483168	ENSG00000087266	SH3BP2
349.2349803	-0.233262877	0.288038211	-0.809833099	0.418036106	0.990226751	ENSG00000006015	REX1BD
344.1705856	-0.270213839	0.279095824	-0.968175859	0.332956549	0.976026345	ENSG00000100142	POLR2F
344.6479589	0.043427165	0.274389522	0.158268307	0.874245381	0.999467288	ENSG00000184990	SIVA1
341.842082	-0.348605154	0.325739568	-1.070195913	0.284531134	0.963310634	ENSG00000103152	MPG
343.7700936	0.185670459	0.220028118	0.84384878	0.398753913	0.988378925	ENSG00000139154	AEBP2
347.7769819	0.342208928	0.273733072	1.250155583	0.211242719	0.954310328	ENSG00000163781	TOPBP1
346.6002686	-0.149076479	0.270128417	-0.551872628	0.581035623	0.99483168	ENSG00000177683	THAP5
342.0267727	0.100460752	0.257257756	0.390506211	0.696162263	0.998869317	ENSG00000089818	NECAP1
341.4138699	-0.083017775	0.275984735	-0.300805679	0.763562678	0.999467288	ENSG00000123353	ORMDL2
347.1491261	-0.118626509	0.388206586	-0.305575724	0.759927707	0.999467288	ENSG00000099308	MAST3

342.5885918	0.125460856	0.321412261	0.390342469	0.696283323	0.998869317	ENSG00000105404	RABAC1
342.3006017	-0.248245255	0.260501351	-0.952951889	0.340614448	0.977691382	ENSG00000135778	NTPCR
335.2373404	-0.556225032	0.486939828	-1.142286994	0.253334746	0.962465655	ENSG00000254851	AP005018.2
342.9027565	0.177007329	0.257939987	0.686234543	0.492565218	0.993513219	ENSG00000100372	SLC25A17
340.1800299	-0.13329429	0.296738545	-0.44919776	0.653289003	0.997527562	ENSG00000011009	LYPLA2
343.4255485	-0.467447231	0.284240989	-1.644545468	0.10006358	0.92169898	ENSG00000099385	BCL7C
341.6224428	-0.364480379	0.324919425	-1.121756196	0.261966116	0.962465655	ENSG00000180155	LYNX1
342.3243783	-0.234466732	0.284555377	-0.823975753	0.409953335	0.990226751	ENSG00000166902	MRPL16
345.4589062	0.378481484	0.239132193	1.582729113	0.11348322	0.923922314	ENSG00000148843	PDCD11
347.0534908	0.247538988	0.283942792	0.871791768	0.383321986	0.985655313	ENSG00000101544	ADNP2
342.4378859	-0.088364241	0.354086233	-0.249555709	0.802930954	0.999467288	ENSG00000204054	LINC00963
335.8083046	0.423254137	0.321887515	1.314913184	0.188539081	0.949094286	ENSG00000073060	SCARB1
345.3786026	0.155281571	0.245696233	0.632006315	0.527382751	0.99483168	ENSG00000135597	REPS1
337.3769406	0.615645399	0.552025955	1.115247196	0.264744506	0.962465655	ENSG00000185022	MAFF
344.3244018	0.174862473	0.270816183	0.645686941	0.51848212	0.993513219	ENSG00000076685	NT5C2
340.9906123	-0.184689738	0.295494397	-0.625019425	0.531958309	0.99483168	ENSG00000171608	PIK3CD
349.1288537	0.016156593	0.38280132	0.042206209	0.966334313	0.999527751	ENSG00000160917	CPSF4
350.9079205	-0.195435927	0.455110016	-0.429425679	0.66761347	0.997568624	ENSG00000105290	APLP1
341.6295182	0.149071363	0.245589107	0.606995011	0.543854223	0.99483168	ENSG00000162623	TYW3
344.5464673	-0.038310293	0.356789516	-0.107375051	0.914491446	0.999527751	ENSG00000158321	AUTS2
341.9852844	-0.13703814	0.268888335	-0.509647026	0.610298772	0.99483168	ENSG00000173889	PHC3
339.0336784	-0.529787633	0.238536215	-2.220994546	0.026351331	0.763890866	ENSG00000233369	GTF2IP4
345.232367	-0.541524352	0.508575415	-1.06478673	0.286972452	0.965284229	ENSG00000166289	PLEKHF1
339.7127201	-0.480542192	0.266205874	-1.805152471	0.071050826	0.88331496	ENSG00000173141	MRPL57
345.5828547	-0.011521549	0.24999046	-0.046087954	0.963240147	0.999527751	ENSG00000055483	USP36
339.360644	0.020457925	0.283501774	0.072161542	0.94247335	0.999527751	ENSG00000127540	UQCR11
349.9783305	-0.683976967	0.396466413	-1.725182627	0.084494566	0.901307093	ENSG00000107821	KAZALD1
341.5095503	-0.160449264	0.241458312	-0.66450089	0.506369779	0.993513219	ENSG00000136522	MRPL47
349.971203	-0.154731763	0.406940579	-0.380231835	0.70377333	0.99900742	ENSG00000184985	SORCS2
336.4520604	-0.335088855	0.298081137	-1.124153171	0.260948052	0.962465655	ENSG00000167515	TRAPPC2L
341.2876603	0.078095558	0.238788328	0.32704931	0.74363059	0.999467288	ENSG00000154889	MPPE1
341.8202896	0.365963907	0.2546622	1.437056252	0.150702011	0.936396974	ENSG00000134255	CEPT1
339.1281844	-0.156496558	0.557375988	-0.280773771	0.778883922	0.999467288	ENSG00000115252	PDE1A
336.7993879	0.337104491	0.423767795	0.795493417	0.426326539	0.991301167	ENSG00000147155	EBP
334.2281817	-0.825139688	0.340374374	-2.424212135	0.015341641	0.694606015	ENSG00000105711	SCN1B
338.3202232	-0.164382519	0.282596439	-0.581686447	0.560777902	0.99483168	ENSG00000155099	PIP4P2
343.058497	0.128636521	0.313051964	0.410911081	0.681137737	0.998869317	ENSG00000132024	CC2D1A
334.9998899	-0.071702173	0.362018911	-0.198061955	0.842996591	0.999467288	ENSG00000211450	SELENOH
339.8540932	-0.221510099	0.234281013	-0.945488909	0.344409331	0.977691382	ENSG00000099910	KLHL22
342.2804431	0.164871148	0.366861384	0.449409928	0.653135971	0.997527562	ENSG00000157150	TIMP4
350.1280817	-0.182311287	0.613274033	-0.297275406	0.766256257	0.999467288	ENSG00000109472	CPE
341.2098951	-0.047536882	0.224327942	-0.211907985	0.832178821	0.999467288	ENSG00000134748	PRPF38A
343.2366007	0.013811451	0.305041113	0.045277344	0.963886246	0.999527751	ENSG00000124160	NCOA5
338.2714234	-0.099076218	0.263172792	-0.376468317	0.706568765	0.99900742	ENSG00000144524	COPS7B
344.7090799	0.23346972	0.329045582	0.709536103	0.477991856	0.99135833	ENSG00000178163	ZNF518B
342.7010886	0.442235796	0.264330161	1.673043265	0.094318779	0.921147998	ENSG00000184863	RBM33
336.6682196	0.147206745	0.303630656	0.484821746	0.627802796	0.99483168	ENSG00000096092	TMEM14A
337.546038	-0.199111479	0.346923209	-0.573935309	0.566011579	0.99483168	ENSG00000110080	ST3GAL4
339.971966	-0.285832676	0.236472161	-1.208737109	0.22676386	0.958865115	ENSG00000181191	PJA1
336.4241202	-0.266965308	0.307351397	-0.868599624	0.38506616	0.985733881	ENSG00000130956	HABP4
339.7085682	-0.085400196	0.262242555	-0.325653463	0.744686558	0.999467288	ENSG00000158796	DEDD
340.0505063	0.13929092	0.249682774	0.557871565	0.57693209	0.99483168	ENSG00000089234	BRAP
339.6634302	-0.154340648	0.267429993	-0.577125426	0.56385473	0.99483168	ENSG00000165914	TTC7B
341.8579465	-0.072062073	0.312142259	-0.230862919	0.817421299	0.999467288	ENSG00000122687	MRM2
339.3210756	0.137084059	0.247693302	0.553442738	0.579960281	0.99483168	ENSG00000089022	MAPKAPK5
338.6725784	0.825627132	0.494425043	1.669873206	0.094944453	0.921147998	ENSG00000156802	ATAD2
341.1606579	-0.071153695	0.242972465	-0.292846743	0.769639298	0.999467288	ENSG00000170903	MSANTD4
340.5083205	-0.193208624	0.432267256	-0.446965669	0.654899848	0.997568624	ENSG00000091972	CD200
342.6174904	-0.092335368	0.330831422	-0.279100961	0.780167346	0.999467288	ENSG00000134909	ARHGAP32
341.2815465	-0.15884603	0.238876546	-0.664971227	0.506068897	0.993513219	ENSG00000121766	ZCCHC17
337.7668496	0.312390808	0.236920958	1.318544422	0.187321465	0.949094286	ENSG00000119906	SLF2
341.3691406	0.305348162	0.279131791	1.09392112	0.273989575	0.962465655	ENSG00000174197	MGA
339.7813346	0.598735974	0.295886931	2.023529634	0.043018572	0.806698895	ENSG00000138829	FBN2
342.8270223	-0.215043046	0.260739219	-0.824743768	0.409517078	0.990226751	ENSG00000215114	UBXN2B

341.0541035	0.01583323	0.2615176	0.06054365	0.951722652	0.999527751	ENSG00000151532	VTI1A
338.5412812	-0.229723821	0.257132672	-0.893405803	0.371639896	0.981955904	ENSG00000163156	SCNM1
338.0629758	-0.045953268	0.26370507	-0.174260086	0.861661066	0.999467288	ENSG00000173065	FAM222B
337.3316135	-0.010991774	0.285234263	-0.038535954	0.969260366	0.999527751	ENSG00000143436	MRPL9
339.901741	-0.271053125	0.225294047	-1.203108243	0.228934436	0.959527137	ENSG00000114956	DGUOK
340.7313625	0.465758216	0.243643224	1.911640343	0.055922341	0.847598083	ENSG00000082258	CCNT2
340.5320632	0.142242886	0.257010735	0.553451148	0.579954523	0.99483168	ENSG00000163913	IFT122
345.9123544	-0.95805301	0.614754929	-1.558430789	0.119131163	0.923922314	ENSG00000178776	C5orf46
334.6713913	-0.205887387	0.287888593	-0.715163405	0.474508079	0.99135833	ENSG00000148450	MSRB2
340.479937	-0.221097894	0.250030334	-0.884284281	0.376542771	0.982609538	ENSG00000144228	SPOPL
337.4846059	-0.04738911	0.224552503	-0.211037997	0.832857622	0.999467288	ENSG00000164117	FBXO8
338.2623143	-0.229862294	0.249999521	-0.919450938	0.357859757	0.979427314	ENSG00000154832	CXXC1
338.5842153	-0.012200625	0.272644325	-0.044749234	0.96430719	0.999527751	ENSG00000136891	TEX10
335.7084326	-0.203887132	0.242532736	-0.840658195	0.400539447	0.988492517	ENSG00000100949	RABGGTA
341.2292008	-0.178265195	0.316959003	-0.56242351	0.573827505	0.99483168	ENSG00000100426	ZBED4
332.5697101	0.513747714	0.419931099	1.223409543	0.221175051	0.956884001	ENSG00000136111	TBC1D4
338.0714712	-0.047886494	0.229955203	-0.208242708	0.835039463	0.999467288	ENSG00000122140	MRPS2
341.1817879	0.495979501	0.288038923	1.721918331	0.085084314	0.901307093	ENSG00000157181	ODR4
336.1788827	0.449091973	0.296845027	1.512883603	0.130309228	0.929373153	ENSG00000178773	CPNE7
336.6412906	0.045652324	0.319406843	0.142928447	0.886346692	0.999467288	ENSG00000204389	HSPA1A
348.1239703	-0.121322644	0.420451131	-0.288553496	0.772923087	0.999467288	ENSG00000164484	TMEM200A
337.9254553	0.243951925	0.249006806	0.979699829	0.327234311	0.976026345	ENSG00000070610	GBA2
353.4617638	-0.856335746	0.38687575	-2.213464518	0.026865637	0.765286378	ENSG00000101463	SYNDIG1
338.3030485	-0.57095911	0.264064272	-2.162197501	0.030602956	0.775963115	ENSG00000242299	AC073861.1
338.1555417	-0.637512032	0.316716853	-2.012876885	0.044127583	0.807325344	ENSG00000132429	POPDC3
333.8396042	-0.500477473	0.284664251	-1.758132505	0.078724966	0.897052166	ENSG00000102871	TRADD
335.3867368	0.886996414	0.468899911	1.891654046	0.05853709	0.852621724	ENSG00000139734	DIAPH3
333.9141751	0.63274135	0.334123046	1.893737523	0.058259858	0.852621724	ENSG00000164211	STARD4
337.0841692	-0.208091257	0.312678823	-0.665511193	0.505723587	0.993513219	ENSG00000254635	WAC-AS1
340.2518456	0.081637914	0.274610792	0.29728589	0.766248253	0.999467288	ENSG00000120685	PROSER1
333.6702835	0.086658266	0.244080456	0.355039759	0.722559805	0.999092366	ENSG00000241685	ARPC1A
339.5300648	0.483166398	0.294210037	1.642249882	0.100538229	0.92169898	ENSG00000111300	NAA25
336.8907818	-0.502336392	0.292201186	-1.719145628	0.085587858	0.901307093	ENSG00000142409	ZNF787
336.4840984	0.525649077	0.271294772	1.937556976	0.052677296	0.847598083	ENSG00000134186	PRPF38B
336.2353667	-0.052998411	0.286213111	-0.185171151	0.853094796	0.999467288	ENSG00000187091	PLCD1
335.2666295	0.689670276	0.297243518	2.320219738	0.020328993	0.73997555	ENSG00000225830	ERCC6
338.7964159	-0.087198584	0.246849787	-0.35324553	0.723904377	0.999456245	ENSG00000196700	ZNF512B
337.0137586	-0.398680362	0.271342835	-1.469286493	0.141755099	0.935644597	ENSG00000167565	SERTAD3
327.8686437	-0.619299339	0.346262691	-1.788524592	0.073691412	0.888663898	ENSG00000130720	FIBCD1
340.0598736	-0.377538167	0.38971797	-0.968747135	0.332671369	0.976026345	ENSG00000103126	AXIN1
333.8924485	0.258245619	0.257607525	1.002477001	0.316113269	0.974768742	ENSG00000133895	MEN1
332.1489731	-0.002798288	0.306358657	-0.009134027	0.992712202	0.999527751	ENSG00000162813	BPNT1
338.0731696	0.256923806	0.307330584	0.835985155	0.403163254	0.988927059	ENSG00000138031	ADCY3
332.1827922	-0.158361284	0.320511047	-0.494089939	0.621242663	0.99483168	ENSG00000167283	ATP5MG
337.3671102	-0.075112224	0.276136325	-0.272011386	0.785613264	0.999467288	ENSG00000160199	PKNOX1
332.2458296	0.154491248	0.260558312	0.592923888	0.55323209	0.99483168	ENSG00000163249	CCNYL1
336.1331045	0.333215547	0.232673931	1.432113799	0.152111256	0.938052926	ENSG00000124228	DDX27
334.040623	-0.03617319	0.254201247	-0.142301389	0.88684195	0.999467288	ENSG00000102897	LYRM1
332.1759187	0.005666113	0.261886903	0.021635724	0.982738537	0.999527751	ENSG00000115226	FNDC4
332.3401898	-0.075316898	0.250711589	-0.300412512	0.763862521	0.999467288	ENSG00000162961	DPY30
336.3582998	-0.177224807	0.228611673	-0.775222035	0.43820847	0.991301167	ENSG00000082213	C5orf22
335.9937214	0.065193699	0.300529142	0.216929708	0.828263125	0.999467288	ENSG00000099219	ERMP1
336.1761662	-0.069853131	0.292117001	-0.239127238	0.811006922	0.999467288	ENSG00000019995	ZRANB1
336.1083324	-0.387631918	0.280554018	-1.381665893	0.167074312	0.943223078	ENSG00000174938	SEZ6L2
339.3500327	-0.345893261	0.300758331	-1.150070426	0.250114866	0.962465655	ENSG00000111275	ALDH2
334.7045672	0.023540486	0.25934833	0.090767834	0.927677069	0.999527751	ENSG00000223501	VPS52
332.7341024	0.107187985	0.252331274	0.424790727	0.670989238	0.997568624	ENSG00000115211	EIF2B4
332.4295924	-0.243846127	0.271707717	-0.897457492	0.369474846	0.981455968	ENSG00000123595	RAB9A
331.4640022	-0.233850741	0.36549287	-0.639822991	0.522287684	0.994515626	ENSG00000213563	C8orf82
333.9006431	-0.038648187	0.290814278	-0.132896457	0.894275269	0.999467288	ENSG00000139722	VPS37B
334.7081849	0.50798621	0.289939862	1.75203991	0.079766946	0.897052166	ENSG00000188811	NHLRC3
332.1853917	0.290764342	0.290930086	0.999430297	0.317586289	0.974768742	ENSG00000010704	HFE
336.7986265	0.204036834	0.274860191	0.74232952	0.457887711	0.99135833	ENSG00000164631	ZNF12
335.0962521	-0.08980117	0.233502123	-0.384583953	0.700545685	0.998869317	ENSG00000094631	HDAC6

334.9637116	1.23850007	0.307709914	4.024894915	5.70E-05	0.054234087	ENSG00000163660	CCNL1
335.2079921	-0.251908649	0.229925784	-1.095608524	0.27325013	0.962465655	ENSG00000170633	RNF34
336.8299828	0.231809122	0.282362838	0.820961866	0.41166799	0.990226751	ENSG00000148606	POLR3A
333.5716061	-0.040390109	0.325370401	-0.124135782	0.901207767	0.999527751	ENSG00000109381	ELF2
332.1096638	0.025620607	0.264715315	0.09678551	0.922896731	0.999527751	ENSG00000144306	SCRN3
330.1071938	-0.207818506	0.298466885	-0.696286645	0.486249335	0.993197447	ENSG00000198585	NUDT16
334.4264117	0.146385351	0.284857737	0.513889329	0.607329369	0.99483168	ENSG00000175931	UBE2O
330.4643073	0.043599651	0.270733925	0.161042437	0.872059977	0.999467288	ENSG00000157657	ZNF618
331.8399704	-0.036789729	0.246630956	-0.149169147	0.881420165	0.999467288	ENSG00000142634	EFHD2
334.9467817	-0.248594742	0.350724414	-0.708803643	0.478446337	0.99135833	ENSG00000125834	STK35
332.3780732	0.523136249	0.343558375	1.522699741	0.127833843	0.928627271	ENSG00000132780	NASP
334.848171	-0.125622843	0.270949462	-0.463639387	0.64290613	0.997096914	ENSG00000197976	AKAP17A
330.904265	0.186137037	0.315805859	0.589403366	0.555590719	0.99483168	ENSG00000187676	B3GLCT
328.5196598	-0.498846698	0.289610694	-1.722473334	0.084983809	0.901307093	ENSG00000130165	ELOF1
326.3918328	-0.033532222	0.329967176	-0.101622901	0.919056001	0.999527751	ENSG00000164237	CMBL
331.580128	0.842695008	0.508490549	1.657248122	0.097469321	0.921147998	ENSG00000104738	MCM4
333.4931159	0.115990048	0.246871067	0.46984059	0.638468912	0.996553586	ENSG00000254004	ZNF260
332.8053414	-0.028465227	0.311877941	-0.091270408	0.927277731	0.999527751	ENSG00000114491	UMPS
329.7711035	0.479414217	0.293589815	1.632938856	0.102481857	0.92169898	ENSG00000155903	RASA2
335.8603708	-0.00438729	0.317958624	-0.013798305	0.988990895	0.999527751	ENSG00000136144	RCBTB1
331.6714244	0.350643197	0.316717683	1.107115946	0.268243802	0.962465655	ENSG00000117262	GPR89A
332.6396693	0.175964792	0.262442442	0.67048908	0.502546065	0.993513219	ENSG00000119403	PHF19
332.6010777	0.290910963	0.235817149	1.233629379	0.21734104	0.956186483	ENSG00000145041	DCAF1
331.6695093	0.130798072	0.23941258	0.546329154	0.584839699	0.99483168	ENSG00000069966	GNB5
330.6658819	0.328179415	0.413485514	0.793690235	0.427375786	0.991301167	ENSG00000102393	GLA
332.047575	-0.318024393	0.315342603	-1.008504368	0.313212392	0.973427483	ENSG00000184281	TSSC4
331.271036	0.16826905	0.23930014	0.703171548	0.481948848	0.991525194	ENSG00000068650	ATP11A
331.5085218	-0.751968842	0.351962966	-2.136499899	0.032638689	0.775963115	ENSG00000090238	YPEL3
330.6780009	0.349842134	0.287341774	1.217512264	0.223409373	0.956884001	ENSG00000172716	SLFN11
333.7861445	0.564806328	0.298606675	1.891472544	0.058561293	0.852621724	ENSG00000176595	KBTBD11
325.9398701	-0.265291801	0.491151261	-0.540142766	0.58909858	0.99483168	ENSG00000151692	RNF144A
329.5100455	1.737250861	0.462073733	3.75968322	0.000170129	0.09924856	ENSG00000237515	SHISA9
320.9106313	-0.467945813	1.520137488	-0.307831244	0.758210746	0.999467288	ENSG00000196126	HLA-DRB1
330.8792069	0.394588014	0.244879618	1.611355066	0.107102355	0.923922314	ENSG00000236287	ZBED5
329.6184619	0.040626135	0.247730774	0.163993089	0.869736584	0.999467288	ENSG00000115816	CEBPZ
329.9072339	-0.07436048	0.226359035	-0.32850679	0.742528511	0.999467288	ENSG00000156735	BAG4
329.5292006	-0.20940516	0.364739744	-0.574122134	0.565885156	0.99483168	ENSG00000173068	BNC2
328.4163861	-0.162946272	0.301526091	-0.540405214	0.588917613	0.99483168	ENSG00000170456	DENND5B
330.4718525	-0.322971901	0.287630382	-1.122871301	0.261492158	0.962465655	ENSG00000168438	CDC40
327.288533	0.002986557	0.298732237	0.009997439	0.992023331	0.999527751	ENSG00000105676	ARMC6
332.5625574	-0.330278201	0.305939186	-1.079555075	0.280340356	0.963267117	ENSG00000102034	ELF4
338.8886205	0.023062402	0.499487369	0.046172143	0.963173046	0.999527751	ENSG00000143367	TUFT1
327.6116521	-0.344164256	0.35184506	-0.978169927	0.327990288	0.976026345	ENSG00000153443	UBALD1
325.7502096	-0.112913517	0.234724997	-0.481045983	0.630483818	0.99483168	ENSG00000090013	BLVRB
329.9030654	-0.317336999	0.247988686	-1.279643054	0.200670701	0.954310328	ENSG00000174547	MRPL11
326.7632997	0.550596924	0.404164172	1.362310077	0.173100049	0.944422693	ENSG00000144369	FAM171B
327.250738	-0.210953163	0.272876594	-0.773071665	0.439479969	0.991301167	ENSG00000004864	SLC25A13
329.8247453	-0.023439138	0.265467463	-0.088293826	0.929643146	0.999527751	ENSG00000105738	SIPA1L3
330.3219707	0.283925194	0.258400771	1.098778432	0.271864722	0.962465655	ENSG00000066422	ZBTB11
327.4818153	-0.742381096	0.510950701	-1.452940753	0.146240205	0.936017041	ENSG00000108551	RASD1
327.550409	0.793677318	0.455815999	1.741223037	0.081644491	0.897807954	ENSG00000101224	CDC25B
330.7653402	0.049995075	0.275783457	0.181283807	0.856144815	0.999467288	ENSG00000123106	CCDC91
323.0045658	-0.111510059	0.434356846	-0.256724534	0.797391431	0.999467288	ENSG00000165804	ZNF219
324.4478706	0.048344067	0.357478386	0.135236335	0.89242502	0.999467288	ENSG00000100911	PSME2
330.3930316	-0.057157248	0.249572942	-0.229020213	0.818853206	0.999467288	ENSG00000100296	THOC5
325.5310237	0.104874085	0.308149536	0.340335043	0.73360423	0.999467288	ENSG00000035141	FAM136A
325.6863914	-0.184228791	0.266735715	-0.690679129	0.489767208	0.993513219	ENSG00000160446	ZDHHC12
337.0658801	-0.026163068	1.172495295	-0.022314006	0.982197476	0.999527751	ENSG00000125845	BMP2
328.8854079	-0.196328755	0.222038444	-0.884210639	0.376582516	0.982609538	ENSG00000137074	APTX
334.2334518	-0.441234068	0.359187895	-1.228421322	0.219288848	0.956884001	ENSG00000070366	SMG6
331.5214591	0.329258528	0.323593538	1.017506501	0.308912541	0.973427483	ENSG00000135164	DMTF1
326.2519819	0.106694982	0.249968299	0.426834052	0.669500203	0.997568624	ENSG00000123352	SPATS2
327.17171	-0.587796518	0.242234011	-2.426564773	0.015242529	0.694606015	ENSG00000084764	MAPRE3
327.4029017	-0.291492136	0.335245742	-0.869487958	0.384580292	0.985655313	ENSG00000123395	ATG101

324.7394087	0.018884645	0.315453437	0.059865079	0.952263093	0.999527751	ENSG00000232112	TMA7
325.207173	0.090465455	0.276290995	0.327428172	0.743344062	0.999467288	ENSG00000120314	WDR55
325.9391638	-0.224821769	0.251065718	-0.895469801	0.370536002	0.981455968	ENSG00000172586	CHCHD1
327.1387594	-0.098403615	0.220572603	-0.446128002	0.655504787	0.997568624	ENSG00000205629	LCMT1
320.8250014	-0.366109463	0.387525718	-0.944735912	0.344793718	0.977691382	ENSG00000185813	PCYT2
330.6198511	0.143628537	0.292474521	0.491080511	0.623369505	0.99483168	ENSG00000127415	IDUA
329.3191837	0.283275676	0.404335605	0.700595428	0.483555532	0.991892545	ENSG00000126001	CEP250
328.179955	0.130744204	0.244140846	0.535527777	0.592284955	0.99483168	ENSG00000132716	DCAF8
324.5562915	1.000467923	0.534372649	1.872228913	0.061174941	0.863606039	ENSG00000183287	CCBE1
327.3616704	0.744188178	0.279438528	2.66315523	0.007741169	0.567541675	ENSG00000221963	APOL6
329.2799801	0.238054626	0.279287627	0.852363669	0.394012278	0.988378925	ENSG00000196950	SLC39A10
330.4058048	0.753598507	0.376529011	2.001435439	0.045345485	0.813244956	ENSG00000118495	PLAGL1
328.0552111	-0.006791253	0.244409615	-0.027786357	0.977832547	0.999527751	ENSG00000117133	RPF1
327.6776269	0.520884836	0.467296027	1.114678503	0.264988216	0.962465655	ENSG00000164647	STEAP1
327.1076878	-0.094314806	0.233594131	-0.403755034	0.686392865	0.998869317	ENSG00000005302	MSL3
328.1989407	-0.122395879	0.254544407	-0.480842932	0.630628135	0.99483168	ENSG00000177981	ASB8
325.7681555	-0.369554636	0.261554974	-1.412913816	0.157681066	0.939464191	ENSG00000185085	INTS5
325.8348675	0.106804985	0.31633294	0.337634724	0.735638471	0.999467288	ENSG00000120253	NUP43
329.1612315	0.133610063	0.285856261	0.467402961	0.640211604	0.996553586	ENSG00000125386	FAM193A
326.8578504	1.274145936	0.942843015	1.351387151	0.176571448	0.946278819	ENSG00000137807	KIF23
324.8486775	0.596427015	0.332118155	1.7958278	0.072521908	0.885710197	ENSG00000118596	SLC16A7
324.8140288	0.098484252	0.299031102	0.329344512	0.741895304	0.999467288	ENSG00000140396	NCOA2
328.7583027	0.425511671	0.376462485	1.130289706	0.258354171	0.962465655	ENSG00000185033	SEMA4B
323.338009	0.506110587	0.332848095	1.520545243	0.128373997	0.928627271	ENSG00000134802	SLC43A3
326.9710134	-0.135762077	0.279862608	-0.485102592	0.627603574	0.99483168	ENSG00000020256	ZFP64
325.2464256	0.338856889	0.294977098	1.148756602	0.250656359	0.962465655	ENSG00000171105	INSR
328.6267024	-0.028995094	0.260956587	-0.111110796	0.911528488	0.999527751	ENSG00000144909	OSBPL11
322.3509637	0.086932978	0.321650762	0.270271327	0.786951522	0.999467288	ENSG00000142197	DOP1B
323.1000011	0.530330696	0.263382495	2.013538131	0.044058048	0.807325344	ENSG00000145014	TMEM44
324.0624107	-0.052834468	0.267793736	-0.197295384	0.843596392	0.999467288	ENSG00000165516	KLHDC2
322.0979619	-0.153185183	0.270149767	-0.567037996	0.570688367	0.99483168	ENSG00000032389	EIPR1
324.5087804	0.282818248	0.265254243	1.066215737	0.28632613	0.964864278	ENSG00000104472	CHRAC1
323.6072885	-0.043880127	0.269070377	-0.163080482	0.870455067	0.999467288	ENSG00000144048	DUSP11
325.7543181	-0.159425649	0.240648099	-0.662484558	0.507660728	0.993513219	ENSG00000137601	NEK1
326.7170373	0.061492817	0.24201171	0.254090253	0.799425833	0.999467288	ENSG00000213965	NUDT19
319.9717253	0.019747506	0.315591121	0.062573074	0.950106471	0.999527751	ENSG00000179152	TCAIM
320.2446042	-0.50838327	0.281807266	-1.804010513	0.071229659	0.883822992	ENSG00000277972	CISD3
320.0553352	0.571354959	0.402844119	1.418302843	0.156102367	0.939464191	ENSG00000173559	NABP1
321.9763062	0.149942709	0.295846144	0.506826648	0.612276457	0.99483168	ENSG00000222041	CYTOR
318.3637332	0.45246307	0.37709303	1.19987121	0.230189363	0.959575179	ENSG00000163840	DTX3L
321.1742165	-0.367526194	0.280447361	-1.31049974	0.190026832	0.949094286	ENSG00000124587	PEX6
322.6818605	0.319404295	0.266869817	1.196854327	0.231363339	0.959575179	ENSG00000120526	NUDCD1
324.4658862	0.345606945	0.442479715	0.781068449	0.434762237	0.991301167	ENSG00000176971	FIBIN
321.7834589	-0.226390068	0.250205265	-0.904817365	0.36556215	0.981269214	ENSG00000172992	DCAKD
321.6681925	-0.354995947	0.377579888	-0.940187648	0.347121316	0.977691382	ENSG00000142694	EVA1B
323.1857512	-0.32960249	0.305497182	-1.078905173	0.280630002	0.963267117	ENSG00000145916	RMND5B
321.6259576	-0.014107904	0.287006021	-0.04915543	0.96079543	0.999527751	ENSG00000112699	GMDS
323.5594508	0.436803986	0.500293522	0.873095427	0.382611066	0.98555814	ENSG00000101194	SLC17A9
325.3576705	0.061766048	0.258805766	0.238657929	0.811370842	0.999467288	ENSG00000164291	ARSK
321.1410723	-0.161755556	0.287030844	-0.563547644	0.573062027	0.99483168	ENSG00000169599	NFU1
323.4749256	-0.571459943	0.23978339	-2.383234058	0.017161279	0.716359406	ENSG00000223768	LINC00205
321.4408854	0.254968976	0.283969384	0.897874878	0.369252259	0.981455968	ENSG00000123080	CDKN2C
323.5365368	0.432325682	0.235668614	1.834464398	0.066585088	0.87420149	ENSG00000135776	ABCB10
330.7270343	-0.107288043	0.616443428	-0.174043615	0.861831185	0.999467288	ENSG00000118785	SPP1
323.662606	0.49737533	0.267916828	1.856454236	0.06338884	0.868202292	ENSG00000164327	RICTOR
321.1607691	0.113905783	0.303129182	0.375766473	0.707090515	0.99900742	ENSG00000111110	PPM1H
319.1580587	-0.209419885	0.32273873	-0.648883648	0.516413585	0.993513219	ENSG00000165233	CARD19
326.3798986	-0.343497198	0.310410625	-1.106589695	0.268471365	0.962465655	ENSG00000087303	NID2
317.4321837	-0.65569927	0.453482155	-1.445920777	0.148199424	0.936396974	ENSG00000143977	SNRPG
322.981967	-0.204915493	0.236334606	-0.867056656	0.385910968	0.986238739	ENSG00000106993	CDC37L1
324.2031764	-0.076668802	0.261241455	-0.293478697	0.769156281	0.999467288	ENSG00000148297	MED22
322.9535468	0.149764935	0.251143219	0.596332784	0.550952934	0.99483168	ENSG00000117533	VAMP4
323.875501	-0.139249181	0.247783734	-0.561978702	0.57413053	0.99483168	ENSG00000105662	CRTC1
323.0719517	-0.167110795	0.235073757	-0.710886647	0.477154481	0.99135833	ENSG00000070814	TCOF1

320.0101669	-0.126158898	0.264595838	-0.476798496	0.633505617	0.995376704	ENSG00000167632	TRAPPC9
321.2878955	0.114213805	0.241893452	0.472165756	0.63680848	0.99653524	ENSG00000138032	PPM1B
326.6492182	0.343304314	0.307063454	1.118024009	0.263556739	0.962465655	ENSG00000272888	LINC01578
321.2108329	-0.023214082	0.308127802	-0.075339134	0.939944885	0.999527751	ENSG00000188641	DPYD
316.0698238	0.237617541	0.357780166	0.664143974	0.506598168	0.993513219	ENSG00000152284	TCF7L1
320.2989584	0.356865988	0.388416197	0.918772159	0.358214758	0.979427314	ENSG00000168621	GDNF
324.4615081	-0.118368174	0.241359694	-0.49042229	0.623835107	0.99483168	ENSG00000139718	SETD1B
324.5512225	-0.741050567	0.359714961	-2.06010494	0.03938851	0.793985738	ENSG00000025708	TYMP
311.7681566	0.852605362	0.53385641	1.597068698	0.110250395	0.923922314	ENSG00000182985	CADM1
319.5787576	-0.336514328	0.319218589	-1.054181491	0.291799832	0.97025964	ENSG00000140854	KATNB1
321.2561499	0.128595666	0.32369204	0.397277815	0.691162605	0.998869317	ENSG00000080608	PUM3
323.5938577	-0.076842318	0.324813418	-0.236573718	0.812987511	0.999467288	ENSG00000135723	FHOD1
321.0537372	0.378417051	0.239799738	1.578054476	0.114553095	0.923922314	ENSG00000165152	TMEM246
319.7242762	0.148933525	0.242307887	0.614645799	0.538788662	0.99483168	ENSG00000204160	ZDHHC18
321.7134707	-0.060155321	0.286962098	-0.209628105	0.833957942	0.999467288	ENSG00000041802	LSG1
322.730177	-0.033075525	0.242558142	-0.136361224	0.891535726	0.999467288	ENSG00000146426	TIAM2
320.4938768	0.02518061	0.288846082	0.087176568	0.930531164	0.999527751	ENSG00000174891	RSRC1
319.7759811	-0.096493555	0.2503386	-0.385452162	0.699902443	0.998869317	ENSG00000065268	WDR18
319.3838276	0.089773698	0.302237915	0.297029901	0.766443681	0.999467288	ENSG00000117174	ZNHIT6
316.8048219	-0.223173755	0.296950603	-0.751551782	0.452320649	0.99135833	ENSG00000108826	MRPL27
319.9623343	0.027903348	0.260632747	0.107060025	0.91474136	0.999527751	ENSG00000131067	GGT7
318.6039801	-0.143345887	0.286153869	-0.500939887	0.61641343	0.99483168	ENSG00000137714	FDX1
322.0140877	0.446074928	0.288925971	1.543907339	0.122610782	0.92570179	ENSG00000153246	PLA2R1
321.1306886	-0.108164476	0.234047397	-0.462147741	0.643975376	0.997332006	ENSG00000060688	SNRNP40
316.9027212	0.14021722	0.282157758	0.496946178	0.619227006	0.99483168	ENSG00000168502	MTCL1
319.2279984	-0.192880023	0.300351362	-0.642181281	0.520755485	0.993513219	ENSG00000175906	ARL4D
327.8074377	0.097459512	0.373958238	0.260616032	0.794388625	0.999467288	ENSG00000095951	HIVEP1
326.5093205	0.270482854	0.508170457	0.532267963	0.594540418	0.99483168	ENSG00000088826	SMOX
318.2407119	-0.192073416	0.267718106	-0.717446491	0.473098642	0.99135833	ENSG00000119878	CRIPT
318.8993219	0.172377337	0.250404681	0.688395028	0.491204054	0.993513219	ENSG00000187097	ENTPD5
317.7021443	-0.231701056	0.329180014	-0.703873402	0.481511617	0.991525194	ENSG00000070785	EIF2B3
317.5652815	-0.416443287	0.272711628	-1.527046315	0.126749495	0.928627271	ENSG00000115661	STK16
317.2639774	-0.172955271	0.266918311	-0.6479708	0.517003837	0.993513219	ENSG00000167987	VPS37C
319.2638363	-0.191883845	0.24785218	-0.774186634	0.438820431	0.991301167	ENSG00000177613	CSTF2T
318.3891994	0.130883657	0.236629177	0.553117153	0.580183192	0.99483168	ENSG00000198001	IRAK4
317.3050777	-0.440217348	0.248183921	-1.773754504	0.076103767	0.897052166	ENSG00000136463	TACO1
319.4012518	-0.044212542	0.26085344	-0.169491886	0.865409754	0.999467288	ENSG00000116809	ZBTB17
316.5167818	-0.164885457	0.271915431	-0.606385068	0.544259082	0.99483168	ENSG00000157014	TATDN2
318.4895621	0.096863638	0.267951801	0.361496498	0.717728322	0.99900742	ENSG00000174720	LARP7
318.3299235	0.420137868	0.248463567	1.690943556	0.090847583	0.912649921	ENSG00000047346	FAM214A
321.4945044	0.226820165	0.260377213	0.871121411	0.383687865	0.985655313	ENSG00000177034	MTX3
323.7118154	0.411263698	0.317574902	1.295013225	0.195315667	0.953843167	ENSG00000160299	PCNT
319.5110449	-0.074752934	0.235912481	-0.316867229	0.751344349	0.999467288	ENSG00000171497	PPID
317.224725	-0.278895781	0.259942366	-1.07291391	0.283309744	0.963267117	ENSG00000103121	CMC2
323.0696732	0.563014556	0.409170458	1.375990239	0.168824655	0.943792179	ENSG00000138018	SELENOI
319.1840657	-0.130000267	0.237176455	-0.548116241	0.58361209	0.99483168	ENSG00000128654	MTX2
320.6905594	0.001668737	0.317553866	0.005254972	0.995807158	0.999527751	ENSG00000184838	PRR16
315.5097661	-0.342706317	0.247662023	-1.383766123	0.166430079	0.943223078	ENSG00000237506	RPSAP15
318.2970599	0.045518432	0.27367867	0.166320715	0.867904564	0.999467288	ENSG00000099381	SETD1A
319.2452706	-0.007537711	0.29470178	-0.02557742	0.979594397	0.999527751	ENSG00000198863	RUNDC1
319.1127372	-0.189395172	0.284482866	-0.665752472	0.505569328	0.993513219	ENSG00000008405	CRY1
319.776457	0.217447585	0.27838093	0.781115233	0.434734723	0.991301167	ENSG00000203880	PCMTD2
319.4574961	0.19451709	0.340316236	0.571577462	0.567608268	0.99483168	ENSG00000162618	ADGRL4
320.6697809	0.297407668	0.362936198	0.819448899	0.412530346	0.990226751	ENSG00000175054	ATR
318.1504944	0.341673435	0.305175928	1.119594971	0.262886399	0.962465655	ENSG00000179241	LDLRAD3
316.5591757	-0.123880767	0.326858552	-0.379004209	0.704684738	0.99900742	ENSG00000261221	ZNF865
321.7825178	-0.431697359	0.316165822	-1.365414374	0.172122848	0.944422693	ENSG00000164684	ZNF704
313.0137881	-0.375981043	0.325944176	-1.153513608	0.248699635	0.962465655	ENSG00000119013	NDUFB3
311.7960103	0.15067686	0.321761758	0.468287036	0.639579338	0.996553586	ENSG00000114166	KAT2B
315.5518926	-0.392529429	0.284170691	-1.381315676	0.16718192	0.943223078	ENSG00000084693	AGBL5
319.3076757	0.431439341	0.283813199	1.520152488	0.128472655	0.928627271	ENSG00000156304	SCAF4
315.6489373	-0.054848504	0.224408363	-0.244413814	0.806910344	0.999467288	ENSG00000143376	SNX27
313.9975401	-0.160141462	0.277827324	-0.576406453	0.564340483	0.99483168	ENSG00000189306	RRP7A
318.5226669	-0.075694414	0.266005877	-0.28455918	0.775981888	0.999467288	ENSG00000205356	TECPR1

318.4238309	-0.275182968	0.298688909	-0.921302933	0.356892291	0.979427314	ENSG00000263753	LINC00667
322.798995	-0.330018536	0.453256305	-0.72810578	0.466548836	0.99135833	ENSG00000232774	FLJ22447
317.343551	0.275648913	0.233674787	1.179626253	0.238148897	0.962465655	ENSG00000101146	RAE1
312.7328007	-0.25168731	0.293781483	-0.856716042	0.391601835	0.988355932	ENSG00000136710	CCDC115
316.1981881	0.17838733	0.248252497	0.718572146	0.47240458	0.99135833	ENSG00000038532	CLEC16A
316.1870032	-0.273402325	0.237930885	-1.149082954	0.250521777	0.962465655	ENSG00000145388	METTL14
318.3362706	0.124741161	0.237580524	0.525047923	0.599549877	0.99483168	ENSG00000112837	TBX18
311.1623571	0.47364537	0.409806661	1.155777627	0.247772127	0.962465655	ENSG00000196569	LAMA2
312.3229974	-0.168693864	0.309238136	-0.545514423	0.585399764	0.99483168	ENSG00000137806	NDUFAF1
316.1692111	0.534366832	0.385979848	1.384442308	0.166223062	0.943223078	ENSG00000040275	SPDL1
316.2938492	0.027114275	0.23296898	0.116385774	0.907346809	0.999527751	ENSG00000106355	LSM5
310.995395	-1.064515784	0.256761111	-4.145938525	3.38E-05	0.037598976	ENSG00000181541	MAB21L2
316.0177156	0.07343253	0.259963609	0.282472343	0.777581351	0.999467288	ENSG00000157600	TMEM164
314.2123247	0.15004771	0.300051493	0.500073199	0.617023536	0.99483168	ENSG00000135336	ORC3
316.6380396	0.008670401	0.235325556	0.036844281	0.970609166	0.999527751	ENSG00000160293	VAV2
313.3354878	-0.114219315	0.3396961	-0.336239702	0.736690116	0.999467288	ENSG00000115840	SLC25A12
316.3457432	-0.035466976	0.293459217	-0.120858279	0.903803288	0.999527751	ENSG00000121481	RNF2
317.5150869	0.190896497	0.289508724	0.659380811	0.509651261	0.993513219	ENSG00000164715	LMTK2
314.373888	-0.005203276	0.235452016	-0.022099092	0.982368911	0.999527751	ENSG00000117222	RBBP5
315.7296678	0.343927091	0.244480865	1.406764866	0.159497124	0.939986013	ENSG00000147251	DOCK11
316.065102	0.10388752	0.2545344	0.408147269	0.683165559	0.998869317	ENSG00000140199	SLC12A6
310.5708803	-0.427635518	0.400961859	-1.066524179	0.286186755	0.964826902	ENSG00000173581	CCDC106
313.586204	-0.116014008	0.229449621	-0.505618653	0.613124384	0.99483168	ENSG00000118096	IFT46
318.2804195	0.315113195	0.270765846	1.16378487	0.244511205	0.962465655	ENSG00000164284	GRPEL2
313.526386	0.124636787	0.222229025	0.560848374	0.574900907	0.99483168	ENSG00000157764	BRAF
312.7250556	-0.291739526	0.299494802	-0.974105476	0.330004171	0.976026345	ENSG00000148300	REXO4
315.0613262	0.002736976	0.235360389	0.011628873	0.990721711	0.999527751	ENSG00000111615	KRR1
314.107624	-0.123047598	0.239803747	-0.513117911	0.607868843	0.99483168	ENSG00000148296	SURF6
323.0969703	0.068413403	0.550056149	0.124375308	0.901018123	0.999527751	ENSG00000123243	ITIH5
311.6242304	-0.625290022	0.3032523	-2.061946508	0.039212829	0.793985738	ENSG00000174348	PODN
312.330066	-0.053346292	0.292085431	-0.182639347	0.855081009	0.999467288	ENSG00000184182	UBE2F
313.9228444	0.093796292	0.475821934	0.197124775	0.843729896	0.999467288	ENSG00000107317	PTGDS
314.2212202	0.276286284	0.272294857	1.014658474	0.310268655	0.973427483	ENSG00000108306	FBXL20
313.6658276	-0.248494198	0.305639278	-0.813030969	0.416200302	0.990226751	ENSG00000171813	PWWP2B
312.4368292	-0.176299296	0.29244524	-0.602845498	0.546611476	0.99483168	ENSG00000235437	LINC01278
314.0449477	-0.235461884	0.238283781	-0.98815741	0.323075563	0.975337095	ENSG00000181481	RNF135
307.0394179	-0.123416634	0.55270715	-0.223294803	0.823306072	0.999467288	ENSG00000109610	SOD3
310.2621632	-0.236399817	0.29504429	-0.801235017	0.422995602	0.991301167	ENSG00000120333	MRPS14
311.0634534	-0.313764474	0.237134129	-1.323151901	0.185784868	0.949094286	ENSG00000072195	SPEG
311.8638878	-0.031831959	0.307938516	-0.103371151	0.917668408	0.999527751	ENSG00000147383	NSDHL
313.2604753	-0.194543468	0.283490822	-0.686242563	0.492560162	0.993513219	ENSG00000173327	MAP3K11
310.5364703	-0.157417523	0.311013216	-0.506144161	0.612755451	0.99483168	ENSG00000109775	UFSP2
312.299497	0.055354153	0.415064165	0.133362881	0.8939064	0.999467288	ENSG00000171444	MCC
311.4482209	0.047975564	0.241245248	0.19886636	0.842367285	0.999467288	ENSG00000241258	CRCP
309.7570126	-0.849303814	0.535535051	-1.585897714	0.112762514	0.923922314	ENSG00000133048	CHI3L1
316.9580687	-0.083165266	0.407315205	-0.204179134	0.838213516	0.999467288	ENSG00000145362	ANK2
308.7729036	-0.084897885	0.319682041	-0.265569766	0.790570573	0.999467288	ENSG00000186577	SMIM29
311.5951986	-0.048344192	0.244398913	-0.197808541	0.843194863	0.999467288	ENSG00000131069	ACSS2
309.3705684	-0.048231595	0.286628931	-0.168271901	0.866369379	0.999467288	ENSG00000145247	OCIAD2
313.3787177	0.723582476	0.241960488	2.990498491	0.002785225	0.383114338	ENSG00000138613	APH1B
311.5602504	-0.092921092	0.343339388	-0.270639186	0.786668554	0.999467288	ENSG00000010610	CD4
310.3299995	0.081259924	0.266909566	0.3044474	0.76078706	0.999467288	ENSG00000153310	FAM49B
310.3450897	-0.020811791	0.369010917	-0.05639885	0.955024073	0.999527751	ENSG00000122873	CISD1
304.8269083	0.151241934	0.449270688	0.336638774	0.736389223	0.999467288	ENSG00000170577	SIX2
310.0969909	0.014602811	0.266176094	0.054861466	0.956248831	0.999527751	ENSG00000100324	TAB1
313.7577179	0.077673896	0.258064801	0.300986015	0.763425159	0.999467288	ENSG00000177485	ZBTB33
311.5868746	0.162694508	0.244836946	0.664501461	0.506369414	0.993513219	ENSG00000141452	RMC1
308.8094584	-0.110081839	0.300242192	-0.366643471	0.713884978	0.99900742	ENSG00000121964	GTDC1
313.6254602	0.254531324	0.257311417	0.989195607	0.322567447	0.975337095	ENSG00000135040	NAA35
311.4524743	-0.250114273	0.242810945	-1.030078249	0.302973275	0.972793733	ENSG00000219201	AC138392.1
309.0068237	-0.514238138	0.394473745	-1.303605485	0.19236813	0.949261383	ENSG00000152642	GPD1L
311.0848469	-0.210284256	0.290731612	-0.7232934	0.469499647	0.99135833	ENSG00000130511	SSBP4
310.9528011	-0.026190306	0.234637927	-0.111620087	0.911124644	0.999527751	ENSG00000197223	C1D
298.4894363	0.343019515	0.977811124	0.35080345	0.725735809	0.999467288	ENSG00000082482	KCNK2

310.9372867	0.248041718	0.290663909	0.85336263	0.393458238	0.988378925	ENSG00000143543	JTB	
312.8004025	0.007398948	0.240821974	0.030723726	0.97548987	0.999527751	ENSG00000144597	EAF1	
311.4018986	0.25033636	0.321345169	0.779026366	0.435964183	0.991301167	ENSG00000110172	CHORDC1	
310.4088348	-0.157801887	0.323530251	-0.48775002	0.625726917	0.99483168	ENSG00000211584	SLC48A1	
309.1766034	0.011582398	0.280341513	0.041315316	0.967044523	0.999527751	ENSG00000073803	MAP3K13	
312.7185993	0.881243916	0.282271537	3.121972287	0.001796438	0.302525475	ENSG00000132424	PNISR	
311.4332715	-0.113498853	0.26938939	-0.421318944	0.673522199	0.99853019	ENSG00000135541	AHI1	
313.2179187	0.282290192	0.24112601	1.170716473	0.241712762	0.962465655	ENSG00000135503	ACVR1B	
309.9580021	-0.213444934	0.348674902	-0.612160304	0.540431702	0.99483168	ENSG00000182749	PAQR7	
310.9609501	-0.362271044	0.284473986	-1.273476877	0.202848873	0.954310328	ENSG00000171161	ZNF672	
312.5743855	0.11023258	0.263736122	0.417965423	0.675972398	0.998869317	ENSG00000172795	DCP2	
308.8705768	0.33049258	0.444573621	0.74339224	0.457244241	0.99135833	ENSG00000170522	ELOVL6	
311.2626596	-0.288775599	0.281384176	-1.026268084	0.304765237	0.973427483	ENSG00000185000	DGAT1	
309.7581235	0.239393204	0.237292352	1.008853433	0.313044931	0.973427483	ENSG00000138641	HERC3	
305.9708158	0.014196449	0.352940087	0.040223396	0.967915025	0.999527751	ENSG00000137364	TPMT	
311.2197946	-0.196360896	0.268932786	-0.730148599	0.465299358	0.99135833	ENSG00000169740	ZNF32	
310.5599155	0.001185929	0.242661292	0.004887176	0.996100613	0.999527751	ENSG00000076248	UNG	
311.4539384	-0.351293579	0.239276182	-1.468151054	0.142063196	0.935644597	ENSG00000179981	TSHZ1	
313.382065	0.468986459	0.283374959	1.655003184	0.097923852	0.921147998	ENSG00000153922	CHD1	
309.2227837	-0.30994706	0.264797487	-1.170506048	0.241797382	0.962465655	ENSG00000136933	RABEPK	
308.8837767	0.035370363	0.27199778	0.13003916	0.896535444	0.999467288	ENSG00000126602	TRAP1	
306.3089005	0.827245376	1.0584193	0.781585687	0.434458101	0.991301167	ENSG00000117152	RGS4	
308.8664909	-0.180420084	0.300937064	-0.599527628	0.548821092	0.99483168	ENSG00000178385	PLEKHM3	
311.273345	0.3518972	0.341216379	1.031302195	0.302399129	0.972793733	ENSG00000033011	ALG1	
312.5740474	-0.028438413	0.266087384	-0.106876219	0.91488718	0.999527751	ENSG00000113851	CRBN	
306.084641	-0.275527334	0.343766288	-0.801496086	0.422844509	0.991301167	ENSG00000160888	IER2	
309.9522084	-0.683425793	0.402473175	-1.698065451	0.089495409	0.910413547	ENSG00000130758	MAP3K10	
308.4146218	0.460804585	0.284195	1.621438042	0.104923728	0.92169898	ENSG00000155158	TTC39B	
304.3266796	-0.994041789	1.431781744	-0.694269076	0.48751348	0.993513219	ENSG00000137745	MMP13	
310.3909467	-0.476631105	0.251742748	-1.89332606	0.058314522	0.852621724	ENSG00000177303	CASKIN2	
309.4487159	0.1660521	0.272894131	0.608485421	0.542865573	0.99483168	ENSG00000080802	CNOT4	
309.0307384	0.176745872	0.279716493	0.631875045	0.527468531	0.99483168	ENSG00000137713	PPP2R1B	
309.7244106	-0.079284584	0.263209964	-0.301221817	0.763245355	0.999467288	ENSG00000110871	COQ5	
309.2821569	-0.37634065	0.226951301	-1.658244079	0.097268211	0.921147998	ENSG00000105364	MRPL4	
311.5292001	0.308681371	0.260770545	1.183727903	0.23652079	0.962465655	ENSG00000272333	KMT2B	
309.6950042	-0.245390992	0.265712677	-0.923520078	0.355736242	0.978364583	ENSG00000253159	PCDHGA12	
312.1109683	0.322968395	0.284033758	1.137077498	0.25550589	0.962465655	ENSG00000075336	TIMM21	
305.8025095	-0.06977297	0.385134747	-0.181165088	0.856237997	0.999467288	ENSG00000067177	PHKA1	
311.7322637	0.238105616	0.293846494	0.810306133	0.417764252	0.990226751	ENSG00000078124	ACER3	
312.177719	0.067647229	0.281036176	0.24070648	0.809782617	0.999467288	ENSG00000103932	RPAP1	
311.2134379	0.606613147	0.37739413	1.607373032	0.107972562	0.923922314	ENSG00000148400	NOTCH1	
306.2789058	0.169484849	0.257819862	0.657377008	0.510938532	0.993513219	ENSG00000130921	C12orf65	
317.2133133	0.0710942	0.516712947	0.137589354	0.89056497	0.999467288	ENSG00000135111	TBX3	
306.854097	0.13556079	0.275563465	0.491940359	0.622761506	0.99483168	ENSG00000140848	CPNE2	
306.889095	0.052665423	0.345696882	0.15234567	0.878914305	0.999467288	ENSG00000168393	DTYMK	
308.4566911	0.063341142	0.234087846	0.27058706	0.786708649	0.999467288	ENSG00000103671	TRIP4	
308.1424154	-0.430163833	0.487779605	-0.881881549	0.377840866	0.982609538	ENSG00000173848	NET1	
307.0960455	-0.177606031	0.257437123	-0.689900622	0.490256685	0.993513219	ENSG00000198042	MAK16	
307.329111	0.097220207	0.258716013	0.375779629	0.707080734	0.99900742	ENSG00000104047	DTWD1	
310.0355875	-1.336125347	0.428085448	-3.121165073	0.00180137	0.302525475	ENSG00000184557	SOCS3	
309.0502217	-0.150851524	0.566458696	-0.266306308	0.790003316	0.999467288	ENSG00000131668	BARX1	
311.4889975	0.069610966	0.334744264	0.207952676	0.835265918	0.999467288	ENSG00000173276	ZBTB21	
304.638751	-0.389264346	0.3530418	-1.102601294	0.270200348	0.962465655	ENSG00000125877	ITPA	
307.5412161	0.228632646	0.369515852	0.61873569	0.536090494	0.99483168	ENSG00000163328	GPR155	
302.2520263	0.218411843	0.400217066	0.545733457	0.58524917	0.99483168	ENSG00000147119	CHST7	
310.1605036	-0.600380736	0.291338684	-2.060765593	0.039325408	0.793985738	ENSG00000104679	R3HCC1	
307.3681253	0.129390909	0.239239849	0.540841792	0.588616635	0.99483168	ENSG00000175110	MRPS22	
307.1106284	0.370851087	0.278924429	1.329575499	0.183658175	0.949094286	ENSG00000165494	PCF11	
312.7391064	-0.169622492	0.448149424	-0.378495393	0.705062616	0.99900742	ENSG00000125398	SOX9	
306.2093382	-0.091630428	0.252941851	-0.362258866	0.717158594	0.99900742	ENSG00000120159	CAAP1	
307.7981095	0.141260183	0.240123882	0.588280442	0.556344071	0.99483168	ENSG00000135775	COG2	
304.875534	0.373990531	0.254694173	1.468390608	0.141998151	0.935644597	ENSG00000136147	PHF11	
305.7930264	-0.094856403	0.271011508	-0.350008765	0.72633212	0.999467288	ENSG00000198276	UCKL1	
301.860776	0.508637945	0.544301438	0.934478416	0.350057165	0.977691382	ENSG00000144583		4-Mar

303.4008523	0.132619701	0.339152637	0.391032493	0.695773219	0.998869317	ENSG00000183527	PSMG1
308.3708873	-0.049341413	0.306191028	-0.161145848	0.87197853	0.999467288	ENSG00000123268	ATF1
305.0506337	-0.000175147	0.251566741	-0.000696226	0.999444492	0.9998586	ENSG00000138382	METTL5
308.327032	0.017063651	0.381432509	0.044735701	0.964317977	0.999527751	ENSG00000130844	ZNF331
307.2851343	1.705425434	1.212873956	1.406102775	0.159693609	0.940573556	ENSG00000111206	FOXM1
304.6654453	-0.393684916	0.243470731	-1.616970199	0.105884714	0.922095158	ENSG00000171421	MRPL36
303.8430723	0.038133319	0.247285891	0.15420742	0.877446192	0.999467288	ENSG00000136273	HUS1
304.9231653	-0.179608304	0.269180804	-0.667240386	0.504618598	0.993513219	ENSG00000181991	MRPS11
305.2954088	0.044670858	0.2791219	0.160040676	0.872849032	0.999467288	ENSG00000121900	TMEM54
304.0853557	0.018762332	0.245064826	0.076560691	0.938973031	0.999527751	ENSG00000094914	AAAS
300.4428455	0.166733964	0.321823513	0.518091306	0.604394566	0.99483168	ENSG00000015475	BID
304.726102	0.399659212	0.28618487	1.396507134	0.162561834	0.943223078	ENSG00000175893	ZDHHC21
303.214849	0.360026729	0.340035876	1.058790424	0.289695236	0.967638364	ENSG00000149269	PAK1
298.8489387	0.176985609	0.38216401	0.463114278	0.643282457	0.997097166	ENSG00000187391	MAGI2
306.1688604	0.501778605	0.319878635	1.568653076	0.116728798	0.923922314	ENSG00000198604	BAZ1A
303.7333678	-0.17449176	0.25997673	-0.671182225	0.502104451	0.993513219	ENSG00000076984	MAP2K7
304.1084524	-0.329530313	0.291822263	-1.129215809	0.258806806	0.962465655	ENSG00000125741	OPA3
300.3808628	0.16091427	0.433183797	0.371468812	0.710288382	0.99900742	ENSG00000153707	PTPRD
300.7098538	0.462153864	0.308488342	1.498124245	0.134100973	0.933741583	ENSG00000080823	MOK
303.8503254	-0.083510591	0.243471594	-0.342999319	0.731598973	0.999467288	ENSG00000139233	LLPH
301.8789487	0.313326545	0.373313252	0.839312675	0.401293872	0.988527641	ENSG00000049759	NEDD4L
303.3819437	-0.575015701	0.256028366	-2.245906227	0.024710015	0.749756093	ENSG00000064490	RFXANK
300.8462493	-0.249789757	0.317029903	-0.787905981	0.430751703	0.991301167	ENSG00000105618	PRPF31
305.874817	0.367419711	0.338239196	1.086271833	0.277358745	0.962465655	ENSG00000005059	MCUB
301.9548581	-0.057717541	0.249256314	-0.231558995	0.816880559	0.999467288	ENSG00000134058	CDK7
301.3607999	-0.402173293	0.333413868	-1.206228449	0.227729417	0.958865115	ENSG00000107960	STN1
304.3980857	-0.31988619	0.253704856	-1.260859547	0.207359455	0.954310328	ENSG00000166912	MTMR10
302.8152497	-0.219486512	0.261417762	-0.839600609	0.401132358	0.988492517	ENSG00000185608	MRPL40
305.0553465	-0.051794947	0.257977695	-0.200772966	0.840876102	0.999467288	ENSG00000162441	LZIC
305.265306	-0.09421817	0.262539482	-0.358872385	0.719690559	0.99900742	ENSG00000163719	MTMR14
305.5770141	-0.091533908	0.268471042	-0.340945181	0.733144851	0.999467288	ENSG00000117036	ETV3
298.6973569	0.15220775	0.515097054	0.295493343	0.767617037	0.999467288	ENSG00000134285	FKBP11
302.0861235	-0.033554003	0.246548623	-0.136094869	0.891746284	0.999467288	ENSG00000163481	RNF25
303.6089535	-0.068276601	0.251941722	-0.271001567	0.786389827	0.999467288	ENSG00000074047	GLI2
303.1340891	0.31034561	0.257062912	1.207274934	0.22732628	0.958865115	ENSG00000151353	TMEM18
302.2267408	-0.339497175	0.245548478	-1.382607528	0.166785239	0.943223078	ENSG00000145979	TBC1D7
301.0105188	-0.394664364	0.270970938	-1.456482258	0.145259351	0.935644597	ENSG00000090263	MRPS33
304.8704345	-0.195332066	0.284157641	-0.687407403	0.491826034	0.993513219	ENSG00000041880	PARP3
297.1462123	-0.540117345	0.365541343	-1.477582098	0.139519669	0.935644597	ENSG00000168101	NUDT16L1
307.9806231	-0.418767048	0.29717661	-1.409152115	0.158790194	0.939464191	ENSG00000182871	COL18A1
297.240355	-0.195200212	0.279824362	-0.697581191	0.485439148	0.992558339	ENSG00000144152	FBLN7
302.2034354	0.003531902	0.358052009	0.009864214	0.992129623	0.999527751	ENSG00000109103	UNC119
297.3387292	-0.154313338	0.41421517	-0.372543908	0.709487927	0.99900742	ENSG00000115350	POLE4
303.0976495	-0.045052014	0.232580753	-0.193704823	0.846407015	0.999467288	ENSG00000166199	ALKBH3
301.6198083	0.153807719	0.228810856	0.672204639	0.501453428	0.993513219	ENSG00000174485	DENND4A
304.5306106	0.063739177	0.300849981	0.211863656	0.832213406	0.999467288	ENSG00000048405	ZNF800
304.3985976	-0.080706308	0.353203942	-0.228497755	0.819259301	0.999467288	ENSG00000165983	PTER
303.4890781	-0.489080179	0.239914165	-2.038563162	0.041493645	0.800562177	ENSG00000159714	ZDHHC1
301.7328699	-0.113934341	0.244276871	-0.466414769	0.640918642	0.996553586	ENSG00000126464	PRR12
303.3501432	0.145932607	0.275807446	0.529110471	0.596728818	0.99483168	ENSG00000127995	CASD1
299.4745262	0.383223706	0.327805627	1.169057743	0.242380368	0.962465655	ENSG00000133858	ZFC3H1
303.3370967	-0.34868363	0.298228734	-1.169181874	0.242330363	0.962465655	ENSG00000244242	IFITM10
303.2126439	-0.269955959	0.588294434	-0.458878995	0.646321063	0.997368416	ENSG00000151617	EDNRA
302.2780713	0.00031785	0.336693343	0.000944035	0.999246769	0.99980056	ENSG00000059588	TARBP1
300.9539098	-0.352755334	0.268824634	-1.312213575	0.189448083	0.949094286	ENSG00000153774	CFDP1
300.3238927	-0.324164228	0.326969236	-0.991421187	0.321479959	0.975337095	ENSG00000168389	MFSD2A
312.6179243	-1.063329687	0.545857104	-1.948000087	0.051414953	0.842890082	ENSG00000069696	DRD4
303.3375655	0.4442286	0.285632138	1.555247263	0.119887192	0.923922314	ENSG00000166532	RIMKLB
302.7741634	-0.107224734	0.248226034	-0.431964095	0.665767509	0.997568624	ENSG00000142751	GPN2
306.545264	-1.019800032	0.363259713	-2.807357921	0.004994971	0.474620817	ENSG00000135636	DYSF
301.5982483	-0.03063926	0.250120081	-0.122498199	0.902504472	0.999527751	ENSG00000101019	UQCC1
300.7194828	1.082983895	0.509925332	2.123808777	0.033686137	0.775963115	ENSG00000146918	NCAPG2
299.3521988	-0.491204408	0.342586557	-1.433811099	0.151626177	0.938052926	ENSG00000166002	SMCO4
301.4038429	0.07439344	0.230804782	0.322321916	0.747208831	0.999467288	ENSG00000177409	SAMD9L

301.5142789	-0.446447681	0.446617348	-0.999620108	0.317494388	0.974768742	ENSG00000092068	SLC7A8
307.0919902	-0.936773863	0.327856667	-2.857266474	0.004273069	0.462224997	ENSG00000177374	HIC1
307.990102	-0.969792453	0.317311843	-3.056275627	0.002241051	0.344335164	ENSG00000215861	AC245297.1
300.3981673	-0.013763195	0.266119907	-0.05171802	0.958753379	0.999527751	ENSG00000080546	SESN1
299.1390415	-0.621731502	0.252261247	-2.464633429	0.013715349	0.672931175	ENSG00000166402	TUB
299.8349384	-0.245860419	0.301144915	-0.816418961	0.414260556	0.990226751	ENSG00000140382	HMG20A
296.8134552	0.104927691	0.398568594	0.263261313	0.792349176	0.999467288	ENSG00000180573	HIST1H2AC
299.6585716	-0.008045762	0.24044992	-0.03346128	0.973306743	0.999527751	ENSG00000108591	DRG2
297.7596509	-0.406296226	0.267826716	-1.517011567	0.129263764	0.928627271	ENSG00000130159	ECSIT
298.798021	-0.484696257	0.241987273	-2.002982436	0.045179174	0.813244956	ENSG00000128626	MRPS12
302.042418	-0.523222423	0.233717261	-2.238698252	0.025175556	0.755947112	ENSG00000143363	PRUNE1
300.3231367	0.064411361	0.259898515	0.247832739	0.804263823	0.999467288	ENSG00000175066	GK5
301.5713622	-0.110497545	0.353113923	-0.312923219	0.754338997	0.999467288	ENSG00000164576	SAP30L
305.5197489	0.134337967	0.331219597	0.405585805	0.685046961	0.998869317	ENSG00000171843	MLLT3
301.7692294	0.285085994	0.255495409	1.115816505	0.264500688	0.962465655	ENSG00000018189	RUFY3
301.4685364	-0.876437869	0.402858543	-2.175547434	0.029589118	0.773201806	ENSG00000025423	HSD17B6
299.5689949	0.002554837	0.261014636	0.009788099	0.992190352	0.999527751	ENSG00000141258	SGSM2
298.9211976	-0.106980739	0.261993785	-0.408333116	0.68302913	0.998869317	ENSG00000172732	MUS81
302.4850698	-0.077281956	0.293551232	-0.263265649	0.792345834	0.999467288	ENSG00000139173	TMEM117
298.8292727	0.206230461	0.314108225	0.656558615	0.511464767	0.993513219	ENSG00000145284	SCD5
296.5164525	0.437072738	0.352599624	1.239572331	0.21513362	0.954310328	ENSG00000177971	IMP3
302.8645976	-0.209640598	0.316299291	-0.662791868	0.507463862	0.993513219	ENSG00000196453	ZNF777
300.0688129	0.209028828	0.255976517	0.816593767	0.414160618	0.990226751	ENSG00000070269	TMEM260
298.5447356	-0.129021259	0.233897816	-0.551613782	0.581212992	0.99483168	ENSG00000182004	SNRPE
294.9030752	-0.0342812	0.401412443	-0.085401439	0.931942249	0.999527751	ENSG00000184178	SCFD2
296.7784366	-0.044610264	0.275294091	-0.162045845	0.871269751	0.999467288	ENSG00000104375	STK3
298.451627	0.037720395	0.242545339	0.155518945	0.87641222	0.999467288	ENSG00000169118	CSNK1G1
298.8781509	-0.114162754	0.244323951	-0.467259771	0.640314035	0.996553586	ENSG00000111875	ASF1A
295.3097815	0.192267941	0.313891029	0.612530859	0.540186588	0.99483168	ENSG00000164983	TMEM65
298.2633789	-0.301702046	0.358538112	-0.841478313	0.400080029	0.988492517	ENSG00000188026	RILPL1
296.5038048	0.076822242	0.378244989	0.203101811	0.839055456	0.999467288	ENSG00000121390	PSPC1
297.3367565	-0.318512625	0.292829834	-1.087705514	0.276725133	0.962465655	ENSG00000037241	RPL26L1
298.0098067	-0.337390144	0.230600689	-1.463092523	0.143442063	0.935644597	ENSG00000064999	ANKS1A
301.3378344	0.062873105	0.247106588	0.254437187	0.799157825	0.999467288	ENSG00000135999	EPC2
302.4426005	-0.030383575	0.332890185	-0.091272067	0.927276412	0.999527751	ENSG00000048028	USP28
300.2082199	0.047887835	0.266064163	0.179986041	0.857163527	0.999467288	ENSG00000088854	C20orf194
300.3632795	-0.265684654	0.266118787	-0.998368651	0.318100629	0.974768742	ENSG00000115947	ORC4
296.9949775	-0.050096219	0.244272233	-0.205083561	0.83750684	0.999467288	ENSG00000136908	DPM2
296.9143087	0.135327851	0.27521786	0.491711734	0.622923142	0.99483168	ENSG00000205903	ZNF316
296.6726036	0.117494387	0.259251593	0.453206036	0.650400386	0.997381078	ENSG00000104626	ERI1
303.2687028	0.153474254	0.305236277	0.502804764	0.615101544	0.99483168	ENSG00000251022	THAP9-AS1
298.8151203	-0.02706045	0.255870709	-0.105758297	0.915774126	0.999527751	ENSG00000104973	MED25
296.4512806	-0.04107501	0.248103807	-0.165555741	0.86850658	0.999467288	ENSG00000163832	ELP6
299.6007229	0.131364222	0.233059942	0.563649937	0.572992394	0.99483168	ENSG00000169762	TAPT1
301.2613873	-0.468330954	0.272859462	-1.716381581	0.086092226	0.901307093	ENSG00000005156	LIG3
294.6879821	-0.276160387	0.272907173	-1.011920589	0.311576024	0.973427483	ENSG00000107882	SUFU
300.2056809	0.856836801	0.363184435	2.35923327	0.01831274	0.726053908	ENSG00000225697	SLC26A6
293.1270866	0.001620452	0.311371935	0.005204231	0.995847643	0.999527751	ENSG00000101220	C20orf27
299.3944225	-0.028057419	0.300000609	-0.09352454	0.925486855	0.999527751	ENSG00000183354	KIAA2026
297.2183952	0.159485198	0.281415814	0.566724365	0.570901465	0.99483168	ENSG00000162642	C1orf52
298.7769058	-0.10961159	0.274698988	-0.399024367	0.68987525	0.998869317	ENSG00000162714	ZNF496
297.1187233	0.484038235	0.354936748	1.363730968	0.172652254	0.944422693	ENSG00000182963	GJC1
299.3633073	0.003057473	0.236898621	0.012906252	0.989702587	0.999527751	ENSG00000184708	EIF4ENIF1
293.3346711	0.125172643	0.312191093	0.400948796	0.688457823	0.998869317	ENSG00000158122	PRXL2C
299.1401012	-0.037503771	0.242760272	-0.154488915	0.877224251	0.999467288	ENSG00000130254	SAFB2
298.5679456	-0.070661842	0.253289584	-0.278976501	0.780262859	0.999467288	ENSG00000001167	NFYA
296.6680302	-0.260303301	0.26399379	-0.986020546	0.324123031	0.975459464	ENSG00000197798	FAM118B
299.1785043	0.257516884	0.316748145	0.813002026	0.416216896	0.990226751	ENSG00000132953	XPO4
298.2773251	-0.090423225	0.273982794	-0.330032494	0.74137541	0.999467288	ENSG00000196182	STK40
296.4683189	0.031725511	0.243570702	0.130251752	0.896367251	0.999467288	ENSG00000152465	NMT2
297.5792543	0.236046934	0.34298191	0.688219779	0.491314391	0.993513219	ENSG00000145734	BDP1
292.3035944	-0.599927459	0.326719901	-1.836213394	0.066326103	0.874040054	ENSG00000137288	UQCC2
295.4430287	-0.114533759	0.251481165	-0.455436729	0.648795077	0.997368416	ENSG00000174744	BRMS1
295.5788174	-0.1757051	0.257174003	-0.68321486	0.494471084	0.993513219	ENSG00000104312	RIPK2

293.5696654	0.175731219	0.355203365	0.494734105	0.620787824	0.99483168	ENSG00000115541	HSPE1
298.4265606	-0.632585564	0.372776662	-1.696955923	0.089704994	0.910413547	ENSG00000244716	BX679664.3
297.7809829	0.123315531	0.279123817	0.441795087	0.658637494	0.997568624	ENSG00000143952	VPS54
296.150774	0.294431316	0.266567238	1.104529267	0.269363615	0.962465655	ENSG00000146247	PHIP
300.3692952	-0.172369589	0.290957923	-0.592421019	0.553568695	0.99483168	ENSG00000171016	PYGO1
296.4158934	0.804147709	0.474332627	1.69532447	0.090013885	0.910413547	ENSG00000175197	DDIT3
296.7544162	-0.424439536	0.308252485	-1.376921703	0.168536457	0.943223078	ENSG00000177383	MAGEF1
297.484081	-0.276300392	0.293311577	-0.942003022	0.346191093	0.977691382	ENSG00000090975	PITPNM2
296.0628361	0.136513996	0.339061603	0.402622988	0.687225596	0.998869317	ENSG00000166477	LEO1
294.541356	0.27239054	0.250607733	1.086919934	0.277072196	0.962465655	ENSG00000160741	CRTC2
296.7693285	0.653541984	0.306306563	2.133620568	0.032873853	0.775963115	ENSG00000112159	MDN1
295.5760892	-0.456622036	0.253676543	-1.800016785	0.071857988	0.884210289	ENSG00000064995	TAF11
293.5930259	-0.09057684	0.264777044	-0.342087211	0.732285262	0.999467288	ENSG00000213015	ZNF580
297.9704229	0.519551635	0.310826338	1.671517407	0.094619523	0.921147998	ENSG00000168476	REEP4
297.8197521	0.160868875	0.29649441	0.542569674	0.587426124	0.99483168	ENSG00000166446	CDYL2
294.7247126	-0.156820288	0.244056267	-0.642557922	0.520510994	0.993513219	ENSG00000126749	EMG1
294.5099201	0.077505036	0.257315646	0.301206077	0.763257356	0.999467288	ENSG00000179941	BBS10
293.0537337	0.120069853	0.234178347	0.512728246	0.608141428	0.99483168	ENSG00000160305	DIP2A
296.3318588	0.079083903	0.239237606	0.330566352	0.740972066	0.999467288	ENSG00000137814	HAUS2
295.0787487	0.27429966	0.24193325	1.133782397	0.256885837	0.962465655	ENSG00000116580	GON4L
295.2286866	-0.027352934	0.234285919	-0.116750225	0.907057989	0.999527751	ENSG00000120688	WBP4
288.8821002	-0.003203873	0.437153066	-0.007328951	0.994152396	0.999527751	ENSG00000176697	BDNF
300.0687965	0.244321273	0.338608601	0.721544795	0.470574391	0.99135833	ENSG00000075407	ZNF37A
294.5080559	0.223781973	0.256966768	0.870859584	0.383830827	0.985655313	ENSG00000040487	PQLC2
295.3019572	-0.344131657	0.235547717	-1.460984895	0.144019587	0.935644597	ENSG00000144231	POLR2D
291.4600489	-0.159033777	0.322718553	-0.492794032	0.622158128	0.99483168	ENSG00000027001	MIPEP
293.7351995	-0.036972642	0.250711392	-0.147470931	0.882760322	0.999467288	ENSG00000122912	SLC25A16
298.2805877	-0.484912705	0.394499561	-1.229184397	0.219002677	0.956884001	ENSG00000158966	CACHD1
293.2064081	0.285714014	0.397718927	0.718381737	0.472521944	0.99135833	ENSG00000151883	PARP8
295.0898484	-0.073939081	0.253718139	-0.291422133	0.770728489	0.999467288	ENSG00000106524	ANKMY2
295.4618609	-0.08291701	0.273252548	-0.303444598	0.761551062	0.999467288	ENSG00000154767	XPC
289.5582744	-0.249000863	0.318506684	-0.781775943	0.434346261	0.991301167	ENSG00000235655	H3F3AP4
296.111284	0.078445547	0.321983999	0.243631817	0.807515984	0.999467288	ENSG00000169155	ZBTB43
291.9378791	-0.054595936	0.239700879	-0.227766941	0.819827428	0.999467288	ENSG00000011105	TSPAN9
291.9181614	-0.091023188	0.272946698	-0.333483384	0.73876943	0.999467288	ENSG00000138639	ARHGAP24
293.4558426	-0.106578874	0.263026108	-0.405202643	0.685328562	0.998869317	ENSG00000005469	CROT
290.0105187	-0.171118819	0.281121906	-0.608699696	0.542723509	0.99483168	ENSG00000170291	ELP5
290.6065607	2.559545113	1.644743911	1.55619674	0.119661316	0.923922314	ENSG00000171848	RRM2
292.2079493	-0.262220225	0.233239899	-1.124251149	0.260906496	0.962465655	ENSG00000084092	NOA1
289.9917587	-0.218250922	0.281924562	-0.774146533	0.438844142	0.991301167	ENSG00000124641	MED20
295.1560877	1.283187986	0.386467666	3.320298433	0.000899213	0.220124215	ENSG00000013441	CLK1
293.4170966	0.020578824	0.269224262	0.07643748	0.939071052	0.999527751	ENSG00000185414	MRPL30
289.6233412	-0.083299266	0.308646102	-0.269886013	0.787247948	0.999467288	ENSG00000115539	PDCL3
294.0252504	0.35137476	0.26344127	1.33378783	0.1822734	0.949094286	ENSG00000136811	ODF2
293.7757568	-0.23343878	0.246978375	-0.945179023	0.344567487	0.977691382	ENSG00000156502	SUPV3L1
291.9184156	0.39127254	0.296988885	1.317465265	0.187682717	0.949094286	ENSG00000138035	PNPT1
290.2438145	0.074403106	0.229538694	0.32414189	0.745830614	0.999467288	ENSG00000197548	ATG7
288.1137938	-0.129738662	0.279157937	-0.464750038	0.64211047	0.996740283	ENSG00000104886	PLEKHJ1
292.8689376	0.327514836	0.237849753	1.376982032	0.168517803	0.943223078	ENSG00000067533	RRP15
289.9816383	0.162298518	0.252428677	0.642948019	0.52025783	0.993513219	ENSG00000153015	CWC27
289.5273356	0.168849259	0.303800591	0.555789765	0.578354583	0.99483168	ENSG00000215712	TMEM242
291.4932179	-0.19164047	0.268185825	-0.714580909	0.474868045	0.99135833	ENSG00000130363	RSPH3
292.8621926	0.010178538	0.258310677	0.03940425	0.968568092	0.999527751	ENSG00000110046	ATG2A
291.5447519	0.417428111	0.340657563	1.225359882	0.220439655	0.956884001	ENSG00000170113	NIPA1
288.9551022	0.200476829	0.313909134	0.638646052	0.523053215	0.994616524	ENSG00000129680	MAP7D3
287.5463097	-0.212490431	0.367381481	-0.578391785	0.562999639	0.99483168	ENSG00000167272	POP5
290.5729851	0.088740329	0.237794346	0.373180987	0.709013746	0.99900742	ENSG00000140455	USP3
293.4197774	-0.186137936	0.267589332	-0.69561045	0.48667282	0.99350043	ENSG00000174749	FAM241A
293.3923627	0.292206665	0.289411103	1.009659482	0.312658461	0.973427483	ENSG00000172954	LCLAT1
290.625055	-0.076299774	0.260434467	-0.292971106	0.769544237	0.999467288	ENSG00000116670	MAD2L2
292.0386002	0.307826536	0.306247441	1.005156271	0.314821608	0.974768742	ENSG00000111581	NUP107
287.3248992	-0.056290019	0.306927805	-0.183398238	0.854485558	0.999467288	ENSG00000166510	CCDC68
291.2080044	-0.013566432	0.23514704	-0.057693399	0.953992852	0.999527751	ENSG00000148019	CEP78
292.3711745	0.382455609	0.26043927	1.468502079	0.141967892	0.935644597	ENSG00000197969	VPS13A

293.2261803	0.96359749	0.326311945	2.952994839	0.003147072	0.408098513	ENSG00000109920	FNBP4
291.1938186	-1.559164289	0.566568594	-2.75194267	0.005924289	0.499617804	ENSG00000124216	SNAI1
294.0356962	-0.037518798	0.342648283	-0.109496529	0.912808675	0.999527751	ENSG00000151746	BICD1
290.7607449	-0.139951337	0.307009074	-0.455854073	0.648494917	0.997368416	ENSG00000146063	TRIM41
288.5819317	-0.147093008	0.258347268	-0.569361577	0.569110787	0.99483168	ENSG00000122696	SLC25A51
290.0358954	0.357898117	0.274564649	1.303511277	0.192400269	0.949261383	ENSG00000023572	GLRX2
293.8310805	0.097554979	0.309520634	0.315180858	0.752624339	0.999467288	ENSG00000106086	PLEKHA8
292.0020246	-0.031290371	0.261244016	-0.119774498	0.904661784	0.999527751	ENSG00000133773	CCDC59
288.6427217	0.066651896	0.294084046	0.226642339	0.820701867	0.999467288	ENSG00000105556	MIER2
286.1007778	-0.20199383	0.265998151	-0.75938058	0.447624927	0.99135833	ENSG00000115317	HTRA2
292.6033127	-0.308713373	0.286219603	-1.078589201	0.280770898	0.963267117	ENSG00000223482	NUTM2A-AS1
287.5389896	1.283364124	1.246750757	1.02936703	0.303307236	0.972793733	ENSG00000141469	SLC14A1
288.8923415	-0.14883518	0.239861711	-0.620504118	0.534925944	0.99483168	ENSG00000164494	PDSS2
289.5481532	0.153710469	0.271519795	0.566111466	0.571318009	0.99483168	ENSG00000171202	TMEM126A
288.8966878	-0.100792578	0.233873846	-0.430969859	0.666490287	0.997568624	ENSG00000099284	H2AFY2
288.8877316	0.090088877	0.262558465	0.3431193	0.731508713	0.999467288	ENSG00000100354	TNRC6B
289.6419735	-0.456288447	0.268506616	-1.699356439	0.089252044	0.910413547	ENSG00000257704	INAFM1
286.8059412	0.262833455	0.279865539	0.939141906	0.347657891	0.977691382	ENSG00000060642	PIGV
287.4260116	-0.338268654	0.251005706	-1.347653245	0.177769936	0.9469453	ENSG00000226950	DANCR
286.5290508	0.015917226	0.272679131	0.058373467	0.953451149	0.999527751	ENSG00000197345	MRPL21
286.9174887	0.681826377	0.351213507	1.941344405	0.052216517	0.846840376	ENSG00000164105	SAP30
291.9483871	-0.401084857	0.3103598	-1.292322192	0.19624559	0.954310328	ENSG00000136141	LRCH1
289.4159012	0.173456613	0.245811723	0.705648256	0.480406907	0.99135833	ENSG00000126216	TUBGCP3
287.9890967	-0.055680378	0.267706301	-0.207990539	0.835236355	0.999467288	ENSG00000172315	TP53RK
288.3535993	0.144052371	0.266314707	0.540910313	0.588569402	0.99483168	ENSG00000188343	FAM92A
285.3259784	0.039611349	0.471938605	0.083933267	0.93310949	0.999527751	ENSG00000118418	HMGN3
287.3475442	-0.105917443	0.236817793	-0.447252891	0.654692476	0.997568624	ENSG00000178974	FBXO34
286.8840519	-0.279756308	0.280467499	-0.997464266	0.31853921	0.974768742	ENSG00000156017	CARNMT1
289.8143467	0.087803259	0.319531995	0.274787065	0.783479836	0.999467288	ENSG00000139436	GIT2
281.5864516	0.124108671	0.387044066	0.32065773	0.748469784	0.999467288	ENSG00000187653	TMSB4XP8
287.0297482	-0.165100966	0.316561353	-0.521544921	0.601987221	0.99483168	ENSG00000104228	TRIM35
282.8317657	0.707849433	0.371038239	1.907753321	0.056423106	0.847990559	ENSG00000111696	NT5DC3
286.9432133	-0.40378356	0.385756127	-1.046732718	0.295222869	0.970706863	ENSG00000182118	FAM89A
283.5934804	1.779820361	1.13113838	1.57347712	0.115608382	0.923922314	ENSG00000167900	TK1
283.632434	-0.15706529	0.303247086	-0.517944928	0.604496694	0.99483168	ENSG00000119711	ALDH6A1
288.9983361	0.20415953	0.313983324	0.650224119	0.515547463	0.993513219	ENSG00000189079	ARID2
287.6635525	-0.604626185	0.242380945	-2.494528537	0.012612458	0.660006295	ENSG00000067365	METTL22
288.8976849	-0.544411193	0.264646474	-2.057126187	0.039674089	0.793985738	ENSG00000197021	CXorf40B
278.2436234	0.044331382	0.474382997	0.093450614	0.925545582	0.999527751	ENSG00000187325	TAF9B
286.944749	0.032837547	0.278915395	0.117733004	0.906279215	0.999527751	ENSG00000064703	DDX20
287.8174468	0.402526666	0.247071432	1.629191461	0.103272494	0.92169898	ENSG00000198874	TYW1
284.6194286	-0.070264025	0.334045025	-0.210342977	0.833399994	0.999467288	ENSG00000141034	GID4
284.1474763	-0.017399496	0.238844684	-0.072848581	0.941926612	0.999527751	ENSG00000132405	TBC1D14
290.1585641	-0.10338268	0.360674967	-0.286636693	0.774390516	0.999467288	ENSG00000068024	HDAC4
284.0434597	-0.198168103	0.277565547	-0.713950652	0.475257695	0.99135833	ENSG00000198912	C1orf174
287.3586289	0.439942583	0.301346349	1.459923392	0.144311129	0.935644597	ENSG00000109689	STIM2
283.8436704	-0.042676549	0.240751395	-0.177263976	0.859301046	0.999467288	ENSG00000184207	PGP
283.4455523	-0.429623884	0.283325428	-1.516361898	0.129427868	0.928627271	ENSG00000118369	USP35
282.384579	-0.262807139	0.280149433	-0.938096275	0.348194936	0.977691382	ENSG00000155330	C16orf87
283.3068222	0.050540513	0.312109336	0.161932078	0.871359342	0.999467288	ENSG00000198721	ECI2
283.7365605	-0.431430596	0.342778465	-1.258628065	0.208164705	0.954310328	ENSG00000166189	HPS6
285.7623679	-0.039219946	0.269839113	-0.145345668	0.88443796	0.999467288	ENSG00000111642	CHD4
284.1050377	0.406424457	0.290617721	1.398484771	0.161967543	0.943223078	ENSG00000106344	RBM28
281.7072296	0.09511436	0.327090544	0.290789086	0.771212633	0.999467288	ENSG00000126561	STAT5A
281.728074	0.123672996	0.409442655	0.302052058	0.762612379	0.999467288	ENSG00000197077	KIAA1671
283.4088044	0.746268082	0.263073519	2.836728242	0.004557839	0.470206187	ENSG00000166801	FAM111A
282.0130386	-0.065208185	0.344038758	-0.189537324	0.849671705	0.999467288	ENSG00000119705	SLIRP
285.7831538	-0.740416065	0.303369814	-2.440638556	0.014661321	0.686410066	ENSG00000107902	LHPP
286.4168895	0.021394964	0.33281612	0.064284638	0.948743585	0.999527751	ENSG00000078699	CBFA2T2
284.7259353	0.009413153	0.245725985	0.038307521	0.969442494	0.999527751	ENSG00000205758	CRYZL1
285.4371134	-0.456314105	0.268191048	-1.701451666	0.088858207	0.910413547	ENSG00000102302	FGD1
285.1785922	-0.177996949	0.916260156	-0.19426464	0.845968671	0.999467288	ENSG00000265107	GJA5
285.9528451	-0.039477789	0.336029797	-0.117483002	0.906477313	0.999527751	ENSG00000231074	HCG18
282.4242448	0.149854889	0.252062035	0.594515906	0.552167107	0.99483168	ENSG00000122674	CCZ1

283.3784039	0.28912338	0.292166652	0.989583781	0.322377601	0.975337095	ENSG00000101464	PIGU
284.4212222	0.127336609	0.237356309	0.536478722	0.591627738	0.99483168	ENSG00000115827	DCAF17
283.6253473	0.286166671	0.299868288	0.954307884	0.339927819	0.977691382	ENSG00000063601	MTMR1
284.3075203	0.291052795	0.278589933	1.044735509	0.296145228	0.970706863	ENSG00000204130	RUFY2
278.7894	-0.539955187	0.353308358	-1.528283083	0.126442268	0.928627271	ENSG00000170906	NDUFA3
282.1664202	-0.154665116	0.250765436	-0.616772064	0.537385081	0.99483168	ENSG00000204304	PBX2
282.7435697	-0.264131501	0.24679261	-1.070256931	0.284503675	0.963310634	ENSG00000117697	NSL1
284.4656219	0.276872561	0.257701625	1.074391989	0.282647032	0.963267117	ENSG00000145476	CYP4V2
280.59896	-0.178671785	0.328475132	-0.543943109	0.586480622	0.99483168	ENSG00000171311	EXOSC1
278.0555608	0.187598324	0.361699717	0.518657647	0.603999501	0.99483168	ENSG00000103047	TANGO6
281.8391222	0.288527769	0.271164959	1.064030437	0.287314911	0.965284229	ENSG00000125447	GGA3
284.867574	0.33779763	0.296086842	1.140873493	0.253922572	0.962465655	ENSG00000104885	DOT1L
280.9429824	0.017307929	0.251351196	0.068859545	0.94510142	0.999527751	ENSG00000079277	MKNK1
285.8696913	0.151692964	0.266598478	0.568994111	0.569360136	0.99483168	ENSG00000065413	ANKRD44
281.0797498	-0.048250293	0.282383935	-0.170867696	0.864327798	0.999467288	ENSG00000166275	BORCS7
282.8823012	0.2439423	0.262580128	0.929020416	0.352878506	0.977691382	ENSG00000172262	ZNF131
282.2042116	0.391189408	0.27764276	1.408966714	0.158845011	0.939464191	ENSG00000198740	ZNF652
282.8776967	0.221498252	0.298067211	0.743115123	0.457411984	0.99135833	ENSG00000103227	LMF1
278.5380749	-0.149977114	0.390241967	-0.38431826	0.700742575	0.998869317	ENSG00000167508	MVD
282.7848268	0.436217039	0.29223695	1.492682697	0.135520265	0.934144427	ENSG00000008869	HEATR5B
282.1874977	-0.335941772	0.237611607	-1.413827279	0.157412622	0.939464191	ENSG00000150990	DHX37
279.5124272	-0.057814339	0.273664918	-0.211259592	0.832684712	0.999467288	ENSG00000212719	C17orf51
281.7913861	0.241317464	0.270259552	0.892910029	0.371905356	0.981955904	ENSG00000163214	DHX57
280.1848558	-0.192762075	0.246558097	-0.781811985	0.434325076	0.991301167	ENSG00000181467	RAP2B
286.4012333	0.069470847	0.533206715	0.13028877	0.896337965	0.999467288	ENSG00000128342	LIF
282.0344486	0.046485228	0.239764533	0.193878668	0.846270888	0.999467288	ENSG00000108799	EZH1
283.053585	0.055024393	0.320588247	0.171635715	0.863723927	0.999467288	ENSG00000101040	ZMYND8
277.1475411	-0.086384798	0.395282652	-0.21853931	0.827008934	0.999467288	ENSG00000006453	BAIAP2L1
280.921094	-0.283391413	0.241924974	-1.171402061	0.241437204	0.962465655	ENSG00000166411	IDH3A
278.4526536	-0.250098449	0.302001505	-0.828136432	0.407593239	0.990226751	ENSG00000141101	NOB1
278.7284662	-0.089950466	0.257237438	-0.349678749	0.726579804	0.999467288	ENSG00000156928	MALSU1
281.9071005	-0.051295778	0.25562495	-0.200668118	0.840958091	0.999467288	ENSG00000128513	POT1
281.9561954	-0.275549715	0.262691756	-1.048946946	0.294202537	0.970706863	ENSG00000109466	KLHL2
282.1300669	0.049720924	0.260016098	0.191222483	0.84835129	0.999467288	ENSG00000113318	MSH3
273.7356211	0.015070238	0.318541136	0.047310179	0.962266016	0.999527751	ENSG00000126803	HSPA2
279.4153385	0.246869663	0.28943737	0.852929472	0.393698417	0.988378925	ENSG00000109016	DHRS7B
277.8000919	-0.216401211	0.359845962	-0.601371793	0.547592381	0.99483168	ENSG00000151376	ME3
281.7844872	0.388384347	0.264634276	1.467626768	0.142205633	0.935644597	ENSG00000129317	PUS7L
280.7307852	0.051044847	0.257064789	0.198568022	0.84260067	0.999467288	ENSG00000112855	HARS2
275.5695418	0.414927475	0.432009451	0.960459253	0.336824129	0.977691382	ENSG00000228716	DHFR
281.6004067	-0.03123895	0.246137502	-0.126916662	0.899006359	0.999527751	ENSG00000174405	LIG4
278.5256027	0.434363252	0.329696104	1.31746553	0.187682628	0.949094286	ENSG00000001561	ENPP4
286.4760548	0.392221331	0.541793456	0.723931467	0.469107811	0.99135833	ENSG00000049192	ADAMTS6
276.4736461	-0.391356941	0.271702438	-1.440388037	0.149757646	0.936396974	ENSG00000169188	APEX2
282.1013782	-0.19281152	0.287283121	-0.671155059	0.502121755	0.993513219	ENSG00000127804	METTL16
279.9638458	-0.132243804	0.278243324	-0.475281138	0.634586601	0.995506752	ENSG00000011376	LARS2
280.1248355	0.157960216	0.234725479	0.672957265	0.500974479	0.993513219	ENSG00000113597	TRAPPC13
275.5102828	-0.050034365	0.32946491	-0.151865535	0.879292992	0.999467288	ENSG00000104976	SNAPC2
279.2538726	0.113127584	0.298969348	0.378391914	0.705139474	0.99900742	ENSG00000091592	NLRP1
281.9341909	-0.641711665	0.352347415	-1.821246978	0.068569322	0.87420149	ENSG00000169515	CCDC8
275.0808953	-0.442435406	0.301340878	-1.46822233	0.142043841	0.935644597	ENSG00000138495	COX17
275.7067995	-0.342352186	0.338449122	-1.011532205	0.311761775	0.973427483	ENSG00000111653	ING4
277.3802843	-0.30504228	0.307947921	-0.990564507	0.321898276	0.975337095	ENSG00000140320	BAHD1
282.5752905	-0.345279732	1.354249703	-0.254960168	0.798753864	0.999467288	ENSG00000061656	SPAG4
280.2902622	-0.01955564	0.275175606	-0.07106604	0.943345196	0.999527751	ENSG00000100979	PLTP
278.7280022	-0.317334464	0.354818459	-0.894357256	0.371130775	0.981955904	ENSG00000108001	EBF3
283.3884707	-0.360294422	0.525163812	-0.686061023	0.492674628	0.993513219	ENSG00000169136	ATF5
277.6859462	0.169199785	0.254610373	0.664543961	0.506342222	0.993513219	ENSG00000147050	KDM6A
286.2776539	0.459128101	0.416773943	1.101623816	0.27062525	0.962465655	ENSG00000006459	KDM7A
278.0409633	-0.058498487	0.326830539	-0.178987212	0.857947743	0.999467288	ENSG00000151229	SLC2A13
281.4472292	-0.877683161	0.411076538	-2.135084541	0.032754105	0.775963115	ENSG00000139946	PELI2
273.628342	-1.75259753	1.104019962	-1.587469059	0.112406449	0.923922314	ENSG00000130751	NPAS1
277.4675164	0.064440035	0.245600201	0.262377778	0.793030203	0.999467288	ENSG00000152904	GGPS1
279.1221552	-0.336842642	0.367359545	-0.916929061	0.359179815	0.979465649	ENSG00000161921	CXCL16

274.5559616	-0.112570795	0.346184205	-0.325176001	0.745047871	0.999467288	ENSG00000168779	SHOX2
279.4518914	0.135437368	0.274932755	0.492619977	0.62228113	0.99483168	ENSG00000086827	ZW10
275.0705455	0.088839039	0.250261068	0.354985454	0.722600488	0.999092366	ENSG00000169241	SLC50A1
273.9842776	0.281184286	0.315239262	0.891971022	0.372408465	0.982230727	ENSG00000138496	PARP9
274.8282535	-0.208119203	0.286575169	-0.726229016	0.467698387	0.99135833	ENSG00000162910	MRPL55
272.244105	-0.334087725	0.306080084	-1.09150429	0.275051045	0.962465655	ENSG00000198355	PIM3
274.7778429	-0.186590464	0.255879074	-0.729213456	0.465871101	0.99135833	ENSG00000109390	NDUFC1
274.4129526	-0.35307304	0.278046687	-1.269833652	0.204143891	0.954310328	ENSG00000112304	ACOT13
276.0968933	1.443358787	0.957629101	1.5072211	0.131753994	0.931891739	ENSG00000084674	APOB
277.7373943	0.223599359	0.291418617	0.76727891	0.442915705	0.99135833	ENSG00000272886	DCP1A
279.1869214	-0.098349452	0.248679346	-0.395487013	0.692483504	0.998869317	ENSG00000132406	TMEM128
275.5896648	0.013349692	0.291496213	0.045797136	0.963471942	0.999527751	ENSG00000086544	ITPKC
275.3588637	0.0271956	0.27304825	0.09959998	0.920661909	0.999527751	ENSG00000143493	INTS7
279.8322978	0.952650875	0.403344929	2.361876417	0.018182702	0.726053908	ENSG00000114857	NKTR
276.3193877	0.229414274	0.255659982	0.897341351	0.369536798	0.981455968	ENSG00000162065	TBC1D24
276.7054453	-0.467818281	0.267448775	-1.749188349	0.080258467	0.897807954	ENSG00000075240	GRAMD4
271.6086664	-0.504297757	0.416972904	-1.209425726	0.22649933	0.958865115	ENSG00000130005	GAMT
274.9175349	-0.381618356	0.270053643	-1.413120562	0.157620278	0.939464191	ENSG00000068354	TBC1D25
278.3457273	-0.411966127	1.05557401	-0.390276876	0.696331821	0.998869317	ENSG00000111341	MGP
275.7840841	-0.108459096	0.325900683	-0.332798002	0.739286766	0.999467288	ENSG00000175938	ORAI3
273.4600166	-0.738522598	0.32176821	-2.295200631	0.021721629	0.73997555	ENSG00000174516	PELI3
272.2424254	0.150027729	0.411669645	0.364437191	0.715531568	0.99900742	ENSG00000170571	EMB
273.5367067	0.047378513	0.313981787	0.150895736	0.880057964	0.999467288	ENSG00000243279	PRAF2
275.9900499	-0.051943949	1.069427588	-0.048571731	0.961260599	0.999527751	ENSG00000164106	SCRG1
277.0535098	-0.038318343	0.319783965	-0.119825719	0.904621208	0.999527751	ENSG00000187601	MAGEH1
275.5710436	-0.186852934	0.246794623	-0.757119146	0.448978485	0.99135833	ENSG00000132434	LANCL2
277.5365723	0.025778938	0.276454041	0.09324855	0.925706105	0.999527751	ENSG00000151332	MBIP
271.5626645	-0.144527078	0.294942901	-0.490017146	0.624121766	0.99483168	ENSG00000127952	STYXL1
276.4962279	-0.009544633	0.281973213	-0.033849431	0.972997218	0.999527751	ENSG00000123607	TTC21B
278.2868812	-0.795263446	1.216003568	-0.653997626	0.513113335	0.993513219	ENSG00000173227	SYT12
272.5724431	-0.328080462	0.294999738	-1.112138145	0.266078758	0.962465655	ENSG00000130332	LSM7
274.4339631	-0.059482632	0.247787224	-0.240055281	0.810287401	0.999467288	ENSG00000136878	USP20
277.085841	0.252064536	0.270803209	0.930803356	0.351955294	0.977691382	ENSG00000177302	TOP3A
274.4333172	0.447258409	0.320015276	1.397615812	0.162228468	0.943223078	ENSG00000120800	UTP20
274.6702098	0.095349579	0.235329405	0.405174945	0.685348921	0.998869317	ENSG00000168813	ZNF507
271.9512815	-0.258186126	0.272411817	-0.947778729	0.343242111	0.977691382	ENSG00000169093	ASMTL
281.3325651	0.285885361	0.527629898	0.541829343	0.587936075	0.99483168	ENSG00000165029	ABCA1
270.9502853	0.042297763	0.315619023	0.134015252	0.893390514	0.999467288	ENSG00000166452	AKIP1
275.6951029	0.367508425	0.260278558	1.411981174	0.157955505	0.939464191	ENSG00000186625	KATNA1
274.4109845	0.246779329	0.298073609	0.827914051	0.407719177	0.990226751	ENSG00000125450	NUP85
276.6206794	-0.037034088	0.298997465	-0.123860877	0.90142543	0.999527751	ENSG00000165118	C9orf64
271.232476	-0.234756576	0.302870498	-0.775105459	0.438277346	0.991301167	ENSG00000162522	KIAA1522
271.2957128	-0.421894086	0.29135128	-1.448059832	0.147600318	0.936396974	ENSG00000137802	MAPKBP1
270.33086	-0.474704659	0.298525307	-1.590165547	0.111797494	0.923922314	ENSG00000005882	PDK2
275.4220128	0.082909555	0.250824643	0.330547883	0.740986018	0.999467288	ENSG00000138686	BBS7
273.6299635	-0.879715457	0.386075353	-2.278610769	0.022690213	0.744905651	ENSG00000102935	ZNF423
273.0745281	0.227872428	0.247741022	0.919800952	0.357676786	0.979427314	ENSG00000142655	PEX14
270.6985912	0.438192744	0.419763255	1.043904484	0.296529583	0.970710964	ENSG00000198189	HSD17B11
274.325822	-0.595547661	0.254248978	-2.34237976	0.019161207	0.728277138	ENSG00000083457	ITGAE
272.1140053	0.374064636	0.264312204	1.415237853	0.156998769	0.939464191	ENSG00000159596	TMEM69
275.3730511	-0.562924119	0.344880771	-1.632228195	0.102631423	0.92169898	ENSG00000136149	RPL13AP25
272.7671729	0.027750726	0.273202106	0.101575814	0.919093378	0.999527751	ENSG00000140463	BBS4
273.0195996	-0.291958118	0.301288701	-0.969031091	0.332529677	0.976026345	ENSG00000123415	SMUG1
273.4855775	-0.383847727	0.263522932	-1.45660085	0.145226593	0.935644597	ENSG00000178498	DTX3
271.3689223	-0.187584116	0.275833818	-0.680062064	0.496465164	0.993513219	ENSG00000115286	NDUFS7
273.1624954	0.039075522	0.256724028	0.152208277	0.879022666	0.999467288	ENSG00000135297	MTO1
274.3895773	0.399021526	0.319288758	1.249719937	0.211401872	0.954310328	ENSG00000104093	DMXL2
273.7244234	1.464628923	1.265984781	1.156908792	0.247309626	0.962465655	ENSG00000145386	CCNA2
274.7418341	-0.254363123	0.31748464	-0.801182454	0.423026027	0.991301167	ENSG00000214389	RPS3AP26
270.4558192	0.11794171	0.300509899	0.392471963	0.694709523	0.998869317	ENSG00000170802	FOXN2
272.0265249	-0.041842438	0.306188598	-0.136655769	0.891302893	0.999467288	ENSG00000183624	HMCES
273.2965483	0.14002561	0.248686891	0.563059875	0.573394113	0.99483168	ENSG00000138767	CNOT6L
274.6564074	-0.117796795	0.305253742	-0.385897956	0.699572244	0.998869317	ENSG00000159388	BTG2
268.8616678	-0.198320008	0.321587195	-0.616691246	0.537438397	0.99483168	ENSG00000138640	FAM13A

266.3339919	0.56266993	0.603213877	0.932786779	0.350930069	0.977691382	ENSG00000163584	RPL22L1
273.1848031	-0.008631796	0.286063099	-0.030174449	0.975927926	0.999527751	ENSG00000185024	BRF1
272.3705458	-0.138974617	0.285192106	-0.487301768	0.626044495	0.99483168	ENSG00000165943	MOAP1
268.6984663	0.320794799	0.303989683	1.055281864	0.291296433	0.969851196	ENSG00000182287	AP1S2
275.7288538	0.249576404	0.37988282	0.656982603	0.511192103	0.993513219	ENSG00000171606	ZNF274
268.6290953	-0.241321803	0.303244701	-0.79579891	0.426148925	0.991301167	ENSG00000243147	MRPL33
273.0371691	-0.376916478	0.269957569	-1.396206373	0.162652358	0.943223078	ENSG00000158773	USF1
272.6673099	0.165840898	0.292965565	0.56607642	0.571341832	0.99483168	ENSG00000227372	TP73-AS1
269.4910302	-0.504236169	0.249373488	-2.022011932	0.043175119	0.806698895	ENSG00000160688	FLAD1
270.9621103	0.213861807	0.264306351	0.809143655	0.41843252	0.990226751	ENSG00000173193	PARP14
270.369502	0.191186557	0.341528598	0.559796626	0.575618165	0.99483168	ENSG00000116667	C1orf21
269.0971161	0.044364867	0.280885226	0.157946602	0.874498876	0.999467288	ENSG00000116455	WDR77
265.1869676	0.420453025	0.440404665	0.954697029	0.339730933	0.977691382	ENSG00000163251	FZD5
273.2053449	0.044588574	0.313974248	0.142013475	0.887069362	0.999467288	ENSG00000181220	ZNF746
271.9468744	0.173294936	0.259448332	0.667936211	0.504174312	0.993513219	ENSG00000197312	DDI2
277.1002035	-0.374391405	1.206705325	-0.310259181	0.75636387	0.999467288	ENSG00000151640	DPYSL4
271.7083664	-0.232514657	0.2500689	-0.929802375	0.352473416	0.977691382	ENSG00000242498	ARPIN
272.6280594	-0.454408342	0.338043245	-1.344231394	0.178873573	0.9469453	ENSG00000221926	TRIM16
269.3793692	-0.079008843	0.257485359	-0.306847904	0.758959144	0.999467288	ENSG00000058600	POLR3E
267.0643311	-0.179442655	0.334418704	-0.536580797	0.591557211	0.99483168	ENSG00000154864	PIEZO2
270.5699756	-0.303177994	0.290663143	-1.043056205	0.296922263	0.971556012	ENSG00000008853	RHOBTB2
272.3346855	-0.219127235	0.258170416	-0.848769733	0.396009435	0.988378925	ENSG00000196922	ZNF252P
270.4211357	0.326898683	0.247479699	1.320911104	0.186531007	0.949094286	ENSG00000126070	AGO3
270.8478339	0.074651816	0.263041782	0.283802122	0.776562029	0.999467288	ENSG00000171792	RHNO1
270.4667149	-0.220691917	0.261071624	-0.84533092	0.397926102	0.988378925	ENSG00000172974	AC007318.1
270.7647888	0.022724045	0.516757934	0.043974255	0.964924926	0.999527751	ENSG00000173705	SUSD5
273.4713555	0.073231574	0.266325063	0.274970642	0.783338793	0.999467288	ENSG00000174989	FBXW8
270.0498724	0.078971511	0.230697337	0.342316526	0.7321127	0.999467288	ENSG00000138433	CIR1
271.7379272	0.112438259	0.228251022	0.49260791	0.622289658	0.99483168	ENSG00000205413	SAMD9
266.4118108	0.128454789	0.407939379	0.314886956	0.752847486	0.999467288	ENSG00000153944	MSI2
269.8361448	-0.504578562	0.456429094	-1.105491672	0.268946601	0.962465655	ENSG00000180875	GREM2
266.3854441	-0.46052065	0.399410837	-1.152999889	0.24891043	0.962465655	ENSG00000139174	PRICKLE1
272.3928341	0.473986868	0.323949569	1.463150173	0.143426291	0.935644597	ENSG00000170949	ZNF160
270.1639098	-0.028619102	0.255176917	-0.112153962	0.910701332	0.999527751	ENSG00000089060	SLC8B1
272.7988543	0.234520622	0.279021911	0.840509699	0.400622666	0.988492517	ENSG00000174606	ANGEL2
269.1688841	0.49932114	0.52840615	0.944957094	0.344680781	0.977691382	ENSG00000113448	PDE4D
265.7516712	0.476571163	0.256536897	1.857710018	0.063210205	0.868202292	ENSG00000151474	FRMD4A
265.7367682	0.41313679	0.335776069	1.230393788	0.218549679	0.956884001	ENSG00000029153	ARNTL2
270.8527697	0.288124992	0.282982154	1.018173716	0.308595408	0.973427483	ENSG00000129566	TEP1
271.9772097	0.177357241	0.274048121	0.64717554	0.51751834	0.993513219	ENSG00000275835	TUBGCP5
266.8652358	-0.165483854	0.272621369	-0.607009841	0.543844381	0.99483168	ENSG00000101473	ACOT8
268.8957329	-0.021190466	0.261718963	-0.080966491	0.935468601	0.999527751	ENSG00000107669	ATE1
266.6422064	0.098081911	0.251825677	0.389483362	0.696918616	0.998869317	ENSG00000163686	ABHD6
268.2260414	-0.22510121	0.305633226	-0.736507653	0.461421812	0.99135833	ENSG00000171943	SRGAP2C
269.7681892	0.0664428	0.268090358	0.247837336	0.804260266	0.999467288	ENSG00000163510	CWC22
269.589961	0.578859177	0.240800476	2.403895485	0.01622141	0.698816035	ENSG00000165704	HPRT1
268.4192448	2.112857009	1.417047025	1.491028153	0.135954104	0.934144427	ENSG00000170312	CDK1
265.4967945	-0.077796893	0.355200319	-0.219022587	0.826632453	0.999467288	ENSG00000105204	DYRK1B
270.3314646	-0.055350649	0.2832025	-0.195445482	0.845044213	0.999467288	ENSG00000167182	SP2
268.8646033	-0.259335243	0.278894276	-0.929869363	0.352438727	0.977691382	ENSG00000079974	RABL2B
270.8212879	0.292766125	0.332886644	0.879476934	0.379142734	0.982778604	ENSG00000172071	EIF2AK3
266.8472732	-0.193537779	0.243993564	-0.79320854	0.427656333	0.991301167	ENSG00000056050	HPF1
265.8049625	-0.259635589	0.281136804	-0.923520455	0.355736046	0.978364583	ENSG00000161654	LSM12
270.1027667	0.314098786	0.241849627	1.298735868	0.19403459	0.950624691	ENSG00000179454	KLHL28
270.0850536	0.129064083	0.380473154	0.339219946	0.734444045	0.999467288	ENSG00000101680	LAMA1
270.3356513	0.463692189	0.250048609	1.854408195	0.063680783	0.868202292	ENSG00000101751	POLI
268.1275848	-0.421683945	0.292839864	-1.43998136	0.149872673	0.936396974	ENSG00000269190	FBXO17
286.095404	0.294478258	0.912196976	0.322823102	0.746829216	0.999467288	ENSG00000187605	TET3
267.5775807	-0.064885363	0.258599674	-0.250910461	0.801883336	0.999467288	ENSG00000204104	TRAF3IP1
267.3828332	-0.110866062	0.250879817	-0.441909052	0.65855502	0.997568624	ENSG00000121486	TRMT1L
265.0080924	-0.126954055	0.293792153	-0.432122007	0.665652741	0.997568624	ENSG00000145781	COMMD10
270.8797175	-0.001098915	0.41542043	-0.002645308	0.997889352	0.999527751	ENSG00000188522	FAM83G
266.3544159	-0.087537509	0.305513145	-0.286526163	0.774475158	0.999467288	ENSG00000133193	FAM104A
268.7686851	-0.079100377	0.248207496	-0.318686496	0.749964256	0.999467288	ENSG00000005100	DHX33

268.6174512	-0.190865155	0.255607084	-0.746713086	0.455236769	0.99135833	ENSG00000142039	CCDC97
267.8371487	-0.369404446	0.297337946	-1.242372359	0.214099209	0.954310328	ENSG00000119574	ZBTB45
269.0496207	-0.568512282	0.355219453	-1.600453685	0.109497974	0.923922314	ENSG00000167487	KLHL26
269.7064984	0.061519315	0.26391088	0.2331064	0.815678785	0.999467288	ENSG00000035115	SH3YL1
269.4197978	-0.074610854	0.308938284	-0.24150731	0.809161951	0.999467288	ENSG00000155256	ZFYVE27
267.8457932	2.647682174	1.344290075	1.969576525	0.048886924	0.83480477	ENSG00000112984	KIF20A
267.1222961	0.038912263	0.259930786	0.149702402	0.880999413	0.999467288	ENSG00000001497	LAS1L
271.7443663	-0.283464528	0.330750028	-0.857035538	0.391425244	0.988355932	ENSG00000158079	PTPDC1
266.8224461	-0.054893639	0.251007784	-0.218692977	0.82688922	0.999467288	ENSG00000006530	AGK
270.053694	-0.007122723	0.316532335	-0.022502355	0.982047233	0.999527751	ENSG00000119522	DENND1A
268.1752832	0.078144047	0.314434589	0.248522426	0.803730221	0.999467288	ENSG00000072756	TRNT1
265.4687376	0.146723494	0.318521284	0.460639528	0.645057248	0.997368416	ENSG00000164463	CREBRF
268.6834273	0.141714837	0.252293794	0.561705603	0.574316617	0.99483168	ENSG00000125863	MKKS
267.3767487	0.282331852	0.244988611	1.152428477	0.249145045	0.962465655	ENSG00000165138	ANKS6
268.9365418	0.021409581	0.295019032	0.07257017	0.942148165	0.999527751	ENSG00000187535	IFT140
264.9425062	-0.622795719	0.333241735	-1.868900723	0.061636628	0.865910462	ENSG00000114383	TUSC2
268.338107	-0.278841973	0.239050803	-1.166454869	0.243430585	0.962465655	ENSG00000105705	SUGP1
264.4313826	-0.813372618	0.349597095	-2.32660005	0.019986562	0.73975641	ENSG00000196465	MYL6B
267.7086625	-0.086982207	0.265810449	-0.327233964	0.743490934	0.999467288	ENSG00000101132	PFDN4
267.2841252	-0.228539651	0.234246551	-0.975637206	0.32924428	0.976026345	ENSG00000250317	SMIM20
266.3237286	0.12315853	0.246675595	0.499273265	0.617586887	0.99483168	ENSG00000148356	LRSAM1
265.7456023	-0.306384742	0.284273851	-1.077780247	0.281131837	0.963267117	ENSG00000144741	SLC25A26
266.3861827	0.021345619	0.227426962	0.093857028	0.92522273	0.999527751	ENSG00000168014	C2CD3
266.0906253	0.526345779	0.425155154	1.238008699	0.215712835	0.954310328	ENSG00000123358	NR4A1
262.6950552	-0.017641235	0.29760241	-0.059277864	0.952730792	0.999527751	ENSG00000158793	NIT1
265.320748	0.646313634	0.342337222	1.887944379	0.059033418	0.853473128	ENSG00000108797	CNTNAP1
268.4436675	-0.255300302	0.351534169	-0.726246049	0.467687947	0.99135833	ENSG00000109654	TRIM2
265.6204672	0.528623174	0.261546617	2.021143228	0.043264941	0.807325344	ENSG00000137842	TMEM62
267.9345855	0.075961558	0.34045642	0.223116833	0.823444578	0.999467288	ENSG00000104064	GABPB1
265.5132051	-0.086086754	0.312401868	-0.275564147	0.78288285	0.999467288	ENSG00000143373	ZNF687
261.9783511	-0.280513161	0.330976636	-0.847531607	0.396698877	0.988378925	ENSG00000136444	RSAD1
269.3704623	0.20670295	0.308502378	0.670020606	0.502844654	0.993513219	ENSG00000182973	CNOT10
265.7259987	0.260162434	0.266629599	0.975744759	0.329190966	0.976026345	ENSG00000160959	LRRC14
267.5191253	-0.120347785	0.314064538	-0.38319444	0.7015756	0.998869317	ENSG00000183762	KREMEN1
266.6378027	0.19295474	0.317842865	0.607075889	0.54380055	0.99483168	ENSG00000164038	SLC9B2
267.8148188	-0.023709077	0.4280428	-0.0553895	0.955828161	0.999527751	ENSG00000105419	MEIS3
262.4164608	0.022877084	0.474029177	0.048260919	0.9615083	0.999527751	ENSG00000186470	BTN3A2
264.577069	0.190360597	0.256609492	0.741829914	0.458190394	0.99135833	ENSG00000033050	ABCF2
263.4797778	-0.134435891	0.262837926	-0.511478282	0.609016192	0.99483168	ENSG00000177548	RABEP2
263.3179174	0.800809002	0.369215118	2.168949651	0.030086507	0.773201806	ENSG00000260549	MT1L
263.7770217	0.105423779	0.261673397	0.40288306	0.687034254	0.998869317	ENSG00000085231	AK6
262.3257555	0.054438667	0.304973997	0.178502652	0.858328239	0.999467288	ENSG00000155287	SLC25A28
264.5912959	-0.113059121	0.232312127	-0.486669045	0.626492885	0.99483168	ENSG00000272779	AC245060.4
267.5764176	-0.225822185	0.30180166	-0.748246995	0.454311189	0.99135833	ENSG00000165533	TTC8
263.9921799	-0.067113421	0.317898917	-0.2111156	0.832797068	0.999467288	ENSG00000072736	NFATC3
267.4438032	0.251408458	0.295701335	0.850210765	0.395207918	0.988378925	ENSG00000198198	SZT2
261.7518159	-0.167721343	0.315832977	-0.531044429	0.595387992	0.99483168	ENSG00000068137	PLEKHH3
266.3273166	0.523862963	0.271628986	1.928597422	0.053780859	0.847598083	ENSG00000047056	WDR37
266.1435439	-0.047986883	0.253259271	-0.1894773	0.849718745	0.999467288	ENSG00000133641	C12orf29
266.4214956	0.156618961	0.336330415	0.46566993	0.641451779	0.996566167	ENSG00000130158	DOCK6
264.0641444	0.159676776	0.30597581	0.521860783	0.601767265	0.99483168	ENSG00000132680	KHDC4
265.0249517	-0.54657353	0.35330346	-1.547037015	0.12185432	0.923922314	ENSG00000272695	GAS6-DT
263.9640537	-0.337909634	0.261682704	-1.291295252	0.196601316	0.954310328	ENSG00000030110	BAK1
262.4967694	-0.298535211	0.268462227	-1.112019423	0.266129799	0.962465655	ENSG00000170430	MGMT
263.9630777	-0.802302602	0.397448038	-2.018635203	0.043525149	0.807325344	ENSG00000236104	ZBTB22
262.6519726	0.064411792	0.265509953	0.242596525	0.808317974	0.999467288	ENSG00000188786	MTF1
262.1195935	-0.095329145	0.280405268	-0.339969165	0.733879749	0.999467288	ENSG00000101928	MOSPD1
265.3333535	0.169202903	0.303854932	0.556854228	0.57762703	0.99483168	ENSG00000205189	ZBTB10
261.5057881	0.090487582	0.293606755	0.308193122	0.757935386	0.999467288	ENSG00000011243	AKAP8L
264.3084998	0.190108364	0.306555319	0.620143745	0.535163154	0.99483168	ENSG00000123737	EXOSC9
262.9438777	2.273209557	1.463864421	1.552882579	0.120451189	0.923922314	ENSG00000089685	BIRC5
259.6285524	-0.132410738	0.338944965	-0.390655571	0.696051844	0.998869317	ENSG00000158526	TSR2
262.9476242	-0.157262688	0.267276593	-0.588389302	0.556271016	0.99483168	ENSG00000129084	PSMA1
262.7999452	0.927854371	0.461149053	2.01204874	0.044214799	0.807325344	ENSG00000154127	UBASH3B

263.2317906	0.0388908	0.280590573	0.138603374	0.889763577	0.999467288	ENSG00000178028	DMAP1
261.6469205	-0.096849559	0.286574137	-0.337956385	0.735396056	0.999467288	ENSG00000173409	ARV1
264.8954191	0.158986226	0.264871801	0.600238398	0.548347367	0.99483168	ENSG00000101901	ALG13
261.4909964	-0.016572354	0.330952285	-0.050074754	0.960062818	0.999527751	ENSG00000166575	TMEM135
262.3161844	-0.318245948	0.255919783	-1.243537892	0.213669687	0.954310328	ENSG00000119650	IFT43
262.3965475	-0.191275754	0.378588869	-0.505233433	0.613394891	0.99483168	ENSG00000254087	LYN
259.9108408	-0.295074983	0.300341874	-0.98246368	0.325871468	0.976026345	ENSG00000126456	IRF3
262.4784746	0.540579898	0.257019749	2.103262108	0.035442864	0.785058983	ENSG00000117360	PRPF3
260.1688944	0.724217324	0.328389965	2.205357661	0.027429007	0.769167109	ENSG00000214944	ARHGEF28
259.453717	-0.466143952	0.275331209	-1.693029836	0.090449788	0.912150398	ENSG00000145912	NHP2
263.4108984	0.688953364	0.293488195	2.347465335	0.018901632	0.726053908	ENSG00000164815	ORC5
260.9408764	0.077371529	0.25066557	0.308664366	0.757576853	0.999467288	ENSG00000180329	CCDC43
257.7784264	0.30434058	0.526986432	0.577511225	0.563594158	0.99483168	ENSG00000120899	PTK2B
262.3065872	-0.243627245	0.257177461	-0.947311804	0.343479917	0.977691382	ENSG00000110497	AMBRA1
262.6800622	0.053446699	0.272252729	0.196312813	0.844365333	0.999467288	ENSG00000126775	ATG14
265.7657338	0.2498305	0.548635721	0.455366812	0.648845367	0.997368416	ENSG00000184226	PCDH9
259.363738	-0.075482059	0.30434042	-0.248018514	0.804120082	0.999467288	ENSG00000101844	ATG4A
266.3077475	-0.248110686	0.306304908	-0.810012116	0.417933212	0.990226751	ENSG00000140406	TLNRD1
260.3486703	0.354089861	0.311195314	1.137838024	0.255188124	0.962465655	ENSG00000171522	PTGER4
259.7970374	-0.564974201	0.933945107	-0.604932984	0.545223526	0.99483168	ENSG00000167244	IGF2
261.5759533	-0.224001843	0.253715884	-0.882884584	0.377298633	0.982609538	ENSG00000141376	BCAS3
261.8150208	-0.047882997	0.23592383	-0.202959563	0.839166638	0.999467288	ENSG00000163257	DCAF16
261.9751071	-0.405939511	0.344028407	-1.179959278	0.238016411	0.962465655	ENSG00000160539	PLPP7
261.8343982	-0.871478152	0.537902865	-1.620140378	0.105202125	0.92169898	ENSG00000129988	LBP
250.208292	-0.865239935	1.558251703	-0.55526327	0.578714598	0.99483168	ENSG00000082196	C1QTNF3
261.1865834	0.245495402	0.291349475	0.842614877	0.399443868	0.988378925	ENSG00000100416	TRMU
257.6903296	0.553331675	0.306161381	1.807320286	0.070712355	0.88094849	ENSG00000145604	SKP2
258.3666138	-0.508558069	0.310517336	-1.637776735	0.101468276	0.92169898	ENSG00000170946	DNAJC24
263.2549983	0.310818074	0.31836163	0.976305073	0.328913307	0.976026345	ENSG00000197603	CPLANE1
257.6869168	-0.193267018	0.302711257	-0.638453357	0.523178607	0.994616524	ENSG00000122507	BBS9
261.0326621	-0.12371855	0.27032414	-0.457667413	0.647191403	0.997368416	ENSG00000165392	WRN
258.3192081	-0.192008116	0.255341576	-0.751965735	0.452071665	0.99135833	ENSG00000171130	ATP6V0E2
263.1412727	-0.226760007	0.351662169	-0.644823433	0.519041615	0.993513219	ENSG00000166188	ZNF319
260.7894762	0.1876534	0.279856678	0.670533937	0.50251748	0.993513219	ENSG00000133597	ADCK2
261.0450623	0.480360773	0.329225714	1.459062136	0.144548007	0.935644597	ENSG00000121057	AKAP1
260.9341147	0.203950366	0.250134621	0.815362407	0.414864896	0.990226751	ENSG00000184677	ZBTB40
259.5243102	-0.396161495	0.260999846	-1.517861029	0.129049436	0.928627271	ENSG00000137947	GTF2B
260.7317225	-0.176155076	0.268336665	-0.656470393	0.511521511	0.993513219	ENSG00000112578	BYSL
259.5779921	-0.097437545	0.344181402	-0.283099388	0.77710065	0.999467288	ENSG00000008086	CDKL5
258.5607189	0.338998003	0.361570301	0.937571482	0.348464673	0.977691382	ENSG00000077312	SNRPA
260.7544336	-0.18911951	0.356925072	-0.529857736	0.596210571	0.99483168	ENSG00000254470	AP5B1
257.5927519	-0.323140325	0.348505835	-0.927216398	0.353814191	0.977691382	ENSG00000166228	PCBD1
259.4201751	0.110095208	0.274185772	0.401535088	0.68802621	0.998869317	ENSG00000111731	C2CD5
260.3203114	-0.256472471	0.379084273	-0.676557929	0.49868648	0.993513219	ENSG00000218891	ZNF579
259.5977242	-0.425659504	0.259262577	-1.641808508	0.100629695	0.92169898	ENSG00000116514	RNF19B
260.6825164	-0.210802429	0.258597433	-0.81517603	0.414971556	0.990226751	ENSG00000235173	HGH1
259.9120679	0.180809391	0.317318904	0.569803402	0.568811048	0.99483168	ENSG00000026652	AGPAT4
263.9292636	0.365544951	0.340206802	1.074478667	0.282608201	0.963267117	ENSG00000121350	PYROXD1
259.4988899	-0.145788063	0.522993235	-0.278757073	0.78043126	0.999467288	ENSG00000125730	C3
258.6041389	-0.110616939	0.279476582	-0.395800386	0.692252292	0.998869317	ENSG00000204406	MBD5
261.8487384	-0.118308882	0.277606389	-0.426174925	0.669980388	0.997568624	ENSG00000155545	MIER3
255.7908215	-0.076134179	0.56664864	-0.134358707	0.893118933	0.999467288	ENSG00000064309	CDON
257.2928466	-0.355991776	0.310208998	-1.147586878	0.251139149	0.962465655	ENSG00000188342	GTF2F2
261.1594976	-0.057918283	0.34046319	-0.170116139	0.864918801	0.999467288	ENSG00000132478	UNK
261.9015822	-0.394507016	0.357517463	-1.103462226	0.269826487	0.962465655	ENSG00000197183	NOL4L
259.500237	0.494197876	0.316572096	1.561091082	0.118502262	0.923922314	ENSG00000168411	RFWD3
259.779314	0.134879331	0.260359152	0.518051047	0.604422654	0.99483168	ENSG00000133678	TMEM254
260.5704209	0.005364587	0.265307971	0.020220224	0.983867695	0.999527751	ENSG00000183475	ASB7
260.4680253	0.09358222	0.287209734	0.325832341	0.744551208	0.999467288	ENSG00000100599	RIN3
259.0317693	-0.012970627	0.24874937	-0.052143357	0.958414466	0.999527751	ENSG00000075089	ACTR6
261.1266332	0.202356645	0.294523934	0.6870635	0.492042714	0.993513219	ENSG00000244306	DUXAP10
257.6898089	-0.158159408	0.264278044	-0.598458374	0.549534126	0.99483168	ENSG00000140153	WDR20
255.2389086	0.003577734	0.34979144	0.010228193	0.991839225	0.999527751	ENSG00000056972	TRAF3IP2
255.3621454	-0.222390571	0.291332676	-0.763356085	0.445251062	0.99135833	ENSG00000214087	ARL16

260.8138011	0.245821041	0.407514441	0.603220441	0.546362051	0.99483168	ENSG00000154429	CCSAP
256.1640264	0.098696865	0.364601668	0.270697787	0.786623479	0.999467288	ENSG00000101935	AMMECR1
256.774881	0.044903234	0.339395229	0.13230367	0.894744105	0.999467288	ENSG00000131269	ABCB7
258.7452424	-0.241066557	0.23126798	-1.042368932	0.297240665	0.971974628	ENSG00000130348	QRSL1
258.7628671	-0.348092388	0.256923315	-1.354849353	0.175465559	0.946278819	ENSG00000139637	C12orf10
254.8537914	-0.175342048	0.31649203	-0.554017261	0.579567033	0.99483168	ENSG00000214113	LYRM4
259.8989469	0.017918007	0.283403054	0.063224466	0.949587763	0.999527751	ENSG00000101407	TTI1
256.7451761	-0.559334141	0.268328259	-2.084514479	0.037113395	0.790190505	ENSG00000136161	RCBTB2
259.4991589	0.001320068	0.256799936	0.005140451	0.995898531	0.999527751	ENSG00000163933	RFT1
258.7533871	0.089104769	0.236175005	0.377282807	0.705963448	0.99900742	ENSG00000108064	TFAM
258.4916973	-0.026852282	0.276854349	-0.096990645	0.922733823	0.999527751	ENSG00000185515	BRCC3
258.4881376	-0.035736493	0.236634115	-0.151020039	0.879959908	0.999467288	ENSG00000147905	ZCCHC7
258.0801335	0.633471776	0.238198298	2.659430316	0.007827292	0.570957466	ENSG00000196233	LCOR
255.4471531	0.065742939	0.30747696	0.213814195	0.830691955	0.999467288	ENSG00000011478	QPCTL
253.9975776	-0.193686438	0.389610194	-0.497128774	0.619098245	0.99483168	ENSG00000135617	PRADC1
254.2155935	0.326352131	0.4578216	0.712836902	0.475946688	0.99135833	ENSG00000106483	SFRP4
254.5675397	-0.367738743	0.277723271	-1.324119297	0.185463428	0.949094286	ENSG00000183597	TANGO2
257.2036247	-0.066173439	0.312258237	-0.211918956	0.832170262	0.999467288	ENSG00000174173	TRMT10C
259.2945748	-0.035006778	0.293611551	-0.119228204	0.905094562	0.999527751	ENSG00000082516	GEMIN5
261.4938413	0.027397232	0.317237675	0.086361847	0.931178775	0.999527751	ENSG00000131196	NFATC1
261.2824566	-0.074802175	0.303873326	-0.246162361	0.805556551	0.999467288	ENSG00000198718	TOGARAM1
257.9146948	0.170718228	0.247382932	0.690097035	0.490133167	0.993513219	ENSG00000068097	HEATR6
258.2101885	-0.435939563	0.254346471	-1.713959551	0.086536155	0.901307093	ENSG00000069974	RAB27A
259.1475339	0.05772565	0.368028474	0.156851042	0.875362245	0.999467288	ENSG00000146083	RNF44
257.7245213	0.425347802	0.333338407	1.276023984	0.201947041	0.954310328	ENSG00000162341	TPCN2
257.7448564	0.260395737	0.309461796	0.84144712	0.400097498	0.988492517	ENSG00000168778	TCTN2
258.7508464	0.267424853	0.265242511	1.008227724	0.313345151	0.973427483	ENSG00000147133	TAF1
256.862516	0.359061272	0.272237032	1.318928837	0.187192904	0.949094286	ENSG00000167904	TMEM68
256.662413	2.699867516	1.549470769	1.74244495	0.081430616	0.897807954	ENSG00000138180	CEP55
254.8461297	0.293094834	0.364402955	0.804315195	0.421214964	0.991301167	ENSG00000034053	APBA2
257.1882035	0.412088427	0.261275891	1.577215659	0.114745911	0.923922314	ENSG00000152520	PAN3
253.7490458	-0.069673151	0.306769709	-0.227118745	0.820331408	0.999467288	ENSG00000185880	TRIM69
253.3987916	-0.187233599	0.302808798	-0.618322849	0.536362543	0.99483168	ENSG00000167461	RAB8A
254.7458592	0.197246662	0.256637826	0.768579854	0.442142769	0.99135833	ENSG00000063438	AHRR
259.3254571	0.500449965	0.313897359	1.594310848	0.110866429	0.923922314	ENSG00000033030	ZCCHC8
258.9011105	0.041445524	0.255268393	0.162360577	0.871021914	0.999467288	ENSG00000047578	KIAA0556
256.5961358	0.214216996	0.267182817	0.801761873	0.422690717	0.991301167	ENSG00000279118	AC093535.2
256.4746627	0.125297784	0.244854842	0.511722711	0.608845089	0.99483168	ENSG00000112130	RNF8
258.0315396	-0.079848211	0.267106332	-0.29893792	0.764987414	0.999467288	ENSG00000224631	RPS27AP16
256.4155022	0.254114922	0.230152482	1.104115496	0.26954304	0.962465655	ENSG00000085872	CHERP
253.9635286	0.397374375	0.352984081	1.125757211	0.2602683	0.962465655	ENSG00000117226	GBP3
255.4736594	-0.280189309	0.274270157	-1.021581466	0.306979033	0.973427483	ENSG00000205981	DNAJC19
257.9810344	0.059195305	0.381811584	0.155038003	0.876791358	0.999467288	ENSG00000174804	FZD4
254.5847727	-0.056500135	0.282024056	-0.200337999	0.841216246	0.999467288	ENSG00000176209	SMIM19
258.6842793	-0.354877967	0.435952682	-0.814028638	0.415628545	0.990226751	ENSG00000115648	MLPH
255.3274349	-0.280317918	0.268132586	-1.045445175	0.295817267	0.970706863	ENSG00000146109	ABT1
256.6936949	0.003172457	0.252241509	0.012577062	0.989965221	0.999527751	ENSG00000014919	COX15
255.9268695	0.053859623	0.360667127	0.149333331	0.881290616	0.999467288	ENSG00000152061	RABGAP1L
256.9155702	0.072747756	0.239373484	0.303909001	0.76119722	0.999467288	ENSG00000164219	PGGT1B
257.3117421	-0.280023089	0.263537785	-1.062553856	0.287984322	0.966288987	ENSG00000133114	GPALPP1
256.5508504	0.199532614	0.249970745	0.798223866	0.42474059	0.991301167	ENSG00000103351	CLUAP1
253.6607794	-0.099421598	0.489624801	-0.203056703	0.839090713	0.999467288	ENSG00000075643	MOCOS
257.5959762	-0.151839598	0.337346111	-0.450100338	0.652638093	0.997471445	ENSG00000101216	GMEB2
258.8107387	-0.072884193	0.308263393	-0.236434797	0.813095298	0.999467288	ENSG00000131697	NPHP4
257.9978381	0.287076936	0.386301747	0.743141697	0.457395897	0.99135833	ENSG00000080200	CRYBG3
251.5776242	-0.191273313	0.350626959	-0.545517987	0.585397313	0.99483168	ENSG00000013563	DNASE1L1
256.796454	0.085353392	0.266655711	0.32008837	0.748901342	0.999467288	ENSG00000147654	EBAG9
253.8804318	0.285784095	0.315955487	0.90450746	0.365726381	0.981269214	ENSG00000143801	PSEN2
253.6259342	0.189259968	0.253442961	0.746755669	0.45521106	0.99135833	ENSG00000196535	MYO18A
254.4179218	-0.189982996	0.28020655	-0.678010544	0.497765008	0.993513219	ENSG00000262919	CCNQ
257.1968929	0.276959317	0.254402832	1.088664442	0.276301888	0.962465655	ENSG00000245680	ZNF585B
253.6073831	0.09593337	0.265793454	0.360932028	0.71815026	0.99900742	ENSG00000165813	CCDC186
254.3236743	-0.612571902	0.280678336	-2.182469477	0.029074902	0.773201806	ENSG00000125901	MRPS26
254.2318305	0.115141104	0.272881481	0.421945465	0.673064823	0.998473603	ENSG00000082515	MRPL22

251.2327184	0.230298498	0.541268194	0.425479458	0.670487193	0.997568624	ENSG00000162645	GBP2
253.3813549	0.035125355	0.279872711	0.125504752	0.900123963	0.999527751	ENSG00000158555	GDPD5
253.3348402	-0.17131954	0.379969176	-0.450877468	0.652077864	0.997449432	ENSG00000033327	GAB2
254.428565	0.216583373	0.930880434	0.232665082	0.816021485	0.999467288	ENSG00000179593	ALOX15B
254.9536566	-0.435197341	0.240592421	-1.808857234	0.070473186	0.87954613	ENSG00000188070	C11orf95
254.9123829	0.141311101	0.284718522	0.496318607	0.61966964	0.99483168	ENSG00000166987	MBD6
251.8865405	0.011431646	0.355698829	0.032138553	0.974361558	0.999527751	ENSG00000140853	NLRC5
250.8905243	-0.082294215	0.495343285	-0.166135724	0.868050141	0.999467288	ENSG00000094963	FMO2
256.467344	-0.327038356	0.366771399	-0.8916681	0.372570857	0.982259938	ENSG00000163818	LZTFL1
250.0577974	-0.441664237	1.170531321	-0.377319453	0.705936218	0.99900742	ENSG00000103175	WFDC1
254.8531838	0.156911271	0.249620654	0.628598911	0.529611673	0.99483168	ENSG00000079313	REXO1
254.5826205	-0.498584983	0.30490689	-1.635204057	0.102006278	0.92169898	ENSG00000233426	EIF3FP3
252.8753247	0.256090719	0.278242905	0.92038544	0.357371374	0.979427314	ENSG00000136897	MRPL50
251.3877607	0.308356704	0.400708427	0.769528873	0.441579411	0.99135833	ENSG00000163710	PCOLCE2
255.5925788	0.196204021	0.433625652	0.452473279	0.650928067	0.997449432	ENSG00000276547	PCDHGB5
259.6730581	2.700604033	1.664138744	1.622823843	0.104627067	0.92169898	ENSG00000166851	PLK1
253.0101759	-0.072792742	0.243525958	-0.298911634	0.76500747	0.999467288	ENSG00000148343	MIGA2
252.8716474	0.109361965	0.371830408	0.294117862	0.768667842	0.999467288	ENSG00000173846	PLK3
247.1634731	-0.015661065	0.527320377	-0.029699336	0.976306841	0.999527751	ENSG00000181649	PHLDA2
253.927244	-0.493892038	0.361612301	-1.365805413	0.172000047	0.944422693	ENSG00000121281	ADCY7
255.4666942	0.924056323	0.398154911	2.320846227	0.020295145	0.73997555	ENSG00000268205	AC005261.1
253.9738965	-0.057762121	0.237001761	-0.24372022	0.807447513	0.999467288	ENSG00000149474	KAT14
253.7030076	-0.296458995	0.265161832	-1.118030422	0.263554001	0.962465655	ENSG00000174151	CYB561D1
250.1673777	-0.149017536	0.341941494	-0.435798341	0.662983047	0.997568624	ENSG00000143390	RFX5
254.8969302	-0.102396276	0.434516056	-0.235655908	0.813699687	0.999467288	ENSG00000186076	AC012085.1
253.0980315	0.164553512	0.250988331	0.655622161	0.512067263	0.993513219	ENSG00000121274	TENT4B
254.3517522	-0.181304824	0.37844633	-0.479076713	0.631884056	0.99483168	ENSG00000134121	CHL1
254.2499159	-0.00984138	0.295064803	-0.033353283	0.973392864	0.999527751	ENSG00000106603	COA1
254.726476	0.052220278	0.280973081	0.185855093	0.852558399	0.999467288	ENSG00000104427	ZC2HC1A
250.0599611	0.166778682	0.408528426	0.408242539	0.683095621	0.998869317	ENSG00000221890	NPTXR
253.882906	0.151120887	0.266816314	0.566385482	0.571131762	0.99483168	ENSG00000171466	ZNF562
253.9688482	0.150583188	0.294609529	0.511128031	0.609261409	0.99483168	ENSG00000130449	ZSWIM6
250.501807	-0.161632219	0.374785765	-0.431265631	0.666275238	0.997568624	ENSG00000197562	RAB40C
247.2649335	1.309586807	0.653252569	2.004717423	0.044993265	0.813244956	ENSG00000100297	MCM5
250.940918	-0.459258988	0.325441381	-1.41118805	0.158189173	0.939464191	ENSG00000165072	MAMDC2
252.9502042	0.193419485	0.472797991	0.409095405	0.682469648	0.998869317	ENSG00000183049	CAMK1D
253.0291608	0.501999061	0.294286362	1.705818297	0.088041915	0.909577527	ENSG00000078403	MLLT10
250.1855136	-0.247859443	0.325404718	-0.761695911	0.446241517	0.99135833	ENSG00000134504	KCTD1
251.8640166	-0.234020334	0.259872247	-0.900520687	0.367843222	0.981269214	ENSG00000186687	LYRM7
253.477321	0.524941672	0.290153743	1.80918456	0.070422336	0.87954613	ENSG00000198040	ZNF84
252.0357318	-0.023230896	0.244783039	-0.094904026	0.924391058	0.999527751	ENSG00000165355	FBXO33
255.480134	0.22910485	0.312208396	0.733820271	0.463058284	0.99135833	ENSG00000197818	SLC9A8
252.0983215	0.095029684	0.265113108	0.358449585	0.72000689	0.99900742	ENSG00000132964	CDK8
251.5248274	-0.180019312	0.291594913	-0.61736095	0.536996673	0.99483168	ENSG00000129480	DTD2
252.936016	0.177586412	0.251494506	0.70612442	0.480110767	0.99135833	ENSG00000168564	CDKN2AIP
247.223105	0.068326018	0.322655386	0.211761591	0.832293035	0.999467288	ENSG00000068383	INPP5A
250.3138965	0.557762645	0.338685873	1.646843551	0.099590207	0.921147998	ENSG00000073910	FRY
250.3191771	0.204009363	0.310577051	0.656871982	0.511263235	0.993513219	ENSG00000168234	TTC39C
252.6094632	0.047132104	0.285961415	0.164819803	0.869085816	0.999467288	ENSG00000103326	CAPN15
249.3687167	0.140862186	0.293762495	0.47951045	0.631575536	0.99483168	ENSG00000173145	NOC3L
251.5548767	-0.022170805	0.248087045	-0.089367041	0.928790216	0.999527751	ENSG00000106477	CEP41
251.6705832	-0.029746643	0.326490909	-0.091110174	0.927405049	0.999527751	ENSG00000127666	TICAM1
249.6737233	-0.488421596	0.287200215	-1.700631023	0.089012295	0.910413547	ENSG00000213190	MLLT11
250.0526907	0.226963373	0.23927799	0.948534266	0.342857537	0.977691382	ENSG00000170037	CNTROB
252.8754397	0.139177224	0.261769062	0.531679426	0.594948044	0.99483168	ENSG00000174013	FBXO45
248.3808132	-0.253759854	0.595725216	-0.425967959	0.670131193	0.997568624	ENSG00000006747	SCIN
250.268476	-0.257011902	0.278219729	-0.923773101	0.355604463	0.978364583	ENSG00000175324	LSM1
249.0845658	-0.340292214	0.292207051	-1.164558533	0.244197737	0.962465655	ENSG00000168005	SPINDOC
251.4574291	-0.24356153	0.262763656	-0.926922444	0.353966803	0.977691382	ENSG00000226964	RHEBP2
251.4915876	0.285521401	0.322936317	0.884141505	0.37661983	0.982609538	ENSG00000116106	EPHA4
252.4689679	-0.186100976	0.47956838	-0.388059313	0.697972141	0.998869317	ENSG00000067445	TRO
250.3110327	0.330577955	0.296562838	1.114697839	0.264979928	0.962465655	ENSG00000157796	WDR19
249.6406872	-0.57070631	0.279550074	-2.041517295	0.041199437	0.800562177	ENSG00000148335	NTMT1
255.1754087	-0.505436344	0.405747736	-1.245691099	0.212877823	0.954310328	ENSG00000152217	SETBP1

247.29151	0.005100759	0.365355952	0.013961067	0.988861042	0.999527751	ENSG00000198618	PPIAP22
252.0823915	0.088081622	0.288348947	0.305468851	0.76000909	0.999467288	ENSG00000111845	PAK1IP1
251.5878324	-0.115402446	0.322785756	-0.357520256	0.720702365	0.99900742	ENSG00000099999	RNF215
255.1265152	0.225952534	0.34433069	0.656207944	0.511690338	0.993513219	ENSG00000146587	RBAK
253.1275835	0.081797114	0.32612789	0.250812998	0.801958692	0.999467288	ENSG00000070476	ZXDC
249.0453422	0.64754439	0.432198525	1.498256826	0.134066537	0.933741583	ENSG00000163516	ANKZF1
249.4207107	0.123522026	0.30007369	0.411638973	0.680604061	0.998869317	ENSG00000141378	PTRH2
248.662537	0.009218545	0.265833635	0.034677872	0.972336606	0.999527751	ENSG00000196116	TDRD7
250.7258262	0.018934896	0.255380231	0.07414394	0.940895852	0.999527751	ENSG00000196652	ZKSCAN5
248.7893311	-0.164782159	0.253916659	-0.648961591	0.516363203	0.993513219	ENSG00000133027	PEMT
249.5096909	-0.391976949	0.266881468	-1.46873049	0.141905904	0.935644597	ENSG00000101298	SNPH
247.0734832	-0.122408795	0.325350031	-0.376237233	0.706740538	0.99900742	ENSG00000132535	DLG4
252.0822261	-0.05215437	0.461286525	-0.113062852	0.909980726	0.999527751	ENSG00000123104	ITPR2
245.8847822	-0.190787603	0.323654151	-0.589479858	0.55553942	0.99483168	ENSG00000247077	PGAM5
248.9289018	0.685618367	0.347035717	1.975642084	0.048195329	0.832637719	ENSG00000101639	CEP192
251.3628057	-0.311302058	0.300454444	-1.036104025	0.300153615	0.972186492	ENSG00000137764	MAP2K5
248.4384435	-0.370779696	0.237620329	-1.560387101	0.118668431	0.923922314	ENSG00000103356	EARS2
248.2701175	0.3696728	0.397213537	0.930665162	0.352026796	0.977691382	ENSG00000144749	LRIG1
249.0538258	0.091321128	0.239210182	0.381761039	0.702638621	0.998869317	ENSG00000146963	LUC7L2
246.9668089	0.442606156	0.318051855	1.391616333	0.16403861	0.943223078	ENSG00000176444	CLK2
247.4262408	-0.150055925	0.265328703	-0.565547277	0.571701577	0.99483168	ENSG00000245937	LINC01184
248.3894482	0.033535767	0.260991263	0.12849383	0.89775818	0.999467288	ENSG00000178105	DDX10
247.1937622	0.342555423	0.250292561	1.36862007	0.171118067	0.944422693	ENSG00000101911	PRPS2
259.2520572	0.248201253	0.56385478	0.440186484	0.659802048	0.997568624	ENSG00000026559	KCNG1
248.1483373	-0.013037304	0.29622729	-0.044011152	0.964895514	0.999527751	ENSG00000121413	ZSCAN18
247.0240052	-0.1189505	0.314068668	-0.378740423	0.704880633	0.99900742	ENSG00000170854	RIOX2
249.4330806	0.160530312	0.264220638	0.607561594	0.543478279	0.99483168	ENSG00000075303	SLC25A40
247.2542867	-0.041915955	0.287651257	-0.145717963	0.88414404	0.999467288	ENSG00000075234	TTC38
251.5097927	0.249936424	0.404924514	0.617242018	0.537075105	0.99483168	ENSG00000177663	IL17RA
247.583948	0.047055851	0.238471553	0.197322703	0.843575014	0.999467288	ENSG00000165055	METTL2B
248.516571	0.179499205	0.264646789	0.678259526	0.497607156	0.993513219	ENSG00000170322	NFRKB
248.3231118	-0.394417668	0.259585405	-1.519413882	0.12865835	0.928627271	ENSG00000065457	ADAT1
248.974622	0.055680793	0.27260851	0.204251854	0.838156692	0.999467288	ENSG00000135845	PIGC
246.7661267	0.079580951	0.249314542	0.319198992	0.749575621	0.999467288	ENSG00000182858	ALG12
247.9163254	0.053530659	0.35789207	0.149572074	0.881102242	0.999467288	ENSG00000185561	TLCD2
250.4150806	0.036799236	0.283325655	0.129883177	0.896658854	0.999467288	ENSG00000166377	ATP9B
248.1506639	0.081945651	0.238434305	0.343682305	0.73108522	0.999467288	ENSG00000118961	LDAH
247.7729833	0.060903836	0.275730813	0.2208815	0.825184702	0.999467288	ENSG00000181472	ZBTB2
245.0775404	-0.282303317	0.289830272	-0.974029784	0.330041751	0.976026345	ENSG00000172336	POP7
245.5123111	-0.115569097	0.273970147	-0.421830984	0.673148388	0.998473603	ENSG00000099899	TRMT2A
247.0586217	0.29357436	0.25159164	1.166868501	0.243263478	0.962465655	ENSG00000108239	TBC1D12
243.9755996	0.263233154	0.335463008	0.784686083	0.432637644	0.991301167	ENSG00000077150	NFKB2
244.8858032	-0.038731802	0.27059326	-0.143136608	0.886182293	0.999467288	ENSG00000157881	PANK4
247.3264808	0.032949085	0.287912697	0.114441235	0.908888028	0.999527751	ENSG00000188559	RALGAPA2
247.5947957	-0.408142647	0.25768172	-1.583902212	0.113215974	0.923922314	ENSG00000164077	MON1A
244.0306134	0.101888795	0.330240392	0.308529174	0.757679705	0.999467288	ENSG00000132256	TRIM5
245.9650735	0.30210572	0.274991139	1.098601656	0.271941855	0.962465655	ENSG00000081019	RSBN1
248.450814	-0.220948346	0.295309605	-0.748192211	0.454344228	0.99135833	ENSG00000168887	C2orf68
243.482917	-0.42120863	0.332020729	-1.268621484	0.204576098	0.954310328	ENSG00000275074	NUDT18
245.3728139	0.295281082	0.27350462	1.07962009	0.280311391	0.963267117	ENSG00000137944	KYAT3
244.3125775	0.039267095	0.310094551	0.126629427	0.899233705	0.999527751	ENSG00000132600	PRMT7
247.8165741	0.16734747	0.433999257	0.38559391	0.699797444	0.998869317	ENSG00000100100	PIK3IP1
252.2516736	-0.471909012	0.40375107	-1.168811792	0.242479468	0.962465655	ENSG00000152413	HOMER1
247.4861881	0.278212502	0.282952864	0.983246813	0.32548598	0.976026345	ENSG00000158636	EMSY
246.5302248	-0.09605643	0.246115469	-0.390290097	0.696322045	0.998869317	ENSG00000141569	TRIM65
247.6170482	-0.214457846	0.256967575	-0.834571621	0.403958946	0.988927059	ENSG00000068400	GRIPAP1
247.2442474	-0.101382998	0.270243465	-0.375154301	0.707545715	0.99900742	ENSG00000161395	PGAP3
241.4014437	-0.204396533	0.354946453	-0.575851742	0.564715397	0.99483168	ENSG00000169738	DCXR
246.8515371	-0.489446876	0.314083657	-1.558332839	0.119154368	0.923922314	ENSG00000005073	HOXA11
244.3106703	0.025030166	0.296703092	0.084360986	0.932769425	0.999527751	ENSG00000244005	NFS1
243.060824	-0.534331071	0.359149527	-1.487767714	0.136812163	0.934144427	ENSG00000131116	ZNF428
245.7650769	0.027244074	0.253913502	0.107296671	0.914553625	0.999527751	ENSG00000196505	GDAP2
249.2970088	0.682797265	0.378738787	1.802818428	0.071416735	0.883866242	ENSG00000130396	AFDN
248.7664733	-0.197941908	0.275040107	-0.719683793	0.471719707	0.99135833	ENSG00000079387	SENP1

245.4032686	0.151724811	0.292882121	0.518040535	0.604429988	0.99483168	ENSG00000119685	TTLL5
246.8772847	0.055259678	0.243288897	0.227136046	0.820317956	0.999467288	ENSG00000158006	PAFAH2
245.0512434	-0.12695325	0.272483976	-0.465910883	0.641279291	0.996553586	ENSG00000162377	COA7
243.779916	-0.278390959	0.294357512	-0.945757957	0.344272054	0.977691382	ENSG00000121068	TBX2
243.7020193	-0.195838547	0.275402075	-0.711100476	0.477021975	0.99135833	ENSG00000002822	MAD1L1
246.3961394	0.209677767	0.267112392	0.784979554	0.432465556	0.991301167	ENSG00000173875	ZNF791
247.2414355	0.416580405	0.335012335	1.243477809	0.213691813	0.954310328	ENSG00000144935	TRPC1
246.2571781	0.32142817	0.26673263	1.205057554	0.228181084	0.958865115	ENSG00000100532	CGRRF1
241.6398224	0.334957911	0.361421398	0.926779411	0.354041077	0.977691382	ENSG00000103876	FAH
245.0399162	0.068498426	0.257373059	0.266144509	0.790127917	0.999467288	ENSG00000170638	TRABD
244.2920441	-0.285067363	0.248897316	-1.145321161	0.252076143	0.962465655	ENSG00000158552	ZFAND2B
237.7149951	0.666683503	0.941078143	0.708425233	0.478681228	0.99135833	ENSG00000108950	FAM20A
249.2376384	0.072706283	0.400992139	0.181315981	0.856119563	0.999467288	ENSG00000179242	CDH4
241.9055389	-0.197555581	0.297252874	-0.664604444	0.506303526	0.993513219	ENSG00000176108	CHMP6
242.3280719	-0.123783646	0.27691258	-0.447013444	0.654865353	0.997568624	ENSG00000130772	MED18
245.0911051	-0.05880985	0.259467576	-0.226655872	0.820691343	0.999467288	ENSG00000135632	SMYD5
247.528824	-0.000834857	0.343725839	-0.002428847	0.998062062	0.999527751	ENSG00000164331	ANKRA2
246.2067234	0.05006789	0.277452037	0.180456018	0.85679458	0.999467288	ENSG00000083844	ZNF264
246.0848691	-0.248042791	0.327023842	-0.758485342	0.448160485	0.99135833	ENSG00000170464	DNAJC18
244.2966243	0.487386303	0.331212357	1.471522103	0.141149975	0.935644597	ENSG00000100350	FOXRED2
242.8251557	-0.199152003	0.28845175	-0.690417039	0.489931965	0.993513219	ENSG00000116353	MECR
244.5805459	0.593346088	0.337667529	1.757190242	0.078885388	0.897052166	ENSG00000107554	DNMBP
245.0085169	0.225762994	0.272710517	0.827848504	0.407756302	0.990226751	ENSG00000143315	PIGM
242.7439209	-0.518830772	0.332394925	-1.560886564	0.118550518	0.923922314	ENSG00000007376	RPUSD1
240.5355551	-0.083621917	0.311627688	-0.268339176	0.788438253	0.999467288	ENSG00000134056	MRPS36
239.2534619	0.00373212	0.425639936	0.008768256	0.993004034	0.999527751	ENSG00000111907	TPD52L1
246.027811	0.010440461	0.281857451	0.037041635	0.970451809	0.999527751	ENSG00000155906	RMND1
241.537924	-0.086933974	0.3386866	-0.256679698	0.797426045	0.999467288	ENSG00000111231	GPN3
239.9223081	-0.040669838	0.300475317	-0.135351677	0.892333829	0.999467288	ENSG00000243449	C4orf48
243.2591689	0.454177258	0.263090478	1.726315838	0.084290608	0.901307093	ENSG00000156504	FAM122B
240.6223432	-0.358717976	0.364601463	-0.983863238	0.325182761	0.976026345	ENSG00000270504	AL391422.4
240.6866442	0.319022642	0.487186499	0.654826524	0.512579451	0.993513219	ENSG00000122254	HS3ST2
244.4005857	-0.102363078	0.25757204	-0.397415331	0.691061212	0.998869317	ENSG00000066135	KDM4A
237.3047591	0.516414167	0.472879483	1.092062957	0.27480543	0.962465655	ENSG00000080573	COL5A3
239.943718	-0.126309963	0.337860967	-0.373851896	0.708514507	0.99900742	ENSG00000149743	TRPT1
242.3387712	0.134086972	0.259986201	0.515746494	0.606031477	0.99483168	ENSG00000103852	TTC23
242.118287	-0.175782276	0.240702893	-0.730287343	0.465214563	0.99135833	ENSG00000107362	ABHD17B
240.5044506	0.144045196	0.284012381	0.507179286	0.612029028	0.99483168	ENSG00000139517	LNX2
244.3944629	0.006800452	0.266370845	0.025530016	0.979632207	0.999527751	ENSG00000163743	RCHY1
245.7437176	0.065242189	0.382748988	0.170456856	0.864650861	0.999467288	ENSG00000168769	TET2
243.798302	-0.252454449	0.281736074	-0.896067179	0.370216885	0.981455968	ENSG00000165724	ZMYND19
240.705343	0.069027765	0.269579301	0.256057364	0.797906543	0.999467288	ENSG00000126814	TRMT5
239.71866	0.377935231	0.508494444	0.743243581	0.457334222	0.99135833	ENSG00000131015	ULBP2
238.0852277	-2.061377776	1.245225112	-1.655425798	0.097838156	0.921147998	ENSG00000170374	SP7
242.785354	-0.316065481	0.267369817	-1.182128503	0.237154716	0.962465655	ENSG00000006194	ZNF263
242.1483316	-0.435018463	0.458240526	-0.94932342	0.342456146	0.977691382	ENSG00000108448	TRIM16L
240.6891814	-0.050829912	0.278917717	-0.182239813	0.85539453	0.999467288	ENSG00000089177	KIF16B
244.0708389	0.536591379	0.293209065	1.830064082	0.067240357	0.87420149	ENSG00000146830	GIGYF1
241.2323425	0.804047945	0.925450857	0.868817549	0.384946933	0.985733881	ENSG00000165304	MELK
241.502114	-0.381242564	0.292619806	-1.302859741	0.192622651	0.949261383	ENSG00000177352	CCDC71
239.6917604	0.087905395	0.302471745	0.290623491	0.771339292	0.999467288	ENSG00000198498	TMA16
241.0586505	0.147900075	0.309078837	0.478518931	0.632280903	0.994860874	ENSG00000101189	MRGBP
240.085453	-0.296330614	0.293167489	-1.010789483	0.312117198	0.973427483	ENSG00000068394	GPKOW
240.9919028	-0.281266276	0.279736319	-1.005469284	0.314670933	0.974768742	ENSG00000282826	FRG1CP
238.4292133	-0.393214953	0.366508301	-1.072867797	0.283330437	0.963267117	ENSG00000105379	ETFB
244.9819809	0.015773979	0.335220486	0.047055534	0.962468967	0.999527751	ENSG00000120616	EPC1
238.6419071	0.368700927	0.289927114	1.271702126	0.203478975	0.954310328	ENSG00000153558	FBXL2
242.8941497	0.003907239	0.357374915	0.010933166	0.991276769	0.999527751	ENSG00000139651	ZNF740
240.7260749	0.166135318	0.287848603	0.577162146	0.563829926	0.99483168	ENSG00000109536	FRG1
238.6417822	-0.363980825	0.362333414	-1.004546669	0.315115188	0.974768742	ENSG00000165806	CASP7
242.6642726	0.440442328	0.294453706	1.495794815	0.134707133	0.933741583	ENSG00000261609	GAN
243.1879384	2.015666959	1.463310749	1.377470206	0.16836692	0.943223078	ENSG00000117399	CDC20
239.6752551	0.076567332	0.336925752	0.227252835	0.820227145	0.999467288	ENSG00000023171	GRAMD1B
242.3133788	0.013666085	0.279299671	0.048929828	0.960975218	0.999527751	ENSG00000132640	BTBD3

238.7356767	0.14063456	0.278735001	0.504545748	0.613877923	0.99483168	ENSG00000101181	MTG2
239.2985194	-0.296665274	0.26054646	-1.138627152	0.254858698	0.962465655	ENSG00000173465	SSSCA1
242.2782425	0.438534599	0.311017144	1.410001368	0.158539279	0.939464191	ENSG00000177917	ARL6IP6
242.0288013	-0.142394372	0.24762209	-0.575047131	0.565259422	0.99483168	ENSG00000197429	IPP
239.8090641	-0.061322371	0.238117929	-0.257529415	0.796770111	0.999467288	ENSG00000172785	CBWD1
240.8585755	-0.006453645	0.277675472	-0.023241682	0.98145749	0.999527751	ENSG00000185219	ZNF445
237.0368663	-0.122935031	0.321150857	-0.382795277	0.701871563	0.998869317	ENSG00000161981	SNRNP25
240.8435528	0.179719156	0.243831579	0.737062675	0.461084236	0.99135833	ENSG00000104907	TRMT1
234.570398	-0.14525128	0.348361118	-0.416956061	0.676710547	0.998869317	ENSG00000187837	HIST1H1C
237.6053383	0.022178832	0.312054768	0.071073524	0.943339239	0.999527751	ENSG00000198960	ARMCX6
240.0255135	-0.51943563	0.313539257	-1.656684508	0.097583277	0.921147998	ENSG00000166171	DPCD
239.838949	-0.082039216	0.260782154	-0.314589075	0.753073675	0.999467288	ENSG00000103510	KAT8
240.6992969	0.053863595	0.261328284	0.206114679	0.836701333	0.999467288	ENSG00000087995	METTL2A
228.8250932	0.101147579	0.459767131	0.219997413	0.82587317	0.999467288	ENSG00000124249	KCNK15
236.5984764	0.154881512	0.309155797	0.500982073	0.61638374	0.99483168	ENSG00000178537	SLC25A20
236.4548821	0.112038311	0.432877294	0.258822333	0.795772326	0.999467288	ENSG00000058056	USP13
239.7918062	-0.25758729	0.275351627	-0.935484905	0.349538459	0.977691382	ENSG00000253738	OTUD6B-AS1
239.6945945	0.084243411	0.313252826	0.268931049	0.787982743	0.999467288	ENSG00000185215	TNFAIP2
237.3683866	-0.108211569	0.391510155	-0.276395307	0.782244461	0.999467288	ENSG00000221955	SLC12A8
239.620371	-0.156363217	0.242522106	-0.644737998	0.519096988	0.993513219	ENSG00000160214	RRP1
239.7040672	0.098115924	0.268991022	0.364755385	0.715294011	0.99900742	ENSG00000068784	SRBD1
239.7619561	0.111212727	0.250330026	0.444264431	0.656851414	0.997568624	ENSG00000170234	PWWP2A
238.5475323	-0.060319433	0.26808984	-0.224997087	0.82198154	0.999467288	ENSG00000100836	PABPN1
239.1399027	0.135921316	0.271919861	0.499857992	0.617175073	0.99483168	ENSG00000175782	SLC35E3
235.7686678	0.094391078	0.278558649	0.338855311	0.734718731	0.999467288	ENSG00000139182	CLSTN3
239.4638433	0.138910732	0.247893305	0.560365	0.5752305	0.99483168	ENSG00000174579	MSL2
240.0720481	-0.984915694	0.30271474	-3.253609964	0.001139486	0.244243321	ENSG00000232320	AC009299.2
238.7530587	-0.100751517	0.249636607	-0.40359272	0.68651224	0.998869317	ENSG00000138231	DBR1
239.4689521	-0.161793775	0.321713381	-0.502912793	0.615025587	0.99483168	ENSG00000151500	THYN1
239.8154096	-0.08179053	0.249527922	-0.327781072	0.7430772	0.999467288	ENSG00000125149	C16orf70
236.5068037	-0.249376113	0.393754126	-0.633329525	0.526518477	0.99483168	ENSG00000182208	MOB2
235.3160509	0.011619016	0.362672216	0.032037238	0.974442355	0.999527751	ENSG00000136159	NUDT15
239.7764231	0.12788509	0.275524535	0.464151368	0.642539301	0.996798617	ENSG00000139620	KANSL2
238.1693639	-0.064770564	0.275581215	-0.235032581	0.814183445	0.999467288	ENSG00000176715	ACSF3
239.4788351	0.055510136	0.260265931	0.213282377	0.831106718	0.999467288	ENSG00000135801	TAF5L
237.6997602	0.186022382	0.284039238	0.654917902	0.512520614	0.993513219	ENSG00000166797	CIAO2A
238.4815434	0.667948204	0.307288493	2.173684403	0.029728845	0.773201806	ENSG00000187800	PEAR1
236.6539538	-0.24795375	0.339094298	-0.73122359	0.464642593	0.99135833	ENSG00000088543	C3orf18
237.3182987	-0.105081858	0.253302265	-0.414847683	0.678253411	0.998869317	ENSG00000153037	SRP19
240.949167	0.289110125	0.31841378	0.907969891	0.36389413	0.98048242	ENSG00000196458	ZNF605
241.3715202	-0.254843794	0.332639962	-0.766125011	0.443601922	0.99135833	ENSG00000196470	SIAH1
238.3041624	0.232453644	0.317639468	0.731815997	0.464280884	0.99135833	ENSG00000151503	NCAPD3
237.1653037	-0.340273274	0.261743439	-1.300025994	0.19359206	0.950382812	ENSG00000124659	TBCC
232.9458904	-0.002461426	0.334696585	-0.007354203	0.994132248	0.999527751	ENSG00000039650	PNKP
231.4017559	-0.167931057	0.428987701	-0.391458909	0.695458056	0.998869317	ENSG00000113578	FGF1
241.3951522	-0.100679937	0.420593568	-0.239375837	0.810814165	0.999467288	ENSG00000154359	LONRF1
239.9872578	0.156186194	0.295606892	0.528357757	0.597251051	0.99483168	ENSG00000089335	ZNF302
233.8749576	0.045668889	0.34608712	0.131957783	0.895017684	0.999467288	ENSG00000239697	TNFSF12
236.5085845	0.278364184	0.252624905	1.101887334	0.270510656	0.962465655	ENSG00000215305	VPS16
236.7545563	0.017844089	0.262623403	0.06794554	0.945828987	0.999527751	ENSG00000065615	CYB5R4
239.3561376	0.339786711	0.281193835	1.208371839	0.226904267	0.958865115	ENSG00000106144	CASP2
236.2898394	-0.096271468	0.38541771	-0.249784753	0.802753811	0.999467288	ENSG00000131080	EDA2R
237.9198531	0.424551922	0.268232702	1.582774651	0.113472836	0.923922314	ENSG00000148690	FRA10AC1
243.9155826	-0.357595363	0.424688142	-0.842018713	0.399777479	0.988384114	ENSG00000167173	C15orf39
237.2610619	-0.369507691	0.28155066	-1.312402147	0.189384483	0.949094286	ENSG00000099992	TBC1D10A
237.1855134	-0.148202539	0.280691184	-0.527991427	0.597505286	0.99483168	ENSG00000237187	NR2F1-AS1
237.2923802	-0.002311847	0.256744545	-0.009004464	0.992815574	0.999527751	ENSG00000100726	TELO2
233.7833124	0.589787402	0.435163472	1.355323781	0.175314422	0.946278819	ENSG00000176454	LPCAT4
237.4162894	-0.233727057	0.282667366	-0.826862545	0.408314975	0.990226751	ENSG00000025770	NCAPH2
237.0565727	-0.215372865	0.307789983	-0.699739684	0.484089889	0.991892545	ENSG00000167700	MFSD3
232.54146	0.144685942	0.357605826	0.404596155	0.685774385	0.998869317	ENSG00000167535	CACNB3
233.3954081	0.693169559	0.379072049	1.828595807	0.067460181	0.87420149	ENSG00000123374	CDK2
237.0358242	0.223305627	0.293919699	0.759750461	0.44740376	0.99135833	ENSG00000168434	COG7
239.9554057	0.010970647	0.320062537	0.034276573	0.972656606	0.999527751	ENSG00000100263	RHBDD3

236.0729973	-0.037915696	0.295773078	-0.128191843	0.897997155	0.999467288	ENSG00000188130	MAPK12
234.6421108	0.387205739	0.296658807	1.30522246	0.191817108	0.949261383	ENSG00000146909	NOM1
237.3467613	0.581094233	0.303528874	1.914461138	0.05556126	0.847598083	ENSG00000253797	UTP14C
237.5711903	0.141813726	0.278896631	0.508481316	0.611115839	0.99483168	ENSG00000121542	SEC22A
237.6966871	-0.206511615	0.242674188	-0.850983025	0.394778782	0.988378925	ENSG00000101457	DNTTIP1
237.9962862	0.104021094	0.2624275	0.396380311	0.691824489	0.998869317	ENSG00000185049	NELFA
235.8826757	0.293107204	0.271130344	1.081056438	0.279672009	0.963267117	ENSG00000004866	ST7
238.5056884	-0.480859803	0.29778027	-1.614814181	0.106350941	0.922165836	ENSG00000171865	RNASEH1
237.2884959	-0.705425821	0.5787926	-1.218788597	0.222924441	0.956884001	ENSG00000186469	GNG2
239.5612056	0.099188275	0.334420102	0.296597827	0.76677357	0.999467288	ENSG00000085382	HACE1
235.113068	0.00220264	0.261374766	0.008427132	0.993276201	0.999527751	ENSG00000130830	MPP1
234.3199481	-0.145824746	0.287154332	-0.50782708	0.611574617	0.99483168	ENSG00000139131	YARS2
234.4279095	-0.037223995	0.244433174	-0.152286999	0.878960578	0.999467288	ENSG00000185808	PIGP
235.8542016	-0.188952646	0.249670138	-0.756809154	0.449164208	0.99135833	ENSG00000169592	INO80E
234.9255185	-0.056263024	0.293278195	-0.191841823	0.847866109	0.999467288	ENSG00000163877	SNIP1
236.4589165	-0.304681933	0.36098154	-0.844037435	0.398648487	0.988378925	ENSG00000122783	CYREN
235.075149	1.099205608	0.313395901	3.507402635	0.000452504	0.181542056	ENSG00000138166	DUSP5
231.4609213	-0.584721962	0.31111243	-1.879455479	0.060182327	0.858996918	ENSG00000178449	COX14
237.5930478	-1.358832361	0.439564336	-3.091316219	0.001992713	0.316272012	ENSG00000099282	TSPAN15
236.7526462	-0.143111438	0.431494673	-0.331664437	0.740142657	0.999467288	ENSG00000196422	PPP1R26
230.3515752	-0.266780925	0.363596	-0.733728988	0.463113927	0.99135833	ENSG00000197903	HIST1H2BK
233.8749356	0.080867806	0.28414745	0.284598037	0.775952116	0.999467288	ENSG00000167113	COQ4
235.2875363	-0.296832067	0.266329189	-1.114530737	0.265051566	0.962465655	ENSG00000156858	PRR14
234.9937712	0.12252551	0.306432529	0.399844983	0.689270696	0.998869317	ENSG00000107951	MTPAP
234.9399953	-0.091777405	0.365436169	-0.251144831	0.801702136	0.999467288	ENSG00000167395	ZNF646
232.2921627	-0.487936199	0.340812123	-1.431686747	0.152233491	0.938052926	ENSG00000173163	COMMD1
232.8999792	-0.599266642	0.44403917	-1.349580583	0.177150556	0.946540546	ENSG00000127220	ABHD8
229.8066489	-0.58128565	0.318069562	-1.827542522	0.067618239	0.87420149	ENSG00000196976	LAGE3
232.6539793	-0.231350114	0.314390301	-0.735869118	0.461810353	0.99135833	ENSG00000169696	ASPSCR1
236.0969931	0.083075259	0.273800551	0.303415235	0.761573436	0.999467288	ENSG00000213762	ZNF134
230.9785827	0.014811627	0.364498151	0.040635672	0.967586345	0.999527751	ENSG00000157873	TNFRSF14
234.6334892	0.259309805	0.267524029	0.969295377	0.332397835	0.976026345	ENSG00000125630	POLR1B
235.489645	0.341771012	0.307460076	1.111594767	0.266312423	0.962465655	ENSG00000069943	PIGB
233.8247039	0.020266086	0.347520073	0.058316304	0.953496681	0.999527751	ENSG00000147324	MFHAS1
233.8865586	0.278747817	0.31119056	0.895746379	0.370388234	0.981455968	ENSG00000184497	TMEM255B
233.291256	-0.156827619	0.437106633	-0.358785722	0.719755394	0.99900742	ENSG00000185339	TCN2
233.0909404	0.306979059	0.267698479	1.146734414	0.251491403	0.962465655	ENSG00000119004	CYP20A1
234.9558579	-0.011970985	0.239536426	-0.049975635	0.960141805	0.999527751	ENSG00000162869	PPP1R21
233.6350777	0.428064695	0.276558727	1.547825663	0.121664276	0.923922314	ENSG00000146828	SLC12A9
231.1851909	1.341036815	0.608855433	2.202553749	0.027626217	0.769167109	ENSG00000137310	TCF19
232.5688929	-0.198549795	0.392589141	-0.50574449	0.613036032	0.99483168	ENSG00000242071	RPL7AP6
233.4342511	-0.098048442	0.242269701	-0.404707817	0.685692296	0.998869317	ENSG00000152133	GPATCH11
234.4724809	-0.427952318	0.273237043	-1.566230963	0.117294557	0.923922314	ENSG00000189319	FAM53B
227.2849364	0.530191807	0.505124287	1.049626438	0.293889897	0.970706863	ENSG00000146376	ARHGAP18
233.5343364	-0.054297555	0.262428042	-0.206904546	0.836084406	0.999467288	ENSG00000185298	CCDC137
233.1245776	0.002587042	0.30761376	0.008410035	0.993289842	0.999527751	ENSG00000115568	ZNF142
234.9931857	-0.365182738	0.314564663	-1.16091469	0.245676594	0.962465655	ENSG00000188997	KCTD21
230.4345603	0.184374853	0.414448406	0.444868048	0.656415115	0.997568624	ENSG00000072864	NDE1
231.6710536	-0.141376949	0.248853598	-0.568112937	0.569958284	0.99483168	ENSG00000183155	RABIF
235.765923	0.4249924	0.306939031	1.384615047	0.166170208	0.943223078	ENSG00000165338	HECTD2
233.8675985	-0.119525673	0.239806537	-0.498425418	0.618184225	0.99483168	ENSG00000119599	DCAF4
233.0036772	0.328183798	0.251328698	1.305795165	0.191622225	0.949261383	ENSG00000119688	ABCD4
235.2497197	0.227385475	0.311304694	0.73042739	0.465128981	0.99135833	ENSG00000043514	TRIT1
231.3397049	-0.317098046	0.244348533	-1.297728461	0.19438066	0.951409476	ENSG00000149260	CAPN5
231.2101583	-0.265412622	0.30199133	-0.878874972	0.37946907	0.983256509	ENSG00000171840	NINJ2
235.510932	0.045570741	0.289197536	0.157576519	0.874790508	0.999467288	ENSG00000090612	ZNF268
233.280661	-0.541832624	0.39293417	-1.378939949	0.16791327	0.943223078	ENSG00000231290	APCDD1L-DT
231.1286196	-0.358647834	0.307591786	-1.165986382	0.243619951	0.962465655	ENSG00000156603	MED19
230.3476759	-0.22116576	0.377658198	-0.585624148	0.558128117	0.99483168	ENSG00000197757	HOXC6
233.5695161	-0.508591205	0.358679536	-1.417954339	0.156204096	0.939464191	ENSG00000100379	KCTD17
233.0204336	-0.143922339	0.330642454	-0.435280883	0.66335856	0.997568624	ENSG00000178764	ZHX2
230.2308512	0.110079803	0.279867725	0.393327967	0.694077265	0.998869317	ENSG00000205707	ETFRF1
234.5962076	0.367199394	0.361698892	1.015207406	0.310006971	0.973427483	ENSG00000196935	SRGAP1
223.654111	0.46654091	0.547861696	0.851566944	0.394454493	0.988378925	ENSG00000196154	S100A4

227.2122066	0.389082273	0.39464917	0.98589406	0.324185102	0.975459464	ENSG00000131634	TMEM204
232.7102162	0.079001375	0.276193415	0.286036417	0.774850229	0.999467288	ENSG00000135945	REV1
224.971976	-3.063596679	0.610018204	-5.022139767	5.11E-07	0.002460075	ENSG00000133063	CHIT1
235.6402791	1.372175326	0.494475187	2.775013511	0.005519941	0.494004338	ENSG00000198563	DDX39B
230.0147775	0.008144265	0.25144944	0.032389273	0.974161617	0.999527751	ENSG00000179409	GEMIN4
233.2481977	-0.023369086	0.341934376	-0.068343774	0.945511979	0.999527751	ENSG00000140265	ZSCAN29
232.2964537	0.527862449	0.390861313	1.350510861	0.176852172	0.946540546	ENSG00000122778	KIAA1549
227.8181622	0.53899532	0.578569801	0.931599471	0.351543557	0.977691382	ENSG00000105889	STEAP1B
230.0341092	-0.132172825	0.384774	-0.343507683	0.731216562	0.999467288	ENSG00000157833	GAREM2
229.2987181	-0.028389431	0.263076373	-0.107913267	0.914064493	0.999527751	ENSG00000020426	MNAT1
231.1708029	0.194704986	0.337266809	0.577302542	0.563735097	0.99483168	ENSG00000170801	HTRA3
229.4956956	0.085118284	0.289364882	0.294155542	0.768639051	0.999467288	ENSG00000162129	CLPB
230.4403699	0.048007477	0.287082832	0.167225177	0.867192875	0.999467288	ENSG00000089048	ESF1
229.2834906	-0.248197061	0.273178018	-0.908554293	0.363585443	0.98048242	ENSG00000100124	ANKRD54
230.4003501	-0.144976369	0.248809054	-0.582681243	0.560107902	0.99483168	ENSG00000125945	ZNF436
231.6955704	0.05754748	0.30490021	0.188742016	0.85029502	0.999467288	ENSG00000149262	INTS4
232.0185702	-0.592049008	0.319203651	-1.854768914	0.063629233	0.868202292	ENSG00000120693	SMAD9
229.8544737	-0.332429881	0.244088063	-1.361926008	0.173221237	0.944422693	ENSG00000136319	TTC5
226.7553743	0.524142695	0.33007298	1.587960018	0.11229538	0.923922314	ENSG00000137767	SQOR
231.5901527	-0.074281175	0.454252747	-0.163523887	0.870105967	0.999467288	ENSG00000070444	MNT
233.2501337	0.115138698	0.322624711	0.356881212	0.721180735	0.99900742	ENSG00000132664	POLR3F
228.8977309	-0.137659261	0.264843598	-0.519775679	0.603219933	0.99483168	ENSG00000154719	MRPL39
229.2004485	0.798306805	0.32387837	2.464835192	0.013707629	0.672931175	ENSG00000144959	NCEH1
227.3115851	0.324283165	0.298427579	1.086639399	0.277196206	0.962465655	ENSG00000145016	RUBCN
230.732338	-0.017972473	0.241551226	-0.074404396	0.940688611	0.999527751	ENSG00000141084	RANBP10
232.0937564	-0.511091072	0.594847769	-0.859196417	0.39023216	0.987407688	ENSG00000117643	MAN1C1
214.135032	2.544985139	1.461705658	1.741106443	0.081664922	0.897807954	ENSG00000102760	RGCC
230.1994026	0.108980289	0.259717181	0.419611397	0.674769361	0.998869317	ENSG00000082068	WDR70
229.0082548	-0.283013345	0.267019899	-1.059896083	0.289191878	0.966513256	ENSG00000121671	CRY2
228.8807216	0.139361382	0.240333584	0.579866451	0.562004681	0.99483168	ENSG00000083535	PIBF1
227.1570649	-0.459798462	0.361593147	-1.271590642	0.203518603	0.954310328	ENSG00000162076	FLYWCH2
229.3987102	0.247104835	0.260539073	0.948436762	0.342907152	0.977691382	ENSG00000011021	CLCN6
228.6179726	0.055839302	0.257222076	0.217085962	0.828141354	0.999467288	ENSG00000167257	RNF214
227.4064501	0.300942356	0.296926743	1.013523919	0.310809977	0.973427483	ENSG00000091009	RBM27
228.2027595	0.097944792	0.265282556	0.369209319	0.71197171	0.99900742	ENSG00000104957	CCDC130
230.3512809	-0.922942941	0.457671178	-2.016607086	0.043736533	0.807325344	ENSG00000236675	MTX1P1
230.0244556	-0.004130473	0.268299208	-0.015395026	0.987717032	0.999527751	ENSG00000186130	ZBTB6
227.9786897	0.022101058	0.262001735	0.084354624	0.932774483	0.999527751	ENSG00000052749	RRP12
228.4833789	0.352544727	0.29341468	1.201523821	0.229548072	0.959575179	ENSG00000095002	MSH2
229.4369936	-0.053088065	0.284052467	-0.18689528	0.85174274	0.999467288	ENSG00000127452	FBXL12
224.4537674	-0.45165702	0.441077406	-1.023985845	0.305841961	0.973427483	ENSG00000163344	PMVK
229.0589788	-0.04728295	0.267676359	-0.176642235	0.859789417	0.999467288	ENSG00000183340	JRKL
227.5176172	0.187946536	0.265667323	0.70745071	0.479286433	0.99135833	ENSG00000167130	DOLPP1
229.9147786	-0.162073482	0.376175518	-0.430845374	0.666580805	0.997568624	ENSG00000077044	DGKD
228.2778114	0.281606208	0.296670497	0.949222154	0.342507636	0.977691382	ENSG00000155393	HEATR3
226.7636696	0.085319101	0.321370928	0.265484814	0.790636007	0.999467288	ENSG00000135698	MPHOSPH6
226.3357549	-0.154522063	0.301496871	-0.512516307	0.608289711	0.99483168	ENSG00000151689	INPP1
229.4173724	-0.069304308	0.279318079	-0.248119663	0.804041822	0.999467288	ENSG00000157869	RAB28
217.5514279	0.555464283	0.65000819	0.854549668	0.392800502	0.988378925	ENSG00000197442	MAP3K5
227.3846584	-0.008867806	0.435510459	-0.020361868	0.983754702	0.999527751	ENSG00000160972	PPP1R16A
227.2894901	-0.196393722	0.244331829	-0.803799171	0.421512969	0.991301167	ENSG00000167862	MRPL58
225.6148777	-0.048147768	0.496398036	-0.096994275	0.922730941	0.999527751	ENSG00000049130	KITLG
230.4825412	-0.065112883	0.373667712	-0.174253436	0.861666292	0.999467288	ENSG00000198855	FICD
225.5359056	0.209825424	0.271552263	0.772688916	0.439706508	0.991301167	ENSG00000101493	ZNF516
228.0681125	0.490733315	0.315386179	1.555975967	0.119713807	0.923922314	ENSG00000271383	NBPF19
228.0262982	-0.336826139	0.403003018	-0.835790612	0.403272709	0.988927059	ENSG00000186310	NAP1L3
225.6395028	-0.06678487	0.269192038	-0.248093777	0.80406185	0.999467288	ENSG00000102030	NAA10
226.4945995	-0.041001543	0.256505285	-0.159846775	0.873001777	0.999467288	ENSG00000105229	PIAS4
227.2972926	-0.336572193	0.292603458	-1.150267309	0.250033791	0.962465655	ENSG00000167685	ZNF444
229.7536743	-0.045682673	0.307666448	-0.148481166	0.881963049	0.999467288	ENSG00000117481	NSUN4
228.4945161	-0.078693211	0.309631573	-0.254151118	0.799378813	0.999467288	ENSG00000164252	AGGF1
226.4748597	0.209110121	0.286294916	0.730401096	0.465145048	0.99135833	ENSG00000141127	PRPSAP2
226.576421	-0.456568897	0.365997086	-1.247465935	0.212226703	0.954310328	ENSG00000184436	THAP7
225.5031861	0.313600676	0.565624056	0.55443306	0.579282506	0.99483168	ENSG00000168955	TM4SF20

224.764739	-0.173270546	0.269170522	-0.643720364	0.519756782	0.993513219	ENSG00000108669	CYTH1
227.318464	-0.311303889	0.300039074	-1.037544494	0.299482171	0.972186492	ENSG00000168795	ZBTB5
227.059946	0.081837636	0.280997284	0.291239954	0.770867807	0.999467288	ENSG00000156232	WHAMM
221.3436751	0.392536812	0.297090763	1.321268991	0.18641169	0.949094286	ENSG00000173801	JUP
226.6623869	-0.259408718	0.286672044	-0.904897158	0.365519873	0.981269214	ENSG00000065029	ZNF76
224.9617867	-0.426027273	0.280943123	-1.516418229	0.129413633	0.928627271	ENSG00000243335	KCTD7
227.252368	0.390520321	0.275643228	1.416760077	0.156553086	0.939464191	ENSG00000051009	FAM160A2
224.7757938	-0.511773291	0.388987957	-1.315653306	0.188290433	0.949094286	ENSG00000234975	FTH1P2
227.0127965	0.122318061	0.298078051	0.410355813	0.681544955	0.998869317	ENSG00000215251	FASTKD5
228.4139516	0.399552206	0.320834755	1.245352005	0.213002387	0.954310328	ENSG00000134698	AGO4
225.9257121	-0.837256838	1.072115613	-0.780938947	0.434838404	0.991301167	ENSG00000083782	EPYC
224.2824701	-0.202960327	0.259942217	-0.780790167	0.434925918	0.991301167	ENSG00000196943	NOP9
230.2928154	-0.062284557	0.458930269	-0.13571682	0.892045151	0.999467288	ENSG00000059728	MXD1
224.0309596	0.028349389	0.295841688	0.095826214	0.923658597	0.999527751	ENSG00000180011	ZADH2
223.6887727	-0.331420644	0.276586292	-1.198254047	0.23081813	0.959575179	ENSG00000157870	PRXL2B
221.899298	0.245026438	0.535338548	0.457703707	0.647165324	0.997368416	ENSG00000171408	PDE7B
225.7234529	-0.011061728	0.262808911	-0.042090384	0.966426646	0.999527751	ENSG00000103091	WDR59
225.2595155	-0.33308671	0.27500892	-1.211185112	0.225824467	0.958865115	ENSG00000101190	TCFL5
221.9691687	-0.146599769	0.292337718	-0.501474012	0.616037563	0.99483168	ENSG00000213445	SIPA1
227.3521011	-0.080973477	0.298548274	-0.271224066	0.786218704	0.999467288	ENSG00000116922	C1orf109
224.3278582	-0.226206956	0.311425355	-0.726360112	0.467618038	0.99135833	ENSG00000170899	GSTA4
225.5998782	0.29248925	0.242928334	1.204014555	0.228583953	0.95944145	ENSG00000167766	ZNF83
224.9461604	-0.110700427	0.27927747	-0.396381515	0.6918236	0.998869317	ENSG00000138785	INTS12
222.5743112	-0.105065687	0.2639131	-0.39810713	0.690551219	0.998869317	ENSG00000112651	MRPL2
226.095715	-0.209631625	0.36947886	-0.567371095	0.570462084	0.99483168	ENSG00000165312	OTUD1
222.6884302	0.554036957	0.482038863	1.149361597	0.250406908	0.962465655	ENSG00000131876	SNRPA1
224.8323323	0.002987326	0.256768529	0.011634317	0.990717367	0.999527751	ENSG00000144736	SHQ1
224.332732	-0.006460307	0.27481228	-0.023508074	0.981244998	0.999527751	ENSG00000143156	NME7
221.6235505	0.011222142	0.315425121	0.035577834	0.971618983	0.999527751	ENSG00000131495	NDUFA2
225.0089326	-0.319229596	0.394723434	-0.808742449	0.418663306	0.990226751	ENSG00000180346	TIGD2
225.3870156	0.747756461	0.30631992	2.44109642	0.014642745	0.686410066	ENSG00000137504	CREBZF
222.9539901	-0.106679342	0.310361045	-0.343726587	0.731051915	0.999467288	ENSG00000094841	UPRT
223.1221589	-0.199224572	0.292415579	-0.681306284	0.495677708	0.993513219	ENSG00000144843	ADPRH
224.9855113	-0.209049825	0.267483069	-0.781544139	0.434482527	0.991301167	ENSG00000185010	F8
220.8026767	-0.3866789	0.418891544	-0.923100275	0.355954951	0.978503494	ENSG00000002330	BAD
222.454141	-0.049994669	0.336916168	-0.148389047	0.882035744	0.999467288	ENSG00000139343	SNRPF
225.9399195	0.619654889	0.420050652	1.475190878	0.140161233	0.935644597	ENSG00000260822	AC004656.1
224.3455836	0.402543601	0.347033495	1.159956045	0.246066703	0.962465655	ENSG00000162664	ZNF326
223.1358289	-0.334082046	0.264756518	-1.261846352	0.20700408	0.954310328	ENSG00000090971	NAT14
225.445948	-0.12598284	0.279126267	-0.451347133	0.65173938	0.997449432	ENSG00000146072	TNFRSF21
223.4507708	-0.158920139	0.330821456	-0.480380386	0.630956939	0.99483168	ENSG00000166813	KIF7
223.6875405	-0.04611225	0.27578835	-0.167201587	0.867211436	0.999467288	ENSG00000119328	FAM206A
224.6297202	0.097752408	0.320315511	0.30517538	0.760232583	0.999467288	ENSG00000176102	CSTF3
223.6755621	0.07683979	0.381855985	0.201227147	0.840520966	0.999467288	ENSG00000075188	NUP37
226.5051566	0.003139091	0.300956905	0.010430368	0.991677921	0.999527751	ENSG00000120942	UBIAD1
222.1218241	0.400430111	0.287724225	1.39171497	0.164008727	0.943223078	ENSG00000174839	DENND6A
236.1703346	-2.385860542	1.34412961	-1.775022679	0.075894143	0.897052166	ENSG00000128045	RASL11B
223.7245785	0.130464102	0.279000134	0.467613046	0.640061334	0.996553586	ENSG00000185716	MOSMO
223.0800188	-0.361290475	0.32174467	-1.122910523	0.261475498	0.962465655	ENSG00000109083	IFT20
220.9347013	0.081803086	0.305876739	0.267438073	0.789131889	0.999467288	ENSG00000184470	TXNRD2
223.2169614	0.12849986	0.452118006	0.284217524	0.776243687	0.999467288	ENSG00000144857	BOC
223.1268332	0.131379481	0.268356764	0.489570224	0.624438051	0.99483168	ENSG00000229119	AC026403.1
223.9538754	1.418720213	1.048876451	1.352609464	0.176180427	0.946278819	ENSG00000138160	KIF11
223.678702	0.560064198	0.287118622	1.950636968	0.051100246	0.842188482	ENSG00000185324	CDK10
223.9438744	0.06943185	0.307843824	0.225542449	0.821557306	0.999467288	ENSG00000188807	TMEM201
224.5702556	-0.636394187	0.338486149	-1.880118842	0.060091883	0.858996918	ENSG00000149761	NUDT22
223.9862231	-0.437886882	0.358398866	-1.221786459	0.221788391	0.956884001	ENSG00000171160	MORN4
219.3262372	0.283088023	0.305780312	0.925788913	0.35455569	0.977691382	ENSG00000165819	METTL3
220.4462224	-0.25105741	0.306525855	-0.819041545	0.412762711	0.990226751	ENSG00000183513	COA5
223.6261947	-0.325592123	0.356973638	-0.912090107	0.361721286	0.979465649	ENSG00000123545	NDUFAF4
222.6850367	-0.040010437	0.277564471	-0.144148264	0.885383393	0.999467288	ENSG00000196177	ACADSB
219.6793385	-0.293810517	0.33625101	-0.873783301	0.382236276	0.98555814	ENSG00000150977	RILPL2
224.3998174	-0.38861291	0.368657611	-1.054129627	0.291823574	0.97025964	ENSG00000177946	CENPBD1
222.7008474	-0.014847087	0.275673784	-0.053857451	0.957048736	0.999527751	ENSG00000124688	MAD2L1BP

223.8496805	-0.047843521	0.302226258	-0.158303655	0.874217528	0.999467288	ENSG00000178381	ZFAND2A
220.6206835	-0.076825448	0.298179994	-0.257647895	0.796678662	0.999467288	ENSG00000131089	ARHGEF9
222.454568	-0.175153655	0.297415721	-0.588918617	0.555915868	0.99483168	ENSG00000103494	RPGRIP1L
223.5207138	-0.033850857	0.32021684	-0.105712295	0.915810625	0.999527751	ENSG00000112624	BICRAL
220.361473	-0.510013366	0.283666625	-1.797932225	0.072187749	0.885710197	ENSG00000139405	RITA1
224.3278831	-0.334366551	0.359949667	-0.928925852	0.352927514	0.977691382	ENSG00000059145	UNKL
222.0239113	0.0284758	0.316804071	0.089884577	0.928378937	0.999527751	ENSG00000145214	DGKQ
221.5979716	-0.554448579	0.350018748	-1.584053943	0.113181444	0.923922314	ENSG00000198680	TUSC1
221.7998614	0.111730208	0.290145426	0.385083472	0.700175573	0.998869317	ENSG00000136104	RNASEH2B
222.2986936	-0.003804906	0.26038817	-0.014612437	0.988341377	0.999527751	ENSG00000163788	SNRK
222.1863831	-0.17134347	0.306652717	-0.558754123	0.576329536	0.99483168	ENSG00000248905	FMN1
221.4348499	-0.107161898	0.335518864	-0.319391572	0.749429601	0.999467288	ENSG00000178567	EPM2AIP1
219.0955275	0.61260362	0.299068037	2.048375435	0.040523227	0.796070921	ENSG00000174136	RGMB
222.5381119	1.651301055	0.60015038	2.751478811	0.005932686	0.499617804	ENSG00000013810	TACC3
217.7420295	-0.33555307	0.332021409	-1.010636847	0.312190273	0.973427483	ENSG00000108479	GALK1
219.3360459	-0.219750272	0.300950915	-0.73018642	0.465276242	0.99135833	ENSG00000175854	SWI5
220.2169629	-0.371538847	0.290723566	-1.277979804	0.201256547	0.954310328	ENSG00000156831	NSMCE2
221.3365103	0.971484188	0.459033355	2.116369492	0.034313395	0.782919999	ENSG00000176438	SYNE3
219.1385252	0.843487913	0.378123926	2.230718175	0.025699803	0.760619373	ENSG00000168496	FEN1
221.4041695	0.321931444	0.296945423	1.08414348	0.278301187	0.96275546	ENSG00000110888	CAPRIN2
219.1977104	0.129920489	0.283219053	0.458727926	0.646429557	0.997368416	ENSG00000169379	ARL13B
223.3188764	-0.231659832	0.422224853	-0.548664605	0.583235641	0.99483168	ENSG00000085644	ZNF213
213.272691	1.167878019	0.578836715	2.017629479	0.043629864	0.807325344	ENSG00000154319	FAM167A
220.3788938	-0.350323593	0.241453208	-1.450896415	0.146808706	0.936396974	ENSG00000122085	MTERF4
217.8212768	0.194936252	0.294618046	0.661657542	0.508190721	0.993513219	ENSG00000166979	EVA1C
220.4374078	0.337627116	0.283615396	1.190440013	0.233873494	0.959884875	ENSG00000128159	TUBGCP6
219.6384408	0.275280636	0.337009411	0.816833677	0.414023484	0.990226751	ENSG00000053900	ANAPC4
222.1381491	0.327351659	0.325390739	1.006026355	0.314402892	0.974768742	ENSG00000187240	DYNC2H1
218.9922175	-0.220940371	0.409843818	-0.539084308	0.589828683	0.99483168	ENSG00000189337	KAZN
220.716017	0.479078406	0.289054207	1.657399869	0.097438658	0.921147998	ENSG00000162408	NOL9
222.2246143	0.522793821	1.047446407	0.499112716	0.61769998	0.99483168	ENSG00000247095	MIR210HG
212.3655438	-0.444563957	0.454605593	-0.977911322	0.328118185	0.976026345	ENSG00000129757	CDKN1C
212.573863	0.378346076	0.520809717	0.726457405	0.467558411	0.99135833	ENSG00000120437	ACAT2
217.3701014	-0.369593252	0.259842903	-1.42237193	0.154918307	0.939432741	ENSG00000158467	AHCYL2
217.8823533	0.309194126	0.278724468	1.109318204	0.267292934	0.962465655	ENSG00000112659	CUL9
218.178974	0.256960841	0.252248733	1.018680401	0.30835472	0.973427483	ENSG00000116903	EXOC8
214.2061208	-0.512743813	0.368350325	-1.392000435	0.163922266	0.943223078	ENSG00000203778	FAM229B
217.9270625	1.355340956	0.587663479	2.306321566	0.021092671	0.73997555	ENSG00000123975	CKS2
222.9482622	-0.314382678	0.355686622	-0.883875464	0.376763443	0.982609538	ENSG00000106948	AKNA
216.9996661	-0.262535491	0.342248212	-0.767090905	0.443027469	0.99135833	ENSG00000156697	UTP14A
216.5548245	-0.568978102	0.413107013	-1.377314071	0.168415167	0.943223078	ENSG00000219507	FTH1P8
217.6230228	-0.381754958	0.284466199	-1.342004638	0.179594492	0.947364225	ENSG00000114767	RRP9
217.5112181	0.119414081	0.425000706	0.280973842	0.778730463	0.999467288	ENSG00000196083	IL1RAP
218.0000616	-0.061563136	0.268320129	-0.229439126	0.818527629	0.999467288	ENSG00000228794	LINC01128
218.1647357	0.011535344	0.275461618	0.04187641	0.966597222	0.999527751	ENSG00000159788	RGS12
216.6688899	-0.197284141	0.251120833	-0.785614395	0.432093429	0.991301167	ENSG00000256269	HMBS
218.3067007	-0.107104437	0.285739862	-0.374831975	0.707785432	0.99900742	ENSG00000130818	ZNF426
215.9703867	-0.05675835	0.307464556	-0.184601279	0.853541783	0.999467288	ENSG00000114626	ABTB1
215.9222749	0.006181655	0.324064797	0.019075368	0.984780982	0.999527751	ENSG00000172375	C2CD2L
216.0028057	-0.031984961	0.257942249	-0.124000473	0.901314901	0.999527751	ENSG00000110171	TRIM3
217.1498663	-0.318970904	0.301541081	-1.057802483	0.290145501	0.967933358	ENSG00000215256	DHRS4-AS1
214.083493	0.377036867	0.346651905	1.087652662	0.276748473	0.962465655	ENSG00000141298	SSH2
219.7848576	0.428970216	0.343494529	1.248841481	0.211723059	0.954310328	ENSG00000167555	ZNF528
215.392162	0.108701161	0.401364501	0.270829036	0.786522526	0.999467288	ENSG00000114395	CYB561D2
216.9281511	0.096658604	0.252935815	0.382146769	0.702352503	0.998869317	ENSG00000175595	ERCC4
216.3848629	-0.609349181	0.295118609	-2.064760282	0.038945688	0.793985738	ENSG00000100271	TTLL1
218.1187507	0.651097097	0.294143639	2.213534516	0.026860816	0.765286378	ENSG00000162601	MYSM1
217.4272497	-0.069835035	0.278958583	-0.250341948	0.802322919	0.999467288	ENSG00000213380	COG8
213.5560758	-0.03958421	0.526735099	-0.075150128	0.940095264	0.999527751	ENSG00000048342	CC2D2A
215.9134552	-0.11552088	0.27184114	-0.42495731	0.670867795	0.997568624	ENSG00000198839	ZNF277
216.6557345	-0.094872844	0.268526941	-0.353308474	0.723857194	0.999456245	ENSG00000140403	DNAJA4
217.3373424	0.192419771	0.29805782	0.645578671	0.518552254	0.993513219	ENSG00000064652	SNX24
214.5350835	-0.073581106	0.293183702	-0.250972702	0.801835215	0.999467288	ENSG00000172113	NME6
216.646972	0.12874837	0.361088871	0.356555908	0.721424291	0.99900742	ENSG00000126870	WDR60

219.0233612	0.184272294	0.432574443	0.425989786	0.670115289	0.997568624	ENSG00000103942	HOMER2
214.3037923	-0.115358493	0.279907363	-0.412130969	0.680243431	0.998869317	ENSG00000173914	RBM4B
214.5557583	0.626151814	0.318082057	1.968522901	0.049007904	0.835680229	ENSG00000182700	IGIP
213.302669	-0.176527077	0.311524051	-0.566656334	0.570947693	0.99483168	ENSG00000205978	NYNRIN
210.2717558	-0.531116394	0.440734267	-1.205071702	0.228175623	0.958865115	ENSG00000187608	ISG15
214.8181953	-0.121003016	0.288733506	-0.419082003	0.675156203	0.998869317	ENSG00000117151	CTBS
214.3894023	-0.303180123	0.258350576	-1.173522151	0.240586479	0.962465655	ENSG00000161036	LRWD1
214.1237191	0.090383148	0.254544054	0.355078607	0.722530703	0.999092366	ENSG00000119392	GLE1
214.1656622	0.313412795	0.247070978	1.268513189	0.204614744	0.954310328	ENSG00000095485	CWF19L1
214.322701	-0.413423018	0.343302651	-1.204252332	0.228492065	0.95933456	ENSG00000186162	CIDECP
214.5503523	0.512122686	0.300463113	1.704444455	0.088298085	0.910413547	ENSG00000105127	AKAP8
215.4867156	0.384444881	0.275779361	1.394030648	0.16330835	0.943223078	ENSG00000119684	MLH3
214.2695377	-0.055171235	0.263977319	-0.208999908	0.83444831	0.999467288	ENSG00000128534	LSM8
214.3260802	-0.367054853	0.294668749	-1.245652466	0.212892012	0.954310328	ENSG00000172830	SSH3
215.2654978	-0.086502247	0.257758576	-0.33559406	0.737177004	0.999467288	ENSG00000124784	RIOK1
216.6235227	0.132831869	0.306767199	0.433005451	0.66501081	0.997568624	ENSG00000070718	AP3M2
214.3429328	0.055315822	0.354678515	0.155960453	0.876064194	0.999467288	ENSG00000280798	LINC00294
209.2482169	0.089674923	0.424543999	0.211226453	0.83271057	0.999467288	ENSG00000109062	SLC9A3R1
215.1733201	0.03778473	0.280634096	0.134640555	0.892896076	0.999467288	ENSG00000122484	RPAP2
211.9080111	-0.358195933	0.277291953	-1.291764617	0.196438672	0.954310328	ENSG00000051596	THOC3
213.2438426	-0.138339196	0.248503576	-0.556688956	0.577739964	0.99483168	ENSG00000273611	ZNHIT3
213.4900294	0.169949628	0.267990276	0.634163414	0.525974181	0.99483168	ENSG00000240857	RDH14
213.0779197	-0.004288982	0.247310086	-0.017342529	0.986163358	0.999527751	ENSG00000105287	PRKD2
211.750104	-0.3498878	0.46844423	-0.746914525	0.455115158	0.99135833	ENSG00000137070	IL11RA
210.4433986	-0.167626231	0.367718206	-0.455855131	0.648494156	0.997368416	ENSG00000161513	FDXR
211.174061	0.91678588	0.445562674	2.057591297	0.039629383	0.793985738	ENSG00000121957	GPSM2
210.5782664	-0.282553616	0.334286106	-0.845244869	0.397974135	0.988378925	ENSG00000120656	TAF12
209.5874884	-0.236790233	0.32848289	-0.720860174	0.470995548	0.99135833	ENSG00000183172	SMDT1
214.3187142	0.231908212	0.294800772	0.786660801	0.431480456	0.991301167	ENSG00000164338	UTP15
214.0955392	-0.394629453	0.334149558	-1.180996482	0.237604121	0.962465655	ENSG00000169083	AR
216.4738727	0.716600998	0.336735061	2.12808549	0.033329996	0.775963115	ENSG00000120699	EXOSC8
212.5755968	0.023077415	0.48403354	0.047677305	0.961973422	0.999527751	ENSG00000138759	FRAS1
213.1362403	-0.039168293	0.352208012	-0.111207841	0.911451534	0.999527751	ENSG00000124104	SNX21
214.5397758	0.123347413	0.29866008	0.413002679	0.679604651	0.998869317	ENSG00000197024	ZNF398
209.2991063	-0.185633683	0.38146939	-0.486627991	0.626521983	0.99483168	ENSG00000139428	MMAB
212.1019129	0.123237274	0.24689043	0.49915776	0.61766825	0.99483168	ENSG00000013523	ANGEL1
209.9099296	1.820602565	0.470993622	3.865450574	0.000110884	0.072795545	ENSG00000162545	CAMK2N1
212.2897849	0.26190722	0.244598058	1.070765735	0.284274777	0.963310634	ENSG00000087206	UIMC1
211.4442314	-0.489715646	0.301299627	-1.625344346	0.104089207	0.92169898	ENSG00000168904	LRRC28
213.7166158	2.315438981	1.671460572	1.385278852	0.165967218	0.943223078	ENSG00000171241	SHCBP1
213.2499315	-0.493724959	0.329448153	-1.498642365	0.133966436	0.933741583	ENSG00000124313	IQSEC2
213.5390811	-0.030899492	0.283669662	-0.108927729	0.913259813	0.999527751	ENSG00000240225	ZNF542P
212.778617	-0.075549118	0.29514867	-0.255969705	0.79797423	0.999467288	ENSG00000229180	AC006001.3
211.2907129	-0.1994969	0.276515143	-0.721468265	0.470621459	0.99135833	ENSG00000100410	PHF5A
213.2232522	-0.505428616	0.259152662	-1.950312267	0.051138911	0.842188482	ENSG00000113838	TBCCD1
213.2018135	-0.339033312	0.291353787	-1.163648208	0.244566606	0.962465655	ENSG00000179967	PPP1R14BP3
213.0124174	0.012469126	0.280357986	0.04447573	0.964525198	0.999527751	ENSG00000168096	ANKS3
211.3532891	0.03012237	0.257064041	0.117178467	0.90671863	0.999527751	ENSG00000181038	METTL23
208.014485	0.086182868	0.497312402	0.173297242	0.862417792	0.999467288	ENSG00000151388	ADAMTS12
218.5487548	0.265784011	0.481019939	0.552542606	0.580576652	0.99483168	ENSG00000210144	MT-TY
213.983571	0.080852217	0.407270427	0.198522191	0.842636524	0.999467288	ENSG00000121039	RDH10
214.4942797	-0.112134309	0.472934071	-0.23710347	0.81257652	0.999467288	ENSG00000123610	TNFAIP6
212.0266207	-0.329151521	0.268418106	-1.22626423	0.220099257	0.956884001	ENSG00000112167	SAYSD1
211.2492737	-0.060004241	0.280589315	-0.213850769	0.830663433	0.999467288	ENSG00000166169	POLL
211.7981464	-0.066527981	0.291557752	-0.228181143	0.81950542	0.999467288	ENSG00000165868	HSPA12A
210.6333809	0.135804913	0.269246014	0.504389687	0.613987564	0.99483168	ENSG00000183298	RPSAP19
212.8637338	0.324617876	0.299273981	1.08468459	0.278061375	0.962465655	ENSG00000174428	GTF2IRD2B
213.2122348	-0.113568177	0.306539494	-0.37048465	0.711021411	0.99900742	ENSG00000196449	YRDC
210.697173	0.094125501	0.289247433	0.325415164	0.74486688	0.999467288	ENSG00000230733	AC092171.2
208.9779759	-0.112343662	0.318942538	-0.352237938	0.724659828	0.999456245	ENSG00000172590	MRPL52
211.2144686	0.128646503	0.391123429	0.328915359	0.742219665	0.999467288	ENSG00000138604	GLCE
210.5046121	-0.188993174	0.414946129	-0.455464363	0.6487752	0.997368416	ENSG00000133943	DGLUCY
211.2189366	0.17580261	0.254884594	0.689734155	0.490361383	0.993513219	ENSG00000176953	NFATC2IP
209.6882704	-0.211373937	0.46572681	-0.453858212	0.649930882	0.997381078	ENSG00000090530	P3H2

207.0637064	-0.040514085	0.351583135	-0.1152333	0.908260205	0.999527751	ENSG00000168310	IRF2
211.0682485	-0.118756621	0.25030694	-0.474443979	0.635183337	0.995506752	ENSG00000150756	FAM173B
208.8384098	-0.037903199	0.278409891	-0.136141712	0.891709253	0.999467288	ENSG00000151065	DCP1B
211.5577386	0.492795355	0.26697636	1.845838917	0.064915598	0.868202292	ENSG00000120798	NR2C1
208.7513611	0.007343797	0.316396897	0.023210711	0.981482195	0.999527751	ENSG00000090470	PDCD7
209.2900701	-0.031379615	0.31776345	-0.098751494	0.921335582	0.999527751	ENSG00000183826	BTBD9
210.4453048	-0.072526508	0.3218412	-0.225348736	0.821707989	0.999467288	ENSG00000175376	EIF1AD
211.7836888	0.283169241	0.335417326	0.84422962	0.398541106	0.988378925	ENSG00000138399	FASTKD1
208.4400329	-0.149689458	0.286378726	-0.522697549	0.601184744	0.99483168	ENSG00000234616	JRK
212.9936374	-0.205541458	0.320559993	-0.641194979	0.521396011	0.993734837	ENSG00000172007	RAB33B
209.5023086	-0.074230661	0.304599996	-0.243698826	0.807464082	0.999467288	ENSG00000105393	BABAM1
208.820777	-0.292973675	0.291802874	-1.004012301	0.315372685	0.974768742	ENSG00000237190	CDKN2AIPNL
210.1794787	0.103022145	0.334196461	0.30826821	0.757878253	0.999467288	ENSG00000219481	NBPF1
209.5038723	-0.260283376	0.313628897	-0.829908782	0.406590359	0.990226751	ENSG00000143374	TARS2
211.075515	0.062341441	0.278223765	0.224069432	0.822703278	0.999467288	ENSG00000164877	MICALL2
210.1476799	0.317023255	0.287862533	1.101300862	0.270765736	0.962465655	ENSG00000149308	NPAT
213.0796273	-0.12791279	0.322978023	-0.396041776	0.69207421	0.998869317	ENSG00000198538	ZNF28
209.8395188	0.158567103	0.265016447	0.598329293	0.549620235	0.99483168	ENSG00000176542	USF3
209.9248327	-0.393446575	0.260094056	-1.51270883	0.130353636	0.929373153	ENSG00000145332	KLHL8
212.4826634	0.177255833	0.372353813	0.476041406	0.634044879	0.99544042	ENSG00000184205	TSPYL2
213.0509041	0.203370052	0.412122893	0.493469436	0.621680931	0.99483168	ENSG00000180921	FAM83H
207.7352275	-0.029654421	0.305606683	-0.097034596	0.922698921	0.999527751	ENSG00000112031	MTRF1L
208.1510645	0.168288986	0.299299953	0.562275351	0.573928429	0.99483168	ENSG00000182504	CEP97
210.1477917	-1.067125613	0.462179683	-2.308897712	0.020949258	0.73997555	ENSG00000132003	ZSWIM4
204.6277522	-0.31742821	0.375816055	-0.844637171	0.398313448	0.988378925	ENSG00000109743	BST1
210.2621903	-0.030595248	0.29841983	-0.10252418	0.918340621	0.999527751	ENSG00000112592	TBP
210.8879726	-0.207352589	0.279401129	-0.742132254	0.45800721	0.99135833	ENSG00000171100	MTM1
209.2351498	-0.017264539	0.346341322	-0.049848337	0.960243247	0.999527751	ENSG00000198690	FAN1
208.2380609	0.33512999	0.340567037	0.984035311	0.325098151	0.976026345	ENSG00000118503	TNFAIP3
209.2133987	-0.199392646	0.401151371	-0.49705089	0.619153165	0.99483168	ENSG00000172379	ARNT2
206.2082958	0.060517278	0.353199982	0.171339979	0.863956447	0.999467288	ENSG00000116096	SPR
208.2376668	-0.144051651	0.25645438	-0.56170478	0.574317177	0.99483168	ENSG00000176261	ZBTB8OS
207.3582464	-0.068393606	0.329669404	-0.207461187	0.835649701	0.999467288	ENSG00000172530	BANP
207.6417554	-0.048427097	0.294627578	-0.164367155	0.869442119	0.999467288	ENSG00000119559	C19orf25
210.6400159	1.251530251	0.660550433	1.894677816	0.058135099	0.852621724	ENSG00000198826	ARHGAP11A
204.1729401	0.243829477	0.495681322	0.491907736	0.622784569	0.99483168	ENSG00000044524	EPHA3
206.8814625	0.145371075	0.24992536	0.581657959	0.560797095	0.99483168	ENSG00000166140	ZFYVE19
207.5004538	2.142910652	1.489436949	1.438738748	0.150224556	0.936396974	ENSG00000101057	MYBL2
209.6068909	-0.149940234	0.26614761	-0.563372463	0.573181284	0.99483168	ENSG00000147679	UTP23
207.3398115	-0.275391424	0.377636837	-0.729249367	0.465849138	0.99135833	ENSG00000144115	THNSL2
205.2802547	0.247563914	0.333406713	0.742528283	0.457767322	0.99135833	ENSG00000078070	MCCC1
207.3449628	0.123498135	0.309825736	0.398605155	0.690184163	0.998869317	ENSG00000075914	EXOSC7
207.0638685	-0.092816154	0.308506413	-0.300856481	0.763523937	0.999467288	ENSG00000233762	AC007969.1
210.5747388	0.155113514	0.294492214	0.526715163	0.598391405	0.99483168	ENSG00000173226	IQCB1
205.8728762	-0.181153628	0.270153849	-0.670557274	0.502502609	0.993513219	ENSG00000162222	TTC9C
208.2662558	0.19762947	0.329986514	0.598901656	0.549238467	0.99483168	ENSG00000278259	MYO19
207.6171019	0.306654202	0.308387356	0.994379946	0.320037928	0.974964731	ENSG00000135249	RINT1
207.646931	0.003138361	0.250241209	0.012541344	0.989993718	0.999527751	ENSG00000130305	NSUN5
207.6334779	0.023717603	0.27329205	0.086784827	0.930842549	0.999527751	ENSG00000122965	RBM19
207.3790152	-0.304269869	0.334769061	-0.908894831	0.363405643	0.98048242	ENSG00000178585	CTNNBIP1
205.0457537	-0.444210494	0.27521471	-1.614050694	0.106516431	0.922165836	ENSG00000156973	PDE6D
206.8857591	-0.330404544	0.324759311	-1.017382819	0.308971352	0.973427483	ENSG00000011523	CEP68
207.5929069	-0.258100071	0.328367244	-0.786010408	0.431861389	0.991301167	ENSG00000126746	ZNF384
207.3379262	0.014588476	0.254296221	0.057368043	0.954252019	0.999527751	ENSG00000141219	C17orf80
208.0411089	0.359141992	0.304284042	1.180285333	0.23788675	0.962465655	ENSG00000121931	LRIF1
203.6356625	-0.274278978	0.352777565	-0.777484186	0.436873161	0.991301167	ENSG00000143545	RAB13
205.3800319	-0.102278	0.371795715	-0.275091929	0.783245612	0.999467288	ENSG00000060762	MPC1
207.3183034	-0.027102476	0.302688101	-0.089539284	0.928653335	0.999527751	ENSG00000167333	TRIM68
207.2033865	-0.039646228	0.257097606	-0.154206912	0.877446593	0.999467288	ENSG00000151498	ACAD8
207.4046534	0.253830165	0.258489978	0.981972946	0.326113177	0.976026345	ENSG00000148187	MRRF
206.1182807	-0.528068777	0.407805795	-1.294902584	0.195353837	0.953843167	ENSG00000100246	DNAL4
200.365702	0.678567807	0.613836855	1.105453021	0.26896334	0.962465655	ENSG00000196730	DAPK1
205.7744533	-0.087089236	0.265641118	-0.327845467	0.743028508	0.999467288	ENSG00000139146	SINHCAF
208.5109022	0.16793462	0.303795005	0.552789275	0.580407714	0.99483168	ENSG00000156983	BRPF1

206.2915853	-0.250596341	0.332992493	-0.752558532	0.451715244	0.99135833	ENSG00000178149	DALRD3
203.6573953	0.351637627	0.38393902	0.915868428	0.359735908	0.979465649	ENSG00000138771	SHROOM3
205.7911308	0.199592691	0.319088637	0.625508616	0.531637296	0.99483168	ENSG00000158156	XKR8
206.3452731	-0.081701794	0.305204803	-0.267694981	0.788934114	0.999467288	ENSG00000100575	TIMM9
203.9464158	-0.25406758	0.370507245	-0.685729046	0.492883987	0.993513219	ENSG00000198130	HIBCH
206.0878435	-0.210191941	0.268112975	-0.783967805	0.433059001	0.991301167	ENSG00000284024	HSPA14
204.1415759	0.266775316	0.305449906	0.873384835	0.382453354	0.98555814	ENSG00000166562	SEC11C
205.8036513	-0.144590293	0.293955131	-0.491878786	0.622805036	0.99483168	ENSG00000162636	FAM102B
205.7601161	0.232011956	0.273404808	0.848602326	0.396102612	0.988378925	ENSG00000188735	TMEM120B
205.7623976	0.162158776	0.266644202	0.608146643	0.54309022	0.99483168	ENSG00000115421	PAPOLG
203.0603741	-0.004755912	0.51906526	-0.009162454	0.992689522	0.999527751	ENSG00000026950	BTN3A1
205.3203255	-0.256585949	0.258840121	-0.991291257	0.321543381	0.975337095	ENSG00000118707	TGIF2
208.460107	-0.155695743	0.344577343	-0.451845562	0.651380244	0.997449432	ENSG00000189114	BLOC1S3
203.0064247	-0.479696026	0.310963519	-1.542611904	0.122924968	0.926620728	ENSG00000105619	TFPT
206.6531827	-0.204184819	0.289053249	-0.706391709	0.479944575	0.99135833	ENSG00000154642	C21orf91
205.1761421	-0.12849089	0.406596118	-0.316016026	0.751990344	0.999467288	ENSG00000269893	SNHG8
204.3717734	-0.178780877	0.302149692	-0.591696373	0.554053926	0.99483168	ENSG00000103245	CIAO3
208.5816795	-0.204909149	0.354244019	-0.578440675	0.56296664	0.99483168	ENSG00000125821	DTD1
193.1770017	0.741209932	2.401563307	0.308636433	0.757598104	0.999467288	ENSG00000107159	CA9
205.4884131	0.225201573	0.264505886	0.851404772	0.394544542	0.988378925	ENSG00000147912	FBXO10
205.7804997	-0.314892481	0.34400467	-0.915372693	0.359996009	0.979465649	ENSG00000122970	IFT81
204.5008628	-0.497451526	0.349341881	-1.423967618	0.154455846	0.939432741	ENSG00000110011	DNAJC4
206.5933942	-0.026685278	0.319182765	-0.08360501	0.933370484	0.999527751	ENSG00000100105	PATZ1
206.8595907	0.337124408	0.307690388	1.095661161	0.273227086	0.962465655	ENSG00000059769	DNAJC25
195.4616954	0.743095916	0.547546572	1.357137374	0.174737565	0.946176003	ENSG00000143382	ADAMTSL4
203.1908072	-0.143061553	0.462754933	-0.309151869	0.757206005	0.999467288	ENSG00000113361	CDH6
200.4453354	1.006357029	1.03151792	0.975607897	0.329258809	0.976026345	ENSG00000160200	CBS
203.5495875	0.218350362	0.334673513	0.652427972	0.514125124	0.993513219	ENSG00000135211	TMEM60
204.3895413	0.090623983	0.275582839	0.3288448	0.742272999	0.999467288	ENSG00000133997	MED6
203.8334721	0.056318378	0.317229385	0.177532034	0.859090505	0.999467288	ENSG00000184898	RBM43
206.9781295	0.348283521	0.368563824	0.944974789	0.344671747	0.977691382	ENSG00000269113	TRABD2B
206.8361399	0.234118303	0.376479075	0.621862724	0.534032138	0.99483168	ENSG00000206195	DUXAP8
200.3473526	-0.2782867	0.44724578	-0.622223199	0.533795114	0.99483168	ENSG00000112667	DNPH1
202.5806985	-0.392541684	0.320722557	-1.223929142	0.220978959	0.956884001	ENSG00000126768	TIMM17B
206.1159478	0.326243724	0.335366777	0.97279679	0.330654308	0.976026345	ENSG00000189308	LIN54
202.7289085	0.210057698	0.304958513	0.688807459	0.490944441	0.993513219	ENSG00000243943	ZNF512
203.8209337	-0.194473832	0.30983946	-0.627659989	0.530226701	0.99483168	ENSG00000137054	POLR1E
206.1276695	0.30964206	0.282418657	1.096393783	0.272906483	0.962465655	ENSG00000185621	LMLN
204.1844759	-0.335157834	0.315773711	-1.061386122	0.288514461	0.966288987	ENSG00000157911	PEX10
205.2871939	-0.249261859	0.30026202	-0.83014781	0.406455219	0.990226751	ENSG00000148429	USP6NL
203.0783023	0.01651139	0.272409661	0.060612351	0.951667937	0.999527751	ENSG00000172943	PHF8
203.9691567	-0.076719887	0.313538925	-0.24469015	0.806696355	0.999467288	ENSG00000087301	TXNDC16
203.0097212	0.421761802	0.429707764	0.981508451	0.326342069	0.976026345	ENSG00000185883	ATP6V0C
200.0968377	1.3346557	0.429750272	3.105654111	0.001898587	0.308164584	ENSG00000137962	ARHGAP29
204.4126873	0.278001657	0.297405188	0.934757255	0.349913413	0.977691382	ENSG00000143353	LYPLAL1
202.7705007	-0.076864798	0.366940904	-0.209474598	0.834077763	0.999467288	ENSG00000164182	NDUFAF2
206.2023881	0.079599297	0.300968124	0.264477501	0.791411999	0.999467288	ENSG00000095574	IKZF5
203.6213205	0.431872563	0.35336999	1.222154046	0.221649378	0.956884001	ENSG00000114770	ABCC5
203.4604882	-0.0438463	0.292498367	-0.149902719	0.880841367	0.999467288	ENSG00000139323	POC1B
203.1828018	0.248155596	0.306187881	0.810468379	0.417671031	0.990226751	ENSG00000151690	MFSD6
210.7497394	1.127405191	0.947868216	1.189411325	0.234277847	0.960525552	ENSG00000128567	PODXL
197.3703689	-0.236099177	0.505945096	-0.466649799	0.640750452	0.996553586	ENSG00000260910	LINC00565
203.031061	-0.178632901	0.412179239	-0.433386461	0.664734035	0.997568624	ENSG00000172059	KLF11
202.5607893	-0.288706361	0.308707209	-0.935210944	0.3496796	0.977691382	ENSG00000171953	ATPAF2
204.9662452	-0.840148193	0.285463531	-2.94310166	0.003249417	0.408098513	ENSG00000198890	PRMT6
203.6676336	-0.217465731	0.267453586	-0.813097084	0.416162398	0.990226751	ENSG00000136682	CBWD2
202.5081012	-0.725908357	0.383689569	-1.891915798	0.058502201	0.852621724	ENSG00000163517	HDAC11
202.8163666	0.199559512	0.258478869	0.772053487	0.440082749	0.99135833	ENSG00000101882	NKAP
204.6269313	-0.28060145	0.313367021	-0.895440269	0.370551783	0.981455968	ENSG00000161847	RAVER1
203.6838495	0.156656705	0.284088918	0.551435467	0.581335193	0.99483168	ENSG00000113593	PPWD1
203.8435043	0.016303651	0.269945838	0.060396009	0.951840237	0.999527751	ENSG00000028839	TBPL1
204.5589393	0.352555619	0.265064127	1.330076701	0.183493001	0.949094286	ENSG00000141446	ESCO1
202.8386857	-0.204109759	0.329223453	-0.619973326	0.535275348	0.99483168	ENSG00000243156	MICAL3
200.4687916	0.353891	0.348288284	1.016086433	0.309588229	0.973427483	ENSG00000160326	SLC2A6

205.2898356	0.557766172	0.371875435	1.499873664	0.133647131	0.933741583	ENSG00000128944	KNSTRN
201.1799224	-0.322571852	0.27323625	-1.180560238	0.237777467	0.962465655	ENSG00000106330	MOSPD3
204.1472364	0.093986107	0.266451496	0.352732519	0.724288978	0.999456245	ENSG00000091127	PUS7
204.350183	-0.131541829	0.273821049	-0.480393417	0.630947674	0.99483168	ENSG00000083544	TDRD3
203.7554633	-0.301614547	0.285032936	-1.058174367	0.289975955	0.967933358	ENSG00000100056	ESS2
203.5124096	0.17024571	0.283958432	0.599544477	0.54880986	0.99483168	ENSG00000148842	CNNM2
201.0567732	1.165582193	0.419413707	2.779075106	0.005451392	0.492090333	ENSG00000166483	WEE1
205.7819555	0.240689391	0.338052582	0.711988026	0.476472191	0.99135833	ENSG00000166024	R3HCC1L
201.5248315	0.107352018	0.26726596	0.401667381	0.687928834	0.998869317	ENSG00000215012	RTL10
199.9200588	-0.399616741	0.340181001	-1.174717989	0.24010756	0.962465655	ENSG00000204392	LSM2
203.1564877	-0.255883721	0.274575529	-0.931924713	0.351375435	0.977691382	ENSG00000119509	INVS
202.2894382	-0.065427933	0.284427156	-0.230034057	0.818065306	0.999467288	ENSG00000154370	TRIM11
201.9405733	-0.019033698	0.27689559	-0.068739621	0.94519688	0.999527751	ENSG00000014164	ZC3H3
198.2633092	-0.381679908	0.372515331	-1.024601877	0.305551078	0.973427483	ENSG00000173992	CCS
202.3860155	0.03058614	0.345126146	0.088623074	0.929381469	0.999527751	ENSG00000123600	METTL8
203.5756318	-0.012256077	0.359737477	-0.034069503	0.972821727	0.999527751	ENSG00000196810	CTBP1-DT
207.3371843	-1.45356474	0.429446394	-3.384740817	0.000712455	0.201177461	ENSG00000169583	CLIC3
202.4510941	0.074330264	0.28571749	0.260153006	0.794745753	0.999467288	ENSG00000054282	SDCCAG8
199.1628814	0.680651877	0.50340961	1.3520836	0.176348573	0.946278819	ENSG00000109861	CTSC
200.6969156	0.090757319	0.389563364	0.232971906	0.815783221	0.999467288	ENSG00000117593	DARS2
200.6093306	0.308928376	0.282814749	1.092334743	0.274685995	0.962465655	ENSG00000131373	HACL1
198.1551119	1.582417476	0.462450464	3.421809686	0.000622058	0.195790358	ENSG00000102554	KLF5
203.4612147	-0.251717883	0.308577717	-0.815735775	0.414651273	0.990226751	ENSG00000114904	NEK4
201.5291628	-0.316562435	0.254029265	-1.246165221	0.212703745	0.954310328	ENSG00000170473	PYM1
201.1509865	-0.203714645	0.373957805	-0.544753024	0.585923391	0.99483168	ENSG00000189129	PLAC9
201.9774898	0.669717816	0.274469263	2.440046687	0.014685364	0.686410066	ENSG00000166881	NEMP1
197.8585979	-0.397833358	0.311840585	-1.275758761	0.20204081	0.954310328	ENSG00000172366	MCRIP2
201.7790868	-0.445153631	0.293109461	-1.518728293	0.128830902	0.928627271	ENSG00000234287	AC099560.2
202.2340416	0.431985957	0.374555849	1.153328557	0.248775553	0.962465655	ENSG00000137337	MDC1
202.7821079	-0.442977358	0.329644659	-1.343802624	0.179012221	0.9469453	ENSG00000135392	DNAJC14
200.5941683	-0.151753673	0.261524213	-0.580266244	0.561735088	0.99483168	ENSG00000143971	ETAA1
203.4727693	0.486297276	0.421221357	1.154493397	0.248297944	0.962465655	ENSG00000197016	ZNF470
199.6637044	-0.292629399	0.310166168	-0.943460082	0.345445624	0.977691382	ENSG00000128228	SDF2L1
201.4797397	0.081944809	0.272090807	0.301167136	0.763287049	0.999467288	ENSG00000085274	MYNN
201.1963759	-0.256982238	0.328938086	-0.78124805	0.434656618	0.991301167	ENSG00000139998	RAB15
202.3754055	0.034167314	0.319344402	0.106992055	0.914795282	0.999527751	ENSG00000103540	CCP110
203.7777561	-0.021153289	0.427511289	-0.049480072	0.960536718	0.999527751	ENSG00000205268	PDE7A
206.3257805	-0.215720819	0.476937915	-0.452303775	0.651050157	0.997449432	ENSG00000135750	KCNK1
200.4504573	0.349458428	0.256858038	1.360511943	0.173667976	0.944422693	ENSG00000145216	FIP1L1
200.2619735	-0.043016937	0.30360728	-0.141686119	0.88732794	0.999467288	ENSG00000102390	PBDC1
197.3742722	0.827688817	0.343487847	2.409659683	0.015967407	0.696728876	ENSG00000172935	MRGPRF
202.9411258	-0.04657541	0.312067352	-0.149247941	0.881357992	0.999467288	ENSG00000135093	USP30
201.6255257	-0.266395312	0.271873358	-0.979850747	0.327159798	0.976026345	ENSG00000025156	HSF2
198.6246336	-0.026539225	0.299869516	-0.088502576	0.929477237	0.999527751	ENSG00000169964	TMEM42
200.7119969	0.100290591	0.26945532	0.37219748	0.709745822	0.99900742	ENSG00000109065	NAT9
197.424656	0.081757486	0.308579758	0.264947663	0.79104978	0.999467288	ENSG00000166770	ZNF667-AS1
199.699034	-0.413415071	0.373345674	-1.107325194	0.268153355	0.962465655	ENSG00000135736	CCDC102A
199.4671882	-0.024068269	0.303292352	-0.079356665	0.936748936	0.999527751	ENSG00000073169	SELENOO
204.0504142	0.11595531	0.393162238	0.294929928	0.768047409	0.999467288	ENSG00000116127	ALMS1
199.2048286	-0.273354599	0.357961986	-0.763641421	0.445080958	0.99135833	ENSG00000105926	MPP6
202.0630101	0.412812462	0.329013264	1.254698542	0.209588215	0.954310328	ENSG00000228253	MT-ATP8
199.0305984	-0.030964769	0.305214962	-0.101452331	0.919191396	0.999527751	ENSG00000178685	PARP10
200.0258841	0.088764846	0.258489104	0.34339879	0.73129847	0.999467288	ENSG00000167967	E4F1
199.2387122	-0.00608313	0.282252275	-0.021552104	0.98280524	0.999527751	ENSG00000104231	ZFAND1
201.9087263	0.021794478	0.28414425	0.076702161	0.938860485	0.999527751	ENSG00000122512	PMS2
195.3023776	-0.044354761	0.626042726	-0.070849416	0.943517602	0.999527751	ENSG00000133169	BEX1
202.2336177	0.269732019	0.303745719	0.888019164	0.374530443	0.982609538	ENSG00000040199	PHLPP2
198.0228232	-0.285253703	0.368288833	-0.774538019	0.438612694	0.991301167	ENSG00000160570	DEDD2
204.3127367	-0.585044418	0.554627774	-1.054841546	0.2914978	0.970069752	ENSG00000117862	TXNDC12
199.9385279	0.24608733	0.285321424	0.862491596	0.388417056	0.986799595	ENSG00000110274	CEP164
199.6326253	0.222135248	0.254432942	0.873060091	0.382630325	0.98555814	ENSG00000129460	NGDN
201.9502305	0.383320608	0.323274857	1.185742098	0.235724164	0.962465655	ENSG00000203668	CHML
201.0346562	0.011896581	0.271088551	0.043884483	0.964996485	0.999527751	ENSG00000147316	MCPH1
196.1731756	0.158582416	0.401258864	0.395212245	0.692686257	0.998869317	ENSG00000110076	NRXN2

200.440075	-0.610725555	0.31982993	-1.909532219	0.05619347	0.847598083	ENSG00000132635	PCED1A
195.6706653	1.14656333	1.053847092	1.087978834	0.276604452	0.962465655	ENSG00000169908	TM4SF1
199.4282682	-0.0307895	0.266269722	-0.115632748	0.907943608	0.999527751	ENSG00000154781	CCDC174
200.1539616	0.262343709	0.280579274	0.935007443	0.349784464	0.977691382	ENSG00000118412	CASP8AP2
198.2267358	0.05965301	0.962569755	0.061972662	0.950584603	0.999527751	ENSG00000213694	S1PR3
198.1617372	0.210011169	0.402489067	0.521781052	0.601822784	0.99483168	ENSG00000160818	GPATCH4
196.798378	-0.203172306	0.355331752	-0.571782017	0.567469662	0.99483168	ENSG00000165912	PACSIN3
201.0444536	-0.160422397	0.327717701	-0.489513983	0.624477858	0.99483168	ENSG00000119661	DNAL1
198.9882926	-0.067057437	0.287298547	-0.23340681	0.815445526	0.999467288	ENSG00000010072	SPRTN
198.2233335	-0.249697153	0.243659618	-1.024778563	0.305467684	0.973427483	ENSG00000141098	GFOD2
199.9968952	0.585272355	0.291650504	2.006759281	0.0447753	0.812424202	ENSG00000138002	IFT172
199.7412872	0.008054272	0.305875703	0.026331845	0.978992655	0.999527751	ENSG00000137574	TGS1
197.5466562	0.06562976	0.26454496	0.248085469	0.804068278	0.999467288	ENSG00000256053	APOPT1
197.9875302	-0.304739527	0.295522951	-1.031187345	0.302452974	0.972793733	ENSG00000186222	BLOC1S4
198.1488257	-0.337810221	0.284012074	-1.189422039	0.234273632	0.960525552	ENSG00000219545	UMAD1
197.141008	-0.268176407	0.26912909	-0.996460124	0.319026632	0.974768742	ENSG00000128311	TST
199.6310491	-0.168810978	0.258633595	-0.652703208	0.513947634	0.993513219	ENSG00000167747	C19orf48
199.2139989	0.102057074	0.30804442	0.331306357	0.74041309	0.999467288	ENSG00000119772	DNMT3A
204.6113459	0.308577348	0.487409501	0.633096703	0.526670495	0.99483168	ENSG00000157680	DGKI
197.7254983	-0.080942304	0.25559149	-0.316686224	0.751481703	0.999467288	ENSG00000165526	RPUSD4
199.8581057	0.042012386	0.364428312	0.115282991	0.90822082	0.999527751	ENSG00000149582	TMEM25
197.918295	-0.086798151	0.243430727	-0.356562018	0.721419716	0.99900742	ENSG00000176463	SLCO3A1
197.7802434	0.195008261	0.308279419	0.632569835	0.527014591	0.99483168	ENSG00000143303	RRNAD1
198.9705652	0.189224798	0.330781313	0.572054076	0.56728534	0.99483168	ENSG00000122779	TRIM24
197.3510276	-0.112897351	0.251352453	-0.449159535	0.653316575	0.997527562	ENSG00000108963	DPH1
195.1417221	-0.477180053	0.373452178	-1.277754104	0.201336142	0.954310328	ENSG00000104518	GSDMD
199.4393456	0.07151715	0.484746668	0.147535105	0.882709673	0.999467288	ENSG00000164976	MYORG
198.8351762	-0.183277618	0.294932284	-0.621422706	0.534321536	0.99483168	ENSG00000155100	OTUD6B
194.5826785	-0.12799501	0.307214036	-0.416631387	0.676948048	0.998869317	ENSG00000099203	TMED1
196.6964139	-0.159920389	0.29655358	-0.539263054	0.589705358	0.99483168	ENSG00000136856	SLC2A8
197.9589768	-0.181650834	0.325190405	-0.558598381	0.576435845	0.99483168	ENSG00000087157	PGS1
194.8873128	-0.107623184	0.364121477	-0.295569447	0.767558909	0.999467288	ENSG00000244694	PTCHD4
195.4681773	-0.175029037	0.363942421	-0.480925078	0.630569749	0.99483168	ENSG00000102103	PQBP1
197.8769028	0.124304114	0.297546137	0.417764167	0.676119552	0.998869317	ENSG00000204084	INPP5B
194.1519066	0.165751981	0.335256085	0.494404094	0.621020823	0.99483168	ENSG00000099949	LZTR1
195.7839365	0.14964215	0.346461014	0.431916274	0.665802266	0.997568624	ENSG00000155893	PXYLP1
194.7265946	0.022732703	0.320655654	0.07089444	0.943481768	0.999527751	ENSG00000104497	SNX16
198.1920716	0.07320933	0.281059437	0.260476328	0.794496373	0.999467288	ENSG00000077514	POLD3
197.2751897	0.507383237	0.440975358	1.150593174	0.249899642	0.962465655	ENSG00000111602	TIMELESS
194.366906	-0.090170086	0.36959465	-0.243970215	0.807253888	0.999467288	ENSG00000090339	ICAM1
196.7571521	0.358945925	0.262550043	1.367152415	0.171577537	0.944422693	ENSG00000130023	ERMARD
195.8363743	-0.040905222	0.270484151	-0.151229646	0.879794565	0.999467288	ENSG00000175895	PLEKHF2
197.0116034	-0.18787974	0.296245119	-0.634203664	0.525947917	0.99483168	ENSG00000010219	DYRK4
196.617976	0.563534015	0.300471313	1.875500221	0.060723939	0.858996918	ENSG00000144802	NFKBIZ
197.0134081	-0.234987583	0.311300892	-0.754856759	0.450334933	0.99135833	ENSG00000117543	DPH5
198.0924115	-0.340163777	0.30165674	-1.127651837	0.259466986	0.962465655	ENSG00000248092	NNT-AS1
194.2241968	-0.111586313	0.38921613	-0.286694986	0.774345877	0.999467288	ENSG00000114107	CEP70
196.6770869	0.536056517	0.291057299	1.841755969	0.065510853	0.868845964	ENSG00000121775	TMEM39B
195.2210374	0.045305166	0.343913214	0.131734297	0.895194457	0.999467288	ENSG00000134138	MEIS2
195.7593981	0.175088545	0.329464081	0.531434397	0.595117791	0.99483168	ENSG00000176390	CRLF3
197.4768546	-0.082119044	0.398416772	-0.206113421	0.836702315	0.999467288	ENSG00000197557	TTC30A
199.6524756	0.097869721	0.43522901	0.224869481	0.822080812	0.999467288	ENSG00000142856	ITGB3BP
195.8166374	0.318918001	0.414503047	0.76939845	0.441656809	0.99135833	ENSG00000051825	MPHOSPH9
195.4169779	0.059323432	0.317472239	0.186861795	0.851768995	0.999467288	ENSG00000143379	SETDB1
196.2703523	0.358821932	0.308977566	1.161320339	0.245511651	0.962465655	ENSG00000138709	LARP1B
193.1325269	0.036674755	0.665307326	0.055124533	0.956039252	0.999527751	ENSG00000219607	PPP1R3G
195.4097892	-0.351043426	0.253665932	-1.383880854	0.16639494	0.943223078	ENSG00000198894	CIPC
192.997191	0.151244672	0.372535346	0.405987441	0.684751828	0.998869317	ENSG00000125746	EML2
194.6014624	-0.029496492	0.267156462	-0.110409053	0.912084974	0.999527751	ENSG00000196290	NIF3L1
186.0862031	0.782995473	0.662247041	1.182331403	0.23707423	0.962465655	ENSG00000145632	PLK2
198.6207978	0.035096683	0.396302764	0.088560277	0.929431378	0.999527751	ENSG00000196204	RNF216P1
201.3835314	-0.679806379	0.442650614	-1.535762874	0.124596548	0.927794614	ENSG00000227036	LINC00511
195.5876522	0.332786108	0.273442269	1.217025114	0.223594661	0.956884001	ENSG00000122643	NT5C3A
195.5003996	0.29774521	0.313583122	0.949493735	0.342369557	0.977691382	ENSG00000140400	MAN2C1

198.0838219	0.028111401	0.400274827	0.070230249	0.944010398	0.999527751	ENSG00000167785	ZNF558
194.6571961	0.110695864	0.365736293	0.302665791	0.762144571	0.999467288	ENSG00000172594	SMPDL3A
195.8832913	0.318663384	0.302932376	1.051929108	0.292832076	0.970706863	ENSG00000132952	USPL1
197.5772915	-0.105634343	0.410557357	-0.257294971	0.796951074	0.999467288	ENSG00000099994	SUSD2
195.4530116	-0.363049499	0.309395657	-1.173414983	0.240629432	0.962465655	ENSG00000168679	SLC16A4
196.8305052	0.001248409	0.327653494	0.003810149	0.996959948	0.999527751	ENSG00000136870	ZNF189
193.2660486	-0.368480781	0.294211081	-1.252436788	0.210410748	0.954310328	ENSG00000099364	FBXL19
192.2933036	-0.52769522	0.364222858	-1.448825102	0.147386432	0.936396974	ENSG00000147889	CDKN2A
193.5225992	0.262345022	0.276155684	0.94998958	0.342117547	0.977691382	ENSG00000165632	TAF3
193.1581293	0.453692777	0.282075748	1.608407601	0.107745938	0.923922314	ENSG00000143669	LYST
194.8517211	0.360310814	0.268433702	1.342271148	0.179508095	0.947254444	ENSG00000115942	ORC2
192.804071	0.392315457	0.325912757	1.203743788	0.228688622	0.959527137	ENSG00000074266	EED
192.8050918	0.522160022	0.340576628	1.533164575	0.12523531	0.928052123	ENSG00000204267	TAP2
193.4858109	-0.026638469	0.34563273	-0.077071603	0.938566583	0.999527751	ENSG00000159082	SYNJ1
191.4008349	-0.328641701	0.330007226	-0.995862137	0.319317134	0.974964731	ENSG00000131368	MRPS25
193.8840717	-0.15223488	0.273603988	-0.556405927	0.577933388	0.99483168	ENSG00000227097	RPS28P7
194.9382955	0.124069506	0.275451635	0.450422108	0.652406107	0.997449432	ENSG00000105053	VRK3
193.6173833	0.216726553	0.305552158	0.709294788	0.478141562	0.99135833	ENSG00000020922	MRE11
191.9624303	-0.051380939	0.310489271	-0.165483782	0.868563214	0.999467288	ENSG00000123297	TSFM
193.4963051	0.372820828	0.36166638	1.030841816	0.302615005	0.972793733	ENSG00000139679	LPAR6
193.9184913	0.251106198	0.445966004	0.563061301	0.573393142	0.99483168	ENSG00000100578	KIAA0586
194.1191963	0.106309743	0.392325003	0.270973664	0.786411288	0.999467288	ENSG00000259781	HMGB1P6
194.3630976	0.252490844	0.315914009	0.799239149	0.424151755	0.991301167	ENSG00000188177	ZC3H6
191.5116454	-0.302902953	0.287772104	-1.052579275	0.29253386	0.970706863	ENSG00000165675	ENOX2
194.1237755	0.257271185	0.250444558	1.027258038	0.304298976	0.973427483	ENSG00000049883	PTCD2
194.9153534	0.402828659	0.336604422	1.196742029	0.23140712	0.959575179	ENSG00000151233	GXYLT1
193.0152398	0.696317987	0.284562965	2.446973335	0.01440615	0.684434275	ENSG00000139636	LMBR1L
194.5564392	-0.000466641	0.283560368	-0.001645651	0.998686961	0.999527751	ENSG00000273559	CWC25
201.3716744	-0.955229767	0.492904682	-1.937960425	0.052628051	0.847598083	ENSG00000155966	AFF2
193.1112889	-0.389835914	0.320681701	-1.215647515	0.224119225	0.956884001	ENSG00000137171	KLC4
194.4271105	-0.460974011	0.262318078	-1.757309346	0.078865096	0.897052166	ENSG00000149476	TKFC
191.528769	0.222271622	0.367082433	0.605508741	0.544841018	0.99483168	ENSG00000138193	PLCE1
192.1498413	-0.407449528	0.326662336	-1.247311009	0.212283482	0.954310328	ENSG00000169851	PCDH7
194.793355	0.170427989	0.303862991	0.560871162	0.574885371	0.99483168	ENSG00000174373	RALGAPA1
193.3092602	0.117467867	0.286145683	0.410517696	0.681426225	0.998869317	ENSG00000175309	PHYKPL
189.8331785	0.268814656	0.345238786	0.778633998	0.436195346	0.991301167	ENSG00000106268	NUDT1
188.4831588	0.679160366	0.560970655	1.210687866	0.226015055	0.958865115	ENSG00000046653	GPM6B
198.3683149	-0.754239445	0.469928374	-1.605009372	0.108491742	0.923922314	ENSG00000167034	NKX3-1
193.4983155	0.852572667	0.412820695	2.065237223	0.03890056	0.793985738	ENSG00000173207	CKS1B
192.095038	0.215600816	0.527188432	0.40896348	0.682566462	0.998869317	ENSG00000213186	TRIM59
191.5900401	0.587275867	0.375603522	1.56355261	0.117922672	0.923922314	ENSG00000276107	AC037198.1
190.8371046	0.076619495	0.298225899	0.256917644	0.797242349	0.999467288	ENSG00000101417	PXMP4
194.3450595	0.20925805	0.323597501	0.646661515	0.517851036	0.993513219	ENSG00000110628	SLC22A18
192.647898	-0.006436168	0.289579873	-0.022225883	0.982267771	0.999527751	ENSG00000088356	PDRG1
193.4926046	0.10419807	0.349643804	0.298012059	0.76569396	0.999467288	ENSG00000198919	DZIP3
192.8195928	0.434870578	0.305045615	1.425591967	0.153986156	0.939432741	ENSG00000187742	SECISBP2
188.6958675	-0.135860742	0.392372672	-0.346254343	0.729151578	0.999467288	ENSG00000116191	RALGPS2
190.8216917	-0.065366856	0.419862237	-0.155686437	0.876280189	0.999467288	ENSG00000135828	RNASEL
191.7544971	0.852389224	0.418524215	2.036654495	0.041684678	0.801666844	ENSG00000110427	KIAA1549L
189.319916	0.699605165	0.47476314	1.473587787	0.140592612	0.935644597	ENSG00000149554	CHEK1
200.1974314	1.663958112	0.553809707	3.004566535	0.002659596	0.380278034	ENSG00000149948	HMGA2
189.8370107	-0.404122351	0.296397082	-1.363449158	0.172740998	0.944422693	ENSG00000164885	CDK5
193.8915614	0.046073611	0.287113419	0.160471815	0.872509422	0.999467288	ENSG00000089195	TRMT6
193.0908997	0.202177541	0.313373519	0.645164728	0.518820441	0.993513219	ENSG00000266338	NBPF15
190.631048	0.10750987	0.278490395	0.386045163	0.699463221	0.998869317	ENSG00000109670	FBXW7
195.0004605	-0.640770141	0.373135758	-1.7172574	0.085932152	0.901307093	ENSG00000111752	PHC1
192.5728305	-0.075046141	0.350799591	-0.213928815	0.83060257	0.999467288	ENSG00000176842	IRX5
191.980358	0.429030607	0.246512323	1.74040227	0.081788407	0.897807954	ENSG00000185404	SP140L
194.6178334	-0.03966638	0.34031897	-0.116556477	0.907211529	0.999527751	ENSG00000177125	ZBTB34
192.3871056	0.446079802	0.305045974	1.462336304	0.143649075	0.935644597	ENSG00000198026	ZNF335
193.4323657	0.087464969	0.346838196	0.252178019	0.800903468	0.999467288	ENSG00000074855	ANO8
191.6008773	0.12331439	0.262978357	0.468914597	0.639130682	0.996553586	ENSG00000131931	THAP1
190.0434169	-0.36073448	0.33969739	-1.061928914	0.288267958	0.966288987	ENSG00000108439	PNPO
190.8471924	-0.137322973	0.373611893	-0.367555144	0.713204967	0.99900742	ENSG00000167074	TEF

192.3983733	0.210662422	0.311767828	0.67570289	0.499229301	0.993513219	ENSG00000182150	ERCC6L2
191.4984881	-0.250459325	0.319549105	-0.783789788	0.433163467	0.991301167	ENSG00000083750	RRAGB
191.3172382	-0.399898283	0.332299549	-1.2034271	0.228811086	0.959527137	ENSG00000006695	COX10
190.9865479	0.207713591	0.292762976	0.709494055	0.478017939	0.99135833	ENSG00000144426	NBEAL1
193.3433654	0.460623426	0.380977082	1.209058097	0.226640526	0.958865115	ENSG00000198455	ZXDB
192.0445101	0.255560631	0.286940829	0.890638783	0.373122986	0.982329093	ENSG00000165512	ZNF22
191.9203058	0.422932676	0.325373163	1.299838847	0.193656208	0.950382812	ENSG00000102901	CENPT
192.3262725	-0.917136069	0.532288612	-1.723005243	0.084887577	0.901307093	ENSG00000171476	HOPX
189.6300972	-0.097454103	0.272846026	-0.357176185	0.720959913	0.99900742	ENSG00000046647	GEMIN8
191.4138602	-0.278261865	0.416065607	-0.668793239	0.50362738	0.993513219	ENSG00000157551	KCNJ15
189.8738866	-0.419123625	0.280534778	-1.494016635	0.135171271	0.933741583	ENSG00000103707	MTFMT
189.3767335	-0.441279892	0.269383016	-1.638113267	0.101398068	0.92169898	ENSG00000141295	SCRN2
191.7196315	-0.283982079	0.27025738	-1.050783807	0.293357895	0.970706863	ENSG00000095794	CREM
188.530497	-0.168702486	0.305386496	-0.552422873	0.580658663	0.99483168	ENSG00000156990	RPUSD3
190.7373948	-0.377917905	0.293573832	-1.287301058	0.197989375	0.954310328	ENSG00000240771	ARHGEF25
186.5544212	1.040356128	0.513989664	2.02407986	0.042961935	0.806698895	ENSG00000240694	PNMA2
191.0249811	0.071502769	0.26126585	0.273678205	0.784331929	0.999467288	ENSG00000169519	METTL15
190.3904126	-0.059330171	0.305214577	-0.194388391	0.845871779	0.999467288	ENSG00000132740	IGHMBP2
193.7893277	0.126210967	0.33252978	0.379547862	0.70428107	0.99900742	ENSG00000106346	USP42
185.6258473	0.986011952	0.502595801	1.961838819	0.049781253	0.836811782	ENSG00000106034	CPED1
190.4422039	0.169657199	0.294738052	0.575620274	0.564871874	0.99483168	ENSG00000181666	HKR1
191.0690442	0.570655938	0.290627329	1.96353158	0.049584439	0.836346249	ENSG00000177054	ZDHHC13
190.2617521	-0.257916603	0.273633417	-0.942562521	0.345904718	0.977691382	ENSG00000091732	ZC3HC1
188.1400368	0.006053812	0.283665228	0.021341398	0.982973321	0.999527751	ENSG00000119929	CUTC
188.4596439	-0.012989625	0.284202989	-0.045705449	0.963545021	0.999527751	ENSG00000156873	PHKG2
190.7430573	0.023054044	0.273093523	0.084418127	0.932723996	0.999527751	ENSG00000167842	MIS12
185.7631136	-0.321692236	0.402418202	-0.799397828	0.42405977	0.991301167	ENSG00000105607	GCDH
176.0926315	0.547134721	0.673765968	0.812054552	0.41676033	0.990226751	ENSG00000142583	SLC2A5
190.6652964	-0.121730566	0.330611772	-0.368197919	0.712725662	0.99900742	ENSG00000184271	POU6F1
192.3850155	-0.230593364	0.355584989	-0.648490152	0.516667978	0.993513219	ENSG00000274211	SOCS7
187.9755224	-0.235560962	0.40498294	-0.581656505	0.560798074	0.99483168	ENSG00000160255	ITGB2
190.5457988	0.448933703	0.523326754	0.857845886	0.390977568	0.988082068	ENSG00000005448	WDR54
191.5535543	0.281248366	0.384636442	0.731205718	0.464653508	0.99135833	ENSG00000196371	FUT4
188.7021686	-0.148710804	0.277928184	-0.535069173	0.592602026	0.99483168	ENSG00000101997	CCDC22
188.8783408	0.242464046	0.316528876	0.766009246	0.443670801	0.99135833	ENSG00000170779	CDCA4
188.3310206	0.020733766	0.307795154	0.067362224	0.946293341	0.999527751	ENSG00000140474	ULK3
188.4070407	0.168497435	0.300511905	0.560701364	0.575001137	0.99483168	ENSG00000164066	INTU
191.4816916	0.213165455	0.395629242	0.538801059	0.590024133	0.99483168	ENSG00000249669	CARMN
189.020635	0.125975172	0.263700063	0.477721432	0.632848488	0.994909189	ENSG00000163626	COX18
188.9631316	-0.301377221	0.324817567	-0.92783535	0.353492984	0.977691382	ENSG00000087095	NLK
189.5516808	-0.68776067	0.377101629	-1.823807213	0.068181223	0.87420149	ENSG00000227063	RPL41P1
191.3622131	0.012349237	0.322935091	0.038240616	0.969495837	0.999527751	ENSG00000204604	ZNF468
190.2477188	-0.052279674	0.275844956	-0.189525574	0.849680913	0.999467288	ENSG00000121058	COIL
189.0265126	0.385337681	0.397691492	0.968936195	0.332577025	0.976026345	ENSG00000185499	MUC1
189.9795126	0.342817195	0.303609286	1.129139361	0.258839049	0.962465655	ENSG00000074935	TUBE1
187.6013681	-0.287989843	0.304558143	-0.945598895	0.344353208	0.977691382	ENSG00000100360	IFT27
189.9757332	0.017832185	0.399919529	0.044589433	0.964434565	0.999527751	ENSG00000214026	MRPL23
190.0145083	-0.453151155	0.35612127	-1.272463045	0.203208646	0.954310328	ENSG00000101084	RAB5IF
193.0172046	-2.798878986	1.106877918	-2.528624828	0.011451036	0.64668571	ENSG00000187634	SAMD11
190.4326026	-0.1043238	0.30355985	-0.34366798	0.731095995	0.999467288	ENSG00000198081	ZBTB14
192.0416446	-0.228674104	0.331116535	-0.690615178	0.489807407	0.993513219	ENSG00000169026	SLC49A3
189.9127327	0.086745436	0.297668132	0.291416603	0.770732718	0.999467288	ENSG00000261801	LOXL1-AS1
188.6708709	-0.436291543	0.291336164	-1.497553673	0.134249251	0.933741583	ENSG00000121680	PEX16
188.7277685	0.635465372	0.311689549	2.038776639	0.041472325	0.800562177	ENSG00000007392	LUC7L
188.842017	0.114827779	0.325880874	0.35236121	0.724567389	0.999456245	ENSG00000228223	HCG11
189.1582144	-0.313132126	0.662698881	-0.47251042	0.636562506	0.99653524	ENSG00000099849	RASSF7
186.7649482	0.29261335	0.366543961	0.798303561	0.424694352	0.991301167	ENSG00000181045	SLC26A11
184.8324984	-0.492198653	0.32341931	-1.521859202	0.128044364	0.928627271	ENSG00000185909	KLHDC8B
187.4378753	-0.15746003	0.303660434	-0.51853983	0.604081678	0.99483168	ENSG00000155363	MOV10
187.7949131	-0.296325488	0.431856265	-0.686166929	0.492607849	0.993513219	ENSG00000116741	RGS2
188.2799447	0.341657367	0.334914479	1.020133166	0.307665309	0.973427483	ENSG00000018699	TTC27
185.2178178	-0.524985372	0.557484883	-0.941703331	0.346344549	0.977691382	ENSG00000004776	HSPB6
187.5028363	-0.245356031	0.427318361	-0.574176197	0.565848575	0.99483168	ENSG00000231389	HLA-DPA1
188.4240453	-0.179597421	0.299008344	-0.60064351	0.548077452	0.99483168	ENSG00000083123	BCKDHB

187.3329573	-0.666115606	0.319448047	-2.085207948	0.037050429	0.790190505	ENSG00000103254	FAM173A
188.7374055	1.227645977	0.629888447	1.948989513	0.051296677	0.842569961	ENSG00000164109	MAD2L1
187.7661802	0.30769555	0.280591225	1.096597191	0.272817516	0.962465655	ENSG00000186260	MRTFB
189.2719051	0.021939677	0.305279914	0.071867409	0.942707427	0.999527751	ENSG00000110583	NAA40
187.3488579	0.302952516	0.281004303	1.078106324	0.28098631	0.963267117	ENSG00000034693	PEX3
187.1419116	-0.038582666	0.280865922	-0.137370407	0.890738021	0.999467288	ENSG00000170836	PPM1D
185.9121553	-0.27730245	0.321615364	-0.862217671	0.388567747	0.986799595	ENSG00000181982	CCDC149
187.2242406	0.339159174	0.258723532	1.310894187	0.189893515	0.949094286	ENSG00000151657	KIN
187.5977427	0.358875416	0.290275385	1.236327412	0.216336886	0.954900762	ENSG00000158161	EYA3
188.5927156	0.141921429	0.278316946	0.509927373	0.610102344	0.99483168	ENSG00000254685	FPGT
188.4379888	0.194091043	0.340871873	0.56939589	0.569087506	0.99483168	ENSG00000129667	RHBDF2
186.4978293	0.728153781	0.383893544	1.896759644	0.057859668	0.852306368	ENSG00000138074	SLC5A6
186.9452786	-0.039880365	0.281063684	-0.14189085	0.887166222	0.999467288	ENSG00000185753	CXorf38
187.725326	0.149731081	0.265851896	0.563212385	0.57329027	0.99483168	ENSG00000132323	ILKAP
192.906045	0.473130041	0.404592273	1.169399596	0.242242672	0.962465655	ENSG00000047936	ROS1
186.4296998	0.339046576	0.267815555	1.265970441	0.205523665	0.954310328	ENSG00000111364	DDX55
187.8923046	0.196386585	0.251425971	0.781091085	0.434748925	0.991301167	ENSG00000104859	CLASRP
185.254045	-0.305413977	0.334230635	-0.913782115	0.360831346	0.979465649	ENSG00000100211	CBY1
186.9535439	-0.239814528	0.298634506	-0.803036901	0.421953406	0.991301167	ENSG00000188010	MORN2
186.5472737	0.074954403	0.314383947	0.238416763	0.811557867	0.999467288	ENSG00000109534	GAR1
186.5495513	0.063398123	0.322406298	0.19664046	0.844108906	0.999467288	ENSG00000107020	PLGRKT
183.116577	-0.027876803	0.605224129	-0.046060297	0.963262191	0.999527751	ENSG00000158023	WDR66
185.5693915	0.70843795	0.339370704	2.087504732	0.036842534	0.790190505	ENSG00000168566	SNRNP48
187.2012845	-0.497648235	0.291605479	-1.706580534	0.087900046	0.908761892	ENSG00000154328	NEIL2
186.9285027	-0.520718162	0.297551223	-1.750011839	0.080116271	0.897807954	ENSG00000129932	DOHH
187.1665425	0.390271684	0.324512395	1.202640298	0.229115546	0.959575179	ENSG00000155957	TMBIM4
185.5759024	-0.26211931	0.314076781	-0.834570799	0.403959409	0.988927059	ENSG00000069509	FUNDC1
185.2307152	0.610665353	0.289611156	2.108569854	0.034981727	0.784512596	ENSG00000187678	SPRY4
185.7348827	0.117486325	0.408952202	0.287286202	0.773893187	0.999467288	ENSG00000182179	UBA7
186.6368933	0.020750448	0.287349702	0.072213221	0.942432224	0.999527751	ENSG00000168792	ABHD15
186.6592612	-0.196994673	0.288429602	-0.682990483	0.494612856	0.993513219	ENSG00000059691	GATB
186.5978442	-0.468432881	0.284163533	-1.648462336	0.099257834	0.921147998	ENSG00000107551	RASSF4
184.124821	0.060583848	0.297216104	0.203837702	0.83848033	0.999467288	ENSG00000174886	NDUFA11
184.9043733	-0.186795326	0.329866626	-0.566275309	0.571206643	0.99483168	ENSG00000125703	ATG4C
185.844024	-0.146886744	0.273017693	-0.538011813	0.590568893	0.99483168	ENSG00000232388	SMIM26
187.1994159	0.092886079	0.302953329	0.306601942	0.759146375	0.999467288	ENSG00000168763	CNNM3
185.4850548	-0.19182596	0.282045064	-0.680125217	0.496425179	0.993513219	ENSG00000100151	PICK1
184.2802568	-0.030247592	0.419054134	-0.072180633	0.942458158	0.999527751	ENSG00000196639	HRH1
184.2121144	-0.124496871	0.300562542	-0.414212863	0.678718222	0.998869317	ENSG00000176396	EID2
186.6562442	0.13678067	0.33824038	0.404388943	0.68592673	0.998869317	ENSG00000132017	DCAF15
185.3595388	-0.171265273	0.342836284	-0.499554107	0.617389079	0.99483168	ENSG00000132768	DPH2
184.9731243	0.268632476	0.269349837	0.997336693	0.318601109	0.974768742	ENSG00000123636	BAZ2B
186.8060399	-0.669247218	0.429726032	-1.557381139	0.119380022	0.923922314	ENSG00000006377	DLX6
188.9671962	0.001934857	0.533306062	0.003628042	0.997105248	0.999527751	ENSG00000175505	CLCF1
183.5194302	-0.636959382	0.301643341	-2.111630841	0.034718125	0.784512596	ENSG00000204220	PFDN6
184.5577344	-0.007609667	0.317277341	-0.023984276	0.980865151	0.999527751	ENSG00000133195	SLC39A11
184.2520332	0.374003122	0.329189277	1.136133977	0.255900497	0.962465655	ENSG00000125871	MGME1
187.4781977	0.026564187	0.317754342	0.08359976	0.933374658	0.999527751	ENSG00000054392	HHAT
183.6224549	-0.236122019	0.354229467	-0.666579269	0.505040914	0.993513219	ENSG00000237765	FAM200B
184.3215907	0.285669037	0.361481955	0.790271916	0.429368984	0.991301167	ENSG00000270629	NBPF14
185.2195349	-0.117530485	0.318808469	-0.368655466	0.712384547	0.99900742	ENSG00000143995	MEIS1
185.2811734	-0.299733664	0.268633038	-1.115773643	0.264519038	0.962465655	ENSG00000126522	ASL
184.0541117	-0.377967382	0.285377263	-1.32444813	0.185354259	0.949094286	ENSG00000115042	FAHD2A
187.4310769	0.417889322	0.365650479	1.142865513	0.253094433	0.962465655	ENSG00000164073	MFSD8
184.3611759	0.387109265	0.277192181	1.39653746	0.162552708	0.943223078	ENSG00000110104	CCDC86
185.9948391	-0.218188264	0.267244419	-0.816437121	0.414250173	0.990226751	ENSG00000134897	BIVM
183.6330628	-0.063112022	0.321557258	-0.196269936	0.844398892	0.999467288	ENSG00000159023	EPB41
182.5069664	0.09210018	0.399352683	0.230623668	0.817607179	0.999467288	ENSG00000146143	PRIM2
184.661408	-0.407979783	0.329654098	-1.237599607	0.21586456	0.954310328	ENSG00000157823	AP3S2
184.7540041	0.476370149	0.437236486	1.089502283	0.275932449	0.962465655	ENSG00000172062	SMN1
184.4643119	0.004402134	0.300003824	0.014673594	0.988292586	0.999527751	ENSG00000111785	RIC8B
184.1974593	-0.247583096	0.292085856	-0.847638086	0.396639557	0.988378925	ENSG00000102547	CAB39L
185.3898401	0.535228323	0.273976546	1.95355526	0.050753836	0.842188482	ENSG00000163728	TTC14
185.2654739	-0.236862939	0.369698855	-0.640691567	0.521723094	0.993980889	ENSG00000214013	GANC

186.4063306	-0.225339031	0.359865234	-0.626176162	0.531199401	0.99483168	ENSG00000175213	ZNF408	
184.9880257	0.071872675	0.265557209	0.270648556	0.786661347	0.999467288	ENSG00000172409	CLP1	
185.1058277	-0.26326417	0.322855549	-0.815424021	0.414829639	0.990226751	ENSG00000108384	RAD51C	
184.850698	0.852121608	0.362838301	2.348488584	0.018849777	0.726053908	ENSG00000185246	PRPF39	
186.3074506	0.337923231	0.311386392	1.085221577	0.27782353	0.962465655	ENSG00000245694	CRNDE	
185.2959342	-0.660812062	0.340006027	-1.943530438	0.051952101	0.845934837	ENSG00000160221	GATD3A	
182.3008583	-0.595231621	0.303257533	-1.96279253	0.049670286	0.836346249	ENSG00000056998	GYG2	
184.0835428	-0.307051718	0.31726401	-0.967811375	0.333138582	0.976026345	ENSG00000166166	TRMT61A	
184.0051554	-0.115389476	0.407848173	-0.282922625	0.777236151	0.999467288	ENSG00000249242	TMEM150C	
183.2323747	-0.492573334	0.290345121	-1.696509765	0.089789382	0.910413547	ENSG00000134809	TIMM10	
186.4911558	0.522587563	0.318466976	1.640947422	0.10080833	0.92169898	ENSG00000149289	ZC3H12C	
182.1599939	-0.738147928	0.365350897	-2.020380775	0.043343906	0.807325344	ENSG00000160256	FAM207A	
182.4651218	0.128921437	0.407997765	0.315985646	0.752013403	0.999467288	ENSG00000162599	NFIA	
182.7696245	-0.16350339	0.350112568	-0.467002344	0.640498201	0.996553586	ENSG00000185532	PRKG1	
182.9322839	-0.058447451	0.278084373	-0.210178839	0.833528094	0.999467288	ENSG00000257218	GATC	
182.6165776	-0.202678194	0.341544672	-0.593416354	0.552902547	0.99483168	ENSG00000139625	MAP3K12	
186.0850154	2.18620094	1.137403231	1.922098408	0.054593376	0.847598083	ENSG00000169679	BUB1	
185.7441456	2.108929176	1.221398323	1.726651441	0.084230281	0.901307093	ENSG00000164611	PTTG1	
182.4898684	0.075530033	0.278556248	0.27114823	0.786277028	0.999467288	ENSG00000139546	TARBP2	
184.3264078	0.035817804	0.408658286	0.087647321	0.93015699	0.999527751	ENSG00000105656	ELL	
182.1260865	-0.144856149	0.299475003	-0.483700299	0.628598581	0.99483168	ENSG00000104825	NFKBIB	
184.1837783	0.59992003	0.341286202	1.757820932	0.078777983	0.897052166	ENSG00000102466	FGF14	
183.524782	-0.092482985	0.271580952	-0.34053561	0.733453211	0.999467288	ENSG00000173614	NMNAT1	
183.724131	-0.034609083	0.350849577	-0.098643651	0.92142121	0.999527751	ENSG00000020633	RUNX3	
180.3039503	-0.034004341	0.379116617	-0.089693617	0.928530688	0.999527751	ENSG00000179085	DPM3	
183.8266044	0.516279616	0.334342718	1.544162884	0.122548878	0.92570179	ENSG00000137871	ZNF280D	
183.6802919	-0.070741713	0.281933007	-0.250916747	0.801878476	0.999467288	ENSG00000157778	PSMG3	
182.3163064	0.212491828	0.256271491	0.829166859	0.407009995	0.990226751	ENSG00000125843	AP5S1	
184.2329847	0.192318118	0.267577128	0.718739	0.472301749	0.99135833	ENSG00000269713	NBPF9	
180.7820204	0.088382118	0.337195053	0.26210977	0.793236815	0.999467288	ENSG00000204435	CSNK2B	
182.9702444	-0.176467974	0.318566795	-0.553943402	0.57961758	0.99483168	ENSG00000226084	AC113935.1	
180.4161875	0.107169354	0.321604329	0.333233555	0.738957991	0.999467288	ENSG00000181192	DHTKD1	
182.5062316	-0.083345335	0.324486682	-0.256852867	0.797292357	0.999467288	ENSG00000205084	TMEM231	
183.2648429	-0.130117848	0.305022958	-0.426583784	0.669682511	0.997568624	ENSG00000240344	PPIL3	
182.1416179	0.084869719	0.349140994	0.243081508	0.807942256	0.999467288	ENSG00000139531	SUOX	
180.3656739	0.214720608	0.3995974	0.537342354	0.591031154	0.99483168	ENSG00000139266		9-Mar
179.0680739	0.155166863	0.40820759	0.380117536	0.703858169	0.99900742	ENSG00000165475	CRYL1	
181.887892	-0.441987584	0.37374455	-1.182592722	0.236970599	0.962465655	ENSG00000161277	THAP8	
180.951203	0.243726423	0.428469912	0.568829727	0.569471699	0.99483168	ENSG00000241852	C8orf58	
183.8335485	-0.177411843	0.377048887	-0.470527428	0.637978243	0.996553586	ENSG00000111058	ACSS3	
183.9650834	-0.264863119	0.284458632	-0.931112962	0.351795135	0.977691382	ENSG00000092978	GPATCH2	
182.8579614	0.187971313	0.410386364	0.458034988	0.646927305	0.997368416	ENSG00000173085	COQ2	
184.2618557	0.43174998	0.325088098	1.328101465	0.18414459	0.949094286	ENSG00000222009	BTBD19	
178.5882215	0.237348822	0.449383175	0.528165797	0.597384266	0.99483168	ENSG00000184588	PDE4B	
181.5126841	0.344726956	0.307025595	1.122795499	0.261524357	0.962465655	ENSG00000138050	THUMPD2	
183.2533917	0.030724512	0.290255586	0.1058533	0.915698747	0.999527751	ENSG00000189180	ZNF33A	
181.1533044	0.192882975	0.330924292	0.582861335	0.559986651	0.99483168	ENSG00000103064	SLC7A6	
180.8609251	0.141731784	0.342500045	0.413815373	0.679009324	0.998869317	ENSG00000164039	BDH2	
181.5611881	0.114152788	0.260038896	0.438983515	0.660673481	0.997568624	ENSG00000114735	HEMK1	
183.1387294	-0.060859461	0.384469975	-0.158294445	0.874224785	0.999467288	ENSG00000075399	VPS9D1	
179.3960081	-0.184364685	0.337127426	-0.546869435	0.584468436	0.99483168	ENSG00000242259	C22orf39	
181.1914975	-0.121056319	0.297103167	-0.407455499	0.683673472	0.998869317	ENSG00000137494	ANKRD42	
179.1340495	-0.306951347	0.439240354	-0.698823192	0.484662533	0.991892545	ENSG00000111052	LIN7A	
182.9935125	0.001143207	0.319977428	0.003572773	0.997149346	0.999527751	ENSG00000066427	ATXN3	
179.3728201	0.127875563	0.309878849	0.412663087	0.679853473	0.998869317	ENSG00000168016	TRANK1	
182.1319894	-0.033026986	0.316125051	-0.104474434	0.916792856	0.999527751	ENSG00000028116	VRK2	
179.1195647	-0.366412507	0.310334179	-1.180703034	0.237720716	0.962465655	ENSG00000182568	SATB1	
179.4479228	-0.08187763	0.292973779	-0.279470847	0.779883508	0.999467288	ENSG00000160325	CACFD1	
180.5880325	0.106949723	0.29365567	0.364201117	0.715707833	0.99900742	ENSG00000153391	INO80C	
182.1898136	0.365810596	0.282456187	1.295105621	0.195283796	0.953843167	ENSG00000186020	ZNF529	
180.1699988	-0.237458387	0.267239033	-0.888561766	0.374238647	0.982609538	ENSG00000197885	NKIRAS1	
185.411998	2.879511368	1.669068843	1.725220252	0.084487787	0.901307093	ENSG00000066279	ASPM	
180.0027802	-0.557885784	0.334849348	-1.666079951	0.095697491	0.921147998	ENSG00000183648	NDUFB1	
179.8274542	-0.10750895	0.327105507	-0.328667501	0.742407021	0.999467288	ENSG00000213339	QTRT1	

180.1040018	0.015342529	0.352511862	0.043523439	0.96528428	0.999527751	ENSG00000151917	BEND6
179.6187001	-0.028088687	0.271850066	-0.103324185	0.917705681	0.999527751	ENSG00000149089	APIP
180.4530734	-0.242154021	0.315567166	-0.767361267	0.442866751	0.99135833	ENSG00000165572	KBTBD6
181.062329	0.119520062	0.265024834	0.45097684	0.652006241	0.997449432	ENSG00000115750	TAF1B
180.5731027	0.260292416	0.377135957	0.690181913	0.490079796	0.993513219	ENSG00000169288	MRPL1
181.6450865	-0.41183647	0.310796388	-1.325100566	0.185137798	0.949094286	ENSG00000174943	KCTD13
183.1573258	0.390939922	0.346232114	1.129126696	0.258844391	0.962465655	ENSG00000197121	PGAP1
180.896674	-0.369374174	0.258704934	-1.427781715	0.153354697	0.939432741	ENSG00000106733	NMRK1
180.7434875	-0.48917314	0.307663492	-1.589961607	0.111843459	0.923922314	ENSG00000116128	BCL9
179.9162843	-0.208746708	0.325063224	-0.642172637	0.520761097	0.993513219	ENSG00000125459	MSTO1
178.5343518	-0.177735062	0.373119555	-0.476348825	0.633825886	0.995376704	ENSG00000179604	CDC42EP4
181.2180292	0.010427423	0.284848858	0.036606864	0.970798471	0.999527751	ENSG00000105821	DNAJC2
178.8721381	1.052935507	0.603690819	1.744163525	0.08113058	0.897807954	ENSG00000174442	ZWILCH
180.5017844	-0.024800047	0.275646592	-0.089970446	0.9283107	0.999527751	ENSG00000113966	ARL6
178.1988644	-0.277994955	0.278711937	-0.997427519	0.31855704	0.974768742	ENSG00000175581	MRPL48
178.536752	0.151703082	0.497997581	0.304626144	0.760650906	0.999467288	ENSG00000259207	ITGB3
182.2688229	0.034397066	0.360904426	0.095307965	0.924070216	0.999527751	ENSG00000151151	IPMK
178.2694932	-0.620590981	0.367046398	-1.690769845	0.090880769	0.912649921	ENSG00000183617	MRPL54
178.6498646	0.36276096	0.29021937	1.249954338	0.211316228	0.954310328	ENSG00000129347	KRI1
181.1362955	-0.105390743	0.290611559	-0.362651587	0.716865169	0.99900742	ENSG00000131437	KIF3A
180.2224885	-0.003964358	0.318594908	-0.012443255	0.990071975	0.999527751	ENSG00000132394	EEFSEC
180.3504467	-0.423703373	0.288117389	-1.47059285	0.141401259	0.935644597	ENSG00000170846	AC093323.1
180.1934732	0.671666888	0.344349428	1.95053871	0.051111944	0.842188482	ENSG00000129534	MIS18BP1
178.9877308	-0.365373333	0.320715893	-1.13924299	0.25460182	0.962465655	ENSG00000161956	SENP3
182.3149917	0.539222754	0.354726537	1.520108301	0.128483759	0.928627271	ENSG00000198707	CEP290
179.0456259	0.119362612	0.273972789	0.435673238	0.663073826	0.997568624	ENSG00000168872	DDX19A
181.3557536	0.426570692	0.298615683	1.428493934	0.153149739	0.939432741	ENSG00000177058	SLC38A9
179.9855396	0.174372292	0.314396984	0.554624568	0.579151481	0.99483168	ENSG00000175104	TRAF6
179.8144429	0.169219641	0.32598899	0.519096186	0.603693668	0.99483168	ENSG00000172339	ALG14
178.553203	0.215303796	0.269681616	0.798362897	0.424659928	0.991301167	ENSG00000153767	GTF2E1
178.8750545	-0.412306588	0.317465699	-1.298743736	0.194031889	0.950624691	ENSG00000080561	MID2
179.3843004	0.07783503	0.280894606	0.27709692	0.781705687	0.999467288	ENSG00000072134	EPN2
177.2773933	0.071096872	0.315207031	0.225556111	0.821546679	0.999467288	ENSG00000109911	ELP4
176.8673285	-0.230178954	0.348769824	-0.659973821	0.509270629	0.993513219	ENSG00000157379	DHRS1
176.0954593	-0.72846469	0.497218197	-1.465080512	0.142898953	0.935644597	ENSG00000163131	CTSS
178.0545424	0.09442945	0.420990018	0.224303298	0.82252131	0.999467288	ENSG00000073849	ST6GAL1
177.9094878	0.005849994	0.280834512	0.020830751	0.983380667	0.999527751	ENSG00000100104	SRRD
178.1076273	0.005492228	0.292376162	0.018784802	0.985012778	0.999527751	ENSG00000065491	TBC1D22B
178.6058005	0.334461322	0.306597698	1.090880083	0.275325652	0.962465655	ENSG00000137411	VARS2
180.4333621	0.220710619	0.37604883	0.586920106	0.557257366	0.99483168	ENSG00000153976	HS3ST3A1
177.375706	-0.027025845	0.335931974	-0.080450351	0.935879083	0.999527751	ENSG00000163170	BOLA3
178.0664257	-0.306002548	0.390849191	-0.782917186	0.433675745	0.991301167	ENSG00000160233	LRRC3
177.0868298	-0.282192659	0.288346676	-0.978657575	0.327749201	0.976026345	ENSG00000185305	ARL15
178.9344495	-0.135768892	0.269260177	-0.504229378	0.614100198	0.99483168	ENSG00000115282	TTC31
176.2457419	0.722555353	0.560729154	1.288599581	0.197537329	0.954310328	ENSG00000170962	PDGFD
180.4226535	-0.725189722	0.458947789	-1.580113773	0.114080814	0.923922314	ENSG00000143473	KCNH1
179.2499114	0.224861913	0.330345227	0.680687641	0.496069158	0.993513219	ENSG00000185238	PRMT3
177.2017236	0.114246498	0.303671259	0.376217683	0.70675507	0.99900742	ENSG00000105483	CARD8
177.6926213	0.214873011	0.270350352	0.794794639	0.42673297	0.991301167	ENSG00000032219	ARID4A
177.0947077	0.397151858	0.324353808	1.224440251	0.220786193	0.956884001	ENSG00000112312	GMNN
178.5109851	0.390191253	0.33217938	1.1746402	0.240138693	0.962465655	ENSG00000163482	STK36
177.3376558	0.095770324	0.374195305	0.255936733	0.79799969	0.999467288	ENSG00000105321	CCDC9
175.7892168	0.382630869	0.295692131	1.294017763	0.195659285	0.954310328	ENSG00000085185	BCORL1
178.1243465	0.212563854	0.303390098	0.700628844	0.483534673	0.991892545	ENSG00000148384	INPP5E
180.607379	2.499391659	1.474238782	1.69537777	0.09000378	0.910413547	ENSG00000157456	CCNB2
177.2595316	0.064586179	0.29404352	0.219648366	0.826145021	0.999467288	ENSG00000114388	NPRL2
178.7478648	0.067574855	0.313189517	0.215763465	0.829172128	0.999467288	ENSG00000128563	PRKRIP1
176.2536475	-0.387120329	0.280825026	-1.378510792	0.168045638	0.943223078	ENSG00000129197	RPAIN
176.8011481	0.008358946	0.364589628	0.022926998	0.981708505	0.999527751	ENSG00000116017	ARID3A
177.2986144	0.006395574	0.373710581	0.017113708	0.986345903	0.999527751	ENSG00000170365	SMAD1
176.0831117	-0.121294501	0.29996958	-0.404356004	0.685950948	0.998869317	ENSG00000074582	BCS1L
175.4781536	0.148347624	0.3419892	0.43377868	0.664449165	0.997568624	ENSG00000180611	MB21D2
177.8576592	0.109400653	0.31343323	0.34903974	0.727059475	0.999467288	ENSG00000125352	RNF113A
177.2913532	-0.084631308	0.268491531	-0.315210345	0.752601951	0.999467288	ENSG00000196756	SNHG17

173.1219889	0.678032165	0.390342278	1.737019539	0.082383722	0.899922174	ENSG00000047634	SCML1
177.6007691	0.049026081	0.290626229	0.168691179	0.866039557	0.999467288	ENSG00000105793	GTPBP10
176.4292035	-0.677706015	0.313020147	-2.165055573	0.03038343	0.775963115	ENSG00000125611	CHCHD5
175.9479787	0.151301964	0.312539268	0.484105453	0.628311031	0.99483168	ENSG00000125744	RTN2
177.1365599	-0.705200909	0.306089672	-2.30390299	0.02122809	0.73997555	ENSG00000196369	SRGAP2B
174.6933458	-0.140484163	0.41182845	-0.341123017	0.733010974	0.999467288	ENSG00000158717	RNF166
176.686193	0.098999795	0.312847039	0.31644792	0.751662549	0.999467288	ENSG00000247315	ZCCHC3
176.155065	-0.00674689	0.285669559	-0.023617811	0.981157465	0.999527751	ENSG00000232677	LINC00665
174.4731802	-0.292009912	0.315333815	-0.926034249	0.354428182	0.977691382	ENSG00000175701	MTLN
173.5515442	0.305550877	0.398655078	0.766454244	0.443406067	0.99135833	ENSG00000135899	SP110
174.826866	-0.323343363	0.323264282	-1.000244633	0.317192134	0.974768742	ENSG00000150433	TMEM218
174.9156975	-0.298149021	0.352645352	-0.845464202	0.397851712	0.988378925	ENSG00000158882	TOMM40L
179.913366	0.108996127	0.405225851	0.268976242	0.787947965	0.999467288	ENSG00000165626	BEND7
175.7371234	-0.017236333	0.325291444	-0.052987355	0.957741983	0.999527751	ENSG00000109458	GAB1
175.459162	0.1738214	0.282795687	0.614653648	0.538783477	0.99483168	ENSG00000175354	PTPN2
177.3162068	-0.017228902	0.320822054	-0.053702362	0.957172301	0.999527751	ENSG00000140386	SCAPER
174.6295651	0.012413834	0.320128015	0.038777718	0.96906761	0.999527751	ENSG00000169371	SNUPN
173.603803	1.421837429	0.636195762	2.234905532	0.025423551	0.759116406	ENSG00000276043	UHRF1
176.6964423	-0.449544321	0.375507377	-1.197165085	0.231242216	0.959575179	ENSG00000182580	EPHB3
176.0311848	0.031929482	0.289978429	0.110109853	0.912322254	0.999527751	ENSG00000147687	TATDN1
175.6994184	0.671817956	0.400639929	1.676862208	0.09356943	0.921147998	ENSG00000149503	INCENP
173.633259	-0.275731076	0.293298535	-0.940103829	0.347164305	0.977691382	ENSG00000106351	AGFG2
174.4581803	0.129693556	0.337267014	0.384542665	0.70057628	0.998869317	ENSG00000129484	PARP2
174.5557654	-0.04630742	0.284190745	-0.162944858	0.870561851	0.999467288	ENSG00000180979	LRRC57
174.5910921	0.236395215	0.296234603	0.798000007	0.424870486	0.991301167	ENSG00000011260	UTP18
177.1565286	-0.016014455	0.347200596	-0.046124503	0.963211016	0.999527751	ENSG00000088179	PTPN4
175.6393727	0.544666985	0.351422789	1.549890906	0.121167703	0.923922314	ENSG00000116815	CD58
173.2338316	0.004943919	0.374201725	0.013211909	0.989458728	0.999527751	ENSG00000110921	MVK
175.2324658	0.431196384	0.28307057	1.523282281	0.127688098	0.928627271	ENSG00000162929	KIAA1841
175.1994071	0.329966674	0.284366825	1.160355726	0.245904005	0.962465655	ENSG00000127980	PEX1
175.692811	-0.336265664	0.443186052	-0.758746045	0.448004487	0.99135833	ENSG00000266208	AC080112.1
174.2943707	0.192776308	0.260315686	0.740548182	0.458967437	0.99135833	ENSG00000162851	TFB2M
180.0991681	0.388080663	0.48081745	0.807126828	0.419593417	0.990226751	ENSG00000178607	ERN1
170.1456401	-0.143480442	0.435063521	-0.329791938	0.74155718	0.999467288	ENSG00000185885	IFITM1
175.6267721	-0.274869473	0.296639314	-0.926611749	0.354128152	0.977691382	ENSG00000175575	PAAF1
175.6422951	-0.315272098	0.322450719	-0.977737309	0.328204264	0.976026345	ENSG00000161618	ALDH16A1
175.8490363	0.274737587	0.28307663	0.970541396	0.331776701	0.976026345	ENSG00000120370	GORAB
175.2229147	-0.6782296	0.29984475	-2.261935887	0.023701365	0.744948012	ENSG00000239264	TXNDC5
175.3322176	0.231520897	0.284301494	0.814349913	0.415444523	0.990226751	ENSG00000160336	ZNF761
179.3699649	0.817571564	0.543651585	1.503852074	0.132619459	0.933741583	ENSG00000163393	SLC22A15
173.8629281	-0.400952117	0.313294391	-1.279793474	0.20061778	0.954310328	ENSG00000162385	MAGOH
172.8868139	-0.334731381	0.387613206	-0.863570632	0.387823803	0.986799595	ENSG00000168765	GSTM4
176.971018	0.072996999	0.333257822	0.219040617	0.826618408	0.999467288	ENSG00000101574	METTL4
174.6934542	0.206395046	0.339377886	0.608157028	0.543083332	0.99483168	ENSG00000115459	ELMOD3
174.8806724	-0.507042349	0.344881553	-1.470192721	0.141509565	0.935644597	ENSG00000129911	KLF16
173.1911207	0.313853717	0.356099479	0.881365279	0.378120144	0.982609538	ENSG00000250742	LINC02381
172.8953385	0.155381508	0.33947717	0.457708271	0.647162045	0.997368416	ENSG00000121753	ADGRB2
173.8848757	0.07481686	0.291113616	0.25700227	0.797177021	0.999467288	ENSG00000164080	RAD54L2
173.1065371	-0.132780974	0.338454235	-0.392315888	0.694824826	0.998869317	ENSG00000170791	CHCHD7
176.6600353	0.391429837	0.359161051	1.089844891	0.275781475	0.962465655	ENSG00000198920	KIAA0753
171.9494496	-0.172151119	0.345058177	-0.498904619	0.61784658	0.99483168	ENSG00000186187	ZNRF1
167.7299858	-0.259136168	0.50000488	-0.518267277	0.6042718	0.99483168	ENSG00000184160	ADRA2C
173.2126695	-0.078399644	0.297957603	-0.263123488	0.792455401	0.999467288	ENSG00000166478	ZNF143
170.7833041	-0.602108915	0.326103014	-1.84637642	0.064837569	0.868202292	ENSG00000198937	CCDC167
165.6059983	0.390171635	0.606891058	0.642902263	0.520287521	0.993513219	ENSG00000013588	GPRC5A
173.3559844	-0.071833782	0.348899188	-0.205886927	0.836879238	0.999467288	ENSG00000103404	USP31
173.8355567	-0.223922976	0.287810507	-0.778022243	0.436555901	0.991301167	ENSG00000170191	NANP
172.6678235	-0.370144672	0.309438059	-1.196183408	0.231624994	0.959575179	ENSG00000135338	LCA5
173.1165392	-0.158217569	0.324904077	-0.486967017	0.626281705	0.99483168	ENSG00000178761	FAM219B
173.5088041	0.344506445	0.411639382	0.836913231	0.402641343	0.988836748	ENSG00000088035	ALG6
178.7255326	-1.532842934	0.456166902	-3.360267757	0.00077867	0.204478655	ENSG00000128422	KRT17
170.9516362	-0.172161307	0.331308539	-0.519640417	0.603314223	0.99483168	ENSG00000129219	PLD2
173.9015827	0.84719471	0.513551249	1.649678997	0.099008608	0.921147998	ENSG00000230630	DNM3OS
172.7772927	-0.224496991	0.306541626	-0.732354018	0.463952517	0.99135833	ENSG00000124459	ZNF45

173.856893	-0.436341243	0.342359812	-1.274510698	0.202482484	0.954310328	ENSG00000116194	ANGPTL1
172.075207	-0.341441508	0.325027371	-1.050500782	0.293487932	0.970706863	ENSG00000179958	DCTPP1
171.8212339	0.293291909	0.289595729	1.012763239	0.311173264	0.973427483	ENSG00000131732	ZCCHC9
172.569583	0.051379989	0.292463768	0.175679842	0.860545472	0.999467288	ENSG00000013503	POLR3B
169.5015686	-0.492436116	0.398570195	-1.235506624	0.216642013	0.954900762	ENSG00000171700	RGS19
173.613974	-0.343264915	0.347436395	-0.987993546	0.323155809	0.975337095	ENSG00000001460	STPG1
171.2764814	-0.599259133	0.295750866	-2.026229512	0.042741271	0.806698895	ENSG00000163795	ZNF513
172.8209635	-0.340272869	0.328318858	-1.03640976	0.300011019	0.972186492	ENSG00000204438	GPANK1
171.1022205	-0.099925453	0.273655955	-0.365149932	0.71499949	0.99900742	ENSG00000132275	RRP8
170.8933589	0.751276352	0.267030041	2.81345256	0.004901262	0.472943531	ENSG00000086712	TXLNG
172.1408369	0.142514977	0.308534343	0.461909606	0.644146145	0.997332006	ENSG00000204116	CHIC1
171.6704779	0.043139253	0.298640411	0.144452163	0.885143428	0.999467288	ENSG00000198917	SPOUT1
171.2550156	-0.172840964	0.341450297	-0.506196554	0.612718674	0.99483168	ENSG00000254122	PCDHGB7
170.7477512	0.44425999	0.32990983	1.346610345	0.17810576	0.9469453	ENSG00000137413	TAF8
170.3162312	0.291685593	0.31497668	0.926054569	0.354417622	0.977691382	ENSG00000137700	SLC37A4
171.2515562	-0.174095237	0.301986711	-0.576499663	0.564277498	0.99483168	ENSG00000166136	NDUFB8
171.1559175	0.316070763	0.32755679	0.964934241	0.334577749	0.976026345	ENSG00000069020	MAST4
172.4528411	-0.063226219	0.35106225	-0.180099737	0.857074269	0.999467288	ENSG00000142082	SIRT3
173.1916419	0.354554291	0.346052268	1.024568607	0.305566783	0.973427483	ENSG00000171365	CLCN5
171.669526	-0.17716178	0.344317989	-0.514529549	0.606881807	0.99483168	ENSG00000006625	GGCT
170.3744601	-0.147099432	0.358874603	-0.409890895	0.681885985	0.998869317	ENSG00000165752	STK32C
174.0914201	-0.488426933	0.402862871	-1.212390042	0.225363111	0.958865115	ENSG00000105088	OLFM2
172.2514456	0.688978058	0.395652516	1.741371607	0.081618462	0.897807954	ENSG00000120539	MASTL
171.9488243	-0.393308211	0.648035821	-0.606923565	0.543901639	0.99483168	ENSG00000069431	ABCC9
172.041935	0.659621449	0.352702795	1.870190593	0.061457356	0.864834166	ENSG00000139974	SLC38A6
171.7759159	-0.236456081	0.312948634	-0.755574733	0.44990421	0.99135833	ENSG00000141198	TOM1L1
169.6355872	0.050288783	0.318919503	0.15768488	0.874705116	0.999467288	ENSG00000114923	SLC4A3
170.7630969	-0.015846443	0.319936207	-0.049530007	0.960496925	0.999527751	ENSG00000140263	SORD
172.5181009	0.921174634	0.386186846	2.385308156	0.01706482	0.715620174	ENSG00000159086	PAXBP1
169.7870352	0.007912512	0.358662258	0.02206118	0.982399153	0.999527751	ENSG00000069399	BCL3
169.9170004	-0.397504365	0.296340997	-1.341374864	0.179798774	0.948095545	ENSG00000157895	C12orf43
171.105736	-0.362279572	0.318867554	-1.136144359	0.255896153	0.962465655	ENSG00000188428	BLOC1S5
170.0910581	0.003502314	0.276703505	0.012657282	0.98990122	0.999527751	ENSG00000180098	TRNAU1AP
170.8403498	-0.162776363	0.437471579	-0.37208443	0.709829988	0.99900742	ENSG00000109906	ZBTB16
172.1350433	-0.227632955	0.303364019	-0.750362403	0.453036468	0.99135833	ENSG00000132005	RFX1
170.6831407	0.008730202	0.30245446	0.028864519	0.976972644	0.999527751	ENSG00000105732	ZNF574
171.6643014	-0.092423563	0.305079035	-0.302949573	0.761928292	0.999467288	ENSG00000143258	USP21
170.2865495	0.518294473	0.28536398	1.816257516	0.069330879	0.877603755	ENSG00000108506	INTS2
166.162698	-0.449513581	0.534102038	-0.841624912	0.399997941	0.988492517	ENSG00000101265	RASSF2
170.8949623	0.136466772	0.265616308	0.513774072	0.607409958	0.99483168	ENSG00000115998	C2orf42
168.4630545	-0.635233641	0.404112132	-1.571924203	0.115968131	0.923922314	ENSG00000226137	BAIAP2-DT
169.3882891	0.200897452	0.289794174	0.693241859	0.488157783	0.993513219	ENSG00000212978	AC016747.1
169.5247746	-0.08637095	0.294675961	-0.293104839	0.769442018	0.999467288	ENSG00000139437	TCHP
170.5660692	0.119986618	0.2721852	0.440827122	0.659338157	0.997568624	ENSG00000196236	XPNPEP3
169.7092095	-1.182104883	0.641132486	-1.843776299	0.065215748	0.868202292	ENSG00000159307	SCUBE1
172.5425641	1.43998188	1.108994976	1.298456631	0.19413047	0.950771915	ENSG00000087586	AURKA
169.0416193	0.198829385	0.389520457	0.510446579	0.609738631	0.99483168	ENSG00000132967	HMGB1P5
169.4913831	-0.205128274	0.317409356	-0.646257808	0.518112407	0.993513219	ENSG00000169193	CCDC126
167.5715821	0.006304207	0.312934469	0.020145454	0.983927341	0.999527751	ENSG00000102309	PIN4
169.6731492	0.04044279	0.288070702	0.140391889	0.888350366	0.999467288	ENSG00000119965	C10orf88
171.4284305	-0.091097739	0.403852895	-0.225571588	0.821534641	0.999467288	ENSG00000118620	ZNF430
170.8714788	-0.586667573	0.319117456	-1.8384064	0.066002543	0.872163518	ENSG00000169184	MN1
166.012454	-0.104751273	0.494063495	-0.212019861	0.83209154	0.999467288	ENSG00000162944	RFTN2
168.6635998	0.100490962	0.361397269	0.278062316	0.780964523	0.999467288	ENSG00000205208	C4orf46
169.2412997	-0.229756015	0.486147384	-0.47260568	0.63649453	0.99653524	ENSG00000148143	ZNF462
169.5845367	-0.594657039	0.326648056	-1.820482409	0.068685573	0.87420149	ENSG00000167528	ZNF641
168.9891961	-0.674967609	0.399286029	-1.69043633	0.09094451	0.912649921	ENSG00000232187	FTH1P7
168.4944158	0.14544402	0.3036572	0.478974381	0.631956854	0.99483168	ENSG00000166965	RCCD1
169.8371318	-0.35882454	0.282944038	-1.268182015	0.204732958	0.954310328	ENSG00000138036	DYNC2LI1
168.2904697	0.400051181	0.334794031	1.194917303	0.232119342	0.959575179	ENSG00000038210	PI4K2B
168.9111339	0.073976558	0.3199847	0.231187798	0.817168909	0.999467288	ENSG00000153487	ING1
168.2031884	0.047014743	0.327519781	0.143547796	0.885857566	0.999467288	ENSG00000116874	WARS2
169.1781928	-0.049718278	0.270837512	-0.183572349	0.854348957	0.999467288	ENSG00000096080	MRPS18A
168.2144831	0.100066583	0.293762432	0.340637781	0.733376284	0.999467288	ENSG00000144535	DIS3L2

168.9288177	-0.28668441	0.277676204	-1.032441404	0.301865384	0.972793733	ENSG00000145945	FAM50B
166.6299815	0.217468156	0.354641514	0.613205582	0.539740414	0.99483168	ENSG00000127124	HIVEP3
168.6538262	-0.231146025	0.299159805	-0.772650674	0.439729146	0.991301167	ENSG00000092531	SNAP23
158.167632	-0.11133833	0.702061846	-0.158587638	0.873993769	0.999467288	ENSG00000117266	CDK18
166.7880325	0.202403492	0.315523625	0.641484427	0.521207995	0.993513219	ENSG00000172728	FUT10
168.6098036	0.426859611	0.31226295	1.366987696	0.171629162	0.944422693	ENSG00000139180	NDUFA9
169.2983056	0.32325586	0.289479619	1.11667917	0.264131527	0.962465655	ENSG00000118965	WDR35
168.6030378	0.029048722	0.304001121	0.095554655	0.92387428	0.999527751	ENSG00000135185	TMEM243
168.3182477	-0.245771414	0.373506586	-0.658010926	0.51053111	0.993513219	ENSG00000026297	RNASET2
167.6905912	-0.218587068	0.309665453	-0.705881352	0.480261925	0.99135833	ENSG00000156958	GALK2
173.8640792	-0.088497293	0.504064958	-0.17556724	0.860633941	0.999467288	ENSG00000173334	TRIB1
167.7004542	0.455365485	0.308511897	1.476006241	0.139942217	0.935644597	ENSG00000064933	PMS1
166.6672144	0.140598879	0.338984196	0.414765291	0.67831373	0.998869317	ENSG00000145982	FARS2
165.3812942	0.056479306	0.398419985	0.141758214	0.887270991	0.999467288	ENSG00000078967	UBE2D4
167.0967692	0.088703503	0.316220986	0.280511119	0.779085396	0.999467288	ENSG00000128881	TTBK2
167.5914055	0.453877426	0.262447907	1.729400057	0.08373752	0.901307093	ENSG00000102125	TAZ
169.101912	0.180557988	0.362725886	0.497780818	0.618638538	0.99483168	ENSG00000089775	ZBTB25
165.7054001	0.389260718	0.503370144	0.773309111	0.439339465	0.991301167	ENSG00000111801	BTN3A3
166.1321151	1.337263475	0.516365268	2.58976263	0.009604213	0.622034307	ENSG00000111981	ULBP1
169.3814245	0.014358049	0.317245183	0.045258525	0.963901245	0.999527751	ENSG00000143748	NVL
167.9973814	-0.011231258	0.300172799	-0.037415974	0.970153336	0.999527751	ENSG00000206562	METTL6
168.0749124	0.059858149	0.307314837	0.194777935	0.845566797	0.999467288	ENSG00000137822	TUBGCP4
164.0980691	-0.510199543	0.344014203	-1.483076971	0.138053962	0.934144427	ENSG00000244187	TMEM141
167.8153834	-0.093137335	0.334901788	-0.278103426	0.780932966	0.999467288	ENSG00000101391	CDK5RAP1
166.4657708	0.049019845	0.341577782	0.143510053	0.885887373	0.999467288	ENSG00000135972	MRPS9
166.5369026	-0.413639146	0.344761835	-1.199782294	0.230223902	0.959575179	ENSG00000169020	ATP5ME
167.1796996	0.042456193	0.308725911	0.137520665	0.89061926	0.999467288	ENSG00000138794	CASP6
165.5573079	0.126250852	0.342716942	0.368382291	0.7125882	0.99900742	ENSG00000187257	RSBN1L
167.0140151	-0.136241212	0.346672152	-0.392997277	0.694321492	0.998869317	ENSG00000067992	PDK3
164.529275	-0.048255677	0.349943723	-0.137895535	0.890322979	0.999467288	ENSG00000143409	MINDY1
166.7989157	0.137667049	0.291910615	0.471606862	0.637207428	0.99653524	ENSG00000196214	ZNF766
167.0350498	-0.007295115	0.286386263	-0.025472994	0.979677689	0.999527751	ENSG00000181894	ZNF329
167.8998914	0.439734015	0.3109651	1.414094428	0.157334179	0.939464191	ENSG00000121310	ECHDC2
165.7514307	-0.090727143	0.329593141	-0.275270118	0.783108721	0.999467288	ENSG00000148362	PAXX
166.0103233	-0.123799856	0.306575693	-0.403814976	0.686348783	0.998869317	ENSG00000114026	OGG1
166.6426686	1.787961772	0.442720101	4.038582772	5.38E-05	0.054234087	ENSG00000153162	BMP6
167.900083	-0.35196288	0.300734793	-1.170343068	0.241862937	0.962465655	ENSG00000173653	RCE1
161.4854629	-0.264107502	0.620508472	-0.425630776	0.670376911	0.997568624	ENSG00000137501	SYTL2
166.0738548	0.241959757	0.273878285	0.883457249	0.376989271	0.982609538	ENSG00000156469	MTERF3
168.2507009	0.657261095	0.501552394	1.310453509	0.190042462	0.949094286	ENSG00000146938	NLGN4X
165.1882654	-0.133151374	0.313212618	-0.425114975	0.670752862	0.997568624	ENSG00000063176	SPHK2
168.2368644	0.707279371	0.350348447	2.01878837	0.04350922	0.807325344	ENSG00000079134	THOC1
162.2849024	0.165849945	0.593364241	0.279507819	0.779855138	0.999467288	ENSG00000186352	ANKRD37
164.1262033	-0.146900932	0.401182251	-0.366170067	0.714238177	0.99900742	ENSG00000132879	FBXO44
165.5437502	0.167689077	0.331989071	0.505104209	0.613485645	0.99483168	ENSG00000130349	C6orf203
162.213146	-0.318687131	0.395106259	-0.806585882	0.41990511	0.9906386	ENSG00000162437	RAVER2
164.7664317	-0.305332188	0.354673603	-0.860882191	0.389302931	0.987216662	ENSG00000176410	DNAJC30
165.8757033	-0.36440411	0.315394191	-1.155392585	0.247929697	0.962465655	ENSG00000196372	ASB13
163.8839641	0.541696547	0.318398889	1.701314188	0.088884005	0.910413547	ENSG00000037042	TUBG2
165.3016673	0.386843447	0.29350892	1.317995537	0.187505142	0.949094286	ENSG00000134758	RNF138
166.0021216	-0.086175225	0.284495537	-0.302905366	0.761961982	0.999467288	ENSG00000018610	CXorf56
161.3249853	0.688060748	0.454786584	1.512931058	0.130297172	0.929373153	ENSG00000147862	NFIB
164.7149216	-0.151127695	0.293091439	-0.515633263	0.606110574	0.99483168	ENSG00000142444	TIMM29
165.2163309	0.017797576	0.286549042	0.062110051	0.950475193	0.999527751	ENSG00000186448	ZNF197
163.2132479	-0.053176518	0.353465857	-0.150443152	0.880414999	0.999467288	ENSG00000132541	RIDA
166.2746164	-0.375026457	0.338793408	-1.106947325	0.268316703	0.962465655	ENSG00000011132	APBA3
166.2937295	0.303106639	0.369916302	0.819392489	0.412562519	0.990226751	ENSG00000157193	LRP8
164.5005127	0.011639425	0.291571096	0.03991968	0.968157161	0.999527751	ENSG00000116213	WRAP73
166.4016833	0.502990473	0.318771364	1.577903569	0.114587765	0.923922314	ENSG00000163867	ZMYM6
162.3344803	-0.318797243	0.294295208	-1.083256657	0.278694518	0.963231086	ENSG00000105655	ISYNA1
166.948883	-0.001441252	0.311733623	-0.004623345	0.996311118	0.999527751	ENSG00000156374	PCGF6
165.7868007	0.722087262	0.349622795	2.06533233	0.038891567	0.793985738	ENSG00000184992	BRI3BP
162.8012179	-0.677323506	0.279311979	-2.424971204	0.015309601	0.694606015	ENSG00000069424	KCNAB2
172.0563523	-0.939884176	0.531431446	-1.768589688	0.076962373	0.897052166	ENSG00000185507	IRF7

166.3996915	-0.190432948	0.299408678	-0.636030155	0.524756768	0.99483168	ENSG00000163684	RPP14
164.3137455	-0.165048286	0.309395193	-0.533454592	0.593718936	0.99483168	ENSG00000204650	LINC02210
162.4269188	0.315968708	0.306569287	1.030660021	0.302700279	0.972793733	ENSG00000173638	SLC19A1
164.601028	0.405232104	0.328307983	1.234304754	0.217089365	0.95592125	ENSG00000164010	ERMAP
165.6740598	2.239483916	1.350124329	1.658724214	0.097171378	0.921147998	ENSG00000175063	UBE2C
163.7350901	-0.067504969	0.284966208	-0.236887626	0.81274397	0.999467288	ENSG00000143458	GABPB2
164.7370851	0.444324791	0.286032849	1.553404766	0.120326465	0.923922314	ENSG00000072832	CRMP1
165.1005903	0.394556618	0.36374233	1.084714606	0.278048077	0.962465655	ENSG00000230590	FTX
163.6442812	0.171420972	0.278476024	0.615568155	0.538179574	0.99483168	ENSG00000102977	ACD
163.5163723	-0.184003861	0.345357467	-0.532792479	0.594177241	0.99483168	ENSG00000123836	PFKFB2
161.8239864	0.494467371	0.348079934	1.420556955	0.1554456	0.939464191	ENSG00000070159	PTPN3
164.7500628	0.122420647	0.315146392	0.388456444	0.697678281	0.998869317	ENSG00000108733	PEX12
165.9529378	1.142348296	0.558108357	2.046821699	0.040675594	0.796070921	ENSG00000196739	COL27A1
164.2502047	-0.024455211	0.291313327	-0.083948136	0.933097668	0.999527751	ENSG00000186532	SMYD4
162.8555649	0.568784844	0.287137611	1.980878931	0.047604852	0.827997046	ENSG00000178921	PFAS
162.9524584	0.179327507	0.379014761	0.473141223	0.636112428	0.996399501	ENSG00000141664	ZCCHC2
162.3792762	0.01488282	0.27518648	0.054082672	0.956869298	0.999527751	ENSG00000168067	MAP4K2
159.3697898	0.380379064	0.501323092	0.758750337	0.448001919	0.99135833	ENSG00000075651	PLD1
163.2202963	0.748281039	0.277410053	2.69738256	0.006988693	0.549501061	ENSG00000198625	MDM4
161.839164	-0.466844986	0.366064472	-1.2753081	0.202200212	0.954310328	ENSG00000177432	NAP1L5
163.7953472	1.045373636	0.518993339	2.014233241	0.043985051	0.807325344	ENSG00000140525	FANCI
162.1310569	-0.289294104	0.294037198	-0.983869067	0.325179895	0.976026345	ENSG00000144451	SPAG16
162.3461258	0.065617251	0.317569616	0.206623201	0.83630414	0.999467288	ENSG00000167985	SDHAF2
161.5274224	0.255354652	0.393086679	0.649614109	0.515941517	0.993513219	ENSG00000125247	TMTC4
162.8786699	-0.402864855	0.288194925	-1.397890176	0.162146049	0.943223078	ENSG00000073050	XRCC1
163.1248345	1.090616351	0.524608095	2.07891636	0.037625039	0.790190505	ENSG00000163535	SGO2
155.7393468	0.26808826	0.410871794	0.652486407	0.514087439	0.993513219	ENSG00000197696	NMB
161.3918268	0.323559486	0.285779613	1.132199327	0.257550643	0.962465655	ENSG00000108469	RECQL5
162.377466	-0.012855306	0.283725441	-0.045308964	0.963861043	0.999527751	ENSG00000137513	NARS2
162.4180192	-0.069462746	0.318263532	-0.218255436	0.827230095	0.999467288	ENSG00000127580	WDR24
161.0031141	-0.049176735	0.2946948	-0.166873438	0.867469633	0.999467288	ENSG00000171612	SLC25A33
162.3314102	0.460328579	0.31939821	1.441237188	0.149517686	0.936396974	ENSG00000108773	KAT2A
161.389102	-0.341737854	0.275880433	-1.238717258	0.215450225	0.954310328	ENSG00000168569	TMEM223
164.7185681	-0.158786102	0.395495199	-0.4014868	0.688061755	0.998869317	ENSG00000078687	TNRC6C
159.7072322	-0.05413453	0.386419784	-0.140092541	0.888586874	0.999467288	ENSG00000110987	BCL7A
163.5552985	-0.112842564	0.359579298	-0.313818301	0.753659044	0.999467288	ENSG00000158480	SPATA2
162.8819286	0.202568734	0.299975186	0.675284969	0.499494732	0.993513219	ENSG00000083097	DOP1A
157.6509083	0.4336075	0.559084816	0.775566582	0.438004938	0.991301167	ENSG00000149571	KIRREL3
161.9612011	0.211399789	0.312191953	0.677146825	0.498312802	0.993513219	ENSG00000196867	ZFP28
161.3050665	0.083704514	0.299064913	0.279887446	0.779563859	0.999467288	ENSG00000117308	GALE
160.4287972	0.04031005	0.288861619	0.139547961	0.889017159	0.999467288	ENSG00000130021	PUDP
160.617108	-0.293862252	0.300956398	-0.976427994	0.328852414	0.976026345	ENSG00000077463	SIRT6
161.9211936	0.371973439	0.27542958	1.350521025	0.176848914	0.946540546	ENSG00000197948	FCHSD1
164.5881698	0.608682255	0.410561372	1.482560942	0.138191101	0.934144427	ENSG00000164953	TMEM67
161.894992	-0.0390599	0.312778474	-0.124880399	0.900618237	0.999527751	ENSG00000101811	CSTF2
159.8226601	0.10362198	0.33221333	0.311913974	0.755105902	0.999467288	ENSG00000104299	INTS9
160.0793179	0.611996862	0.343688225	1.780674513	0.074965639	0.893390506	ENSG00000179431	FJX1
160.1842288	0.440687158	0.531141091	0.829698861	0.406709066	0.990226751	ENSG00000120875	DUSP4
159.6598852	0.086782776	0.303725588	0.285727577	0.775086781	0.999467288	ENSG00000111203	ITFG2
160.7490505	-0.109265868	0.305337503	-0.357852759	0.720453505	0.99900742	ENSG00000168556	ING2
162.9484147	0.280500296	0.412336777	0.680269895	0.496333583	0.993513219	ENSG00000135540	NHSL1
159.7765637	-0.001447009	0.314919381	-0.004594856	0.996333848	0.999527751	ENSG00000121851	POLR3GL
162.486831	-0.433132188	0.366010037	-1.183388827	0.236655083	0.962465655	ENSG00000136925	TSTD2
157.7758853	-0.503873846	0.376522327	-1.338230989	0.18082115	0.949094286	ENSG00000178184	PARD6G
162.4098765	-0.598094389	0.361940174	-1.65246754	0.098439277	0.921147998	ENSG00000119771	KLHL29
158.4476455	-0.683327448	0.37574775	-1.818580278	0.068975489	0.875406843	ENSG00000106992	AK1
161.4171002	-0.205616282	0.340218086	-0.60436611	0.545600262	0.99483168	ENSG00000058453	CROCC
158.5364719	2.496461926	1.158873954	2.154213507	0.031223432	0.775963115	ENSG00000113368	LMNB1
159.9679847	0.009302908	0.442616962	0.021017966	0.983231324	0.999527751	ENSG00000156675	RAB11FIP1
160.370593	0.229490055	0.294442282	0.779405911	0.435740643	0.991301167	ENSG00000139719	VPS33A
159.7990411	-0.073897019	0.279587586	-0.264307223	0.791543195	0.999467288	ENSG00000184967	NOC4L
160.1983268	0.178387842	0.347684573	0.513073792	0.607899704	0.99483168	ENSG00000140691	ARMC5
159.8935473	-0.14382056	0.286052649	-0.502776535	0.615121393	0.99483168	ENSG00000174796	THAP6
159.1217535	-0.040592431	0.288306824	-0.140795942	0.888031149	0.999467288	ENSG00000155329	ZCCHC10

160.5368931	3.303342375	1.931368481	1.710363614	0.087198655	0.90492251	ENSG00000126787	DLGAP5
160.5163954	0.088982206	0.278174087	0.319879566	0.749059629	0.999467288	ENSG00000198131	ZNF544
158.6714646	-0.595107016	0.338476663	-1.758192162	0.078714818	0.897052166	ENSG00000100294	MCAT
159.5134691	0.348343745	0.338116814	1.030246741	0.302894193	0.972793733	ENSG00000115526	CHST10
161.283802	0.657318397	0.319288807	2.058695396	0.039523428	0.793985738	ENSG00000139668	WDFY2
161.3595602	0.035131495	0.302329329	0.116202736	0.907491869	0.999527751	ENSG00000166526	ZNF3
160.0208601	0.268773495	0.38759661	0.693436136	0.488035892	0.993513219	ENSG00000110721	CHKA
157.7672411	2.770697479	0.622368967	4.45185674	8.51E-06	0.02049244	ENSG00000221852	KRTAP1-5
160.3544999	0.201198848	0.382963113	0.525373963	0.59932325	0.99483168	ENSG00000261824	LINC00662
162.5721259	0.016909787	0.399323438	0.042346093	0.966222801	0.999527751	ENSG00000204186	ZDBF2
157.6914998	-0.398854507	0.377735195	-1.055910363	0.291009169	0.969337045	ENSG00000204344	STK19
158.689879	0.101238948	0.304477016	0.332501116	0.739510897	0.999467288	ENSG00000066651	TRMT11
159.4255971	0.270400642	0.274213815	0.986094161	0.324086908	0.975459464	ENSG00000047230	CTPS2
158.8998117	-0.246283431	0.35016095	-0.703343507	0.481841704	0.991525194	ENSG00000197620	CXorf40A
157.7269556	0.89525199	0.378615942	2.364538551	0.018052548	0.726053908	ENSG00000069702	TGFBR3
157.2167529	-0.227836056	0.383455708	-0.594165247	0.552401595	0.99483168	ENSG00000244026	FAM86DP
159.568446	-0.095195701	0.319209638	-0.29822314	0.765532862	0.999467288	ENSG00000203705	TATDN3
159.1365237	0.171169081	0.287919247	0.594503781	0.552175214	0.99483168	ENSG00000096872	IFT74
158.9785397	0.076834638	0.289277848	0.26560844	0.790540785	0.999467288	ENSG00000130713	EXOSC2
162.3011553	0.134599096	0.397820341	0.338341413	0.735105919	0.999467288	ENSG00000150401	DCUN1D2
157.9902815	-0.390282256	0.270001755	-1.445480441	0.148322983	0.936396974	ENSG00000157326	DHRS4
158.5118497	0.090428716	0.29952438	0.301907699	0.762722426	0.999467288	ENSG00000157349	DDX19B
159.3725691	-0.411441268	0.36486372	-1.127657383	0.259464642	0.962465655	ENSG00000146859	TMEM140
159.2075874	-0.743960254	0.296565319	-2.508588179	0.012121471	0.648356243	ENSG00000198324	PHETA1
158.4286167	0.861335889	0.327654407	2.628793846	0.008568829	0.604581883	ENSG00000177084	POLE
157.137372	-0.428186545	0.535161399	-0.800107306	0.423648629	0.991301167	ENSG00000143416	SELENBP1
157.8250674	-0.031800594	0.382766444	-0.083080935	0.933787185	0.999527751	ENSG00000130193	THEM6
159.2413574	-0.327669223	0.302140914	-1.084491402	0.278146977	0.962465655	ENSG00000168806	LCMT2
156.7039913	0.299001283	0.303957571	0.983694146	0.325265919	0.976026345	ENSG00000144445	KANSL1L
159.877358	-0.2332735	0.346809135	-0.6726279	0.501184047	0.993513219	ENSG00000013392	RWDD2A
156.2665201	0.578415069	0.351757919	1.644355499	0.100102792	0.92169898	ENSG00000214455	RCN1P2
158.9434187	0.250421352	0.31705741	0.78982968	0.429627243	0.991301167	ENSG00000185163	DDX51
158.1198953	0.163078214	0.284179667	0.573856024	0.566065234	0.99483168	ENSG00000102543	CDADC1
156.8702804	-0.291415526	0.296281737	-0.98357573	0.325324163	0.976026345	ENSG00000162419	GMEB1
158.6735758	0.201319743	0.284512779	0.70759473	0.479196967	0.99135833	ENSG00000174718	KIAA1551
157.2554951	-0.335641046	0.465375123	-0.721226877	0.470769938	0.99135833	ENSG00000074370	ATP2A3
158.1817022	0.499711343	0.346904811	1.440485479	0.149730096	0.936396974	ENSG00000070950	RAD18
157.7587086	-0.186905021	0.323559843	-0.577652095	0.563499028	0.99483168	ENSG00000178053	MLF1
160.9313118	0.041659031	1.051040183	0.039636002	0.968383325	0.999527751	ENSG00000142178	SIK1
156.9751773	-0.241070282	0.300933544	-0.801074814	0.423088336	0.991301167	ENSG00000185641	AC034236.1
157.5197769	0.441125056	0.326343388	1.351720527	0.176464736	0.946278819	ENSG00000130589	HELZ2
158.088744	-0.697191716	0.276273956	-2.52355208	0.011617585	0.648356243	ENSG00000173171	MTX1
158.5045442	0.034360662	0.293641377	0.117015736	0.906847584	0.999527751	ENSG00000189369	GSPT2
157.9778765	0.35488843	0.317123954	1.119084273	0.263104188	0.962465655	ENSG00000165288	BRWD3
157.2477777	-0.26104822	0.284818414	-0.916542638	0.359382355	0.979465649	ENSG00000163961	RNF168
157.9778837	0.142458818	0.276153983	0.515867332	0.605947072	0.99483168	ENSG00000063169	BICRA
159.1230093	-0.007068663	0.348992826	-0.020254465	0.98384038	0.999527751	ENSG00000073331	ALPK1
157.4464126	0.377775384	0.407799896	0.9263744	0.354251443	0.977691382	ENSG00000049541	RFC2
155.1663076	0.266819104	0.388518308	0.686760697	0.492233541	0.993513219	ENSG00000151702	FLI1
155.7745708	-0.382939529	0.351624919	-1.089056857	0.276128813	0.962465655	ENSG00000254858	MPV17L2
158.0106432	0.393145197	0.286258653	1.373391486	0.169630675	0.944422693	ENSG00000144589	STK11IP
155.3492154	-0.209883504	0.379099888	-0.553636416	0.579827698	0.99483168	ENSG00000164978	NUDT2
154.4451918	-0.623312879	0.353920787	-1.761164934	0.078210491	0.897052166	ENSG00000107872	FBXL15
156.892079	0.293358227	0.353417097	0.830062353	0.406503531	0.990226751	ENSG00000065600	TMEM206
157.7421881	-0.036349527	0.288670075	-0.125920662	0.899794727	0.999527751	ENSG00000096968	JAK2
157.5207346	-0.800577953	0.296967428	-2.695844318	0.007021047	0.549501061	ENSG00000232472	EEF1B2P3
155.2628156	-0.084837304	0.318349636	-0.266490973	0.78986111	0.999467288	ENSG00000178718	RPP25
157.3566227	0.245841866	0.3031325	0.811004648	0.417363001	0.990226751	ENSG00000103168	TAF1C
156.7873622	-0.198891525	0.295460743	-0.673157194	0.50084729	0.993513219	ENSG00000221838	AP4M1
155.8629013	0.727475334	0.319134343	2.279526945	0.02263576	0.744905651	ENSG00000102805	CLN5
157.4228835	0.431685045	0.398265284	1.083913316	0.278403235	0.962877855	ENSG00000198795	ZNF521
156.5511401	0.404428147	0.391659965	1.032600172	0.301791048	0.972793733	ENSG00000092094	OSGEP
153.9150553	0.55031665	0.518737154	1.060877644	0.28874551	0.966288987	ENSG00000157510	AFAP1L1
155.3980503	0.799990107	0.277801446	2.879719017	0.003980297	0.45638972	ENSG00000146067	FAM193B

156.4119144	0.072190521	0.383029778	0.188472346	0.850506392	0.999467288	ENSG00000082458	DLG3
156.4790463	0.037621458	0.348190779	0.108048403	0.913957297	0.999527751	ENSG00000214413	BBIP1
153.4106963	0.794267877	0.386247187	2.056371943	0.039746679	0.793985738	ENSG00000137404	NRM
157.2144818	-0.486969993	0.335801232	-1.450173335	0.147010189	0.936396974	ENSG00000006757	PNPLA4
157.081945	0.040537506	0.530522717	0.0764105	0.939092516	0.999527751	ENSG00000197046	SIGLEC15
157.028553	-0.491284089	0.30489941	-1.611298914	0.107114587	0.923922314	ENSG00000161179	YDJC
156.6368813	0.830922458	0.511103957	1.625740607	0.104004848	0.92169898	ENSG00000225177	FLJ46906
155.6991891	0.39780816	0.281804526	1.411645748	0.158054295	0.939464191	ENSG00000130699	TAF4
155.7989546	-0.211589005	0.320267649	-0.660663061	0.508828418	0.993513219	ENSG00000156239	N6AMT1
157.7739452	-0.244611578	0.395325997	-0.618759149	0.536075038	0.99483168	ENSG00000141338	ABCA8
153.889959	0.188454346	0.378597344	0.497769859	0.618646263	0.99483168	ENSG00000148444	COMMD3
155.2053221	-0.107861988	0.328412704	-0.328434272	0.742583333	0.999467288	ENSG00000127337	YEATS4
156.0121693	-0.243769356	0.293638515	-0.830168196	0.406443694	0.990226751	ENSG00000113638	TTC33
152.8329708	-1.276486809	0.657431329	-1.941627591	0.0521822	0.846840376	ENSG00000164379	FOXQ1
155.6237395	1.008465335	0.321995843	3.131920352	0.00173667	0.302201472	ENSG00000138834	MAPK8IP3
155.5410216	0.994852774	0.320341951	3.105596288	0.001898958	0.308164584	ENSG00000114933	INO80D
152.3589169	-0.157376835	0.399127725	-0.394301937	0.693358134	0.998869317	ENSG00000152240	HAUS1
158.1576428	-0.104791932	0.398818645	-0.262755849	0.792738767	0.999467288	ENSG00000126003	PLAGL2
154.1033875	-0.272044413	0.289590775	-0.939409802	0.347520382	0.977691382	ENSG00000176182	MYPOP
153.423572	0.009151157	0.393321497	0.023266353	0.981437811	0.999527751	ENSG00000142330	CAPN10
154.7903128	-0.034525149	0.289509853	-0.119253797	0.905074287	0.999527751	ENSG00000113761	ZNF346
151.4857922	-0.527931483	0.418330843	-1.26199512	0.206950542	0.954310328	ENSG00000229619	MBNL1-AS1
153.0555446	0.061723378	0.289106927	0.213496712	0.830939553	0.999467288	ENSG00000170634	ACYP2
153.9120086	0.4108895	0.657303487	0.62511383	0.531896352	0.99483168	ENSG00000136160	EDNRB
152.8525983	-0.284126204	0.35719853	-0.795429376	0.426363777	0.991301167	ENSG00000125449	ARMC7
152.3523874	-0.570280535	0.399798778	-1.426418906	0.15374746	0.939432741	ENSG00000105298	CACTIN
155.4162195	-0.028768194	0.33524591	-0.085812215	0.931615696	0.999527751	ENSG00000221923	ZNF880
153.8577183	-0.056235017	0.320657863	-0.17537389	0.860785855	0.999467288	ENSG00000115289	PCGF1
154.197765	0.309846646	0.365722966	0.847216812	0.396874285	0.988378925	ENSG00000104691	UBXN8
154.614061	0.314010939	0.310756414	1.010472914	0.31226877	0.973427483	ENSG00000148985	PGAP2
155.1147668	0.360668468	0.370084162	0.974557967	0.329779572	0.976026345	ENSG00000186814	ZSCAN30
154.8178524	0.224498872	0.346291029	0.648295374	0.516793926	0.993513219	ENSG00000044446	PHKA2
153.7370897	-0.039069835	0.30412152	-0.128467842	0.897778745	0.999467288	ENSG00000141179	PCTP
156.8319147	1.225794998	0.441923048	2.77377476	0.005541003	0.494004338	ENSG00000243716	NPIPB5
152.7098329	0.049166156	0.331383114	0.148366511	0.882053528	0.999467288	ENSG00000145414	NAF1
151.6648515	-0.515268425	0.460882047	-1.118004983	0.263564865	0.962465655	ENSG00000171443	ZNF524
154.7132978	-0.117061702	0.301303175	-0.388517984	0.697632748	0.998869317	ENSG00000213047	DENND1B
151.9603222	0.603280644	1.291702812	0.467042913	0.640469176	0.996553586	ENSG00000158258	CLSTN2
156.2570125	0.634122604	0.393282755	1.612383446	0.106878525	0.923786078	ENSG00000163945	UVSSA
151.9919917	-0.122142098	0.63206739	-0.193242209	0.846769284	0.999467288	ENSG00000148426	PROSER2
152.53041	0.280482717	0.277506624	1.010724404	0.312148353	0.973427483	ENSG00000100564	PIGH
153.4091143	0.22249309	0.35838394	0.620823271	0.53471591	0.99483168	ENSG00000120868	APAF1
160.8075933	-0.91874467	1.024702335	-0.896596639	0.369934192	0.981455968	ENSG00000095752	IL11
150.4336199	0.100322305	0.409188136	0.245174031	0.806321683	0.999467288	ENSG00000133466	C1QTNF6
153.3170132	0.146737636	0.374509971	0.391812362	0.69519686	0.998869317	ENSG00000177000	MTHFR
149.8742558	0.121053779	0.421851625	0.286958191	0.774144333	0.999467288	ENSG00000118473	SGIP1
155.7345175	2.011565881	1.038138566	1.937666076	0.052663975	0.847598083	ENSG00000142945	KIF2C
150.1735681	-1.364966661	0.414639139	-3.291938778	0.000994993	0.228234741	ENSG00000019582	CD74
151.7886907	-0.196184506	0.322162437	-0.608961452	0.54254999	0.99483168	ENSG00000134597	RBMX2
152.3180812	-0.115259693	0.332685787	-0.346452112	0.729002968	0.999467288	ENSG00000186106	ANKRD46
152.8865322	-0.23021567	0.296956009	-0.775251766	0.438190905	0.991301167	ENSG00000167186	COQ7
154.2306644	2.241311992	1.660893129	1.349461897	0.177188651	0.946540546	ENSG00000024526	DEPDC1
152.250543	0.523616394	0.288905188	1.812416029	0.069921938	0.878158738	ENSG00000155744	FAM126B
152.8804037	0.291512846	0.306662982	0.950596791	0.341809098	0.977691382	ENSG00000196312	MFSD14C
151.9085385	0.247646131	0.344669288	0.718503619	0.472446817	0.99135833	ENSG00000081721	DUSP12
152.0177489	0.131108618	0.275922928	0.475163911	0.634670148	0.995506752	ENSG00000118197	DDX59
151.0073195	-0.234809981	0.384969978	-0.609943618	0.541899157	0.99483168	ENSG00000175198	PCCA
150.0307256	-0.134348212	0.33657908	-0.399157939	0.689776833	0.998869317	ENSG00000126790	L3HYPDH
151.0511529	-0.117193684	0.385716996	-0.303833342	0.761254864	0.999467288	ENSG00000132357	CARD6
150.1285017	0.085805305	0.489226921	0.175389581	0.860773527	0.999467288	ENSG00000121361	KCNJ8
150.7260622	-0.138188444	0.288816642	-0.478464271	0.632319798	0.994860874	ENSG00000213977	TAX1BP3
153.342319	0.333167584	0.319110004	1.044052456	0.296461121	0.970706863	ENSG00000173040	EVC2
152.6357167	0.015781136	0.317899293	0.049641936	0.960407728	0.999527751	ENSG00000188917	TRMT2B
152.3641105	0.540891643	0.278192074	1.944310043	0.051858074	0.845356848	ENSG00000157741	UBN2

154.8130315	-0.306389753	0.421811612	-0.72636633	0.467614226	0.99135833	ENSG00000143891	GALM
150.0873259	-0.067265333	0.335731702	-0.200354427	0.841203399	0.999467288	ENSG00000047579	DTNBP1
154.293129	1.596693239	0.450102823	3.547396632	0.000389058	0.173156217	ENSG00000229807	XIST
152.308658	0.372193283	0.2820837	1.319442714	0.18702115	0.949094286	ENSG00000143033	MTF2
149.7070459	-0.146128014	0.524097166	-0.278818555	0.780384074	0.999467288	ENSG00000131370	SH3BP5
153.5127558	-0.283411975	0.369531326	-0.766949795	0.443111366	0.99135833	ENSG00000253846	PCDHGA10
153.1812524	-0.143627633	0.344320002	-0.417134154	0.676580285	0.998869317	ENSG00000140650	PMM2
148.982013	-0.285776727	0.331548892	-0.861944449	0.388718088	0.986803154	ENSG00000132109	TRIM21
150.9543331	0.117635126	0.309358688	0.380254801	0.703756284	0.99900742	ENSG00000197283	SYNGAP1
150.922467	-0.058177808	0.405190715	-0.143581295	0.885831112	0.999467288	ENSG00000123427	EEF1AKMT3
149.2340863	-0.691337343	0.289458897	-2.388378282	0.016922913	0.714671438	ENSG00000122971	ACADS
146.5875181	-0.221570965	0.4995524	-0.443538985	0.657375927	0.997568624	ENSG00000143126	CELSR2
152.3966886	1.975941168	1.132524442	1.744722758	0.08103314	0.897807954	ENSG00000068489	PRR11
149.6467618	-0.064143653	0.337794665	-0.189889478	0.849395738	0.999467288	ENSG00000161048	NAPEPLD
149.9029716	-0.387871918	0.295735002	-1.311552286	0.189671241	0.949094286	ENSG00000101888	NXT2
149.9011931	0.034730011	0.272523109	0.127438774	0.898593129	0.999490224	ENSG00000070501	POLB
151.5282023	0.933134532	0.584204678	1.597273297	0.1102048	0.923922314	ENSG00000094804	CDC6
148.2386528	0.127488261	0.349141112	0.365148235	0.715000757	0.99900742	ENSG00000179456	ZBTB18
146.9679884	0.410475716	0.437772419	0.93764636	0.348426178	0.977691382	ENSG00000204219	TCEA3
150.4391176	-0.0654328	0.27101946	-0.241432109	0.80922023	0.999467288	ENSG00000145439	CBR4
151.6365587	0.13386372	0.366618919	0.365130421	0.715014053	0.99900742	ENSG00000197928	ZNF677
151.1781369	-0.168869233	0.325503995	-0.51879312	0.603905016	0.99483168	ENSG00000169016	E2F6
148.2104756	-0.391942923	0.429658161	-0.912220361	0.361652727	0.979465649	ENSG00000253982	AC100810.1
149.5319108	0.319607351	0.325265717	0.982603867	0.32580244	0.976026345	ENSG00000196781	TLE1
151.572371	-0.103034667	0.327751774	-0.314367994	0.753241562	0.999467288	ENSG00000117155	SSX2IP
150.3265145	-0.182869853	0.311187137	-0.587652353	0.556765663	0.99483168	ENSG00000128059	PPAT
150.0604409	-0.087335872	0.274246396	-0.318457684	0.750137788	0.999467288	ENSG00000197062	ZSCAN26
151.2793063	0.297691257	0.325930803	0.913357235	0.36105469	0.979465649	ENSG00000204576	PRR3
147.3678022	0.146684345	0.424892552	0.345226915	0.729923786	0.999467288	ENSG00000163644	PPM1K
149.9380648	0.186559615	0.287152505	0.649688271	0.515893601	0.993513219	ENSG00000277203	F8A1
149.2128378	0.020189796	0.2788694	0.072398749	0.94228458	0.999527751	ENSG00000112874	NUDT12
147.1955488	-0.803926621	0.483274555	-1.663498756	0.09621264	0.921147998	ENSG00000184481	FOXO4
148.4406803	-0.02263655	0.315321713	-0.071788744	0.942770031	0.999527751	ENSG00000105559	PLEKHA4
151.8343135	0.331968224	0.350712237	0.946554438	0.34386587	0.977691382	ENSG00000143190	POU2F1
150.1610819	-0.147150162	0.28781472	-0.511266978	0.609164125	0.99483168	ENSG00000171453	POLR1C
147.6361219	0.36379466	0.447255626	0.813393144	0.415992689	0.990226751	ENSG00000010295	IFFO1
149.4175032	-0.443062242	0.457597948	-0.968234766	0.332927136	0.976026345	ENSG00000166387	PPFIBP2
149.4231737	-0.453044585	0.355491334	-1.274418086	0.202515286	0.954310328	ENSG00000103150	MLYCD
150.949758	0.64634977	0.327549071	1.973291416	0.04846237	0.833264304	ENSG00000198799	LRIG2
149.7620768	0.122602014	0.665626176	0.184190494	0.853864017	0.999467288	ENSG00000188112	C6orf132
147.614224	-0.549889881	0.384151881	-1.43143873	0.152304515	0.938052926	ENSG00000242612	DECR2
150.378214	-0.261590112	0.592141851	-0.441769336	0.65865613	0.997568624	ENSG00000141068	KSR1
151.1221118	0.080062441	0.371844503	0.215311618	0.829524373	0.999467288	ENSG00000181007	ZFP82
148.3516885	0.024350401	0.311946506	0.078059539	0.937780692	0.999527751	ENSG00000198464	ZNF480
151.8825297	0.229050649	0.340966652	0.671768479	0.501731099	0.993513219	ENSG00000132950	ZMYM5
147.187985	-0.372024181	0.337655898	-1.101784933	0.270555182	0.962465655	ENSG00000171425	ZNF581
147.3391585	0.120865149	0.332716336	0.363267853	0.716404805	0.99900742	ENSG00000132906	CASP9
149.9164606	0.264548143	0.373828892	0.707671743	0.479149129	0.99135833	ENSG00000157036	EXOG
146.618496	-0.209527651	0.377719512	-0.554717574	0.579087854	0.99483168	ENSG00000240356	RPL23AP7
147.6391047	0.328857939	0.717566032	0.458296413	0.6467395	0.997368416	ENSG00000182379	NXPH4
144.9098815	0.108373322	0.508732472	0.213026154	0.831306563	0.999467288	ENSG00000104888	SLC17A7
150.571388	0.003497203	0.340698564	0.010264801	0.991810018	0.999527751	ENSG00000158158	CNNM4
148.7216312	0.934761319	0.409858103	2.280694983	0.022566502	0.744905651	ENSG00000172349	IL16
148.9558125	-0.135100074	0.373753089	-0.361468782	0.717749038	0.99900742	ENSG00000151576	QTRT2
148.890793	-0.203577652	0.307707222	-0.661595299	0.508230621	0.993513219	ENSG00000155858	LSM11
149.0384449	-0.216003665	0.289318527	-0.746594654	0.455308277	0.99135833	ENSG00000111196	MAGOHB
147.5872953	0.26464893	0.315929563	0.837683335	0.402208575	0.988592117	ENSG00000187630	DHRS4L2
146.2984676	0.295714253	0.346269956	0.853999164	0.393105453	0.988378925	ENSG00000100592	DAAM1
148.5661173	-0.301816167	0.330867991	-0.912195122	0.361666011	0.979465649	ENSG00000049167	ERCC8
145.4093372	0.053572527	0.369998081	0.144791364	0.884875601	0.999467288	ENSG00000100916	BRMS1L
147.9701127	0.372730214	0.296601709	1.256669137	0.208873468	0.954310328	ENSG00000068885	IFT80
145.6765701	-0.300654389	0.415731136	-0.723194303	0.469560518	0.99135833	ENSG00000198933	TBKBP1
149.5206073	-0.44527693	0.300461307	-1.481977609	0.138346253	0.934144427	ENSG00000266074	BAHCC1
146.49267	0.227227498	0.372988496	0.609207791	0.542386717	0.99483168	ENSG00000064012	CASP8

148.2675468	0.125113985	0.280079451	0.446708907	0.65508525	0.997568624	ENSG00000154240	CEP112	
148.1783273	-0.021056	0.354609695	-0.059377958	0.952651069	0.999527751	ENSG00000125457	MIF4GD	
146.3846789	-0.27692172	0.374756061	-0.738938602	0.459944281	0.99135833	ENSG00000245060	LINC00847	
147.4674044	0.255602551	0.281109847	0.909262175	0.363211752	0.98048242	ENSG00000177694	NAALADL2	
148.5042743	0.306153602	0.279955668	1.093578866	0.274139723	0.962465655	ENSG00000178966	RMI1	
147.4233882	-0.230222581	0.424927374	-0.541792775	0.587961269	0.99483168	ENSG00000141337	ARSG	
149.1824002	-0.038159988	0.378304317	-0.10087114	0.919652753	0.999527751	ENSG00000198046	ZNF667	
147.0446718	-0.471249416	0.299572878	-1.573071029	0.115702372	0.923922314	ENSG00000053501	USE1	
147.9329645	-0.179682955	0.320732236	-0.560227302	0.575324407	0.99483168	ENSG00000170222	ADPRM	
145.5632138	0.316781977	0.408982838	0.774560562	0.438599369	0.991301167	ENSG00000130813	C19orf66	
146.7529141	0.114653193	0.287163312	0.399261285	0.68970069	0.998869317	ENSG00000167920	TMEM99	
146.5763174	-0.07378288	0.281225121	-0.262362336	0.793042107	0.999467288	ENSG00000168275	COA6	
148.6561983	1.40410064	0.700937799	2.003174377	0.045158575	0.813244956	ENSG00000138182	KIF20B	
146.6337705	0.229389619	0.458111169	0.500729156	0.61656175	0.99483168	ENSG00000276045	ORAI1	
146.1065645	0.450801563	0.376248989	1.198146908	0.23085983	0.959575179	ENSG00000162600	OMA1	
148.003066	0.053034166	0.364751279	0.145398164	0.884396515	0.999467288	ENSG00000253304	TMEM200B	
148.3266632	0.230189583	0.337311468	0.682424421	0.494970618	0.993513219	ENSG00000163412	EIF4E3	
149.2350897	0.076362068	0.450716135	0.169423862	0.865463255	0.999467288	ENSG00000111271	ACAD10	
147.1361782	-0.181797508	0.354004735	-0.513545412	0.607569853	0.99483168	ENSG00000166263	STXBP4	
147.7243072	-0.053494009	0.301180482	-0.177614461	0.859025767	0.999467288	ENSG00000162972	MAIP1	
144.2466586	-0.006801805	0.396730219	-0.017144661	0.986321209	0.999527751	ENSG00000177854	TMEM187	
146.6766345	-0.406895944	0.318627456	-1.277027251	0.201592626	0.954310328	ENSG00000141026	MED9	
144.7301999	-0.221405927	0.312272932	-0.709014149	0.478315697	0.99135833	ENSG00000178896	EXOSC4	
146.1749774	-0.496578705	0.428657635	-1.158450624	0.24668019	0.962465655	ENSG00000112425	EPM2A	
144.918822	-0.737313151	0.395237151	-1.865495561	0.062111974	0.865910462	ENSG00000262814	MRPL12	
147.1712701	-0.129627803	0.349591467	-0.370797961	0.71078802	0.99900742	ENSG00000173436	MINOS1	
148.3671109	-0.286694354	0.38572132	-0.743268104	0.457319379	0.99135833	ENSG00000112305	SMAP1	
146.0780606	-0.046035925	0.40824088	-0.112766573	0.91021562	0.999527751	ENSG00000197070	ARRDC1	
147.132582	0.416316944	0.524657377	0.793502508	0.427485109	0.991301167	ENSG00000250251	PKD1P6	
146.489046	-0.103784409	0.285264707	-0.363817907	0.71599399	0.99900742	ENSG00000215041	NEURL4	
146.3864308	-0.307445708	0.302425183	-1.016600883	0.309343334	0.973427483	ENSG00000179943	FIZ1	
146.548586	-0.009381987	0.271783506	-0.034520074	0.972462435	0.999527751	ENSG00000125482	TTF1	
146.45017	-0.06487143	0.306491753	-0.211657995	0.832373861	0.999467288	ENSG00000188785	ZNF548	
143.8727669	-0.255575563	0.36027708	-0.709386128	0.478084894	0.99135833	ENSG00000160908	ZNF394	
147.3367429	0.592376059	0.306493856	1.93275019	0.053266979	0.847598083	ENSG00000177853	ZNF518A	
145.8328778	0.260593444	0.53756833	0.484763387	0.627844197	0.99483168	ENSG00000100058	CRYBB2P1	
146.662229	-0.291920931	0.295343185	-0.988412616	0.322950611	0.975337095	ENSG00000118600	RXYLT1	
142.1677312	0.212930682	0.51548443	0.413069085	0.679555998	0.998869317	ENSG00000134508	CABLES1	
144.4564799	-0.004426137	0.283824556	-0.015594625	0.987557794	0.999527751	ENSG00000100077	GRK3	
147.4745952	-1.413835793	0.360966914	-3.916801622	8.97E-05	0.067295389	ENSG00000187699	C2orf88	
143.1197744	-0.148484869	0.439979301	-0.337481486	0.735753966	0.999467288	ENSG00000125354		6-Sep
147.3907536	-0.241027033	0.32595537	-0.73944796	0.459635029	0.99135833	ENSG00000170468	RIOX1	
147.4175004	0.60157747	0.45259113	1.329185284	0.183786849	0.949094286	ENSG00000173166	RAPH1	
146.2816236	0.036919938	0.400073505	0.092282887	0.926473285	0.999527751	ENSG00000186416	NKRF	
145.3385941	0.591840654	0.290936966	2.034257323	0.041925658	0.804159726	ENSG00000119720	NRDE2	
145.3412314	-0.30566282	0.370965251	-0.823966178	0.409958776	0.990226751	ENSG00000197951	ZNF71	
145.101983	-0.175090152	0.313975943	-0.557654673	0.577080215	0.99483168	ENSG00000143633	C1orf131	
144.6445629	0.409871213	0.326155051	1.256675963	0.208870995	0.954310328	ENSG00000124508	BTN2A2	
144.9829636	0.467859474	0.412936894	1.133004779	0.257212246	0.962465655	ENSG00000146263	MMS22L	
145.7635309	0.779704089	0.318450832	2.44842849	0.014348091	0.684434275	ENSG00000136158	SPRY2	
146.1382054	-0.147914987	0.364848077	-0.405415285	0.685172278	0.998869317	ENSG00000257365	FNTB	
142.833803	0.188249165	0.384558522	0.489520201	0.624473457	0.99483168	ENSG00000129355	CDKN2D	
145.7473315	-0.039287796	0.38379944	-0.102365432	0.918466621	0.999527751	ENSG00000116525	TRIM62	
143.7075539	-0.183713033	0.344900127	-0.532655742	0.594271908	0.99483168	ENSG00000163050	COQ8A	
146.0353149	0.109779779	0.290771648	0.377546365	0.705767615	0.99900742	ENSG00000005436	GCFC2	
145.4592898	0.042526126	0.316982561	0.134159198	0.89327669	0.999467288	ENSG00000184402	SS18L1	
144.7555377	0.308175304	0.304757847	1.01121368	0.31191417	0.973427483	ENSG00000170448	NFXL1	
144.7072412	-0.215631735	0.360844276	-0.597575602	0.550123148	0.99483168	ENSG00000148950	IMMP1L	
144.1704309	-0.384192127	0.311437231	-1.233610143	0.217348212	0.956186483	ENSG00000041988	THAP3	
143.4661371	-0.015227597	0.380579538	-0.040011602	0.968083877	0.999527751	ENSG00000168306	ACOX2	
144.7903673	-0.08315993	0.30406133	-0.273497226	0.784471024	0.999467288	ENSG00000135521	LTV1	
143.4881436	-0.236748283	0.31938422	-0.74126481	0.458532894	0.99135833	ENSG00000126970	ZC4H2	
143.9253793	-0.285488489	0.368315672	-0.775119036	0.438269324	0.991301167	ENSG00000170364	SETMAR	
144.2656648	-0.558963909	0.345273363	-1.618902497	0.105468244	0.92169898	ENSG00000164197	RNF180	

146.8521835	-0.326112106	0.414462323	-0.786831729	0.431380377	0.991301167	ENSG00000185960	SHOX
142.7385869	-0.125183447	0.457843768	-0.273419571	0.78453071	0.999467288	ENSG00000077157	PPP1R12B
144.9787734	-0.139572282	0.319251609	-0.437185836	0.661976581	0.997568624	ENSG00000137760	ALKBH8
143.8750538	-0.300588473	0.344249815	-0.873169599	0.382570643	0.98555814	ENSG00000169169	CPT1C
143.6751433	2.110709169	1.234034176	1.710413869	0.087189368	0.90492251	ENSG00000109805	NCAPG
142.329849	0.997571928	1.052720825	0.947612989	0.343326511	0.977691382	ENSG00000137393	RNF144B
141.7960623	0.156390044	0.507094178	0.308404338	0.757774681	0.999467288	ENSG00000139117	CPNE8
144.6317174	0.378418564	0.334393275	1.131657222	0.257778573	0.962465655	ENSG00000168395	ING5
147.423801	0.406657283	0.438422004	0.927547611	0.353642284	0.977691382	ENSG00000102468	HTR2A
143.6998701	0.156788095	0.319893045	0.490126614	0.624044307	0.99483168	ENSG00000044459	CNTLN
143.3007877	0.121364313	0.333631683	0.363767348	0.716031747	0.99900742	ENSG00000204611	ZNF616
144.6607165	-0.257557246	0.326295074	-0.789338445	0.429914223	0.991301167	ENSG00000107371	EXOSC3
150.9401925	-0.796896986	0.619139027	-1.287105079	0.198057665	0.954310328	ENSG00000258545	RHOXF1-AS1
143.6003767	-0.197283301	0.310822652	-0.634713398	0.525615354	0.99483168	ENSG00000197763	TXNRD3
142.1732394	-0.075690123	0.396756865	-0.190772056	0.848704182	0.999467288	ENSG00000188921	HACD4
143.4723258	0.808503714	0.375469738	2.153312589	0.03129412	0.775963115	ENSG00000128617	OPN1SW
142.3180728	-0.152626086	0.430175078	-0.354799926	0.722739484	0.999104299	ENSG00000196368	NUDT11
141.0950623	-0.977200951	0.577396136	-1.692427245	0.090564541	0.912150398	ENSG00000077092	RARB
143.7496317	0.173688786	0.361992409	0.479813337	0.631360129	0.99483168	ENSG00000013619	MAMLD1
145.6474875	0.331572599	0.423904729	0.782186601	0.434104918	0.991301167	ENSG00000186417	GLDN
151.7673528	-0.427771718	0.73481129	-0.582151803	0.560464434	0.99483168	ENSG00000113070	HBEGF
142.5616284	0.617480669	0.353278321	1.747858933	0.080488457	0.897807954	ENSG00000102780	DGKH
143.3639449	0.180818153	0.299126187	0.604487874	0.545519329	0.99483168	ENSG00000135763	URB2
143.5740908	0.562781237	0.301636164	1.865761814	0.062074697	0.865910462	ENSG00000174652	ZNF266
143.5784498	-0.352125666	0.438858812	-0.80236663	0.42234091	0.991301167	ENSG00000079257	LXN
143.1002109	0.31635746	0.367284656	0.861341346	0.38905007	0.986937571	ENSG00000179630	LACC1
141.824869	0.470863075	0.429789536	1.095566632	0.273268471	0.962465655	ENSG00000163918	RFC4
140.1947955	0.061978072	0.450892466	0.137456437	0.890670024	0.999467288	ENSG00000119917	IFIT3
143.0794581	-0.36219636	0.2994745	-1.209439736	0.226493951	0.958865115	ENSG00000184517	ZFP1
142.9477026	-0.246754184	0.290589982	-0.849148968	0.395798405	0.988378925	ENSG00000132661	NXT1
141.2994287	-0.065199178	0.294254723	-0.22157394	0.824645571	0.999467288	ENSG00000170954	ZNF415
140.5425884	0.278674809	0.377109864	0.738975125	0.459922102	0.99135833	ENSG00000170545	SMAGP
141.8439898	-0.037460016	0.326885858	-0.114596623	0.908764856	0.999527751	ENSG00000174977	AC026271.1
144.0775483	0.190820322	0.352782899	0.540900147	0.588576409	0.99483168	ENSG00000108666	C17orf75
141.2586174	-0.09468057	0.373392747	-0.253568316	0.799829077	0.999467288	ENSG00000184208	C22orf46
140.4970851	0.027696175	0.359317025	0.077080053	0.938559861	0.999527751	ENSG00000137941	TTLL7
143.1864921	-0.110599676	0.328682262	-0.336494204	0.736498222	0.999467288	ENSG00000144034	TPRKB
141.2432068	-0.390434292	0.402405989	-0.970249705	0.331922041	0.976026345	ENSG00000215039	CD27-AS1
149.1794902	-0.694674068	1.147261174	-0.605506474	0.544842524	0.99483168	ENSG00000158859	ADAMTS4
141.2398359	-0.203220647	0.523910052	-0.387892246	0.698095776	0.998869317	ENSG00000169122	FAM110B
141.5761492	0.279732747	0.548972151	0.509557264	0.61036167	0.99483168	ENSG00000078725	BRINP1
140.4534932	0.070958883	0.344686127	0.205865213	0.8368962	0.999467288	ENSG00000110074	FOXRED1
142.8260019	0.048358258	0.29754467	0.162524363	0.870892944	0.999467288	ENSG00000128692	EIF2S2P4
140.9317077	-0.409847591	0.353974019	-1.157846536	0.246926669	0.962465655	ENSG00000160799	CCDC12
142.509665	0.207065665	0.287129219	0.721158458	0.470812028	0.99135833	ENSG00000147124	ZNF41
140.8745147	0.221260218	0.287083086	0.770718404	0.440873861	0.99135833	ENSG00000215421	ZNF407
142.19921	0.577335266	0.331132651	1.743516577	0.081243421	0.897807954	ENSG00000104290	FZD3
141.3199912	-0.176642949	0.295066372	-0.598654967	0.549402993	0.99483168	ENSG00000116691	MIIP
140.9555731	2.486667611	1.648786968	1.50818005	0.131508452	0.931066946	ENSG00000168078	PBK
145.4732989	1.1210111	0.653647882	1.715007623	0.086343829	0.901307093	ENSG00000162772	ATF3
141.8483155	0.224573689	0.288960663	0.777177372	0.437054131	0.991301167	ENSG00000007545	CRAMP1
141.8121715	0.130447056	0.306556609	0.425523548	0.670455059	0.997568624	ENSG00000124217	MOCS3
140.3100056	0.258895948	0.348003725	0.743945909	0.4569092	0.99135833	ENSG00000198169	ZNF251
141.6983344	0.117545829	0.306917175	0.382988761	0.701728097	0.998869317	ENSG00000164118	CEP44
140.2081899	-0.143120511	0.360868404	-0.396600282	0.691662244	0.998869317	ENSG00000164949	GEM
141.14201	-0.456795644	0.350137791	-1.304616798	0.192023365	0.949261383	ENSG00000123191	ATP7B
139.9898306	1.716207692	1.147856711	1.495141053	0.134877634	0.933741583	ENSG00000071539	TRIP13
140.1755486	-0.013145668	0.314438629	-0.041806786	0.966652726	0.999527751	ENSG00000129696	TTI2
139.7423368	-0.142159311	0.3638981	-0.390656919	0.696050847	0.998869317	ENSG00000174446	SNAPC5
140.8673683	0.484445454	0.331355988	1.462009051	0.14373873	0.935644597	ENSG00000081870	HSPB11
139.4002103	-0.518552677	0.385319519	-1.345773186	0.178375675	0.9469453	ENSG00000185630	PBX1
140.1201999	0.050057326	0.370659192	0.135049466	0.892572764	0.999467288	ENSG00000138617	PARP16
141.2271298	0.393939648	0.308733151	1.275987524	0.201959929	0.954310328	ENSG00000096093	EFHC1
142.5439516	0.295964074	0.530649108	0.557739699	0.577022145	0.99483168	ENSG00000181195	PENK

140.7091102	-0.149845373	0.38950758	-0.384704638	0.700456259	0.998869317	ENSG00000129158	SERGEF
141.869911	-0.297658583	0.335095364	-0.888280216	0.374390039	0.982609538	ENSG00000133316	WDR74
146.7456574	-0.127979873	0.709256904	-0.180442196	0.85680543	0.999467288	ENSG00000116183	PAPPA2
138.9718526	0.635373892	0.368847505	1.722592352	0.084962269	0.901307093	ENSG00000146386	ABRACL
140.1282064	-0.080753694	0.329588555	-0.245013646	0.806445865	0.999467288	ENSG00000073536	NLE1
137.6446575	0.027225995	0.402232974	0.067687129	0.946034695	0.999527751	ENSG00000105755	ETHE1
140.9534337	0.358731892	0.331255435	1.082946432	0.278832201	0.963231086	ENSG00000046651	OFD1
140.1481235	-0.267154786	0.29540706	-0.904361546	0.365803722	0.981269214	ENSG00000166352	C11orf74
139.5788482	-0.308683341	0.322112464	-0.958309209	0.337906861	0.977691382	ENSG00000266469	AC005288.1
140.7472834	0.186741324	0.326491073	0.571964563	0.567345983	0.99483168	ENSG00000163607	GTPBP8
139.4777403	-0.515872341	0.348211086	-1.481493157	0.138475207	0.934578231	ENSG00000037965	HOXC8
140.2613118	0.690982592	0.321351406	2.150239831	0.031536249	0.775963115	ENSG00000177192	PUS1
138.1559045	1.231799828	0.360675017	3.415262413	0.000637206	0.195790358	ENSG00000242125	SNHG3
138.3057965	0.875698206	0.422069823	2.074770944	0.038007769	0.790190505	ENSG00000115841	RMDN2
140.0946457	0.426164471	0.45575291	0.935077893	0.349748158	0.977691382	ENSG00000253953	PCDHGB4
139.3124478	-0.203275555	0.319518382	-0.636193617	0.524650233	0.99483168	ENSG00000173451	THAP2
140.1284004	-0.232569004	0.377552508	-0.61599115	0.537900361	0.99483168	ENSG00000181315	ZNF322
142.7030039	2.057101544	1.160516797	1.772573692	0.076299374	0.897052166	ENSG00000076382	SPAG5
139.9196127	-0.115407914	0.535295776	-0.215596533	0.829302258	0.999467288	ENSG00000159263	SIM2
139.5297923	0.114199042	0.325961707	0.350344964	0.726079825	0.999467288	ENSG00000170085	SIMC1
137.9646348	0.579342	0.333990785	1.734604746	0.082810838	0.899952548	ENSG00000091073	DTX2
138.3135025	1.822505593	0.639157479	2.851418709	0.004352461	0.462224997	ENSG00000185697	MYBL1
137.7438753	-0.369254702	0.327047129	-1.129056546	0.258873981	0.962465655	ENSG00000215271	HOMEZ
140.07774	0.112759923	0.375956967	0.299927738	0.764232276	0.999467288	ENSG00000169239	CA5B
138.4389444	0.486558316	0.419354189	1.160256242	0.245944495	0.962465655	ENSG00000198554	WDHD1
140.0535556	0.171852478	0.277820023	0.618574847	0.536196476	0.99483168	ENSG00000103248	MTHFSD
139.9832742	-0.537428257	0.336648241	-1.596408926	0.110397525	0.923922314	ENSG00000143479	DYRK3
137.1611169	-0.336329373	0.504555298	-0.666585753	0.505036772	0.993513219	ENSG00000158560	DYNC1I1
138.5922771	-0.141576296	0.32091001	-0.441171331	0.659088966	0.997568624	ENSG00000127554	GFER
140.1842849	-0.185707829	0.382633118	-0.485341754	0.627433942	0.99483168	ENSG00000196814	MVB12B
139.681156	-0.124803144	0.285420249	-0.437261003	0.661922073	0.997568624	ENSG00000111224	PARP11
139.6998424	0.032783056	0.281962513	0.116267428	0.907440599	0.999527751	ENSG00000088451	TGDS
140.2612152	0.427832662	0.349975779	1.222463632	0.221532349	0.956884001	ENSG00000257093	KIAA1147
138.7929907	-0.015451471	0.322051488	-0.047978263	0.961733566	0.999527751	ENSG00000078399	HOXA9
139.3923491	0.093089815	0.284664586	0.327015791	0.743655942	0.999467288	ENSG00000071243	ING3
138.893335	0.007023209	0.311125758	0.022573539	0.981990452	0.999527751	ENSG00000090776	EFNB1
136.1661789	-0.520851914	0.411363525	-1.266159691	0.205455916	0.954310328	ENSG00000242732	RTL5
139.8163984	0.316084187	0.357351235	0.884519642	0.376415764	0.982609538	ENSG00000130787	HIP1R
138.0515938	0.158288731	0.287019711	0.551490805	0.581297268	0.99483168	ENSG00000009780	FAM76A
139.8170565	0.319991478	0.445692437	0.717964793	0.472778995	0.99135833	ENSG00000166451	CENPN
132.0814056	-1.884074365	1.363035311	-1.382263797	0.166890718	0.943223078	ENSG00000163017	ACTG2
139.1040678	-0.280367943	0.377096858	-0.743490531	0.457184752	0.99135833	ENSG00000167394	ZNF668
141.1257538	0.823426018	0.517060415	1.592514132	0.11126923	0.923922314	ENSG00000164171	ITGA2
138.2301996	-0.236851309	0.403199033	-0.587430249	0.556914783	0.99483168	ENSG00000133315	MACROD1
140.4820079	0.611941704	0.366336839	1.670434525	0.094833424	0.921147998	ENSG00000164180	TMEM161B
139.1291046	-0.002297587	0.321219711	-0.007152695	0.994293024	0.999527751	ENSG00000151304	SRFBP1
137.1744541	0.47656784	0.371357662	1.283312257	0.1993827	0.954310328	ENSG00000042088	TDP1
138.0353689	0.227723812	0.33038847	0.689260773	0.490659179	0.993513219	ENSG00000104714	ERICH1
138.7530436	0.316612305	0.368125343	0.860066579	0.389752343	0.987216662	ENSG00000153551	CMTM7
135.7251709	0.052228717	0.462808767	0.112851616	0.910148196	0.999527751	ENSG00000136002	ARHGEF4
136.7085706	0.039901611	0.400625267	0.099598338	0.920663214	0.999527751	ENSG00000123609	NMI
139.2823592	-0.169063907	0.37108247	-0.455596587	0.648680098	0.997368416	ENSG00000174028	FAM3C2
138.4849759	-0.412705468	0.29359814	-1.405681481	0.159818728	0.940612019	ENSG00000236698	EIF1AXP1
140.2146584	-0.285914282	0.629242275	-0.454378691	0.649556287	0.997381078	ENSG00000187479	C11orf96
136.7316043	-0.084794413	0.364105474	-0.232884203	0.815851325	0.999467288	ENSG00000164070	HSPA4L
135.041948	-0.222421952	0.488205447	-0.455590885	0.648684199	0.997368416	ENSG00000124107	SLPI
137.2268523	0.575242061	0.384977126	1.494224	0.135117082	0.933741583	ENSG00000113240	CLK4
137.2358405	-0.500640401	0.300202112	-1.667677814	0.095379702	0.921147998	ENSG00000089639	GMIP
136.9024842	0.243168754	0.305386971	0.796264338	0.425878409	0.991301167	ENSG00000153140	CETN3
138.3154493	-0.299443213	0.444522513	-0.673628904	0.500547272	0.993513219	ENSG00000216285	AC078819.1
137.300217	0.521759034	0.296709055	1.758487062	0.078664671	0.897052166	ENSG00000138162	TACC2
137.0006763	-0.478285898	0.349093398	-1.370080045	0.170661916	0.944422693	ENSG00000169972	PUSL1
133.6636659	-0.203763693	0.479740908	-0.424736956	0.67102844	0.997568624	ENSG00000068079	IFI35
136.7680716	-0.264085774	0.347909973	-0.759063536	0.447814551	0.99135833	ENSG00000160392	C19orf47

137.1766222	-0.113398335	0.349016709	-0.32490804	0.745250672	0.999467288	ENSG00000198513	ATL1
138.5786199	-0.326101731	0.341121411	-0.955969694	0.339087546	0.977691382	ENSG00000142556	ZNF614
143.4248834	-1.068210629	0.646157425	-1.65317396	0.098295465	0.921147998	ENSG00000154118	JPH3
136.6294512	-1.028400513	0.400772124	-2.566048016	0.010286459	0.632201379	ENSG00000158008	EXTL1
138.814441	-0.067479834	0.374137526	-0.180361041	0.856869138	0.999467288	ENSG00000078177	N4BP2
136.4267176	-0.286008987	0.302823031	-0.944475673	0.344926627	0.977691382	ENSG00000112357	PEX7
137.8616697	-0.027216294	0.29040379	-0.093718799	0.925332537	0.999527751	ENSG00000106459	NRF1
135.2494575	0.124002512	0.598916907	0.2070446	0.835975027	0.999467288	ENSG00000128340	RAC2
136.9250065	-0.171411183	0.353038999	-0.485530447	0.62730012	0.99483168	ENSG00000204439	C6orf47
135.3450415	-0.059628203	0.651652116	-0.091503122	0.927092825	0.999527751	ENSG00000008517	IL32
137.7184393	-0.63067229	0.405438236	-1.555532344	0.119819338	0.923922314	ENSG00000149531	FRG1BP
136.0679563	-0.330546754	0.304321206	-1.086177196	0.277400604	0.962465655	ENSG00000104524	PYCR3
135.0971333	0.605197077	0.369544104	1.637685652	0.101487285	0.92169898	ENSG00000172123	SLFN12
135.4869422	-0.0661424	0.397220212	-0.166513178	0.867753113	0.999467288	ENSG00000278970	HEIH
136.1149071	-0.173487283	0.300744248	-0.576859855	0.564034131	0.99483168	ENSG00000133619	KRBA1
137.614348	0.6492883	0.330214936	1.966259636	0.049268627	0.836346249	ENSG00000132199	ENOSF1
136.0670074	-0.133776259	0.290538845	-0.460441903	0.645199063	0.997368416	ENSG00000240682	ISY1
134.1753602	0.497938295	0.557805933	0.892673	0.372032314	0.981955904	ENSG00000149636	DSN1
135.9065845	0.302268706	0.39009197	0.774865234	0.438419297	0.991301167	ENSG00000176273	SLC35G1
135.5114503	-0.212099722	0.357110517	-0.593933004	0.552556923	0.99483168	ENSG00000180806	HOXC9
137.5266563	0.181605975	0.331983356	0.547033374	0.584355804	0.99483168	ENSG00000152443	ZNF776
136.2049673	0.120673022	0.299471036	0.4029539	0.686982138	0.998869317	ENSG00000173681	BCLAF3
134.9040448	-0.282949384	0.310620562	-0.910916462	0.362339391	0.980012702	ENSG00000167136	ENDOG
136.0048874	-0.326694287	0.300763998	-1.086214737	0.277383999	0.962465655	ENSG00000169372	CRADD
139.0372091	0.332832902	0.443108048	0.751132604	0.452572856	0.99135833	ENSG00000196387	ZNF140
136.3339917	-0.198263817	0.390380285	-0.507873538	0.611542033	0.99483168	ENSG00000177427	MIEF2
135.7884775	-0.062466681	0.334846912	-0.186552954	0.852011156	0.999467288	ENSG00000148814	LRRC27
136.2249265	-0.571758864	0.290001429	-1.971572574	0.048658421	0.83480477	ENSG00000102409	BEX4
136.0417807	0.113882411	0.346821837	0.328359978	0.7426395	0.999467288	ENSG00000007341	ST7L
136.8055868	-0.081611993	0.34593165	-0.235919418	0.8134952	0.999467288	ENSG00000198453	ZNF568
134.852249	0.286939766	0.316183374	0.907510608	0.364136843	0.98048242	ENSG00000145220	LYAR
135.1046567	-0.791932756	0.344200058	-2.30079205	0.021403388	0.73997555	ENSG00000204642	HLA-F
134.7528768	0.152883001	0.302032653	0.506180372	0.612730032	0.99483168	ENSG00000024862	CCDC28A
136.6141913	0.339853282	0.325349582	1.044578819	0.296217673	0.970706863	ENSG00000147789	ZNF7
136.2016121	0.291479246	0.373188331	0.78105134	0.4347723	0.991301167	ENSG00000138780	GSTCD
131.6189846	-0.730099258	1.431584856	-0.5099937	0.610055875	0.99483168	ENSG00000185100	ADSSL1
135.7119787	0.807687049	0.342107551	2.360915589	0.01822988	0.726053908	ENSG00000187531	SIRT7
135.5506326	-0.297016874	0.373310076	-0.795630477	0.426246847	0.991301167	ENSG00000188827	SLX4
131.2778056	-0.370050267	0.380325548	-0.972982932	0.330561785	0.976026345	ENSG00000137486	ARRB1
134.3798781	-0.391958555	0.365171682	-1.073354191	0.28311223	0.963267117	ENSG00000163026	WDCP
134.0191102	0.047658303	0.359028106	0.13274254	0.894396998	0.999467288	ENSG00000090061	CCNK
132.7053379	0.024400792	0.336447837	0.07252474	0.942184317	0.999527751	ENSG00000135299	ANKRD6
136.1703978	-0.1306853	0.303904874	-0.430020414	0.667180792	0.997568624	ENSG00000135749	PCNX2
137.3184942	-0.504908704	0.436163443	-1.157613534	0.247021784	0.962465655	ENSG00000100767	PAPLN
133.8004887	-0.479457842	0.425452234	-1.126936948	0.25976914	0.962465655	ENSG00000081189	MEF2C
134.6421411	0.059000194	0.30403	0.194060435	0.846128561	0.999467288	ENSG00000123552	USP45
128.4213717	0.817224823	1.485431586	0.550159853	0.582209737	0.99483168	ENSG00000166592	RRAD
136.3509135	-0.060455492	0.342107662	-0.176714815	0.859732404	0.999467288	ENSG00000198924	DCLRE1A
134.6184623	-0.177828989	0.327106199	-0.543642979	0.586687178	0.99483168	ENSG00000081665	ZNF506
135.049301	0.493444002	0.61762234	0.798941311	0.424324444	0.991301167	ENSG00000048740	CELF2
135.3143773	-0.386352972	0.340420961	-1.134927096	0.256405866	0.962465655	ENSG00000198551	ZNF627
134.9936482	0.04822133	0.334390906	0.144206462	0.885337438	0.999467288	ENSG00000137338	PGBD1
133.3088863	-0.369935577	0.309188572	-1.196472352	0.231512281	0.959575179	ENSG00000155621	C9orf85
133.5563931	0.043657979	0.315899453	0.138202136	0.890080667	0.999467288	ENSG00000141428	C18orf21
133.7385672	0.427065237	0.38616841	1.105904124	0.26876802	0.962465655	ENSG00000151665	PIGF
133.1642745	-0.254671677	0.367710534	-0.692587386	0.488568528	0.993513219	ENSG00000134490	TMEM241
134.1783099	0.010769584	0.367540616	0.029301752	0.97662393	0.999527751	ENSG00000021826	CPS1
133.8272748	0.276112506	0.30275501	0.911999794	0.361768826	0.979465649	ENSG00000155868	MED7
134.3293025	0.085833123	0.320826131	0.267537818	0.7890551	0.999467288	ENSG00000125485	DDX31
128.6277113	-0.017257291	0.747364369	-0.023090867	0.981577791	0.999527751	ENSG00000130600	H19
133.0477282	-0.276381792	0.30233293	-0.914163706	0.360630832	0.979465649	ENSG00000111554	MDM1
135.0549548	0.115089245	0.332824223	0.34579588	0.729496121	0.999467288	ENSG00000102738	MRPS31
132.8071727	7.56E-05	0.286455707	0.000263983	0.999789372	0.9998586	ENSG00000105258	POLR2I
133.1434438	-0.220556968	0.304386492	-0.724595124	0.468700451	0.99135833	ENSG00000181264	TMEM136

135.0976546	3.468408427	1.625177758	2.13417173	0.032828726	0.775963115	ENSG00000183856	IQGAP3
132.848309	0.070482981	0.279283685	0.252370564	0.800754651	0.999467288	ENSG00000136243	NUPL2
133.3792711	-0.097228513	0.298068176	-0.326195552	0.744276406	0.999467288	ENSG00000241127	YAE1
130.9109972	-0.298799025	0.416602527	-0.717228067	0.473233384	0.99135833	ENSG00000167617	CDC42EP5
132.8896039	-0.420243533	0.417042515	-1.00767552	0.313610261	0.973633446	ENSG00000106031	HOXA13
133.0581485	-0.049890989	0.307273845	-0.162366533	0.871017224	0.999467288	ENSG00000155636	RBM45
130.9337339	-0.601704589	0.676272555	-0.889736815	0.373607221	0.982521685	ENSG00000078053	AMPH
133.5934989	-0.306820614	0.297199524	-1.032372493	0.301897653	0.972793733	ENSG00000198105	ZNF248
133.634892	-0.234623543	0.336079424	-0.698119332	0.485102569	0.992117871	ENSG00000198783	ZNF830
132.1716219	0.581813766	0.421678214	1.37975771	0.167661258	0.943223078	ENSG00000159147	DONSON
133.4383108	-0.561075753	0.416381168	-1.347505115	0.177817607	0.9469453	ENSG00000156711	MAPK13
133.0634329	0.431026624	0.317298298	1.358427154	0.174328183	0.945838447	ENSG00000257621	PSMA3-AS1
132.8586814	-0.200431885	0.30456439	-0.65809363	0.510477969	0.993513219	ENSG00000169609	C15orf40
135.2459655	-0.012336547	0.469827996	-0.026257581	0.979051889	0.999527751	ENSG00000138316	ADAMTS14
133.5466533	0.179787483	0.368588725	0.487772606	0.625710917	0.99483168	ENSG00000090376	IRAK3
132.7756434	-0.001433068	0.326313309	-0.004391692	0.996495948	0.999527751	ENSG00000104731	KLHDC4
127.7071746	0.942873958	0.648429292	1.454089089	0.145921608	0.936017041	ENSG00000163975	MELTF
133.2203521	-0.061958382	0.286537968	-0.216230968	0.828807715	0.999467288	ENSG00000198246	SLC29A3
132.2031566	0.185901919	0.428079175	0.434269944	0.664092427	0.997568624	ENSG00000048162	NOP16
132.5781828	-0.351271147	0.319161261	-1.100607089	0.271067699	0.962465655	ENSG00000167625	ZNF526
132.814622	0.002053151	0.322671813	0.006362971	0.994923118	0.999527751	ENSG00000163312	HELQ
130.8283567	0.00438377	0.390281104	0.01123234	0.991038078	0.999527751	ENSG00000159884	CCDC107
131.9709064	-0.046673143	0.414289853	-0.112658186	0.910301553	0.999527751	ENSG00000237296	SMG1P1
131.947343	1.349470651	1.314909379	1.026284147	0.304757667	0.973427483	ENSG00000080986	NDC80
131.1855257	0.003619665	0.309598357	0.011691488	0.990671755	0.999527751	ENSG00000114993	RTKN
132.4376551	-0.179408251	0.360159707	-0.498135265	0.618388706	0.99483168	ENSG00000083093	PALB2
131.5568306	0.082128003	0.343230148	0.239279689	0.810888714	0.999467288	ENSG00000172296	SPTLC3
126.7977734	1.02252627	0.540129354	1.893113681	0.058342753	0.852621724	ENSG00000146950	SHROOM2
131.6662907	-0.137455106	0.324494432	-0.423597735	0.671859208	0.997568624	ENSG00000151413	NUBPL
130.7838445	-0.115483967	0.368715426	-0.313206225	0.754123989	0.999467288	ENSG00000214941	ZSWIM7
129.7985607	0.176989679	0.351263738	0.503865502	0.614355894	0.99483168	ENSG00000183077	AFMID
130.8563367	0.19135759	0.331325112	0.577552328	0.5635664	0.99483168	ENSG00000157426	AASDH
130.9936824	-0.829600021	0.411106134	-2.017970429	0.043594341	0.807325344	ENSG00000165895	ARHGAP42
129.2675934	-0.223138402	0.366968919	-0.608058041	0.543148979	0.99483168	ENSG00000159685	CHCHD6
131.6445171	-0.561169667	0.387684022	-1.447492376	0.14775907	0.936396974	ENSG00000204498	NFKBIL1
128.318282	-0.178521365	0.424191713	-0.420850666	0.673864132	0.998869317	ENSG00000169689	CENPX
132.8849132	0.38660917	0.420001454	0.920494837	0.35731423	0.979427314	ENSG00000008083	JARID2
131.8739287	-0.016790806	0.348147074	-0.04822906	0.961533691	0.999527751	ENSG00000148399	DPH7
130.8533583	0.09682713	0.328061185	0.295149607	0.767879596	0.999467288	ENSG00000100601	ALKBH1
131.3084976	-0.976881178	0.428493443	-2.279804261	0.0226193	0.744905651	ENSG00000168040	FADD
132.8005778	0.007320892	0.322012242	0.022734825	0.981861796	0.999527751	ENSG00000197037	ZSCAN25
131.3794701	-0.038541638	0.292942941	-0.131567047	0.895326751	0.999467288	ENSG00000170190	SLC16A5
128.0224803	-0.378098804	0.483941286	-0.781290654	0.434631565	0.991301167	ENSG00000130748	TMEM160
131.6272243	0.060359097	0.67787615	0.089041481	0.929048944	0.999527751	ENSG00000116711	PLA2G4A
129.0513859	0.018192464	0.32194493	0.056508001	0.954937122	0.999527751	ENSG00000185904	LINC00839
132.2836463	0.09955312	0.338871918	0.293778017	0.768927534	0.999467288	ENSG00000197841	ZNF181
131.9142886	-0.234253902	0.329332236	-0.711299643	0.476898573	0.99135833	ENSG00000138756	BMP2K
130.1871943	-0.446201214	0.341181425	-1.307812155	0.19093703	0.949234949	ENSG00000141040	ZNF287
130.6820133	0.476429894	0.431259162	1.1047415	0.269271615	0.962465655	ENSG00000189050	RNFT1
129.9011645	0.396365245	0.398410008	0.994867692	0.319800618	0.974964731	ENSG00000109452	INPP4B
130.7813278	0.049928466	0.29800606	0.167541781	0.866943775	0.999467288	ENSG00000074621	SLC24A1
130.3887877	-0.442002506	0.549198285	-0.804814068	0.420926982	0.991301167	ENSG00000183876	ARSI
130.4239872	0.808365855	0.62963681	1.28386054	0.199190756	0.954310328	ENSG00000170271	FAXDC2
130.6849451	-0.322059584	0.356016362	-0.904620174	0.365666644	0.981269214	ENSG00000088970	KIZ
130.3332639	0.28671718	0.540477298	0.53048885	0.595773038	0.99483168	ENSG00000134343	ANO3
131.3757613	0.240134192	0.303606922	0.790937805	0.428980286	0.991301167	ENSG00000135473	PAN2
130.5492942	0.179264888	0.305192532	0.587382946	0.556946545	0.99483168	ENSG00000105948	TTC26
129.9249847	-0.318151333	0.363951623	-0.874158302	0.382032051	0.985477569	ENSG00000179886	TIGD5
131.6041916	0.67909893	0.344677563	1.970244087	0.048810403	0.83480477	ENSG00000149716	LTO1
130.2533572	0.036831082	0.317156133	0.11612918	0.907550163	0.999527751	ENSG00000112365	ZBTB24
131.1350811	0.729700322	0.416489223	1.7520269	0.079769182	0.897052166	ENSG00000106415	GLCCI1
131.6339639	-0.200341369	0.347581793	-0.576386258	0.564354131	0.99483168	ENSG00000131845	ZNF304
130.1250178	-0.136130046	0.316489674	-0.430124764	0.667104887	0.997568624	ENSG00000167280	ENGASE
130.2850653	-0.301704146	0.357176967	-0.844690933	0.398283423	0.988378925	ENSG00000130734	ATG4D

131.0069974	0.399806486	0.357918692	1.11703159	0.263980819	0.962465655	ENSG00000196417	ZNF765
130.4402556	-0.248724484	0.339391824	-0.732853493	0.463647791	0.99135833	ENSG00000136874	STX17
129.9293248	1.618152824	0.415350843	3.895869845	9.78E-05	0.067295389	ENSG00000255717	SNHG1
128.8068096	-0.421961587	0.383904393	-1.099131956	0.271710513	0.962465655	ENSG00000204237	OXLD1
129.6996251	0.267939716	0.318580315	0.841042912	0.400323895	0.988492517	ENSG00000196459	TRAPPC2
128.8153854	0.043554717	0.328704093	0.132504334	0.894585395	0.999467288	ENSG00000101247	NDUFAF5
129.8101468	0.672777058	0.622494986	1.080775064	0.279797183	0.963267117	ENSG00000079156	OSBPL6
128.4643541	0.186062176	0.353326302	0.526601543	0.59847032	0.99483168	ENSG00000135045	C9orf40
128.36528	-0.55810267	0.348716794	-1.600446781	0.109499504	0.923922314	ENSG00000109944	JHY
131.3674521	0.282058929	0.364379565	0.774079987	0.438883491	0.991301167	ENSG00000258890	CEP95
128.546593	0.102662925	0.30776194	0.333579014	0.738697256	0.999467288	ENSG00000124444	ZNF576
127.6290011	0.29540676	0.365724917	0.80772938	0.419246387	0.990226751	ENSG00000242193	CRYZL2P
128.5472172	0.333331581	0.291531046	1.143382791	0.252879694	0.962465655	ENSG00000185418	TARSL2
126.8832989	-0.056863216	0.365622175	-0.155524526	0.87640782	0.999467288	ENSG00000181754	AMIGO1
128.5197123	0.563033002	0.393769845	1.42985302	0.152759208	0.939432741	ENSG00000196352	CD55
127.6392452	0.029046209	0.34122729	0.085122762	0.932163795	0.999527751	ENSG00000196636	SDHAF3
126.9351925	-0.021944548	0.400791022	-0.054753093	0.956335171	0.999527751	ENSG00000198720	ANKRD13B
129.1606286	-0.214130839	0.377080381	-0.567865236	0.570126479	0.99483168	ENSG00000160145	KALRN
128.7160774	-0.198030988	0.33170795	-0.597004045	0.55050468	0.99483168	ENSG00000167264	DUS2
126.982634	-0.034667857	0.37416006	-0.092655151	0.926177528	0.999527751	ENSG00000106399	RPA3
127.9147008	0.457639426	0.293194963	1.560870699	0.118554262	0.923922314	ENSG00000127993	RBM48
126.6203447	0.258590307	0.361855499	0.714623125	0.474841952	0.99135833	ENSG00000107201	DDX58
127.4395739	-0.137186806	0.373452588	-0.367347316	0.713359964	0.99900742	ENSG00000105248	YJU2
128.8140995	0.346565816	0.352747538	0.982475507	0.325865644	0.976026345	ENSG00000183137	CEP57L1
127.8216471	-0.334778059	0.374937618	-0.892890025	0.371916069	0.981955904	ENSG00000143067	ZNF697
128.268606	0.298910684	0.324479109	0.92120163	0.356945167	0.979427314	ENSG00000144791	LIMD1
129.7938626	0.33029512	0.455175553	0.725643365	0.468057429	0.99135833	ENSG00000240230	COX19
125.9314614	1.112289218	0.507987406	2.189599987	0.02855326	0.770696352	ENSG00000106484	MEST
129.1205948	-0.107859664	0.374433867	-0.288060653	0.773300311	0.999467288	ENSG00000171533	MAP6
127.68106	-0.007593869	0.325489952	-0.023330579	0.98138658	0.999527751	ENSG00000168116	KIAA1586
129.7174018	-0.642135106	1.256378412	-0.51110008	0.60928098	0.99483168	ENSG00000198729	PPP1R14C
128.6490134	-0.077378785	0.321854914	-0.24041511	0.810008465	0.999467288	ENSG00000160961	ZNF333
126.7433389	0.082750151	0.404603481	0.204521599	0.837945915	0.999467288	ENSG00000145779	TNFAIP8
128.5427079	0.166043333	0.313628348	0.529427056	0.596509233	0.99483168	ENSG00000185917	SETD4
125.9671865	0.027477148	0.339354727	0.080968808	0.935466758	0.999527751	ENSG00000105173	CCNE1
128.5314055	0.549018632	0.460868958	1.191268413	0.23354823	0.959684953	ENSG00000230409	TCEA1P2
127.337658	-0.030807824	0.342237097	-0.09001895	0.928272156	0.999527751	ENSG00000233016	SNHG7
128.6868247	-0.003663011	0.400670644	-0.009142198	0.992705683	0.999527751	ENSG00000149679	CABLES2
125.1621376	0.117142722	0.427846628	0.273796062	0.78424135	0.999467288	ENSG00000085276	MECOM
127.6499533	0.478701872	0.306528957	1.561685646	0.118362062	0.923922314	ENSG00000164306	PRIMPOL
127.9552611	0.392328122	0.375629254	1.044455718	0.296274597	0.970706863	ENSG00000140830	TXNL4B
127.3734409	-0.411040752	0.391867639	-1.048927524	0.294211477	0.970706863	ENSG00000182013	PNMA8A
128.0136015	0.395949654	0.338556479	1.169523193	0.242192902	0.962465655	ENSG00000109618	SEPSECS
127.8318815	-0.176821657	0.323327028	-0.546881767	0.584459963	0.99483168	ENSG00000184719	RNLS
127.1117794	-0.314729679	0.358405321	-0.87813897	0.379868307	0.983585589	ENSG00000176473	WDR25
125.7092989	0.298901872	0.376810272	0.793242368	0.427636627	0.991301167	ENSG00000166821	PEX11A
127.4996948	0.234823045	0.359641711	0.652936067	0.513797496	0.993513219	ENSG00000115109	EPB41L5
127.2503921	-0.803184301	0.345263182	-2.326295832	0.020002774	0.73975641	ENSG00000188856	RPSAP47
129.9854499	1.361666362	0.488989556	2.78465326	0.005358499	0.487175435	ENSG00000256087	ZNF432
126.4339139	-0.778920747	0.404643729	-1.924954448	0.054235057	0.847598083	ENSG00000054277	OPN3
126.7922301	0.087280641	0.295454099	0.295411845	0.767679286	0.999467288	ENSG00000172977	KAT5
127.5742247	-0.144437397	0.312490844	-0.462213213	0.643928429	0.997332006	ENSG00000128872	TMOD2
124.8836084	0.139146759	0.442314239	0.314588015	0.75307448	0.999467288	ENSG00000138378	STAT4
125.7008629	-0.005426759	0.317878363	-0.017071811	0.986379327	0.999527751	ENSG00000181004	BBS12
126.7010204	0.154223841	0.315480468	0.48885385	0.62494517	0.99483168	ENSG00000135953	MFSD9
125.0726448	-0.146417337	0.426467352	-0.343326017	0.73135321	0.999467288	ENSG00000066583	ISOC1
127.5819943	0.192625338	0.359155862	0.53632798	0.591731896	0.99483168	ENSG00000169131	ZNF354A
126.8048974	-0.450975505	0.297267041	-1.517072003	0.129248506	0.928627271	ENSG00000092098	RNF31
127.4085239	-0.507856801	0.371163863	-1.368281915	0.17122385	0.944422693	ENSG00000243199	AC115223.1
127.0904962	0.290602821	0.359599258	0.808129646	0.419015953	0.990226751	ENSG00000183605	SFXN4
127.4867818	0.021029654	0.339007399	0.062033022	0.950536535	0.999527751	ENSG00000213799	ZNF845
133.8646047	-0.583763872	0.733329546	-0.796045755	0.426005442	0.991301167	ENSG00000188580	NKAIN2
128.3069013	0.098659905	0.4673421	0.211108533	0.832802582	0.999467288	ENSG00000167995	BEST1
127.5291506	0.475887727	0.453821258	1.048623702	0.294351343	0.970706863	ENSG00000133874	RNF122

124.516129	-0.098306924	0.327582828	-0.300097916	0.764102469	0.999467288	ENSG00000172572	PDE3A
127.9152662	0.201024067	0.35000975	0.574338477	0.565738777	0.99483168	ENSG00000259623	AC125257.1
124.3615757	0.156529347	0.318528819	0.491413455	0.62313405	0.99483168	ENSG00000176055	MBLAC2
123.5494684	-0.142855458	0.398040444	-0.358896842	0.719672262	0.99900742	ENSG00000175182	FAM131A
120.6030006	1.043567896	0.640965672	1.628118229	0.103499819	0.92169898	ENSG00000185585	OLFML2A
125.9019704	0.024310291	0.377520877	0.06439456	0.948656062	0.999527751	ENSG00000100926	TM9SF1
125.1742107	-0.264050185	0.358023726	-0.73752147	0.460805291	0.99135833	ENSG00000187098	MITF
124.5897736	-0.069846346	0.308395246	-0.226483214	0.820825614	0.999467288	ENSG00000167380	ZNF226
125.259689	-0.280490978	0.382121852	-0.734035431	0.462927145	0.99135833	ENSG00000151611	MMAA
126.3317121	0.456318946	0.306614298	1.488250709	0.136684789	0.934144427	ENSG00000147121	KRBOX4
126.0347307	0.501951152	0.393823814	1.274557641	0.202465858	0.954310328	ENSG00000197056	ZMYM1
126.5942173	0.545182885	0.424132474	1.285407079	0.198650069	0.954310328	ENSG00000262576	PCDHGA4
126.289039	0.996589892	1.091010747	0.913455614	0.361002968	0.979465649	ENSG00000138778	CENPE
124.8849464	0.792563167	0.425396278	1.863117303	0.062445764	0.868050216	ENSG00000213551	DNAJC9
124.5314432	0.377601068	0.354630774	1.064772421	0.286978928	0.965284229	ENSG00000161642	ZNF385A
125.2174041	0.237068113	0.436422148	0.543208254	0.586986424	0.99483168	ENSG00000085871	MGST2
125.4304704	0.13196679	0.302247245	0.43661867	0.66238792	0.997568624	ENSG00000124608	AARS2
124.2289743	0.435135854	0.389542474	1.117043412	0.263975764	0.962465655	ENSG00000228594	FNDC10
125.7535928	-0.612137442	0.33189693	-1.844360063	0.065130684	0.868202292	ENSG00000050327	ARHGEF5
125.8433086	0.34318702	0.364326718	0.941975987	0.346204934	0.977691382	ENSG00000134815	DHX34
124.5535245	-0.079997789	0.363340686	-0.220172947	0.825736465	0.999467288	ENSG00000175352	NRIP3
124.1399435	-0.545736325	0.303014971	-1.801020993	0.071699571	0.884210289	ENSG00000163528	CHCHD4
124.5969424	-0.576929871	0.404759688	-1.425363957	0.154052021	0.939432741	ENSG00000218175	AC016739.1
123.667505	0.328375126	0.35227583	0.932153438	0.351257234	0.977691382	ENSG00000062822	POLD1
125.7429663	0.157414803	0.389889177	0.403742428	0.686402136	0.998869317	ENSG00000132334	PTPRE
126.652745	2.791554305	1.681211709	1.660441864	0.096825594	0.921147998	ENSG00000072571	HMMR
123.308781	0.041884646	0.326052711	0.128459738	0.897785159	0.999467288	ENSG00000204536	CCHCR1
124.8822491	-0.363077115	0.511420384	-0.709938685	0.47774216	0.99135833	ENSG00000164604	GPR85
125.0739438	-0.279287397	0.501098545	-0.557350246	0.577288152	0.99483168	ENSG00000153094	BCL2L11
124.6861168	0.011867487	0.315636443	0.037598595	0.970007728	0.999527751	ENSG00000152348	ATG10
123.5729585	-0.07636407	0.333927482	-0.228684593	0.819114071	0.999467288	ENSG00000152404	CWF19L2
124.5175361	0.401138254	0.312150182	1.285080955	0.198763996	0.954310328	ENSG00000198089	SFI1
122.206103	1.152307152	0.401638243	2.869017509	0.00411749	0.460999293	ENSG00000117616	RSRP1
125.0470017	0.150105312	0.300482324	0.499547893	0.617393456	0.99483168	ENSG00000138468	SENP7
122.8066231	-0.376764941	0.399089941	-0.944060229	0.345138871	0.977691382	ENSG00000155034	FBXL18
123.8944569	-0.909704215	0.397417574	-2.28903872	0.022077103	0.744905651	ENSG00000184227	ACOT1
132.4483102	-0.438073402	1.307316964	-0.335093488	0.737554565	0.999467288	ENSG00000123689	G0S2
125.0837818	0.316059339	0.317425747	0.995695347	0.319398192	0.974964731	ENSG00000145996	CDKAL1
124.4348463	0.478978021	0.308375217	1.553231239	0.120367901	0.923922314	ENSG00000153975	ZUP1
123.5775326	-0.216331533	0.329536032	-0.656473077	0.511519785	0.993513219	ENSG00000156853	ZNF689
123.9979069	-0.22532663	0.339714895	-0.663281573	0.507150236	0.993513219	ENSG00000120696	KBTBD7
121.3827402	-0.038478654	0.554086614	-0.069445196	0.944635255	0.999527751	ENSG00000103253	HAGHL
123.7917258	0.42538562	0.324049591	1.312717656	0.189278106	0.949094286	ENSG00000099377	HSD3B7
124.4997679	-0.100052948	0.394960976	-0.25332363	0.800018137	0.999467288	ENSG00000249673	NOP14-AS1
122.9546866	-0.280891829	0.32507874	-0.864073205	0.387547678	0.986799595	ENSG00000176058	TPRN
124.5697882	0.601796977	0.326561453	1.842829188	0.065353954	0.868202292	ENSG00000118276	B4GALT6
119.9269832	-0.40841085	0.734995692	-0.555664278	0.578440381	0.99483168	ENSG00000122035	RASL11A
123.2971165	-0.083738494	0.367458065	-0.227885851	0.819734982	0.999467288	ENSG00000119862	LGALSL
126.0499205	0.600303337	0.458349981	1.309705164	0.190295596	0.949094286	ENSG00000065665	SEC61A2
122.4614872	-0.455633067	0.411561751	-1.107083121	0.268257992	0.962465655	ENSG00000224870	AL391244.1
122.0224518	-0.070921325	0.457121286	-0.155147719	0.876704864	0.999467288	ENSG00000134602	STK26
123.7324585	-0.089143716	0.347891257	-0.256240174	0.79776539	0.999467288	ENSG00000081386	ZNF510
122.4383671	0.112643918	0.346041711	0.325521214	0.74478663	0.999467288	ENSG00000177595	PIDD1
122.5697358	-0.128045514	0.315728748	-0.405555448	0.68506927	0.998869317	ENSG00000138101	DTNB
124.585091	0.603290555	0.38955411	1.548669464	0.121461198	0.923922314	ENSG00000204681	GABBR1
123.2121462	0.016061818	0.344526047	0.046620039	0.962816061	0.999527751	ENSG00000213860	RPL21P75
123.839377	-0.106286378	0.333851619	-0.318364125	0.750208748	0.999467288	ENSG00000104221	BRF2
120.5726671	-0.406190938	0.547584522	-0.741786741	0.458216555	0.99135833	ENSG00000100116	GCAT
122.7763791	0.110117756	0.290886048	0.378559773	0.705014799	0.99900742	ENSG00000003509	NDUFAF7
122.8712584	-0.034527956	0.312377099	-0.110532929	0.911986736	0.999527751	ENSG00000163138	PACRGL
122.5274503	1.709451229	1.29260142	1.322489054	0.186005352	0.949094286	ENSG00000090889	KIF4A
122.1008287	0.14666456	0.305595176	0.479930875	0.631276546	0.99483168	ENSG00000141542	RAB40B
123.3779306	-0.050391745	0.315751529	-0.159593035	0.873201666	0.999467288	ENSG00000183579	ZNRF3
122.8177769	0.037579953	0.299758162	0.125367573	0.900232559	0.999527751	ENSG00000124596	OARD1

123.0029314	-0.379574275	0.393294013	-0.965115823	0.334486802	0.976026345	ENSG00000158292	GPR153
122.7626157	0.479040192	0.311523243	1.537734993	0.124113424	0.927794614	ENSG00000226752	CUTALP
120.7513136	0.011528235	0.392399788	0.029378801	0.97656248	0.999527751	ENSG00000234608	MAPKAPK5-AS1
122.6341125	0.780037134	0.614037033	1.270342166	0.203962774	0.954310328	ENSG00000168952	STXBP6
119.5130203	-0.621424778	0.404079064	-1.537879177	0.12407816	0.927794614	ENSG00000196839	ADA
122.2864985	0.109069173	0.370347707	0.294504788	0.768372205	0.999467288	ENSG00000102221	JADE3
121.2206562	0.093348031	0.643358851	0.145094811	0.884636015	0.999467288	ENSG00000149150	SLC43A1
120.7922863	-0.445328409	0.356747823	-1.248300285	0.21192111	0.954310328	ENSG00000176973	FAM89B
127.9104327	0.230326753	2.070543946	0.111239732	0.911426246	0.999527751	ENSG00000149968	MMP3
120.5234281	-0.642826228	0.494216179	-1.30069847	0.193361686	0.950382812	ENSG00000198624	CCDC69
127.9957994	-1.190489222	0.526951179	-2.259202122	0.023870815	0.744948012	ENSG00000095303	PTGS1
122.9178023	-0.090158202	0.456431609	-0.197528392	0.843414065	0.999467288	ENSG00000134569	LRP4
131.9035375	1.145550136	1.302185085	0.879713759	0.379014394	0.982778604	ENSG00000139269	INHBE
122.0416149	0.962930566	0.496216238	1.940546264	0.052313337	0.846840376	ENSG00000167670	CHAF1A
121.8972863	-0.231907488	0.339895209	-0.682291135	0.495054877	0.993513219	ENSG00000101134	DOK5
121.621251	-0.075248208	0.37600841	-0.200123736	0.84138381	0.999467288	ENSG00000133250	ZNF414
122.6309119	0.203033747	0.341675896	0.594229062	0.552358918	0.99483168	ENSG00000184083	FAM120C
127.7075975	-0.582871235	1.289118682	-0.45214707	0.651163036	0.997449432	ENSG00000095713	CRTAC1
121.4358208	0.375188118	0.300209428	1.249754616	0.211389199	0.954310328	ENSG00000158411	MITD1
122.8924978	0.322382279	0.307196093	1.049434826	0.293978037	0.970706863	ENSG00000165792	METTL17
122.7008604	2.638114455	1.324021444	1.992501305	0.046316082	0.818574412	ENSG00000075218	GTSE1
122.8897462	-0.753369078	0.413300578	-1.822811578	0.068331934	0.87420149	ENSG00000049769	PPP1R3F
120.8278753	0.890644184	0.385223906	2.312016909	0.020776752	0.73997555	ENSG00000091409	ITGA6
120.9355844	1.9391004	1.244933853	1.557593117	0.119329731	0.923922314	ENSG00000146670	CDCA5
117.7035406	-0.035037838	0.682968011	-0.051302312	0.959084626	0.999527751	ENSG00000112297	CRYBG1
121.9590897	0.36235109	0.347033891	1.044137473	0.296421791	0.970706863	ENSG00000276855	AC015922.3
121.749152	0.18877572	0.34224361	0.55158289	0.581234162	0.99483168	ENSG00000101868	POLA1
121.8302435	0.630894907	0.365921348	1.724127083	0.084684904	0.901307093	ENSG00000268043	NBPF12
121.6046755	-0.144086313	0.330562079	-0.435882766	0.66292179	0.997568624	ENSG00000111860	CEP85L
119.6627665	-0.245950293	0.446309431	-0.551075725	0.581581765	0.99483168	ENSG00000147408	CSGALNACT1
121.6318747	-0.230657354	0.350089121	-0.658853246	0.509990012	0.993513219	ENSG00000136169	SETDB2
119.4723313	-0.385368506	0.397497075	-0.969487652	0.332301938	0.976026345	ENSG00000164967	RPP25L
121.7075515	-0.105607145	0.358143743	-0.294873629	0.768090418	0.999467288	ENSG00000099822	HCN2
121.2096505	-0.485299981	0.401856578	-1.207644736	0.227183944	0.958865115	ENSG00000241399	CD302
120.1505728	0.410740057	0.385737663	1.064817093	0.286958709	0.965284229	ENSG00000151150	ANK3
121.1099728	-0.508786683	0.319269726	-1.593595141	0.111026744	0.923922314	ENSG00000196227	FAM217B
117.7390894	0.262021097	0.759139334	0.345155474	0.729977491	0.999467288	ENSG00000138449	SLC40A1
121.7868456	0.472292152	0.366316705	1.289300066	0.197293788	0.954310328	ENSG00000145241	CENPC
121.8514091	0.040127508	0.332479719	0.120691595	0.903935316	0.999527751	ENSG00000137656	BUD13
118.9224044	-0.192550564	0.457784026	-0.420614423	0.67403666	0.998869317	ENSG00000119922	IFIT2
121.4008856	-0.031120829	0.373119715	-0.083407088	0.933527852	0.999527751	ENSG00000122678	POLM
120.8386024	-0.173841332	0.302690518	-0.574320375	0.565751024	0.99483168	ENSG00000128512	DOCK4
121.1855155	0.185537174	0.297862467	0.622895446	0.533353231	0.99483168	ENSG00000171827	ZNF570
118.5133524	-0.692241425	0.44143048	-1.568177678	0.116839672	0.923922314	ENSG00000183828	NUDT14
120.6448109	-0.105108835	0.329667425	-0.318832941	0.749853198	0.999467288	ENSG00000225484	NUTM2B-AS1
119.6990622	-0.165334544	0.335858661	-0.49227417	0.622525537	0.99483168	ENSG00000132840	BHMT2
120.8418735	0.123864563	0.347852783	0.356083289	0.721778192	0.99900742	ENSG00000179029	TMEM107
120.5826721	-0.404743554	0.383595197	-1.055131964	0.291364975	0.969851196	ENSG00000177045	SIX5
120.981822	0.185203575	0.330058773	0.561123019	0.574713677	0.99483168	ENSG00000107938	EDRF1
119.5113136	-0.12717715	0.333917894	-0.380863537	0.70330451	0.99900742	ENSG00000165898	ISCA2
119.0735059	0.040108526	0.359039009	0.111710776	0.911052735	0.999527751	ENSG00000100376	FAM118A
118.8230905	-0.053303739	0.406968768	-0.130977469	0.895793131	0.999467288	ENSG00000079691	CARMIL1
119.5296678	0.180872256	0.306222779	0.590655786	0.554751078	0.99483168	ENSG00000183808	RBM12B
118.1529316	-0.604444622	0.434683895	-1.390538341	0.164365465	0.943223078	ENSG00000167994	RAB3IL1
120.3069316	0.245154451	0.380008435	0.645128972	0.51884361	0.993513219	ENSG00000064687	ABCA7
119.9886509	0.561327324	0.408914923	1.372723986	0.169838169	0.944422693	ENSG00000116830	TTF2
121.1648517	-0.169535047	0.364500115	-0.465116579	0.641847974	0.996696873	ENSG00000147421	HMBOX1
120.3953111	-0.441428673	0.296784736	-1.487369864	0.136917152	0.934144427	ENSG00000108187	PBLD
133.2858186	-1.434548808	1.453003527	-0.987298916	0.323496122	0.975412329	ENSG00000186832	KRT16
119.7444252	0.507759956	0.523616523	0.969717215	0.332187467	0.976026345	ENSG00000138821	SLC39A8
119.8968819	0.012788789	0.339648194	0.037653046	0.969964313	0.999527751	ENSG00000253873	PCDHGA11
121.4284444	0.451072085	0.369460888	1.220892658	0.222126665	0.956884001	ENSG00000165195	PIGA
117.7098438	0.497046456	0.372331245	1.334957683	0.181890197	0.949094286	ENSG00000136383	ALPK3
117.9017363	-0.231089867	0.41266374	-0.559995572	0.575482458	0.99483168	ENSG00000131652	THOC6

120.1666887	0.557250786	0.348127616	1.600708364	0.109441528	0.923922314	ENSG00000125814	NAPB
120.1447325	0.060872956	0.31088219	0.195807152	0.844761112	0.999467288	ENSG00000171169	NAIF1
119.4517725	-0.136512757	0.395894455	-0.344821088	0.730228879	0.999467288	ENSG00000184232	OAF
119.0592079	-0.103034815	0.312706933	-0.329493222	0.741782916	0.999467288	ENSG00000170089	AC106795.1
119.7833479	-0.131672968	0.307315872	-0.428461334	0.668315279	0.997568624	ENSG00000105875	WDR91
120.7662478	0.076196863	0.321090392	0.237306582	0.812418956	0.999467288	ENSG00000107815	TWNK
119.6362953	-0.010337676	0.307102966	-0.033661922	0.973146743	0.999527751	ENSG00000123815	COQ8B
118.5895167	0.090048836	0.340437978	0.264508784	0.791387897	0.999467288	ENSG00000118655	DCLRE1B
120.5881495	-0.155638571	0.423839731	-0.367210905	0.713461706	0.99900742	ENSG00000132846	ZBED3
122.9201217	-0.52569361	0.525069005	-1.001189566	0.31673517	0.974768742	ENSG00000177679	SRRM3
118.8878454	-0.209929497	0.395506057	-0.53078706	0.595566348	0.99483168	ENSG00000102078	SLC25A14
119.2273389	0.034101599	0.499785388	0.068232485	0.945600568	0.999527751	ENSG00000135454	B4GALNT1
119.1151567	1.086597438	0.578586119	1.878021963	0.060378161	0.858996918	ENSG00000079616	KIF22
120.3844424	0.16080176	0.355739495	0.452021106	0.651253778	0.997449432	ENSG00000184986	TMEM121
121.740648	-0.084173868	0.378965702	-0.222114739	0.824224565	0.999467288	ENSG00000138411	HECW2
121.108698	0.168682758	0.440595789	0.382851498	0.701829875	0.998869317	ENSG00000145819	ARHGAP26
116.0422087	0.442864598	0.518071278	0.854833334	0.392643421	0.988378925	ENSG00000168405	CMAHP
118.7061712	0.561832485	0.495189085	1.134581723	0.256550615	0.962465655	ENSG00000111261	MANSC1
120.2635642	0.126145649	0.32109969	0.392855095	0.694426509	0.998869317	ENSG00000011258	MBTD1
118.8951207	0.003216585	0.445583133	0.00721882	0.994240265	0.999527751	ENSG00000168916	ZNF608
119.8570024	-0.425987591	0.391425263	-1.088298664	0.27646328	0.962465655	ENSG00000134463	ECHDC3
118.6664449	-0.076948475	0.337818388	-0.227780598	0.819816811	0.999467288	ENSG00000164610	RP9
120.8319638	0.237631897	0.381892572	0.62224802	0.533778795	0.99483168	ENSG00000175548	ALG10B
118.0575421	0.243266739	0.451542002	0.538746645	0.590061683	0.99483168	ENSG00000088899	LZTS3
120.6036555	0.213453461	0.397327377	0.537223141	0.591113489	0.99483168	ENSG00000134830	C5AR2
119.3659561	0.262196109	0.349192061	0.750865036	0.452733884	0.99135833	ENSG00000168917	SLC35G2
116.5469257	-0.075728272	0.519879528	-0.14566504	0.884185821	0.999467288	ENSG00000153721	CNKSR3
118.0096216	0.366491875	0.37791383	0.969776298	0.33215801	0.976026345	ENSG00000146826	C7orf43
118.5214715	-0.066771408	0.330854811	-0.201814833	0.840061485	0.999467288	ENSG00000132825	PPP1R3D
119.9027183	0.527188941	0.346305377	1.522323865	0.127927951	0.928627271	ENSG00000100036	SLC35E4
119.3687721	0.498373149	0.297283373	1.676424564	0.093655061	0.921147998	ENSG00000119778	ATAD2B
117.2548058	-0.06279457	0.405661752	-0.154795391	0.876982625	0.999467288	ENSG00000029639	TFB1M
120.6543162	-0.459879331	0.362191966	-1.269711574	0.204187389	0.954310328	ENSG00000183665	TRMT12
118.2725293	0.108250555	0.313660026	0.345120658	0.730003663	0.999467288	ENSG00000157353	FUK
118.9846455	0.085674183	0.348552026	0.245800274	0.805836845	0.999467288	ENSG00000163704	PRRT3
121.2442641	0.314972212	0.419294064	0.75119645	0.452534436	0.99135833	ENSG00000273033	LINC02035
117.7432251	-0.468722062	0.334068337	-1.403072395	0.160595252	0.941856092	ENSG00000137285	TUBB2B
120.9804654	0.601251642	0.697310869	0.862243325	0.388553633	0.986799595	ENSG00000076554	TPD52
119.6784736	-0.25928742	0.350740034	-0.739258125	0.459750271	0.99135833	ENSG00000196678	ERI2
118.9309054	-0.028811623	0.307305384	-0.093755673	0.925303244	0.999527751	ENSG00000253710	ALG11
121.3990173	-0.566202143	0.448653074	-1.262004375	0.206947212	0.954310328	ENSG00000267023	LRRC37A16P
118.232178	-0.241774016	0.317886697	-0.760566636	0.446915954	0.99135833	ENSG00000170260	ZNF212
116.3353556	0.120649765	0.426277911	0.28303077	0.77715325	0.999467288	ENSG00000213178	RPL22P1
116.5672827	0.054759436	0.371160785	0.14753562	0.882709267	0.999467288	ENSG00000178177	LCORL
119.2188535	0.027314458	0.339955434	0.080347173	0.935961141	0.999527751	ENSG00000172273	HINFP
117.9911401	-0.000468794	0.356438289	-0.001315217	0.998950609	0.999573482	ENSG00000145882	PCYOX1L
118.3801445	-0.266623964	0.393131894	-0.678204868	0.497641806	0.993513219	ENSG00000185973	TMLHE
118.3279422	-0.100668029	0.299842593	-0.335736255	0.737069764	0.999467288	ENSG00000110400	NECTIN1
118.8809659	0.324102408	0.32351389	1.001819142	0.31643095	0.974768742	ENSG00000184922	FMNL1
116.2625703	-0.30404128	0.386132006	-0.787402429	0.431046325	0.991301167	ENSG00000079482	OPHN1
118.1148033	0.665157962	0.480802562	1.383432648	0.166532246	0.943223078	ENSG00000247903	AC024896.1
119.1744121	1.567399938	1.25709203	1.246845816	0.212454037	0.954310328	ENSG00000134690	CDCA8
117.9062617	0.392906902	0.331297982	1.185962256	0.235637206	0.962465655	ENSG00000197375	SLC22A5
119.2906611	-0.005725346	0.403739031	-0.014180808	0.988685732	0.999527751	ENSG00000285756	BX890604.2
117.4885064	-0.044546907	0.315869362	-0.141029527	0.887846616	0.999467288	ENSG00000103550	KNOP1
115.5420451	0.34403533	0.424933059	0.809622416	0.418157221	0.990226751	ENSG00000049246	PER3
118.3190141	-0.3018205	0.310801307	-0.971104344	0.331496321	0.976026345	ENSG00000136490	LIMD2
116.6425536	0.620232233	0.450420398	1.377007424	0.168509952	0.943223078	ENSG00000214114	MYCBP
117.2732405	0.011522419	0.555061041	0.020758832	0.983438038	0.999527751	ENSG00000107833	NPM3
116.8052597	-0.086221231	0.398145482	-0.216557099	0.828553522	0.999467288	ENSG00000187118	CMC1
121.8225159	-0.122284958	0.734103283	-0.16657732	0.86770264	0.999467288	ENSG00000144681	STAC
118.6639609	0.33265291	0.396812605	0.838312358	0.401855297	0.988592117	ENSG00000152439	ZNF773
117.5780828	0.222819549	0.336444375	0.662277528	0.507793376	0.993513219	ENSG00000197937	ZNF347
115.8761621	-0.159330588	0.327828423	-0.486018225	0.626954244	0.99483168	ENSG00000143793	C1orf35

116.6270018	0.445013295	0.346596186	1.28395324	0.199158317	0.954310328	ENSG00000159733	ZFYVE28
115.0084545	0.321445959	0.637441765	0.504275021	0.614068128	0.99483168	ENSG00000185652	NTF3
117.2778296	-0.135057769	0.371059918	-0.363978329	0.715874192	0.99900742	ENSG00000165506	DNAAF2
116.2251446	-0.803004882	0.346648621	-2.31648082	0.02053203	0.73997555	ENSG00000066468	FGFR2
117.9979936	-0.270858786	0.371406637	-0.729278258	0.465831469	0.99135833	ENSG00000180035	ZNF48
117.0740144	-0.101979367	0.335115059	-0.304311503	0.760890583	0.999467288	ENSG00000101452	DHX35
117.4019494	0.750541824	0.365198479	2.055161417	0.039863416	0.793985738	ENSG00000269958	AL049840.4
115.4983642	-0.715452957	0.760108708	-0.941250837	0.34657633	0.977691382	ENSG00000041353	RAB27B
115.9870228	0.37568503	0.343703178	1.093050788	0.274371501	0.962465655	ENSG00000213930	GALT
116.7776956	0.128900318	0.343190673	0.375593885	0.707218838	0.99900742	ENSG00000110801	PSMD9
118.5316838	0.293793638	0.397220945	0.739622726	0.459528948	0.99135833	ENSG00000130856	ZNF236
116.0435022	0.063781483	0.3216276	0.198308487	0.842803712	0.999467288	ENSG00000104205	SGK3
117.7841012	0.317723778	0.387160316	0.820651717	0.41184468	0.990226751	ENSG00000215790	SLC35E2A
117.8169926	0.120272854	0.364248376	0.330194621	0.741252911	0.999467288	ENSG00000100625	SIX4
116.446432	-0.256948809	0.308873033	-0.831891363	0.405470267	0.990045328	ENSG00000114473	IQCG
116.4245759	-0.014429369	0.324971983	-0.044401886	0.964584059	0.999527751	ENSG00000264281	AC016596.1
118.909926	-0.265819754	0.453346105	-0.586350586	0.557639943	0.99483168	ENSG00000224032	EPB41L4A-AS1
122.3511819	-0.446704425	0.608598416	-0.733988806	0.46295556	0.99135833	ENSG00000004660	CAMKK1
117.3733428	0.244570444	0.325994032	0.750229819	0.453116302	0.99135833	ENSG00000186812	ZNF397
116.6763534	0.472101182	0.373465388	1.2641096	0.206190692	0.954310328	ENSG00000273136	NBPF26
116.6380484	0.121771083	0.341968738	0.356088348	0.721774403	0.99900742	ENSG00000082269	FAM135A
115.4195535	-0.054226494	0.335505932	-0.161626035	0.871600354	0.999467288	ENSG00000181035	SLC25A42
115.1027061	-0.018692317	0.333583027	-0.056034977	0.955313943	0.999527751	ENSG00000162542	TMCO4
116.3244453	-0.156047355	0.312481103	-0.499381732	0.617510486	0.99483168	ENSG00000177042	TMEM80
116.4479691	0.263044401	0.400077426	0.657483735	0.510869926	0.993513219	ENSG00000166788	SAAL1
116.9993509	-0.718989323	0.464674404	-1.547297026	0.121791639	0.923922314	ENSG00000105327	BBC3
116.3431439	-0.134711059	0.389830094	-0.34556352	0.729670766	0.999467288	ENSG00000197128	ZNF772
116.7156074	0.366783192	0.349069026	1.050746884	0.293374857	0.970706863	ENSG00000214367	HAUS3
118.9219556	1.476342782	0.605854165	2.436795631	0.014818051	0.688157931	ENSG00000170379	TCAF2
115.4020535	-0.183363939	0.310656583	-0.590246431	0.555025448	0.99483168	ENSG00000142252	GEMIN7
115.4569464	0.429690566	0.400331843	1.073335967	0.283120403	0.963267117	ENSG00000130695	CEP85
119.1563964	0.467540237	0.501620088	0.932060433	0.351305295	0.977691382	ENSG00000152117	AC073869.1
114.007734	0.083108361	0.420325961	0.197723597	0.843261326	0.999467288	ENSG00000165948	IFI27L1
116.7968319	0.682723392	0.325320298	2.098619102	0.035850491	0.785058983	ENSG00000197961	ZNF121
117.2565377	1.961571618	1.432094327	1.369722358	0.170773587	0.944422693	ENSG00000101447	FAM83D
115.2200272	0.03552956	0.33009954	0.107632868	0.914286924	0.999527751	ENSG00000183323	CCDC125
114.941376	0.019405552	0.300191032	0.064644009	0.948457444	0.999527751	ENSG00000179476	C14orf28
115.9926769	-0.003857883	0.308216101	-0.012516812	0.99001329	0.999527751	ENSG00000100084	HIRA
114.8479045	0.277715191	0.330637315	0.839939048	0.400942563	0.988492517	ENSG00000171103	TRMT61B
115.5056922	0.149829102	0.377754677	0.3966307	0.69163981	0.998869317	ENSG00000165714	BORCS5
116.6493174	-0.074877479	0.386272314	-0.193846353	0.846296191	0.999467288	ENSG00000086619	ERO1B
118.2287735	0.150335185	1.164254957	0.129125656	0.897258222	0.999467288	ENSG00000154545	MAGED4
117.2723729	0.740664098	0.400398852	1.84981574	0.064340111	0.868202292	ENSG00000005801	ZNF195
115.5554168	-0.003464731	0.353599168	-0.00979847	0.992182077	0.999527751	ENSG00000140548	ZNF710
116.2900687	2.595824346	1.411651721	1.838856077	0.065936358	0.872086825	ENSG00000123485	HJURP
115.0680269	-0.341367288	0.304398976	-1.121446901	0.262097682	0.962465655	ENSG00000179041	RRS1
115.3555178	-0.001902009	0.377890945	-0.005033221	0.995984088	0.999527751	ENSG00000166439	RNF169
116.5531141	1.031177647	0.576715899	1.78801668	0.073773317	0.888663898	ENSG00000184445	KNTC1
114.4676584	-0.491896813	0.358186454	-1.3732982	0.169659662	0.944422693	ENSG00000185019	UBOX5
116.2673188	0.001323344	0.402648355	0.003286601	0.997377677	0.999527751	ENSG00000099326	MZF1
113.9187606	-0.432147003	0.702950523	-0.614761621	0.538712158	0.99483168	ENSG00000164638	SLC29A4
117.0350116	-0.384885329	0.412821953	-0.932327667	0.351167214	0.977691382	ENSG00000176293	ZNF135
115.7913013	0.364999442	0.380791319	0.95852879	0.337796181	0.977691382	ENSG00000128973	CLN6
116.6018148	1.006971868	0.670201431	1.502491374	0.132970254	0.933741583	ENSG00000123473	STIL
115.8015343	-0.049386524	0.305685324	-0.161560009	0.871652352	0.999467288	ENSG00000160062	ZBTB8A
113.9815617	-0.128011563	0.310562482	-0.41219262	0.680198246	0.998869317	ENSG00000213516	RBMXL1
115.2055396	-0.029154288	0.320350351	-0.091007512	0.927486622	0.999527751	ENSG00000151093	OXSM
114.9102643	0.358159728	0.39402331	0.908981064	0.363360121	0.98048242	ENSG00000156521	TYSND1
114.3205965	0.909978843	0.387178704	2.350281233	0.018759231	0.726053908	ENSG00000100344	PNPLA3
113.6464367	-1.139883416	0.610673885	-1.86659925	0.061957572	0.865910462	ENSG00000004799	PDK4
113.3525647	0.348194179	0.316229158	1.101081828	0.270861045	0.962465655	ENSG00000008324	SS18L2
113.5432477	-0.378184161	0.395316154	-0.956662552	0.338737605	0.977691382	ENSG00000164169	PRMT9
114.0538159	-0.195317218	0.446849145	-0.437098783	0.66203971	0.997568624	ENSG00000184209	SNRNP35
113.9513764	-0.048385466	0.330152761	-0.146554783	0.883483446	0.999467288	ENSG00000172731	LRRC20

116.100218	-0.532405394	0.374402781	-1.422012394	0.155022653	0.939432741	ENSG00000259863	SH3RF3-AS1
114.8282643	0.463914396	0.515537119	0.899866137	0.368191493	0.981269214	ENSG00000012171	SEMA3B
114.9116357	0.008291538	0.320026933	0.025908875	0.979330021	0.999527751	ENSG00000196150	ZNF250
114.1059574	0.611815682	0.326187764	1.875654911	0.060702681	0.858996918	ENSG00000084444	FAM234B
114.670573	0.527118078	0.36104013	1.459998584	0.144290463	0.935644597	ENSG00000117602	RCAN3
114.5064788	0.315011941	0.393507204	0.800523948	0.423407294	0.991301167	ENSG00000128203	ASPHD2
115.0288414	-0.48921722	0.328664696	-1.488499451	0.136619227	0.934144427	ENSG00000171574	ZNF584
114.2545957	-0.159685688	0.319511702	-0.499780407	0.617229708	0.99483168	ENSG00000156795	WDYHV1
110.8507153	0.230010483	0.594024894	0.387206808	0.69860311	0.998869317	ENSG00000138028	CGREF1
114.2092631	-0.314233827	0.369573446	-0.850260835	0.395180086	0.988378925	ENSG00000163521	GLB1L
113.8021044	-0.011308323	0.398933583	-0.028346379	0.97738589	0.999527751	ENSG00000204388	HSPA1B
115.1130692	0.464052197	0.338923232	1.369195597	0.170938142	0.944422693	ENSG00000077458	FAM76B
112.6312123	-0.244443278	0.355213721	-0.688158322	0.491353087	0.993513219	ENSG00000188191	PRKAR1B
115.0528449	0.007064154	0.586239097	0.012049954	0.990385761	0.999527751	ENSG00000132561	MATN2
113.0316034	-0.352367383	0.388489717	-0.90701856	0.364396983	0.980802389	ENSG00000162885	B3GALNT2
114.3042632	0.144441008	0.315144159	0.458333128	0.646713127	0.997368416	ENSG00000104450	SPAG1
113.878934	-0.377068874	0.345109335	-1.092606998	0.274566389	0.962465655	ENSG00000124074	ENKD1
112.8903488	0.918526284	1.151152667	0.797918739	0.424917648	0.991301167	ENSG00000122952	ZWINT
113.4481018	-0.568980205	0.363184962	-1.566640318	0.117198788	0.923922314	ENSG00000169955	ZNF747
110.6496493	-0.426957447	0.465081448	-0.91802726	0.358604594	0.979427314	ENSG00000099330	OCEL1
111.1796373	-0.016557603	0.461057286	-0.035912246	0.971352331	0.999527751	ENSG00000163947	ARHGEF3
112.7012444	-0.798703711	0.450514917	-1.772868513	0.076250497	0.897052166	ENSG00000087842	PIR
112.8964151	0.107017311	0.319660452	0.334784333	0.73778778	0.999467288	ENSG00000012211	PRICKLE3
114.09513	-0.815795289	0.381987569	-2.135659259	0.032707197	0.775963115	ENSG00000125967	NECAB3
113.2894181	0.172816177	0.63511667	0.272101466	0.785544002	0.999467288	ENSG00000101000	PROCR
115.9325425	-0.137858493	0.469674117	-0.293519459	0.769125128	0.999467288	ENSG00000100228	RAB36
114.4267549	-0.026279848	0.451563367	-0.058197475	0.953591332	0.999527751	ENSG00000269335	IKBKG
112.4241589	0.375115425	0.326850776	1.1476657	0.251106596	0.962465655	ENSG00000262454	MIR193BHG
114.0591964	0.528558909	0.338957132	1.559368012	0.118909303	0.923922314	ENSG00000105497	ZNF175
112.9644111	0.252453469	0.309484429	0.815722682	0.414658763	0.990226751	ENSG00000160055	TMEM234
112.6469706	-0.764698433	0.3762397	-2.032476724	0.042105418	0.804797577	ENSG00000250786	SNHG18
114.3912713	-0.048125692	0.360031272	-0.133670866	0.893662844	0.999467288	ENSG00000156050	FAM161B
113.9578999	-0.459288196	0.340649797	-1.348270865	0.177571279	0.946844293	ENSG00000168781	PPIP5K1
113.7366586	0.063026548	0.428265636	0.147166951	0.883000246	0.999467288	ENSG00000103199	ZNF500
115.1252096	0.26338628	0.398701998	0.660609381	0.508862852	0.993513219	ENSG00000154265	ABCA5
112.9026562	0.075442218	0.35428913	0.212939692	0.831374003	0.999467288	ENSG00000145365	TIFA
112.4381805	0.759719505	0.398561729	1.906152673	0.056630401	0.84929896	ENSG00000092470	WDR76
114.5123987	0.272785788	0.36065021	0.756372186	0.449426079	0.99135833	ENSG00000162086	ZNF75A
113.3487151	0.497722011	0.354279716	1.404884301	0.160055686	0.941239523	ENSG00000080839	RBL1
110.6761066	-0.435854256	0.383567673	-1.136316448	0.255824149	0.962465655	ENSG00000100577	GSTZ1
113.444306	0.129741186	0.337652385	0.384244839	0.700796987	0.998869317	ENSG00000152464	RPP38
114.1647237	0.138610303	0.387959378	0.357280454	0.720881861	0.99900742	ENSG00000140104	CLBA1
112.0777643	0.126408989	0.370898335	0.340818433	0.733240273	0.999467288	ENSG00000147592	LACTB2
112.9390059	0.05228347	0.340922121	0.153358985	0.878115187	0.999467288	ENSG00000182919	C11orf54
113.4970226	0.256640477	0.330827337	0.775753537	0.437894523	0.991301167	ENSG00000168228	ZCCHC4
114.0336223	-0.210085683	0.357957931	-0.586900485	0.557270544	0.99483168	ENSG00000152422	XRCC4
113.7541188	0.086246711	0.398337758	0.216516534	0.828585139	0.999467288	ENSG00000223547	ZNF844
112.2563703	0.383894178	0.345374033	1.111531679	0.266339562	0.962465655	ENSG00000111445	RFC5
111.8101553	-0.076376322	0.33278405	-0.229507159	0.818474757	0.999467288	ENSG00000125388	GRK4
111.9158362	-0.813025383	0.335614614	-2.422496964	0.015414255	0.695712746	ENSG00000164051	CCDC51
112.6368021	-0.277024609	0.348162476	-0.795676237	0.426220242	0.991301167	ENSG00000037897	METTL1
112.4030243	-0.176278235	0.412774146	-0.427057355	0.669337553	0.997568624	ENSG00000125375	ATP5S
111.9845122	-0.012944719	0.357174082	-0.036242044	0.971089362	0.999527751	ENSG00000253626	EIF5AL1
110.6523849	-0.474797784	0.380270497	-1.248579071	0.211819071	0.954310328	ENSG00000154217	PITPNC1
112.8168018	0.120599353	0.379477692	0.317803538	0.750633967	0.999467288	ENSG00000164236	ANKRD33B
113.5915103	-0.811871865	0.656274121	-1.23709261	0.216052703	0.954563123	ENSG00000156103	MMP16
111.8990921	-0.333731953	0.328521631	-1.015859904	0.309696105	0.973427483	ENSG00000162227	TAF6L
111.7741467	-0.628757465	0.344960861	-1.822692183	0.068350025	0.87420149	ENSG00000176641	RNF152
112.6727643	0.658538438	0.330065505	1.99517498	0.046023799	0.815583282	ENSG00000196705	ZNF431
112.7952314	0.1765957	0.320931721	0.550259411	0.582141459	0.99483168	ENSG00000270696	AC005034.3
112.0986575	0.372275525	0.305707405	1.217751087	0.223318577	0.956884001	ENSG00000152382	TADA1
112.6441671	-0.109834042	0.435459077	-0.252225864	0.800866488	0.999467288	ENSG00000145736	GTF2H2
111.3762991	0.207697741	0.358541913	0.57928441	0.562397283	0.99483168	ENSG00000174010	KLHL15
112.0822752	0.033772278	0.334206224	0.101052212	0.919509013	0.999527751	ENSG00000100908	EMC9

111.584529	0.210387447	0.298482604	0.704856647	0.480899452	0.991417002	ENSG00000126453	BCL2L12
111.7217308	-0.048745307	0.31063473	-0.156921628	0.875306614	0.999467288	ENSG00000083635	NUFIP1
111.4035038	0.704972221	0.331813323	2.124604925	0.033619593	0.775963115	ENSG00000140326	CDAN1
110.2324986	-0.008023956	0.425294336	-0.01886683	0.984947341	0.999527751	ENSG00000275464	FP565260.1
111.1537391	0.175711333	0.365395882	0.480879348	0.630602251	0.99483168	ENSG00000116885	OSCP1
111.5961914	0.444578812	0.376293626	1.181467826	0.237416928	0.962465655	ENSG00000081913	PHLPP1
111.0204856	-0.466177315	0.343571807	-1.356855555	0.174827111	0.946176003	ENSG00000037757	MRI1
111.193955	-0.49209195	0.327819303	-1.501107306	0.13332781	0.933741583	ENSG00000178694	NSUN3
110.0425314	0.012744242	0.358555145	0.035543325	0.9716465	0.999527751	ENSG00000173275	ZNF449
111.5676766	0.105826953	0.374206277	0.282803789	0.77732725	0.999467288	ENSG00000196911	KPNA5
111.2932152	0.404710232	0.345767095	1.170470635	0.241811625	0.962465655	ENSG00000187522	HSPA14
110.9960863	0.22186516	0.306583815	0.723668863	0.469269054	0.99135833	ENSG00000174109	C16orf91
110.2271525	-0.349087483	0.353835472	-0.986581366	0.323847909	0.975412329	ENSG00000254893	AC113404.3
108.7648417	-0.22698979	0.482417675	-0.470525444	0.63797966	0.996553586	ENSG00000105516	DBP
111.9542659	-0.014792735	0.307969378	-0.048033138	0.961689833	0.999527751	ENSG00000162971	TYW5
111.7564014	-0.047325921	0.336619479	-0.140591749	0.888192467	0.999467288	ENSG00000165684	SNAPC4
111.2547895	-0.413892439	0.313921654	-1.318457758	0.187350457	0.949094286	ENSG00000010361	FUZ
112.1399642	0.079324172	0.382616741	0.20732018	0.835759815	0.999467288	ENSG00000135315	CEP162
111.0181471	-0.039901495	0.435989011	-0.091519498	0.927079814	0.999527751	ENSG00000146858	ZC3HAV1L
109.3526456	-0.102091045	0.409377105	-0.249381422	0.803065754	0.999467288	ENSG00000168061	SAC3D1
110.1383653	-0.289287809	0.305736182	-0.946200764	0.344046197	0.977691382	ENSG00000176422	SPRYD4
112.6873472	0.221174423	0.394665392	0.560409976	0.575199829	0.99483168	ENSG00000152193	RNF219
110.2065263	0.497056655	0.633516615	0.78459924	0.432688576	0.991301167	ENSG00000077152	UBE2T
111.5453256	-0.36219916	0.531040598	-0.6820555	0.495203857	0.993513219	ENSG00000155816	FMN2
111.5504378	0.554728801	0.392209022	1.414370323	0.157253199	0.939464191	ENSG00000138092	CENPO
108.0930724	-0.164604726	0.493789439	-0.333350034	0.738870075	0.999467288	ENSG00000154917	RAB6B
110.2711097	0.298139492	0.378056893	0.788610119	0.430339915	0.991301167	ENSG00000145375	SPATA5
111.1318935	-2.210586432	0.756858241	-2.920740387	0.003492007	0.42382399	ENSG00000223764	LINC02593
111.1454735	-0.235515358	0.335002668	-0.703025321	0.48203997	0.991525194	ENSG00000179611	DGKZP1
108.7087477	0.499697308	0.38990517	1.281586771	0.199987643	0.954310328	ENSG00000177542	SLC25A22
110.7166137	0.216846888	0.296476104	0.731414388	0.46452608	0.99135833	ENSG00000095066	HOOK2
111.231717	-0.313014143	0.423741517	-0.738691232	0.460094511	0.99135833	ENSG00000110063	DCPS
109.6021154	0.216630401	0.482533503	0.448943752	0.653472233	0.997527562	ENSG00000167972	ABCA3
112.1405387	0.297147863	0.48865635	0.608091685	0.543126667	0.99483168	ENSG00000179859	RNF227
110.7911687	-0.179830703	0.480357982	-0.374368096	0.708130476	0.99900742	ENSG00000101017	CD40
111.2701421	0.33035839	0.407927488	0.809845865	0.418028768	0.990226751	ENSG00000109576	AADAT
111.1253382	-0.373815352	0.355756643	-1.05076141	0.293368184	0.970706863	ENSG00000227615	AP001324.1
110.6091759	0.146034668	0.319709727	0.456772677	0.647834447	0.997368416	ENSG00000168803	ADAL
110.3964061	0.254366454	0.333495736	0.762727757	0.445625773	0.99135833	ENSG00000169612	RAMAC
110.7014089	-0.177447702	0.346947487	-0.511454064	0.609033146	0.99483168	ENSG00000105538	RASIP1
108.8203827	0.170156403	0.463521863	0.367094666	0.713548406	0.99900742	ENSG00000165501	LRR1
108.5560053	-0.447890361	0.449366472	-0.996715129	0.318902804	0.974768742	ENSG00000167642	SPINT2
108.8781992	-0.5247581	0.42890047	-1.223496211	0.221142334	0.956884001	ENSG00000231312	AC007388.1
109.8430055	-0.360135299	0.347622575	-1.03599514	0.30020441	0.972186492	ENSG00000183161	FANCF
116.7535207	-0.461526589	0.750173841	-0.615226183	0.538405359	0.99483168	ENSG00000119714	GPR68
109.3439112	-0.37840153	0.328951319	-1.150326836	0.250009282	0.962465655	ENSG00000189046	ALKBH2
107.8924803	0.47304331	0.489908676	0.96557447	0.334257156	0.976026345	ENSG00000205364	MT1M
108.2736599	-0.227608936	0.438628357	-0.518910674	0.603823034	0.99483168	ENSG00000169683	LRRC45
112.7408176	-0.600924201	0.474868645	-1.265453526	0.205708798	0.954310328	ENSG00000114315	HES1
107.4846048	0.385459633	0.39989079	0.963912254	0.33508992	0.976928484	ENSG00000204516	MICB
109.5342801	-0.175188841	0.371767197	-0.471232649	0.637474606	0.99653524	ENSG00000171033	PKIA
108.6835082	-0.192218536	0.371387505	-0.517568669	0.604759247	0.99483168	ENSG00000184428	TOP1MT
110.740705	2.694206587	1.478829407	1.821850833	0.068477622	0.87420149	ENSG00000137812	KNL1
109.5618619	0.426303407	0.320554034	1.329895622	0.183552664	0.949094286	ENSG00000109881	CCDC34
104.4769532	0.037279088	0.691326204	0.05392402	0.9569957	0.999527751	ENSG00000132821	VSTM2L
107.4672159	0.31728838	0.520946836	0.609060959	0.542484034	0.99483168	ENSG00000100307	CBX7
108.0351089	0.644421126	0.438527442	1.469511514	0.141694101	0.935644597	ENSG00000120334	CENPL
108.7203571	-0.279597316	0.439002523	-0.636892275	0.524195015	0.99483168	ENSG00000122386	ZNF205
107.8218808	0.219571771	0.385819321	0.569105172	0.569284769	0.99483168	ENSG00000186654	PRR5
110.6522439	0.464609304	0.370650703	1.25349635	0.210025126	0.954310328	ENSG00000100483	VCPKMT
108.7711889	0.305880285	0.357212871	0.856296932	0.391833558	0.988378925	ENSG00000171723	GPHN
107.5688102	0.005613609	0.482454222	0.011635526	0.990716403	0.999527751	ENSG00000088756	ARHGAP28
109.9537639	0.43973423	0.424587255	1.035674589	0.300353981	0.972186492	ENSG00000188321	ZNF559
109.1993049	-2.038098117	0.728056488	-2.799368112	0.005120273	0.483347066	ENSG00000167749	KLK4

107.643905	0.159176095	0.361844055	0.439902474	0.660007744	0.997568624	ENSG00000180902	D2HGDH
109.0570317	0.207245247	0.379367048	0.546292167	0.584865119	0.99483168	ENSG00000178814	OPLAH
108.0416137	0.722112062	0.731692493	0.986906479	0.323688488	0.975412329	ENSG00000139354	GAS2L3
110.6198366	2.561683003	1.382000115	1.853605492	0.063795621	0.868202292	ENSG00000122966	CIT
109.6392429	0.311674195	0.314692113	0.990409933	0.321973792	0.975337095	ENSG00000180667	YOD1
109.5361146	-0.595221422	0.404554919	-1.471299431	0.141210157	0.935644597	ENSG00000235217	TSPY26P
107.6914635	0.009287271	0.392053731	0.02368877	0.981100864	0.999527751	ENSG00000137274	BPHL
108.2557919	-0.405364206	0.381606719	-1.062256467	0.288119272	0.966288987	ENSG00000186493	C5orf38
107.8340311	0.192021944	0.409496337	0.46892225	0.639125211	0.996553586	ENSG00000112964	GHR
108.1404068	0.195981888	0.321727558	0.609154805	0.542421834	0.99483168	ENSG00000156170	NDUFAF6
110.4648256	0.419295724	0.644573471	0.650501057	0.515368619	0.993513219	ENSG00000228589	SPCS2P4
107.3938952	0.09730354	0.382695715	0.254258243	0.799296057	0.999467288	ENSG00000164053	ATRIP
108.2488524	-0.033699762	0.355290763	-0.09485122	0.924433002	0.999527751	ENSG00000110060	PUS3
106.9950357	-0.153992122	0.730542923	-0.21079134	0.833050096	0.999467288	ENSG00000139629	GALNT6
109.3863423	-0.158670942	0.499321588	-0.317773046	0.750657098	0.999467288	ENSG00000180190	TDRP
107.3416502	-0.252148927	0.45858262	-0.549844055	0.582426339	0.99483168	ENSG00000205155	PSENEN
108.4302251	0.606551365	0.585303735	1.036301886	0.300061326	0.972186492	ENSG00000006468	ETV1
107.4445473	-0.198697684	0.348454687	-0.570225315	0.568524889	0.99483168	ENSG00000270170	NCBP2-AS2
108.71415	0.191377588	0.323499124	0.591586109	0.554127778	0.99483168	ENSG00000221909	FAM200A
108.7513607	-0.461582932	0.409211349	-1.127981745	0.259327629	0.962465655	ENSG00000198353	HOXC4
109.8096296	-0.911563632	1.47574799	-0.617696001	0.536775748	0.99483168	ENSG00000187260	WDR86
107.9521612	0.361345842	0.334254061	1.081051464	0.279674222	0.963267117	ENSG00000188158	NHS
106.8001443	0.429666875	0.418105208	1.027652531	0.304113304	0.973427483	ENSG00000073111	MCM2
104.8776076	0.026112404	0.545617182	0.047858472	0.961829036	0.999527751	ENSG00000260604	AL590004.3
105.6660166	1.311034524	0.480991683	2.725690631	0.006416711	0.516907781	ENSG00000138675	FGF5
107.4546797	-0.239262873	0.403869216	-0.592426617	0.553564947	0.99483168	ENSG00000249353	NPM1P27
108.0342933	0.735628182	0.468902189	1.568830771	0.116687376	0.923922314	ENSG00000006634	DBF4
107.8875477	-0.505912476	0.430235876	-1.175895606	0.239636595	0.962465655	ENSG00000173818	ENDOV
107.5107381	-0.248488153	0.365996866	-0.678935194	0.497178925	0.993513219	ENSG00000204152	TIMM23B
109.0155487	0.196612394	0.376724075	0.521900264	0.601739775	0.99483168	ENSG00000167232	ZNF91
106.2067508	-0.342712684	0.380061114	-0.901730462	0.367200067	0.981269214	ENSG00000205659	LIN52
106.9756058	0.381567852	0.319402078	1.194631714	0.232230953	0.959575179	ENSG00000141994	DUS3L
106.9269469	0.535643377	0.492125977	1.088427357	0.276406489	0.962465655	ENSG00000196110	ZNF699
106.4592136	0.458710486	0.396685159	1.156359083	0.247534311	0.962465655	ENSG00000151876	FBXO4
109.9112286	0.419777346	0.749072455	0.560396186	0.575209233	0.99483168	ENSG00000164850	GPER1
107.3436431	0.713269842	0.441707167	1.61480251	0.106353469	0.922165836	ENSG00000196876	SCN8A
105.5549788	-0.067979943	0.45746409	-0.148601703	0.88186793	0.999467288	ENSG00000144891	AGTR1
106.2840461	-0.552608577	0.381888904	-1.44704015	0.147885679	0.936396974	ENSG00000182165	TP53TG1
108.4249712	0.32130975	0.469574522	0.684257204	0.493812766	0.993513219	ENSG00000154589	LY96
107.5443909	-0.230566678	0.322783015	-0.714308581	0.475036387	0.99135833	ENSG00000185379	RAD51D
107.0788926	0.273468294	0.528160787	0.517774703	0.604615471	0.99483168	ENSG00000151466	SCLT1
106.6964262	0.545980508	0.540841637	1.009501617	0.312734127	0.973427483	ENSG00000112796	ENPP5
105.5701048	0.4535433	0.388247504	1.168180853	0.242733822	0.962465655	ENSG00000198598	MMP17
108.4188189	0.555073382	0.353537736	1.570054126	0.11640252	0.923922314	ENSG00000276234	TADA2A
108.9815601	-0.144733217	0.416773535	-0.347270651	0.728387997	0.999467288	ENSG00000197363	ZNF517
107.3651831	-0.019640903	0.379216245	-0.051793413	0.958693304	0.999527751	ENSG00000171428	NAT1
106.1747689	-1.156032906	0.342000576	-3.380207483	0.000724311	0.201177461	ENSG00000067836	ROGDI
109.0634931	0.797078641	0.579452664	1.375571622	0.168954297	0.943792179	ENSG00000074211	PPP2R2C
105.3522646	0.109657504	0.358734549	0.305678683	0.759849305	0.999467288	ENSG00000127191	TRAF2
107.6060751	-0.136715802	0.35806532	-0.381818051	0.702596329	0.998869317	ENSG00000197782	ZNF780A
105.2335905	-0.210042049	0.378208553	-0.555360389	0.578648181	0.99483168	ENSG00000151014	NOCT
104.8932085	0.484781363	0.465292005	1.041886295	0.297464399	0.972186492	ENSG00000152229	PSTPIP2
106.1636795	0.293455756	0.388038078	0.75625505	0.449496293	0.99135833	ENSG00000134262	AP4B1
105.5437047	-0.275497479	0.32683382	-0.842928308	0.399268541	0.988378925	ENSG00000163155	LYSMD1
108.0783775	-0.680221559	0.539724451	-1.26031266	0.207556595	0.954310328	ENSG00000179715	PCED1B
105.7228881	-0.035256524	0.337654698	-0.104415913	0.916839295	0.999527751	ENSG00000089123	TASP1
105.1110406	0.97972441	0.376624963	2.601326267	0.009286409	0.618081117	ENSG00000116761	CTH
105.4133721	-0.416169004	0.312350097	-1.332379941	0.18273537	0.949094286	ENSG00000066827	ZFAT
109.8689957	-1.364459947	1.247136945	-1.094073872	0.273922581	0.962465655	ENSG00000227619	AL391056.1
108.9809684	0.320021043	0.516050828	0.620134733	0.535169087	0.99483168	ENSG00000166016	ABTB2
107.0879356	1.668160262	1.532274396	1.088682462	0.276293938	0.962465655	ENSG00000156970	BUB1B
105.8771232	-0.014875949	0.314360085	-0.047321369	0.962257098	0.999527751	ENSG00000197302	ZNF720
106.464163	0.138507974	0.338446599	0.40924617	0.682359015	0.998869317	ENSG00000186017	ZNF566
105.8111217	0.159991251	0.322462972	0.496153867	0.619785856	0.99483168	ENSG00000076650	GPATCH1

108.0282007	-0.584237584	0.435064042	-1.342877205	0.179311739	0.9469453	ENSG00000133433	GSTT2B
106.2182706	-0.924919976	0.329068719	-2.810719834	0.004943081	0.472943531	ENSG00000158352	SHROOM4
104.1340908	0.048494302	0.328508537	0.147619611	0.882642978	0.999467288	ENSG00000148339	SLC25A25
106.0023047	0.614231661	0.397985576	1.543351564	0.122745499	0.926234713	ENSG00000183426	NPIPA1
103.7609893	-0.649502551	0.438119183	-1.482479143	0.138212849	0.934144427	ENSG00000167100	SAMD14
103.7184796	-1.138160959	0.458274006	-2.483581753	0.013006847	0.667111125	ENSG00000127472	PLA2G5
109.7384164	-1.340964508	0.509740193	-2.630682312	0.008521365	0.604581883	ENSG00000260428	SCX
101.0948418	-1.517182348	0.435494547	-3.483814796	0.000494321	0.1830637	ENSG00000090382	LYZ
103.6398612	0.062578062	0.408249282	0.153283948	0.878174358	0.999467288	ENSG00000104856	RELB
105.9981548	0.366255973	0.506875274	0.722576129	0.469940338	0.99135833	ENSG00000197646	PDCD1LG2
105.2970312	-0.255189515	0.368765528	-0.692010223	0.488930909	0.993513219	ENSG00000162300	ZFPL1
102.0382501	0.527004912	0.568398034	0.927175818	0.353835256	0.977691382	ENSG00000185745	IFIT1
104.852631	2.511624058	1.453209146	1.728329376	0.083929189	0.901307093	ENSG00000129195	PIMREG
103.6606709	0.386448638	0.603151084	0.640716146	0.521707122	0.993980889	ENSG00000109084	TMEM97
104.6832971	-0.343333473	0.360666409	-0.951941919	0.341126439	0.977691382	ENSG00000160226	CFAP410
104.3039923	-0.099097024	0.401728111	-0.246676847	0.805158326	0.999467288	ENSG00000065717	TLE2
105.1499418	0.27438059	0.327268421	0.838396169	0.40180824	0.988592117	ENSG00000159445	THEM4
105.6825675	-0.236382053	0.433633484	-0.545119466	0.585671355	0.99483168	ENSG00000111684	LPCAT3
103.858066	0.32220801	0.330138215	0.975979137	0.329074803	0.976026345	ENSG00000204564	C6orf136
105.0052789	2.461451845	1.421204681	1.731947466	0.083282916	0.901307093	ENSG00000237649	KIFC1
105.9012228	0.336266388	0.400390119	0.839846868	0.400994251	0.988492517	ENSG00000186088	GSAP
105.1568005	0.583876209	0.572416166	1.020020475	0.30771875	0.973427483	ENSG00000178295	GEN1
104.8422808	-0.021160358	0.331998933	-0.063736223	0.949180261	0.999527751	ENSG00000154957	ZNF18
103.9649203	-0.399112126	0.347403807	-1.14884212	0.250621088	0.962465655	ENSG00000100319	ZMAT5
103.6437366	-0.200166876	0.377733841	-0.529915126	0.596170778	0.99483168	ENSG00000143772	ITPKB
105.0426846	1.18214649	0.465177285	2.541281632	0.011044691	0.644117389	ENSG00000112029	FBXO5
105.0454992	0.182945992	0.312475646	0.585472803	0.558229849	0.99483168	ENSG00000250462	LRRC37BP1
104.2562768	0.309956931	0.351304722	0.882302207	0.377613403	0.982609538	ENSG00000125846	ZNF133
101.6947338	-0.026259267	0.593937221	-0.044212193	0.964735263	0.999527751	ENSG00000164318	EGFLAM
103.9316033	-0.490286291	0.395625056	-1.239270068	0.215245499	0.954310328	ENSG00000168060	NAALADL1
105.4677008	0.366451203	0.376799839	0.972535454	0.330784236	0.976026345	ENSG00000166822	TMEM170A
104.4467797	0.184346603	0.478435899	0.385310976	0.700007031	0.998869317	ENSG00000157557	ETS2
104.7940966	-0.136119792	0.374825965	-0.363154649	0.716489363	0.99900742	ENSG00000188002	AC026412.1
103.0427495	1.363401287	0.421916381	3.231449046	0.001231643	0.247064127	ENSG00000187720	THSD4
104.7055687	-0.11582652	0.315287389	-0.367368072	0.713344484	0.99900742	ENSG00000168876	ANKRD49
105.04948	-0.121392486	0.396537665	-0.306131036	0.759504878	0.999467288	ENSG00000196151	WDSUB1
105.2942649	-0.36260016	0.52424206	-0.691665527	0.489147401	0.993513219	ENSG00000136274	NACAD
104.3058466	-0.085498225	0.490549524	-0.174290709	0.861637	0.999467288	ENSG00000232024	LSM12P1
101.7423118	-0.344356605	0.597787987	-0.576051396	0.564580443	0.99483168	ENSG00000176532	PRR15
101.5866743	-0.19041718	0.458748541	-0.415079641	0.678083603	0.998869317	ENSG00000205138	SDHAF1
103.5993309	-0.392748576	0.38480637	-1.020639487	0.307425274	0.973427483	ENSG00000135835	KIAA1614
104.7134577	-0.375341913	0.438532467	-0.855904502	0.392050605	0.988378925	ENSG00000154153	RETREG1
104.1495826	-0.033364343	0.36747407	-0.090793734	0.927656489	0.999527751	ENSG00000154309	DISP1
103.0211683	0.724223132	0.357351611	2.026640173	0.042699225	0.806698895	ENSG00000181381	DDX60L
102.9159761	0.702918512	0.648160111	1.084482832	0.278150775	0.962465655	ENSG00000125885	MCM8
104.5215797	0.794325205	0.389323067	2.040272647	0.041323178	0.800562177	ENSG00000187953	PMS2CL
103.1504501	0.722442155	0.319454346	2.261487951	0.023729058	0.744948012	ENSG00000107890	ANKRD26
104.8297699	-0.375083306	0.342126577	-1.09632905	0.272934801	0.962465655	ENSG00000188171	ZNF626
102.3920354	-0.120695666	0.384580032	-0.31383758	0.753644401	0.999467288	ENSG00000113272	THG1L
103.9308725	0.501758735	0.31538298	1.590950578	0.111620699	0.923922314	ENSG00000196295	GARS-DT
103.1949807	0.084284982	0.31581182	0.266883558	0.789558817	0.999467288	ENSG00000116205	TCEANC2
103.2331361	0.906582043	0.409854933	2.211958355	0.026969543	0.765286378	ENSG00000113805	CNTN3
103.7455148	-0.436525542	0.347730698	-1.255355205	0.209349843	0.954310328	ENSG00000165698	SPACA9
101.8457993	-0.034255314	0.492921889	-0.069494406	0.944596086	0.999527751	ENSG00000121690	DEPDC7
102.5312805	-1.446364558	0.799719452	-1.808589944	0.070514732	0.87954613	ENSG00000270885	RASL10B
103.7475289	-0.087591914	0.369580333	-0.23700372	0.812653905	0.999467288	ENSG00000177051	FBXO46
98.90736504	1.239999152	0.735133007	1.686768436	0.091647887	0.916034901	ENSG00000185274	GALNT17
103.9318806	0.360780235	0.4487422	0.803981073	0.421407906	0.991301167	ENSG00000125351	UPF3B
102.9892962	0.740819568	0.358416874	2.066921569	0.038741545	0.793985738	ENSG00000173611	SCAI
104.8656664	0.135586191	0.605371854	0.223971746	0.822779288	0.999467288	ENSG00000054967	RELT
102.740915	0.238479633	0.334446496	0.713057651	0.475810083	0.99135833	ENSG00000225137	DYNC1I2P1
103.1903584	0.042876042	0.380129256	0.112793324	0.910194411	0.999527751	ENSG00000097096	SYDE2
103.9635496	-0.147827621	0.39139984	-0.377689529	0.705661247	0.99900742	ENSG00000196878	LAMB3
103.0123767	-0.570467305	0.396944452	-1.437146438	0.150676388	0.936396974	ENSG00000182518	FAM104B

103.3271958	0.649381561	0.322688516	2.012409892	0.044176747	0.807325344	ENSG00000147996	CBWD5	
104.816765	0.37846259	0.398684561	0.949278271	0.342479102	0.977691382	ENSG00000188283	ZNF383	
103.1731698	-0.311130303	0.381328189	-0.815912151	0.414550382	0.990226751	ENSG00000142961	MOB3C	
103.5370942	0.395727959	0.425619028	0.929770366	0.352489993	0.977691382	ENSG00000180758	GPR157	
105.0127948	0.114835689	0.401843143	0.285772423	0.775052431	0.999467288	ENSG00000175087	PDIK1L	
102.3555862	0.44852708	0.380583099	1.178526009	0.23858697	0.962465655	ENSG00000119227	PIGZ	
103.299486	-0.301630144	0.385014596	-0.783425217	0.433377453	0.991301167	ENSG00000146414	SHPRH	
103.4050693	0.587801773	0.355729641	1.652383454	0.098456406	0.921147998	ENSG00000132793	LPIN3	
100.1692075	0.568119994	0.440620574	1.289363294	0.197271816	0.954310328	ENSG00000232956	SNHG15	
102.9086676	-0.238456758	0.342193092	-0.696848544	0.485897583	0.992923464	ENSG00000238103	RPL9P7	
101.8981253	0.45709552	0.386370812	1.183048785	0.236789813	0.962465655	ENSG00000185386	MAPK11	
102.1962016	-0.940831961	0.429739555	-2.189307336	0.02857451	0.770696352	ENSG00000156384	SFR1	
101.8919546	0.384253394	0.379924782	1.011393341	0.311828207	0.973427483	ENSG00000142794	NBPF3	
103.9211571	1.764163857	1.365600236	1.29185966	0.19640575	0.954310328	ENSG00000112742	TTK	
103.867149	0.084240739	0.397490307	0.211931555	0.832160432	0.999467288	ENSG00000188566	NDOR1	
102.4729145	0.075250601	0.321638837	0.233959935	0.815016083	0.999467288	ENSG00000141499	WRAP53	
102.9043336	-0.056061034	0.324727284	-0.172640356	0.862934128	0.999467288	ENSG00000162775	RBM15	
103.078897	0.127966192	0.330817432	0.386818165	0.698890828	0.998869317	ENSG00000000457	SCYL3	
102.8582731	-0.3973171	0.396793774	-1.001318886	0.316672665	0.974768742	ENSG00000240024	LINC00888	
100.0694392	-0.172591691	0.533967109	-0.323225323	0.746524605	0.999467288	ENSG00000198483	ANKRD35	
100.8710422	-0.080102002	0.353587728	-0.226540674	0.820780928	0.999467288	ENSG00000184924	PTRHD1	
102.7257666	0.240433894	0.39324599	0.611408381	0.540929256	0.99483168	ENSG00000204271	SPIN3	
101.8888956	0.140601627	0.411012311	0.342086168	0.732286047	0.999467288	ENSG00000175866	BAIAP2	
102.3786986	-0.168596803	0.35762073	-0.471440241	0.637326385	0.99653524	ENSG00000160284	SPATC1L	
102.3748229	-0.268065598	0.316616528	-0.846656994	0.397186336	0.988378925	ENSG00000124523	SIRT5	
102.4098235	-0.355199246	0.376391756	-0.943695605	0.34532522	0.977691382	ENSG00000071246	VASH1	
101.5875194	1.384547346	0.612510314	2.2604474	0.023793498	0.744948012	ENSG00000012048	BRCA1	
102.0904195	0.270707053	0.326821047	0.82830361	0.407498579	0.990226751	ENSG00000120784	ZFP30	
100.7995011	-0.308893662	0.412535672	-0.748768368	0.453996828	0.99135833	ENSG00000158792	SPATA2L	
99.97992395	0.157857454	0.499956629	0.315742296	0.75219812	0.999467288	ENSG00000121104	FAM117A	
98.40698972	0.016018637	0.55789941	0.02871241	0.977093959	0.999527751	ENSG00000175315	CST6	
101.2759306	-0.415552712	0.381314384	-1.089790287	0.275805534	0.962465655	ENSG00000183621	ZNF438	
101.5908939	0.22280874	0.381768741	0.583622272	0.559474473	0.99483168	ENSG00000179588	ZFPM1	
102.5097238	-0.463585148	0.36808899	-1.259437691	0.207872283	0.954310328	ENSG00000204599	TRIM39	
102.4192385	2.426112635	1.50849263	1.60830261	0.107768919	0.923922314	ENSG00000169607	CKAP2L	
100.0390412	-0.316105843	0.433930573	-0.728471011	0.466325308	0.99135833	ENSG00000154079	SDHAF4	
98.19495878	1.27589447	0.596461077	2.139107678	0.032426949	0.775963115	ENSG00000145506	NKD2	
101.7921039	0.330770266	0.322763759	1.024806091	0.305454692	0.973427483	ENSG00000063761	ADCK1	
102.7434255	0.473085529	0.641328121	0.737665344	0.460717837	0.99135833	ENSG00000108852	MPP2	
101.5679589	-0.354956707	0.345073243	-1.028641642	0.303648102	0.973213238	ENSG00000198093	ZNF649	
102.0298496	0.034494761	0.372955387	0.092490314	0.926308487	0.999527751	ENSG00000127989	MTERF1	
101.5303176	-0.55800252	0.395103916	-1.412293062	0.157863688	0.939464191	ENSG00000229809	ZNF688	
100.2784953	-0.099774111	0.419627032	-0.237768551	0.812060612	0.999467288	ENSG00000167733	HSD11B1L	
102.2847399	0.207945776	0.413820287	0.502502614	0.615314014	0.99483168	ENSG00000121406	ZNF549	
101.1537903	-0.054151534	0.331573163	-0.163316999	0.870268851	0.999467288	ENSG00000157184	CPT2	
100.8969831	0.268717311	0.31940923	0.841294759	0.400182826	0.988492517	ENSG00000117791		2-Mar
100.5599798	-0.516701757	0.440221117	-1.173732329	0.240502257	0.962465655	ENSG00000197905	TEAD4	
101.0649562	0.341501321	0.348702301	0.979349204	0.327407466	0.976026345	ENSG00000205808	PLPP6	
101.6855858	-0.52591952	0.332701957	-1.580752708	0.113934592	0.923922314	ENSG00000138738	PRDM5	
100.765494	-0.804056606	0.365200227	-2.20168704	0.027687423	0.769167109	ENSG00000160993	ALKBH4	
101.688062	0.095408477	0.344194865	0.277193203	0.781631759	0.999467288	ENSG00000111266	DUSP16	
99.14817681	0.197822365	0.444598437	0.444946154	0.656358668	0.997568624	ENSG00000167363	FN3K	
103.9174794	-0.476597478	0.532047694	-0.895779614	0.37037048	0.981455968	ENSG00000125347	IRF1	
99.63689745	-0.110066977	0.378970819	-0.290436548	0.771482286	0.999467288	ENSG00000141933	TPGS1	
100.6367621	0.43905005	0.531654795	0.825817906	0.408907395	0.990226751	ENSG00000196440	ARMCX4	
101.2211408	0.007179437	0.37993636	0.01889642	0.984923736	0.999527751	ENSG00000099204	ABLIM1	
99.71882291	-0.069507103	0.376914256	-0.184410917	0.853691107	0.999467288	ENSG00000170270	GON7	
99.89320644	0.19164603	0.40904794	0.468517284	0.639414714	0.996553586	ENSG00000113790	EHHADH	
100.990033	0.017738375	0.342497581	0.051791241	0.958695035	0.999527751	ENSG00000106077	ABHD11	
101.022077	-0.543962849	0.330757139	-1.644598967	0.10005254	0.92169898	ENSG00000224861	YBX1P1	
99.48196946	0.765858237	0.4633947	1.652712553	0.098389378	0.921147998	ENSG00000154027	AK5	
101.45472	0.338226706	0.394116806	0.858188996	0.390788111	0.987833983	ENSG00000164074	ABHD18	
101.1023698	-0.012930271	0.313325493	-0.041267854	0.96708236	0.999527751	ENSG00000096654	ZNF184	
99.82919353	0.50871153	0.360277061	1.412000888	0.1579497	0.939464191	ENSG00000172840	PDP2	

100.5523878	-0.105833289	0.315727665	-0.335204356	0.737470936	0.999467288	ENSG00000169981	ZNF35
99.80551318	-0.154461566	0.416777387	-0.370609276	0.710928572	0.99900742	ENSG00000148655	LRMDA
101.0522443	-0.003867074	0.334207623	-0.011570874	0.990767985	0.999527751	ENSG00000105750	ZNF85
98.15915595	0.39567933	0.527272432	0.750426737	0.452997733	0.99135833	ENSG00000150556	LYPD6B
102.4190582	0.831438997	0.453370562	1.833906008	0.066667948	0.87420149	ENSG00000267519	AC020916.1
99.93866449	0.005221593	0.391558244	0.013335419	0.989360191	0.999527751	ENSG00000224578	HNRNPA1P48
100.4779979	0.194840235	0.370734505	0.525551932	0.599199562	0.99483168	ENSG00000122435	TRMT13
100.376117	-0.396226282	0.33593636	-1.179468284	0.238211759	0.962465655	ENSG00000171861	MRM3
101.0066469	-0.289212507	0.345264222	-0.837655595	0.402224159	0.988592117	ENSG00000163874	ZC3H12A
99.48693741	-0.41691114	0.325705176	-1.280026141	0.200535942	0.954310328	ENSG00000119636	BBOF1
100.7014911	0.184472038	0.377779874	0.488305627	0.625333377	0.99483168	ENSG00000196437	ZNF569
100.2328752	0.37357844	0.425226199	0.878540506	0.379650466	0.983373687	ENSG00000124802	EEF1E1
100.9290304	0.421058638	0.410776646	1.025030616	0.305348742	0.973427483	ENSG00000198753	PLXNB3
100.0305213	-0.178917436	0.315945378	-0.566292305	0.571195091	0.99483168	ENSG00000140006	WDR89
98.49341527	-0.403815257	0.560071021	-0.721007232	0.470905065	0.99135833	ENSG00000124343	XG
97.82033587	-0.229819455	0.422705337	-0.543687138	0.586656785	0.99483168	ENSG00000161920	MED11
99.42896462	0.77399362	0.472176744	1.639203177	0.101170952	0.92169898	ENSG00000117569	PTBP2
97.98867568	-0.580987253	0.539753124	-1.076394424	0.281750895	0.963267117	ENSG00000206341	HLA-H
99.99136396	2.007523067	1.387295219	1.447077045	0.147875346	0.936396974	ENSG00000100526	CDKN3
99.28058841	-0.073020288	0.359132431	-0.203324128	0.838881698	0.999467288	ENSG00000182810	DDX28
99.47797897	0.00819237	0.337190453	0.024295973	0.980616525	0.999527751	ENSG00000113734	BNIP1
98.73922213	0.079778998	0.348929448	0.228639337	0.819149248	0.999467288	ENSG00000162139	NEU3
99.57616237	1.786469934	0.681416031	2.621702239	0.008749183	0.604581883	ENSG00000214548	MEG3
98.61750075	-0.163653756	0.442266575	-0.370034194	0.711357012	0.99900742	ENSG00000176974	SHMT1
99.71993977	-0.68495678	0.417189846	-1.641834732	0.100624259	0.92169898	ENSG00000198746	GPATCH3
98.61449709	0.196225856	0.399112899	0.49165501	0.622963248	0.99483168	ENSG00000171960	PPIH
98.1540556	0.322774598	0.39986525	0.807208423	0.419546414	0.990226751	ENSG00000092208	GEMIN2
100.6161824	0.148801442	0.388717378	0.382801106	0.701867241	0.998869317	ENSG00000107443	CCNJ
99.68039855	0.533965873	0.414711491	1.287559871	0.197899216	0.954310328	ENSG00000275131	AC241952.1
103.5222446	-2.114035975	1.204500747	-1.755113876	0.079239831	0.897052166	ENSG00000188338	SLC38A3
97.66858113	0.106141234	0.479989276	0.221132511	0.824989256	0.999467288	ENSG00000170542	SERPINB9
99.13568641	-0.216461675	0.330387957	-0.655174228	0.512355585	0.993513219	ENSG00000214174	AMZ2P1
100.2514534	-0.124402298	0.431380249	-0.288381997	0.773054347	0.999467288	ENSG00000132849	PATJ
96.29360443	0.53615268	0.639148316	0.838854874	0.401550754	0.988592117	ENSG00000170293	CMTM8
97.83225014	-0.136005392	0.512715991	-0.26526458	0.790805649	0.999467288	ENSG00000141448	GATA6
98.5570287	0.030514791	0.439645634	0.069407698	0.944665101	0.999527751	ENSG00000101546	RBFA
98.42631553	-0.405644072	0.360810625	-1.124257556	0.260903779	0.962465655	ENSG00000182362	YBEY
100.038764	0.148003089	0.398106243	0.371767817	0.710065727	0.99900742	ENSG00000186665	C17orf58
98.50491419	-0.397249443	0.356281982	-1.114986059	0.264856396	0.962465655	ENSG00000132004	FBXW9
98.70625765	0.152172574	0.351159246	0.433343491	0.664765247	0.997568624	ENSG00000042317	SPATA7
100.000673	0.232746341	0.433569617	0.536814233	0.591395939	0.99483168	ENSG00000166261	ZNF202
97.84061458	-0.049575508	0.346513858	-0.143069337	0.886235421	0.999467288	ENSG00000150456	EEF1AKMT1
98.45009175	-0.022399463	0.493660558	-0.045374221	0.963809029	0.999527751	ENSG00000136982	DSCC1
96.47229865	-0.742387951	0.780195126	-0.951541385	0.34132962	0.977691382	ENSG00000103485	QPRT
96.89235624	-0.684630764	0.455875085	-1.501794649	0.133150151	0.933741583	ENSG00000152137	HSPB8
97.20822221	-0.903059174	0.498098954	-1.813011584	0.069830035	0.878035218	ENSG00000133107	TRPC4
98.48312803	-0.426821904	0.332700597	-1.282900926	0.199526788	0.954310328	ENSG00000166133	RPUSD2
97.77308372	0.168589173	0.348244978	0.484110852	0.6283072	0.99483168	ENSG00000197619	ZNF615
97.03015981	-0.299488807	0.497572369	-0.601899996	0.547240707	0.99483168	ENSG00000099889	ARVCF
101.1932058	1.232080888	0.76171606	1.617506776	0.105768934	0.921919564	ENSG00000275895	U2AF1L5
99.45562954	-0.284404286	0.564359844	-0.503941394	0.614302561	0.99483168	ENSG00000226415	TPI1P1
91.95750358	1.425426114	0.840237472	1.696456252	0.089799508	0.910413547	ENSG00000158270	COLEC12
96.72583122	-0.526064012	0.346778351	-1.517003618	0.129265771	0.928627271	ENSG00000174177	CTU2
96.22525913	-0.64303247	0.441998697	-1.454828884	0.14571664	0.935644597	ENSG00000003096	KLHL13
97.3654179	0.194304503	0.401714881	0.483687592	0.628607601	0.99483168	ENSG00000175806	MSRA
96.99879749	0.440508578	0.466356505	0.944574748	0.344876024	0.977691382	ENSG00000010278	CD9
98.7485361	0.995116057	0.670473494	1.484198952	0.137756148	0.934144427	ENSG00000162063	CCNF
96.63044237	-0.355550219	0.364989561	-0.974138049	0.329987999	0.976026345	ENSG00000227496	AC099066.2
98.1924904	0.032362224	0.310899516	0.104092232	0.917096156	0.999527751	ENSG00000106610	STAG3L4
97.34396585	-0.187581761	0.362538435	-0.517412068	0.604868537	0.99483168	ENSG00000103343	ZNF174
97.40071911	0.000594139	0.407158592	0.001459233	0.998835701	0.999527751	ENSG00000019485	PRDM11
97.69648906	0.158326139	0.388121607	0.407929205	0.683325651	0.998869317	ENSG00000267534	S1PR2
97.71551017	-0.071959328	0.401553281	-0.179202441	0.857778747	0.999467288	ENSG00000186073	C15orf41
95.49619543	-0.226501173	0.436723836	-0.518637076	0.604013849	0.99483168	ENSG00000170175	CHRNB1

98.92497999	-0.075575962	0.416815832	-0.181317397	0.856118451	0.999467288	ENSG00000179818	PCBP1-AS1
97.8268642	-0.693696578	0.36318243	-1.910049937	0.056126784	0.847598083	ENSG00000204634	TBC1D8
97.03191379	0.157759805	0.680961804	0.231672032	0.816792756	0.999467288	ENSG00000164687	FABP5
99.02708779	0.115053888	0.36774575	0.312862592	0.754385059	0.999467288	ENSG00000032742	IFT88
97.88837207	-0.352701797	0.403885945	-0.873270787	0.3825155	0.98555814	ENSG00000163093	BBS5
95.91426482	0.128131572	0.439405166	0.291602333	0.770590691	0.999467288	ENSG00000151470	C4orf33
98.07003315	-0.151861351	0.364895935	-0.41617715	0.677280379	0.998869317	ENSG00000165630	PRPF18
90.79724275	0.932484248	1.396191556	0.667877014	0.504212101	0.993513219	ENSG00000162630	B3GALT2
96.97123115	-0.005428642	0.379160934	-0.014317515	0.988576666	0.999527751	ENSG00000186312	CA5BP1
96.83248553	0.450583269	0.341470701	1.319537132	0.186989605	0.949094286	ENSG00000100150	DEPDC5
97.54816525	0.968162627	0.446175824	2.16991279	0.030013453	0.773201806	ENSG00000160072	ATAD3B
98.67221457	1.026628572	0.763794527	1.344116168	0.178910825	0.9469453	ENSG00000143847	PPFIA4
95.72098805	0.404398457	0.393487289	1.027729403	0.304077133	0.973427483	ENSG00000161682	FAM171A2
96.83513835	-0.645086319	0.411513512	-1.567594502	0.116975796	0.923922314	ENSG00000122547	EEPD1
95.26650711	-0.152574759	0.383519919	-0.39782747	0.690757367	0.998869317	ENSG00000275342	PRAG1
96.87724678	-0.045909576	0.359781498	-0.12760405	0.898462326	0.999490224	ENSG00000135049	AGTPBP1
96.99840272	0.130424988	0.349428912	0.37325185	0.708961009	0.99900742	ENSG00000183060	LYSMD4
94.93115657	-0.400983935	0.358566896	-1.118296029	0.263440582	0.962465655	ENSG00000119431	HDHD3
96.6555445	-0.06220461	0.331377179	-0.187715431	0.851099733	0.999467288	ENSG00000152359	POC5
94.8984011	0.494461165	0.444929821	1.111323946	0.266428936	0.962465655	ENSG00000184785	SMIM10
96.28278897	-0.004097191	0.34852756	-0.011755717	0.990620511	0.999527751	ENSG00000153574	RPIA
95.68557506	-0.224057899	0.430146944	-0.520886878	0.602445577	0.99483168	ENSG00000235863	B3GALT4
94.76550646	-0.004231968	0.488092625	-0.008670419	0.993082093	0.999527751	ENSG00000180660	MAB21L1
94.87917115	-0.658772443	0.404886437	-1.627054856	0.103725448	0.92169898	ENSG00000273270	AC090114.2
96.66862141	-0.127476521	0.379248763	-0.336129037	0.736773563	0.999467288	ENSG00000103021	CCDC113
96.83662876	0.312258688	0.367557256	0.849551144	0.395574684	0.988378925	ENSG00000092871	RFFL
95.63899319	0.486644683	0.320376572	1.518977118	0.128768256	0.928627271	ENSG00000152078	TMEM56
95.39748013	-0.357737008	0.354187399	-1.010021838	0.312484827	0.973427483	ENSG00000139239	RPL14P1
96.97702126	0.491044954	0.518353361	0.947317007	0.343477267	0.977691382	ENSG00000166046	TCP11L2
96.76267448	0.054363746	0.366320543	0.148404853	0.88202327	0.999467288	ENSG00000187607	ZNF286A
95.75499037	0.025338524	0.343656341	0.07373216	0.941223508	0.999527751	ENSG00000160783	PMF1
94.81092207	0.20754094	0.415924043	0.4989876	0.61778812	0.99483168	ENSG00000183474	GTF2H2C
97.44123947	0.4452251	0.43053508	1.034120377	0.301079891	0.972793733	ENSG00000110660	SLC35F2
94.72351603	-0.370768226	0.411643464	-0.900702328	0.367746611	0.981269214	ENSG00000243701	DUBR
96.23540474	-0.050672801	0.621791569	-0.081494835	0.935048432	0.999527751	ENSG00000204257	HLA-DMA
98.48515426	0.2907292	0.619840075	0.469039049	0.639041724	0.996553586	ENSG00000169594	BNC1
98.36642143	0.237315769	1.178037028	0.201450178	0.840346583	0.999467288	ENSG00000162373	BEND5
94.26265376	0.042198032	0.37019399	0.113988972	0.909246535	0.999527751	ENSG00000135441	BLOC1S1
94.47506415	-0.395141197	0.47872336	-0.825406132	0.409141055	0.990226751	ENSG00000259721	AC090877.2
97.18355347	0.472111976	0.725243308	0.650970468	0.515065551	0.993513219	ENSG00000261888	AC144831.1
96.705533	0.880364682	0.428406066	2.054977164	0.03988121	0.793985738	ENSG00000215908	CROCCP2
93.62265577	0.021873026	0.494598197	0.04422383	0.964725987	0.999527751	ENSG00000112039	FANCE
97.39183349	0.326784147	0.444484557	0.735197978	0.46221893	0.99135833	ENSG00000255455	AP003486.1
99.06246394	0.075255886	0.535617699	0.140502985	0.888262594	0.999467288	ENSG00000104783	KCNN4
94.56798412	0.759168974	0.451895517	1.679965712	0.092963987	0.921147998	ENSG00000273038	AL365203.2
93.54177375	0.714622025	0.577084648	1.238331373	0.215593215	0.954310328	ENSG00000139209	SLC38A4
94.45586196	-0.128083971	0.400397177	-0.319892294	0.74904998	0.999467288	ENSG00000185187	SIGIRR
94.34942824	0.822099659	0.397084873	2.070337389	0.038420761	0.793985738	ENSG00000279453	Z99129.4
94.45553726	-0.288668938	0.3698661	-0.780468765	0.435115006	0.991301167	ENSG00000141441	GAREM1
96.09271496	0.062986287	0.379097517	0.166147981	0.868040495	0.999467288	ENSG00000260917	AL158212.3
94.29015511	-0.071506302	0.324758478	-0.220183017	0.825728622	0.999467288	ENSG00000077348	EXOSC5
95.47338111	0.251569093	0.379124166	0.663553305	0.506976252	0.993513219	ENSG00000089682	RBM41
94.77452049	-0.193104354	0.555461885	-0.347646453	0.728105716	0.999467288	ENSG00000110900	TSPAN11
93.72807404	-0.427912753	0.367237432	-1.16522096	0.243929563	0.962465655	ENSG00000147804	SLC39A4
95.18446231	0.697127303	0.665288181	1.04785764	0.294704201	0.970706863	ENSG00000133119	RFC3
95.99239872	0.154360483	0.447445216	0.344981861	0.730108007	0.999467288	ENSG00000076351	SLC46A1
95.68918775	0.167470128	0.47099064	0.35556997	0.722162636	0.99906455	ENSG00000105136	ZNF419
93.5363093	0.099300233	0.384746165	0.258092846	0.796335252	0.999467288	ENSG00000169249	ZRSR2
96.21793064	0.371775091	0.457168475	0.813212439	0.416096269	0.990226751	ENSG00000180787	ZFP3
92.97136921	0.521190348	0.380933422	1.368192757	0.171251748	0.944422693	ENSG00000128739	SNRPN
96.94032041	1.181320352	0.725521197	1.628236855	0.103474673	0.92169898	ENSG00000224259	LINC01133
94.10468553	-0.491941111	0.37440435	-1.313930007	0.18886976	0.949094286	ENSG00000123444	KBTBD4
94.74809662	-0.117286936	0.47453959	-0.247159432	0.80478484	0.999467288	ENSG00000171132	PRKCE
93.53723907	0.226051116	0.476277831	0.474620276	0.635057652	0.995506752	ENSG00000121210	TMEM131L

94.41095359	0.349939712	0.3279839	1.066941737	0.285998146	0.964826902	ENSG00000155970	MICU3
93.99776431	-0.192885682	0.326294775	-0.591139351	0.554427055	0.99483168	ENSG00000181135	ZNF707
94.60921784	-0.353424518	0.400745465	-0.881917698	0.377821316	0.982609538	ENSG00000226221	RPL26P19
94.58325185	0.460411856	0.405693471	1.13487618	0.256427202	0.962465655	ENSG00000136379	ABHD17C
94.05928303	0.311247309	0.350916956	0.886954319	0.375103495	0.982609538	ENSG00000279207	AC015813.6
92.93538444	1.759601638	0.669972199	2.626380081	0.008629839	0.604581883	ENSG00000101412	E2F1
95.17635361	-0.156355907	0.671059869	-0.232998446	0.815762612	0.999467288	ENSG00000205221	VIT
93.49343299	-0.035353532	0.404848095	-0.087325425	0.930412845	0.999527751	ENSG00000179965	ZNF771
93.11150532	-0.273343725	0.430194398	-0.635395826	0.52517029	0.99483168	ENSG00000162066	AMDHD2
93.41056098	0.227552429	0.34048159	0.668325208	0.503926026	0.993513219	ENSG00000011143	MKS1
93.63344645	0.247133906	0.342992162	0.72052348	0.471202748	0.99135833	ENSG00000270231	NBPF8
92.77612534	0.927218344	0.677988187	1.367602507	0.171436531	0.944422693	ENSG00000171488	LRRC8C
93.96543806	-0.229972815	0.477766459	-0.481349854	0.630267871	0.99483168	ENSG00000109819	PPARGC1A
94.80119841	0.048847878	0.354702006	0.137715256	0.890465462	0.999467288	ENSG00000204348	DXO
93.77641207	0.576972904	0.383542252	1.504326842	0.132497231	0.933741583	ENSG00000234545	FAM133B
92.34066413	-0.182954814	0.512409785	-0.357047853	0.721055982	0.99900742	ENSG00000122378	PRXL2A
93.99414254	-0.196352123	0.36320048	-0.540616363	0.588772037	0.99483168	ENSG00000167720	SRR
93.55294123	-0.283510241	0.358528171	-0.790761407	0.429083234	0.991301167	ENSG00000144040	SFXN5
95.07695281	-0.499597598	0.418190791	-1.194664276	0.232218225	0.959575179	ENSG00000163040	CCDC74A
91.82817484	-0.177627353	0.443245896	-0.400742238	0.68860991	0.998869317	ENSG00000069011	PITX1
93.0659737	-0.456936244	0.439947246	-1.038615988	0.298983367	0.972186492	ENSG00000197483	ZNF628
92.71993248	0.248182827	0.395036323	0.628253181	0.529838097	0.99483168	ENSG00000186104	CYP2R1
93.66922925	-0.959465991	0.461837253	-2.077498045	0.037755615	0.790190505	ENSG00000115896	PLCL1
92.172248	1.667039996	1.20087681	1.388185684	0.16508051	0.943223078	ENSG00000180139	ACTA2-AS1
94.55028874	0.03095243	0.581687134	0.053211474	0.957563414	0.999527751	ENSG00000125740	FOSB
93.41554965	-0.161417593	0.343989611	-0.46925136	0.638889978	0.996553586	ENSG00000164296	TIGD6
93.40184526	0.227884488	0.404733157	0.563048726	0.573401705	0.99483168	ENSG00000130299	GTPBP3
92.39402604	-0.450410117	0.399448224	-1.127580724	0.259497031	0.962465655	ENSG00000232533	AC093673.1
91.67481486	-0.655900327	1.244530455	-0.527026337	0.5981753	0.99483168	ENSG00000185924	RTN4RL1
93.91283988	0.154840338	0.344451769	0.449526906	0.653051603	0.997527562	ENSG00000157212	PAXIP1
94.17870469	-1.323724536	1.235772621	-1.0711716	0.284092279	0.963310634	ENSG00000151136	BTBD11
95.15832183	-0.477620992	0.633443192	-0.754007618	0.450844648	0.99135833	ENSG00000188092	GPR89B
94.35147859	0.375188693	0.374608146	1.001549745	0.316561103	0.974768742	ENSG00000086848	ALG9
91.18373132	-0.314858626	0.545297682	-0.577406866	0.563664637	0.99483168	ENSG00000110002	VWA5A
93.46814516	0.250933041	0.458876552	0.546842151	0.584487182	0.99483168	ENSG00000174365	SNHG11
92.21955235	0.118030438	0.354853131	0.332617717	0.739422868	0.999467288	ENSG00000197568	HHLA3
92.66902672	-0.007445048	0.370508589	-0.020094132	0.983968282	0.999527751	ENSG00000137720	C11orf1
89.85470829	0.246349237	0.512182505	0.480979405	0.630531136	0.99483168	ENSG00000059378	PARP12
95.22284751	1.387516941	0.52015817	2.667490434	0.007642006	0.563130099	ENSG00000209082	MT-TL1
91.93400373	-0.215766209	0.382427373	-0.564201793	0.572616808	0.99483168	ENSG00000130244	FAM98C
91.93685017	0.513886191	0.61504625	0.835524468	0.403422476	0.988927059	ENSG00000166845	C18orf54
93.59899754	-0.331026093	0.50483224	-0.65571504	0.51200749	0.993513219	ENSG00000171385	KCND3
92.67544924	0.101692647	0.346069821	0.293850087	0.76887246	0.999467288	ENSG00000143951	WDPCP
92.65508006	0.256958296	0.371384537	0.691892825	0.489004637	0.993513219	ENSG00000154814	OXNAD1
92.38784291	-0.867614586	0.377623624	-2.297564377	0.021586595	0.73997555	ENSG00000218426	AL590867.2
90.84741237	0.104285857	0.582967918	0.178887814	0.858025791	0.999467288	ENSG00000139832	RAB20
93.20344615	-0.086036277	0.363684101	-0.236568704	0.812991401	0.999467288	ENSG00000198794	SCAMP5
92.19810253	0.431781815	0.335350215	1.287554906	0.197900945	0.954310328	ENSG00000186132	C2orf76
93.02288711	0.26495703	0.3967803	0.667767603	0.504281949	0.993513219	ENSG00000198168	SVIP
92.2914377	-0.184712473	0.495037531	-0.373128221	0.709053016	0.99900742	ENSG00000118292	C1orf54
95.09150467	0.080627017	0.559488206	0.144108519	0.885414778	0.999467288	ENSG00000137273	FOXF2
92.15684754	0.385267606	0.350056996	1.100585364	0.271077159	0.962465655	ENSG00000244045	TMEM199
92.15002476	0.344171674	0.519139187	0.662966083	0.507352276	0.993513219	ENSG00000104356	POP1
93.12088939	-0.067450809	0.389673213	-0.173095832	0.862576101	0.999467288	ENSG00000213020	ZNF611
91.56418951	-0.228467988	0.379703058	-0.601701733	0.547372696	0.99483168	ENSG00000176386	CDC26
92.53615277	0.804712057	0.453358329	1.775002257	0.075897514	0.897052166	ENSG00000013573	DDX11
90.25965296	0.464104644	0.453570318	1.023225341	0.306201314	0.973427483	ENSG00000133106	EPSTI1
92.07298981	0.638717211	0.331257621	1.92815854	0.053835409	0.847598083	ENSG00000144559	TAMM41
91.7989291	0.018974836	0.3191299	0.059458032	0.952587292	0.999527751	ENSG00000213066	FGFR1OP
93.1382928	-0.530333028	0.441319966	-1.201697337	0.229480813	0.959575179	ENSG00000134864	GGACT
91.33984629	0.373061369	0.349754426	1.066638023	0.286135324	0.964826902	ENSG00000128694	OSGEPL1
92.59430247	-0.663291889	0.409054024	-1.621526378	0.104904798	0.92169898	ENSG00000225791	TRAM2-AS1
90.26491518	-1.371001644	0.662260145	-2.070185945	0.038434935	0.793985738	ENSG00000174945	AMZ1
91.55338098	0.117507006	0.332533731	0.353368683	0.723812061	0.999456245	ENSG00000141577	CEP131

92.56204226	0.574287857	0.363490899	1.579923619	0.114124359	0.923922314	ENSG00000171649	ZIK1
91.16824848	-0.349373657	0.439650655	-0.794661973	0.426810158	0.991301167	ENSG00000116663	FBXO6
91.04656867	0.164191066	0.348484138	0.471157931	0.637527958	0.99653524	ENSG00000197329	PELI1
92.30586501	-0.051382253	0.367381562	-0.139860729	0.88877003	0.999467288	ENSG00000178700	DHFR2
92.02359506	0.361325799	0.671515025	0.538075524	0.590524909	0.99483168	ENSG00000183111	ARHGEF37
91.17280754	-0.14541669	0.41798369	-0.347900393	0.72791499	0.999467288	ENSG00000226742	HSBP1L1
91.42011945	-0.315959974	0.388224193	-0.813859569	0.415725405	0.990226751	ENSG00000229132	EIF4A1P10
94.58553149	-0.907904139	0.622341204	-1.458852689	0.144605657	0.935644597	ENSG00000230487	PSMG3-AS1
90.16004187	-0.277970688	0.382399946	-0.726910898	0.467280541	0.99135833	ENSG00000148291	SURF2
91.54864603	-0.013431388	0.61833225	-0.021721959	0.982669747	0.999527751	ENSG00000235194	PPP1R3E
90.23679718	0.188713692	0.368466491	0.512159711	0.608539237	0.99483168	ENSG00000168301	KCTD6
91.47230337	0.423285292	0.33016031	1.282059894	0.199821637	0.954310328	ENSG00000107672	NSMCE4A
90.70877364	0.220963581	0.346127672	0.638387506	0.523221462	0.994616524	ENSG00000010539	ZNF200
92.02789295	0.876760285	0.626714721	1.398978285	0.161819495	0.943223078	ENSG00000170425	ADORA2B
90.57589837	0.434781284	0.466555071	0.931897028	0.351389744	0.977691382	ENSG00000183506	PI4KAP2
91.63675179	-0.685521147	0.3916144	-1.750500356	0.080032013	0.897807954	ENSG00000092036	HAUS4
88.48176553	0.541104411	0.809650977	0.66831811	0.503930555	0.993513219	ENSG00000126860	EVI2A
90.93018621	-0.519619892	0.383178719	-1.356077113	0.175074634	0.946278819	ENSG00000132205	EMILIN2
89.97784407	0.289582002	0.363033637	0.797672646	0.425060481	0.991301167	ENSG00000144199	FAHD2B
92.50019927	-0.562936362	0.4727512	-1.190766648	0.233745205	0.959884875	ENSG00000166435	XRRA1
89.78217333	0.540689981	0.413274626	1.308306747	0.190769287	0.949234949	ENSG00000249115	HAUS5
91.2748394	0.437294482	0.338923001	1.290247285	0.196964813	0.954310328	ENSG00000170325	PRDM10
91.05704476	-0.074904937	0.38482025	-0.194649156	0.845667618	0.999467288	ENSG00000135482	ZC3H10
85.98656205	-0.149741584	0.804668049	-0.186091127	0.8523733	0.999467288	ENSG00000174600	CMKLR1
90.28430028	-0.213738688	0.346351935	-0.617114172	0.537159422	0.99483168	ENSG00000126106	TMEM53
91.29648085	0.453152655	0.455215383	0.995468676	0.319508372	0.974964731	ENSG00000067191	CACNB1
85.78496488	1.570224609	1.543132538	1.017556542	0.308888749	0.973427483	ENSG00000110203	FOLR3
90.57844738	0.405565931	0.429322423	0.94466515	0.344829855	0.977691382	ENSG00000257727	CNPY2
90.13772101	-0.187763936	0.417680245	-0.449539901	0.653042231	0.997527562	ENSG00000187792	ZNF70
89.08320193	-0.689916971	0.491541305	-1.403578832	0.160444302	0.941607907	ENSG00000112320	SOBP
90.18952782	0.143615585	0.348757137	0.411792535	0.680491493	0.998869317	ENSG00000106479	ZNF862
91.76938106	0.211087665	0.436551448	0.483534451	0.628716304	0.99483168	ENSG00000150967	ABCB9
91.97092221	-0.095217283	0.560311657	-0.169936288	0.865060242	0.999467288	ENSG00000144218	AFF3
89.86877282	0.725406733	0.505612059	1.434710111	0.151369722	0.937894849	ENSG00000171951	SCG2
91.65373694	0.655569327	0.398182297	1.646405005	0.099680403	0.921147998	ENSG00000172766	NAA16
90.11071683	0.30228051	0.357675653	0.845124647	0.398041248	0.988378925	ENSG00000164403	SHROOM1
91.59083055	0.018221956	0.431002461	0.042278079	0.96627702	0.999527751	ENSG00000177932	ZNF354C
89.77788309	-0.38180939	0.476931569	-0.800553823	0.423389993	0.991301167	ENSG00000180233	ZNRF2
91.2511187	0.621280947	0.474674245	1.308857503	0.190582624	0.949094286	ENSG00000221829	FANCG
89.45562676	0.40077203	0.394718687	1.015335839	0.309945766	0.973427483	ENSG00000183943	PRKX
87.97907675	-0.958231829	0.523342235	-1.83098509	0.067102769	0.87420149	ENSG00000165949	IFI27
88.91682953	-0.014785196	0.417952623	-0.035375291	0.971780487	0.999527751	ENSG00000065057	NTHL1
90.44188022	0.282273389	0.44204888	0.638556959	0.523111189	0.994616524	ENSG00000213096	ZNF254
88.26509151	-0.137142412	0.569263817	-0.240911873	0.80962342	0.999467288	ENSG00000061337	LZTS1
90.33620401	0.489535106	0.448364934	1.091822908	0.274910948	0.962465655	ENSG00000140471	LINS1
89.40769733	0.599078884	0.39431166	1.519302988	0.128686248	0.928627271	ENSG00000099625	CBARP
90.88965171	0.213053375	0.516012506	0.41288413	0.679691509	0.998869317	ENSG00000081059	TCF7
89.46034074	0.673946895	0.325328511	2.071588785	0.038303807	0.793985738	ENSG00000178971	CTC1
89.68176217	0.08397379	0.358507766	0.234231439	0.814805309	0.999467288	ENSG00000204859	ZBTB48
90.53990809	-0.183605637	0.351870587	-0.521798763	0.601810451	0.99483168	ENSG00000185149	NPY2R
88.99019426	0.436564476	0.424450192	1.02854112	0.303695359	0.973213238	ENSG00000187049	TMEM216
90.27883819	-0.070223936	0.351924185	-0.199542796	0.84183817	0.999467288	ENSG00000185798	WDR53
90.59889179	0.329277964	0.449977226	0.731765843	0.4643115	0.99135833	ENSG00000120519	SLC10A7
90.15139336	0.464450023	0.391929063	1.185035933	0.23600324	0.962465655	ENSG00000167393	PPP2R3B
90.57981809	0.899557896	0.390951982	2.300942154	0.021394901	0.73997555	ENSG00000198393	ZNF26
88.77256025	0.690955482	0.470761485	1.467740042	0.142174849	0.935644597	ENSG00000182621	PLCB1
89.81097299	0.013928208	0.350423291	0.03974681	0.968294982	0.999527751	ENSG00000081026	MAGI3
90.43279296	-0.089967521	0.472303651	-0.190486608	0.848927836	0.999467288	ENSG00000011201	ANOS1
89.90106752	0.095642502	0.398478605	0.240019166	0.810315399	0.999467288	ENSG00000155592	ZKSCAN2
88.23101469	0.242815865	0.610203545	0.397926015	0.690684723	0.998869317	ENSG00000149925	ALDOA
88.0514364	1.188625186	0.675792836	1.758860292	0.078601241	0.897052166	ENSG00000176890	TYMS
86.05218755	-1.047993307	0.504219427	-2.078446903	0.037668217	0.790190505	ENSG00000102575	ACP5
89.04685626	0.126234851	0.358836752	0.351789081	0.724996448	0.999467288	ENSG00000215190	LINC00680
89.11671878	-0.393481432	0.407416456	-0.96579661	0.334145965	0.976026345	ENSG00000153832	FBXO36

87.93314697	-0.010990714	0.577378452	-0.019035546	0.984812749	0.999527751	ENSG00000132623	ANKEF1
90.55536844	0.062595493	0.469188447	0.133412265	0.893867346	0.999467288	ENSG00000159208	CIART
89.49927157	0.178049125	0.352775777	0.504709043	0.613763208	0.99483168	ENSG00000214135	AC132008.2
92.85413705	-1.92137679	0.68717209	-2.796063487	0.005172924	0.483572934	ENSG00000145536	ADAMTS16
89.29527637	0.079705736	0.327309743	0.243517761	0.807604328	0.999467288	ENSG00000196670	ZFP62
88.24193827	-0.177598285	0.367222008	-0.483626474	0.628650983	0.99483168	ENSG00000198225	FKBP1C
88.73531583	-0.027442859	0.406696286	-0.067477526	0.946201553	0.999527751	ENSG00000185875	THNSL1
88.03351045	-0.121453032	0.403783683	-0.30078737	0.76357664	0.999467288	ENSG00000178096	BOLA1
88.86174774	-0.34183265	0.38526355	-0.887269637	0.374933749	0.982609538	ENSG00000169570	DTWD2
89.85204952	0.256668489	0.345938747	0.74194779	0.458118969	0.99135833	ENSG00000121417	ZNF211
90.16165247	-0.537450064	0.377912171	-1.422156007	0.154980967	0.939432741	ENSG00000185252	ZNF74
89.42080073	-0.253109322	0.435393555	-0.581334564	0.56101499	0.99483168	ENSG00000229638	RPL4P4
89.02174223	0.487301684	0.352432171	1.382682187	0.166762336	0.943223078	ENSG00000164414	SLC35A1
86.68818461	0.928063489	0.602506669	1.540337288	0.123478159	0.927794614	ENSG00000261115	TMEM178B
87.40803543	-0.380075223	0.470672469	-0.807515306	0.41936966	0.990226751	ENSG00000180263	FGD6
89.63978129	1.242270674	0.409964696	3.030189396	0.002444004	0.360084787	ENSG00000173209	AHSA2P
89.8178817	-0.850614313	0.513159018	-1.65760375	0.097397472	0.921147998	ENSG00000272841	AL139393.2
88.19871448	0.404702629	0.403894499	1.002000845	0.316343185	0.974768742	ENSG00000135912	TTLL4
89.39299374	0.888313998	0.61062774	1.454755393	0.145736991	0.935644597	ENSG00000153044	CENPH
89.88504646	0.321627976	0.365226728	0.880625516	0.378520543	0.982609538	ENSG00000204946	ZNF783
88.06264476	-0.054778119	0.347718629	-0.15753576	0.874822627	0.999467288	ENSG00000114670	NEK11
88.61867792	-0.020596454	0.426040755	-0.048343858	0.961442202	0.999527751	ENSG00000205269	TMEM170B
87.82021605	-0.258754537	0.498611351	-0.518950355	0.603795362	0.99483168	ENSG00000184831	APOO
87.50775979	0.174805748	0.381327475	0.458413725	0.646655233	0.997368416	ENSG00000152147	GEMIN6
88.86445591	0.195407962	0.387676658	0.504048819	0.614227071	0.99483168	ENSG00000133302	SLF1
87.16789433	-0.313066861	0.448189032	-0.698515222	0.484855042	0.991892545	ENSG00000181524	RPL24P4
88.76164763	-0.611341311	0.454149902	-1.3461223	0.178263078	0.9469453	ENSG00000150281	CTF1
89.46413906	0.973756591	0.741253031	1.31366288	0.188959678	0.949094286	ENSG00000214193	SH3D21
88.32536692	-0.017002658	0.35179527	-0.048331117	0.961452356	0.999527751	ENSG00000213839	TMX2P1
88.76612053	-0.129451086	0.393761807	-0.328754805	0.742341026	0.999467288	ENSG00000123411	IKZF4
88.69308389	0.342879067	0.393684581	0.870948684	0.383782173	0.985655313	ENSG00000100749	VRK1
88.75640766	-0.278252247	0.321080622	-0.866611774	0.38615476	0.986422569	ENSG00000143224	PPOX
87.81120077	-0.722615594	0.376303768	-1.92029859	0.054820194	0.847598083	ENSG00000112561	TFEB
84.8796158	0.251228989	0.639341859	0.392949384	0.694356866	0.998869317	ENSG00000002746	HECW1
91.07907474	-0.490147469	0.45537124	-1.076368961	0.281762278	0.963267117	ENSG00000124191	TOX2
89.19773253	0.817077802	0.397252936	2.056820043	0.039703539	0.793985738	ENSG00000161010	MRNIP
87.8194548	0.487019835	0.35547137	1.370067682	0.170665775	0.944422693	ENSG00000187961	KLHL17
88.06210303	0.106361741	0.379935312	0.279946975	0.779518187	0.999467288	ENSG00000231365	AL359915.2
89.1109547	0.006303188	0.4909011	0.012840037	0.989755414	0.999527751	ENSG00000197608	ZNF841
87.08344719	-0.030759154	0.467520348	-0.065792118	0.947543331	0.999527751	ENSG00000261040	WFDC21P
88.71478281	-0.282366571	0.439714336	-0.64215912	0.520769873	0.993513219	ENSG00000137185	ZSCAN9
84.88350795	1.937547149	0.64618302	2.998449494	0.002713571	0.380278034	ENSG00000139132	FGD4
88.45940144	0.44741774	0.372854185	1.199980471	0.230146925	0.959575179	ENSG00000185278	ZBTB37
86.93311943	-0.498382852	0.397356028	-1.254247618	0.209752017	0.954310328	ENSG00000163132	MSX1
88.21490345	-0.17326083	0.396915538	-0.436518133	0.662460846	0.997568624	ENSG00000166860	ZBTB39
86.56250186	0.062341668	0.387355845	0.160941597	0.872139399	0.999467288	ENSG00000205181	LINC00654
87.92741094	-0.480869387	0.345238735	-1.39286047	0.163661988	0.943223078	ENSG00000160190	SLC37A1
86.6410339	1.087068704	0.665614685	1.633180169	0.10243111	0.92169898	ENSG00000175264	CHST1
86.87592068	-0.480717025	0.56811319	-0.846164168	0.397461168	0.988378925	ENSG00000102032	RENBP
87.64744688	0.704914016	0.476933087	1.478014497	0.139403898	0.935644597	ENSG00000166004	CEP295
89.76694012	-0.206721035	0.493550263	-0.418844949	0.675329452	0.998869317	ENSG00000264247	LINC00909
86.86747179	0.240743801	0.396560929	0.607078972	0.543798504	0.99483168	ENSG00000170417	TMEM182
86.97424945	-0.512355326	0.378530613	-1.353537357	0.175884024	0.946278819	ENSG00000107338	SHB
87.85614006	-0.109975948	0.352472793	-0.312012586	0.755030958	0.999467288	ENSG00000168612	ZSWIM1
86.45650341	1.234101613	0.588298434	2.097747573	0.03592745	0.785058983	ENSG00000164087	POC1A
85.40619284	0.164997348	0.450424679	0.36631507	0.714129985	0.99900742	ENSG00000131188	PRR7
86.71394961	-0.096915428	0.412454567	-0.234972372	0.814230177	0.999467288	ENSG00000196284	SUPT3H
85.45947503	0.192821423	0.547849475	0.351960588	0.72486782	0.999456245	ENSG00000154654	NCAM2
88.2266426	0.93619446	0.405538903	2.308519488	0.02097026	0.73997555	ENSG00000152926	ZNF117
86.43292125	-0.470992673	0.445450165	-1.057340889	0.290356039	0.968170184	ENSG00000135469	COQ10A
87.31659939	0.171439149	0.392685527	0.436581277	0.662415043	0.997568624	ENSG00000075131	TIPIN
85.73435519	-0.106174485	0.406177784	-0.261399045	0.793784793	0.999467288	ENSG00000100209	HSCB
87.54086722	0.108783252	0.362900488	0.299760555	0.764359805	0.999467288	ENSG00000156787	TBC1D31
86.6444006	-0.10753483	0.385545918	-0.278915754	0.780309478	0.999467288	ENSG00000215883	CYB5RL

86.452228	0.320758317	0.392439743	0.817344122	0.413731799	0.990226751	ENSG00000136932	TRMO
86.60854573	-0.190587333	0.458928765	-0.415287399	0.677931525	0.998869317	ENSG00000237854	LINC00674
86.97906499	1.412347357	0.654978207	2.15632725	0.031058121	0.775963115	ENSG00000165891	E2F7
85.87434029	0.182636416	0.361697495	0.504942443	0.613599263	0.99483168	ENSG00000275111	ZNF2
85.29322818	0.229129104	0.398104469	0.575550193	0.564919255	0.99483168	ENSG00000188493	C19orf54
83.31310354	-0.339027095	0.625023577	-0.54242289	0.587527214	0.99483168	ENSG00000099260	PALMD
86.84873722	0.472681642	0.428041711	1.10428874	0.269467906	0.962465655	ENSG00000258441	LINC00641
85.8076367	-0.17546741	0.337858278	-0.519352112	0.603515219	0.99483168	ENSG00000128891	CCDC32
85.17621998	-0.376981158	0.413012659	-0.91275933	0.361369132	0.979465649	ENSG00000162639	HENMT1
85.14115329	0.312471754	0.382908085	0.816048987	0.414472119	0.990226751	ENSG00000172878	METAP1D
87.63305008	0.305188501	0.666492179	0.457902599	0.647022419	0.997368416	ENSG00000257219	LINC02407
82.5600023	0.161106137	0.499770912	0.322359972	0.747180005	0.999467288	ENSG00000179862	CITED4
84.80183677	0.588264573	0.384996321	1.527974532	0.126518861	0.928627271	ENSG00000220848	RPS18P9
86.00401932	0.448616639	0.496544698	0.903476848	0.36627287	0.981269214	ENSG00000151287	TEX30
84.72138031	0.075002726	0.471698835	0.159005536	0.873664512	0.999467288	ENSG00000251322	SHANK3
84.50367848	-0.062999407	0.416658415	-0.151201571	0.879816711	0.999467288	ENSG00000144401	METTL21A
85.3053634	-0.503266875	0.382009267	-1.317420594	0.187697682	0.949094286	ENSG00000010626	LRRC23
86.41856484	0.691537816	0.714636496	0.967677722	0.333205348	0.976026345	ENSG00000163507	CIP2A
85.19464822	-0.068780406	0.428533673	-0.160501754	0.87248584	0.999467288	ENSG00000197119	SLC25A29
84.73505696	0.040335615	0.40628938	0.099278044	0.92091751	0.999527751	ENSG00000138439	FAM117B
85.45886254	-0.208443559	0.408243858	-0.510585903	0.609641049	0.99483168	ENSG00000132763	MMACHC
85.97031519	-0.017805478	0.377637267	-0.047149686	0.962393928	0.999527751	ENSG00000170561	IRX2
84.14580859	2.890591283	1.640218261	1.76232112	0.078015058	0.897052166	ENSG00000128510	CPA4
84.44794359	-0.235006908	0.346815082	-0.677614442	0.498016187	0.993513219	ENSG00000104983	CCDC61
85.10656825	0.40677706	0.370600756	1.097615301	0.272372509	0.962465655	ENSG00000166704	ZNF606
84.40127781	-0.084695541	0.337756905	-0.250758875	0.802000539	0.999467288	ENSG00000198945	L3MBTL3
84.56271786	0.237517409	0.353543736	0.671819027	0.501698914	0.993513219	ENSG00000273015	AC008124.1
82.75574158	0.024577288	0.530566426	0.046322735	0.963053019	0.999527751	ENSG00000144366	GULP1
90.13398593	-0.269781679	1.369115844	-0.197048103	0.843789895	0.999467288	ENSG00000054356	PTPRN
83.90438629	0.392104677	0.442856669	0.885398605	0.375941685	0.982609538	ENSG00000120647	CCDC77
84.61766986	1.118916143	0.401382264	2.78765716	0.00530907	0.487175435	ENSG00000105339	DENND3
84.38990334	0.037185893	0.390973631	0.095111	0.924226661	0.999527751	ENSG00000136936	XPA
86.86752887	-0.080825263	0.438718544	-0.184230332	0.853832765	0.999467288	ENSG00000271254	AC240274.1
85.15225438	-0.155304701	0.431506104	-0.359913103	0.718912118	0.99900742	ENSG00000175879	HOXD8
84.66719869	0.030894097	0.369402443	0.083632627	0.933348525	0.999527751	ENSG00000197050	ZNF420
83.40379706	-0.353967725	0.426662348	-0.829620254	0.406753522	0.990226751	ENSG00000226328	NUP50-DT
85.89513006	-0.127415499	0.401223749	-0.31756719	0.750813265	0.999467288	ENSG00000196275	GTF2IRD2
84.41642503	0.423324009	0.4240309	0.998332927	0.318117946	0.974768742	ENSG00000131773	KHDRBS3
84.202623	-0.096338807	0.407394974	-0.236475198	0.813063951	0.999467288	ENSG00000181284	TMEM102
83.84493271	-0.084455305	0.436703042	-0.193392986	0.846651208	0.999467288	ENSG00000188290	HES4
84.32920939	-0.064158329	0.458764028	-0.139850391	0.888778198	0.999467288	ENSG00000137965	IFI44
83.19703881	0.146496319	0.427162751	0.342951998	0.731634573	0.999467288	ENSG00000248487	ABHD14A
82.43444099	0.143591613	0.470548373	0.305158026	0.760245799	0.999467288	ENSG00000132563	REEP2
81.21399357	1.023785704	0.712574598	1.436741792	0.150791375	0.936396974	ENSG00000170458	CD14
84.34915809	0.267835081	0.379548595	0.70566743	0.48039498	0.99135833	ENSG00000189227	C15orf61
84.24061164	-0.12471738	0.394356669	-0.316255282	0.75180875	0.999467288	ENSG00000022556	NLRP2
84.93052151	0.647210532	0.348682286	1.856161205	0.063430584	0.868202292	ENSG00000198466	ZNF587
85.32252703	0.306240918	0.426748347	0.717614771	0.472994847	0.99135833	ENSG00000160124	CCDC58
83.37446162	-0.619545112	0.4232971	-1.463617661	0.143298444	0.935644597	ENSG00000233328	PFN1P1
83.41594399	-0.302215056	0.440043329	-0.686784769	0.492218369	0.993513219	ENSG00000170745	KCNS3
83.64632475	0.340928068	0.378725716	0.90019783	0.368014981	0.981269214	ENSG00000088808	PPP1R13B
83.87118782	-0.414844858	0.411178264	-1.008917284	0.313014305	0.973427483	ENSG00000125458	NT5C
84.55899412	-0.14419308	0.336418846	-0.428611779	0.668205772	0.997568624	ENSG00000198440	ZNF583
83.72756767	-0.250984915	0.359175019	-0.698781658	0.484688493	0.991892545	ENSG00000256060	TRAPPC2B
83.50981013	0.029033273	0.422632335	0.068696289	0.945231372	0.999527751	ENSG00000107282	APBA1
84.55612981	0.010737755	0.344916428	0.031131469	0.975164693	0.999527751	ENSG00000229944	EIF4EP2
82.36589472	-0.134886582	0.378515625	-0.356356707	0.721573447	0.99900742	ENSG00000087884	AAMDC
83.34457592	1.080214146	0.538965252	2.004237085	0.04504467	0.813244956	ENSG00000125618	PAX8
85.05763133	-0.296937345	0.422253132	-0.703221178	0.481917923	0.991525194	ENSG00000138380	CARF
83.85284058	-0.218022753	0.493072804	-0.442171524	0.658365091	0.997568624	ENSG00000233608	TWIST2
83.10318115	-0.713682065	2.337252745	-0.30535083	0.760098967	0.999467288	ENSG00000164266	SPINK1
84.30760803	-0.286982804	0.374447071	-0.766417543	0.443427898	0.99135833	ENSG00000170631	ZNF16
83.66919221	0.215340922	0.333685264	0.64534142	0.518705957	0.993513219	ENSG00000278129	ZNF8
84.65370212	-0.113979391	0.368722358	-0.309119825	0.757230379	0.999467288	ENSG00000120158	RCL1

83.97888777	0.125743962	0.413860737	0.303831582	0.761256204	0.999467288	ENSG00000186889	TMEM17
82.98591091	0.258415126	0.356287397	0.725299656	0.468268217	0.99135833	ENSG00000146707	POMZP3
84.81823858	0.593055496	0.57511885	1.031187721	0.302452797	0.972793733	ENSG00000147394	ZNF185
83.30450153	0.199458005	0.420243825	0.474624476	0.635054657	0.995506752	ENSG00000147799	ARHGAP39
84.55980376	0.578339757	0.374166568	1.545674591	0.122183177	0.923922314	ENSG00000161298	ZNF382
82.85419642	-0.225048753	0.382620766	-0.588177049	0.556413461	0.99483168	ENSG00000185453	ZSWIM9
83.13456636	0.309341137	0.491779389	0.629024202	0.529333212	0.99483168	ENSG00000137959	IFI44L
83.43551187	0.560920471	0.377831879	1.484576875	0.137655945	0.934144427	ENSG00000114405	C3orf14
84.71051109	0.114434184	0.393605941	0.290732867	0.771255632	0.999467288	ENSG00000248049	UBA6-AS1
83.52882102	-0.003012493	0.346646969	-0.008690377	0.99306617	0.999527751	ENSG00000184378	ACTRT3
83.607824	-0.188135221	0.343065335	-0.548394728	0.583420896	0.99483168	ENSG00000137691	CFAP300
83.12872889	-0.289258382	0.384512098	-0.752273814	0.451886412	0.99135833	ENSG00000182173	TSEN54
81.56423689	-0.335015815	0.492999665	-0.679545727	0.496792145	0.993513219	ENSG00000143842	SOX13
83.26721957	0.352389496	0.383512069	0.918848517	0.358174811	0.979427314	ENSG00000163319	MRPS18C
83.31702472	-0.149162083	0.382034202	-0.390441699	0.696209958	0.998869317	ENSG00000142528	ZNF473
82.93746653	0.024756116	0.360994899	0.068577468	0.945325955	0.999527751	ENSG00000125962	ARMCX5
85.24705839	-0.375455571	0.424475193	-0.884517111	0.37641713	0.982609538	ENSG00000136630	HLX
84.32814573	1.115089942	0.47571646	2.344022199	0.019077036	0.727643954	ENSG00000132436	FIGNL1
83.01442196	-0.253925457	0.463554651	-0.5477789	0.583843728	0.99483168	ENSG00000272419	LINC01145
82.53416282	-0.634754491	0.511792458	-1.240257611	0.214880126	0.954310328	ENSG00000057657	PRDM1
81.94897588	-0.971379436	0.575400923	-1.688178446	0.091376979	0.915862397	ENSG00000133069	TMCC2
83.79887395	0.002928096	0.42342599	0.006915248	0.994482474	0.999527751	ENSG00000176834	VSIG10
81.46034458	-0.543853791	0.512355782	-1.06147683	0.288473257	0.966288987	ENSG00000183044	ABAT
81.84105563	-0.052460428	0.354171767	-0.148121429	0.882246938	0.999467288	ENSG00000180537	RNF182
81.46183684	-0.288562386	0.435837215	-0.662087532	0.507915126	0.993513219	ENSG00000180822	PSMG4
83.42292311	1.004645746	0.427581083	2.349602883	0.01879345	0.726053908	ENSG00000213398	LCAT
83.45320059	-1.268684384	1.579104543	-0.803420134	0.421731942	0.991301167	ENSG00000160201	U2AF1
82.86526104	-0.252750839	0.453848749	-0.556905444	0.577592035	0.99483168	ENSG00000273748	AL592183.1
84.08540262	0.681077013	0.419775948	1.622477457	0.104701156	0.92169898	ENSG00000174799	CEP135
80.50950387	-0.873040758	0.409067724	-2.134220585	0.032824728	0.775963115	ENSG00000206337	HCP5
82.37719873	-0.218510339	0.360099513	-0.606805427	0.543980046	0.99483168	ENSG00000001630	CYP51A1
81.90756708	0.535760425	0.404056323	1.32595481	0.184854666	0.949094286	ENSG00000171763	SPATA5L1
84.19027218	-1.059152986	0.446162019	-2.373920102	0.017600359	0.726053908	ENSG00000181031	RPH3AL
83.87050605	-0.28323247	0.417073926	-0.679094168	0.497078198	0.993513219	ENSG00000196659	TTC30B
82.62579749	-0.119754622	0.350243739	-0.341917953	0.732412638	0.999467288	ENSG00000154743	TSEN2
82.58731123	0.70972044	0.367925824	1.92897697	0.053733721	0.847598083	ENSG00000182903	ZNF721
83.60147446	0.616001723	0.683473008	0.901281712	0.367438555	0.981269214	ENSG00000131094	C1QL1
82.13181719	-0.21896817	0.375209503	-0.583589084	0.559496806	0.99483168	ENSG00000169660	HEXDC
81.61923867	-0.147171927	0.383722949	-0.383536945	0.701321683	0.998869317	ENSG00000162396	PARS2
83.15035063	0.383749631	0.52204514	0.735088983	0.462285304	0.99135833	ENSG00000196843	ARID5A
82.48379787	0.12778755	0.425963559	0.299996438	0.764179872	0.999467288	ENSG00000133739	LRRCC1
79.96890973	-0.501687819	0.448675328	-1.118153344	0.263501507	0.962465655	ENSG00000184678	HIST2H2BE
82.14529148	0.168335206	0.371162652	0.453534871	0.65016364	0.997381078	ENSG00000155275	TRMT44
82.90996431	0.081124583	0.413433579	0.196221562	0.844436752	0.999467288	ENSG00000166348	USP54
83.09534446	-0.228641934	0.634468833	-0.360367479	0.718572343	0.99900742	ENSG00000100626	GALNT16
83.02646337	0.246458927	0.394966287	0.62399991	0.532627644	0.99483168	ENSG00000167384	ZNF180
83.35428151	0.249946682	0.416309228	0.60038708	0.548248296	0.99483168	ENSG00000181638	ZFP41
83.16063506	-0.105642375	0.418109206	-0.252666943	0.800525595	0.999467288	ENSG00000124279	FASTKD3
80.1757908	-0.297392283	0.641793442	-0.463376942	0.643094203	0.997097166	ENSG00000196196	HRCT1
82.87666746	0.146716586	0.479931054	0.305703464	0.759830436	0.999467288	ENSG00000072071	ADGRL1
80.37261242	0.353827494	0.49898174	0.709099082	0.478262993	0.99135833	ENSG00000197147	LRRC8B
82.14823059	0.037629513	0.346079128	0.108730951	0.913415892	0.999527751	ENSG00000189362	NEMP2
82.43366978	0.4154988	0.376233268	1.104364857	0.269434899	0.962465655	ENSG00000163935	SFMBT1
78.64473871	0.435676725	0.745716405	0.584239159	0.559059419	0.99483168	ENSG00000169429	CXCL8
82.96640009	0.648914985	0.505145685	1.284609579	0.19892875	0.954310328	ENSG00000178026	LRRC75B
82.65880508	-0.220649282	1.212532002	-0.181973986	0.855603141	0.999467288	ENSG00000188487	INSC
80.80460046	0.551428213	0.512426447	1.076111929	0.281877201	0.963267117	ENSG00000006062	MAP3K14
80.76458087	0.045492488	0.360913091	0.126048317	0.899693678	0.999527751	ENSG00000254901	BORCS8
81.4323896	-0.824131929	0.45291936	-1.819599693	0.068819989	0.87420149	ENSG00000196358	NTNG2
80.94433923	-0.21199077	0.419912985	-0.50484452	0.613668043	0.99483168	ENSG00000102743	SLC25A15
81.18599415	0.340539045	0.350570015	0.971386685	0.331355757	0.976026345	ENSG00000174021	GNG5
84.07619877	-0.196920227	0.480415223	-0.409895894	0.681882317	0.998869317	ENSG00000132326	PER2
82.74439678	-0.034836051	0.479043856	-0.072719962	0.942028963	0.999527751	ENSG00000164124	TMEM144
83.61254716	0.118864487	0.468828046	0.253535358	0.799854541	0.999467288	ENSG00000100330	MTMR3

80.28162052	0.15995788	0.430159191	0.371857405	0.70999902	0.99900742	ENSG00000163879	DNALI1
81.38578651	0.075633102	0.396327964	0.190834634	0.848655153	0.999467288	ENSG00000116396	KCNC4
81.33947833	1.107781836	0.472666208	2.343687399	0.019094167	0.727643954	ENSG00000064225	ST3GAL6
81.05003485	0.397772841	0.47163924	0.843383686	0.399013893	0.988378925	ENSG00000182957	SPATA13
81.63535163	-0.33680195	0.372804727	-0.903427252	0.366299182	0.981269214	ENSG00000171448	ZBTB26
82.01408958	-0.00353668	0.531626191	-0.006652569	0.994692057	0.999527751	ENSG00000170919	TPT1-AS1
80.26024871	-0.150181991	0.468692195	-0.320427761	0.748644084	0.999467288	ENSG00000117877	CD3EAP
81.60572374	0.6219586	0.347466894	1.789979449	0.073457215	0.888663898	ENSG00000141956	PRDM15
81.80994853	-0.2419319	0.50084918	-0.483043417	0.629064909	0.99483168	ENSG00000171823	FBXL14
80.84645598	2.479154745	1.471906295	1.684315608	0.092120691	0.920123883	ENSG00000143476	DTL
81.38355201	-0.691722713	0.438988512	-1.575719394	0.115090486	0.923922314	ENSG00000142544	CTU1
81.96470524	0.736576768	0.475482924	1.549112976	0.121354564	0.923922314	ENSG00000279095	AC243964.3
81.10148976	0.045058869	0.396932733	0.113517644	0.909620175	0.999527751	ENSG00000213462	ERV3-1
80.25712104	0.676567166	0.458318985	1.476192756	0.139892154	0.935644597	ENSG00000204856	FAM216A
79.72383759	1.145396055	0.501348072	2.284632413	0.022334391	0.744905651	ENSG00000054654	SYNE2
80.65946704	-0.336112476	0.381597248	-0.880804244	0.378423782	0.982609538	ENSG00000108423	TUBD1
81.49914926	0.378302808	0.531438373	0.711846995	0.476559528	0.99135833	ENSG00000105486	LIG1
80.64434183	-0.046743641	0.361916194	-0.12915598	0.897234227	0.999467288	ENSG00000280433	FP565260.6
81.77122256	-0.464020043	0.457234538	-1.014840316	0.310181952	0.973427483	ENSG00000164929	BAALC
81.42862448	0.328570744	0.385564323	0.852181398	0.394113419	0.988378925	ENSG00000182325	FBXL6
80.22530824	-0.445079838	0.419246749	-1.061617862	0.288409201	0.966288987	ENSG00000186153	WWOX
78.21306186	0.08890993	0.567356843	0.156709011	0.875474185	0.999467288	ENSG00000104313	EYA1
80.86191775	0.432122439	0.465986521	0.927328194	0.35375616	0.977691382	ENSG00000118402	ELOVL4
80.84690841	-0.551289261	0.660557158	-0.834582222	0.403952975	0.988927059	ENSG00000007944	MYLIP
79.57619894	-0.533953205	0.509560072	-1.047870966	0.294698061	0.970706863	ENSG00000241644	INMT
81.58908956	-0.3551234	0.583128313	-0.60899701	0.54252642	0.99483168	ENSG00000232434	AJM1
80.49590357	-0.210006005	0.337148077	-0.622889526	0.533357122	0.99483168	ENSG00000034533	ASTE1
80.00304114	-0.525347639	0.434786147	-1.208289738	0.226935834	0.958865115	ENSG00000161980	POLR3K
79.31340213	0.041053274	0.398617532	0.102989133	0.917971596	0.999527751	ENSG00000104880	ARHGEF18
80.0346022	-0.040903021	0.378235432	-0.108141695	0.913883294	0.999527751	ENSG00000105518	TMEM205
78.43122329	-0.117811912	0.479796342	-0.245545666	0.806033953	0.999467288	ENSG00000177380	PPFIA3
80.13794083	0.624028212	0.378857394	1.647132197	0.099530876	0.921147998	ENSG00000133460	SLC2A11
79.37001226	0.480176958	0.367481999	1.306667971	0.191325501	0.949261383	ENSG00000131591	C1orf159
78.85993265	-0.051373776	0.403914076	-0.127189863	0.898790128	0.999527751	ENSG00000066379	ZNRD1
80.31666524	-0.075234587	0.414864385	-0.181347423	0.856094885	0.999467288	ENSG00000198478	SH3BGRL2
79.78695863	1.248787246	0.507416582	2.461069049	0.01385237	0.673635639	ENSG00000160957	RECQL4
80.37300917	-0.180499044	0.471734931	-0.382628108	0.701995526	0.998869317	ENSG00000152782	PANK1
79.57378166	-0.101143783	0.407734692	-0.248062737	0.804085867	0.999467288	ENSG00000153485	TMEM251
79.61266255	-0.058705019	0.397336477	-0.147746363	0.882542941	0.999467288	ENSG00000225973	PIGBOS1
78.06879441	1.741645691	0.517703254	3.3641776	0.000767721	0.204478655	ENSG00000139318	DUSP6
80.20833482	0.36208277	0.362590531	0.998599629	0.31798868	0.974768742	ENSG00000203791	EEF1AKMT2
80.07242718	-0.078850809	0.377352047	-0.208958213	0.83448086	0.999467288	ENSG00000278318	ZNF229
81.3206019	0.404714771	0.429975336	0.941251129	0.346576181	0.977691382	ENSG00000158691	ZSCAN12
78.11423592	0.468269026	0.690486695	0.678172409	0.497662384	0.993513219	ENSG00000164342	TLR3
80.45063134	-0.035733189	0.423940213	-0.084288274	0.932827235	0.999527751	ENSG00000182400	TRAPPC6B
80.06095368	-0.028377972	0.391896072	-0.072411984	0.942274048	0.999527751	ENSG00000144026	ZNF514
78.91590217	0.316888186	0.346181262	0.91538226	0.359990988	0.979465649	ENSG00000173933	RBM4
83.20649927	0.029808102	1.373813689	0.021697339	0.982689386	0.999527751	ENSG00000101096	NFATC2
75.94835567	1.578232762	0.605452815	2.606698199	0.00914199	0.611285922	ENSG00000189058	APOD
80.79406312	0.099029188	0.62154026	0.159328678	0.873409928	0.999467288	ENSG00000108932	SLC16A6
79.08450725	-0.067706595	0.409806709	-0.165215926	0.868774031	0.999467288	ENSG00000152455	SUV39H2
77.33387835	-0.971537503	1.459121275	-0.665837391	0.505515042	0.993513219	ENSG00000144057	ST6GAL2
78.23367344	0.076187992	0.453851613	0.167869828	0.866685687	0.999467288	ENSG00000182511	FES
79.71223257	-0.089046664	0.378327608	-0.235369194	0.813922194	0.999467288	ENSG00000112294	ALDH5A1
78.39209912	0.40313602	0.474513644	0.849577299	0.395560137	0.988378925	ENSG00000132423	COQ3
78.9180009	0.361291536	0.428410895	0.843329477	0.399044201	0.988378925	ENSG00000147894	C9orf72
79.72365115	-0.0314874	0.433618742	-0.072615404	0.942112168	0.999527751	ENSG00000110318	CEP126
78.35234131	0.159087513	0.353174524	0.450450138	0.6523859	0.997449432	ENSG00000256229	ZNF486
77.7472913	0.077995898	0.60090255	0.129797915	0.896726313	0.999467288	ENSG00000197093	GAL3ST4
78.23599313	-0.327321406	0.362155056	-0.903815647	0.366093164	0.981269214	ENSG00000180353	HCLS1
76.59569282	-0.070629302	0.483143312	-0.146187064	0.883773717	0.999467288	ENSG00000276180	HIST1H4I
78.11237429	-0.193812164	0.384720002	-0.503774596	0.614419781	0.99483168	ENSG00000184675	AMER1
78.5124671	0.15235151	0.409821723	0.371750693	0.710078478	0.99900742	ENSG00000130038	CRACR2A
78.84444744	0.189866213	0.452967432	0.419160849	0.675098583	0.998869317	ENSG00000196123	KIAA0895L

78.83230874	0.1662735	0.36541554	0.455025804	0.649090675	0.997381078	ENSG00000180376	CCDC66
77.96786183	-0.175403361	0.394098547	-0.445074875	0.656265645	0.997568624	ENSG00000137269	LRRC1
78.45962848	-0.149212725	0.456298099	-0.327007114	0.743662504	0.999467288	ENSG00000140961	OSGIN1
77.33988166	0.388479527	0.418533136	0.928193	0.353307465	0.977691382	ENSG00000164162	ANAPC10
79.0390267	0.989795726	0.603955531	1.638855303	0.101243398	0.92169898	ENSG00000106462	EZH2
78.11680749	0.957063237	0.826041364	1.15861418	0.246613486	0.962465655	ENSG00000131398	KCNC3
78.92661495	0.138092467	0.399306275	0.345830947	0.729469766	0.999467288	ENSG00000284770	TBCE
80.26478798	-0.46907376	0.538987721	-0.870286543	0.384143831	0.985655313	ENSG00000138795	LEF1
78.50237027	-0.079923661	0.366349701	-0.218162213	0.827302726	0.999467288	ENSG00000196646	ZNF136
78.3612399	0.470914426	0.487322413	0.966330327	0.333878916	0.976026345	ENSG00000171208	NETO2
78.08243593	0.281325328	0.391705299	0.718206592	0.472629914	0.99135833	ENSG00000182378	PLCXD1
77.75547884	0.118885514	0.361957701	0.328451402	0.742570383	0.999467288	ENSG00000277161	PIGW
77.94088296	-0.080903485	0.397294423	-0.203636095	0.838637884	0.999467288	ENSG00000147813	NAPRT
79.02807723	0.553104328	0.61477984	0.899678702	0.368291261	0.981269214	ENSG00000151849	CENPJ
78.11164039	-0.203431725	0.355771019	-0.571805218	0.567453942	0.99483168	ENSG00000271122	AC018647.2
77.64481716	-0.066926747	0.389810351	-0.171690533	0.863680829	0.999467288	ENSG00000147099	HDAC8
78.98294296	2.565846951	0.592911592	4.32753717	1.51E-05	0.02722251	ENSG00000175265	GOLGA8A
77.6763587	0.148390642	0.383704178	0.38673189	0.698954705	0.998869317	ENSG00000205643	CDPF1
77.6559576	-0.17368172	0.384669757	-0.451508642	0.651622998	0.997449432	ENSG00000132122	SPATA6
76.10435624	-0.064851015	0.490101672	-0.132321554	0.89472996	0.999467288	ENSG00000112541	PDE10A
74.53123114	1.308374045	0.857176737	1.526376053	0.126916238	0.928627271	ENSG00000160801	PTH1R
77.69321412	0.096865757	0.347159544	0.279023749	0.780226599	0.999467288	ENSG00000285967	NIPBL-DT
77.72636045	0.900749904	0.424119166	2.123813249	0.033685763	0.775963115	ENSG00000272674	PCDHB16
74.05970236	1.113275493	0.727907731	1.529418423	0.126160748	0.928627271	ENSG00000003989	SLC7A2
78.07807413	0.223516095	0.369667987	0.604640117	0.545418145	0.99483168	ENSG00000148680	HTR7
77.52553597	0.224648012	0.38150387	0.588848578	0.555962855	0.99483168	ENSG00000133627	ACTR3B
75.07167055	-0.23916572	0.560761054	-0.426502015	0.66974208	0.997568624	ENSG00000148803	FUOM
77.06647086	0.300584354	0.378503839	0.794138191	0.427114986	0.991301167	ENSG00000175193	PARL
76.62572043	0.052047411	0.421641098	0.123440081	0.901758619	0.999527751	ENSG00000158301	GPRASP2
77.45103589	-0.233294532	0.380189055	-0.613627692	0.53946138	0.99483168	ENSG00000148090	AUH
77.69505219	0.380992476	0.373961867	1.018800338	0.308297765	0.973427483	ENSG00000076555	ACACB
74.62437355	-0.347707554	0.656997928	-0.52923691	0.596641115	0.99483168	ENSG00000130303	BST2
78.20855884	0.379731175	0.398398929	0.953143061	0.340517591	0.977691382	ENSG00000196967	ZNF585A
76.62461551	0.115801214	0.364490773	0.317706845	0.750707319	0.999467288	ENSG00000088876	ZNF343
76.95402142	0.845279225	0.884320349	0.955851831	0.339147098	0.977691382	ENSG00000242221	PSG2
77.67561523	-0.390093618	0.424840067	-0.918212873	0.358507431	0.979427314	ENSG00000178082	TWF1P1
76.76286342	0.477010065	0.470186739	1.014511949	0.310338531	0.973427483	ENSG00000136098	NEK3
75.90194049	-0.831318957	0.535117221	-1.553526824	0.120297326	0.923922314	ENSG00000175899	A2M
75.98433138	-0.422330002	0.348576321	-1.21158546	0.225671103	0.958865115	ENSG00000141480	ARRB2
76.69479712	0.073124854	0.429647924	0.170197154	0.86485509	0.999467288	ENSG00000163002	NUP35
76.70996664	-0.35677991	0.341112913	-1.045929066	0.295593783	0.970706863	ENSG00000118162	KPTN
76.87039595	-0.230275027	0.394786443	-0.583290109	0.55969802	0.99483168	ENSG00000181826	RELL1
76.6699934	0.2646329	0.451826516	0.585695815	0.558079947	0.99483168	ENSG00000107968	MAP3K8
75.46359277	-0.057574087	0.516690742	-0.111428524	0.911276542	0.999527751	ENSG00000250320	AC113383.1
76.77110669	0.564693844	0.470226825	1.200896702	0.229791274	0.959575179	ENSG00000099256	PRTFDC1
75.87730763	0.310521885	0.411604539	0.75441803	0.450598249	0.99135833	ENSG00000132359	RAP1GAP2
76.81490243	-0.210891851	0.364714751	-0.578237786	0.563103591	0.99483168	ENSG00000213326	RPS7P11
75.84771854	-0.339648949	0.385663578	-0.880687127	0.378487186	0.982609538	ENSG00000008382	MPND
75.99485903	-0.463663908	0.448805447	-1.033106685	0.301553975	0.972793733	ENSG00000235098	ANKRD65
75.95895491	0.214419023	0.420561611	0.509839742	0.610163741	0.99483168	ENSG00000101624	CEP76
75.69892553	0.249529944	0.368840568	0.676525215	0.498707242	0.993513219	ENSG00000145331	TRMT10A
77.6299227	0.173996228	0.414043386	0.420236704	0.674312544	0.998869317	ENSG00000256683	ZNF350
76.83048094	0.005095296	0.3740841	0.013620724	0.989132571	0.999527751	ENSG00000186376	ZNF75D
75.22866739	-0.532578819	0.505124226	-1.054352161	0.291721716	0.97025964	ENSG00000117461	PIK3R3
75.61673993	-0.03273671	0.846089899	-0.038691763	0.969136141	0.999527751	ENSG00000212864	RNF208
75.90086847	-0.260961784	0.364400507	-0.716140013	0.473904899	0.99135833	ENSG00000124370	MCEE
76.50488275	0.069635056	0.408166945	0.170604348	0.864534879	0.999467288	ENSG00000280088	AC126474.2
75.73502772	-0.549809256	0.450543649	-1.220324062	0.222342052	0.956884001	ENSG00000160469	BRSK1
75.0476232	0.281744737	0.432333148	0.651684328	0.514604835	0.993513219	ENSG00000163923	RPL39L
75.84271392	0.209958511	0.495240816	0.423952358	0.671600559	0.997568624	ENSG00000135083	CCNJL
74.54553302	-0.715074839	0.478012795	-1.495932423	0.134671266	0.933741583	ENSG00000240065	PSMB9
74.34423604	-0.06045403	0.522145989	-0.115779939	0.90782695	0.999527751	ENSG00000102362	SYTL4
74.86657741	-0.072469029	0.540394628	-0.134103904	0.893320413	0.999467288	ENSG00000110446	SLC15A3
75.63858585	-0.057239	0.374243652	-0.152945814	0.878441007	0.999467288	ENSG00000108590	MED31

74.88828602	1.735940742	0.904253833	1.919749388	0.054889563	0.847598083	ENSG00000105011	ASF1B	
77.7107917	0.90909934	0.625836419	1.452614954	0.146330692	0.936017041	ENSG00000162409	PRKAA2	
77.71285801	0.757909684	0.606351316	1.249951413	0.211317297	0.954310328	ENSG00000263535	AK4P1	
76.00127962	2.92899506	1.407612247	2.080825218	0.037449907	0.790190505	ENSG00000143228	NUF2	
76.38808473	-0.018446688	0.474162187	-0.038903751	0.968967126	0.999527751	ENSG00000204147	ASAH2B	
76.20786247	-0.162338627	0.369291149	-0.439595229	0.660230297	0.997568624	ENSG00000160193	WDR4	
74.57024785	-0.380045438	0.43146882	-0.880817848	0.378416418	0.982609538	ENSG00000105617	LENG1	
75.68255159	-0.227819133	0.415642017	-0.548113817	0.583613754	0.99483168	ENSG00000146350	TBC1D32	
75.69999742	-0.125805082	0.375760279	-0.334801438	0.737774876	0.999467288	ENSG00000123388	HOXC11	
75.47430773	-0.211370739	0.380734585	-0.555165585	0.578781406	0.99483168	ENSG00000183655	KLHL25	
76.47413194	0.49153521	0.468966002	1.048125468	0.294580804	0.970706863	ENSG00000251369	ZNF550	
74.89797642	-0.392848028	0.387700101	-1.013278115	0.310927338	0.973427483	ENSG00000078319	PMS2P1	
75.14815108	-0.514210752	0.465658238	-1.104266414	0.269477588	0.962465655	ENSG00000204442	FAM155A	
74.95600939	2.282988681	1.372392601	1.663509902	0.09621041	0.921147998	ENSG00000121152	NCAPH	
75.43753764	-0.162759161	0.396793054	-0.410186518	0.68166913	0.998869317	ENSG00000164430	CGAS	
75.68533046	1.795067574	0.824504972	2.177145845	0.029469687	0.773201806	ENSG00000111665	CDCA3	
75.96418145	0.537744779	0.377791199	1.423391495	0.154622697	0.939432741	ENSG00000128000	ZNF780B	
75.38863446	-0.128799734	0.356912172	-0.36087235	0.718194874	0.99900742	ENSG00000161558	TMEM143	
75.60928751	0.552516875	0.458056381	1.206220233	0.227732584	0.958865115	ENSG00000120458	MSANTD2	
75.03604994	-0.342849508	0.393994209	-0.870189206	0.384197014	0.985655313	ENSG00000149929	HIRIP3	
72.34915683	-0.657662418	0.556616156	-1.181536704	0.237389581	0.962465655	ENSG00000108387		4-Sep
72.34386635	-0.93119392	0.794336562	-1.1722914	0.241080083	0.962465655	ENSG00000158246	TENT5B	
75.06214547	0.678792121	0.42601237	1.593362467	0.1110789	0.923922314	ENSG00000162687	KCNT2	
75.68392791	-0.939109264	0.424859744	-2.210398319	0.02707753	0.765324405	ENSG00000027075	PRKCH	
73.16530539	0.727637568	0.643471952	1.130799199	0.258139617	0.962465655	ENSG00000115041	KCNIP3	
75.65087208	-0.552619886	0.365717024	-1.511058685	0.1307735	0.929373153	ENSG00000144061	NPHP1	
74.50941627	-0.333743731	0.384944284	-0.866992304	0.385946227	0.986238739	ENSG00000167637	ZNF283	
74.92333436	-0.034284793	0.371952286	-0.092175246	0.926558805	0.999527751	ENSG00000159079	CFAP298	
76.91765042	-1.938319939	1.284095755	-1.509482397	0.13117555	0.930077797	ENSG00000115844	DLX2	
78.88402068	0.605198334	0.907040521	0.66722304	0.504629676	0.993513219	ENSG00000136541	ERMN	
73.87759949	1.125857849	0.741271755	1.5188193	0.128807986	0.928627271	ENSG00000167513	CDT1	
75.35355448	0.387389111	0.396191484	0.977782529	0.328181894	0.976026345	ENSG00000132773	TOE1	
75.76453231	-0.037579223	0.632655312	-0.059399206	0.952634145	0.999527751	ENSG00000118507	AKAP7	
73.69142354	-0.448315967	0.407059034	-1.101353684	0.270742755	0.962465655	ENSG00000175482	POLD4	
76.91609781	-1.050212631	0.509569903	-2.060978531	0.039305088	0.793985738	ENSG00000138311	ZNF365	
73.68115636	0.158903173	0.516225724	0.307817232	0.758221408	0.999467288	ENSG00000151131	C12orf45	
74.75393055	-3.046851349	1.535347867	-1.984469718	0.047203501	0.827156057	ENSG00000185269	NOTUM	
74.71928375	0.155622835	0.399755696	0.389294852	0.697058044	0.998869317	ENSG00000164221	CCDC112	
74.15617158	-0.025833913	0.357750682	-0.072212058	0.942433149	0.999527751	ENSG00000176024	ZNF613	
73.35888923	0.106024582	0.425274415	0.249308631	0.803122055	0.999467288	ENSG00000119943	PYROXD2	
73.40433436	1.942276288	1.797425805	1.08058774	0.279880539	0.963267117	ENSG00000118193	KIF14	
72.66370912	-0.097302164	0.445376596	-0.218471659	0.827061638	0.999467288	ENSG00000228305	AC016734.1	
74.84718742	-0.126114191	0.383958234	-0.328458097	0.742565322	0.999467288	ENSG00000198406	BZW1P2	
73.65043441	-0.768813235	0.79794343	-0.963493409	0.33529997	0.977237217	ENSG00000169116	PARM1	
73.21327909	-0.099186018	0.386606	-0.256555817	0.797521687	0.999467288	ENSG00000130119	GNL3L	
73.80068839	-0.611936462	0.589840263	-1.037461328	0.29952091	0.972186492	ENSG00000119699	TGFB3	
73.44617377	0.151568289	0.425575221	0.356149234	0.721728808	0.99900742	ENSG00000006740	ARHGAP44	
74.07264513	0.305297397	0.38182417	0.799575879	0.423956567	0.991301167	ENSG00000179909	ZNF154	
73.4798626	0.033471493	0.351012991	0.095356849	0.92403139	0.999527751	ENSG00000169087	HSPBAP1	
74.24261606	-0.14708912	0.444616973	-0.330822097	0.740778869	0.999467288	ENSG00000246695	RASSF8-AS1	
74.08607942	-0.339059285	0.356272473	-0.951685328	0.341256593	0.977691382	ENSG00000204253	HNRNPCP2	
73.68542423	-0.499136995	0.354828956	-1.406697468	0.159517117	0.939986013	ENSG00000221930	FAM45BP	
75.1162347	-0.190202533	0.497875379	-0.382028398	0.702440301	0.998869317	ENSG00000083812	ZNF324	
74.03922228	0.32111971	0.394241493	0.814525401	0.415344027	0.990226751	ENSG00000197808	ZNF461	
72.14775021	0.050282878	0.535948905	0.093820284	0.925251919	0.999527751	ENSG00000137628	DDX60	
72.95032639	0.228844703	0.363665575	0.629272384	0.529170748	0.99483168	ENSG00000176731	C8orf59	
73.00906826	-0.379579565	0.434812154	-0.872973678	0.382677425	0.98555814	ENSG00000203993	ARRDC1-AS1	
72.77171887	-0.782391691	0.497107868	-1.573887162	0.115513538	0.923922314	ENSG00000267106	ZNF561-AS1	
74.40914932	0.87952487	0.60825967	1.4459694	0.148185785	0.936396974	ENSG00000276141	WHAMMP3	
72.50854522	0.015943592	0.409469376	0.038937202	0.968940456	0.999527751	ENSG00000072954	TMEM38A	
74.00917883	0.769910135	0.624998614	1.231858948	0.218001779	0.956884001	ENSG00000203760	CENPW	
73.54249511	-0.005075478	0.368354149	-0.013778799	0.989006457	0.999527751	ENSG00000135913	USP37	
73.5760279	-0.587201435	0.616008374	-0.953236125	0.340470447	0.977691382	ENSG00000160963	COL26A1	
73.30849497	-0.014632571	0.382574681	-0.03824762	0.969490253	0.999527751	ENSG00000165644	COMTD1	

72.47319541	-0.390638914	0.472406665	-0.826912367	0.408286734	0.990226751	ENSG00000125966	MMP24
72.71242771	0.150618481	0.569882069	0.264297632	0.791550585	0.999467288	ENSG00000186684	CYP27C1
70.96008922	1.031691314	0.539767075	1.911363924	0.055957829	0.847598083	ENSG00000178031	ADAMTSL1
72.14131392	0.493811857	0.504792872	0.978246492	0.327952427	0.976026345	ENSG00000178878	APOLD1
72.72561771	-0.205335921	0.347128064	-0.591527859	0.554166794	0.99483168	ENSG00000196981	WDR5B
73.55046608	0.506515664	0.395899555	1.279404479	0.200754657	0.954310328	ENSG00000164099	PRSS12
73.15022962	0.793905825	0.396932682	2.000101932	0.045489258	0.813244956	ENSG00000196263	ZNF471
72.34077536	0.124998409	0.445897163	0.280330129	0.779224237	0.999467288	ENSG00000165209	STRBP
72.37655995	0.107877945	0.406239981	0.26555226	0.790584056	0.999467288	ENSG00000139192	TAPBPL
73.67566542	2.866383681	1.825875205	1.569868342	0.116445744	0.923922314	ENSG00000117650	NEK2
71.85766583	-0.405977319	0.56803181	-0.714708774	0.474789015	0.99135833	ENSG00000135378	PRRG4
71.80617924	-0.092851807	0.405535605	-0.228960926	0.818899286	0.999467288	ENSG00000204954	C12orf73
72.24223711	0.034880745	0.343336719	0.101593401	0.919079418	0.999527751	ENSG00000189298	ZKSCAN3
70.9736581	0.001215246	0.483179717	0.002515101	0.997993242	0.999527751	ENSG00000167840	ZNF232
74.12061308	-0.165315654	0.47732193	-0.346339951	0.729087248	0.999467288	ENSG00000081818	PCDHB4
72.64643849	0.630154918	0.420364142	1.499069153	0.133855693	0.933741583	ENSG00000185920	PTCH1
72.1753815	-1.194645363	0.432533653	-2.761970904	0.00574536	0.499617804	ENSG00000148219	ASTN2
71.60249362	0.459630904	0.446476723	1.029462188	0.303262539	0.972793733	ENSG00000100321	SYNGR1
72.747685	-0.09175107	0.376983347	-0.243382289	0.807709263	0.999467288	ENSG00000162927	PUS10
73.1258608	-0.247522	0.371942497	-0.665484589	0.505740597	0.993513219	ENSG00000166394	CYB5R2
72.97801003	-0.352870602	0.403575678	-0.874360427	0.381922001	0.985369679	ENSG00000083817	ZNF416
71.56683339	0.701983291	0.42110261	1.667012442	0.095511931	0.921147998	ENSG00000174791	RIN1
70.73460521	0.3431761	0.420371504	0.816363852	0.414292064	0.990226751	ENSG00000111850	SMIM8
74.90872305	-0.811091716	0.791469605	-1.024791996	0.305461344	0.973427483	ENSG00000091622	PITPNM3
69.82999935	0.662058401	0.62619919	1.057264863	0.290390725	0.968170184	ENSG00000115232	ITGA4
72.33960013	0.38559492	0.361562742	1.066467517	0.286212355	0.964826902	ENSG00000111877	MCM9
71.36548354	0.22967997	0.529862729	0.433470704	0.664672845	0.997568624	ENSG00000172348	RCAN2
71.12518511	0.286535575	0.429154702	0.667674323	0.504341504	0.993513219	ENSG00000185596	WASH3P
71.25597775	-0.19596652	0.376837631	-0.520029062	0.603043319	0.99483168	ENSG00000224831	TMEM183B
69.93606452	1.149314932	0.537932944	2.136539404	0.032635473	0.775963115	ENSG00000118971	CCND2
72.39774099	-0.249513511	0.405052066	-0.616003551	0.537892176	0.99483168	ENSG00000259642	ST20-AS1
71.40209929	-0.379653017	0.396112472	-0.958447521	0.337837142	0.977691382	ENSG00000118894	EEF2KMT
71.80224079	-0.452564416	0.42823167	-1.056821455	0.290593081	0.968621247	ENSG00000233830	EIF4HP1
71.29946852	-0.487099551	0.402583357	-1.209934645	0.226303972	0.958865115	ENSG00000174276	ZNHIT2
71.64120509	0.283010239	0.39775653	0.711516261	0.476764378	0.99135833	ENSG00000031691	CENPQ
71.59449432	-0.179877612	0.433123357	-0.41530342	0.677919798	0.998869317	ENSG00000258102	MAP1LC3B2
74.21629845	-0.275865343	0.517724376	-0.532842098	0.59414289	0.99483168	ENSG00000248334	WHAMMP2
71.6661242	0.639540244	0.445249159	1.436364854	0.150898549	0.936396974	ENSG00000277149	TYW1B
72.71051582	-1.158160181	0.488964681	-2.368596802	0.017855707	0.726053908	ENSG00000061455	PRDM6
68.86917709	0.629901618	0.631747307	0.997078438	0.318726438	0.974768742	ENSG00000126016	AMOT
72.46636351	-0.273107992	0.440159194	-0.620475491	0.534944785	0.99483168	ENSG00000225210	DUXAP9
70.91641852	0.433070986	0.415541325	1.042185121	0.29732586	0.971995789	ENSG00000155085	AK9
69.70087846	3.03815228	1.341712676	2.264383675	0.023550527	0.744948012	ENSG00000165244	ZNF367
71.49755308	-0.941066439	0.469499536	-2.004403341	0.045026872	0.813244956	ENSG00000007255	TRAPPC6A
71.15438343	0.209843961	0.422983011	0.496104938	0.619820375	0.99483168	ENSG00000236088	COX10-AS1
70.60109617	0.059698632	0.413004185	0.144547281	0.885068324	0.999467288	ENSG00000203965	EFCAB7
70.76144607	-0.34846163	0.396136567	-0.87965025	0.379048808	0.982778604	ENSG00000214106	PAXIP1-AS2
71.6260603	0.28148979	0.475202614	0.59235741	0.55361128	0.99483168	ENSG00000117242	PINK1-AS
70.29742525	0.61079031	0.415521471	1.46993682	0.141578866	0.935644597	ENSG00000158169	FANCC
71.77965222	-0.128920995	0.458789305	-0.281002616	0.778708393	0.999467288	ENSG00000169951	ZNF764
70.41483425	-0.277371012	0.560837112	-0.49456608	0.620906451	0.99483168	ENSG00000064989	CALCRL
71.59017951	-0.23016355	0.372285424	-0.618244861	0.536413943	0.99483168	ENSG00000189042	ZNF567
71.72798702	0.507536859	0.4092823	1.240065499	0.214951169	0.954310328	ENSG00000113971	NPHP3
71.03427049	-0.111013493	0.387790214	-0.286272034	0.774669776	0.999467288	ENSG00000197044	ZNF441
70.36718758	0.568489962	0.402399207	1.4127512	0.157728891	0.939464191	ENSG00000076641	PAG1
69.53023042	0.761017701	0.56789331	1.340071608	0.180222066	0.948941777	ENSG00000134222	PSRC1
72.59088502	-1.680426102	0.884663693	-1.899508384	0.057497665	0.852306368	ENSG00000047617	ANO2
70.08357926	-0.187670318	0.402312097	-0.46647943	0.640872368	0.996553586	ENSG00000234380	LINC01426
71.38767564	0.486627229	0.521103689	0.933839539	0.350386671	0.977691382	ENSG00000223960	AC009948.1
69.84236542	0.163523878	0.421452729	0.388000521	0.698015648	0.998869317	ENSG00000006638	TBXA2R
70.11871344	0.494762595	0.443227733	1.116271746	0.264305832	0.962465655	ENSG00000160352	ZNF714
71.30402218	-0.00926753	0.44769544	-0.020700523	0.983484552	0.999527751	ENSG00000263002	ZNF234
69.26401369	0.660866405	0.448420011	1.473766534	0.140544462	0.935644597	ENSG00000001631	KRIT1
69.85394823	-0.40843353	0.617016192	-0.661949452	0.508003617	0.993513219	ENSG00000188312	CENPP

70.62420415	2.105455839	1.582424384	1.330525401	0.183345223	0.949094286	ENSG00000165480	SKA3
70.17996064	0.779936308	0.382525414	2.038913699	0.041458641	0.800562177	ENSG00000171291	ZNF439
70.00397648	-0.256002199	0.392121624	-0.652864273	0.513843783	0.993513219	ENSG00000081692	JMJD4
70.95094146	0.519826107	0.665040616	0.781645654	0.434422849	0.991301167	ENSG00000125848	FLRT3
69.92999132	0.007714999	0.457026924	0.016880842	0.986531676	0.999527751	ENSG00000230629	RPS23P8
70.07819947	0.616541539	0.418871216	1.471911928	0.141044662	0.935644597	ENSG00000141391	PRELID3A
69.43839506	-0.212087149	0.400491691	-0.529566913	0.59641224	0.99483168	ENSG00000241506	PSMC1P1
68.98258474	-0.113064812	0.399678685	-0.282889271	0.777261719	0.999467288	ENSG00000171097	KYAT1
69.7522685	-0.170361778	0.451972159	-0.376929806	0.70622577	0.99900742	ENSG00000148908	RGS10
71.80643037	-0.356310954	0.549247857	-0.648725253	0.516515979	0.993513219	ENSG00000137821	LRRC49
70.085249	0.010479689	0.463480454	0.022610854	0.981960686	0.999527751	ENSG00000164542	KIAA0895
70.72390641	0.074418018	0.384182861	0.193704678	0.846407128	0.999467288	ENSG00000175741	RWDD4P2
70.54570919	-0.662262433	0.415515801	-1.593832129	0.110973639	0.923922314	ENSG00000285410	GABPB1-IT1
68.72530883	0.229787895	0.418457101	0.549131307	0.582915341	0.99483168	ENSG00000103061	SLC7A6OS
70.56191519	0.040402747	0.558577177	0.072331539	0.942338066	0.999527751	ENSG00000178531	CTXN1
70.69944302	0.186310233	0.423930746	0.43948271	0.660311808	0.997568624	ENSG00000171295	ZNF440
71.02356711	1.024087904	0.615044812	1.665062259	0.095900335	0.921147998	ENSG00000257354	AC048341.1
70.01400788	0.08857612	0.41550163	0.213178755	0.831187539	0.999467288	ENSG00000104129	DNAJC17
69.72044484	0.586891747	0.463311217	1.266733302	0.205250668	0.954310328	ENSG00000271425	NBPF10
69.98074195	0.036168382	0.48514304	0.074551996	0.94057117	0.999527751	ENSG00000163472	TMEM79
70.37517325	-0.413451317	0.407062674	-1.015694496	0.30977489	0.973427483	ENSG00000197927	C2orf27A
70.63959782	0.076101515	0.431735452	0.176268858	0.860082726	0.999467288	ENSG00000284976	BX255925.3
70.37955745	0.754897628	0.49919512	1.512229583	0.130475468	0.929373153	ENSG00000161996	WDR90
69.93053076	-0.115913491	0.379635385	-0.305328469	0.760115996	0.999467288	ENSG00000203666	EFCAB2
68.92840969	0.836442764	0.587356023	1.424081361	0.154422921	0.939432741	ENSG00000138376	BARD1
69.74257018	-0.05662876	0.35797918	-0.158190095	0.874307009	0.999467288	ENSG00000105865	DUS4L
68.63704112	-0.329481652	0.405300105	-0.812932559	0.416256726	0.990226751	ENSG00000213366	GSTM2
68.61114083	-0.374373878	0.575806698	-0.650172843	0.515580581	0.993513219	ENSG00000188985	DHFRP1
69.87270366	-0.31937066	0.44031451	-0.725323952	0.468253315	0.99135833	ENSG00000131115	ZNF227
70.69394915	0.773455666	0.420516317	1.83930001	0.065871072	0.872086825	ENSG00000169914	OTUD3
69.81349058	2.597415964	0.875408816	2.96708911	0.003006338	0.402042054	ENSG00000184661	CDCA2
69.11638288	0.079342949	0.44765694	0.177240521	0.859319469	0.999467288	ENSG00000107614	TRDMT1
70.14160768	1.353128806	0.838292689	1.614148404	0.10649524	0.922165836	ENSG00000131737	KRT34
69.2680903	-0.571778602	0.532849453	-1.07305844	0.283244896	0.963267117	ENSG00000101605	MYOM1
68.33568412	0.544690671	0.586132343	0.92929639	0.352735505	0.977691382	ENSG00000141682	PMAIP1
68.8470133	0.534665718	0.47807431	1.118373664	0.263407437	0.962465655	ENSG00000187266	EPOR
69.65643528	0.316824864	0.400611637	0.790852872	0.429029853	0.991301167	ENSG00000189164	ZNF527
68.42368816	-0.31739733	0.406016759	-0.781734555	0.434370589	0.991301167	ENSG00000185838	GNB1L
72.52251588	-0.523850607	0.738815048	-0.709041604	0.47829866	0.99135833	ENSG00000236609	ZNF853
69.26968232	-0.584533429	0.3510012	-1.665331711	0.095846595	0.921147998	ENSG00000156345	CDK20
69.97331728	-0.916731306	0.429823609	-2.132808174	0.032940465	0.775963115	ENSG00000095397	WHRN
68.8789428	0.624167809	0.484087002	1.289371139	0.19726909	0.954310328	ENSG00000138134	STAMBPL1
68.59279215	-0.042939779	0.486505156	-0.088261713	0.929668669	0.999527751	ENSG00000138185	ENTPD1
69.60015691	0.474155705	0.417758466	1.134999632	0.256375473	0.962465655	ENSG00000177465	ACOT4
70.55144942	-1.011334741	0.480392264	-2.105226952	0.035271557	0.784512596	ENSG00000240376	AC010343.1
68.62621354	-0.792333277	0.440204421	-1.799921217	0.071873079	0.884210289	ENSG00000213866	YBX1P10
68.9591279	0.188925235	0.412540434	0.457955679	0.646984283	0.997368416	ENSG00000095637	SORBS1
69.11367817	0.757902007	0.369098209	2.05338847	0.040034917	0.794441233	ENSG00000002016	RAD52
69.58845344	0.344240349	0.452330744	0.761036816	0.446635078	0.99135833	ENSG00000115392	FANCL
70.22805298	0.064046638	0.463080091	0.138305747	0.889998783	0.999467288	ENSG00000124613	ZNF391
71.42287711	1.435653244	0.914814684	1.569337779	0.116569254	0.923922314	ENSG00000088340	FER1L4
68.27383149	-0.128166151	0.456851345	-0.280542351	0.779061438	0.999467288	ENSG00000198841	KTI12
68.89075684	0.599299984	0.369003346	1.624104472	0.104353514	0.92169898	ENSG00000170915	PAQR8
68.89256555	-0.116781213	0.358174129	-0.326045919	0.744389613	0.999467288	ENSG00000077454	LRCH4
69.65575044	-0.06128695	0.386992167	-0.158367417	0.874167287	0.999467288	ENSG00000083814	ZNF671
69.69841989	0.551511326	0.476322636	1.157852439	0.246924259	0.962465655	ENSG00000116574	RHOU
68.30793236	-0.073218654	0.407489465	-0.179682324	0.857401971	0.999467288	ENSG00000163322	ABRAXAS1
69.17146812	0.184108395	0.419540448	0.43883348	0.660782199	0.997568624	ENSG00000101004	NINL
67.99745135	-0.463847346	0.413258649	-1.122414129	0.2616864	0.962465655	ENSG00000177868	SVBP
69.25571588	0.899811591	0.405248874	2.220392576	0.026392131	0.763890866	ENSG00000160796	NBEAL2
68.3915501	0.400910133	0.408713232	0.980908132	0.326638047	0.976026345	ENSG00000173214	MFSD4B
67.89014475	0.357143192	0.492628503	0.724974681	0.468467563	0.99135833	ENSG00000167702	KIFC2
68.27732149	-0.270309824	0.562049147	-0.480936277	0.630561789	0.99483168	ENSG00000204682	CASC10
66.83837415	-0.287936759	0.610141972	-0.471917639	0.636985577	0.99653524	ENSG00000173947	PIFO

68.29882622	1.486304773	0.694804526	2.139169676	0.032421929	0.775963115	ENSG00000091651	ORC6	
70.3079338	1.271985301	0.526589329	2.415516669	0.015712903	0.696728876	ENSG00000276550	HERC2P2	
67.73702937	0.513422208	0.661414036	0.776249338	0.43760178	0.991301167	ENSG00000167703	SLC43A2	
67.37517851	0.338519924	0.418264235	0.809344658	0.418316926	0.990226751	ENSG00000101442	ACTR5	
68.30842107	-0.154364586	0.494761363	-0.311998061	0.755041997	0.999467288	ENSG00000198270	TMEM116	
69.10240804	0.235595949	0.423628241	0.55613844	0.578116219	0.99483168	ENSG00000145723	GIN1	
67.30832375	0.009467048	0.441785694	0.02142905	0.982903401	0.999527751	ENSG00000185442	FAM174B	
67.64168879	0.820763232	0.747028169	1.098704528	0.271896967	0.962465655	ENSG00000136492	BRIP1	
68.40350785	0.162830994	0.558254308	0.291678886	0.770532154	0.999467288	ENSG00000223459	TCAF1P1	
67.16390342	0.496162603	0.55284413	0.897472861	0.369466648	0.981455968	ENSG00000173588	CEP83	
67.05344778	0.340578594	0.651502501	0.522758691	0.60114219	0.99483168	ENSG00000184988	TMEM106A	
68.87296404	1.243349191	0.557255799	2.231200092	0.025667878	0.760619373	ENSG00000159259	CHAF1B	
68.11512099	0.850969976	0.442790221	1.921835526	0.054626457	0.847598083	ENSG00000138696	BMPR1B	
68.62481638	0.873955473	0.479169337	1.823896911	0.068167659	0.87420149	ENSG00000145908	ZNF300	
68.79868568	-0.252560727	0.435274572	-0.580233129	0.561757415	0.99483168	ENSG00000176826	FKBP9P1	
67.7048456	0.421344907	0.616779472	0.683137047	0.494520248	0.993513219	ENSG00000198331	HYLS1	
69.15751934	0.090181774	0.442688079	0.203714032	0.838576976	0.999467288	ENSG00000176476	SGF29	
68.24823128	0.219229539	0.369560114	0.593217533	0.553035581	0.99483168	ENSG00000181896	ZNF101	
68.07757822	0.141870221	0.386506881	0.367057426	0.713576183	0.99900742	ENSG00000186205		1-Mar
66.57638005	1.79677549	1.430652755	1.255913068	0.20914749	0.954310328	ENSG00000151725	CENPU	
68.01324875	-0.071901746	0.447390035	-0.160713786	0.872318831	0.999467288	ENSG00000135537	AFG1L	
67.75812801	-0.133496901	0.398880286	-0.334679115	0.737867158	0.999467288	ENSG00000182310	SPACA6	
67.46448745	1.154057668	0.440307297	2.621027802	0.008766511	0.604581883	ENSG00000184465	WDR27	
68.80038324	-0.396709329	0.563112122	-0.704494388	0.481124944	0.991525194	ENSG00000151090	THRB	
68.55065735	0.277037955	0.453228936	0.6112539	0.541031505	0.99483168	ENSG00000103995	CEP152	
67.44530329	-0.666635089	0.388662404	-1.71520343	0.086307936	0.901307093	ENSG00000248008	NRAV	
67.06898079	-0.857575129	0.436653767	-1.963970527	0.04953351	0.836346249	ENSG00000223891	OSER1-DT	
66.46676372	0.222599827	0.458401865	0.485599741	0.62725098	0.99483168	ENSG00000130958	SLC35D2	
66.99185029	0.358659944	0.401369029	0.893591479	0.371540507	0.981955904	ENSG00000110536	PTPMT1	
65.70043391	0.160728578	0.585875867	0.274338964	0.783824144	0.999467288	ENSG00000165323	FAT3	
65.66928759	0.190951208	0.402111154	0.474871702	0.634878422	0.995506752	ENSG00000130775	THEMIS2	
67.10083131	0.850330383	0.386889521	2.19786357	0.02795883	0.769167109	ENSG00000258634	AL160006.1	
66.93497549	0.608560157	0.445760661	1.365217279	0.172184769	0.944422693	ENSG00000014914	MTMR11	
67.52559472	0.141280854	0.419396105	0.336867348	0.736216901	0.999467288	ENSG00000149292	TTC12	
66.71699423	-0.458885098	0.584429334	-0.785184917	0.432345157	0.991301167	ENSG00000137142	IGFBPL1	
67.00884567	-0.597816392	0.357427615	-1.672552337	0.094415457	0.921147998	ENSG00000108641	B9D1	
66.00935173	0.137012669	0.478875759	0.286113186	0.774791432	0.999467288	ENSG00000205763	RP9P	
67.25164899	-0.278379514	0.410263306	-0.678538662	0.497430219	0.993513219	ENSG00000257698	AC084033.3	
68.49049357	-0.961761374	0.44417048	-2.165297824	0.030364885	0.775963115	ENSG00000008277	ADAM22	
66.80950318	2.060760277	1.391808292	1.480635148	0.138703824	0.934605957	ENSG00000186185	KIF18B	
66.70422447	1.324981248	0.897503502	1.476296465	0.139864323	0.935644597	ENSG00000123219	CENPK	
67.68909657	0.071014019	0.419127084	0.169433143	0.865455956	0.999467288	ENSG00000167595	PROSER3	
67.15364917	0.229384703	0.403034092	0.569144665	0.569257969	0.99483168	ENSG00000197774	EME2	
67.71143841	0.151738686	0.663658366	0.228639755	0.819148923	0.999467288	ENSG00000183458	AC138932.1	
67.04837524	0.16584648	0.369887522	0.448370033	0.653886165	0.997527562	ENSG00000134780	DAGLA	
65.80465747	0.628754802	0.381901069	1.646381363	0.099685268	0.921147998	ENSG00000160949	TONSL	
66.2304183	0.371572741	0.49875187	0.745005209	0.456268571	0.99135833	ENSG00000172613	RAD9A	
66.40752141	-0.841735624	0.574811694	-1.464367605	0.143093534	0.935644597	ENSG00000179277	MEIS3P1	
67.34796073	-0.268385937	0.458300227	-0.585611617	0.55813654	0.99483168	ENSG00000186300	ZNF555	
67.11061783	0.536832014	0.546673949	0.981996701	0.326101473	0.976026345	ENSG00000279457	FO538757.1	
65.84703463	0.058562116	0.410546061	0.142644447	0.886570993	0.999467288	ENSG00000174599	TRAM1L1	
67.00459479	0.254931373	0.461434897	0.552475278	0.580622768	0.99483168	ENSG00000253537	PCDHGA7	
65.73778512	-0.257052066	0.391085087	-0.65727913	0.511001453	0.993513219	ENSG00000175928	LRRN1	
64.8168757	-0.568880587	0.562884674	-1.010652117	0.312182962	0.973427483	ENSG00000154310	TNIK	
66.32052481	-0.463933236	0.445953359	-1.04031784	0.298192258	0.972186492	ENSG00000132881	CPLANE2	
66.63103611	-0.219168364	0.378901053	-0.578431658	0.562972725	0.99483168	ENSG00000212664	AC064799.1	
65.55057503	-0.366616672	0.458570817	-0.799476675	0.424014066	0.991301167	ENSG00000125898	FAM110A	
65.29842704	-0.486344334	0.571738559	-0.85064113	0.394968734	0.988378925	ENSG00000090661	CERS4	
65.74020699	0.072463706	0.392983379	0.18439382	0.853704518	0.999467288	ENSG00000105866	SP4	
67.273329	0.839571631	0.501006315	1.675770558	0.093783143	0.921147998	ENSG00000110031	LPXN	
66.38593182	-0.090099861	0.450661142	-0.199928178	0.841536753	0.999467288	ENSG00000144504	ANKMY1	
65.8877823	0.516467069	0.406734626	1.269788791	0.204159875	0.954310328	ENSG00000176222	ZNF404	
65.02798197	0.23568123	0.57141593	0.412451277	0.680008686	0.998869317	ENSG00000163364	LINC01116	
64.84449029	-0.058820703	0.58183636	-0.101094924	0.919475107	0.999527751	ENSG00000164649	CDCA7L	

65.93413521	-0.58287634	0.418699148	-1.392112553	0.163888318	0.943223078	ENSG00000161243	FBXO27
65.45192169	0.025479453	0.406194528	0.062727218	0.949983723	0.999527751	ENSG00000179922	ZNF784
65.47564449	-1.764379101	1.41121951	-1.250251353	0.211207743	0.954310328	ENSG00000100302	RASD2
65.37909783	-1.100514668	0.437022151	-2.518212553	0.011795212	0.648356243	ENSG00000270872	SRGAP2D
66.31110337	0.58705692	0.471909113	1.244004202	0.213498017	0.954310328	ENSG00000196476	C20orf96
66.77569543	0.007853679	0.459481728	0.017092472	0.986362844	0.999527751	ENSG00000163596	ICA1L
64.42911494	-0.191591776	0.501900232	-0.38173279	0.702659576	0.998869317	ENSG00000168273	SMIM4
64.58657557	0.338398319	0.473271586	0.715019302	0.474597116	0.99135833	ENSG00000181804	SLC9A9
65.69776996	-0.534350234	0.409097344	-1.306168918	0.19149512	0.949261383	ENSG00000141642	ELAC1
65.97770984	-0.210599157	0.515543832	-0.408499033	0.68290734	0.998869317	ENSG00000142611	PRDM16
65.10089992	-0.318266376	0.427441627	-0.744584419	0.45652299	0.99135833	ENSG00000205464	ATP6AP1L
66.11275359	-0.003932492	0.437661237	-0.008985242	0.992830911	0.999527751	ENSG00000285976	AL135905.2
64.77355896	-1.22749468	0.485506519	-2.528276416	0.011462407	0.64668571	ENSG00000103274	NUBP1
65.92521097	-0.171028876	0.471190943	-0.362971485	0.716626186	0.99900742	ENSG00000243364	EFNA4
64.88867163	-0.326993467	0.518901678	-0.630164597	0.5285869	0.99483168	ENSG00000133111	RFXAP
65.34345683	-0.570456245	0.41683467	-1.36854318	0.171142116	0.944422693	ENSG00000132801	ZSWIM3
65.18362782	-0.32694491	0.440030564	-0.743005003	0.457478652	0.99135833	ENSG00000185730	ZNF696
65.02784567	0.083036045	0.401784315	0.206668209	0.836268987	0.999467288	ENSG00000127328	RAB3IP
64.01873131	1.835682801	0.858182901	2.139034463	0.032432877	0.775963115	ENSG00000214357	NEURL1B
64.95353894	0.224030101	0.405700851	0.552205155	0.580807803	0.99483168	ENSG00000137343	ATAT1
64.70984136	0.224558075	0.658710624	0.3409055	0.733174724	0.999467288	ENSG00000146409	SLC18B1
64.935244	0.141448687	0.475341508	0.297572766	0.766029263	0.999467288	ENSG00000106025	TSPAN12
64.41575845	0.451593149	0.456814238	0.988570653	0.322873251	0.975337095	ENSG00000162073	PAQR4
65.13699261	-0.370567344	0.41240845	-0.898544498	0.368895333	0.981455968	ENSG00000230383	AC009245.1
65.29637513	-0.357922013	0.703032893	-0.509111333	0.610674189	0.99483168	ENSG00000169933	FRMPD4
64.43324004	-0.024606656	0.380444946	-0.064678626	0.948429881	0.999527751	ENSG00000123119	NECAB1
64.2234989	-0.695187448	0.46540587	-1.493722992	0.135248037	0.933741583	ENSG00000136425	CIB2
64.50792614	0.222246426	0.396302306	0.560800234	0.574933727	0.99483168	ENSG00000159212	CLIC6
63.65255283	0.313249112	0.412754624	0.758923324	0.447898426	0.99135833	ENSG00000243477	NAA80
62.17140581	0.28313057	0.773678082	0.365953976	0.714399419	0.99900742	ENSG00000111335	OAS2
64.55608912	-1.03738327	0.48127771	-2.155477487	0.031124488	0.775963115	ENSG00000139410	SDSL
63.34292791	0.590402775	0.459252004	1.285574737	0.198591518	0.954310328	ENSG00000263528	IKBKE
63.69188859	0.180628958	0.517153716	0.349275181	0.726882729	0.999467288	ENSG00000221988	PPT2
64.2492065	0.29628624	0.413592594	0.716372208	0.473761551	0.99135833	ENSG00000176225	RTTN
63.8468688	0.135337498	0.376206171	0.359742896	0.71903941	0.99900742	ENSG00000189190	ZNF600
66.22160511	-0.145537031	0.63494276	-0.229212836	0.818703496	0.999467288	ENSG00000271643	AC112220.2
63.97389379	-0.525725604	0.450700761	-1.16646265	0.243427441	0.962465655	ENSG00000178458	H3F3AP6
65.18814296	0.67831196	0.547028426	1.239993988	0.214977618	0.954310328	ENSG00000185684	EP400P1
63.44382637	0.151515443	0.637905159	0.237520328	0.812253151	0.999467288	ENSG00000168404	MLKL
67.29460587	-1.355551883	0.783958858	-1.729110998	0.083789231	0.901307093	ENSG00000272168	CASC15
64.47698344	0.851111025	0.493678123	1.724020137	0.084704208	0.901307093	ENSG00000153933	DGKE
63.52224876	0.155525863	0.472030767	0.329482469	0.741791042	0.999467288	ENSG00000243667	WDR92
63.29760582	-0.130486248	0.58712098	-0.222247634	0.824121115	0.999467288	ENSG00000132170	PPARG
64.13865541	-0.098573756	0.536487823	-0.183739037	0.854218183	0.999467288	ENSG00000187824	TMEM220
63.97010328	-0.198801149	0.513891051	-0.386854662	0.698863807	0.998869317	ENSG00000164167	LSM6
59.82795848	1.733917443	0.840887605	2.062008564	0.039206921	0.793985738	ENSG00000255364	SMILR
62.95787598	0.609139325	0.546735867	1.114138219	0.265219895	0.962465655	ENSG00000008311	AASS
63.13999705	0.412540487	0.405275848	1.017925172	0.308713518	0.973427483	ENSG00000275764	AC092747.4
62.12246661	0.143992322	0.431671611	0.333569125	0.738704719	0.999467288	ENSG00000205476	CCDC85C
63.83384891	-0.165957348	0.452822522	-0.366495349	0.713995482	0.99900742	ENSG00000265681	RPL17
63.65706616	0.234510819	0.412421638	0.568619096	0.569614663	0.99483168	ENSG00000146021	KLHL3
63.64047671	-0.213487753	0.473553537	-0.450820733	0.652118757	0.997449432	ENSG00000130347	RTN4IP1
63.85972112	-0.112810704	0.516501287	-0.218413209	0.827107174	0.999467288	ENSG00000184903	IMMP2L
62.5338366	-0.362169013	0.410733275	-0.881762046	0.3779055	0.982609538	ENSG00000204922	UQCC3
62.79295628	0.748380013	0.521121831	1.436094151	0.150975552	0.936396974	ENSG00000053747	LAMA3
63.68670832	0.010876141	0.462012289	0.023540805	0.98121889	0.999527751	ENSG00000256294	ZNF225
62.98171207	-0.231985618	0.478648866	-0.484667643	0.627912122	0.99483168	ENSG00000187244	BCAM
64.20619683	0.002011517	0.464777438	0.004327914	0.996546835	0.999527751	ENSG00000169228	RAB24
62.39608501	0.231104279	0.544763495	0.424228644	0.671399073	0.997568624	ENSG00000187764	SEMA4D
64.19048064	1.565676431	0.58505384	2.676123674	0.007447916	0.563130099	ENSG00000198113	TOR4A
64.11223009	-0.057536923	0.442862235	-0.12992059	0.896629254	0.999467288	ENSG00000175356	SCUBE2
63.11654346	0.087086949	0.420539947	0.207083655	0.835944527	0.999467288	ENSG00000087299	L2HGDH
61.36145038	0.398512153	0.603612214	0.660212208	0.509117659	0.993513219	ENSG00000108771	DHX58
63.65331866	0.038505969	0.418748754	0.091954826	0.926733931	0.999527751	ENSG00000181873	IBA57

62.60240457	-0.417976403	0.442417547	-0.944755482	0.344783725	0.977691382	ENSG00000147439	BIN3
62.91848168	-0.018561814	0.399751905	-0.046433334	0.962964869	0.999527751	ENSG00000119723	COQ6
63.02842292	0.732659726	0.463072311	1.582171312	0.113610467	0.923922314	ENSG00000103037	SETD6
62.85666367	0.810892462	0.424526684	1.910109522	0.056119113	0.847598083	ENSG00000173041	ZNF680
63.04449528	0.375737558	0.423733781	0.886730241	0.375224153	0.982609538	ENSG00000198300	PEG3
63.58259213	-0.083342444	0.387741871	-0.21494311	0.829811673	0.999467288	ENSG00000167981	ZNF597
63.42200523	-0.161960479	0.414159448	-0.391058273	0.695754163	0.998869317	ENSG00000267317	AC027307.2
62.59726074	0.44333313	0.383042578	1.157399086	0.247109347	0.962465655	ENSG00000105072	C19orf44
63.60617072	0.703064514	0.467380582	1.504265564	0.132513002	0.933741583	ENSG00000253731	PCDHGA6
61.73755095	1.790117896	0.89656978	1.996629751	0.045865418	0.815583282	ENSG00000131153	GINS2
62.57059766	0.029582888	0.415197933	0.071250084	0.943198722	0.999527751	ENSG00000152763	WDR78
62.8393471	-0.040062111	0.369929349	-0.108296654	0.913760377	0.999527751	ENSG00000204685	STARD7-AS1
63.05691045	0.436681186	0.501402825	0.870918879	0.383798448	0.985655313	ENSG00000113108	APBB3
63.06236997	0.005928686	0.458682318	0.012925473	0.989687252	0.999527751	ENSG00000239789	MRPS17
61.32983741	-0.279807484	0.669727446	-0.417793068	0.67609842	0.998869317	ENSG00000126785	RHOJ
62.12631554	0.309110382	0.381237081	0.810808804	0.417475478	0.990226751	ENSG00000141013	GAS8
63.94350039	0.189696203	0.489590554	0.387458871	0.698416528	0.998869317	ENSG00000176809	LRRC37A3
62.92022901	-0.428418297	0.450502208	-0.95097935	0.341614858	0.977691382	ENSG00000132819	RBM38
62.85010322	0.449849893	0.644952783	0.697492754	0.485494473	0.992558339	ENSG00000173114	LRRN3
61.73927326	-0.279412794	0.565150727	-0.494404024	0.621020872	0.99483168	ENSG00000283154	IQCJ-SCHIP1
62.27123022	-0.254352224	0.406017831	-0.626455799	0.53101602	0.99483168	ENSG00000165060	FXN
62.04656576	0.025423675	0.451917392	0.05625735	0.955136795	0.999527751	ENSG00000169330	KIAA1024
62.51601485	-0.571367174	0.409349125	-1.395794296	0.162776449	0.943223078	ENSG00000196605	ZNF846
62.56892824	-0.054268965	0.474000533	-0.114491358	0.908848297	0.999527751	ENSG00000120318	ARAP3
61.93988771	0.66986125	0.410356063	1.632390283	0.102597295	0.92169898	ENSG00000152457	DCLRE1C
64.18650774	-0.511867906	0.50810106	-1.007413576	0.31373607	0.973633446	ENSG00000156218	ADAMTSL3
62.40590133	-0.032059742	0.420907895	-0.076168069	0.939285386	0.999527751	ENSG00000175984	DENND2C
62.24700382	-0.051177361	0.503146079	-0.101714717	0.91898312	0.999527751	ENSG00000157064	NMNAT2
61.30438905	-0.272955965	0.533770556	-0.511373214	0.609089747	0.99483168	ENSG00000166337	TAF10
62.18962571	-0.58973544	0.455183675	-1.295598838	0.19511373	0.953843167	ENSG00000259146	AC005476.2
63.68649924	-1.150152606	0.809342648	-1.421094773	0.155289209	0.939464191	ENSG00000143217	NECTIN4
62.11202891	0.115353155	0.555499831	0.207656507	0.83549718	0.999467288	ENSG00000235770	LINC00607
61.17355044	-1.115712534	0.846995869	-1.31725853	0.187751981	0.949094286	ENSG00000068078	FGFR3
61.09278316	-0.448551711	0.648909106	-0.691239662	0.489414943	0.993513219	ENSG00000228232	GAPDHP1
61.85324577	0.198647685	0.363502959	0.546481618	0.58473492	0.99483168	ENSG00000180257	ZNF816
62.23454985	0.117955635	0.461023836	0.255855827	0.798062165	0.999467288	ENSG00000081853	PCDHGA2
62.66264556	-0.454328358	0.426217485	-1.065954294	0.286444304	0.96503734	ENSG00000253305	PCDHGB6
61.98252781	-0.37987059	0.416248977	-0.91260426	0.361450713	0.979465649	ENSG00000175764	TTLL11
62.167546	0.784203114	0.686360531	1.142552753	0.253224332	0.962465655	ENSG00000111247	RAD51AP1
60.9266546	-0.119807695	0.483806148	-0.247635743	0.804416253	0.999467288	ENSG00000235423	AC068768.1
61.85822283	0.521785854	0.432345905	1.206871274	0.227481722	0.958865115	ENSG00000120662	MTRF1
61.5847568	0.996577699	0.67118693	1.484799024	0.137597071	0.934144427	ENSG00000101003	GINS1
62.41070475	0.165104922	0.46745335	0.353200852	0.723937869	0.999456245	ENSG00000161914	ZNF653
61.70923964	0.148812607	0.476653384	0.31220298	0.754886267	0.999467288	ENSG00000124787	RPP40
60.59270508	-1.770928356	0.643669803	-2.75129942	0.005935936	0.499617804	ENSG00000198523	PLN
60.70622726	0.774314435	0.511743062	1.513092201	0.13025624	0.929373153	ENSG00000079102	RUNX1T1
62.09674968	-0.132470886	0.386658151	-0.342604664	0.731895893	0.999467288	ENSG00000165275	TRMT10B
63.09608402	-0.221053347	0.717082888	-0.308267497	0.757878796	0.999467288	ENSG00000157445	CACNA2D3
60.73940677	-0.080677265	0.419497497	-0.192318823	0.847492475	0.999467288	ENSG00000104689	TNFRSF10A
61.52477215	0.650024615	0.382057528	1.701378896	0.088871862	0.910413547	ENSG00000171316	CHD7
62.50951783	0.048803136	0.428387175	0.113922962	0.909298863	0.999527751	ENSG00000269001	AC092070.2
61.7150707	0.307244909	0.530011532	0.579694763	0.562120475	0.99483168	ENSG00000226210	WASH8P
61.03915659	0.505584459	0.453050894	1.115955108	0.264441351	0.962465655	ENSG00000120832	MTERF2
61.37067632	-0.252249794	0.413804572	-0.609586775	0.542135574	0.99483168	ENSG00000151338	MIPOL1
61.94597048	1.459068877	0.529939442	2.753274737	0.005900236	0.499617804	ENSG00000166922	SCG5
59.54633322	0.743851662	0.585457809	1.270546998	0.203889853	0.954310328	ENSG00000102096	PIM2
60.89110805	0.20650504	0.549527075	0.375786835	0.707075376	0.99900742	ENSG00000151117	TMEM86A
62.04008282	-0.090119495	0.455058755	-0.198039251	0.843014354	0.999467288	ENSG00000172671	ZFAND4
61.07323037	0.161228393	0.421972946	0.382082298	0.702400322	0.998869317	ENSG00000135587	SMPD2
66.22769367	-0.867227993	1.032050435	-0.840296136	0.400742367	0.988492517	ENSG00000147642	SYBU
61.43231498	0.603354885	0.429682062	1.404189142	0.160262534	0.941607907	ENSG00000214765	SEPT7P2
61.62573087	0.012684318	0.440797617	0.028775833	0.977043376	0.999527751	ENSG00000142065	ZFP14
61.30359719	-0.171628242	0.409976573	-0.418629389	0.675487008	0.998869317	ENSG00000197863	ZNF790
61.64273257	0.077712512	0.427948837	0.181592998	0.855902145	0.999467288	ENSG00000106100	NOD1

60.6053189	-1.117653119	0.8213454	-1.360758969	0.173589873	0.944422693	ENSG00000240583	AQP1
61.7115061	-0.185846065	0.520708584	-0.356909932	0.721159234	0.99900742	ENSG00000231764	DLX6-AS1
61.78160391	1.940421343	0.590727704	3.284798276	0.001020554	0.23031036	ENSG00000139618	BRCA2
59.81169972	1.973684648	1.41907533	1.390824438	0.164278671	0.943223078	ENSG00000127564	PKMYT1
60.38704006	-0.064357322	0.424743373	-0.151520485	0.879565153	0.999467288	ENSG00000158062	UBXN11
59.90113364	0.048539355	0.461042183	0.105281808	0.916152198	0.999527751	ENSG00000164011	ZNF691
59.9094788	-0.928830377	0.452663402	-2.051922846	0.040177161	0.795004104	ENSG00000283196	AC006453.2
60.25164975	-0.030939509	0.468861481	-0.065988592	0.947386908	0.999527751	ENSG00000182263	FIGN
60.87972114	0.540955736	0.406436596	1.330972016	0.183198219	0.949094286	ENSG00000185485	SDHAP1
60.35798862	0.677351827	0.454809421	1.489309138	0.136405983	0.934144427	ENSG00000235043	TECRP1
61.1371105	0.254947785	0.481484892	0.529503188	0.596456434	0.99483168	ENSG00000272335	AC093297.2
63.40234242	-1.621053525	0.656890981	-2.467766449	0.013595899	0.672931175	ENSG00000187243	MAGED4B
61.11827416	0.108603227	0.425456392	0.255262887	0.798520062	0.999467288	ENSG00000234743	EIF5AP4
60.99248313	0.045304983	0.465467391	0.097332238	0.922462555	0.999527751	ENSG00000227124	ZNF717
60.46490402	-0.454867067	0.441042757	-1.031344602	0.302379249	0.972793733	ENSG00000127080	IPPK
60.78849928	-0.128041606	0.411214396	-0.311374328	0.755516067	0.999467288	ENSG00000115137	DNAJC27
61.03721793	0.170055202	0.464718598	0.36593156	0.714416146	0.99900742	ENSG00000120327	PCDHB14
59.32239648	0.338920215	0.66353188	0.510782111	0.609503637	0.99483168	ENSG00000140807	NKD1
60.40018692	2.469056842	1.493711477	1.652967711	0.098337435	0.921147998	ENSG00000178999	AURKB
60.13347965	0.242896859	0.596070807	0.407496654	0.683643251	0.998869317	ENSG00000143434	SEMA6C
60.77844205	0.638284543	0.454617848	1.404002386	0.160318139	0.941607907	ENSG00000183814	LIN9
60.65774062	1.271197268	0.855444716	1.486007505	0.137277136	0.934144427	ENSG00000142731	PLK4
61.93816646	-0.652699651	0.539982141	-1.208743032	0.226761584	0.958865115	ENSG00000276291	FRG1HP
60.72302561	0.879864176	0.413703961	2.126796593	0.033436988	0.775963115	ENSG00000204514	ZNF814
60.0398317	-0.435196356	0.425072269	-1.023817331	0.305921563	0.973427483	ENSG00000172456	FGGY
59.95921731	-0.107697574	0.529834129	-0.203266586	0.838926671	0.999467288	ENSG00000162999	DUSP19
60.22686815	-0.746745666	0.630624288	-1.18413718	0.236358765	0.962465655	ENSG00000144488	ESPNL
60.08862723	1.008259557	0.550466662	1.831645085	0.067004315	0.87420149	ENSG00000186166	CCDC84
60.13458226	0.311104738	0.441942534	0.703948396	0.481464911	0.991525194	ENSG00000182796	TMEM198B
61.17047852	0.36742969	0.461541868	0.796091786	0.425978688	0.991301167	ENSG00000254681	PKD1P5
60.32594613	0.927977355	0.435756447	2.12957803	0.033206466	0.775963115	ENSG00000159055	MIS18A
58.95204893	-0.415247912	0.530167415	-0.783239219	0.433486649	0.991301167	ENSG00000125657	TNFSF9
59.90103072	0.273656726	0.383166135	0.714198621	0.475104369	0.99135833	ENSG00000130544	ZNF557
60.95485604	0.234412129	0.404049387	0.580157121	0.561808667	0.99483168	ENSG00000197857	ZNF44
59.48226767	0.615285059	0.445997454	1.379570788	0.167718837	0.943223078	ENSG00000172687	ZNF738
60.13023435	0.714623521	0.43776608	1.632432373	0.102588434	0.92169898	ENSG00000188315	C3orf62
60.65483288	0.573139481	0.399030383	1.436330429	0.150908339	0.936396974	ENSG00000022567	SLC45A4
59.33346362	0.693102979	0.402023899	1.724034272	0.084701656	0.901307093	ENSG00000102967	DHODH
59.93676049	0.389166107	0.470234257	0.827600501	0.407896784	0.990226751	ENSG00000198182	ZNF607
59.54628859	1.047178612	0.463877649	2.25744572	0.023980238	0.744948012	ENSG00000104177	MYEF2
60.64653751	0.254664868	0.503089117	0.5062023	0.61271464	0.99483168	ENSG00000156869	FRRS1
59.48094163	-0.21770972	0.432185294	-0.503741619	0.614442958	0.99483168	ENSG00000184857	TMEM186
59.62010541	-0.319508458	0.451546991	-0.707586284	0.479202213	0.99135833	ENSG00000262879	AC068152.1
59.65373424	0.084402195	0.425248165	0.198477506	0.842671482	0.999467288	ENSG00000232098	AC012313.1
59.76901416	0.258514139	0.449493795	0.575122821	0.565208235	0.99483168	ENSG00000166831	RBPMS2
60.73573903	1.247302021	0.462620021	2.696169563	0.007014195	0.549501061	ENSG00000267680	ZNF224
57.67822527	1.003158868	0.751975692	1.334030978	0.182193704	0.949094286	ENSG00000157111	TMEM171
59.37140806	-0.081488001	0.433492846	-0.187980036	0.850892301	0.999467288	ENSG00000146243	IRAK1BP1
58.94630908	0.327421956	0.773994025	0.423029048	0.672274068	0.9977039	ENSG00000154529	CNTNAP3B
59.78560969	0.330708675	0.417649819	0.79183244	0.428458384	0.991301167	ENSG00000167523	SPATA33
58.54066442	0.178275123	0.550107027	0.324073523	0.745882371	0.999467288	ENSG00000121897	LIAS
59.98451103	-0.15284988	0.448989001	-0.340431234	0.7335318	0.999467288	ENSG00000164758	MED30
59.54873233	0.22031675	0.384783777	0.572572868	0.566933935	0.99483168	ENSG00000258429	PDF
58.84766254	-0.218333634	0.450675508	-0.484458618	0.628060426	0.99483168	ENSG00000166582	CENPV
59.36381225	-0.550704574	0.484931139	-1.135634588	0.256109527	0.962465655	ENSG00000166896	ATP23
61.49499976	-0.555613767	0.671490554	-0.827433482	0.40799141	0.990226751	ENSG00000102445	RUBCNL
59.45813613	-0.070459098	0.520928429	-0.135256772	0.892408862	0.999467288	ENSG00000143554	SLC27A3
58.79636789	0.513922656	0.403009836	1.275211198	0.202234499	0.954310328	ENSG00000162836	ACP6
58.9309081	1.42647057	0.544319768	2.620648107	0.00877628	0.604581883	ENSG00000003137	CYP26B1
60.09980732	-0.032709102	0.414332273	-0.078944132	0.937077061	0.999527751	ENSG00000250571	GLI4
59.37642014	-0.136167393	0.437170805	-0.311474123	0.755440212	0.999467288	ENSG00000181690	PLAG1
59.71292425	-0.67821332	0.443570754	-1.528985657	0.126268	0.928627271	ENSG00000134461	ANKRD16
59.98184306	0.920760085	0.61533295	1.496360769	0.134559667	0.933741583	ENSG00000129467	ADCY4
57.45082619	0.931422962	0.76101052	1.223929154	0.220978954	0.956884001	ENSG00000030419	IKZF2

60.0378329	0.728077331	0.469126997	1.551983441	0.120666186	0.923922314	ENSG00000197182	MIRLET7BHG
59.04203695	0.316030013	0.425277476	0.743114862	0.457412143	0.99135833	ENSG00000185946	RNPC3
58.94175572	-0.008622342	0.525891564	-0.016395665	0.986918738	0.999527751	ENSG00000169621	APLF
58.4049319	-0.296395304	0.428428371	-0.691819971	0.489050393	0.993513219	ENSG00000146232	NFKBIE
59.79495218	-0.479822747	0.417192733	-1.150122496	0.250093422	0.962465655	ENSG00000196152	ZNF79
58.22158877	0.204278061	0.401280102	0.509066012	0.610705955	0.99483168	ENSG00000187609	EXD3
58.37339274	-0.153376012	0.436911328	-0.351046088	0.725553773	0.999467288	ENSG00000197162	ZNF785
58.12819205	-0.59445658	0.464616204	-1.27945727	0.200736077	0.954310328	ENSG00000144120	TMEM177
61.63098972	-0.746341554	0.691030915	-1.08004076	0.28012403	0.963267117	ENSG00000132530	XAF1
60.03883662	-0.084354016	0.498559782	-0.169195389	0.865642956	0.999467288	ENSG00000042429	MED17
58.76143664	-0.588714595	0.594115873	-0.990908714	0.321730157	0.975337095	ENSG00000123364	HOXC13
59.54971906	0.222289046	0.441873426	0.503060454	0.614921769	0.99483168	ENSG00000158805	ZNF276
59.1329852	-0.026839267	0.45930343	-0.05843472	0.95340236	0.999527751	ENSG00000203326	ZNF525
59.01267919	-0.746710435	0.522620638	-1.428780994	0.15306719	0.939432741	ENSG00000244586	WNT5A-AS1
58.51032665	0.191778138	0.44468668	0.431265757	0.666275146	0.997568624	ENSG00000283498	MIR1244-2
59.62242086	-0.43714325	0.409050232	-1.068678649	0.285214495	0.964493786	ENSG00000187626	ZKSCAN4
59.69237063	0.589477816	0.538151961	1.09537428	0.273352697	0.962465655	ENSG00000106089	STX1A
59.2621917	0.912100809	0.426800877	2.137064045	0.032592783	0.775963115	ENSG00000104218	CSPP1
58.81163424	-0.399839719	0.434998135	-0.919175708	0.358003675	0.979427314	ENSG00000151743	AMN1
58.06975975	-0.892868279	0.433489987	-2.059720655	0.039425254	0.793985738	ENSG00000259431	THTPA
57.46788198	-0.278669329	0.514560433	-0.541567737	0.588116323	0.99483168	ENSG00000176928	GCNT4
59.13050675	0.264859754	0.528074527	0.501557526	0.615978803	0.99483168	ENSG00000079335	CDC14A
57.36547572	-0.292121071	0.561119091	-0.520604405	0.60264238	0.99483168	ENSG00000276600	RAB7B
57.89405426	-0.532387291	0.436520373	-1.219616137	0.222610426	0.956884001	ENSG00000126953	TIMM8A
59.04865579	0.588635064	0.534580197	1.10111648	0.270845965	0.962465655	ENSG00000156876	SASS6
59.46587617	-0.191974388	0.581390899	-0.330198475	0.741249999	0.999467288	ENSG00000078295	ADCY2
58.49168091	0.728265116	0.442305122	1.646522005	0.099656333	0.921147998	ENSG00000158987	RAPGEF6
58.28317277	0.161788322	0.450976984	0.358750729	0.719781575	0.99900742	ENSG00000140905	GCSH
57.1245486	0.460691781	0.530167524	0.868955114	0.384871682	0.985733881	ENSG00000072201	LNX1
57.43820585	-0.157679862	0.433916979	-0.363387168	0.716315686	0.99900742	ENSG00000197779	ZNF81
57.82140089	0.969406196	0.566925409	1.709936052	0.087277699	0.90492251	ENSG00000186767	SPIN4
58.55382156	0.287908944	0.767506559	0.375122454	0.707569399	0.99900742	ENSG00000149346	SLX4IP
58.07203764	0.15512543	0.436326653	0.355525909	0.722195638	0.99906455	ENSG00000282057	AC092807.3
58.53494366	-0.008044485	0.468162323	-0.01718311	0.986290536	0.999527751	ENSG00000168038	ULK4
58.80457961	0.270688583	0.486108377	0.556848217	0.577631137	0.99483168	ENSG00000112852	PCDHB2
58.13075331	0.410344118	0.392977658	1.044191978	0.296396578	0.970706863	ENSG00000204947	ZNF425
57.11832743	0.008661669	0.571326664	0.015160625	0.987904035	0.999527751	ENSG00000164920	OSR2
58.63366756	0.808225762	0.684881155	1.180096366	0.23796189	0.962465655	ENSG00000081181	ARG2
58.07566249	0.293736534	0.678644352	0.432828377	0.665139456	0.997568624	ENSG00000125869	LAMP5
57.77825773	0.28443977	0.490010581	0.580476792	0.561593133	0.99483168	ENSG00000172889	EGFL7
58.89119833	0.660676269	0.500893648	1.318995104	0.187170749	0.949094286	ENSG00000196268	ZNF493
57.62424581	-0.168025013	0.579717963	-0.289839238	0.771939228	0.999467288	ENSG00000196693	ZNF33B
57.15221754	0.22901564	0.474371298	0.482777185	0.629253952	0.99483168	ENSG00000167562	ZNF701
58.95486615	-0.30399626	2.167163663	-0.14027379	0.888443672	0.999467288	ENSG00000019505	SYT13
58.70586356	0.011195401	0.450282229	0.024863075	0.98016418	0.999527751	ENSG00000138336	TET1
57.12990597	-0.120243175	0.658614738	-0.182569821	0.855135565	0.999467288	ENSG00000061918	GUCY1B1
57.74717671	0.713223811	0.785860731	0.907570239	0.364105324	0.98048242	ENSG00000185480	PARPBP
61.60710928	-2.55823774	0.743120075	-3.442563086	0.000576229	0.195790358	ENSG00000196557	CACNA1H
57.8822236	0.207618005	0.559367679	0.371165536	0.710514241	0.99900742	ENSG00000185761	ADAMTSL5
53.05114101	0.435863975	0.962191822	0.45299073	0.650555417	0.997381078	ENSG00000106809	OGN
58.09949303	0.766407723	0.442441929	1.732222179	0.083234012	0.901307093	ENSG00000133805	AMPD3
54.86853316	-1.663688261	0.901319655	-1.845835994	0.064916023	0.868202292	ENSG00000133392	MYH11
58.3620525	2.14707633	0.846498341	2.536421191	0.011199195	0.644117389	ENSG00000264112	AC015813.1
58.26175828	0.22291583	0.492840393	0.452308361	0.651046854	0.997449432	ENSG00000198346	ZNF813
57.0466174	0.085400291	0.561677325	0.152045111	0.879151356	0.999467288	ENSG00000174842	GLMN
58.52099487	0.612723252	0.642088539	0.954265985	0.339949021	0.977691382	ENSG00000169031	COL4A3
57.37551873	0.242917126	0.758864222	0.320106178	0.748887842	0.999467288	ENSG00000125089	SH3TC1
58.08862115	-0.596610158	0.443887455	-1.344057263	0.17892987	0.9469453	ENSG00000169220	RGS14
56.60350651	-0.426224159	0.47860564	-0.890553983	0.373168496	0.982329093	ENSG00000121988	ZRANB3
57.03315821	0.108004097	0.445692524	0.242328716	0.808525465	0.999467288	ENSG00000174007	CEP19
57.74220029	-0.067303798	0.399254651	-0.168573612	0.866132038	0.999467288	ENSG00000215375	MYL5
56.52762718	-0.786155974	0.546406042	-1.438776137	0.15021396	0.936396974	ENSG00000196544	BORCS6
57.65335831	0.090667606	0.450559073	0.20123356	0.840515951	0.999467288	ENSG00000105708	ZNF14
56.70556016	1.477955252	0.743241644	1.988525891	0.046753558	0.82348981	ENSG00000144554	FANCD2

57.44801259	-0.050903447	0.510380197	-0.099736329	0.920553658	0.999527751	ENSG00000245958	AC093752.1
57.81673779	-0.004268812	0.48604146	-0.008782815	0.992992418	0.999527751	ENSG00000260231	KDM7A-DT
56.01074241	-0.443791757	0.633208518	-0.700861951	0.483389172	0.991892545	ENSG00000110881	ASIC1
56.87975507	-0.952002846	0.646297703	-1.473009793	0.140748395	0.935644597	ENSG00000104081	BMF
56.91656563	0.82341096	0.534426586	1.540737271	0.123380742	0.927794614	ENSG00000146281	PM20D2
57.57491665	0.740002168	0.444456327	1.664960365	0.095920664	0.921147998	ENSG00000226445	BX322234.1
57.78854596	-0.158783046	0.466912477	-0.340070256	0.73380362	0.999467288	ENSG00000111199	TRPV4
57.67738593	-0.27001937	0.757709844	-0.356362494	0.721569113	0.99900742	ENSG00000261105	LMO7-AS1
57.40165248	-0.240863117	0.38602967	-0.623949752	0.532660585	0.99483168	ENSG00000172171	TEFM
57.33024056	-1.016590954	1.638044432	-0.620612563	0.534854571	0.99483168	ENSG00000017427	IGF1
56.82812208	-0.084910416	0.462461907	-0.183605211	0.854323175	0.999467288	ENSG00000196793	ZNF239
57.41908853	-0.266719061	0.402822669	-0.66212525	0.507890955	0.993513219	ENSG00000073584	SMARCE1
56.18340403	0.309070374	0.445028099	0.694496313	0.487371012	0.993513219	ENSG00000167554	ZNF610
56.98238296	1.03872787	0.553114299	1.877962426	0.060386305	0.858996918	ENSG00000162825	NBPF20
56.35601008	-1.3693678	0.582161043	-2.352214764	0.018661997	0.726053908	ENSG00000271303	SRXN1
56.4959642	2.041848477	0.519954397	3.926976076	8.60E-05	0.067295389	ENSG00000162706	CADM3
55.22259731	0.067807314	0.639450244	0.106040015	0.915550604	0.999527751	ENSG00000156299	TIAM1
55.95602874	-0.070327725	0.456945326	-0.153908401	0.87768196	0.999467288	ENSG00000279267	AL078621.3
55.1505923	0.688018428	0.57304819	1.200629266	0.229895043	0.959575179	ENSG00000078401	EDN1
54.83097194	0.577547259	0.59838114	0.965182926	0.334453197	0.976026345	ENSG00000165959	CLMN
56.11306459	0.395247149	0.55000261	0.71862777	0.472370299	0.99135833	ENSG00000158201	ABHD3
54.90016811	-1.528831996	0.708863234	-2.156737607	0.031026115	0.775963115	ENSG00000175920	DOK7
55.92307692	0.163530285	0.460922302	0.354789266	0.72274747	0.999104299	ENSG00000196511	TPK1
56.7304783	2.118028086	0.46044867	4.599922261	4.23E-06	0.015260786	ENSG00000215252	GOLGA8B
56.28668542	-0.492164895	0.447350201	-1.100178104	0.271254529	0.962465655	ENSG00000182318	ZSCAN22
55.96600493	0.375008316	0.450949381	0.831597364	0.405636251	0.990045328	ENSG00000152939	MARVELD2
54.80804156	0.984707146	0.647816064	1.520041261	0.128500606	0.928627271	ENSG00000143631	FLG
55.37704674	-0.606271578	0.576449376	-1.051734294	0.292921472	0.970706863	ENSG00000239779	WBP1
56.8767212	-0.154897303	0.483206296	-0.320561433	0.748542768	0.999467288	ENSG00000171045	TSNARE1
56.06128885	0.086874038	0.568278273	0.152872355	0.878498937	0.999467288	ENSG00000139567	ACVRL1
56.30212196	0.727408043	0.551267834	1.319518385	0.186995868	0.949094286	ENSG00000187790	FANCM
56.91846156	-0.161817247	0.431108133	-0.375351876	0.70739879	0.99900742	ENSG00000022976	ZNF839
56.51739819	-1.091625047	0.496280938	-2.199611073	0.027834501	0.769167109	ENSG00000103522	IL21R
54.97054005	-0.032493651	0.484129955	-0.067117622	0.946488065	0.999527751	ENSG00000167705	RILP
56.4390419	0.535057864	0.593794232	0.90108296	0.367544212	0.981269214	ENSG00000095015	MAP3K1
55.23445104	-0.054111557	0.544066213	-0.099457669	0.920774896	0.999527751	ENSG00000120256	LRP11
55.41676082	-0.190391104	0.449556142	-0.423509069	0.671923883	0.997568624	ENSG00000198546	ZNF511
55.47008587	0.074192777	0.433109333	0.171302651	0.863985796	0.999467288	ENSG00000167549	CORO6
55.89171197	-0.331241818	0.438163675	-0.75597736	0.44966277	0.99135833	ENSG00000166292	TMEM100
57.23954934	1.927042512	1.033753768	1.86412139	0.062304659	0.866923116	ENSG00000198576	ARC
56.35998752	-0.16802926	0.466237524	-0.360394115	0.718552427	0.99900742	ENSG00000131849	ZNF132
55.67701969	0.239045331	0.427366076	0.559345593	0.575925885	0.99483168	ENSG00000261799	AC007406.5
56.09866028	0.167475212	0.436150327	0.383985065	0.700989517	0.998869317	ENSG00000170100	ZNF778
55.78300436	-0.670284918	0.44055163	-1.521467344	0.1281426	0.928627271	ENSG00000144134	RABL2A
55.43109762	-0.441589648	0.485312022	-0.909908733	0.362870644	0.98048242	ENSG00000229647	MYOSLID
56.13921477	0.198326287	0.755748584	0.26242363	0.792994856	0.999467288	ENSG00000214110	LDHAP4
55.28478818	-0.249516893	0.407799427	-0.611861804	0.540629195	0.99483168	ENSG00000203780	FANK1
55.55997389	-0.416419688	0.515271649	-0.808155639	0.419000991	0.990226751	ENSG00000121316	PLBD1
54.97989939	0.077790832	0.42473012	0.183153557	0.854677534	0.999467288	ENSG00000268516	AC020915.3
55.97748746	0.015587095	0.439548079	0.035461638	0.971711635	0.999527751	ENSG00000187815	ZFP69
55.24696221	0.706899517	0.48772193	1.449390468	0.147228569	0.936396974	ENSG00000183765	CHEK2
55.68886117	0.702872927	0.487879091	1.440670322	0.149677843	0.936396974	ENSG00000261662	AL359752.1
56.53795312	0.409413003	0.513835808	0.796777874	0.425580048	0.991301167	ENSG00000160766	GBAP1
55.65782607	-0.159206225	0.540563522	-0.294518995	0.768361351	0.999467288	ENSG00000153930	ANKFN1
54.35040431	0.00823466	0.853094446	0.009652694	0.992298384	0.999527751	ENSG00000144868	TMEM108
53.58999232	-0.330292348	0.60711779	-0.544033387	0.586418498	0.99483168	ENSG00000170214	ADRA1B
55.5314888	-0.240423598	0.427163565	-0.562837323	0.573545663	0.99483168	ENSG00000160813	PPP1R35
54.30493987	-0.406765982	0.488567039	-0.832569432	0.405087605	0.989962822	ENSG00000223865	HLA-DPB1
55.52438094	0.314275691	0.467099553	0.672823789	0.501059402	0.993513219	ENSG00000198521	ZNF43
55.2495827	-0.281680063	0.704736804	-0.399695406	0.689380876	0.998869317	ENSG00000261189	AL031058.1
55.0570995	0.715262567	0.409548252	1.746467146	0.080729811	0.897807954	ENSG00000101938	CHRDL1
53.78412851	0.799133689	0.640902395	1.246888287	0.212438462	0.954310328	ENSG00000103811	CTSH
55.96819935	-0.280734821	0.450444432	-0.623239628	0.533127064	0.99483168	ENSG00000186272	ZNF17
53.6670741	-0.043880371	0.561656186	-0.078126748	0.937727231	0.999527751	ENSG00000144355	DLX1

55.67783779	0.172091738	0.510701722	0.336971132	0.736138662	0.999467288	ENSG00000198205	ZXDA
54.35618506	0.481379758	0.412701066	1.166412684	0.243447633	0.962465655	ENSG00000166192	SENP8
55.27703657	0.441159416	0.40852129	1.079893329	0.280189684	0.963267117	ENSG00000108474	PIGL
53.55432404	0.69472832	0.613488366	1.132422975	0.25745665	0.962465655	ENSG00000028137	TNFRSF1B
55.2475231	-0.039200888	0.433526245	-0.090423333	0.927950816	0.999527751	ENSG00000196653	ZNF502
54.3781391	0.110047539	0.51546487	0.21349183	0.830943361	0.999467288	ENSG00000234851	RPL23AP42
54.29741329	-0.674602609	0.493332612	-1.36743972	0.17148752	0.944422693	ENSG00000272325	NUDT3
55.21204696	0.133017901	0.425050262	0.312946286	0.754321472	0.999467288	ENSG00000254598	CSNK2A3
55.17370902	-0.06422726	0.482912336	-0.132999833	0.894193512	0.999467288	ENSG00000184860	SDR42E1
54.32880669	-0.672310962	0.447838577	-1.501235037	0.133294781	0.933741583	ENSG00000254612	AP001000.1
55.66890377	0.102414271	0.531800018	0.192580421	0.84728758	0.999467288	ENSG00000148459	PDSS1
54.60287479	-0.678730097	0.485582887	-1.397763628	0.16218406	0.943223078	ENSG00000005379	TSPOAP1
53.83200539	0.420843043	0.460373559	0.914133826	0.36064653	0.979465649	ENSG00000258484	SPESP1
54.57270927	-0.451404491	0.483946127	-0.932757732	0.35094507	0.977691382	ENSG00000124243	BCAS4
54.36101735	0.352666217	0.409191006	0.861862093	0.388763411	0.986803154	ENSG00000156313	RPGR
53.61625324	0.020234025	0.912432988	0.022175902	0.98230764	0.999527751	ENSG00000188613	NANOS1
49.7957139	-1.696023755	2.599128647	-0.652535517	0.514055768	0.993513219	ENSG00000087250	MT3
53.88233309	0.673867161	0.416058387	1.619645659	0.105308415	0.92169898	ENSG00000189195	BTBD8
55.1296768	-0.206569004	0.486095432	-0.424955658	0.670869	0.997568624	ENSG00000215105	TTC3P1
53.25345327	-0.37014734	0.516978025	-0.715982735	0.47400201	0.99135833	ENSG00000247134	AC090204.1
54.85262011	-0.102632094	0.518066148	-0.198106158	0.842962007	0.999467288	ENSG00000214274	ANG
54.87366309	1.041442591	0.428317766	2.431471849	0.015037617	0.691682485	ENSG00000263956	NBPF11
54.03915334	0.181673994	0.472823134	0.384232455	0.700806165	0.998869317	ENSG00000116141	MARK1
53.26298063	-0.268548896	0.474258555	-0.566249977	0.57122386	0.99483168	ENSG00000225439	BOLA3-AS1
54.19587006	0.863156419	0.442352272	1.95128741	0.051022866	0.842188482	ENSG00000118939	UCHL3
50.80608813	-0.258995003	0.968943304	-0.267296345	0.789241001	0.999467288	ENSG00000100593	ISM2
53.19476554	-0.020118965	0.516193653	-0.038975615	0.96890983	0.999527751	ENSG00000183549	ACSM5
53.92414294	-0.022116093	0.470673784	-0.046988155	0.962522668	0.999527751	ENSG00000247626	MARS2
53.91458239	0.079315596	0.432414225	0.183425038	0.854464532	0.999467288	ENSG00000174740	PABPC5
53.64963574	0.63667638	0.48782459	1.305133841	0.191847277	0.949261383	ENSG00000148288	GBGT1
53.27858166	-0.192473605	0.498547075	-0.386069068	0.699445518	0.998869317	ENSG00000138621	PPCDC
53.17805455	0.090103619	0.542725105	0.166020731	0.868140636	0.999467288	ENSG00000164418	GRIK2
53.62061264	-0.17647384	0.397980961	-0.443422821	0.657459933	0.997568624	ENSG00000146757	ZNF92
53.03320096	0.002269501	0.485632648	0.004673288	0.996271269	0.999527751	ENSG00000275183	LENG9
53.89471624	0.180954591	0.447848286	0.404053329	0.686173504	0.998869317	ENSG00000186792	HYAL3
51.24200249	1.230578859	0.84017719	1.464665875	0.1430121	0.935644597	ENSG00000163884	KLF15
51.32565606	0.568088466	0.778191884	0.730010782	0.465383594	0.99135833	ENSG00000184349	EFNA5
52.80585002	-0.562366206	0.522766141	-1.075751014	0.282038626	0.963267117	ENSG00000269293	ZSCAN16-AS1
54.41634925	-0.300116356	0.416461779	-0.720633611	0.471134968	0.99135833	ENSG00000213189	BTF3L4P2
54.06371019	0.474590871	0.41135529	1.153724975	0.248612941	0.962465655	ENSG00000182774	RPS17
54.38277182	0.1278845	0.428776946	0.298254142	0.765509202	0.999467288	ENSG00000269343	ZNF587B
53.11538837	0.148390367	0.446467917	0.33236513	0.739613566	0.999467288	ENSG00000141570	CBX8
52.80040311	-0.312745573	0.529600217	-0.590531429	0.554834421	0.99483168	ENSG00000176531	PHLDB3
53.35279743	0.384004394	0.463386219	0.828691874	0.407278785	0.990226751	ENSG00000222011	FAM185A
53.86548687	-0.938394246	0.431208938	-2.176193868	0.029540767	0.773201806	ENSG00000231528	FAM225A
53.88100491	-0.753229362	0.446359043	-1.687496587	0.091507906	0.915903452	ENSG00000181274	FRAT2
54.18579273	0.097646693	0.604905578	0.161424687	0.871758924	0.999467288	ENSG00000273604	EPOP
54.90666854	0.976828324	0.538828682	1.81287366	0.06985131	0.878035218	ENSG00000015568	RGPD5
52.96676192	0.297696205	0.462859976	0.643166875	0.520115825	0.993513219	ENSG00000178222	RNF212
54.23416477	-0.085082191	0.455446644	-0.186810448	0.851809255	0.999467288	ENSG00000261534	AL596244.1
53.98770197	0.828181499	0.43041238	1.924158176	0.05433476	0.847598083	ENSG00000213390	ARHGAP19
53.28721384	-0.519200468	0.42269173	-1.228319437	0.219327077	0.956884001	ENSG00000235609	AF127577.4
53.35113355	0.219721803	0.409790234	0.536181158	0.591833354	0.99483168	ENSG00000146776	ATXN7L1
54.38056839	1.023824438	0.536297803	1.909059541	0.056254411	0.847598083	ENSG00000106701	FSD1L
52.95010928	0.091243799	0.415031428	0.219847926	0.825989593	0.999467288	ENSG00000138190	EXOC6
53.64872298	-0.030605001	0.468177665	-0.065370485	0.947879023	0.999527751	ENSG00000143443	C1orf56
53.84150928	0.31852079	0.498375768	0.63911773	0.522746346	0.994616524	ENSG00000106004	HOXA5
53.34441426	0.003521801	0.480829055	0.007324435	0.994155999	0.999527751	ENSG00000179523	EIF3J-DT
53.18396168	0.413780822	0.462372419	0.894908098	0.370836217	0.981849219	ENSG00000140563	MCTP2
52.98567132	0.557195831	0.510684068	1.091077372	0.275238839	0.962465655	ENSG00000115271	GCA
53.06489281	0.675043186	0.506138812	1.333711563	0.182298404	0.949094286	ENSG00000112619	PRPH2
55.18567616	0.575630383	0.566862412	1.015467547	0.309883009	0.973427483	ENSG00000250644	AC068580.4
54.47699068	1.898290976	1.617504861	1.173592131	0.240558435	0.962465655	ENSG00000121621	KIF18A
53.09886064	-0.07196367	0.414118181	-0.173775684	0.862041755	0.999467288	ENSG00000180423	HARBI1

54.37184496	0.349356129	0.543206165	0.643137269	0.520135034	0.993513219	ENSG00000239665	AL157392.3
53.16837391	-0.163179079	0.434025409	-0.375966649	0.706941691	0.99900742	ENSG00000148832	PAOX
53.91369237	0.674513132	0.45481254	1.483057461	0.138059145	0.934144427	ENSG00000196757	ZNF700
52.11233181	-0.089090213	0.513879965	-0.173367749	0.862362373	0.999467288	ENSG00000235501	AC105942.1
52.89505946	0.4915673	0.432909764	1.135495988	0.256167562	0.962465655	ENSG00000135637	CCDC142
53.84369441	-0.379256782	0.42011772	-0.902739313	0.366664267	0.981269214	ENSG00000226121	AHCTF1P1
53.33884138	0.040934736	0.416426443	0.098300041	0.921694046	0.999527751	ENSG00000171806	METTL18
51.98171311	0.386721697	0.525375137	0.736086787	0.461677883	0.99135833	ENSG00000188372	ZP3
53.60923687	-0.340474871	0.480608959	-0.708423895	0.478682058	0.99135833	ENSG00000127720	METTL25
52.79081994	-0.01986934	0.451782561	-0.043979875	0.964920446	0.999527751	ENSG00000056558	TRAF1
52.51857275	-0.298278804	0.419563608	-0.710926303	0.477129906	0.99135833	ENSG00000149016	TUT1
53.64061417	0.058520587	0.470627271	0.124345932	0.901041381	0.999527751	ENSG00000185947	ZNF267
52.60237368	-0.222509753	0.478402357	-0.465110069	0.641852636	0.996696873	ENSG00000228343	AC115618.2
54.42205061	0.564015768	0.554061197	1.017966555	0.30869385	0.973427483	ENSG00000139116	KIF21A
48.15752146	1.326051933	2.376796821	0.557915562	0.576902045	0.99483168	ENSG00000125378	BMP4
53.08348452	-0.538700114	0.49350162	-1.091587326	0.275014529	0.962465655	ENSG00000149090	PAMR1
53.23644199	1.047128367	0.48947358	2.139294969	0.032411787	0.775963115	ENSG00000159433	STARD9
52.83271961	-0.273425787	0.448149231	-0.610122182	0.541780874	0.99483168	ENSG00000183401	CCDC159
52.7253436	-0.37853322	0.514435356	-0.735822715	0.461838595	0.99135833	ENSG00000151778	SERP2
53.49049383	0.87136226	0.506230878	1.721274419	0.085201039	0.901307093	ENSG00000124224	PPP4R1L
53.02271086	-0.163785309	0.47831182	-0.342423713	0.732032046	0.999467288	ENSG00000196267	ZNF836
50.11035887	-2.152193637	0.941515452	-2.285882437	0.022261137	0.744905651	ENSG00000158050	DUSP2
52.83908202	-0.191492779	0.501481577	-0.381854066	0.702569613	0.998869317	ENSG00000177873	ZNF619
54.85590376	-0.346055963	0.833378939	-0.415244431	0.677962976	0.998869317	ENSG00000251320	AC011352.3
52.60840649	-0.331953944	0.566799137	-0.585664166	0.558101219	0.99483168	ENSG00000123453	SARDH
51.67699609	-1.626093362	0.545031753	-2.983483718	0.002849872	0.384679448	ENSG00000125864	BFSP1
52.88294953	0.218570752	0.598653881	0.365103708	0.715033993	0.99900742	ENSG00000131351	HAUS8
51.8435356	0.069106695	0.509820364	0.135551069	0.89217619	0.999467288	ENSG00000123810	B9D2
52.82233187	-0.209174259	0.455872263	-0.458844015	0.646346184	0.997368416	ENSG00000116668	SWT1
51.71432111	-0.517122313	0.705694	-0.732785475	0.463689282	0.99135833	ENSG00000242265	PEG10
52.13573237	0.161503684	0.413361144	0.39070843	0.696012767	0.998869317	ENSG00000073905	VDAC1P1
53.47927904	0.639963367	0.468091969	1.367174422	0.171570641	0.944422693	ENSG00000163462	TRIM46
52.16152886	1.065253005	0.45886168	2.321512237	0.020259214	0.73997555	ENSG00000137462	TLR2
52.84645677	-0.797969972	0.519765011	-1.535251422	0.124722082	0.927794614	ENSG00000164743	C8orf48
53.60895857	0.548072701	0.630920513	0.868687402	0.385018134	0.985733881	ENSG00000149243	KLHL35
52.51500425	2.147588962	0.984115848	2.182252186	0.029090926	0.773201806	ENSG00000154839	SKA1
51.49637321	-0.37829461	0.45003768	-0.840584305	0.400580854	0.988492517	ENSG00000130988	RGN
53.18637146	0.543725707	0.501324821	1.084577671	0.278108749	0.962465655	ENSG00000134627	PIWIL4
52.48283937	-0.227659017	0.398126138	-0.571826352	0.567439622	0.99483168	ENSG00000256393	RPL41P5
52.96864718	-0.295360943	0.575430756	-0.513286682	0.607750799	0.99483168	ENSG00000117016	RIMS3
51.70331385	-0.574416858	0.891796593	-0.644111968	0.51950283	0.993513219	ENSG00000178772	CPN2
52.2565468	0.735573148	0.638681041	1.151706565	0.249441674	0.962465655	ENSG00000138792	ENPEP
51.22649199	0.019353229	0.455358164	0.042501113	0.966099224	0.999527751	ENSG00000226976	COX6A1P2
52.32618504	0.113595854	0.413006426	0.275046214	0.783280734	0.999467288	ENSG00000152527	PLEKHH2
52.70074848	0.014068932	0.66963267	0.021009924	0.983237739	0.999527751	ENSG00000121552	CSTA
51.9098647	0.355474163	0.396399229	0.896757959	0.369848086	0.981455968	ENSG00000158163	DZIP1L
53.15126123	1.435228484	0.652348593	2.200094398	0.027800198	0.769167109	ENSG00000230551	AC021078.1
52.95029534	-0.632595256	0.612637166	-1.03257734	0.301801737	0.972793733	ENSG00000224914	LINC00863
50.51654907	0.916278916	0.648886662	1.412078518	0.157926843	0.939464191	ENSG00000185158	LRRC37B
52.05857537	0.218307368	0.397429549	0.54929828	0.582800768	0.99483168	ENSG00000185869	ZNF829
50.99561876	0.644501076	0.522889278	1.232576576	0.217733781	0.956884001	ENSG00000203706	SERTAD4-AS1
51.49155622	0.147922574	1.657305238	0.089254877	0.928879354	0.999527751	ENSG00000124731	TREM1
51.74273708	-0.919936202	0.510193151	-1.803113584	0.071370378	0.883866242	ENSG00000235652	AL356599.1
51.66249631	-0.214004007	0.475346391	-0.45020644	0.652561593	0.997471445	ENSG00000198934	MAGEE1
50.96635298	-1.505410542	0.848245136	-1.77473525	0.075941612	0.897052166	ENSG00000205426	KRT81
49.36162538	0.390488878	0.747422592	0.52244725	0.601358965	0.99483168	ENSG00000163545	NUAK2
52.08341566	0.211689403	0.56800035	0.372692382	0.709377407	0.99900742	ENSG00000106804	C5
51.72705281	0.284757094	0.529514594	0.537770058	0.590735806	0.99483168	ENSG00000186281	GPAT2
50.73891007	-0.457180276	0.503792904	-0.907476609	0.364154814	0.98048242	ENSG00000155754	C2CD6
51.64537135	-0.038520166	0.64928314	-0.059327223	0.952691478	0.999527751	ENSG00000254343	AC091563.1
52.20432365	0.769694411	0.50373182	1.527984496	0.126516388	0.928627271	ENSG00000234420	ZNF37BP
50.97242222	-0.250194364	0.508922617	-0.49161573	0.622991021	0.99483168	ENSG00000115425	PECR
51.21132451	0.363660954	0.449459784	0.809106769	0.418453735	0.990226751	ENSG00000079931	MOXD1
51.51431921	-0.357636562	0.543198157	-0.658390602	0.510287173	0.993513219	ENSG00000197566	ZNF624

53.47392304	-1.263498831	0.801560754	-1.576298271	0.11495708	0.923922314	ENSG00000070031	SCT
52.81854332	-0.35856288	0.707437148	-0.506847684	0.612261696	0.99483168	ENSG00000277758	FO681492.1
51.88253561	-0.21298946	0.467499763	-0.455592659	0.648682923	0.997368416	ENSG00000174206	C12orf66
51.36480005	1.485355484	0.960063891	1.547142328	0.121828929	0.923922314	ENSG00000075702	WDR62
51.2320737	-0.145225435	0.432696589	-0.335628795	0.737150807	0.999467288	ENSG00000251192	ZNF674
51.86452343	2.384436407	0.722425589	3.300597932	0.00096479	0.228234741	ENSG00000135476	ESPL1
52.22801357	-0.479869021	0.481182105	-0.997271128	0.318632924	0.974768742	ENSG00000133740	E2F5
51.24536616	0.898057063	0.464233959	1.934492396	0.053052616	0.847598083	ENSG00000244879	GABPB1-AS1
51.61341501	-0.160083105	0.504488305	-0.317317771	0.751002494	0.999467288	ENSG00000177576	C18orf32
50.1343347	0.836824831	0.501319949	1.669243032	0.095069225	0.921147998	ENSG00000203930	LINC00632
50.76442328	-0.180438599	0.456089106	-0.395621375	0.692384366	0.998869317	ENSG00000158483	FAM86C1
52.05217793	0.451138989	0.51875574	0.869655898	0.38448848	0.985655313	ENSG00000234444	ZNF736
50.05867723	-0.455029815	0.726672935	-0.626182417	0.531195298	0.99483168	ENSG00000111671	SPSB2
51.83149464	0.505606236	0.500001373	1.011209694	0.311916077	0.973427483	ENSG00000204519	ZNF551
49.88409857	0.516283936	0.568375404	0.908350242	0.363693206	0.98048242	ENSG00000235374	SSR4P1
50.51939814	-0.541037161	0.464908241	-1.163750422	0.244525169	0.962465655	ENSG00000253250	C8orf88
51.21580782	0.286264161	0.475235537	0.602362698	0.546932734	0.99483168	ENSG00000140025	EFCAB11
48.42940128	-0.331772973	1.007094871	-0.32943567	0.74182641	0.999467288	ENSG00000169627	BOLA2B
50.67772789	-1.077055303	0.513027315	-2.099411222	0.035780666	0.785058983	ENSG00000197380	DACT3
51.33491059	-0.028921458	0.515070282	-0.056150508	0.955221908	0.999527751	ENSG00000185842	DNAH14
50.66073502	-0.581349767	0.418083849	-1.390509986	0.164374069	0.943223078	ENSG00000187566	NHLRC1
50.32403958	-0.221787895	0.444540882	-0.498914507	0.617839614	0.99483168	ENSG00000204315	FKBPL
51.20311891	0.670993202	0.4471593	1.500568591	0.13346718	0.933741583	ENSG00000136059	VILL
50.76462436	0.180119778	0.544236409	0.330958707	0.740675676	0.999467288	ENSG00000138111	MFSD13A
50.33013992	0.165067714	0.510541987	0.323318588	0.746453978	0.999467288	ENSG00000087903	RFX2
50.85369342	0.126273999	0.453141833	0.278663301	0.780503229	0.999467288	ENSG00000161551	ZNF577
51.75864929	-0.192517507	0.494951755	-0.388962167	0.697304134	0.998869317	ENSG00000197362	ZNF786
50.473833	0.890597577	0.407874241	2.183510227	0.028998257	0.773201806	ENSG00000173715	C11orf80
51.05620801	0.033693005	0.462386643	0.072867601	0.941911476	0.999527751	ENSG00000100478	AP4S1
49.84125977	0.01757184	0.491274353	0.035767876	0.971467448	0.999527751	ENSG00000172006	ZNF554
50.6247452	-1.079011297	0.505495191	-2.134562932	0.032796728	0.775963115	ENSG00000138669	PRKG2
50.89511941	0.40393705	0.415652836	0.97181353	0.331143324	0.976026345	ENSG00000127081	ZNF484
49.77304242	0.634702627	0.745827867	0.851004173	0.394767035	0.988378925	ENSG00000111331	OAS3
51.07220652	-0.29510669	0.474035812	-0.622540919	0.533586246	0.99483168	ENSG00000198756	COLGALT2
50.30902863	0.005118998	0.451350333	0.011341517	0.990950973	0.999527751	ENSG00000254093	PINX1
48.39704343	0.867954297	0.855722105	1.014294584	0.310442208	0.973427483	ENSG00000111728	ST8SIA1
50.00661954	0.365326326	0.544404648	0.671056588	0.502184481	0.993513219	ENSG00000136367	ZFHX2
50.84703803	-0.029270494	0.46894927	-0.062417187	0.950230608	0.999527751	ENSG00000182405	PGBD4
49.70207659	-0.298048095	0.491409179	-0.606517151	0.544171397	0.99483168	ENSG00000165821	SALL2
49.52984751	-0.010032036	0.702855576	-0.014273254	0.988611978	0.999527751	ENSG00000048540	LMO3
48.46907363	0.037955681	0.623338995	0.060890914	0.951446086	0.999527751	ENSG00000275226	LINC00547
49.06918068	0.248552603	0.659620321	0.376811623	0.706313603	0.99900742	ENSG00000132481	TRIM47
50.40505822	-0.018743505	0.466773794	-0.040155436	0.967969206	0.999527751	ENSG00000173480	ZNF417
49.76339919	0.278654308	0.552147904	0.504673306	0.613788313	0.99483168	ENSG00000154358	OBSCN
50.63676348	0.307119413	0.468119524	0.656070507	0.51177876	0.993513219	ENSG00000196345	ZKSCAN7
49.42320659	-0.411671409	0.631644559	-0.651745359	0.514565456	0.993513219	ENSG00000148541	FAM13C
51.03995331	-0.226144661	0.467414084	-0.483820811	0.628513044	0.99483168	ENSG00000139675	HNRNPA1L2
50.14484368	0.914948248	0.42015224	2.177658859	0.029431444	0.773201806	ENSG00000171163	ZNF692
49.34103928	-0.299463992	0.458844133	-0.652648624	0.513982831	0.993513219	ENSG00000162959	MEMO1
48.85103489	0.542019656	0.61925529	0.875276586	0.381423431	0.985209339	ENSG00000239332	LINC01119
50.50835306	0.472170232	0.62687703	0.753210294	0.451323555	0.99135833	ENSG00000253910	PCDHGB2
50.7825385	-0.376001267	0.704797022	-0.533488728	0.593695312	0.99483168	ENSG00000120057	SFRP5
50.27826425	-0.01944975	0.480627809	-0.040467384	0.96772051	0.999527751	ENSG00000105085	MED26
50.28116436	-0.104928269	0.474720514	-0.221031673	0.825067771	0.999467288	ENSG00000127399	LRRC61
50.70919212	-1.021215623	0.495379703	-2.061480548	0.039257217	0.793985738	ENSG00000223803	RPS20P14
48.86320584	-0.133551584	0.494197752	-0.270239157	0.78697627	0.999467288	ENSG00000177406	AC021054.1
48.58042723	-0.112839003	0.498626505	-0.226299649	0.820968371	0.999467288	ENSG00000280071	GATD3B
49.40277504	0.051963005	0.422292277	0.123049858	0.902067617	0.999527751	ENSG00000185015	CA13
50.74711219	1.743766011	0.545825789	3.194729977	0.001399617	0.264250513	ENSG00000204149	AGAP6
49.22028685	-0.713440296	0.512642957	-1.391690428	0.164016162	0.943223078	ENSG00000237149	ZNF503-AS2
48.58321141	-0.604547203	0.473030802	-1.278029254	0.201239112	0.954310328	ENSG00000205544	TMEM256
49.60640547	-0.201588539	0.470385042	-0.428560692	0.668242957	0.997568624	ENSG00000115266	APC2
49.86281069	-0.486121332	0.614058791	-0.791652752	0.428563179	0.991301167	ENSG00000232679	LINC01705
48.63899013	-0.615143885	0.609865605	-1.008654824	0.313140204	0.973427483	ENSG00000162981	FAM84A

50.10368629	-0.52925495	0.463615325	-1.141582086	0.253627774	0.962465655	ENSG00000025434	NR1H3
49.55112958	0.094964388	0.846025946	0.112247607	0.910627083	0.999527751	ENSG00000138347	MYPN
49.13636877	0.24635779	0.535280066	0.460240919	0.645343303	0.997368416	ENSG00000168646	AXIN2
49.21664479	-0.526222462	0.506513813	-1.038910388	0.298846415	0.972186492	ENSG00000265688	MAFG-DT
49.24090945	1.104496586	0.593292885	1.861638012	0.062654132	0.868202292	ENSG00000260804	LINC01963
49.14619358	0.225811713	0.440924222	0.512132702	0.608558138	0.99483168	ENSG00000112981	NME5
49.32885956	0.496640482	0.811770366	0.611799227	0.540670601	0.99483168	ENSG00000019991	HGF
49.65820925	0.820078319	0.60445975	1.356712864	0.174872463	0.946176003	ENSG00000139364	TMEM132B
48.87876126	0.199462733	0.444657338	0.448576277	0.65373735	0.997527562	ENSG00000164048	ZNF589
50.8822122	-0.969942244	0.701088164	-1.383481129	0.16651739	0.943223078	ENSG00000182578	CSF1R
48.32440026	-0.214936669	0.555999889	-0.38657682	0.699069521	0.998869317	ENSG00000196456	ZNF775
50.27054746	0.667476999	0.546501519	1.221363484	0.221948427	0.956884001	ENSG00000213793	ZNF888
49.52843663	0.074553877	0.446049937	0.167142445	0.867257969	0.999467288	ENSG00000133247	KMT5C
49.68987867	0.647786938	0.45253881	1.431450571	0.152301124	0.938052926	ENSG00000147118	ZNF182
49.85550933	0.821080005	0.438649292	1.87183707	0.061229148	0.863606039	ENSG00000256223	ZNF10
49.19379461	0.516904649	0.412030706	1.254529434	0.209649634	0.954310328	ENSG00000164663	USP49
49.17066884	0.328030969	0.457068919	0.717683823	0.47295226	0.99135833	ENSG00000080298	RFX3
49.34593313	-0.864033015	0.508052711	-1.700675926	0.089003858	0.910413547	ENSG00000176399	DMRTA1
47.79092126	0.719564962	0.843439858	0.853131323	0.393586483	0.988378925	ENSG00000148082	SHC3
49.50304847	0.177481353	0.586314502	0.302706742	0.762113359	0.999467288	ENSG00000108352	RAPGEFL1
49.93468242	-0.17044209	0.484269562	-0.351957057	0.724870468	0.999456245	ENSG00000174332	GLIS1
48.50207868	1.489883282	0.790579799	1.884545094	0.059491281	0.858374199	ENSG00000213347	MXD3
49.57248317	-0.921192259	0.547978241	-1.681074521	0.092748441	0.921147998	ENSG00000265519	AC015922.2
48.19280851	0.504776648	0.505895585	0.997788207	0.31838207	0.974768742	ENSG00000173727	AP000769.1
49.46344768	-0.301449472	0.437099448	-0.689658781	0.490408793	0.993513219	ENSG00000148835	TAF5
49.81724675	0.124080549	0.562416998	0.220620197	0.825388173	0.999467288	ENSG00000227398	KIF9-AS1
48.16066482	-0.027645637	0.682855216	-0.040485356	0.967706181	0.999527751	ENSG00000122986	HVCN1
48.85124013	0.203316334	0.501058343	0.405773772	0.684908831	0.998869317	ENSG00000120324	PCDHB10
48.7164236	0.083042336	0.497529385	0.166909409	0.867441329	0.999467288	ENSG00000135740	SLC9A5
49.09870357	-0.086445783	0.524300629	-0.164878274	0.869039793	0.999467288	ENSG00000160606	TLCD1
48.05511986	0.428495744	0.569138891	0.752884315	0.451519434	0.99135833	ENSG00000263142	LRRC37A17P
47.15702875	-0.33847633	4.712827687	-0.071820222	0.94274498	0.999527751	ENSG00000134184	GSTM1
48.80979297	0.542002617	0.594398549	0.911850505	0.36184742	0.979465649	ENSG00000176749	CDK5R1
48.86640694	0.623889993	0.684518386	0.911429124	0.362069314	0.979465649	ENSG00000161692	DBF4B
48.60917521	0.827587036	0.47431543	1.744803108	0.081019148	0.897807954	ENSG00000198429	ZNF69
49.59130928	0.244259895	0.576529447	0.423672886	0.671804392	0.997568624	ENSG00000182141	ZNF708
48.9014449	-0.908196608	0.432030167	-2.102160166	0.035539248	0.785058983	ENSG00000153896	ZNF599
48.06098326	0.089940958	0.47918686	0.187694958	0.851115783	0.999467288	ENSG00000106588	PSMA2
50.08830188	0.250415945	0.617348725	0.405631267	0.685013552	0.998869317	ENSG00000110693	SOX6
53.30494233	-2.351726194	1.838061644	-1.279459915	0.200735147	0.954310328	ENSG00000131620	ANO1
48.97042254	0.525075848	0.584448286	0.898412846	0.36896549	0.981455968	ENSG00000261512	AC092368.3
47.85356624	-1.439283003	0.692688616	-2.077821073	0.037725842	0.790190505	ENSG00000105649	RAB3A
48.3637897	-0.070138539	0.468231523	-0.149794568	0.880926695	0.999467288	ENSG00000226360	RPL10AP6
49.2722392	0.087009688	0.578321859	0.150452014	0.880408007	0.999467288	ENSG00000189134	NKAPL
48.22684639	0.335010534	0.500111	0.669872356	0.502939164	0.993513219	ENSG00000249859	PVT1
47.43397469	-0.772593704	0.598093387	-1.291760986	0.19643993	0.954310328	ENSG00000181513	ACBD4
47.93939946	0.126672584	0.54562337	0.232161213	0.816412802	0.999467288	ENSG00000115267	IFIH1
49.10254988	0.48337387	0.564992846	0.855539807	0.392252377	0.988378925	ENSG00000144730	IL17RD
49.37293759	0.253665928	0.472945876	0.536352976	0.591714624	0.99483168	ENSG00000152582	SPEF2
47.12313113	-0.413379201	0.609363268	-0.678378929	0.497531465	0.993513219	ENSG00000198873	GRK5
47.94897108	0.154827752	0.538193284	0.287680572	0.773591263	0.999467288	ENSG00000168672	FAM84B
47.83401744	0.364839735	0.4816291	0.757511818	0.448743288	0.99135833	ENSG00000215440	NPEPL1
47.03182781	0.63037453	0.613987443	1.026689613	0.304566642	0.973427483	ENSG00000142102	PGGHG
47.0776135	0.478112379	0.568517075	0.840981564	0.400358263	0.988492517	ENSG00000104998	IL27RA
48.3262135	0.315307295	0.492714769	0.639938795	0.522212391	0.994503371	ENSG00000267454	ZNF582-AS1
47.94419166	-0.284251342	0.457325013	-0.621552142	0.534236398	0.99483168	ENSG00000284691	AC073111.5
48.87794072	-0.592147261	0.465145747	-1.273035956	0.203005283	0.954310328	ENSG00000214189	ZNF788P
46.98040395	0.368367222	0.558788959	0.65922423	0.50975179	0.993513219	ENSG00000272031	ANKRD34A
48.63973063	0.338779448	0.436105563	0.776828999	0.437259665	0.991301167	ENSG00000206573	THUMPD3-AS1
48.30365333	0.72697248	0.491029611	1.480506397	0.138738155	0.934605957	ENSG00000163689	C3orf67
48.0368347	-0.382347993	0.506561855	-0.754790337	0.450374793	0.99135833	ENSG00000150477	KIAA1328
47.95444839	0.640717068	0.449222203	1.426280945	0.153787263	0.939432741	ENSG00000114739	ACVR2B
47.73056721	-0.987309137	0.521666127	-1.892607332	0.058410108	0.852621724	ENSG00000066382	MPPED2
47.40839889	1.251549433	0.604624076	2.069962945	0.038455815	0.793985738	ENSG00000233024	AC126755.2

48.14612529	-0.111793239	0.495404605	-0.225660476	0.821465501	0.999467288	ENSG00000223804	AC244669.1
47.15882763	-0.320247541	0.58354898	-0.548792908	0.583147578	0.99483168	ENSG00000228288	PCAT6
48.24576568	-0.370014602	0.499654917	-0.7405403	0.458972218	0.99135833	ENSG00000006025	OSBPL7
47.85119054	1.767108339	0.970043943	1.821678649	0.068503759	0.87420149	ENSG00000174371	EXO1
47.46132091	0.357739397	0.433254195	0.825703249	0.408972449	0.990226751	ENSG00000177738	AC025171.1
46.11161921	0.618337622	0.704644409	0.877517247	0.380205755	0.983585589	ENSG00000150054	MPP7
47.55066299	-0.371011582	0.484188268	-0.766254794	0.443524711	0.99135833	ENSG00000167613	LAIR1
47.11225415	0.081294	0.52373842	0.155218706	0.876648902	0.999467288	ENSG00000113212	PCDHB7
47.67693715	-0.430301937	0.568445933	-0.756979534	0.449062124	0.99135833	ENSG00000274265	AC245297.3
48.2818371	-0.220067193	0.492419571	-0.446909925	0.654940098	0.997568624	ENSG00000172817	CYP7B1
48.39341156	-0.454931647	0.458169558	-0.992932941	0.320742635	0.975337095	ENSG00000254837	AP001372.2
48.22252728	0.559119225	0.443511566	1.260664359	0.2074298	0.954310328	ENSG00000110844	PRPF40B
47.51175477	-0.204927313	0.412234562	-0.497113372	0.619109106	0.99483168	ENSG00000125510	OPRL1
48.67764195	0.237165322	0.553960146	0.428127048	0.668558625	0.997568624	ENSG00000073150	PANX2
46.61811249	0.410048962	0.628852223	0.652059335	0.514362896	0.993513219	ENSG00000185361	TNFAIP8L1
47.58346874	-0.174739201	0.426104192	-0.410085617	0.681743143	0.998869317	ENSG00000203644	AC083799.1
47.46264629	-0.058679983	0.428075343	-0.137078634	0.890968641	0.999467288	ENSG00000214293	APTR
48.76233948	-0.596459332	0.454304853	-1.312905483	0.189214799	0.949094286	ENSG00000007237	GAS7
48.03753787	-0.093605561	0.658234321	-0.14220705	0.886916463	0.999467288	ENSG00000154262	ABCA6
46.52037361	-0.061673897	0.522814796	-0.117965095	0.906095315	0.999527751	ENSG00000158373	HIST1H2BD
50.11710094	-0.455028526	0.827241105	-0.550055508	0.582281302	0.99483168	ENSG00000261578	AP003119.3
47.71463708	-0.715335763	0.521096393	-1.372751323	0.169829668	0.944422693	ENSG00000233621	LINC01137
47.90640183	-0.405149715	0.527902019	-0.767471425	0.442801276	0.99135833	ENSG00000106976	DNM1
46.91963679	0.052670041	0.45967937	0.114579953	0.90877807	0.999527751	ENSG00000279117	AP001972.5
46.87207164	0.348773472	0.645696552	0.540150744	0.589093079	0.99483168	ENSG00000160999	SH2B2
48.2411697	0.233500553	0.502687858	0.464504065	0.642286648	0.996740283	ENSG00000121653	MAPK8IP1
46.97901309	0.039112846	0.498942176	0.07839154	0.937516603	0.999527751	ENSG00000241058	NSUN6
47.04502019	1.203150226	0.584789194	2.057408446	0.039646953	0.793985738	ENSG00000205746	AC126755.1
48.52152094	0.283055891	0.877147058	0.32270061	0.74692199	0.999467288	ENSG00000161653	NAGS
46.74866777	0.039268169	0.618190019	0.063521196	0.949351482	0.999527751	ENSG00000134201	GSTM5
47.46147768	0.515040567	0.43972542	1.171277675	0.241487182	0.962465655	ENSG00000143630	HCN3
46.88978147	2.463239887	0.79427802	3.101231337	0.001927177	0.309269027	ENSG00000163808	KIF15
47.30706125	1.129539708	0.906462959	1.246095824	0.212729218	0.954310328	ENSG00000102384	CENPI
45.8616353	-0.222070115	0.706591493	-0.314283595	0.753305657	0.999467288	ENSG00000140280	LYSMD2
47.20306768	0.006697365	0.499919086	0.013396898	0.989311142	0.999527751	ENSG00000131848	ZSCAN5A
47.14382939	-0.041538799	0.536517377	-0.077423026	0.938287023	0.999527751	ENSG00000241945	PWP2
44.51959308	0.73480726	0.806074889	0.911586838	0.361986253	0.979465649	ENSG00000138435	CHRNA1
46.92916206	0.72050075	1.544868462	0.466383235	0.64094121	0.996553586	ENSG00000204128	C2orf72
47.49227636	-0.654480927	0.561715177	-1.165147308	0.24395937	0.962465655	ENSG00000198010	DLGAP2
47.43917241	-0.15871983	0.435364232	-0.364567915	0.715433969	0.99900742	ENSG00000139160	ETFBKMT
47.16063775	-0.065845648	0.431238394	-0.152689669	0.878643008	0.999467288	ENSG00000184162	NR2C2AP
45.80843371	1.040203967	0.649014796	1.60274307	0.10899139	0.923922314	ENSG00000105855	ITGB8
47.21643576	-0.099728817	0.52904906	-0.188505802	0.850480168	0.999467288	ENSG00000129910	CDH15
47.59981393	-0.163432955	0.456172714	-0.358269906	0.720141337	0.99900742	ENSG00000083828	ZNF586
46.79266143	0.222318024	0.56489279	0.393557907	0.693907464	0.998869317	ENSG00000169218	RSPO1
46.73182505	-0.235102635	0.451515338	-0.520696895	0.602577937	0.99483168	ENSG00000185220	PGBD2
47.50481402	1.090930656	0.476172149	2.291042554	0.021960953	0.743057397	ENSG00000176155	CCDC57
46.81496689	-0.25140137	0.427061896	-0.588676657	0.556078199	0.99483168	ENSG00000233184	AC093157.1
47.06397411	0.672719705	0.499814786	1.345937982	0.178322518	0.9469453	ENSG00000157399	ARSE
45.03777234	-0.197990662	0.688083209	-0.287742324	0.773543989	0.999467288	ENSG00000103490	PYCARD
46.38677134	0.412357897	0.46046171	0.89553135	0.370503115	0.981455968	ENSG00000231205	ZNF826P
47.2173986	0.45059177	0.495842323	0.908740035	0.363487366	0.98048242	ENSG00000196873	CBWD3
48.05762393	-0.170080407	0.709087742	-0.239858056	0.810440298	0.999467288	ENSG00000186446	ZNF501
46.52111165	0.070871507	0.498694086	0.142114192	0.886989809	0.999467288	ENSG00000239521	CASTOR3
46.06028586	-0.427763513	0.710039961	-0.602449914	0.546874693	0.99483168	ENSG00000117069	ST6GALNAC5
45.0894811	-0.114509793	0.644850097	-0.177575833	0.859056105	0.999467288	ENSG00000162946	DISC1
45.63950334	0.665886969	0.506025905	1.315914782	0.188202647	0.949094286	ENSG00000260025	AC009414.2
46.50448445	-0.252355741	0.49619798	-0.508578735	0.611047537	0.99483168	ENSG00000197208	SLC22A4
46.83060517	0.405186103	0.498857506	0.812228139	0.416660736	0.990226751	ENSG00000144161	ZC3H8
46.91119372	0.031989547	0.5530571	0.05784131	0.953875033	0.999527751	ENSG00000093217	XYLB
46.71076801	0.063150364	0.457611031	0.13800009	0.890240346	0.999467288	ENSG00000183666	GUSBP1
46.65738251	-0.430299954	0.496226877	-0.867143588	0.385863341	0.986238739	ENSG00000219626	FAM228B
45.56947503	-0.457878318	0.543214443	-0.842905272	0.399281425	0.988378925	ENSG00000278962	AC092645.1
47.055802	-0.323521287	0.94800311	-0.34126606	0.732903296	0.999467288	ENSG00000113532	ST8SIA4

47.00232266	-0.152878384	0.798897117	-0.191361792	0.848242152	0.999467288	ENSG00000072818	ACAP1
45.96402399	0.860684225	0.481565231	1.787264049	0.073894822	0.889199003	ENSG00000175048	ZDHHC14
45.355291	0.137993277	0.660059199	0.209061971	0.834399861	0.999467288	ENSG00000139597	N4BP2L1
45.06874621	-0.421626271	0.882085375	-0.477988052	0.632658709	0.994909189	ENSG00000197361	FBXL22
45.35802103	-1.151085661	0.982552257	-1.171526149	0.241387353	0.962465655	ENSG00000150394	CDH8
46.40490682	2.436852808	1.645301376	1.481098141	0.138580423	0.934605957	ENSG00000115163	CENPA
45.40467073	-0.3001031	0.568545288	-0.527843791	0.597607761	0.99483168	ENSG00000143919	CAMKMT
46.8406374	-0.337727798	0.501827996	-0.672995132	0.500950388	0.993513219	ENSG00000250565	ATP6V1E2
46.57205573	1.08307808	0.577217399	1.876378087	0.060603381	0.858996918	ENSG00000246859	STARD4-AS1
46.08971367	0.539071353	0.568035845	0.949009393	0.342615835	0.977691382	ENSG00000261061	AC092718.4
46.6689885	1.291367781	0.638046917	2.023938594	0.04297647	0.806698895	ENSG00000242086	MUC20-OT1
45.30281511	0.186920344	0.683429963	0.273503291	0.784466363	0.999467288	ENSG00000069122	ADGRF5
46.40166834	0.499226186	0.452150209	1.104115792	0.269542912	0.962465655	ENSG00000239900	ADSL
46.26106463	0.099690189	0.486083313	0.205088689	0.837502834	0.999467288	ENSG00000164002	EXO5
44.645526	0.973653696	0.583975936	1.667283934	0.09545796	0.921147998	ENSG00000164056	SPRY1
45.27037806	-0.010182872	0.565983093	-0.017991477	0.985645653	0.999527751	ENSG00000165259	HDX
46.54245033	-0.882576718	0.512782834	-1.72115106	0.085223416	0.901307093	ENSG00000225684	FAM225B
45.44627517	-0.249664123	0.552162502	-0.452156967	0.651155907	0.997449432	ENSG00000277147	LINC00869
44.41808965	0.408613656	0.699497841	0.584152848	0.559117482	0.99483168	ENSG00000234177	LINC01114
46.15358428	0.085543349	0.457764874	0.186871807	0.851761144	0.999467288	ENSG00000136866	ZFP37
45.24624388	2.034957357	0.88196866	2.307289871	0.021038666	0.73997555	ENSG00000119969	HELLS
46.34232111	1.280661113	0.533037046	2.402574311	0.016280126	0.698816035	ENSG00000140534	TICRR
46.52638478	0.623787478	0.48400635	1.288800193	0.197467559	0.954310328	ENSG00000127329	PTPRB
45.66969249	0.257044005	0.579169207	0.443815041	0.657176313	0.997568624	ENSG00000197405	C5AR1
45.21051508	-0.048662876	0.532671546	-0.091356253	0.927209522	0.999527751	ENSG00000128394	APOBEC3F
44.75513425	0.045291515	0.50374624	0.089909385	0.928359223	0.999527751	ENSG00000213337	ANKRD39
45.67283137	1.015681278	0.487136836	2.085002003	0.037069119	0.790190505	ENSG00000125637	PSD4
45.03947578	-0.715124402	0.462643115	-1.545736613	0.122168191	0.923922314	ENSG00000270194	AC097359.2
44.68494687	0.448771068	0.492089364	0.911970672	0.361784156	0.979465649	ENSG00000135722	FBXL8
45.8585332	0.087540807	0.448487459	0.195191204	0.845243264	0.999467288	ENSG00000267080	ASB16-AS1
46.68454174	0.465734829	1.724981663	0.269994075	0.787164812	0.999467288	ENSG00000112379	ARFGEF3
44.19577479	-0.191687125	0.709889664	-0.270023829	0.787141921	0.999467288	ENSG00000160050	CCDC28B
45.25422155	0.055052814	0.456706344	0.120543134	0.904052912	0.999527751	ENSG00000097046	CDC7
45.22360561	-0.315124644	0.47458759	-0.663996805	0.506692356	0.993513219	ENSG00000177822	AC098864.1
44.98988901	0.133525164	0.46325663	0.288231522	0.773169521	0.999467288	ENSG00000238072	AC009244.1
46.41375111	-1.0963833	0.836033288	-1.311411058	0.189718925	0.949094286	ENSG00000165061	ZMAT4
46.14150011	0.365093156	0.529629267	0.689337201	0.490611093	0.993513219	ENSG00000149054	ZNF215
45.70734617	1.671841117	0.780255857	2.142683201	0.032138545	0.775963115	ENSG00000127586	CHTF18
45.57540823	-0.083207474	0.450415486	-0.184734931	0.853436946	0.999467288	ENSG00000278974	AC093909.6
45.77402163	0.14963748	0.477239003	0.313548304	0.753864128	0.999467288	ENSG00000214300	SPDYE3
45.67003016	-0.362463813	0.430295618	-0.842359991	0.399586481	0.988384114	ENSG00000236801	RPL24P8
45.0520722	1.044321735	0.664170857	1.572369105	0.115864975	0.923922314	ENSG00000187741	FANCA
45.10777291	-0.251863509	0.433018699	-0.581645802	0.560805285	0.99483168	ENSG00000249936	RAC1P2
45.25547827	-0.102320507	0.457559449	-0.223622324	0.823051192	0.999467288	ENSG00000169258	GPRIN1
44.76975989	0.62223308	0.443264367	1.403751636	0.16039282	0.941607907	ENSG00000179988	PSTK
45.75464424	0.066400808	0.46838041	0.141766833	0.887264183	0.999467288	ENSG00000182185	RAD51B
45.45597584	0.406219719	0.474486409	0.85612509	0.391928592	0.988378925	ENSG00000163013	FBXO41
44.12213623	2.357392322	0.663824225	3.55122973	0.000383436	0.173156217	ENSG00000214402	LCNL1
44.882738	0.268634864	0.580376439	0.462863146	0.643462466	0.997097166	ENSG00000166839	ANKDD1A
45.46784051	-0.208284771	0.500122251	-0.416467715	0.677067787	0.998869317	ENSG00000176371	ZSCAN2
43.55472281	-0.039853291	0.700679862	-0.056878032	0.954642354	0.999527751	ENSG00000151789	ZNF385D
44.95718216	0.674300809	0.521678517	1.292560048	0.196163265	0.954310328	ENSG00000214960	ISPD
44.64763489	0.801543023	0.682347912	1.174683778	0.240121252	0.962465655	ENSG00000137819	PAQR5
44.70261003	0.926918728	0.523220132	1.771565488	0.076466713	0.897052166	ENSG00000169629	RGPD8
45.29078934	0.99657941	0.603474833	1.651401776	0.098656563	0.921147998	ENSG00000106404	CLDN15
45.18242426	-0.197879433	0.458434915	-0.43164128	0.666002151	0.997568624	ENSG00000153790	C7orf31
45.5032773	0.095798251	0.526814441	0.181844391	0.855704847	0.999467288	ENSG00000229644	NAMPTP1
44.65616634	-0.204096865	0.530602464	-0.384651181	0.700495869	0.998869317	ENSG00000100802	C14orf93
44.68406942	-0.61785816	0.605174219	-1.020959156	0.30727379	0.973427483	ENSG00000185338	SOCS1
46.15510738	-0.230141593	0.574932759	-0.400293059	0.68894068	0.998869317	ENSG00000279415	AC099494.2
45.78034973	-0.664877194	0.636308523	-1.044897514	0.296070339	0.970706863	ENSG00000228451	SDAD1P1
44.39719496	-0.664390589	0.508835836	-1.305707149	0.191652166	0.949261383	ENSG00000268858	AL118506.1
45.26395163	0.100598271	0.446086329	0.22551301	0.821580206	0.999467288	ENSG00000205593	DENND6B
44.78031293	-0.118545	0.49409261	-0.239924657	0.810388665	0.999467288	ENSG00000250312	ZNF718

45.08239926	-0.163128466	0.450106403	-0.362422007	0.717036697	0.99900742	ENSG00000234284	ZNF879
43.90099176	0.730697428	0.551602277	1.324681674	0.185276754	0.949094286	ENSG00000223705	NSUN5P1
43.6069065	-0.585063056	0.588963107	-0.993378108	0.320525726	0.975316319	ENSG00000196972	SMIM10L2B
43.33771999	-0.889469688	4.713058159	-0.188724531	0.850308724	0.999467288	ENSG00000126890	CTAG2
46.21297262	-1.174841812	0.892369198	-1.316542318	0.187992085	0.949094286	ENSG00000237471	AC073115.2
44.99968485	0.503111333	0.468907666	1.07294329	0.283296561	0.963267117	ENSG00000273820	USP27X
45.53656648	1.211117268	0.597586747	2.026680267	0.042695122	0.806698895	ENSG00000111452	ADGRD1
45.02221371	1.717008706	0.602025777	2.852051809	0.004343802	0.462224997	ENSG00000270055	AC127502.2
44.1069461	0.570483023	0.522319892	1.092210025	0.274740797	0.962465655	ENSG00000131061	ZNF341
44.62569404	0.395073288	0.444920586	0.887963606	0.374560328	0.982609538	ENSG00000271976	AC012467.2
46.00056189	1.547591618	0.606935162	2.549846698	0.01077703	0.638240244	ENSG00000215788	TNFRSF25
45.10868883	0.120513078	0.53633069	0.224699203	0.822213285	0.999467288	ENSG00000182983	ZNF662
43.87380891	-0.485649175	0.492919846	-0.985249792	0.324501388	0.975801281	ENSG00000205220	PSMB10
42.95602139	-0.103498858	0.665832786	-0.155442718	0.876472309	0.999467288	ENSG00000226067	LINC00623
44.21715801	0.144037959	0.530018386	0.271760307	0.785806327	0.999467288	ENSG00000198556	ZNF789
44.25762425	0.22539621	0.481432856	0.468177871	0.639657396	0.996553586	ENSG00000160013	PTGIR
44.52957007	-0.38517939	0.45978627	-0.837735737	0.402179138	0.988592117	ENSG00000167065	DUSP18
44.17142165	-0.575066344	0.507452946	-1.133240724	0.257113177	0.962465655	ENSG00000254726	MEX3A
43.75317907	-0.023292705	0.557090797	-0.041811326	0.966649106	0.999527751	ENSG00000174899	PQLC2L
45.58696469	1.25432656	0.838378991	1.496133101	0.134618974	0.933741583	ENSG00000070669	ASNS
44.28777062	0.5895662	0.544446572	1.082872462	0.278865036	0.963231086	ENSG00000198157	HMGN5
44.38056592	-0.130598495	0.469851071	-0.277957215	0.781045202	0.999467288	ENSG00000250305	TRMT9B
47.19221283	-1.634907061	0.854050493	-1.914297896	0.055582103	0.847598083	ENSG00000183715	OPCML
42.83823619	0.66620455	0.634841444	1.049403054	0.293992653	0.970706863	ENSG00000136114	THSD1
44.15487707	-0.279521152	0.587332439	-0.47591642	0.634133923	0.99544042	ENSG00000000460	C1orf112
43.91638308	-0.906698842	0.523831891	-1.730896607	0.083470207	0.901307093	ENSG00000237493	AC034102.1
43.87036972	-0.027763735	0.558435007	-0.049717038	0.960347879	0.999527751	ENSG00000076258	FMO4
43.61706072	-0.237851228	0.504517194	-0.471443254	0.637324234	0.99653524	ENSG00000110455	ACCS
43.80138991	0.51452861	0.719478941	0.715140612	0.474522161	0.99135833	ENSG00000134594	RAB33A
45.04450979	-1.284844931	0.560842659	-2.290918693	0.021968117	0.743057397	ENSG00000150510	FAM124A
44.06255212	0.523404226	0.63749028	0.821038755	0.411624193	0.990226751	ENSG00000181938	GINS3
43.9525267	0.284600608	0.474404878	0.599910796	0.548565687	0.99483168	ENSG00000159882	ZNF230
42.41590865	-0.974676323	0.630533797	-1.545795528	0.122153958	0.923922314	ENSG00000118640	VAMP8
43.86140048	2.126756853	0.838723257	2.535707498	0.011222042	0.644117389	ENSG00000261051	AC107021.2
44.80199538	0.325422525	0.602566018	0.540061197	0.58915483	0.99483168	ENSG00000230797	YY2
43.16086516	0.196522222	0.52409926	0.374971379	0.707681753	0.99900742	ENSG00000155542	SETD9
42.64027571	-0.623261941	0.58040963	-1.073831151	0.282898365	0.963267117	ENSG00000111348	ARHGDIB
43.77092123	-0.490329413	0.483263852	-1.014620505	0.310286761	0.973427483	ENSG00000213862	AC044787.1
43.93412748	0.073650776	0.442198851	0.166555784	0.867719587	0.999467288	ENSG00000125650	PSPN
44.24783712	-0.214982629	0.898689717	-0.239217858	0.810936656	0.999467288	ENSG00000128052	KDR
44.23790017	0.36149156	0.512823927	0.704903848	0.480870075	0.991417002	ENSG00000137218	FRS3
43.41537847	-0.304082914	0.4527945	-0.671569363	0.501857888	0.993513219	ENSG00000154710	RABGEF1
43.95720436	0.979868266	0.471163385	2.079678296	0.03755505	0.790190505	ENSG00000184056	VPS33B
43.27260556	0.242479709	0.492048974	0.492795884	0.622156819	0.99483168	ENSG00000184635	ZNF93
43.59851537	-0.442724987	0.468620868	-0.94474023	0.344791513	0.977691382	ENSG00000168661	ZNF30
43.31506281	-0.095102568	0.462536547	-0.205610927	0.837094841	0.999467288	ENSG00000263072	ZNF213-AS1
43.14561758	0.771642774	0.443575132	1.739598813	0.081929488	0.898487162	ENSG00000221886	ZBED8
42.25397759	0.058653299	0.78356889	0.074854042	0.940330843	0.999527751	ENSG00000149809	TM7SF2
43.58227031	-0.108620079	0.582225953	-0.18656001	0.852005624	0.999467288	ENSG00000139899	CBLN3
44.08153285	-0.402631067	0.604187678	-0.666400659	0.505155042	0.993513219	ENSG00000214575	CPEB1
42.81070254	-0.051537245	0.493417726	-0.10444952	0.916812626	0.999527751	ENSG00000249042	AC008771.1
44.62744979	-0.142484508	0.636580082	-0.223828097	0.822891066	0.999467288	ENSG00000280128	AL662795.2
42.99908217	0.278706347	0.675805318	0.412406265	0.680041672	0.998869317	ENSG00000105894	PTN
40.51912747	-1.016330196	0.818880922	-1.241120861	0.214561104	0.954310328	ENSG00000170442	KRT86
43.01144658	-0.766021099	0.507813212	-1.508470203	0.131434227	0.930997815	ENSG00000116991	SIPA1L2
44.80324368	-1.293973238	0.602162173	-2.148878319	0.031644047	0.775963115	ENSG00000155926	SLA
41.12871973	1.565370518	0.80500132	1.94455646	0.051828384	0.845356848	ENSG00000274012	RN7SL2
42.81092845	2.228030471	1.536473476	1.450093676	0.147032398	0.936396974	ENSG00000092853	CLSPN
43.7076725	-0.264224326	0.576743867	-0.458131141	0.646858227	0.997368416	ENSG00000247572	CKMT2-AS1
42.88274661	-0.506103789	0.544729406	-0.929092103	0.352841357	0.977691382	ENSG00000166333	ILK
43.21787064	0.299154141	0.57429625	0.520905614	0.602432524	0.99483168	ENSG00000156414	TDRD9
43.41191179	0.288341512	0.478032896	0.603183409	0.546386684	0.99483168	ENSG00000125434	SLC25A35
44.10757117	0.373223751	0.772289403	0.483269289	0.628904543	0.99483168	ENSG00000162415	ZSWIM5
43.81138124	-0.097354409	0.498891984	-0.195141257	0.845282365	0.999467288	ENSG00000178338	ZNF354B

42.70162428	-0.058349597	0.442061412	-0.131994323	0.894988782	0.999467288	ENSG00000160439	RDH13
43.11221694	0.333756651	0.555247053	0.601095763	0.547776206	0.99483168	ENSG00000274020	LINC01138
42.79282241	-0.122657926	0.504192775	-0.24327585	0.807791711	0.999467288	ENSG00000056277	ZNF280C
43.86534583	0.134642108	0.581492738	0.23154564	0.816890933	0.999467288	ENSG00000143178	TBX19
42.50355251	-0.224579024	0.463443476	-0.484587734	0.627968817	0.99483168	ENSG00000213740	SERBP1P1
42.24192649	1.846548758	1.577525804	1.170534741	0.241785842	0.962465655	ENSG00000152253	SPC25
42.46097129	1.714231766	0.608201476	2.818526151	0.004824468	0.472943531	ENSG00000048052	HDAC9
41.47187112	1.607909032	1.133604693	1.418403648	0.156072951	0.939464191	ENSG00000100162	CENPM
43.83491424	0.556469979	0.697329255	0.798001769	0.424869463	0.991301167	ENSG00000283787	PRR33
42.08111651	-0.205473302	0.544134535	-0.377614889	0.705716702	0.99900742	ENSG00000228630	HOTAIR
42.47017179	-1.153927377	0.859437457	-1.342654276	0.179383947	0.9469453	ENSG00000224982	TMEM233
42.57941068	-0.090014978	0.495651638	-0.181609362	0.855889301	0.999467288	ENSG00000214223	HNRNPA1P10
42.1869529	-0.596939035	0.507032944	-1.177318046	0.239068587	0.962465655	ENSG00000104863	LIN7B
41.95262238	-0.342351146	0.485451465	-0.705222192	0.480671973	0.991417002	ENSG00000258056	AC009779.2
41.62474416	0.627003627	0.661882142	0.94730404	0.343483873	0.977691382	ENSG00000005189	REXO5
42.60439716	0.150892719	0.447905701	0.336885014	0.736203582	0.999467288	ENSG00000083838	ZNF446
41.57207605	-0.37405083	0.590636073	-0.633301701	0.526536643	0.99483168	ENSG00000118804	STBD1
42.18768973	2.143777793	1.814367945	1.181556254	0.23738182	0.962465655	ENSG00000171320	ESCO2
42.4742608	0.89705475	0.467472715	1.918945684	0.054991208	0.847598083	ENSG00000166436	TRIM66
40.14091288	0.642644522	0.884539061	0.726530405	0.467513675	0.99135833	ENSG00000021645	NRXN3
41.98976454	0.35351426	0.505301166	0.699611012	0.484170264	0.991892545	ENSG00000278133	AC135050.6
39.7070535	1.847639467	1.832769798	1.008113223	0.313400111	0.973427483	ENSG00000168685	IL7R
43.85454055	-0.450795219	0.619451197	-0.727733227	0.466776906	0.99135833	ENSG00000188211	NCR3LG1
42.56797076	-0.174549883	0.504507662	-0.345980638	0.729357266	0.999467288	ENSG00000131944	FAAP24
43.16169702	1.58254741	0.604004204	2.62009337	0.00879057	0.604581883	ENSG00000206149	HERC2P9
41.57895037	0.155529909	0.589238	0.263950915	0.791817742	0.999467288	ENSG00000226124	FTCDNL1
42.28941125	-0.009046988	0.634295479	-0.01426305	0.988620119	0.999527751	ENSG00000206052	DOK6
41.26148073	-0.554186239	0.545484614	-1.0159521	0.309652197	0.973427483	ENSG00000229833	PET100
42.30888091	0.628060325	0.483049261	1.300199329	0.19353266	0.950382812	ENSG00000188227	ZNF793
42.78859159	-0.442624246	0.509566933	-0.868628274	0.385050484	0.985733881	ENSG00000142396	ERVK3-1
40.10598374	0.941957109	0.791824073	1.189604031	0.234202061	0.960525552	ENSG00000183486	MX2
41.1294582	-0.194523725	0.555475054	-0.35019345	0.726193522	0.999467288	ENSG00000158406	HIST1H4H
41.98103155	0.174684582	0.642009619	0.272090288	0.785552597	0.999467288	ENSG00000184194	GPR173
42.10247311	0.347188792	0.464314528	0.747744838	0.45461408	0.99135833	ENSG00000189376	C8orf76
41.38808269	0.820595244	0.523499734	1.567517977	0.116993668	0.923922314	ENSG00000121236	TRIM6
41.29456518	-0.146618976	0.498202705	-0.294295826	0.768531862	0.999467288	ENSG00000239713	APOBEC3G
41.93520537	0.582287316	0.504702525	1.153723802	0.248613422	0.962465655	ENSG00000260920	AL031985.3
42.91040271	-0.003705989	0.543695907	-0.00681629	0.99456143	0.999527751	ENSG00000178752	ERFE
42.02753444	-0.336764252	0.872018675	-0.386189267	0.699356502	0.998869317	ENSG00000177453	NIM1K
41.75913874	0.303350188	0.459415492	0.660295948	0.50906393	0.993513219	ENSG00000160404	TOR2A
41.20440798	0.955397315	0.464988314	2.054669521	0.039910936	0.793985738	ENSG00000110852	CLEC2B
42.00550592	-0.198382197	0.564290222	-0.35156058	0.725167833	0.999467288	ENSG00000205363	INSYN1
41.39177646	0.384822807	0.502338115	0.766063325	0.443638624	0.99135833	ENSG00000152380	FAM151B
41.37067633	-0.244902322	0.519167851	-0.471720891	0.637126023	0.99653524	ENSG00000078237	TIGAR
41.3764096	-0.344110827	0.517291471	-0.66521651	0.505912022	0.993513219	ENSG00000269439	AC010618.3
42.59760019	-0.333237616	0.518515795	-0.642675922	0.520434408	0.993513219	ENSG00000132837	DMGDH
40.73195004	-1.218232426	0.62417015	-1.951763354	0.050966307	0.842188482	ENSG00000113319	RASGRF2
41.30902783	0.16986	0.481948708	0.352444144	0.724505201	0.999456245	ENSG00000237036	ZEB1-AS1
41.52745897	0.042262411	0.520650437	0.081172334	0.935304901	0.999527751	ENSG00000256525	POLG2
39.61091539	0.230848189	0.774736047	0.297970115	0.765725973	0.999467288	ENSG00000169750	RAC3
41.83195987	-0.343566678	0.499289951	-0.68811054	0.491383174	0.993513219	ENSG00000236439	AC099336.2
42.123935	-0.032517505	0.69106877	-0.047053935	0.962470241	0.999527751	ENSG00000251615	AC104825.1
42.96463839	-0.549418796	0.680663903	-0.807180745	0.419562357	0.990226751	ENSG00000167771	RCOR2
41.41139188	0.279908176	0.731778126	0.382504159	0.702087445	0.998869317	ENSG00000141404	GNAL
40.67511978	0.272219212	0.498030564	0.546591377	0.584659494	0.99483168	ENSG00000277072	STAG3L2
41.12886668	-0.213728144	0.494972334	-0.431798162	0.665888116	0.997568624	ENSG00000141519	CCDC40
41.94996228	-0.230146612	0.589434998	-0.390452913	0.696201667	0.998869317	ENSG00000164989	CCDC171
41.81620948	0.683009725	0.531640941	1.284719954	0.198890163	0.954310328	ENSG00000176597	B3GNT5
40.28523909	-0.568605497	0.653386734	-0.870243406	0.3841674	0.985655313	ENSG00000248429	FAM198B-AS1
39.86212058	0.136452431	0.845631002	0.16136167	0.871808554	0.999467288	ENSG00000107105	ELAVL2
41.1785178	-0.371214909	0.524529676	-0.707710023	0.479125352	0.99135833	ENSG00000198774	RASSF9
41.41843329	-0.338194513	0.49779131	-0.679390152	0.496890688	0.993513219	ENSG00000174628	IQCK
40.35694707	0.725784727	0.55450665	1.308883719	0.190573742	0.949094286	ENSG00000132196	HSD17B7
41.02960701	0.266289442	0.603886999	0.440959057	0.659242638	0.997568624	ENSG00000146556	WASH2P

40.31889191	1.460433934	0.782236994	1.866996762	0.06190204	0.865910462	ENSG00000248323	LUCAT1
41.11963836	-0.306231306	0.479711152	-0.638366035	0.523235435	0.994616524	ENSG00000118690	ARMC2
41.46812855	0.389074567	0.664303748	0.58568775	0.558085368	0.99483168	ENSG00000175471	MCTP1
40.54096525	-0.41885076	0.537141968	-0.779776642	0.435522357	0.991301167	ENSG00000102984	ZNF821
41.25916356	-0.657428266	0.458837744	-1.432812089	0.151911545	0.938052926	ENSG00000249471	ZNF324B
40.52449375	0.154577507	0.484425738	0.319094332	0.749654982	0.999467288	ENSG00000243547	HNRNPKP4
41.4943295	-0.017032195	0.574015149	-0.02967203	0.976328619	0.999527751	ENSG00000166450	PRTG
40.87983623	-0.953164127	0.839986445	-1.134737509	0.256485316	0.962465655	ENSG00000223485	LINC01615
41.11813234	0.37282777	0.484866878	0.768928106	0.441935992	0.99135833	ENSG00000181404	WASHC1
40.10240778	2.673075229	1.164651657	2.295171448	0.021723301	0.73997555	ENSG00000189057	FAM111B
40.16369543	0.254619818	0.561717388	0.453288118	0.650341287	0.997381078	ENSG00000079462	PAFAH1B3
40.64887534	-0.212278689	0.595514293	-0.356462795	0.72149401	0.99900742	ENSG00000143786	CNIH3
40.69207264	0.641897585	0.530566037	1.209835422	0.226342051	0.958865115	ENSG00000198908	BHLHB9
40.52707932	0.073053248	0.495882774	0.147319592	0.882879769	0.999467288	ENSG00000173137	ADCK5
41.61358849	-1.033498265	0.597834541	-1.728736287	0.083856304	0.901307093	ENSG00000255642	PABPC1P4
40.39746849	0.318768254	0.528955359	0.602637347	0.54674997	0.99483168	ENSG00000204851	PNMA8B
40.76340356	0.333641496	0.517226685	0.645058551	0.518889243	0.993513219	ENSG00000018869	ZNF582
40.32732367	-0.290516562	0.539323092	-0.538668872	0.590115356	0.99483168	ENSG00000117010	ZNF684
40.81946976	0.232103695	0.601760226	0.385707937	0.699712984	0.998869317	ENSG00000115556	PLCD4
40.17935631	-0.172487916	0.471994827	-0.365444504	0.714779625	0.99900742	ENSG00000270959	LPP-AS2
40.48809127	-0.578477519	0.584333305	-0.989978688	0.322184536	0.975337095	ENSG00000250182	EEF1A1P13
41.25073793	0.05193606	0.558185328	0.093044474	0.925868229	0.999527751	ENSG00000230417	LINC00856
39.91506255	0.84781154	0.504515896	1.680445644	0.092870642	0.921147998	ENSG00000174456	C12orf76
39.58368457	-0.544639408	0.75150624	-0.724730386	0.46861745	0.99135833	ENSG00000081803	CADPS2
40.53537284	-0.112340159	0.470665062	-0.238683871	0.811350724	0.999467288	ENSG00000147586	MRPS28
40.90829795	0.580976234	0.648263139	0.896204332	0.370143642	0.981455968	ENSG00000186314	PRELID2
41.16179659	-0.066514277	0.74692007	-0.089051398	0.929041062	0.999527751	ENSG00000213071	LPAL2
39.44886015	0.277716972	0.577683465	0.480742464	0.630699547	0.99483168	ENSG00000064199	SPA17
40.38135427	-0.44354837	0.582803599	-0.761059765	0.446621371	0.99135833	ENSG00000213846	AC098614.1
39.77047037	-0.699078315	0.505046497	-1.384186047	0.166301495	0.943223078	ENSG00000038945	MSR1
40.19594518	-0.525077638	0.647467392	-0.810971555	0.417382006	0.990226751	ENSG00000213713	PIGCP1
39.83678952	2.080286814	0.777267822	2.676409282	0.007441571	0.563130099	ENSG00000260578	AC110597.1
39.8745762	-0.008901258	0.555250875	-0.016031057	0.987209615	0.999527751	ENSG00000147144	CCDC120
40.39161799	-0.566744008	0.732994928	-0.773189535	0.439410218	0.991301167	ENSG00000130052	STARD8
39.89998052	0.00112956	0.533354768	0.002117839	0.99831021	0.999527751	ENSG00000234742	AC144530.1
38.83414976	-0.514250431	0.748815282	-0.686752051	0.49223899	0.993513219	ENSG00000120262	CCDC170
40.86916802	0.311920167	0.575916913	0.541606193	0.588089825	0.99483168	ENSG00000215126	CBWD6
40.17163552	0.107082831	0.471067222	0.22731964	0.820175202	0.999467288	ENSG00000186666	BCDIN3D
39.83779213	0.176179612	0.452409777	0.389424855	0.696961889	0.998869317	ENSG00000259291	ZNF710-AS1
39.83849465	-0.493579291	0.76430039	-0.645792279	0.518413889	0.993513219	ENSG00000279369	AC046185.3
40.22326823	0.148440287	0.49442178	0.300230073	0.764001667	0.999467288	ENSG00000277150	F8A3
39.24514379	-0.150888847	0.775860243	-0.194479416	0.845800511	0.999467288	ENSG00000164265	SCGB3A2
40.11941639	0.632629279	0.513728547	1.231446613	0.218155872	0.956884001	ENSG00000229043	AC091729.3
39.89783613	0.250567843	0.519815725	0.482032057	0.629783174	0.99483168	ENSG00000106246	PTCD1
40.00626595	-0.380200434	0.510456425	-0.744824467	0.456377842	0.99135833	ENSG00000242779	ZNF702P
39.88140833	0.140738316	0.628752607	0.223837348	0.822883867	0.999467288	ENSG00000164674	SYTL3
39.69826952	0.90827328	0.605445773	1.500172799	0.133569647	0.933741583	ENSG00000196247	ZNF107
35.90788941	1.269469253	1.794124638	0.707570269	0.479212161	0.99135833	ENSG00000127954	STEAP4
40.31584216	1.082131144	0.534352229	2.025127031	0.042854322	0.806698895	ENSG00000126215	XRCC3
39.91834642	-0.672586637	0.474644152	-1.417033443	0.15647315	0.939464191	ENSG00000183250	LINC01547
39.30390458	-0.353588934	0.444794844	-0.794948364	0.426643539	0.991301167	ENSG00000183935	HTR7P1
40.00367661	0.694676767	0.54253926	1.280417508	0.20039834	0.954310328	ENSG00000139133	ALG10
39.44899791	-1.493013116	0.593011002	-2.517681985	0.011812993	0.648356243	ENSG00000132965	ALOX5AP
39.34332933	-0.08501308	0.520921311	-0.163197547	0.870362897	0.999467288	ENSG00000102931	ARL2BP
39.99110351	0.123078429	0.495780445	0.248251884	0.803939525	0.999467288	ENSG00000241015	TPM3P9
39.68624312	0.411993389	0.518961048	0.793881142	0.427264629	0.991301167	ENSG00000184381	PLA2G6
39.02218363	-1.92778578	0.522499848	-3.689543238	0.000224657	0.118725853	ENSG00000237276	ANO7L1
38.42661572	-0.473363828	0.57934154	-0.817072134	0.413887207	0.990226751	ENSG00000272602	ZNF595
39.36278442	0.001162095	0.45051613	0.002579476	0.997941879	0.999527751	ENSG00000132570	PCBD2
39.81185958	-0.288935655	0.510003923	-0.566536141	0.571029372	0.99483168	ENSG00000248275	TRIM52-AS1
38.58046464	0.707005174	0.763874763	0.925551162	0.354679284	0.977691382	ENSG00000135643	KCNMB4
39.54301297	-0.382209179	0.534756455	-0.714735044	0.474772779	0.99135833	ENSG00000259494	MRPL46
38.51532342	-0.431213431	0.593098933	-0.727051437	0.467194447	0.99135833	ENSG00000174939	ASPHD1
38.41765609	0.083202404	0.637079261	0.130599768	0.896091926	0.999467288	ENSG00000182134	TDRKH

38.58156119	0.52874332	0.688696617	0.767744907	0.442638751	0.99135833	ENSG00000124496	TRERF1
39.76328322	3.336526744	1.672380701	1.995076086	0.046034582	0.815583282	ENSG00000135451	TROAP
39.36927819	0.159586694	0.503305558	0.317077154	0.75118506	0.999467288	ENSG00000121903	ZSCAN20
39.21358951	0.151751057	0.519265537	0.292241726	0.770101811	0.999467288	ENSG00000153064	BANK1
38.79840362	0.511877311	0.493605928	1.037016134	0.299728338	0.972186492	ENSG00000182484	WASH6P
38.88137017	-0.071107984	0.54815165	-0.129723196	0.89678543	0.999467288	ENSG00000226688	ENTPD1-AS1
37.82309463	0.787042432	0.599676141	1.312445798	0.189369763	0.949094286	ENSG00000235823	OLMALINC
39.00760029	0.494880783	0.523173636	0.945920722	0.344189023	0.977691382	ENSG00000166669	ATF7IP2
39.60140543	-0.157527956	0.537097287	-0.293295014	0.769296664	0.999467288	ENSG00000062370	ZNF112
39.65194977	0.453162784	0.499068165	0.908017813	0.36386881	0.98048242	ENSG00000198482	ZNF808
38.0784558	0.557065032	0.573115201	0.971994864	0.331053104	0.976026345	ENSG00000216775	AL109918.1
40.07255946	-0.005643788	0.647018297	-0.008722764	0.993040329	0.999527751	ENSG00000178409	BEND3
39.55931785	1.092372429	0.564559918	1.934909644	0.053001385	0.847598083	ENSG00000101945	SUV39H1
39.22556559	0.23602344	0.508194047	0.464435664	0.642335643	0.996740283	ENSG00000196632	WNK3
37.45041475	1.442760906	0.627023995	2.300966018	0.021393552	0.73997555	ENSG00000003249	DBNDD1
39.03748501	-0.502571944	0.595216556	-0.84435142	0.398473061	0.988378925	ENSG00000159214	CCDC24
38.86686787	0.811339896	0.727500724	1.115242733	0.264746418	0.962465655	ENSG00000213700	RPL17P50
38.37468782	0.165003533	0.659108861	0.250343369	0.802321821	0.999467288	ENSG00000153363	LINC00467
39.95554389	0.249867335	0.68230389	0.366211212	0.714207476	0.99900742	ENSG00000272870	AC097534.2
38.65269982	0.005777549	0.471347639	0.012257511	0.990220166	0.999527751	ENSG00000185261	KIAA0825
38.75316815	-0.985341433	0.887563974	-1.110163845	0.26692843	0.962465655	ENSG00000115919	KYNU
39.38776213	-0.452604905	0.647882102	-0.698591463	0.484807381	0.991892545	ENSG00000046889	PREX2
38.48042419	0.448860743	0.485105683	0.925284445	0.354817968	0.977691382	ENSG00000213918	DNASE1
39.07537344	0.241092603	0.513398838	0.469600991	0.638640116	0.996553586	ENSG00000268713	AC005261.3
39.09861196	0.751105126	0.589098758	1.275007147	0.202306712	0.954310328	ENSG00000181450	ZNF678
39.07143472	-1.10897352	0.505254342	-2.19488172	0.028172083	0.769167109	ENSG00000225031	EIF4BP7
38.76444397	0.701060173	0.470113372	1.491257673	0.135893857	0.934144427	ENSG00000278768	BACE1-AS
37.41028425	-0.776816964	0.568498598	-1.366436024	0.171802148	0.944422693	ENSG00000143429	LSP1P4
37.60511807	-1.514761402	0.846511733	-1.789415719	0.07354789	0.888663898	ENSG00000245812	LINC02202
38.2567717	0.791927497	0.535655297	1.478427454	0.139293401	0.935644597	ENSG00000198039	ZNF273
37.88105271	-0.537014391	0.622880168	-0.862147197	0.388606522	0.986799595	ENSG00000110200	ANAPC15
37.69612691	-0.514712407	0.781032085	-0.659015702	0.509885686	0.993513219	ENSG00000268894	PLCE1-AS1
37.6675953	1.728233986	0.7335056	2.356129232	0.018466492	0.726053908	ENSG00000175643	RMI2
37.98282001	-1.080768085	0.661007293	-1.635032012	0.102042337	0.92169898	ENSG00000233098	CCDC144NL-AS1
38.11993388	0.336991334	0.467399985	0.720991323	0.470914854	0.99135833	ENSG00000264538	SUZ12P1
37.83282409	0.464736727	0.571805596	0.812753024	0.416359674	0.990226751	ENSG00000105639	JAK3
38.69299594	-0.693899359	0.474973161	-1.460923303	0.144036491	0.935644597	ENSG00000230979	AC079250.1
37.04629558	0.398311066	0.613115669	0.649650769	0.515917831	0.993513219	ENSG00000173926	3-Mar
39.0334782	1.669977081	0.669379555	2.494813396	0.012602338	0.660006295	ENSG00000274602	PI4KAP1
37.77730734	-0.552545165	0.542079025	-1.019307405	0.30805705	0.973427483	ENSG00000162738	VANGL2
37.65580641	0.122890042	0.637388904	0.192802293	0.847113808	0.999467288	ENSG00000247828	TMEM161B-AS1
37.50194502	-0.879897174	0.5778165	-1.522796898	0.127809526	0.928627271	ENSG00000149201	CCDC81
38.42643528	0.057023333	0.501783609	0.113641284	0.90952216	0.999527751	ENSG00000143498	TAF1A
38.29545974	-0.587990756	0.475416161	-1.236791689	0.216164428	0.954759278	ENSG00000204179	PTPN20
37.99094496	0.071939449	0.626981613	0.114739328	0.908651741	0.999527751	ENSG00000154040	CABYR
38.21781556	0.385226559	0.542303327	0.710352564	0.477485531	0.99135833	ENSG00000169598	DFFB
38.25318026	0.193317712	0.581759763	0.332298183	0.739664111	0.999467288	ENSG00000148225	WDR31
38.429734	0.534371706	0.536962318	0.99517543	0.319650949	0.974964731	ENSG00000166341	DCHS1
37.81923208	0.745970771	0.608545396	1.225826005	0.220264159	0.956884001	ENSG00000188599	NPIPP1
37.76562336	-0.009494733	0.499229808	-0.019018762	0.984826139	0.999527751	ENSG00000119782	FKBP1B
38.42366007	0.04434367	0.550531443	0.080547026	0.935802197	0.999527751	ENSG00000169994	MYO7B
37.85223063	-0.335926221	0.569953728	-0.589392093	0.555598279	0.99483168	ENSG00000180769	WDFY3-AS2
37.28752512	0.80674565	0.517024407	1.560362798	0.118674171	0.923922314	ENSG00000214176	PLEKHM1P1
37.93169456	-0.141174067	0.596210777	-0.2367855	0.812823201	0.999467288	ENSG00000105227	PRX
38.46559608	0.348503605	0.782010911	0.445650566	0.655849678	0.997568624	ENSG00000145242	EPHA5
38.05376895	0.102284167	0.522380846	0.195803823	0.844763717	0.999467288	ENSG00000187187	ZNF546
37.75849539	-0.325775739	0.591011759	-0.551217017	0.581484916	0.99483168	ENSG00000132781	MUTYH
37.7961079	1.557613289	0.88363724	1.762729339	0.07794615	0.897052166	ENSG00000006606	CCL26
36.89556691	-0.938497011	0.614118195	-1.52820258	0.126462248	0.928627271	ENSG00000237807	AC022034.1
37.3641998	2.315645407	1.084175528	2.135858398	0.032690957	0.775963115	ENSG00000166803	PCLAF
37.09639564	0.156107793	0.572734477	0.272565735	0.785187055	0.999467288	ENSG00000233461	AL445524.1
38.17169629	-0.573330095	0.523066046	-1.096095033	0.273037188	0.962465655	ENSG00000221843	C2orf16
37.45541399	-1.084962187	0.675453294	-1.606272701	0.108214006	0.923922314	ENSG00000135114	OASL
37.55320117	0.32636783	0.515932748	0.632578241	0.5270091	0.99483168	ENSG00000197776	KLHDC1

37.77559001	1.067792476	0.475187931	2.247095111	0.024633949	0.749756093	ENSG00000228526	MIR34AHG
37.75866754	0.241692577	0.817055021	0.295809426	0.767375624	0.999467288	ENSG00000146205	ANO7
37.70842318	0.861515097	0.489848133	1.758739167	0.078621822	0.897052166	ENSG00000163738	MTHFD2L
37.27308189	0.691489256	0.539328877	1.282129116	0.199797357	0.954310328	ENSG00000173917	HOXB2
37.55994956	0.125554749	0.581810665	0.215800013	0.829143638	0.999467288	ENSG00000271605	MILR1
38.65187874	-0.456213199	0.575726553	-0.792412989	0.428119908	0.991301167	ENSG00000152076	CCDC74B
36.43319411	-0.394132498	0.632093897	-0.623534731	0.532933186	0.99483168	ENSG00000156463	SH3RF2
37.11434104	-0.644434188	0.639041839	-1.008438179	0.313244152	0.973427483	ENSG00000117676	RPS6KA1
38.14573581	0.410100183	0.522207398	0.785320515	0.43226567	0.991301167	ENSG00000162994	CLHC1
35.68412771	0.184033811	0.855253427	0.215180443	0.829626638	0.999467288	ENSG00000047457	CP
37.31885314	0.119092724	0.45126932	0.263906096	0.791852278	0.999467288	ENSG00000134146	DPH6
36.80251416	-0.226420213	0.530661421	-0.426675474	0.669615717	0.997568624	ENSG00000272667	AC012306.2
37.17197675	-0.559423897	0.497128586	-1.125310257	0.260457585	0.962465655	ENSG00000242110	AMACR
37.33536059	-0.155998248	0.541657473	-0.288001654	0.773345473	0.999467288	ENSG00000150455	TIRAP
36.92901758	1.520039758	1.005187743	1.51219488	0.130484293	0.929373153	ENSG00000123407	HOXC12
37.3443459	0.152689132	0.575266642	0.265423233	0.790683441	0.999467288	ENSG00000174327	SLC16A13
37.02487449	2.4385953	1.11038136	2.196178167	0.028079193	0.769167109	ENSG00000161888	SPC24
36.75677987	0.648611158	0.652370174	0.99423791	0.320107056	0.974969677	ENSG00000113645	WWC1
36.95648469	-1.44593784	0.792005052	-1.825667445	0.06790037	0.87420149	ENSG00000100336	APOL4
37.83586723	-0.020539456	0.58361902	-0.03519326	0.971925637	0.999527751	ENSG00000144792	ZNF660
37.02523757	0.272765604	0.540091028	0.505036356	0.613533301	0.99483168	ENSG00000204789	ZNF204P
36.17957402	0.030557041	0.670848754	0.045549822	0.963669064	0.999527751	ENSG00000259345	AC013652.1
36.57053138	0.297950287	0.597605598	0.498573453	0.618079911	0.99483168	ENSG00000259768	AC004943.2
36.92629913	-0.782434632	0.839550294	-0.931968743	0.351352679	0.977691382	ENSG00000135324	MRAP2
36.91838526	0.212075212	0.709067168	0.299090442	0.764871039	0.999467288	ENSG00000182107	TMEM30B
36.856029	-0.104550682	0.58079287	-0.180013714	0.857141802	0.999467288	ENSG00000267100	ILF3-DT
36.70851792	-0.166049944	0.505788647	-0.328299074	0.742685544	0.999467288	ENSG00000145990	GFOD1
37.44829966	0.372540727	0.558351778	0.667215081	0.50463476	0.993513219	ENSG00000238083	LRRC37A2
36.6617458	-0.57960555	0.565781443	-1.024433651	0.305630494	0.973427483	ENSG00000180448	ARHGAP45
35.78161588	-0.641777059	0.702724655	-0.913269593	0.361100771	0.979465649	ENSG00000176472	ZNF575
36.65850169	0.078993759	0.574475656	0.137505842	0.890630975	0.999467288	ENSG00000257702	LBX2-AS1
36.5711097	0.570732189	0.663493244	0.860192916	0.389682709	0.987216662	ENSG00000189007	ADAT2
36.11138458	-0.390018927	0.48389046	-0.806006647	0.420239016	0.990778994	ENSG00000135218	CD36
36.9609325	-0.62945366	0.499372351	-1.260489611	0.207492793	0.954310328	ENSG00000184508	HDDC3
36.47006693	1.819717374	1.154520701	1.576166951	0.114987333	0.923922314	ENSG00000051341	POLQ
36.70976538	0.13577635	0.495507622	0.274014655	0.784073359	0.999467288	ENSG00000256745	AP002784.2
35.41846309	0.14633982	0.833148946	0.175646649	0.860571552	0.999467288	ENSG00000137673	MMP7
37.44688275	-0.751303266	0.7175892	-1.046982405	0.295107694	0.970706863	ENSG00000241360	PDXP
37.25974577	-0.445660232	0.617626773	-0.721568836	0.470559605	0.99135833	ENSG00000146411	SLC2A12
36.76735873	3.081598278	1.214512036	2.537313906	0.011170674	0.644117389	ENSG00000065328	MCM10
36.02979938	0.695695304	0.671957663	1.035326096	0.300516647	0.972186492	ENSG00000285928	AC103591.4
36.12332642	0.593667377	0.488152943	1.216150359	0.22392765	0.956884001	ENSG00000269973	AC010969.2
36.60730964	0.590461267	0.591087568	0.998940426	0.317823551	0.974768742	ENSG00000280206	AC026401.3
36.3986666	-0.043342782	0.547181129	-0.079211032	0.93686477	0.999527751	ENSG00000234009	RPL5P34
37.57435941	-0.892562511	0.761141598	-1.172662897	0.240931016	0.962465655	ENSG00000174564	IL20RB
36.13711896	0.319119737	0.488905099	0.652723274	0.513934695	0.993513219	ENSG00000160447	PKN3
36.78473018	0.543834694	0.600519784	0.905606624	0.365144101	0.981269214	ENSG00000162769	FLVCR1
35.99988219	0.823079564	0.516530888	1.593475983	0.111053452	0.923922314	ENSG00000229320	KRT8P12
37.62273934	0.590549526	1.038261002	0.568787159	0.56950059	0.99483168	ENSG00000183775	KCTD16
36.37240138	1.112905982	0.493034716	2.257256835	0.023992031	0.744948012	ENSG00000232593	KANTR
36.64033182	-0.324578291	0.598774504	-0.542070996	0.587769599	0.99483168	ENSG00000062282	DGAT2
36.5020856	1.205824583	0.888222314	1.357570694	0.174599947	0.946176003	ENSG00000165490	DDIAS
37.36816702	0.088290185	0.71908772	0.122780827	0.902280657	0.999527751	ENSG00000078018	MAP2
35.8912091	0.928763998	0.735727254	1.262375416	0.206813731	0.954310328	ENSG00000138642	HERC6
36.37792883	-0.280068195	0.5330903	-0.525367269	0.599327903	0.99483168	ENSG00000254535	PABPC4L
36.29622426	0.394545264	0.477269897	0.826671168	0.408423468	0.990226751	ENSG00000184154	LRTOMT
36.15664664	0.329982447	0.624583961	0.528323601	0.597274754	0.99483168	ENSG00000138587	MNS1
36.00519526	-0.747612525	0.546324129	-1.368441345	0.17117397	0.944422693	ENSG00000237440	ZNF737
36.84853245	-0.550211175	0.616695741	-0.892192272	0.372289884	0.98211172	ENSG00000233682	AL356417.2
36.40651505	0.100102098	0.484758018	0.206499108	0.836401061	0.999467288	ENSG00000196378	ZNF34
36.66943748	1.11982025	0.584760349	1.91500715	0.055491592	0.847598083	ENSG00000186638	KIF24
35.85526371	0.054735861	0.544035757	0.100610778	0.919859441	0.999527751	ENSG00000179627	ZBTB42
36.33868096	0.589106598	0.47591884	1.237829959	0.215779117	0.954310328	ENSG00000188868	ZNF563
36.02996944	-0.694925021	0.568179977	-1.223072	0.221302503	0.956884001	ENSG00000170549	IRX1

36.44353922	-0.256120245	0.560672866	-0.456808704	0.647808549	0.997368416	ENSG00000171791	BCL2
35.72904061	-1.370667282	0.562207322	-2.438010372	0.01476835	0.688062202	ENSG00000087076	HSD17B14
37.80103545	0.812733228	0.859450786	0.945642544	0.344330937	0.977691382	ENSG00000274276	CBSL
35.86437559	0.714979004	0.553076043	1.29273183	0.196103825	0.954310328	ENSG00000198814	GK
37.2206016	-2.797069928	0.744446826	-3.757246092	0.000171794	0.09924856	ENSG00000130208	APOC1
36.43716494	0.269292494	0.518390834	0.51947773	0.603427639	0.99483168	ENSG00000172748	ZNF596
34.91200143	-0.164512214	0.83694293	-0.19656324	0.84416934	0.999467288	ENSG00000198133	TMEM229B
36.47887821	0.878791465	0.537619176	1.634598437	0.102133256	0.92169898	ENSG00000180626	ZNF594
35.38059648	-0.248743191	0.773832001	-0.321443402	0.747874397	0.999467288	ENSG00000108984	MAP2K6
36.14938868	0.060868702	0.500340867	0.121654468	0.903172674	0.999527751	ENSG00000197013	ZNF429
35.68508991	1.33308478	0.60716927	2.195573532	0.028122482	0.769167109	ENSG00000197989	SNHG12
35.70052588	0.072464967	0.503331694	0.143970602	0.885523684	0.999467288	ENSG00000267640	AC016582.3
34.68034725	0.383114988	0.688164985	0.556719677	0.57771897	0.99483168	ENSG00000240875	LINC00886
35.12160512	-0.542189663	0.585425462	-0.926146364	0.354369922	0.977691382	ENSG00000221990	EXOC3-AS1
35.22743327	-1.285409309	0.66030055	-1.946703374	0.051570307	0.843646143	ENSG00000204131	NHSL2
36.24026074	0.289226068	0.649329165	0.445422882	0.656014179	0.997568624	ENSG00000177575	CD163
35.27770691	-0.092273565	0.513808311	-0.179587529	0.857476396	0.999467288	ENSG00000183048	SLC25A10
36.59244072	-0.586972083	0.876251614	-0.669867049	0.502942547	0.993513219	ENSG00000268941	LINC01711
35.3926082	0.014888	0.481571195	0.030915471	0.975336952	0.999527751	ENSG00000256771	ZNF253
35.59094687	0.343169074	0.509095312	0.674076279	0.500262819	0.993513219	ENSG00000105792	CFAP69
35.9430976	0.317501809	0.599063029	0.529997335	0.596113779	0.99483168	ENSG00000204959	ARHGEF34P
35.60936055	0.970876041	0.764772873	1.269495918	0.204264246	0.954310328	ENSG00000147536	GINS4
35.7626194	0.37392502	0.524837274	0.712458963	0.476180615	0.99135833	ENSG00000160271	RALGDS
36.30462842	-0.892566359	0.567210145	-1.57360789	0.115578128	0.923922314	ENSG00000198417	MT1F
35.77352608	0.723361209	0.605717064	1.194222934	0.232390774	0.959575179	ENSG00000180479	ZNF571
35.97112377	-0.212600048	0.561266438	-0.378786319	0.704846547	0.99900742	ENSG00000010318	PHF7
35.71285799	-0.8693273	0.567014193	-1.533166738	0.125234777	0.928052123	ENSG00000120915	EPHX2
35.10978422	0.433870194	0.532360274	0.814993559	0.415075997	0.990226751	ENSG00000172458	IL17D
34.77803406	-0.80286541	0.785825443	-1.021684163	0.306930409	0.973427483	ENSG00000136167	LCP1
36.53821714	-0.348778788	0.598848364	-0.582415864	0.560286598	0.99483168	ENSG00000046604	DSG2
35.0070585	1.117658192	0.926519371	1.206297706	0.227702722	0.958865115	ENSG00000124134	KCNS1
35.74385938	-0.227122805	0.487400659	-0.465987891	0.641224168	0.996553586	ENSG00000197372	ZNF675
35.74340818	0.156859002	0.508269551	0.308613809	0.757615316	0.999467288	ENSG00000100147	CCDC134
34.65346655	-0.033371093	0.683842372	-0.048799393	0.961079166	0.999527751	ENSG00000143845	ETNK2
34.70386232	-0.274092647	0.777316572	-0.352613924	0.724377897	0.999456245	ENSG00000178662	CSRNP3
35.78121471	-0.521803649	0.642671376	-0.811929189	0.416832264	0.990226751	ENSG00000111319	SCNN1A
35.22906867	-0.505005237	0.547905031	-0.921702134	0.356683967	0.979427314	ENSG00000249565	SERBP1P5
36.11889282	0.521272951	0.579261203	0.899892739	0.368177335	0.981269214	ENSG00000285517	LINC00941
35.03250661	-0.081027155	0.712676362	-0.113694181	0.909480225	0.999527751	ENSG00000250722	SELENOP
35.81969774	-0.854269708	0.649082687	-1.316118463	0.188134285	0.949094286	ENSG00000254245	PCDHGA3
35.46503338	-0.062021577	0.502809334	-0.123350091	0.901829877	0.999527751	ENSG00000127589	TUBBP1
35.65855139	0.639011762	0.494916214	1.291151398	0.196651184	0.954310328	ENSG00000250091	DNAH10OS
35.42357001	0.482575466	0.481633032	1.001956748	0.316364483	0.974768742	ENSG00000167302	TEPSIN
35.44812562	0.786706509	0.578966306	1.35881225	0.174206091	0.945531217	ENSG00000269834	ZNF528-AS1
34.72075596	-0.422250296	0.595606013	-0.708942299	0.478360284	0.99135833	ENSG00000113356	POLR3G
35.34955398	0.950601137	0.601651753	1.579985651	0.114110152	0.923922314	ENSG00000133624	ZNF767P
36.39200271	0.522168986	0.64139	0.814120871	0.415575711	0.990226751	ENSG00000182010	RTKN2
36.86744696	0.003659858	0.716905613	0.005105076	0.995926756	0.999527751	ENSG00000004139	SARM1
35.32238435	0.648247769	0.651770673	0.994594871	0.319933343	0.974964731	ENSG00000117586	TNFSF4
35.26527758	-0.918823658	0.515437287	-1.78260999	0.074649812	0.893390506	ENSG00000225953	SATB2-AS1
35.1932083	0.683757909	0.552233587	1.238167917	0.215653805	0.954310328	ENSG00000169282	KCNAB1
34.69268867	0.713730729	0.660687602	1.080284733	0.280015406	0.963267117	ENSG00000014138	POLA2
34.92279498	-0.031882868	0.485396297	-0.0656842	0.947629252	0.999527751	ENSG00000182359	KBTBD3
36.75612032	-0.302087425	0.84615426	-0.357012237	0.721082645	0.99900742	ENSG00000205710	C17orf107
34.11169801	0.406011081	0.606264391	0.669693102	0.50305345	0.993513219	ENSG00000076944	STXBP2
35.39596348	-0.057170036	0.524493047	-0.10900056	0.913202046	0.999527751	ENSG00000235954	TTC28-AS1
34.68008733	-1.540215295	0.738516515	-2.085552948	0.037019137	0.790190505	ENSG00000227456	LINC00310
34.42556038	0.284762938	0.597726129	0.47641039	0.633782034	0.995376704	ENSG00000170989	S1PR1
35.63033112	1.027313813	0.606548094	1.69370545	0.090321268	0.912150398	ENSG00000285867	BX470102.2
35.41785059	0.092296097	0.654190769	0.141084377	0.887803286	0.999467288	ENSG00000176887	SOX11
35.21775067	1.146416894	0.502685637	2.280584144	0.022573066	0.744905651	ENSG00000155657	TTN
34.47780347	0.084246078	0.640124519	0.13160889	0.895293653	0.999467288	ENSG00000109794	FAM149A
35.63194276	0.027644948	0.638252798	0.043313477	0.965451649	0.999527751	ENSG00000251136	AF117829.1
35.40736198	0.246454544	0.498116224	0.494773171	0.620760244	0.99483168	ENSG00000168010	ATG16L2

35.64082319	0.096002326	1.075074762	0.089298279	0.928844861	0.999527751	ENSG00000050438	SLC4A8
35.80278134	-0.228487748	0.571007145	-0.400148668	0.68904702	0.998869317	ENSG00000161835	GRASP
35.38881246	-0.339101447	0.516640808	-0.656358232	0.511593658	0.993513219	ENSG00000175106	TVP23C
34.61863443	0.515334339	0.595489894	0.865395609	0.38682169	0.986730073	ENSG00000188766	SPRED3
38.04399605	-1.277512405	1.234121038	-1.035159734	0.30059432	0.972186492	ENSG00000066248	NGEF
34.36600369	-0.090448647	0.703390865	-0.128589453	0.897682512	0.999467288	ENSG00000164620	RELL2
35.21162253	-0.187322649	0.578370647	-0.323879938	0.746028934	0.999467288	ENSG00000185972	CCIN
35.326142	0.483495172	0.604681174	0.799586944	0.423950154	0.991301167	ENSG00000119397	CNTRL
34.59299332	-0.161127082	0.548992537	-0.293495942	0.769143101	0.999467288	ENSG00000173898	SPTBN2
34.6710936	0.171662637	0.811789145	0.2114621	0.832526704	0.999467288	ENSG00000139880	CDH24
35.12309583	-0.072246772	0.610315257	-0.118376152	0.905769621	0.999527751	ENSG00000205534	SMG1P2
36.01222506	-0.934116082	0.642147549	-1.454675151	0.145759215	0.935644597	ENSG00000089091	DZANK1
35.50472591	0.061985589	0.525735414	0.117902632	0.906144808	0.999527751	ENSG00000198185	ZNF334
34.2317949	0.599013851	0.574040307	1.043504861	0.296714531	0.971096299	ENSG00000185900	POMK
34.69670645	-0.617560209	0.579040283	-1.06652374	0.286186953	0.964826902	ENSG00000125122	LRRC29
34.83168085	0.973888986	0.551937593	1.764491129	0.077649328	0.897052166	ENSG00000131470	PSMC3IP
34.80813041	0.043614155	0.511932118	0.085195191	0.932106213	0.999527751	ENSG00000162777	DENND2D
35.4331022	0.548084248	0.780837348	0.701918587	0.482729937	0.991759385	ENSG00000164078	MST1R
34.70503432	0.048602102	0.601648922	0.080781499	0.935615722	0.999527751	ENSG00000165185	KIAA1958
34.63236082	0.303511636	0.64693108	0.469156059	0.638958091	0.996553586	ENSG00000257027	AC010186.3
34.21804088	0.108972883	0.590226662	0.184628873	0.853520138	0.999467288	ENSG00000109667	SLC2A9
35.46489136	-0.186987032	0.601706089	-0.310761409	0.755982009	0.999467288	ENSG00000102053	ZC3H12B
34.76265593	0.276333553	0.703790625	0.392636025	0.694588328	0.998869317	ENSG00000184545	DUSP8
35.80007223	-0.615883518	0.780444353	-0.789144692	0.430027443	0.991301167	ENSG00000099869	IGF2-AS
34.55106807	0.798110633	0.492283298	1.621242558	0.104965629	0.92169898	ENSG00000064763	FAR2
34.79922853	0.339412009	0.522795478	0.64922522	0.516192813	0.993513219	ENSG00000230896	AL604028.1
34.60319978	0.034213628	0.570316949	0.059990551	0.95216316	0.999527751	ENSG00000176896	TCEANC
34.70234386	0.30291738	0.576547259	0.525399047	0.599305816	0.99483168	ENSG00000102349	KLF8
35.21612976	-0.850686807	0.530240828	-1.604340446	0.10863903	0.923922314	ENSG00000224546	EIF4BP3
34.75405459	0.718102858	0.541564933	1.325977394	0.184847185	0.949094286	ENSG00000069493	CLEC2D
34.05779425	-0.049899239	0.693608877	-0.071941466	0.942648491	0.999527751	ENSG00000152475	ZNF837
34.45459472	0.810824762	0.606925226	1.335954955	0.181563997	0.949094286	ENSG00000163827	LRRC2
33.56491429	0.032796246	0.590315489	0.05555715	0.955694601	0.999527751	ENSG00000215218	UBE2QL1
34.97848889	0.090537592	0.622079187	0.145540301	0.884284299	0.999467288	ENSG00000182648	LINC01006
34.02936278	-0.73782038	0.626109713	-1.178420274	0.238629099	0.962465655	ENSG00000108176	DNAJC12
34.22370126	0.043262779	0.547128789	0.079072387	0.936975047	0.999527751	ENSG00000171224	FAM241B
34.99562542	-0.252568131	0.545630023	-0.462892657	0.643441311	0.997097166	ENSG00000180881	CAPS2
34.03451636	-0.872257488	0.546136252	-1.597142627	0.110233918	0.923922314	ENSG00000188681	TEKT4P2
34.466761	-1.158455995	0.539958708	-2.145452936	0.031916651	0.775963115	ENSG00000116299	KIAA1324
34.75951509	0.127542992	0.542665471	0.235030601	0.814184982	0.999467288	ENSG00000072133	RPS6KA6
34.9131143	0.587387937	0.604324499	0.97197439	0.331063289	0.976026345	ENSG00000008300	CELSR3
34.52323552	-0.392878474	0.717504143	-0.547562655	0.583992238	0.99483168	ENSG00000133401	PDZD2
34.42229568	0.244040208	0.474464002	0.51434926	0.607007828	0.99483168	ENSG00000164241	C5orf63
34.6154082	0.175443853	0.575082563	0.305075938	0.760308317	0.999467288	ENSG00000147180	ZNF711
33.9406946	-0.002135304	0.858736187	-0.002486566	0.99801601	0.999527751	ENSG00000124126	PREX1
33.45622163	2.804840561	1.006504419	2.786714601	0.005324535	0.487175435	ENSG00000093009	CDC45
35.08522286	-0.314396542	0.593579627	-0.529661949	0.596346335	0.99483168	ENSG00000168826	ZBTB49
34.26698626	-0.483433385	0.527077567	-0.917195904	0.359039993	0.979465649	ENSG00000078487	ZCWPW1
33.90412439	0.673948795	0.492539295	1.368314777	0.171213567	0.944422693	ENSG00000165568	AKR1E2
33.74765577	-0.524980387	0.583008662	-0.900467561	0.367871481	0.981269214	ENSG00000278709	NKILA
33.74788103	-0.18035491	0.588995968	-0.306207376	0.759446757	0.999467288	ENSG00000258777	HIF1A-AS1
33.61218272	-0.005574051	0.507683026	-0.010979393	0.991239888	0.999527751	ENSG00000125454	SLC25A19
33.78840366	-0.054924129	0.684963015	-0.08018554	0.936089691	0.999527751	ENSG00000160712	IL6R
33.91281825	-0.47440707	0.49939187	-0.949969549	0.342127725	0.977691382	ENSG00000188295	ZNF669
34.38070226	-1.154912571	0.601583664	-1.919787122	0.054884794	0.847598083	ENSG00000247081	BAALC-AS1
33.58088408	0.45033829	0.565285716	0.796656058	0.425650811	0.991301167	ENSG00000254615	AC027031.2
33.97841949	-0.896885111	0.534781343	-1.677106208	0.093521715	0.921147998	ENSG00000176533	GNG7
33.93209065	0.87364232	0.586687901	1.489109149	0.136458629	0.934144427	ENSG00000172244	C5orf34
33.797895	-1.00341339	0.86193367	-1.164142236	0.244366375	0.962465655	ENSG00000135547	HEY2
33.41916627	-0.216268998	0.600045744	-0.360420851	0.718532436	0.99900742	ENSG00000126822	PLEKHG3
31.57773113	0.730391053	1.114227586	0.655513346	0.512137296	0.993513219	ENSG00000128849	CGNL1
33.71628489	-0.147970222	0.714626518	-0.207059517	0.835963378	0.999467288	ENSG00000183196	CHST6
33.16807807	-0.486226865	0.588548006	-0.826146482	0.408721003	0.990226751	ENSG00000242960	FTH1P23
33.52899778	-1.799393846	0.639794127	-2.812457587	0.004916451	0.472943531	ENSG00000109705	NKX3-2

33.98107854	-0.119695337	0.685782327	-0.174538381	0.86144237	0.999467288	ENSG00000187372	PCDHB13
34.48630003	-0.292455909	0.59116298	-0.494712826	0.620802847	0.99483168	ENSG00000177640	CASC2
33.66152441	-0.779400733	0.627433153	-1.242205212	0.214160857	0.954310328	ENSG00000157021	FAM92A1P1
34.29803861	0.674012806	0.586209163	1.149782107	0.250233627	0.962465655	ENSG00000267397	AC090229.1
33.72269493	0.367138468	0.532544489	0.689404314	0.490568869	0.993513219	ENSG00000197933	ZNF823
34.67708341	-0.948873275	0.69744569	-1.360497726	0.173672472	0.944422693	ENSG00000177989	ODF3B
32.75907456	-1.349493751	0.68695878	-1.964446471	0.049478338	0.836346249	ENSG00000116661	FBXO2
33.3589377	-0.143182391	0.581567566	-0.246200784	0.805526809	0.999467288	ENSG00000234171	RNASEH1-AS1
33.28464784	0.256576139	0.533360099	0.481056118	0.630476615	0.99483168	ENSG00000144182	LIPT1
33.92243239	-0.258908418	0.548700973	-0.471857042	0.637028832	0.99653524	ENSG00000213214	ARHGEF35
33.78557238	0.455664837	0.632419017	0.720510967	0.47121045	0.99135833	ENSG00000129028	THAP10
35.73829076	-0.586670657	2.014710986	-0.291193457	0.770903366	0.999467288	ENSG00000186847	KRT14
33.72547422	-0.748545119	0.549314757	-1.362688893	0.17298058	0.944422693	ENSG00000260077	AC104794.2
33.81819188	0.560279442	0.975974959	0.574071534	0.565919395	0.99483168	ENSG00000156298	TSPAN7
33.47212691	0.613977679	0.509207749	1.205750857	0.227913569	0.958865115	ENSG00000253161	LINC01605
34.54481485	-0.583389474	0.631443814	-0.923897678	0.355539592	0.978364583	ENSG00000278727	AC000403.1
34.01718289	-0.089383522	0.591484263	-0.151117329	0.879883163	0.999467288	ENSG00000100890	KIAA0391
33.25498684	0.991633803	0.608434731	1.629811305	0.103141384	0.92169898	ENSG00000119640	ACYP1
33.26594852	1.411295923	0.588076593	2.399850529	0.016401768	0.700860152	ENSG00000100629	CEP128
32.86675036	0.100823597	0.593725581	0.169815148	0.865155514	0.999467288	ENSG00000278619	MRM1
33.26321333	0.261719793	0.57272255	0.456974836	0.647689132	0.997368416	ENSG00000154065	ANKRD29
33.71653303	-0.512342581	0.54817803	-0.934628081	0.349980002	0.977691382	ENSG00000088727	KIF9
33.28702278	-0.482587626	0.5726245	-0.842764545	0.399360141	0.988378925	ENSG00000250303	AP002884.1
33.35760863	0.352290656	0.714734395	0.492897303	0.622085153	0.99483168	ENSG00000260257	AL035071.1
33.50606952	0.097754014	0.521558915	0.1874266	0.851326168	0.999467288	ENSG00000272106	AL691432.2
33.94971022	0.760894888	0.680062013	1.118861035	0.263199428	0.962465655	ENSG00000115687	PASK
33.81507647	-0.722379832	0.616485009	-1.171771935	0.241288633	0.962465655	ENSG00000126882	FAM78A
33.44125065	-0.595218338	0.514968985	-1.155833372	0.24774932	0.962465655	ENSG00000240087	RPSAP12
33.9859821	0.613583294	0.654081084	0.93808445	0.348201012	0.977691382	ENSG00000248124	RRN3P1
31.22110772	1.233307532	0.968008851	1.274066379	0.202639893	0.954310328	ENSG00000006210	CX3CL1
33.44091034	-0.29497201	0.51605897	-0.571585859	0.567602578	0.99483168	ENSG00000178935	ZNF552
33.2921036	-0.64409141	0.507476862	-1.2692035	0.204368493	0.954310328	ENSG00000118420	UBE3D
33.3584114	-0.21005252	0.520422161	-0.403619476	0.686492562	0.998869317	ENSG00000156172	C8orf37
33.87389169	-0.496285643	0.668075149	-0.742859009	0.457567045	0.99135833	ENSG00000177335	C8orf31
33.62976126	-0.76359905	0.76614682	-0.996674567	0.318922499	0.974768742	ENSG00000170412	GPRC5C
33.62930487	-0.415567055	0.587415123	-0.707450385	0.479286635	0.99135833	ENSG00000163749	CCDC158
33.28357884	0.495582078	0.561473787	0.882645084	0.377428062	0.982609538	ENSG00000183718	TRIM52
33.03479576	-0.135391698	0.496810379	-0.27252188	0.78522077	0.999467288	ENSG00000261652	C15orf65
33.11072376	0.196448761	0.578204192	0.339756722	0.734039742	0.999467288	ENSG00000198496	NBR2
33.32381231	0.729923934	0.59463319	1.227519665	0.219627335	0.956884001	ENSG00000153982	GDPD1
32.31616774	-0.381369885	0.77923695	-0.489414529	0.624548252	0.99483168	ENSG00000253163	AC109479.1
32.72884318	0.234967298	0.546454846	0.429984838	0.667206671	0.997568624	ENSG00000134769	DTNA
32.14298958	-0.248900066	0.66853672	-0.372305752	0.709665216	0.99900742	ENSG00000159231	CBR3
32.94025803	0.111570052	0.568136895	0.196378818	0.844313674	0.999467288	ENSG00000234518	PTGES3P1
32.41427112	-0.670559339	0.659927055	-1.016111302	0.309576388	0.973427483	ENSG00000232973	CYP1B1-AS1
32.47015134	0.513094377	0.612801515	0.837292931	0.402427932	0.988658335	ENSG00000231584	FAHD2CP
32.81484483	0.7957848	0.56114792	1.418137308	0.156150681	0.939464191	ENSG00000240032	LNCSRLR
32.3413852	0.181694015	0.896081108	0.202765144	0.839318603	0.999467288	ENSG00000129596	CDO1
33.41406582	0.025710671	0.536975474	0.047880531	0.961811456	0.999527751	ENSG00000224411	HSP90AA2P
32.36320678	-0.456066285	0.70347798	-0.648302147	0.516789546	0.993513219	ENSG00000137727	ARHGAP20
32.6646083	-0.257178453	0.533449686	-0.482104423	0.629731768	0.99483168	ENSG00000152784	PRDM8
35.95578141	-0.386471315	1.986934578	-0.194506311	0.845779454	0.999467288	ENSG00000221971	TTC4P1
33.26220318	-1.018966043	0.649981277	-1.567685223	0.116954612	0.923922314	ENSG00000035664	DAPK2
31.97736953	0.218052651	0.631694578	0.345186833	0.729953917	0.999467288	ENSG00000259426	AC027237.3
32.50998011	-0.101834814	0.551553635	-0.184632658	0.853517169	0.999467288	ENSG00000197114	ZGPAT
32.48335607	0.86525345	0.567049709	1.525886418	0.127038154	0.928627271	ENSG00000233527	ZNF529-AS1
32.54222072	0.795247027	0.676142193	1.17615353	0.23953353	0.962465655	ENSG00000128578	STRIP2
31.69145189	0.907595619	0.716836624	1.266112234	0.205472903	0.954310328	ENSG00000167536	DHRS13
33.06692454	-0.549759574	0.615186442	-0.89364709	0.371510743	0.981955904	ENSG00000235109	ZSCAN31
32.8683079	1.39240505	0.542207743	2.568028707	0.010227869	0.631286776	ENSG00000223959	AFG3L1P
33.32504722	0.177306001	0.675307111	0.2625561	0.79289274	0.999467288	ENSG00000130810	PPAN
33.35468333	0.137094713	0.559464995	0.245046096	0.806420739	0.999467288	ENSG00000127507	ADGRE2
32.38563086	-0.497367841	0.559110627	-0.889569643	0.373697012	0.98257891	ENSG00000234546	LNCTAM34A
32.73368013	-0.048881171	0.708454239	-0.068996935	0.944992059	0.999527751	ENSG00000236859	NIFK-AS1

32.42703048	1.675502794	0.68328758	2.452119491	0.014201749	0.68371955	ENSG00000138346	DNA2
32.65193568	-0.342102588	0.548249756	-0.623990406	0.532633886	0.99483168	ENSG00000257337	AC068888.1
33.492704	-1.572332396	0.729199896	-2.156243306	0.031064672	0.775963115	ENSG00000246985	SOCS2-AS1
32.84060394	-0.652594246	0.570678405	-1.143541162	0.252813975	0.962465655	ENSG00000105519	CAPS
33.16753042	-1.481341247	0.828418473	-1.78815574	0.073750885	0.888663898	ENSG00000203721	LINC00862
32.37145906	0.112004988	0.499880245	0.224063641	0.822707784	0.999467288	ENSG00000221821	C6orf226
32.69446895	-0.32602448	0.702651677	-0.463991606	0.642653759	0.996869106	ENSG00000248690	HAS2-AS1
32.19486399	0.616333188	0.575788928	1.070415143	0.284432486	0.963310634	ENSG00000187164	SHTN1
32.71919633	0.059105899	0.610687484	0.096785837	0.922896472	0.999527751	ENSG00000140451	PIF1
32.2608369	0.66483697	0.488898428	1.35986727	0.17387193	0.94495857	ENSG00000197020	ZNF100
32.24149616	-0.22892159	0.636349288	-0.359742038	0.719040052	0.99900742	ENSG00000250903	GMDS-DT
32.06504499	0.441798977	0.696957352	0.633896717	0.526148228	0.99483168	ENSG00000122824	NUDT10
31.95427654	-0.742266631	0.709603885	-1.046029547	0.295547391	0.970706863	ENSG00000103034	NDRG4
32.79022321	-0.635004718	0.521726421	-1.217122024	0.223557792	0.956884001	ENSG00000159885	ZNF222
31.16906345	-4.377377904	3.493706671	-1.252932291	0.210230349	0.954310328	ENSG00000188624	IGFL3
32.06995221	-0.289624279	0.560969779	-0.516292126	0.605650395	0.99483168	ENSG00000223745	CCDC18-AS1
31.94941058	-0.666253287	0.536712809	-1.24135902	0.21447315	0.954310328	ENSG00000198468	FLVCR1-DT
33.90977107	-0.887145062	1.028732868	-0.86236679	0.388485709	0.986799595	ENSG00000229628	AC073115.1
32.66917399	0.163744593	0.619349541	0.264381552	0.791485925	0.999467288	ENSG00000166529	ZSCAN21
32.16716796	-0.282099731	0.559354212	-0.50433111	0.614028719	0.99483168	ENSG00000186787	SPIN2B
32.41298214	0.614559168	0.51027282	1.204373707	0.22844517	0.95933456	ENSG00000160323	ADAMTS13
31.82092667	0.021201367	0.546927302	0.038764507	0.969078143	0.999527751	ENSG00000247271	ZBED5-AS1
32.55802491	0.553672242	0.880394826	0.62889084	0.529420523	0.99483168	ENSG00000076344	RGS11
33.05016118	-0.178229156	0.703343825	-0.253402603	0.799957116	0.999467288	ENSG00000244165	P2RY11
31.80233365	0.412638472	0.777497189	0.530726642	0.595608221	0.99483168	ENSG00000099957	P2RX6
32.32554104	-0.93111028	0.627084968	-1.484823154	0.137590677	0.934144427	ENSG00000220749	RPL21P28
32.0911608	-0.496314416	0.50236805	-0.987949803	0.323177233	0.975337095	ENSG00000259917	HNRNPLP2
32.47005835	0.532475367	1.052611562	0.505861218	0.612954079	0.99483168	ENSG00000171877	FRMD5
32.17725707	-0.465539082	0.549839483	-0.846681797	0.397172508	0.988378925	ENSG00000187994	RINL
32.59351823	-0.135979708	0.555999504	-0.244568039	0.806790914	0.999467288	ENSG00000143578	CREB3L4
31.90858865	0.284051316	0.730645638	0.388767552	0.697448106	0.998869317	ENSG00000112139	MDGA1
32.02490807	-0.276440851	0.761518172	-0.363012809	0.716595316	0.99900742	ENSG00000176428	VPS37D
32.90096531	0.309268069	0.722807767	0.427870429	0.668745458	0.997568624	ENSG00000119508	NR4A3
32.06717267	0.394603025	0.554190869	0.712034512	0.476443405	0.99135833	ENSG00000136828	RALGPS1
32.24875206	-0.126842499	0.765097778	-0.165785998	0.868325365	0.999467288	ENSG00000149294	NCAM1
31.79146475	0.619816625	0.669799854	0.925375873	0.354770424	0.977691382	ENSG00000184916	JAG2
32.01334267	0.535791994	0.690675957	0.775750174	0.437896509	0.991301167	ENSG00000137869	CYP19A1
32.23044514	-0.021371835	0.61476079	-0.034764473	0.97226755	0.999527751	ENSG00000257335	MGAM
32.33237824	1.130104325	0.532082104	2.123928461	0.033676126	0.775963115	ENSG00000246263	UBR5-AS1
32.58786619	0.331124312	0.721295457	0.459068899	0.646184689	0.997368416	ENSG00000260196	AC124798.1
33.80366093	-0.607147186	1.011881649	-0.600017982	0.548494251	0.99483168	ENSG00000285877	AC007448.4
31.37903229	-0.642821912	0.714365026	-0.89985076	0.368199677	0.981269214	ENSG00000180884	ZNF792
31.8295796	0.326736136	0.66540534	0.491033235	0.623402942	0.99483168	ENSG00000143036	SLC44A3
32.27128341	0.459089426	0.515668629	0.89027992	0.3733156	0.982329093	ENSG00000272750	AL592148.3
31.75286141	-0.608502186	0.510276511	-1.192494998	0.233067211	0.959575179	ENSG00000116035	VAX2
31.22370362	0.061208556	0.741684483	0.082526408	0.934228119	0.999527751	ENSG00000248610	HSPA8P4
32.23555151	-0.068363385	0.583972363	-0.11706613	0.906807649	0.999527751	ENSG00000120217	CD274
31.2016271	0.938828733	0.588727689	1.59467399	0.110785157	0.923922314	ENSG00000260400	AL513534.1
31.4552119	1.42151782	0.787529382	1.805034647	0.07106926	0.88331496	ENSG00000197467	COL13A1
31.80170581	-0.130367672	0.506300385	-0.257490763	0.796799945	0.999467288	ENSG00000176124	DLEU1
31.07951261	-0.611134715	0.742245846	-0.823358889	0.410303934	0.990226751	ENSG00000179841	AKAP5
31.54100862	-0.516041065	0.576184526	-0.895617708	0.370456975	0.981455968	ENSG00000138606	SHF
31.16796491	0.22157129	0.732055751	0.302669967	0.762141388	0.999467288	ENSG00000235027	AC068580.3
31.6161662	-0.149489139	0.69083479	-0.21638913	0.828684439	0.999467288	ENSG00000167799	NUDT8
31.59069657	0.092603431	0.596901065	0.155140334	0.876710685	0.999467288	ENSG00000107864	CPEB3
32.34481293	-0.820649243	0.557817443	-1.471178882	0.141242747	0.935644597	ENSG00000226049	TLK2P1
30.38252552	0.912219976	0.819362923	1.113328356	0.265567432	0.962465655	ENSG00000108691	CCL2
31.98171199	0.271812842	0.588311464	0.462022005	0.64406554	0.997332006	ENSG00000224837	GCSHP5
32.26840543	1.003432038	0.625529341	1.604132647	0.108684817	0.923922314	ENSG00000214021	TTLL3
31.82008498	0.765679872	0.583076353	1.313172569	0.189124805	0.949094286	ENSG00000178038	ALS2CL
32.10219565	1.42830141	0.784952591	1.819602134	0.068819617	0.87420149	ENSG00000183763	TRAIP
31.44778432	-0.37033158	0.647058813	-0.572330633	0.567098001	0.99483168	ENSG00000172780	RAB43
32.99902885	0.999258783	0.74397117	1.343141808	0.17922606	0.9469453	ENSG00000146094	DOK3
31.47882222	0.439939652	0.582513274	0.755243994	0.450102596	0.99135833	ENSG00000175567	UCP2

31.47478305	-1.293677788	0.702132193	-1.842498893	0.065402208	0.868202292	ENSG00000166816	LDHD
32.32726187	0.252377595	0.581285046	0.434171835	0.664163664	0.997568624	ENSG00000108379	WNT3
31.25171727	-0.95305817	0.608579233	-1.566037943	0.117339735	0.923922314	ENSG00000261371	PECAM1
31.03151345	0.177191221	0.730314255	0.242623254	0.808297265	0.999467288	ENSG00000144712	CAND2
32.43435788	-1.059318177	0.705039658	-1.502494457	0.132969458	0.933741583	ENSG00000055163	CYFIP2
31.62832065	0.054551471	0.528811436	0.103158645	0.917837061	0.999527751	ENSG00000151773	CCDC122
32.09086632	-1.254824045	0.649968783	-1.930591252	0.053533621	0.847598083	ENSG00000142920	AZIN2
31.03128248	0.079363502	0.617957883	0.128428659	0.897809752	0.999467288	ENSG00000144158	AL078621.1
31.24668024	3.468822997	1.805478989	1.921275749	0.054696953	0.847598083	ENSG00000129810	SGO1
31.98289536	-0.728916208	0.542261546	-1.344215193	0.17887881	0.9469453	ENSG00000214654	B3GNT10
31.48431355	0.528860924	0.627409921	0.842927257	0.399269128	0.988378925	ENSG00000138207	RBP4
31.49860969	0.345622811	0.538065284	0.642343636	0.520650088	0.993513219	ENSG00000185347	TEDC1
31.08903464	-0.501735867	0.578891104	-0.86671891	0.386096042	0.986422569	ENSG00000131781	FMO5
31.46424456	0.402113595	0.572279928	0.702651929	0.482272692	0.991525194	ENSG00000153234	NR4A2
31.5607694	-0.003999731	0.540438074	-0.007400904	0.994094987	0.999527751	ENSG00000113522	RAD50
31.57701233	0.403284024	0.604432894	0.667210585	0.504637631	0.993513219	ENSG00000267508	ZNF285
31.50490963	0.101341146	0.530464422	0.191042305	0.848492449	0.999467288	ENSG00000213533	STIMATE
31.95000804	0.163476305	0.539915576	0.302781235	0.762056585	0.999467288	ENSG00000165359	INTS6L
31.69226169	2.529628117	1.098818437	2.302134758	0.021327574	0.73997555	ENSG00000186193	SAPCD2
31.66591036	-1.286414376	0.77966001	-1.649968395	0.0989494	0.921147998	ENSG00000233588	CYP51A1P2
30.72536865	0.844131843	0.619077694	1.363531347	0.172715112	0.944422693	ENSG00000133983	COX16
30.79778139	-0.828550473	0.625041649	-1.325592421	0.184974738	0.949094286	ENSG00000214140	PRCD
31.8512387	1.484234289	1.003343729	1.479287951	0.139063372	0.935644597	ENSG00000116883	AL591845.1
31.27329845	0.814248998	0.593261599	1.372495706	0.169909175	0.944422693	ENSG00000117480	FAAH
31.6535667	-0.09458726	0.551942224	-0.171371668	0.863931531	0.999467288	ENSG00000280173	AC104447.1
30.86893716	0.228838094	0.656865366	0.348378992	0.727555577	0.999467288	ENSG00000273314	AC005229.4
31.32830743	-0.02720766	0.558163741	-0.048744943	0.961122559	0.999527751	ENSG00000243660	ZNF487
31.1482497	0.1493917	0.635681705	0.235010224	0.814200798	0.999467288	ENSG00000134070	IRAK2
30.25917506	0.281978685	0.972647433	0.289908425	0.771886296	0.999467288	ENSG00000112149	CD83
31.09695618	0.689884766	0.843979607	0.817418763	0.413689157	0.990226751	ENSG00000112761	WISP3
31.10781854	0.226747602	0.534256471	0.424417138	0.671261624	0.997568624	ENSG00000240527	AL365273.1
31.28724921	0.214689173	0.541029108	0.396816308	0.691502924	0.998869317	ENSG00000089723	OTUB2
30.54028061	-1.106798658	0.695581082	-1.591185681	0.111567794	0.923922314	ENSG00000260630	SNAI3-AS1
31.30332963	0.809401317	0.753526948	1.074150458	0.282755252	0.963267117	ENSG00000116985	BMP8B
31.70886733	-0.02237161	0.589477589	-0.037951587	0.969726282	0.999527751	ENSG00000166546	BEAN1
31.41510302	0.631675786	0.633449185	0.997200408	0.318667243	0.974768742	ENSG00000179598	PLD6
31.01493735	0.503640419	0.979147249	0.514366372	0.606995865	0.99483168	ENSG00000205129	C4orf47
30.11659208	-0.231225906	0.669037451	-0.345609809	0.729635973	0.999467288	ENSG00000105991	HOXA1
30.99118015	-0.045982714	0.485486301	-0.094714752	0.924541401	0.999527751	ENSG00000281332	LINC00997
31.56986325	0.715602169	0.729281342	0.981242942	0.326472953	0.976026345	ENSG00000183889	PKD1P1
31.10392207	0.56860773	0.551918717	1.030238172	0.302898215	0.972793733	ENSG00000226419	SLC16A1-AS1
31.03357625	-0.048257271	0.658377725	-0.073297242	0.941569587	0.999527751	ENSG00000158715	SLC45A3
31.03947118	0.300402553	0.595163588	0.504739468	0.613741836	0.99483168	ENSG00000188542	DUSP28
30.84589678	-0.554414213	0.692830712	-0.800215988	0.423585668	0.991301167	ENSG00000173421	CCDC36
29.4709468	-0.314928185	1.127712277	-0.279262886	0.780043086	0.999467288	ENSG00000137198	GMPR
30.50469675	0.101485405	0.538779925	0.188361519	0.850593263	0.999467288	ENSG00000168939	SPRY3
30.73366168	0.600467696	0.736440199	0.815365181	0.414863309	0.990226751	ENSG00000139190	VAMP1
30.7307543	-0.097379771	0.576751575	-0.168841795	0.865921083	0.999467288	ENSG00000182612	TSPAN10
30.92714998	0.39329475	0.534081654	0.73639442	0.461490699	0.99135833	ENSG00000152454	ZNF256
30.23040846	-0.682572206	0.616419046	-1.107318487	0.268156254	0.962465655	ENSG00000253683	AC027309.2
30.50289943	0.757870201	0.615752552	1.230803184	0.218396484	0.956884001	ENSG00000217648	AL136116.3
30.53014748	0.224690105	0.662823901	0.338989141	0.734617911	0.999467288	ENSG00000169891	REPS2
31.95544548	-0.983138715	0.987366197	-0.995718426	0.319386975	0.974964731	ENSG00000180720	CHRM4
30.69784181	0.666323637	0.547256116	1.217571841	0.223386721	0.956884001	ENSG00000107518	ATRNL1
30.7596553	-0.494607059	0.500662403	-0.987905335	0.323199012	0.975337095	ENSG00000225377	NRSN2-AS1
31.68100461	0.156997959	0.608699425	0.257923619	0.796465856	0.999467288	ENSG00000100181	TPTEP1
31.9973551	0.950854012	0.821939467	1.156841897	0.247336961	0.962465655	ENSG00000177943	MAMDC4
30.61479887	0.678883158	0.538346044	1.26105349	0.207289576	0.954310328	ENSG00000162241	SLC25A45
31.0547221	1.289363146	0.588840108	2.189665969	0.028548471	0.770696352	ENSG00000161647	MPP3
30.97829383	-0.012873656	0.688226942	-0.01870554	0.985076009	0.999527751	ENSG00000088881	EBF4
29.88723947	-0.456884064	0.746324879	-0.612178526	0.540419648	0.99483168	ENSG00000176659	C20orf197
30.98313294	0.545722908	0.543699063	1.003722362	0.315512455	0.974768742	ENSG00000212916	MAP10
31.04274378	-0.108675297	0.633841837	-0.171454913	0.86386608	0.999467288	ENSG00000113209	PCDHB5
30.26023521	0.537136629	0.70188957	0.765272276	0.444109429	0.99135833	ENSG00000165084	C8orf34

30.31450047	1.784686107	0.94129698	1.895986224	0.057961866	0.852306368	ENSG00000104267	CA2
30.33927985	-1.17393088	0.670987482	-1.74955705	0.080194777	0.897807954	ENSG00000196388	INCA1
30.02339051	-0.941372697	0.619797886	-1.518838187	0.128803231	0.928627271	ENSG00000186806	VSIG10L
30.01828927	1.09260785	0.746474469	1.463690849	0.143278437	0.935644597	ENSG00000143942	CHAC2
30.34676846	1.804847096	2.386881248	0.756152866	0.449557549	0.99135833	ENSG00000186871	ERCC6L
30.06035449	-1.299213354	0.788224575	-1.648278163	0.099295604	0.921147998	ENSG00000179104	TMTC2
30.78829277	-0.922899976	0.621608672	-1.484696107	0.137624343	0.934144427	ENSG00000178726	THBD
31.44222443	-1.08734814	0.684639598	-1.588205158	0.112239955	0.923922314	ENSG00000163909	HEYL
30.62454915	0.308794026	0.606503558	0.50913803	0.610655477	0.99483168	ENSG00000178199	ZC3H12D
30.244555	-0.681342449	0.707588779	-0.962907368	0.335594012	0.977411637	ENSG00000185345	PRKN
30.97828793	-0.233669386	0.579845313	-0.402985729	0.686958723	0.998869317	ENSG00000225470	JPX
27.98132515	2.366435763	1.299431417	1.821131713	0.068586838	0.87420149	ENSG00000145824	CXCL14
31.01501445	-0.655745323	0.740935397	-0.885023614	0.376143895	0.982609538	ENSG00000205090	TMEM240
31.13181333	0.948239324	0.58582992	1.618625631	0.105527837	0.92169898	ENSG00000227671	AL390728.4
30.62519639	-0.136466174	0.735556051	-0.185527906	0.852814995	0.999467288	ENSG00000170681	CAVIN4
29.98537809	-0.232614271	0.570633965	-0.407641826	0.683536652	0.998869317	ENSG00000245571	FAM111A-DT
30.49313662	1.480397105	0.554888199	2.667919603	0.007632252	0.563130099	ENSG00000139631	CSAD
30.19172077	0.272321987	0.709020471	0.384081981	0.700917686	0.998869317	ENSG00000169744	LDB2
30.69631715	-0.663713578	0.54528606	-1.217184204	0.223534139	0.956884001	ENSG00000218418	AL591135.1
30.41398628	0.029356062	0.528533064	0.055542528	0.95570625	0.999527751	ENSG00000136895	GARNL3
31.68344168	-0.353746643	0.794780929	-0.44508698	0.656256898	0.997568624	ENSG00000128346	C22orf23
30.54223307	0.387396044	0.641136647	0.604233194	0.545688615	0.99483168	ENSG00000162924	REL
30.2740822	0.089892944	0.686320852	0.130978017	0.895792698	0.999467288	ENSG00000131019	ULBP3
30.09786355	0.054096566	0.612752374	0.088284547	0.929650521	0.999527751	ENSG00000197647	ZNF433
29.90592453	-0.415919945	0.77686876	-0.535379933	0.592387163	0.99483168	ENSG00000170909	OSCAR
29.57270009	-0.244739529	0.641300535	-0.38163001	0.702735821	0.998879389	ENSG00000158825	CDA
30.31432225	0.230188986	0.533079082	0.431810202	0.665879364	0.997568624	ENSG00000168152	THAP9
30.48723446	0.027159118	0.534968345	0.05076771	0.959510621	0.999527751	ENSG00000197258	EIF4BP6
29.46309357	0.027696978	0.602884701	0.045940754	0.963357472	0.999527751	ENSG00000279528	AC115618.3
29.60991855	-0.086091401	0.632083429	-0.136202592	0.891661126	0.999467288	ENSG00000130224	LRCH2
29.73369383	-0.036609394	0.524772127	-0.06976246	0.944382727	0.999527751	ENSG00000223508	RPL23AP53
31.58924363	-0.048203431	0.831272881	-0.057987494	0.95375859	0.999527751	ENSG00000187796	CARD9
29.74388058	1.560335196	0.527146442	2.959965336	0.003076737	0.403975514	ENSG00000269044	AC024075.2
29.39041055	-0.103176299	0.592098458	-0.174255307	0.861664822	0.999467288	ENSG00000105499	PLA2G4C
28.13760899	-0.688931165	1.172512028	-0.587568527	0.556821941	0.99483168	ENSG00000143320	CRABP2
29.59525854	0.952667734	0.659300152	1.44496817	0.148466826	0.936396974	ENSG00000197008	ZNF138
29.3153473	-1.481872489	0.632257059	-2.343781645	0.019089343	0.727643954	ENSG00000122367	LDB3
29.48307458	-1.015115246	0.56543392	-1.795285373	0.072608244	0.885710197	ENSG00000100445	SDR39U1
30.70169424	-0.847305431	0.58647188	-1.44475031	0.148528033	0.936396974	ENSG00000166262	FAM227B
29.50591088	-0.321412356	0.630982995	-0.509383548	0.610483406	0.99483168	ENSG00000141384	TAF4B
29.36653948	1.081086853	0.792778239	1.363668678	0.172671866	0.944422693	ENSG00000164045	CDC25A
29.72420346	-0.752245364	0.746094036	-1.008244708	0.313337	0.973427483	ENSG00000129295	LRRC6
29.53272753	-0.058048295	0.639102395	-0.090827848	0.927629382	0.999527751	ENSG00000258168	AC025569.1
30.73265672	0.205096339	0.800637901	0.256166162	0.797822536	0.999467288	ENSG00000013297	CLDN11
30.32253214	0.727862554	0.827618546	0.879466219	0.379148541	0.982778604	ENSG00000146555	SDK1
29.4153078	-0.079963865	0.57892131	-0.13812562	0.890141138	0.999467288	ENSG00000168000	BSCL2
29.82202191	0.446685808	0.941616087	0.474382091	0.635227462	0.995506752	ENSG00000137872	SEMA6D
29.92437959	0.665921771	0.559319205	1.190593431	0.233813232	0.959884875	ENSG00000197415	VEPH1
28.59971493	-0.748959524	0.764961052	-0.979081905	0.327539512	0.976026345	ENSG00000135549	PKIB
28.71686867	0.352823243	0.710879146	0.496319586	0.61966895	0.99483168	ENSG00000171729	TMEM51
30.04740251	-0.195116919	0.604108767	-0.322983094	0.746708045	0.999467288	ENSG00000152977	ZIC1
29.54873645	-0.158843351	0.537146253	-0.295717136	0.76744611	0.999467288	ENSG00000164093	PITX2
28.90719435	1.915861328	0.616172169	3.109295459	0.001875341	0.308164584	ENSG00000155511	GRIA1
28.84461956	1.994025647	1.051246189	1.89682081	0.057851592	0.852306368	ENSG00000204764	RANBP17
29.79179049	1.066551606	0.647105989	1.648186888	0.099314327	0.921147998	ENSG00000165194	PCDH19
29.30884818	-0.253381083	0.574120411	-0.441337876	0.65896841	0.997568624	ENSG00000271147	ARMCX5-GPRASP2
29.07486205	0.252147632	0.653820875	0.385652465	0.699754072	0.998869317	ENSG00000196466	ZNF799
28.87516394	-0.450883677	0.629742945	-0.715980513	0.474003382	0.99135833	ENSG00000083807	SLC27A5
28.83244793	-0.665118411	0.565966142	-1.175191166	0.239918244	0.962465655	ENSG00000247516	MIR4458HG
30.28085016	-0.071392796	0.842031005	-0.084786422	0.932431189	0.999527751	ENSG00000233381	AK4P3
29.04227513	0.589483738	0.538531955	1.094612368	0.273686495	0.962465655	ENSG00000274605	AL355338.1
29.59523324	0.22441783	0.58576556	0.383118854	0.70163164	0.998869317	ENSG00000132141	CCT6B
30.14722924	-0.75201025	1.03041571	-0.729812485	0.465504812	0.99135833	ENSG00000271447	MMP28
28.92931229	-0.348123563	0.627915334	-0.554411629	0.57929717	0.99483168	ENSG00000175711	B3GNTL1

29.42885118	-0.139839339	0.515306896	-0.271370984	0.786105717	0.999467288	ENSG00000036448	MYOM2
29.78747213	0.549138063	0.732393108	0.749785952	0.453383631	0.99135833	ENSG00000274307	AC023449.2
28.63111972	0.239226951	0.57163254	0.418497783	0.675583207	0.998869317	ENSG00000186496	ZNF396
29.1638843	-0.68762902	0.691133142	-0.994929889	0.319770365	0.974964731	ENSG00000280099	AL603750.1
29.13676617	-0.718617734	0.565252931	-1.271320669	0.203614592	0.954310328	ENSG00000162757	C1orf74
28.39521297	-0.224727076	0.65101998	-0.345192287	0.729949817	0.999467288	ENSG00000213160	KLHL23
29.55665767	-0.958205692	0.64635623	-1.482473049	0.13821447	0.934144427	ENSG00000134072	CAMK1
28.98847516	0.694170791	0.6524943	1.063872575	0.287386428	0.965284229	ENSG00000114841	DNAH1
28.45539145	-0.701347585	0.728070392	-0.963296397	0.335398801	0.977237217	ENSG00000254531	FLJ20021
29.23743039	1.316333543	0.580900315	2.266023117	0.023449968	0.744948012	ENSG00000257151	PWAR6
29.05229957	0.132180841	0.613026846	0.215619988	0.829283974	0.999467288	ENSG00000158315	RHBDL2
29.17479716	0.661345948	0.673518905	0.981926334	0.326136141	0.976026345	ENSG00000144485	HES6
29.83625096	-0.829931757	0.642874372	-1.290970357	0.196713957	0.954310328	ENSG00000185250	PPIL6
29.54849209	-0.269491066	0.531875144	-0.506681069	0.612378616	0.99483168	ENSG00000274349	ZNF658
29.54993207	-0.503312359	0.561049947	-0.897090109	0.369670836	0.981455968	ENSG00000151575	TEX9
28.89990479	-1.166398193	1.080993405	-1.079005837	0.280585126	0.963267117	ENSG00000183114	FAM43B
29.27138279	-0.382531745	0.572146287	-0.668590802	0.503756541	0.993513219	ENSG00000162650	ATXN7L2
29.10093954	-0.447468973	0.598749391	-0.747339336	0.454858754	0.99135833	ENSG00000109339	MAPK10
29.46165769	0.506466503	0.625805947	0.809302796	0.418340998	0.990226751	ENSG00000228439	TSTD3
29.44268913	1.380371375	0.652197175	2.11649395	0.034302819	0.782919999	ENSG00000095203	EPB41L4B
29.13738513	0.823488006	0.540083526	1.524741945	0.127323475	0.928627271	ENSG00000105464	GRIN2D
28.35682385	-1.778205455	0.623157364	-2.853541589	0.004323487	0.462224997	ENSG00000114115	RBP1
29.06965436	-0.218583297	0.543194532	-0.402403346	0.687387208	0.998869317	ENSG00000113205	PCDHB3
28.45996613	-0.341470266	0.580438604	-0.588296959	0.556332986	0.99483168	ENSG00000204366	ZBTB12
28.54974694	0.085869816	0.662638207	0.129587783	0.896892569	0.999467288	ENSG00000110876	SELPLG
28.25020131	1.622612276	0.64715249	2.507310565	0.012165377	0.648356243	ENSG00000259250	AC018904.1
28.89855539	1.342040764	0.641663477	2.091502498	0.036483043	0.789688071	ENSG00000103742	IGDCC4
29.10333422	1.276313354	0.723358513	1.764427087	0.077660101	0.897052166	ENSG00000142494	SLC47A1
28.49510851	0.238556348	0.628355851	0.379651669	0.704204001	0.99900742	ENSG00000152580	IGSF10
27.37926095	1.571514842	0.98521086	1.595105074	0.110688741	0.923922314	ENSG00000134533	RERG
28.66993414	0.148026353	0.550039204	0.26911964	0.787837617	0.999467288	ENSG00000258738	AL121603.2
28.87081136	0.091673582	0.571802019	0.160323991	0.872625861	0.999467288	ENSG00000228672	PROB1
29.35573353	0.741256002	0.763951267	0.970292261	0.331900834	0.976026345	ENSG00000262209	PCDHGB3
29.147028	-0.012420851	0.54925939	-0.022613816	0.981958323	0.999527751	ENSG00000261934	PCDHGA9
28.33873229	0.337740532	0.719313677	0.469531642	0.638689673	0.996553586	ENSG00000233223	AC016876.1
29.28760348	-0.753682858	0.658886015	-1.14387442	0.252675721	0.962465655	ENSG00000205309	NT5M
29.26456778	0.741309651	0.600426651	1.234638151	0.216965204	0.955665886	ENSG00000267002	AC060780.1
27.46455068	0.567901466	0.688306154	0.825071028	0.409331267	0.990226751	ENSG00000121769	FABP3
29.45408199	-1.175141557	0.930390939	-1.263062126	0.206566854	0.954310328	ENSG00000171227	TMEM37
28.22174197	0.009781971	0.649264837	0.015066227	0.987979345	0.999527751	ENSG00000179348	GATA2
28.57638408	-0.267383191	0.531729953	-0.502855237	0.615066055	0.99483168	ENSG00000237350	CDC42P6
28.20906388	-2.683319374	0.840417175	-3.192842143	0.001408799	0.264250513	ENSG00000205572	SERF1B
28.80154425	-0.423768792	0.537722199	-0.788081268	0.430649171	0.991301167	ENSG00000135314	KHDC1
28.89485269	0.321402619	0.618929862	0.519287627	0.60356018	0.99483168	ENSG00000105997	HOXA3
27.44220187	0.922980622	0.778696281	1.185289624	0.235902954	0.962465655	ENSG00000120658	ENOX1
28.08317665	-0.154373336	0.752443209	-0.205162774	0.837444953	0.999467288	ENSG00000090447	TFAP4
28.76181531	-0.097173978	0.555025235	-0.175080288	0.861016547	0.999467288	ENSG00000232859	LYRM9
29.08640993	-0.296927127	0.624468956	-0.475487412	0.634439604	0.995506752	ENSG00000187860	CCDC157
28.23179964	0.948717131	0.681112421	1.392893599	0.163651968	0.943223078	ENSG00000205583	STAG3L1
28.80548558	1.875323787	0.764722082	2.452294541	0.014194842	0.68371955	ENSG00000243302	AC018638.4
28.5058043	-0.225679201	0.544238092	-0.414669984	0.678383508	0.998869317	ENSG00000185670	ZBTB3
28.58259039	-1.164984268	0.554605466	-2.100563985	0.035679258	0.785058983	ENSG00000176723	ZNF843
28.3209716	1.534106882	0.667707014	2.297574907	0.021585995	0.73997555	ENSG00000120156	TEK
28.22105425	-0.550377887	0.530632158	-1.03721171	0.299637202	0.972186492	ENSG00000256073	URB1-AS1
28.43147551	-0.118450402	0.565540438	-0.209446388	0.834099783	0.999467288	ENSG00000160298	C21orf58
30.46920922	1.339712289	1.264089094	1.05982426	0.289224558	0.966513256	ENSG00000156453	PCDH1
28.26199115	0.086796537	0.624758524	0.138928136	0.889506938	0.999467288	ENSG00000115255	REEP6
27.04449789	-0.087280793	0.953935671	-0.091495471	0.927098905	0.999527751	ENSG00000142623	PADI1
28.03492882	-0.300838689	0.621709446	-0.483889525	0.628464275	0.99483168	ENSG00000136237	RAPGEF5
28.49177717	1.341365175	0.688355692	1.948651242	0.051337088	0.842569961	ENSG00000183793	NPIPA5
28.277228	0.474612175	0.668297256	0.710181242	0.477591752	0.99135833	ENSG00000185495	AC138393.1
28.07299356	0.329838161	0.614652458	0.536625465	0.59152635	0.99483168	ENSG00000213430	HSPD1P1
28.10751847	0.073114932	0.662420759	0.110375363	0.912111691	0.999527751	ENSG00000229852	AC019205.1
27.915694	0.99800627	0.724269771	1.37794826	0.168219263	0.943223078	ENSG00000179869	ABCA13

27.95267129	-0.804374079	0.769795545	-1.044919114	0.296060355	0.970706863	ENSG00000214309	MBLAC1
29.00312425	-0.073909918	0.930265807	-0.079450322	0.936674444	0.999527751	ENSG00000236404	VLDLR-AS1
28.31478845	-0.729786892	0.637863756	-1.144110925	0.252577638	0.962465655	ENSG00000254473	AL354920.1
28.26392988	0.473400248	0.715147822	0.661961393	0.507995964	0.993513219	ENSG00000198056	PRIM1
28.55689768	0.022091824	0.653060738	0.033828131	0.973014203	0.999527751	ENSG00000178229	ZNF543
28.00883077	0.049210788	0.614192078	0.080122798	0.936139591	0.999527751	ENSG00000251495	PPIAP11
28.91287718	-1.444071094	0.821241999	-1.758398982	0.078679646	0.897052166	ENSG00000171889	MIR31HG
28.8016924	-0.67351221	0.6140639	-1.096811278	0.272723899	0.962465655	ENSG00000130222	GADD45G
27.64286751	0.120973903	0.645378163	0.187446539	0.851310536	0.999467288	ENSG00000188266	HYKK
28.25428408	-0.565954253	0.537776159	-1.052397439	0.292617243	0.970706863	ENSG00000160345	C9orf116
26.86007604	-0.483720236	0.889764253	-0.543649887	0.586682423	0.99483168	ENSG00000167767	KRT80
27.90492363	-0.728314059	0.659873439	-1.103717799	0.269715572	0.962465655	ENSG00000213654	GPSM3
27.82685768	-0.926020553	0.559841712	-1.654075666	0.09811214	0.921147998	ENSG00000149489	ROM1
28.45660686	-0.750670228	0.58334774	-1.286831467	0.198153037	0.954310328	ENSG00000225422	RBMS1P1
28.36477619	-0.293027226	0.574177747	-0.510342359	0.609811631	0.99483168	ENSG00000264885	AC026271.3
27.54149702	-0.596274801	0.661234546	-0.901759904	0.367184423	0.981269214	ENSG00000233593	AL590094.1
28.2463689	0.531214326	0.621670163	0.854495452	0.392830529	0.988378925	ENSG00000276334	AL133243.2
27.77527543	0.11874558	0.769975582	0.154219929	0.877436329	0.999467288	ENSG00000163536	SERPINI1
27.885773	2.914856593	0.748164602	3.896009763	9.78E-05	0.067295389	ENSG00000270069	MIR222HG
28.52485209	-0.79376417	0.64026691	-1.239739486	0.215071767	0.954310328	ENSG00000104381	GDAP1
27.13779697	0.741093568	0.655908894	1.129872723	0.258529859	0.962465655	ENSG00000177570	SAMD12
26.42574642	3.081656012	0.95150403	3.238720925	0.00120067	0.244243321	ENSG00000023445	BIRC3
27.64890939	-0.373992435	0.625839554	-0.597585168	0.550116763	0.99483168	ENSG00000251602	AL928654.1
28.49522235	-0.343232178	0.579842215	-0.59194065	0.553890332	0.99483168	ENSG00000187624	C17orf97
28.06625929	0.049744625	0.865413191	0.05748078	0.954162216	0.999527751	ENSG00000177602	HASPIN
27.87886896	-0.411801571	0.646073402	-0.637391309	0.523869988	0.99483168	ENSG00000118242	MREG
28.18047528	0.599513031	0.661486172	0.906312265	0.364770595	0.981076669	ENSG00000205571	SMN2
27.21427451	-0.454436003	0.666690362	-0.681629778	0.495473081	0.993513219	ENSG00000215483	LINC00598
27.84833838	0.384611182	0.556060575	0.691671374	0.489143728	0.993513219	ENSG00000181619	GPR135
28.00392367	0.624712001	0.563320431	1.108981615	0.267438113	0.962465655	ENSG00000080603	SRCAP
27.59328339	-0.73081414	0.663433037	-1.101564286	0.270651142	0.962465655	ENSG00000172264	MACROD2
28.78131833	-0.66870407	0.669617995	-0.998635156	0.317971463	0.974768742	ENSG00000130702	LAMA5
27.98825921	-0.749790933	0.540255768	-1.387844382	0.165184437	0.943223078	ENSG00000224687	RASAL2-AS1
28.30130814	-0.205369435	0.634328921	-0.323758586	0.746120812	0.999467288	ENSG00000170006	TMEM154
27.76634382	-0.164360555	0.63320438	-0.259569516	0.795195853	0.999467288	ENSG00000184489	PTP4A3
27.25659993	-0.254354866	0.683726467	-0.372012607	0.709883463	0.99900742	ENSG00000211448	DIO2
27.00528868	-1.544176516	0.859357916	-1.796895667	0.072352185	0.885710197	ENSG00000142185	TRPM2
27.62559785	0.337506843	0.642206844	0.52554227	0.599206276	0.99483168	ENSG00000281490	CICP14
27.87507741	-0.078635294	0.543580467	-0.144661735	0.884977952	0.999467288	ENSG00000230311	TOMM20P4
26.97229301	0.165639859	0.632829022	0.261745042	0.793518012	0.999467288	ENSG00000253661	ZFHX4-AS1
27.83990734	0.178800441	0.579209633	0.308697285	0.757551809	0.999467288	ENSG00000226525	RPS7P10
26.73118008	2.820614396	1.009156649	2.795021366	0.005189629	0.483572934	ENSG00000100678	SLC8A3
28.1476676	0.121363269	0.639179026	0.189873673	0.849408123	0.999467288	ENSG00000267041	ZNF850
27.77425626	-0.066462704	0.6557361	-0.101355872	0.919267965	0.999527751	ENSG00000250132	AC004803.1
27.86889221	0.25141775	0.619211751	0.406028712	0.684721504	0.998869317	ENSG00000280202	AC005831.1
27.08711223	-0.516031195	0.675029915	-0.76445678	0.444595083	0.99135833	ENSG00000011600	TYROBP
27.01564946	0.709604231	0.680777211	1.04234428	0.29725209	0.971974628	ENSG00000184068	SREBF2-AS1
28.01786357	-0.114477076	0.68548974	-0.167000422	0.867369717	0.999467288	ENSG00000196418	ZNF124
28.03001208	-1.303224012	0.679064137	-1.919147163	0.054965712	0.847598083	ENSG00000229419	RALGAPA1P1
27.30182263	0.268808292	0.965449106	0.278428236	0.780683647	0.999467288	ENSG00000143590	EFNA3
27.03751458	-1.347448595	0.627900798	-2.145957767	0.031876348	0.775963115	ENSG00000102271	KLHL4
27.08426142	0.321924304	0.598338619	0.538030295	0.590556134	0.99483168	ENSG00000042832	TG
27.52867571	0.254961713	0.803215582	0.317426253	0.750920189	0.999467288	ENSG00000197142	ACSL5
27.85294968	0.377949743	0.680016233	0.555795178	0.578350882	0.99483168	ENSG00000255150	EID3
27.11362842	-0.591911124	0.652760667	-0.906781235	0.364522495	0.980957406	ENSG00000170296	GABARAP
26.90016246	0.737676044	0.624377602	1.181458211	0.237420745	0.962465655	ENSG00000213963	AC019080.1
27.19153144	0.150729829	0.555562037	0.271310526	0.786152211	0.999467288	ENSG00000234327	AC012146.1
27.07061823	0.648927882	0.557722147	1.163532567	0.244613493	0.962465655	ENSG00000266088	AC004585.1
27.70045475	-0.638918886	0.600101373	-1.064684927	0.287018533	0.965284229	ENSG00000178502	KLHL11
27.0151388	-0.471727917	0.648445576	-0.727474956	0.466935051	0.99135833	ENSG00000247796	AC008966.1
27.0683734	-0.190507768	0.622405876	-0.306082856	0.759541561	0.999467288	ENSG00000100490	CDKL1
27.30816158	-0.460568726	0.701343776	-0.65669468	0.511377256	0.993513219	ENSG00000196357	ZNF565
28.67038675	-0.678380608	0.908357655	-0.746821039	0.455171594	0.99135833	ENSG00000174343	CHRNA9
27.43230145	1.583358342	0.696197927	2.274293389	0.022948352	0.744948012	ENSG00000260404	AC110079.1

27.18482826	0.63428263	0.674986225	0.939697147	0.347372929	0.977691382	ENSG00000130653	PNPLA7
26.775917	-0.549760883	0.564656652	-0.973619775	0.330245364	0.976026345	ENSG00000232004	CAP1P2
26.79613446	-0.166128761	0.565193318	-0.293932634	0.768809381	0.999467288	ENSG00000260267	AC026471.1
26.37954448	0.590496221	0.654649133	0.902004128	0.367054675	0.981269214	ENSG00000176678	FOXL1
27.47256577	-0.444735974	0.601087198	-0.739885954	0.459369198	0.99135833	ENSG00000204524	ZNF805
25.75540145	0.669973926	0.934738456	0.716750148	0.473528277	0.99135833	ENSG00000188452	CERKL
27.25298969	0.697515641	0.552775754	1.26184196	0.20700566	0.954310328	ENSG00000131127	ZNF141
26.86842581	-0.792664297	0.665410537	-1.191240975	0.233558998	0.959684953	ENSG00000112183	RBM24
27.55127442	-1.83220842	0.758131246	-2.416743051	0.015660067	0.696728876	ENSG00000103449	SALL1
27.931177	-0.333933596	1.048608727	-0.318453955	0.750140617	0.999467288	ENSG00000172137	CALB2
27.13642735	-0.347871831	0.865816858	-0.401784544	0.687842598	0.998869317	ENSG00000149451	ADAM33
27.15461606	-0.06903908	0.560260606	-0.123226726	0.901927563	0.999527751	ENSG00000219665	ZNF433-AS1
26.60287526	-0.079146816	0.581520542	-0.136103216	0.891739685	0.999467288	ENSG00000123154	WDR83
27.95024205	-0.665423221	0.92687285	-0.717922875	0.472804843	0.99135833	ENSG00000226054	MEMO1P1
27.28843735	-0.353689968	0.630900492	-0.560611336	0.575062522	0.99483168	ENSG00000116151	MORN1
26.77763444	-0.050966702	0.617633077	-0.082519386	0.934233703	0.999527751	ENSG00000070759	TESK2
27.51273895	-0.035718406	0.606040219	-0.058937353	0.953002007	0.999527751	ENSG00000221994	ZNF630
26.21774079	0.436324065	0.70900306	0.615405052	0.538287256	0.99483168	ENSG00000246100	LINC00900
27.52487757	0.455021185	0.75621007	0.601712677	0.54736541	0.99483168	ENSG00000168398	BDKRB2
26.30020231	-0.948446714	0.617370348	-1.536268655	0.124472504	0.927794614	ENSG00000093072	ADA2
28.29967639	1.23054948	1.318127263	0.933558932	0.350531458	0.977691382	ENSG00000081277	PKP1
26.65994407	-0.315324431	0.603036157	-0.522894734	0.60104751	0.99483168	ENSG00000285906	AC083855.2
26.73409385	0.004056091	0.591670039	0.006855327	0.994530283	0.999527751	ENSG00000219200	RNASEK
26.37239228	0.946235181	0.615775208	1.536656833	0.124377367	0.927794614	ENSG00000162426	SLC45A1
26.50239207	0.024378585	0.673302994	0.036207451	0.971116945	0.999527751	ENSG00000248587	GDNF-AS1
26.47033469	1.298025779	0.883846955	1.46860921	0.141938815	0.935644597	ENSG00000106785	TRIM14
26.67298248	1.662804683	1.033771548	1.608483699	0.107729284	0.923922314	ENSG00000197299	BLM
26.78462478	0.037174573	0.527242985	0.070507477	0.94378975	0.999527751	ENSG00000278535	DHRS11
27.3107846	0.320076153	0.83252385	0.384464846	0.700633947	0.998869317	ENSG00000101115	SALL4
26.97231425	-0.504975913	0.577387694	-0.874587246	0.38179853	0.985369679	ENSG00000236540	AC006547.1
26.42764069	-0.330470614	0.89310805	-0.370023106	0.711365274	0.99900742	ENSG00000134516	DOCK2
26.8155742	0.888757855	0.784720019	1.132579562	0.257390855	0.962465655	ENSG00000126950	TMEM35A
27.10822674	0.859130866	0.604290531	1.421718232	0.155108065	0.939432741	ENSG00000260565	ERVK13-1
27.87101992	-2.174727293	0.961844776	-2.260996105	0.023759498	0.744948012	ENSG00000179772	FOXS1
26.656407	0.067053044	0.695247158	0.096444902	0.923167232	0.999527751	ENSG00000267510	AC011451.1
26.86808647	0.532658829	0.644145651	0.826922961	0.408280728	0.990226751	ENSG00000124549	BTN2A3P
26.35376204	1.240840722	0.584245549	2.123834276	0.033684004	0.775963115	ENSG00000261438	AL157394.1
26.3144233	0.419895869	0.672372813	0.624498583	0.532300201	0.99483168	ENSG00000197497	ZNF665
26.75171913	-0.11510286	0.734410484	-0.156728236	0.875459033	0.999467288	ENSG00000157554	ERG
26.15078068	0.09541669	0.593371632	0.160804266	0.872247565	0.999467288	ENSG00000113231	PDE8B
26.73161144	0.306537456	0.72632734	0.422037611	0.672997565	0.998473603	ENSG00000280077	AL353763.2
26.89929855	0.279010522	0.795873888	0.350571272	0.725910012	0.999467288	ENSG00000095539	SEMA4G
26.2151262	-0.179524452	0.718739854	-0.249776677	0.802760057	0.999467288	ENSG00000248593	DSTNP2
26.10829148	-1.080754562	0.646137457	-1.672638771	0.094398429	0.921147998	ENSG00000214029	ZNF891
25.93595432	-1.120025725	0.668418711	-1.675634909	0.093809727	0.921147998	ENSG00000131979	GCH1
26.2882132	1.32633091	1.160721815	1.142677679	0.253172441	0.962465655	ENSG00000137804	NUSAP1
26.17785531	-0.372199272	0.693346177	-0.536815929	0.591394768	0.99483168	ENSG00000102981	PARD6A
26.39578897	0.407897437	0.618694337	0.65928749	0.509711174	0.993513219	ENSG00000215808	LINC01139
26.4976114	-0.98603536	0.668656929	-1.474650629	0.140306496	0.935644597	ENSG00000189283	FHIT
26.42320555	-0.357569499	0.576915498	-0.619795273	0.535392581	0.99483168	ENSG00000163959	SLC51A
26.2979143	1.731204129	1.116597712	1.550427796	0.121038871	0.923922314	ENSG00000109674	NEIL3
25.98573697	-0.08370122	0.591991371	-0.141389257	0.88756244	0.999467288	ENSG00000123570	RAB9B
25.96032262	0.476769956	0.640030521	0.744917532	0.456321576	0.99135833	ENSG00000262246	CORO7
26.16386587	-0.690406624	0.973125764	-0.709473174	0.478030893	0.99135833	ENSG00000230479	AP000695.1
26.71549065	0.118599088	0.585057348	0.202713612	0.839358883	0.999467288	ENSG00000277462	ZNF670
25.79541204	0.194030288	0.778306147	0.249298157	0.803130157	0.999467288	ENSG00000175287	PHYHD1
26.0457846	0.283841284	0.614842543	0.461648738	0.644333238	0.997332006	ENSG00000188611	ASAH2
26.22977705	-0.466967567	0.764490251	-0.610822134	0.541317338	0.99483168	ENSG00000256806	C17orf100
26.55755143	1.683822721	0.970321786	1.735324039	0.082683426	0.899922174	ENSG00000265415	AC099850.3
26.89026356	0.108299556	0.626922061	0.172748038	0.862849482	0.999467288	ENSG00000204920	ZNF155
25.54297915	-0.01487627	0.662475993	-0.022455561	0.98208456	0.999527751	ENSG00000235105	AL356968.2
26.36201364	-0.164053272	0.547642381	-0.299562777	0.764510681	0.999467288	ENSG00000197872	FAM49A
26.72159139	-0.134316195	0.719283448	-0.186736113	0.85186754	0.999467288	ENSG00000197140	ADAM32
25.74560124	0.175342088	0.658005159	0.266475249	0.789873219	0.999467288	ENSG00000167992	VWCE

25.58124821	0.141422809	0.722119186	0.195844137	0.844732162	0.999467288	ENSG00000129465	RIPK3
25.86518735	-0.694585447	0.643716847	-1.079023255	0.280577361	0.963267117	ENSG00000126249	PDCD2L
26.47366957	0.916951751	0.766084234	1.196933327	0.231332543	0.959575179	ENSG00000230615	AL139220.2
27.26083463	0.170925245	0.842689948	0.202832899	0.839265643	0.999467288	ENSG00000239467	AC007405.3
25.93760576	-0.022642175	0.991988309	-0.022825042	0.981789832	0.999527751	ENSG00000111816	FRK
25.60494492	0.638323847	0.778299777	0.82015165	0.412129661	0.990226751	ENSG00000136231	IGF2BP3
26.40828666	0.174054582	0.753951406	0.2308565	0.817426286	0.999467288	ENSG00000119915	ELOVL3
25.4077805	0.302442996	0.674231589	0.448574349	0.653738741	0.997527562	ENSG00000277954	AC092376.2
25.99380852	-0.280677293	0.656546655	-0.42750548	0.669011194	0.997568624	ENSG00000187650	VMAC
25.74929894	-0.39279422	0.671374367	-0.5850599	0.558507441	0.99483168	ENSG00000284693	AL928921.2
25.15299085	0.747980665	0.979106016	0.763942466	0.444901529	0.99135833	ENSG00000134259	NGF
26.08489963	-0.317145292	0.737825647	-0.429837718	0.667313694	0.997568624	ENSG00000173175	ADCY5
25.63569991	0.475351252	0.643690481	0.738477988	0.460224038	0.99135833	ENSG00000171757	LRRC34
26.32943754	1.653600871	0.743522365	2.22400959	0.026147799	0.762934669	ENSG00000138658	ZGRF1
26.11470028	0.094947672	0.66284808	0.14324198	0.886099076	0.999467288	ENSG00000274070	CASTOR2
26.16582321	1.380707963	0.970864151	1.422143315	0.15498465	0.939432741	ENSG00000182261	NLRP10
25.21856294	-0.55589128	0.620082929	-0.896478928	0.369997029	0.981455968	ENSG00000228436	AL139260.1
25.32981733	-1.389956116	0.713263099	-1.948728481	0.051327859	0.842569961	ENSG00000231351	AC111200.2
25.45822342	0.156001875	0.595860138	0.26180955	0.793468276	0.999467288	ENSG00000261338	AC021016.2
26.15648268	-0.424607037	0.616758891	-0.688448992	0.491170081	0.993513219	ENSG00000248866	USP46-AS1
27.07305226	0.853105657	1.099189872	0.776122196	0.43767684	0.991301167	ENSG00000243753	HLA-L
24.83229955	0.573249632	0.829051282	0.691452561	0.489281183	0.993513219	ENSG00000197457	STMN3
26.05421737	-0.565999213	0.620087773	-0.912772736	0.36136208	0.979465649	ENSG00000159917	ZNF235
24.7522782	1.485379948	0.88767005	1.673346924	0.09425902	0.921147998	ENSG00000185760	KCNQ5
26.6717297	-0.713091112	0.770133797	-0.925931462	0.3544816	0.977691382	ENSG00000104321	TRPA1
25.41588047	-0.233629982	0.63330849	-0.368903916	0.712199344	0.99900742	ENSG00000180574	EIF2S3B
25.52174192	0.104581546	0.563753104	0.185509482	0.852829444	0.999467288	ENSG00000168237	GLYCTK
25.97610091	0.620793424	0.597767888	1.038519192	0.299028405	0.972186492	ENSG00000004777	ARHGAP33
25.16868931	-0.755410826	0.67808732	-1.114031782	0.265265552	0.962465655	ENSG00000114646	CSPG5
25.79157018	-2.179995298	0.767543972	-2.840222031	0.004508214	0.469826318	ENSG00000244564	AC096888.1
24.65703065	0.241402456	0.892818151	0.270382558	0.786865957	0.999467288	ENSG00000229512	AC068580.1
25.48894238	-1.018579808	0.830530691	-1.22642043	0.220040502	0.956884001	ENSG00000165879	FRAT1
25.61960247	0.328364558	0.653323535	0.502606351	0.615241063	0.99483168	ENSG00000130590	SAMD10
26.11829914	-1.066545618	1.226408048	-0.869649885	0.384491767	0.985655313	ENSG00000159261	CLDN14
25.72059296	0.116666731	0.827090661	0.141056762	0.887825101	0.999467288	ENSG00000104722	NEFM
25.21059334	0.584476172	0.600090185	0.973980555	0.330066194	0.976026345	ENSG00000255366	AC120036.4
25.25626274	0.058147578	0.596321667	0.097510423	0.922321057	0.999527751	ENSG00000119686	FLVCR2
25.77055027	0.129477351	0.602878568	0.214765224	0.829950367	0.999467288	ENSG00000106526	ACTR3C
24.89735196	-0.211931322	0.906593343	-0.233766687	0.815166113	0.999467288	ENSG00000260454	AL355607.2
24.37001035	-0.53469347	0.817945427	-0.653703111	0.513303099	0.993513219	ENSG00000185742	C11orf87
25.61027183	0.645289735	0.791837996	0.81492646	0.415114406	0.990226751	ENSG00000261327	AC134312.5
25.46959376	0.064127961	0.605632253	0.105885974	0.915672823	0.999527751	ENSG00000237975	FLG-AS1
25.06482008	0.565013791	0.860273354	0.656784019	0.511319801	0.993513219	ENSG00000143195	ILDR2
24.67685901	1.111187195	0.73352642	1.514856406	0.129808772	0.929373153	ENSG00000163701	IL17RE
25.54991961	-0.641313181	0.709120667	-0.90437807	0.365794962	0.981269214	ENSG00000155465	SLC7A7
25.24827353	1.607762764	0.912977112	1.761011031	0.078236536	0.897052166	ENSG00000187951	AC091057.1
25.83290293	1.121459112	0.669848687	1.674197673	0.094091757	0.921147998	ENSG00000280670	CCDC163
25.66952523	0.087058008	0.609967899	0.142725558	0.886506931	0.999467288	ENSG00000177839	PCDHB9
25.63876334	1.557036012	1.06960069	1.455717097	0.145470841	0.935644597	ENSG00000157782	CABP1
25.51312252	0.44357624	0.724956327	0.611866154	0.540626317	0.99483168	ENSG00000180998	GPR137C
25.15186259	0.456905118	0.79360421	0.575734242	0.564794827	0.99483168	ENSG00000233818	AP000695.2
24.85824821	-1.005972601	0.787335278	-1.277692781	0.201357772	0.954310328	ENSG00000121966	CXCR4
24.3117493	-0.344586859	0.647811539	-0.531924547	0.594778255	0.99483168	ENSG00000170917	NUDT6
24.55103993	0.28656339	1.106629728	0.258951465	0.795672689	0.999467288	ENSG00000164849	GPR146
24.96687172	-0.361213071	0.668913645	-0.539999556	0.589197338	0.99483168	ENSG00000134297	PLEKHA8P1
25.11721996	0.480459128	0.683390014	0.703052602	0.482022969	0.991525194	ENSG00000270015	AC087481.3
25.02650603	1.157114441	0.716847657	1.61417064	0.106490418	0.922165836	ENSG00000270580	PKD1P6-NPIPP1
25.25296431	-0.549823804	0.73521743	-0.74783837	0.454557655	0.99135833	ENSG00000274487	AC244154.1
24.78800896	0.198952752	0.692694224	0.287215838	0.77394706	0.999467288	ENSG00000134253	TRIM45
25.55787829	-0.225096818	0.648988602	-0.346842483	0.728709659	0.999467288	ENSG00000249348	UGDH-AS1
24.92694526	0.090195822	0.728578666	0.123796957	0.901476041	0.999527751	ENSG00000198939	ZFP2
25.07882462	0.312954953	0.561656081	0.557200329	0.577390565	0.99483168	ENSG00000204623	ZNRD1ASP
25.44314012	-0.089057631	0.682334924	-0.130518939	0.896155871	0.999467288	ENSG00000223559	AC073136.1
24.65203499	-0.000318574	0.585277479	-0.000544313	0.999565701	0.9998586	ENSG00000280789	PAGR1

24.54416505	-0.01378617	0.690712682	-0.019959342	0.984075807	0.999527751	ENSG00000267575	AC006504.5
25.44528551	0.118780463	0.723939539	0.164075115	0.869672012	0.999467288	ENSG00000171174	RBKS
23.26067255	1.051346085	1.220395062	0.861480121	0.388973665	0.986937571	ENSG00000168497	CAVIN2
24.01920989	-0.289046705	0.682693204	-0.423391801	0.672009426	0.997568624	ENSG00000185614	INKA1
24.10064866	0.76021636	0.76944813	0.988002089	0.323151625	0.975337095	ENSG00000113119	TMCO6
24.94861449	-0.243341528	0.742386025	-0.327783012	0.743075733	0.999467288	ENSG00000273230	AC102953.2
24.04368097	-0.515662352	1.059361802	-0.486766987	0.626423468	0.99483168	ENSG00000111057	KRT18
24.935057	-1.129227589	0.69235984	-1.630983663	0.102893767	0.92169898	ENSG00000171984	SHLD1
25.85039857	0.409757898	0.806431875	0.508112229	0.611374639	0.99483168	ENSG00000183054	RGPD6
24.25957656	1.211668959	0.905739872	1.337767053	0.180972385	0.949094286	ENSG00000111913	RIPOR2
24.47891297	0.798412985	0.607375206	1.314530091	0.188667879	0.949094286	ENSG00000165046	LETM2
24.5964083	-0.129918353	0.580741608	-0.223711115	0.822982097	0.999467288	ENSG00000204839	MROH6
24.31496194	0.699444184	0.773215327	0.904591722	0.365681723	0.981269214	ENSG00000185862	EVI2B
24.83651314	-0.135080751	0.56319168	-0.239848626	0.810447609	0.999467288	ENSG00000236814	AC046176.1
24.70035322	-0.869372294	0.581405513	-1.495294203	0.134837677	0.933741583	ENSG00000254332	AF201337.1
23.17256168	-1.128604563	0.885382748	-1.274708102	0.202412578	0.954310328	ENSG00000154330	PGM5
24.51909702	-0.035589502	0.705477166	-0.050447419	0.95976585	0.999527751	ENSG00000077943	ITGA8
24.89345112	0.503560633	0.562166547	0.895749909	0.370386348	0.981455968	ENSG00000272631	AC067750.1
25.19673002	-0.741948341	0.727250545	-1.020210086	0.307628835	0.973427483	ENSG00000116701	NCF2
24.51823567	-1.263492482	0.636441768	-1.985244442	0.047117282	0.827156057	ENSG00000244490	RWDD4P1
24.32607063	0.4845936	0.637667803	0.75994679	0.447286392	0.99135833	ENSG00000188707	ZBED6CL
25.60912636	0.869504352	0.855412144	1.016474174	0.30940364	0.973427483	ENSG00000210194	MT-TE
24.07458293	-0.0767208	0.672586526	-0.114068298	0.909183652	0.999527751	ENSG00000137752	CASP1
24.28705752	-0.168355164	0.706489098	-0.238298319	0.811649725	0.999467288	ENSG00000267321	LINC02001
23.91015125	1.371573622	0.912024934	1.50387733	0.132612955	0.933741583	ENSG00000178078	STAP2
24.22073495	-0.879779568	0.606525492	-1.450523647	0.14691255	0.936396974	ENSG00000173013	CCDC96
24.53046607	0.065020654	0.690955979	0.094102455	0.92502777	0.999527751	ENSG00000109771	LRP2BP
24.20543383	-0.105972322	0.704302185	-0.150464282	0.880398329	0.999467288	ENSG00000279204	AC134043.2
25.11114941	0.921880191	0.760103003	1.212835875	0.225192576	0.958865115	ENSG00000136122	BORA
24.69279661	-2.919772006	1.286772339	-2.269066498	0.023264282	0.744948012	ENSG00000205336	ADGRG1
24.7902429	1.157005012	0.740595132	1.562263863	0.118225841	0.923922314	ENSG00000196155	PLEKHG4
24.6895188	-0.454383625	0.629233418	-0.722122526	0.47021915	0.99135833	ENSG00000249459	ZNF286B
23.956489	-0.89112275	0.686696962	-1.297694323	0.194392395	0.951409476	ENSG00000226608	FTLP3
25.26172615	-0.369297824	1.197790739	-0.308315811	0.757842036	0.999467288	ENSG00000235513	AL035681.1
24.82751161	-0.113585049	0.661403317	-0.171733413	0.863647116	0.999467288	ENSG00000215817	ZC3H11B
24.42351317	0.081149233	0.629371773	0.12893688	0.897407594	0.999467288	ENSG00000056736	IL17RB
24.01086744	0.221460935	0.636455776	0.34795966	0.727870479	0.999467288	ENSG00000217094	PPIAP31
24.86407907	-1.114431765	0.658070408	-1.693484089	0.090363361	0.912150398	ENSG00000103599	IQCH
24.63121703	0.724534646	0.627411272	1.154800174	0.248172266	0.962465655	ENSG00000279059	AC007485.2
23.70041805	-0.912905138	0.832478572	-1.096610974	0.272811488	0.962465655	ENSG00000206535	LNP1
23.89551825	0.680933621	1.265365027	0.538132164	0.590485808	0.99483168	ENSG00000158402	CDC25C
23.98212146	-0.252891129	0.652233497	-0.387730974	0.698215132	0.998869317	ENSG00000272142	LYRM4-AS1
24.31220215	-0.052998965	0.584205139	-0.090719786	0.927715248	0.999527751	ENSG00000156500	FAM122C
23.13870975	-1.452039573	1.305490739	-1.112255744	0.266028206	0.962465655	ENSG00000166920	C15orf48
24.31758335	-0.648948741	0.703930474	-0.921893233	0.356584269	0.979427314	ENSG00000140459	CYP11A1
23.9656506	-0.096106284	0.824310891	-0.116589851	0.907185081	0.999527751	ENSG00000141574	SECTM1
24.39923999	0.667627837	0.577524448	1.156016579	0.247674376	0.962465655	ENSG00000175322	ZNF519
23.99794793	-0.359001543	0.655718591	-0.547493312	0.584039864	0.99483168	ENSG00000274423	SEC22B2
23.51630519	-0.468769528	0.621759061	-0.753940806	0.450884767	0.99135833	ENSG00000122592	HOXA7
23.18810808	-0.405710475	4.713695933	-0.086070566	0.931410322	0.999527751	ENSG00000226278	PSPHP1
24.17974175	-0.477052522	0.652315152	-0.731322154	0.464582402	0.99135833	ENSG00000280734	LINC01232
23.94548268	1.184356765	0.757439455	1.563632259	0.117903955	0.923922314	ENSG00000161960	EIF4A1
24.0718873	-0.030487482	0.577597915	-0.052783227	0.957904625	0.999527751	ENSG00000104883	PEX11G
23.80058708	0.411667642	0.60752933	0.677609495	0.498019324	0.993513219	ENSG00000203724	C1orf53
24.72103305	0.59308412	0.62264468	0.952524192	0.340831203	0.977691382	ENSG00000168026	TTC21A
23.96554879	-0.642113999	0.609601185	-1.053334564	0.292187683	0.970706863	ENSG00000220472	AL139095.2
23.88595787	-0.30751864	0.620535666	-0.495569645	0.620198074	0.99483168	ENSG00000158856	DMTN
24.25401318	-0.38524098	0.653572772	-0.58943854	0.55556713	0.99483168	ENSG00000234664	HMGN2P5
24.0624869	-0.178084959	0.656565533	-0.271237142	0.786208648	0.999467288	ENSG00000259673	IQCH-AS1
24.2374531	-0.193891258	1.013770558	-0.191257535	0.848323829	0.999467288	ENSG00000241684	ADAMTS9-AS2
23.33701692	-1.776408976	0.707584085	-2.510527036	0.012055109	0.648356243	ENSG00000254109	RBPMS-AS1
23.60532131	-0.225224117	0.841830595	-0.267540903	0.789052726	0.999467288	ENSG00000278266	AC079949.2
23.39117852	-0.676290674	0.616925345	-1.096227736	0.272979125	0.962465655	ENSG00000258920	FOXN3-AS1
23.67681096	0.29247977	0.722723398	0.40469116	0.685704541	0.998869317	ENSG00000105514	RAB3D

23.95431522	-0.145170349	0.778371182	-0.186505297	0.852048525	0.999467288	ENSG00000105767	CADM4
23.69344764	0.0679033	0.627406533	0.108228551	0.913814398	0.999527751	ENSG00000273344	PAXIP1-AS1
23.60819173	1.020051486	0.682348297	1.494913214	0.134937093	0.933741583	ENSG00000102098	SCML2
23.62747398	-0.223447753	0.605399894	-0.369091167	0.712059773	0.99900742	ENSG00000225071	AC004552.1
24.55406916	-0.42469955	0.732916648	-0.579465006	0.562275452	0.99483168	ENSG00000240445	FOXO3B
24.15341921	0.569406697	0.611337657	0.93141113	0.351640936	0.977691382	ENSG00000237438	CECR7
23.64786356	-0.752186164	0.682151969	-1.102666558	0.270171994	0.962465655	ENSG00000156875	MFSD14A
23.68558089	0.282893217	0.74788829	0.378255978	0.705240445	0.99900742	ENSG00000186364	NUDT17
24.19742875	1.637997	0.679478061	2.410669447	0.015923273	0.696728876	ENSG00000259865	AL390728.6
23.14530733	0.413145653	0.718485901	0.57502263	0.565275992	0.99483168	ENSG00000242759	LINC00882
23.7859238	-1.376441941	0.591293034	-2.327850764	0.019920029	0.73975641	ENSG00000284753	EEF1AKMT4
22.69901923	2.105124476	0.893021491	2.357305503	0.018408096	0.726053908	ENSG00000165899	OTOGL
24.51082502	-0.413812344	0.699120886	-0.59190385	0.553914975	0.99483168	ENSG00000172915	NBEA
24.34770408	0.639682127	0.703381621	0.909438217	0.363118856	0.98048242	ENSG00000213983	AP1G2
23.40282351	1.562789353	1.095524149	1.426522049	0.153717707	0.939432741	ENSG00000083454	P2RX5
23.44305111	-0.9100278	0.718480902	-1.266599846	0.205298407	0.954310328	ENSG00000217716	RPS10P3
23.65701272	0.091605025	0.636815084	0.143848705	0.885619942	0.999467288	ENSG00000130558	OLFM1
23.47423781	-0.520631027	0.613226576	-0.849002714	0.395879782	0.988378925	ENSG00000164107	HAND2
23.48286854	0.336656877	0.686761778	0.490209106	0.623985938	0.99483168	ENSG00000050344	NFE2L3
24.00769896	-0.002157612	0.686777983	-0.003141644	0.997493335	0.999527751	ENSG00000148057	IDNK
22.99255015	0.233242384	0.664220274	0.35115216	0.725474198	0.999467288	ENSG00000246067	RAB30-AS1
24.00860444	-0.028754304	0.723977836	-0.039717105	0.968318664	0.999527751	ENSG00000188878	FBF1
22.49077016	0.237757582	1.009598833	0.235497085	0.813822941	0.999467288	ENSG00000185860	CCDC190
23.27659253	0.255081071	0.634548321	0.40198841	0.687692556	0.998869317	ENSG00000272913	AC009237.14
23.95850084	-1.096457939	0.773808039	-1.416963748	0.156493527	0.939464191	ENSG00000242516	LINC00960
23.01562265	-0.306292534	0.656863049	-0.466295882	0.641003726	0.996553586	ENSG00000086300	SNX10
23.90244232	0.70533599	1.414034598	0.498810985	0.617912548	0.99483168	ENSG00000181652	ATG9B
23.57537195	-0.072254849	0.596418552	-0.12114789	0.903573898	0.999527751	ENSG00000269743	SLC25A53
23.74916096	-0.164272986	0.766983884	-0.214180492	0.830406308	0.999467288	ENSG00000236739	CLIC4P1
24.37204363	0.427562772	0.713674879	0.599100212	0.54910606	0.99483168	ENSG00000165115	KIF27
24.0051837	0.857823913	0.696863198	1.230978929	0.218330745	0.956884001	ENSG00000179532	DNHD1
23.61492278	0.058377314	0.622881198	0.093721426	0.92533045	0.999527751	ENSG00000134899	ERCC5
22.63804424	0.289261257	0.867357573	0.333497125	0.738759059	0.999467288	ENSG00000185634	SHC4
23.75037818	-0.025485005	1.011040533	-0.025206709	0.979890086	0.999527751	ENSG00000259518	LINC01583
23.00305287	-2.384869789	0.991273098	-2.40586554	0.016134202	0.698816035	ENSG00000162882	HAAO
23.98950857	-0.76248876	0.794465279	-0.959750892	0.337180603	0.977691382	ENSG00000231890	DARS-AS1
23.3825746	-0.362231057	0.561960386	-0.644584683	0.519196364	0.993513219	ENSG00000212994	RPS26P6
23.36940914	0.706399115	0.739613473	0.955092276	0.339531035	0.977691382	ENSG00000229847	EMX2OS
23.34853327	0.790778392	0.604884551	1.307321193	0.19110365	0.949234949	ENSG00000266173	STRADA
23.07514591	0.886550892	0.688079229	1.288443037	0.197591786	0.954310328	ENSG00000082397	EPB41L3
23.88371424	-0.030033405	0.655035926	-0.045850012	0.963429797	0.999527751	ENSG00000167216	KATNAL2
22.98776273	-0.831440379	1.096845126	-0.758028968	0.448433641	0.99135833	ENSG00000177757	FAM87B
22.95190311	0.527235666	0.748032054	0.704830311	0.480915843	0.991417002	ENSG00000281398	SNHG4
23.0757067	-1.461896927	0.657025712	-2.225022401	0.026079734	0.762934669	ENSG00000229720	AL109924.2
22.87498947	0.703578135	0.831819551	0.845830246	0.39764745	0.988378925	ENSG00000132321	IQCA1
22.45877184	-0.551409027	0.831213801	-0.663378094	0.507088432	0.993513219	ENSG00000228495	LINC01013
22.33756146	0.949803963	0.75422659	1.259308509	0.207918921	0.954310328	ENSG00000106123	EPHB6
22.95993235	1.534223689	0.658628719	2.329421183	0.019836764	0.73975641	ENSG00000244198	AC004889.1
23.09833892	-0.24949245	0.669644987	-0.372574207	0.709465372	0.99900742	ENSG00000275160	AL354718.1
24.47965229	1.814781456	3.27048001	0.554897584	0.578964715	0.99483168	ENSG00000196611	MMP1
23.329627	0.026603637	0.588132046	0.045234122	0.963920697	0.999527751	ENSG00000179361	ARID3B
22.37318871	0.271927847	0.787757447	0.345192354	0.729949766	0.999467288	ENSG00000266865	AC138207.8
24.07994928	-0.797387774	0.939787448	-0.848476723	0.39617253	0.988378925	ENSG00000169432	SCN9A
21.84098367	2.150491465	1.009669472	2.12989649	0.03318016	0.775963115	ENSG00000166924	NYAP1
22.73094297	0.099979739	0.666889362	0.149919529	0.880828104	0.999467288	ENSG00000261455	LINC01003
22.64724753	0.274042333	0.720704566	0.380242259	0.703765592	0.99900742	ENSG00000235453	SMIM27
23.56976346	-0.477238746	0.762141317	-0.626181437	0.531195941	0.99483168	ENSG00000036672	USP2
22.95828957	-0.671364914	0.661687092	-1.01462598	0.31028415	0.973427483	ENSG00000227825	SLC9A7P1
23.3467709	0.549388544	0.7458084	0.736634963	0.461344367	0.99135833	ENSG00000231607	DLEU2
23.25978866	-0.41983782	0.724355975	-0.579601515	0.562183371	0.99483168	ENSG00000238045	AC009133.1
22.99356495	0.266050657	0.609505342	0.436502585	0.662472124	0.997568624	ENSG00000273311	DGCR11
23.14152497	1.233649994	0.611464271	2.017534061	0.04363981	0.807325344	ENSG00000158286	RNF207
23.0911127	3.254359621	1.174624723	2.770552635	0.005596125	0.495857916	ENSG00000085999	RAD54L
23.12282458	-0.118514177	0.682514628	-0.173643423	0.862145704	0.999467288	ENSG00000179388	EGR3

22.37951419	0.905691733	0.72819401	1.243750596	0.213591368	0.954310328	ENSG00000165511	C10orf25
22.72340586	-0.045301525	0.622184992	-0.072810379	0.941957012	0.999527751	ENSG00000221817	PPP3CB-AS1
22.7010065	0.051310013	0.610236609	0.084082162	0.932991107	0.999527751	ENSG00000237489	C10orf143
22.70496766	0.147449611	0.647737428	0.227637936	0.819927726	0.999467288	ENSG00000164008	C1orf50
22.33355928	-0.950431151	0.861368208	-1.103397063	0.269854772	0.962465655	ENSG00000186472	PCLO
24.03233659	-0.022563623	0.824889737	-0.027353502	0.978177785	0.999527751	ENSG00000223509	AC135983.2
22.92528717	1.862933916	0.714351984	2.60786553	0.009110874	0.611285922	ENSG00000261556	SMG1P7
22.80538557	0.087568485	0.589358561	0.148582698	0.881882926	0.999467288	ENSG00000159110	IFNAR2
23.15812612	0.295985629	0.884468842	0.334647887	0.737890717	0.999467288	ENSG00000090539	CHRD
22.58747501	-0.518444735	0.721277726	-0.718786559	0.47227244	0.99135833	ENSG00000223361	FTH1P10
22.94595508	-0.401575254	0.706701355	-0.568238975	0.56987271	0.99483168	ENSG00000159784	FAM131B
23.05354977	-0.647131792	0.692509104	-0.934474057	0.350059412	0.977691382	ENSG00000182916	TCEAL7
21.38213279	1.648816555	0.862955861	1.910661516	0.056048093	0.847598083	ENSG00000275882	IKBKGP1
22.53836211	1.507053682	0.599846069	2.512400697	0.011991285	0.648356243	ENSG00000188234	AGAP4
22.48238942	-1.105057964	0.708410368	-1.559912184	0.118780635	0.923922314	ENSG00000234494	SP2-AS1
22.78710587	0.106691628	0.66529926	0.160366371	0.872592479	0.999467288	ENSG00000264278	ZNF236-DT
23.17347949	-0.448123258	0.743114847	-0.60303365	0.546486304	0.99483168	ENSG00000100399	CHADL
21.41507671	-0.224964305	0.935904375	-0.240371037	0.810042629	0.999467288	ENSG00000163815	CLEC3B
22.99515885	-0.211034963	0.670207862	-0.314879868	0.752852868	0.999467288	ENSG00000164136	IL15
22.93896346	0.560682519	0.591643115	0.947670149	0.343297402	0.977691382	ENSG00000177082	WDR73
22.66029304	-0.140077301	0.712075196	-0.196717006	0.844049001	0.999467288	ENSG00000196502	SULT1A1
22.81845001	-0.594901065	0.691822043	-0.859904755	0.389841547	0.987216662	ENSG00000276075	AC027682.6
22.93741605	-1.189034276	0.801963119	-1.482654561	0.138166213	0.934144427	ENSG00000205670	SMIM11A
22.66167942	0.023545253	0.591337567	0.039816941	0.96823907	0.999527751	ENSG00000170264	FAM161A
22.72177751	-0.408940577	0.709350352	-0.57650014	0.564277175	0.99483168	ENSG00000196872	KIAA1211L
22.67401103	-0.075629375	1.025911554	-0.073719196	0.941233824	0.999527751	ENSG00000225205	AC078883.1
23.08384994	0.066491565	0.684323567	0.097163927	0.922596215	0.999527751	ENSG00000154928	EPHB1
22.79842454	0.388510165	0.582528917	0.666937131	0.504812292	0.993513219	ENSG00000231686	Z97180.1
22.92433009	0.771720376	0.651760516	1.184055121	0.236391244	0.962465655	ENSG00000154134	ROBO3
22.26094802	1.763848544	0.869476442	2.028632932	0.042495692	0.806698895	ENSG00000256663	AC112777.1
22.78448738	-0.613355762	0.641683542	-0.955853972	0.339146016	0.977691382	ENSG00000125246	CLYBL
22.80166399	0.099930108	0.599958653	0.166561657	0.867714965	0.999467288	ENSG00000186026	ZNF284
22.49938571	-0.381903202	0.649791144	-0.587732236	0.556712035	0.99483168	ENSG00000248569	CRSP8P
21.7380685	-0.071741122	0.789713569	-0.090844485	0.927616163	0.999527751	ENSG00000062582	MRPS24
22.51520141	0.579838791	0.752034303	0.771027052	0.440690897	0.99135833	ENSG00000256940	AP001453.2
22.43430371	0.213097358	0.566556846	0.376127056	0.706822441	0.99900742	ENSG00000213638	ADAT3
22.12391742	0.675406294	0.667418546	1.011968125	0.311553294	0.973427483	ENSG00000236144	TMEM147-AS1
22.05651029	0.319286881	0.681396453	0.468577257	0.639371837	0.996553586	ENSG00000135838	NPL
22.13310211	0.081132016	0.589241637	0.137688872	0.890486315	0.999467288	ENSG00000114529	C3orf52
22.4765302	-0.591106936	0.62721789	-0.942426779	0.345974182	0.977691382	ENSG00000248927	AC114284.1
22.68004185	-0.395451501	0.627286438	-0.630416149	0.528422348	0.99483168	ENSG00000233247	AC002524.1
22.01526012	-0.230018544	0.726950008	-0.316415904	0.751686847	0.999467288	ENSG00000130045	NXNL2
22.21033489	0.16163403	0.743123926	0.217506158	0.827813908	0.999467288	ENSG00000234964	FABP5P7
22.35496145	-1.242665649	0.655052435	-1.897047598	0.057821656	0.852306368	ENSG00000168282	MGAT2
23.31648535	-0.42669419	0.952167398	-0.448129385	0.654059822	0.997527562	ENSG00000180777	ANKRD30B
22.37961706	-0.242643885	0.599593231	-0.404680828	0.685712136	0.998869317	ENSG00000224086	AC245452.1
22.8071183	0.207367635	0.971589275	0.213431375	0.83099051	0.999467288	ENSG00000132688	NES
22.49496129	-0.696244503	0.697509693	-0.998186133	0.318189109	0.974768742	ENSG00000178187	ZNF454
21.55955577	-0.715766711	0.890463237	-0.803813882	0.421504471	0.991301167	ENSG00000184363	PKP3
22.11282892	0.495584196	0.691921413	0.716243473	0.473841024	0.99135833	ENSG00000167220	HDHD2
22.30525623	0.355519274	0.70704008	0.502827611	0.615085479	0.99483168	ENSG00000173200	PARP15
22.38768013	-0.169209704	0.63789362	-0.265263202	0.79080671	0.999467288	ENSG00000248632	AC106872.5
22.21319756	-1.30765675	0.780738641	-1.674896927	0.093954457	0.921147998	ENSG00000238271	IFNWP19
22.77200067	-0.0563772	0.853340981	-0.066066439	0.94732493	0.999527751	ENSG00000234323	LINC01505
22.31903396	-0.816731397	0.909699458	-0.897803544	0.369290295	0.981455968	ENSG00000064195	DLX3
22.51649169	-0.170831541	0.736397356	-0.231982827	0.816551352	0.999467288	ENSG00000141965	FEM1A
22.25125593	-0.138161221	0.648689515	-0.212985131	0.831338561	0.999467288	ENSG00000240990	HOXA11-AS
21.17387572	0.059824687	0.892072178	0.067062608	0.946531861	0.999527751	ENSG00000130822	PNCK
22.25510129	-0.189721948	0.638812377	-0.296991659	0.766472877	0.999467288	ENSG00000136040	PLXNC1
21.88124749	-0.476627756	0.696644896	-0.684176054	0.493864001	0.993513219	ENSG00000185834	RPL12P4
22.70145885	1.296277683	0.745810155	1.738079957	0.082196726	0.899345331	ENSG00000130270	ATP8B3
21.46091895	-0.561426565	0.743334887	-0.755280796	0.450080519	0.99135833	ENSG00000064886	CHI3L2
21.7333476	-0.169220694	0.661180826	-0.255937086	0.797999417	0.999467288	ENSG00000233429	HOTAIRM1
22.40374817	-0.481546966	0.649618776	-0.741276244	0.458525963	0.99135833	ENSG00000197180	CH17-340M24.3

22.63691275	-0.325925068	0.679572463	-0.479603112	0.631509633	0.99483168	ENSG00000254198	AC113191.1
22.37931135	0.780947355	0.766318069	1.019090358	0.308160071	0.973427483	ENSG00000166689	PLEKHA7
22.12534409	-0.486320373	0.594906857	-0.817473135	0.413658096	0.990226751	ENSG00000285331	AC090517.5
21.77295962	-0.561846553	0.671301534	-0.836951093	0.402620059	0.988836748	ENSG00000125775	SDCBP2
22.074893	-1.042823031	0.740508918	-1.40825182	0.159056517	0.939523519	ENSG00000128284	APOL3
21.50264876	-0.435196514	0.742615744	-0.586031898	0.557854079	0.99483168	ENSG00000175279	CENPS
22.10551814	-0.7978941	0.794047329	-1.004844512	0.314971727	0.974768742	ENSG00000245146	MALINC1
22.11530685	0.363763737	0.617368488	0.58921656	0.55571601	0.99483168	ENSG00000196391	ZNF774
22.29590044	1.350880317	0.719760701	1.87684645	0.060539142	0.858996918	ENSG00000233966	UBE2SP1
22.26765213	-0.088289822	0.626180764	-0.140997339	0.887872044	0.999467288	ENSG00000161681	SHANK1
22.06158204	0.475929299	0.615349497	0.773429249	0.439268386	0.991301167	ENSG00000183647	ZNF530
22.03254695	0.009366025	0.675786675	0.01385944	0.988942121	0.999527751	ENSG00000174137	FAM53A
22.302535	-0.401072291	0.935391546	-0.428774766	0.668087145	0.997568624	ENSG00000226259	GTF2H2B
22.22085532	0.464352682	0.788544427	0.588873202	0.555946335	0.99483168	ENSG00000113211	PCDHB6
22.06278542	-1.279147646	0.691083081	-1.850931793	0.064179366	0.868202292	ENSG00000231889	TRAF3IP2-AS1
21.81538049	-0.528102101	0.581514374	-0.908149696	0.363799137	0.98048242	ENSG00000005249	PRKAR2B
22.31843667	-1.811689873	0.933780837	-1.940166045	0.052359514	0.846840376	ENSG00000161249	DMKN
21.48921239	1.833002008	1.080310568	1.696736163	0.089746552	0.910413547	ENSG00000143869	GDF7
21.7330991	-0.417026231	0.938607613	-0.444303057	0.656823492	0.997568624	ENSG00000146592	CREB5
21.96820844	-0.602703085	0.65894851	-0.914643673	0.360378724	0.979465649	ENSG00000257038	AP002761.3
21.94166656	-0.37810022	0.628436692	-0.601652044	0.547405778	0.99483168	ENSG00000207652	MIR621
20.71552414	1.384296303	1.017294397	1.360762731	0.173588683	0.944422693	ENSG00000157601	MX1
21.87943101	0.010628777	0.613010232	0.017338661	0.986166443	0.999527751	ENSG00000256628	ZBTB11-AS1
21.38527663	-0.696590019	0.633218479	-1.100078475	0.271297932	0.962465655	ENSG00000280832	GSEC
22.10720722	0.311442704	0.668383047	0.465964397	0.641240985	0.996553586	ENSG00000274964	AC026356.1
21.23384108	1.63618832	1.102487678	1.484087625	0.137785676	0.934144427	ENSG00000063180	CA11
22.12232817	-0.163471549	0.759568311	-0.215216389	0.829598614	0.999467288	ENSG00000152433	ZNF547
22.3185722	-0.350551991	0.751530615	-0.466450712	0.64089292	0.996553586	ENSG00000128266	GNAZ
21.42187548	1.158655585	0.723049636	1.602456494	0.109054701	0.923922314	ENSG00000156265	MAP3K7CL
21.460403	-0.496519223	0.909352258	-0.546014175	0.585056193	0.99483168	ENSG00000237945	LINC00649
21.46991335	-0.131841712	0.938603188	-0.140465869	0.888291918	0.999467288	ENSG00000185565	LSAMP
22.11685786	0.709227406	0.71733986	0.988690919	0.322814387	0.975337095	ENSG00000280347	AC000123.3
21.99736501	0.760420907	0.704950856	1.078686409	0.280727547	0.963267117	ENSG00000259953	AL138756.1
21.72039158	0.68061639	0.583451065	1.166535518	0.243397997	0.962465655	ENSG00000259994	AL353796.1
22.45143501	1.112117528	0.889224795	1.250659603	0.211058695	0.954310328	ENSG00000241749	RPSAP52
21.49572017	-0.787246085	0.689787337	-1.141288109	0.253750049	0.962465655	ENSG00000233954	UQCRHL
21.25647101	-0.317937759	0.744518948	-0.427037835	0.669351771	0.997568624	ENSG00000227855	DPY19L2P3
21.77070925	-0.93621777	0.628815903	-1.48885829	0.136524689	0.934144427	ENSG00000236753	MKLN1-AS
21.73436258	-0.181346389	0.779024714	-0.232786438	0.815927244	0.999467288	ENSG00000149043	SYT8
21.28936609	0.451981163	0.710152991	0.636456044	0.524479222	0.99483168	ENSG00000248508	SRP14-AS1
21.76523283	0.432997897	0.671304799	0.645009388	0.518921102	0.993513219	ENSG00000274654	AC022467.1
21.51283993	0.176909427	0.762451204	0.232027212	0.816516879	0.999467288	ENSG00000170537	TMC7
21.45470002	0.102036895	0.918511503	0.111089403	0.911545452	0.999527751	ENSG00000143867	OSR1
21.68794101	1.065320319	0.657646535	1.619898019	0.105254186	0.92169898	ENSG00000204822	MRPL53
22.00740689	0.561489469	0.751528769	0.747129708	0.454985269	0.99135833	ENSG00000253485	PCDHGA5
22.20922837	0.163895303	1.30522454	0.125568665	0.900073368	0.999527751	ENSG00000126500	FLRT1
21.99843559	0.428730198	0.654089519	0.655461042	0.512170961	0.993513219	ENSG00000180953	ST20
21.73239489	-0.138952986	0.761941125	-0.182367091	0.85529465	0.999467288	ENSG00000102934	PLLP
20.92914639	0.356424481	0.681833936	0.522743827	0.601152535	0.99483168	ENSG00000177337	DLGAP1-AS1
21.68320614	0.297902902	0.712708552	0.417986989	0.67595663	0.998869317	ENSG00000251247	ZNF345
20.52756128	1.024555784	0.830338396	1.23390149	0.217239614	0.956186483	ENSG00000138395	CDK15
21.97883626	0.12997156	0.817899122	0.158909035	0.873740542	0.999467288	ENSG00000138670	RASGEF1B
21.47137403	-0.339699469	0.749130717	-0.453458204	0.650218833	0.997381078	ENSG00000133937	GSC
21.8885192	0.065444774	0.642450267	0.101867455	0.918861882	0.999527751	ENSG00000261971	MMP25-AS1
21.13616958	-0.555103872	0.705346632	-0.786994432	0.431285126	0.991301167	ENSG00000234684	SDCBP2-AS1
21.29820481	0.222710049	0.709432486	0.313927052	0.753576444	0.999467288	ENSG00000244625	MIATNB
21.06847946	-0.559914133	0.82631665	-0.677602385	0.498023833	0.993513219	ENSG00000188060	RAB42
21.62333022	-0.935687844	0.695203913	-1.345918553	0.178328785	0.9469453	ENSG00000272086	AC025181.2
21.71861812	0.095021208	0.838798999	0.113282453	0.909806628	0.999527751	ENSG00000235272	RAMACL
21.03733763	0.577186187	0.924537703	0.624297079	0.532432502	0.99483168	ENSG00000259583	AC015712.2
22.24983217	0.234513487	0.948310362	0.247296135	0.80467905	0.999467288	ENSG00000108556	CHRNE
21.2454061	-2.011950597	0.912653264	-2.204507096	0.027488701	0.769167109	ENSG00000205795	CYS1
21.48486099	-0.455032561	0.710852652	-0.640122196	0.52209316	0.994503371	ENSG00000285219	AL591485.1
20.94687809	0.105327089	0.690416129	0.15255595	0.878748465	0.999467288	ENSG00000205485	AC004980.1

21.59729062	-0.678018951	0.635229077	-1.067361328	0.285808704	0.964826902	ENSG00000165028	NIPSNAP3B
20.71557268	0.334932963	0.694150931	0.482507404	0.62944554	0.99483168	ENSG00000198298	ZNF485
21.09336665	-0.120264123	0.691824956	-0.173836058	0.861994306	0.999467288	ENSG00000272447	AL135925.1
21.68365037	0.34349984	0.646172568	0.531591492	0.595008958	0.99483168	ENSG00000203772	SPRN
21.34351333	-1.062892477	0.830787384	-1.279379655	0.200763395	0.954310328	ENSG00000256894	AC022509.3
20.73010039	0.157978962	1.23516043	0.127901573	0.898226866	0.999490224	ENSG00000162069	BICDL2
21.28308465	0.734005124	0.658682277	1.114353839	0.265127418	0.962465655	ENSG00000257122	RRN3P3
21.72962609	1.603792459	0.68886188	2.328177108	0.019902701	0.73975641	ENSG00000269858	EGLN2
21.71858566	0.45586036	0.954128498	0.477776695	0.63280915	0.994909189	ENSG00000116690	PRG4
21.02076696	0.735937409	0.637905829	1.153677198	0.248632536	0.962465655	ENSG00000144031	ANKRD53
20.43767357	-0.403467033	0.840716087	-0.479908782	0.631292256	0.99483168	ENSG00000236397	DDX11L2
20.79465745	0.160388538	0.641725847	0.249933112	0.802639076	0.999467288	ENSG00000144362	PHOSPHO2
21.21656429	0.034046077	0.628028991	0.054210996	0.95676706	0.999527751	ENSG00000223773	CD99P1
21.10416652	-0.070618458	0.674637988	-0.104676077	0.916632846	0.999527751	ENSG00000215492	HNRNPA1P7
20.66474811	1.741101142	0.702542732	2.478285037	0.013201563	0.670420654	ENSG00000260001	TGFBR3L
20.94544195	0.051614691	0.933239479	0.055307016	0.955893873	0.999527751	ENSG00000116771	AGMAT
21.4466629	0.155210574	0.747523029	0.207633168	0.835515405	0.999467288	ENSG00000127957	PMS2P3
20.79748131	0.334231818	0.738539475	0.452557824	0.650867174	0.997449432	ENSG00000260461	AL133355.1
20.88234398	-1.475443524	0.769546218	-1.917290333	0.055201057	0.847598083	ENSG00000130755	GMFG
20.64989358	0.60097791	0.675064027	0.890253199	0.373329945	0.982329093	ENSG00000161896	IP6K3
20.86288132	0.066170385	0.693267703	0.09544709	0.923959714	0.999527751	ENSG00000172167	MTBP
20.14397119	-0.299582867	0.873459687	-0.342984194	0.731610351	0.999467288	ENSG00000106789	CORO2A
21.2584999	-1.050805654	1.050045132	-1.000724276	0.316960128	0.974768742	ENSG00000169918	OTUD7A
20.81571492	0.207162085	0.619688487	0.334300361	0.738152918	0.999467288	ENSG00000155666	KDM8
20.75207731	0.229255236	0.745095051	0.307685893	0.758321355	0.999467288	ENSG00000160602	NEK8
20.60483363	0.459667091	0.714249182	0.643566842	0.519856358	0.993513219	ENSG00000279253	AL121753.2
20.45821637	0.261165299	0.678991954	0.38463681	0.700506518	0.998869317	ENSG00000227232	WASH7P
20.80466853	-0.90335273	0.683880037	-1.320922796	0.186527108	0.949094286	ENSG00000162643	WDR63
20.85309073	0.817848144	0.619504275	1.320165456	0.186779783	0.949094286	ENSG00000204923	FBXO48
20.71625455	-0.140082236	0.687829623	-0.203658335	0.838620503	0.999467288	ENSG00000213300	HNRNPA3P6
20.7145233	1.009899226	0.871535601	1.158758432	0.246554666	0.962465655	ENSG00000169760	NLGN1
20.67256127	1.002195139	0.797886985	1.25606152	0.209093666	0.954310328	ENSG00000225138	SLC9A3-AS1
21.33526888	0.391471553	0.877850283	0.445943415	0.65563812	0.997568624	ENSG00000131941	RHPN2
20.7775766	0.491386858	0.682500969	0.719979722	0.471537481	0.99135833	ENSG00000134245	WNT2B
21.37641338	0.024831162	0.714932355	0.034732184	0.972293297	0.999527751	ENSG00000272150	NBPF25P
21.0456695	1.227230793	0.665299333	1.844629526	0.065091449	0.868202292	ENSG00000243679	AC018638.5
20.33786159	-0.488492026	0.743036547	-0.657426648	0.510906622	0.993513219	ENSG00000029534	ANK1
21.23982495	-0.756220689	0.808184775	-0.935702717	0.349426272	0.977691382	ENSG00000254860	TMEM9B-AS1
20.45433389	0.649717203	1.221980468	0.531691971	0.594939354	0.99483168	ENSG00000101230	ISM1
20.65535117	0.441633453	0.719393486	0.61389693	0.539283439	0.99483168	ENSG00000142046	TMEM91
20.40128796	-0.569497025	0.806457371	-0.706171269	0.480081635	0.99135833	ENSG00000100027	YPEL1
20.58368235	0.422721939	0.585704508	0.721732432	0.470458998	0.99135833	ENSG00000176714	CCDC121
20.51625075	0.236811042	1.024226012	0.231209752	0.817151854	0.999467288	ENSG00000164112	TMEM155
20.69344366	0.654440732	0.761128845	0.859829103	0.389883254	0.987216662	ENSG00000162062	TEDC2
20.17885452	1.412485403	0.679340615	2.079200584	0.037598918	0.790190505	ENSG00000101104	PABPC1L
20.64059462	-0.002392036	0.727472558	-0.003288146	0.997376444	0.999527751	ENSG00000254221	PCDHGB1
19.9284861	-0.114689079	0.788606449	-0.14543259	0.884369335	0.999467288	ENSG00000237523	LINC00857
20.41940097	-0.012993062	0.627922835	-0.020692132	0.983491245	0.999527751	ENSG00000180581	SRP9P1
20.99310318	-0.631029803	0.908379959	-0.694676051	0.48725834	0.993513219	ENSG00000259969	AL049838.1
20.29209057	1.100344703	0.677054314	1.625194141	0.104121199	0.92169898	ENSG00000223797	ENTPD3-AS1
19.83407649	-0.669937777	0.755408178	-0.886855342	0.375156788	0.982609538	ENSG00000217130	AL139100.1
20.68988821	-0.003568295	0.695730881	-0.005128844	0.995907792	0.999527751	ENSG00000161912	ADCY10P1
20.85401451	0.041655786	0.683625582	0.060933627	0.951412069	0.999527751	ENSG00000280123	AC023632.6
20.90497725	-0.745415238	0.87341002	-0.853453957	0.39340761	0.988378925	ENSG00000254458	AP001107.3
20.63137744	0.93950002	0.733893962	1.280157719	0.200489673	0.954310328	ENSG00000187801	ZFP69B
19.85249349	-0.052037981	0.7402297	-0.070299775	0.943955062	0.999527751	ENSG00000143412	ANXA9
20.43571007	-0.389997214	0.749708872	-0.520198212	0.602925432	0.99483168	ENSG00000213222	AC093724.1
19.81313325	0.705573529	0.823331332	0.85697398	0.391459265	0.988355932	ENSG00000145794	MEGF10
20.33687637	1.105743001	1.005958976	1.099192937	0.271683919	0.962465655	ENSG00000146410	MTFR2
20.12412987	1.146702826	1.0107262	1.134533592	0.256570791	0.962465655	ENSG00000196584	XRCC2
20.92998057	-0.227779944	0.778640368	-0.292535493	0.769877226	0.999467288	ENSG00000226808	LINC00840
20.86001708	2.13278321	1.022837607	2.085163075	0.0370545	0.790190505	ENSG00000073605	GSDMB
20.64365094	0.506244067	0.669488093	0.7561659	0.449549735	0.99135833	ENSG00000129646	QRICH2
20.3079158	0.20722834	0.635477929	0.326098406	0.744349903	0.999467288	ENSG00000237424	FOXD2-AS1

20.37134468	-1.485446245	1.054286869	-1.408958309	0.158847496	0.939464191	ENSG00000064692	SNCAIP
19.82200449	0.552019939	0.777058258	0.710397108	0.477457916	0.99135833	ENSG00000260653	AC237221.1
20.8112799	1.384709594	0.988338762	1.401047544	0.161199856	0.942964826	ENSG00000166448	TMEM130
20.69033969	0.841383109	1.320336174	0.637249153	0.523962565	0.99483168	ENSG00000151025	GPR158
20.19390853	0.723196229	0.63461374	1.139584889	0.254459284	0.962465655	ENSG00000213903	LTB4R
19.50214809	-0.156195749	0.883312469	-0.176829553	0.859642275	0.999467288	ENSG00000197971	MBP
19.08720649	0.543584769	1.01993243	0.53296155	0.594060197	0.99483168	ENSG00000188015	S100A3
20.40210815	0.321354598	0.678860703	0.473373399	0.635946805	0.996399501	ENSG00000174529	TMEM81
20.35180556	-0.384702235	0.703445333	-0.546882917	0.584459173	0.99483168	ENSG00000204745	AC083899.1
20.3747638	-0.224266269	0.728645786	-0.307785035	0.75824591	0.999467288	ENSG00000241316	SUCLG2-AS1
19.97623135	2.164781359	0.972957968	2.224948488	0.026084696	0.762934669	ENSG00000147174	GCNA
19.93072254	0.400727319	0.644136824	0.622115215	0.533866111	0.99483168	ENSG00000079337	RAPGEF3
19.89652503	0.757252798	0.677749734	1.117304455	0.263864171	0.962465655	ENSG00000163611	SPICE1
19.31154339	0.088506191	0.717713009	0.123316966	0.901856107	0.999527751	ENSG00000151623	NR3C2
19.374601	0.143253765	0.736163806	0.194594958	0.84571005	0.999467288	ENSG00000166278	C2
19.92386504	-0.19606473	0.656007008	-0.298875969	0.765034684	0.999467288	ENSG00000237264	FTH1P11
20.69824899	-0.412578029	0.972419703	-0.424279791	0.671361775	0.997568624	ENSG00000188488	SERPINA5
20.46988203	-0.085017944	0.784671012	-0.108348522	0.913719234	0.999527751	ENSG00000283633	AP000547.3
20.13457878	0.021074756	0.711486636	0.029620733	0.97636953	0.999527751	ENSG00000251333	RTN3P1
19.46901715	0.337147552	0.878658426	0.383707186	0.701195486	0.998869317	ENSG00000104432	IL7
19.88167078	-0.687143938	0.692380969	-0.9924362	0.320984787	0.975337095	ENSG00000257556	LINC02298
19.77481353	-1.702023568	0.690605956	-2.464536475	0.01371906	0.672931175	ENSG00000159761	C16orf86
20.82210519	-1.100380574	0.989921077	-1.111584145	0.266316993	0.962465655	ENSG00000123700	KCNJ2
19.79190586	0.135428301	0.636171913	0.212880038	0.831420533	0.999467288	ENSG00000230562	FAM133DP
20.19607733	-1.028711097	0.990041867	-1.039058176	0.298777681	0.972186492	ENSG00000091428	RAPGEF4
20.11645097	1.668093274	0.732916193	2.275967279	0.022847968	0.744905651	ENSG00000224660	SH3BP5-AS1
20.08472813	-0.075115763	0.694915602	-0.108093361	0.913921634	0.999527751	ENSG00000163590	PPM1L
19.59258531	0.574223926	0.660961259	0.868770927	0.384972438	0.985733881	ENSG00000152669	CCNO
19.82220773	-0.35878792	0.682866994	-0.52541406	0.599295381	0.99483168	ENSG00000233822	HIST1H2BN
19.95932282	-0.203616928	0.681326775	-0.298853553	0.765051787	0.999467288	ENSG00000219891	ZSCAN12P1
19.39823109	-0.369984304	0.772299737	-0.479068276	0.631890057	0.99483168	ENSG00000272288	AL451165.2
19.98314816	-0.264364509	0.620085433	-0.426335622	0.669863305	0.997568624	ENSG00000217165	ANKRD18EP
20.24004644	0.308975961	0.71514024	0.432049469	0.665705459	0.997568624	ENSG00000147036	LANCL3
20.0102018	0.506030145	0.937054258	0.540022246	0.589181691	0.99483168	ENSG00000068976	PYGM
20.89891916	1.894900015	0.90951746	2.083412467	0.037213643	0.790190505	ENSG00000189423	USP32P3
19.8056713	0.408324091	0.696924184	0.585894564	0.557946371	0.99483168	ENSG00000268912	AC012313.5
19.58427295	-0.521787421	0.846464748	-0.616431366	0.537609857	0.99483168	ENSG00000272711	AC019069.1
20.12485144	1.390725409	0.832344453	1.670853219	0.094750674	0.921147998	ENSG00000251474	RPL32P3
19.90893941	-1.231782785	0.756936555	-1.62732633	0.103667809	0.92169898	ENSG00000120149	MSX2
19.4517233	-0.472298421	4.714061649	-0.100189275	0.92019406	0.999527751	ENSG00000198502	HLA-DRB5
19.59085029	-0.215308116	0.709682847	-0.303386389	0.761595417	0.999467288	ENSG00000272476	AL024507.2
20.25263836	-1.150200156	0.789920597	-1.456095917	0.145366106	0.935644597	ENSG00000230291	AC078817.1
19.02048572	-0.009517409	0.938385092	-0.010142328	0.991907732	0.999527751	ENSG00000267102	AC060766.1
20.16447766	1.663527209	0.942402844	1.765197568	0.077530567	0.897052166	ENSG00000122483	CCDC18
19.89275876	0.826980884	1.054362381	0.784342176	0.432839358	0.991301167	ENSG00000115657	ABCB6
19.27364452	0.54974396	0.778487641	0.706169155	0.480082949	0.99135833	ENSG00000262185	AC005736.1
20.17257803	1.444073105	0.8225819	1.755537175	0.079167468	0.897052166	ENSG00000183604	SMG1P5
20.24508842	-0.79300465	0.658048174	-1.205086013	0.228170099	0.958865115	ENSG00000267787	AC027097.2
19.9543301	1.252760455	0.684998468	1.82885147	0.067421862	0.87420149	ENSG00000088305	DNMT3B
19.5529985	-0.590826101	0.606797877	-0.97367859	0.330216151	0.976026345	ENSG00000177599	ZNF491
19.69489594	0.221100067	0.670495683	0.329756138	0.741584232	0.999467288	ENSG00000266904	LINC00663
20.02701247	1.79434434	1.06953584	1.677685098	0.09340859	0.921147998	ENSG00000186567	CEACAM19
19.30472675	0.262267278	0.67028356	0.391278099	0.695591685	0.998869317	ENSG00000245556	SCAMP1-AS1
19.90828182	0.429053632	0.698028901	0.614664567	0.538776265	0.99483168	ENSG00000236603	RANP1
19.48706926	0.229407991	0.714297147	0.321166046	0.748084561	0.999467288	ENSG00000228506	AL513550.1
19.84518897	-1.013407405	0.686799179	-1.475551276	0.140064393	0.935644597	ENSG00000232160	RAP2C-AS1
20.08227242	-0.832112356	0.879790177	-0.945807736	0.344246659	0.977691382	ENSG00000172733	PURG
19.56130404	0.730451033	0.735690447	0.992878235	0.320769298	0.975337095	ENSG00000188906	LRRK2
19.60171376	2.116921238	0.816145457	2.593803863	0.009492062	0.622034307	ENSG00000158296	SLC13A3
20.05930586	-0.385481199	0.675404032	-0.570741631	0.568174794	0.99483168	ENSG00000175691	ZNF77
19.45911312	1.476461589	0.84570455	1.745836166	0.080839425	0.897807954	ENSG00000085840	ORC1
20.06204009	0.216244477	0.742276071	0.291326213	0.770801841	0.999467288	ENSG00000115604	IL18R1
19.7693311	-0.581614489	0.719983529	-0.807816381	0.419196294	0.990226751	ENSG00000273186	AL359091.5
19.78624298	1.21729537	1.018545487	1.195131082	0.23203582	0.959575179	ENSG00000154493	C10orf90

19.4117612	0.012687702	0.739367939	0.0171602	0.986308813	0.999527751	ENSG00000102796	DHRS12
19.70035303	0.181861417	0.728932942	0.249489913	0.802981842	0.999467288	ENSG00000160460	SPTBN4
19.16536661	-0.608726936	0.771527669	-0.788989118	0.430118367	0.991301167	ENSG00000140323	DISP2
20.33830181	1.804690233	2.363506416	0.76356477	0.44512665	0.99135833	ENSG00000101210	EEF1A2
19.86886498	0.060095192	0.720063951	0.083458131	0.933487268	0.999527751	ENSG00000180855	ZNF443
18.94483889	-0.45698237	1.03484217	-0.4415962	0.658781434	0.997568624	ENSG00000128606	LRRC17
19.1769015	-0.148846608	0.837815085	-0.177660453	0.858989645	0.999467288	ENSG00000228065	LINC01515
19.31226339	-0.667465756	0.691288211	-0.965539041	0.334274891	0.976026345	ENSG00000170370	EMX2
19.17638661	0.8838721	0.769237826	1.149023189	0.250546419	0.962465655	ENSG00000129538	RNASE1
19.70692441	-1.969708169	1.11219798	-1.771004986	0.076559873	0.897052166	ENSG00000188501	LCTL
19.34622364	0.309828677	0.743504754	0.416713781	0.676887773	0.998869317	ENSG00000176593	AC008969.1
19.28479484	-0.197613876	0.680446388	-0.290417994	0.771496479	0.999467288	ENSG00000169635	HIC2
19.3818745	1.726939623	0.738802416	2.337485078	0.019413976	0.73594765	ENSG00000163376	KBTBD8
19.73459298	1.004175785	0.953910864	1.05269352	0.29248148	0.970706863	ENSG00000163735	CXCL5
18.86833306	1.975618456	1.159466413	1.703903135	0.088399185	0.910413547	ENSG00000109321	AREG
19.06046377	0.060516992	0.718893564	0.08418074	0.932912731	0.999527751	ENSG00000039139	DNAH5
19.17121784	-0.635647371	0.801841827	-0.792734115	0.42793275	0.991301167	ENSG00000203797	DDO
19.36829098	-0.290333513	0.721531205	-0.402385248	0.687400525	0.998869317	ENSG00000253320	AZIN1-AS1
19.2212113	0.353124789	0.617398022	0.571956463	0.56735147	0.99483168	ENSG00000260912	AL158206.1
19.30371279	-0.673092383	0.675446884	-0.996514159	0.319000391	0.974768742	ENSG00000179899	PHC1P1
18.29368767	0.017721863	0.826910109	0.021431426	0.982901505	0.999527751	ENSG00000021300	PLEKHB1
19.27645576	2.235820084	0.847301387	2.638754189	0.008321129	0.600910346	ENSG00000204172	AGAP9
19.14394538	0.306922362	0.995426878	0.308332404	0.757829412	0.999467288	ENSG00000138135	CH25H
18.92603297	-1.020620389	0.698324334	-1.461527745	0.143870667	0.935644597	ENSG00000155629	PIK3AP1
19.11654597	-0.16610192	0.687350608	-0.241655304	0.809047266	0.999467288	ENSG00000197417	SHPK
19.35931315	-0.211614194	0.729927897	-0.289911092	0.771884255	0.999467288	ENSG00000171970	ZNF57
19.53503423	0.334990438	0.683775551	0.489912862	0.624195561	0.99483168	ENSG00000269837	IPO5P1
19.44295229	0.213161254	0.766733685	0.278012117	0.781003058	0.999467288	ENSG00000122481	RWDD3
19.57647966	4.239760141	1.315391598	3.223192353	0.001267704	0.250814355	ENSG00000180287	PLD5
18.90105132	-0.319288258	0.648064765	-0.492679553	0.622239028	0.99483168	ENSG00000214975	PPIAP29
19.37465403	0.040479903	1.169419179	0.034615392	0.972386428	0.999527751	ENSG00000106327	TFR2
19.16573203	-0.446677505	0.681834203	-0.655111614	0.512395895	0.993513219	ENSG00000246089	AC016065.1
19.22157075	0.195972137	1.254317296	0.156238089	0.875845355	0.999467288	ENSG00000275216	AL161431.1
19.03771574	-0.794934911	1.300287134	-0.611353362	0.540965671	0.99483168	ENSG00000166473	PKD1L2
18.4554961	-0.025667508	0.708964624	-0.036204216	0.971119524	0.999527751	ENSG00000196754	S100A2
18.76473497	-0.275812353	0.705606429	-0.390886962	0.695880792	0.998869317	ENSG00000164659	KIAA1324L
18.55504074	-0.131112891	0.76274466	-0.171896177	0.863519152	0.999467288	ENSG00000134028	ADAMDEC1
19.09307694	-0.315136106	0.855373605	-0.368419254	0.712560643	0.99900742	ENSG00000100558	PLEK2
18.93555642	0.461561245	0.647083237	0.71329501	0.475663224	0.99135833	ENSG00000163617	CCDC191
19.01215604	-0.96274994	0.713246091	-1.349814534	0.177075482	0.946540546	ENSG00000067601	PMS2P4
18.94181894	-0.402508982	0.629951302	-0.638952537	0.522853809	0.994616524	ENSG00000214182	PTMAP5
18.40779063	-0.760957236	0.898869779	-0.846571165	0.397234192	0.988378925	ENSG00000159871	LYPD5
18.8440008	-1.017901961	0.679976253	-1.496966926	0.134401864	0.933741583	ENSG00000198835	GJC2
18.6139305	-0.048931073	0.7253489	-0.067458671	0.946216563	0.999527751	ENSG00000250120	PCDHA10
19.79343435	0.633659664	1.035011312	0.612224868	0.540388991	0.99483168	ENSG00000171462	DLK2
18.55987165	-0.90964854	1.262331165	-0.720610063	0.471149461	0.99135833	ENSG00000109063	MYH3
18.8891345	1.94219847	1.052259918	1.845740236	0.064929932	0.868202292	ENSG00000125319	C17orf53
18.72861454	-0.537205119	0.66645256	-0.806066555	0.420204475	0.990778994	ENSG00000223612	AC241585.1
18.76944631	-0.637391925	0.70868712	-0.899398206	0.368440594	0.981443656	ENSG00000158869	FCER1G
18.43467493	1.143041881	0.811117232	1.409219082	0.158770397	0.939464191	ENSG00000120756	PLS1
18.45707853	0.315310978	0.730589442	0.431584361	0.666043527	0.997568624	ENSG00000280047	AC091825.1
18.94429378	-0.172628736	0.77902743	-0.221595196	0.824629023	0.999467288	ENSG00000204390	HSPA1L
18.48667073	0.250322805	0.923053317	0.271189974	0.786244924	0.999467288	ENSG00000235944	ZNF815P
18.36452072	1.229361812	0.773726606	1.588883983	0.112086588	0.923922314	ENSG00000231721	LINC-PINT
18.50819769	-0.078187182	0.694504403	-0.112579822	0.910363683	0.999527751	ENSG00000059377	TBXAS1
18.46317332	0.515876245	0.62803278	0.821416113	0.411409289	0.990226751	ENSG00000127311	HELB
19.10190255	0.472456323	0.69541992	0.679382786	0.496895354	0.993513219	ENSG00000132932	ATP8A2
18.81900244	0.386121538	0.633279343	0.609717564	0.542048918	0.99483168	ENSG00000279423	AL445363.3
18.60076265	0.328063674	0.637685761	0.514459776	0.606930576	0.99483168	ENSG00000171962	DRC3
18.60587215	-1.919022555	0.746091466	-2.572100932	0.010108342	0.630908184	ENSG00000227082	AC244021.1
18.7603116	0.276644124	0.949437034	0.29137701	0.770762996	0.999467288	ENSG00000213139	CRYGS
18.65422889	0.169231637	0.836627522	0.202278352	0.839699122	0.999467288	ENSG00000161267	BDH1
18.91227326	0.514264373	0.792031113	0.649298196	0.516145652	0.993513219	ENSG00000041515	MYO16
18.75033714	0.146393606	0.819054121	0.178734961	0.858145816	0.999467288	ENSG00000231106	LINC01436

18.0958557	-0.392239632	0.89525517	-0.438131658	0.661290847	0.997568624	ENSG00000163485	ADORA1
18.76744117	0.278204026	0.695830502	0.399815796	0.689292196	0.998869317	ENSG00000213123	TCTEX1D2
19.29223504	-0.952070459	0.915143675	-1.040350805	0.298176949	0.972186492	ENSG00000266680	AL135905.1
18.81056979	-1.039893207	0.780741633	-1.331930004	0.182883191	0.949094286	ENSG00000225511	LINC00475
17.73386134	-1.283798552	0.873501443	-1.469715434	0.141638841	0.935644597	ENSG00000255517	AP002748.3
18.59803955	-0.066169261	0.66745868	-0.099136116	0.921030197	0.999527751	ENSG00000226200	SGMS1-AS1
18.46943997	0.234591956	0.717548472	0.326935343	0.743716789	0.999467288	ENSG00000279088	AC022400.7
19.31670829	-0.729340178	1.051045642	-0.693918655	0.487733224	0.993513219	ENSG00000162490	DRAXIN
19.03111481	0.762623098	0.860029143	0.886740995	0.375218362	0.982609538	ENSG00000153898	MCOLN2
18.48403406	0.419167666	0.885710048	0.473256081	0.636030492	0.996399501	ENSG00000163006	CCDC138
18.09824009	1.425617137	1.054294006	1.352200742	0.176311106	0.946278819	ENSG00000138646	HERC5
18.3365676	0.021916965	0.799524251	0.027412508	0.978130722	0.999527751	ENSG00000255690	TRIL
18.79967316	-1.475737576	0.728312388	-2.026242586	0.042739932	0.806698895	ENSG00000137699	TRIM29
18.42520809	-0.107814568	0.864411618	-0.124725959	0.900740506	0.999527751	ENSG00000204176	SYT15
17.64234103	0.174281519	0.968068616	0.18003013	0.857128914	0.999467288	ENSG00000186862	PDZD7
17.88700034	-0.558394757	0.796037149	-0.701468214	0.483010865	0.991892545	ENSG00000186615	KTN1-AS1
18.30430618	1.40031384	0.90339553	1.550056197	0.121128028	0.923922314	ENSG00000159314	ARHGAP27
18.69719394	0.113231004	1.123629983	0.100772502	0.919731056	0.999527751	ENSG00000231924	PSG1
18.32917717	-0.48211785	0.766418114	-0.629053308	0.529314157	0.99483168	ENSG00000259943	AL050341.2
18.23137313	0.12574044	0.626899713	0.200575048	0.84103087	0.999467288	ENSG00000230082	PRRT3-AS1
18.16524628	-0.036692339	0.786138251	-0.046674156	0.962772928	0.999527751	ENSG00000184343	SRPK3
18.46983586	1.205554015	0.722039171	1.669651818	0.094988272	0.921147998	ENSG00000275004	ZNF280B
17.61925472	0.630900031	0.883202946	0.714331891	0.475021976	0.99135833	ENSG00000184792	OSBP2
18.29109708	1.791857069	0.703074887	2.548600585	0.010815609	0.638240244	ENSG00000225313	AL513327.1
18.15891672	-0.165892212	0.676739781	-0.245134418	0.806352354	0.999467288	ENSG00000187068	C3orf70
18.45114422	0.921467386	0.7914781	1.164236112	0.244328339	0.962465655	ENSG00000269821	KCNQ1OT1
19.56304284	-0.491605892	0.93395895	-0.526367773	0.598632703	0.99483168	ENSG00000085741	WNT11
18.27787166	0.274914135	0.64459452	0.426491579	0.669749683	0.997568624	ENSG00000151320	AKAP6
18.42993461	0.260443279	0.712897033	0.36533085	0.714864452	0.99900742	ENSG00000234769	WASH4P
18.38765578	-1.223163143	0.832217118	-1.46976446	0.141625558	0.935644597	ENSG00000248121	SMURF2P1
18.03935657	-0.944539706	0.663990724	-1.422519429	0.154875515	0.939432741	ENSG00000101353	MROH8
18.39140687	0.147695137	0.650296291	0.227119759	0.82033062	0.999467288	ENSG00000211643	IGLV5-52
18.64688222	-0.505848105	0.77635301	-0.651569709	0.514678794	0.993513219	ENSG00000203761	MSTO2P
18.03083561	0.684264957	0.660558928	1.035887833	0.300254474	0.972186492	ENSG00000174130	TLR6
17.97815071	-0.293759882	0.707600447	-0.415149373	0.678032558	0.998869317	ENSG00000272686	AC006333.2
17.76331581	0.728624032	0.821230341	0.887234671	0.37495257	0.982609538	ENSG00000181544	FANCB
18.06504194	0.12477078	0.704855195	0.177016188	0.859495674	0.999467288	ENSG00000179304	FAM156B
18.61286334	0.27344393	0.890438798	0.307088966	0.758775656	0.999467288	ENSG00000188153	COL4A5
18.30715845	0.275063046	0.672794201	0.408836826	0.682659412	0.998869317	ENSG00000215154	AC141586.1
18.11587929	0.600737895	0.720272546	0.834042472	0.404257051	0.98927221	ENSG00000161265	U2AF1L4
19.05559905	-0.569613589	1.062449278	-0.536132501	0.591866979	0.99483168	ENSG00000222022	AC112721.1
18.20795341	-0.133433635	0.955599568	-0.139633419	0.888949634	0.999467288	ENSG00000280219	AC093908.1
17.299322	0.759592824	1.15388345	0.658292503	0.510350195	0.993513219	ENSG00000203727	SAMD5
18.16954394	-0.548751675	0.690340783	-0.794899692	0.426671854	0.991301167	ENSG00000178665	ZNF713
17.17735005	1.876141486	1.19125742	1.57492533	0.115273681	0.923922314	ENSG00000146966	DENND2A
18.15020432	-0.983624409	0.646830817	-1.520682663	0.128339491	0.928627271	ENSG00000233270	SNRPEP4
18.07251055	-0.42289458	0.716655654	-0.590094529	0.555127277	0.99483168	ENSG00000196172	ZNF681
18.36923742	-0.360935097	0.82302136	-0.438548882	0.660988443	0.997568624	ENSG00000186230	ZNF749
17.86107663	0.25690113	0.788643821	0.325750514	0.744613122	0.999467288	ENSG00000186976	EFCAB6
18.12378509	0.230329564	0.798388024	0.28849326	0.772969189	0.999467288	ENSG00000231711	LINC00899
18.27917682	-1.208654369	0.785233981	-1.539228305	0.12374857	0.927794614	ENSG00000238042	LINC02257
18.2746288	-0.293123561	0.6687295	-0.438329042	0.661147776	0.997568624	ENSG00000253958	CLDN23
18.05243736	-1.641287048	1.448259842	-1.133282164	0.257095779	0.962465655	ENSG00000283162	AL390726.6
18.40454686	1.965269496	0.875114768	2.245727724	0.024721453	0.749756093	ENSG00000137496	IL18BP
18.44867323	1.230546481	0.929705647	1.323587186	0.185640183	0.949094286	ENSG00000119946	CNNM1
17.65280991	-0.009272272	0.764352214	-0.012130889	0.990321189	0.999527751	ENSG00000150667	FSIP1
17.38978908	-1.002182004	0.889609863	-1.126541021	0.259936587	0.962465655	ENSG00000232480	TGFB2-AS1
17.47818932	-0.629849367	0.874133838	-0.720541111	0.471191897	0.99135833	ENSG00000154553	PDLIM3
18.04144666	-1.991250652	1.041169162	-1.912514051	0.055810292	0.847598083	ENSG00000156427	FGF18
17.80922002	-0.522396793	0.727309555	-0.718259219	0.47259747	0.99135833	ENSG00000173535	TNFRSF10C
18.27579897	0.327776335	0.894955258	0.366248851	0.714179393	0.99900742	ENSG00000160588	MPZL3
18.38546304	-2.638044636	0.96083331	-2.745579912	0.006040405	0.499617804	ENSG00000188013	MEIS3P2
18.97256533	3.019029861	1.179358524	2.559891499	0.010470485	0.635400047	ENSG00000173110	HSPA6
18.29407047	-1.475542358	0.785906899	-1.877502742	0.060449222	0.858996918	ENSG00000106565	TMEM176B

18.27570724	-0.134592141	0.788862228	-0.170615522	0.864526092	0.999467288	ENSG00000253669	GASAL1
17.9695781	-0.242024332	0.684367571	-0.353646699	0.723603672	0.999456245	ENSG00000269940	AL049840.3
17.75918989	-0.473399968	0.66822084	-0.708448374	0.478666861	0.99135833	ENSG00000183208	GDPGP1
17.54194845	0.145723023	0.686177826	0.212369182	0.831819027	0.999467288	ENSG00000127774	EMC6
17.46185033	-0.062204721	0.760972946	-0.081743669	0.934850552	0.999527751	ENSG00000161955	TNFSF13
18.10640449	1.166910564	0.955278329	1.221539868	0.22188168	0.956884001	ENSG00000176208	ATAD5
17.90475943	-0.871157751	0.694477495	-1.254407462	0.209693942	0.954310328	ENSG00000183397	C19orf71
17.70202235	0.626478495	0.799297459	0.783786421	0.433165443	0.991301167	ENSG00000188167	TMPPE
18.14825539	0.423931343	0.725730397	0.584144394	0.559123169	0.99483168	ENSG00000128596	CCDC136
17.80147522	-0.961697345	0.821191452	-1.171100043	0.241558566	0.962465655	ENSG00000175611	LINC00476
17.67273894	-1.275708212	0.940756676	-1.356044813	0.17508491	0.946278819	ENSG00000177234	LINC01561
18.05717651	-0.556204664	0.755625709	-0.736084886	0.46167904	0.99135833	ENSG00000283103	AC010642.2
17.51355973	-1.383240593	1.061453557	-1.303156963	0.19252118	0.949261383	ENSG00000143494	VASH2
17.32790292	-0.133761141	0.822180191	-0.162690785	0.870761903	0.999467288	ENSG00000186854	TRABD2A
17.4072944	0.858292783	1.061891078	0.808268193	0.418936208	0.990226751	ENSG00000131730	CKMT2
16.8495661	0.194963673	1.171134889	0.16647414	0.867783833	0.999467288	ENSG00000075213	SEMA3A
17.69889249	0.99224185	0.717613306	1.382697118	0.166757756	0.943223078	ENSG00000230715	AC018638.2
17.9670258	0.022427767	0.767242399	0.029231657	0.976679833	0.999527751	ENSG00000186280	KDM4D
16.8749025	0.623317659	1.134119198	0.549605068	0.582590282	0.99483168	ENSG00000018236	CNTN1
18.10355232	0.212510515	0.928973781	0.228758356	0.819056736	0.999467288	ENSG00000197358	BNIP3P1
17.52224549	0.757410112	0.744420766	1.01744893	0.308939915	0.973427483	ENSG00000180229	HERC2P3
17.70566343	-1.27606895	0.843798634	-1.512290846	0.130459889	0.929373153	ENSG00000132522	GPS2
17.39799605	-0.201525325	0.784420818	-0.25690971	0.797248474	0.999467288	ENSG00000274828	AC068473.5
17.45263534	-0.539575332	0.71528326	-0.754351964	0.450637907	0.99135833	ENSG00000182584	ACTL10
17.54628874	-0.066834515	0.673899913	-0.099175728	0.920998746	0.999527751	ENSG00000237039	AC018738.1
18.04804408	1.433725703	0.854162921	1.678515501	0.093246507	0.921147998	ENSG00000273142	AC073335.2
17.39395708	-0.423974746	0.842418587	-0.503282753	0.61476549	0.99483168	ENSG00000254510	AP001107.5
17.21964315	0.590278994	0.778645499	0.758084384	0.448400468	0.99135833	ENSG00000232653	GOLGA8N
17.5852033	-0.743713076	0.710066632	-1.04738491	0.294922088	0.970706863	ENSG00000179044	EXOC3L1
18.07254855	0.57200747	1.339090368	0.427161216	0.669261908	0.997568624	ENSG00000161958	FGF11
17.51802518	0.399433814	0.658863179	0.606246982	0.544350759	0.99483168	ENSG00000185829	ARL17A
17.49692468	0.154283915	0.702115768	0.219741419	0.826072545	0.999467288	ENSG00000234912	SNHG20
17.73594102	0.469812678	0.707012504	0.664504058	0.506367753	0.993513219	ENSG00000225507	AC069282.1
17.30380061	0.40623931	0.699699276	0.580591296	0.56151594	0.99483168	ENSG00000280355	AL132656.4
17.17116237	0.959907979	0.665248446	1.442931562	0.149039751	0.936396974	ENSG00000244687	UBE2V1
17.7252797	-0.873809717	0.992803504	-0.880143667	0.378781486	0.982609538	ENSG00000235649	MXRA5Y
17.15977869	0.260100226	0.813977947	0.319542104	0.749315469	0.999467288	ENSG00000128262	POM121L9P
17.31192311	0.609788521	0.749048976	0.814083646	0.415597034	0.990226751	ENSG00000284968	AC093827.4
17.80804791	-0.353324415	0.839857447	-0.420695699	0.673977302	0.998869317	ENSG00000248362	AC011352.1
17.52374009	0.093324246	0.727404888	0.128297524	0.897913524	0.999467288	ENSG00000233117	LINC00702
17.61546755	-0.535613631	0.721841881	-0.742009636	0.458081497	0.99135833	ENSG00000197653	DNAH10
17.38587608	0.240380854	0.681841044	0.352546764	0.724428254	0.999456245	ENSG00000259319	AF111167.2
16.77817201	-0.362143543	1.092350523	-0.331526864	0.740246553	0.999467288	ENSG00000172183	ISG20
17.06878258	0.886335257	0.707253824	1.253206737	0.210130479	0.954310328	ENSG00000207561	MIR635
16.48412708	0.95716799	1.146963129	0.834523767	0.4039859	0.988927059	ENSG00000125965	GDF5
17.05306815	-0.390090048	0.802069636	-0.486354339	0.626715959	0.99483168	ENSG00000160321	ZNF208
17.63613692	1.073140132	0.7790718	1.37745986	0.168370117	0.943223078	ENSG00000267152	AC093227.1
17.10410202	-0.732582511	0.822665866	-0.890498246	0.37319841	0.982329093	ENSG00000128011	LRFN1
17.33046332	-0.093578995	0.734250898	-0.127448254	0.898585627	0.999490224	ENSG00000220785	MTMR9LP
16.85660999	-0.783829028	0.770546625	-1.017237637	0.309040396	0.973427483	ENSG00000227533	SLC2A1-AS1
17.52780195	0.243057706	0.721384422	0.336932291	0.736167943	0.999467288	ENSG00000144230	GPR17
18.06370627	0.315755865	0.816139418	0.386889615	0.698837929	0.998869317	ENSG00000145040	UCN2
17.04376106	0.85568043	0.87050107	0.982974588	0.325619946	0.976026345	ENSG00000118849	RARRES1
17.36732289	0.556817279	0.748759956	0.743652589	0.457086679	0.99135833	ENSG00000259959	AC107068.1
16.79205582	-0.110274335	0.723159178	-0.152489712	0.878800704	0.999467288	ENSG00000183199	HSP90AB3P
17.6719406	1.516292259	0.929065239	1.632062202	0.102666383	0.92169898	ENSG00000197083	ZNF300P1
17.28982899	-1.513774272	0.781534925	-1.936924664	0.052754553	0.847598083	ENSG00000275765	AC091982.3
17.32237874	-0.393382313	0.698199635	-0.563423831	0.573146313	0.99483168	ENSG00000268592	RAET1E-AS1
17.024704	-0.564188894	0.817103227	-0.690474441	0.489895878	0.993513219	ENSG00000226237	GAS1RR
17.28840102	0.64550208	0.757411911	0.852247067	0.394076978	0.988378925	ENSG00000236710	AC108448.2
17.49822283	0.234949015	0.821577426	0.285973065	0.774898751	0.999467288	ENSG00000166823	MESP1
17.55404032	0.492348481	0.840277985	0.585935237	0.557919036	0.99483168	ENSG00000125144	MT1G
17.2024189	0.183417526	0.902588551	0.203212777	0.838968726	0.999467288	ENSG00000214331	AC009053.1
16.79793771	0.285334153	0.814478411	0.35032746	0.726092959	0.999467288	ENSG00000228782	MRPL45P2

16.79672199	0.238221998	0.800719302	0.297509999	0.766077175	0.999467288	ENSG00000228340	MIR646HG
16.83615374	1.546514237	1.035333572	1.493735236	0.135244835	0.933741583	ENSG00000205835	GMNC
16.81265759	0.154119133	0.757954326	0.203335647	0.838872695	0.999467288	ENSG00000130429	ARPC1B
17.18032555	0.986562674	0.863898691	1.141988852	0.253458654	0.962465655	ENSG00000175832	ETV4
17.18441521	-0.222974814	0.679121993	-0.328328072	0.742663621	0.999467288	ENSG00000064547	LPAR2
16.57651367	-1.412920795	0.928988412	-1.520924025	0.128278904	0.928627271	ENSG00000231123	SPATA20P1
16.93022633	0.784418606	1.064569989	0.736840803	0.461219166	0.99135833	ENSG00000189410	SH2D5
16.76809901	0.619761261	0.729701068	0.849335829	0.395694449	0.988378925	ENSG00000114631	PODXL2
17.47091112	-0.650328903	0.760759602	-0.854841531	0.392638883	0.988378925	ENSG00000214050	FBXO16
17.10562835	0.433365051	0.667286531	0.649443726	0.516051609	0.993513219	ENSG00000177076	ACER2
17.10178229	-0.770883341	0.746059788	-1.033272874	0.301476218	0.972793733	ENSG00000255508	AP002990.1
17.21887624	1.534848483	0.763410993	2.010513991	0.044376817	0.809260564	ENSG00000162194	LBHD1
15.32211056	1.391557823	1.295521572	1.074129411	0.282764684	0.963267117	ENSG00000166106	ADAMTS15
17.24341822	0.175238565	0.757745206	0.231263178	0.817110351	0.999467288	ENSG00000089505	CMTM1
17.15673875	0.96654907	0.851054572	1.135707512	0.256078996	0.962465655	ENSG00000197124	ZNF682
16.72022247	0.604825006	0.751275737	0.805063943	0.420782782	0.991301167	ENSG00000213080	AL354714.2
17.17594166	-1.307410242	0.720919862	-1.813530617	0.069750021	0.878035218	ENSG00000250461	AC122718.1
17.11930181	0.140656326	0.691557476	0.203390652	0.838829705	0.999467288	ENSG00000232389	AL583856.1
17.05504412	0.247786676	0.744059247	0.333020088	0.739119119	0.999467288	ENSG00000146250	PRSS35
17.13210454	-0.034896549	0.778999081	-0.044796649	0.964269396	0.999527751	ENSG00000146677	AC004453.1
17.58611721	0.006406676	1.065361512	0.006013617	0.995201857	0.999527751	ENSG00000148948	LRRC4C
17.24590952	0.057613746	0.701853727	0.082087968	0.934576761	0.999527751	ENSG00000135407	AVIL
17.27979683	-0.437865193	0.834566744	-0.524661683	0.599818398	0.99483168	ENSG00000136371	MTHFS
17.24169227	-0.154255397	0.864658822	-0.178400304	0.85840861	0.999467288	ENSG00000285043	AC093512.2
16.90837409	-0.12644187	0.702917578	-0.179881502	0.857245597	0.999467288	ENSG00000108786	HSD17B1
16.87070509	-0.179168822	0.869991251	-0.205943246	0.836835244	0.999467288	ENSG00000134242	PTPN22
16.90099149	0.084776643	0.684391703	0.123871524	0.901417	0.999527751	ENSG00000162755	KLHDC9
15.98627331	-0.242068587	1.292132124	-0.187340429	0.851393726	0.999467288	ENSG00000232788	ITGA6-AS1
17.14235904	2.088600379	1.37025001	1.524247665	0.127446855	0.928627271	ENSG00000035499	DEPDC1B
17.92640744	1.272413489	1.135174255	1.120897064	0.262331681	0.962465655	ENSG00000247627	MTND4P12
17.55957604	0.169154372	0.872167138	0.193947197	0.846217228	0.999467288	ENSG00000279821	AC145098.2
17.187757	-0.543669927	0.910891565	-0.596854717	0.550604382	0.99483168	ENSG00000204052	LRRC73
16.83575855	-0.906219829	0.82725877	-1.095449045	0.273319957	0.962465655	ENSG00000255135	AP002360.1
16.57822767	-0.659001094	0.839585105	-0.784912797	0.432504698	0.991301167	ENSG00000248265	FLJ12825
17.21700438	-0.129366393	0.711030209	-0.181942189	0.855628095	0.999467288	ENSG00000160207	HSF2BP
16.49197827	2.732177654	0.828147799	3.299142565	0.000969806	0.228234741	ENSG00000198914	POU3F3
17.2142389	0.064252557	0.832578011	0.077173017	0.938485907	0.999527751	ENSG00000170356	OR2A20P
17.30550408	-0.514094235	0.768459482	-0.668993287	0.50349976	0.993513219	ENSG00000215374	FAM66B
16.38708974	2.053106868	0.9768909	2.101674677	0.035581783	0.785058983	ENSG00000175305	CCNE2
16.39133582	0.634903754	0.806931832	0.78681213	0.431391852	0.991301167	ENSG00000246273	SBF2-AS1
16.30330151	-0.924069812	1.041289597	-0.887428257	0.374848376	0.982609538	ENSG00000133321	RARRES3
17.30132349	-0.732852921	1.039411043	-0.705065553	0.480769442	0.991417002	ENSG00000196932	TMEM26
17.51880738	-0.151564186	0.89661568	-0.169040303	0.86576494	0.999467288	ENSG00000178401	DNAJC22
16.96047893	0.777288723	0.878738189	0.884550976	0.376398857	0.982609538	ENSG00000215302	AC127502.1
16.96436837	-0.604933612	0.722117672	-0.837721655	0.402187048	0.988592117	ENSG00000283538	AC005972.3
16.6186755	0.129399502	0.90521741	0.142948535	0.886330827	0.999467288	ENSG00000226287	TMEM191A
16.49521796	-0.174552364	0.791570235	-0.220514057	0.825470826	0.999467288	ENSG00000186409	CCDC30
16.81563819	1.117418749	0.722632783	1.546316158	0.122028231	0.923922314	ENSG00000183891	TTC32
17.1335485	0.208914056	0.702521929	0.297377274	0.766178492	0.999467288	ENSG00000112877	CEP72
16.54957551	0.675050452	0.721577291	0.935520644	0.34952005	0.977691382	ENSG00000197536	C5orf56
17.04488158	-0.172416791	0.719486969	-0.239638518	0.810610502	0.999467288	ENSG00000164627	KIF6
17.35210677	0.447552188	0.871847733	0.51333756	0.607715214	0.99483168	ENSG00000157927	RADIL
16.76154913	-0.383745179	0.740360946	-0.518321747	0.604233802	0.99483168	ENSG00000136244	IL6
16.70010051	-0.604724971	1.014312117	-0.596192199	0.551046837	0.99483168	ENSG00000197576	HOXA4
17.24342154	2.2311236	1.036304278	2.152961873	0.031321675	0.775963115	ENSG00000271533	Z83843.1
17.15615097	-0.539788567	0.830773989	-0.649741777	0.515859034	0.993513219	ENSG00000165121	AL353743.1
16.3466775	1.254360055	1.200641771	1.044741308	0.296142547	0.970706863	ENSG00000186081	KRT5
16.60508904	-0.396683687	0.720291448	-0.550726637	0.581821082	0.99483168	ENSG00000277782	AC068870.2
16.69659486	-0.68159844	0.731485362	-0.931800519	0.351439627	0.977691382	ENSG00000267248	AC025048.2
16.5456271	-0.153104294	0.747106149	-0.204929774	0.837626992	0.999467288	ENSG00000079435	LIPE
16.99730364	-0.95345159	0.810163515	-1.176863154	0.239250131	0.962465655	ENSG00000127903	ZNF835
16.98150071	0.553029975	0.738078102	0.749283814	0.453686161	0.99135833	ENSG00000272977	AL008721.2
16.17573742	0.064816962	0.78644215	0.082417965	0.934314351	0.999527751	ENSG00000003400	CASP10
16.90704287	1.744734764	0.828763705	2.105225836	0.035271654	0.784512596	ENSG00000206530	CFAP44

16.55867127	1.007482798	0.761162643	1.323610409	0.185632466	0.949094286	ENSG00000229334	AC046143.1
16.86403882	-0.118784395	0.813518976	-0.14601306	0.883911078	0.999467288	ENSG00000135605	TEC
16.66969604	-0.593867836	0.796742078	-0.745370243	0.456047928	0.99135833	ENSG00000197465	GYPE
16.69847843	1.058517972	0.908326562	1.165349574	0.24387752	0.962465655	ENSG00000172179	PRL
15.90104556	0.006752657	0.814461843	0.008290943	0.99338486	0.999527751	ENSG00000188763	FZD9
16.60888036	0.441989738	0.790723031	0.558969096	0.576182812	0.99483168	ENSG00000008056	SYN1
15.89548624	1.019483699	1.064515343	0.957697515	0.33821531	0.977691382	ENSG00000107611	CUBN
17.3226243	-1.387240662	1.294640453	-1.071525812	0.283933071	0.963310634	ENSG00000272636	DOC2B
16.11776375	-0.563729992	1.33161417	-0.423343341	0.672044777	0.997568624	ENSG00000264301	LINC01444
16.83995196	-0.138729685	0.752421155	-0.184377704	0.85371716	0.999467288	ENSG00000197714	ZNF460
17.05126731	-0.596203988	1.015039662	-0.58737014	0.556955144	0.99483168	ENSG00000084710	EFR3B
16.70845808	0.523269228	0.901010426	0.580758238	0.561403405	0.99483168	ENSG00000173531	MST1
16.86852692	1.153977457	0.831163719	1.388387669	0.165019029	0.943223078	ENSG00000254996	ANKHD1-EIF4EBP3
16.61890162	0.526043818	0.828919896	0.634613575	0.525680473	0.99483168	ENSG00000247679	AC139795.2
16.41296852	-0.846865424	0.694918729	-1.218653906	0.22297558	0.956884001	ENSG00000233225	AC004987.2
15.9968289	0.263411699	0.907050756	0.290404585	0.771506736	0.999467288	ENSG00000233554	B4GALT1-AS1
16.56202875	0.241405527	0.762171209	0.316733989	0.751445456	0.999467288	ENSG00000169508	GPR183
16.5064644	-0.201628788	0.69099605	-0.291794414	0.770443816	0.999467288	ENSG00000157429	ZNF19
16.2883825	0.188067592	0.920174365	0.20438256	0.838054558	0.999467288	ENSG00000173894	CBX2
16.09428343	1.806819908	0.940842755	1.920427082	0.054803975	0.847598083	ENSG00000166396	SERPINB7
16.76948006	0.218107644	0.821691051	0.265437532	0.790672427	0.999467288	ENSG00000178093	TSSK6
16.21837557	-0.011266304	0.839448223	-0.013421083	0.989291847	0.999527751	ENSG00000241990	PRR34-AS1
16.27365107	-0.820465933	0.900110317	-0.911517085	0.362022988	0.979465649	ENSG00000198286	CARD11
16.91670057	-0.87430659	0.837182044	-1.04434465	0.296325962	0.970706863	ENSG00000230844	ZNF674-AS1
16.38557647	0.187166817	0.828691754	0.225858186	0.82131172	0.999467288	ENSG00000196741	LINC01560
16.25871747	-0.006434732	0.700138913	-0.00919065	0.992667025	0.999527751	ENSG00000230074	AL162231.2
16.75788347	-0.670429042	0.79764905	-0.840506288	0.400624578	0.988492517	ENSG00000269609	RPARP-AS1
16.62063958	0.200483395	0.905595268	0.221382998	0.82479423	0.999467288	ENSG00000250899	AC125807.2
16.40019853	-0.230253088	0.832316186	-0.276641367	0.782055497	0.999467288	ENSG00000256594	AC010186.2
16.59912577	-1.051249279	0.788754323	-1.332796852	0.182598479	0.949094286	ENSG00000255737	AGAP2-AS1
16.26178428	0.014063163	0.774744031	0.018152013	0.985517585	0.999527751	ENSG00000100902	PSMA6
16.59356967	-0.349478846	0.780915574	-0.447524493	0.654496408	0.997568624	ENSG00000214401	KANSL1-AS1
16.97468713	0.383798003	0.908556616	0.422426073	0.672714051	0.998254293	ENSG00000141837	CACNA1A
16.33233857	-1.893315208	1.002733862	-1.888153258	0.059005379	0.853473128	ENSG00000187556	NANOS3
16.25603897	0.905822437	0.67758477	1.336840018	0.181274863	0.949094286	ENSG00000135951	TSGA10
16.3166947	0.985838042	0.7214792	1.366412283	0.171809595	0.944422693	ENSG00000261468	AC096921.2
16.26900109	-0.123554302	0.726777261	-0.170002982	0.865007792	0.999467288	ENSG00000174353	STAG3L3
16.13668905	-1.065182145	0.870922936	-1.223049826	0.221310878	0.956884001	ENSG00000275964	AL355001.2
17.60547965	-0.757942753	1.205267615	-0.628858474	0.529441714	0.99483168	ENSG00000102683	SGCG
16.51546339	0.257559513	0.836526487	0.307891642	0.758164786	0.999467288	ENSG00000267414	AC120049.1
16.13804147	1.503459662	0.799650248	1.880146558	0.060088107	0.858996918	ENSG00000198342	ZNF442
16.3015315	-0.795059345	0.693925054	-1.145742383	0.251901761	0.962465655	ENSG00000238142	BX284668.5
16.248993	1.524883994	0.695477067	2.192572646	0.028338184	0.769339074	ENSG00000228409	CCT6P1
16.61196743	0.902179401	0.783489566	1.151488724	0.249531232	0.962465655	ENSG00000149548	CCDC15
15.90036493	-0.764180464	0.802898693	-0.951776944	0.341210117	0.977691382	ENSG00000257605	AC073611.1
16.19425694	-0.373927753	0.842755299	-0.44369671	0.657261874	0.997568624	ENSG00000133116	KL
16.29014797	-1.098817922	0.71974675	-1.526672989	0.126842347	0.928627271	ENSG00000258655	ARHGAP5-AS1
16.44448682	-0.173424484	0.798779224	-0.217111911	0.828121132	0.999467288	ENSG00000205771	CATSPER2P1
16.32725779	0.094966011	0.872601029	0.108830964	0.913336564	0.999527751	ENSG00000228695	CES1P1
16.56470765	0.548506618	0.740498004	0.740726667	0.458859187	0.99135833	ENSG00000267254	ZNF790-AS1
16.92617323	-0.422674687	0.920135007	-0.459361598	0.64597452	0.997368416	ENSG00000187474	FPR3
16.00409069	-0.40932562	0.934857817	-0.437847994	0.661496477	0.997568624	ENSG00000040608	RTN4R
16.33318464	-0.5342824	0.74081967	-0.721204392	0.47078377	0.99135833	ENSG00000143248	RGS5
16.2146688	0.43441543	0.703029588	0.617919128	0.53662865	0.99483168	ENSG00000151687	ANKAR
16.04045892	0.063169446	0.983704816	0.064215855	0.948798353	0.999527751	ENSG00000280241	AC079298.3
16.29982428	0.48167563	0.689273449	0.698816458	0.484666742	0.991892545	ENSG00000249485	RBBP4P1
16.18372125	-0.52211644	0.668125113	-0.781465073	0.434529011	0.991301167	ENSG00000279483	AC090498.1
16.4197924	0.812646958	0.694024899	1.170919026	0.241631327	0.962465655	ENSG00000179071	CCDC89
16.17410291	0.999350245	0.781915641	1.278079363	0.201221444	0.954310328	ENSG00000244733	AL132656.2
15.82613848	-1.01138121	0.747404724	-1.353190818	0.175994678	0.946278819	ENSG00000260086	AC007611.1
15.28996982	1.206215172	1.084769298	1.111955486	0.26615729	0.962465655	ENSG00000141750	STAC2
16.54690859	-0.998253182	0.774197092	-1.289404458	0.197257512	0.954310328	ENSG00000157578	LCA5L
15.94084169	0.589329057	0.854027546	0.690058605	0.490157334	0.993513219	ENSG00000152760	TCTEX1D1
15.96217677	0.395803197	0.709988989	0.557477938	0.577200929	0.99483168	ENSG00000277406	SEC22B4P

15.89702918	0.138092006	0.76794906	0.179819226	0.85729449	0.999467288	ENSG00000225265	TAF1A-AS1
15.51602391	-0.513575064	0.96882785	-0.530099402	0.596043014	0.99483168	ENSG00000179528	LBX2
15.98857243	0.554996541	0.680213853	0.815914787	0.414548874	0.990226751	ENSG00000196597	ZNF782
15.83879916	-0.229959844	0.785214119	-0.292862594	0.769627182	0.999467288	ENSG00000176894	PXMP2
16.30416762	0.058364505	0.724788237	0.080526286	0.935818691	0.999527751	ENSG00000273679	AC100757.1
15.86672802	0.388902904	0.79427869	0.489630288	0.62439554	0.99483168	ENSG00000172345	STARD5
16.26620051	0.148495195	0.8163145	0.181909295	0.85565391	0.999467288	ENSG00000259728	LINC00933
16.03491528	0.983372694	0.781837643	1.257770974	0.208474596	0.954310328	ENSG00000140993	TIGD7
16.27495957	-0.739303376	1.033383321	-0.715420271	0.47434939	0.99135833	ENSG00000064300	NGFR
15.72181046	0.201720349	0.814169182	0.247762201	0.804318402	0.999467288	ENSG00000160051	IQCC
15.80681553	-0.302926533	0.904249992	-0.335003081	0.737622762	0.999467288	ENSG00000231770	TMEM44-AS1
16.12933629	0.242568319	0.78120147	0.310506737	0.756175637	0.999467288	ENSG00000130997	POLN
15.93630848	-1.157753455	0.984663727	-1.175785625	0.239680552	0.962465655	ENSG00000095587	TLL2
16.12439396	-0.096425803	0.685099759	-0.140747098	0.888069737	0.999467288	ENSG00000250133	HOXC-AS2
15.73450193	1.030881913	0.806217449	1.278664849	0.201015103	0.954310328	ENSG00000280120	AC073857.1
16.20413595	1.052487853	0.772391916	1.362634475	0.172997738	0.944422693	ENSG00000229152	ANKRD10-IT1
15.86908461	0.170711694	0.994285418	0.171692847	0.863679009	0.999467288	ENSG00000132639	SNAP25
16.28204756	-0.381317797	0.794982026	-0.479655873	0.63147211	0.99483168	ENSG00000276975	HYDIN2
15.48729452	0.178819659	0.771022319	0.231925399	0.816595957	0.999467288	ENSG00000115138	POMC
15.94807821	-0.197884334	0.689780035	-0.286880344	0.774203942	0.999467288	ENSG00000246560	AC018797.2
15.90558553	-0.252857365	0.873039514	-0.289628775	0.772100251	0.999467288	ENSG00000204618	RNF39
15.56432082	0.000318778	0.788968104	0.000404044	0.99967762	0.9998586	ENSG00000171631	P2RY6
15.34083257	0.425737528	0.81914878	0.519731626	0.603250641	0.99483168	ENSG00000150672	DLG2
15.99985016	-0.605478337	0.738234155	-0.820171125	0.41211856	0.990226751	ENSG00000214562	NUTM2D
15.45286986	-0.567648076	0.984096846	-0.576821355	0.564060142	0.99483168	ENSG00000214900	LINC01588
15.50615602	-1.409036473	0.977818566	-1.440999918	0.149584706	0.936396974	ENSG00000248079	DPH6-DT
15.48568518	-0.486907608	0.77192443	-0.630771082	0.528190214	0.99483168	ENSG00000285230	RALY-AS1
16.23690131	0.820136045	0.877763499	0.934347402	0.35012472	0.977691382	ENSG00000272872	AP000525.1
16.0871203	0.743853016	0.909430803	0.817932507	0.413395728	0.990226751	ENSG00000279519	AC007382.1
15.85359555	0.15834861	0.696955669	0.227200404	0.820267913	0.999467288	ENSG00000157152	SYN2
15.56240748	0.4659542	0.770515317	0.604730613	0.545358004	0.99483168	ENSG00000152953	STK32B
15.89258867	1.210460596	0.859675487	1.408043634	0.15911815	0.939523519	ENSG00000253251	SHLD3
15.62898283	0.660757393	0.736016569	0.897747986	0.369319921	0.981455968	ENSG00000080007	DDX43
15.89946124	-0.054306093	0.772352856	-0.070312542	0.9439449	0.999527751	ENSG00000272655	POLR2J4
16.87504697	-0.425529261	1.182945585	-0.359720063	0.719056487	0.99900742	ENSG00000078114	NEBL
15.76828424	-0.617697731	0.697071944	-0.886131964	0.37554642	0.982609538	ENSG00000229932	YWHAZP3
15.59515094	-0.024099325	0.791405523	-0.030451297	0.975707135	0.999527751	ENSG00000259032	ENSAP2
15.93995269	-1.186138788	1.167445042	-1.016012528	0.309623421	0.973427483	ENSG00000274370	AC130371.2
15.72680794	0.133811482	0.773349723	0.173028422	0.862629087	0.999467288	ENSG00000215386	MIR99AHG
15.99686244	-0.998439251	0.747469131	-1.335759845	0.181627782	0.949094286	ENSG00000177692	DNAJC28
16.7495831	3.707917912	1.218778334	3.042323456	0.002347595	0.355371917	ENSG00000228560	AL513323.1
16.25199393	1.157981389	0.940202552	1.231629702	0.218087441	0.956884001	ENSG00000235363	SNRPGP10
15.7926893	-1.926558204	1.095200429	-1.759091901	0.0785619	0.897052166	ENSG00000168427	KLHL30
15.81539755	0.762699468	0.780513316	0.977176753	0.328481655	0.976026345	ENSG00000216937	CCDC7
15.83931982	0.532771906	0.678930705	0.784722066	0.432616542	0.991301167	ENSG00000276805	AL133216.2
15.77191638	-0.813034068	0.84687539	-0.960039786	0.337035192	0.977691382	ENSG00000166145	SPINT1
15.85535672	0.713975971	0.872975489	0.817864854	0.413434361	0.990226751	ENSG00000196689	TRPV1
16.04216342	0.23608116	0.808752956	0.291907632	0.770357248	0.999467288	ENSG00000277534	AC007996.1
15.42976854	-0.483097882	0.879539721	-0.549262155	0.582825555	0.99483168	ENSG00000186564	FOXD2
15.648366	-1.540282235	0.821092336	-1.875894059	0.060669829	0.858996918	ENSG00000135744	AGT
15.71234153	-0.254208378	0.810331031	-0.313709297	0.753741839	0.999467288	ENSG00000233230	AC079807.1
15.28780253	0.44453191	0.838463576	0.530174385	0.595991029	0.99483168	ENSG00000137463	MGARP
15.25943728	0.366368388	0.797183051	0.459578748	0.645818616	0.997368416	ENSG00000204956	PCDHGA1
15.59557842	-0.432893388	1.440790405	-0.300455491	0.763829742	0.999467288	ENSG00000235385	LINC02154
15.52407285	1.780638532	1.140778142	1.560898186	0.118547775	0.923922314	ENSG00000137135	ARHGEF39
14.86335577	0.092991346	1.286274047	0.072295127	0.942367042	0.999527751	ENSG00000184350	MRGPRE
15.58076604	0.600181079	0.711473	0.843575342	0.398906748	0.988378925	ENSG00000280385	AP000648.3
15.63933667	-0.168859074	0.732437794	-0.230543911	0.817669146	0.999467288	ENSG00000227268	KLLN
15.29082065	-0.530930117	0.894351959	-0.593647849	0.552747671	0.99483168	ENSG00000259295	CSPG4P12
14.52883535	-0.652092945	3.011286424	-0.216549625	0.828559348	0.999467288	ENSG00000116014	KISS1R
15.37941884	0.031335192	0.750766767	0.041737586	0.966707891	0.999527751	ENSG00000232882	PHKA1P1
15.78760177	-0.029126005	0.937603855	-0.031064297	0.975218263	0.999527751	ENSG00000223396	RPS10P7
16.24385568	-0.598073568	0.939601242	-0.636518494	0.52443853	0.99483168	ENSG00000163803	PLB1
15.88837782	-0.669714592	1.107691596	-0.604603839	0.545442255	0.99483168	ENSG00000164128	NPY1R

15.55282295	-0.097600157	0.756106997	-0.129082467	0.897292395	0.999467288	ENSG00000130584	ZBTB46
15.70016039	0.055120843	0.734984903	0.074995885	0.940217986	0.999527751	ENSG00000058085	LAMC2
15.24023248	1.093398533	0.779633699	1.402451605	0.160780432	0.942049406	ENSG00000234072	AC074117.1
16.23584516	-0.396456507	1.010653211	-0.392277492	0.694853192	0.998869317	ENSG00000174963	ZIC4
15.0921793	-1.474308344	0.862924071	-1.708502976	0.087543055	0.906368702	ENSG00000239857	GET4
15.61621844	0.753757855	0.844847561	0.892182082	0.372295345	0.98211172	ENSG00000272604	AC073073.2
16.01277779	2.296942515	1.047389357	2.193016856	0.028306165	0.769339074	ENSG00000270607	AC009549.1
15.36610375	0.072958929	0.80826249	0.09026638	0.928075536	0.999527751	ENSG00000160172	FAM86C2P
15.165314	1.136763347	0.865176065	1.313909841	0.188876546	0.949094286	ENSG00000156398	SFXN2
15.35577809	1.316898546	0.799345876	1.647470245	0.099461427	0.921147998	ENSG00000135119	RNFT2
15.49308551	-0.729222054	0.743451112	-0.980860803	0.326661389	0.976026345	ENSG00000165807	PPP1R36
15.19873031	2.265628333	1.071839622	2.1137755	0.034534446	0.783572981	ENSG00000133985	TTC9
15.48709974	-0.791543526	0.838077163	-0.944475713	0.344926607	0.977691382	ENSG00000108839	ALOX12
15.87600148	1.417300722	1.13231715	1.25168176	0.210685849	0.954310328	ENSG00000260075	NSFP1
15.22371839	0.463655615	0.70223425	0.660257762	0.50908843	0.993513219	ENSG00000108622	ICAM2
15.77488164	-0.0144382	0.705312514	-0.020470642	0.983667931	0.999527751	ENSG00000196724	ZNF418
15.15592455	2.615691788	1.535954583	1.702974695	0.088572804	0.910413547	ENSG00000179750	APOBEC3B
14.95398796	-0.663889855	0.92546756	-0.71735616	0.473154363	0.99135833	ENSG00000170498	KISS1
15.43685989	-0.90350677	0.815690312	-1.107659066	0.268009084	0.962465655	ENSG00000224152	AC009506.1
15.15023643	-0.078474077	0.868810561	-0.090323576	0.928030086	0.999527751	ENSG00000198846	TOX
15.31902139	-1.186684465	0.766437619	-1.548311873	0.121547227	0.923922314	ENSG00000245105	A2M-AS1
15.48164168	0.255640884	0.827040166	0.309103347	0.757242913	0.999467288	ENSG00000169783	LINGO1
15.30160969	0.719537415	0.799704019	0.899754657	0.36825083	0.981269214	ENSG00000136404	TM6SF1
15.84147923	-0.072001198	0.893302956	-0.080601097	0.935759194	0.999527751	ENSG00000266053	NDUFV2-AS1
15.5152624	-1.070241058	1.121629426	-0.954184184	0.339990419	0.977691382	ENSG00000101280	ANGPT4
15.55287172	0.168875724	0.758130814	0.22275275	0.823727944	0.999467288	ENSG00000115598	IL1RL2
15.36227823	-0.159241823	0.863265756	-0.184464427	0.853649132	0.999467288	ENSG00000114859	CLCN2
15.35449311	0.685899271	0.749932575	0.914614586	0.360393999	0.979465649	ENSG00000180481	GLIPR1L2
15.30323798	1.667179484	0.869458698	1.917491293	0.055175546	0.847598083	ENSG00000245849	RAD51-AS1
15.34962502	-0.429928411	0.785748561	-0.547157745	0.584270363	0.99483168	ENSG00000215067	ALOX12-AS1
15.10060845	-2.472772584	0.860198682	-2.874652841	0.00404472	0.45638972	ENSG00000105699	LSR
14.9731259	-0.998901786	1.0094879	-0.989513382	0.322412026	0.975337095	ENSG00000234465	PINLYP
15.05219742	0.64055004	0.975859807	0.656395555	0.51156965	0.993513219	ENSG00000143816	WNT9A
15.04315727	0.426126577	0.950899057	0.448130192	0.65405924	0.997527562	ENSG00000198885	ITPRIPL1
15.03414933	0.105217496	0.717756536	0.146592181	0.883453926	0.999467288	ENSG00000145217	SLC26A1
15.39998957	-1.62852311	0.870676463	-1.870411318	0.061426722	0.864834166	ENSG00000244459	AC147067.1
14.76106141	-0.458017472	0.913178081	-0.501564243	0.615974077	0.99483168	ENSG00000242715	CCDC169
14.9974587	1.448678743	0.936040306	1.547667054	0.121702478	0.923922314	ENSG00000102524	TNFSF13B
15.39940617	1.684772137	1.110902165	1.516580119	0.129372728	0.928627271	ENSG00000154920	EME1
15.35699582	-0.277589974	1.026797359	-0.270345431	0.786894517	0.999467288	ENSG00000267480	AP001542.3
15.16473334	0.203129426	0.764763324	0.265610837	0.790538939	0.999467288	ENSG00000267169	AC022098.1
15.25437588	0.285835789	0.711030385	0.402002214	0.687682397	0.998869317	ENSG00000099338	CATSPERG
15.2899735	0.203968239	0.81279643	0.250946278	0.801855644	0.999467288	ENSG00000230513	THAP7-AS1
15.54099216	1.423336254	1.266174464	1.124123329	0.26096071	0.962465655	ENSG00000188610	FAM72B
14.83439538	1.195709172	0.820487828	1.457314942	0.145029464	0.935644597	ENSG00000221944	TIGD1
15.16284885	-1.091472744	0.780705788	-1.398058987	0.162095354	0.943223078	ENSG00000249129	SUDS3P1
15.11241701	-1.410531596	1.043486664	-1.351748561	0.176455765	0.946278819	ENSG00000155093	PTPRN2
15.20929064	1.135409991	0.777795268	1.459780019	0.144350542	0.935644597	ENSG00000178301	AQP11
15.59668129	2.33779001	0.988996887	2.363799158	0.018088616	0.726053908	ENSG00000197978	GOLGA6L9
14.60685795	0.862668219	1.067636824	0.808016546	0.419081057	0.990226751	ENSG00000140538	NTRK3
14.90981729	1.312826098	0.742160941	1.768923726	0.076906604	0.897052166	ENSG00000178852	EFCAB13
15.15016461	2.129852264	0.957113875	2.225286165	0.026062034	0.762934669	ENSG00000233695	GAS6-AS1
15.11679113	-0.510341257	0.798648952	-0.63900573	0.522819204	0.994616524	ENSG00000279495	AL928654.4
15.50009762	2.151367718	0.857703457	2.508288501	0.012131757	0.648356243	ENSG00000108465	CDK5RAP3
15.33365096	1.345411671	0.987736125	1.362116498	0.173161122	0.944422693	ENSG00000267470	ZNF571-AS1
15.25934701	-0.49102749	0.783634075	-0.626603036	0.530919478	0.99483168	ENSG00000228106	AL392172.1
14.70208943	-0.916452437	0.918568201	-0.997696672	0.318426468	0.974768742	ENSG00000163064	EN1
14.94052535	0.489453493	0.728305293	0.672044399	0.501555431	0.993513219	ENSG00000181722	ZBTB20
15.15863836	0.259430696	0.817417838	0.317378314	0.75095656	0.999467288	ENSG00000196782	MAML3
14.6909963	0.429304784	0.984456259	0.436083148	0.662776404	0.997568624	ENSG00000184408	KCND2
14.90876643	-0.10316952	0.816259203	-0.126393086	0.899420775	0.999527751	ENSG00000165272	AQP3
15.12384251	-0.683353594	1.305932197	-0.523268815	0.600787199	0.99483168	ENSG00000224513	AC109309.1
15.18205524	0.553421002	0.972898385	0.568837415	0.569466481	0.99483168	ENSG00000177990	DPY19L2
14.61059012	-0.231741675	1.219200487	-0.190076757	0.849248983	0.999467288	ENSG00000165474	GJB2

14.90193711	0.608280234	0.906033996	0.671365795	0.501987529	0.993513219	ENSG00000255769	GOLGA2P10
14.84156944	-0.51231467	0.803058762	-0.63795415	0.523503527	0.99483168	ENSG00000166246	C16orf71
14.97157026	-0.540344843	0.811789469	-0.665621892	0.50565281	0.993513219	ENSG00000267745	AC060766.7
15.10229362	0.988377958	0.892140175	1.10787294	0.267916694	0.962465655	ENSG00000262001	DLGAP1-AS2
14.41498839	0.366174758	0.861128369	0.425226681	0.670671436	0.997568624	ENSG00000268895	A1BG-AS1
14.56796968	-0.973793067	0.837996313	-1.162049345	0.245215422	0.962465655	ENSG00000267858	MZF1-AS1
14.90646148	-0.451262059	0.878221856	-0.513836061	0.607366614	0.99483168	ENSG00000225544	AC245452.2
14.96286083	0.821503207	0.783706245	1.048228482	0.294533351	0.970706863	ENSG00000273145	BX537318.1
14.61410421	-0.001549978	0.769820743	-0.002013428	0.998393518	0.999527751	ENSG00000143119	CD53
14.74019186	0.513129136	0.796093116	0.644559193	0.519212887	0.993513219	ENSG00000269982	AC018809.2
14.68982869	-0.20870908	0.744576357	-0.280305812	0.779242892	0.999467288	ENSG00000246174	KCTD21-AS1
14.61889585	-0.135511797	0.752643224	-0.180047854	0.857115	0.999467288	ENSG00000182912	TSPEAR-AS2
14.54105702	0.038495847	0.856166588	0.044963033	0.964136774	0.999527751	ENSG00000154822	PLCL2
15.36367271	0.6698327	1.146167095	0.584411037	0.558943803	0.99483168	ENSG00000067840	PDZD4
14.62491761	0.381078939	0.940314039	0.405267734	0.685280721	0.998869317	ENSG00000154760	SLFN13
13.99785283	-0.065944531	1.317455804	-0.050054454	0.960078994	0.999527751	ENSG00000186765	FSCN2
14.93558022	1.582667291	0.857731036	1.845178995	0.065011505	0.868202292	ENSG00000215424	MCM3AP-AS1
14.58802549	0.244591868	0.771470382	0.317046349	0.751208433	0.999467288	ENSG00000115239	ASB3
14.24581233	-0.163191892	0.819375882	-0.199166092	0.842132823	0.999467288	ENSG00000010030	ETV7
14.7035836	1.419035107	0.901289406	1.574450002	0.115383452	0.923922314	ENSG00000229124	VIM-AS1
15.03698713	1.860709789	0.853077576	2.181173015	0.029170622	0.773201806	ENSG00000163463	KRTCAP2
14.57289505	0.205487296	0.766434296	0.268108169	0.788616058	0.999467288	ENSG00000174928	C3orf33
14.68307349	-0.934612625	0.803094473	-1.163764235	0.24451957	0.962465655	ENSG00000214192	UBE2V1P2
14.28076984	-0.916127274	0.855897082	-1.070370835	0.284452422	0.963310634	ENSG00000250986	LINC02600
14.42578839	-0.068934979	0.783556573	-0.087977029	0.929894934	0.999527751	ENSG00000219755	AL137784.1
14.519338	0.617554283	0.86396371	0.714791925	0.474737625	0.99135833	ENSG00000003987	MTMR7
15.12399309	-1.271319395	0.929790215	-1.367318535	0.171525485	0.944422693	ENSG00000245573	BDNF-AS
14.80249498	0.244360915	0.749400935	0.326075007	0.744367606	0.999467288	ENSG00000065621	GSTO2
14.87573373	-2.178650619	1.306750543	-1.667227636	0.095469149	0.921147998	ENSG00000249641	HOXC13-AS
14.69547921	0.228421663	0.768200399	0.297346452	0.766202021	0.999467288	ENSG00000279722	AC007342.7
14.84791475	1.091903751	0.749262481	1.457304722	0.145032284	0.935644597	ENSG00000224543	SNRPGP15
14.62833558	0.242849647	0.904298239	0.268550392	0.788275692	0.999467288	ENSG00000162390	ACOT11
14.13055562	0.364019586	0.802929284	0.45336444	0.650286338	0.997381078	ENSG00000185839	AL035411.1
14.7392638	-0.219679707	1.387964465	-0.158274734	0.874240316	0.999467288	ENSG00000143469	SYT14
14.2829051	-0.385527036	0.880450882	-0.437874552	0.661477224	0.997568624	ENSG00000280187	AC022107.1
14.43766677	0.237314385	0.773458113	0.306822542	0.758978449	0.999467288	ENSG00000203722	RAET1G
14.49509269	0.278852856	0.869560277	0.320682606	0.74845093	0.999467288	ENSG00000185267	CDNF
14.48503079	0.065701583	0.835896235	0.078600166	0.937350655	0.999527751	ENSG00000179008	C14orf39
14.63768619	0.601329138	0.951230422	0.63215928	0.527282803	0.99483168	ENSG00000156968	MPV17L
13.83517007	0.652513177	0.964535429	0.676505141	0.498719983	0.993513219	ENSG00000141854	MISP3
14.03228531	1.999454989	1.205348829	1.658818543	0.097152363	0.921147998	ENSG00000162723	SLAMF9
13.96849702	-0.312097924	0.979044235	-0.318778164	0.749894738	0.999467288	ENSG00000127241	MASP1
14.4215928	0.316126082	0.729826076	0.433152627	0.664903892	0.997568624	ENSG00000249700	SRD5A3-AS1
13.64625287	0.521827608	1.15803979	0.450612848	0.652268605	0.997449432	ENSG00000106511	MEOX2
14.30079738	-0.504302274	0.86088133	-0.585797666	0.558011493	0.99483168	ENSG00000269313	MAGIX
14.18639821	-0.145703167	0.770753848	-0.189039817	0.85006161	0.999467288	ENSG00000204072	ARMCX7P
13.96968696	0.000453267	1.005939737	0.00045059	0.999640481	0.9998586	ENSG00000134339	SAA2
14.46455843	-0.243859929	0.789612173	-0.308835068	0.757446992	0.999467288	ENSG00000240036	AC104563.1
13.87684701	-0.224559154	0.872604998	-0.25734342	0.796913677	0.999467288	ENSG00000151651	ADAM8
14.55857878	-0.459861697	1.125734074	-0.408499403	0.682907069	0.998869317	ENSG00000111087	GLI1
14.29608428	-0.588042644	0.706102428	-0.832800768	0.404957101	0.989962822	ENSG00000184979	USP18
13.84523749	0.352824839	0.816742785	0.431990151	0.665748571	0.997568624	ENSG00000116990	MYCL
13.67511295	-0.21016658	1.033373321	-0.203379142	0.838838701	0.999467288	ENSG00000124374	PAIP2B
14.31454419	-0.698010202	1.115687923	-0.625632121	0.531556265	0.99483168	ENSG00000168743	NPNT
14.72074337	-0.547295926	0.828199708	-0.660826031	0.508723887	0.993513219	ENSG00000112182	BACH2
14.78377398	-1.711819828	0.893847259	-1.915114478	0.055477906	0.847598083	ENSG00000146700	SSC4D
13.65400621	-0.170839462	0.961949824	-0.177597062	0.859039432	0.999467288	ENSG00000128573	FOXP2
14.38889431	0.89910627	0.74728379	1.203165761	0.228912182	0.959527137	ENSG00000253106	AC090198.1
14.50361062	-0.733603469	0.821979258	-0.892484162	0.372133478	0.982043455	ENSG00000259676	AC103982.1
13.7664039	1.899055054	1.059610665	1.792219649	0.073097788	0.888663898	ENSG00000158445	KCNB1
14.00364113	-0.307357277	0.824868346	-0.372613737	0.709435948	0.99900742	ENSG00000187534	PRR13P5
14.251372	0.423623767	0.770099794	0.550089443	0.582258027	0.99483168	ENSG00000242861	AL591895.1
14.58618924	-0.042193026	0.875320313	-0.048202955	0.961554495	0.999527751	ENSG00000246082	NUDT16P1
13.99531598	0.427393184	0.999072008	0.427790169	0.668803895	0.997568624	ENSG00000058866	DGKG

14.38383732	0.201950501	0.767604117	0.263091998	0.792479672	0.999467288	ENSG00000253716	MINCR
14.41811902	-0.918008351	0.865708288	-1.060413032	0.288956736	0.966288987	ENSG00000268996	MAN1B1-DT
13.89701504	-0.441203134	0.786169131	-0.561206382	0.574656853	0.99483168	ENSG00000255320	AP000759.1
14.12527211	-1.225365532	0.842282179	-1.45481593	0.145720227	0.935644597	ENSG00000264230	ANXA8L1
14.00868451	-0.783404971	0.830854927	-0.942890204	0.345737066	0.977691382	ENSG00000255874	LINC00346
13.84131587	0.057523719	0.980155424	0.058688365	0.953200327	0.999527751	ENSG00000152056	AP1S3
14.33935144	2.536961513	0.969669488	2.616315707	0.008888433	0.608415359	ENSG00000261786	AC006058.1
14.2536877	-0.388185995	0.742657929	-0.522698244	0.601184261	0.99483168	ENSG00000217416	ISCA1P1
14.60309395	0.305385555	1.476430903	0.206840398	0.836134506	0.999467288	ENSG00000053108	FSTL4
14.49872739	0.123538383	0.903520093	0.136730089	0.891244145	0.999467288	ENSG00000235720	GABPAP
14.30270811	-1.088746234	0.856824541	-1.270675829	0.203843998	0.954310328	ENSG00000223546	LINC00630
13.61040767	-0.884154412	1.230543049	-0.718507502	0.472444424	0.99135833	ENSG00000076770	MBNL3
14.08563351	-0.059559929	0.852380985	-0.069874775	0.944293331	0.999527751	ENSG00000110195	FOLR1
14.05473746	-0.33590685	0.758317839	-0.442963138	0.657792397	0.997568624	ENSG00000134955	SLC37A2
14.43966827	-0.958272878	0.85332114	-1.122992075	0.26144086	0.962465655	ENSG00000183346	CABCOCO1
13.99332859	-1.393851143	1.078629689	-1.292242516	0.196273172	0.954310328	ENSG00000258824	AL122035.1
14.21143464	0.402721616	0.868694729	0.463593944	0.642938694	0.997096914	ENSG00000108515	ENO3
14.23898242	1.029252313	0.736438341	1.397608266	0.162230735	0.943223078	ENSG00000196381	ZNF781
14.24735287	1.063688368	0.795124038	1.337764068	0.180973359	0.949094286	ENSG00000184441	AP001062.1
14.62990031	-1.032564762	1.186684703	-0.870125619	0.384231759	0.985655313	ENSG00000210195	MT-TT
14.24680882	0.575817495	0.751964834	0.765750563	0.443824735	0.99135833	ENSG00000224536	AC096677.1
13.74913517	2.355564588	0.896251233	2.628241392	0.008582759	0.604581883	ENSG00000187123	LYPD6
14.1547928	-0.308055075	0.766701339	-0.401792796	0.687836524	0.998869317	ENSG00000182890	GLUD2
13.73296669	0.606148706	0.828093442	0.731981049	0.464180135	0.99135833	ENSG00000171860	C3AR1
14.03552927	-1.734494923	1.369759097	-1.266277353	0.205413802	0.954310328	ENSG00000141052	MYOCD
13.85386885	0.072807579	1.270342734	0.057313335	0.954295598	0.999527751	ENSG00000167676	PLIN4
13.95424734	0.375389848	0.934591163	0.401662098	0.687932722	0.998869317	ENSG00000213996	TM6SF2
14.60663489	0.509001205	1.16596235	0.436550293	0.662437518	0.997568624	ENSG00000115602	IL1RL1
14.21674195	0.247366709	0.759145429	0.325848908	0.744538673	0.999467288	ENSG00000188760	TMEM198
14.18503555	0.338804051	0.885006225	0.382826743	0.701848231	0.998869317	ENSG00000175697	GPR156
14.11843649	2.279108248	1.139013254	2.000949717	0.045397809	0.813244956	ENSG00000158220	ESYT3
14.10041138	-0.849211556	0.807028274	-1.052269893	0.29267574	0.970706863	ENSG00000241627	UBQLN4P1
14.15373872	-1.713302541	0.886634376	-1.932366472	0.053314289	0.847598083	ENSG00000272970	AC107294.2
13.78072072	1.098348603	0.735537168	1.493260506	0.135369009	0.934130245	ENSG00000204428	LY6G5C
13.87629846	0.564291212	0.767110702	0.73560597	0.461970528	0.99135833	ENSG00000273014	AC018645.2
13.75234943	0.457667895	0.866221808	0.528349542	0.597256752	0.99483168	ENSG00000136197	C7orf25
14.43246097	0.588248509	0.847935869	0.693741744	0.487844182	0.993513219	ENSG00000260966	AP001486.2
13.81171157	-0.556826133	0.957417508	-0.58159176	0.560841695	0.99483168	ENSG00000278126	AC139768.1
14.12401997	-0.340885505	0.770040621	-0.442685094	0.657993526	0.997568624	ENSG00000259065	AC005520.2
14.42494347	0.889173467	1.072287012	0.829230847	0.406973793	0.990226751	ENSG00000176700	SCAND2P
13.67041283	0.706955035	1.021946673	0.691772921	0.489079945	0.993513219	ENSG00000131746	TNS4
14.30291685	-1.193738569	0.904691947	-1.319497286	0.187002917	0.949094286	ENSG00000177133	LINC00982
14.10233747	3.419222696	1.130630913	3.024172306	0.002493144	0.360084787	ENSG00000232931	LINC00342
13.56486572	-0.242331726	0.843190071	-0.287398695	0.77380706	0.999467288	ENSG00000226564	FTH1P20
13.91964897	-0.247746061	0.851871468	-0.290825636	0.771184678	0.999467288	ENSG00000113248	PCDHB15
14.21706774	-0.448069192	0.812196984	-0.551675518	0.581170686	0.99483168	ENSG00000197935	ZNF311
14.1074319	-0.805582469	0.900697478	-0.894398496	0.371108717	0.981955904	ENSG00000161328	LRRC56
15.55864814	-2.424003348	1.521091049	-1.59359517	0.111026737	0.923922314	ENSG00000167178	ISLR2
13.95961827	-0.258941544	0.838937484	-0.308654159	0.757584618	0.999467288	ENSG00000176401	EID2B
13.33990955	-0.1577732	0.922034832	-0.171114142	0.864134016	0.999467288	ENSG00000117245	KIF17
13.70051859	0.006539248	0.71933327	0.009090708	0.992746765	0.999527751	ENSG00000261716	HIST2H2BC
13.49696711	0.064872017	0.96071323	0.06752485	0.946163879	0.999527751	ENSG00000234432	AC092171.3
13.66317939	1.082348583	0.896679052	1.207063531	0.227407678	0.958865115	ENSG00000272734	ADIRF-AS1
13.58985535	0.808811369	0.849793116	0.951774442	0.341211387	0.977691382	ENSG00000261819	AC138932.3
13.88038134	-1.089322315	0.966275134	-1.127341765	0.259598011	0.962465655	ENSG00000140832	MARVELD3
13.9701419	-0.794970581	0.835968799	-0.950957239	0.341626083	0.977691382	ENSG00000280254	AC233723.2
13.81333522	2.582111582	1.045385088	2.470009963	0.013510929	0.672931175	ENSG00000212724	KRTAP2-3
14.14275037	0.999362606	0.803744467	1.243383496	0.213726549	0.954310328	ENSG00000279377	AC003973.3
13.9158976	0.923882131	0.946392425	0.976214631	0.328958114	0.976026345	ENSG00000243970	PPIEL
13.68055502	-0.187626172	0.72630167	-0.258330911	0.796151532	0.999467288	ENSG00000236432	AC097662.1
14.30649184	-0.48496179	1.006974657	-0.481602776	0.630088154	0.99483168	ENSG00000144668	ITGA9
13.24706348	1.102583248	0.864709978	1.275090235	0.202277305	0.954310328	ENSG00000261490	AC005674.2
13.44763333	1.172534896	0.776450502	1.510121886	0.131012326	0.929833427	ENSG00000091831	ESR1
13.63319658	0.81314607	0.737782256	1.102149129	0.270396844	0.962465655	ENSG00000218226	TATDN2P2

13.84617195	1.115880998	0.927502624	1.203102793	0.228936545	0.959527137	ENSG00000135205	CCDC146
13.47136042	-0.425900852	0.798895504	-0.53311209	0.59395599	0.99483168	ENSG00000224897	POT1-AS1
13.86638479	-0.200844012	0.934242401	-0.214980622	0.829782427	0.999467288	ENSG00000232229	LINC00865
13.59010631	-0.580376123	0.786900528	-0.737546999	0.460789773	0.99135833	ENSG00000260260	SNHG19
13.49758058	-0.237085334	0.873067021	-0.271554564	0.785964539	0.999467288	ENSG00000268001	CARD8-AS1
13.59200267	0.759787176	0.766402891	0.991367836	0.321506	0.975337095	ENSG00000133056	PIK3C2B
13.74408983	-0.925010804	0.792561006	-1.167116218	0.243163439	0.962465655	ENSG00000270820	AC016727.1
13.5419246	0.519933914	0.834433859	0.623097814	0.533220246	0.99483168	ENSG00000237753	FLJ42351
13.76295997	-0.620215973	0.776802426	-0.798421777	0.42462577	0.991301167	ENSG00000263327	TAPT1-AS1
13.63408306	0.081706642	0.899639149	0.090821573	0.927634368	0.999527751	ENSG00000272338	AC067838.1
13.81855913	0.344430864	0.738194411	0.466585576	0.640796409	0.996553586	ENSG00000234160	AL513165.1
13.39511679	-0.443104887	0.85236182	-0.51985539	0.60316437	0.99483168	ENSG00000230091	TMEM254-AS1
13.4885078	-0.908890543	0.921478948	-0.986338912	0.323966831	0.975412329	ENSG00000277283	AC004812.2
13.61965018	0.752340969	0.759863563	0.990100072	0.322125208	0.975337095	ENSG00000100583	SAMD15
13.29053894	0.343541864	0.879780653	0.390485814	0.696177343	0.998869317	ENSG00000269386	RAB11B-AS1
13.86043539	0.256960483	0.936614953	0.274350182	0.783815524	0.999467288	ENSG00000160111	CPAMD8
13.24714456	-0.549720344	1.260999709	-0.435940104	0.662880187	0.997568624	ENSG00000183668	PSG9
13.5441604	-0.288871003	0.992085439	-0.291175529	0.770917077	0.999467288	ENSG00000100065	CARD10
13.59788249	-1.715615148	0.900996797	-1.90413013	0.056893239	0.851511963	ENSG00000114790	ARHGEF26
13.82240462	0.979484012	1.173858071	0.834414344	0.404047537	0.988927059	ENSG00000255389	Z97989.1
13.61515366	0.458270648	0.917398808	0.49953264	0.617404199	0.99483168	ENSG00000234390	USP27X-AS1
13.54959386	1.471467606	1.034740934	1.422063781	0.155007736	0.939432741	ENSG00000123496	IL13RA2
13.91975366	0.954159455	1.173785895	0.812890544	0.416280816	0.990226751	ENSG00000011347	SYT7
13.55041756	0.783417739	1.158458604	0.676258725	0.498876393	0.993513219	ENSG00000127324	TSPAN8
13.6504045	1.387445082	0.849458205	1.633329425	0.102399731	0.92169898	ENSG00000212694	LINC01089
13.39474934	-0.946492901	1.021443105	-0.926623222	0.354122194	0.977691382	ENSG00000259705	AC084757.3
13.28339693	1.494124803	0.965185553	1.548018201	0.121617915	0.923922314	ENSG00000159640	ACE
13.32313595	0.627051955	0.752448851	0.83334828	0.404648335	0.989741812	ENSG00000100079	LGALS2
13.49979897	0.038608345	0.821211378	0.047013894	0.962502154	0.999527751	ENSG00000279348	AC012513.3
13.09527899	1.178977487	0.874484046	1.348197822	0.177594765	0.946844293	ENSG00000251595	ABCA11P
13.38527134	-0.07747169	0.821446173	-0.094311341	0.924861842	0.999527751	ENSG00000128594	LRRC4
13.72018766	0.176229156	0.970343302	0.181615265	0.855884669	0.999467288	ENSG00000164707	SLC13A4
13.56896603	-0.203330523	0.835731367	-0.243296508	0.807775709	0.999467288	ENSG00000198932	GPRASP1
13.17951133	0.417868479	0.852890799	0.489943706	0.624173735	0.99483168	ENSG00000237984	PTENP1
13.17926529	0.240003346	0.893174933	0.268708108	0.788154312	0.999467288	ENSG00000110042	DTX4
13.71760023	0.655479708	0.935087577	0.700982159	0.48331415	0.991892545	ENSG00000214756	CSKMT
13.02916709	0.475436169	0.981686889	0.484305305	0.628169211	0.99483168	ENSG00000149781	FERMT3
13.27464057	-0.263822415	0.871031676	-0.302884984	0.761977515	0.999467288	ENSG00000175591	P2RY2
12.95682288	-1.136276211	0.858491854	-1.323572503	0.185645062	0.949094286	ENSG00000140678	ITGAX
13.47976144	1.177875514	0.960655948	1.22611588	0.220155071	0.956884001	ENSG00000267547	AC060766.4
13.59366461	-0.215709758	0.844803849	-0.255337092	0.798462754	0.999467288	ENSG00000279329	AC020910.5
13.24637604	-0.26767242	1.040301388	-0.257302762	0.796945061	0.999467288	ENSG00000272455	AL391244.3
13.24289736	1.255485704	1.201680193	1.044775233	0.296126864	0.970706863	ENSG00000152154	TMEM178A
13.34634019	-0.074513682	0.785458864	-0.094866434	0.924420918	0.999527751	ENSG00000261167	AC107027.3
13.33256922	-0.143779579	1.02052584	-0.140887739	0.887958628	0.999467288	ENSG00000248810	LINC02432
13.21501124	1.542261002	0.805800013	1.913950084	0.055626534	0.847598083	ENSG00000188185	LINC00265
13.99874557	0.802952239	1.480026406	0.542525617	0.587456465	0.99483168	ENSG00000077063	CTTNBP2
13.82760723	-0.008996183	0.909136431	-0.009895305	0.992104818	0.999527751	ENSG00000158457	TSPAN33
13.3080072	0.343835383	0.835815844	0.411376962	0.680796144	0.998869317	ENSG00000247746	USP51
13.55871193	-1.521670576	1.110104039	-1.370745914	0.170454177	0.944422693	ENSG00000165325	DEUP1
13.20033275	-0.802521961	0.85899611	-0.93425564	0.350172041	0.977691382	ENSG00000170421	KRT8
13.13778728	-0.423847372	1.005371704	-0.421582755	0.673329596	0.99853019	ENSG00000139835	GRTP1
13.43614841	0.140587087	0.872746414	0.161085838	0.872025794	0.999467288	ENSG00000100918	REC8
13.62835291	0.916164484	1.175245322	0.779551697	0.435654797	0.991301167	ENSG00000100479	POLE2
13.49109538	-1.111220084	0.841521286	-1.320489574	0.186671615	0.949094286	ENSG00000258376	AC004846.1
14.02483925	-1.986627474	1.110970174	-1.788191547	0.07374511	0.888663898	ENSG00000234648	AL162151.2
13.19764662	-0.111314773	0.859870319	-0.129455303	0.89699739	0.999467288	ENSG00000267493	CIRBP-AS1
13.61826501	-0.024097205	1.173918414	-0.020527155	0.98362285	0.999527751	ENSG00000187942	LDLRAD2
13.55954422	-0.295366914	1.129049997	-0.261606585	0.793624767	0.999467288	ENSG00000247708	STX18-AS1
13.8396228	-0.864672124	0.944703959	-0.915283689	0.36004272	0.979465649	ENSG00000251396	LINC01301
12.90151785	-0.200643465	1.167316095	-0.171884432	0.863528386	0.999467288	ENSG00000104490	NCALD
13.16507362	0.490599135	0.953911841	0.514302384	0.607040595	0.99483168	ENSG00000251003	ZFPM2-AS1
13.83927486	-0.540873148	0.923559452	-0.585639774	0.558117614	0.99483168	ENSG00000203499	IQANK1
13.00707101	0.416771583	0.780679615	0.533857392	0.593440203	0.99483168	ENSG00000160401	CFAP157

13.24679576	-1.129490199	1.25021489	-0.903436847	0.366294091	0.981269214	ENSG00000152402	GUCY1A2
13.09478036	-0.041193597	0.789207606	-0.052196148	0.958372402	0.999527751	ENSG00000247934	AC022364.1
13.49363076	0.54803283	0.889577177	0.616059904	0.537854984	0.99483168	ENSG00000167984	NLRC3
13.46940222	0.379724022	0.957730691	0.396483089	0.691748681	0.998869317	ENSG00000260852	FBXL19-AS1
13.78123798	0.598286517	0.99340005	0.602261412	0.547000142	0.99483168	ENSG00000131797	CLUHP3
13.08591133	0.840886077	0.734453825	1.14491347	0.252245004	0.962465655	ENSG00000271851	AC087501.4
13.38775621	-0.722368613	0.755828427	-0.955730941	0.339208186	0.977691382	ENSG00000243678	NME2
13.66703578	0.043948709	1.0362082	0.04241301	0.966169457	0.999527751	ENSG00000220804	LINC01881
12.7886151	0.553435418	0.870490136	0.635774485	0.52492342	0.99483168	ENSG00000271270	TMCC1-AS1
12.82781829	-1.175294052	0.944244767	-1.244692153	0.213244932	0.954310328	ENSG00000236548	RNF217-AS1
13.37962456	-0.908009787	0.80401679	-1.129341822	0.258753665	0.962465655	ENSG00000150773	PIH1D2
13.08055346	-0.244487044	0.873196511	-0.279990862	0.779484516	0.999467288	ENSG00000059915	PSD
13.23857543	-0.979174986	0.881959807	-1.110226314	0.266901516	0.962465655	ENSG00000249456	AL731577.2
12.84941475	-0.626487155	0.878775525	-0.712909199	0.475901946	0.99135833	ENSG00000213250	RBMS2P1
12.99576958	1.377032819	1.125314429	1.223687161	0.221070265	0.956884001	ENSG00000104147	OIP5
13.56544922	0.334030961	0.85336078	0.391429942	0.695479464	0.998869317	ENSG00000263006	ROCK1P1
12.92248719	-0.686831199	0.800073388	-0.858460248	0.390638372	0.987743348	ENSG00000171017	LRRC8E
12.69403596	1.989855597	0.944631609	2.106488475	0.035161943	0.784512596	ENSG00000237624	OXCT2P1
13.05940284	1.422076028	0.985382337	1.443171827	0.148972074	0.936396974	ENSG00000174827	PDZK1
13.08244017	-0.215105342	0.810623536	-0.265357878	0.790733781	0.999467288	ENSG00000230756	RHOQP3
12.80626854	-1.141108824	1.009923312	-1.129896509	0.258519835	0.962465655	ENSG00000238278	ALG1L6P
12.99616642	-0.669246119	0.822036839	-0.814131541	0.415569599	0.990226751	ENSG00000285813	AP000813.1
13.01106751	0.461367376	0.774343001	0.595817842	0.551296923	0.99483168	ENSG00000130649	CYP2E1
13.1131124	0.601399254	0.868346867	0.692579517	0.488573468	0.993513219	ENSG00000255857	PXN-AS1
13.17940929	0.198099243	0.805978791	0.245787166	0.805846992	0.999467288	ENSG00000277639	AC007906.2
12.72774572	0.49432363	0.93500796	0.528683874	0.597024766	0.99483168	ENSG00000227992	AC108463.1
12.87069137	0.339781471	0.790708818	0.429717569	0.667401102	0.997568624	ENSG00000197385	ZNF860
13.84080918	-0.563131756	1.437066226	-0.391862077	0.695160125	0.998869317	ENSG00000038295	TLL1
13.18514052	-0.618749324	0.81816081	-0.756268592	0.449488175	0.99135833	ENSG00000170469	SPATA24
12.92983939	-0.456283494	0.886722188	-0.514573223	0.606851281	0.99483168	ENSG00000083067	TRPM3
12.99418502	0.420942774	0.757476713	0.555717116	0.578404254	0.99483168	ENSG00000149050	ZNF214
13.11925993	0.355796248	0.768752089	0.462823131	0.64349115	0.997097166	ENSG00000255561	FDXACB1
13.09453381	0.558241821	0.871329076	0.640678519	0.521731574	0.993980889	ENSG00000166321	NUDT13
12.38095498	0.514981087	1.447987534	0.355652984	0.722100459	0.99906455	ENSG00000251301	LINC02384
12.89634203	-0.611985936	0.825801056	-0.741081561	0.458643989	0.99135833	ENSG00000258839	MC1R
12.76418211	0.21157438	0.950840938	0.222512906	0.823914627	0.999467288	ENSG00000271503	CCL5
13.2334361	-2.419807499	1.343860942	-1.800638313	0.071759906	0.884210289	ENSG00000149633	KIAA1755
12.98680088	-0.279758247	0.963769636	-0.290275017	0.77160585	0.999467288	ENSG00000073282	TP63
12.74548631	-1.493051989	0.962019269	-1.551998007	0.1206627	0.923922314	ENSG00000170891	CYTL1
12.55688189	-0.011499669	1.185501365	-0.009700258	0.992260435	0.999527751	ENSG00000121211	MND1
12.68794316	0.038687171	0.757329133	0.051083696	0.959258828	0.999527751	ENSG00000232150	ST13P4
12.79699701	0.40196643	0.746061748	0.538784398	0.59003563	0.99483168	ENSG00000091262	ABCC6
12.5690895	-1.302171179	0.957716784	-1.359662064	0.173936888	0.94495857	ENSG00000132470	ITGB4
12.97572854	0.999214774	0.856099841	1.167170844	0.243141383	0.962465655	ENSG00000213988	ZNF90
13.14664395	-2.114333858	0.945658072	-2.235833353	0.025362689	0.759116406	ENSG00000213888	LINC01521
12.31618262	1.380480252	1.189522338	1.160533272	0.245831755	0.962465655	ENSG00000137960	GIPC2
12.87301532	-2.37586515	0.9451291	-2.513799597	0.011943828	0.648356243	ENSG00000150681	RGS18
12.79558711	0.758221214	1.171492057	0.647226935	0.517485081	0.993513219	ENSG00000235831	BHLHE40-AS1
12.90882752	-0.610643487	0.803054013	-0.760401514	0.447014619	0.99135833	ENSG00000251669	FAM86EP
12.56940017	0.542099512	1.035530029	0.523499557	0.600626659	0.99483168	ENSG00000249790	AC092490.1
12.80756226	-0.496120896	0.877669634	-0.565270664	0.571889679	0.99483168	ENSG00000279800	BCLAF1P2
13.04589532	-5.067479314	1.59964795	-3.167871602	0.001535593	0.280741431	ENSG00000087258	GNAO1
13.2681061	-0.858919906	0.850524762	-1.009870546	0.312557316	0.973427483	ENSG00000226243	RPL37AP1
13.12328216	2.275811065	1.119835601	2.032272472	0.04212608	0.804797577	ENSG00000129951	PLPPR3
12.56507458	0.485542708	1.004839852	0.483204072	0.628950845	0.99483168	ENSG00000121904	CSMD2
12.54664583	-1.007747917	0.878585685	-1.147011538	0.251376853	0.962465655	ENSG00000224550	AC114491.1
13.08838844	2.015816439	0.970166742	2.077804104	0.037727406	0.790190505	ENSG00000272645	GTF2IP20
12.63910199	0.669784974	0.784397934	0.853884163	0.393169175	0.988378925	ENSG00000232531	AC027612.3
12.6840487	0.429941521	0.749501881	0.573636346	0.566213911	0.99483168	ENSG00000235734	HMGN1P36
12.79896023	-0.499475833	0.789951932	-0.632286362	0.527199774	0.99483168	ENSG00000213763	ACTBP2
12.49135051	-0.094804392	0.945967768	-0.100219474	0.920170086	0.999527751	ENSG00000231113	AL035587.1
13.66166629	-1.233081073	3.244550013	-0.380046869	0.703910624	0.99900742	ENSG00000233952	FTLP15
12.72460386	0.452324902	1.3172342	0.343389886	0.731305167	0.999467288	ENSG00000123560	PLP1
13.10201941	-1.45856428	1.086256924	-1.342743368	0.179355087	0.9469453	ENSG00000175445	LPL

12.93268634	1.334482381	1.056415246	1.263217647	0.206510973	0.954310328	ENSG00000160886	LY6K	
12.11112742	0.128885216	1.20069448	0.107342224	0.914517488	0.999527751	ENSG00000183644	C11orf88	
12.41504156	-1.187483191	0.955249892	-1.243112614	0.213826338	0.954310328	ENSG00000149418	ST14	
12.47008755	0.884288303	0.972707915	0.909099525	0.363297594	0.98048242	ENSG00000279822	AC016397.2	
12.80995235	1.528706268	0.880867292	1.735455818	0.082660101	0.899922174	ENSG00000272821	U62317.2	
12.32984126	0.75269021	0.958953822	0.784907669	0.432507705	0.991301167	ENSG00000187642	PERM1	
12.14098643	0.043740082	0.978899524	0.044682912	0.964360054	0.999527751	ENSG00000117472	TSPAN1	
12.99430174	-0.058534587	0.997978392	-0.05865316	0.953228368	0.999527751	ENSG00000128655	PDE11A	
12.703365	0.402423713	0.745529219	0.539782617	0.589346956	0.99483168	ENSG00000230457	PA2G4P4	
12.41536895	-0.829348276	1.122535976	-0.738816656	0.460018336	0.99135833	ENSG00000215068	AC025171.2	
12.18909869	0.566225156	1.168934991	0.484394051	0.628106239	0.99483168	ENSG00000274750	HIST1H3E	
12.53716137	-0.954591546	0.88314142	-1.080904512	0.279739591	0.963267117	ENSG00000235238	SUMO2P1	
12.29236978	-1.653596284	1.08648774	-1.521964973	0.128017858	0.928627271	ENSG00000204287	HLA-DRA	
12.47271017	0.508882292	0.903906542	0.562981092	0.573447759	0.99483168	ENSG00000268350	FAM156A	
12.51668214	1.485860936	0.976125791	1.522202311	0.127958396	0.928627271	ENSG00000224596	ZMIZ1-AS1	
12.38691522	-0.598159565	0.961913425	-0.621843452	0.534044811	0.99483168	ENSG00000233325	MIPEPP3	
12.85715453	0.213166106	0.926352897	0.230113282	0.818003744	0.999467288	ENSG00000258667	HIF1A-AS2	
12.77527378	-0.003317397	0.983950559	-0.003371507	0.997309931	0.999527751	ENSG00000197249	SERPINA1	
12.52475344	-0.958250313	0.849238819	-1.128363767	0.259166323	0.962465655	ENSG00000214226	C17orf67	
12.51850475	1.999388552	1.076729528	1.856908816	0.063324128	0.868202292	ENSG00000134321	RSAD2	
12.4239816	-0.693631282	0.895019567	-0.774990075	0.438345524	0.991301167	ENSG00000184139	RPL7AP28	
12.42393902	0.378975905	0.96690799	0.391946192	0.695097972	0.998869317	ENSG00000146054	TRIM7	
12.22947729	-1.263339745	1.150917787	-1.097680268	0.272344129	0.962465655	ENSG00000176125	UFSP1	
11.8507011	-0.042784733	1.333366813	-0.032087744	0.974402077	0.999527751	ENSG00000047648	ARHGAP6	
12.12369681	-0.496551473	0.970657547	-0.511561956	0.608957617	0.99483168	ENSG00000164683	HEY1	
12.64765098	-0.064667809	0.944486541	-0.068468746	0.945412499	0.999527751	ENSG00000203497	PDCD4-AS1	
12.29474516	0.00948916	0.971084404	0.009771715	0.992203424	0.999527751	ENSG00000151846	PABPC3	
12.63101401	2.104870938	0.90242172	2.332469278	0.019676017	0.73975641	ENSG00000214967	NPIPA7	
12.55404332	0.362906617	0.773410755	0.469228822	0.638906086	0.996553586	ENSG00000171817	ZNF540	
12.3347	-0.062379389	1.011299807	-0.06168239	0.950815764	0.999527751	ENSG00000269388	AC018755.3	
11.94518589	0.832318805	1.036931679	0.802674681	0.422162791	0.991301167	ENSG00000176771	NCKAP5	
12.12325268	-0.07160141	1.366591319	-0.052394164	0.958214624	0.999527751	ENSG00000239828	AC063944.1	
12.68038422	-1.570988294	1.039481354	-1.511319359	0.130707104	0.929373153	ENSG00000215217	C5orf49	
12.49204189	-1.115319296	0.930502588	-1.198620305	0.230675619	0.959575179	ENSG00000253520	AC136628.3	
12.27784452	-0.254498296	0.931334824	-0.273261871	0.784651923	0.999467288	ENSG00000272462	U91328.1	
12.51773883	0.548276243	0.936792073	0.585269943	0.558366222	0.99483168	ENSG00000229036	VDAC1P8	
12.48957305	-0.192148709	0.801874073	-0.239624544	0.810621336	0.999467288	ENSG00000229358	DPY19L1P1	
12.23507299	-0.619843756	0.964669973	-0.642544885	0.520519456	0.993513219	ENSG00000237004	ZNRF2P1	
12.46418054	-0.340318905	1.07882551	-0.31545315	0.752417617	0.999467288	ENSG00000102383	ZDHHC15	
12.72363249	-0.155645214	0.804811555	-0.193393364	0.846650912	0.999467288	ENSG00000253837	AC090197.1	
12.3751535	-0.355059814	0.857121257	-0.414246889	0.678693305	0.998869317	ENSG00000234618	RPSAP9	
12.2248871	0.248057705	1.050015008	0.236242057	0.813244846	0.999467288	ENSG00000166349	RAG1	
12.51041239	-0.933503179	0.891317835	-1.047329181	0.294947782	0.970706863	ENSG00000123338	NCKAP1L	
12.13227542	0.182472507	0.82276487	0.221779653	0.824485419	0.999467288	ENSG00000250548	LINC01303	
12.81324963	-0.338879853	0.959296158	-0.353258845	0.723894396	0.999456245	ENSG00000253954	HMGN1P38	
12.79914155	1.485260467	0.854708604	1.73773899	0.082256815	0.899345331	ENSG00000278864	AC055811.4	
12.58465889	0.455992908	0.889116446	0.512860728	0.608048746	0.99483168	ENSG00000069188	SDK2	
12.54016458	0.694428236	1.016846086	0.682923645	0.494655092	0.993513219	ENSG00000071073	MGAT4A	
12.39755295	0.824692442	0.792988361	1.039980512	0.298348955	0.972186492	ENSG00000272077	AC124045.1	
12.2841188	1.034487991	1.035609461	0.998917092	0.317834856	0.974768742	ENSG00000114378	HYAL1	
12.7371323	-0.815695765	1.122789492	-0.72649038	0.467538203	0.99135833	ENSG00000285725	AC004967.2	
12.55715651	-1.590372117	0.958499684	-1.659230717	0.09706931	0.921147998	ENSG00000106236	NPTX2	
12.58495821	0.349070161	0.970194586	0.359793969	0.719001213	0.99900742	ENSG00000255495	AC145124.1	
12.62039897	-0.016082273	1.205214009	-0.013343915	0.989353412	0.999527751	ENSG00000237512	UNC5B-AS1	
12.29115179	-0.611748384	0.779907865	-0.784385453	0.432813971	0.991301167	ENSG00000213621	RPSAP54	
12.48009313	-0.536547402	0.894233314	-0.600008291	0.54850071	0.99483168	ENSG00000226085	UQCRFS1P1	
12.18207642	-0.483084279	0.832959095	-0.579961588	0.561940522	0.99483168	ENSG00000100167		3-Sep
12.35543844	0.280948816	0.778830006	0.360731885	0.718299887	0.99900742	ENSG00000126091	ST3GAL3	
11.97651406	-0.361275723	0.940670435	-0.384061952	0.700932531	0.998869317	ENSG00000137975	CLCA2	
12.37071172	0.602813066	0.97832016	0.616171567	0.537781292	0.99483168	ENSG00000145777	TSLP	
11.87924166	0.333840031	1.011240199	0.330129312	0.741302256	0.999467288	ENSG00000128536	CDHR3	
12.40844942	1.05748342	1.025810648	1.030875846	0.302599045	0.972793733	ENSG00000091137	SLC26A4	
11.95364242	2.021320445	1.049309443	1.926333988	0.054062684	0.847598083	ENSG00000170775	GPR37	
12.56692952	-1.679588765	0.956107524	-1.756694433	0.078969907	0.897052166	ENSG00000228960	OR2A9P	

12.57744127	0.247705743	1.274879118	0.194297435	0.845942994	0.999467288	ENSG00000120659	TNFSF11
12.14499634	1.173129902	0.849524175	1.38092586	0.167301758	0.943223078	ENSG00000259856	RAB43P1
12.19675026	-0.320511211	0.852901158	-0.375789396	0.707073472	0.99900742	ENSG00000277969	AC006449.6
11.79272156	-0.345590045	1.098246192	-0.314674476	0.753008825	0.999467288	ENSG00000085831	TTC39A
12.20252102	0.198774021	0.88997455	0.223347984	0.823264685	0.999467288	ENSG00000272789	AC010976.2
12.14630046	0.201795824	0.986108713	0.204638517	0.83785456	0.999467288	ENSG00000227502	LINC01268
11.9860273	2.628051695	1.034372944	2.540719679	0.011062457	0.644117389	ENSG00000223749	MIR503HG
12.35775356	-0.830419183	0.865752923	-0.959187271	0.337464412	0.977691382	ENSG00000272767	JMJD1C-AS1
12.29599766	0.757524615	0.847155927	0.894197386	0.37121629	0.981955904	ENSG00000130684	ZNF337
11.87114403	-0.689816691	1.069177474	-0.64518446	0.518807655	0.993513219	ENSG00000042062	RIPOR3
11.99739818	0.114538888	0.890107173	0.128679884	0.897610953	0.999467288	ENSG00000232442	MHENCR
12.18836366	-0.036797896	0.862963592	-0.042641308	0.965987466	0.999527751	ENSG00000268362	AC092279.1
12.13828446	0.256629651	0.855851644	0.299852963	0.764289314	0.999467288	ENSG00000171049	FPR2
11.94784076	-0.45511093	0.91399871	-0.497933887	0.618530642	0.99483168	ENSG00000227295	ELL2P1
12.14253837	0.091382518	0.757773878	0.120593387	0.904013107	0.999527751	ENSG00000235386	AC113174.1
12.26735057	1.116458006	0.94958912	1.175727462	0.239703801	0.962465655	ENSG00000174125	TLR1
12.27794076	-0.463515238	0.954472997	-0.485624255	0.627233596	0.99483168	ENSG00000180596	HIST1H2BC
11.98034067	0.253757445	0.763034638	0.332563467	0.739463824	0.999467288	ENSG00000237310	GS1-124K5.4
12.06237009	-0.067992951	0.950850696	-0.071507495	0.94299386	0.999527751	ENSG00000272899	ATP6V1FNB
12.26209297	0.615644755	0.83567057	0.736707474	0.461300261	0.99135833	ENSG00000213865	C8orf44
12.13170138	0.661177634	0.80752897	0.818766457	0.412919672	0.990226751	ENSG00000137857	DUOX1
12.15644145	0.587400022	0.795259814	0.738626561	0.460133791	0.99135833	ENSG00000180747	SMG1P3
12.20647203	-0.14194666	0.942435736	-0.150616805	0.880278004	0.999467288	ENSG00000070731	ST6GALNAC2
12.40066781	0.463420322	0.870323319	0.532469155	0.5944011	0.99483168	ENSG00000205517	RGL3
11.76306925	-0.534765478	0.974933676	-0.548514726	0.583338521	0.99483168	ENSG00000276345	AC004556.1
12.0065136	-0.854363438	0.877388283	-0.973757519	0.330176951	0.976026345	ENSG00000232742	RHOQP2
11.57617253	-0.064220225	1.186271826	-0.05413618	0.956826667	0.999527751	ENSG00000145569	OTULINL
12.17138008	-0.499516804	1.081116766	-0.462037793	0.644054219	0.997332006	ENSG00000234882	EIF3EP1
11.89702422	-0.86918557	0.910187144	-0.95495259	0.339601673	0.977691382	ENSG00000226784	PGAM4
12.36806752	0.79545308	0.894808014	0.888965082	0.374021846	0.982609538	ENSG00000232630	PRPS1P2
12.07078711	-0.270933102	0.902782755	-0.300108859	0.764094122	0.999467288	ENSG00000280351	AC127496.7
11.84017735	-0.845563632	0.794018227	-1.064917157	0.28691342	0.965284229	ENSG00000179743	FLJ37453
11.96803789	-0.177466723	0.764271099	-0.23220389	0.816379656	0.999467288	ENSG00000244513	AC109587.1
11.95299305	-0.367461033	0.833485206	-0.440872892	0.65930502	0.997568624	ENSG00000196812	ZSCAN16
12.20302738	-0.219007656	0.983821408	-0.222609159	0.823839708	0.999467288	ENSG00000125355	TMEM255A
11.96874068	-0.808399137	0.848625194	-0.952598559	0.340793508	0.977691382	ENSG00000182993	C12orf60
11.79067852	-0.531844207	0.857829356	-0.61998835	0.535265457	0.99483168	ENSG00000089163	SIRT4
11.83509448	0.4707377	1.107493793	0.425047709	0.670801896	0.997568624	ENSG00000102878	HSF4
11.82156227	-0.750089199	0.940912172	-0.79719364	0.425338579	0.991301167	ENSG00000204815	TTC25
11.88178712	-0.711032917	0.913880054	-0.778037461	0.436546929	0.991301167	ENSG00000264920	AC018521.5
12.32634863	-2.941325805	1.144717197	-2.569478133	0.010185182	0.631286776	ENSG00000189001	SBSN
12.36804946	1.562272777	0.989071596	1.579534568	0.114213494	0.923922314	ENSG00000184949	FAM227A
11.81380958	-0.2362361	0.81644645	-0.289346717	0.772316065	0.999467288	ENSG00000228463	AP006222.1
11.864181	1.675639012	0.853511313	1.963229996	0.049619455	0.836346249	ENSG00000187583	PLEKHN1
11.63083885	-0.346569423	0.975292975	-0.35534904	0.722328121	0.999092366	ENSG00000231789	PIK3CD-AS2
11.76340363	1.224714765	0.864340179	1.416936058	0.156501623	0.939464191	ENSG00000173369	C1QB
11.54393012	0.29785337	1.037533294	0.287078373	0.774052311	0.999467288	ENSG00000228109	MELTF-AS1
11.62101797	0.215848182	0.878756348	0.245629158	0.805969315	0.999467288	ENSG00000172426	RSPH9
11.68327939	0.141417003	0.83857079	0.168640507	0.866079417	0.999467288	ENSG00000235859	AC006978.1
11.69093303	-0.6054932	0.7687928	-0.787589583	0.43093681	0.991301167	ENSG00000280303	ERICD
11.90612978	0.498732191	1.189931769	0.419126713	0.675123529	0.998869317	ENSG00000077616	NAALAD2
11.74866677	-0.23605521	0.931970425	-0.25328616	0.80004709	0.999467288	ENSG00000273312	AL121749.1
11.89169889	-0.245464505	0.95381287	-0.2573508	0.79690798	0.999467288	ENSG00000230330	HMGN2P3
11.76411768	-0.319587074	0.95217414	-0.335639313	0.737142874	0.999467288	ENSG00000197943	PLCG2
11.63146332	0.080842068	0.806661597	0.10021807	0.920171201	0.999527751	ENSG00000188825	LINC00910
11.78384876	-0.626137891	0.811353214	-0.771720479	0.440279999	0.99135833	ENSG00000235776	AC000089.1
11.80266939	2.133032769	1.108487569	1.924273062	0.054320365	0.847598083	ENSG00000100068	LRP5L
11.57079992	-0.479600083	1.111028049	-0.431672345	0.66597957	0.997568624	ENSG00000100156	SLC16A8
11.29451624	0.038077042	1.04094721	0.036579224	0.970820509	0.999527751	ENSG00000226674	TEX41
12.17724129	-1.442807848	0.991420991	-1.455292818	0.145588214	0.935644597	ENSG00000221883	ARIH2OS
11.87859588	-0.82571678	0.988618074	-0.835223229	0.403592033	0.988927059	ENSG00000171658	NMRAL2P
11.97327461	-0.059059304	1.025590695	-0.057585647	0.954078683	0.999527751	ENSG00000120322	PCDHB8
11.78472539	-0.14181749	0.918059449	-0.15447528	0.877235001	0.999467288	ENSG00000204444	APOM
11.53325889	-0.463769749	0.922198306	-0.502895903	0.615037462	0.99483168	ENSG00000146233	CYP39A1

11.52119119	1.255993635	0.870516109	1.442814926	0.149072613	0.936396974	ENSG00000120278	PLEKHG1
11.51315187	0.370361222	0.916286429	0.404198087	0.68606706	0.998869317	ENSG00000232818	RPS2P32
11.89699065	0.646786784	0.927992516	0.696974138	0.485818979	0.992923464	ENSG00000279413	AC112497.1
11.79702952	-0.613447802	0.833429584	-0.736052348	0.461698841	0.99135833	ENSG00000223478	AL441992.1
11.69537126	-0.002275008	0.930992482	-0.002443638	0.998050261	0.999527751	ENSG00000139044	B4GALNT3
12.12663192	-1.023640686	0.868332593	-1.178857841	0.238454788	0.962465655	ENSG00000226711	FAM66C
11.56568482	-1.021954187	1.330190132	-0.768276777	0.442322769	0.99135833	ENSG00000277734	TRAC
11.48548673	-0.228124925	0.907372942	-0.251412528	0.801495183	0.999467288	ENSG00000154930	ACSS1
11.83829296	-0.070952492	0.826437455	-0.085853433	0.93158293	0.999527751	ENSG00000167604	NFKBID
11.33041911	0.392562969	1.24439601	0.315464664	0.752408877	0.999467288	ENSG00000269825	AC022150.4
11.71760301	0.306739347	0.916590307	0.334652619	0.737887147	0.999467288	ENSG00000066185	ZMYND12
11.14897633	-0.406388118	1.029377271	-0.394790257	0.692997686	0.998869317	ENSG00000246575	AC093162.2
11.99777913	-0.3813395	0.985122943	-0.387098385	0.698683373	0.998869317	ENSG00000285793	AC125232.2
11.44453212	0.905910191	1.047179184	0.865095683	0.386986274	0.986799595	ENSG00000088538	DOCK3
11.47771792	-0.751106762	0.959274252	-0.782994812	0.433630158	0.991301167	ENSG00000204228	HSD17B8
11.57593531	1.050580663	1.447769569	0.725654611	0.468050533	0.99135833	ENSG00000175556	LONRF3
11.6976131	-0.775817744	0.975900706	-0.794976107	0.426627401	0.991301167	ENSG00000174672	BRSK2
11.42229874	-0.387630429	0.949506474	-0.408244114	0.683094464	0.998869317	ENSG00000214425	LRRC37A4P
11.67484634	0.78302227	1.057286699	0.740595971	0.458938452	0.99135833	ENSG00000100368	CSF2RB
11.56303361	-1.146616945	0.915326386	-1.252686432	0.210319846	0.954310328	ENSG00000188672	RHCE
11.35283534	-1.77328316	1.195604447	-1.483168755	0.13802958	0.934144427	ENSG00000000938	FGR
11.35998713	2.971203709	1.155566089	2.57121054	0.010134369	0.630908184	ENSG00000196550	FAM72A
11.28438617	1.745848746	0.994992755	1.754634632	0.079321824	0.897052166	ENSG00000196912	ANKRD36B
11.57760383	-0.785185103	0.850030426	-0.923714116	0.355635181	0.978364583	ENSG00000132330	SCLY
11.55520419	-0.281947351	0.907912667	-0.310544572	0.75614687	0.999467288	ENSG00000144648	ACKR2
11.02826537	1.536265878	1.283731139	1.196719337	0.231415967	0.959575179	ENSG00000091513	TF
11.70599426	0.973960924	0.812596375	1.198578966	0.230691701	0.959575179	ENSG00000179406	LINC00174
11.75581729	-1.052165149	0.833165471	-1.262852562	0.206642171	0.954310328	ENSG00000235636	NUS1P1
11.4313365	1.020020384	0.931808869	1.094666962	0.273662568	0.962465655	ENSG00000241343	RPL36A
11.10432364	-0.558870509	1.036362792	-0.539261457	0.58970646	0.99483168	ENSG00000285533	AP001362.2
11.37043605	1.158444687	1.283066958	0.902871577	0.366594057	0.981269214	ENSG00000177675	CD163L1
11.55869329	-0.130006185	1.24863652	-0.104118519	0.917075295	0.999527751	ENSG00000168229	PTGDR
11.77515324	-2.327711724	1.128190755	-2.063225314	0.039091226	0.793985738	ENSG00000262089	AC040977.1
11.54886479	0.661469148	1.020647203	0.648087944	0.516928071	0.993513219	ENSG00000136449	MYCBPAP
11.28309007	0.331690223	0.984548394	0.336895805	0.736195448	0.999467288	ENSG00000186481	ANKRD20A5P
11.2656766	-0.225112145	0.86853608	-0.259185715	0.795491952	0.999467288	ENSG00000267809	NDUFV2P1
11.68634345	2.083012392	0.915175883	2.276078765	0.022841296	0.744905651	ENSG00000284747	AL034417.4
11.59589023	-0.6584638	1.007476097	-0.653577591	0.513383986	0.993513219	ENSG00000224183	SDHDP6
11.13151349	0.889727329	1.030038655	0.863780524	0.387708469	0.986799595	ENSG00000181773	GPR3
11.33937808	0.113908479	0.921847	0.123565493	0.901659315	0.999527751	ENSG00000226015	CCT8P1
11.49884496	-0.071933926	0.894671305	-0.08040263	0.935917035	0.999527751	ENSG00000266094	RASSF5
11.42975639	0.157377714	0.926725326	0.169821315	0.865150663	0.999467288	ENSG00000224080	UBE2FP1
10.75212061	0.776766273	1.379559892	0.563053679	0.573398332	0.99483168	ENSG00000114541	FRMD4B
11.40125972	1.691510144	1.300237716	1.300923765	0.193284551	0.950382812	ENSG00000198838	RYR3
11.49918421	1.331825085	1.001646655	1.329635633	0.183638352	0.949094286	ENSG00000234409	CCDC188
10.88961719	-1.515675551	1.13345616	-1.337215858	0.181152187	0.949094286	ENSG00000049089	COL9A2
11.55897169	0.948688825	1.159841661	0.817946843	0.413387541	0.990226751	ENSG00000143028	SYPL2
11.43287247	0.136175184	1.126662762	0.120865967	0.903797199	0.999527751	ENSG00000196208	GREB1
11.68396048	-1.04766648	0.901974615	-1.16152546	0.245428275	0.962465655	ENSG00000112293	GPLD1
11.60666081	-1.146483272	1.00407553	-1.14182971	0.25352481	0.962465655	ENSG00000131831	RAI2
11.53844378	-1.393564031	1.102429776	-1.264084173	0.206199817	0.954310328	ENSG00000189420	ZFP92
11.27200941	0.212210969	0.863179674	0.245847968	0.805799923	0.999467288	ENSG00000237410	AP001092.1
11.0601852	1.420959317	0.902434388	1.574584631	0.115352353	0.923922314	ENSG00000174370	C11orf45
11.45601603	-0.368688174	0.751695637	-0.490475342	0.623797574	0.99483168	ENSG00000230555	AL450326.1
11.4346143	1.212381925	0.993392283	1.220446288	0.222295739	0.956884001	ENSG00000259479	SORD2P
11.48394268	-0.273503406	0.817312812	-0.334637366	0.737898655	0.999467288	ENSG00000214185	XPOTP1
11.22739287	1.135591032	0.863355199	1.315323095	0.18840134	0.949094286	ENSG00000227001	NBPF2P
11.59431131	0.320106636	0.980605015	0.326437894	0.744093071	0.999467288	ENSG00000163449	TMEM169
11.49036079	1.164421219	1.268958245	0.917619806	0.358817945	0.979465649	ENSG00000164161	HHIP
11.05915553	0.154637935	1.152269044	0.134202976	0.893242073	0.999467288	ENSG00000237125	HAND2-AS1
11.28363211	1.360863822	1.068901217	1.273142738	0.202967396	0.954310328	ENSG00000235314	LINC00957
10.84974981	-0.184409531	1.074695306	-0.171592385	0.863757994	0.999467288	ENSG00000156049	GNA14
11.08686194	-0.168832542	1.197715327	-0.140962162	0.887899834	0.999467288	ENSG00000136842	TMOD1
11.17958356	-2.600167384	1.262636632	-2.059315656	0.03946401	0.793985738	ENSG00000265190	ANXA8

11.48061218	3.724380731	1.145519445	3.251259285	0.00114895	0.244243321	ENSG00000075826	SEC31B
11.04030203	-1.537257832	1.146501818	-1.340824591	0.17997741	0.948583952	ENSG00000257261	AC008014.1
11.60438973	-1.259408391	1.06417721	-1.183457397	0.236627921	0.962465655	ENSG00000140839	CLEC18B
11.26942764	-0.063717632	1.184092725	-0.053811353	0.957085464	0.999527751	ENSG00000065325	GLP2R
11.07197636	-2.108466804	0.945952512	-2.228935148	0.02581822	0.762561457	ENSG00000158769	F11R
11.01046822	0.968919109	1.369069741	0.707720783	0.479118669	0.99135833	ENSG00000143850	PLEKHA6
11.05009197	-0.993893621	0.899229586	-1.105272376	0.269041584	0.962465655	ENSG00000181218	HIST3H2A
11.08949227	1.594694246	1.052493441	1.51515837	0.129732303	0.929373153	ENSG00000243415	AC107021.1
10.77780443	1.027793747	1.030982686	0.996906894	0.318809705	0.974768742	ENSG00000137460	FHDC1
11.13424974	-1.16356721	1.323875143	-0.878910082	0.379450032	0.983256509	ENSG00000124772	CPNE5
11.06796484	0.333374283	0.884886641	0.376742362	0.706365078	0.99900742	ENSG00000204934	ATP6V0E2-AS1
11.21248159	-0.610476372	0.892715135	-0.683842301	0.494074752	0.993513219	ENSG00000178947	SMIM10L2A
11.24142397	-0.018367085	0.980600436	-0.018730448	0.985056139	0.999527751	ENSG00000168481	LGI3
11.00162924	0.912481731	0.838981364	1.087606674	0.276768783	0.962465655	ENSG00000255224	AC109322.1
10.90898489	-0.519415652	1.251296211	-0.415102074	0.678067181	0.998869317	ENSG00000165449	SLC16A9
11.34460125	-0.598919613	0.852516791	-0.702531164	0.482347974	0.991538826	ENSG00000180764	PIPSL
10.87119161	-0.695664148	0.993067343	-0.700520617	0.483602234	0.991892545	ENSG00000139200	PIANP
10.71325054	-0.803469048	1.093212934	-0.734961161	0.462363148	0.99135833	ENSG00000258701	LINC00638
11.25575444	-1.109841724	0.836305486	-1.32707694	0.184483233	0.949094286	ENSG00000247240	UBL7-AS1
11.22768083	2.079111683	0.845906984	2.457849056	0.013977191	0.677424718	ENSG00000169246	NPIPB3
11.43950467	-0.553554582	1.037052728	-0.5337767	0.593496036	0.99483168	ENSG00000274615	AC233968.1
11.41294372	0.283674088	0.867658632	0.326942045	0.74371172	0.999467288	ENSG00000185774	KCNIP4
11.70388593	0.523886711	1.399365102	0.374374572	0.708125658	0.99900742	ENSG00000243836	WDR86-AS1
10.97754317	-0.742650768	0.863015043	-0.860530502	0.389496677	0.987216662	ENSG00000228801	AC064807.1
11.13475079	0.291398873	0.844287624	0.345141708	0.729987839	0.999467288	ENSG00000235387	SPAAR
10.99609887	0.150668526	1.085370816	0.138817558	0.889594319	0.999467288	ENSG00000111325	OGFOD2
10.90940402	0.407151681	1.242032965	0.327810688	0.743054806	0.999467288	ENSG00000184564	SLITRK6
11.2033549	-1.054529001	1.222847794	-0.862355075	0.388492154	0.986799595	ENSG00000279145	AC011912.1
11.49636952	0.69809519	0.959762627	0.72736234	0.467004018	0.99135833	ENSG00000189136	UBE2Q2P1
11.22181036	-0.341576443	0.925291164	-0.369155631	0.712011725	0.99900742	ENSG00000261659	Z92544.2
11.06114938	-0.121778748	0.958270886	-0.127081757	0.89887569	0.999527751	ENSG00000266916	ZNF793-AS1
11.10496533	0.156393008	0.942650544	0.165907726	0.868229567	0.999467288	ENSG00000159905	ZNF221
11.03901853	-0.270298903	0.834165047	-0.324035278	0.745911326	0.999467288	ENSG00000063127	SLC6A16
11.02076238	2.005241948	1.197437261	1.674611283	0.094010524	0.921147998	ENSG00000258405	ZNF578
11.43967626	0.762926291	1.062029631	0.718366295	0.472531463	0.99135833	ENSG00000231064	AC234582.1
11.2903996	0.681389081	1.015431212	0.67103421	0.502198736	0.993513219	ENSG00000279227	AC009303.4
10.75412863	-0.35686881	1.224574003	-0.291422821	0.770727963	0.999467288	ENSG00000168032	ENTPD3
11.20855237	0.313336403	0.971572774	0.322504306	0.747070676	0.999467288	ENSG00000197479	PCDHB11
10.74265477	-0.193041983	1.093149487	-0.176592484	0.859828498	0.999467288	ENSG00000155846	PPARGC1B
10.85572229	-0.431621144	0.976634756	-0.441947352	0.658527304	0.997568624	ENSG00000228283	KATNBL1P6
10.79049977	-0.412963828	1.017566876	-0.405834582	0.684864147	0.998869317	ENSG00000273084	AC092171.5
10.95872067	0.627043835	0.888479062	0.705749704	0.480343805	0.99135833	ENSG00000221947	XKR9
11.4164332	-1.340716354	1.108287115	-1.209719337	0.226386607	0.958865115	ENSG00000151023	ENKUR
11.17292726	0.122361367	0.89688452	0.136429344	0.891481877	0.999467288	ENSG00000278784	AL136295.7
11.05545984	1.038417891	1.026949756	1.011167182	0.31193642	0.973427483	ENSG00000261270	AC012181.2
10.76757776	-0.413832635	1.117787047	-0.370224934	0.7112149	0.99900742	ENSG00000154102	C16orf74
11.07698376	-0.16582171	0.854694349	-0.194012877	0.846165799	0.999467288	ENSG00000259877	AC009113.1
11.09617389	1.872007511	0.993820371	1.883647755	0.059612638	0.858410096	ENSG00000263731	AC145207.5
11.12650937	0.680341314	1.032042509	0.659218305	0.509755594	0.993513219	ENSG00000213707	HMGB1P10
10.62102148	-1.974614978	1.033931173	-1.90981279	0.056157322	0.847598083	ENSG00000233521	LINC01638
10.64429827	-0.907140798	0.914091034	-0.99239656	0.321004116	0.975337095	ENSG00000236682	AC068282.1
11.20192347	1.035085299	1.355318013	0.763721347	0.445033317	0.99135833	ENSG00000135917	SLC19A3
10.7826602	-0.43132979	0.928796321	-0.464396531	0.642363675	0.996740283	ENSG00000135525	MAP7
10.9847625	0.803170905	0.896115312	0.896280751	0.370102837	0.981455968	ENSG00000205085	FAM71F2
10.88554297	-0.572869411	0.937704661	-0.610927336	0.541247687	0.99483168	ENSG00000233382	NKAPP1
11.15333959	0.330052445	1.019049311	0.323882703	0.74602684	0.999467288	ENSG00000261087	AP003469.4
10.52666519	-1.729519251	1.216716057	-1.421464968	0.155181631	0.939432741	ENSG00000165140	FBP1
10.6394632	1.411260821	0.931275605	1.515406195	0.12966957	0.929373153	ENSG00000285796	AL162458.1
10.43702775	-1.586383857	1.044885786	-1.518236613	0.128954761	0.928627271	ENSG00000186818	LILRB4
11.93149819	1.473885057	1.573783821	0.936523198	0.349003874	0.977691382	ENSG00000204334	ERICH2
10.40639955	0.443429426	0.98848449	0.448595229	0.653723676	0.997527562	ENSG00000145088	EAF2
10.55358755	-0.701673549	1.243551354	-0.564249757	0.57258417	0.99483168	ENSG00000230438	SERPINB9P1
10.89568594	0.800975489	0.982433219	0.815297644	0.414901957	0.990226751	ENSG00000272316	AL021368.2
10.76939914	2.033422341	0.865887979	2.34836652	0.018855956	0.726053908	ENSG00000105982	RNF32

10.98453045	1.873218335	1.377813877	1.359558331	0.173969732	0.94495857	ENSG00000155897	ADCY8
11.00673362	-1.288701326	1.012782511	-1.272436394	0.20321811	0.954310328	ENSG00000278156	TSC22D1-AS1
10.54871938	1.546402567	1.270874091	1.216802339	0.22367943	0.956884001	ENSG00000172575	RASGRP1
11.14306787	0.253393305	1.030460637	0.245902945	0.805757364	0.999467288	ENSG00000217555	CKLF
10.85841331	-0.815855675	0.945144904	-0.863206977	0.388023678	0.986799595	ENSG00000183153	GJD3
10.73451788	0.114718966	0.789365855	0.14533054	0.884449903	0.999467288	ENSG00000280852	AC025048.6
11.02137289	0.571019506	0.925662078	0.616876849	0.537315958	0.99483168	ENSG00000127533	F2RL3
10.54086615	-1.997586451	1.523221686	-1.311422014	0.189715225	0.949094286	ENSG00000162552	WNT4
10.73975614	-1.272061972	0.99793112	-1.274699171	0.20241574	0.954310328	ENSG00000018625	ATP1A2
11.25637418	-0.336253669	0.955336606	-0.351974023	0.724857744	0.999456245	ENSG00000200033	RNU6-403P
10.48258286	0.091622662	1.207255405	0.075893354	0.939503945	0.999527751	ENSG00000237510	GPAT2P1
10.85355859	2.332551397	0.910179743	2.562737103	0.010385065	0.635400047	ENSG00000135976	ANKRD36
11.47317378	-0.036248968	1.211788285	-0.029913615	0.976135948	0.999527751	ENSG00000222032	AC112721.2
10.43243932	-0.430622676	0.996217419	-0.432257726	0.665554108	0.997568624	ENSG00000178917	ZNF852
10.82017013	-0.805561028	0.831821841	-0.968429762	0.332829782	0.976026345	ENSG00000273117	AC144652.1
10.332116	0.154606673	1.288271999	0.120010893	0.904474519	0.999527751	ENSG00000245522	AC026250.1
10.62813332	-0.141378174	0.884998323	-0.159749652	0.873078287	0.999467288	ENSG00000180425	C11orf71
10.78657854	-1.374402365	0.930498119	-1.477060873	0.139659321	0.935644597	ENSG00000152804	HHEX
10.5736242	0.939511722	0.853204093	1.101157074	0.270828301	0.962465655	ENSG00000249628	LINC00942
10.6380981	0.42238996	0.85983628	0.491244635	0.623253433	0.99483168	ENSG00000036530	CYP46A1
10.58659207	-1.291445128	0.991722804	-1.30222389	0.192839861	0.949321643	ENSG00000245975	AC090515.2
10.65274604	0.496551933	1.04056433	0.477194844	0.633223382	0.995281893	ENSG00000157368	IL34
10.74965283	-0.779813454	1.020273363	-0.764318154	0.444677669	0.99135833	ENSG00000265666	RARA-AS1
11.18544656	0.377007412	1.278714949	0.294833037	0.768121428	0.999467288	ENSG00000101670	LIPG
10.78817782	0.558320654	0.941790227	0.592829101	0.55329553	0.99483168	ENSG00000125731	SH2D3A
10.55594546	-0.729570244	1.205845376	-0.605028023	0.545160377	0.99483168	ENSG00000050628	PTGER3
10.38787703	-0.088815643	0.954246153	-0.093074143	0.925844659	0.999527751	ENSG00000285184	AC244033.2
10.51388616	0.119863112	0.856648214	0.139921043	0.888722375	0.999467288	ENSG00000143450	OAZ3
10.58670041	-0.193012807	1.098328129	-0.175733282	0.860503487	0.999467288	ENSG00000285053	TBCE
10.6438527	0.28879361	1.062464871	0.271814737	0.785764473	0.999467288	ENSG00000284879	AC133644.3
10.41309307	-0.210553934	1.0287924	-0.204661246	0.837836801	0.999467288	ENSG00000248932	AC097103.2
10.36751842	-0.202692343	0.87872331	-0.230666856	0.817573624	0.999467288	ENSG00000184100	BRD7P2
10.57452214	1.343428011	1.051344564	1.277818954	0.20131327	0.954310328	ENSG00000156234	CXCL13
10.64442157	0.447188655	0.91862081	0.486804403	0.62639695	0.99483168	ENSG00000164142	FAM160A1
10.50689865	0.388985036	0.886679931	0.438698364	0.660880112	0.997568624	ENSG00000228649	SNHG26
11.17504569	1.377718764	1.092324913	1.261271941	0.207210887	0.954310328	ENSG00000174469	CNTNAP2
10.42150916	-0.84816829	0.998338298	-0.849580039	0.395558614	0.988378925	ENSG00000147509	RGS20
10.28730281	-0.139467705	0.916728758	-0.152136282	0.879079448	0.999467288	ENSG00000254682	AP002387.1
10.78735302	-0.823096884	0.903842949	-0.910663611	0.362472643	0.980189549	ENSG00000277879	AL391988.1
10.90595632	-0.349746107	0.992885828	-0.352252089	0.724649216	0.999456245	ENSG00000277559	AC018553.1
10.73912207	-0.233889651	0.858918644	-0.272307107	0.785385892	0.999467288	ENSG00000099974	DDTL
10.3041595	0.55609507	0.979443318	0.567766465	0.570193554	0.99483168	ENSG00000276256	AC011043.1
10.21917286	-0.234536711	1.01887662	-0.230191474	0.817942987	0.999467288	ENSG00000117013	KCNQ4
10.82537593	-0.600810836	0.892361987	-0.673281521	0.500768206	0.993513219	ENSG00000273382	AL356488.3
10.26131416	-0.57396194	0.968309806	-0.592746181	0.55335103	0.99483168	ENSG00000280649	AC245100.8
10.44378437	-0.778299256	0.914899779	-0.850693456	0.394939659	0.988378925	ENSG00000230154	AC018463.1
10.45388031	-1.291106006	0.981287963	-1.315725918	0.188266052	0.949094286	ENSG00000224424	PRKAR2A-AS1
10.39005262	0.231463663	1.117832919	0.207064633	0.835959382	0.999467288	ENSG00000164122	ASB5
10.36627441	-0.160622477	0.852282274	-0.188461596	0.850514818	0.999467288	ENSG00000271646	AC099343.3
10.58925225	0.748483566	0.949123213	0.788605268	0.430342751	0.991301167	ENSG00000178015	GPR150
10.31402484	0.85050822	0.996069197	0.853864594	0.39318002	0.988378925	ENSG00000250510	GPR162
10.62259975	-1.31502134	1.100139338	-1.195322533	0.231961039	0.959575179	ENSG00000135094	SDS
10.80943725	0.573433857	1.111190852	0.516053436	0.605817089	0.99483168	ENSG00000182870	GALNT9
10.64261074	-0.446734897	0.916541564	-0.487413681	0.6259652	0.99483168	ENSG00000278922	AC002310.6
10.71307159	0.393566208	0.811501407	0.484985244	0.627686813	0.99483168	ENSG00000264575	LINC00526
10.39916916	0.097249084	0.878695336	0.110674405	0.911874543	0.999527751	ENSG00000267279	AC090409.1
10.45577032	-0.801154103	1.181596001	-0.678027094	0.497754514	0.993513219	ENSG00000267213	AC007773.1
10.14870822	2.234151279	1.148897457	1.9446046	0.051822585	0.845356848	ENSG00000144354	CDCA7
10.46073705	-0.615880614	0.948947092	-0.649014702	0.516328874	0.993513219	ENSG00000235257	ITGA9-AS1
10.14872795	0.476286067	0.958142264	0.497093266	0.619123283	0.99483168	ENSG00000164898	FMC1
10.31046439	0.695018788	0.909311289	0.764335378	0.444667408	0.99135833	ENSG00000269899	AC025857.2
10.12822747	-0.778171491	1.023028162	-0.760655004	0.446863157	0.99135833	ENSG00000168490	PHYHIP
10.44075353	-0.582657869	0.879621604	-0.662396043	0.507717439	0.993513219	ENSG00000180938	ZNF572
10.32794958	0.286680455	1.087775576	0.263547427	0.792128673	0.999467288	ENSG00000198963	RORB

10.0043927	0.80788416	0.998213734	0.809329838	0.418325447	0.990226751	ENSG00000165113	GKAP1
10.56199839	-2.203074789	0.975359997	-2.258729901	0.023900191	0.744948012	ENSG00000228623	ZNF883
10.0292868	0.641262076	1.223998245	0.523907676	0.600342756	0.99483168	ENSG00000169413	RNASE6
10.56251243	-1.221594395	0.840448922	-1.453502246	0.146084357	0.936017041	ENSG00000168961	LGALS9
10.77373141	1.508499873	1.041783499	1.447997472	0.147617758	0.936396974	ENSG00000221995	TIAF1
10.53926555	-1.988586781	1.215602418	-1.635885839	0.101863482	0.92169898	ENSG00000173868	PHOSPHO1
10.58475786	1.67570987	0.954903084	1.754848107	0.079285292	0.897052166	ENSG00000160951	PTGER1
10.3754657	0.52966131	0.917816612	0.577088389	0.563879747	0.99483168	ENSG00000100285	NEFH
10.37963579	0.864444113	0.830638988	1.040697735	0.298015855	0.972186492	ENSG00000240731	AL139287.1
10.2476757	0.598540734	0.894396015	0.66921221	0.503360119	0.993513219	ENSG00000188739	RBM34
10.19683111	-2.145478549	1.173453383	-1.828345788	0.067497672	0.87420149	ENSG00000050730	TNIP3
10.49450631	1.369661158	1.051041241	1.303146921	0.192524607	0.949261383	ENSG00000214922	HLA-F-AS1
10.85114931	0.499719042	1.260605985	0.396411764	0.691801289	0.998869317	ENSG00000102287	GABRE
10.0025419	0.004272419	1.386201841	0.003082105	0.99754084	0.999527751	ENSG00000166793	YPEL4
10.41510228	0.451957746	0.901564981	0.501303573	0.616157491	0.99483168	ENSG00000229373	LINC00452
10.40888233	0.534740979	0.917999313	0.582506949	0.560225262	0.99483168	ENSG00000260136	AC008915.2
10.34334476	-0.317831411	0.821042949	-0.387106924	0.698677052	0.998869317	ENSG00000281468	AC006504.8
10.1652776	0.636982203	0.848301873	0.750890954	0.452718285	0.99135833	ENSG00000273899	NOL12
10.07200551	1.154298503	1.348037217	0.856280886	0.391842431	0.988378925	ENSG00000159189	C1QC
10.45341666	-1.585583598	0.938114511	-1.690181293	0.090993276	0.912649921	ENSG00000235912	AL031729.1
10.15492039	-1.259239747	1.003628867	-1.254686656	0.209592532	0.954310328	ENSG00000143570	SLC39A1
10.50964262	3.239501846	1.06816946	3.032760219	0.002423281	0.360084787	ENSG00000015479	MATR3
10.26358694	-0.505606436	0.968083475	-0.52227566	0.601478414	0.99483168	ENSG00000240859	AC093627.4
9.991953223	-0.088297511	1.09839439	-0.080387802	0.935928828	0.999527751	ENSG00000230333	AC004160.1
10.38872122	-0.608652352	0.974248642	-0.624740263	0.532141543	0.99483168	ENSG00000165092	ALDH1A1
9.91800979	1.743498287	1.51522883	1.150650155	0.249876191	0.962465655	ENSG00000129173	E2F8
10.5591098	1.585795905	1.281876647	1.237089316	0.216053926	0.954563123	ENSG00000243742	RPLP0P2
10.49771099	-0.471944233	1.089699	-0.433095959	0.664945059	0.997568624	ENSG00000246523	AP001528.1
10.6079413	-1.24800482	1.281022829	-0.974225276	0.329944697	0.976026345	ENSG00000159713	TPPP3
9.879040088	0.188103772	1.115623886	0.168608591	0.866104522	0.999467288	ENSG00000260456	C16orf95
10.35552944	0.904397838	1.037230226	0.871935483	0.383243575	0.985655313	ENSG00000266709	MGC12916
10.37299229	0.293582874	0.935309253	0.313888559	0.75360568	0.999467288	ENSG00000183570	PCBP3
10.12298544	-0.07395049	0.893983247	-0.082720219	0.934074008	0.999527751	ENSG00000284308	C2orf81
10.29681986	0.397166966	1.00853113	0.393807345	0.693723281	0.998869317	ENSG00000171121	KCNMB3
10.40605931	-0.416273416	1.047974365	-0.397217174	0.691207319	0.998869317	ENSG00000164283	ESM1
10.06158098	-1.680897525	0.951155967	-1.767215456	0.077192152	0.897052166	ENSG00000216819	TUBB2BP1
10.44993187	0.67460734	0.911375335	0.740208029	0.459173777	0.99135833	ENSG00000213760	ATP6V1G2
10.11821955	0.906837261	1.003300405	0.903854177	0.36607273	0.981269214	ENSG00000124593	AL365205.1
10.04045045	0.004632417	1.173750386	0.00394668	0.996851014	0.999527751	ENSG00000233237	LINC00472
10.24507629	2.189668267	1.043398539	2.098592421	0.035852845	0.785058983	ENSG00000146678	IGFBP1
9.573553292	0.184149697	1.326742945	0.138798324	0.889609519	0.999467288	ENSG00000197506	SLC28A3
10.05359141	-0.423088135	0.906198898	-0.4668822	0.640584161	0.996553586	ENSG00000151062	CACNA2D4
10.05058337	0.245215997	1.001684533	0.244803617	0.806608493	0.999467288	ENSG00000250451	HOXC-AS1
10.16716986	0.54079105	0.885445411	0.610755946	0.541361162	0.99483168	ENSG00000260329	AC007541.1
10.03076619	0.302127087	1.057527264	0.285692008	0.775114026	0.999467288	ENSG00000184047	DIABLO
10.09161015	-1.457833446	0.975684953	-1.494164116	0.135132729	0.933741583	ENSG00000133958	UNC79
10.19194966	-1.210847614	0.897326263	-1.349395046	0.177210111	0.946540546	ENSG00000259838	ELOCP2
10.13040552	1.502244942	1.039750241	1.444813267	0.148510344	0.936396974	ENSG00000246922	UBAP1L
10.57279718	1.947583203	1.175521865	1.656781775	0.097563603	0.921147998	ENSG00000259972	AC009120.2
10.35969753	0.049510081	0.961974849	0.051467126	0.958953297	0.999527751	ENSG00000121075	TBX4
10.08460645	-0.285226263	0.959031739	-0.29741066	0.766153006	0.999467288	ENSG00000226686	LINC01535
10.0442841	0.151637185	1.040756341	0.145699026	0.88415899	0.999467288	ENSG00000183615	FAM167B
9.922656713	0.602100606	1.066014028	0.564814899	0.572199672	0.99483168	ENSG00000121577	POPDC2
10.02534523	-0.596575768	0.840469863	-0.709812207	0.477820598	0.99135833	ENSG00000251580	LINC02482
10.10637211	-1.512122062	0.942175328	-1.604926405	0.108510002	0.923922314	ENSG00000272523	LINC01023
9.911049305	0.717902034	1.043177786	0.688187617	0.491334641	0.993513219	ENSG00000233452	STXBP5-AS1
10.0888766	-0.003959981	0.901462723	-0.004392839	0.996495033	0.999527751	ENSG00000178460	MCMDC2
10.11480703	1.903530123	1.039397697	1.831378046	0.067044136	0.87420149	ENSG00000204778	CBWD4P
9.971606253	-0.416524706	0.894678614	-0.465557911	0.641531976	0.996566167	ENSG00000255284	AP006621.3
10.41959296	-1.858781046	1.136469018	-1.635575644	0.101928431	0.92169898	ENSG00000166927	MS4A7
9.804678565	-0.386514542	1.259372228	-0.306910486	0.758911508	0.999467288	ENSG00000135477	KRT87P
10.01937033	1.593958337	1.058273245	1.506187883	0.132018949	0.932626545	ENSG00000257167	TMPO-AS1
10.36225562	0.085763885	0.946457356	0.090615689	0.927797965	0.999527751	ENSG00000158113	LRRC43
9.847480724	-0.478416905	1.080909808	-0.442605758	0.658050919	0.997568624	ENSG00000283689	AC018553.2

10.04059044	-0.472650672	1.195045493	-0.395508518	0.692467636	0.998869317	ENSG00000104903	LYL1
10.75887427	-0.839556542	1.353861375	-0.620120019	0.535178773	0.99483168	ENSG00000133519	ZDHHC8P1
10.22516232	-0.09901953	0.898769447	-0.110172336	0.912272701	0.999527751	ENSG00000143226	FCGR2A
9.842910969	0.843102299	0.974015742	0.865594119	0.386712782	0.986711841	ENSG00000134326	CMPK2
9.529822519	1.815724267	1.553902714	1.168492886	0.242608007	0.962465655	ENSG00000158125	XDH
10.15670673	-0.339212026	1.059133901	-0.320273033	0.748761364	0.999467288	ENSG00000138483	CCDC54
9.821755303	-0.181835853	0.927818532	-0.195982131	0.844624152	0.999467288	ENSG00000246090	AP002026.1
9.958725245	1.083462556	0.911685807	1.188416609	0.234669316	0.961085539	ENSG00000272221	AL645933.2
9.992364795	-0.795708814	1.358375192	-0.585779848	0.558023467	0.99483168	ENSG00000122574	WIPF3
10.13253858	-1.185819089	1.08590788	-1.092007076	0.274829991	0.962465655	ENSG00000172476	RAB40A
9.703309677	-0.600960582	1.301277956	-0.461823378	0.644207984	0.997332006	ENSG00000221818	EBF2
9.954486113	1.340077062	0.95618684	1.401480344	0.161070481	0.942888225	ENSG00000224594	RPL29P19
9.816154818	0.137769249	1.198302073	0.114970384	0.908468596	0.999527751	ENSG00000214376	VSTM5
9.968232745	-0.108250089	0.915499706	-0.118241534	0.905876282	0.999527751	ENSG00000263050	AC090617.5
9.84005462	-0.078885639	1.231491379	-0.064056997	0.948924843	0.999527751	ENSG00000149599	DUSP15
9.832373911	-1.199348189	0.957672019	-1.252357973	0.210439453	0.954310328	ENSG00000183569	SERHL2
9.848500604	-0.874283898	1.447727873	-0.603900716	0.545909653	0.99483168	ENSG00000204632	HLA-G
10.16818782	0.058102474	1.094035461	0.053108401	0.957645538	0.999527751	ENSG00000250474	WBP1LP2
9.752686813	1.660830615	1.077036907	1.542036864	0.123064636	0.927012392	ENSG00000261542	AC011978.2
10.27024288	-0.136940587	0.955835647	-0.143267922	0.886078589	0.999467288	ENSG00000226864	ATE1-AS1
9.983328409	-0.233005652	1.250697595	-0.186300552	0.852209075	0.999467288	ENSG00000111664	GNB3
9.06045423	2.140370769	1.70350807	1.256448858	0.208953277	0.954310328	ENSG00000167941	SOST
9.914943011	0.257028087	0.875151862	0.293695412	0.768990661	0.999467288	ENSG00000088836	SLC4A11
9.970333771	1.231840915	0.937909957	1.313389314	0.189051796	0.949094286	ENSG00000182841	RRP7BP
9.725055725	2.953740895	1.177923669	2.507582598	0.012156017	0.648356243	ENSG00000157542	KCNJ6
9.851250508	-0.541999129	0.940672157	-0.576182812	0.564491622	0.99483168	ENSG00000278817	AC007325.4
9.379112744	-1.467451343	1.382144847	-1.061720373	0.288362648	0.966288987	ENSG00000204060	FOXO6
10.19110858	-0.073521087	1.210496514	-0.060736306	0.951569217	0.999527751	ENSG00000203747	FCGR3A
9.418955271	-1.290636337	1.290390764	-1.000190309	0.317218418	0.974768742	ENSG00000171303	KCNK3
9.968134237	-0.109255979	1.161852117	-0.094036046	0.925080523	0.999527751	ENSG00000280916	FOXCUT
9.944873694	-0.977238163	0.908641174	-1.075494035	0.282153602	0.963267117	ENSG00000279881	AC041040.1
9.910174397	1.080918573	1.475837326	0.732410377	0.463918127	0.99135833	ENSG00000231345	BEND3P1
10.29849656	0.390571277	0.988125083	0.395265016	0.692647315	0.998869317	ENSG00000054690	PLEKHH1
9.968255438	0.763142322	0.975744336	0.782112993	0.434148172	0.991301167	ENSG00000119703	ZC2HC1C
9.672498692	0.271473695	1.117630759	0.242901059	0.808082045	0.999467288	ENSG00000271780	AL118558.3
9.797841889	0.122385557	0.926181348	0.13213995	0.894873597	0.999467288	ENSG00000232838	PET117
10.26176593	-0.088612251	1.141094103	-0.077655515	0.938102081	0.999527751	ENSG00000261342	AC006538.1
9.763021815	0.402225109	0.912636997	0.440728472	0.659409582	0.997568624	ENSG00000268658	LINC00664
10.02259717	2.440766134	1.398662364	1.745071718	0.080972386	0.897807954	ENSG00000261326	LINC01355
9.515902149	3.342657955	1.988042482	1.681381552	0.092688826	0.921147998	ENSG00000007968	E2F2
9.817340205	1.81367363	1.308280823	1.386303	0.165654401	0.943223078	ENSG00000127423	AUNIP
9.591619072	-0.299827053	1.132380694	-0.264775843	0.791182147	0.999467288	ENSG00000186453	FAM228A
9.754352352	0.756647458	0.891943004	0.848313687	0.396263297	0.988378925	ENSG00000280374	AC019080.5
10.2570406	-0.50041943	1.260501023	-0.397000416	0.691367154	0.998869317	ENSG00000124406	ATP8A1
9.495752456	-1.444046059	1.050550623	-1.374561136	0.169267544	0.944276223	ENSG00000235961	PNMA6A
10.0164287	0.500233109	1.092854843	0.457730606	0.647145997	0.997368416	ENSG00000272010	AC100814.1
9.843723087	-0.806137901	0.983273791	-0.819850899	0.412301111	0.990226751	ENSG00000165695	AK8
9.846841066	-0.380028816	1.051561088	-0.361394902	0.717804259	0.99900742	ENSG00000236264	RPL26P30
9.852398272	-0.684099658	0.89829498	-0.761553469	0.446326555	0.99135833	ENSG00000129535	NRL
9.66390729	-2.034803637	1.483429344	-1.371688949	0.17016029	0.944422693	ENSG00000100628	ASB2
9.680973431	-0.963759578	1.11012458	-0.868154435	0.385309795	0.986047049	ENSG00000183632	TP53TG3
9.794472022	0.155630299	0.910832953	0.170865907	0.864329204	0.999467288	ENSG00000275734	AC010538.1
10.16401913	1.15414694	1.079941873	1.068712093	0.285199421	0.964493786	ENSG00000276851	AC002401.4
9.716037924	-1.488147786	1.149400333	-1.294716682	0.195417983	0.953843167	ENSG00000267530	LINC01836
9.8030997	-0.181602309	0.909589032	-0.199653143	0.841751863	0.999467288	ENSG00000267309	AC092295.2
9.372713553	2.121906602	1.216125235	1.744809285	0.081018072	0.897807954	ENSG00000116819	TFAP2E
9.241556508	-0.097862514	1.43346838	-0.06826974	0.945570912	0.999527751	ENSG00000162631	NTNG1
10.13342635	-0.24472637	1.326888897	-0.184436218	0.853671259	0.999467288	ENSG00000228626	AC245100.3
9.87934784	-0.100253128	0.896702093	-0.111802045	0.910980366	0.999527751	ENSG00000237298	TTN-AS1
9.945036258	-0.675078651	0.916636491	-0.736473681	0.461442479	0.99135833	ENSG00000163053	SLC16A14
9.900026057	0.717681123	0.968232027	0.741228448	0.458554937	0.99135833	ENSG00000144649	FAM198A
9.82883959	-2.262299973	1.009999646	-2.239901749	0.025097302	0.755167362	ENSG00000188001	TPRG1
9.636522226	-0.199265866	1.105359729	-0.180272413	0.856938712	0.999467288	ENSG00000223652	AC106786.1
9.762382993	-1.453536281	0.927538229	-1.567090428	0.117093556	0.923922314	ENSG00000213433	RPLP1P6

9.52656567	-0.642806178	1.000318086	-0.642601776	0.520482531	0.993513219	ENSG00000226824	AC006001.2
9.876275948	-1.783659527	0.975824432	-1.827848809	0.067572246	0.87420149	ENSG00000110079	MS4A4A
9.770459543	0.642035315	0.960723124	0.668283399	0.503952708	0.993513219	ENSG00000278616	BEND3P3
9.856297886	0.983535387	0.994216208	0.989257045	0.322537395	0.975337095	ENSG00000279233	AC122688.3
9.590691149	-0.323435323	1.204187471	-0.268592167	0.78824354	0.999467288	ENSG00000276248	AL442125.1
9.191521404	-0.435086768	1.197262683	-0.36340126	0.716305161	0.99900742	ENSG00000140557	ST8SIA2
9.522225162	0.150123057	0.97363834	0.1541877	0.87746174	0.999467288	ENSG00000182108	DEXI
9.651944902	-1.008937809	0.991708699	-1.017373156	0.308975947	0.973427483	ENSG00000203865	ATP1A1-AS1
10.07639297	-0.166242693	1.135122737	-0.146453496	0.883563399	0.999467288	ENSG00000225234	TRAPPC12-AS1
9.526791435	0.336821301	1.244879464	0.270565393	0.786725316	0.999467288	ENSG00000223658	C1GALT1C1L
9.751192854	0.016457804	0.892438592	0.018441385	0.985286737	0.999527751	ENSG00000185986	SDHAP3
9.491392074	0.715419544	0.966323727	0.740351835	0.459086536	0.99135833	ENSG00000175449	RFESD
10.07860935	-0.730444465	1.348472513	-0.541682873	0.58803699	0.99483168	ENSG00000240764	PCDHGC5
9.889755643	5.198514408	1.4492204	3.587110978	0.000334362	0.166523826	ENSG00000181016	LSMEM1
9.572723042	-0.38414547	0.905534749	-0.424219469	0.671405764	0.997568624	ENSG00000146856	AGBL3
9.38209195	-1.226860771	1.000563168	-1.226170231	0.220134621	0.956884001	ENSG00000216866	RPS2P55
9.264965758	2.278450025	1.28989185	1.766388419	0.077330707	0.897052166	ENSG00000278989	AP001148.1
9.889857349	-1.144329591	1.052914627	-1.08682087	0.277115982	0.962465655	ENSG00000271614	ATP2B1-AS1
9.648176281	1.016815146	1.472649305	0.690466591	0.489900812	0.993513219	ENSG00000205918	PDPK2P
9.593685416	0.147411882	0.834542573	0.176637941	0.85979279	0.999467288	ENSG00000261461	UBE2MP1
9.699803732	-0.716697334	0.900644611	-0.79576042	0.426171301	0.991301167	ENSG00000263400	TMEM220-AS1
9.693600318	-0.831573418	0.87918011	-0.945851036	0.34422457	0.977691382	ENSG00000186868	MAPT
9.679587527	0.359510115	0.927064904	0.387793901	0.69816856	0.998869317	ENSG00000225828	FAM229A
9.812312869	-1.178455116	1.217155324	-0.96820438	0.332942308	0.976026345	ENSG00000162595	DIRAS3
9.91409708	0.808144594	1.061247063	0.761504669	0.446355691	0.99135833	ENSG00000225855	RUSC1-AS1
9.849602928	-0.011839046	1.060237479	-0.01116641	0.991090679	0.999527751	ENSG00000214595	EML6
9.307770471	1.676520547	1.080137967	1.552135559	0.120629791	0.923922314	ENSG00000196787	HIST1H2AG
9.428234953	1.102690023	1.053498088	1.046693901	0.295240778	0.970706863	ENSG00000153291	SLC25A27
9.223113912	-0.377385892	1.194587803	-0.315913063	0.752068497	0.999467288	ENSG00000003147	ICA1
9.483145465	0.259591462	0.950436117	0.273128785	0.784754221	0.999467288	ENSG00000227014	AC007285.1
9.512684397	0.93372174	0.884108343	1.056116875	0.290914822	0.969246313	ENSG00000160219	GAB3
8.916081298	-0.071753539	1.325650279	-0.05412705	0.956833941	0.999527751	ENSG00000104213	PDGFRL
9.434667751	-2.2956279	1.022016166	-2.246175722	0.024692755	0.749756093	ENSG00000228395	AL356481.1
9.411052142	-2.135858096	1.069769263	-1.996559605	0.045873045	0.815583282	ENSG00000066336	SPI1
9.075408253	-0.382527211	1.304431239	-0.293252108	0.769329457	0.999467288	ENSG00000261377	PDCD6IPP2
9.227201938	0.226702686	0.945320245	0.239815752	0.810473095	0.999467288	ENSG00000137825	ITPKA
9.452241363	2.612524647	1.245406229	2.097728907	0.0359291	0.785058983	ENSG00000198901	PRC1
9.471100007	1.186890227	1.041567697	1.139522885	0.254485129	0.962465655	ENSG00000283213	AC130466.1
9.522786935	-0.13504294	1.035060094	-0.130468696	0.89619562	0.999467288	ENSG00000129946	SHC2
9.497719445	-0.370652679	0.915963882	-0.404658619	0.685728464	0.998869317	ENSG00000188033	ZNF490
9.254629967	0.995975929	0.868683346	1.14653508	0.251573821	0.962465655	ENSG00000269843	AC008537.2
9.149651872	0.587062845	1.180433619	0.497328131	0.618957678	0.99483168	ENSG00000235890	TSPEAR-AS1
9.591705732	-1.93858024	1.082399428	-1.791002647	0.07329287	0.888663898	ENSG00000248458	AL139147.1
9.284571419	-1.186646203	1.022810636	-1.16018172	0.245974828	0.962465655	ENSG00000277702	AC239859.6
9.506821107	-2.485362892	1.261066421	-1.970842178	0.048741931	0.83480477	ENSG00000145287	PLAC8
9.312288985	0.363341431	1.0004422	0.363180832	0.716469805	0.99900742	ENSG00000268471	MIR4453HG
9.653513011	0.181036881	1.01222744	0.178850003	0.858055481	0.999467288	ENSG00000120328	PCDHB12
9.272932459	1.063907412	1.319278286	0.806431382	0.419994158	0.990680811	ENSG00000169252	ADRB2
9.577661587	-0.956462742	0.965025235	-0.991127182	0.321623481	0.975337095	ENSG00000219992	AL391422.1
9.196152384	0.050687278	0.956182871	0.053010026	0.95772392	0.999527751	ENSG00000124635	HIST1H2BJ
9.44288022	-3.285689086	1.33152081	-2.467621281	0.013601414	0.672931175	ENSG00000204301	NOTCH4
9.442593032	-0.375228361	0.973520058	-0.385434648	0.699915416	0.998869317	ENSG00000241764	AC002467.1
9.125786841	1.588955616	1.191422645	1.333662427	0.182314514	0.949094286	ENSG00000198910	L1CAM
9.488277705	-1.621277866	0.985907578	-1.64445218	0.100082834	0.92169898	ENSG00000227473	TSSK5P
9.408179129	0.869686332	1.334304593	0.651789956	0.514536682	0.993513219	ENSG00000186642	PDE2A
9.062212963	-0.371036575	0.960879411	-0.386142705	0.699390984	0.998869317	ENSG00000205791	LOH12CR2
9.686274834	-0.460716298	1.092391923	-0.421750005	0.673207501	0.998473603	ENSG00000177875	CCDC184
9.193600697	0.684296174	1.232615891	0.555157676	0.578786815	0.99483168	ENSG00000268087	AC008764.2
9.592720337	-0.43410046	1.149303502	-0.377707419	0.705647956	0.99900742	ENSG00000128709	HOXD9
9.45941062	-1.472990411	1.295887575	-1.136665278	0.25567824	0.962465655	ENSG00000227946	AC007383.2
9.232400457	-1.74956494	1.011822408	-1.729122548	0.083787164	0.901307093	ENSG00000204967	PCDHA4
9.325225628	-1.018789103	0.935161383	-1.089425977	0.275966081	0.962465655	ENSG00000244041	LINC01011
9.491296805	-0.470332982	1.310608644	-0.358866077	0.719695278	0.99900742	ENSG00000124749	COL21A1
8.804362609	1.280206099	1.337130776	0.957427742	0.338351401	0.977691382	ENSG00000079841	RIMS1

9.093847484	3.325841585	1.149674317	2.892855425	0.00381757	0.451943936	ENSG00000091844	RGS17
9.346853792	1.405086806	0.996837971	1.409543825	0.158674424	0.939464191	ENSG00000234585	CCT6P3
9.322241982	-0.359975583	1.204631929	-0.298826201	0.765072658	0.999467288	ENSG00000285090	AC002074.1
9.193655543	-0.580945393	1.046276001	-0.555250615	0.578723253	0.99483168	ENSG00000168071	CCDC88B
9.280954448	2.269886674	1.120491394	2.025795723	0.042785722	0.806698895	ENSG00000256667	KLRA1P
9.311122892	0.345547229	0.922635944	0.374521751	0.708016177	0.99900742	ENSG00000139537	CCDC65
9.274197001	0.444964197	1.081507966	0.411429422	0.680757684	0.998869317	ENSG00000279148	AC126474.1
9.133641086	1.968197018	1.006934833	1.954641903	0.050625352	0.842188482	ENSG00000140398	NEIL1
9.311300986	-1.029957406	1.050368328	-0.980567844	0.326805898	0.976026345	ENSG00000108947	EFNB3
8.938511103	1.428428722	0.990280986	1.442447893	0.149176061	0.936396974	ENSG00000231551	AC245100.4
8.740836344	1.605101446	1.432349544	1.120607363	0.262455029	0.962465655	ENSG00000175874	CREG2
9.313424229	0.642051847	1.113829815	0.576436219	0.564320369	0.99483168	ENSG00000236255	AC009404.1
8.942423753	-0.33004976	1.278015478	-0.258251771	0.796212605	0.999467288	ENSG00000273472	AC096733.2
9.207102101	-0.009786358	1.153535885	-0.008483792	0.993230995	0.999527751	ENSG00000166432	ZMAT1
9.124730661	-2.200913027	1.202039716	-1.830981953	0.067103237	0.87420149	ENSG00000215187	FAM166B
9.239284234	0.30934985	0.953694192	0.324370068	0.745657878	0.999467288	ENSG00000156689	GLYATL2
9.235045236	0.889803387	0.868948495	1.024000148	0.305835205	0.973427483	ENSG00000075073	TACR2
9.428767307	0.125962822	1.0496472	0.120004914	0.904479255	0.999527751	ENSG00000188596	CFAP54
9.472907586	3.315253572	1.361845696	2.434382678	0.014917215	0.690542737	ENSG00000274471	AC242376.2
9.562419564	1.938783786	1.006163834	1.926906653	0.053991264	0.847598083	ENSG00000154898	CCDC144CP
9.571529161	-0.238135803	1.107519917	-0.215017174	0.829753929	0.999467288	ENSG00000130518	IQCN
9.206867922	0.455781463	0.885446698	0.514747488	0.606729484	0.99483168	ENSG00000105717	PBX4
9.188826308	1.702072627	0.896759646	1.898025446	0.05769273	0.852306368	ENSG00000011332	DPF1
8.994594089	0.015586196	1.00582818	0.015495883	0.987636569	0.999527751	ENSG00000182902	SLC25A18
9.42744034	0.069626823	1.02030447	0.068241221	0.945593614	0.999527751	ENSG00000278948	AL031587.5
9.105971137	-1.058024851	1.087867729	-0.972567549	0.330768277	0.976026345	ENSG00000225826	LINC00626
9.067014586	-0.876142273	0.957879678	-0.9146684	0.360365738	0.979465649	ENSG00000196220	SRGAP3
9.068042276	-0.526334096	1.147931639	-0.458506481	0.646588608	0.997368416	ENSG00000174640	SLCO2A1
8.850445637	0.856183086	0.989566034	0.865210664	0.386923173	0.986799595	ENSG00000168491	CCDC110
9.255940851	-1.67157448	1.074922698	-1.555064828	0.119930631	0.923922314	ENSG00000182366	FAM87A
8.809088584	-0.183218435	1.079309867	-0.169755175	0.865202681	0.999467288	ENSG00000181634	TNFSF15
9.186996272	0.363310628	0.858415896	0.423233807	0.672124683	0.997584709	ENSG00000228544	CCDC183-AS1
8.937726557	-0.486359122	0.994187622	-0.489202553	0.624698303	0.99483168	ENSG00000271335	AL117336.3
9.488827599	-1.435486412	1.189087419	-1.207216887	0.227348629	0.958865115	ENSG00000230018	PPIAP39
8.812412148	-0.588656463	1.092713535	-0.53871069	0.590086496	0.99483168	ENSG00000151490	PTPRO
9.231898346	0.381381304	0.882518317	0.432151148	0.665631562	0.997568624	ENSG00000277156	AL589743.5
8.807174475	1.547153326	1.105138377	1.399963442	0.161524266	0.942964826	ENSG00000261114	AC012181.1
9.532186899	-0.616782518	0.966190555	-0.638365294	0.523235917	0.994616524	ENSG00000274565	AC080038.1
9.091716875	-1.25103149	1.151337549	-1.086589672	0.277218192	0.962465655	ENSG00000167850	CD300C
9.162938547	1.296950209	0.899410942	1.441999588	0.14930249	0.936396974	ENSG00000280407	AC132872.4
9.105098976	-1.432888374	1.328845428	-1.078295747	0.280901794	0.963267117	ENSG00000265778	AC018413.1
8.824953247	0.057729755	1.432251037	0.040307008	0.967848366	0.999527751	ENSG00000226012	AP001434.1
9.311895421	1.155371695	0.944331222	1.22348141	0.221147921	0.956884001	ENSG00000210151	MT-TS1
9.116962851	0.966796045	1.246621967	0.775532656	0.438024977	0.991301167	ENSG00000270728	AL035413.2
9.021024179	-0.749293135	1.214164215	-0.617126683	0.53715117	0.99483168	ENSG00000224081	SLC44A3-AS1
8.948622873	-0.936767408	1.027400888	-0.911783724	0.36188258	0.979465649	ENSG00000177144	NUDT4B
9.168884969	0.099782677	0.956252556	0.104347618	0.91689349	0.999527751	ENSG00000234028	AC062029.1
8.821981675	-0.88875085	1.114559609	-0.797400913	0.425218229	0.991301167	ENSG00000228857	AC104653.1
9.37202671	-0.181342395	1.307770044	-0.138665353	0.889714598	0.999467288	ENSG00000170837	GPR27
9.178224004	0.065697036	1.099307814	0.059762184	0.952345045	0.999527751	ENSG00000283050	GTF2IP12
8.86563405	-0.214310891	1.151031489	-0.186190294	0.852295535	0.999467288	ENSG00000224786	CETN4P
9.143396991	0.672490104	1.022466685	0.657713462	0.51072227	0.993513219	ENSG00000268942	CKS1BP3
9.153186547	-0.997158874	0.907079358	-1.099307205	0.271634091	0.962465655	ENSG00000272341	AL137003.2
8.786734497	-1.341679569	1.138490682	-1.178472156	0.238608426	0.962465655	ENSG00000234336	JAZF1-AS1
9.095472473	0.939473945	0.935940819	1.003774946	0.315487103	0.974768742	ENSG00000274272	AC069281.2
9.088697073	-0.128026691	1.010904936	-0.126645628	0.899220882	0.999527751	ENSG00000260947	AL356489.2
8.813504414	0.326615156	1.15654997	0.282404708	0.777633205	0.999467288	ENSG00000174669	SLC29A2
9.332308996	0.436469479	1.092122519	0.399652485	0.689412493	0.998869317	ENSG00000261098	AP000766.1
9.322337251	-0.954512796	1.076666098	-0.886544861	0.375323992	0.982609538	ENSG00000188916	INSYN2
9.288624071	0.747394173	0.994728131	0.75135522	0.452438905	0.99135833	ENSG00000148824	MTG1
8.648313402	0.085348738	1.070009695	0.079764453	0.936424596	0.999527751	ENSG00000111254	AKAP3
9.502733992	-1.864524805	1.488064417	-1.252986621	0.210210576	0.954310328	ENSG00000176387	HSD11B2
8.760660908	0.04742716	1.072739513	0.044211255	0.964736011	0.999527751	ENSG00000270049	AC009061.2
9.07874709	-0.244428237	1.362066524	-0.179453964	0.857581262	0.999467288	ENSG00000108785	HSD17B1P1

8.941437996	2.11128938	1.31285591	1.608165347	0.10779897	0.923922314	ENSG00000189144	ZNF573
8.834825651	1.040853292	0.949333054	1.096404774	0.272901676	0.962465655	ENSG00000179168	GGN
8.691506043	-0.067938281	1.082678479	-0.06275019	0.94996543	0.999527751	ENSG00000278384	AL354822.1
9.048939483	0.547002635	0.991584396	0.551645062	0.581191557	0.99483168	ENSG00000268575	AL031282.2
9.648034382	-0.367954415	1.55153856	-0.237154541	0.812536902	0.999467288	ENSG00000130762	ARHGEF16
8.549548867	3.823691662	1.837784443	2.080598558	0.037470666	0.790190505	ENSG00000177181	RIMKLA
9.035363116	-0.522274047	0.98642622	-0.529460831	0.596485809	0.99483168	ENSG00000198658	ABHD17AP1
9.043226487	-0.298276926	0.968439369	-0.307997522	0.758084218	0.999467288	ENSG00000231822	AC019097.1
8.969774732	0.778739284	0.887693788	0.877261162	0.380344802	0.983746147	ENSG00000175772	LINC01106
8.950321615	-0.5032187	0.893788902	-0.563017396	0.573423038	0.99483168	ENSG00000270362	HMGN3-AS1
9.071205732	0.134481204	0.912162599	0.147431175	0.8827917	0.999467288	ENSG00000089041	P2RX7
8.913455619	-0.501472135	0.973790643	-0.514969145	0.606574581	0.99483168	ENSG00000279730	SETD8P1
8.90363515	-1.567427329	1.18063195	-1.327617238	0.184304587	0.949094286	ENSG00000103569	AQP9
9.224359218	0.156050422	1.206831732	0.129305866	0.89711563	0.999467288	ENSG00000172382	PRSS27
9.143616089	-0.102590353	1.142504592	-0.089794259	0.928450711	0.999527751	ENSG00000234719	NPIPB2
9.143953562	1.30026689	0.980821103	1.325692204	0.184941671	0.949094286	ENSG00000167723	TRPV3
9.305594279	-0.371014863	1.18581655	-0.312877116	0.754374024	0.999467288	ENSG00000131480	AOC2
9.17603426	-0.445062459	1.042768205	-0.42680862	0.669518727	0.997568624	ENSG00000176681	LRRC37A
8.81412514	0.473721244	1.134493615	0.417561842	0.6762675	0.998869317	ENSG00000171873	ADRA1D
9.049089196	1.853370816	0.967212385	1.91619839	0.055339848	0.847598083	ENSG00000250067	YJEFN3
9.235439371	-0.660915953	1.022991933	-0.646061745	0.518239369	0.993513219	ENSG00000267475	AC008736.1
9.073356484	-0.995305364	0.929097017	-1.071260962	0.284052108	0.963310634	ENSG00000267422	AC016582.2
8.739223908	-0.077095129	1.007979217	-0.07648484	0.939033375	0.999527751	ENSG00000215014	AL645728.1
8.889574869	-0.952531907	1.002621307	-0.950041557	0.342091137	0.977691382	ENSG00000171357	LURAP1
9.033842591	-0.010032062	0.88626736	-0.011319453	0.990968576	0.999527751	ENSG00000228929	RPS13P2
9.644779412	-1.526548331	1.534980008	-0.994506979	0.319976109	0.974964731	ENSG00000178623	GPR35
8.956359978	-0.432315768	0.946085194	-0.45695226	0.64770536	0.997368416	ENSG00000251442	LINC01094
8.916359458	1.248776287	1.05800465	1.180312663	0.237875883	0.962465655	ENSG00000215246	AC116351.1
8.70144766	-0.016224952	0.968389429	-0.016754574	0.98663241	0.999527751	ENSG00000172058	SERF1A
9.058405677	-0.985521115	1.020893928	-0.965351138	0.334368966	0.976026345	ENSG00000213316	LTC4S
8.653021466	-0.674772202	0.974729674	-0.692265989	0.488770305	0.993513219	ENSG00000145949	MYLK4
8.937274402	-0.069908204	1.085968882	-0.06437404	0.9486724	0.999527751	ENSG00000188732	FAM221A
8.978476081	-0.641235539	1.106677759	-0.579423896	0.562303184	0.99483168	ENSG00000228775	WEE2-AS1
8.943966391	0.363720371	1.212947211	0.299864963	0.76428016	0.999467288	ENSG00000260317	AC009812.4
9.072525945	-0.200763843	1.014885991	-0.197819109	0.843186594	0.999467288	ENSG00000283959	AP002851.1
8.900501278	0.085088426	0.912812747	0.093215642	0.925732248	0.999527751	ENSG00000168517	HEXIM2
8.818841097	0.261202842	0.972468959	0.268597614	0.788239349	0.999467288	ENSG00000279332	AC090772.4
8.92113088	-0.286592236	1.062251362	-0.269797005	0.787316427	0.999467288	ENSG00000228274	AL021707.2
8.782587055	1.275838379	0.893449374	1.427991799	0.153294219	0.939432741	ENSG00000080947	CROCCP3
8.704356381	-0.31406744	0.97090959	-0.323477533	0.746333619	0.999467288	ENSG00000228502	EEF1A1P11
8.633126244	-0.87948258	1.039483493	-0.846076524	0.397510056	0.988378925	ENSG00000027869	SH2D2A
9.260223426	-0.137646832	1.113461212	-0.12362068	0.901615618	0.999527751	ENSG00000114948	ADAM23
8.785404851	0.828023337	0.904202363	0.915750025	0.359798021	0.979465649	ENSG00000271870	AC024060.1
8.652509158	-1.358800159	1.05379531	-1.289434624	0.197247031	0.954310328	ENSG00000230943	LINC02541
8.847861318	0.018718736	1.008996131	0.018551841	0.985198621	0.999527751	ENSG00000230825	AC005532.1
8.998291738	-1.395038484	1.235761196	-1.128890022	0.258944231	0.962465655	ENSG00000184616	SPDYE12P
8.869871235	0.880846374	1.291187317	0.68219875	0.495113285	0.993513219	ENSG00000179542	SLITRK4
8.890384132	-0.745924655	1.319784203	-0.565186833	0.571946691	0.99483168	ENSG00000130595	TNNT3
8.529310484	-2.00717802	1.126526705	-1.781740293	0.074791593	0.893390506	ENSG00000260233	SSSCA1-AS1
9.0206793	-1.130817431	1.297621107	-0.871454253	0.383506174	0.985655313	ENSG00000198915	RASGEF1A
8.707457403	-0.700823571	1.037373149	-0.675575198	0.499310393	0.993513219	ENSG00000229927	RHEBP1
8.749813892	-0.327900814	1.107341568	-0.29611533	0.767142007	0.999467288	ENSG00000240370	RPL13P5
9.041653072	0.706679483	1.297286872	0.544736479	0.585934772	0.99483168	ENSG00000151322	NPAS3
9.015743401	0.130429307	1.113642636	0.117119535	0.90676533	0.999527751	ENSG00000100505	TRIM9
8.747556937	0.248823236	1.182652658	0.21039418	0.833360035	0.999467288	ENSG00000139985	ADAM21
8.683220571	-0.563534656	1.089604196	-0.517192076	0.605022083	0.99483168	ENSG00000140022	STON2
9.039169824	-1.506661107	1.081597986	-1.39299548	0.163621157	0.943223078	ENSG00000260643	AC092718.3
9.092714509	0.592872115	1.305791964	0.454032596	0.649805366	0.997381078	ENSG00000277476	AC005332.5
8.706121154	-1.121694208	1.16243047	-0.964955959	0.334566871	0.976026345	ENSG00000177191	B3GNT8
8.793609482	0.072317252	1.038657797	0.069625677	0.944491599	0.999527751	ENSG00000178386	ZNF223
8.681186772	-2.56687698	1.352552246	-1.897802461	0.057722109	0.852306368	ENSG00000105357	MYH14
8.775650565	-1.270581362	0.933914499	-1.360490028	0.173674906	0.944422693	ENSG00000213790	OLA1P1
8.589570187	0.32562487	1.041840503	0.312547716	0.754624305	0.999467288	ENSG00000260708	AL118516.1
8.530618058	-0.688170386	0.940205599	-0.731936065	0.464207592	0.99135833	ENSG00000187010	RHD

8.774459197	3.84513539	1.331909848	2.886933674	0.003890162	0.454844605	ENSG00000213088	ACKR1
8.718589233	0.545577095	1.222231469	0.446377883	0.655324306	0.997568624	ENSG00000272668	AL590560.1
8.743692154	0.732864949	0.915052307	0.800899515	0.423189821	0.991301167	ENSG00000242282	AC108488.1
8.521699638	-2.55385016	1.062335419	-2.403996059	0.016216948	0.698816035	ENSG00000115956	PLEK
8.95474004	-1.621595973	1.303349999	-1.244175374	0.213435025	0.954310328	ENSG00000163075	CFAP221
8.623920722	-1.563552248	1.345106385	-1.162400435	0.245072846	0.962465655	ENSG00000224858	RPL29P11
8.47322752	1.569810355	1.054836555	1.488202458	0.136697509	0.934144427	ENSG00000273356	LINC02019
8.91580722	0.605472202	1.336438143	0.453049178	0.65051333	0.997381078	ENSG00000215018	COL28A1
8.397339623	-1.697054835	1.483874207	-1.143664892	0.252762638	0.962465655	ENSG00000183690	EFHC2
8.643166119	-0.476103875	0.994052988	-0.478952209	0.631972628	0.99483168	ENSG00000089820	ARHGAP4
8.564991613	3.126574361	1.162500466	2.689525255	0.007155373	0.555618581	ENSG00000147437	GNRH1
8.858913446	0.397882721	1.0491921	0.379227714	0.704518773	0.99900742	ENSG00000274026	FAM27E3
8.861678112	1.257890831	0.98680466	1.274711076	0.202411525	0.954310328	ENSG00000249839	AC011330.1
8.666484609	-0.80397916	1.083867957	-0.741768547	0.45822758	0.99135833	ENSG00000108958	AC130689.1
8.382997703	0.21618878	2.023094998	0.106860419	0.914899714	0.999527751	ENSG00000125895	TMEM74B
8.445543376	-0.274818392	1.138856085	-0.241310904	0.80931416	0.999467288	ENSG00000196074	SYCP2
8.819597063	-0.210329527	1.033142715	-0.203582259	0.838679958	0.999467288	ENSG00000089847	ANKRD24
8.939978719	-1.35662949	1.182321015	-1.147429059	0.251204337	0.962465655	ENSG00000142347	MYO1F
8.663211293	1.137793955	0.998804882	1.13915538	0.254638352	0.962465655	ENSG00000233493	TMEM238
8.394343634	-1.719128184	1.295949971	-1.326539005	0.184661224	0.949094286	ENSG00000160307	S100B
8.582891642	0.200799767	1.232927215	0.162864251	0.870625319	0.999467288	ENSG00000185519	FAM131C
8.511867461	0.510678429	1.034968527	0.493424114	0.621712947	0.99483168	ENSG00000232022	FAAHP1
8.794366086	0.117174991	1.139659586	0.102815782	0.91810918	0.999527751	ENSG00000213225	NOC2LP1
8.942846312	1.672432357	1.099343806	1.521300569	0.128184428	0.928627271	ENSG00000236841	AC007750.1
8.708727783	-1.209934479	1.109014729	-1.090999468	0.275273117	0.962465655	ENSG00000018280	SLC11A1
8.523363508	0.685101748	1.463157559	0.468235115	0.639616463	0.996553586	ENSG00000205403	CFI
8.727283589	1.062450281	1.058758035	1.003487337	0.315625784	0.974768742	ENSG00000213073	AL353625.1
8.955550137	1.552351256	1.473285609	1.053666204	0.292035767	0.97066771	ENSG00000248498	ASNSP1
8.430825583	0.524790492	1.458078354	0.359919267	0.718907509	0.99900742	ENSG00000165985	C1QL3
8.442465542	1.01544976	1.043741665	0.972893767	0.330606103	0.976026345	ENSG00000275202	AL161421.1
8.516026629	1.438470965	0.937682957	1.534069649	0.125012519	0.927794614	ENSG00000217930	PAM16
8.314818648	-0.462560181	1.464145878	-0.315924928	0.75205949	0.999467288	ENSG00000205362	MT1A
8.732052555	0.599356393	0.97572973	0.614264764	0.539040383	0.99483168	ENSG00000204316	MRPL38
8.72755936	-0.286518994	0.960218701	-0.298389309	0.765406048	0.999467288	ENSG00000173928	SWSAP1
8.910425174	1.378919724	1.174104166	1.174444112	0.240217185	0.962465655	ENSG00000100429	HDAC10
8.514888679	1.190063005	1.124986837	1.057846159	0.290125585	0.967933358	ENSG00000162572	SCNN1D
8.741161507	0.355935615	0.962686585	0.369731562	0.711582512	0.99900742	ENSG00000182873	PRKCZ-AS1
8.384263684	2.161951507	1.048622393	2.061706408	0.039235696	0.793985738	ENSG00000200087	SNORA73B
8.356367272	-1.509874949	1.339665964	-1.127053302	0.259719945	0.962465655	ENSG00000160097	FNDC5
8.187203855	0.518506772	1.26603037	0.409553186	0.682133744	0.998869317	ENSG00000261737	AL049597.2
8.42751152	1.337730539	1.157589291	1.155617584	0.247837613	0.962465655	ENSG00000270210	AC104695.2
8.655397643	-0.277979254	1.203079274	-0.231056473	0.81727093	0.999467288	ENSG00000163219	ARHGAP25
8.774991188	1.853449625	1.559254659	1.188676663	0.234566927	0.961085539	ENSG00000237686	AL109615.3
8.639837458	-1.527696632	1.358235305	-1.124765809	0.260688286	0.962465655	ENSG00000077264	PAK3
8.384164674	1.15926034	1.232492858	0.940581791	0.346919217	0.977691382	ENSG00000251364	AC107884.1
8.529866788	-0.857425945	1.045163669	-0.820374809	0.412002471	0.990226751	ENSG00000238041	AP004245.1
8.474094276	0.086343162	0.993249528	0.08692998	0.930727169	0.999527751	ENSG00000255062	AP001318.2
8.526715196	-0.608291995	0.997038358	-0.61009889	0.541796302	0.99483168	ENSG00000134470	IL15RA
8.785285909	0.120997994	1.03262879	0.117174725	0.906721596	0.999527751	ENSG00000239883	PARGP1
8.396072094	0.47361357	1.075771153	0.440254945	0.659752469	0.997568624	ENSG00000273888	FRMD6-AS1
8.68415009	1.646125454	1.048679363	1.569712833	0.116481934	0.923922314	ENSG00000246451	AL049840.1
8.736593551	1.516861166	1.100697474	1.378090895	0.168175226	0.943223078	ENSG00000279520	AC093525.8
8.602747748	0.951096487	0.957501395	0.993310811	0.32055851	0.975316319	ENSG00000227827	AC138969.1
8.518369658	1.483745979	1.314439237	1.128805301	0.258979976	0.962465655	ENSG00000196296	ATP2A1
8.423684231	0.502674623	1.027067906	0.489426863	0.624539522	0.99483168	ENSG00000159217	IGF2BP1
8.36738375	0.745976574	1.113688411	0.66982521	0.502969221	0.993513219	ENSG00000105146	AURKC
8.577238363	-0.463822979	0.953920222	-0.48622827	0.62680533	0.99483168	ENSG00000276529	AP001505.1
8.530058235	0.045994149	0.996752806	0.046143988	0.963195486	0.999527751	ENSG00000234810	AL603840.1
8.334533907	-0.07343171	1.04078691	-0.070554029	0.943752699	0.999527751	ENSG00000144063	MALL
8.490473325	0.32763898	0.957289476	0.342256954	0.732157527	0.999467288	ENSG00000204311	PJVK
8.346808379	1.335957708	1.088236135	1.227635865	0.219583692	0.956884001	ENSG00000237126	AC073254.1
8.439544351	1.636012405	0.980303914	1.668882866	0.095140596	0.921147998	ENSG00000227252	AC105760.2
8.381424286	-0.501651592	0.904959218	-0.554336131	0.579348827	0.99483168	ENSG00000280739	EIF1B-AS1
8.673316918	1.171635216	1.359016707	0.862119803	0.388621595	0.986799595	ENSG00000230177	AL080317.1

8.064483802	1.631529964	1.044747556	1.561649946	0.118370477	0.923922314	ENSG00000274791	F8A2
8.561692578	-0.490601664	0.899120158	-0.545646385	0.585309033	0.99483168	ENSG00000251034	AC037459.2
8.42179385	-0.260354432	0.943091493	-0.276064871	0.782498241	0.999467288	ENSG00000137101	CD72
8.198353729	0.439896096	1.064798626	0.413126093	0.679514233	0.998869317	ENSG00000243444	PALM2
8.375963913	0.023725428	0.932085599	0.02545413	0.979692735	0.999527751	ENSG00000167103	PIP5KL1
8.236395558	-1.224484266	1.456426227	-0.840745823	0.400490344	0.988492517	ENSG00000274897	PANO1
8.386564436	-0.42062083	0.950221767	-0.442655435	0.658014981	0.997568624	ENSG00000165874	SHLD2P1
8.316593471	0.605778958	1.05215512	0.575750616	0.564783758	0.99483168	ENSG00000134532	SOX5
8.220814956	1.493491327	1.145409622	1.303892772	0.192270145	0.949261383	ENSG00000214353	VAC14-AS1
8.737415374	0.079805198	1.06382146	0.075017474	0.940200809	0.999527751	ENSG00000197134	ZNF257
8.489303147	0.07357874	1.095834253	0.067144041	0.946467033	0.999527751	ENSG00000182986	ZNF320
8.233237344	3.336761665	1.249218523	2.671079242	0.00756078	0.563130099	ENSG00000215784	FAM72D
8.262970833	-0.13160435	1.13225547	-0.116232029	0.907468654	0.999527751	ENSG00000246203	AL353807.3
8.010823291	-1.298320948	1.196172344	-1.085396226	0.277746203	0.962465655	ENSG00000260686	AC008669.1
8.215198511	1.208267879	1.251834354	0.965197891	0.334445703	0.976026345	ENSG00000053328	METTL24
8.231837983	-1.215741276	1.090807884	-1.114532901	0.265050638	0.962465655	ENSG00000002933	TMEM176A
8.401933327	0.637739207	1.042004811	0.612030963	0.540517272	0.99483168	ENSG00000280195	AC245140.2
8.276838644	0.242164359	1.016413149	0.238253862	0.811684203	0.999467288	ENSG00000225259	ST13P6
8.360208542	0.481990349	1.066973154	0.451736154	0.65145907	0.997449432	ENSG00000273226	AL391834.2
8.279587151	0.007934099	1.100618825	0.007208762	0.99424829	0.999527751	ENSG00000172350	ABCG4
8.470310105	-0.445199153	1.171661795	-0.379972407	0.703965897	0.99900742	ENSG00000107719	PALD1
8.040490467	1.706506321	1.039841511	1.641121558	0.100772185	0.92169898	ENSG00000139304	PTPRQ
8.122766692	0.286036869	1.025844414	0.278830654	0.780374788	0.999467288	ENSG00000246877	DNM1P35
8.243609695	-0.967701214	1.123575554	-0.861269374	0.389089699	0.986937571	ENSG00000255198	SNHG9
8.395201636	0.178059165	1.245879308	0.142918471	0.886354571	0.999467288	ENSG00000258947	TUBB3
7.866156174	1.464781488	1.225891606	1.194870314	0.232137703	0.959575179	ENSG00000108830	RND2
8.675663988	0.578573774	1.238884969	0.467011699	0.640491508	0.996553586	ENSG00000127530	OR7C1
8.072611724	0.404614409	0.976389137	0.41439872	0.678582126	0.998869317	ENSG00000279108	AC008537.3
8.319684832	-0.437913861	1.212731305	-0.361097186	0.718026797	0.99900742	ENSG00000268041	AC010616.1
8.272862609	-0.864469882	1.01259603	-0.853716443	0.393262121	0.988378925	ENSG00000174586	ZNF497
8.433800728	-1.140811425	1.153018231	-0.989413171	0.322461033	0.975337095	ENSG00000250318	AC003072.1
8.291229197	-0.300574018	1.087545446	-0.276378352	0.782257482	0.999467288	ENSG00000138964	PARVG
8.274230258	0.055904807	1.229913503	0.045454259	0.963745233	0.999527751	ENSG00000182257	PRR34
8.559330148	-0.749867541	1.21957222	-0.61486112	0.538646441	0.99483168	ENSG00000163501	IHH
7.980116683	0.76226798	1.429612364	0.533199068	0.593895787	0.99483168	ENSG00000160808	MYL3
8.096481512	-1.204777577	1.237369562	-0.973660266	0.330225252	0.976026345	ENSG00000206532	AC117402.1
8.156542028	0.781404324	1.080797038	0.722988958	0.469686668	0.99135833	ENSG00000118407	FILIP1
8.048886284	-0.51452903	1.003032877	-0.512973245	0.607970036	0.99483168	ENSG00000168255	POLR2J3
7.947336831	0.590253399	1.43476576	0.411393563	0.680783973	0.998869317	ENSG00000086717	PPEF1
8.315260354	1.842661537	0.951935595	1.935699796	0.052904479	0.847598083	ENSG00000253746	AC091182.2
8.145326719	2.398047729	1.269780311	1.888553247	0.058951717	0.853473128	ENSG00000172927	MYEOV
8.106341689	-0.709084901	1.077178941	-0.658279581	0.510358497	0.993513219	ENSG00000109927	TECTA
8.129237526	-0.603684879	1.212294925	-0.49796866	0.618506132	0.99483168	ENSG00000254944	ATP5PBP5
8.257452685	2.381519806	1.053539088	2.260494967	0.023790548	0.744948012	ENSG00000176244	ACBD7
8.203631998	0.685005482	0.935443024	0.732279214	0.463998164	0.99135833	ENSG00000151164	RAD9B
8.302362761	-0.750752983	1.234966189	-0.607913795	0.543244649	0.99483168	ENSG00000247092	SNHG10
8.180732333	-0.488277427	1.120758275	-0.435667028	0.663078332	0.997568624	ENSG00000178226	PRSS36
7.814040647	-0.686240505	1.225123237	-0.560139979	0.575383963	0.99483168	ENSG00000108961	RANGRF
8.494134178	3.264546398	1.325091469	2.463638529	0.013753474	0.672931175	ENSG00000274512	TBC1D3L
8.027138463	0.590158536	1.328988652	0.444065896	0.656994943	0.997568624	ENSG00000267040	AC027097.1
8.253435944	-0.914612123	1.098331427	-0.832728719	0.404997744	0.989962822	ENSG00000150636	CCDC102B
8.322061309	0.728098749	1.404636152	0.518353986	0.604211313	0.99483168	ENSG00000210174	MT-TR
8.289793131	2.643973364	1.390433109	1.901546609	0.057230454	0.852306368	ENSG00000228327	AL669831.1
8.159161642	-0.713994577	0.975789632	-0.731709535	0.464345875	0.99135833	ENSG00000143061	IGSF3
8.181761851	-0.682779464	1.008639932	-0.676930828	0.498449843	0.993513219	ENSG00000117477	CCDC181
7.910484072	0.858273815	1.036498688	0.828051039	0.407641596	0.990226751	ENSG00000168334	XIRP1
8.075674573	-0.883222896	1.138841547	-0.775545025	0.438017671	0.991301167	ENSG00000204261	PSMB8-AS1
8.292867279	1.187396647	1.030331584	1.152441277	0.249139787	0.962465655	ENSG00000104728	ARHGEF10
8.301881918	0.351051039	1.064117621	0.329898718	0.741476492	0.999467288	ENSG00000237840	FAM21FP
8.252545729	0.469017042	4.717092819	0.099429259	0.920797453	0.999527751	ENSG00000258791	LINC00520
8.158945659	0.127071714	0.996076749	0.127572212	0.898487523	0.999490224	ENSG00000261008	LINC01572
8.279339794	0.184907791	0.969384024	0.190747719	0.848723251	0.999467288	ENSG00000274561	AC005332.3
8.143708818	0.232589394	0.9881805	0.235371366	0.813920508	0.999467288	ENSG00000236830	CBR3-AS1
7.95158056	1.376046694	1.46469232	0.93947833	0.347485213	0.977691382	ENSG00000232284	GNG12-AS1

8.24249253	0.260435895	1.315700228	0.197944706	0.843088326	0.999467288	ENSG00000117594	HSD11B1	
7.752225025	-1.349724182	1.243283186	-1.085612833	0.27765032	0.962465655	ENSG00000168918	INPP5D	
7.966139072	0.343588909	1.225238273	0.280426197	0.779150541	0.999467288	ENSG00000240747	KRBOX1	
7.968387559	-0.694543332	1.047728033	-0.662904217	0.507391901	0.993513219	ENSG00000278934	AC117489.1	
7.995617561	0.239502788	1.197110495	0.200067403	0.841427867	0.999467288	ENSG00000172738	TMEM217	
8.152789417	-0.38899565	0.976112813	-0.398515053	0.690250564	0.998869317	ENSG00000254810	AP001189.3	
8.112703259	1.381177113	1.006494816	1.372264507	0.169981111	0.944422693	ENSG00000139187	KLRG1	
7.813581607	0.763980518	1.425585389	0.535906529	0.592023152	0.99483168	ENSG00000187773	FAM69C	
7.970027416	0.727337819	1.104240029	0.658677281	0.510103025	0.993513219	ENSG00000115257	PCSK4	
8.18111942	1.179621832	1.00856556	1.169603522	0.242160559	0.962465655	ENSG00000267481	AC011477.2	
8.100889252	-0.014035238	1.180454145	-0.011889693	0.990513621	0.999527751	ENSG00000100031	GGT1	
8.060601313	-1.549665217	1.114339289	-1.390658332	0.164329059	0.943223078	ENSG00000203667	COX20	
7.727860227	3.362516595	1.49469962	2.249626981	0.024472634	0.749756093	ENSG00000196460	RFX8	
8.09768566	-0.497358986	1.018009366	-0.488560324	0.625153008	0.99483168	ENSG00000188993	LRRC66	
7.933110538	0.061378904	1.016353763	0.060391279	0.951844004	0.999527751	ENSG00000233330	AL078581.1	
7.975967027	3.15143649	1.326395179	2.37594085	0.017504269	0.726053908	ENSG00000121716	PILRB	
8.003998083	0.482911307	1.073289496	0.449935743	0.652756774	0.997471445	ENSG00000238832	RF01233	
8.145313517	-0.148186128	1.35101301	-0.109685197	0.912659041	0.999527751	ENSG00000042980	ADAM28	
7.792420527	-0.582152057	1.149027505	-0.506647626	0.612402085	0.99483168	ENSG00000255153	TOLLIP-AS1	
8.151995165	1.48166767	1.390565316	1.065514617	0.286643114	0.965284229	ENSG00000166343	MSS51	
7.764976039	-0.735057377	1.04443847	-0.703782366	0.481568317	0.991525194	ENSG00000222047	C10orf55	
7.86144514	0.839493895	1.03690845	0.809612358	0.418163003	0.990226751	ENSG00000205683	DPF3	
8.01526361	-0.214114746	1.191025584	-0.179773423	0.857330449	0.999467288	ENSG00000173838		10-Mar
7.870932237	0.290354957	1.266187046	0.229314427	0.818624541	0.999467288	ENSG00000182324	KCNJ14	
8.043122184	-0.628978688	0.939113283	-0.669758057	0.503012035	0.993513219	ENSG00000261188	Z95115.1	
7.717323939	0.050837758	1.068526456	0.047577445	0.962053008	0.999527751	ENSG00000203739	AL645568.1	
8.223853549	-1.001418396	1.531704469	-0.653793481	0.513244867	0.993513219	ENSG00000236090	LDHAP3	
8.014962462	0.494435737	1.182819763	0.418014437	0.675936562	0.998869317	ENSG00000284052	AC006460.2	
7.822165559	1.871587438	1.460640493	1.281347085	0.200071781	0.954310328	ENSG00000229056	AC020571.1	
7.58427794	0.937285511	1.019042477	0.919770797	0.357692547	0.979427314	ENSG00000163694	RBM47	
7.767527678	0.392478918	1.138668412	0.344682362	0.730333179	0.999467288	ENSG00000250486	FAM218A	
7.610298771	2.207186314	1.171697588	1.883750839	0.059598686	0.858410096	ENSG00000070193	FGF10	
8.006772624	1.042711442	1.046195458	0.996669823	0.318924802	0.974768742	ENSG00000246763	RGMB-AS1	
7.683724214	-0.490143166	1.045315422	-0.468894992	0.639144696	0.996553586	ENSG00000233859	ADH5P4	
7.916172323	1.121862382	1.01878795	1.101173588	0.270821115	0.962465655	ENSG00000235086	FNDC1-IT1	
7.91263465	-1.531189758	1.164035379	-1.315415138	0.188370421	0.949094286	ENSG00000224116	INHBA-AS1	
7.692255184	-1.21472741	1.091144613	-1.113259778	0.265596876	0.962465655	ENSG00000231638	LUARIS	
7.825852103	-0.491753335	1.107146424	-0.444162871	0.656924834	0.997568624	ENSG00000270157	AC004918.3	
8.035922796	0.709627818	1.005554311	0.705708095	0.480369686	0.99135833	ENSG00000173698	ADGRG2	
7.928469175	1.929298331	1.680609971	1.147975059	0.25097886	0.962465655	ENSG00000224945	AL353150.1	
8.03670517	1.054641726	1.043533764	1.010644564	0.312186578	0.973427483	ENSG00000242375	AL590705.3	
7.96833983	0.553791015	1.522858045	0.363652421	0.716117576	0.99900742	ENSG00000182771	GRID1	
7.993794745	0.72314566	1.145375205	0.631361372	0.527804267	0.99483168	ENSG00000148798	INA	
7.855769126	0.234692153	1.05886361	0.22164531	0.824590007	0.999467288	ENSG00000221949	LINC01465	
8.230665655	0.098541271	1.184364162	0.083201834	0.933691054	0.999527751	ENSG00000180730	SHISA2	
7.752463945	-1.827188843	1.313655503	-1.390919338	0.164249888	0.943223078	ENSG00000260992	DOCK9-DT	
8.059044651	2.14781372	1.32382807	1.622426482	0.104712063	0.92169898	ENSG00000258768	AL356019.2	
7.924582308	0.686967404	1.440444483	0.476913489	0.633423726	0.995376704	ENSG00000269927	AC004817.3	
8.800953257	-1.458950222	2.054955336	-0.709966877	0.477724677	0.99135833	ENSG00000130487	KLHDC7B	
7.949446868	-0.296355143	1.052665707	-0.281528258	0.778305258	0.999467288	ENSG00000243646	IL10RB	
7.927059224	0.536260556	1.262021525	0.424921877	0.670893626	0.997568624	ENSG00000055732	MCOLN3	
7.855384565	-0.805029957	1.196872213	-0.672611452	0.501194514	0.993513219	ENSG00000233396	LINC01719	
7.692063283	-0.949391351	1.072518678	-0.885197966	0.376049869	0.982609538	ENSG00000066294	CD84	
7.46984873	1.52166398	1.259059134	1.208572289	0.226827208	0.958865115	ENSG00000206567	AC022007.1	
7.798526132	1.417271979	1.236917332	1.145809783	0.251873867	0.962465655	ENSG00000253540	FAM86HP	
7.689400066	-0.575285018	1.427406537	-0.403028152	0.686927515	0.998869317	ENSG00000138769	CDKL2	
7.877641846	-0.064850123	1.128513489	-0.057465085	0.954174719	0.999527751	ENSG00000146005	PSD2	
7.447602031	0.004899651	1.2455404	0.003933755	0.996861326	0.999527751	ENSG00000081842	PCDHA6	
7.715737366	-0.331464868	1.012373603	-0.327413582	0.743355095	0.999467288	ENSG00000272693	AC073107.1	
7.639243864	1.435523224	1.070900138	1.340482807	0.18008843	0.948583952	ENSG00000213420	GPC2	
7.77503923	0.07518878	1.471223781	0.051106283	0.959240829	0.999527751	ENSG00000197191	CYSRT1	
7.779452975	0.715613257	1.023570455	0.699134342	0.484468078	0.991892545	ENSG00000186907	RTN4RL2	
7.60303266	-0.734263019	1.050420199	-0.699018374	0.484540548	0.991892545	ENSG00000179240	GVQW3	
8.307072887	0.66209021	1.192734702	0.555102663	0.578824441	0.99483168	ENSG00000215146	BX322639.1	

7.79530999	-0.27495401	1.044562826	-0.263224004	0.79237793	0.999467288	ENSG00000212127	TAS2R14
7.725942618	1.209199169	1.209430193	0.999808981	0.317402958	0.974768742	ENSG00000151006	PRSS53
7.910126374	0.092325318	1.166345248	0.079157795	0.936907114	0.999527751	ENSG00000102904	TSNAXIP1
7.654735775	-0.345611439	1.148283664	-0.30098089	0.763429067	0.999467288	ENSG00000161381	PLXDC1
7.847042246	2.375275954	1.201530018	1.976876081	0.048055639	0.831218677	ENSG00000260160	AC011468.1
8.012991395	-0.42852288	1.207147552	-0.354987987	0.722598591	0.999092366	ENSG00000123870	ZNF137P
7.716723678	-0.789063772	0.958599755	-0.823142055	0.410427216	0.990226751	ENSG00000205559	CHKB-DT
7.789316867	1.156246456	1.038169662	1.113735546	0.265392655	0.962465655	ENSG00000234883	MIR155HG
8.14386815	0.499290799	1.372569267	0.363763645	0.716034513	0.99900742	ENSG00000238078	LINC01352
7.822559179	0.139327876	1.148925644	0.121267966	0.903478792	0.999527751	ENSG00000278978	AC092611.3
7.733694337	-1.347908496	1.08248662	-1.24519645	0.213059547	0.954310328	ENSG00000132915	PDE6A
7.634343698	-0.4519481	1.432514963	-0.315492761	0.752387547	0.999467288	ENSG00000250999	AC136604.3
8.159174008	-2.990618251	1.569285432	-1.905719757	0.056686575	0.84929896	ENSG00000154548	SRSF12
7.709253713	-0.446246205	1.021896109	-0.436684514	0.662340162	0.997568624	ENSG00000232640	AL354892.2
8.031044409	-0.749170914	1.291491438	-0.580081982	0.561859334	0.99483168	ENSG00000086730	LAT2
7.589481353	0.363179805	1.012673752	0.358634559	0.71986849	0.99900742	ENSG00000216895	AC009403.1
7.893067498	-1.031952738	1.120254046	-0.921177426	0.356957802	0.979427314	ENSG00000186523	FAM86B1
7.861225297	1.639703377	1.223041703	1.340676588	0.180025479	0.948583952	ENSG00000204177	BMS1P1
7.95808047	-2.547774037	1.972056269	-1.2919378	0.196378686	0.954310328	ENSG00000266524	GDF10
7.879220604	1.109220298	1.035442447	1.071252489	0.284055916	0.963310634	ENSG00000276710	CSPG4P10
7.812636798	0.116313673	1.118413285	0.10399883	0.917170277	0.999527751	ENSG00000172361	CFAP53
7.506040806	-0.190743872	1.069509128	-0.17834712	0.858450376	0.999467288	ENSG00000124466	LYPD3
7.632029457	-1.325176307	1.013999965	-1.306880033	0.191253458	0.949234949	ENSG00000275793	RIMBP3
7.747041932	1.397282065	1.433859064	0.97449052	0.329813044	0.976026345	ENSG00000099984	GSTT2
8.014083659	-0.737374905	1.200091299	-0.61443234	0.538929671	0.99483168	ENSG00000162814	SPATA17
7.63153157	-1.765593329	1.253075732	-1.40900768	0.158832897	0.939464191	ENSG00000159374	M1AP
7.61479309	-0.38681213	4.717065115	-0.082002712	0.934644557	0.999527751	ENSG00000163145	C1QTNF7
7.759793721	-0.842300773	1.076929274	-0.782131931	0.434137043	0.991301167	ENSG00000226887	ERVMER34-1
7.580197302	0.258934078	1.184632099	0.218577631	0.826979079	0.999467288	ENSG00000251359	WWC2-AS2
7.759303158	1.134992275	1.119637353	1.013714193	0.31071915	0.973427483	ENSG00000170629	DPY19L2P2
7.692584489	-1.180071442	1.630201539	-0.723880706	0.469138976	0.99135833	ENSG00000002745	WNT16
7.46603166	-1.390947949	1.787177496	-0.77829312	0.436396231	0.991301167	ENSG00000227471	AKR1B15
7.776804429	-0.448066892	1.308894061	-0.342324795	0.732106478	0.999467288	ENSG00000102385	DRP2
7.656789698	0.284167442	1.046449955	0.271553781	0.785965142	0.999467288	ENSG00000225032	AL162586.1
7.374451904	0.204760459	1.392422305	0.147053417	0.883089858	0.999467288	ENSG00000177685	CRACR2B
7.728164688	0.714266555	1.064986425	0.67068137	0.502423533	0.993513219	ENSG00000172803	SNX32
7.45775324	1.143469346	1.362368358	0.839324651	0.401287154	0.988527641	ENSG00000256268	LINC02454
7.58839116	-0.170965981	1.320330835	-0.129487229	0.89697213	0.999467288	ENSG00000257894	AC027288.3
7.862652243	-0.859312909	1.410893712	-0.609055736	0.542487496	0.99483168	ENSG00000275880	AL139385.1
7.415897524	0.458542059	1.39654252	0.328340923	0.742653906	0.999467288	ENSG00000258733	LINC02328
7.773515251	2.835475674	1.289298835	2.19923853	0.027860966	0.769167109	ENSG00000196696	PDXDC2P-NPIPB14P
7.560740617	-0.137459168	1.171397617	-0.117346293	0.906585642	0.999527751	ENSG00000274712	AC005332.4
7.538479366	1.452396259	1.484439182	0.978414123	0.327869546	0.976026345	ENSG00000101187	SLCO4A1
7.820403784	2.163511384	1.108504367	1.951739162	0.050969181	0.842188482	ENSG00000284428	IPO5P1
7.13555841	0.137969069	1.439084571	0.095872801	0.923621596	0.999527751	ENSG00000104892	KLC3
7.140168577	0.96986137	1.461066504	0.663803713	0.506815949	0.993513219	ENSG00000277117	FP565260.3
7.572142683	-1.435877837	1.104289728	-1.300272746	0.193507505	0.950382812	ENSG00000237380	HOXD-AS2
7.42381009	-0.369213608	1.141248079	-0.323517397	0.746303434	0.999467288	ENSG00000271474	AC106881.1
7.59571637	0.167853812	1.14609238	0.146457489	0.883560247	0.999467288	ENSG00000154608	CEP170P1
7.446223159	-1.37337218	1.145168295	-1.199275412	0.230420872	0.959575179	ENSG00000263432	RN7SL689P
7.658443379	-0.568358366	1.082993825	-0.524802961	0.599720173	0.99483168	ENSG00000135077	HAVCR2
7.630476534	-0.751740422	1.253391186	-0.599765205	0.548662725	0.99483168	ENSG00000135604	STX11
7.896034668	-0.933912995	1.132767858	-0.824452237	0.409682644	0.990226751	ENSG00000253552	HOXA-AS2
7.745409084	-1.006499193	1.42690259	-0.705373443	0.480577866	0.991417002	ENSG00000123965	PMS2P5
7.860578465	-2.043490337	1.259476804	-1.622491443	0.104698164	0.92169898	ENSG00000198416	ZNF658B
7.271271478	-0.014791929	1.159813399	-0.012753715	0.989824284	0.999527751	ENSG00000197859	ADAMTSL2
7.654792777	-0.330087825	1.512138226	-0.218292097	0.827201532	0.999467288	ENSG00000196917	HCAR1
7.574820332	-0.492140634	1.350565897	-0.36439587	0.715562419	0.99900742	ENSG00000256001	AC079949.1
7.328309653	1.160662099	1.10539842	1.049994353	0.293720709	0.970706863	ENSG00000260285	AL133367.1
7.42952786	0.402220223	1.228931875	0.327292531	0.743446641	0.999467288	ENSG00000188659	SAXO2
7.46756607	0.533407869	1.1694201	0.456130238	0.648296327	0.997368416	ENSG00000140470	ADAMTS17
7.495663291	-0.766214236	1.346701004	-0.56895646	0.569385688	0.99483168	ENSG00000198650	TAT
7.546465867	0.225572291	1.244065697	0.181318632	0.856117482	0.999467288	ENSG00000170049	KCNAB3
7.195865111	0.888172072	1.132295014	0.784399878	0.43280551	0.991301167	ENSG00000268573	AC011815.1

7.196907435	0.879554589	1.220347952	0.720740825	0.471068989	0.99135833	ENSG00000181781	ODF3L2
7.379282274	-0.422871157	1.125691533	-0.375654559	0.707173724	0.99900742	ENSG00000230163	AL122010.1
7.458472263	0.94824403	1.126006246	0.842130346	0.399714997	0.988384114	ENSG00000162711	NLRP3
7.228730744	-0.794782905	1.357773276	-0.585357599	0.558307293	0.99483168	ENSG00000114737	CISH
7.380343427	0.279191352	1.187950685	0.235019312	0.814193744	0.999467288	ENSG00000278828	HIST1H3H
7.236503131	0.033801095	1.149459362	0.029406081	0.976540723	0.999527751	ENSG00000183807	FAM162B
7.292879832	0.022795441	1.097079514	0.020778295	0.983422512	0.999527751	ENSG00000218305	CDC14C
7.589818494	-0.506042233	1.079434494	-0.468803096	0.639210387	0.996553586	ENSG00000231245	C1DP1
7.317169327	-0.354230967	1.04247348	-0.33979854	0.734008247	0.999467288	ENSG00000273729	AC007686.3
7.470300196	1.61855937	1.241002392	1.304235496	0.192153301	0.949261383	ENSG00000278600	AC015871.3
7.400567875	1.400394487	1.311180309	1.068041122	0.285501963	0.96478822	ENSG00000171345	KRT19
7.473768468	0.271430014	0.951437591	0.285284097	0.775426496	0.999467288	ENSG00000267280	TBX2-AS1
7.524013982	-0.868973526	1.148216153	-0.756803085	0.449167845	0.99135833	ENSG00000108370	RGS9
7.525931673	-0.825564417	1.054134452	-0.783168044	0.433528438	0.991301167	ENSG00000198691	ABCA4
7.326376894	0.49499274	0.935960787	0.528860554	0.596902188	0.99483168	ENSG00000197959	DNM3
7.598702512	0.154671978	1.193822894	0.129560238	0.896914364	0.999467288	ENSG00000206559	ZCWPW2
7.005639239	0.223679034	1.146092203	0.195166701	0.845262447	0.999467288	ENSG00000085514	PILRA
7.141255982	0.91547954	1.065467483	0.859228043	0.390214714	0.987407688	ENSG00000213199	ASIC3
7.224704659	2.175998621	1.59336206	1.365664889	0.17204417	0.944422693	ENSG00000132554	RGS22
7.210411648	1.602746706	1.250443573	1.281742527	0.199932981	0.954310328	ENSG00000177363	LRRN4CL
7.307631199	1.124441216	1.424038276	0.789614461	0.42975296	0.991301167	ENSG00000149328	GLB1L2
7.470057445	0.005881401	1.056920464	0.005564658	0.995560068	0.999527751	ENSG00000232807	AL137186.2
7.355088504	-0.626334026	1.000937863	-0.625747161	0.531480795	0.99483168	ENSG00000250790	AC127070.2
7.409988566	0.103035728	1.179238357	0.087374811	0.930373591	0.999527751	ENSG00000166206	GABRB3
7.526876094	-0.425062723	1.125856226	-0.377546185	0.705767748	0.99900742	ENSG00000255346	NOX5
7.31454444	0.086422589	1.197811756	0.072150393	0.942482223	0.999527751	ENSG00000184451	CCR10
6.833104768	1.02401591	1.447688003	0.707345718	0.479351661	0.99135833	ENSG00000124116	WFDC3
7.225059143	-1.048865541	1.099324582	-0.954099961	0.340033046	0.977691382	ENSG00000224397	SMIM25
7.366305242	0.128889347	1.095276387	0.117677463	0.906323224	0.999527751	ENSG00000242615	AC022415.1
7.738936393	0.264259033	1.403181032	0.188328538	0.850619115	0.999467288	ENSG00000227925	LINC01655
7.05644251	-0.44382102	1.21775907	-0.364457167	0.715516654	0.99900742	ENSG00000273763	AC007318.2
7.522573743	1.596679941	1.199675078	1.33092699	0.183213035	0.949094286	ENSG00000184307	ZDHHC23
7.557771429	1.120737645	1.24897172	0.89732828	0.36954377	0.981455968	ENSG00000174912	METTL15P1
7.475446621	0.526879581	1.433453017	0.367559714	0.713201559	0.99900742	ENSG00000250241	AC105383.1
7.452314875	-1.11694892	1.357227981	-0.822963375	0.410528821	0.990226751	ENSG00000183439	TRIM61
7.371827784	0.359364266	1.190263611	0.301919896	0.762713128	0.999467288	ENSG00000162148	PPP1R32
7.453279949	-1.149983011	1.3506835	-0.851408203	0.394542637	0.988378925	ENSG00000165495	PKNOX2
7.562586087	0.292727908	1.215972464	0.240735639	0.809760015	0.999467288	ENSG00000260314	MRC1
7.485388556	-2.397200472	1.335871797	-1.79448393	0.072735961	0.886289726	ENSG00000260293	AC106820.4
7.348054908	2.105382496	1.239186697	1.699003468	0.08931853	0.910413547	ENSG00000198028	ZNF560
7.051193426	1.714154934	1.052328264	1.628916558	0.103330684	0.92169898	ENSG00000269399	AC008764.6
7.118492825	-0.078756617	1.17252441	-0.067168424	0.946447622	0.999527751	ENSG00000142530	FAM71E1
7.416141282	0.42554911	1.012347724	0.420358638	0.674223479	0.998869317	ENSG00000268225	AC010487.1
7.131018484	0.429416127	0.972132045	0.441726131	0.658687398	0.997568624	ENSG00000233903	Z83851.1
7.089730964	-0.156574463	1.197176445	-0.130786455	0.895944238	0.999467288	ENSG00000272156	AC008280.3
7.275554757	1.071211754	1.118758901	0.957500095	0.338314898	0.977691382	ENSG00000204305	AGER
7.580041703	-0.541595148	1.672343519	-0.323854006	0.746048567	0.999467288	ENSG00000112499	SLC22A2
6.919143971	-0.899845434	1.280487749	-0.702736465	0.482219999	0.991525194	ENSG00000254827	SLC22A18AS
7.142392973	1.51899779	1.045500257	1.452890881	0.146254053	0.936017041	ENSG00000254703	SENCR
7.113947014	0.212678133	1.008952316	0.210791065	0.833050311	0.999467288	ENSG00000258311	BLOC1S1-RDH5
7.202948017	0.020149972	1.366272764	0.014748132	0.988233119	0.999527751	ENSG00000153233	PTPRR
6.810571557	-1.075740514	1.351320082	-0.796066401	0.425993442	0.991301167	ENSG00000184185	KCNJ12
7.346000556	0.845277266	1.090011541	0.77547552	0.438058725	0.991301167	ENSG00000269693	AC010422.6
7.257200946	-1.936099388	1.196438028	-1.618219533	0.105615295	0.92169898	ENSG00000172346	CSDC2
7.086834979	1.266603539	1.117334184	1.13359419	0.256964811	0.962465655	ENSG00000225972	MTND1P23
6.746908163	0.650748775	1.435371904	0.45336597	0.650285237	0.997381078	ENSG00000115290	GRB14
6.960466238	-0.913996629	1.199140988	-0.76220948	0.445934989	0.99135833	ENSG00000138400	MDH1B
7.028382212	-0.35391776	1.26397056	-0.28000475	0.779473861	0.999467288	ENSG00000164309	CMYA5
6.933325752	-0.318685725	1.086534079	-0.293304859	0.76928914	0.999467288	ENSG00000255408	PCDHA3
6.722833253	0.735314157	1.317856329	0.557962306	0.576870124	0.99483168	ENSG00000120162	MOB3B
6.977193547	1.37902725	1.093043513	1.261639847	0.207078411	0.954310328	ENSG00000187984	ANKRD19P
7.157562574	-0.350403084	1.135561021	-0.30857266	0.757646621	0.999467288	ENSG00000247157	LINC01252
6.878430492	1.099983165	1.147670434	0.958448638	0.337836579	0.977691382	ENSG00000110944	IL23A
7.333134238	0.21894131	1.241699289	0.176323939	0.860039456	0.999467288	ENSG00000258818	RNASE4

6.855075331	-0.822059728	1.28457246	-0.639948117	0.52220633	0.994503371	ENSG00000239246	AC008026.1
7.428846539	-1.752729526	1.228610928	-1.426594446	0.153696826	0.939432741	ENSG00000105672	ETV2
6.865012605	1.828090901	1.070889087	1.707077721	0.087807608	0.908456501	ENSG00000237118	CYP2F2P
7.049770916	-3.029117132	1.693868291	-1.788283746	0.073730241	0.888663898	ENSG00000197588	KLKP1
7.03637206	1.188951186	0.991773788	1.198812875	0.230600715	0.959575179	ENSG00000273271	AP000254.1
7.04060334	0.124748805	1.211148987	0.10300038	0.91796267	0.999527751	ENSG00000143110	C1orf162
6.731718245	0.10972657	1.192244683	0.0920336	0.926671344	0.999527751	ENSG00000123685	BATF3
6.97836642	1.124710064	1.197754446	0.939015562	0.347722755	0.977691382	ENSG00000119737	GPR75
7.214554377	-0.771419899	1.411374205	-0.546573613	0.584671701	0.99483168	ENSG00000145428	RNF175
6.904416998	0.138147016	1.16667765	0.118410613	0.905742317	0.999527751	ENSG00000172901	LVRN
6.895089638	-1.761525339	1.405568546	-1.253247551	0.210115629	0.954310328	ENSG00000133878	DUSP26
6.876431434	1.144422808	1.406414961	0.813716321	0.415807482	0.990226751	ENSG00000164764	SBSPON
7.251177021	-0.239654755	1.40023639	-0.171153068	0.864103409	0.999467288	ENSG00000106714	CNTNAP3
7.080777373	1.118655751	1.428522938	0.783085606	0.433576843	0.991301167	ENSG00000154143	PANX3
6.947534846	-0.578429794	1.051330561	-0.550188319	0.582190215	0.99483168	ENSG00000275457	AL117332.1
6.910263451	2.966807439	1.198086749	2.476287667	0.013275657	0.670420654	ENSG00000272644	AC097468.3
6.672444347	1.323062977	1.464918428	0.903164949	0.366438358	0.981269214	ENSG00000228242	AC093495.1
7.276725347	-0.155517787	1.175917078	-0.132252342	0.894784702	0.999467288	ENSG00000227354	RBM26-AS1
7.006865046	-1.448560159	1.345219608	-1.07682058	0.281560429	0.963267117	ENSG00000269906	AL606834.1
6.934222229	3.155846678	1.354740923	2.329483539	0.019833464	0.73975641	ENSG00000262801	U91319.1
7.061314812	-1.176763943	1.334981917	-0.881483059	0.37805642	0.982609538	ENSG00000049249	TNFRSF9
7.092020596	-0.081249765	1.305104825	-0.062255356	0.95035948	0.999527751	ENSG00000282849	AL359834.1
6.781083485	-2.005789651	1.644140241	-1.219962629	0.222479042	0.956884001	ENSG00000119283	TRIM67
6.693526317	1.878834926	1.511283575	1.243204755	0.213792391	0.954310328	ENSG00000224376	AC017104.1
6.8749147	0.276614571	1.174890573	0.235438582	0.813868343	0.999467288	ENSG00000169302	STK32A
7.129841752	1.380330687	1.320635187	1.045202112	0.295929568	0.970706863	ENSG00000230795	HLA-K
7.130422912	1.672587104	1.66301136	1.005758075	0.314531959	0.974768742	ENSG00000204622	HLA-J
6.818958671	1.275515334	1.169673172	1.090488664	0.275497944	0.962465655	ENSG00000273329	AC078846.1
6.605423514	-3.041771202	1.997257785	-1.522973761	0.12776527	0.928627271	ENSG00000211895	IGHA1
6.843753557	-2.060264815	1.804630906	-1.141654401	0.253597702	0.962465655	ENSG00000211896	IGHG1
6.826366464	0.823072607	1.310559567	0.628031436	0.529983347	0.99483168	ENSG00000169684	CHRNA5
6.743722916	0.009449208	1.167671387	0.008092352	0.993543308	0.999527751	ENSG00000260123	AC013565.1
6.884258301	-0.62096824	1.3161475	-0.471807483	0.637064209	0.99653524	ENSG00000167105	TMEM92
6.973367399	-1.323858422	1.197222633	-1.105774637	0.268824076	0.962465655	ENSG00000181029	TRAPPC5
6.525518738	-0.390934446	1.506464579	-0.259504572	0.795245955	0.999467288	ENSG00000169174	PCSK9
6.54649162	-3.125989361	4.79499406	-0.651927682	0.514447826	0.993513219	ENSG00000204792	LINC01291
6.973271882	-0.346915969	1.10758559	-0.313218204	0.754114889	0.999467288	ENSG00000153237	CCDC148
6.815306256	0.694682627	1.114832963	0.623127096	0.533201005	0.99483168	ENSG00000163491	NEK10
6.744998124	-0.070757283	1.637771095	-0.043203402	0.965539393	0.999527751	ENSG00000112936	C7
6.715039324	0.350910003	1.199758526	0.292483859	0.7699167	0.999467288	ENSG00000197744	PTMAP2
6.77190325	0.026574895	1.247503473	0.021302462	0.98300438	0.999527751	ENSG00000204963	PCDHA7
6.55214704	1.060105819	1.222115016	0.867435393	0.385703496	0.986238739	ENSG00000245275	SAP30L-AS1
6.713421807	1.002204482	1.121967691	0.893256099	0.371720042	0.981955904	ENSG00000272345	AL031775.1
6.628866563	-1.437327068	1.81116894	-0.793590833	0.427433671	0.991301167	ENSG00000275591	XKR5
6.831017465	0.279332967	1.396820279	0.199977743	0.841497988	0.999467288	ENSG00000254876	SUGT1P4-STRA6LP
6.638331654	1.597946096	1.037558936	1.540101522	0.123535609	0.927794614	ENSG00000205885	C1RL-AS1
6.875645725	1.005561386	1.127854933	0.89156979	0.372623568	0.982259938	ENSG00000181350	LRRC75A
6.357212067	0.642574103	1.563223395	0.411057117	0.681030653	0.998869317	ENSG00000077984	CST7
6.622220077	-2.249958538	1.359303356	-1.65522915	0.097878024	0.921147998	ENSG00000104879	CKM
6.402740205	-2.784123323	1.618776414	-1.719893679	0.08545177	0.901307093	ENSG00000146070	PLA2G7
6.630441366	2.507160938	1.353825878	1.851907973	0.064039039	0.868202292	ENSG00000184156	KCNQ3
6.765493877	-0.465668552	1.167951174	-0.398705496	0.690110217	0.998869317	ENSG00000235618	FAM21EP
6.582008292	1.21760199	1.079704939	1.127717347	0.259439309	0.962465655	ENSG00000261644	AC007728.2
6.577087349	0.592445067	1.127769713	0.525324505	0.599357625	0.99483168	ENSG00000279443	AL513497.1
6.478198295	1.255597247	1.254456831	1.000909091	0.316870761	0.974768742	ENSG00000241288	AC092902.2
6.430736943	-0.042117719	1.216781161	-0.034614046	0.972387501	0.999527751	ENSG00000156140	ADAMTS3
6.283372037	3.622807272	1.506430963	2.404894323	0.016177143	0.698816035	ENSG00000138772	ANXA3
6.432582223	0.125416593	1.807206054	0.069398059	0.944672774	0.999527751	ENSG00000248329	APELA
6.381866915	-1.192878758	1.529225082	-0.780054403	0.435358854	0.991301167	ENSG00000198865	CCDC152
6.83348177	-0.284752002	1.146569288	-0.248351326	0.803862591	0.999467288	ENSG00000260000	AL133338.1
6.486938265	0.851516064	1.049222873	0.811568339	0.417039365	0.990226751	ENSG00000172159	FRMD3
6.554956964	-3.151008827	1.872379485	-1.682890061	0.092396376	0.921147998	ENSG00000102837	OLFM4
6.240911512	-0.901728655	1.352833162	-0.666548308	0.505060697	0.993513219	ENSG00000275557	AC242842.1
6.485535254	0.356782068	1.650804765	0.216126144	0.828889421	0.999467288	ENSG00000026751	SLAMF7

6.350251825	-0.047115113	1.070094039	-0.044028946	0.96488133	0.999527751	ENSG00000138030	KHK
6.402995183	-1.269260579	1.442171405	-0.880103832	0.378803063	0.982609538	ENSG00000235997	LINC01936
6.633399229	-0.938407838	1.336530721	-0.702122161	0.482602983	0.991759385	ENSG00000173890	GPR160
6.774837205	1.283314488	1.517352658	0.845758882	0.397687268	0.988378925	ENSG00000272556	GTF2IP13
6.333997183	0.502904479	1.41150726	0.356288978	0.721624163	0.99900742	ENSG00000118113	MMP8
6.275911652	2.64652261	1.510641125	1.751920139	0.079787541	0.897052166	ENSG00000137877	SPTBN5
6.302790545	0.422194037	1.356468732	0.311244946	0.755614417	0.999467288	ENSG00000266554	LINC01443
6.356143819	1.241523118	1.131424594	1.097309643	0.272506057	0.962465655	ENSG00000185513	L3MBTL1
6.761907664	0.676100693	1.563430604	0.432446884	0.665416649	0.997568624	ENSG00000210154	MT-TD
6.293949412	1.526779302	1.295492904	1.178531582	0.238584749	0.962465655	ENSG00000070831	CDC42
6.312588409	-0.50911853	1.195539297	-0.425848428	0.670218296	0.997568624	ENSG00000243422	RPL23AP49
6.715386614	-2.91193007	1.569674459	-1.855117188	0.063579494	0.868202292	ENSG00000228412	AL022068.1
6.175495104	-1.506695228	1.301351052	-1.157793068	0.246948493	0.962465655	ENSG00000198870	STKLD1
6.567392564	1.052196944	1.293082124	0.813712389	0.415809735	0.990226751	ENSG00000226268	AC135977.1
6.260167554	0.263573421	1.09301127	0.241144285	0.809443291	0.999467288	ENSG00000166153	DEPDC4
6.19110436	-0.137038971	1.27860384	-0.107178601	0.914647292	0.999527751	ENSG00000236423	LINC01134
6.675779333	0.940126138	1.42284296	0.660737808	0.508780473	0.993513219	ENSG00000272994	AC012360.3
6.205505117	-1.556306683	1.630046839	-0.95476194	0.339698098	0.977691382	ENSG00000106066	CPVL
6.242302654	0.434958677	1.140668125	0.381319217	0.702966396	0.99900742	ENSG00000260563	AC132872.1
6.354540774	-0.854137299	1.41695888	-0.602796109	0.546644336	0.99483168	ENSG00000203883	SOX18
5.981381091	-1.350535815	1.410266814	-0.957645604	0.338241495	0.977691382	ENSG00000167680	SEMA6B
6.259188419	2.722667794	1.472093686	1.849520733	0.064382657	0.868202292	ENSG00000273373	AL355488.1
6.073836607	-2.743708022	1.431566596	-1.916577287	0.055291656	0.847598083	ENSG00000170396	ZNF804A
6.165489093	-0.243637218	1.135856954	-0.214496391	0.830159981	0.999467288	ENSG00000178075	GRAMD1C
6.077245961	-1.426984025	1.462426476	-0.975764627	0.329181118	0.976026345	ENSG00000244300	GATA2-AS1
6.284583129	0.713624279	1.266324124	0.563539986	0.57306724	0.99483168	ENSG00000280927	CTBP1-AS
6.309092296	-0.169988924	1.622391121	-0.104776784	0.916552932	0.999527751	ENSG00000111846	GCNT2
6.36503258	-0.732219057	1.45741316	-0.502410077	0.615379092	0.99483168	ENSG00000261499	AC233699.1
6.241731933	1.482421311	1.611944781	0.9196477	0.357756891	0.979427314	ENSG00000167851	CD300A
6.201754687	0.545063884	1.37315846	0.396941722	0.691410437	0.998869317	ENSG00000223799	IL10RB-DT
6.184749918	1.940767309	1.170727203	1.657745122	0.097368922	0.921147998	ENSG00000162878	PKDCC
6.207752761	-0.126789123	1.276526634	-0.099323524	0.920881402	0.999527751	ENSG00000254614	AP003068.2
6.373489403	1.769789622	1.267705293	1.39605761	0.162697147	0.943223078	ENSG00000237399	PITRM1-AS1
6.183560259	-0.863607924	1.623739768	-0.531863505	0.594820535	0.99483168	ENSG00000181409	AATK
5.803112251	-0.328699684	1.50521358	-0.218374115	0.827137632	0.999467288	ENSG00000238164	TNFRSF14-AS1
5.968161298	0.907673024	1.282377607	0.707804798	0.479066486	0.99135833	ENSG00000264343	NOTCH2NLA
5.913026436	-4.564339271	1.791169228	-2.548245693	0.010826619	0.638240244	ENSG00000211592	IGKC
5.987451806	-1.041787352	1.190536071	-0.875057361	0.381542696	0.985271081	ENSG00000214820	MPRIPP1
5.990140754	-0.51148582	2.240932702	-0.228246846	0.819454345	0.999467288	ENSG00000174004	NRROS
6.010513136	0.345893007	1.319533889	0.262132719	0.793219122	0.999467288	ENSG00000186212	SOWAHB
5.981214774	2.009478454	1.648933172	1.218653665	0.222975671	0.956884001	ENSG00000164669	INTS4P1
6.291357342	-2.275292658	1.422274271	-1.599756604	0.109652589	0.923922314	ENSG00000169884	WNT10B
6.330374585	0.293090533	4.718412634	0.062116342	0.950470183	0.999527751	ENSG00000140297	GCNT3
6.117570575	-1.960507684	1.321122369	-1.483971303	0.137816534	0.934144427	ENSG00000188095	MESP2
5.902522015	1.993692209	1.384889869	1.439603432	0.149979628	0.936396974	ENSG00000267648	AC060766.5
6.213719839	1.238731953	1.483914446	0.834773161	0.40384544	0.988927059	ENSG00000104899	AMH
5.818404454	0.710370506	1.568031287	0.453033375	0.650524709	0.997381078	ENSG00000277400	AC145212.1
6.130262499	1.09727973	1.512965973	0.725250766	0.468298204	0.99135833	ENSG00000259820	AC083843.2
5.742957711	-0.45698013	1.332409213	-0.342972808	0.731618917	0.999467288	ENSG00000233532	LINC00460
5.888466307	2.20972529	1.297438197	1.703144931	0.088540949	0.910413547	ENSG00000174292	TNK1
5.699336042	-0.29707999	1.702578078	-0.174488321	0.861481709	0.999467288	ENSG00000232855	AF165147.1
5.809308396	3.490054899	1.510731936	2.310174834	0.020878477	0.73997555	ENSG00000204362	AL590644.1
5.727124127	2.852389854	1.408048114	2.025775843	0.042787761	0.806698895	ENSG00000235927	NEXN-AS1
5.712614237	-1.149634195	1.54115026	-0.745958538	0.455692462	0.99135833	ENSG00000150551	LYPD1
5.978185735	0.827124409	1.20293959	0.68758599	0.491713533	0.993513219	ENSG00000239388	ASB14
6.145216997	-0.994154845	1.448621423	-0.686276504	0.492538763	0.993513219	ENSG00000066923	STAG3
5.930423008	0.373600467	1.609745913	0.232086607	0.816470747	0.999467288	ENSG00000149403	GRIK4
5.67653873	2.788954464	1.408781957	1.979692066	0.047738141	0.828704284	ENSG00000187908	DMBT1
5.894197289	0.071526308	4.718087538	0.015160021	0.987904516	0.999527751	ENSG00000137878	GCOM1
5.735055599	1.586019247	1.256098122	1.262655535	0.206713	0.954310328	ENSG00000259658	AC027559.1
6.033309776	-0.642042028	1.237604933	-0.518777851	0.603915665	0.99483168	ENSG00000275496	CU633906.1
5.560815577	-2.326628579	1.645809892	-1.413667879	0.157459441	0.939464191	ENSG00000169442	CD52
5.776814929	1.033998541	1.292373446	0.80007721	0.423666064	0.991301167	ENSG00000273340	MICE
5.85288755	2.229352808	1.381099988	1.614186393	0.106487003	0.922165836	ENSG00000231760	AL355312.2

5.692292592	1.481682996	1.512637669	0.979535963	0.327315228	0.976026345	ENSG00000224975	INE1
5.684701251	1.425378395	1.232555678	1.156441385	0.247500662	0.962465655	ENSG00000269427	AC024075.3
5.646309754	0.95887132	1.289263108	0.743735948	0.457036237	0.99135833	ENSG00000280721	LINC01943
6.057972269	0.064587343	1.665040095	0.038790263	0.969057608	0.999527751	ENSG00000235381	AL596202.1
5.806405904	-1.435195875	1.41588305	-1.013640127	0.310754503	0.973427483	ENSG00000164675	IQUB
5.573289497	1.619022644	1.498704956	1.080281104	0.280017022	0.963267117	ENSG00000155659	VSIG4
5.777214308	-2.425536676	1.412441916	-1.717264724	0.085930814	0.901307093	ENSG00000254560	BBOX1-AS1
5.729571067	-4.215640124	1.782845787	-2.364556797	0.018051659	0.726053908	ENSG00000164120	HPGD
5.422937604	0.391225409	1.538371642	0.254311376	0.799255012	0.999467288	ENSG00000204758	AC008429.1
5.473345411	1.131651794	1.444416939	0.783466161	0.433353417	0.991301167	ENSG00000227486	AL445472.1
5.406415026	-1.133545991	1.529515357	-0.741114489	0.458624025	0.99135833	ENSG00000205456	TP53TG3D
5.727454594	5.156340921	1.786840405	2.885731096	0.003905057	0.454844605	ENSG00000275888	AC132872.3
5.723249898	-0.077582465	1.421720218	-0.054569432	0.956481492	0.999527751	ENSG00000184828	ZBTB7C
5.318522652	-0.217146842	4.720060589	-0.046005096	0.963306188	0.999527751	ENSG00000241104	CEACAMP10
5.75575802	1.618795841	1.525564169	1.061112914	0.288638589	0.966288987	ENSG00000164037	SLC9B1
5.447707541	1.003388879	1.550215207	0.647257796	0.517465111	0.993513219	ENSG00000261324	AC010168.2
5.889327154	1.072800115	1.607323099	0.667445217	0.504487791	0.993513219	ENSG00000100784	RPS6KA5
5.346643954	0.977584157	1.67478101	0.583708647	0.559416349	0.99483168	ENSG00000103313	MEFV
5.352731466	2.29227222	1.479662565	1.549185791	0.121337064	0.923922314	ENSG00000263293	THCAT158
5.435825033	2.102341344	1.296092656	1.622060996	0.104790288	0.92169898	ENSG00000267577	AC010327.3
5.250042225	1.157781101	1.218652146	0.950050517	0.342086585	0.977691382	ENSG00000251867	AC009812.1
5.577318646	0.036539311	1.581573165	0.023103143	0.981567999	0.999527751	ENSG00000266405	CBX3P2
5.237058357	2.376608678	1.397268479	1.700896223	0.088962476	0.910413547	ENSG00000260942	CAPN10-DT
5.438888187	1.567136902	1.588768554	0.986384642	0.323944398	0.975412329	ENSG00000245552	AP000787.1
5.476501034	-0.297369583	4.719673931	-0.063006383	0.949761422	0.999527751	ENSG00000203837	PNLIPRP3
5.154399524	2.276829655	1.371031171	1.660669505	0.096779841	0.921147998	ENSG00000179111	HES7
5.113964403	-0.626121981	1.889185231	-0.331424347	0.740323977	0.999467288	ENSG00000153902	LGI4
5.0246006	1.702698253	1.732060664	0.9830477	0.325583963	0.976026345	ENSG00000143882	ATP6V1C2
5.410783213	-0.373610871	1.521835235	-0.245500211	0.806069145	0.999467288	ENSG00000135472	FAIM2
5.248184856	4.676484199	2.219652292	2.1068544	0.035130202	0.784512596	ENSG00000100739	BDKRB1
5.147996008	0.449670682	1.618564458	0.277820683	0.781150014	0.999467288	ENSG00000103316	CRYM
5.161104647	-1.65638333	1.642558298	-1.008416768	0.313254426	0.973427483	ENSG00000281376	ABALON
5.124154316	0.854299008	1.594676239	0.535719407	0.592152489	0.99483168	ENSG00000141934	PLPP2
5.355781646	2.994702002	1.741527675	1.719583355	0.085508204	0.901307093	ENSG00000280332	AC020917.4
5.197743981	-1.363357781	1.406825429	-0.969102316	0.332494142	0.976026345	ENSG00000262468	LINC01569
4.903152222	1.451584255	1.765491856	0.822198217	0.410964094	0.990226751	ENSG00000126460	PRRG2
5.194671319	4.756168082	2.268258419	2.096836958	0.036008011	0.785478429	ENSG00000213928	IRF9
5.175838821	-0.631463013	1.546376484	-0.408350114	0.683016652	0.998869317	ENSG00000210077	MT-TV
4.954779476	4.803211724	1.962189768	2.447883381	0.014369816	0.684434275	ENSG00000279312	AL136164.4
4.941457569	-0.280099677	1.843030884	-0.151977745	0.879204489	0.999467288	ENSG00000099960	SLC7A4
4.821358137	2.365449747	1.523835431	1.552300004	0.120590457	0.923922314	ENSG00000157303	SUSD3
4.829768572	-0.345625008	4.71219351	-0.073346947	0.941530034	0.999527751	ENSG00000259846	AC092131.1
4.747018182	0.201543017	4.722747504	0.042674951	0.965960648	0.999527751	ENSG00000140465	CYP1A1
4.668886392	-0.04687026	1.405153299	-0.033355976	0.973390716	0.999527751	ENSG00000175426	PCSK1
4.305504892	2.042694907	2.154651062	0.948039774	0.343109206	0.977691382	ENSG00000232765	AL158819.1
4.296389389	-0.837738928	2.07561909	-0.403609184	0.686500131	0.998869317	ENSG00000198074	AKR1B10
3.751983224	4.251646441	2.485261196	1.710744306	0.087128325	0.90492251	ENSG00000010327	STAB1
4.087423991	2.8198898	2.080866259	1.355151869	0.175369176	0.946278819	ENSG00000283236	AC074141.1
3.795711852	1.239733388	2.174423513	0.570143479	0.568580387	0.99483168	ENSG00000260228	AC009119.1
3.812191071	0.943677693	2.031880757	0.464435568	0.642335712	0.996740283	ENSG00000273295	AP000350.6

DMOG_d7 vs CTR_d7

baseMean	log2FoldChange	lfcSE	stat	pvalue	padj	gene_id	gene_name
836617.341	0.256102976	0.411473478	0.622404578	0.533675871	0.999975616	ENSG00000115414	FN1
607209.4415	-0.23371275	0.846838358	-0.275982716	0.782561342	0.999975616	ENSG00000108821	COL1A1
377493.6585	-0.128210722	0.181995037	-0.704473727	0.481137806	0.999975616	ENSG00000164692	COL1A2
276619.4416	0.417635097	0.450429164	0.927193731	0.353825957	0.999975616	ENSG00000120708	TGFBI
221928.4399	-0.285083676	0.175391566	-1.625412687	0.104074654	0.987682951	ENSG00000113140	SPARC
211230.9357	-0.424615546	1.045808747	-0.406016442	0.68473052	0.999975616	ENSG00000198804	MT-CO1
148280.5096	0.197399674	0.236082163	0.836148194	0.403071539	0.999975616	ENSG00000141753	IGFBP4
138468.9625	-0.114868653	0.290420987	-0.395524627	0.69245575	0.999975616	ENSG00000137801	THBS1
117801.0114	-0.220306156	0.214434685	-1.027381165	0.304241017	0.999975616	ENSG00000156508	EEF1A1
118430.1866	-0.478197557	0.267823886	-1.785492566	0.074181456	0.950333634	ENSG00000168542	COL3A1
115574.4082	1.363155374	0.840986361	1.620900692	0.105038938	0.987682951	ENSG00000146674	IGFBP3
112693.5513	-0.198654744	0.38765998	-0.51244584	0.608339016	0.999975616	ENSG00000198886	MT-ND4
103486.6261	-0.223317184	0.349648914	-0.63868977	0.523024769	0.999975616	ENSG00000133110	POSTN
106699.4452	-0.010407809	0.400965087	-0.025956896	0.979291719	0.999975616	ENSG00000105664	COMP
97763.92112	-0.408481242	0.303833683	-1.344423824	0.178811374	0.999975616	ENSG00000210082	MT-RNR2
91610.65186	-0.123127982	0.201982663	-0.609596786	0.542128941	0.999975616	ENSG00000075624	ACTB
83506.4108	-0.087621465	0.351831231	-0.249044022	0.80332673	0.999975616	ENSG00000198712	MT-CO2
74009.85526	1.016533399	0.648380883	1.567802854	0.116927149	0.999975616	ENSG00000106366	SERPINE1
71158.15676	-0.388619757	0.374810706	-1.036842735	0.299809156	0.999975616	ENSG00000198938	MT-CO3
68770.8487	0.100884559	0.19304772	0.522588713	0.601260497	0.999975616	ENSG00000184009	ACTG1
65040.01421	-0.231938322	0.353767463	-0.655623669	0.512066293	0.999975616	ENSG00000198727	MT-CYB
63307.6516	-0.28545754	0.310052357	-0.920675277	0.357219988	0.999975616	ENSG00000144810	COL8A1
61510.12721	0.123997068	0.169861299	0.729990108	0.465396231	0.999975616	ENSG00000163430	FSTL1
58724.66708	-0.469895754	0.273575411	-1.717609604	0.085867847	0.973741696	ENSG00000087086	FTL
57129.01918	-0.065203244	0.188661862	-0.345609033	0.729636556	0.999975616	ENSG00000196924	FLNA
56096.11109	-0.288603435	0.213767679	-1.350079846	0.176990372	0.999975616	ENSG00000060718	COL11A1
56743.08966	0.464998356	0.701176447	0.663168819	0.507222439	0.999975616	ENSG00000130635	COL5A1
54810.87725	1.189054375	0.651222036	1.825881664	0.067868089	0.939405498	ENSG00000134333	LDHA
53292.75387	0.097024377	0.289999814	0.334567031	0.737951718	0.999975616	ENSG00000111799	COL12A1
51918.76701	-0.093455565	0.229051229	-0.408011627	0.683265139	0.999975616	ENSG00000182492	BGN
50980.594	0.847552922	0.626882376	1.352012682	0.176371258	0.999975616	ENSG00000111640	GAPDH
52425.69871	-0.193509523	0.365209809	-0.529858505	0.596210038	0.999975616	ENSG00000198786	MT-ND5
50476.64981	-0.244941204	0.39964326	-0.612899624	0.539942712	0.999975616	ENSG00000198899	MT-ATP6
48792.28758	0.019316305	0.170665474	0.113182269	0.909886053	0.999975616	ENSG00000087245	MMP2
50560.21165	0.22994633	0.376720623	0.610389544	0.541603793	0.999975616	ENSG00000163359	COL6A3
48464.61863	0.328232388	0.214983201	1.526781562	0.126815338	0.999975616	ENSG00000118523	CTGF
46133.68263	0.087502177	0.224003534	0.390628556	0.696071815	0.999975616	ENSG00000122786	CALD1
46304.07993	0.104077346	0.241392438	0.431154127	0.666356306	0.999975616	ENSG00000166033	HTRA1
45389.13007	0.054974625	0.243422125	0.225840707	0.821325315	0.999975616	ENSG00000187498	COL4A1
41790.81693	0.190676366	0.19716854	0.967072972	0.333507556	0.999975616	ENSG00000164733	CTSB
41726.45627	-0.113193439	0.273052737	-0.414547902	0.678472894	0.999975616	ENSG00000183087	GAS6
41783.49206	0.815564853	0.812071172	1.004302185	0.315232981	0.999975616	ENSG00000100234	TIMP3
41820.9389	-0.485652387	0.301650912	-1.6099815	0.107401895	0.989669467	ENSG00000150687	PRSS23
41894.64371	0.050937661	0.21926958	0.232306099	0.816300276	0.999975616	ENSG00000011465	DCN
39170.92811	0.01709343	0.252545858	0.067684459	0.946036821	0.999975616	ENSG00000186340	THBS2
38111.68277	-0.212766224	0.202609864	-1.050127669	0.293659419	0.999975616	ENSG00000167460	TPM4
36184.82522	0.087185038	0.241002198	0.361760345	0.717531128	0.999975616	ENSG00000182718	ANXA2
36316.05462	0.018080413	0.196911726	0.091819891	0.92684114	0.999975616	ENSG00000142173	COL6A2
35856.89208	-0.386517329	0.190872048	-2.025007504	0.042866594	0.855568497	ENSG00000137809	ITGA11
35024.25571	-0.116474775	0.32903116	-0.353993144	0.723344021	0.999975616	ENSG00000166147	FBN1
34194.66747	-0.096933272	0.207072825	-0.468111989	0.639704506	0.999975616	ENSG00000128595	CALU
33296.79179	-0.462501555	0.451531804	-1.024294525	0.305696183	0.999975616	ENSG00000139329	LUM
33004.13867	0.077460094	0.277810417	0.278823575	0.780380221	0.999975616	ENSG00000134871	COL4A2
32829.04842	-0.005517028	0.179093216	-0.030805342	0.97542478	0.999975616	ENSG00000150093	ITGB1
32782.32368	1.002089987	0.414215659	2.419246993	0.015552676	0.666269005	ENSG00000074800	ENO1
32069.82685	1.049363891	0.39424391	2.661712369	0.007774428	0.507415783	ENSG00000135919	SERPINE2
31986.37454	-0.306661	0.318478975	-0.962892448	0.3356015	0.999975616	ENSG00000167996	FTH1
32366.78973	-0.107094128	0.322369183	-0.332209572	0.739731016	0.999975616	ENSG00000198763	MT-ND2
31841.52048	0.025961753	0.181858405	0.14275806	0.886481261	0.999975616	ENSG00000100345	MYH9
31372.31377	0.496365442	0.23884291	2.078208817	0.03769013	0.835184568	ENSG00000067225	PKM

30670.96394	0.273296514	0.263988667	1.035258511	0.3005482	0.999975616	ENSG00000026025	VIM	
29548.26541	0.060186514	0.308927865	0.194823843	0.845530856	0.999975616	ENSG00000161638	ITGA5	
30259.14507	0.096784147	0.414283782	0.233617996	0.815281556	0.999975616	ENSG00000049540	ELN	
28336.76926	-0.883704062	0.372288567	-2.373707224	0.017610508	0.670428193	ENSG00000107796	ACTA2	
28409.42277	1.402888651	0.436694791	3.212515193	0.001315782	0.274639877	ENSG00000152952	PLOD2	
27652.73637	-0.419678755	0.291414894	-1.440141748	0.1498273	0.999975616	ENSG00000149591	TAGLN	
27389.22967	-0.077587938	0.230230867	-0.337000588	0.736116457	0.999975616	ENSG00000197746	PSAP	
27392.96074	-0.030148755	0.332539817	-0.090662092	0.927761093	0.999975616	ENSG00000117984	CTSD	
26584.13069	0.609751782	0.239476999	2.546180986	0.010890871	0.567086032	ENSG00000166923	GREM1	
26355.51285	-0.256783782	0.275895318	-0.93072903	0.351993749	0.999975616	ENSG00000101335	MYL9	
26472.09916	-0.518503318	0.251456662	-2.061998727	0.039207857	0.848069004	ENSG00000125868	DSTN	
26260.76334	-0.007516518	0.169202345	-0.044423248	0.964567031	0.999975616	ENSG00000204262	COL5A2	
25783.96577	0.056062379	0.268453874	0.208834307	0.834577589	0.999975616	ENSG00000140545	MFGE8	
26116.77625	-0.037561389	0.310598832	-0.120932164	0.903744766	0.999975616	ENSG00000123384	LRP1	
25571.05347	-0.029970244	0.208561219	-0.143699983	0.885737385	0.999975616	ENSG00000164176	EDIL3	
25777.4986	0.039970128	0.26082718	0.153243721	0.87820608	0.999975616	ENSG00000168036	CTNNB1	
25525.12132	-0.415081191	0.203139302	-2.04333276	0.041019509	0.854443069	ENSG00000167658	EEF2	
24587.69524	-0.272891808	0.25283998	-1.079306401	0.28045116	0.999975616	ENSG00000166710	B2M	
24710.73282	1.133169103	0.433992894	2.611031464	0.009026959	0.526777378	ENSG00000113739	STC2	
24360.54709	1.050964172	0.413125245	2.543935972	0.010961117	0.568653138	ENSG00000113083	LOX	
23070.33227	0.048159262	0.148597224	0.324092607	0.745867924	0.999975616	ENSG00000142156	COL6A1	
23191.05697	-0.274667004	0.341320967	-0.804717644	0.420982636	0.999975616	ENSG00000198888	MT-ND1	
22248.48224	0.109132989	0.229850228	0.474800436	0.634929222	0.999975616	ENSG00000125148	MT2A	
22717.93142	-0.234406142	0.350681373	-0.668430547	0.503858802	0.999975616	ENSG00000198695	MT-ND6	
21979.71542	-0.064452069	0.194037978	-0.332162133	0.739766835	0.999975616	ENSG00000082781	ITGB5	
22264.58879	-0.174044173	0.270349255	-0.6437753	0.519721153	0.999975616	ENSG00000119681	LTBP2	
21719.95951	0.200730281	0.181985507	1.103001466	0.270026529	0.999975616	ENSG00000087460	GNAS	
21577.88254	-0.00502942	0.250817728	-0.02005209	0.984001819	0.999975616	ENSG00000163453	IGFBP7	
21634.67525	-0.280849752	0.266135295	-1.055289385	0.291292994	0.999975616	ENSG00000152661	GJA1	
21632.12089	-0.06831588	0.204225729	-0.33451162	0.737993524	0.999975616	ENSG00000087116	ADAMTS2	
21394.2198	-0.183160963	0.181257489	-1.01050149	0.312255086	0.999975616	ENSG00000072110	ACTN1	
21198.02628	-0.054457035	0.182361423	-0.298621466	0.765228885	0.999975616	ENSG00000140416	TPM1	
21142.30039	-0.179873053	0.207689429	-0.866067441	0.386453179	0.999975616	ENSG00000089157	RPLP0	
20308.85716	-0.246300452	0.247274174	-0.996062178	0.319219935	0.999975616	ENSG00000038427	VCAN	
19460.72559	0.05462535	0.200088418	0.273006059	0.784848558	0.999975616	ENSG00000149257	SERPINH1	
19139.8078	0.161626851	0.251075569	0.643737865	0.519745432	0.999975616	ENSG00000185624	P4HB	
18390.19484	-0.00763574	0.194779837	-0.039201899	0.968729419	0.999975616	ENSG00000106211	HSPB1	
18766.80717	0.286511728	0.205854192	1.39181877	0.163977285	0.999975616	ENSG00000169504	CLIC4	
18318.67559	-0.686326629	0.678492278	-1.011546706	0.311754838	0.999975616	ENSG00000100644	HIF1A	
18407.38737	0.014059256	0.182072016	0.077218106	0.938450038	0.999975616	ENSG00000138061	CYP1B1	
18405.39355	0.230942483	0.193578303	1.193018429	0.232862156	0.999975616	ENSG00000115310	RTN4	
18211.84073	-0.1506168	0.196891674	-0.764972927	0.444287666	0.999975616	ENSG00000133112	TPT1	
17259.75467	-0.237841868	0.176790548	-1.345331356	0.178518252	0.999975616	ENSG00000035862	TIMP2	
17825.14095	0.808678619	0.681981496	1.185777948	0.235710002	0.999975616	ENSG00000134013	LOXL2	
16817.20538	1.049735447	0.752163175	1.395621963	0.162828365	0.999975616	ENSG00000102144	PGK1	
16192.88485	-0.401543044	0.317986849	-1.262766198	0.206673216	0.999975616	ENSG00000198467	TPM2	
16497.9693	0.230464574	0.247813341	0.929992602	0.352374915	0.999975616	ENSG00000134294	SLC38A2	
16197.7282	-0.107305729	0.356494467	-0.301002509	0.763412582	0.999975616	ENSG00000044574	HSPA5	
15907.28784	-0.380156768	0.244681181	-1.553682092	0.120260267	0.999975616	ENSG00000157227	MMP14	
15855.78397	0.110194782	0.144848717	0.760757737	0.446801782	0.999975616	ENSG00000110321	EIF4G2	
15534.25906	0.015836239	0.259716026	0.060975211	0.951378951	0.999975616	ENSG00000135404	CD63	
15628.16899	-0.186588659	0.139659787	-1.336022796	0.181541822	0.999975616	ENSG00000115677	HDLBP	
15388.11596	0.364147438	0.24191619	1.505262789	0.132256528	0.999975616	ENSG00000150991	UBC	
15460.93301	-0.215609324	0.28376045	-0.759828664	0.447357007	0.999975616	ENSG00000011028	MRC2	
15311.76414	0.042107247	0.250949497	0.167791715	0.86674714	0.999975616	ENSG00000166598	HSP90B1	
15425.18546	0.44107264	0.284717523	1.549158745	0.121343564	0.999975616	ENSG00000091986	CCDC80	
15107.7331	0.545258008	0.199812102	2.728853768	0.006355488	0.483939657	ENSG00000102265	TIMP1	
14847.59317	0.037680462	0.319617243	0.117892457	0.90615287	0.999975616	ENSG00000141429	GALNT1	
14282.47785	-0.107060129	0.293208207	-0.365133467	0.71501178	0.999975616	ENSG00000205542	TMSB4X	
14286.09099	0.37970037	0.223157341	1.70149173	0.088850689	0.9781243	ENSG00000034510	TMSB10	
14409.595	0.09769466	0.168132828	0.581056425	0.561202425	0.999975616	ENSG00000164111	ANXA5	
14553.55817	-0.07313427	0.178176857	-0.410458862	0.681469375	0.999975616	ENSG00000138758		11-Sep
14083.66717	0.383149584	0.269194586	1.423318314	0.1546439	0.999975616	ENSG00000142871	CYR61	
14011.77016	0.450018407	0.25657043	1.753976118	0.079434599	0.962537177	ENSG00000100097	LGALS1	

14085.80676	0.414081696	0.20230876	2.046780854	0.040679606	0.852456137	ENSG00000124942	AHNAK
14446.24864	0.153423835	0.330664522	0.463986382	0.642657503	0.999975616	ENSG00000130508	PXDN
14031.77336	-0.379164632	0.222736558	-1.702300852	0.088698985	0.9781243	ENSG00000109971	HSPA8
13559.98103	-0.219741428	0.211174422	-1.04056839	0.298075908	0.999975616	ENSG00000142192	APP
13564.91962	-0.366306838	0.181698533	-2.016014286	0.043798482	0.860814587	ENSG00000174444	RPL4
13361.59477	-0.025170565	0.225833838	-0.111456128	0.911254653	0.999975616	ENSG00000179218	CALR
13134.83224	-0.278741968	0.225359106	-1.236879101	0.21613197	0.999975616	ENSG00000142541	RPL13A
13035.17263	0.101366054	0.160853014	0.630178145	0.528578036	0.999975616	ENSG00000183255	PTTG1IP
13158.61836	0.284812408	0.718153464	0.396589897	0.691669904	0.999975616	ENSG00000137573	SULF1
12995.31561	-0.197079344	0.208042702	-0.947302366	0.343484725	0.999975616	ENSG00000100316	RPL3
13107.04106	0.020646341	0.257883462	0.08006074	0.936188947	0.999975616	ENSG00000127022	CANX
12353.97944	0.17436727	0.207654062	0.839700742	0.401076198	0.999975616	ENSG00000067560	RHOA
12864.0602	0.273994889	0.638737136	0.428963455	0.66794982	0.999975616	ENSG00000041982	TNC
12124.71943	0.096532534	0.246450478	0.391691407	0.69528624	0.999975616	ENSG00000177469	CAVIN1
12076.05961	-0.295903677	0.269188386	-1.099243846	0.271661719	0.999975616	ENSG00000129116	PALLD
12235.90148	-0.23784137	0.246687678	-0.964139644	0.334975919	0.999975616	ENSG00000140937	CDH11
12140.65935	-0.226973585	0.206846574	-1.097304055	0.272508499	0.999975616	ENSG00000137818	RPLP1
12099.55332	-0.070256469	0.177871794	-0.394983753	0.692854878	0.999975616	ENSG00000136156	ITM2B
12112.67539	-0.652587448	0.24076615	-2.710461782	0.006718959	0.492649482	ENSG00000138448	ITGAV
12057.48812	-0.613061948	0.330309104	-1.856024977	0.063449998	0.937668618	ENSG00000211459	MT-RNR1
11927.04718	0.300597372	0.223379449	1.345680518	0.178405572	0.999975616	ENSG00000086062	B4GALT1
11817.50089	0.132350623	0.171972387	0.769603916	0.441534882	0.999975616	ENSG00000139644	TMBIM6
11792.3872	0.80755585	0.423627018	1.906289768	0.056612621	0.917136198	ENSG00000111669	TPI1
11577.05612	-0.023564037	0.204488234	-0.115234195	0.908259496	0.999975616	ENSG00000169604	ANTXR1
11636.90902	-0.138971945	0.188515324	-0.737191769	0.461005738	0.999975616	ENSG00000185896	LAMP1
11426.0024	-0.135976418	0.204998684	-0.663303858	0.507135966	0.999975616	ENSG00000135862	LAMC1
10935.42532	0.042350456	0.271485856	0.155995072	0.876036905	0.999975616	ENSG00000092841	MYL6
11082.33266	0.30592542	0.333568819	0.917128349	0.359075388	0.999975616	ENSG00000196230	TUBB
11239.87856	0.03127285	0.246821552	0.126702266	0.899176052	0.999975616	ENSG00000006016	CRLF1
11129.38546	0.420858271	0.305878901	1.375898339	0.16885311	0.999975616	ENSG00000083444	PLOD1
10946.91787	-0.28400636	0.293186227	-0.968689295	0.332700235	0.999975616	ENSG00000136235	GPNMB
10926.22891	0.143940981	0.169866177	0.847378703	0.396784071	0.999975616	ENSG00000164924	YWHAZ
10891.5412	-0.208745641	0.200945697	-1.038816173	0.298890238	0.999975616	ENSG00000140988	RPS2
10903.30452	-0.332702481	0.161718941	-2.057288269	0.039658505	0.848069004	ENSG00000070756	PABPC1
10835.6103	-0.319572442	0.249579513	-1.280443411	0.200389235	0.999975616	ENSG00000166741	NNMT
10806.17779	-0.127917582	0.308924157	-0.414074391	0.678819627	0.999975616	ENSG00000164694	FNDC1
10503.18336	0.110915773	0.254330518	0.436108784	0.662757804	0.999975616	ENSG00000196262	PPIA
10727.70693	0.23054728	0.233789593	0.986131488	0.324068594	0.999975616	ENSG00000188783	PRELP
10510.72107	-0.097809235	0.180220041	-0.542721189	0.587321783	0.999975616	ENSG00000147403	RPL10
10325.75907	0.012169742	0.213145104	0.057096043	0.954468689	0.999975616	ENSG00000134243	SORT1
10337.17725	-0.17574285	0.16125883	-1.089818467	0.275793117	0.999975616	ENSG00000168028	RPSA
10363.26904	0.182143778	0.229150793	0.79486427	0.42669246	0.999975616	ENSG00000182534	MXRA7
10161.04013	-0.130315978	0.334378239	-0.389726254	0.696738981	0.999975616	ENSG00000124762	CDKN1A
10256.04896	0.617275582	0.264872972	2.33045893	0.019781908	0.682776641	ENSG00000083857	FAT1
10117.63405	-0.061399841	0.140558528	-0.436827578	0.662236397	0.999975616	ENSG00000009307	CSDE1
10187.193	-0.163225825	0.211596604	-0.771400965	0.440469303	0.999975616	ENSG00000058262	SEC61A1
10118.67358	-0.362286035	0.189280449	-1.914017194	0.055617959	0.916132452	ENSG00000198034	RPS4X
9987.239551	-0.12208947	0.23092624	-0.52869466	0.597017282	0.999975616	ENSG00000096384	HSP90AB1
9836.460983	0.141700508	0.251715245	0.562939713	0.573475937	0.999975616	ENSG00000100243	CYB5R3
9940.85848	-0.087057451	0.215706573	-0.403592015	0.686512758	0.999975616	ENSG00000084234	APLP2
9970.63155	0.099414969	0.167268808	0.594342548	0.552283027	0.999975616	ENSG00000134250	NOTCH2
9867.783581	0.463626079	0.244691907	1.894734012	0.058127649	0.922712543	ENSG00000150938	CRIM1
9891.133849	-0.277296528	0.173915601	-1.594431588	0.110839402	0.997355609	ENSG00000174748	RPL15
9811.705189	0.102279047	0.239071083	0.42781856	0.668783223	0.999975616	ENSG00000113389	NPR3
9718.950593	-0.338310132	0.34616868	-0.977298502	0.328421394	0.999975616	ENSG00000154096	THY1
9757.806183	0.113205698	0.192999151	0.586560598	0.557498852	0.999975616	ENSG00000067167	TRAM1
9800.732982	-0.287587146	0.228950632	-1.25610986	0.209076142	0.999975616	ENSG00000167526	RPL13
9837.888166	-0.53468702	0.336403326	-1.589422511	0.111965036	0.997355609	ENSG00000198840	MT-ND3
9705.941146	0.022588152	0.165662686	0.136350274	0.891544382	0.999975616	ENSG00000137076	TLN1
9601.042423	0.076891064	0.172169232	0.446601654	0.655162701	0.999975616	ENSG00000065978	YBX1
9458.826656	0.205805501	0.27355782	0.752329073	0.451853188	0.999975616	ENSG00000110092	CCND1
9472.45754	-0.234013355	0.181540116	-1.289044867	0.197382489	0.999975616	ENSG00000130402	ACTN4
9367.558377	0.048953096	0.231930016	0.211068395	0.832833901	0.999975616	ENSG00000172757	CFL1
9501.508071	0.158583442	0.225017682	0.704759912	0.48095966	0.999975616	ENSG00000173812	EIF1

9494.115672	-0.512385833	0.250759591	-2.043334933	0.041019294	0.854443069	ENSG00000158747	NBL1	
9455.816265	-0.059424288	0.171556739	-0.346382711	0.729055116	0.999975616	ENSG00000141756	FKBP10	
9248.490102	-0.119508476	0.150947153	-0.791723945	0.428521657	0.999975616	ENSG00000204628	RACK1	
9281.806554	0.189620353	0.227988216	0.831711199	0.405571978	0.999975616	ENSG00000167004	PDIA3	
9109.810063	0.177267122	0.32567743	0.544302755	0.586233151	0.999975616	ENSG00000085063	CD59	
9149.155993	-0.225240047	0.207437229	-1.085822676	0.277557452	0.999975616	ENSG00000148303	RPL7A	
9364.517135	-0.288636616	0.249124358	-1.158604556	0.246617411	0.999975616	ENSG00000113721	PDGFRB	
9197.091567	0.203482001	0.301495048	0.674909928	0.499732993	0.999975616	ENSG00000004399	PLXND1	
9127.104641	-0.05382752	0.155919344	-0.345226695	0.729923951	0.999975616	ENSG00000197324	LRP10	
8892.265936	0.077785454	0.218783997	0.355535391	0.722188536	0.999975616	ENSG00000108518	PFN1	
9008.065783	-0.241127837	0.390519335	-0.617454285	0.536935125	0.999975616	ENSG00000116774	OLFML3	
8986.337143	-0.020158094	0.336400671	-0.059922871	0.952217064	0.999975616	ENSG00000122641	INHBA	
9062.659944	0.354185376	0.395606713	0.895296678	0.370628516	0.999975616	ENSG00000142798	HSPG2	
8840.570075	-0.369150233	0.220356306	-1.675242429	0.093886676	0.9781243	ENSG00000105372	RPS19	
8686.903539	0.224045693	0.169530866	1.321562843	0.186313763	0.999975616	ENSG00000078369	GNB1	
8551.422826	0.080170062	0.229551784	0.349246086	0.72690457	0.999975616	ENSG00000071127	WDR1	
8652.023975	0.047682914	0.196803185	0.242287308	0.808557548	0.999975616	ENSG00000080824	HSP90AA1	
8558.223275	0.253779923	0.175891719	1.442819051	0.149071451	0.999975616	ENSG00000177697	CD151	
8744.625655	-1.041842045	0.288328936	-3.613380115	0.000302231	0.129712105	ENSG00000106624	AEBP1	
8531.916456	0.193923366	0.210141784	0.922821549	0.356100209	0.999975616	ENSG00000162734	PEA15	
8563.292137	-0.030927133	0.250941211	-0.123244536	0.901913459	0.999975616	ENSG00000136240	KDELR2	
8564.469905	-0.237962048	0.222738085	-1.068349169	0.285363037	0.999975616	ENSG00000154380	ENAH	
8633.068742	0.075427275	0.222451931	0.339072241	0.734555309	0.999975616	ENSG00000196576	PLXNB2	
8496.150642	0.713630274	0.397653684	1.794602445	0.072717063	0.946591699	ENSG00000141526	SLC16A3	
8400.994147	-0.147086937	0.207419172	-0.709128938	0.478244467	0.999975616	ENSG00000168374	ARF4	
8483.756881	0.009952679	0.269986042	0.036863681	0.970593698	0.999975616	ENSG00000049323	LTBP1	
8272.71137	0.117500063	0.256520037	0.458054134	0.64691355	0.999975616	ENSG00000143933	CALM2	
8264.910805	0.049524681	0.215522968	0.229788413	0.818256189	0.999975616	ENSG00000172270	BSG	
8285.810046	-0.190437071	0.17481181	-1.089383327	0.275984881	0.999975616	ENSG00000089009	RPL6	
8196.324938	-0.246785895	0.197005571	-1.252684859	0.210320418	0.999975616	ENSG00000137154	RPS6	
8130.147698	-0.18210248	0.191507004	-0.950892008	0.341659199	0.999975616	ENSG00000161016	RPL8	
8079.665786	0.112995323	0.317283523	0.356133599	0.721740516	0.999975616	ENSG00000116260	QSOX1	
8108.085754	-0.276860646	0.22247138	-1.24447759	0.213323842	0.999975616	ENSG00000142534	RPS11	
8106.018108	-0.131070635	0.184580042	-0.710101881	0.47764096	0.999975616	ENSG00000138760	SCARB2	
8182.062988	0.05029915	0.296790743	0.169476816	0.865421606	0.999975616	ENSG00000171603	CLSTN1	
7998.452907	0.034285895	0.184243869	0.186089749	0.852374381	0.999975616	ENSG00000138071	ACTR2	
8170.714858	-0.003489927	0.33212714	-0.010507805	0.991616139	0.999975616	ENSG00000112655	PTK7	
7938.041303	0.294112611	0.198771464	1.479652085	0.138966119	0.999975616	ENSG00000178209	PLEC	
7961.685641	0.064329137	0.179178474	0.359022686	0.719578118	0.999975616	ENSG00000141367	CLTC	
7875.593601	0.017642615	0.187280759	0.094204096	0.924947031	0.999975616	ENSG00000277443	MARCKS	
7928.560506	-0.076682448	0.36991823	-0.207295672	0.835778954	0.999975616	ENSG00000148848	ADAM12	
7958.360478	0.079270789	0.33343897	0.237737026	0.812085064	0.999975616	ENSG00000124225	PMEPA1	
7714.121049	0.112262936	0.172301721	0.651548548	0.514692449	0.999975616	ENSG00000101160	CTSZ	
7697.248902	-0.153798447	0.25352703	-0.606635305	0.544092965	0.999975616	ENSG00000035403	VCL	
7716.509289	0.151669016	0.158427931	0.957337601	0.338396882	0.999975616	ENSG00000111897	SERINC1	
7750.597594	0.049288756	0.199231474	0.247394426	0.804602987	0.999975616	ENSG00000082641	NFE2L1	
7745.195872	0.127431797	0.21417402	0.594991854	0.551848916	0.999975616	ENSG00000073712	FERMT2	
7674.425758	-0.10471938	0.171067991	-0.612150638	0.540438097	0.999975616	ENSG00000168385		2-Sep
7682.781916	0.127286572	0.197207455	0.645445034	0.518638828	0.999975616	ENSG00000110651	CD81	
7726.379816	0.644639454	0.328066561	1.964965438	0.049418238	0.893790775	ENSG00000171314	PGAM1	
7674.367612	0.948077256	0.782208574	1.212051731	0.225492579	0.999975616	ENSG00000114480	GBE1	
7471.812146	-0.707802987	0.260493105	-2.717165923	0.006584359	0.492649482	ENSG00000198959	TGM2	
7634.765152	-0.228376392	0.183224286	-1.246430793	0.212606282	0.999975616	ENSG00000108298	RPL19	
7628.208565	-0.228294916	0.15615658	-1.461961552	0.143751746	0.999975616	ENSG00000229117	RPL41	
7642.772993	-0.394164704	0.190736925	-2.066535908	0.038777905	0.848069004	ENSG00000231500	RPS18	
7866.095953	-0.365453835	0.362529415	-1.008066711	0.313422437	0.999975616	ENSG00000212907	MT-ND4L	
7556.75922	0.193149874	0.179495312	1.076071972	0.281895069	0.999975616	ENSG00000104904	OAZ1	
7748.619181	1.083580361	0.724488042	1.495649752	0.134744951	0.999975616	ENSG00000122884	P4HA1	
7554.714508	-0.058760576	0.160456113	-0.366209647	0.714208644	0.999975616	ENSG00000161203	AP2M1	
7554.228213	0.229604722	0.219455895	1.046245408	0.295447743	0.999975616	ENSG00000145730	PAM	
7547.219193	-0.206539966	0.200996102	-1.027581944	0.304146521	0.999975616	ENSG00000149273	RPS3	
7530.481226	0.30668911	0.209794881	1.461852206	0.143781714	0.999975616	ENSG00000148248	SURF4	
7507.418517	-0.136353558	0.181582098	-0.750919606	0.45270104	0.999975616	ENSG00000112697	TMEM30A	
7503.486447	0.128940767	0.197349823	0.65336145	0.513523285	0.999975616	ENSG00000147065	MSN	

7505.517368	-0.03607181	0.183436094	-0.196645108	0.844105269	0.999975616	ENSG00000170348	TMED10
7383.466402	-0.036166373	0.174821907	-0.206875522	0.836107074	0.999975616	ENSG00000123562	MORF4L2
7450.589071	-0.170204545	0.273756823	-0.621736266	0.5341153	0.999975616	ENSG00000206503	HLA-A
7386.909347	-0.019309576	0.205027395	-0.094180467	0.924965801	0.999975616	ENSG00000068697	LAPTM4A
7388.404333	-0.024792125	0.231681315	-0.1070096	0.914781363	0.999975616	ENSG00000030582	GRN
7436.195623	-0.262068181	0.200469523	-1.307271932	0.191120373	0.999975616	ENSG00000196205	EEF1A1P5
7253.674441	0.346993206	0.21715105	1.597934737	0.110057502	0.995962615	ENSG00000135046	ANXA1
7329.219106	0.326665427	0.256702941	1.272542595	0.2031804	0.999975616	ENSG00000157514	TSC22D3
7286.928251	0.111519699	0.181379572	0.61484156	0.53865936	0.999975616	ENSG00000167123	CERCAM
7323.118765	0.174434747	0.191314745	0.911768439	0.361890628	0.999975616	ENSG00000198542	ITGBL1
7210.11223	-0.353347534	0.269361592	-1.31179628	0.18958888	0.999975616	ENSG00000115419	GLS
7320.922971	0.095560779	0.25860264	0.36952747	0.711734601	0.999975616	ENSG00000172037	LAMB2
7207.233254	0.427455617	0.178793482	2.390778521	0.016812692	0.670428193	ENSG00000158710	TAGLN2
7257.41198	0.141780693	0.176800845	0.801923163	0.422597407	0.999975616	ENSG00000047849	MAP4
7151.35254	-0.19635959	0.21725058	-0.903839197	0.366080674	0.999975616	ENSG00000159176	CSRP1
7222.994626	0.487353817	0.263335656	1.850694373	0.064213534	0.937668618	ENSG00000070404	FSTL3
7113.30262	-0.150140361	0.23635327	-0.63523708	0.525273803	0.999975616	ENSG00000197043	ANXA6
7144.681774	0.392287876	0.267841372	1.464627638	0.143022537	0.999975616	ENSG00000136830	FAM129B
7075.496	0.937974133	0.328368741	2.856465972	0.004283859	0.447970647	ENSG00000105974	CAV1
7127.977221	0.013340551	0.217569489	0.061316275	0.95110733	0.999975616	ENSG00000101439	CST3
7058.316714	-0.082143637	0.210369753	-0.390472661	0.696187067	0.999975616	ENSG00000173706	HEG1
7069.557106	-0.085315166	0.154851959	-0.550946633	0.581670259	0.999975616	ENSG00000122642	FKBP9
6990.108805	3.656332454	1.063692478	3.437396173	0.000587336	0.207084475	ENSG00000167772	ANGPTL4
7040.959298	-0.147743941	0.202839802	-0.728377467	0.466382553	0.999975616	ENSG00000123989	CHPF
7078.773206	-0.231956219	0.200217304	-1.158522336	0.246650942	0.999975616	ENSG00000142937	RPS8
7032.417119	1.593504242	0.932286373	1.709243305	0.08740589	0.975734123	ENSG00000117394	SLC2A1
6857.44383	0.000857877	0.230354103	0.003724168	0.997028551	0.999975616	ENSG00000101608	MYL12A
6913.804711	0.007400171	0.159117292	0.04650765	0.962905637	0.999975616	ENSG00000143761	ARF1
6956.149669	-0.257814573	0.196708836	-1.310640527	0.18997924	0.999975616	ENSG00000105640	RPL18A
6931.662727	0.287661172	0.23966399	1.200268643	0.230035024	0.999975616	ENSG00000136026	CKAP4
6960.372999	-0.967027611	0.26282372	-3.679377226	0.000233804	0.118313828	ENSG00000168461	RAB31
6856.921216	-0.202498906	0.274693973	-0.737180009	0.461012889	0.999975616	ENSG00000204525	HLA-C
6845.103998	-0.266942053	0.272081654	-0.981110079	0.326538461	0.999975616	ENSG00000110955	ATP5F1B
6876.171639	-0.087346044	0.16282157	-0.536452537	0.59164583	0.999975616	ENSG00000174437	ATP2A2
6754.098803	-0.14906332	0.248644063	-0.599504841	0.548836283	0.999975616	ENSG00000136153	LMO7
6847.537884	0.314492513	0.20578372	1.528267219	0.126446205	0.999975616	ENSG00000044115	CTNNA1
6714.130847	0.076368964	0.201103849	0.379748894	0.704131822	0.999975616	ENSG00000114353	GNAI2
6777.626156	0.010952806	0.177171549	0.061820343	0.950705903	0.999975616	ENSG00000135535	CD164
6753.339607	0.273868836	0.272753833	1.004087946	0.315336225	0.999975616	ENSG00000024422	EHD2
6742.45737	-0.227817342	0.18930031	-1.20347052	0.228794293	0.999975616	ENSG00000135677	GNS
6628.029001	-0.166816102	0.21258116	-0.784717245	0.432619369	0.999975616	ENSG00000002586	CD99
6654.457235	-0.058170012	0.219030777	-0.265579169	0.790563331	0.999975616	ENSG00000179222	MAGED1
6590.018707	-0.068052574	0.248794408	-0.273529356	0.784446329	0.999975616	ENSG00000050165	DKK3
6546.80093	1.476890154	0.786760463	1.877178917	0.060493576	0.92582322	ENSG00000104419	NDRG1
6697.881527	-0.00414042	0.255562501	-0.016201202	0.987073876	0.999975616	ENSG00000089597	GANAB
6706.233399	0.502889719	0.396035387	1.269810061	0.204152296	0.999975616	ENSG00000072682	P4HA2
6460.160291	0.486347324	0.222967256	2.181249985	0.029164932	0.773485712	ENSG00000109846	CRYAB
6538.236402	-0.534710945	0.180354032	-2.964785086	0.003028944	0.383026258	ENSG00000065534	MYLK
6510.510622	-0.013400847	0.213928676	-0.062641658	0.950051856	0.999975616	ENSG00000114850	SSR3
6466.578176	0.02488487	0.22766644	0.109304077	0.912961313	0.999975616	ENSG00000197879	MYO1C
6543.634461	-0.049003567	0.193707906	-0.252976596	0.800286299	0.999975616	ENSG00000134352	IL6ST
6400.943676	-0.162241569	0.166037785	-0.977136434	0.328501612	0.999975616	ENSG00000077549	CAPZB
6432.863435	-0.211302031	0.184282944	-1.146617402	0.251539781	0.999975616	ENSG00000008988	RPS20
6379.117151	-0.040341774	0.218970269	-0.184234026	0.853829867	0.999975616	ENSG00000115091	ACTR3
6422.743444	-0.108620831	0.166270276	-0.653278707	0.513576617	0.999975616	ENSG00000162576	MXRA8
6366.40529	-0.074777067	0.17666848	-0.423262074	0.672104062	0.999975616	ENSG00000104763	ASAH1
6418.91495	-0.299168968	0.242069207	-1.235881967	0.216502441	0.999975616	ENSG00000118257	NRP2
6289.557795	0.071949198	0.291398158	0.246910271	0.804977668	0.999975616	ENSG00000115380	EFEMP1
6391.527892	0.344681659	0.256249522	1.345101669	0.178592404	0.999975616	ENSG00000170017	ALCAM
6206.369668	-0.038974746	0.333984296	-0.116696344	0.907100688	0.999975616	ENSG00000077942	FBLN1
6174.622557	0.010690178	0.189604585	0.056381432	0.955037949	0.999975616	ENSG00000108828	VAT1
6268.761676	0.086937675	0.203314936	0.427601025	0.66894162	0.999975616	ENSG00000116209	TMEM59
6234.389927	-0.356198662	0.194884955	-1.827738125	0.067588863	0.939405498	ENSG00000166441	RPL27A
6348.749697	-0.129579169	0.297046235	-0.436225589	0.662673064	0.999975616	ENSG00000133816	MICAL2

6261.06314	-0.251631516	0.17700825	-1.421580719	0.155148005	0.999975616	ENSG00000145425	RPS3A
6279.969621	0.374256525	0.253080317	1.47880534	0.139192348	0.999975616	ENSG00000106105	GARS
6237.352426	-0.397640514	0.194876384	-2.040475634	0.041302975	0.855344797	ENSG00000197756	RPL37A
6097.979112	0.095010677	0.217874712	0.436079415	0.662779112	0.999975616	ENSG00000134531	EMP1
5983.880118	0.18908475	0.331826373	0.569830385	0.568792745	0.999975616	ENSG00000197956	S100A6
6139.083284	0.071140841	0.276537732	0.257255458	0.796981575	0.999975616	ENSG00000196549	MME
6144.039977	-0.164457952	0.184253226	-0.892564842	0.372090254	0.999975616	ENSG00000197965	MPZL1
5970.031256	-0.131551048	0.296903081	-0.44307741	0.657709744	0.999975616	ENSG00000142089	IFITM3
6076.610362	0.020064403	0.158359147	0.126701889	0.899176351	0.999975616	ENSG00000114867	EIF4G1
6052.055158	-0.243152864	0.199345756	-1.21975441	0.222557989	0.999975616	ENSG00000170275	CRTAP
5944.675189	0.008973964	0.342566161	0.026196295	0.979100771	0.999975616	ENSG00000115457	IGFBP2
6196.501933	0.727119122	0.338829396	2.14597414	0.031875042	0.794279453	ENSG00000099194	SCD
6045.679014	2.047395876	0.996786927	2.053995513	0.039976126	0.84890004	ENSG00000112715	VEGFA
6171.613985	1.196925176	0.731993599	1.635157983	0.102015934	0.987682951	ENSG00000103257	SLC7A5
6172.8038	-0.156196389	0.282011251	-0.553865807	0.579670688	0.999975616	ENSG00000265972	TXNIP
6061.017366	-0.21085698	0.183264122	-1.150563335	0.249911924	0.999975616	ENSG00000122406	RPL5
6018.888896	-0.01567726	0.188277856	-0.083266615	0.933639546	0.999975616	ENSG00000005893	LAMP2
5988.08992	-0.223201995	0.304431968	-0.733175286	0.463451527	0.999975616	ENSG00000103196	CRISPLD2
5947.064053	-0.37650503	0.301182756	-1.250088269	0.211267305	0.999975616	ENSG00000116016	EPAS1
5961.724936	-0.272853415	0.187871493	-1.452340697	0.146406897	0.999975616	ENSG00000108107	RPL28
5897.588008	-0.453596496	0.253307399	-1.790695799	0.073342124	0.947863913	ENSG00000169100	SLC25A6
5881.713286	0.233650306	0.18258159	1.279703535	0.200649421	0.999975616	ENSG00000113719	ERGIC1
5985.60767	-0.100016403	0.315757142	-0.31675104	0.751432517	0.999975616	ENSG00000197081	IGF2R
5873.109276	-0.362808861	0.629255243	-0.576568674	0.564230866	0.999975616	ENSG00000117318	ID3
5843.713221	0.041763572	0.199093436	0.209768703	0.833848201	0.999975616	ENSG00000163466	ARPC2
6063.700677	-0.130121129	0.311564512	-0.417637838	0.676211928	0.999975616	ENSG00000107731	UNC5B
5990.731765	-0.906662767	0.370441281	-2.447520872	0.014384279	0.6423619	ENSG00000143153	ATP1B1
5839.033633	0.136234633	0.155798265	0.874429714	0.381884282	0.999975616	ENSG00000134308	YWHAQ
5780.979832	-0.518403876	0.244027196	-2.12436927	0.033639278	0.802075905	ENSG00000127314	RAP1B
5840.490999	0.193501721	0.177997619	1.087102864	0.276991352	0.999975616	ENSG00000172638	EFEMP2
5568.916562	1.456774942	0.440489933	3.307169664	0.000942438	0.251240009	ENSG00000147872	PLIN2
5807.320877	0.033408947	0.222089436	0.15043015	0.880425256	0.999975616	ENSG00000118705	RPN2
5729.364963	-0.204182364	0.225780007	-0.904342095	0.365814032	0.999975616	ENSG00000162909	CAPN2
5668.865636	0.381347221	0.276840885	1.377496034	0.16835894	0.999975616	ENSG00000160789	LMNA
5733.717283	0.063782591	0.158182183	0.403222345	0.686784663	0.999975616	ENSG00000165119	HNRNPK
5697.338901	-0.219932948	0.216377875	-1.016429931	0.309424698	0.999975616	ENSG00000075415	SLC25A3
5744.429309	0.082266325	0.182412425	0.450990798	0.651996181	0.999975616	ENSG00000163399	ATP1A1
5693.55996	-0.208139718	0.177955664	-1.169615583	0.242155704	0.999975616	ENSG00000138674	SEC31A
5743.416853	0.105364413	0.221180452	0.476373081	0.633808609	0.999975616	ENSG00000131711	MAP1B
5692.3653	-0.194764104	0.172749639	-1.127435664	0.259558327	0.999975616	ENSG00000112306	RPS12
5656.489432	1.446610152	0.828664178	1.745713391	0.080860767	0.965624825	ENSG00000148344	PTGES
5587.308027	-0.172673955	0.208490611	-0.828209737	0.40755173	0.999975616	ENSG00000139926	FRMD6
5560.796527	-0.21304586	0.178316647	-1.194761472	0.232180237	0.999975616	ENSG00000163110	PDLIM5
5620.7279	-0.257183639	0.243381058	-1.056711813	0.290643132	0.999975616	ENSG00000164574	GALNT10
5604.091642	-0.001398459	0.173728759	-0.008049666	0.993577365	0.999975616	ENSG00000061676	NCKAP1
5579.429646	-0.101930162	0.179799311	-0.566910749	0.570774821	0.999975616	ENSG00000117519	CNN3
5600.557898	0.656597339	0.323971709	2.026711966	0.042691878	0.855568497	ENSG00000128272	ATF4
5518.661231	-0.458844463	0.214556815	-2.13856858	0.032470624	0.794279453	ENSG00000096060	FKBP5
5589.127031	0.90475633	0.41970618	2.155689799	0.031107895	0.789571901	ENSG00000104765	BNIP3L
5519.672071	0.12552416	0.165375448	0.759025363	0.447837385	0.999975616	ENSG00000077147	TM9SF3
5473.460701	0.949368854	0.412554597	2.301195677	0.021380573	0.686650924	ENSG00000067057	PFKP
5448.691627	-0.197285507	0.247194716	-0.798097589	0.42481386	0.999975616	ENSG00000102024	PLS3
5477.01369	-0.249005027	0.194333197	-1.281330368	0.200077651	0.999975616	ENSG00000164587	RPS14
5392.489413	0.335684021	0.211005304	1.590879542	0.111636688	0.997355609	ENSG00000106991	ENG
5339.398261	0.569135482	0.2268258	2.509130273	0.012102884	0.599346666	ENSG00000115641	FHL2
5376.124816	0.04694639	0.164734046	0.284982926	0.775657223	0.999975616	ENSG00000185787	MORF4L1
5354.653406	0.098539562	0.169727336	0.580575672	0.561526472	0.999975616	ENSG00000108953	YWHAE
5398.194047	-0.471598178	0.194025585	-2.430597893	0.015073933	0.65785616	ENSG00000234745	HLA-B
5316.665464	0.140924642	0.210753901	0.668669196	0.503706522	0.999975616	ENSG00000063660	GPC1
5317.037527	0.003597462	0.176240875	0.020412188	0.983714562	0.999975616	ENSG00000152558	TMEM123
5335.427028	0.131894866	0.211446486	0.623774218	0.532775874	0.999975616	ENSG00000163902	RPN1
5290.077117	0.046076799	0.206915917	0.222683688	0.823781698	0.999975616	ENSG00000163191	S100A11
5261.519914	0.17016684	0.248149503	0.685743223	0.492875045	0.999975616	ENSG00000168615	ADAM9
5326.023612	0.671576186	0.347930549	1.93020184	0.053581834	0.903530068	ENSG00000105220	GPI

5343.607083	-0.006553113	0.228243203	-0.028711098	0.977095005	0.999975616	ENSG00000187109	NAP1L1
5308.89743	-0.099298419	0.210376103	-0.47200427	0.636923741	0.999975616	ENSG00000163527	STT3B
5246.816429	-0.08680751	0.242459227	-0.358029312	0.720321377	0.999975616	ENSG00000122862	SRGN
5220.962319	-0.065425443	0.249705798	-0.262010109	0.793313649	0.999975616	ENSG00000088832	FKBP1A
5276.692993	-0.262035286	0.199472065	-1.313644021	0.188966027	0.999975616	ENSG00000071967	CYBRD1
5245.052505	0.185451386	0.189683822	0.977686893	0.328229206	0.999975616	ENSG00000162704	ARPC5
5249.766963	-0.137659841	0.203117595	-0.677734695	0.497939924	0.999975616	ENSG00000105193	RPS16
5233.070695	-0.564983286	0.291069779	-1.941057875	0.052251258	0.903530068	ENSG00000076706	MCAM
5214.193099	-0.237917522	0.204096033	-1.1657136	0.24373026	0.999975616	ENSG00000112977	DAP
5320.668592	0.686464469	0.394846288	1.738561283	0.082111962	0.969369989	ENSG00000135069	PSAT1
5172.740614	-0.278083108	0.175493876	-1.584574431	0.113063058	0.997355609	ENSG00000144713	RPL32
5177.108672	-0.171156357	0.244268648	-0.700689006	0.483497118	0.999975616	ENSG00000140092	FBLN5
5165.077155	-0.105982965	0.150557702	-0.70393586	0.481472718	0.999975616	ENSG00000221983	UBA52
5091.208432	0.138123613	0.234024029	0.590211244	0.555049035	0.999975616	ENSG00000138119	MYOF
5105.507453	-0.0883301	0.181050991	-0.487874161	0.625638978	0.999975616	ENSG00000197702	PARVA
5105.066524	0.038563549	0.189450719	0.203554515	0.838701641	0.999975616	ENSG00000129353	SLC44A2
5111.521471	-0.007132367	0.172233643	-0.041410997	0.966968246	0.999975616	ENSG00000086598	TMED2
5122.840184	-0.334237699	0.195768124	-1.707314203	0.087763668	0.976257053	ENSG00000125691	RPL23
5118.101557	0.194727731	0.202254368	0.962786285	0.335654785	0.999975616	ENSG00000198363	ASPH
5095.909994	0.047911066	0.159159944	0.301024648	0.7633957	0.999975616	ENSG00000166913	YWHAB
5065.490464	0.430080748	0.343911001	1.250558275	0.211095682	0.999975616	ENSG00000167552	TUBA1A
4838.616538	2.181973866	0.826905509	2.638722132	0.008321916	0.515028071	ENSG00000115461	IGFBP5
5084.568994	-0.229748496	0.176223126	-1.303736356	0.192323489	0.999975616	ENSG00000138326	RPS24
5077.625064	0.594671422	0.24423109	2.434871914	0.014897062	0.657280648	ENSG00000077782	FGFR1
4957.563868	0.419092789	0.364295952	1.150418461	0.24997156	0.999975616	ENSG00000120129	DUSP1
5073.73191	0.139405534	0.205398305	0.678708299	0.497322707	0.999975616	ENSG00000140575	IQGAP1
5008.436446	0.588403672	0.237217396	2.480440652	0.013122011	0.622303364	ENSG00000176014	TUBB6
5000.494327	0.267924179	0.171356675	1.563546789	0.117924039	0.999975616	ENSG00000148180	GSN
5132.456318	-0.762952926	0.805882243	-0.946730038	0.34377636	0.999975616	ENSG00000123500	COL10A1
5050.789643	0.228479617	0.189808264	1.203739033	0.22869046	0.999975616	ENSG00000075420	FNDC3B
5020.260226	-0.21854934	0.195225469	-1.119471458	0.262939061	0.999975616	ENSG00000083845	RPS5
4995.076006	-0.106910354	0.156488283	-0.683184404	0.494490326	0.999975616	ENSG00000260032	NORAD
4906.203025	0.059279438	0.265289819	0.223451613	0.823184038	0.999975616	ENSG00000002834	LASP1
4941.439659	0.196791793	0.293916614	0.669549742	0.503144862	0.999975616	ENSG00000138080	EMILIN1
4931.96433	0.250205114	0.238330641	1.049823529	0.293799255	0.999975616	ENSG00000065308	TRAM2
4970.165011	-0.250843338	0.199000479	-1.260516254	0.207483188	0.999975616	ENSG00000147604	RPL7
4970.33691	0.118108206	0.201641434	0.585733811	0.558054409	0.999975616	ENSG00000162733	DDR2
4921.856166	0.129459187	0.152050079	0.85142466	0.394533498	0.999975616	ENSG00000165280	VCP
4967.306043	-0.306092435	0.184069946	-1.662913695	0.096329713	0.9781243	ENSG00000162244	RPL29
4903.036541	-0.171863017	0.167424093	-1.026513056	0.304649813	0.999975616	ENSG00000143947	RPS27A
5001.961284	1.084763251	0.874825334	1.239976951	0.214983919	0.999975616	ENSG00000176171	BNIP3
4876.29646	-0.255480614	0.217424049	-1.175033837	0.239981179	0.999975616	ENSG00000197958	RPL12
4905.121143	0.01429317	0.15446713	0.092532111	0.92627528	0.999975616	ENSG00000132824	SERINC3
4825.067037	-0.052092858	0.322954126	-0.161301107	0.871856252	0.999975616	ENSG00000119280	C1orf198
4857.582724	0.147939875	0.292445604	0.505871428	0.612946911	0.999975616	ENSG00000206538	VGLL3
4873.987854	0.45065856	0.310186517	1.452863151	0.146261754	0.999975616	ENSG00000079308	TNS1
4872.769315	0.323810691	0.233119591	1.389032512	0.164822864	0.999975616	ENSG00000118816	CCNI
4794.784138	-0.315105952	0.243611922	-1.293475085	0.195846796	0.999975616	ENSG00000064666	CNN2
4855.362872	0.075533716	0.225682597	0.334690032	0.737858922	0.999975616	ENSG00000070614	NDST1
4804.168556	-0.243550809	0.175304552	-1.389301113	0.164741206	0.999975616	ENSG00000131469	RPL27
4765.481916	-0.11433857	0.21959496	-0.520679389	0.602590135	0.999975616	ENSG00000213625	LEPROT
4768.587521	0.068216401	0.189023332	0.360888787	0.718182586	0.999975616	ENSG00000126247	CAPNS1
4829.079369	-0.041379363	0.179159935	-0.230963264	0.817343342	0.999975616	ENSG00000152291	TGOLN2
4783.4558	-0.16782549	0.253277743	-0.662614441	0.507577519	0.999975616	ENSG00000181163	NPM1
4845.082838	0.007903313	0.179958159	0.043917503	0.964970163	0.999975616	ENSG00000213639	PPP1CB
4858.417221	0.742491833	0.30154969	2.462253681	0.013806697	0.634883942	ENSG00000096696	DSP
4807.005191	0.087471439	0.187547197	0.466396942	0.6409314	0.999975616	ENSG00000169439	SDC2
4784.173485	-0.063273988	0.190881474	-0.33148313	0.740279582	0.999975616	ENSG00000133030	MPRIP
4802.01719	-0.174373399	0.200889679	-0.868005763	0.385391178	0.999975616	ENSG00000075223	SEMA3C
4783.270769	0.093430458	0.226539636	0.412424333	0.680028431	0.999975616	ENSG00000074695	LMAN1
4787.311159	0.261484791	0.194675096	1.343185626	0.179211875	0.999975616	ENSG00000197102	DYNC1H1
4782.949715	-0.24856502	0.228319118	-1.088673701	0.276297803	0.999975616	ENSG00000182752	PAPPA
4739.834462	-0.15646937	0.159767184	-0.979358628	0.327402811	0.999975616	ENSG00000108946	PRKAR1A
4734.122038	-0.042107666	0.242282193	-0.173795958	0.862025821	0.999975616	ENSG00000109099	PMP22

4796.700292	0.071196811	0.249803102	0.285011718	0.775635165	0.999975616	ENSG00000196305	IARS
4683.562643	0.094726115	0.171536565	0.552221127	0.580796861	0.999975616	ENSG00000136238	RAC1
4662.872722	-0.173068946	0.404556887	-0.427798788	0.668797619	0.999975616	ENSG00000156804	FBXO32
4682.18321	0.179944304	0.16975164	1.06004457	0.289124323	0.999975616	ENSG00000215301	DDX3X
4618.89525	0.153279937	0.182900281	0.838051949	0.402001528	0.999975616	ENSG00000204592	HLA-E
4701.589748	-0.034292517	0.196338151	-0.174660485	0.861346419	0.999975616	ENSG00000108654	DDX5
4640.044279	-0.301201812	0.244937967	-1.22970651	0.218807028	0.999975616	ENSG00000170889	RPS9
4653.109074	0.110606469	0.17446843	0.633962653	0.526105196	0.999975616	ENSG00000170027	YWHAG
4625.493089	-0.511553834	0.268178488	-1.907512557	0.056454247	0.917136198	ENSG00000198431	TXNRD1
4625.852447	-0.273614259	0.207260591	-1.32014609	0.186786247	0.999975616	ENSG00000064601	CTSA
4651.7364	0.316708664	0.228278992	1.387375429	0.165327313	0.999975616	ENSG00000166825	ANPEP
4598.421448	-0.047135873	0.177831673	-0.265058931	0.790964064	0.999975616	ENSG00000130985	UBA1
4656.976384	0.465945792	0.427720968	1.089368601	0.275991372	0.999975616	ENSG00000107562	CXCL12
4570.909811	0.16482454	0.212614925	0.775225637	0.438206342	0.999975616	ENSG00000198668	CALM1
4555.549302	-0.009290853	0.209503632	-0.044346978	0.964627825	0.999975616	ENSG00000113732	ATP6V0E1
4580.265928	-0.171252834	0.208883069	-0.81985024	0.412301486	0.999975616	ENSG00000198755	RPL10A
4586.815155	-0.073338102	0.195346404	-0.375425915	0.707343735	0.999975616	ENSG00000182220	ATP6AP2
4500.987982	-0.776204867	0.431355577	-1.799454809	0.071946766	0.945224913	ENSG00000183160	TMEM119
4554.204618	-0.212329436	0.1745806	-1.216225839	0.223898903	0.999975616	ENSG00000142676	RPL11
4537.750363	0.040022448	0.156972114	0.254965339	0.79874987	0.999975616	ENSG00000078808	SDF4
4529.500154	-0.170730151	0.1980381	-0.862107596	0.388628312	0.999975616	ENSG00000182670	TTC3
4537.108977	-0.003331556	0.176565603	-0.018868659	0.984945881	0.999975616	ENSG00000143384	MCL1
4672.425231	0.094747452	0.30163067	0.314117435	0.753431848	0.999975616	ENSG00000104415	WISP1
4493.210374	0.12597409	0.217938133	0.578026839	0.563245999	0.999975616	ENSG00000110880	CORO1C
4495.554344	0.186490862	0.275737854	0.676333914	0.498828665	0.999975616	ENSG00000213626	LBH
4500.477197	-0.040220561	0.153813803	-0.261488633	0.793715715	0.999975616	ENSG00000075785	RAB7A
4509.328042	0.022057389	0.18294487	0.120568502	0.904032818	0.999975616	ENSG00000104805	NUCB1
4517.424225	0.614323802	0.259253956	2.369583135	0.017808151	0.672578247	ENSG00000145623	OSMR
4523.556257	0.551778956	0.196319026	2.810623946	0.004944554	0.458380363	ENSG00000103335	PIEZO1
4469.357512	0.295598839	0.181347085	1.630017037	0.103097896	0.987682951	ENSG00000135926	TMBIM1
4502.710254	-0.066238431	0.160396809	-0.412966015	0.679631514	0.999975616	ENSG00000105887	MTPN
4473.685922	-0.164201336	0.256774658	-0.639476408	0.522513057	0.999975616	ENSG00000161011	SQSTM1
4424.127197	0.691798195	0.450057798	1.537131895	0.124261014	0.999975616	ENSG00000148677	ANKRD1
4596.601229	-0.099918753	0.289193662	-0.345508102	0.72971242	0.999975616	ENSG00000107957	SH3PXD2A
4501.20087	-0.32566625	0.188822622	-1.72472052	0.084577851	0.971784007	ENSG00000071082	RPL31
4567.886494	0.673761032	0.383468197	1.757019325	0.078914515	0.962537177	ENSG00000168003	SLC3A2
4477.245016	-0.187913122	0.1713982	-1.096354118	0.272923835	0.999975616	ENSG00000163682	RPL9
4496.045824	0.193909059	0.233114034	0.831820614	0.405510207	0.999975616	ENSG00000125844	RRBP1
4457.677523	0.470920096	0.336671663	1.398751807	0.161887423	0.999975616	ENSG00000069849	ATP1B3
4409.407716	1.561363283	0.404230868	3.862553328	0.000112208	0.093482515	ENSG00000074410	CA12
4418.044621	-0.10202121	0.213379793	-0.478120296	0.632564587	0.999975616	ENSG00000073921	PICALM
4459.675905	0.310347503	0.293535769	1.057273202	0.29038692	0.999975616	ENSG00000184575	XPOT
4362.995517	0.400292365	0.228874384	1.748960967	0.080297767	0.965624825	ENSG00000111186	WNT5B
4408.01383	0.156663555	0.181216942	0.864508325	0.387308709	0.999975616	ENSG00000137845	ADAM10
4381.037828	0.034276887	0.188987352	0.181371326	0.856076124	0.999975616	ENSG00000166311	SMPD1
4371.806965	-0.388114766	0.261971545	-1.481514973	0.138469398	0.999975616	ENSG00000152234	ATP5F1A
4385.725145	-0.130714643	0.178663167	-0.731626138	0.46439679	0.999975616	ENSG00000095139	ARCN1
4362.215055	-0.140098327	0.156897711	-0.892927799	0.371895839	0.999975616	ENSG00000100934	SEC23A
4426.369917	0.055877785	0.2540984	0.219906087	0.825944296	0.999975616	ENSG00000090863	GLG1
4327.025146	0.146510866	0.203961574	0.718325824	0.472556411	0.999975616	ENSG00000118680	MYL12B
4388.308043	0.07434312	0.21098251	0.35236627	0.724563595	0.999975616	ENSG00000125304	TM9SF2
4317.440006	-0.065883617	0.264786081	-0.248818281	0.80350135	0.999975616	ENSG00000130309	COLGALT1
4327.803219	0.173893617	0.341106286	0.50979306	0.610196448	0.999975616	ENSG00000143878	RHOB
4324.200537	-0.157269482	0.168811009	-0.931630481	0.351527525	0.999975616	ENSG00000063177	RPL18
4312.49961	0.112328393	0.233695009	0.480662353	0.630756492	0.999975616	ENSG00000117385	P3H1
4328.490705	0.390357529	0.272636505	1.431787462	0.152204657	0.999975616	ENSG00000169764	UGP2
4284.564931	-0.022356147	0.188911533	-0.118341884	0.905796772	0.999975616	ENSG00000170759	KIF5B
4262.134718	-0.055532054	0.283500433	-0.195879962	0.844704122	0.999975616	ENSG00000135486	HNRNPA1
4262.823123	0.165470641	0.243621486	0.679212018	0.497003534	0.999975616	ENSG00000142552	RCN3
4295.917492	-0.095722113	0.204444988	-0.468204743	0.639638181	0.999975616	ENSG00000105223	PLD3
4239.947058	0.364358946	0.274353453	1.328064004	0.184156964	0.999975616	ENSG00000114019	AMOTL2
4225.908163	-0.453515628	0.391881454	-1.157277597	0.247158963	0.999975616	ENSG00000140285	FGF7
4308.725925	-0.044868569	0.346058931	-0.129655861	0.896838705	0.999975616	ENSG00000072274	TFRC
4287.581192	0.281751958	0.295942047	0.952051125	0.341071054	0.999975616	ENSG00000051108	HERPUD1

4297.878373	-0.194185578	0.185712489	-1.045624766	0.29573431	0.999975616	ENSG00000174807	CD248
4214.322159	-0.160258485	0.159771782	-1.003046239	0.315838552	0.999975616	ENSG00000196923	PDLIM7
4254.658368	0.112812207	0.24141273	0.467300156	0.640285145	0.999975616	ENSG00000168487	BMP1
4264.277179	0.0319307	0.307054526	0.103990325	0.917177026	0.999975616	ENSG00000071537	SEL1L
4295.731346	0.229971461	0.270137102	0.851313867	0.394595024	0.999975616	ENSG00000105281	SLC1A5
4220.172658	0.122343077	0.231746746	0.527917129	0.597556855	0.999975616	ENSG00000140941	MAP1LC3B
4221.2582	-0.254827218	0.256557676	-0.993255091	0.320585657	0.999975616	ENSG00000182326	C1S
4170.194397	0.245822333	0.207058712	1.187210767	0.235144495	0.999975616	ENSG00000023191	RNH1
4155.633756	-0.219708388	0.148164402	-1.482868924	0.138109239	0.999975616	ENSG00000138069	RAB1A
4166.058097	0.068053679	0.227193625	0.299540442	0.76452772	0.999975616	ENSG00000151348	EXT2
4152.250553	-0.17803741	0.165269519	-1.077254964	0.281366376	0.999975616	ENSG00000104388	RAB2A
4133.154832	-0.082780782	0.18139439	-0.456358005	0.648132559	0.999975616	ENSG00000122218	COPA
4109.968065	-0.031077314	0.144625155	-0.214881803	0.829859472	0.999975616	ENSG00000162368	CMPK1
4103.760198	0.112427107	0.193085816	0.582264973	0.560388215	0.999975616	ENSG00000140391	TSPAN3
4116.287242	0.048002391	0.174258407	0.275466718	0.782957692	0.999975616	ENSG00000180398	MCFD2
4111.273145	0.072586091	0.178972514	0.405571161	0.685057722	0.999975616	ENSG00000182934	SRPRA
4074.055061	0.096655112	0.177796257	0.543628498	0.586697145	0.999975616	ENSG00000071054	MAP4K4
4058.259072	-0.020999216	0.193246609	-0.10866538	0.913467902	0.999975616	ENSG00000172380	GNG12
4060.135434	-0.054642027	0.194250064	-0.281297342	0.778482349	0.999975616	ENSG00000159840	ZYX
4093.069713	-0.320622364	0.295317205	-1.085688062	0.277617024	0.999975616	ENSG00000115415	STAT1
4104.202998	0.496868188	0.237157219	2.095100413	0.036162067	0.816847461	ENSG00000106799	TGFBR1
4032.655201	0.054224491	0.156821815	0.345771351	0.729514557	0.999975616	ENSG00000179010	MRFAP1
4137.524209	0.605201778	0.391577843	1.545546532	0.122214123	0.999975616	ENSG00000170558	CDH2
4043.182878	0.572568372	0.220472826	2.597002008	0.009404137	0.526777378	ENSG00000115306	SPTBN1
4054.094617	-0.316124062	0.243650488	-1.297448921	0.194476769	0.999975616	ENSG00000060237	WNK1
4025.72953	-0.244497646	0.267889579	-0.91268069	0.361410503	0.999975616	ENSG00000091136	LAMB1
4080.339024	0.341766696	0.316426336	1.080082968	0.280105235	0.999975616	ENSG00000163171	CDC42EP3
4020.825305	-0.557802584	0.365373667	-1.526663342	0.126844747	0.999975616	ENSG00000125249	RAP2A
4092.513875	-0.307081893	0.359404104	-0.854419552	0.392872567	0.999975616	ENSG00000116679	IVNS1ABP
3957.172724	-0.178888856	0.258934442	-0.690865436	0.489650109	0.999975616	ENSG00000140264	SERF2
4037.388989	0.227884563	0.167581849	1.359840367	0.173880445	0.999975616	ENSG00000132475	H3F3B
3954.831162	-0.191770272	0.239553928	-0.800530691	0.423403389	0.999975616	ENSG00000163513	TGFBR2
4000.688147	0.406533558	0.180155024	2.256576303	0.024034562	0.734505671	ENSG00000082153	BZW1
3966.839359	0.195943214	0.185347691	1.057165662	0.290435988	0.999975616	ENSG00000143753	DEGS1
3995.558412	0.120773066	0.256819349	0.470264669	0.638165936	0.999975616	ENSG00000152049	KCNE4
4035.279894	0.074730781	0.291849645	0.25605918	0.797905141	0.999975616	ENSG00000113013	HSPA9
3916.0381	0.201014459	0.252433349	0.796307063	0.425853581	0.999975616	ENSG00000175274	TP53I11
3967.469159	0.218749569	0.199131547	1.0985179	0.271978406	0.999975616	ENSG00000179820	MYADM
3962.917156	-0.062363514	0.182279718	-0.342130846	0.732252425	0.999975616	ENSG00000135387	CAPRIN1
3935.432651	0.501222733	0.194569575	2.576059141	0.009993355	0.53208981	ENSG00000187514	PTMA
3995.443968	-0.153907926	0.353114677	-0.435858196	0.662939617	0.999975616	ENSG00000162407	PLPP3
3960.204675	0.961597194	0.292227056	3.290582357	0.000999802	0.251240009	ENSG00000167601	AXL
3947.953849	-0.064562812	0.241012436	-0.267881662	0.78879041	0.999975616	ENSG00000124783	SSR1
3932.114233	0.3611938	0.177139301	2.039038192	0.041446216	0.855344797	ENSG00000213585	VDAC1
3946.660035	-0.185277459	0.19785209	-0.936444284	0.349044487	0.999975616	ENSG00000188846	RPL14
3912.341825	-0.026882931	0.221650422	-0.121285267	0.903465089	0.999975616	ENSG00000168268	NT5DC2
3920.212264	-0.046653829	0.183479459	-0.254272761	0.799284842	0.999975616	ENSG00000071553	ATP6AP1
3940.230513	0.127728532	0.289230945	0.441614339	0.658768306	0.999975616	ENSG00000136205	TNS3
3889.705292	0.050030787	0.197695904	0.253069416	0.800214573	0.999975616	ENSG00000164294	GPX8
3818.009704	0.082301171	0.193974076	0.424289535	0.671354671	0.999975616	ENSG00000129250	KIF1C
3909.139128	-0.415819631	0.227830277	-1.825128937	0.067981574	0.939405498	ENSG00000021762	OSBPL5
3886.397614	0.064548664	0.220082332	0.293293257	0.769298007	0.999975616	ENSG00000131473	ACLY
3864.918563	-0.386812285	0.336047442	-1.151064513	0.249705696	0.999975616	ENSG00000100600	LGMN
3822.955752	0.074575071	0.271263057	0.274917903	0.783379313	0.999975616	ENSG00000119655	NPC2
3845.965732	-0.161596523	0.187514087	-0.861783375	0.388806735	0.999975616	ENSG00000131236	CAP1
3843.420524	0.097374272	0.200224256	0.486326053	0.62673601	0.999975616	ENSG00000115053	NCL
3859.390898	0.056757836	0.15820233	0.358767386	0.719769113	0.999975616	ENSG00000184743	ATL3
3783.560002	-0.293144824	0.309941043	-0.945808341	0.344246351	0.999975616	ENSG00000156976	EIF4A2
3827.414743	0.254909445	0.175727343	1.450596367	0.146892287	0.999975616	ENSG00000197694	SPTAN1
3785.141497	-0.205628874	0.169355042	-1.214188082	0.224675911	0.999975616	ENSG00000085733	CTTN
3805.616258	-0.187625821	0.19075934	-0.983573446	0.325325286	0.999975616	ENSG00000136942	RPL35
3853.068972	0.351140757	0.302049527	1.162527089	0.245021428	0.999975616	ENSG00000104635	SLC39A14
3789.893805	-0.093869962	0.256826929	-0.365498909	0.714739021	0.999975616	ENSG00000049860	HEXB
3813.996673	0.165529844	0.179307969	0.923159439	0.355924123	0.999975616	ENSG00000153187	HNRNPU

3833.153214	0.030085542	0.210791354	0.142726639	0.886506077	0.999975616	ENSG00000134910	STT3A
3788.562664	0.198125471	0.217923286	0.909152369	0.363269703	0.999975616	ENSG00000104331	IMPAD1
3776.241836	0.210482025	0.169065329	1.244974509	0.21314112	0.999975616	ENSG00000133872	SARAF
3759.505338	-0.01068641	0.184858396	-0.057808627	0.953901066	0.999975616	ENSG00000092199	HNRNPC
3803.655567	0.241417102	0.252239898	0.957093242	0.338520194	0.999975616	ENSG00000145431	PDGFC
3733.604579	0.108377119	0.188306774	0.575534893	0.564929599	0.999975616	ENSG00000137409	MTCH1
3740.653994	0.299916782	0.254847825	1.176846542	0.239256762	0.999975616	ENSG00000050405	LIMA1
3723.39952	0.129300021	0.242506214	0.533182301	0.593907392	0.999975616	ENSG00000144746	ARL6IP5
3655.733041	-0.024839746	0.366107318	-0.067848265	0.945906422	0.999975616	ENSG00000135821	GLUL
3726.08981	0.281841032	0.263618179	1.069125937	0.285012926	0.999975616	ENSG00000005884	ITGA3
3761.378574	-0.08456482	0.233042346	-0.362873191	0.716699614	0.999975616	ENSG00000135506	OS9
3763.312215	-0.188486435	0.225945872	-0.834210572	0.404162335	0.999975616	ENSG00000166340	TPP1
3746.747745	-0.078422409	0.200291171	-0.391542014	0.695396639	0.999975616	ENSG00000244038	DDOST
3709.239416	0.029270111	0.248883159	0.117605833	0.906379983	0.999975616	ENSG00000206527	HACD2
3710.200473	-0.010840221	0.245962447	-0.044072668	0.96484648	0.999975616	ENSG00000105438	KDELR1
3713.508811	0.294763383	0.186364348	1.581651142	0.113729232	0.997355609	ENSG00000132341	RAN
3707.839722	0.139657414	0.179820752	0.776647923	0.437366519	0.999975616	ENSG00000106682	EIF4H
3678.034966	-0.082848065	0.159270027	-0.520173613	0.602942575	0.999975616	ENSG00000184432	COPB2
3680.509837	0.095886637	0.167416036	0.572744639	0.566817608	0.999975616	ENSG00000083312	TNPO1
3721.167799	-0.432847868	0.275841772	-1.569188977	0.116603912	0.999975616	ENSG00000120594	PLXDC2
3736.150797	0.215302714	0.258563037	0.832689453	0.405019894	0.999975616	ENSG00000157613	CREB3L1
3669.725566	0.040437487	0.20414294	0.198084181	0.842979201	0.999975616	ENSG00000132507	EIF5A
3653.001539	0.326422047	0.23884279	1.366681599	0.171725128	0.999975616	ENSG00000213719	CLIC1
3658.872386	0.232381756	0.192459143	1.207434221	0.227264963	0.999975616	ENSG00000184840	TMED9
3671.267156	-0.023867918	0.164620708	-0.144987339	0.884720868	0.999975616	ENSG00000067182	TNFRSF1A
3691.954537	0.985031057	0.315083869	3.126250354	0.001770508	0.302395792	ENSG00000026508	CD44
3659.797504	0.167080738	0.183954747	0.908270867	0.363735131	0.999975616	ENSG00000175166	PSMD2
3655.887668	-1.310399115	0.803408267	-1.631050075	0.102879754	0.987682951	ENSG00000143387	CTSK
3630.312389	0.038435168	0.289270458	0.132869318	0.894296733	0.999975616	ENSG00000122566	HNRNPA2B1
3700.492256	-0.050825441	0.278272174	-0.182646509	0.855075389	0.999975616	ENSG00000152377	SPOCK1
3656.666835	0.16468506	0.210199208	0.783471363	0.433350364	0.999975616	ENSG00000069275	NUCKS1
3622.781106	0.3147298	0.20949248	1.50234414	0.133008255	0.999975616	ENSG00000143549	TPM3
3669.611344	-0.042524845	0.17478292	-0.243300918	0.807772293	0.999975616	ENSG00000183853	KIRREL1
3616.512434	-0.571657389	0.277717036	-2.058416714	0.039550149	0.848069004	ENSG00000143819	EPHX1
3623.066325	0.255010223	0.356106428	0.716106769	0.473925424	0.999975616	ENSG00000103888	CEMIP
3601.038357	0.035504026	0.252679399	0.140510173	0.888256915	0.999975616	ENSG00000103855	CD276
3612.818229	-0.271132475	0.20372936	-1.330846349	0.183239574	0.999975616	ENSG00000108679	LGALS3BP
3606.878787	1.11958287	0.41787773	2.679211624	0.007379574	0.502485114	ENSG00000197930	ERO1A
3510.652718	-0.858050498	0.393015326	-2.183249462	0.029017444	0.772694606	ENSG00000137124	ALDH1B1
3611.155661	-0.200549055	0.161840088	-1.239180337	0.215278721	0.999975616	ENSG00000125991	ERGIC3
3613.567889	0.106641856	0.187639617	0.568333371	0.569808623	0.999975616	ENSG00000143870	PDIA6
3583.920799	0.018883191	0.175179633	0.107793299	0.914159658	0.999975616	ENSG00000147140	NONO
3600.627392	-0.0286625	0.207728167	-0.137980804	0.890255588	0.999975616	ENSG00000127603	MACF1
3571.190603	-0.2741526	0.240946466	-1.137815401	0.255197573	0.999975616	ENSG00000129038	LOXL1
3558.380039	0.419260221	0.203312868	2.062143063	0.039194118	0.848069004	ENSG00000131446	MGAT1
3566.185953	0.017944199	0.221541271	0.080997091	0.935444266	0.999975616	ENSG00000156642	NPTN
3545.292653	0.316259717	0.21352887	1.481109867	0.138577299	0.999975616	ENSG00000197226	TBC1D9B
3524.265414	-0.161278318	0.222893144	-0.723567873	0.469331071	0.999975616	ENSG00000071282	LMCD1
3543.023022	-0.050646299	0.174347012	-0.290491349	0.771440368	0.999975616	ENSG00000145741	BTF3
3555.933645	0.465928707	0.215632701	2.160751614	0.030714532	0.788369914	ENSG00000156515	HK1
3616.029193	0.727086308	0.394883975	1.841265672	0.065582636	0.937668618	ENSG00000128591	FLNC
3582.054951	0.506499536	0.271788638	1.863578772	0.062380881	0.936048409	ENSG00000122863	CHST3
3553.310991	-0.291534663	0.222701628	-1.309081868	0.19050662	0.999975616	ENSG00000145592	RPL37
3548.131178	0.145332777	0.225297508	0.645070503	0.518881498	0.999975616	ENSG00000187079	TEAD1
3509.206355	0.067145015	0.175204865	0.383237164	0.701543925	0.999975616	ENSG00000197712	FAM114A1
3497.224749	0.769592826	0.24278111	3.169904062	0.001524893	0.286776293	ENSG00000136068	FLNB
3540.319609	0.117656475	0.176253086	0.667542781	0.504425493	0.999975616	ENSG00000130175	PRKCSH
3530.919174	-0.0013048	0.207917632	-0.006275561	0.99499286	0.999975616	ENSG00000130147	SH3BP4
3597.856007	0.91284082	0.743801356	1.227264259	0.219723284	0.999975616	ENSG00000168209	DDIT4
3532.704784	0.298918669	0.229843633	1.300530561	0.193419189	0.999975616	ENSG00000031698	SARS
3492.988014	-0.357986357	0.196344712	-1.823254383	0.068264872	0.940818254	ENSG00000177156	TALDO1
3476.644159	-0.053691771	0.242078827	-0.221794578	0.824473801	0.999975616	ENSG00000198856	OSTC
3601.726078	0.295189721	0.861170881	0.342777174	0.7317661	0.999975616	ENSG00000139211	AMIGO2
3515.241882	0.232701679	0.193127662	1.204911177	0.228237593	0.999975616	ENSG00000171867	PRNP

3596.118811	0.544081225	0.442015659	1.230909389	0.218356756	0.999975616	ENSG00000074181	NOTCH3		
3413.675751	1.721691181	0.881375855	1.953413145	0.05077066	0.899956016	ENSG00000111674	ENO2		
3463.419198	-0.299421802	0.204042703	-1.467446755	0.142254564	0.999975616	ENSG00000182899	RPL35A		
3446.891673	-0.089601041	0.175836909	-0.509569014	0.610353437	0.999975616	ENSG00000131143	COX4I1		
3436.781657	-0.840788964	0.763340224	-1.101460315	0.270696367	0.999975616	ENSG00000122861	PLAU		
3475.763679	0.407492564	0.282654308	1.441664083	0.149397161	0.999975616	ENSG00000075618	FSCN1		
3439.731065	0.247704335	0.171661054	1.44298505	0.149024683	0.999975616	ENSG00000117868	ESYT2		
3444.74345	-0.188665004	0.189534083	-0.995414658	0.319534633	0.999975616	ENSG00000168610	STAT3		
3430.899239	-0.602596191	0.353628633	-1.704036765	0.088374219	0.977083578	ENSG00000127083	OMD		
3391.682595	0.110176853	0.339437452	0.324586614	0.74549396	0.999975616	ENSG00000164932	CTHRC1		
3378.007278	-0.193219096	0.283079197	-0.682561975	0.494883669	0.999975616	ENSG00000197614	MFAP5		
3394.591747	0.595573102	0.316557871	1.881403552	0.059917044	0.92582322	ENSG00000164096	C4orf3		
3416.977402	0.052855758	0.314605019	0.168006721	0.866577992	0.999975616	ENSG00000112769	LAMA4		
3400.422802	-0.147706113	0.178831033	-0.825953475	0.408830484	0.999975616	ENSG00000114391	RPL24		
3387.245401	0.164850444	0.192404874	0.856789336	0.39156132	0.999975616	ENSG00000093010	COMT		
3353.539376	0.233423992	0.266546103	0.875735903	0.381173622	0.999975616	ENSG00000153904	DDAH1		
3382.095401	0.287566595	0.260254476	1.104943895	0.269183901	0.999975616	ENSG00000147027	TMEM47		
3375.09043	0.047805866	0.195367098	0.244697632	0.806690561	0.999975616	ENSG00000163069	SGCB		
3343.585766	-0.409362202	0.221487137	-1.848243685	0.064567098	0.937668618	ENSG00000176658	MYO1D		
3395.055269	0.058250006	0.184967869	0.314919592	0.752822706	0.999975616	ENSG00000147649	MTDH		
3403.655821	-0.368578739	0.199503349	-1.847481461	0.064677393	0.937668618	ENSG00000136010	ALDH1L2		
3320.000297	0.745611185	0.426280002	1.749111337	0.080271776	0.965624825	ENSG00000130821	SLC6A8		
3339.275726	-0.704753449	0.33555355	-2.100271178	0.035704993	0.809103708	ENSG00000154188	ANGPT1		
3354.372466	0.030698211	0.198770519	0.154440466	0.877262449	0.999975616	ENSG00000079246	XRCC5		
3379.23328	-0.252690024	0.236569112	-1.068144618	0.285455282	0.999975616	ENSG00000151176	PLBD2		
3322.735028	0.040993846	0.177072677	0.231508589	0.816919714	0.999975616	ENSG00000112335	SNX3		
3336.405728	-0.12910461	0.204998206	-0.629784095	0.528835853	0.999975616	ENSG00000057608	GDI2		
3345.780644	-0.002577679	0.201722726	-0.012778326	0.989804648	0.999975616	ENSG00000129083	COPB1		
3257.783722	-1.131137115	0.364616092	-3.10226877	0.001920435	0.302395792	ENSG00000172403	SYNPO2		
3319.120958	0.141921111	0.226829065	0.625674276	0.531528609	0.999975616	ENSG00000105701	FKBP8		
3315.726669	-0.343665071	0.193861082	-1.772738842	0.076271992	0.954810416	ENSG00000174099	MSRB3		
3318.658161	-0.069238433	0.201753366	-0.343183535	0.731460391	0.999975616	ENSG00000081087	OSTM1		
3334.310054	-0.138208377	0.235958511	-0.585731687	0.558055837	0.999975616	ENSG00000078098	FAP		
3331.957778	0.066774679	0.229939089	0.290401599	0.771509021	0.999975616	ENSG00000106397	PLOD3		
3305.325237	0.129241114	0.161436566	0.800569025	0.423381189	0.999975616	ENSG00000039560	RAI14		
3310.287084	0.158255542	0.228609826	0.692251708	0.488779272	0.999975616	ENSG00000133318	RTN3		
3300.898089	-0.277541132	0.214684868	-1.29278386	0.196085824	0.999975616	ENSG00000177600	RPLP2		
3290.627024	-0.02305283	0.179907985	-0.128136779	0.89804073	0.999975616	ENSG00000181789	COPG1		
3254.568255	0.043841624	0.200999725	0.218117831	0.827337305	0.999975616	ENSG00000137575	SDCBP		
3262.092848	-0.245438396	0.188289253	-1.303517818	0.192398037	0.999975616	ENSG00000265808	SEC22B		
3247.853511	0.252099831	0.299648705	0.841317939	0.400169844	0.999975616	ENSG00000116678	LEPR		
3266.467624	-0.068283554	0.194612155	-0.350869935	0.725685927	0.999975616	ENSG00000196531	NACA		
3296.538755	0.42132731	0.235499847	1.789076791	0.073602449	0.947863913	ENSG00000099860	GADD45B		
3307.204895	0.35226343	0.291923586	1.206697395	0.227548703	0.999975616	ENSG00000168140	VASN		
3250.296066	0.077980647	0.211229928	0.369174236	0.711997858	0.999975616	ENSG00000123091	RNF11		
3285.500283	0.086890425	0.178685344	0.486276171	0.626771372	0.999975616	ENSG00000134440	NARS		
3257.992284	-0.298722835	0.19139632	-1.560755375	0.11858148	0.999975616	ENSG00000063046	EIF4B		
3253.94005	-0.343270994	0.173419744	-1.979422798	0.047768424	0.886084716	ENSG00000114942	EEF1B2		
3238.652912	-0.048714802	0.186845273	-0.260722687	0.794306369	0.999975616	ENSG00000110917	MLEC		
3214.205907	-0.189361404	0.245361353	-0.771765406	0.440253384	0.999975616	ENSG00000071242	RPS6KA2		
3240.129786	0.04306628	0.158761648	0.27126375	0.786188185	0.999975616	ENSG00000109332	UBE2D3		
3232.316066	-0.212829283	0.295567566	-0.720069816	0.471482011	0.999975616	ENSG00000134363	FST		
3224.691007	-0.060790688	0.153232286	-0.396722451	0.691572143	0.999975616	ENSG00000116871	MAP7D1		
3223.613028	-0.031069562	0.15340235	-0.202536414	0.839497394	0.999975616	ENSG00000087274	ADD1		
3188.219775	0.117527718	0.177118482	0.663554232	0.506975658	0.999975616	ENSG00000118985	ELL2		
3185.52411	-0.104716467	0.239117752	-0.437928452	0.661438149	0.999975616	ENSG00000128791	TWSG1		
3198.646097	-0.139540464	0.167848468	-0.831347855	0.405777147	0.999975616	ENSG00000065150	IPO5		
3213.520695	-0.064770225	0.182425956	-0.35504939	0.72255259	0.999975616	ENSG00000107581	EIF3A		
3316.534347	0.460156329	0.651933615	0.705833108	0.480291931	0.999975616	ENSG00000154175	ABI3BP		
3220.82689	0.133536205	0.191354743	0.697846329	0.485273302	0.999975616	ENSG00000136628	EPRS		
3188.717301	-0.259566791	0.193357917	-1.342416155	0.1794611	0.999975616	ENSG00000118181	RPS25		
3182.876534	-0.122242263	0.178635125	-0.684312575	0.493777808	0.999975616	ENSG00000115268	RPS15		
3195.914729	0.108469208	0.162645098	0.666907332	0.504831328	0.999975616	ENSG00000122545			7-Sep
3216.830749	0.012951712	0.22024659	0.058805506	0.953107023	0.999975616	ENSG00000196498	NCOR2		

3303.094317	0.003658837	0.386269203	0.009472246	0.992442354	0.999975616	ENSG00000235750	KIAA0040
3158.87045	-0.173126038	0.23332607	-0.74199183	0.458092286	0.999975616	ENSG00000142864	SERBP1
3150.074974	0.111937991	0.173456374	0.645338009	0.518708167	0.999975616	ENSG00000172354	GNB2
3244.611526	0.714680721	0.852339183	0.838493332	0.401753691	0.999975616	ENSG00000163661	PTX3
3186.457927	0.383630339	0.226593999	1.693029564	0.09044984	0.9781243	ENSG00000105329	TGFB1
3191.442178	0.309799739	0.233260927	1.328125303	0.184136716	0.999975616	ENSG00000100664	EIF5
3142.022097	0.028326678	0.18171772	0.155882861	0.876125355	0.999975616	ENSG00000141522	ARHGDIA
3139.004676	0.229605203	0.237123997	0.968291721	0.332898698	0.999975616	ENSG00000049449	RCN1
3149.781879	-0.128986789	0.175893761	-0.733322141	0.463361974	0.999975616	ENSG00000163697	APBB2
3153.997829	0.333255406	0.20188128	1.650749416	0.098789753	0.982968395	ENSG00000150403	TMCO3
3165.329619	-0.044482997	0.199236737	-0.223267044	0.823327675	0.999975616	ENSG00000165678	GHITM
3114.702744	-0.001474702	0.184639893	-0.007986909	0.993627437	0.999975616	ENSG00000105568	PPP2R1A
3152.637019	0.263518718	0.237957995	1.107416954	0.268113699	0.999975616	ENSG00000151414	NEK7
3149.242942	-0.084179885	0.190945058	-0.440859196	0.659314936	0.999975616	ENSG00000114251	WNT5A
3119.666732	0.171734892	0.163490383	1.050428097	0.293521333	0.999975616	ENSG00000116005	PCYOX1
3132.520642	0.375252481	0.199932081	1.876899791	0.06053183	0.92582322	ENSG00000128245	YWHAH
3095.93273	0.189799892	0.240348602	0.789685857	0.429711253	0.999975616	ENSG00000142669	SH3BGRL3
3127.28103	-0.376178531	0.194484855	-1.934230465	0.053084798	0.903530068	ENSG00000102181	CD99L2
3107.105261	-0.160434943	0.226286399	-0.708990658	0.478330274	0.999975616	ENSG00000182197	EXT1
3080.470368	-0.16958411	0.228614955	-0.741789227	0.458215049	0.999975616	ENSG00000160691	SHC1
3116.021407	-0.262821645	0.192959733	-1.362054354	0.173180732	0.999975616	ENSG00000198561	CTNND1
3083.417036	0.016481363	0.200977686	0.082005936	0.934641993	0.999975616	ENSG00000119318	RAD23B
3100.763049	-0.081533672	0.173917749	-0.468805926	0.639208364	0.999975616	ENSG00000150961	SEC24D
3087.718966	-0.265931496	0.285631126	-0.931031223	0.351837413	0.999975616	ENSG00000178695	KCTD12
3041.427714	0.047777388	0.238987527	0.199915821	0.841546417	0.999975616	ENSG00000143774	GUK1
3084.978646	0.064090371	0.189612881	0.338006422	0.735358348	0.999975616	ENSG00000137693	YAP1
3045.349048	-0.715306967	0.309621938	-2.310259316	0.020873802	0.68388746	ENSG00000106333	PCOLCE
3076.670325	-0.04421023	0.166476178	-0.265564901	0.79057432	0.999975616	ENSG00000153113	CAST
3068.573172	-0.165399733	0.186025375	-0.889124579	0.373936131	0.999975616	ENSG00000100380	ST13
3077.511585	0.018315371	0.216244517	0.084697505	0.932501879	0.999975616	ENSG00000183291	SELENOF
3054.723661	0.035528793	0.181840461	0.195384418	0.845092014	0.999975616	ENSG00000088986	DYNLL1
3051.791066	-0.145651341	0.216049585	-0.674157005	0.500211501	0.999975616	ENSG00000060138	YBX3
3070.646426	0.314648741	0.219737358	1.431931029	0.152163561	0.999975616	ENSG00000133657	ATP13A3
3110.888628	0.767989097	0.324979442	2.363192857	0.018118238	0.673513405	ENSG00000134369	NAV1
3076.34853	0.052090418	0.196362633	0.265276632	0.790796365	0.999975616	ENSG00000134970	TMED7
3105.796824	0.296481518	0.247150319	1.19959998	0.230294734	0.999975616	ENSG00000100219	XBP1
3044.706171	0.060865126	0.170353636	0.357286918	0.720877022	0.999975616	ENSG00000196419	XRCC6
3034.781902	0.020675114	0.221460178	0.093358156	0.925619032	0.999975616	ENSG00000184216	IRAK1
3015.395859	-0.071544207	0.226683838	-0.315612298	0.752296802	0.999975616	ENSG00000140319	SRP14
3041.46269	0.310325945	0.208798629	1.486245124	0.137214296	0.999975616	ENSG00000164040	PGRMC2
3057.784749	0.069444317	0.177396376	0.391464122	0.695454203	0.999975616	ENSG00000175582	RAB6A
3080.038905	-0.127324523	0.243555426	-0.522774321	0.601131311	0.999975616	ENSG00000157933	SKI
3052.437855	-0.146106497	0.230686972	-0.633353914	0.526502553	0.999975616	ENSG00000171812	COL8A2
3037.396643	-0.070083533	0.183466188	-0.381996995	0.702463593	0.999975616	ENSG00000138757	G3BP2
3038.364278	0.122692954	0.181811015	0.674837848	0.499778792	0.999975616	ENSG00000006125	AP2B1
3054.128969	0.191094443	0.225419388	0.847728514	0.396589183	0.999975616	ENSG00000126777	KTN1
3008.714688	0.035271851	0.167431833	0.210663946	0.83314951	0.999975616	ENSG00000079332	SAR1A
3055.181098	0.391017257	0.259353082	1.507663812	0.131640592	0.999975616	ENSG00000008710	PKD1
3009.398215	0.098947104	0.171071234	0.578397091	0.562996057	0.999975616	ENSG00000049656	CLPTM1L
3027.365734	0.272271887	0.160956927	1.691582289	0.090725646	0.9781243	ENSG00000110958	PTGES3
3046.907453	0.276645638	0.267146216	1.035558884	0.300407982	0.999975616	ENSG00000166250	CLMP
2995.895558	-0.070834853	0.143178317	-0.494731706	0.620789518	0.999975616	ENSG00000167986	DDB1
2945.681325	0.726393891	0.67285861	1.079563939	0.280336407	0.999975616	ENSG00000119630	PGF
2984.73384	-0.015894031	0.215503554	-0.073752989	0.941206934	0.999975616	ENSG00000102316	MAGED2
2975.149771	-0.353982184	0.217316016	-1.628882172	0.103337965	0.987682951	ENSG00000155254	MARVELD1
2952.736476	-0.139834461	0.220265645	-0.634844627	0.525529754	0.999975616	ENSG00000162458	FBLIM1
2986.858614	-0.095803082	0.17966364	-0.533235785	0.593870373	0.999975616	ENSG00000196141	SPATS2L
2968.145582	-0.313337925	0.181768516	-1.723829473	0.084738632	0.971784007	ENSG00000197894	ADH5
2957.180642	-0.194468679	0.173640928	-1.119947244	0.262736244	0.999975616	ENSG00000089063	TMEM230
2998.455901	0.087625438	0.194441896	0.450651016	0.652241092	0.999975616	ENSG00000112081	SRSF3
2951.582664	0.199785628	0.291915697	0.684394945	0.493725807	0.999975616	ENSG00000152518	ZFP36L2
2962.588321	-0.144742898	0.145702946	-0.993410924	0.32050974	0.999975616	ENSG00000084733	RAB10
2946.209403	0.203020415	0.218172688	0.930549176	0.352086815	0.999975616	ENSG00000152601	MBNL1
2989.218664	1.156226617	0.696428475	1.660223065	0.096869586	0.97912728	ENSG00000119938	PPP1R3C

2948.682563	-0.235485099	0.16617986	-1.417049568	0.156468436	0.999975616	ENSG00000197157	SND1
2953.162616	0.176280805	0.205146577	0.85929196	0.390179459	0.999975616	ENSG00000123131	PRDX4
3027.243309	0.868322165	0.445390825	1.949573533	0.051226971	0.903521275	ENSG00000134107	BHLHE40
2829.375113	1.042775037	0.874396198	1.192565841	0.23303945	0.999975616	ENSG00000165507	DEPP1
2960.035628	0.161077867	0.183356896	0.878493644	0.379675885	0.999975616	ENSG00000125977	EIF2S2
2942.518277	-0.09191966	0.188853193	-0.486725474	0.626452891	0.999975616	ENSG00000178104	PDE4DIP
2937.023049	-0.256120456	0.170384238	-1.503193362	0.132789189	0.999975616	ENSG00000122026	RPL21
2940.400413	-0.015117844	0.193361548	-0.078184334	0.937681423	0.999975616	ENSG00000155660	PDIA4
2909.007893	0.011892229	0.206096519	0.057702233	0.953985815	0.999975616	ENSG00000255302	EID1
2935.70711	-0.337031049	0.196577659	-1.714493143	0.086438195	0.974422538	ENSG00000266412	NCOA4
2939.277239	0.042978249	0.149610307	0.287267966	0.773907149	0.999975616	ENSG00000140943	MBTPS1
2916.47012	0.219836377	0.176387994	1.24632279	0.212645914	0.999975616	ENSG00000074842	MYDGF
2918.940169	0.708336151	0.356507793	1.986874241	0.046936335	0.881360317	ENSG00000135318	NT5E
2933.405504	0.184065833	0.186851314	0.985092523	0.324578626	0.999975616	ENSG00000129625	REEP5
2978.688395	-0.345974238	0.341774901	-1.012286851	0.31140092	0.999975616	ENSG00000248527	MTATP6P1
2932.758728	-0.278874194	0.188316424	-1.48088089	0.138638316	0.999975616	ENSG00000110700	RPS13
2907.968576	-0.088475348	0.250310576	-0.353462285	0.723741898	0.999975616	ENSG00000164713	BRI3
2925.429257	0.14887253	0.175273516	0.849372648	0.395673968	0.999975616	ENSG00000178719	GRINA
2906.33006	0.150729672	0.199521422	0.755456084	0.449975373	0.999975616	ENSG00000183726	TMEM50A
2908.459955	0.201479586	0.170967625	1.178466307	0.238610757	0.999975616	ENSG00000185825	BCAP31
2952.859694	0.036354368	0.391393651	0.092884409	0.925995392	0.999975616	ENSG00000198743	SLC5A3
2917.065031	-0.158362727	0.182379793	-0.8683129	0.385223062	0.999975616	ENSG00000171863	RPS7
2897.745587	-0.066146353	0.216285099	-0.305829449	0.759734505	0.999975616	ENSG00000136810	TXN
2942.709928	0.155163454	0.263728601	0.58834519	0.556300619	0.999975616	ENSG00000168264	IRF2BP2
2894.484086	0.256047173	0.185384792	1.381166004	0.167227925	0.999975616	ENSG00000173402	DAG1
2910.963162	0.096441129	0.192586612	0.500767566	0.616534715	0.999975616	ENSG00000141424	SLC39A6
2893.92135	-0.343180037	0.340913002	-1.006649892	0.314103049	0.999975616	ENSG00000159403	C1R
2959.828846	0.557036063	0.32948727	1.690614822	0.090910392	0.9781243	ENSG00000065911	MTHFD2
2879.539694	0.050021441	0.181241202	0.27599376	0.782552859	0.999975616	ENSG00000124155	PIGT
2865.430641	-0.420030794	0.357372769	-1.175329602	0.239862876	0.999975616	ENSG00000115594	IL1R1
2849.460371	0.012898527	0.196047462	0.065792881	0.947542724	0.999975616	ENSG00000112473	SLC39A7
2815.749693	0.026916218	0.219864705	0.122421732	0.902565028	0.999975616	ENSG00000084207	GSTP1
2838.981808	-0.083489196	0.210491289	-0.396639671	0.691633194	0.999975616	ENSG00000129562	DAD1
2861.083706	0.020057333	0.151539622	0.132357023	0.894701906	0.999975616	ENSG00000119335	SET
2970.520816	-0.314589918	0.893097095	-0.352246043	0.72465375	0.999975616	ENSG00000119927	GPAM
2857.350827	-0.234941301	0.175964537	-1.335162786	0.181823074	0.999975616	ENSG00000205339	IPO7
2834.940011	0.1183629	0.20680846	0.572331035	0.567097728	0.999975616	ENSG00000143641	GALNT2
2842.681773	0.224585994	0.216924594	1.035318265	0.300520302	0.999975616	ENSG00000105971	CAV2
2841.027993	-0.222803993	0.176116866	-1.265091742	0.205838443	0.999975616	ENSG00000177239	MAN1B1
2832.235303	-0.033900853	0.228985072	-0.148048309	0.882304643	0.999975616	ENSG00000101294	HM13
2837.628457	0.133475343	0.187859105	0.710507714	0.477389349	0.999975616	ENSG00000104853	CLPTM1
2785.621033	-0.327864807	0.325521732	-1.007197909	0.313839678	0.999975616	ENSG00000233276	GPX1
2823.948615	0.126352981	0.184162747	0.686094135	0.492653749	0.999975616	ENSG00000008441	NFIX
2844.684616	-0.30233132	0.175113613	-1.726486681	0.084259893	0.971784007	ENSG00000160710	ADAR
2831.643907	0.091204465	0.185517631	0.49162155	0.622986906	0.999975616	ENSG00000124486	USP9X
2811.117814	-0.254862951	0.204844655	-1.244176718	0.21343453	0.999975616	ENSG00000111321	LTBR
2840.40059	-0.016543245	0.191377634	-0.086442939	0.931114314	0.999975616	ENSG00000157637	SLC38A10
2791.794025	0.130600623	0.223762289	0.583657879	0.559450512	0.999975616	ENSG00000104964	AES
2795.242944	0.43852032	0.405273102	1.082036577	0.279236275	0.999975616	ENSG00000123416	TUBA1B
2807.816899	-0.095360956	0.186521094	-0.511260974	0.609168328	0.999975616	ENSG00000166734	CASC4
2816.721907	-0.057113367	0.154809303	-0.36892723	0.712181966	0.999975616	ENSG00000155506	LARP1
2827.370401	0.362494319	0.280665485	1.291552895	0.196512026	0.999975616	ENSG00000006327	TNFRSF12A
2824.807541	-0.185000527	0.181053678	-1.021799332	0.306875886	0.999975616	ENSG00000197858	GPAA1
2791.674373	-0.108299719	0.183522552	-0.590116678	0.555112428	0.999975616	ENSG00000143612	C1orf43
2793.101377	0.331879278	0.176622108	1.879035879	0.060239594	0.92582322	ENSG00000058668	ATP2B4
2817.547187	-0.332731353	0.418308675	-0.795420638	0.426368859	0.999975616	ENSG00000171617	ENC1
2792.37222	-0.125796457	0.215312117	-0.584251637	0.559051025	0.999975616	ENSG00000120438	TCP1
2785.694058	-0.088500312	0.180321312	-0.490792306	0.623573353	0.999975616	ENSG00000113758	DBN1
2759.170481	0.095746048	0.224642941	0.426214362	0.669951653	0.999975616	ENSG00000155366	RHOC
2780.222832	-0.088180403	0.164858117	-0.534886632	0.592728253	0.999975616	ENSG00000163956	LRPAP1
2743.284608	-0.005361959	0.260270787	-0.020601464	0.983563573	0.999975616	ENSG00000100300	TSPO
2801.038823	0.052790838	0.191598934	0.275527828	0.782910749	0.999975616	ENSG00000124422	USP22
2786.095201	0.084870287	0.207931646	0.408164357	0.683153014	0.999975616	ENSG00000075426	FOSL2
2784.992916	-0.079482447	0.154515909	-0.514396526	0.606974788	0.999975616	ENSG00000023734	STRAP

2779.193504	-0.278586164	0.203853315	-1.366601099	0.171750372	0.999975616	ENSG00000111817	DSE	
2808.354141	1.131727776	0.426838765	2.651417512	0.008015469	0.513679125	ENSG00000162433	AK4	
2766.403006	0.423672365	0.244203262	1.734916891	0.082755527	0.970687665	ENSG00000079739	PGM1	
2756.367904	-0.035140144	0.178991525	-0.196322947	0.844357402	0.999975616	ENSG00000116237	ICMT	
2754.492319	-0.283741203	0.18972111	-1.495570014	0.134765742	0.999975616	ENSG00000198918	RPL39	
2735.548578	0.014146934	0.230457401	0.06138633	0.951051539	0.999975616	ENSG00000176871	WSB2	
2758.79996	-0.272934194	0.204574732	-1.334153984	0.182153397	0.999975616	ENSG00000048828	FAM120A	
2777.093315	0.322302698	0.24601914	1.31007164	0.190171601	0.999975616	ENSG00000167978	SRRM2	
2753.983103	0.081485488	0.163302535	0.498984834	0.617790068	0.999975616	ENSG00000184007	PTP4A2	
2737.645305	0.000549254	0.244455609	0.002246844	0.99820728	0.999975616	ENSG00000033100	CHPF2	
2749.074369	0.12394852	0.20322258	0.609915101	0.541918049	0.999975616	ENSG00000124067	SLC12A4	
2726.026167	0.689790472	0.285332905	2.417493602	0.015627809	0.666269005	ENSG00000102007	PLP2	
2752.275889	-0.163688074	0.20372385	-0.803480176	0.421697251	0.999975616	ENSG00000077232	DNAJC10	
2729.030758	0.113274802	0.169540256	0.668129235	0.504051102	0.999975616	ENSG00000113558	SKP1	
2749.371049	-0.070532493	0.191640228	-0.368046386	0.712838647	0.999975616	ENSG00000173517	PEAK1	
2747.567208	0.037946647	0.194020547	0.19558056	0.844938477	0.999975616	ENSG00000177311	ZBTB38	
2727.021333	0.175215132	0.190078322	0.921804916	0.356630343	0.999975616	ENSG00000159335	PTMS	
2724.847388	0.030145899	0.180066929	0.167414968	0.867043548	0.999975616	ENSG00000169714	CNBP	
2743.607155	0.172659892	0.197373026	0.874789711	0.381688337	0.999975616	ENSG00000170248	PDCD6IP	
2713.233989	0.339884978	0.214370683	1.585501211	0.112852502	0.997355609	ENSG00000184640		9-Sep
2709.191424	-0.030609318	0.220397749	-0.13888217	0.88954326	0.999975616	ENSG00000152894	PTPRK	
2739.686151	-0.161677336	0.218320164	-0.740551548	0.458965395	0.999975616	ENSG00000198612	COPS8	
2783.461229	0.342971879	0.438448467	0.782239886	0.434073608	0.999975616	ENSG00000168159	RNF187	
2796.29796	0.095013187	0.355356792	0.267374056	0.789181173	0.999975616	ENSG00000187735	TCEA1	
2699.192231	-0.468748907	0.26905552	-1.74220141	0.081473207	0.968853935	ENSG00000244486	SCARF2	
2719.982	0.71132361	0.367175162	1.937286839	0.05271029	0.903530068	ENSG00000141959	PFKL	
2752.313979	0.289257066	0.245158969	1.179875519	0.238049728	0.999975616	ENSG00000204217	BMPR2	
2711.732638	0.193686164	0.155061857	1.249089673	0.211632277	0.999975616	ENSG00000173726	TOMM20	
2695.895855	-0.231797595	0.193022611	-1.20088312	0.229796543	0.999975616	ENSG00000197728	RPS26	
2699.471612	0.060061354	0.198088478	0.303204685	0.761733879	0.999975616	ENSG00000166272	WBP1L	
2672.429651	-0.252958187	0.218283464	-1.158851808	0.246516596	0.999975616	ENSG00000136436	CALCOCO2	
2696.67617	0.075834651	0.179653547	0.42211608	0.672940291	0.999975616	ENSG00000106609	TMEM248	
2631.506563	0.426603147	0.288785279	1.477233013	0.139613187	0.999975616	ENSG00000067082	KLF6	
2690.852748	0.061018735	0.190474514	0.320351177	0.748702132	0.999975616	ENSG00000153827	TRIP12	
2701.946797	-0.27599548	0.219867698	-1.255279796	0.209377207	0.999975616	ENSG00000168710	AHCYL1	
2695.45262	-0.177437698	0.174044059	-1.019498734	0.307966254	0.999975616	ENSG00000107372	ZFAND5	
2664.905614	0.053616938	0.144283888	0.37160724	0.710185297	0.999975616	ENSG00000197256	KANK2	
2669.353023	0.214016294	0.204062113	1.048780152	0.294279314	0.999975616	ENSG00000157240	FZD1	
2684.209217	0.39303713	0.210035666	1.871287567	0.061305233	0.932616556	ENSG00000113407	TARS	
2627.37903	-0.101981778	0.254083384	-0.401371298	0.688146777	0.999975616	ENSG00000013364	MVP	
2668.566786	-0.274647565	0.208641794	-1.316359299	0.188053476	0.999975616	ENSG00000175390	EIF3F	
2648.206353	-0.024163299	0.176360803	-0.1370106	0.891022416	0.999975616	ENSG00000022840	RNF10	
2687.266483	0.062766805	0.269504716	0.23289687	0.815841489	0.999975616	ENSG00000175215	CTDSP2	
2617.078823	-0.43533758	0.296021529	-1.470628103	0.141391719	0.999975616	ENSG00000189043	NDUFA4	
2696.599276	0.509529169	0.347960561	1.464330232	0.143103741	0.999975616	ENSG00000097007	ABL1	
2651.33429	0.095015586	0.16647477	0.570750666	0.568168669	0.999975616	ENSG00000169223	LMAN2	
2662.368404	-0.473536307	0.175806242	-2.693512485	0.00707035	0.493287504	ENSG00000166963	MAP1A	
2648.601113	0.252518916	0.173076848	1.458998814	0.144565434	0.999975616	ENSG00000011304	PTBP1	
2573.830983	0.365654548	0.36941339	0.989824836	0.322259743	0.999975616	ENSG00000185201	IFITM2	
2657.509798	0.036741886	0.161674539	0.227258329	0.820222874	0.999975616	ENSG00000007168	PAFAH1B1	
2643.311965	0.226982573	0.160823011	1.411381196	0.158132245	0.999975616	ENSG00000067141	NEO1	
2645.759867	0.099603672	0.173966155	0.572546266	0.566951951	0.999975616	ENSG00000008952	SEC62	
2628.947628	-0.756570595	0.292843669	-2.583530651	0.009779478	0.528651689	ENSG00000181019	NQO1	
2655.41475	-0.015436548	0.160193821	-0.096361695	0.923233313	0.999975616	ENSG00000100528	CNIH1	
2608.56583	0.262230875	0.308460507	0.850127874	0.395253996	0.999975616	ENSG00000176046	NUPR1	
2627.908842	0.031652998	0.166570599	0.19002752	0.849287565	0.999975616	ENSG00000128989	ARPP19	
2596.158642	-0.054870157	0.334334609	-0.16411749	0.869638654	0.999975616	ENSG00000107738	VSIR	
2629.570454	0.207574228	0.207111561	1.002233906	0.316230636	0.999975616	ENSG00000129128	SPCS3	
2707.516256	-0.15881634	0.333253551	-0.476563083	0.633673277	0.999975616	ENSG00000144476	ACKR3	
2625.513603	0.103317591	0.17784997	0.580925546	0.561290634	0.999975616	ENSG00000100030	MAPK1	
2630.593344	0.106053105	0.268282606	0.395303692	0.692618775	0.999975616	ENSG00000140564	FURIN	
2611.810499	-0.070611625	0.159835421	-0.441777074	0.65865053	0.999975616	ENSG00000136758	YME1L1	
2617.301749	-0.208580704	0.181756238	-1.147584846	0.251139988	0.999975616	ENSG00000179295	PTPN11	
2558.768328	-0.154204226	0.302166547	-0.510328584	0.60982128	0.999975616	ENSG00000000971	CFH	

2602.689509	0.469566116	0.326498852	1.438186119	0.150381253	0.999975616	ENSG00000122203	KIAA1191
2570.558504	-0.17873888	0.239807546	-0.745343019	0.456064381	0.999975616	ENSG00000122359	ANXA11
2603.268154	0.222446571	0.169103847	1.315443589	0.188360864	0.999975616	ENSG00000106263	EIF3B
2560.108325	-0.28348877	0.272936122	-1.038663435	0.298961292	0.999975616	ENSG00000131378	RFTN1
2643.117554	0.178438282	0.315639445	0.565323138	0.571853994	0.999975616	ENSG00000140105	WARS
2609.430518	-0.235909998	0.229369522	-1.028515016	0.303707631	0.999975616	ENSG00000085662	AKR1B1
2557.424911	0.038548896	0.20785991	0.185456136	0.852871282	0.999975616	ENSG00000134590	RTL8C
2604.092882	0.031102048	0.185108669	0.168020483	0.866567165	0.999975616	ENSG00000198663	C6orf89
2572.959491	-0.11556238	0.207609363	-0.556633761	0.577777682	0.999975616	ENSG00000135624	CCT7
2600.091138	0.408061877	0.241700481	1.688295674	0.091354485	0.9781243	ENSG00000168214	RBPJ
2605.274573	-0.455287714	0.284324079	-1.601298475	0.109310827	0.995962615	ENSG00000177954	RPS27
2576.475743	-0.352879456	0.233913453	-1.508589827	0.131403635	0.999975616	ENSG00000172667	ZMAT3
2589.83554	-0.295568394	0.205120869	-1.440947454	0.149599528	0.999975616	ENSG00000138801	PAPSS1
2558.264511	0.184141732	0.158876963	1.159020969	0.246447638	0.999975616	ENSG00000049245	VAMP3
2597.046272	-0.15260584	0.668229834	-0.228373281	0.819356059	0.999975616	ENSG00000146197	SCUBE3
2595.143949	-0.555727905	0.223529593	-2.486149139	0.012913384	0.622303364	ENSG00000101955	SRPX
2566.939351	-0.02920129	0.155041131	-0.188345438	0.850605869	0.999975616	ENSG00000097033	SH3GLB1
2596.638959	-0.122028386	0.233341567	-0.522960341	0.601001852	0.999975616	ENSG00000103512	NOMO1
2557.571225	1.189294167	0.38311857	3.10424568	0.001907649	0.302395792	ENSG00000116285	ERRFI1
2577.635163	0.111937728	0.182647386	0.612862467	0.539967283	0.999975616	ENSG00000174231	PRPF8
2528.152958	0.463437699	0.248668042	1.863680173	0.062366631	0.936048409	ENSG00000166073	GPR176
2535.554558	-0.135380283	0.228139117	-0.593411096	0.552906065	0.999975616	ENSG00000117592	PRDX6
2545.845164	-0.172015531	0.166409402	-1.033688774	0.301281683	0.999975616	ENSG00000116489	CAPZA1
2546.17356	0.084527645	0.180891134	0.467284623	0.640296257	0.999975616	ENSG00000198961	PJA2
2545.966198	0.757982602	0.272770444	2.778829664	0.005455512	0.474027133	ENSG00000188229	TUBB4B
2545.259062	-0.369223855	0.242611081	-1.521875481	0.128040284	0.999975616	ENSG00000134686	PHC2
2535.445508	-0.04817052	0.190337081	-0.253080062	0.800206345	0.999975616	ENSG00000055208	TAB2
2543.55423	0.076246256	0.192840007	0.395386088	0.692557975	0.999975616	ENSG00000086758	HUWE1
2526.945129	0.055090562	0.180882761	0.304565022	0.760697463	0.999975616	ENSG00000143742	SRP9
2495.078012	0.131629046	0.29839322	0.441126129	0.659121689	0.999975616	ENSG00000134986	NREP
2526.699973	-0.138550716	0.179420543	-0.772212109	0.439988811	0.999975616	ENSG00000155096	AZIN1
2484.082377	0.462328849	0.323812454	1.427767349	0.153358834	0.999975616	ENSG00000134824	FADS2
2530.16784	0.03134573	0.169264937	0.185187381	0.853082067	0.999975616	ENSG00000131507	NDFIP1
2527.929445	-0.401408994	0.200544401	-2.001596616	0.045328133	0.871859801	ENSG00000157483	MYO1E
2532.843949	-0.092610061	0.27594616	-0.335609168	0.73716561	0.999975616	ENSG00000008394	MGST1
2540.47935	0.104372712	0.184921104	0.564417525	0.572470015	0.999975616	ENSG00000108424	KPNB1
2485.541544	-0.240218441	0.268735042	-0.89388581	0.371382991	0.999975616	ENSG00000131171	SH3BGRL
2562.188762	-0.127392554	0.229394177	-0.555343453	0.578659762	0.999975616	ENSG00000151491	EPS8
2543.088562	0.440510218	0.310325912	1.419508333	0.155750869	0.999975616	ENSG00000120889	TNFRSF10B
2550.705651	0.323240968	0.308318902	1.048398153	0.294455204	0.999975616	ENSG00000206190	ATP10A
2499.500338	-0.129172081	0.216631918	-0.596274465	0.550991887	0.999975616	ENSG00000166794	PPIB
2483.668052	-0.008513772	0.258185885	-0.03297536	0.973694237	0.999975616	ENSG00000166557	TMED3
2505.544141	0.092429614	0.175625399	0.52628842	0.598687828	0.999975616	ENSG00000017260	ATP2C1
2497.821383	0.161257339	0.174946611	0.921751713	0.3566581	0.999975616	ENSG00000114554	PLXNA1
2491.492197	0.204630229	0.237239382	0.862547467	0.388386324	0.999975616	ENSG00000185650	ZFP36L1
2474.29206	1.010740922	0.391558998	2.581324724	0.009842196	0.528713791	ENSG00000142949	PTPRF
2417.948921	0.086750528	0.383547765	0.226179205	0.821062044	0.999975616	ENSG00000180447	GAS1
2516.537339	0.096507515	0.206958991	0.466312262	0.640992003	0.999975616	ENSG00000025796	SEC63
2473.433322	-0.008259479	0.16048754	-0.051464923	0.958955052	0.999975616	ENSG00000165476	REEP3
2454.398524	0.23973273	0.169714325	1.412566263	0.157783295	0.999975616	ENSG00000161013	MGAT4B
2450.328128	-0.027869872	0.191647707	-0.145422416	0.884377368	0.999975616	ENSG00000108829	LRRC59
2450.030948	-0.098227569	0.161785538	-0.607146786	0.543753504	0.999975616	ENSG00000120705	ETF1
2447.276156	-0.010940213	0.175558852	-0.062316497	0.950310791	0.999975616	ENSG00000180879	SSR4
2479.221453	0.198894584	0.251798794	0.789894903	0.429589148	0.999975616	ENSG00000038382	TRIO
2477.068159	0.300573732	0.191409961	1.57031395	0.11634209	0.999975616	ENSG00000184014	DENND5A
2486.486246	-0.016759979	0.427601833	-0.039195292	0.968734687	0.999975616	ENSG00000105509	HAS1
2469.23638	0.393258734	0.244143089	1.610771519	0.107229531	0.989669467	ENSG00000166986	MARS
2430.56223	0.535121462	0.249416477	2.145493628	0.0319134	0.794279453	ENSG00000128641	MYO1B
2445.568647	0.009044814	0.168591081	0.053649419	0.957214483	0.999975616	ENSG00000101367	MAPRE1
2445.333835	0.367980228	0.26530692	1.386998226	0.165442304	0.999975616	ENSG00000189403	HMGB1
2474.228781	0.064041545	0.296317022	0.216125096	0.828890238	0.999975616	ENSG00000198380	GFPT1
2420.331006	0.022508353	0.177478112	0.126823264	0.899080283	0.999975616	ENSG00000198898	CAPZA2
2427.41412	-0.129238723	0.178972325	-0.722115687	0.470223354	0.999975616	ENSG00000145391	SETD7
2417.732665	-0.022188887	0.178216578	-0.124505179	0.9009153	0.999975616	ENSG00000176788	BASP1

2359.333235	0.320507649	0.370226369	0.865707244	0.386650727	0.999975616	ENSG00000162616	DNAJB4
2426.625515	-0.160188061	0.169669687	-0.944117148	0.345109787	0.999975616	ENSG00000070214	SLC44A1
2392.95357	0.121453965	0.258254288	0.470288281	0.638149069	0.999975616	ENSG00000168994	PXDC1
2417.148231	-0.088276653	0.171963781	-0.513344453	0.607710394	0.999975616	ENSG00000105323	HNRNPUL1
2449.87308	0.257278666	0.217611858	1.182282381	0.237093674	0.999975616	ENSG00000176915	ANKLE2
2433.225517	-0.336145792	0.240079465	-1.400143875	0.161470239	0.999975616	ENSG00000187134	AKR1C1
2433.827256	-0.065779651	0.26412305	-0.249049264	0.803322674	0.999975616	ENSG00000173482	PTPRM
2421.032626	0.030790931	0.20965188	0.146866945	0.883237043	0.999975616	ENSG00000115806	GORASP2
2413.102016	0.01543448	0.192527048	0.080167855	0.936103756	0.999975616	ENSG00000148358	GPR107
2401.639488	0.225947207	0.175628001	1.286510155	0.198265077	0.999975616	ENSG00000104695	PPP2CB
2429.044819	-0.517679168	0.275024231	-1.882303851	0.059794772	0.92582322	ENSG00000150907	FOXO1
2399.833189	0.063435404	0.156597143	0.405086599	0.685413856	0.999975616	ENSG00000091527	CDV3
2379.304334	-0.032771241	0.21511478	-0.152343049	0.878916373	0.999975616	ENSG00000117450	PRDX1
2406.93134	-0.134387411	0.235247316	-0.571260122	0.567823329	0.999975616	ENSG00000144381	HSPD1
2383.387962	-0.196175456	0.195575122	-1.003069582	0.31582729	0.999975616	ENSG00000070087	PFN2
2388.548027	-0.002282611	0.195484575	-0.011676679	0.99068357	0.999975616	ENSG00000101150	TPD52L2
2395.556187	-0.19572036	0.210768095	-0.928605253	0.353093699	0.999975616	ENSG00000104408	EIF3E
2412.010038	0.104509264	0.232766086	0.448988362	0.653440052	0.999975616	ENSG00000072364	AFF4
2395.449728	0.083479994	0.189159888	0.441319746	0.658981533	0.999975616	ENSG00000131724	IL13RA1
2404.028323	0.541751311	0.323230422	1.676052976	0.093727816	0.9781243	ENSG00000182199	SHMT2
2380.133407	-0.064808923	0.197978681	-0.32735304	0.74340088	0.999975616	ENSG00000186591	UBE2H
2417.362137	0.201349486	0.273127109	0.737200661	0.461000332	0.999975616	ENSG00000149115	TNKS1BP1
2370.621503	-0.048304309	0.15538288	-0.310872788	0.755897332	0.999975616	ENSG00000196961	AP2A1
2363.795898	-0.02142064	0.178341293	-0.120110375	0.904395714	0.999975616	ENSG00000123240	OPTN
2344.936722	0.255916024	0.238325435	1.073809116	0.282908243	0.999975616	ENSG00000235162	C12orf75
2355.158915	0.960800918	0.38169604	2.517188591	0.011829549	0.593932564	ENSG00000176845	METRNL
2377.602325	0.014822214	0.211320899	0.070140787	0.944081604	0.999975616	ENSG00000112531	QKI
2371.492393	1.435179915	0.407463579	3.522228707	0.000427935	0.163806497	ENSG00000197747	S100A10
2356.160887	-0.178637884	0.217223753	-0.822368094	0.410867432	0.999975616	ENSG00000116288	PARK7
2388.165746	0.271251777	0.221320914	1.225603905	0.220347767	0.999975616	ENSG00000139641	ESYT1
2357.766134	0.015619278	0.178431444	0.087536579	0.930245011	0.999975616	ENSG00000106153	CHCHD2
2346.297687	0.07405365	0.209281822	0.353846548	0.723453887	0.999975616	ENSG00000154978	VOPP1
2362.318302	0.056228414	0.245725659	0.228825976	0.819004177	0.999975616	ENSG00000171604	CXXC5
2329.892084	-0.198319234	0.365286326	-0.542914475	0.587188689	0.999975616	ENSG00000100196	KDELR3
2383.634256	0.106956683	0.203373495	0.525912596	0.598948937	0.999975616	ENSG00000112308	C6orf62
2357.672675	-0.359583236	0.256126692	-1.403927222	0.160340523	0.999975616	ENSG00000103502	CDIPT
2349.429672	-0.052452389	0.209116405	-0.250828668	0.801946576	0.999975616	ENSG00000163468	CCT3
2333.159376	0.048998393	0.174962372	0.280051035	0.77943835	0.999975616	ENSG00000131100	ATP6V1E1
2348.320887	-0.157930631	0.215169443	-0.733982618	0.462959332	0.999975616	ENSG00000163931	TKT
2397.174112	-0.184049416	0.325050321	-0.566218225	0.571245442	0.999975616	ENSG00000149428	HYOU1
2358.870637	0.188042307	0.182472586	1.030523605	0.302764277	0.999975616	ENSG00000130726	TRIM28
2354.560739	-0.123397519	0.254828505	-0.484237504	0.628217323	0.999975616	ENSG00000008294	SPAG9
2342.987551	-0.073118502	0.19669481	-0.371735799	0.710089568	0.999975616	ENSG00000084754	HADHA
2343.191137	0.133356544	0.195088196	0.683570544	0.49424639	0.999975616	ENSG00000170266	GLB1
2346.756681	-0.006756081	0.167546643	-0.040323583	0.967835153	0.999975616	ENSG00000134108	ARL8B
2346.732015	0.242296493	0.300328207	0.806772349	0.419797653	0.999975616	ENSG00000140945	CDH13
2318.34653	-0.001936458	0.213535249	-0.009068562	0.992764434	0.999975616	ENSG00000122705	CLTA
2328.193736	-0.114147467	0.168631493	-0.676904797	0.49846636	0.999975616	ENSG00000151135	TMEM263
2328.101973	-0.239948976	0.19968396	-1.201643716	0.229501596	0.999975616	ENSG00000109475	RPL34
2293.025763	-0.813512887	0.670006583	-1.21418641	0.224676549	0.999975616	ENSG00000164125	FAM198B
2309.280244	0.092291286	0.201987199	0.456916509	0.647731057	0.999975616	ENSG00000111328	CDK2AP1
2304.458953	0.264912988	0.176738005	1.498902218	0.133899001	0.999975616	ENSG00000203879	GDI1
2302.350448	-0.095229123	0.184557127	-0.515987245	0.605863318	0.999975616	ENSG00000126524	SBDS
2317.578916	-0.055176897	0.17254045	-0.319791082	0.749126708	0.999975616	ENSG00000162736	NCSTN
2311.25951	-0.037783892	0.213408598	-0.177049528	0.859469486	0.999975616	ENSG00000177628	GBA
2287.662813	0.208645225	0.332791363	0.62695505	0.530688701	0.999975616	ENSG00000174307	PHLDA3
2314.134005	-0.003906312	0.195610709	-0.019969828	0.984067442	0.999975616	ENSG00000145817	YIPF5
2293.770801	-0.239961472	0.196629562	-1.220373323	0.222323386	0.999975616	ENSG00000233927	RPS28
2305.532916	-0.036531102	0.165740228	-0.220411798	0.825550457	0.999975616	ENSG00000065000	AP3D1
2308.895196	-0.073925132	0.181989665	-0.406205113	0.684591898	0.999975616	ENSG00000162434	JAK1
2316.006707	0.157003431	0.2454502	0.639654932	0.522396962	0.999975616	ENSG00000113657	DPYSL3
2276.280455	0.114569959	0.209529925	0.546795206	0.584519437	0.999975616	ENSG00000165915	SLC39A13
2277.835265	-0.23331485	0.199034931	-1.172230666	0.241104459	0.999975616	ENSG00000065154	OAT
2278.429945	0.524982601	0.193852034	2.708161428	0.00676571	0.492649482	ENSG00000101856	PGRMC1

2282.641016	0.017678247	0.236932355	0.074613057	0.940522585	0.999975616	ENSG00000163479	SSR2
2266.827105	0.04915836	0.17550499	0.280096652	0.779403353	0.999975616	ENSG00000159346	ADIPOR1
2263.108546	-0.037577334	0.176838169	-0.212495606	0.831720405	0.999975616	ENSG00000010256	UQCRC1
2263.196103	0.006076673	0.181379466	0.033502544	0.973273837	0.999975616	ENSG00000138107	ACTR1A
2279.6046	-0.047278375	0.241676882	-0.195626385	0.844902607	0.999975616	ENSG00000134851	TMEM165
2222.112462	0.116263205	0.21095159	0.551136897	0.581539834	0.999975616	ENSG00000173641	HSPB7
2294.274202	0.018088009	0.193831431	0.093318247	0.925650737	0.999975616	ENSG00000100201	DDX17
2281.590803	0.133878993	0.212197916	0.630915686	0.528095655	0.999975616	ENSG00000159140	SON
2246.071199	0.120967101	0.212141077	0.570220075	0.568528442	0.999975616	ENSG00000160014	CALM3
2255.394318	-0.121977311	0.180513968	-0.675722284	0.499216985	0.999975616	ENSG00000135916	ITM2C
2253.790383	0.218706585	0.208207974	1.050423674	0.293523366	0.999975616	ENSG00000136295	TTYH3
2245.13357	-0.104495599	0.198082175	-0.527536608	0.597821002	0.999975616	ENSG00000074416	MGLL
2246.777713	-0.233201234	0.213698935	-1.091260626	0.275158218	0.999975616	ENSG00000126934	MAP2K2
2268.788291	0.05521237	0.189960836	0.290651333	0.771317996	0.999975616	ENSG00000118762	PKD2
2235.614473	-0.030587659	0.208202253	-0.146913197	0.883200535	0.999975616	ENSG00000159377	PSMB4
2260.668719	0.378634318	0.201542613	1.878681206	0.060288035	0.92582322	ENSG00000159461	AMFR
2266.617688	0.006252979	0.2350384	0.026604075	0.978775523	0.999975616	ENSG00000171862	PTEN
2252.313155	0.068844101	0.156054651	0.441153791	0.659101664	0.999975616	ENSG00000135018	UBQLN1
2261.301488	-0.044750845	0.203430069	-0.219981465	0.82588559	0.999975616	ENSG00000108582	CPD
2241.159349	-0.159116066	0.267266533	-0.595346018	0.551612201	0.999975616	ENSG00000167468	GPX4
2231.402799	-0.059819308	0.170296852	-0.351264905	0.725389621	0.999975616	ENSG00000129925	TMEM8A
2254.74266	0.18058083	0.177765456	1.015837574	0.30970674	0.999975616	ENSG00000170876	TMEM43
2262.35336	-0.12543975	0.196184882	-0.639395598	0.522565612	0.999975616	ENSG00000106723	SPIN1
2243.257314	0.154190106	0.208643268	0.73901309	0.459899049	0.999975616	ENSG00000142208	AKT1
2205.671857	-0.484946933	0.353324222	-1.372526712	0.169899529	0.999975616	ENSG00000133142	TCEAL4
2216.883857	-0.647481465	0.910860236	-0.71084612	0.477179597	0.999975616	ENSG00000172061	LRRC15
2250.48214	-0.188803281	0.255803486	-0.73807939	0.460466207	0.999975616	ENSG00000174705	SH3PXD2B
2226.643193	0.182781171	0.222362238	0.821997353	0.411078403	0.999975616	ENSG00000180628	PCGF5
2240.748393	-0.171593556	0.173051862	-0.99157301	0.321405861	0.999975616	ENSG00000138768	USO1
2214.646718	-0.237521785	0.230294886	-1.031381063	0.302362158	0.999975616	ENSG00000102804	TSC22D1
2239.430757	-0.081181439	0.188929016	-0.429692805	0.667419118	0.999975616	ENSG00000117122	MFAP2
2291.273428	-0.69814448	0.34439633	-2.027154236	0.042646642	0.855568497	ENSG00000139508	SLC46A3
2269.263098	0.351687816	0.289539838	1.214643961	0.224501914	0.999975616	ENSG00000155304	HSPA13
2269.269502	0.091403405	0.289067476	0.31620093	0.751850002	0.999975616	ENSG00000135269	TES
2241.264505	-0.130354656	0.184881252	-0.705072337	0.480765221	0.999975616	ENSG00000120451	SNX19
2228.337476	-0.219942205	0.165671477	-1.327580394	0.184316765	0.999975616	ENSG00000146535	GNA12
2299.278732	-0.000879682	0.400044619	-0.002198959	0.998245486	0.999975616	ENSG00000162591	MEGF6
2220.305475	0.102244655	0.203617271	0.502141366	0.615568084	0.999975616	ENSG00000148700	ADD3
2208.939668	-0.099014347	0.209766417	-0.472021922	0.636911141	0.999975616	ENSG00000182240	BACE2
2243.009135	-0.119098128	0.203796208	-0.584398157	0.558952466	0.999975616	ENSG00000166479	TMX3
2213.524019	0.03830152	0.190351824	0.201214357	0.840530966	0.999975616	ENSG00000113583	C5orf15
2199.436643	-0.376586397	0.225365844	-1.67100032	0.094721615	0.9781243	ENSG00000103187	COTL1
2209.869746	0.159045599	0.23919264	0.664926808	0.506097308	0.999975616	ENSG00000132155	RAF1
2199.15317	-0.024668311	0.242838971	-0.101583	0.919087673	0.999975616	ENSG00000112078	KCTD20
2233.603425	-0.267392795	0.214915161	-1.244178367	0.213433924	0.999975616	ENSG00000237550	RPL9P9
2214.657754	-0.079806665	0.31727382	-0.251538766	0.801397594	0.999975616	ENSG00000151914	DST
2153.045244	-0.181745934	0.325312994	-0.558680216	0.576379984	0.999975616	ENSG00000120738	EGR1
2210.433519	0.083134685	0.158437103	0.524717276	0.599779745	0.999975616	ENSG00000114354	TFG
2201.07307	-0.123129164	0.183870278	-0.669652353	0.503079432	0.999975616	ENSG00000086289	EPDR1
2188.319981	0.19449309	0.211855374	0.918046525	0.358594509	0.999975616	ENSG00000160209	PDXK
2219.645926	0.030329241	0.18904024	0.160438018	0.872536044	0.999975616	ENSG00000158195	WASF2
2196.067055	0.05346146	0.163703158	0.326575617	0.743988888	0.999975616	ENSG00000089737	DDX24
2216.23808	0.239993164	0.250451933	0.958240413	0.337941543	0.999975616	ENSG00000164741	DLC1
2176.645088	0.370244357	0.151332922	2.446555267	0.014422868	0.6423619	ENSG00000072778	ACADVL
2185.329335	-0.110153362	0.172473483	-0.638668391	0.52303868	0.999975616	ENSG00000169567	HINT1
2199.927189	0.100778125	0.16616006	0.606512328	0.544174598	0.999975616	ENSG00000151239	TWF1
2208.681576	-0.281623583	0.196055936	-1.436445071	0.150875736	0.999975616	ENSG00000177425	PAWR
2169.010616	-0.030258532	0.233674585	-0.129490042	0.896969904	0.999975616	ENSG00000111229	ARPC3
2195.695066	0.020283767	0.21632965	0.09376323	0.925297241	0.999975616	ENSG00000116044	NFE2L2
2182.021844	-0.071106025	0.161022178	-0.441591498	0.658784837	0.999975616	ENSG00000007202	KIAA0100
2195.49111	0.034202235	0.20655621	0.165583183	0.868484982	0.999975616	ENSG00000115524	SF3B1
2179.199031	-0.008704229	0.362808423	-0.023991254	0.980859585	0.999975616	ENSG00000198732	SMOC1
2164.915534	0.254052891	0.277223928	0.916417614	0.3594479	0.999975616	ENSG00000123143	PKN1
2179.287476	-0.003868271	0.150260613	-0.025743745	0.979461732	0.999975616	ENSG00000204469	PRRC2A

2195.492829	-0.01057793	0.228055921	-0.046383054	0.963004943	0.999975616	ENSG00000177733	HNRNPA0
2180.423152	-0.150806657	0.171196674	-0.880897116	0.378373509	0.999975616	ENSG00000174695	TMEM167A
2172.370674	-0.033172169	0.31055816	-0.106814677	0.914936004	0.999975616	ENSG00000022267	FHL1
2159.727018	-0.067956086	0.178290125	-0.381154517	0.703088597	0.999975616	ENSG00000115234	SNX17
2163.517698	0.179971538	0.209999193	0.85701062	0.391439016	0.999975616	ENSG00000137207	YIPF3
2172.494129	0.160863351	0.146248367	1.099932626	0.271361478	0.999975616	ENSG00000101558	VAPA
2135.418822	0.667388391	0.412882798	1.61641123	0.106005432	0.987682951	ENSG00000164442	CITED2
2159.428479	0.046636861	0.194525037	0.239747344	0.81052613	0.999975616	ENSG00000119777	TMEM214
2151.363709	0.052713884	0.186611317	0.282479569	0.77757581	0.999975616	ENSG00000183283	DAZAP2
2176.590455	-0.154560262	0.197436834	-0.782833978	0.433724612	0.999975616	ENSG00000168906	MAT2A
2148.702864	-0.211231061	0.210086968	-1.005445806	0.314682233	0.999975616	ENSG00000129103	SUMF2
2190.765649	0.501077437	0.326523642	1.534582409	0.124886436	0.999975616	ENSG00000139514	SLC7A1
2147.47906	-0.073217775	0.193387991	-0.378605593	0.704980769	0.999975616	ENSG00000164292	RHOBTB3
2146.106015	-0.076845355	0.150836275	-0.509462029	0.610428407	0.999975616	ENSG00000078618	NRDC
2154.742936	0.102778425	0.207993758	0.49414187	0.62120599	0.999975616	ENSG00000137509	PRCP
2156.991959	0.356455402	0.227278209	1.568365936	0.116795756	0.999975616	ENSG00000155876	RRAGA
2180.007655	0.136804962	0.362316247	0.377584399	0.705739356	0.999975616	ENSG00000134030	CTIF
2140.271546	0.467093194	0.256689937	1.819678636	0.068807959	0.941152905	ENSG00000092964	DPYSL2
2154.69776	-0.094116931	0.260373671	-0.361468694	0.717749104	0.999975616	ENSG00000109929	SC5D
2154.448848	-0.228214899	0.173988978	-1.311662969	0.189633876	0.999975616	ENSG00000135956	TMEM127
2165.578122	0.047568666	0.180954922	0.262875777	0.792646327	0.999975616	ENSG00000137710	RDX
2140.813105	0.294105379	0.225882231	1.302029726	0.192906224	0.999975616	ENSG00000138944	SHISAL1
2119.725482	0.209669646	0.365748086	0.573262455	0.566467003	0.999975616	ENSG00000197381	ADARB1
2151.981572	0.101501994	0.227575017	0.44601554	0.655586021	0.999975616	ENSG00000074696	HACD3
2140.93556	-0.045113589	0.292521858	-0.154222966	0.877433935	0.999975616	ENSG00000048392	RRM2B
2118.513741	-0.057123996	0.262472504	-0.217638021	0.827711158	0.999975616	ENSG00000122870	BICC1
2116.61354	0.621591543	0.395327652	1.572345218	0.115870512	0.999975616	ENSG00000106538	RARRES2
2159.626885	0.151190497	0.284178713	0.532026117	0.594707907	0.999975616	ENSG00000168056	LTBP3
2160.934264	0.750314505	0.408724183	1.835747761	0.066394971	0.937668618	ENSG00000133639	BTG1
2134.046605	-0.055479924	0.25925798	-0.213995047	0.83055092	0.999975616	ENSG00000062716	VMP1
2119.920807	-0.040314713	0.269072921	-0.149828206	0.880900156	0.999975616	ENSG00000072163	LIMS2
2124.675528	0.21666523	0.220210634	0.983899941	0.325164713	0.999975616	ENSG00000134684	YARS
2156.16694	0.120999241	0.264483566	0.457492473	0.647317111	0.999975616	ENSG00000182158	CREB3L2
2096.698801	0.170877814	0.254645513	0.67104192	0.502193825	0.999975616	ENSG00000110811	P3H3
2107.687939	-0.071141738	0.22964462	-0.309790572	0.756720221	0.999975616	ENSG00000156261	CCT8
2146.945315	-0.33370724	0.231030707	-1.444428078	0.148618597	0.999975616	ENSG00000132294	EFR3A
2123.851273	-0.01979389	0.242265328	-0.081703354	0.934882611	0.999975616	ENSG00000100241	SBF1
2104.012925	-0.090619587	0.182429828	-0.496736682	0.619374751	0.999975616	ENSG00000146701	MDH2
2124.154616	-0.194000289	0.204898627	-0.946811074	0.343735058	0.999975616	ENSG00000120742	SERP1
2143.115909	0.18347914	0.340513601	0.538830576	0.590003764	0.999975616	ENSG00000150630	VEGFC
2104.761441	0.297339915	0.177940437	1.671008115	0.094720075	0.9781243	ENSG00000164754	RAD21
2097.296388	0.216809962	0.194182838	1.116524838	0.264197544	0.999975616	ENSG00000177706	FAM20C
2112.392011	0.092935003	0.188488277	0.493054554	0.621974041	0.999975616	ENSG00000103342	GSPT1
2133.578445	0.487162636	0.321117114	1.517087113	0.129244692	0.999975616	ENSG00000198053	SIRPA
2072.076965	0.305791284	0.290315789	1.053305731	0.292200893	0.999975616	ENSG00000188042	ARL4C
2097.777057	-0.045047331	0.157684401	-0.285680323	0.775122977	0.999975616	ENSG00000084623	EIF3I
2109.853973	-0.251114974	0.151165472	-1.661192673	0.096674757	0.9781243	ENSG00000196914	ARHGEF12
2104.914074	-0.157738864	0.187356709	-0.841917352	0.399834217	0.999975616	ENSG00000198242	RPL23A
2091.819396	0.601884282	0.417282296	1.442391129	0.149192065	0.999975616	ENSG00000167553	TUBA1C
2096.879464	0.523703786	0.274618679	1.907021719	0.056517775	0.917136198	ENSG00000090861	AARS
2094.70653	0.003606499	0.16342433	0.022068309	0.982393466	0.999975616	ENSG00000129636	ITFG1
2089.786749	-0.37602971	0.197645946	-1.902541983	0.057100336	0.917136198	ENSG00000143162	CREG1
2111.617502	-0.318674177	0.275172696	-1.158087929	0.246828155	0.999975616	ENSG00000171298	GAA
2084.111465	-0.12719049	0.163066704	-0.779990561	0.435396431	0.999975616	ENSG00000147677	EIF3H
2107.463871	0.216129353	0.177134084	1.220145488	0.222409727	0.999975616	ENSG00000162512	SDC3
2073.116797	-0.056484727	0.19800682	-0.285266572	0.775439921	0.999975616	ENSG00000110906	KCTD10
2076.933359	0.212382064	0.192204013	1.104982463	0.269167188	0.999975616	ENSG00000143321	HDGF
2089.542608	0.236095642	0.237973697	0.992108142	0.321144775	0.999975616	ENSG00000169715	MT1E
2079.890555	-0.310678238	0.234904223	-1.322574082	0.185977058	0.999975616	ENSG00000145012	LPP
2072.187037	0.382835362	0.215345837	1.777769971	0.075441642	0.953148956	ENSG00000117298	ECE1
2093.82409	0.482007681	0.28890356	1.668403397	0.095235674	0.9781243	ENSG00000144320	LNPK
2084.010365	-0.034585899	0.205299084	-0.168465921	0.866216751	0.999975616	ENSG00000167323	STIM1
2081.79776	0.253979338	0.20979202	1.210624397	0.22603939	0.999975616	ENSG00000169180	XPO6
2081.275395	0.54350538	0.229701226	2.366140529	0.01797462	0.673513405	ENSG00000145860	RNF145

2080.029435	0.120262105	0.161593529	0.744225991	0.456739766	0.999975616	ENSG00000115216	NRBP1
2062.441185	-0.007457703	0.169037399	-0.044118658	0.96480982	0.999975616	ENSG00000204463	BAG6
2047.226863	-0.497829667	0.269787603	-1.845265169	0.064998975	0.937668618	ENSG00000162614	NEXN
2085.261383	0.386089608	0.260085497	1.484471886	0.137683776	0.999975616	ENSG00000124831	LRRFIP1
2081.397635	0.060286758	0.201495372	0.299196738	0.764789938	0.999975616	ENSG00000152102	FAM168B
2072.888717	-0.101318683	0.158043218	-0.641082133	0.521469322	0.999975616	ENSG00000090060	PAPOLA
2095.141727	0.062159979	0.266874224	0.232918632	0.81582459	0.999975616	ENSG00000197555	SIPA1L1
2059.062717	-0.283516828	0.226356704	-1.252522339	0.210379594	0.999975616	ENSG00000165757	JCAD
2059.727352	0.050169846	0.163141193	0.307524085	0.758444493	0.999975616	ENSG00000119396	RAB14
2067.743472	-0.045947439	0.162458875	-0.28282505	0.777310951	0.999975616	ENSG00000107863	ARHGAP21
2075.567227	-0.024038672	0.211437388	-0.11369168	0.909482208	0.999975616	ENSG00000013288	MAN2B2
2056.425374	0.139368508	0.153816502	0.906069936	0.364898837	0.999975616	ENSG00000165637	VDAC2
2156.1189	0.76415672	0.992516133	0.769918689	0.441348127	0.999975616	ENSG00000101255	TRIB3
2023.68644	0.129483855	0.22777233	0.568479302	0.569709556	0.999975616	ENSG00000198832	SELENOM
2068.570388	-0.092093206	0.195798407	-0.470347065	0.638107077	0.999975616	ENSG00000144724	PTPRG
2050.039106	-0.127412254	0.23902905	-0.533040876	0.594005285	0.999975616	ENSG00000068912	ERLEC1
2049.551389	-0.140184187	0.222879007	-0.628969903	0.52936876	0.999975616	ENSG00000146731	CCT6A
2034.771783	-0.223820708	0.216383254	-1.034371671	0.300962443	0.999975616	ENSG00000148484	RSU1
2044.214745	-0.072435023	0.172783658	-0.419223806	0.675052575	0.999975616	ENSG00000113384	GOLPH3
2039.068539	0.011891446	0.183863234	0.064675497	0.948432373	0.999975616	ENSG00000147526	TACC1
2044.998649	-0.065146735	0.190229764	-0.34246342	0.732002168	0.999975616	ENSG00000012660	ELOVL5
2044.851825	-0.241420713	0.172591819	-1.398795806	0.161874224	0.999975616	ENSG00000090621	PABPC4
2080.383793	-0.249752571	0.4253414	-0.587181428	0.557081864	0.999975616	ENSG00000101665	SMAD7
2019.933811	-0.072824107	0.199652668	-0.364753989	0.715295053	0.999975616	ENSG00000185088	RPS27L
2025.915208	0.029095508	0.16900263	0.172160091	0.863311672	0.999975616	ENSG00000014216	CAPN1
1989.752465	0.192162452	0.373291281	0.51477884	0.606707574	0.999975616	ENSG00000204381	LAYN
2024.111995	0.108365026	0.220679103	0.491052503	0.623389314	0.999975616	ENSG00000117411	B4GALT2
2049.722391	-0.051511569	0.189490215	-0.271842898	0.785742819	0.999975616	ENSG00000225733	FGD5-AS1
2039.597237	-0.197575447	0.209478591	-0.943177277	0.345590234	0.999975616	ENSG00000154122	ANKH
2021.46844	-0.058409063	0.226820567	-0.257512197	0.796783401	0.999975616	ENSG00000153071	DAB2
2037.65801	0.08370344	0.160097598	0.52282758	0.601094245	0.999975616	ENSG00000168066	SF1
2026.913171	0.016103632	0.17664492	0.091163853	0.927362396	0.999975616	ENSG00000143418	CERS2
2038.758964	0.221388284	0.203483015	1.087993927	0.276597789	0.999975616	ENSG00000173457	PPP1R14B
2009.723178	0.110672191	0.173832984	0.636658183	0.524347518	0.999975616	ENSG00000204843	DCTN1
2019.683925	-0.094069096	0.161345006	-0.583030729	0.559872614	0.999975616	ENSG00000188554	NBR1
2024.727033	0.072211332	0.198525934	0.363737523	0.716054021	0.999975616	ENSG00000095787	WAC
2029.516388	0.030223401	0.166326403	0.181711385	0.855809231	0.999975616	ENSG00000087365	SF3B2
2013.85209	-0.068683511	0.16613749	-0.41341368	0.679303554	0.999975616	ENSG00000135720	DYNC1LI2
1993.919106	0.016100791	0.247634317	0.065018417	0.948159337	0.999975616	ENSG00000231991	ANXA2P2
1998.539242	0.159171965	0.166671095	0.955006418	0.339574452	0.999975616	ENSG00000157593	SLC35B2
2001.067561	-0.211411134	0.26730884	-0.790887178	0.429009832	0.999975616	ENSG00000167291	TBC1D16
2020.861623	0.105808172	0.164621913	0.642734433	0.520396434	0.999975616	ENSG00000144224	UBXN4
1999.674654	0.047851513	0.192104449	0.249091125	0.803290294	0.999975616	ENSG00000184900	SUMO3
2009.876889	-0.058606087	0.289865579	-0.202183672	0.839773138	0.999975616	ENSG00000135047	CTSL
1998.774167	0.032824423	0.174500116	0.188105448	0.85079399	0.999975616	ENSG00000106636	YKT6
2038.891653	0.438120107	0.339262283	1.291390551	0.196568285	0.999975616	ENSG00000196365	LONP1
1999.591052	0.157636647	0.175255938	0.899465369	0.368404834	0.999975616	ENSG00000105993	DNAJB6
1983.330916	0.006768457	0.192530873	0.03515518	0.971956002	0.999975616	ENSG00000165410	CFL2
2006.66662	-0.01454785	0.204270968	-0.071218393	0.943223943	0.999975616	ENSG00000059573	ALDH18A1
2005.973198	0.034305194	0.202821164	0.169140111	0.865686436	0.999975616	ENSG00000135316	SYNCRIP
2011.398163	0.084656868	0.187328667	0.451916244	0.651329322	0.999975616	ENSG00000111530	CAND1
1997.24528	0.139927437	0.211193133	0.662556755	0.507614475	0.999975616	ENSG00000134153	EMC7
1977.628508	0.01290906	0.201292623	0.064130816	0.948866065	0.999975616	ENSG00000170004	CHD3
2021.257479	-0.458567569	0.305966789	-1.498749492	0.133938632	0.999975616	ENSG00000136960	ENPP2
2052.644929	0.228042027	0.433126542	0.526502084	0.598539404	0.999975616	ENSG00000171793	CTPS1
1958.421292	-1.155158829	0.307840661	-3.752456952	0.00017511	0.101985805	ENSG00000132386	SERPINF1
1973.906602	0.002162663	0.189637398	0.011404202	0.99090096	0.999975616	ENSG00000157020	SEC13
2007.479292	0.179968161	0.223559511	0.805012322	0.420812569	0.999975616	ENSG00000165527	ARF6
1979.674349	0.048369141	0.186538431	0.259298528	0.795404915	0.999975616	ENSG00000119729	RHOQ
1995.474863	-0.029646587	0.161509914	-0.183558933	0.854359483	0.999975616	ENSG00000099246	RAB18
1998.946847	0.018783208	0.21000917	0.089439944	0.92873228	0.999975616	ENSG00000081154	PCNP
1994.136874	0.243446968	0.270043678	0.901509599	0.367317433	0.999975616	ENSG00000057019	DCBLD2
1993.184996	-0.017171443	0.155114598	-0.110701654	0.911852934	0.999975616	ENSG00000188643	S100A16
1980.938952	-0.121054625	0.19333078	-0.626152882	0.531214669	0.999975616	ENSG00000170606	HSPA4

1988.466251	-0.195824714	0.172684563	-1.134002426	0.25679353	0.999975616	ENSG00000091039	OSBPL8
1975.229992	0.004058407	0.169181589	0.023988468	0.980861807	0.999975616	ENSG00000018408	WWTR1
2004.119746	0.461003614	0.283416678	1.626593101	0.103823546	0.987682951	ENSG00000183688	RFLNB
1970.179343	0.003511621	0.23137434	0.015177227	0.98789079	0.999975616	ENSG00000118855	MFSD1
2017.620251	0.109557393	0.319440295	0.342966729	0.73162349	0.999975616	ENSG00000136603	SKIL
1974.31308	-0.073031345	0.21836004	-0.3344538	0.738037148	0.999975616	ENSG00000123983	ACSL3
1965.398537	-0.106574579	0.153656312	-0.693590631	0.487938971	0.999975616	ENSG00000009335	UBE3C
1942.097757	-0.017730273	0.331082183	-0.053552484	0.957291714	0.999975616	ENSG00000173511	VEGFB
1964.04355	-0.199849825	0.15611915	-1.280110893	0.200506138	0.999975616	ENSG00000153250	RBMS1
1958.614841	0.480064593	0.235487349	2.038600355	0.04148993	0.855344797	ENSG00000149131	SERPING1
1958.312811	0.112903104	0.222550868	0.5073137	0.611934728	0.999975616	ENSG00000159202	UBE2Z
1963.013736	0.004698238	0.176621879	0.026600544	0.978778339	0.999975616	ENSG00000148730	EIF4EBP2
1945.348535	0.01126056	0.276591682	0.040711852	0.967525613	0.999975616	ENSG00000119138	KLF9
1946.579786	-0.049221015	0.190588447	-0.258258124	0.796207703	0.999975616	ENSG00000164054	SHISA5
1995.271577	-0.302091383	0.215176458	-1.403923954	0.160341496	0.999975616	ENSG00000084636	COL16A1
1951.951051	0.17145284	0.175577137	0.976510061	0.328811765	0.999975616	ENSG00000113575	PPP2CA
1939.397424	0.9598534	0.401252232	2.392144702	0.016750237	0.670428193	ENSG00000159399	HK2
1938.721016	-0.25521628	0.244012472	-1.045914902	0.295600324	0.999975616	ENSG00000014641	MDH1
1923.255109	0.048207947	0.222642153	0.216526594	0.828577298	0.999975616	ENSG00000254999	BRK1
1969.671887	0.046744943	0.234332322	0.199481415	0.84188618	0.999975616	ENSG00000127463	EMC1
1933.270137	-0.064326009	0.215984056	-0.297827583	0.765834761	0.999975616	ENSG00000141696	P3H4
1933.758343	-0.279483106	0.184827665	-1.512128104	0.130501277	0.999975616	ENSG00000147416	ATP6V1B2
1936.414057	0.089182622	0.156619054	0.569423833	0.569068547	0.999975616	ENSG00000110696	C11orf58
1939.315531	0.053703257	0.168754956	0.318232176	0.750308828	0.999975616	ENSG00000100320	RBFOX2
1931.760577	-0.349385661	0.299816212	-1.165332783	0.243884314	0.999975616	ENSG00000005022	SLC25A5
1950.638578	-0.054587456	0.213498696	-0.255680513	0.798197543	0.999975616	ENSG00000102580	DNAJC3
1944.418239	0.615328408	0.259075428	2.375093669	0.017544497	0.670428193	ENSG00000105976	MET
1955.300477	0.185642559	0.265958494	0.698013276	0.485168892	0.999975616	ENSG00000140526	ABHD2
1925.633308	0.010606695	0.217035558	0.048870773	0.961022281	0.999975616	ENSG00000117335	CD46
1926.966884	-0.197494559	0.184132576	-1.072567189	0.283465353	0.999975616	ENSG00000158186	MRAS
1937.955517	-0.309193417	0.180035419	-1.717403264	0.085905516	0.973741696	ENSG00000027697	IFNGR1
1926.403014	0.050181235	0.179386498	0.279738083	0.779678458	0.999975616	ENSG00000165102	HGSNAT
1918.523556	-0.611622577	0.245163617	-2.494752624	0.012604497	0.613462161	ENSG00000184371	CSF1
1925.814107	0.420584798	0.325131046	1.29358547	0.195808644	0.999975616	ENSG00000074590	NUAK1
1934.482894	0.116913216	0.25686491	0.455154487	0.648998101	0.999975616	ENSG00000141458	NPC1
1921.311804	-0.059064697	0.176365913	-0.334898599	0.737701579	0.999975616	ENSG00000136802	LRRC8A
1909.168067	-0.097311013	0.215296561	-0.451985917	0.651279128	0.999975616	ENSG00000198911	SREBF2
1923.610049	0.16986024	0.189314553	0.897238153	0.369591851	0.999975616	ENSG00000056097	ZFR
1895.832526	0.257872353	0.334567588	0.770763104	0.440847361	0.999975616	ENSG00000138623	SEMA7A
1909.630121	-0.283937799	0.189191007	-1.500799657	0.133407387	0.999975616	ENSG00000283041	AC008038.1
1896.022021	0.123039961	0.190335749	0.646436423	0.517996758	0.999975616	ENSG00000198792	TMEM184B
1916.735399	0.033611001	0.24409159	0.137698317	0.89047885	0.999975616	ENSG00000107331	ABCA2
1882.953994	0.214853165	0.175235522	1.226082265	0.220167719	0.999975616	ENSG00000107130	NCS1
1902.936014	0.14506195	0.16171303	0.897033158	0.369701224	0.999975616	ENSG00000145907	G3BP1
1891.249849	-0.368345727	0.204654155	-1.799844848	0.07188514	0.945224913	ENSG00000084090	STARD7
1899.594442	-0.059285435	0.155745187	-0.38065661	0.70345807	0.999975616	ENSG00000129515	SNX6
1891.045133	-0.186644989	0.216690065	-0.861345388	0.389047844	0.999975616	ENSG00000116459	ATP5PB
1900.797195	0.137546264	0.167934144	0.819048832	0.412758553	0.999975616	ENSG00000069329	VPS35
1885.726093	0.283545585	0.237001329	1.196388161	0.231545119	0.999975616	ENSG00000104332	SFRP1
1904.455485	-0.114457957	0.171487424	-0.667442277	0.504489669	0.999975616	ENSG00000169710	FASN
1893.849499	-0.044036267	0.245878142	-0.179097932	0.857860806	0.999975616	ENSG00000101474	APMAP
1927.924984	-0.16326508	0.272297721	-0.599582983	0.548784191	0.999975616	ENSG00000144136	SLC20A1
1892.708738	0.045855225	0.202357184	0.226605373	0.820730614	0.999975616	ENSG00000148175	STOM
1897.966461	0.020784413	0.167841903	0.123833278	0.901447282	0.999975616	ENSG00000162923	WDR26
1887.504798	-0.127788471	0.214253748	-0.596435174	0.550884548	0.999975616	ENSG00000161202	DVL3
1885.044493	0.128927537	0.181942587	0.7086166	0.478562432	0.999975616	ENSG00000119801	YPEL5
1916.221315	0.886698739	0.322684352	2.747882673	0.005998147	0.483939657	ENSG00000092621	PHGDH
1900.084578	-0.208886735	0.273463028	-0.763857316	0.444952276	0.999975616	ENSG00000064042	LIMCH1
1896.288028	0.754703711	0.286312545	2.635943564	0.008390368	0.5166643	ENSG00000170540	ARL6IP1
1887.366499	0.154534193	0.184577339	0.837232752	0.402461751	0.999975616	ENSG00000140612	SEC11A
1888.284888	-0.541487661	0.278662976	-1.943163276	0.051996434	0.903530068	ENSG00000151632	AKR1C2
1894.829517	0.379308818	0.281802946	1.346007287	0.178300167	0.999975616	ENSG00000130779	CLIP1
1842.289674	0.07360035	0.328112309	0.224314506	0.82251259	0.999975616	ENSG00000131389	SLC6A6
1850.089705	-0.073733649	0.23888483	-0.308657728	0.757581903	0.999975616	ENSG00000089220	PEBP1

1869.589204	-0.173184448	0.197090497	-0.87870522	0.379561128	0.999975616	ENSG00000178952	TUFM	
1869.386944	-0.327329245	0.204819357	-1.598136276	0.110012652	0.995962615	ENSG00000076716	GPC4	
1885.354524	0.10114956	0.177487093	0.569898116	0.568746803	0.999975616	ENSG00000127947	PTPN12	
1873.235988	0.039168784	0.159053366	0.246261896	0.805479504	0.999975616	ENSG00000070367	EXOC5	
1865.786869	0.052947964	0.173225007	0.305660049	0.759863495	0.999975616	ENSG00000124214	STAU1	
1868.816211	0.168447879	0.210724973	0.799373117	0.424074094	0.999975616	ENSG00000169564	PCBP1	
1873.583336	-0.38021576	0.214200773	-1.775043829	0.075890651	0.954564195	ENSG00000151726	ACSL1	
1834.295575	0.691492526	0.25601808	2.700951931	0.006914133	0.492649482	ENSG00000170961	HAS2	
1924.626088	-0.103548962	0.689750445	-0.150125255	0.880665795	0.999975616	ENSG00000171223	JUNB	
1869.670288	0.178837043	0.184432151	0.969663051	0.332214474	0.999975616	ENSG00000065135	GNAI3	
1872.745909	0.188360393	0.16240609	1.159811143	0.246125706	0.999975616	ENSG00000132463	GRSF1	
1888.241306	-0.388962799	0.225099237	-1.727961431	0.083995139	0.971784007	ENSG00000163297	ANTXR2	
1866.86617	-0.036164113	0.198437258	-0.182244572	0.855390795	0.999975616	ENSG00000166226	CCT2	
1863.554651	0.006995593	0.16448074	0.042531379	0.966075098	0.999975616	ENSG00000129351	ILF3	
1871.279056	-0.699111014	0.309679522	-2.257530655	0.023974936	0.734505671	ENSG00000210140	MT-TC	
1849.624674	-0.074138276	0.186927143	-0.396615895	0.691650729	0.999975616	ENSG00000167193	CRK	
1859.15273	0.053692148	0.194671374	0.27580916	0.782694648	0.999975616	ENSG00000143183	TMCO1	
1876.045255	-0.314069432	0.192632953	-1.630403456	0.103016255	0.987682951	ENSG00000128283	CDC42EP1	
1833.676127	-1.308553035	0.406886015	-3.216018709	0.001299824	0.274639877	ENSG00000162551	ALPL	
1836.43055	-1.051672616	0.43786422	-2.401823598	0.016313573	0.66646844	ENSG00000120549	KIAA1217	
1856.932114	-0.094700728	0.170133047	-0.556627473	0.577781979	0.999975616	ENSG00000182446	NPLOC4	
1849.867475	0.10636139	0.216951034	0.490255281	0.623953267	0.999975616	ENSG00000182087	TMEM259	
1852.673572	0.015967015	0.272872529	0.058514557	0.953338768	0.999975616	ENSG00000172216	CEBPB	
1865.016053	-0.050193799	0.188566907	-0.266185619	0.790096258	0.999975616	ENSG00000090054	SPTLC1	
1858.923927	0.007779926	0.163720163	0.047519659	0.962099063	0.999975616	ENSG00000102753	KPNA3	
1872.738609	0.457926054	0.342760595	1.33599387	0.181551277	0.999975616	ENSG00000091542	ALKBH5	
1837.367922	-0.031513156	0.231489799	-0.136131941	0.891716977	0.999975616	ENSG00000155324	GRAMD2B	
1875.034365	0.876999785	0.452124927	1.939728897	0.052412646	0.903530068	ENSG00000135766	EGLN1	
1859.299555	-0.297415118	0.180918792	-1.643915015	0.100193758	0.986706896	ENSG00000163531	NFASC	
1869.575565	-0.085374983	0.328388069	-0.259981988	0.794877668	0.999975616	ENSG00000124766	SOX4	
1845.684297	-0.052872394	0.217388852	-0.243215756	0.807838262	0.999975616	ENSG00000115484	CCT4	
1838.743727	-0.088616444	0.166875584	-0.531033016	0.5953959	0.999975616	ENSG00000111540	RAB5B	
1856.928408	0.786489826	0.367934923	2.137578624	0.032550958	0.794279453	ENSG00000105825	TFPI2	
1847.916768	0.322538297	0.215616173	1.495891024	0.134682056	0.999975616	ENSG00000100242	SUN2	
1808.039739	0.44681123	0.211439864	2.113183488	0.034585066	0.803388455	ENSG00000162337	LRP5	
1849.417865	-0.045346936	0.168795162	-0.268650687	0.788198503	0.999975616	ENSG00000106052	TAX1BP1	
1875.757787	0.113485587	0.298938721	0.379628261	0.704221379	0.999975616	ENSG00000151468	CCDC3	
1840.587387	0.050970285	0.175032344	0.291204951	0.770894576	0.999975616	ENSG00000162889	MAPKAPK2	
1849.419379	0.16112687	0.231683941	0.695459813	0.486767188	0.999975616	ENSG00000145495		6-Mar
1818.808357	-0.051896735	0.227215967	-0.228402676	0.819333209	0.999975616	ENSG00000142168	SOD1	
1834.302482	0.150421204	0.190802538	0.788360603	0.430485809	0.999975616	ENSG00000168092	PAFAH1B2	
1815.748453	1.056550976	0.431358552	2.449356737	0.014311163	0.6423619	ENSG00000156466	GDF6	
1823.667894	0.023448677	0.191383532	0.122521918	0.902485689	0.999975616	ENSG00000157916	RER1	
1822.694146	-0.100599336	0.187552137	-0.536380646	0.591695504	0.999975616	ENSG00000115762	PLEKHB2	
1840.429976	-0.009180928	0.21504104	-0.042693842	0.965945589	0.999975616	ENSG00000146433	TMEM181	
1822.11389	0.090311696	0.161702406	0.558505578	0.576499197	0.999975616	ENSG00000273749	CYFIP1	
1837.092433	-0.255883407	0.264388419	-0.967831374	0.333128592	0.999975616	ENSG00000175061	LRRC75A-AS1	
1822.817669	-0.001749174	0.158646404	-0.011025613	0.991203012	0.999975616	ENSG00000141905	NFIC	
1820.322029	-0.258713453	0.19891227	-1.300640997	0.193381367	0.999975616	ENSG00000042753	AP2S1	
1819.411028	-0.193937903	0.227957511	-0.850763381	0.394900807	0.999975616	ENSG00000149806	FAU	
1827.431256	0.006162069	0.197117213	0.03126094	0.975061441	0.999975616	ENSG00000162430	SELENON	
1822.019405	-0.113992433	0.179439929	-0.635267933	0.525253684	0.999975616	ENSG00000075151	EIF4G3	
1797.782119	0.195590226	0.245560904	0.796503934	0.42573919	0.999975616	ENSG00000149485	FADS1	
1819.500066	0.168919044	0.254420405	0.663936702	0.506730825	0.999975616	ENSG00000125912	NCLN	
1830.505585	-0.029543663	0.175632096	-0.168213348	0.866415441	0.999975616	ENSG00000156599	ZDHHC5	
1845.096565	0.318175243	0.365674155	0.870105907	0.38424253	0.999975616	ENSG00000198853	RUSC2	
1816.098305	-0.0112649	0.199049841	-0.056593364	0.954869121	0.999975616	ENSG00000077380	DYNC1I2	
1811.764947	-0.220490228	0.173671126	-1.269584839	0.204232553	0.999975616	ENSG00000165672	PRDX3	
1817.641432	-0.019941516	0.199842595	-0.099786114	0.920514133	0.999975616	ENSG00000172260	NEGR1	
1812.438037	-0.020331911	0.226395331	-0.089807111	0.928440497	0.999975616	ENSG00000176978	DPP7	
1861.259284	-0.180730932	0.310313709	-0.582413626	0.560288106	0.999975616	ENSG00000166750	SLFN5	
1823.022278	-0.067643323	0.215642791	-0.313682285	0.753762357	0.999975616	ENSG00000169926	KLF13	
1816.757813	-0.016523977	0.173907355	-0.095015974	0.924302139	0.999975616	ENSG00000163320	CGGBP1	
1806.318679	-0.139376206	0.173145612	-0.804965278	0.420839717	0.999975616	ENSG00000171858	RPS21	

1803.797599	-0.080200724	0.208008712	-0.385564253	0.699819412	0.999975616	ENSG00000173692	PSMD1
1813.581094	-0.338314736	0.194520854	-1.739220907	0.081995913	0.969369989	ENSG00000122042	UBL3
1778.906505	-0.094648008	0.312945062	-0.302442888	0.762314465	0.999975616	ENSG00000116133	DHCR24
1800.649647	-0.047543876	0.16620211	-0.286060603	0.774831706	0.999975616	ENSG00000132963	POMP
1803.200027	-0.837271862	0.744561168	-1.124517229	0.260793666	0.999975616	ENSG00000101825	MXRA5
1801.294218	-0.059630702	0.174288951	-0.34213702	0.732247779	0.999975616	ENSG00000086061	DNAJA1
1807.915895	-0.045940324	0.163342151	-0.281252106	0.778517042	0.999975616	ENSG00000127946	HIP1
1790.562282	-0.24908195	0.192014144	-1.297206261	0.194560227	0.999975616	ENSG00000110237	ARHGEF17
1793.975529	-0.035771063	0.161484907	-0.221513351	0.824692742	0.999975616	ENSG00000099341	PSMD8
1776.891319	-0.091099912	0.258672774	-0.352182065	0.724701726	0.999975616	ENSG00000168528	SERINC2
1810.203081	0.075077822	0.224808188	0.333963913	0.73840679	0.999975616	ENSG00000198752	CDC42BPB
1774.753832	-0.114192445	0.245613249	-0.46492787	0.641983112	0.999975616	ENSG00000198682	PAPSS2
1777.660929	0.551076873	0.244259246	2.256114693	0.024063449	0.734505671	ENSG00000142227	EMP3
1801.704921	0.041701339	0.198309025	0.210284625	0.833445534	0.999975616	ENSG00000138600	SPPL2A
1787.504171	0.089428755	0.164478166	0.543712014	0.586639664	0.999975616	ENSG00000177731	FLII
1756.658243	-0.482232981	0.356523858	-1.352596666	0.176184518	0.999975616	ENSG00000167641	PPP1R14A
1794.053323	0.030668572	0.166771881	0.183895338	0.854095562	0.999975616	ENSG00000084112	SSH1
1797.816846	0.173053836	0.189107358	0.915108947	0.360134438	0.999975616	ENSG00000144028	SNRNP200
1766.60465	-0.002950984	0.276586607	-0.010669295	0.991487295	0.999975616	ENSG00000105698	USF2
1787.047104	0.125059502	0.175039029	0.714466383	0.474938836	0.999975616	ENSG00000100353	EIF3D
1788.380946	-0.124643405	0.210475754	-0.592198403	0.553717738	0.999975616	ENSG00000161671	EMC10
1762.102007	-0.219014331	0.268939888	-0.814361648	0.415437803	0.999975616	ENSG00000116171	SCP2
1799.570146	0.281476126	0.241071503	1.16760431	0.242966411	0.999975616	ENSG00000116685	KIAA2013
1787.527874	-0.05007017	0.149667524	-0.33454265	0.737970113	0.999975616	ENSG00000149547	EI24
1799.979755	-0.045837392	0.229032139	-0.200135196	0.841374848	0.999975616	ENSG00000100994	PYGB
1803.859214	0.356088309	0.290956546	1.22385392	0.22100734	0.999975616	ENSG00000127481	UBR4
1791.950914	0.429042659	0.254092037	1.688532488	0.091309058	0.9781243	ENSG00000177119	ANO6
1797.02699	0.954115747	0.423391646	2.253506314	0.024227243	0.734754698	ENSG00000173530	TNFRSF10D
1812.550176	0.786894523	0.354787392	2.217932601	0.026559428	0.763004428	ENSG00000155380	SLC16A1
1775.076108	0.35585529	0.250974091	1.41789652	0.156220979	0.999975616	ENSG00000130382	MLLT1
1819.399419	-0.162862118	0.277922962	-0.585997347	0.557877298	0.999975616	ENSG00000020181	ADGRA2
1788.002674	0.015852919	0.175584331	0.090286638	0.928059438	0.999975616	ENSG00000121892	PDS5A
1766.303192	0.105503631	0.205783554	0.512692239	0.608166619	0.999975616	ENSG00000156052	GNAQ
1773.305084	0.09802537	0.208061731	0.47113599	0.637543625	0.999975616	ENSG00000066322	ELOVL1
1807.242846	-0.217927611	0.233521234	-0.933223963	0.350704345	0.999975616	ENSG00000047932	GOPC
1794.169415	0.41355697	0.253303358	1.632654904	0.102541597	0.987682951	ENSG00000117139	KDM5B
1770.242619	-0.148858387	0.197830974	-0.75245238	0.451779057	0.999975616	ENSG00000154556	SORBS2
1765.672883	0.210300511	0.183345715	1.147016234	0.251374911	0.999975616	ENSG00000010404	IDS
1752.884306	0.172136556	0.217852251	0.790152757	0.429438562	0.999975616	ENSG00000203485	INF2
1799.182614	-0.211244569	0.347728505	-0.607498568	0.543520092	0.999975616	ENSG00000165617	DACT1
1750.895109	0.15575067	0.215283036	0.723469312	0.469391601	0.999975616	ENSG00000124795	DEK
1768.168088	-0.094090756	0.177892388	-0.52891952	0.59686128	0.999975616	ENSG00000114978	MOB1A
1760.362812	-0.240265618	0.20464924	-1.174036212	0.240380522	0.999975616	ENSG00000130741	EIF2S3
1779.368841	0.426913143	0.220368855	1.93726624	0.052712807	0.903530068	ENSG00000198408	OGA
1766.198085	-0.081701376	0.160746622	-0.508261855	0.611269717	0.999975616	ENSG00000177830	CHID1
1751.578863	-0.132199518	0.210520492	-0.627965085	0.530026813	0.999975616	ENSG00000159069	FBXW5
1759.462456	0.10574899	0.183743859	0.575523942	0.564937003	0.999975616	ENSG00000107223	EDF1
1762.299623	0.163047937	0.172065236	0.94759372	0.343336324	0.999975616	ENSG00000106615	RHEB
1761.718784	-0.24976612	0.182516115	-1.368460642	0.171167933	0.999975616	ENSG00000177565	TBL1XR1
1771.290107	0.203322824	0.18675826	1.08869522	0.27628831	0.999975616	ENSG00000067704	IARS2
1770.877568	-0.017869657	0.193845524	-0.092185036	0.926551027	0.999975616	ENSG00000186111	PIP5K1C
1759.984973	-0.1897894	0.195929789	-0.968660259	0.332714727	0.999975616	ENSG00000084073	ZMPSTE24
1738.713227	0.079171418	0.222938902	0.355126077	0.722495142	0.999975616	ENSG00000140682	TGFB1I1
1753.469731	-0.349746096	0.300531365	-1.163759052	0.244521671	0.999975616	ENSG00000196937	FAM3C
1762.160765	0.048187445	0.176072263	0.273679934	0.7843306	0.999975616	ENSG00000119537	KDSR
1783.714053	-0.02189504	0.293258986	-0.074661106	0.940484354	0.999975616	ENSG00000159216	RUNX1
1742.977379	0.013737458	0.23741451	0.057862756	0.953857949	0.999975616	ENSG00000167671	UBXN6
1737.797252	-0.047663084	0.191541744	-0.248839145	0.80348521	0.999975616	ENSG00000103769	RAB11A
1752.287406	-0.208379433	0.173179017	-1.203260285	0.228875613	0.999975616	ENSG00000257923	CUX1
1790.72427	0.26666917	0.347067384	0.768349844	0.442279369	0.999975616	ENSG00000103489	XYLT1
1761.09534	-0.043877151	0.225825196	-0.19429697	0.845943358	0.999975616	ENSG00000104723	TUSC3
1755.395265	-0.038575305	0.184995163	-0.208520615	0.834822488	0.999975616	ENSG00000125534	PPDPF
1758.831172	0.403625071	0.21959548	1.838039067	0.066056649	0.937668618	ENSG00000131873	CHSY1
1733.476247	-0.088406583	0.260321356	-0.339605573	0.73415358	0.999975616	ENSG00000158470	B4GALT5

1729.045279	-0.056681102	0.189927055	-0.298436163	0.765370292	0.999975616	ENSG00000101182	PSMA7
1731.920215	-0.0632328	0.237967215	-0.265720637	0.790454368	0.999975616	ENSG00000231925	TAPBP
1736.789684	0.125610861	0.207197639	0.606236931	0.544357432	0.999975616	ENSG00000171067	C11orf24
1765.307191	-0.317664382	0.248731931	-1.277135512	0.201554409	0.999975616	ENSG00000031081	ARHGAP31
1749.281346	-0.056838111	0.206716532	-0.274956773	0.783349449	0.999975616	ENSG00000133706	LARS
1738.953246	0.105506632	0.225696651	0.467470971	0.640162956	0.999975616	ENSG00000082898	XPO1
1746.662327	-0.189379059	0.191427559	-0.989298823	0.32251696	0.999975616	ENSG00000169756	LIMS1
1733.065746	0.082819673	0.182003048	0.455045528	0.649076485	0.999975616	ENSG00000180370	PAK2
1742.881311	-0.024354824	0.184406362	-0.132071496	0.894927741	0.999975616	ENSG00000136813	ECPAS
1732.543464	-0.119329302	0.219538574	-0.543545948	0.586753964	0.999975616	ENSG00000133104	SPART
1738.606374	-0.023836712	0.278492533	-0.085591924	0.931790819	0.999975616	ENSG00000156639	ZFAND3
1738.597806	0.048108002	0.255841857	0.188038043	0.850846829	0.999975616	ENSG00000171206	TRIM8
1724.49459	-0.483708634	0.234545677	-2.062321675	0.039177121	0.848069004	ENSG00000175220	ARHGAP1
1721.013061	-0.072023915	0.168432359	-0.427613288	0.66893269	0.999975616	ENSG00000153786	ZDHHC7
1715.27071	0.033711128	0.180343987	0.186926822	0.851718009	0.999975616	ENSG00000089693	MLF2
1716.198288	0.116827469	0.190187959	0.614273743	0.53903445	0.999975616	ENSG00000165458	INPPL1
1736.307892	0.26214407	0.280900572	0.933227256	0.350702645	0.999975616	ENSG00000160007	ARHGAP35
1728.508867	-0.598904385	0.294774431	-2.031737908	0.042180196	0.855568497	ENSG00000157570	TSPAN18
1711.966214	-0.189827597	0.187933632	-1.010077839	0.312457999	0.999975616	ENSG00000112079	STK38
1728.050282	0.567232418	0.288375121	1.966994986	0.049183789	0.891920616	ENSG00000115902	SLC1A4
1747.010586	-0.168865616	0.263833081	-0.640047166	0.522141936	0.999975616	ENSG00000260916	CCPG1
1707.835251	-0.035726562	0.168404302	-0.212147559	0.831991918	0.999975616	ENSG00000169813	HNRNPF
1685.465559	-0.218724867	0.654673726	-0.334097518	0.738305973	0.999975616	ENSG00000136542	GALNT5
1715.584386	-0.231914724	0.183958631	-1.26068955	0.20742072	0.999975616	ENSG00000198677	TTC37
1702.879964	0.060673637	0.216652446	0.280050551	0.779438722	0.999975616	ENSG00000136193	SCRN1
1677.115457	-0.036841193	0.269395923	-0.136754829	0.89122459	0.999975616	ENSG00000178980	SELENOW
1702.931669	0.763799919	0.255844877	2.985402435	0.002832055	0.374056652	ENSG00000164032	H2AFZ
1697.223322	-0.190865244	0.212890726	-0.896540904	0.369963944	0.999975616	ENSG00000115504	EHBP1
1718.717713	0.105676447	0.219011175	0.482516229	0.629439272	0.999975616	ENSG00000115963	RND3
1734.396026	0.213554974	0.269599993	0.792117875	0.428291948	0.999975616	ENSG00000091490	SEL1L3
1700.035273	0.068712238	0.187329501	0.366798813	0.713769093	0.999975616	ENSG00000115694	STK25
1688.012391	-0.228110687	0.19668321	-1.159787287	0.246135422	0.999975616	ENSG00000113328	CCNG1
1712.067444	0.072100426	0.182479172	0.39511592	0.692757341	0.999975616	ENSG00000112851	ERBIN
1695.960949	-0.067969673	0.262849857	-0.258587446	0.795953571	0.999975616	ENSG00000170144	HNRNPA3
1695.616439	0.015157648	0.168648108	0.089877365	0.928384668	0.999975616	ENSG00000143569	UBAP2L
1705.54271	0.271220776	0.194722582	1.392857336	0.163662936	0.999975616	ENSG00000008282	SYPL1
1684.943179	0.099792554	0.256913071	0.388429259	0.697698395	0.999975616	ENSG00000102317	RBM3
1696.148774	-0.00061769	0.193398328	-0.003193875	0.997451661	0.999975616	ENSG00000117906	RCN2
1701.891224	0.165433599	0.222584827	0.743238435	0.457337338	0.999975616	ENSG00000088812	ATRN
1690.440906	-0.186828503	0.171006409	-1.092523395	0.274603113	0.999975616	ENSG00000129187	DCTD
1681.368627	-0.198040728	0.212346926	-0.932628182	0.351011977	0.999975616	ENSG00000163041	H3F3A
1704.685105	0.077742408	0.225335494	0.345007378	0.730088824	0.999975616	ENSG00000109180	OCIAD1
1696.715731	0.257480198	0.324500028	0.793467415	0.427505547	0.999975616	ENSG00000185950	IRS2
1684.776339	-0.058830365	0.189236174	-0.31088329	0.755889348	0.999975616	ENSG00000111843	TMEM14C
1681.254083	0.014819006	0.18209987	0.081378455	0.935140983	0.999975616	ENSG00000125817	CENPB
1679.088118	0.290798573	0.181118643	1.605569521	0.108368527	0.993926078	ENSG00000113648	H2AFY
1682.349794	-0.454907092	0.203733215	-2.232856785	0.02555839	0.752780145	ENSG00000140740	UQCRC2
1692.876014	0.261423118	0.202881626	1.288549994	0.197554578	0.999975616	ENSG00000100258	LMF2
1672.930936	-0.13754173	0.222011268	-0.619525896	0.535569968	0.999975616	ENSG00000138029	HADHB
1681.838493	-0.258979607	0.238112346	-1.087636198	0.276755744	0.999975616	ENSG00000186468	RPS23
1697.382553	0.076756438	0.240642658	0.318964388	0.749753518	0.999975616	ENSG00000173905	GOLIM4
1690.335696	-0.053932802	0.249773619	-0.215926735	0.829044857	0.999975616	ENSG00000116560	SFPQ
1684.585813	-0.265462391	0.192218976	-1.381041543	0.167266188	0.999975616	ENSG00000130255	RPL36
1703.583267	0.102644104	0.286793798	0.357902107	0.720416574	0.999975616	ENSG00000054598	FOXC1
1688.798928	0.03177146	0.207499297	0.153115991	0.878306806	0.999975616	ENSG00000108100	CCNY
1685.333873	0.079010852	0.186086201	0.424592754	0.671133576	0.999975616	ENSG00000135829	DHX9
1674.229499	1.3577388	0.680634359	1.994813781	0.046063194	0.876872329	ENSG00000017483	SLC38A5
1665.70193	-0.098346217	0.222274925	-0.442453043	0.658161403	0.999975616	ENSG00000137312	FLOT1
1665.877908	-0.080296157	0.191289684	-0.419762087	0.674659264	0.999975616	ENSG00000160075	SSU72
1682.564394	-0.017818567	0.175023835	-0.101806514	0.918910255	0.999975616	ENSG00000164091	WDR82
1724.936311	-0.140458819	0.882805145	-0.15910512	0.873586054	0.999975616	ENSG00000100292	HMOX1
1675.513292	-0.586747763	0.22515977	-2.6059174	0.009162856	0.526777378	ENSG00000213949	ITGA1
1684.668123	0.13984615	0.225176924	0.621050098	0.534566662	0.999975616	ENSG00000101337	TM9SF4
1700.196842	0.089190887	0.349118758	0.255474347	0.798356754	0.999975616	ENSG00000151718	WWC2

1660.57172	0.329228738	0.272299737	1.209067408	0.226636949	0.999975616	ENSG00000145901	TNIP1
1667.49179	0.03934464	0.178656263	0.220225362	0.825695645	0.999975616	ENSG00000124767	GLO1
1662.501243	-0.140289871	0.282717586	-0.496219118	0.619739824	0.999975616	ENSG00000105953	OGDH
1654.216477	0.150303849	0.179083319	0.839295639	0.40130343	0.999975616	ENSG00000136279	DBNL
1627.818063	0.218264493	0.41636146	0.524218771	0.600126387	0.999975616	ENSG00000186480	INSIG1
1664.732164	-0.250810597	0.222848573	-1.125475446	0.260387616	0.999975616	ENSG00000091317	CMTM6
1660.963665	-0.163250667	0.192151278	-0.849594487	0.395550578	0.999975616	ENSG00000158290	CUL4B
1652.863333	0.120067989	0.184369547	0.651235475	0.514894494	0.999975616	ENSG00000188612	SUMO2
1668.311082	0.160413631	0.176887775	0.906866692	0.364477297	0.999975616	ENSG00000152104	PTPN14
1657.172104	0.044871091	0.172691235	0.259834214	0.794991658	0.999975616	ENSG00000175203	DCTN2
1675.589163	0.089336379	0.202963169	0.440160544	0.659820834	0.999975616	ENSG00000113712	CSNK1A1
1655.267429	1.289799145	0.422696453	3.051360227	0.002278071	0.337641006	ENSG00000059804	SLC2A3
1682.988852	-0.483184502	0.310738663	-1.554954566	0.11995689	0.999975616	ENSG00000169902	TPST1
1649.838766	0.094756443	0.17781509	0.532893148	0.594107549	0.999975616	ENSG00000166888	STAT6
1672.005396	-0.043791904	0.198051784	-0.221113406	0.825004132	0.999975616	ENSG00000092148	HECTD1
1653.919502	-0.018772604	0.161945076	-0.11591957	0.907716286	0.999975616	ENSG00000078304	PPP2R5C
1652.286921	-0.157586068	0.197638366	-0.79734553	0.425250384	0.999975616	ENSG00000079950	STX7
1667.908297	0.056859427	0.242752136	0.234228328	0.814807724	0.999975616	ENSG00000204256	BRD2
1654.006855	-0.116613699	0.212363903	-0.549122037	0.582921703	0.999975616	ENSG00000052802	MSMO1
1665.861786	0.540792893	0.305345534	1.771084993	0.076546569	0.954810416	ENSG00000120254	MTHFD1L
1647.126993	0.056676499	0.170255287	0.332891272	0.739216358	0.999975616	ENSG00000168439	STIP1
1660.019928	-0.03841705	0.245651802	-0.156388227	0.875727017	0.999975616	ENSG00000111711	GOLT1B
1686.244638	-0.228910645	0.311822484	-0.734105641	0.462884356	0.999975616	ENSG00000225630	MTND2P28
1658.415809	-0.005359912	0.206103748	-0.026005893	0.979252638	0.999975616	ENSG00000127483	HP1BP3
1652.937216	-0.034830899	0.189048054	-0.184243628	0.853822335	0.999975616	ENSG00000186432	KPNA4
1636.163245	-0.004600243	0.165732976	-0.027756955	0.977855997	0.999975616	ENSG00000177885	GRB2
1640.995544	-0.015994923	0.209926769	-0.076192869	0.939265656	0.999975616	ENSG00000198843	SELENOT
1652.236882	-0.15892651	0.174459119	-0.910967058	0.362312731	0.999975616	ENSG00000110367	DDX6
1661.904091	-0.12743659	0.24590358	-0.518238043	0.604292195	0.999975616	ENSG00000131016	AKAP12
1653.552363	0.216719306	0.283237018	0.765151773	0.444181174	0.999975616	ENSG00000127526	SLC35E1
1630.234109	-0.288845639	0.246117361	-1.173609365	0.240551529	0.999975616	ENSG00000154518	ATP5MC3
1643.724043	0.060240142	0.182283531	0.330474957	0.741041112	0.999975616	ENSG00000150753	CCT5
1633.69824	-0.292456478	0.246622872	-1.185844911	0.235683552	0.999975616	ENSG00000102401	ARMCX3
1638.582733	0.313374315	0.16187259	1.935931933	0.052876038	0.903530068	ENSG00000124570	SERPINB6
1643.593757	0.164367833	0.160583498	1.023566156	0.306040238	0.999975616	ENSG00000132128	LRRC41
1666.994556	-0.201985439	0.266376592	-0.758270229	0.448289226	0.999975616	ENSG00000162849	KIF26B
1638.784348	0.129917699	0.182141807	0.713277755	0.4756739	0.999975616	ENSG00000133687	TMTC1
1659.936377	-0.032698465	0.287721265	-0.113646325	0.909518163	0.999975616	ENSG00000245532	NEAT1
1620.446397	-0.12638634	0.212048163	-0.596026574	0.551157474	0.999975616	ENSG00000123349	PFDN5
1636.412554	0.134482372	0.214908675	0.625765207	0.531468956	0.999975616	ENSG00000101152	DNAJC5
1660.255198	0.744378286	0.279103304	2.667035019	0.00765237	0.507415783	ENSG00000117143	UAP1
1638.903302	0.055043172	0.187960624	0.292844166	0.769641268	0.999975616	ENSG00000175115	PACS1
1646.035552	0.247589513	0.268164528	0.923274661	0.355864089	0.999975616	ENSG00000183723	CMTM4
1619.933318	0.568176536	0.246838469	2.301815186	0.021345597	0.686650924	ENSG00000124145	SDC4
1635.673527	-0.137680875	0.168998654	-0.814686223	0.415251942	0.999975616	ENSG00000119487	MAPKAP1
1642.37278	0.243793377	0.178506678	1.365738131	0.172021171	0.999975616	ENSG00000108588	CCDC47
1620.044048	0.024291633	0.193702657	0.125406816	0.900201493	0.999975616	ENSG00000008018	PSMB1
1674.547499	0.171538523	0.389903543	0.439951179	0.659972467	0.999975616	ENSG00000090020	SLC9A1
1659.409653	-0.068435609	0.310133982	-0.220664657	0.825353553	0.999975616	ENSG00000104368	PLAT
1626.281374	-0.114767785	0.172663758	-0.664689488	0.506249119	0.999975616	ENSG00000116688	MFN2
1649.804445	-0.38147391	0.208780193	-1.82715565	0.06767637	0.939405498	ENSG00000166482	MFAP4
1629.008225	0.191657765	0.291211852	0.658138613	0.510449066	0.999975616	ENSG00000221968	FADS3
1647.398555	0.223006803	0.242527999	0.919509515	0.357829131	0.999975616	ENSG00000136868	SLC31A1
1617.044128	-0.064359155	0.157104005	-0.409659544	0.682055711	0.999975616	ENSG00000159720	ATP6V0D1
1632.781033	0.141430526	0.186656797	0.757703592	0.448628448	0.999975616	ENSG00000109111	SUPT6H
1616.621314	-0.194362696	0.188641781	-1.03032687	0.30285659	0.999975616	ENSG00000128829	EIF2AK4
1628.70032	-0.141096446	0.215162896	-0.655765693	0.511974893	0.999975616	ENSG00000182095	TNRC18
1581.34021	1.692600439	0.419202737	4.037665522	5.40E-05	0.069509109	ENSG00000148926	ADM
1642.707502	-0.219551425	0.212398024	-1.033679222	0.30128615	0.999975616	ENSG00000123066	MED13L
1603.48335	0.210066434	0.423695752	0.495795469	0.620038722	0.999975616	ENSG00000161544	CYGB
1636.978735	0.010401347	0.233543295	0.044537126	0.964476259	0.999975616	ENSG00000019144	PHLDB1
1618.941441	0.04849975	0.159670755	0.303748483	0.761319518	0.999975616	ENSG00000086232	EIF2AK1
1616.482135	-0.181325859	0.167702014	-1.081238409	0.279591077	0.999975616	ENSG00000111676	ATN1
1607.347591	-0.386247935	0.296990899	-1.300537951	0.193416658	0.999975616	ENSG00000168938	PPIC

1631.521549	-0.131578703	0.286619983	-0.459070233	0.646183732	0.999975616	ENSG00000132613	MTSS1L
1621.606005	-0.533470014	0.397842169	-1.340908672	0.179950106	0.999975616	ENSG00000116132	PRRX1
1593.020189	-0.398300334	0.311905466	-1.276990556	0.201605581	0.999975616	ENSG00000116717	GADD45A
1629.4265	0.32373591	0.446429876	0.725166319	0.468350003	0.999975616	ENSG00000102359	SRPX2
1603.859023	-0.096222228	0.194747374	-0.494087423	0.62124444	0.999975616	ENSG00000114416	FXR1
1612.754017	0.191660093	0.203376912	0.942388646	0.345993698	0.999975616	ENSG00000144567	RETREG2
1602.420572	-0.050554753	0.212092981	-0.238361276	0.8116009	0.999975616	ENSG00000196743	GM2A
1606.305595	0.026656456	0.161123213	0.165441435	0.868596543	0.999975616	ENSG00000087053	MTMR2
1620.291028	0.044116649	0.206727557	0.213404782	0.831011251	0.999975616	ENSG00000015171	ZMYND11
1624.199154	1.309751481	0.7909051	1.656015975	0.097718584	0.981485048	ENSG00000170525	PFKFB3
1611.60527	-0.146764434	0.218498835	-0.671694354	0.501778296	0.999975616	ENSG00000162413	KLHL21
1604.648344	-0.04007719	0.169424814	-0.236548528	0.813007056	0.999975616	ENSG00000121579	NAA50
1599.411622	0.068295115	0.199902302	0.341642465	0.732619975	0.999975616	ENSG00000177963	RIC8A
1590.692964	0.046997613	0.188745928	0.24899935	0.803361284	0.999975616	ENSG00000174282	ZBTB4
1597.266898	-0.022698752	0.187957139	-0.120765574	0.903876718	0.999975616	ENSG00000130340	SNX9
1592.552718	0.135081868	0.183683192	0.735406799	0.462091782	0.999975616	ENSG00000110108	TMEM109
1588.113076	0.046464822	0.176768558	0.262856824	0.792660936	0.999975616	ENSG00000185651	UBE2L3
1614.33199	-0.068956675	0.179077723	-0.385065622	0.700188797	0.999975616	ENSG00000140931	CMTM3
1599.925932	0.050142315	0.191554622	0.261765101	0.793502546	0.999975616	ENSG00000173821	RNF213
1607.183077	0.095335115	0.216349322	0.440653637	0.659463766	0.999975616	ENSG00000115170	ACVR1
1575.072201	-0.183251256	0.263331873	-0.695894703	0.486494775	0.999975616	ENSG00000198121	LPAR1
1602.651766	-0.37675795	0.420461348	-0.896058464	0.370221539	0.999975616	ENSG00000179403	VWA1
1590.586024	0.171365821	0.179538065	0.954481832	0.339839801	0.999975616	ENSG00000196776	CD47
1599.977767	0.195821055	0.17998652	1.087976228	0.276605602	0.999975616	ENSG00000164880	INTS1
1612.155505	0.107529138	0.288863559	0.372248884	0.709707553	0.999975616	ENSG00000103222	ABCC1
1574.693684	-0.164707826	0.220157225	-0.74813727	0.454377364	0.999975616	ENSG00000105401	CDC37
1592.256626	0.113019818	0.200471481	0.563770052	0.572910636	0.999975616	ENSG00000151929	BAG3
1592.118363	0.088201547	0.192112166	0.459114843	0.646151698	0.999975616	ENSG00000100991	TRPC4AP
1578.474847	-0.294877709	0.233071962	-1.265178815	0.205807235	0.999975616	ENSG00000172809	RPL38
1561.515341	-0.309798189	0.291150502	-1.064048273	0.287306832	0.999975616	ENSG00000240342	RPS2P5
1563.972699	-0.228561317	0.278994957	-0.819231001	0.412654631	0.999975616	ENSG00000042445	RETSAT
1614.073975	0.395949421	0.306479885	1.291926289	0.196382673	0.999975616	ENSG00000150764	DIXDC1
1597.193912	-0.389903251	0.201909812	-1.93107629	0.053473619	0.903530068	ENSG00000100129	EIF3L
1585.995292	-0.133078873	0.163703325	-0.812927122	0.416259844	0.999975616	ENSG00000001629	ANKIB1
1578.995932	-0.185858263	0.175804884	-1.05718487	0.290427223	0.999975616	ENSG00000179889	PDXDC1
1591.024395	0.304003783	0.306014116	0.993430588	0.320500161	0.999975616	ENSG00000060982	BCAT1
1572.934683	-0.107045196	0.177254004	-0.603908481	0.54590449	0.999975616	ENSG00000115935	WIPF1
1576.13657	-0.048044359	0.245364817	-0.195807855	0.844760561	0.999975616	ENSG00000156535	CD109
1568.053803	-0.145603164	0.170025267	-0.856361918	0.391797622	0.999975616	ENSG00000055070	SZRD1
1597.445367	-0.296842241	0.24040025	-1.234783412	0.216911123	0.999975616	ENSG00000130592	LSP1
1579.645976	0.351837993	0.234274581	1.501818898	0.133143886	0.999975616	ENSG00000110619	CARS
1574.156024	-0.04344638	0.185668837	-0.233999309	0.814985515	0.999975616	ENSG00000131051	RBM39
1566.21275	0.138830235	0.173015639	0.802414375	0.422313301	0.999975616	ENSG00000065882	TBC1D1
1594.307178	0.90998368	0.423825932	2.147069379	0.031787759	0.794279453	ENSG00000181458	TMEM45A
1575.494414	-0.218663757	0.158820695	-1.376796369	0.168575214	0.999975616	ENSG00000174780	SRP72
1550.013767	-1.132354563	0.878309497	-1.289243219	0.197313544	0.999975616	ENSG00000162745	OLFML2B
1526.440207	-0.407001228	0.413886801	-0.983363634	0.325428502	0.999975616	ENSG00000007933	FMO3
1604.193398	0.073397156	0.444336495	0.165183722	0.868799377	0.999975616	ENSG00000115468	EFHD1
1586.687166	-0.186082952	0.215408667	-0.863860099	0.387664748	0.999975616	ENSG00000140262	TCF12
1572.920681	0.204629555	0.19903355	1.02811589	0.303895317	0.999975616	ENSG00000032444	PNPLA6
1570.759644	0.035502166	0.191915323	0.184988698	0.853237901	0.999975616	ENSG00000067900	ROCK1
1592.082631	0.100252624	0.291341409	0.34410702	0.730765804	0.999975616	ENSG00000108175	ZMIZ1
1568.38043	0.244508893	0.32320767	0.756507088	0.449345224	0.999975616	ENSG00000196526	AFAP1
1543.21255	-0.20912825	0.260826437	-0.801790849	0.422673953	0.999975616	ENSG00000123933	MXD4
1566.261636	-0.317054115	0.279547045	-1.134170871	0.25672288	0.999975616	ENSG00000062485	CS
1556.129362	0.174815056	0.227616624	0.768024116	0.442472858	0.999975616	ENSG00000050820	BCAR1
1569.370693	-0.043547024	0.19202078	-0.226782873	0.820592582	0.999975616	ENSG00000101346	POFUT1
1561.375645	-0.03214757	0.191658632	-0.167733483	0.866792953	0.999975616	ENSG00000148110	MFSD14B
1566.060551	0.730216243	0.44943475	1.624743622	0.104217198	0.987682951	ENSG00000107984	DKK1
1557.596621	0.110217559	0.16978725	0.649150976	0.516240796	0.999975616	ENSG00000108344	PSMD3
1559.826051	0.531099246	0.204787946	2.593410681	0.009502922	0.526777378	ENSG00000118689	FOXO3
1561.741975	0.241530162	0.295675431	0.816875993	0.413999298	0.999975616	ENSG00000198830	HMGN2
1598.129825	0.930294064	0.442217774	2.103701203	0.035404519	0.80685679	ENSG00000104812	GYS1
1600.594387	0.278955897	0.332503277	0.838956838	0.401493531	0.999975616	ENSG00000155090	KLF10

1548.52008	0.008974155	0.190072168	0.047214461	0.962342302	0.999975616	ENSG00000134779	TPGS2
1565.889064	-0.046771891	0.191317337	-0.24447283	0.806864642	0.999975616	ENSG00000049239	H6PD
1551.051661	-0.387506425	0.264282112	-1.466260514	0.142577328	0.999975616	ENSG00000172115	CYCS
1552.592411	0.055217371	0.172765996	0.319607864	0.749265612	0.999975616	ENSG00000198176	TFDP1
1553.971264	-0.164511934	0.200101794	-0.822141221	0.410996528	0.999975616	ENSG00000065427	KARS
1549.060527	-0.224675314	0.168460077	-1.33370065	0.182301982	0.999975616	ENSG00000151327	FAM177A1
1559.039011	0.199408544	0.211759071	0.941676514	0.346358283	0.999975616	ENSG00000100811	YY1
1571.435117	0.415577863	0.241439872	1.721247857	0.085205857	0.972981818	ENSG00000115866	DARS
1555.569132	-0.131504802	0.209959172	-0.626335116	0.531095158	0.999975616	ENSG00000079785	DDX1
1551.128828	-0.293978952	0.207594703	-1.416119715	0.156740457	0.999975616	ENSG00000116251	RPL22
1549.018919	-0.079892566	0.204509998	-0.390653597	0.696053303	0.999975616	ENSG00000186318	BACE1
1536.104858	-0.068642674	0.171890073	-0.399340538	0.689642301	0.999975616	ENSG00000119408	NEK6
1555.358839	-1.90E-05	0.19347351	-9.80E-05	0.999921805	0.999975616	ENSG00000134247	PTGFRN
1534.115185	-0.037641279	0.253494543	-0.148489505	0.881956468	0.999975616	ENSG00000008256	CYTH3
1541.59113	0.176543645	0.231572621	0.762368382	0.445840171	0.999975616	ENSG00000169554	ZEB2
1565.028128	-0.435344641	0.237088812	-1.83620913	0.066326733	0.937668618	ENSG00000104687	GSR
1569.308007	0.459230529	0.356784687	1.287136323	0.198046777	0.999975616	ENSG00000138685	FGF2
1540.000642	0.106426943	0.184569481	0.576622649	0.564194396	0.999975616	ENSG00000153130	SCOC
1551.155586	-0.118351474	0.194614889	-0.608131655	0.543100159	0.999975616	ENSG00000082701	GSK3B
1544.55591	0.049757232	0.162789411	0.305653983	0.759868114	0.999975616	ENSG00000055211	GINM1
1538.724687	-0.237845604	0.16077167	-1.479399976	0.139033447	0.999975616	ENSG00000115993	TRAK2
1547.043662	0.257886846	0.178728781	1.442894899	0.14905008	0.999975616	ENSG00000099331	MYO9B
1526.760836	-0.1828628	0.245437324	-0.745048868	0.456242179	0.999975616	ENSG00000170315	UBB
1546.079397	-0.657531013	0.240129157	-2.738238956	0.006176918	0.483939657	ENSG00000128335	APOL2
1517.217545	-0.040535109	0.312719061	-0.129621485	0.896865904	0.999975616	ENSG00000112096	SOD2
1522.349583	-0.234176683	0.23897664	-0.979914533	0.327128308	0.999975616	ENSG00000127184	COX7C
1543.468819	-0.174960381	0.194118554	-0.901306841	0.367425197	0.999975616	ENSG00000215021	PHB2
1545.467499	0.139639859	0.179666367	0.777217583	0.43703041	0.999975616	ENSG00000100504	PYGL
1538.852988	0.431373332	0.286993164	1.503078772	0.132818732	0.999975616	ENSG00000256235	SMIM3
1543.259266	0.309971856	0.224224248	1.38241898	0.166843092	0.999975616	ENSG00000172939	OXSR1
1549.077266	-0.007561543	0.232621362	-0.032505797	0.974068693	0.999975616	ENSG00000253729	PRKDC
1546.910004	0.207296	0.1901284	1.090294765	0.27558332	0.999975616	ENSG00000160216	AGPAT3
1562.389436	-0.155462867	0.316023772	-0.49193409	0.622765938	0.999975616	ENSG00000105429	MEGF8
1536.531318	-0.627261984	0.290613034	-2.158409671	0.030895995	0.788432379	ENSG00000019549	SNAI2
1532.931038	-0.313736066	0.202925078	-1.546068474	0.122088032	0.999975616	ENSG00000149577	SIDT2
1515.005522	0.067456612	0.206211526	0.327123384	0.743574566	0.999975616	ENSG00000125971	DYNLRB1
1522.109531	-0.202193051	0.246998497	-0.81860033	0.413014479	0.999975616	ENSG00000275832	ARHGAP23
1516.53297	0.035313832	0.315972313	0.111762424	0.911011782	0.999975616	ENSG00000118564	FBXL5
1539.100055	-0.034378131	0.217304532	-0.158202551	0.874297194	0.999975616	ENSG00000167693	NXN
1532.370748	-0.212917245	0.158588264	-1.34257883	0.179408389	0.999975616	ENSG00000131467	PSME3
1553.038864	-0.020061309	0.222247946	-0.090265444	0.92807628	0.999975616	ENSG00000124193	SRSF6
1530.799755	0.041222411	0.173170068	0.238045822	0.811845554	0.999975616	ENSG00000197111	PCBP2
1524.910692	-0.079575523	0.200460872	-0.39696287	0.691394841	0.999975616	ENSG00000129657	SEC14L1
1521.349972	-0.055272248	0.195426534	-0.282828777	0.777308093	0.999975616	ENSG00000197006	METTL9
1513.186215	-0.168502436	0.224266034	-0.751350674	0.45244164	0.999975616	ENSG00000100804	PSMB5
1527.139365	0.075022651	0.267857509	0.280084181	0.779412921	0.999975616	ENSG00000070081	NUCB2
1528.552361	0.097842426	0.418552186	0.233763984	0.815168212	0.999975616	ENSG00000136859	ANGPTL2
1522.182079	0.412538458	0.262762208	1.570006815	0.116413526	0.999975616	ENSG00000182809	CRIP2
1535.130199	0.309313664	0.225996814	1.368663821	0.171104384	0.999975616	ENSG00000164889	SLC4A2
1534.651606	0.373403064	0.215121025	1.735781354	0.082602502	0.970687665	ENSG00000175216	CKAP5
1531.718845	0.153333515	0.24710528	0.620518974	0.534916166	0.999975616	ENSG00000126012	KDM5C
1528.227114	0.220261204	0.261265264	0.843055831	0.39919722	0.999975616	ENSG00000099783	HNRNPM
1517.707588	0.620063288	0.226155112	2.741761092	0.006111077	0.483939657	ENSG00000189060	H1F0
1530.111788	-0.404180465	0.249051265	-1.622880595	0.104614932	0.987682951	ENSG00000138413	IDH1
1534.696293	0.175041714	0.215644194	0.811715406	0.416954952	0.999975616	ENSG00000080845	DLGAP4
1546.382769	0.334110999	0.273658287	1.220905832	0.222121676	0.999975616	ENSG00000075568	TMEM131
1511.25879	-0.461352601	0.209610677	-2.200997624	0.027736192	0.768050664	ENSG00000173598	NUDT4
1530.575336	0.117713902	0.311954493	0.377343183	0.705918584	0.999975616	ENSG00000173546	CSPG4
1513.362659	-0.022880646	0.172351437	-0.132755762	0.894386541	0.999975616	ENSG00000148672	GLUD1
1496.653523	-0.139325333	0.823546424	-0.169177267	0.865657211	0.999975616	ENSG00000173432	SAA1
1528.464205	0.007219594	0.240108789	0.030068012	0.976012812	0.999975616	ENSG00000211455	STK38L
1520.034443	0.013782806	0.193318571	0.071295819	0.943162323	0.999975616	ENSG00000101557	USP14
1518.108807	0.085784786	0.163061259	0.526089314	0.598826153	0.999975616	ENSG00000143621	ILF2
1511.861342	-0.360190467	0.24505958	-1.469807734	0.141613834	0.999975616	ENSG00000178996	SNX18

1515.388183	0.190806481	0.283004997	0.674215944	0.500174034	0.999975616	ENSG00000135631	RAB11FIP5
1510.095008	0.036164542	0.187788274	0.192581473	0.847286756	0.999975616	ENSG00000116962	NID1
1524.472362	0.052531812	0.19146543	0.274367087	0.783802534	0.999975616	ENSG00000100266	PACSIN2
1508.199814	-0.407370261	0.175227019	-2.324814201	0.020081897	0.682776641	ENSG00000114573	ATP6V1A
1514.711652	-0.230038016	0.220657699	-1.042510713	0.297174961	0.999975616	ENSG00000128487	SPECC1
1507.689309	-0.23027108	0.195576096	-1.177398899	0.239036329	0.999975616	ENSG00000138279	ANXA7
1518.570667	0.295525216	0.20159266	1.465952266	0.142661291	0.999975616	ENSG00000134954	ETS1
1512.285304	0.164929948	0.171029507	0.964336217	0.334877389	0.999975616	ENSG00000089154	GCN1
1506.344439	-0.118749906	0.200050754	-0.593598893	0.552780422	0.999975616	ENSG00000197451	HNRNPAB
1498.532636	0.12345477	0.241754789	0.510661115	0.609588374	0.999975616	ENSG00000141736	ERBB2
1504.956711	-0.241263819	0.225832974	-1.068328571	0.285372325	0.999975616	ENSG00000156482	RPL30
1524.158639	0.133266499	0.257217407	0.5181084	0.60438264	0.999975616	ENSG00000147010	SH3KBP1
1507.30951	0.126773282	0.226524115	0.559645857	0.57572102	0.999975616	ENSG00000115307	AUP1
1499.934953	0.360207625	0.164876005	2.184718291	0.028909508	0.772694606	ENSG00000178913	TAF7
1509.281868	0.043392819	0.204254981	0.212444363	0.831760379	0.999975616	ENSG00000152795	HNRNPDL
1496.379314	-0.019775569	0.168350102	-0.117466924	0.906490054	0.999975616	ENSG00000126067	PSMB2
1503.652277	0.10795696	0.152861456	0.706240557	0.480038553	0.999975616	ENSG00000146223	RPL7L1
1514.964336	-0.043335106	0.238636504	-0.181594623	0.855900869	0.999975616	ENSG00000136731	UGGT1
1485.889589	0.011036185	0.254228307	0.043410528	0.965374286	0.999975616	ENSG00000183722	LHFPL6
1501.934554	-0.108918108	0.161574687	-0.674103782	0.500245335	0.999975616	ENSG00000134882	UBAC2
1489.78142	-0.113344616	0.208579107	-0.543413086	0.586845418	0.999975616	ENSG00000165629	ATP5F1C
1489.455751	0.036257209	0.214556865	0.168986477	0.865807279	0.999975616	ENSG00000142166	IFNAR1
1464.714119	0.293523458	0.204237546	1.437166986	0.150670551	0.999975616	ENSG00000167815	PRDX2
1500.076909	-0.26329397	0.20424636	-1.289099938	0.197363345	0.999975616	ENSG00000143776	CDC42BPA
1471.607393	-0.142315664	0.241535966	-0.589211064	0.555719696	0.999975616	ENSG00000112514	CUTA
1486.741595	0.397209885	0.272098619	1.459801182	0.144344724	0.999975616	ENSG00000166130	IKBIP
1481.562671	0.028465858	0.167295572	0.170153084	0.864889747	0.999975616	ENSG00000160310	PRMT2
1516.644477	0.192741901	0.325791496	0.591611211	0.554110965	0.999975616	ENSG00000182704	TSKU
1461.089718	-0.173731016	0.287319984	-0.6046604	0.545404665	0.999975616	ENSG00000160213	CSTB
1490.680999	0.16837998	0.176729644	0.952754598	0.340714423	0.999975616	ENSG00000106771	TMEM245
1481.657303	0.104900987	0.173076462	0.606096207	0.544450869	0.999975616	ENSG00000122565	CBX3
1480.890174	0.012005005	0.156598735	0.076660933	0.938893284	0.999975616	ENSG00000171055	FEZ2
1479.443755	-0.013957463	0.186568623	-0.074811415	0.94036476	0.999975616	ENSG00000139684	ESD
1487.515949	-6.93E-05	0.175522838	-0.000395049	0.999684797	0.999975616	ENSG00000085719	CPNE3
1494.128803	0.217762598	0.329093952	0.661703434	0.508161303	0.999975616	ENSG00000154734	ADAMTS1
1464.101084	0.069485053	0.23200945	0.299492339	0.764564416	0.999975616	ENSG00000160932	LY6E
1483.535421	0.135891458	0.176330216	0.770664617	0.44090575	0.999975616	ENSG00000121774	KHDRBS1
1456.661679	0.11713149	0.262611422	0.446025878	0.655578553	0.999975616	ENSG00000126458	RRAS
1461.891753	0.025854281	0.241919754	0.106871308	0.914891076	0.999975616	ENSG00000105355	PLIN3
1474.712365	-0.080433621	0.207690151	-0.387277009	0.698551144	0.999975616	ENSG00000115084	SLC35F5
1504.551246	0.245797387	0.306522162	0.801891077	0.422615968	0.999975616	ENSG00000134109	EDEM1
1459.505769	-0.053311718	0.241225803	-0.221003381	0.825089801	0.999975616	ENSG00000126432	PRDX5
1487.743418	-0.564134559	0.344294772	-1.638522004	0.101312848	0.987682951	ENSG00000198848	CES1
1496.714266	0.27162427	0.221360349	1.227068313	0.219796916	0.999975616	ENSG00000181222	POLR2A
1477.007375	-0.274736147	0.205659207	-1.335880611	0.181588299	0.999975616	ENSG00000213741	RPS29
1468.520366	-0.189202369	0.219727522	-0.861077246	0.389195501	0.999975616	ENSG00000124172	ATP5F1E
1468.850869	0.187257709	0.434898797	0.430577666	0.666775483	0.999975616	ENSG00000221869	CEBPD
1473.350137	-0.029767096	0.177575574	-0.167630577	0.866873915	0.999975616	ENSG00000089248	ERP29
1473.571782	0.034407634	0.164625151	0.209005935	0.834443605	0.999975616	ENSG00000182149	IST1
1469.863319	0.03141985	0.166997458	0.188145681	0.850762452	0.999975616	ENSG00000100554	ATP6V1D
1466.665681	-0.168272593	0.203938081	-0.825116095	0.409305683	0.999975616	ENSG00000110756	HPS5
1337.049567	1.521212781	1.276104694	1.192075218	0.233231753	0.999975616	ENSG00000174697	LEP
1448.768445	0.00807479	0.254067026	0.031782124	0.974645802	0.999975616	ENSG00000143198	MGST3
1467.24706	-0.205711114	0.216962506	-0.948141305	0.343057523	0.999975616	ENSG00000089053	ANAPC5
1471.60093	0.011719071	0.228666168	0.051249694	0.959126554	0.999975616	ENSG00000160746	ANO10
1470.613224	0.160998388	0.254986734	0.631399074	0.527779621	0.999975616	ENSG00000107438	PDLIM1
1474.995408	-0.043637036	0.151347722	-0.288323046	0.773099468	0.999975616	ENSG00000108256	NUFIP2
1474.911222	-0.101741978	0.19438058	-0.523416374	0.600684532	0.999975616	ENSG00000143515	ATP8B2
1483.378152	0.099874789	0.201443007	0.495796754	0.620037815	0.999975616	ENSG00000148396	SEC16A
1464.326607	0.059867742	0.188088987	0.318294778	0.750261345	0.999975616	ENSG00000101421	CHMP4B
1469.735509	0.044774733	0.162889355	0.274878204	0.783409813	0.999975616	ENSG00000269378	AC022149.1
1483.266369	0.416427598	0.320749861	1.298293934	0.19418635	0.999975616	ENSG00000125629	INSIG2
1451.042299	0.206533457	0.212422867	0.972275067	0.330913724	0.999975616	ENSG00000125970	RALY
1464.285511	0.108190584	0.163579533	0.661394379	0.50835943	0.999975616	ENSG00000119242	CCDC92

1477.339263	-0.210872215	0.181008177	-1.164987233	0.244024161	0.999975616	ENSG00000012983	MAP4K5
1445.309232	-0.154143948	0.256732475	-0.600406895	0.548235094	0.999975616	ENSG00000168653	NDUFS5
1487.601821	0.3827915	0.273885719	1.397632198	0.162223544	0.999975616	ENSG00000169045	HNRNPH1
1462.082749	-0.134199634	0.159718976	-0.840223484	0.400783094	0.999975616	ENSG00000143337	TOR1AIP1
1459.880538	0.092251846	0.1680203	0.549051787	0.582969911	0.999975616	ENSG00000196715	VKORC1L1
1454.632193	-0.056910953	0.235324531	-0.241840291	0.808903918	0.999975616	ENSG00000117410	ATP6V0B
1456.31053	0.167321141	0.177309472	0.943667243	0.345339718	0.999975616	ENSG00000102158	MAGT1
1478.920931	0.283798407	0.28465449	0.996992554	0.318768124	0.999975616	ENSG00000106070	GRB10
1479.696768	0.21287015	0.249706521	0.85248134	0.393946991	0.999975616	ENSG00000101782	RIOK3
1453.4488	0.368203411	0.27042497	1.361573273	0.173332595	0.999975616	ENSG00000105137	SYDE1
1440.386388	-0.122181385	0.229683768	-0.53195481	0.594757295	0.999975616	ENSG00000186298	PPP1CC
1465.487324	0.112111606	0.215257039	0.520826669	0.602487523	0.999975616	ENSG00000064393	HIPK2
1460.181329	0.428046964	0.275556384	1.553391572	0.120329615	0.999975616	ENSG00000141580	WDR45B
1471.40046	0.07255164	0.267293442	0.271430676	0.786059811	0.999975616	ENSG00000136986	DERL1
1447.942325	-0.010867656	0.181542971	-0.059862718	0.952264974	0.999975616	ENSG00000116786	PLEKHM2
1444.410564	0.085344125	0.182947188	0.466495965	0.640860535	0.999975616	ENSG00000159842	ABR
1455.117872	-0.038276217	0.17370031	-0.220357795	0.825592512	0.999975616	ENSG00000029364	SLC39A9
1455.600797	0.05712076	0.177370435	0.322042173	0.747420745	0.999975616	ENSG00000257103	LSM14A
1449.273786	0.33717478	0.212649601	1.585588583	0.112832668	0.997355609	ENSG00000068366	ACSL4
1447.292878	-0.155285431	0.165112798	-0.94048089	0.346970948	0.999975616	ENSG00000087302	RTRAF
1466.913201	0.464124331	0.2745121	1.690724492	0.090889434	0.9781243	ENSG00000069869	NEDD4
1439.726433	0.140781736	0.193464182	0.72768889	0.466804053	0.999975616	ENSG00000109113	RAB34
1441.156823	0.123643483	0.177492934	0.69661073	0.486046439	0.999975616	ENSG00000177879	AP3S1
1444.491993	-0.190486091	0.230387732	-0.826806571	0.408346705	0.999975616	ENSG00000005243	COPZ2
1431.87471	0.061497647	0.22871874	0.268878918	0.788022861	0.999975616	ENSG00000185222	TCEAL9
1438.986833	-0.48120121	0.240591492	-2.000075753	0.045492085	0.871859801	ENSG00000100439	ABHD4
1432.685878	-0.058461992	0.220561565	-0.265059743	0.790963439	0.999975616	ENSG00000141985	SH3GL1
1446.858739	-0.060941373	0.170582732	-0.357254058	0.72090162	0.999975616	ENSG00000198833	UBE2J1
1442.487363	0.175140131	0.166826992	1.049830902	0.293795865	0.999975616	ENSG00000144043	TEX261
1438.613388	0.144598944	0.264886766	0.545889651	0.585141793	0.999975616	ENSG00000099139	PCSK5
1446.662719	0.202933549	0.187786351	1.080661869	0.279847551	0.999975616	ENSG00000187555	USP7
1454.508089	0.19368252	0.275407689	0.703257489	0.481895298	0.999975616	ENSG00000167522	ANKRD11
1447.608223	-0.521075888	0.399058483	-1.305763215	0.191633093	0.999975616	ENSG00000170345	FOS
1430.628372	-0.154582752	0.178246705	-0.867240448	0.385810279	0.999975616	ENSG00000120805	ARL1
1413.383347	-0.656822604	0.344907802	-1.904342552	0.056865586	0.917136198	ENSG00000130176	CNN1
1428.644113	-0.220087659	0.178490673	-1.233048512	0.217557665	0.999975616	ENSG00000135679	MDM2
1427.696746	-0.322692969	0.259523494	-1.243405611	0.213718403	0.999975616	ENSG00000164543	STK17A
1434.844329	-0.02002308	0.2505669	-0.079911115	0.936307949	0.999975616	ENSG00000063245	EPN1
1430.372465	-0.134060334	0.194504704	-0.689239545	0.490672535	0.999975616	ENSG00000111142	METAP2
1536.035815	1.263143298	1.169150201	1.080394373	0.2799666	0.999975616	ENSG00000185633	NDUFA4L2
1422.439756	0.370122263	0.208016256	1.779294898	0.075191427	0.952536833	ENSG00000092820	EZR
1433.041145	-0.081760089	0.180405749	-0.453201124	0.650403923	0.999975616	ENSG00000122068	FYTTD1
1407.065909	-0.235724661	0.241053235	-0.977894616	0.328126448	0.999975616	ENSG00000228474	OST4
1449.152424	0.272820421	0.361398436	0.754902053	0.450307754	0.999975616	ENSG00000159200	RCAN1
1422.971329	0.040394531	0.156021657	0.258903357	0.795709808	0.999975616	ENSG00000078140	UBE2K
1426.423209	-0.021198922	0.197839044	-0.107152365	0.914668105	0.999975616	ENSG00000139921	TMX1
1439.960624	-0.132669373	0.208917665	-0.635031857	0.525407638	0.999975616	ENSG00000134313	KIDINS220
1417.268038	-0.02196335	0.208545037	-0.105317057	0.916124228	0.999975616	ENSG00000174851	YIF1A
1438.578506	0.253402595	0.235636693	1.075395315	0.282197778	0.999975616	ENSG00000179051	RCC2
1414.76151	-0.456133178	0.747965829	-0.609831574	0.541973384	0.999975616	ENSG00000157168	NRG1
1416.942797	-0.039665049	0.209059041	-0.189731325	0.849519673	0.999975616	ENSG00000161057	PSMC2
1415.991595	-0.116870594	0.169610953	-0.689050982	0.490791185	0.999975616	ENSG00000136448	NMT1
1414.564566	-0.198914161	0.767965696	-0.259014383	0.795624143	0.999975616	ENSG00000115738	ID2
1400.692493	0.858167051	0.348846422	2.460013909	0.013893163	0.635512264	ENSG00000177606	JUN
1423.58197	0.110361583	0.172817891	0.63860045	0.523082889	0.999975616	ENSG00000101266	CSNK2A1
1422.738889	0.049600431	0.294177334	0.168607249	0.866105578	0.999975616	ENSG00000174080	CTSF
1423.196989	-0.07047155	0.167630001	-0.420399388	0.674193715	0.999975616	ENSG00000138085	ATRAID
1412.28973	-0.290590039	0.178448045	-1.62842938	0.103433872	0.987682951	ENSG00000107745	MICU1
1429.109167	-0.006809285	0.202062297	-0.033698938	0.973117226	0.999975616	ENSG00000160551	TAOK1
1413.105628	-0.382377611	0.262058911	-1.459128444	0.144529759	0.999975616	ENSG00000184867	ARMCX2
1410.103512	0.009347709	0.180682055	0.051735678	0.958739308	0.999975616	ENSG00000138434	ITPRID2
1418.985527	-0.127862538	0.224389278	-0.569824633	0.568796646	0.999975616	ENSG00000029363	BCLAF1
1402.145959	-0.167276988	0.256437821	-0.652310129	0.514201127	0.999975616	ENSG00000139112	GABARAPL1
1410.581609	-0.099793947	0.161627278	-0.617432581	0.536949437	0.999975616	ENSG00000064726	BTBD1

1410.848722	-0.017314487	0.186553601	-0.092812396	0.926052603	0.999975616	ENSG00000167930	FAM234A
1386.049823	0.002531207	0.264527027	0.009568802	0.992365317	0.999975616	ENSG00000131981	LGALS3
1413.055799	-0.164652815	0.165245051	-0.996416011	0.319048057	0.999975616	ENSG00000072062	PRKACA
1402.869588	0.011138898	0.201333876	0.055325501	0.955879146	0.999975616	ENSG00000135272	MDFIC
1401.633034	0.213171448	0.238885523	0.892358172	0.372200983	0.999975616	ENSG00000113621	TXNDC15
1412.805643	-0.04509067	0.156816188	-0.287538362	0.773700133	0.999975616	ENSG00000189091	SF3B3
1447.146091	-1.061377092	0.388843037	-2.729577212	0.00634156	0.483939657	ENSG00000169231	THBS3
1411.417779	0.162232026	0.194240592	0.835211756	0.403598492	0.999975616	ENSG00000102225	CDK16
1396.018897	-0.165156443	0.268427837	-0.615273156	0.538374342	0.999975616	ENSG00000204386	NEU1
1410.412219	0.050914177	0.20115162	0.253113431	0.80018056	0.999975616	ENSG00000167110	GOLGA2
1407.301353	0.084762472	0.176356574	0.4806312	0.630778637	0.999975616	ENSG00000084652	TXLNA
1408.548079	-0.41205205	0.186456636	-2.209908208	0.027111534	0.768050664	ENSG00000214717	ZBED1
1392.483345	0.013168982	0.190827994	0.069009698	0.9449819	0.999975616	ENSG00000085998	POMGNT1
1398.075546	-0.414871516	0.324684855	-1.27776676	0.201331678	0.999975616	ENSG00000105373	NOP53
1399.30148	0.042545868	0.259616511	0.163879668	0.869825873	0.999975616	ENSG00000105810	CDK6
1405.647762	0.889992322	0.738075104	1.205828942	0.227883453	0.999975616	ENSG00000175040	CHST2
1421.556008	0.447854267	0.290372493	1.542343979	0.122990027	0.999975616	ENSG00000131018	SYNE1
1383.093708	-0.054242674	0.261272919	-0.207609249	0.835534082	0.999975616	ENSG00000093167	LRRFIP2
1412.789031	-0.002384003	0.182529221	-0.013060939	0.989579175	0.999975616	ENSG00000182831	C16orf72
1394.930168	-0.019486683	0.20850076	-0.093460967	0.925537358	0.999975616	ENSG00000132002	DNAJB1
1393.022414	0.034950807	0.192212276	0.181834419	0.855712673	0.999975616	ENSG00000146112	PPP1R18
1394.843094	0.022358827	0.156736873	0.142651989	0.886565036	0.999975616	ENSG00000077721	UBE2A
1398.293242	0.107601403	0.205608125	0.523332449	0.600742923	0.999975616	ENSG00000016864	GLT8D1
1394.17727	0.33290555	0.194117197	1.714971961	0.086350368	0.974422538	ENSG00000089280	FUS
1403.989918	0.326909636	0.202124496	1.617367722	0.105798929	0.987682951	ENSG00000125505	MBOAT7
1385.267195	-0.373854069	0.339761381	-1.100343037	0.271182687	0.999975616	ENSG00000068878	PSME4
1400.437127	-0.168073645	0.185255035	-0.907255475	0.364271714	0.999975616	ENSG00000153989	NUS1
1402.468432	0.060274585	0.184131065	0.327346092	0.743406135	0.999975616	ENSG00000196591	HDAC2
1392.616245	-0.310649232	0.37972589	-0.818088101	0.413306883	0.999975616	ENSG00000099250	NRP1
1392.972991	0.09738583	0.238456001	0.408401674	0.682978805	0.999975616	ENSG00000150457	LATS2
1383.80291	0.012302148	0.282664804	0.043522037	0.965285398	0.999975616	ENSG00000163346	PBXIP1
1401.501629	-0.133791703	0.253449899	-0.527882249	0.597581066	0.999975616	ENSG00000179195	ZNF664
1386.899887	-0.115680701	0.161116756	-0.717992988	0.47276161	0.999975616	ENSG00000168288	MMADHC
1420.375993	0.210689764	0.290648092	0.724896427	0.468515573	0.999975616	ENSG00000101384	JAG1
1383.987258	-0.026995671	0.198858882	-0.135752905	0.892016622	0.999975616	ENSG00000073578	SDHA
1383.592433	-0.163293854	0.22629076	-0.721610791	0.470533803	0.999975616	ENSG00000064115	TM7SF3
1383.196218	-0.215396876	0.189420378	-1.137136765	0.255481117	0.999975616	ENSG00000164506	STXBP5
1376.174992	0.053873863	0.183597556	0.293434532	0.769190033	0.999975616	ENSG00000079805	DNM2
1386.657242	0.079493778	0.224138725	0.35466329	0.722841856	0.999975616	ENSG00000104164	BLOC1S6
1385.66347	-0.03253716	0.170721004	-0.190586744	0.848849376	0.999975616	ENSG00000119689	DLST
1388.077	-0.234448688	0.186443953	-1.257475415	0.208581536	0.999975616	ENSG00000117859	OSBPL9
1385.291769	0.10714027	0.183678328	0.583303814	0.559688795	0.999975616	ENSG00000147475	ERLIN2
1377.042254	-0.127553946	0.208478095	-0.611833807	0.54064772	0.999975616	ENSG00000136888	ATP6V1G1
1360.331497	0.023211716	0.246079472	0.094326096	0.924850121	0.999975616	ENSG00000103202	NME4
1371.046046	-0.223560588	0.230393838	-0.970341003	0.331876546	0.999975616	ENSG00000173786	CNP
1364.777941	-0.182712016	0.243987128	-0.748859244	0.453942048	0.999975616	ENSG00000182054	IDH2
1385.839845	0.035467395	0.160512468	0.22096349	0.825120861	0.999975616	ENSG00000109814	UGDH
1378.512412	-0.051130335	0.194476924	-0.262912094	0.792618334	0.999975616	ENSG00000107341	UBE2R2
1385.433302	0.240101482	0.175240481	1.370125677	0.170647674	0.999975616	ENSG00000136827	TOR1A
1387.06589	-0.295222647	0.244393454	-1.20798099	0.227054575	0.999975616	ENSG00000142627	EPHA2
1356.901523	-0.031236001	0.277228901	-0.112672239	0.910290411	0.999975616	ENSG00000166681	BEX3
1387.988804	0.450955025	0.273384426	1.649527117	0.099039692	0.982968395	ENSG00000130827	PLXNA3
1372.241096	0.053598445	0.174563966	0.30704186	0.758811511	0.999975616	ENSG00000150459	SAP18
1365.46368	-0.037537927	0.223104071	-0.168252991	0.866384255	0.999975616	ENSG00000168575	SLC20A2
1361.924737	-0.274186285	0.213213798	-1.285968771	0.198453961	0.999975616	ENSG00000118515	SGK1
1390.986299	0.300448711	0.239907672	1.252351413	0.210441842	0.999975616	ENSG00000116641	DOCK7
1383.905211	-0.127681114	0.245362267	-0.52037795	0.602800175	0.999975616	ENSG00000144674	GOLGA4
1385.387238	-0.057597611	0.174875272	-0.329363955	0.741880609	0.999975616	ENSG00000138138	ATAD1
1382.11258	0.059573477	0.179836629	0.331264423	0.740444762	0.999975616	ENSG00000074657	ZNF532
1388.415656	-0.071307749	0.249124442	-0.28623345	0.774699325	0.999975616	ENSG00000185164	NOMO2
1384.171818	0.079880794	0.182195827	0.438433719	0.661071908	0.999975616	ENSG00000180776	ZDHHC20
1354.970683	0.629059678	0.320308165	1.963920207	0.049539346	0.893790775	ENSG00000068305	MEF2A
1352.003237	-0.208580399	0.416792092	-0.500442315	0.616763664	0.999975616	ENSG00000130164	LDLR
1364.669141	-0.076283454	0.223382241	-0.341492922	0.732732532	0.999975616	ENSG00000123144	TRIR

1387.732986	0.104282418	0.288289683	0.361727887	0.717555385	0.999975616	ENSG00000164465	DCBLD1
1369.908914	-0.006931342	0.183918799	-0.037686971	0.969937264	0.999975616	ENSG00000090615	GOLGA3
1366.45813	-0.058839086	0.185043098	-0.317975037	0.750503873	0.999975616	ENSG00000006007	GDE1
1374.026439	-0.009920737	0.167979201	-0.059059317	0.952904862	0.999975616	ENSG00000185728	YTHDF3
1354.96389	0.125766902	0.195621749	0.642908589	0.520283416	0.999975616	ENSG00000127884	ECHS1
1368.808316	-0.016789592	0.181862745	-0.092320126	0.926443698	0.999975616	ENSG00000164244	PRRC1
1369.667549	0.063791231	0.174881619	0.364768073	0.715284539	0.999975616	ENSG00000168175	MAPK1IP1L
1372.048843	0.135675692	0.158350396	0.856806769	0.391551684	0.999975616	ENSG00000080815	PSEN1
1366.922447	0.01957866	0.201262937	0.097279013	0.922504822	0.999975616	ENSG00000112249	ASCC3
1364.213182	0.041433872	0.222681556	0.186067819	0.852391578	0.999975616	ENSG00000100284	TOM1
1358.256658	-0.12668566	0.197228372	-0.642329793	0.520659074	0.999975616	ENSG00000111481	COPZ1
1365.092099	-0.444357082	0.221322491	-2.007735771	0.044671377	0.867769731	ENSG00000139793	MBNL2
1374.092814	0.198369298	0.2414995	0.821406659	0.411414672	0.999975616	ENSG00000214517	PPME1
1361.006921	0.047115822	0.161449605	0.2918299	0.770416683	0.999975616	ENSG00000196235	SUPT5H
1368.888345	0.137452356	0.199620233	0.688569256	0.491094374	0.999975616	ENSG00000108061	SHOC2
1385.552703	-0.531693367	0.316171455	-1.68166151	0.092634496	0.9781243	ENSG00000155850	SLC26A2
1359.876165	-0.236464856	0.198767874	-1.189653291	0.234182691	0.999975616	ENSG00000100403	ZC3H7B
1356.939455	0.063345831	0.182798499	0.346533651	0.728941699	0.999975616	ENSG00000152492	CCDC50
1362.735421	-0.106123542	0.207206552	-0.512163064	0.608536891	0.999975616	ENSG00000122591	FAM126A
1360.590498	0.231222512	0.226724858	1.019837499	0.307805536	0.999975616	ENSG00000106392	C1GALT1
1359.048649	0.140958887	0.200360652	0.703525796	0.481728137	0.999975616	ENSG00000108861	DUSP3
1369.500234	0.367045104	0.239906856	1.529948369	0.126029509	0.999975616	ENSG00000173120	KDM2A
1347.045282	-0.048809018	0.201637915	-0.2420627	0.80873158	0.999975616	ENSG00000158109	TPRG1L
1370.462153	-0.056385685	0.232144214	-0.242890762	0.808090022	0.999975616	ENSG00000010017	RANBP9
1355.541107	-0.295351845	0.190113427	-1.55355595	0.120290374	0.999975616	ENSG00000174306	ZHX3
1351.435876	0.046021613	0.177424536	0.259386971	0.795336681	0.999975616	ENSG00000122034	GTF3A
1346.62666	0.133857802	0.240512825	0.556551618	0.577833818	0.999975616	ENSG00000106348	IMPDH1
1364.474227	-0.105064355	0.205386809	-0.511543828	0.608970307	0.999975616	ENSG00000130723	PRRC2B
1346.046503	-0.039185888	0.182352602	-0.214890753	0.829852495	0.999975616	ENSG00000175387	SMAD2
1350.320405	0.346223985	0.222772704	1.554158018	0.120146729	0.999975616	ENSG00000152127	MGAT5
1357.584011	-0.148354512	0.182718468	-0.811929491	0.416832091	0.999975616	ENSG00000106460	TMEM106B
1352.416078	-0.143762759	0.188318645	-0.76340162	0.445223914	0.999975616	ENSG00000166887	VPS39
1345.746452	-0.216256758	0.229150053	-0.943734268	0.345305458	0.999975616	ENSG00000100823	APEX1
1356.223493	-0.106746211	0.204546447	-0.52186783	0.601762358	0.999975616	ENSG00000165732	DDX21
1349.811068	-0.035947088	0.172630761	-0.20823107	0.83504855	0.999975616	ENSG00000117505	DR1
1346.051507	-0.113942184	0.217095575	-0.524848027	0.599688842	0.999975616	ENSG00000147853	AK3
1363.940211	-0.12808391	0.212348035	-0.603179161	0.546389509	0.999975616	ENSG00000074054	CLASP1
1349.897952	0.311198282	0.176717759	1.760990432	0.078240022	0.962537177	ENSG00000099917	MED15
1342.060182	0.126723862	0.183718232	0.689772925	0.490336998	0.999975616	ENSG00000010244	ZNF207
1362.307327	0.175084881	0.228491095	0.766265667	0.443518243	0.999975616	ENSG00000117020	AKT3
1341.868304	-0.036892232	0.194742692	-0.189440904	0.849747268	0.999975616	ENSG00000090487	SPG21
1358.537815	0.050583924	0.199286047	0.253825716	0.799630205	0.999975616	ENSG00000117523	PRRC2C
1351.212512	-0.06469246	0.164605802	-0.393014459	0.694308802	0.999975616	ENSG00000153147	SMARCA5
1340.859652	-0.018305647	0.163979492	-0.11163376	0.911113803	0.999975616	ENSG00000136930	PSMB7
1332.777819	-0.075665855	0.20254137	-0.373582219	0.708715165	0.999975616	ENSG00000099817	POLR2E
1343.923566	-0.007849511	0.184470941	-0.042551476	0.966059077	0.999975616	ENSG00000088247	KHSRP
1368.220077	0.695563332	0.359395599	1.935369645	0.052944951	0.903530068	ENSG00000198768	APCDD1L
1350.80099	0.624015652	0.395548287	1.577596647	0.114658303	0.997513711	ENSG00000182481	KPNA2
1345.002634	0.08700873	0.166539348	0.522451485	0.601356017	0.999975616	ENSG00000141551	CSNK1D
1336.18742	0.063692397	0.180187965	0.353477528	0.723730473	0.999975616	ENSG00000088256	GNA11
1345.093857	0.091156038	0.204868968	0.444948004	0.65635733	0.999975616	ENSG00000173230	GOLGB1
1331.840986	-0.070067273	0.205933245	-0.340242651	0.733673801	0.999975616	ENSG00000006042	TMEM98
1331.456401	-0.02790012	0.19947147	-0.13987023	0.888762523	0.999975616	ENSG00000106244	PDAP1
1329.959752	-0.00663986	0.186241294	-0.03565192	0.971559909	0.999975616	ENSG00000125743	SNRPD2
1350.737558	0.085154304	0.190558864	0.446866138	0.654971715	0.999975616	ENSG00000005007	UPF1
1327.905656	-0.376862447	0.232291214	-1.622370648	0.10472401	0.987682951	ENSG00000124098	FAM210B
1337.036352	-0.061584324	0.239151821	-0.257511414	0.796784005	0.999975616	ENSG00000125944	HNRNPR
1344.02651	0.165504675	0.234532467	0.705679162	0.480387683	0.999975616	ENSG00000054965	FAM168A
1332.19159	-0.18463732	0.166059266	-1.111876045	0.26619145	0.999975616	ENSG00000017797	RALBP1
1338.217757	0.027340168	0.188251815	0.145231898	0.884527782	0.999975616	ENSG00000139218	SCAF11
1342.115248	-0.203994325	0.18049262	-1.130208676	0.258388305	0.999975616	ENSG00000136051	WASHC4
1331.692572	0.063328099	0.161877187	0.391210772	0.695641446	0.999975616	ENSG00000105612	DNASE2
1348.792858	1.572957577	0.445992077	3.526873366	0.000420498	0.163806497	ENSG00000147852	VLDLR
1318.100562	0.144159894	0.202346897	0.712439362	0.476192748	0.999975616	ENSG00000167657	DAPK3

1337.490684	0.223531743	0.273794312	0.816422158	0.414258728	0.999975616	ENSG00000127418	FGFRL1
1336.560714	-0.086456247	0.191860071	-0.450621367	0.652262464	0.999975616	ENSG00000114439	BBX
1336.396375	0.121306982	0.178470064	0.679704928	0.496691315	0.999975616	ENSG00000198952	SMG5
1333.893607	0.291245621	0.219838575	1.324815813	0.185232249	0.999975616	ENSG00000176407	KCMF1
1315.945838	0.033261419	0.194130373	0.17133547	0.863959992	0.999975616	ENSG00000159348	CYB5R1
1321.036025	-0.076500732	0.194305395	-0.393713886	0.693792288	0.999975616	ENSG00000196975	ANXA4
1317.801721	-0.080181044	0.216468945	-0.370404374	0.711081215	0.999975616	ENSG00000125753	VASP
1306.460268	0.249042617	0.19214443	1.296121969	0.194933467	0.999975616	ENSG00000175550	DRAP1
1331.0846	0.328756344	0.254159424	1.293504443	0.195836648	0.999975616	ENSG00000004455	AK2
1314.989307	0.046270868	0.192220706	0.240717396	0.809774156	0.999975616	ENSG00000230989	HSBP1
1318.976644	0.044021363	0.255423293	0.172346706	0.863164968	0.999975616	ENSG00000255112	CHMP1B
1333.963384	0.387171077	0.268616966	1.441350048	0.149485815	0.999975616	ENSG00000150347	ARID5B
1317.848902	-0.089314492	0.162715046	-0.548901252	0.583073219	0.999975616	ENSG00000142186	SCYL1
1334.231984	0.249461446	0.233596209	1.067917354	0.285557794	0.999975616	ENSG00000110422	HIPK3
1319.449021	-0.106438422	0.228462869	-0.465889368	0.641294692	0.999975616	ENSG00000149084	HSD17B12
1327.428068	0.010600314	0.164773095	0.064332796	0.94870524	0.999975616	ENSG00000205581	HMGN1
1336.815316	0.150790251	0.261105488	0.577507013	0.563597002	0.999975616	ENSG00000169967	MAP3K2
1327.79818	-0.08774874	0.209282497	-0.419283702	0.675008807	0.999975616	ENSG00000152767	FARP1
1311.279996	-0.148345728	0.205465636	-0.721997757	0.470295856	0.999975616	ENSG00000117758	STX12
1326.133763	0.103064714	0.211116804	0.488188113	0.625416604	0.999975616	ENSG00000152332	UHMK1
1327.53615	0.039379513	0.169287482	0.232619165	0.816057144	0.999975616	ENSG00000154845	PPP4R1
1325.170393	0.042397624	0.196063806	0.216244012	0.828797549	0.999975616	ENSG00000120686	UFM1
1333.089289	-0.006130492	0.225211008	-0.027221103	0.978283384	0.999975616	ENSG00000069956	MAPK6
1328.926611	0.244818271	0.25353834	0.965606508	0.334241118	0.999975616	ENSG00000010810	FYN
1322.0016	0.071993065	0.203320351	0.354086862	0.723273788	0.999975616	ENSG00000072042	RDH11
1318.500343	-0.043880691	0.178307819	-0.24609516	0.80560857	0.999975616	ENSG00000107862	GBF1
1307.836298	-0.071589251	0.233515584	-0.306571619	0.759169459	0.999975616	ENSG00000214063	TSPAN4
1311.537455	-0.373248395	0.24019853	-1.553916234	0.120204399	0.999975616	ENSG00000135048	CEMIP2
1322.99702	-0.037657991	0.177196677	-0.212520866	0.831700701	0.999975616	ENSG00000134001	EIF2S1
1321.868493	-0.076455046	0.219132203	-0.348899182	0.727164999	0.999975616	ENSG00000058272	PPP1R12A
1318.315125	-0.014595124	0.202568528	-0.072050302	0.942561877	0.999975616	ENSG00000117114	ADGRL2
1309.822052	0.112427831	0.177519389	0.63332705	0.526520093	0.999975616	ENSG00000100225	FBXO7
1321.118032	-0.019998862	0.177340269	-0.112771129	0.910212008	0.999975616	ENSG00000054118	THRAP3
1310.730913	-0.063812231	0.19035158	-0.335233526	0.737448934	0.999975616	ENSG00000243317	STMP1
1300.031171	0.195035104	0.216473417	0.900965608	0.367606606	0.999975616	ENSG00000132471	WBP2
1303.945183	0.011226104	0.221765842	0.050621432	0.959627185	0.999975616	ENSG00000078902	TOLLIP
1316.531564	0.015806289	0.155590355	0.101589131	0.919082807	0.999975616	ENSG00000055917	PUM2
1308.810305	-0.325403465	0.312521057	-1.04122093	0.297773024	0.999975616	ENSG00000149218	ENDOD1
1313.824988	-0.047554439	0.175148958	-0.271508544	0.78599993	0.999975616	ENSG00000087111	PIGS
1293.469879	-0.342877061	0.246314732	-1.392028229	0.16391385	0.999975616	ENSG00000132274	TRIM22
1309.615578	-0.06005752	0.177556486	-0.338244586	0.735178879	0.999975616	ENSG00000166200	COPS2
1317.050535	0.148319447	0.216462528	0.685196873	0.493219697	0.999975616	ENSG00000213593	TMX2
1311.852209	0.871501229	0.441065114	1.975901518	0.048165932	0.887092455	ENSG00000119950	MXI1
1296.469502	0.050485531	0.185008267	0.272882569	0.784943486	0.999975616	ENSG00000071859	FAM50A
1310.527155	0.095756416	0.158972351	0.602346356	0.54694361	0.999975616	ENSG00000167881	SRP68
1294.467919	0.237305998	0.2310001	1.027298245	0.304280048	0.999975616	ENSG00000159363	ATP13A2
1294.122274	-0.175702326	0.213152366	-0.824303896	0.409766906	0.999975616	ENSG00000143252	SDHC
1305.684393	-0.152263522	0.293093884	-0.519504262	0.603409142	0.999975616	ENSG00000144867	SRPRB
1292.709624	0.299052382	0.189324733	1.579573764	0.114204511	0.997355609	ENSG00000130202	NECTIN2
1304.183164	-0.113949348	0.253976798	-0.448660465	0.653676609	0.999975616	ENSG00000113273	ARSB
1312.539204	-0.029470234	0.217323429	-0.135605417	0.892133223	0.999975616	ENSG00000125875	TBC1D20
1304.443205	0.080690293	0.165404069	0.487837412	0.62566501	0.999975616	ENSG00000119383	PTPA
1310.505794	-0.42227994	0.274551426	-1.538072291	0.12403094	0.999975616	ENSG00000132000	PODNL1
1308.383427	0.020000244	0.167435906	0.119450147	0.904918734	0.999975616	ENSG00000092201	SUPT16H
1300.692269	-0.408196701	0.221951226	-1.839127939	0.06589637	0.937668618	ENSG00000112992	NNT
1292.070543	0.121626826	0.229312661	0.530397342	0.595836468	0.999975616	ENSG00000133243	BTBD2
1301.651369	0.190274832	0.180249241	1.055620709	0.291141536	0.999975616	ENSG00000182944	EWSR1
1307.29673	0.003219186	0.167106197	0.019264315	0.984630251	0.999975616	ENSG00000141380	SS18
1299.842653	0.025249394	0.189199826	0.133453578	0.893834675	0.999975616	ENSG00000070413	DGCR2
1295.973184	0.004524796	0.178197611	0.025392012	0.979742283	0.999975616	ENSG00000109919	MTCH2
1309.033116	0.126207866	0.187569897	0.672857789	0.501037769	0.999975616	ENSG00000126261	UBA2
1297.732022	0.042424017	0.165465547	0.256391848	0.797648282	0.999975616	ENSG00000115839	RAB3GAP1
1300.910246	-0.04633856	0.173679353	-0.266805232	0.789619126	0.999975616	ENSG00000131966	ACTR10
1302.670674	0.261493414	0.220266779	1.187166831	0.235161822	0.999975616	ENSG00000163602	RYBP

1292.413225	0.343094715	0.186682224	1.837854234	0.066083888	0.937668618	ENSG00000164828	SUN1
1299.480927	-0.127588477	0.184467708	-0.691657515	0.489152433	0.999975616	ENSG00000114650	SCAP
1306.969949	0.11414408	0.271914932	0.41977864	0.67464717	0.999975616	ENSG00000131435	PDLIM4
1300.530231	0.105498277	0.205992601	0.512145956	0.608548864	0.999975616	ENSG00000153201	RANBP2
1285.261475	0.141432885	0.200926042	0.703905199	0.481491813	0.999975616	ENSG00000186866	POFUT2
1298.555234	-0.04824799	0.179885612	-0.268214837	0.788533955	0.999975616	ENSG00000138246	DNAJC13
1314.031882	0.297655884	0.252269894	1.17991045	0.238035833	0.999975616	ENSG00000100596	SPTLC2
1300.99264	0.16486991	0.191071008	0.862872458	0.388207595	0.999975616	ENSG00000009954	BAZ1B
1291.499063	-0.472851987	0.286813576	-1.648638792	0.099221657	0.982968395	ENSG00000170581	STAT2
1286.952157	0.130350754	0.185634806	0.70218919	0.482561186	0.999975616	ENSG00000130177	CDC16
1290.630018	-0.085407649	0.16122201	-0.529751794	0.596284032	0.999975616	ENSG00000134287	ARF3
1275.615016	0.065153172	0.249932102	0.260683487	0.794336602	0.999975616	ENSG00000123159	GIPC1
1290.06829	0.270343466	0.183887012	1.470160744	0.141518224	0.999975616	ENSG00000109606	DHX15
1274.379635	0.032282341	0.235128608	0.137296524	0.890796418	0.999975616	ENSG00000051523	CYBA
1293.038438	-0.220009216	0.256447211	-0.857912301	0.390940891	0.999975616	ENSG00000120694	HSPH1
1286.400957	-0.154347608	0.209577433	-0.736470552	0.461444383	0.999975616	ENSG00000116649	SRM
1281.017572	0.175649942	0.246773158	0.711787067	0.476596642	0.999975616	ENSG00000105854	PON2
1268.303727	-0.140023964	0.269003029	-0.520529322	0.602694696	0.999975616	ENSG00000079459	FDFT1
1285.996537	-0.264734266	0.272944709	-0.969919022	0.332086858	0.999975616	ENSG00000131238	PPT1
1290.906942	-0.058743721	0.158102393	-0.371554912	0.710224264	0.999975616	ENSG00000173674	EIF1AX
1274.739627	0.230424309	0.202143732	1.139903312	0.254326585	0.999975616	ENSG00000143537	ADAM15
1296.754026	-0.01072206	0.232695969	-0.046077549	0.96324844	0.999975616	ENSG00000169826	CSGALNACT2
1285.104242	0.102486017	0.175276961	0.584709002	0.5587434	0.999975616	ENSG00000140367	UBE2Q2
1287.488673	-0.107211746	0.217828351	-0.492184539	0.622588893	0.999975616	ENSG00000125107	CNOT1
1271.438026	-0.152366602	0.219802044	-0.693199204	0.488184548	0.999975616	ENSG00000004142	POLDIP2
1285.49022	0.065477334	0.226360282	0.289261583	0.772381208	0.999975616	ENSG00000077097	TOP2B
1281.749064	0.796371385	0.27937837	2.850511961	0.004364891	0.447970647	ENSG00000169032	MAP2K1
1289.772076	-1.052125396	0.449889613	-2.33863011	0.019354585	0.682776641	ENSG00000143341	HMCN1
1272.289744	0.030956629	0.206199532	0.150129483	0.88066246	0.999975616	ENSG00000148834	GSTO1
1279.153917	-0.168902358	0.167960908	-1.005605171	0.314605535	0.999975616	ENSG00000134419	RPS15A
1278.925888	0.0113695	0.157470672	0.072200749	0.942442149	0.999975616	ENSG00000204574	ABCF1
1282.366593	-0.398884801	0.256521347	-1.554977023	0.119951542	0.999975616	ENSG00000142657	PGD
1279.021442	-0.024970386	0.159569743	-0.156485718	0.875650176	0.999975616	ENSG00000132670	PTPRA
1279.733293	-0.06216207	0.165258132	-0.376151353	0.706804379	0.999975616	ENSG00000080371	RAB21
1273.727891	0.195907816	0.188988984	1.036609711	0.299917786	0.999975616	ENSG00000117362	APH1A
1274.608054	0.060988966	0.173488915	0.351543879	0.72518036	0.999975616	ENSG00000185627	PSMD13
1264.581505	0.610609848	0.2647992	2.305935396	0.021114242	0.686354235	ENSG00000163659	TIPARP
1292.027666	-0.600840923	0.328074808	-1.831414386	0.067038716	0.938628653	ENSG00000099998	GGT5
1273.86141	-0.04048952	0.19380334	-0.208920652	0.834510182	0.999975616	ENSG00000151240	DIP2C
1280.057784	-0.052150501	0.172567556	-0.302203392	0.762497019	0.999975616	ENSG00000105270	CLIP3
1279.632001	-0.283242698	0.253817953	-1.11592854	0.264452724	0.999975616	ENSG00000134853	PDGFRA
1244.057888	-0.738346795	0.345349301	-2.137971015	0.032519096	0.794279453	ENSG00000154856	APCDD1
1268.672883	-0.128950352	0.218630947	-0.589808323	0.555319162	0.999975616	ENSG00000105221	AKT2
1242.592566	1.502606362	1.355706777	1.108356459	0.267707901	0.999975616	ENSG00000159167	STC1
1252.071077	-0.297872736	0.340932453	-0.873700153	0.382281568	0.999975616	ENSG00000170624	SGCD
1263.715002	-0.044410371	0.202713627	-0.219079357	0.826588231	0.999975616	ENSG00000179364	PACS2
1277.847269	0.03623852	0.170929445	0.212008643	0.832100291	0.999975616	ENSG00000103429	BFAR
1263.622666	-0.137259715	0.209920178	-0.653866228	0.513197994	0.999975616	ENSG00000041357	PSMA4
1283.507897	-0.055566952	0.354096709	-0.156925922	0.87530323	0.999975616	ENSG00000149380	P4HA3
1272.832061	-0.306017769	0.183547091	-1.667243908	0.095465915	0.9781243	ENSG00000179134	SAMD4B
1260.489886	-0.11073642	0.234227949	-0.472772016	0.636375841	0.999975616	ENSG00000138698	RAP1GDS1
1280.289789	0.22872652	0.231942265	0.98613558	0.324066586	0.999975616	ENSG00000176903	PNMA1
1270.611877	0.03509391	0.161137676	0.217788357	0.827594017	0.999975616	ENSG00000076043	REXO2
1257.964972	-0.180007163	0.203142539	-0.886112595	0.375556857	0.999975616	ENSG00000111652	COPS7A
1272.493076	0.380427723	0.297360497	1.27934856	0.200774339	0.999975616	ENSG00000144579	CTDSP1
1238.654669	-0.121862798	0.344518945	-0.353718712	0.723549698	0.999975616	ENSG00000177700	POLR2L
1268.041141	0.183600454	0.265541014	0.691420322	0.489301437	0.999975616	ENSG00000183010	PYCR1
1267.679168	-0.016769053	0.233206933	-0.071906323	0.942676458	0.999975616	ENSG00000140450	ARRDC4
1271.165216	0.021382327	0.192971538	0.1108056	0.911770505	0.999975616	ENSG00000118217	ATF6
1288.270302	0.112811794	0.218894263	0.515371178	0.606293669	0.999975616	ENSG00000080503	SMARCA2
1250.570564	-0.736290496	0.318102987	-2.314629307	0.020633228	0.682776641	ENSG00000128656	CHN1
1247.364144	0.086111534	0.283392794	0.303859294	0.761235091	0.999975616	ENSG00000148516	ZEB1
1259.824452	0.179322503	0.1891873	0.947856982	0.343202267	0.999975616	ENSG00000120265	PCMT1
1268.138871	-0.020080836	0.204408512	-0.098238745	0.921742717	0.999975616	ENSG00000103249	CLCN7

1270.984648	-0.201077	0.191207396	-1.051617268	0.292975182	0.999975616	ENSG00000101350	KIF3B	
1285.732919	-0.064976788	0.227251538	-0.285924526	0.774935929	0.999975616	ENSG00000131791	PRKAB2	
1254.315023	-0.035284389	0.223159903	-0.158112585	0.874368084	0.999975616	ENSG00000102038	SMARCA1	
1272.833903	0.282395444	0.217608099	1.297724876	0.194381892	0.999975616	ENSG00000113580	NR3C1	
1287.147974	0.395830314	0.294069949	1.346041361	0.178289178	0.999975616	ENSG00000128050	PAICS	
1250.509645	0.043002441	0.200637016	0.21432955	0.830290076	0.999975616	ENSG00000126698	DNAJC8	
1236.936741	0.769623157	0.370418824	2.077710708	0.037736012	0.835184568	ENSG00000099953	MMP11	
1261.599485	-0.00113797	0.168803211	-0.006741403	0.994621179	0.999975616	ENSG00000113282	CLINT1	
1275.282196	0.164456015	0.215001163	0.764907559	0.444326593	0.999975616	ENSG00000113194	FAF2	
1251.247208	0.142185024	0.175687283	0.809307436	0.41833833	0.999975616	ENSG00000185236	RAB11B	
1261.659867	-0.18620538	0.190072564	-0.979654171	0.327256855	0.999975616	ENSG00000159658	EFCAB14	
1243.510951	0.133649667	0.198469559	0.673401341	0.500691996	0.999975616	ENSG00000104824	HNRNPL	
1248.175159	-0.112432865	0.23679413	-0.474812719	0.634920467	0.999975616	ENSG00000054148	PHPT1	
1258.13302	-0.2327128	0.201757866	-1.153426156	0.248735511	0.999975616	ENSG00000175224	ATG13	
1258.730927	0.136403346	0.204734111	0.666246308	0.505253678	0.999975616	ENSG00000139645	ANKRD52	
1241.519951	0.252460884	0.285820783	0.883283856	0.377082924	0.999975616	ENSG00000131408	NR1H2	
1259.469864	0.118910645	0.164855913	0.721300457	0.470724676	0.999975616	ENSG00000140299	BNIP2	
1256.789896	0.005566296	0.250273295	0.022240871	0.982255816	0.999975616	ENSG00000171148	TADA3	
1256.775288	-0.24841173	0.305654619	-0.812720354	0.416378409	0.999975616	ENSG00000102996	MMP15	
1258.810245	-0.052003061	0.160174033	-0.324665989	0.745433879	0.999975616	ENSG00000108312	UBTF	
1279.634328	0.651659548	0.39328909	1.65694794	0.097530001	0.981485048	ENSG00000116584	ARHGEF2	
1253.734845	0.058876644	0.173361124	0.339618493	0.73414385	0.999975616	ENSG00000119414	PPP6C	
1255.023362	-0.371626645	0.182254605	-2.039052156	0.041444822	0.855344797	ENSG00000101444	AHCY	
1228.917883	-0.453478211	0.39720514	-1.141672568	0.253590148	0.999975616	ENSG00000127863	TNFRSF19	
1250.879052	0.000600823	0.173121323	0.003470534	0.99723092	0.999975616	ENSG00000182827	ACBD3	
1247.377763	0.244191479	0.261516617	0.933751289	0.350432202	0.999975616	ENSG00000140511	HAPLN3	
1256.397732	0.012328205	0.158258309	0.077899262	0.937908187	0.999975616	ENSG00000166181	API5	
1253.519296	0.251436702	0.257055276	0.978142547	0.328003827	0.999975616	ENSG00000110841	PPFIBP1	
1253.184481	0.104591146	0.206332016	0.506907013	0.612220065	0.999975616	ENSG00000188706	ZDHHC9	
1245.951306	-0.162489893	0.210345484	-0.772490525	0.439823956	0.999975616	ENSG00000134375	TIMM17A	
1255.441732	-0.054637001	0.170539697	-0.320377026	0.748682539	0.999975616	ENSG00000116221	MRPL37	
1258.301912	0.113010387	0.176271446	0.641115675	0.52144753	0.999975616	ENSG00000123684	LPGAT1	
1241.026102	-0.384066721	0.271642211	-1.413869809	0.157400132	0.999975616	ENSG00000119812	FAM98A	
1247.086496	-0.019600679	0.16471737	-0.118995826	0.905278663	0.999975616	ENSG00000063244	U2AF2	
1253.223756	0.006400946	0.165268718	0.038730537	0.969105227	0.999975616	ENSG00000122958	VPS26A	
1259.255956	0.080543907	0.20834811	0.386583334	0.699064698	0.999975616	ENSG00000065613	SLK	
1256.170298	-0.072221655	0.190506343	-0.379103676	0.704610877	0.999975616	ENSG00000006451	RALA	
1247.873403	-0.066396795	0.202456264	-0.327956238	0.742944751	0.999975616	ENSG00000087470	DNM1L	
1245.035435	-0.597880235	0.223151883	-2.679252482	0.007378673	0.502485114	ENSG00000156467	UQCRB	
1257.224686	-0.024941814	0.231426206	-0.107774371	0.914174673	0.999975616	ENSG00000152700	SAR1B	
1249.446303	0.147726086	0.188025919	0.785668736	0.432061584	0.999975616	ENSG00000127616	SMARCA4	
1248.520404	-0.065118351	0.198508109	-0.328038745	0.742882368	0.999975616	ENSG00000132305	IMMT	
1249.373409	0.119802034	0.167041064	0.717201095	0.473250024	0.999975616	ENSG00000131508	UBE2D2	
1252.658948	0.089218519	0.159010891	0.561084328	0.574740051	0.999975616	ENSG00000186522		10-Sep
1246.414837	0.166949512	0.21742426	0.767851354	0.442575501	0.999975616	ENSG00000131871	SELENOS	
1238.21515	-0.030619014	0.195943076	-0.156264844	0.875824266	0.999975616	ENSG00000122729	ACO1	
1250.914873	0.137051836	0.180835517	0.757881185	0.448522115	0.999975616	ENSG00000188895	MSL1	
1235.386671	0.240748005	0.18933482	1.271546379	0.203534339	0.999975616	ENSG00000105520	PLPPR2	
1230.535095	-0.535864648	0.225115577	-2.380397908	0.017293952	0.670428193	ENSG00000205302	SNX2	
1239.933378	0.29386237	0.218559564	1.344541341	0.178773397	0.999975616	ENSG00000105968	H2AFV	
1251.199054	-0.140847662	0.181238693	-0.777139029	0.43707675	0.999975616	ENSG00000104324	CPQ	
1242.569294	-0.122949236	0.156003094	-0.788120495	0.430626228	0.999975616	ENSG00000014824	SLC30A9	
1245.576425	-0.291959491	0.192212186	-1.518943712	0.128776665	0.999975616	ENSG00000113716	HMGXB3	
1240.808089	-0.210309462	0.195035119	-1.078315859	0.280892822	0.999975616	ENSG00000115944	COX7A2L	
1242.842166	-0.007056249	0.165645521	-0.042598492	0.966021597	0.999975616	ENSG00000183020	AP2A2	
1241.36473	0.417197988	0.226294072	1.843609886	0.065240014	0.937668618	ENSG00000132432	SEC61G	
1246.911736	-0.018726108	0.204775534	-0.091447	0.927137418	0.999975616	ENSG00000118200	CAMSAP2	
1238.62898	-0.139899578	0.170100192	-0.822453965	0.410818577	0.999975616	ENSG00000181904	C5orf24	
1241.568337	-0.088154816	0.153735443	-0.573418948	0.566361064	0.999975616	ENSG00000107758	PPP3CB	
1241.876599	-0.050391362	0.226155863	-0.222816961	0.823677967	0.999975616	ENSG00000099622	CIRBP	
1239.094853	0.121470886	0.162533526	0.747358952	0.454846916	0.999975616	ENSG00000157954	WIPI2	
1234.554901	-0.253731227	0.160170917	-1.584127955	0.113164604	0.997355609	ENSG00000058091	CDK14	
1246.662292	0.195825588	0.266908959	0.733679336	0.463144195	0.999975616	ENSG00000077235	GTF3C1	
1230.199247	-0.069632694	0.168246259	-0.413873651	0.678966642	0.999975616	ENSG00000276293	PIP4K2B	

1240.10512	-0.147575944	0.176356051	-0.836806809	0.402701169	0.999975616	ENSG00000171490	RSL1D1
1232.536906	-0.050615793	0.16661194	-0.303794514	0.761284446	0.999975616	ENSG00000116473	RAP1A
1231.433785	-0.084775744	0.209342166	-0.40496258	0.685505017	0.999975616	ENSG00000130638	ATXN10
1198.568532	0.153199907	0.256804003	0.596563545	0.550798816	0.999975616	ENSG00000151729	SLC25A4
1224.094673	-0.056242395	0.190004994	-0.296004823	0.767226398	0.999975616	ENSG00000179950	PUF60
1233.822589	-0.080054283	0.276950306	-0.289056487	0.772538151	0.999975616	ENSG00000154511	FAM69A
1227.206966	-0.124307147	0.184220532	-0.674773576	0.499819631	0.999975616	ENSG00000160714	UBE2Q1
1240.158754	-0.005437238	0.193267171	-0.028133273	0.977555857	0.999975616	ENSG00000004897	CDC27
1226.243989	-0.048130944	0.160448263	-0.299977971	0.764193959	0.999975616	ENSG00000116199	FAM20B
1241.285639	0.128708582	0.204504492	0.629367994	0.529108167	0.999975616	ENSG00000169018	FEM1B
1226.688834	-0.040295079	0.20967088	-0.192182523	0.847599235	0.999975616	ENSG00000158417	EIF5B
1217.590394	-0.13888565	0.191214456	-0.726334463	0.467633757	0.999975616	ENSG00000205531	NAP1L4
1235.048565	-0.057121273	0.1999222	-0.285717509	0.775094494	0.999975616	ENSG00000107854	TNKS2
1227.326001	0.355764245	0.226972811	1.567431107	0.117013958	0.999975616	ENSG00000140443	IGF1R
1225.755631	-0.02131358	0.16252141	-0.131143214	0.895662016	0.999975616	ENSG00000025800	KPNA6
1232.05716	0.235668969	0.205392543	1.147407618	0.251213194	0.999975616	ENSG00000085365	SCAMP1
1251.189207	0.429548098	0.667384085	0.643629519	0.519815704	0.999975616	ENSG00000204291	COL15A1
1224.851133	0.134399568	0.211743726	0.634727509	0.525606149	0.999975616	ENSG00000107566	ERLIN1
1231.782971	-0.932379478	1.013111006	-0.920313246	0.357409089	0.999975616	ENSG00000115884	SDC1
1216.966863	0.151943111	0.24439718	0.621705665	0.534135426	0.999975616	ENSG00000214022	REPIN1
1219.708774	-0.257548071	0.16846558	-1.528787493	0.126317134	0.999975616	ENSG00000103353	UBFD1
1224.84062	0.161147755	0.179019451	0.900168972	0.368030335	0.999975616	ENSG00000170242	USP47
1200.056314	0.002912511	0.29663865	0.009818381	0.992166191	0.999975616	ENSG00000145349	CAMK2D
1226.248938	0.407281164	0.210735238	1.932667584	0.053277161	0.903530068	ENSG00000146648	EGFR
1226.873982	-0.03657506	0.172684513	-0.211802779	0.832260901	0.999975616	ENSG00000198231	DDX42
1226.739968	0.081402699	0.186787647	0.435803442	0.662979347	0.999975616	ENSG00000172239	PAIP1
1214.010687	-0.25554486	0.182637576	-1.399191038	0.161755703	0.999975616	ENSG00000115561	CHMP3
1209.275279	0.31273677	0.229467497	1.362880471	0.172920184	0.999975616	ENSG00000159128	IFNGR2
1230.897623	-0.140175347	0.188484318	-0.743697667	0.457059401	0.999975616	ENSG00000119638	NEK9
1224.349901	-0.217881812	0.196404037	-1.109355061	0.267277041	0.999975616	ENSG00000138095	LRPPRC
1222.37961	0.153442417	0.205128259	0.748031589	0.454441104	0.999975616	ENSG00000118363	SPCS2
1208.104174	-0.208861758	0.260722474	-0.801088432	0.423080452	0.999975616	ENSG00000178035	IMPDH2
1212.886937	0.109063317	0.303215277	0.359689386	0.71907943	0.999975616	ENSG00000104341	LAPTM4B
1200.003676	0.005730099	0.207211178	0.027653427	0.977938569	0.999975616	ENSG00000196821	C6orf106
1183.995632	0.595163213	0.299611612	1.986449086	0.046983481	0.881360317	ENSG00000136861	CDK5RAP2
1220.208774	0.140071692	0.188664919	0.742436339	0.457823009	0.999975616	ENSG00000058063	ATP11B
1212.528561	0.128955201	0.244488946	0.527447982	0.59788253	0.999975616	ENSG00000164253	WDR41
1221.398482	0.278983591	0.187855969	1.485093033	0.137519181	0.999975616	ENSG00000156113	KCNMA1
1220.716606	-0.065161184	0.238381351	-0.273348499	0.784585337	0.999975616	ENSG00000159592	GPBP1L1
1208.386728	0.054456422	0.175683595	0.309968739	0.756584729	0.999975616	ENSG00000186063	AIDA
1190.18926	0.778661355	0.435477151	1.788064776	0.073765558	0.947863913	ENSG00000183691	NOG
1207.963095	-0.120759945	0.281345981	-0.429222213	0.667761519	0.999975616	ENSG00000121691	CAT
1214.3991	0.073102786	0.211028675	0.346411625	0.72903339	0.999975616	ENSG00000061273	HDAC7
1195.482498	-0.101192269	0.25381324	-0.398687905	0.690123181	0.999975616	ENSG00000087088	BAX
1216.032627	-0.059762692	0.179509372	-0.332922404	0.739192857	0.999975616	ENSG00000196504	PRPF40A
1202.006724	-0.112236391	0.210074102	-0.534270478	0.593154414	0.999975616	ENSG00000112062	MAPK14
1219.120474	-0.047060055	0.194578682	-0.241856172	0.808891612	0.999975616	ENSG00000104067	TJP1
1222.540804	-0.002526461	0.221686122	-0.01139657	0.99090705	0.999975616	ENSG00000253352	TUG1
1246.729014	0.686300478	0.415478073	1.651833206	0.098568557	0.982968395	ENSG00000100889	PCK2
1198.581046	-0.185066944	0.20870755	-0.886728553	0.375225062	0.999975616	ENSG00000028528	SNX1
1212.249321	0.198783571	0.205041302	0.969480631	0.33230544	0.999975616	ENSG00000054793	ATP9A
1203.621965	-0.059995184	0.166742532	-0.359807325	0.718991225	0.999975616	ENSG00000141002	TCF25
1201.776205	0.082786405	0.208336736	0.397368253	0.691095923	0.999975616	ENSG00000136816	TOR1B
1206.674731	-0.118241414	0.159616622	-0.74078384	0.458824515	0.999975616	ENSG00000189241	TSPYL1
1208.072694	-0.141348048	0.271593254	-0.520440203	0.602756795	0.999975616	ENSG00000184500	PROS1
1207.774787	-0.029666915	0.203673687	-0.145659048	0.884190552	0.999975616	ENSG00000166747	AP1G1
1200.281531	-0.083661912	0.175751116	-0.476024928	0.634056619	0.999975616	ENSG00000186660	ZFP91
1195.672394	0.082618766	0.172958806	0.477678866	0.632878788	0.999975616	ENSG00000124357	NAGK
1191.888911	0.269783055	0.268128206	1.006171856	0.314332908	0.999975616	ENSG00000114450	GNB4
1200.905314	0.016622437	0.166865527	0.099615764	0.920649378	0.999975616	ENSG00000144455	SUMF1
1199.900239	-0.149707729	0.338331942	-0.442487717	0.658136317	0.999975616	ENSG00000135052	GOLM1
1187.701358	0.29981625	0.203748019	1.471505103	0.141154569	0.999975616	ENSG00000177889	UBE2N
1204.199161	-0.000459883	0.168096641	-0.002735824	0.997817131	0.999975616	ENSG00000115486	GGCX
1152.680399	0.000105959	0.368994058	0.000287156	0.999770882	0.999975616	ENSG00000064205	WISP2

1213.991632	-0.477030641	0.302792512	-1.575437379	0.115155522	0.998842831	ENSG00000149212	SESN3
1198.082773	-0.077301986	0.172213021	-0.448874223	0.653522392	0.999975616	ENSG00000241973	PI4KA
1200.522408	0.0568154	0.179942872	0.315741319	0.752198861	0.999975616	ENSG00000055130	CUL1
1195.08667	0.170324623	0.207590397	0.820484116	0.411940181	0.999975616	ENSG00000136527	TRA2B
1201.3966	-0.060895227	0.189471404	-0.321395345	0.74791081	0.999975616	ENSG00000143164	DCAF6
1144.360623	-1.014542343	0.450711022	-2.25098188	0.024386685	0.736436292	ENSG00000184292	TACSTD2
1189.180241	-0.072700088	0.275687326	-0.26370486	0.792007351	0.999975616	ENSG00000119977	TCTN3
1195.339205	-0.052748706	0.208815253	-0.252609451	0.800570026	0.999975616	ENSG00000153317	ASAP1
1195.228576	0.295996109	0.268049998	1.104257083	0.269481634	0.999975616	ENSG00000073008	PVR
1196.289344	-0.222158577	0.292417646	-0.759730406	0.44741575	0.999975616	ENSG00000067113	PLPP1
1201.132667	-0.093386512	0.244172687	-0.382460924	0.702119508	0.999975616	ENSG00000100221	JOSD1
1184.695595	0.03291355	0.23155585	0.14214087	0.886968737	0.999975616	ENSG00000068323	TFE3
1185.678647	0.013998195	0.184026196	0.076066316	0.939366338	0.999975616	ENSG00000111666	CHPT1
1187.124082	0.217564183	0.176200622	1.23475264	0.216922578	0.999975616	ENSG00000165169	DYNLT3
1197.323573	0.300958912	0.244488878	1.230971789	0.218333416	0.999975616	ENSG00000099942	CRKL
1182.061976	-0.163399904	0.176190139	-0.927406639	0.353715445	0.999975616	ENSG00000159352	PSMD4
1191.802226	-0.049048229	0.159328864	-0.307842711	0.75820202	0.999975616	ENSG00000163930	BAP1
1200.55	0.081924699	0.301477417	0.271744066	0.785818816	0.999975616	ENSG00000137507	LRRC32
1179.137906	0.364042363	0.281944017	1.291186691	0.196638949	0.999975616	ENSG00000107816	LZTS2
1190.932763	0.260585991	0.192272908	1.355292294	0.175324449	0.999975616	ENSG00000137876	RSL24D1
1187.516723	0.014094805	0.274026884	0.051435849	0.958978219	0.999975616	ENSG00000179632	MAF1
1218.29703	-0.95033907	0.347115547	-2.737817651	0.006184836	0.483939657	ENSG00000160161	CILP2
1177.994284	-0.172437343	0.224391882	-0.768465158	0.442210883	0.999975616	ENSG00000113387	SUB1
1163.253476	-0.316782797	0.318397906	-0.994927388	0.319771581	0.999975616	ENSG00000112576	CCND3
1193.470148	-0.131708713	0.245110697	-0.537343796	0.591030158	0.999975616	ENSG00000100299	ARSA
1170.033865	-0.352589504	0.222858498	-1.582122774	0.113621546	0.997355609	ENSG00000124299	PEPD
1193.817844	-0.019188202	0.198096655	-0.096862825	0.922835332	0.999975616	ENSG00000109572	CLCN3
1193.456837	0.314972866	0.223920854	1.406625866	0.159538359	0.999975616	ENSG00000112378	PERP
1193.426983	0.455929136	0.287456169	1.586082278	0.112720646	0.997355609	ENSG00000147650	LRP12
1185.689283	0.019991166	0.159663154	0.125208386	0.900358579	0.999975616	ENSG00000066044	ELAVL1
1177.313445	-0.014842157	0.210438381	-0.070529706	0.943772057	0.999975616	ENSG00000163683	SMIM14
1210.734908	-0.571921445	0.376766552	-1.517972978	0.129021211	0.999975616	ENSG00000237973	MTCO1P12
1188.678476	-0.465182393	0.258363772	-1.800493889	0.071782688	0.945224913	ENSG00000121064	SCPEP1
1185.886787	0.310221546	0.245465165	1.263810879	0.206297915	0.999975616	ENSG00000173039	RELA
1162.713703	0.443674718	0.169749706	2.613699473	0.008956778	0.526777378	ENSG00000255248	MIR100HG
1170.626582	0.048433344	0.192247042	0.251932845	0.801092971	0.999975616	ENSG00000172531	PPP1CA
1177.868638	0.360125324	0.188105266	1.914488268	0.055557797	0.916132452	ENSG00000138594	TMOD3
1180.304126	0.221359875	0.229517601	0.964457081	0.334816816	0.999975616	ENSG00000168488	ATXN2L
1180.970451	0.057808798	0.216938941	0.26647497	0.789873433	0.999975616	ENSG00000104517	UBR5
1183.347914	0.226588692	0.272169961	0.832526453	0.405111853	0.999975616	ENSG00000133065	SLC41A1
1185.370314	0.061476247	0.204339703	0.30085317	0.763526462	0.999975616	ENSG00000101126	ADNP
1171.622992	0.087214639	0.175813062	0.496064614	0.619848824	0.999975616	ENSG00000119820	YIPF4
1161.550005	-0.192860037	0.27325455	-0.705788932	0.480319406	0.999975616	ENSG00000103994	ZNF106
1176.819792	0.020075788	0.28650474	0.070071401	0.944136829	0.999975616	ENSG00000186350	RXRA
1179.127202	0.217430009	0.203720677	1.067294749	0.285838759	0.999975616	ENSG00000154473	BUB3
1176.917947	0.144493596	0.183005199	0.789560059	0.429784742	0.999975616	ENSG00000121741	ZMYM2
1174.050435	-0.011024352	0.159455498	-0.069137486	0.944880183	0.999975616	ENSG00000198700	IPO9
1176.296729	-0.180989093	0.24464162	-0.739813173	0.459413365	0.999975616	ENSG00000137831	UACA
1177.053309	0.078917614	0.236709669	0.333394128	0.738836795	0.999975616	ENSG00000103978	TMEM87A
1176.976442	-0.173558957	0.204149404	-0.850156569	0.395238044	0.999975616	ENSG00000166326	TRIM44
1177.761969	0.467698002	0.289731257	1.614247652	0.10647372	0.989151081	ENSG00000135480	KRT7
1168.654877	-0.032051309	0.204100291	-0.157037056	0.875215644	0.999975616	ENSG00000147533	GOLGA7
1217.088857	0.099062837	0.778491741	0.127249696	0.898742773	0.999975616	ENSG00000177283	FZD8
1167.905398	-0.168977919	0.172706544	-0.978410629	0.327871273	0.999975616	ENSG00000165934	CPSF2
1160.198879	0.250170228	0.211091956	1.185124401	0.235968265	0.999975616	ENSG00000100997	ABHD12
1176.644732	0.052114807	0.20147504	0.258666318	0.79589271	0.999975616	ENSG00000197217	ENTPD4
1163.02008	0.207963157	0.193603469	1.07417061	0.282746222	0.999975616	ENSG00000117500	TMED5
1156.71365	-0.194813656	0.202714564	-0.961024465	0.336539867	0.999975616	ENSG00000013306	SLC25A39
1163.04777	0.096235858	0.185788582	0.517985859	0.604468136	0.999975616	ENSG00000025293	PHF20
1158.710104	0.153734167	0.203119722	0.756864795	0.449130869	0.999975616	ENSG00000131165	CHMP1A
1172.175732	0.222673911	0.185298688	1.201702575	0.229478783	0.999975616	ENSG00000197771	MCMBP
1169.10816	-0.065151045	0.260737773	-0.249871909	0.802686408	0.999975616	ENSG00000213614	HEXA
1155.852466	-0.582251705	0.232916377	-2.499831541	0.012425237	0.606822891	ENSG00000136003	ISCU
1192.662547	0.342317545	0.391191701	0.875063414	0.381539402	0.999975616	ENSG00000166173	LARP6

1164.663307	-0.101013785	0.181799317	-0.555633469	0.578461447	0.999975616	ENSG00000125166	GOT2
1159.27654	0.054517924	0.198900958	0.274095836	0.784010973	0.999975616	ENSG00000138385	SSB
1168.562994	-0.058413535	0.192382485	-0.303632293	0.761408046	0.999975616	ENSG00000100422	CERK
1164.501194	-0.039954701	0.1768978	-0.225863189	0.821307829	0.999975616	ENSG00000139687	RB1
1165.402438	-0.463874535	0.266892827	-1.738055461	0.082201042	0.969369989	ENSG00000106823	ECM2
1174.669829	-0.099850394	0.251103763	-0.397645947	0.690891187	0.999975616	ENSG00000107771	CCSER2
1181.499471	0.021784733	0.267751973	0.081361617	0.935154373	0.999975616	ENSG00000120063	GNA13
1159.593185	-0.061393917	0.18315016	-0.335210831	0.737466053	0.999975616	ENSG00000134318	ROCK2
1165.423741	0.228481623	0.331626161	0.688973457	0.490839971	0.999975616	ENSG00000178202	KDELC2
1161.28565	-0.130438629	0.211820461	-0.615798061	0.538027807	0.999975616	ENSG00000116521	SCAMP3
1168.417186	0.117124473	0.170825125	0.685639616	0.492940394	0.999975616	ENSG00000149136	SSRP1
1157.394255	-0.048030782	0.20213616	-0.237615981	0.812178955	0.999975616	ENSG00000064655	EYA2
1158.799931	0.066428345	0.193394097	0.343486933	0.73123217	0.999975616	ENSG00000125037	EMC3
1166.02656	-0.02546394	0.236916543	-0.107480635	0.914407688	0.999975616	ENSG00000259956	RBM15B
1153.875435	-0.054839456	0.218582148	-0.250887166	0.801901347	0.999975616	ENSG00000114902	SPCS1
1162.048569	-0.065221168	0.186794323	-0.349160332	0.726968945	0.999975616	ENSG00000023228	NDUFS1
1143.122022	-0.137231085	0.22813775	-0.601527302	0.547488833	0.999975616	ENSG00000112695	COX7A2
1150.41564	0.007905697	0.273368796	0.02891953	0.97692877	0.999975616	ENSG00000104870	FCGRT
1139.47126	0.078461884	0.35928695	0.218382227	0.827131312	0.999975616	ENSG00000251562	MALAT1
1150.987916	-0.16470417	0.169457298	-0.97195088	0.331074986	0.999975616	ENSG00000125818	PSMF1
1144.907857	0.028860639	0.225414524	0.128033624	0.898122364	0.999975616	ENSG00000105677	TMEM147
1153.832395	-0.003655266	0.168539862	-0.021687845	0.982696959	0.999975616	ENSG00000102531	FNDC3A
1150.320558	0.08088259	0.189182455	0.427537478	0.668987893	0.999975616	ENSG00000100568	VTI1B
1163.640908	0.08937529	0.249258861	0.358564145	0.719921174	0.999975616	ENSG00000013375	PGM3
1159.635004	0.202531312	0.320651106	0.631625179	0.52763183	0.999975616	ENSG00000181852	RNF41
1148.000543	0.022046759	0.160285769	0.137546577	0.89059878	0.999975616	ENSG00000148337	CIZ1
1146.916333	-0.187238164	0.166782077	-1.12265159	0.261585496	0.999975616	ENSG00000006715	VPS41
1144.152214	0.139217062	0.169266755	0.822471381	0.410808668	0.999975616	ENSG00000132383	RPA1
1152.756119	0.171206023	0.281083048	0.609094088	0.542462076	0.999975616	ENSG00000124207	CSE1L
1149.617988	-0.168896157	0.163036834	-1.035938646	0.300230767	0.999975616	ENSG00000211460	TSN
1129.574096	-0.429583629	0.254989715	-1.684709631	0.092044607	0.9781243	ENSG00000204941	PSG5
1116.654268	0.304052137	0.679776146	0.447282739	0.654670928	0.999975616	ENSG00000159251	ACTC1
1150.151471	-0.099651294	0.28592387	-0.348523871	0.72744679	0.999975616	ENSG00000143013	LMO4
1137.451591	0.102578893	0.20092959	0.510521584	0.609686097	0.999975616	ENSG00000142230	SAE1
1152.706558	-0.003715371	0.171885818	-0.021615343	0.982754794	0.999975616	ENSG00000109171	SLAIN2
1161.119589	0.436193934	0.294118314	1.483056012	0.13805953	0.999975616	ENSG00000148841	ITPRIP
1142.597423	-0.286472556	0.228011691	-1.256394157	0.208973099	0.999975616	ENSG00000178982	EIF3K
1151.786101	0.012780047	0.191813619	0.066627424	0.946878313	0.999975616	ENSG00000120137	PANK3
1134.168848	-0.102537178	0.214113084	-0.478892633	0.632015012	0.999975616	ENSG00000152818	UTRN
1142.51758	0.094423463	0.26647981	0.354336273	0.723086887	0.999975616	ENSG00000142327	RNPEPL1
1138.55234	-0.109926487	0.229273833	-0.479455005	0.631614971	0.999975616	ENSG00000185122	HSF1
1142.228141	0.021447829	0.185322991	0.115732154	0.907864823	0.999975616	ENSG00000158669	GPAT4
1150.609955	-0.230126727	0.315453145	-0.729511595	0.465688778	0.999975616	ENSG00000188157	AGRN
1142.400993	-0.179826771	0.197788733	-0.909186121	0.363251889	0.999975616	ENSG00000149100	EIF3M
1143.532819	-0.162026085	0.179037676	-0.904983179	0.365474299	0.999975616	ENSG00000164961	WASHC5
1140.023523	-0.017283387	0.192883103	-0.0896055	0.928600714	0.999975616	ENSG00000156471	PTDSS1
1140.807423	-0.135336326	0.199806087	-0.677338353	0.498191302	0.999975616	ENSG00000131626	PPFIA1
1142.301227	0.323765096	0.203535231	1.590707885	0.111675332	0.997355609	ENSG00000141279	NPEPPS
1135.61091	-0.145357593	0.211961911	-0.68577223	0.492856751	0.999975616	ENSG00000107959	PITRM1
1132.35325	0.08684428	0.169228484	0.513177676	0.60782704	0.999975616	ENSG00000134046	MBD2
1137.792935	0.283394885	0.253463631	1.118088951	0.263529005	0.999975616	ENSG00000155760	FZD7
1130.962379	-0.028715573	0.196485247	-0.146146206	0.883805971	0.999975616	ENSG00000105058	FAM32A
1114.002506	-0.407996858	0.290133292	-1.406239374	0.159653056	0.999975616	ENSG00000163431	LMOD1
1131.952436	0.177928781	0.192919598	0.922295003	0.35637472	0.999975616	ENSG00000140497	SCAMP2
1146.02418	-0.035085827	0.199245847	-0.176093141	0.860220769	0.999975616	ENSG00000047315	POLR2B
1129.006572	-0.045616769	0.235212185	-0.193938799	0.846223803	0.999975616	ENSG00000132842	AP3B1
1124.738972	0.076299586	0.208673521	0.365640957	0.714633009	0.999975616	ENSG00000165916	PSMC3
1122.770272	-0.164407769	0.232564173	-0.706935065	0.479606832	0.999975616	ENSG00000140464	PML
1127.489302	-0.084743856	0.176853407	-0.479175708	0.631813634	0.999975616	ENSG00000112658	SRF
1149.934775	0.297400866	0.342928693	0.86723821	0.385811505	0.999975616	ENSG00000187840	EIF4EBP1
1129.625297	-0.053190305	0.222384448	-0.239181766	0.810964641	0.999975616	ENSG00000113643	RARS
1129.954723	-0.274890124	0.26262641	-1.046696423	0.295239614	0.999975616	ENSG00000091140	DLD
1123.296596	-0.036175349	0.210993384	-0.171452528	0.863867955	0.999975616	ENSG00000137106	GRHPR
1139.584608	-0.107877762	0.265275436	-0.406663218	0.684255359	0.999975616	ENSG00000165655	ZNF503

1143.34492	0.211380624	0.261057436	0.80970926	0.418107295	0.999975616	ENSG00000133026	MYH10
1124.860753	-0.020928209	0.230121433	-0.090944198	0.927536931	0.999975616	ENSG00000165802	NSMF
1110.478081	0.10431169	0.265107079	0.393470028	0.693972357	0.999975616	ENSG00000182551	ADI1
1122.175323	0.077118017	0.230820558	0.334103763	0.738301261	0.999975616	ENSG00000109079	TNFAIP1
1114.857405	-0.068519988	0.223224134	-0.306956004	0.75887686	0.999975616	ENSG00000198258	UBL5
1138.262025	-0.263930311	0.2497767	-1.056665057	0.290664478	0.999975616	ENSG00000142892	PIGK
1149.804259	0.19607628	0.288393382	0.679891745	0.496573009	0.999975616	ENSG00000112773	TENT5A
1131.681021	0.065730084	0.176278074	0.372877256	0.7092398	0.999975616	ENSG00000010322	NISCH
1125.118895	-0.121806116	0.16428516	-0.741431035	0.458432133	0.999975616	ENSG00000185963	BICD2
1111.144314	0.107948068	0.229035778	0.471315306	0.637415587	0.999975616	ENSG00000174903	RAB1B
1123.800085	0.027989186	0.163994246	0.170671758	0.864481871	0.999975616	ENSG00000116030	SUMO1
1134.192761	0.222446963	0.201651299	1.103126854	0.269972081	0.999975616	ENSG00000165671	NSD1
1115.040918	0.127093981	0.237291534	0.535602679	0.592233177	0.999975616	ENSG00000167645	YIF1B
1116.023666	-0.177299222	0.212135125	-0.83578437	0.403276221	0.999975616	ENSG00000124333	VAMP7
1122.977578	0.028989662	0.158976981	0.182351315	0.85530703	0.999975616	ENSG00000015676	NUDCD3
1122.294025	0.155448331	0.167324804	0.92902144	0.352877975	0.999975616	ENSG00000158604	TMED4
1129.779038	-0.079948509	0.18853137	-0.424059449	0.671522458	0.999975616	ENSG00000013561	RNF14
1130.147727	1.41832311	0.769163254	1.843981888	0.06518578	0.937668618	ENSG00000114023	FAM162A
1132.422403	-0.050635468	0.214395486	-0.236177865	0.813294655	0.999975616	ENSG00000115464	USP34
1114.856975	-0.12921604	0.209918969	-0.615551995	0.538190243	0.999975616	ENSG00000168090	COPS6
1132.801501	0.023080896	0.171795156	0.134351259	0.893124822	0.999975616	ENSG00000136021	SCYL2
1136.740818	0.901764388	0.320459916	2.813969371	0.004893389	0.458380363	ENSG00000006652	IFRD1
1113.071699	-0.293545738	0.252301987	-1.163469783	0.244638951	0.999975616	ENSG00000128294	TPST2
1075.555091	-1.110280242	0.690474496	-1.607996021	0.10783605	0.991741545	ENSG00000145681	HAPLN1
1129.011872	-0.397915974	0.264622169	-1.503713674	0.132655107	0.999975616	ENSG00000182541	LIMK2
1121.337189	-0.11167992	0.168842183	-0.661445604	0.508326588	0.999975616	ENSG00000088930	XRN2
1112.508889	-0.267522876	0.225659961	-1.185513258	0.235814576	0.999975616	ENSG00000197496	SLC2A10
1112.393102	-0.022758075	0.200680732	-0.113404385	0.909709964	0.999975616	ENSG00000184304	PRKD1
1106.51444	-0.05715692	0.19248117	-0.29694811	0.766506125	0.999975616	ENSG00000149357	LAMTOR1
1155.627361	1.159112079	0.965085514	1.201045982	0.229733365	0.999975616	ENSG00000213859	KCTD11
1119.353	0.120954975	0.175598499	0.688815538	0.490939356	0.999975616	ENSG00000227500	SCAMP4
1123.404847	0.116519742	0.193529655	0.602076938	0.547122925	0.999975616	ENSG00000114062	UBE3A
1118.571853	-0.001445496	0.185521492	-0.007791528	0.993783323	0.999975616	ENSG00000242247	ARFGAP3
1110.342373	0.198451852	0.186776998	1.062506918	0.288005619	0.999975616	ENSG00000078668	VDAC3
1114.307578	0.163603828	0.269720533	0.606567941	0.544137681	0.999975616	ENSG00000107404	DVL1
1111.940811	-0.355266712	0.211187214	-1.682235892	0.092523107	0.9781243	ENSG00000102893	PHKB
1112.308051	-0.043787412	0.180668242	-0.242363634	0.80849841	0.999975616	ENSG00000161217	PCYT1A
1106.905764	0.578129905	0.262647806	2.201160231	0.027724683	0.768050664	ENSG00000130522	JUND
1110.586197	-0.16411425	0.175996885	-0.932483834	0.351086537	0.999975616	ENSG00000056586	RC3H2
1118.009484	0.102804425	0.188569942	0.545179279	0.585630222	0.999975616	ENSG00000023318	ERP44
1114.337462	-0.035602381	0.196284236	-0.181381766	0.85606793	0.999975616	ENSG00000185989	RASA3
1113.714736	0.493953864	0.305538644	1.616665759	0.105950449	0.987682951	ENSG00000103507	BCKDK
1129.752568	-0.147676812	0.256876217	-0.574894843	0.565362418	0.999975616	ENSG00000115758	ODC1
1127.166974	-0.527740475	0.313590647	-1.682896095	0.092395208	0.9781243	ENSG00000122176	FMOD
1112.709737	-0.248287865	0.209695443	-1.18404035	0.236397091	0.999975616	ENSG00000076067	RBMS2
1109.073769	0.055329269	0.183287402	0.301871639	0.762749916	0.999975616	ENSG00000047249	ATP6V1H
1097.570714	-0.245785124	0.23100778	-1.063969028	0.28734273	0.999975616	ENSG00000126267	COX6B1
1098.657278	0.073579335	0.190021307	0.387216236	0.698596131	0.999975616	ENSG00000163812	ZDHHC3
1106.900312	0.579593598	0.270660691	2.141402931	0.032241559	0.794279453	ENSG00000155508	CNOT8
1113.627643	0.179749883	0.193048496	0.931112575	0.351795334	0.999975616	ENSG00000078674	PCM1
1106.557609	0.046764532	0.178926633	0.261361494	0.793813749	0.999975616	ENSG00000108349	CASC3
1103.915168	-0.003532868	0.175797402	-0.020096245	0.983966595	0.999975616	ENSG00000134644	PUM1
1105.050508	0.151431382	0.16345942	0.926415755	0.35422996	0.999975616	ENSG00000136938	ANP32B
1105.92332	0.052857511	0.193795175	0.272749367	0.785045884	0.999975616	ENSG00000169905	TOR1AIP2
1123.106501	0.10362169	0.268801641	0.385495003	0.699870708	0.999975616	ENSG00000175727	MLXIP
1096.90559	-0.240591248	0.230847631	-1.042208001	0.297315254	0.999975616	ENSG00000180817	PPA1
1102.138644	0.077375213	0.159459129	0.485235391	0.62750938	0.999975616	ENSG00000205937	RNPS1
1113.612237	-0.292766865	0.176774919	-1.656156127	0.097690205	0.981485048	ENSG00000157216	SSBP3
1124.51339	3.83330535	1.185152651	3.234440176	0.001218815	0.269719843	ENSG00000163083	INHBB
1100.96976	-0.217732896	0.19097844	-1.140091501	0.254248183	0.999975616	ENSG00000101752	MIB1
1088.971519	0.24469911	0.209461608	1.168228928	0.242714435	0.999975616	ENSG00000180304	OAZ2
1093.180788	0.094655805	0.189226889	0.500223864	0.616917457	0.999975616	ENSG00000102898	NUTF2
1101.625182	0.009121168	0.19613011	0.046505702	0.96290719	0.999975616	ENSG00000117899	MESD
1103.628374	-0.254909214	0.382751416	-0.665991562	0.505416494	0.999975616	ENSG00000130066	SAT1

1094.242927	0.052806357	0.172550336	0.306034508	0.759578372	0.999975616	ENSG00000128463	EMC4
1100.202255	-0.095932063	0.218701344	-0.438644143	0.660919406	0.999975616	ENSG00000132466	ANKRD17
1093.588328	0.012549999	0.242677119	0.051714803	0.958755942	0.999975616	ENSG00000147955	SIGMAR1
1095.504652	-0.133690271	0.233257154	-0.573145428	0.56654623	0.999975616	ENSG00000165424	ZCCHC24
1101.793175	0.159397376	0.173903903	0.916583086	0.359361151	0.999975616	ENSG00000197045	GMFB
1097.845281	-0.047061498	0.17041668	-0.276155465	0.782428661	0.999975616	ENSG00000174238	PITPNA
1100.64804	0.255698385	0.213015442	1.200374875	0.229993782	0.999975616	ENSG00000179833	SERTAD2
1103.192738	-0.068167894	0.186463065	-0.365583898	0.714675592	0.999975616	ENSG00000153560	UBP1
1096.633397	-0.027774166	0.164708563	-0.168626124	0.866090731	0.999975616	ENSG00000126457	PRMT1
1100.176366	-0.199666023	0.154291231	-1.294085357	0.195635939	0.999975616	ENSG00000105176	URI1
1091.484492	-0.01075631	0.187929825	-0.05723578	0.954357377	0.999975616	ENSG00000001461	NIPAL3
1105.399914	-0.053717873	0.184645124	-0.290924945	0.771108723	0.999975616	ENSG00000275052	PPP4R3B
1085.210906	-0.554992184	0.303862187	-1.826460178	0.067780974	0.939405498	ENSG00000197448	GSTK1
1095.905813	0.08104968	0.204109113	0.397089965	0.69130112	0.999975616	ENSG00000168216	LMBRD1
1100.641115	0.084382641	0.19199954	0.439493977	0.660303645	0.999975616	ENSG00000110713	NUP98
1096.95001	0.070436694	0.157980035	0.445858201	0.655699678	0.999975616	ENSG00000139842	CUL4A
1091.075608	-0.010876517	0.192270117	-0.056568942	0.954888575	0.999975616	ENSG00000083799	CYLD
1109.824068	0.113701253	0.40903777	0.277972504	0.781033466	0.999975616	ENSG00000172164	SNTB1
1098.05769	0.344092788	0.281019616	1.224444019	0.220784772	0.999975616	ENSG00000068438	FTSJ1
1098.856732	-0.023003396	0.202400308	-0.113652971	0.909512894	0.999975616	ENSG00000145740	SLC30A5
1087.959927	-0.199276494	0.208783183	-0.954466214	0.339847703	0.999975616	ENSG00000131653	TRAF7
1100.54902	-0.019459261	0.184431198	-0.105509593	0.915971458	0.999975616	ENSG00000139505	MTMR6
1102.052822	-0.016464055	0.196248468	-0.083893929	0.933140766	0.999975616	ENSG00000126773	PCNX4
1085.10469	-0.016655775	0.176569974	-0.094329602	0.924847336	0.999975616	ENSG00000034713	GABARAPL2
1095.538656	0.232335494	0.205504375	1.130562273	0.258239374	0.999975616	ENSG00000173744	AGFG1
1092.949579	-5.01E-06	0.164006543	-3.06E-05	0.999975616	0.999975616	ENSG00000130227	XPO7
1097.065534	0.069825264	0.18304201	0.381471246	0.702853603	0.999975616	ENSG00000144566	RAB5A
1090.15603	-0.011129712	0.208642818	-0.053343373	0.957458323	0.999975616	ENSG00000101199	ARFGAP1
1086.97666	0.213429097	0.17138468	1.245321911	0.213013445	0.999975616	ENSG00000122707	RECK
1093.131329	0.141490425	0.179885633	0.786557674	0.431540845	0.999975616	ENSG00000124181	PLCG1
1090.193818	-0.01599735	0.200911225	-0.079623971	0.936536329	0.999975616	ENSG00000139324	TMTC3
1100.486549	-0.092888598	0.194719118	-0.477038921	0.633334407	0.999975616	ENSG00000132912	DCTN4
1090.351719	0.304850001	0.202865139	1.502722461	0.132910628	0.999975616	ENSG00000116857	TMEM9
1098.529848	0.019287954	0.252689487	0.076330654	0.939156038	0.999975616	ENSG00000070961	ATP2B1
1118.929876	-0.048687677	0.341427027	-0.142600535	0.886605675	0.999975616	ENSG00000105643	ARRDC2
1094.131736	-0.079468034	0.192035319	-0.413819888	0.679006018	0.999975616	ENSG00000141027	NCOR1
1096.290872	-0.086861916	0.196549951	-0.441933034	0.658537665	0.999975616	ENSG00000023516	AKAP11
1089.846448	0.038708806	0.212432053	0.182217352	0.855412156	0.999975616	ENSG00000143079	CTTNBP2NL
1084.851934	-0.590138453	0.261520001	-2.256571011	0.024034893	0.734505671	ENSG00000124813	RUNX2
1091.68067	0.01931685	0.19203955	0.100587872	0.919877624	0.999975616	ENSG00000072803	FBXW11
1078.022272	0.032752261	0.180351998	0.181601877	0.855895176	0.999975616	ENSG00000102882	MAPK3
1081.321641	-0.580805947	0.274762541	-2.113846908	0.034528345	0.803388455	ENSG00000106772	PRUNE2
1082.198552	-0.192423937	0.181293195	-1.061396362	0.288509809	0.999975616	ENSG00000187446	CHP1
1098.400886	0.041721753	0.305811839	0.13642949	0.891481762	0.999975616	ENSG00000177169	ULK1
1082.907172	-0.032403733	0.207029748	-0.156517281	0.875625299	0.999975616	ENSG00000108784	NAGLU
1078.167865	0.070935794	0.208732873	0.33984007	0.73397697	0.999975616	ENSG00000146425	DYNLT1
1082.170447	0.143712765	0.23442135	0.613053227	0.539841146	0.999975616	ENSG00000102054	RBBP7
1088.996452	0.010251451	0.233857347	0.043836342	0.965034858	0.999975616	ENSG00000108510	MED13
1090.418566	0.06342321	0.216191728	0.293365574	0.769242736	0.999975616	ENSG00000170471	RALGAPB
1087.762435	-0.232651722	0.194710187	-1.194861587	0.232141113	0.999975616	ENSG00000029725	RABEP1
1076.917845	-0.555300522	0.230356388	-2.410614817	0.015925658	0.66646844	ENSG00000146122	DAAM2
1074.619027	-0.344191402	0.188962412	-1.821480785	0.068533805	0.941152905	ENSG00000132356	PRKAA1
1083.47982	-0.127005071	0.214681768	-0.591596914	0.554120541	0.999975616	ENSG00000170921	TANC2
1087.163526	0.200289948	0.20515758	0.976273689	0.328928855	0.999975616	ENSG00000198492	YTHDF2
1092.430824	-0.534253853	0.250861988	-2.129672407	0.033198669	0.79736798	ENSG00000136848	DAB2IP
1073.259067	-0.10908966	0.204295685	-0.533979267	0.593355879	0.999975616	ENSG00000206560	ANKRD28
1067.905018	0.072811635	0.202507755	0.35954986	0.719183785	0.999975616	ENSG00000053254	FOXN3
1088.523462	0.032820706	0.224665	0.146087312	0.883852463	0.999975616	ENSG00000169641	LUZP1
1075.086605	-0.056732022	0.220461959	-0.257332476	0.796922124	0.999975616	ENSG00000120725	SIL1
1075.413219	-0.057888601	0.16598425	-0.348759599	0.727269797	0.999975616	ENSG00000111077	TNS2
1066.122297	-0.164584924	0.193335785	-0.851290534	0.394607982	0.999975616	ENSG00000165502	RPL36AL
1094.340678	0.144082589	0.259193174	0.555888827	0.578286857	0.999975616	ENSG00000166224	SGPL1
1079.330164	-0.007014566	0.204167059	-0.034356993	0.972592478	0.999975616	ENSG00000088888	MAVS
1073.366484	-0.164821387	0.197951377	-0.832635718	0.405050208	0.999975616	ENSG00000204673	AKT1S1

1067.878163	-0.099449711	0.175407696	-0.566963215	0.570739174	0.999975616	ENSG00000169895	SYAP1
1069.74181	0.213824263	0.241516505	0.88534017	0.375973191	0.999975616	ENSG00000089159	PXN
1055.278497	0.013769904	0.255805271	0.053829633	0.9570709	0.999975616	ENSG00000204580	DDR1
1069.110807	0.24925122	0.171662884	1.451980851	0.14650693	0.999975616	ENSG00000171302	CANT1
1075.779341	-0.06055486	0.18809638	-0.321935279	0.747501726	0.999975616	ENSG00000005700	IBTK
1079.974933	0.551560041	0.291061565	1.894994417	0.058093141	0.922712543	ENSG00000114120	SLC25A36
1060.034883	-0.077801102	0.213622573	-0.36419888	0.715709503	0.999975616	ENSG00000181061	HIGD1A
1066.758005	-0.097354567	0.211700119	-0.459870157	0.645609422	0.999975616	ENSG00000119314	PTBP3
1061.415225	-0.132241391	0.184500774	-0.716752498	0.473526827	0.999975616	ENSG00000176087	SLC35A4
1062.146418	-0.04282297	0.178858686	-0.239423485	0.810777221	0.999975616	ENSG00000136854	STXBP1
1068.717929	-0.251564366	0.171413661	-1.467586447	0.142216592	0.999975616	ENSG00000162521	RBBP4
1075.399195	0.422152091	0.291934231	1.446052042	0.148162606	0.999975616	ENSG00000107679	PLEKHA1
1076.045278	-0.086775212	0.207764411	-0.417661578	0.676194568	0.999975616	ENSG00000154305	MIA3
1061.844298	0.094814654	0.199061791	0.476307649	0.633855217	0.999975616	ENSG00000157538	VPS26C
1061.549555	0.060964472	0.197489205	0.308697742	0.757551462	0.999975616	ENSG00000166086	JAM3
1059.816831	-0.028614419	0.167107751	-0.171233344	0.86404029	0.999975616	ENSG00000100280	AP1B1
1057.773391	-0.211962553	0.205728591	-1.030301877	0.302868318	0.999975616	ENSG00000164023	SGMS2
1059.7536	-0.077092466	0.169139816	-0.455791354	0.648540022	0.999975616	ENSG00000013275	PSMC4
1061.857994	-0.414340381	0.186434668	-2.222442783	0.026253397	0.759780745	ENSG00000100412	ACO2
1059.349289	0.125706887	0.185304397	0.678380487	0.497530478	0.999975616	ENSG00000133961	NUMB
1050.413134	-0.141995661	0.318046284	-0.446462253	0.655263373	0.999975616	ENSG00000116729	WLS
1052.585471	-0.352940864	0.230605784	-1.530494413	0.125894395	0.999975616	ENSG00000173660	UQCRH
1054.359553	-0.318082703	0.282582487	-1.125627801	0.260323095	0.999975616	ENSG00000106080	FKBP14
1060.288114	-0.091242695	0.164294948	-0.555359104	0.57864906	0.999975616	ENSG00000198162	MAN1A2
1070.605444	-0.148337695	0.226939238	-0.653644984	0.513340555	0.999975616	ENSG00000153823	PID1
1066.694337	-0.013937484	0.223174062	-0.062451182	0.950203537	0.999975616	ENSG00000023287	RB1CC1
1060.559657	0.089868074	0.16368833	0.549019433	0.582992114	0.999975616	ENSG00000169139	UBE2V2
1054.215669	-0.237661634	0.187337038	-1.268631323	0.204572587	0.999975616	ENSG00000166847	DCTN5
1057.90002	0.206545575	0.324263278	0.636968749	0.5241452	0.999975616	ENSG00000165030	NFIL3
1057.43643	-0.099513911	0.160000187	-0.621961217	0.53396737	0.999975616	ENSG00000100220	RTCB
1060.174015	-0.290995792	0.260954084	-1.115122582	0.264797896	0.999975616	ENSG00000176170	SPHK1
1054.829734	0.427202217	0.227434749	1.878350687	0.060333207	0.92582322	ENSG00000109736	MFSD10
1057.032651	-0.083331859	0.222089692	-0.375217141	0.707498983	0.999975616	ENSG00000012232	EXTL3
1063.362311	-0.091521528	0.191469545	-0.477995223	0.632653605	0.999975616	ENSG00000066455	GOLGA5
1050.295188	0.565916751	0.284303844	1.990534995	0.046532033	0.877406326	ENSG00000123146	ADGRE5
1054.286686	0.596253293	0.248703582	2.397445536	0.016509835	0.66646844	ENSG00000198925	ATG9A
1054.671653	0.114505873	0.177235077	0.646067783	0.518235459	0.999975616	ENSG00000183576	SETD3
1054.667119	0.156497615	0.224509934	0.697063207	0.485763239	0.999975616	ENSG00000071564	TCF3
1061.651782	0.00729118	0.20768334	0.035107198	0.971994262	0.999975616	ENSG00000078747	ITCH
1047.684129	-0.285468796	0.218755537	-1.30496718	0.191904024	0.999975616	ENSG00000172053	QARS
1060.463473	0.349799868	0.253275418	1.381104693	0.167246773	0.999975616	ENSG00000130024	PHF10
1064.557994	0.153045424	0.225564705	0.678498989	0.497455365	0.999975616	ENSG00000092607	TBX15
1056.155828	0.025473486	0.187217538	0.136063568	0.891771028	0.999975616	ENSG00000166579	NDEL1
1034.570767	-0.09396046	0.236230912	-0.397748368	0.69081568	0.999975616	ENSG00000172725	CORO1B
1048.314278	0.324816804	0.256711025	1.265301343	0.205763325	0.999975616	ENSG00000118508	RAB32
1045.148466	-0.275652767	0.249883489	-1.103125176	0.269972809	0.999975616	ENSG00000164163	ABCE1
1044.929684	-0.261452368	0.222576366	-1.174663659	0.240129304	0.999975616	ENSG00000127920	GNG11
1068.481689	0.720078503	0.935586244	0.769654864	0.441504651	0.999975616	ENSG00000146477	SLC22A3
1044.854362	-0.264514247	0.187115793	-1.413639339	0.157467825	0.999975616	ENSG00000155463	OXA1L
1037.089346	0.251266256	0.217708594	1.15414027	0.248442666	0.999975616	ENSG00000107819	SFXN3
1045.383182	-0.240894134	0.352633971	-0.683127986	0.494525972	0.999975616	ENSG00000143127	ITGA10
1041.694807	-0.038435186	0.266540763	-0.14420003	0.885342517	0.999975616	ENSG00000026103	FAS
1052.402415	0.038722347	0.211399494	0.18317143	0.854663511	0.999975616	ENSG00000169446	MMGT1
1037.486846	-0.051702802	0.197494205	-0.261794022	0.793480248	0.999975616	ENSG00000115241	PPM1G
1053.753904	0.251522307	0.194660088	1.292110311	0.196318945	0.999975616	ENSG00000067955	CBFB
1040.347874	-0.345939238	0.206602582	-1.674418754	0.09404833	0.9781243	ENSG00000135390	ATP5MC2
1037.007258	-0.568460778	0.214276261	-2.652933998	0.007979547	0.513679125	ENSG00000163681	SLMAP
1044.986713	0.008934656	0.219820595	0.040645219	0.967578735	0.999975616	ENSG00000197386	HTT
1040.077897	0.155411426	0.154933251	1.00308633	0.31581921	0.999975616	ENSG00000093183	SEC22C
1049.622722	0.056038252	0.209446181	0.267554421	0.789042319	0.999975616	ENSG00000166503	HDGFL3
1051.63684	0.656924151	0.326958071	2.009199985	0.044515929	0.867769731	ENSG00000183258	DDX41
1024.997056	-0.047868746	0.262483813	-0.182368374	0.855293644	0.999975616	ENSG00000103363	ELOB
1045.548609	0.042843563	0.192952836	0.222041635	0.824281473	0.999975616	ENSG00000108039	XPNPEP1
1036.931255	0.173918975	0.203608851	0.854181799	0.393004267	0.999975616	ENSG00000004059	ARF5

1034.194944	0.10495534	0.213195759	0.492295626	0.622510372	0.999975616	ENSG00000101333	PLCB4
1039.827672	-0.491244846	0.264638819	-1.856284152	0.063413067	0.937668618	ENSG00000116406	EDEM3
1041.807803	0.061531603	0.212574772	0.289458632	0.772230432	0.999975616	ENSG00000138668	HNRNPD
1039.828116	-0.186116287	0.205383034	-0.906191146	0.364834689	0.999975616	ENSG00000111885	MAN1A1
1038.243961	0.063500079	0.206520565	0.307475817	0.758481227	0.999975616	ENSG00000171928	TVP23B
1038.228049	-0.389966672	0.255445762	-1.526612418	0.126857417	0.999975616	ENSG00000075391	RASAL2
1033.093478	-0.164538898	0.271590822	-0.605833793	0.544625127	0.999975616	ENSG00000172893	DHCR7
1035.550966	-0.320486057	0.242978487	-1.318989432	0.187172646	0.999975616	ENSG00000143924	EML4
1037.613594	-0.087242098	0.182153639	-0.47894787	0.631975715	0.999975616	ENSG00000119402	FBXW2
1028.962866	0.078719169	0.250469098	0.314286952	0.753303108	0.999975616	ENSG00000204520	MICA
1040.650429	-0.096888671	0.195902653	-0.494575591	0.620899735	0.999975616	ENSG00000205730	ITPRIPL2
1039.1913	0.770732596	0.240032391	3.210952456	0.001322958	0.274639877	ENSG00000102172	SMS
1034.64306	0.061731441	0.200821012	0.307395329	0.758542483	0.999975616	ENSG00000159692	CTBP1
1049.10163	0.903413839	0.312326351	2.892531603	0.003821507	0.432992068	ENSG00000020577	SAMD4A
1032.79522	-0.074758748	0.175055449	-0.427057533	0.669337423	0.999975616	ENSG00000108671	PSMD11
1031.050568	0.117963921	0.266029764	0.443423771	0.657459245	0.999975616	ENSG00000136378	ADAMTS7
1034.725456	-0.195727558	0.246905345	-0.792723053	0.427939196	0.999975616	ENSG00000158864	NDUFS2
1033.61461	-0.280471936	0.232059217	-1.208622263	0.226807999	0.999975616	ENSG00000114686	MRPL3
1035.121859	-0.137049423	0.17418341	-0.786811001	0.431392513	0.999975616	ENSG00000110768	GTF2H1
1036.833171	0.098714517	0.240521156	0.410419269	0.681498414	0.999975616	ENSG00000116157	GPX7
1034.11759	0.081475481	0.213378239	0.381835942	0.702583057	0.999975616	ENSG00000214078	CPNE1
1040.405533	-0.013265599	0.210634904	-0.062979112	0.949783137	0.999975616	ENSG00000196704	AMZ2
1044.95455	-0.122349639	0.262027679	-0.466934026	0.64054708	0.999975616	ENSG00000185070	FLRT2
1030.16292	-0.067059726	0.17490066	-0.38341608	0.701411282	0.999975616	ENSG00000090581	GNPTG
1029.452563	0.028979417	0.17988885	0.161096238	0.872017604	0.999975616	ENSG00000090316	MAEA
1027.224254	0.025190509	0.192758726	0.130684142	0.896025178	0.999975616	ENSG00000085832	EPS15
1031.365833	0.099757129	0.180415256	0.552930674	0.580310883	0.999975616	ENSG00000115233	PSMD14
1033.882774	-0.269843521	0.186550876	-1.446487558	0.148040499	0.999975616	ENSG00000115365	LANCL1
1029.627541	-0.017345396	0.17490529	-0.09917022	0.92100312	0.999975616	ENSG00000086065	CHMP5
1037.205672	-0.460226515	0.200400284	-2.296536241	0.021645239	0.693034246	ENSG00000065833	ME1
1031.265845	-0.002244418	0.170135015	-0.013191982	0.989474626	0.999975616	ENSG00000110048	OSBP
1033.665519	-0.086089439	0.201288946	-0.42769084	0.66887622	0.999975616	ENSG00000003056	M6PR
1024.161876	-0.023743357	0.247864121	-0.095791829	0.923685907	0.999975616	ENSG00000111670	GNPTAB
1029.474298	-0.169154464	0.174030837	-0.971979832	0.331060582	0.999975616	ENSG00000113845	TIMMDC1
1014.616762	0.325424994	0.240145802	1.355114229	0.175381166	0.999975616	ENSG00000184347	SLIT3
1028.47904	0.246508582	0.284478065	0.866529312	0.386199959	0.999975616	ENSG00000185567	AHNAK2
1027.724459	0.117754778	0.192461608	0.611835155	0.540646828	0.999975616	ENSG00000119048	UBE2B
1028.703791	0.092195929	0.165123806	0.558344257	0.576609329	0.999975616	ENSG00000198791	CNOT7
1021.17724	0.231359024	0.245936131	0.940728079	0.346844226	0.999975616	ENSG00000189067	LITAF
1038.034457	0.052165	0.206258653	0.252910602	0.800337297	0.999975616	ENSG00000197601	FAR1
991.7872429	0.958101181	0.399477994	2.398382875	0.016467642	0.66646844	ENSG00000154721	JAM2
1031.977936	-0.223283373	0.190004748	-1.175146278	0.239936199	0.999975616	ENSG00000167491	GATAD2A
1036.13102	0.110233693	0.292303978	0.37712006	0.706084385	0.999975616	ENSG00000186174	BCL9L
1027.762697	-0.410849576	0.182890165	-2.246427932	0.024676611	0.741369653	ENSG00000131374	TBC1D5
1025.913307	0.136634815	0.195692925	0.698210292	0.485045691	0.999975616	ENSG00000039523	RIPOR1
1026.942589	0.374063406	0.248227689	1.506936663	0.131826893	0.999975616	ENSG00000100106	TRIOBP
1053.079477	-0.008932883	0.334948056	-0.026669458	0.978723374	0.999975616	ENSG00000070371	CLTCL1
1023.508633	0.159309824	0.192239571	0.828704636	0.407271562	0.999975616	ENSG00000126461	SCAF1
1020.460864	-0.210351581	0.283318607	-0.742455933	0.457811141	0.999975616	ENSG00000123505	AMD1
1013.114066	0.478341481	0.247444791	1.933124069	0.053220916	0.903530068	ENSG00000003436	TFPI
1027.543323	0.186482771	0.250374789	0.744814489	0.456383875	0.999975616	ENSG00000143702	CEP170
1033.867753	0.139215285	0.259412605	0.536655823	0.591505377	0.999975616	ENSG00000100647	SUSD6
1020.110803	0.256424926	0.178643406	1.435401016	0.151172857	0.999975616	ENSG00000188986	NELFB
1020.415986	0.193339439	0.186092941	1.038940212	0.298832543	0.999975616	ENSG00000140350	ANP32A
1026.008561	-0.085617744	0.193052898	-0.443493702	0.657408674	0.999975616	ENSG00000102910	LONP2
1027.135625	0.312698228	0.207172043	1.509364992	0.131205534	0.999975616	ENSG00000213281	NRAS
1021.877823	0.082512753	0.184612573	0.446950883	0.654910524	0.999975616	ENSG00000136451	VEZF1
1014.872118	-0.51188529	0.279496855	-1.83145277	0.067032991	0.938628653	ENSG00000162144	CYB561A3
1009.823711	-0.350912844	0.221099333	-1.587127557	0.112483758	0.997355609	ENSG00000130811	EIF3G
1017.241385	0.300248449	0.227134368	1.321897921	0.186202145	0.999975616	ENSG00000124006	OBSL1
1010.007203	-0.255698131	0.220183841	-1.161293809	0.245522436	0.999975616	ENSG00000143420	ENSA
1020.386081	-0.05708081	0.19541799	-0.292095985	0.770213236	0.999975616	ENSG00000160877	NACC1
1017.558537	-0.742157665	0.24880036	-2.982944502	0.002854898	0.374056652	ENSG00000154277	UCHL1
1019.526032	0.142221372	0.185599957	0.766279123	0.443510238	0.999975616	ENSG00000137936	BCAR3

1014.335201	0.07374485	0.170214476	0.433246643	0.664835597	0.999975616	ENSG00000089351	GRAMD1A
1016.688317	0.234501244	0.207451773	1.130389203	0.258312262	0.999975616	ENSG00000137497	NUMA1
1013.739555	-0.102963048	0.239298288	-0.430270726	0.666998719	0.999975616	ENSG00000133275	CSNK1G2
1016.176145	-0.032733503	0.166343566	-0.196782503	0.843997744	0.999975616	ENSG00000099995	SF3A1
1008.197429	0.058181935	0.21602526	0.269329314	0.787676276	0.999975616	ENSG00000108819	PPP1R9B
1018.716352	1.082037512	0.7061137	1.532384249	0.125427642	0.999975616	ENSG00000275993	SIK1B
1011.372897	0.030179448	0.176820942	0.170678019	0.864476948	0.999975616	ENSG00000188529	SRSF10
1004.23802	-0.068660867	0.249049027	-0.27569217	0.78278451	0.999975616	ENSG00000140374	ETFA
1011.515447	-0.146790322	0.21692381	-0.676690685	0.498602228	0.999975616	ENSG00000178033	CALHM5
1014.363993	0.009578124	0.165540412	0.057859734	0.953860357	0.999975616	ENSG00000116906	GNPAT
1012.306483	0.000599492	0.161277865	0.003717139	0.997034159	0.999975616	ENSG00000111667	USP5
1014.233523	0.028996265	0.170817131	0.169750336	0.865206486	0.999975616	ENSG00000108883	EFTUD2
1016.110496	-0.098452928	0.203293837	-0.4842888	0.628180923	0.999975616	ENSG00000198836	OPA1
1014.6085	0.004941027	0.184141921	0.02683271	0.978593164	0.999975616	ENSG00000174574	AKIRIN1
1008.282554	-0.17660036	0.249463366	-0.707921015	0.478994308	0.999975616	ENSG00000175183	CSRP2
1016.642143	-0.103869252	0.175958393	-0.590305753	0.554985683	0.999975616	ENSG00000136485	DCAF7
1003.828631	0.007684717	0.194945656	0.039419793	0.968555699	0.999975616	ENSG00000240849	TMEM189
1019.846137	-0.394449787	0.258263433	-1.527315664	0.126682536	0.999975616	ENSG00000163629	PTPN13
1011.098418	-0.130907512	0.19374118	-0.675682433	0.499242291	0.999975616	ENSG00000092969	TGFB2
1013.833041	-0.023919367	0.16990759	-0.140778683	0.888044784	0.999975616	ENSG00000215717	TMEM167B
998.2068776	-0.15742536	0.237810618	-0.661977843	0.507985422	0.999975616	ENSG00000164405	UQCRQ
1017.80121	-0.152933608	0.179188306	-0.853479849	0.393393258	0.999975616	ENSG00000163785	RYK
1010.630597	0.019704046	0.19605268	0.100503833	0.91994434	0.999975616	ENSG00000130881	LRP3
1015.260354	-0.461929936	0.296629194	-1.557263905	0.119407841	0.999975616	ENSG00000122644	ARL4A
1018.374448	0.144206073	0.216794793	0.665173136	0.50593976	0.999975616	ENSG00000168137	SETD5
1006.307368	-0.140116735	0.241978171	-0.579047006	0.562557456	0.999975616	ENSG00000163378	EOGT
1000.55442	-0.114602106	0.205023003	-0.558971942	0.576180869	0.999975616	ENSG00000163444	TMEM183A
1005.542297	-0.099258082	0.251201771	-0.39513289	0.692744817	0.999975616	ENSG00000142784	WDTC1
1005.65493	0.548148345	0.256863263	2.134008337	0.032842098	0.796477121	ENSG00000139370	SLC15A4
1011.72553	-0.233685567	0.204509068	-1.142666039	0.253177275	0.999975616	ENSG00000138078	PREPL
1008.821965	-0.083189232	0.18871671	-0.440815401	0.659346643	0.999975616	ENSG00000204394	VARS
1005.840461	0.205070736	0.230356497	0.890232045	0.373341301	0.999975616	ENSG00000160445	ZER1
1001.56154	0.264346563	0.257612974	1.026138392	0.304826356	0.999975616	ENSG00000177666	PNPLA2
1003.575874	0.249995389	0.235906426	1.059722676	0.289270783	0.999975616	ENSG00000071655	MBD3
1003.522858	-0.02718103	0.162466406	-0.167302463	0.867132066	0.999975616	ENSG00000166233	ARIH1
1004.281427	-0.259491399	0.207496684	-1.250580946	0.211087406	0.999975616	ENSG00000104979	C19orf53
995.6268223	0.036946073	0.193546484	0.19088992	0.848611838	0.999975616	ENSG00000141552	ANAPC11
1011.577946	0.039264699	0.211592499	0.185567538	0.852783912	0.999975616	ENSG00000124201	ZNFX1
991.5687446	-0.057217699	0.217713295	-0.26281215	0.79269537	0.999975616	ENSG00000170043	TRAPPC1
1006.573635	-0.169888198	0.185025956	-0.918185761	0.358521622	0.999975616	ENSG00000169251	NMD3
1001.803139	-0.075314337	0.190423596	-0.395509478	0.692466928	0.999975616	ENSG00000196396	PTPN1
1014.407049	0.221136807	0.252967712	0.874170088	0.382025633	0.999975616	ENSG00000134575	ACP2
1011.686528	0.262045248	0.181503827	1.443745031	0.148810709	0.999975616	ENSG00000110330	BIRC2
1002.262424	0.087875353	0.187443323	0.468810257	0.639205268	0.999975616	ENSG00000160058	BSDC1
997.9614311	-0.255008671	0.23356156	-1.091826369	0.274909426	0.999975616	ENSG00000183741	CBX6
992.5874435	-0.163213236	0.19357327	-0.843159987	0.399138973	0.999975616	ENSG00000160211	G6PD
1003.197794	0.149652786	0.202319594	0.739685083	0.459491101	0.999975616	ENSG00000110047	EHD1
1009.632765	0.359893972	0.280628936	1.282454964	0.199683094	0.999975616	ENSG00000072310	SREBF1
1006.236103	-0.061869207	0.2065762	-0.299498233	0.76455992	0.999975616	ENSG00000109323	MANBA
984.5737037	-0.086427624	0.234723096	-0.36821099	0.712715916	0.999975616	ENSG00000128524	ATP6V1F
1004.443536	0.259998248	0.198607576	1.309105392	0.190498653	0.999975616	ENSG00000096746	HNRNPH3
990.954141	0.093134756	0.216181918	0.430816587	0.666601738	0.999975616	ENSG00000175334	BANF1
999.9989623	-0.309733492	0.207380144	-1.493554234	0.135292169	0.999975616	ENSG00000136478	TEX2
998.0693217	-0.195455428	0.170324351	-1.147548347	0.251155063	0.999975616	ENSG00000164209	SLC25A46
1004.368706	-0.015770308	0.235576495	-0.066943468	0.946626708	0.999975616	ENSG00000107263	RAPGEF1
1009.869409	0.000413835	0.220582306	0.001876102	0.998503088	0.999975616	ENSG00000100731	PCNX1
1006.756331	-0.159237666	0.203131592	-0.783913837	0.43309067	0.999975616	ENSG00000176853	FAM91A1
1006.031145	0.14648351	0.198475837	0.738042033	0.460488907	0.999975616	ENSG00000151553	FAM160B1
1001.962104	0.209930515	0.224552647	0.934883279	0.349848455	0.999975616	ENSG00000080822	CLDND1
997.327336	-0.101871025	0.247433626	-0.411710514	0.680551618	0.999975616	ENSG00000140743	CDR2
1010.143362	0.019806421	0.215949281	0.091717928	0.926922153	0.999975616	ENSG00000165006	UBAP1
994.4669348	-0.082187346	0.180819275	-0.454527572	0.649449152	0.999975616	ENSG00000170142	UBE2E1
999.145732	0.158383367	0.208601429	0.759263097	0.447695188	0.999975616	ENSG00000133606	MKRN1
999.9458652	0.601780007	0.32389575	1.857943511	0.063177037	0.937668618	ENSG00000167695	FAM57A

1000.967881	0.624255683	0.34816369	1.792994792	0.072973757	0.947863913	ENSG00000087074	PPP1R15A
988.6614039	0.12537172	0.173175537	0.723957445	0.469091861	0.999975616	ENSG00000140553	UNC45A
1000.272801	-0.054519767	0.209253848	-0.260543675	0.79444443	0.999975616	ENSG00000163349	HIPK1
1005.236423	-0.077493687	0.181413896	-0.427165114	0.669259069	0.999975616	ENSG00000180228	PRKRA
999.9413242	-0.35930741	0.244245684	-1.471090107	0.14126675	0.999975616	ENSG00000259330	INAFM2
1002.337946	0.054457736	0.219365004	0.248251703	0.803939665	0.999975616	ENSG00000113615	SEC24A
998.0735841	0.047340461	0.179136282	0.264270648	0.791571376	0.999975616	ENSG00000006831	ADIPOR2
1007.660538	-0.164793239	0.234774568	-0.701921166	0.482728328	0.999975616	ENSG00000166454	ATMIN
997.8101648	0.271879647	0.258494517	1.051781098	0.292899993	0.999975616	ENSG00000079432	CIC
1013.784605	-0.021277661	0.394586439	-0.053923954	0.956995752	0.999975616	ENSG00000112759	SLC29A1
994.7686425	5.02E-05	0.239165378	0.000209898	0.999832526	0.999975616	ENSG00000001036	FUCA2
997.1753534	-0.107544876	0.17554974	-0.61261769	0.540129159	0.999975616	ENSG00000131558	EXOC4
991.7138651	0.138919159	0.173775778	0.799416127	0.424049162	0.999975616	ENSG00000107175	CREB3
984.6280779	0.407084945	0.234068789	1.739167988	0.082005219	0.969369989	ENSG00000119899	SLC17A5
990.1471543	-0.026322677	0.176825622	-0.148862344	0.881662255	0.999975616	ENSG00000167778	SPRYD3
991.4219962	-0.090076982	0.221789677	-0.406136946	0.684641981	0.999975616	ENSG00000134905	CARS2
983.4958011	-0.462239154	0.29956324	-1.543043645	0.122820187	0.999975616	ENSG00000107798	LIPA
990.5800473	0.167958769	0.232122914	0.723576861	0.469325551	0.999975616	ENSG00000142002	DPP9
993.0497323	0.082244666	0.231686185	0.354983038	0.722602299	0.999975616	ENSG00000033627	ATP6V0A1
993.816073	-0.705476738	0.259010377	-2.723739283	0.006454744	0.488869185	ENSG00000149256	TENM4
995.2795543	0.32165929	0.318088168	1.011226833	0.311907876	0.999975616	ENSG00000139278	GLIPR1
987.4620987	0.025488891	0.189197788	0.13472087	0.892832572	0.999975616	ENSG00000166848	TERF2IP
987.1165819	0.149357823	0.185881562	0.803510693	0.421679619	0.999975616	ENSG00000149923	PPP4C
989.4750809	0.150977656	0.187934479	0.803352617	0.421770953	0.999975616	ENSG00000099956	SMARCB1
997.6749779	-2.35E-05	0.241269541	-9.72E-05	0.999922416	0.999975616	ENSG00000115295	CLIP4
983.353237	-0.093842511	0.254275072	-0.369059027	0.712083728	0.999975616	ENSG00000115107	STEAP3
981.35707	-0.093658141	0.210364324	-0.445218748	0.656161679	0.999975616	ENSG00000022277	RTF2
992.5369948	-0.016927212	0.246538843	-0.068659411	0.945260728	0.999975616	ENSG00000103043	VAC14
992.911251	-0.27311861	0.208110687	-1.312371867	0.189394694	0.999975616	ENSG00000169047	IRS1
1001.396753	-0.175707239	0.213444604	-0.82319832	0.410395224	0.999975616	ENSG00000141012	GALNS
991.2916191	-0.156852733	0.179820605	-0.872273417	0.383059236	0.999975616	ENSG00000018510	AGPS
983.0939588	0.112821925	0.225461107	0.500405265	0.616789747	0.999975616	ENSG00000099797	TECR
990.0071573	-0.065438249	0.205376173	-0.318626296	0.750009911	0.999975616	ENSG00000102858	MGRN1
981.9183144	0.029448026	0.208151422	0.141474057	0.887495453	0.999975616	ENSG00000179262	RAD23A
998.6849451	0.211534415	0.227653026	0.929196585	0.352787217	0.999975616	ENSG00000063978	RNF4
987.6948917	0.093987015	0.209785775	0.448014241	0.654142919	0.999975616	ENSG00000187231	SESTD1
985.7782945	0.078529311	0.167517179	0.468783631	0.639224302	0.999975616	ENSG00000155097	ATP6V1C1
1006.946702	0.032073215	0.265257852	0.120913347	0.90375967	0.999975616	ENSG00000170385	SLC30A1
990.1927365	0.00956499	0.223441762	0.042807531	0.96585496	0.999975616	ENSG00000057252	SOAT1
984.724575	-0.159350398	0.177674425	-0.896867395	0.36978968	0.999975616	ENSG00000143393	PI4KB
982.4389584	-0.054285799	0.21878934	-0.24811903	0.804042312	0.999975616	ENSG00000187210	GCNT1
991.3015126	-0.216065044	0.183975788	-1.174421083	0.240226405	0.999975616	ENSG00000142207	URB1
984.7435049	0.053767555	0.199707004	0.269232194	0.787751008	0.999975616	ENSG00000178252	WDR6
984.2291365	-0.062611309	0.18272777	-0.342648022	0.73186327	0.999975616	ENSG00000148634	HERC4
989.7333295	0.263697475	0.271381512	0.971685481	0.331207042	0.999975616	ENSG00000076513	ANKRD13A
986.7418962	-0.171350664	0.186804536	-0.917272501	0.358999863	0.999975616	ENSG00000140718	FTO
988.6323896	-0.136303983	0.163219732	-0.835095007	0.403664218	0.999975616	ENSG00000166068	SPRED1
989.9044556	-0.686970814	1.15063806	-0.59703467	0.550484234	0.999975616	ENSG00000132854	KANK4
984.6996709	0.066449944	0.165427534	0.401686121	0.68791504	0.999975616	ENSG00000104969	SGTA
977.0335378	0.318684317	0.304307999	1.047242656	0.294987676	0.999975616	ENSG00000173548	SNX33
985.9450056	0.244537672	0.183368565	1.333585566	0.182339716	0.999975616	ENSG00000090520	DNAJB11
986.5192092	0.117806551	0.183789535	0.640986175	0.521531666	0.999975616	ENSG00000116754	SRSF11
982.702312	0.074969116	0.15781479	0.475044931	0.634754948	0.999975616	ENSG00000149187	CELF1
977.7402789	-0.470823174	0.351419788	-1.339774222	0.180318759	0.999975616	ENSG00000189184	PCDH18
981.632006	0.033782409	0.157772143	0.214121505	0.830452306	0.999975616	ENSG00000103197	TSC2
980.6371878	0.105356001	0.172590143	0.610440429	0.541570094	0.999975616	ENSG00000137177	KIF13A
986.2973341	0.023522129	0.195194963	0.120505819	0.90408247	0.999975616	ENSG00000109133	TMEM33
1006.859011	-0.352367774	0.340504056	-1.034841636	0.300742875	0.999975616	ENSG00000234741	GAS5
983.738912	-0.008512753	0.255773365	-0.033282407	0.973449383	0.999975616	ENSG00000112210	RAB23
977.0729423	-0.231625859	0.189923487	-1.219574593	0.222626183	0.999975616	ENSG00000111832	RWDD1
1013.435829	-0.407874971	0.387511353	-1.052549734	0.292547405	0.999975616	ENSG00000099864	PALM
985.3879442	-0.039552108	0.205795787	-0.192191047	0.847592559	0.999975616	ENSG00000011451	WIZ
978.1598717	0.105812157	0.188420367	0.561574943	0.574405656	0.999975616	ENSG00000075790	BCAP29
963.1231154	-0.146520664	0.278110444	-0.526843443	0.598302312	0.999975616	ENSG00000087077	TRIP6

974.5509645	-0.143400978	0.185736039	-0.772068675	0.440073754	0.999975616	ENSG00000103018	CYB5B
992.9291437	0.209827635	0.305500865	0.686831557	0.492188881	0.999975616	ENSG00000127663	KDM4B
976.2192607	-0.088077111	0.158826671	-0.554548621	0.57920344	0.999975616	ENSG00000148120	C9orf3
974.3874454	-0.156446678	0.184113997	-0.849727237	0.395476752	0.999975616	ENSG00000196712	NF1
973.0403016	-0.037416775	0.206755366	-0.180971242	0.856390149	0.999975616	ENSG00000079999	KEAP1
979.8066752	-0.128863642	0.232349863	-0.554610362	0.5791612	0.999975616	ENSG00000165175	MID1IP1
976.5158142	0.17832295	0.181279997	0.983687956	0.325268963	0.999975616	ENSG00000124164	VAPB
975.0347619	0.529079927	0.213017016	2.483744907	0.01300089	0.622303364	ENSG00000139624	CERS5
976.1905676	0.328079204	0.170120389	1.92851195	0.053791479	0.903735138	ENSG00000100401	RANGAP1
970.0472234	0.06657592	0.221205142	0.300969133	0.763438032	0.999975616	ENSG00000106299	WASL
965.7786974	0.249268432	0.228016298	1.093204452	0.274304043	0.999975616	ENSG00000213853	EMP2
979.1387247	0.160531574	0.187561509	0.855887625	0.39205994	0.999975616	ENSG00000122299	ZC3H7A
967.5758145	-0.158364655	0.20104862	-0.787693321	0.430876113	0.999975616	ENSG00000099968	BCL2L13
970.4819524	0.028144828	0.3063734	0.091864464	0.926805726	0.999975616	ENSG00000006576	PHTF2
975.9040399	0.459891098	0.276676396	1.662198529	0.096472975	0.9781243	ENSG00000138764	CCNG2
965.8232719	-0.128628344	0.225820574	-0.569604185	0.568946189	0.999975616	ENSG00000133835	HSD17B4
971.0807848	-0.110844755	0.190944686	-0.580507146	0.561572669	0.999975616	ENSG00000151502	VPS26B
973.6261782	-0.087996175	0.1857311	-0.473782664	0.635654898	0.999975616	ENSG00000104131	EIF3J
969.0672681	0.125829561	0.156112361	0.806019204	0.420231776	0.999975616	ENSG00000107779	BMPR1A
960.7483768	0.183382067	0.205481169	0.892451936	0.372150744	0.999975616	ENSG00000167315	ACAA2
979.6723443	-0.17284297	0.327728025	-0.52739759	0.597917517	0.999975616	ENSG00000128590	DNAJB9
971.6328558	0.002890432	0.220355215	0.013117149	0.989534329	0.999975616	ENSG00000050130	JKAMP
972.5353775	0.0737499	0.166630825	0.44259458	0.658059006	0.999975616	ENSG00000163605	PPP4R2
967.2726162	-0.07532035	0.167489503	-0.449701913	0.652925392	0.999975616	ENSG00000114503	NCBP2
964.2194335	-0.156173033	0.181526388	-0.860332403	0.389605836	0.999975616	ENSG00000121067	SPOP
958.890856	-0.024567814	0.193466346	-0.126987534	0.898950265	0.999975616	ENSG00000169976	SF3B5
968.4621345	0.017019982	0.229999824	0.073999977	0.941010403	0.999975616	ENSG00000111145	ELK3
976.9486489	0.075757352	0.250426495	0.302513325	0.762260777	0.999975616	ENSG00000136048	DRAM1
970.2007088	-0.173976717	0.176471184	-0.985864735	0.324199494	0.999975616	ENSG00000164323	CFAP97
970.20378	-0.087566634	0.207365543	-0.422281506	0.672819555	0.999975616	ENSG00000181704	YIPF6
965.5166174	-0.001166993	0.176497841	-0.006611939	0.994724474	0.999975616	ENSG00000132153	DHX30
969.0790038	0.28964329	0.280389472	1.033003442	0.301602288	0.999975616	ENSG00000170153	RNF150
960.1090571	0.091252138	0.16756561	0.544575572	0.586045459	0.999975616	ENSG00000072958	AP1M1
960.1115575	-0.162751258	0.213477772	-0.762380347	0.445833032	0.999975616	ENSG00000130414	NDUFA10
954.5032554	-0.269173931	0.195500408	-1.376845881	0.168559902	0.999975616	ENSG00000186815	TPCN1
969.3202265	-0.202349848	0.190728177	-1.060933162	0.288720277	0.999975616	ENSG00000182606	TRAK1
971.0564695	0.034326286	0.262388744	0.13082225	0.895915922	0.999975616	ENSG00000102572	STK24
994.6988101	0.469395107	0.73296136	0.640409076	0.521906684	0.999975616	ENSG00000171246	NPTX1
951.9423251	0.001480641	0.203813363	0.00726469	0.994203667	0.999975616	ENSG00000105254	TBCB
967.4866247	-0.103846249	0.222024175	-0.467724962	0.639981288	0.999975616	ENSG00000151779	NBAS
963.968343	-0.040106881	0.192092206	-0.208789734	0.834612385	0.999975616	ENSG00000147162	OGT
964.6718065	0.057829567	0.234267978	0.246852206	0.805022606	0.999975616	ENSG00000154229	PRKCA
963.5665549	0.446538165	0.209161241	2.134899198	0.032769245	0.796477121	ENSG00000142599	RERE
961.6642604	0.216393777	0.238044967	0.909045797	0.363325952	0.999975616	ENSG00000054523	KIF1B
955.2571866	-0.028845952	0.175202539	-0.164643458	0.869224624	0.999975616	ENSG00000169727	GPS1
962.5341052	0.080602386	0.191944047	0.419926467	0.674539172	0.999975616	ENSG00000136536	7-Mar
960.0160455	0.026517075	0.185240005	0.143149829	0.886171853	0.999975616	ENSG00000197170	PSMD12
949.8061033	0.067717439	0.207038249	0.327076952	0.743609684	0.999975616	ENSG00000106628	POLD2
947.2001822	0.078863244	0.223817081	0.352355787	0.724571455	0.999975616	ENSG00000162627	SNX7
949.4487925	1.018630758	0.358575112	2.840773729	0.004500423	0.452053143	ENSG00000162496	DHRS3
951.5249223	0.031465543	0.175713694	0.179072799	0.85788054	0.999975616	ENSG00000174243	DDX23
959.4980162	0.111397468	0.214532899	0.519255871	0.603582322	0.999975616	ENSG00000188636	RTL6
953.358437	-0.205088917	0.222854207	-0.920282908	0.357424939	0.999975616	ENSG00000006282	SPATA20
966.9937937	0.423316595	0.294660794	1.436623413	0.150825027	0.999975616	ENSG00000108854	SMURF2
954.4290478	0.144638153	0.261932736	0.552195785	0.580814222	0.999975616	ENSG00000147274	RBMX
959.4168045	0.229087269	0.198789482	1.152411419	0.249152051	0.999975616	ENSG00000099904	ZDHHC8
956.9555019	-0.045506362	0.219405842	-0.207407249	0.835691821	0.999975616	ENSG00000085224	ATRX
955.2185804	-0.108276205	0.210230932	-0.515034606	0.606528838	0.999975616	ENSG00000154174	TOMM70
940.8049001	-0.051357961	0.300300199	-0.171022068	0.864206413	0.999975616	ENSG00000147224	PRPS1
952.6202532	-0.090833248	0.196184321	-0.462999528	0.643364705	0.999975616	ENSG00000100883	SRP54
941.6850416	0.032776958	0.182903968	0.179203099	0.85777823	0.999975616	ENSG00000134248	LAMTOR5
954.3208896	0.035424212	0.19489169	0.181763584	0.855768264	0.999975616	ENSG00000110911	SLC11A2
952.7451036	-0.200562893	0.22080465	-0.908327307	0.36370532	0.999975616	ENSG00000091436	MAP3K20
952.6368581	0.117511477	0.180018213	0.652775491	0.513901026	0.999975616	ENSG00000109501	WFS1

959.4197888	-0.219333932	0.217012421	-1.010697598	0.312161187	0.999975616	ENSG00000139726	DENR
951.4591656	0.090961437	0.185691793	0.48985168	0.624238857	0.999975616	ENSG00000068308	OTUD5
935.5109634	-0.155143755	0.277446324	-0.559184757	0.576035635	0.999975616	ENSG00000013583	HEBP1
954.7600701	-0.208142175	0.286057806	-0.727622775	0.466844535	0.999975616	ENSG00000012822	CALCOCO1
954.0074697	0.271037457	0.174979847	1.548963844	0.121390412	0.999975616	ENSG00000161904	LEMD2
946.0683425	-0.178317427	0.188037224	-0.948309187	0.342972075	0.999975616	ENSG00000169021	UQCRFS1
952.4491392	0.329125823	0.18122009	1.816166322	0.069344863	0.943613549	ENSG00000108468	CBX1
953.4880856	0.025206395	0.206245025	0.122215772	0.902728136	0.999975616	ENSG00000168246	UBTD2
952.0751125	-0.056007349	0.178183073	-0.314324744	0.753274407	0.999975616	ENSG00000180957	PITPNB
948.9980295	-0.187565797	0.164224194	-1.142132553	0.253398926	0.999975616	ENSG00000144895	EIF2A
951.7568628	-0.239188155	0.223233013	-1.07147304	0.283956787	0.999975616	ENSG00000213442	RPL18AP3
936.7751612	0.72787754	0.318844295	2.282862049	0.022438496	0.706214251	ENSG00000137331	IER3
948.0686008	0.049958688	0.18474205	0.270424021	0.786834062	0.999975616	ENSG00000139990	DCAF5
945.5431332	0.135836258	0.184994704	0.73427106	0.462783552	0.999975616	ENSG00000168958	MFF
944.7927369	-0.201137175	0.181447078	-1.108517027	0.26763859	0.999975616	ENSG00000113141	IK
954.9536063	0.221429452	0.312573702	0.708407173	0.478692439	0.999975616	ENSG00000134717	BTF3L4
949.2967548	0.021016794	0.174984725	0.120106451	0.904398822	0.999975616	ENSG00000112701	SENP6
946.5572565	0.062832963	0.171338456	0.366718392	0.713829086	0.999975616	ENSG00000134371	CDC73
904.4880864	1.628604208	0.428718955	3.798768842	0.000145417	0.098073135	ENSG00000073756	PTGS2
949.0487801	0.219128458	0.187297687	1.169947485	0.242022103	0.999975616	ENSG00000152223	EPG5
948.6558785	-0.013265056	0.187840019	-0.070618903	0.943701065	0.999975616	ENSG00000151923	TIAL1
956.3689241	0.511752183	0.393862107	1.299318149	0.193834769	0.999975616	ENSG00000140044	JDP2
943.4074423	-0.136775705	0.180818802	-0.756424125	0.449394948	0.999975616	ENSG00000170035	UBE2E3
941.7537397	0.036354664	0.19114116	0.190197991	0.849153986	0.999975616	ENSG00000103275	UBE2I
963.3831343	0.270336196	0.405980511	0.665884663	0.505484824	0.999975616	ENSG00000123095	BHLHE41
946.2793747	0.428942294	0.331867924	1.292509048	0.196180915	0.999975616	ENSG00000129474	AJUBA
941.3995623	0.117446873	0.237069865	0.495410384	0.620310467	0.999975616	ENSG00000120896	SORBS3
940.9182623	0.052126497	0.21018351	0.248004694	0.804130775	0.999975616	ENSG00000175826	CTDNEP1
937.7825743	-0.02326608	0.257860847	-0.090227269	0.928106615	0.999975616	ENSG00000131504	DIAPH1
944.3256984	0.100738257	0.18502312	0.544463075	0.586122851	0.999975616	ENSG00000132388	UBE2G1
938.8733751	-0.144994738	0.247297355	-0.586317384	0.557662251	0.999975616	ENSG00000087191	PSMC5
936.5897257	-0.099351819	0.199604395	-0.497743646	0.618664741	0.999975616	ENSG00000111412	C12orf49
935.6254614	-0.141523327	0.214332848	-0.660296958	0.509063282	0.999975616	ENSG00000196642	RABL6
940.9075109	0.152781638	0.167580626	0.911690341	0.36193175	0.999975616	ENSG00000144118	RALB
941.0803143	0.033627893	0.182584997	0.184176648	0.853874878	0.999975616	ENSG00000105576	TNPO2
948.1101298	0.126234337	0.20585002	0.613234512	0.539721288	0.999975616	ENSG00000141867	BRD4
941.8827231	0.024476482	0.257932985	0.094894735	0.924398438	0.999975616	ENSG00000109762	SNX25
937.1574888	-0.043861429	0.199421109	-0.219943762	0.825914953	0.999975616	ENSG00000264364	DYNLL2
935.4494196	0.089438378	0.216267235	0.413554918	0.679200095	0.999975616	ENSG00000115073	ACTR1B
941.2342047	-0.085326202	0.190225108	-0.448553836	0.653753542	0.999975616	ENSG00000107651	SEC23IP
950.9118174	-0.101828301	0.222069391	-0.458542716	0.646562581	0.999975616	ENSG00000141627	DYM
939.8007182	0.133606761	0.231597238	0.576892719	0.564011929	0.999975616	ENSG00000051620	HEBP2
933.0778303	0.446127324	0.186563358	2.391291235	0.016789229	0.670428193	ENSG00000023902	PLEKHO1
934.9869165	-0.207917848	0.252456484	-0.823578956	0.410178837	0.999975616	ENSG00000182667	NTM
941.6476413	0.248472864	0.217768563	1.140995102	0.253871961	0.999975616	ENSG00000133818	RRAS2
934.4129064	0.007018131	0.170155335	0.041245438	0.96710023	0.999975616	ENSG00000141543	EIF4A3
929.8200383	-0.227627888	0.220854625	-1.030668425	0.302696337	0.999975616	ENSG00000011454	RABGAP1
942.7273962	-0.027726555	0.18062951	-0.153499589	0.878004315	0.999975616	ENSG00000107290	SETX
952.8742288	0.702492108	0.392254565	1.790908689	0.073307949	0.947863913	ENSG00000166123	GPT2
934.2738467	-0.052981586	0.162225012	-0.326593199	0.743975588	0.999975616	ENSG00000125841	NRSN2
943.8932732	-0.427998777	0.30119277	-1.421012783	0.155313043	0.999975616	ENSG00000146374	RSPO3
933.6135318	-0.270383027	0.195685521	-1.381722196	0.167057016	0.999975616	ENSG00000093144	ECHDC1
940.834599	-0.027364694	0.177025643	-0.154580395	0.877152127	0.999975616	ENSG00000121644	DESI2
938.6004485	-0.067005974	0.169302981	-0.395775513	0.692270642	0.999975616	ENSG00000120008	WDR11
936.8487997	-0.059421373	0.17139506	-0.346692449	0.728822384	0.999975616	ENSG00000075711	DLG1
935.5247082	0.098720772	0.192006495	0.514153296	0.607144818	0.999975616	ENSG00000047410	TPR
929.3328349	-0.048554092	0.173942363	-0.279138968	0.780138179	0.999975616	ENSG00000148411	NACC2
922.9805654	-0.025767272	0.258865973	-0.099539044	0.920710289	0.999975616	ENSG00000135678	CPM
920.3552937	-0.089451202	0.226626115	-0.394708271	0.693058198	0.999975616	ENSG00000149932	TMEM219
934.7096747	0.019388977	0.16068989	0.120660836	0.90395968	0.999975616	ENSG00000085491	SLC25A24
940.027407	0.046707128	0.217622295	0.21462474	0.830059904	0.999975616	ENSG00000174227	PIGG
921.081396	-0.198833011	0.231817361	-0.857714067	0.39105037	0.999975616	ENSG00000168291	PDHB
927.2630593	0.043096627	0.260250284	0.165596851	0.868474226	0.999975616	ENSG00000101940	WDR13
932.3800362	0.038670272	0.158429436	0.244085147	0.807164876	0.999975616	ENSG00000130640	TUBGCP2

925.6141412	-0.045541496	0.172249809	-0.264392142	0.791477766	0.999975616	ENSG00000164062	APEH
939.8471796	0.222711353	0.187555055	1.187445218	0.235052053	0.999975616	ENSG00000136044	APPL2
932.0662054	-0.14978368	0.167418686	-0.894665248	0.370966061	0.999975616	ENSG00000134452	FBH1
922.49946	-0.388609357	0.24004951	-1.618871691	0.105474873	0.987682951	ENSG00000128923	MINDY2
922.4114769	-0.071207882	0.229171179	-0.310719185	0.756014112	0.999975616	ENSG00000175416	CLTB
922.3522566	0.099982451	0.191076866	0.523257749	0.600794899	0.999975616	ENSG00000135930	EIF4E2
934.3918534	0.008859191	0.18882157	0.046918319	0.962578328	0.999975616	ENSG00000172845	SP3
928.0963069	0.269787239	0.263171403	1.025138886	0.305297659	0.999975616	ENSG00000137216	TMEM63B
921.9193902	0.03280603	0.20674495	0.158678751	0.87392198	0.999975616	ENSG00000085449	WDFY1
928.8481277	-0.008369123	0.199942432	-0.041857662	0.966612167	0.999975616	ENSG00000105063	PPP6R1
925.2427111	-0.356001675	0.233021607	-1.527762508	0.126571514	0.999975616	ENSG00000167792	NDUFV1
931.7297586	0.020853828	0.193920357	0.107538106	0.914362097	0.999975616	ENSG00000163428	LRRC58
934.0999772	0.28863995	0.263997847	1.093342059	0.274243643	0.999975616	ENSG00000106638	TBL2
929.9095112	0.135665405	0.172134903	0.788134209	0.430618207	0.999975616	ENSG00000069345	DNAJA2
923.7011091	0.543504789	0.201235355	2.70084145	0.00691643	0.492649482	ENSG00000169213	RAB3B
927.1415912	-0.002693568	0.223865139	-0.012032102	0.990400004	0.999975616	ENSG00000087502	ERGIC2
930.3527389	0.323341744	0.202372524	1.597755163	0.110097477	0.995962615	ENSG00000164930	FZD6
917.8852092	-0.009727499	0.302458963	-0.032161384	0.974343352	0.999975616	ENSG00000132646	PCNA
926.4875419	-0.224726868	0.171552323	-1.309961087	0.190209	0.999975616	ENSG00000135966	TGFBRAP1
918.7826206	-0.055299291	0.192426069	-0.287379413	0.773821823	0.999975616	ENSG00000100138	SNU13
920.1838457	-0.384173086	0.202251249	-1.899484372	0.057500819	0.918331448	ENSG00000166619	BLCAP
924.0450117	-0.043570746	0.16697618	-0.260939888	0.794138864	0.999975616	ENSG00000101577	LPIN2
924.4910113	-0.041588538	0.232489766	-0.178883305	0.858029332	0.999975616	ENSG00000106683	LIMK1
935.3841129	-0.103371709	0.298308351	-0.346526367	0.728947173	0.999975616	ENSG00000179918	SEPHS2
923.0479118	0.094506597	0.170965121	0.552782911	0.580412071	0.999975616	ENSG00000135932	CAB39
921.638553	-0.268846333	0.227873311	-1.179806145	0.238077325	0.999975616	ENSG00000089472	HEPH
928.2473133	0.108399768	0.183642305	0.590276668	0.555005179	0.999975616	ENSG00000120690	ELF1
923.1714418	-0.118452109	0.175511078	-0.674898192	0.499740449	0.999975616	ENSG00000134759	ELP2
920.1362172	0.325019658	0.223584081	1.453679781	0.146035106	0.999975616	ENSG00000104756	KCTD9
916.8624488	-0.172804284	0.212414866	-0.813522553	0.415918521	0.999975616	ENSG00000152484	USP12
922.1490112	0.173462804	0.211167683	0.821445786	0.411392393	0.999975616	ENSG00000186635	ARAP1
922.7343555	-0.217538922	0.177700882	-1.22418594	0.220882092	0.999975616	ENSG00000128731	HERC2
934.299968	0.149489989	0.278929591	0.535941664	0.591998868	0.999975616	ENSG00000131149	GSE1
922.8822676	-0.14525447	0.182246372	-0.797022558	0.42543793	0.999975616	ENSG00000198900	TOP1
926.2536564	0.885528118	0.694079391	1.275831166	0.202015208	0.999975616	ENSG00000187957	DNER
943.0346881	0.279794386	0.391772715	0.714175274	0.475118804	0.999975616	ENSG00000172432	GTPBP2
914.9443614	-0.060529105	0.200626377	-0.301700631	0.762880288	0.999975616	ENSG00000141503	MINK1
925.060823	0.062925267	0.186683356	0.33706951	0.736064501	0.999975616	ENSG00000125827	TMX4
923.7779377	-0.104395445	0.260387378	-0.4009236	0.688476374	0.999975616	ENSG00000153814	JAZF1
936.1748135	0.074350626	0.398320892	0.186660122	0.851927125	0.999975616	ENSG00000081052	COL4A4
919.9249107	-0.040042896	0.172845069	-0.231669298	0.816794879	0.999975616	ENSG00000163636	PSMD6
921.8208718	-0.057950499	0.218095287	-0.265711834	0.790461148	0.999975616	ENSG00000131263	RLIM
917.5332517	0.060711389	0.163125855	0.372175147	0.709762449	0.999975616	ENSG00000074319	TSG101
914.9763328	-0.112130022	0.20139453	-0.556767961	0.577685977	0.999975616	ENSG00000141349	G6PC3
911.3723211	-0.140331036	0.212538713	-0.660261059	0.509086315	0.999975616	ENSG00000172500	FIBP
918.8465924	-0.129523611	0.21540732	-0.601296237	0.547642695	0.999975616	ENSG00000038274	MAT2B
917.3754289	0.02239753	0.181940707	0.123103457	0.902025174	0.999975616	ENSG00000100614	PPM1A
912.2662416	0.21660598	0.209046389	1.036162262	0.300126449	0.999975616	ENSG00000120948	TARDBP
921.7783587	0.54336895	0.269183721	2.018580275	0.043530863	0.860814587	ENSG00000163872	YEATS2
939.6825745	0.553866051	0.403770471	1.371734911	0.170145976	0.999975616	ENSG00000130766	SESN2
908.4219429	-0.102830131	0.190973103	-0.538453477	0.590264016	0.999975616	ENSG00000141759	TXNL4A
905.3699165	-0.133396111	0.212652704	-0.627295628	0.530465469	0.999975616	ENSG00000130724	CHMP2A
901.4846344	0.00026313	0.262395179	0.001002802	0.99919988	0.999975616	ENSG00000132589	FLOT2
919.5378062	0.283904347	0.425875377	0.666637149	0.505003934	0.999975616	ENSG00000183671	GPR1
902.0270191	0.008590069	0.227680924	0.037728543	0.969904118	0.999975616	ENSG00000142507	PSMB6
913.1808129	-0.096661341	0.179856951	-0.537434557	0.590967478	0.999975616	ENSG00000135341	MAP3K7
904.2277542	0.005209047	0.214092147	0.024330865	0.980588694	0.999975616	ENSG00000138867	GUCD1
900.5202294	-0.048973954	0.244062352	-0.200661648	0.84096315	0.999975616	ENSG00000103381	CPPED1
914.3373549	-0.096886039	0.953807603	-0.101578178	0.919091501	0.999975616	ENSG00000125968	ID1
909.9984977	0.162034038	0.167919846	0.964948707	0.334570504	0.999975616	ENSG00000143727	ACP1
903.1660761	-0.048811063	0.203499177	-0.239858773	0.810439742	0.999975616	ENSG00000100364	KIAA0930
915.0823915	0.356955188	0.172786783	2.065870903	0.038840671	0.848069004	ENSG00000270647	TAF15
909.2376625	0.074646132	0.208304149	0.358351634	0.720080182	0.999975616	ENSG00000108010	GLRX3
905.5391983	0.333252061	0.176285661	1.890409344	0.058703235	0.922712543	ENSG00000005486	RHBDD2

905.3968676	-0.163269837	0.202183086	-0.807534598	0.41935855	0.999975616	ENSG00000179091	CYC1
898.2448828	-0.033514242	0.232566647	-0.144105968	0.885416792	0.999975616	ENSG00000117448	AKR1A1
907.4546038	-0.428126585	0.210779607	-2.031157523	0.042239018	0.855568497	ENSG00000108091	CCDC6
910.61171	0.048563003	0.183458046	0.26470904	0.791233613	0.999975616	ENSG00000112339	HBS1L
909.8211844	-0.225493508	0.176253001	-1.279374001	0.200765385	0.999975616	ENSG00000103544	VPS35L
916.2187381	-0.078395807	0.225623078	-0.347463598	0.728243061	0.999975616	ENSG00000213064	SFT2D2
908.2565393	-0.073120922	0.185762648	-0.393625538	0.693857523	0.999975616	ENSG00000274523	RCC1L
918.8990248	0.202860724	0.245511483	0.826277949	0.408646439	0.999975616	ENSG00000100813	ACIN1
914.9992424	0.13947602	0.236260736	0.590347859	0.554957459	0.999975616	ENSG00000100393	EP300
903.6924055	0.129495807	0.20261639	0.639118126	0.522746089	0.999975616	ENSG00000078269	SYNJ2
952.784989	-0.138640247	0.317926858	-0.436075919	0.662781649	0.999975616	ENSG00000179954	SSC5D
900.3276127	0.096453969	0.191531636	0.503592882	0.614547496	0.999975616	ENSG00000160948	VPS28
905.9326971	0.253437803	0.250558942	1.011489756	0.311782082	0.999975616	ENSG00000111859	NEDD9
915.2710372	-0.100074293	0.18230429	-0.548940963	0.583045965	0.999975616	ENSG00000167635	ZNF146
898.7036486	-0.121182844	0.257853403	-0.469967986	0.63837789	0.999975616	ENSG00000198715	GLMP
900.673683	0.319075492	0.204540672	1.559961105	0.118769073	0.999975616	ENSG00000071626	DAZAP1
902.6389456	0.180834564	0.211162494	0.856376343	0.391789646	0.999975616	ENSG00000184887	BTBD6
901.8465923	0.162623069	0.20584783	0.79001595	0.429518453	0.999975616	ENSG00000142687	KIAA0319L
901.3184192	-0.107699579	0.184406382	-0.584033901	0.559197504	0.999975616	ENSG00000271601	LIX1L
900.95557	-0.236085289	0.226602039	-1.041849797	0.297481323	0.999975616	ENSG00000107521	HPS1
908.1069932	0.368795509	0.292173075	1.262250155	0.206858786	0.999975616	ENSG00000157985	AGAP1
909.9356841	0.143644984	0.196272414	0.731865375	0.464250742	0.999975616	ENSG00000052841	TTC17
915.8307597	-0.258734948	0.395586224	-0.654054496	0.513076697	0.999975616	ENSG00000130513	GDF15
901.7587271	-0.239823224	0.182046538	-1.317373164	0.187713572	0.999975616	ENSG00000092108	SCFD1
912.6600032	0.038790837	0.33879284	0.114497216	0.908843653	0.999975616	ENSG00000138942	RNF185
892.8563895	-0.158084895	0.227061015	-0.696222095	0.486289753	0.999975616	ENSG00000167863	ATP5PD
899.0595307	-0.066669646	0.213196584	-0.312714421	0.754497638	0.999975616	ENSG00000100632	ERH
904.7701256	-0.052487836	0.203751324	-0.257607335	0.796709968	0.999975616	ENSG00000124209	RAB22A
897.524407	-0.044723035	0.183717023	-0.243434355	0.807668933	0.999975616	ENSG00000162517	PEF1
899.7450105	-0.280767668	0.215339032	-1.303840116	0.192288102	0.999975616	ENSG00000168936	TMEM129
897.9656342	0.468505318	0.233315795	2.008030871	0.044640011	0.867769731	ENSG00000153395	LPCAT1
900.9354777	0.025414873	0.167214617	0.15198954	0.879195185	0.999975616	ENSG00000009844	VTA1
902.7313515	-0.28774076	0.222794978	-1.291504693	0.196528729	0.999975616	ENSG00000236552	RPL13AP5
891.1776932	-0.113150975	0.262508931	-0.431036667	0.666441709	0.999975616	ENSG00000173915	ATP5MD
901.0786394	0.333817061	0.263325774	1.267696114	0.204906493	0.999975616	ENSG00000175137	SH3BP5L
911.8248276	-0.147258924	0.288783968	-0.509927628	0.610102165	0.999975616	ENSG00000238227	TMEM250
899.8447744	-0.0170916	0.161283568	-0.105972355	0.915604286	0.999975616	ENSG00000150760	DOCK1
957.2090541	1.312647362	0.437373464	3.00120485	0.002689136	0.366311929	ENSG00000157766	ACAN
897.5642122	0.11182069	0.177278543	0.630762688	0.528195703	0.999975616	ENSG00000145782	ATG12
896.2427939	0.034470906	0.218452315	0.157796021	0.874617534	0.999975616	ENSG00000130725	UBE2M
895.6346671	0.16134232	0.29515439	0.546637033	0.584628121	0.999975616	ENSG00000161547	SRSF2
900.3827478	0.557124123	0.251859841	2.21204032	0.026963879	0.768050664	ENSG00000100968	NFATC4
885.2617144	0.15381902	0.220732475	0.696857227	0.485892148	0.999975616	ENSG00000243927	MRPS6
885.0561229	-0.077686603	0.247142982	-0.314338698	0.75326381	0.999975616	ENSG00000106400	ZNHIT1
892.5062487	-0.456176924	0.260839883	-1.748877198	0.080312249	0.965624825	ENSG00000136521	NDUFB5
874.9202778	-0.670833013	0.391497862	-1.713503645	0.086619923	0.974422538	ENSG00000109686	SH3D19
913.1646023	0.133413427	0.294758635	0.452619233	0.650822947	0.999975616	ENSG00000096433	ITPR3
884.2311031	0.750581162	0.849833895	0.883209256	0.377123221	0.999975616	ENSG00000117632	STMN1
900.4002988	-0.008400551	0.204427383	-0.041093079	0.967221692	0.999975616	ENSG00000144357	UBR3
897.0109609	0.062575429	0.173534741	0.360593093	0.718403653	0.999975616	ENSG00000142453	CARM1
896.5940809	0.058351844	0.188961803	0.308802325	0.757471901	0.999975616	ENSG00000106261	ZKSCAN1
910.9074391	0.146639029	0.273844827	0.535482194	0.592316467	0.999975616	ENSG00000153885	KCTD15
934.5432377	0.488265254	0.476738282	1.024178827	0.305750818	0.999975616	ENSG00000134198	TSPAN2
896.8937775	-0.082461341	0.159599197	-0.51667767	0.605381187	0.999975616	ENSG00000102699	PARP4
900.1852593	0.138806616	0.210913808	0.6581201	0.510460961	0.999975616	ENSG00000135317	SNX14
894.8259914	-0.014297609	0.214630953	-0.06661485	0.946888323	0.999975616	ENSG00000114999	TTL
921.4679505	-0.284746825	0.34546526	-0.824241561	0.409802316	0.999975616	ENSG00000130707	ASS1
896.6991646	0.027553301	0.182320014	0.151126036	0.879876294	0.999975616	ENSG00000070770	CSNK2A2
885.4980536	-0.0179762	0.204666728	-0.087831568	0.930010547	0.999975616	ENSG00000108528	SLC25A11
895.4306047	0.138388678	0.213366223	0.648596934	0.516598938	0.999975616	ENSG00000185359	HGS
894.2234362	-0.008842076	0.176814207	-0.050007724	0.960116233	0.999975616	ENSG00000090372	STRN4
894.3479343	-0.014044751	0.191723307	-0.073255313	0.941602951	0.999975616	ENSG00000084463	WBP11
890.8142922	-0.260027235	0.165983504	-1.566584803	0.117211772	0.999975616	ENSG00000184787	UBE2G2
891.4688421	-0.106872618	0.208026893	-0.513744238	0.607430818	0.999975616	ENSG00000064419	TNPO3

887.6513532	-0.011856039	0.198865243	-0.059618457	0.952459518	0.999975616	ENSG00000090273	NUDC
895.3896939	-0.059400693	0.189464349	-0.313519104	0.753886309	0.999975616	ENSG00000119541	VPS4B
885.7074832	0.010379075	0.19118771	0.054287356	0.956706223	0.999975616	ENSG00000083937	CHMP2B
895.4460985	-0.107555845	0.225025655	-0.477971477	0.632670506	0.999975616	ENSG00000100815	TRIP11
890.5140078	-0.321716625	0.248377715	-1.295271701	0.195226518	0.999975616	ENSG00000232573	RPL3P4
876.383097	0.018501096	0.288254085	0.064183291	0.948824282	0.999975616	ENSG00000180891	CUEDC1
901.846138	-0.372398594	0.221044346	-1.68472345	0.09204194	0.9781243	ENSG00000023909	GCLM
898.1808439	0.132105291	0.27556521	0.479397564	0.631655826	0.999975616	ENSG00000123094	RASSF8
879.1571222	-0.185493964	0.207273551	-0.894923464	0.370828003	0.999975616	ENSG00000198356	ASNA1
885.4493802	0.124766608	0.190361843	0.655418153	0.512198567	0.999975616	ENSG00000204619	PPP1R11
886.816385	0.276078716	0.193845827	1.424217997	0.154383377	0.999975616	ENSG00000164466	SFXN1
889.2261143	0.055219395	0.240370219	0.229726442	0.818304347	0.999975616	ENSG00000118263	KLF7
886.2209729	0.14083618	0.259403257	0.542923716	0.587182326	0.999975616	ENSG00000173281	PPP1R3B
899.5466696	0.090435372	0.253494898	0.356754208	0.72127582	0.999975616	ENSG00000127870	RNF6
863.0825982	0.590850619	0.284653657	2.075682512	0.037923324	0.83661689	ENSG00000108389	MTMR4
875.7590532	-0.140653073	0.180064911	-0.78112427	0.434729409	0.999975616	ENSG00000145050	MANF
886.059106	0.061177123	0.225978312	0.270721215	0.786605458	0.999975616	ENSG00000173020	GRK2
885.2734892	0.429704102	0.34415979	1.248559866	0.211826099	0.999975616	ENSG00000176720	BOK
878.7153773	0.218018025	0.223874929	0.973838502	0.330136733	0.999975616	ENSG00000139433	GLTP
889.6682891	0.137860187	0.18951231	0.727447135	0.466952089	0.999975616	ENSG00000090432	MUL1
879.733701	-0.104550769	0.186480568	-0.560652352	0.575034555	0.999975616	ENSG00000078804	TP53INP2
873.8526663	-0.066794296	0.21902022	-0.304968626	0.760390048	0.999975616	ENSG00000277791	PSMB3
886.1467099	-0.082023093	0.193775866	-0.423288486	0.672084794	0.999975616	ENSG00000107164	FUBP3
888.2932249	0.261395601	0.335173202	0.779882162	0.435460239	0.999975616	ENSG00000037637	FBXO42
877.5359215	0.232201743	0.316148576	0.734470311	0.462662148	0.999975616	ENSG00000146278	PNRC1
885.3298449	-0.084123713	0.180828023	-0.46521392	0.641778271	0.999975616	ENSG00000138814	PPP3CA
884.0551296	-0.065309872	0.186098468	-0.350942555	0.725631445	0.999975616	ENSG00000124198	ARFGEF2
887.3718515	0.240821479	0.224772452	1.071401217	0.283989066	0.999975616	ENSG00000161091	MFSD12
882.9521063	0.161572599	0.200207597	0.807025313	0.419651899	0.999975616	ENSG00000164944	VIRMA
875.5535794	-0.061400608	0.234688583	-0.261625884	0.793609886	0.999975616	ENSG00000265354	TIMM23
880.4182387	-0.007526579	0.251856665	-0.029884375	0.976159268	0.999975616	ENSG00000071051	NCK2
878.2414299	-0.22903943	0.190927624	-1.199613893	0.230289329	0.999975616	ENSG00000105939	ZC3HAV1
875.6634594	-0.092932933	0.191419185	-0.48549435	0.627325719	0.999975616	ENSG00000247556	OIP5-AS1
873.7114611	0.086260256	0.301796857	0.285822249	0.775014266	0.999975616	ENSG00000137267	TUBB2A
876.8185429	-0.023215295	0.169615986	-0.136869737	0.89113376	0.999975616	ENSG00000101161	PRPF6
881.1474067	-0.033876376	0.213475622	-0.158689672	0.873913376	0.999975616	ENSG00000185591	SP1
882.6931019	-0.102804182	0.316644394	-0.32466762	0.745432644	0.999975616	ENSG00000197063	MAFG
870.7193567	-0.085226845	0.179400908	-0.475063622	0.634741627	0.999975616	ENSG00000054116	TRAPPC3
877.9618801	-0.026633756	0.191135107	-0.139345182	0.889177388	0.999975616	ENSG00000011405	PIK3C2A
871.4750326	0.302000524	0.199655225	1.512610169	0.13037871	0.999975616	ENSG00000116350	SRSF4
892.9385872	0.275825665	0.403599564	0.683414179	0.494345163	0.999975616	ENSG00000099875	MKNK2
879.1801323	0.017144798	0.195668913	0.087621474	0.930177534	0.999975616	ENSG00000154001	PPP2R5E
878.8388424	-0.059764545	0.201949275	-0.2959384	0.767277125	0.999975616	ENSG00000143324	XPR1
864.5949154	-0.093923599	0.217324219	-0.432181924	0.665609196	0.999975616	ENSG00000106367	AP1S1
872.237172	-0.103860951	0.199154847	-0.521508525	0.602012568	0.999975616	ENSG00000011007	ELOA
876.1225022	-0.167829211	0.195243497	-0.859589249	0.390015503	0.999975616	ENSG00000198793	MTOR
869.5640405	-0.602725048	0.336875591	-1.789162126	0.073588709	0.947863913	ENSG00000196139	AKR1C3
890.4800941	0.51456509	0.326399976	1.576486302	0.114913773	0.99848084	ENSG00000075275	CELSR1
866.0476456	-0.095264137	0.213144954	-0.446945309	0.654914549	0.999975616	ENSG00000126088	UROD
864.1008265	-0.021827797	0.251070701	-0.086938844	0.930720123	0.999975616	ENSG00000225663	MCRIP1
863.810762	-1.198197637	0.446286803	-2.684815303	0.00725699	0.501369489	ENSG00000196562	SULF2
874.9264873	0.222206358	0.268322742	0.828130913	0.407596364	0.999975616	ENSG00000073792	IGF2BP2
867.6940382	-0.075411027	0.199348389	-0.378287617	0.705216944	0.999975616	ENSG00000147164	SNX12
885.0329831	0.047005446	0.250989726	0.18728036	0.851440821	0.999975616	ENSG00000176624	MEX3C
866.7038861	0.048671478	0.19807619	0.245720993	0.80589822	0.999975616	ENSG00000242372	EIF6
863.6114484	-0.011821413	0.257522878	-0.045904321	0.96338651	0.999975616	ENSG00000129968	ABHD17A
871.8815417	-0.044982668	0.165823866	-0.271267756	0.786185104	0.999975616	ENSG00000125755	SYMPK
874.0141211	-0.027210757	0.172059482	-0.158147386	0.874340662	0.999975616	ENSG00000173950	XXYLT1
882.8703266	-0.68243424	0.27759055	-2.458420288	0.013954975	0.635512264	ENSG00000100342	APOL1
870.1127408	0.143514243	0.198328642	0.723618342	0.469300077	0.999975616	ENSG00000108523	RNF167
869.8614419	-0.056193238	0.166352297	-0.337796585	0.735516483	0.999975616	ENSG00000138593	SECISBP2L
880.6441785	-0.340335779	0.255600323	-1.331515447	0.183019466	0.999975616	ENSG00000125826	RBCK1
872.2245366	0.14749147	0.228334585	0.645944503	0.518315297	0.999975616	ENSG00000153066	TXNDC11
874.9618089	0.442536007	0.385319783	1.148490232	0.250766243	0.999975616	ENSG00000145555	MYO10

866.067484	-0.19311259	0.192767592	-1.001789707	0.316445169	0.999975616	ENSG00000110931	CAMKK2
874.3032759	-0.193667863	0.207233449	-0.934539593	0.350025623	0.999975616	ENSG00000137414	FAM8A1
872.1353567	-0.087567721	0.244312576	-0.358424943	0.720025328	0.999975616	ENSG00000173852	DPY19L1
870.0724729	0.24486181	0.233546217	1.048451197	0.294430776	0.999975616	ENSG00000094916	CBX5
[truncated: 881,633 more chars]
